# The Role of microRNAs in Organismal and Skin Aging

**DOI:** 10.3390/ijms21155281

**Published:** 2020-07-25

**Authors:** Marta Gerasymchuk, Viktoriia Cherkasova, Olga Kovalchuk, Igor Kovalchuk

**Affiliations:** Department of Biological Sciences, University of Lethbridge, Lethbridge, AB T1K 3M4, Canada; marta.gerasymchuk@uleth.ca (M.G.); viktoriia.cherkasova@uleth.ca (V.C.); olga.kovalchuk@uleth.ca (O.K.)

**Keywords:** microRNA, aging, skin

## Abstract

The aging process starts directly after birth and lasts for the entire lifespan; it manifests itself with a decline in an organism’s ability to adapt and is linked to the development of age-related diseases that eventually lead to premature death. This review aims to explore how microRNAs (miRNAs) are involved in skin functioning and aging. Recent evidence has suggested that miRNAs regulate all aspects of cutaneous biogenesis, functionality, and aging. It has been noted that some miRNAs were down-regulated in long-lived individuals, such as let-7, miR-17, and miR-34 (known as longevity-related miRNAs). They are conserved in humans and presumably promote lifespan prolongation; conversely, they are up-regulated in age-related diseases, like cancers. The analysis of the age-associated cutaneous miRNAs revealed the increased expression of miR-130, miR-138, and miR-181a/b in keratinocytes during replicative senescence. These miRNAs affected cell proliferation pathways via targeting the p63 and Sirtuin 1 mRNAs. Notably, miR-181a was also implicated in skin immunosenescence, represented by the Langerhans cells. Dermal fibroblasts also expressed increased the levels of the biomarkers of aging that affect telomere maintenance and all phases of the cellular life cycle, such as let-7, miR-23a-3p, 34a-5p, miR-125a, miR-181a-5p, and miR-221/222-3p. Among them, the miR-34 family, stimulated by ultraviolet B irradiation, deteriorates collagen in the extracellular matrix due to the activation of the matrix metalloproteinases and thereby potentiates wrinkle formation. In addition to the pro-aging effects of miRNAs, the plausible antiaging activity of miR-146a that antagonized the UVA-induced inhibition of proliferation and suppressed aging-related genes (e.g., *p21WAF-1*, *p16*, and *p53*) through targeting Smad4 has also been noticed. Nevertheless, the role of miRNAs in skin aging is still not fully elucidated and needs to be further discovered and explained.

## 1. Introduction

The aging process starts directly after birth and lasts through the entire lifespan. For the majority of the population, it becomes a pressing problem around age 40–60 when the middle-age period of life free of diseases is followed by the period associated with the beginning of age-related diseases (ARDs) that eventually leads to a shortening of life. There are numerous theories describing the causative effects that deteriorate health, and almost all of them are based on the mechanisms that affect homeostasis at the genetic, molecular, cellular, tissue, and organismal levels. Many theories suggest that the appearance of various aging phenotypes occurs because of the age-dependent accumulation of damages [1]. At the same time, some individuals demonstrate the delayed development of aging features or ARDs and have a significantly longer life span despite their socioeconomic status. 

An increase in life expectancy around the world is positive, but it is a double-edged sword. On the one side, older people have more opportunities to live an active lifestyle, stay with their families, or continue to work and contribute to multiple areas of society. The extent of this possibility mainly depends on health. On the other side, an increased longevity is directly associated with a higher odds of developing ARDs. Therefore, this means that long-lived people will suffer from pain and disability for a much more extended period of time due to degenerative processes and ARDs (cardiovascular, diabetes, cancer, Parkinson’s, Alzheimer’s, and others). These morbidities drastically complicate a patient’s life and hasten mortality. It is worth noting that clinical manifestations of the abovementioned ARDs are preceded by long (10–20 years) asymptomatic periods of illness development [2]. If no changes are implemented, these trends will continue, and aging will become a significant social and economic burden [3]. The increased life spans have created a pressing need for a better understanding of the pathogenesis of the aging process and the identification of biomarkers and possible therapeutic targets for ARDs. 

We know that human longevity is determined by multiple factors, including genetic and environmental influences. About a quarter of the changes in our genetic material over our life period are due to various mutations or epigenetic regulation [4]. The results of epidemiological studies show that centenarians usually demonstrate features of healthy aging, accompanied by the lack or the delayed onset of ARDs. The process of aging is associated with various physiological and pathological molecular mechanisms, including intricate patterns of changes in gene expression leading to changes in the performance of multiple cell types, tissues, and organs. The insulated genetic aberrations in aging-relevant pathways trigger segmental, tissue-selective aging phenotypes [5]. Nevertheless, for a majority of tissues, it remains unknown which age-related modifications play a prominent etiological role in the mechanism of aging, or which ones are just epiphenomena [6]. 

The most evident and visible symptoms of aging in humans are manifested by changing skin appearance due to continuous exposure to exogenous irritants. Skin is the first line of defense against abiotic and biotic environmental factors [7,8]. Despite an ongoing renewal process, the cutaneous regenerative capacity reduces with age. A decrease in regenerative potential is often linked to a decline in the elimination of senescent cells, and their gradual accumulation results in the physiological aging of the tissue itself [9]. Many questions remain as to how to achieve longevity and healthy aging [10]. Skin seems to be a perfect health sensor because an alteration in its appearance directly signals the ongoing pathological changes in the organism. Thus, research on dermal health and aging is essential for an overall understanding of the processes of aging.

Current the biology of aging includes studies that explore or combine the issues of genomic instability, telomere length, deterioration of mitochondrial function, cellular senescence, epigenetic modifications, proteostasis, exhaustion of stem cells, autophagy, altered intercellular communication, as well as the deteriorated sensing accuracy or trophic function [1,11,12]. 

A typical signature of aging is the down-regulation of the expression of proteins involved in the electron transport chain. It remains disputable whether this is a reason or an outcome of the aging process. The respiratory chain is controlled by the communication between mitochondria and the nucleus, and changes in the expression of mitochondrial genes depend on energetic demand and a reductive load [13]. A disruption or reductive overload of the respiratory chain leads to the overproduction and release of reactive oxygen species (ROS) or Ca^2+^ by mitochondria or elevated levels of AMP and ADP. Signals of the abovementioned mitochondrial dysfunction are sensed by the mammalian target of rapamycin (mTOR) or AMP-dependent protein kinase (AMPK) and calmodulin. Subsequently, these signals are transduced to PPARγ-coactivator 1α (PGC1A), which activates the switch in the nuclear mitochondrial biogenic program [14,15]. Dysfunction in these pathways has been described in association with age-related human pathologies and has been observed in aged tissues [13]. Nevertheless, AMPK/mTOR and PGC1A are not unique pathways involved in the regulation of mitochondrial and nuclear interactions in the functionality of the respiratory chain but also require the cellular redox state via Sirtuin 1 (SIRT1) and *c-myc*. It was found that the SIRT1 expression and nicotinamide adenine dinucleotide (NAD^+^) levels decrease throughout aging in murine skeletal muscle and lead to dysfunction in mitochondrial biogenesis activation via SIRT1 and *c-myc* in the AMPK/PGC1A-independent fashion [16]. 

The majority of genetics-based longevity studies are primarily focused on sirtuins SIRT1 and SIRT3 and calorie restriction. Sirtuins have been reported to contribute to the extension of lifespan due to the improvement of mitochondrial function during dietary restrictions [17,18]. Additionally, sirtuins SIRT1, SIRT6, and SIRT7 are involved in regulating the telomere length and integrity via deacetylase activity, which also facilitates chromatin condensation and histone modification [19,20]. The potential mechanism might include the TOR signaling pathway. 

It is intriguing that within the last decade, it was discovered that microRNAs (miRNAs) were widely involved in the modulation of almost all processes in an organism, including aging. The potential role of miRNAs in aging biology is linked to the regulation of the expression of proteins maintaining the insulin-like growth factor (IGF)-1 and TOR signaling pathways [21]. Moreover, publications show that microRNAs play an essential role in regulating the balance between cellular proliferative capacity and replicative senescence [22]. The expression of various miRNAs was found to be directly associated with lifespan duration, ARDs, or their predisposition. Therefore, the expression of a subset of miRNAs also might be considered as one of the aging markers and as a predictor of chronological age, longevity and premature aging, the development of pathologies, or even a risk of mortality [23]. Unfortunately, there is a deficiency of robust information about miRNA functions in the processes of human aging and longevity, and many of these aspects still need to be discovered [24].

To date, there is a lack of information about the relation between miRNA involvement in the dermal and organismal aging process from the perspective of longevity and presumably skin disorders. Furthermore, there is currently a paucity of data that demonstrate possible links between miRNA expression and aging or age-related clinical outcomes [25]. In this review, we will focus on associations between miRNA expression and organismal and cutaneous aging.

## 2. Skin Structure and Biology

**Figure 1:**
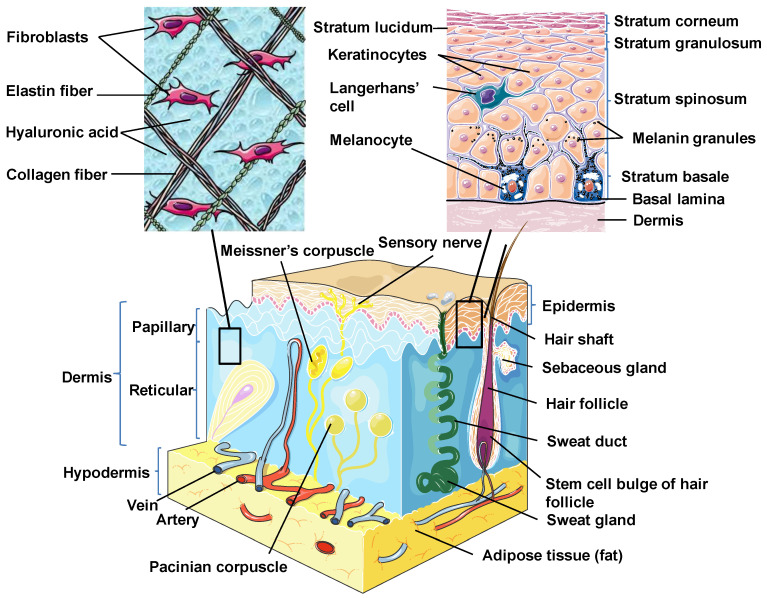
Skin structure. Schematic diagram showing the structural components of the normal human skin. This figure was created using images from Servier Medical Art Commons Attribution 3.0 Unported License (http://smart.servier.com). Servier Medical Art by Servier is licensed under a Creative Commons Attribution 3.0 Unported License.

Skin is the outermost organ and the largest one in the human body. It occupies about 8% of the total body mass of an adult human, covering 1.8 m^2^ of surface area [26]. Apart from being a protective barrier, the skin is also responsible for maintaining homeostasis, including the prevention of the percutaneous loss of electrolytes, fluid, and proteins; it also controls immune activity and sensory perception as well as temperature regulation [27,28,29]. The skin consists of three separate but functionally interdependent layers: the epidermis, dermis, and hypodermis (Figure 1). Their cellular components maintain the mechanical defense, photoprotection, immunosurveillance, nutrient metabolism, repair, and rejuvenation [30,31].

### 2.1. Epidermis

The epidermis is the outermost layer of the skin derived from the ectoderm and is tightly interlocked with dermal ridges. It mainly serves as a protective bulwark between the outside world and the internal environment of the body. Other epidermal functions are related to immunoprotection, thermoregulation, ultraviolet protection, resistance to trauma, and the maintenance of energy metabolism. The epidermis consists of keratinocytes, Langerhans cells, melanocytes, neuroendocrine (Merkel) cells, and inflammatory cells (Figure 1) [32]. Keratinocytes comprise over 90% of the cellular population of the epidermis and are connected by desmosomes and tight junctions. They produce and store an intracellular fibrous protein—keratin—that provides hardness and water-resistant properties to the skin and its appendages (hair and nails) [33]. Langerhans cells resemble macrophages in their functions and perform phagocytic activity. Melanocytes produce the pigment melanin that gives hair and skin its color and is also involved in UVR damage protection. Merkel cells function as a touch-perception receptor [32].

Depending on the location in the body, the skin is classified into two types: thin skin and thick skin. They are respectively made of four or five layers of epithelial cells in the epidermis. Thin skin consists of the following layers or strata (from the innermost layer to the outermost one): the stratum basale (the basal layer or stratum germinativum) which contains stem cells that attach the epidermis to the basal lamina; stratum spinosum (the spinous or prickle cell layer); stratum granulosum (the granular cell layer); stratum corneum (the cornified cell layer) (Figure 1). The fifth layer, located between the stratum corneum and the stratum granulosum, is known as the stratum lucidum (the clear cell layer), found predominantly in thick skin which covers the palms of the hands and the soles of the feet [31,33].

### 2.2. Dermis

The dermal layer delivers nutrients and supports the circulation of the skin [34]. It is separated from the epidermis by a structurally and chemically complex basement membrane zone presented by intertwining collagen fibers. The dermal-epidermal interface is tightly interlocked and stabilized by hemidesmosomes [33]. Structurally, the dermis is a connective tissue layer composed of an interconnected net of collagenous and elastin fibers embedded within the ground substance produced by fibroblasts; it is abundant with lymph and haemocirculation, nerves, and additional structural components (hair follicles and sweat glands). The majority of dermis cells are presented by fibroblasts that possess multifunctional activity but that are predominantly involved in the production of extracellular matrix (ECM) components [31]. Apart from fibroblasts, the dermal layer is composed of a broad number of endothelial and neural cells, supporting elements as well as mast cells embedded within a matrix of collagen and glycosaminoglycans. In the dermis, there are also dendritic and nondendritic monocyte/macrophages, myofibroblasts, along with dermal dendrocytes expressing factor XIIIa that are implicated in the hemostatic and inflammatory processes [35,36]. 

The dermis consists of two layers. The first one is a papillary layer, composed of loose areolar connective tissue with fibroblasts, collagen bundles, and thin elastic fibers that form a loose mesh and provide a mechanical anchorage and nutrients to the overlying epidermis. Additionally, it contains phagocytes, lymphatic capillaries, nerve fibers, and touch receptors known as the Meissner corpuscles [33]. The second layer is a reticular layer, which is a thicker layer located between the papillary dermis and the subcutaneous adipose tissue. It is made up of irregular dense connective tissue with thick collagen bundles and coarse elastic fibers and is supported by wide vascularization and the sensory and sympathetic nerve supply. Elastin fibers in this layer render the dermal elasticity and movements. While collagen fibers maintain the structural and tensile robustness, they are also responsible for the support of skin hydration by binding water. They are widespread in the papillary compartment and hypodermal layer [31]. 

### 2.3. Hypodermis

The hypodermis (also called the subcutaneous layer of the skin or superficial fascia) is a layer directly below the dermis which transitions to the fascial layers. It consists of well-vascularized, loose, areolar connective tissue with a lesser collagen content and abundant adipose tissue that form a layer of variable thickness depending on its location in the body. The hypodermis facilitates cutaneous mobility. The adipose tissue serves as a storage of metabolic energy and provides thermal insulation, cushioning properties, and shock absorption for the integumentary system [31,33]. 

About 30,000 dermal cells die every minute. At the same time, cutaneous development and normal physiology is a highly adapted process that depends on the cooperation between genetic networks and various regulatory factors [37]. Despite daily exposure to environmental damage, the skin sustains continuous self-renewal to replace old or injured cells and repair the damaged tissue [38]. However, when pathogenic effects exceed the protective cutaneous capabilities or regenerative properties depleted with age, it potentiates cellular senescence and different dermatological diseases [39].

## 3. Molecular Aspects of Skin Development

Cutaneous formation begins within the first two weeks of development. The ectoderm eventually forms the epidermis and melanocytes as well as the nervous system. The latter one starts developing during the third week of fetal life, when the ectoderm creates the neural plate within it and initiates neural crest development. The mesoderm gives rise to blood vessels, muscles, bones, and fibroblasts, except for some subpopulations that are derived from the neural crest of ectoderm. Besides this, the endoderm is not involved in cutaneous formation [32]. It is believed that neural crest cells secrete the Wnt1 ligand, a signaling molecule that activates the transcription factor and cytoskeletal protein, β-catenin. The latter controls the epithelial differentiation and the functioning of stem cells and appendages. Fibroblast growth factors induce the neural fate, while bone morphogenetic proteins (BMPs) and WNT signaling regulate the epidermal fate [38,40].

### 3.1. The Development of Epidermis

The surface ectoderm stimulates the generation of a single layer of basal keratinocytes from the germinativum—a layer of cuboidal undifferentiated and mitotically active cells. Keratinocytes are famous for an abundant synthesis of intermediate filaments keratins. Germinativum expresses the gene *p63*, which is essential for epidermal differentiation. Experiments demonstrate mice lethality immediately following birth due to *p63* deficiency [41]. In humans, *TP63* mutation causes several autosomal dominant ectodermal dysplasias, which are characterized by different combinations of limb, ectodermal, and orofacial abnormalities as well as alopecia, suggesting the role of *p63* in maintaining stem cell proliferation [42,43]. 

Numerous signaling pathways control the epidermis formation and stratification. For instance, the ligand Delta or Jagged binds the receptor Notch, which initiates transcription and epidermal differentiation. The inhibition of the Notch pathway results in the deficiency of the development of the cutaneous barrier [44]. The epidermis fails to stratify in cases with a low level of expression of the p63 transcription factor, which consequently deteriorates appendage formation [45]. The development of hair follicles and epidermal interfollicular lineages is regulated by the notch 1 and 2 signaling pathways [46]. The mitogen-activated protein kinases (MAPK) signal transduction pathway (also known as the Ras-Raf-MEK-ERK pathway) likewise regulates the epidermal proliferation and differentiation. The deletion of its key enzyme Mek1/2 (mitogen-activated protein dual kinases 1/2) results in cutaneous underdevelopment [47]. Conversely, the augmented activity of the epidermal growth factor (EGF) involved in the MAPK pathway potentiates proliferation and epidermal tumor growth [48]. 

Three distinct pools of stem cells are located in the interfollicular epidermis, the bulge, and the sebaceous gland supporting epidermal homeostasis. Skin stem cells undergo continuous self-renewal and differentiation into the required cell lineages to replenish cells such as keratinocytes, which die due to programmed termination or injury [28]. The renewal of the cutaneous barrier occurs via the spinous transition of basal cells in mature skin in parallel with changes in gene expression (e.g., the down-regulation of Keratin5/14 and up-regulation of Keratin1/10) controlled by p63 and the canonical Notch pathway [45].

### 3.2. The Development of Dermis

The dermis is formed from the mesodermal layer. The distinct boundary between the epidermis and the dermis appears at the eighth week of gestation. Fibroblasts represent the primary cell type that compose the dermis and demonstrate the ability to regulate the epithelial cell function. They also abundantly secrete collagens and other extracellular matrix molecules. In addition, fibroblasts show a high level of heterogeneity depending on the skin location. Thus, early embryonic dermal fibroblast progenitors can potentially differentiate into several cell types. For instance, the upper dermal fibroblast progenitor cells (PDGFRa, Blimp1, Dlk−, Irig1 markers) become the dermal papillae (a ball of fibroblasts that control hair keratinocytes) and the arrector pili muscle (the muscle attached to small hairs that causes goosebumps) [49,50]. In the upper dermis, there are papillary fibroblasts. The lower dermal fibroblast progenitor (PDGFRa, Blimp1−, Dlk1) generates the lower reticular fibroblasts and intradermal adipocytes/dermal white adipose tissues [40,50]. Reticular fibroblasts have a lower density and are biased for collagen I over collagen III production. Moreover, after an injury they differentiate into myofibroblasts, which promotes wound closure and presumably scarring [50]. The formation of the appendages is directly interrelated with the papillary dermis [40]. Skin development is not complete at birth because the final full barrier formation occurs afterwards.

## 4. Processes Involved in Skin Aging 

Biological aging is an integral process. It consists of multiple interrelated processes based on internal biochemical reactions, genetic programs, and ongoing external influences. Aging affects all organs and systems, albeit at a different rate [51]. The skin, like all other organs, undergoes distinguishable changes due to the progressive deterioration of morphological and physiological functions with increasing age. Thus, the barrier function and mechanical protection are compromised as a result of a gradual diminishing in the replacement of cellular components. Therefore, wound healing and immune responses are delayed, which is also accompanied by disrupted thermoregulation along with depleted sebum and sweat production. Furthermore, the skin provides the first visible evidence of the aging process [52].

Cutaneous aging is a complex phenomenon involving two simultaneously occurring processes: intrinsic aging and extrinsic aging. Intrinsic aging, known as chronological or natural aging, is genetically determined, whereas extrinsic aging is caused by environmental factors, such as chronic sun exposure (the total sunlight spectrum contains 45% ultraviolet light), and is known as photoaging [53]. The main symptoms of dermal aging are represented by the gradual process of wrinkle development, combined with skin sagging and drooping. Naturally aged skin looks dry and has fine wrinkles, but is still smooth and light [54]. In contrast, extrinsically photo-aged skin has thick layers (a leathery aspect), coarse wrinkles, irregular pigmentation (“age-spots” which are actinic lentigines), capillary telangiectasia, elastosis, and precancerous lesions such as actinic keratosis and malignant tumors [55,56,57,58]. 

Both types of cutaneous aging reduce the proliferative capacity of fibroblasts, keratinocytes, and melanocytes [53]. The morphological manifestations are the result of a decreased extracellular matrix (ECM) synthesis in the dermis due to the increased expression of matrix-degrading enzymes and matrix metalloproteinases (MMPs), which are mainly secreted by epidermal keratinocytes and dermal fibroblasts [55]. MMPs lead to the degradation and accumulation of a nonfunctional matrix due to cross-links in collagen fibers (intrinsic aging) or partially degraded elastin fibers (extrinsic aging) in combination with an increased oxidative stress and inflammatory process [57].

Extrinsic and intrinsic types of dermal aging are based on the common pathogenic molecular pathways [59]. The interactive features of both types of cutaneous aging are the generation of reactive oxygen species (ROS), resulting in the degradation of the ECM by the overexpressed MMPs [60]. The accumulation of ROS predominantly leads to the activation of tyrosine kinases receptors (TKRs) via the inactivation of protein tyrosine phosphatases. Then, TKRs are phosphorylated, and their subsequent signaling pathways are activated. These include the three families of mitogen-activated protein kinases (MAPKs): p38MAPK, c-Jun N-terminal kinase (JNK), and ERK (extracellular signal-regulated kinases). The activation of these MAPKs is followed by the increased expression of transcription factor activator protein-1 (AP-1). It stimulates the expression of different metalloproteinases (MMP-1, MMP-3, and MMP-9) and prevents the appearance of procollagen-1 [57,60,61]. The enhanced activities of MMPs eventually lead to ECM degradation, which deteriorates the cutaneous structure and manifests as aged skin.

Although the fundamental mechanisms are still poorly understood, a growing body of evidence is in favor of multiple pathogenic pathways of skin aging. Chronological and photoaging skin types may occur in parallel or overlap, and their leading mechanism is intensified oxidative stress, which presumably has one of the most detrimental effects on cutaneous aging. At the same time, all the vital processes are simultaneously controlled by genetic and epigenetic mechanisms, including DNA methylation, histone modification, and noncoding RNA (ncRNA) regulation (long ncRNAs, microRNAs, and circular RNAs) [62]. Despite a lot of accumulated data, microRNAs remain one of the most puzzling parts of aging biology.

## 5. Biogenesis of microRNAs

To date, nearly 2700 human mature miRNAs have been identified [63]; they are presumed to target over 60% of protein-coding genes in humans [24]. Additionally, miRNAs are thought to modulate the expression of approximately one-third of all human genetic codes [22]. 

MicroRNAs are a class of small non-coding endogenous, evolutionarily conserved RNAs (~19–24 nucleotides in length) that generally function to modulate the levels/translation of messenger RNA (mRNA). Their primary function is related to the posttranscriptional regulation of gene expression [64,65]. miRNAs block the translation and perform the cleavage of mRNA targets, resulting in a reduction in protein levels. Additionally, it was shown that the binding of miRNA stimulates the initiation of mRNA decay factors and a subsequent potentiation of mRNA instability and degradation, resulting in a reduction in expression levels [25]. Furthermore, miRNAs were proven to participate in numerous pathobiological processes, such as cell proliferation, differentiation, apoptosis/necrosis, tissue degeneration, cancer, and age-related diseases [22]. 

The biogenesis of miRNAs occurs through a multi-step process that requires both nuclear and cytoplasmic phases. Typically, miRNAs are transcribed by RNA polymerase II and III as a long primary miRNA (pri-miRNA) with a cap and a poly-A tail in the nucleus. Pri-miRNA contains a terminal loop, which consists of two flanking unstructured single-stranded tails and a double-stranded stem of about 30 base pairs [34]. Pri-miRNAs get processed into short 60–70 nucleotide stem-loop structures called precursor miRNAs (pre-miRNAs) by the microprocessor complex, which consists of the RNase III enzyme Drosha and double-stranded RNA binding protein DGCR8 (Di George syndrome critical region 8 gene) [66,67,68]. The translocation of pre-miRNA into cytoplasm across the nuclear pore complex occurs with the aid of the Exportin5 (XPO5) complex, which belongs to the karyopherin β family bound to the Ras-related nuclear protein (Ran) GTPase [34,69]. In the cytoplasm, the hydrolysis of guanosine triphosphate (GTP) to guanosine diphosphate (GDP) results in pre-miRNA discharge [66]. The RNase III enzyme Dicer processes pre-miRNA and generates the miRNA duplex with phosphate at the 5′ end and a two-nucleotide overhang with a hydroxyl group at the 3′ end. The miRNA duplex gets loaded onto Argonaute (AGO) protein in the presence of the RNA-induced silencing complex (RISC). RISC consists of Dicer, its partner protein, which is also known as the transactivation response RNA binding protein (TRBP), and AGO. TRBP detects the guide and passenger strands in the miRNA duplex. Once it recognizes the strand with the less stable 5’ end, the protein loads the miRNA duplex in the correct orientation onto the AGO protein [70]. AGO unwinds the duplex and removes the passenger strand retaining the mature miRNA molecule, and only one strand remains to form an effector complex RISC. After being loaded into RISC, miRNA correctly positions itself alongside the target mRNA to direct the posttranscriptional repression [34], [66,68,71].

MicroRNAs interact with the 3′ untranslated region (UTR) of the target mRNA and, once bound, there appears to be two ways in which it can inactivate the mRNA, thus causing mRNA degradation and the inhibition of the target mRNA translation. The first mechanism is based on the complementary binding of miRNA to mRNA, with a subsequent deadenylation and degradation of the target mRNA. The second mechanism leads to the inhibition of translation, where the RISC complex prevents the ribosome subunit from binding [72].

## 6. MicroRNAs as Aging Biomarkers

Many miRNAs have been previously demonstrated to be differentially expressed in cells, tissues, whole blood [10,73,74,75], plasma [76,77,78], and serum [79,80,81] in relation to age. 

The microRNA expression has been analyzed in the whole blood of 5221 adults, and 127 microRNAs that were differentially expressed depending on age and age-related diseases were identified. The targets of these miRNAs were associated with the main aging mechanisms, such as the regulation of transcription, translation, and immune responses. The authors suggested an epigenetic age prediction model based on the profiling of mRNA complementary to miRNA [82]. Kinser and Pincus (2020) reviewed the microRNA involvement in the modulation of aging, longevity, and age-related diseases within different species, including humans. They noticed that lin-4, let-7, miR-17, and miR-34 (known as longevity-related miRNAs) are conserved in humans and presumably promote lifespan prolongation [23].

Numerous experimental and computational data suggest that miRNAs regulate the expression of the majority of the known protein-coding genes at both the transcriptional and translational levels. In humans, microRNAs predominantly down-regulate their gene targets. The accurate prediction of gene targets and their involvement in the cellular regulations is necessary for understanding the physiological and pathological processes, pathogenesis of various diseases, and development of treatment strategies. Within the last decade, Dr. Xiaowei Wang and colleagues developed and improved an online database, miRDB (http://mirdb.org), for miRNA target prediction and functional annotations [83]. The miRDB database contains information about 7000 miRNAs and their 3.5 million predicted targets in five species: human, mouse, rat, dog, and chicken. Recently, the authors implemented an updated version of miRDB, the new MirTarget algorithm that allows analyzing custom miRNA data or gene target sequences (Tables S1–S10) for the transcriptome-wide prediction of gene targets or miRNA regulators for a flexible analysis of miRNA functions [83]. 

Zhang et al. (2015) profiled circulating miRNAs in serum from adults (40–70 years old) and identified significantly down-regulated age-dependent miRNAs, including miR-29b, miR-106b, miR-130b, miR-142-5p, and miR-340; and up-regulated miRNAs, including miR-92a, miR-222, and miR-375 [81]. Notably, miR-130b-5p was also involved in ribosome biogenesis and RNA processing and translation [10]. Interestingly, in long- versus short-lived individuals, there was a significant down-regulation of miR-340-3p, miR-374a-5p, and miR-376c in serum, which was negatively correlated with their lifespan [80]. miR-340-3p is predicted to have 316 targets (e.g., the *RPTOR* gene, a regulator of MTOR complex 1; the *GNAI3* gene, G protein subunit alpha i3; the *JAK* gene, Janus kinase 3, etc.) outlined in the miRDB online database [63,83]. In addition, a longer lifespan was positively correlated with the up-regulation of miR-211-5p, miR-1225-3p, and miR-5095 [80]. Another large population-based study profiling the miRNAs in plasma from adults identified the age-dependent elevation of the following miRNAs: let-7a-5p, miR-30b-5p, miR-30c-5p (Table S2), miR-126-3p, miR-142-3p, and miR-210; meanwhile, the miR-93-5p (Table S7) expression level was decreased [77]. Similarly, there is an age-dependent up-regulation of miR-126-3p in the plasma of older individuals [78]. Noren Hooten et al. (2013) also found down-regulated miR-151a-5p, miR-181a-5p, and miR-1248 in the human serum of older individuals (a mean age of 64.6 years); these miRNAs were often found to be differentially expressed in the inflammatory pathways of chronic age-related pathologies [79,84].

Table 1 introduces the list of differentially expressed miRNAs with respect to their role in normal skin development.

The analysis of plasma circulating miRNA from a twin study conducted by the National Heart, Lung, and Blood Institute identified significantly up-regulated miR-3615 and miR-619 in younger deceased twins compared to their longer-lived co-twin. Additionally, the increased expression of miR-423-5p and miR-4305 was detected in less than 70% of short-lived twins [76]. In this study, changes in the remaining longevity-related miRNAs were not significant, potentially due to a small sample size.

The immune system deteriorates with age. There is significant evidence of miRNA participation in pathways regulating immunological function. For instance, the miR-24 identified in whole blood [10] and peripheral blood mononuclear cells [94] was shown to be involved in the activation of the immune response (Table 1). Furthermore, Brunner et al. (2012) discovered that the down-regulation of the expression of histone H2A family member X, which plays an essential role in DNA damage response, was initiated by the increased expression of miR-24 in T-cell lines [97]. 

In addition, miR-181a was reported to facilitate local immunological stability [98]. It was shown to be co-expressed with the *CXCL16, RAB27A*, and *SPON2* genes responsible for immune response [82]. Cytotoxic T lymphocytes express RAB27A, which modulates the hexosaminidase and granzyme A granules secretion and immunological synapse activity [99]. Recent studies using senescence-accelerated mouse models discovered miR-301a-3p and miR-181a-1-3p to be differentially expressed in CD4+ T cells. Additionally, the up-regulated miR-301a was shown to facilitate the Th17 subset generation via the IL-6/23-STAT3 pathway, and miR-301a-3p was found to be significantly reduced in the CD4+ T cells of old mice. In contrast, miR-181a-1-3p was strongly elevated in the development of the age-related inflammatory phenotype, characterized by the enhanced levels of IL-6, IL-2, and TNF-α and the decreased levels of IL-10 in CD4+ T cells. Concomitantly, age-related alterations were found in the T cell differentiation-related pathways and their potential target genes, such as *GNG12, MAPK8, PIK3CA, PIK3R1, PRKACB, PRKCA*, and *PRKCB* regulated by miR-17-5p (Table S4), miR-144-3p, miR-451a, miR-301a-3p, and miR-582-5p [100]. Notably, numerous immune function-related genes (e.g., *APOBEC3G, CCR7, CD28, CST7, CTSW, DPP4, ETS1, FAIM3, FLT3LG, ICOS, KIF13B, SLAMF7, TCF7, TGFBR3, TNFRSF17, TRAT1*, etc.) were potential targets of miR-193b-3p. Similarly, miR-31-5p was predicted to target *CD27, CST7, DPP4, ETS1, FASLG, F2R, FLT3LG, GIMAP5, GZMA, ICOS, INPP5D, KYNU, TCF7, TGFBR3, TRAT1*, and many other immune function-related genes [82]. 

El Sharawy et al. (2012) validated eight miRNAs in the samples of 15 centenarians (a mean age of 96.4 years) and middle-aged individuals (a mean age of 45.9 years) that were proposed as longevity biomarkers. Among them, miR-106a, miR-126, miR-20a, miR-144*, and miR-18a were down-regulated, while miR-30d, miR-320d, and miR-320b were up-regulated in long-lived individuals [10]. Seven of these miRNAs were in line with the results of another study [101]. Storci et al. (2019) discovered longevity-related miRNAs in peripheral blood mononuclear cells and dermal fibroblasts from centenarians: down-regulated miR-21, miR-125a, miR-125b, miR-146a, and miR-155a (pro-inflammatory); up-regulated miR-335-5p, miR-532-5p, and miR-508-3p (anti-inflammatory). Simultaneously, the overexpressed pro-inflammatory and diminished levels of anti-inflammatory miRNAs were often linked to age-related diseases [74].

Due to the differential expression of microRNAs in long-lived individuals compared to younger ones, it was suggested that changes in the appearance of these miRNAs during physiological aging might inhibit carcinogenesis [21] and multiple pathologies associated with age [102]. For example, miR-17 (Table S1), miR-18a, miR18b, miR-20a (Table S5), and miR-106a (Table S8) in inflammation-associated conditions remained poorly expressed in centenarians [103,104], while they were up-regulated in cancers [21]. Conversely, the up-regulated miRNAs related to longevity were reported to be down-regulated in cancers; these miRNAs included miR-320d in colon cancer stem cells [105], miR-339-5p in breast cancer tissues [106], and miR-30d in chronic lymphoblastic leukemia [107] (Table 2). In comparison to healthy individuals, some age-related neurodegenerative diseases such as Alzheimer’s disease and spinocerebellar ataxia type 1 were associated with the increased expression of miR-101, miR-130, and miR-144 in the cerebellum and cortex [108]. The up-regulation of miR-130a was also found in the brain tissues of rats with cerebral infarction and was correlated with subsequent elevated neuronal apoptosis, accompanied by the liberation of inflammatory mediators and the inhibition of the PTEN/PI3K/Akt signaling pathway and anti-apoptotic genes [109]. 

The inverse expression of microRNAs in long-lived individuals and age-related diseases may signify the pathogenetic mechanisms of the latter and could potentially be used for diagnostic preventive purposes and treatment strategies. However, there is still incomplete information on the involvement of miRNA in various mechanisms of organismal aging and the skin in particular. 

**Figure 2:**
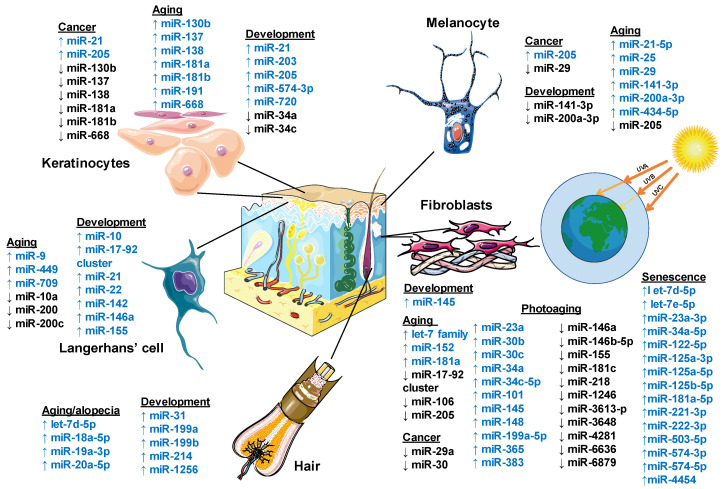
Role of microRNA regulation in human skin. Schematic diagram showing the involvement of miRNA in the cutaneous components’ development, aging processes, and cancer. MicroRNAs are shown as up-regulated in blue color and down-regulated in black color. This figure was created using images from Servier Medical Art Commons Attribution 3.0 Unported License. (http://smart.servier.com). Servier Medical Art by Servier is licensed under a Creative Commons Attribution 3.0 Unported License.

Figure 2 summarizes the miRNAs involved in normal development and skin pathologies.

Table 2 introduces the list of age-related differentially expressed miRNAs with respect to their involvement in various pathological conditions.

## 7. Epigenetic Regulation in Cutaneous Cellular Senescence

Cellular senescence is associated with the halting of mitotic cell divisions and the loss of proliferative potential, along with preserved viability or metabolic activity [126,127]. The senescent stage is represented by deviations in the protein expression levels and activity and, in particular, by changes in the p53/p21 and the pRB/p16 tumor suppressor pathways [128]. Indeed, tumor suppressor *p53*, also known as the Guardian of the Genome, down-regulates the expression of genes required for telomere maintenance, DNA repair, and centromere structure; its expression is enhanced by the progressive passaging of human fibroblasts [129]. One of the leading reasons for replicative senescence is telomere attrition, which works as a mitotic clock [130]. MicroRNAs likely play a role in cellular aging due to their involvement in proliferation-associated gene silencing. To date, about 30 proteins have been discovered to be implicated in cutaneous aging. Furthermore, most of the miRNAs associated with these proteins in the aging skin were reported to be down-regulated. For instance, filaggrin that undertakes the modulation of the cutaneous barrier function, hydration, and pH, and presumably the buffering capacity (via its breakdown products), was decreased 2.7-fold in aged skin [131]. Epigenetic mechanisms, including DNA methylation, histone modification, chromatin remodeling, and non-coding RNA control, also guide cellular senescence [132].

Within recent decades, robust evidence has supported an essential role of chromatin changes, including DNA methylation and post-translational histone modifications, in the regulation of gene expression. The latter depends on chromatin accessibility: more “relaxed” chromatin makes a gene more accessible for transcription. DNA and histone modifications establish an epigenetic “code” that controls gene function by favoring or limiting the transcriptional activity of genomic domains. DNA can be chemically modified by the methylation of cytosine nucleotides in a CpG context. DNA methylation is established and maintained by methylating enzymes, DNA methyltransferases (DNMTs). The hypermethylated DNA segment has a decreased chance of being transcribed into mRNA, and therefore genes located in this region typically have a low expression level. In contrast, hypomethylated DNA regions allow active transcription. Simultaneously, DNA methylation changes DNA–histone interactions, where unmethylated DNA is typically associated with acetylated histones, whereas methylated DNA is associated with deacetylated histones [133].

Epigenetic regulation plays a critical role in controlling the expression of various genetic elements. For example, DNA methylation and histone modifications are responsible for X chromosome inactivation and the repression of repetitive elements [134], such as transposable elements, telomeres, and centromeric DNA [4]. In turn, condensed chromatin limits recombination and decreases the risk of mutation, therefore facilitating genomic stability. Apart from the control of gene expression, epigenetic modifications control recombination, repair, stem cell development, and various processes of cellular differentiation. The impairment of epigenetic regulation is associated with aging and aging-related diseases, such as cancer [135]. For instance, extracellular matrix molecules such as ECM1 maintain the differentiation and maturation of the epidermis, keratinocyte adhesion and signaling, neovascularization, and the dermis functioning via ECM formation. The increased expression of ECM1 is associated with enhanced invasiveness and metastasis in different cancers. Recent studies demonstrated that DNA methylation and hydroxymethylation regulate the tumor-suppressive role of *ECM1*, *ATF5*, and *EOMES* in human hepatocellular carcinoma [136]. Other studies found significant differences in the DNA methylation levels in skin samples from young and old as well as sun-exposed and protected body sites; differences in DNA methylation were found at particular gene loci [137], correlating with the level of damage in the skin [29]. Site-specific changes in DNA methylation in response to aging or skin damage can be used for predicting the aging phenotype or the risk of development of specific dermal diseases.

The epigenetic regulation of the majority of biological processes depends on DNA methyltransferase 1 (DNMT1). DNMT1 maintains CpG methylation, and its decline in activity with age leads to a decrease in global methylation or epigenome changes (drift) in aging cells [138]. A recent study demonstrated that the DNMT1 expression was decreased in passage-aged fibroblasts, and its silencing in young fibroblasts induced the senescence phenotype. However, the microarray analyses showed that the down-regulation of miR-217 in old fibroblasts stimulated a partial reversion of the senescence phenotype. The authors also demonstrated that miR-217 promoted senescence by suppressing the DNMT1-mediated methylation of *p16* and *pRb* by targeting the DNMT1 3′-untranslated region (3′-UTR) in skin tissues and aged fibroblasts [139]. A more detailed description of the role of methylation and DNMT1 in aging is beyond the scope of this review.

## 8. The Role of microRNAs in the Dermal Aging

The significance of miRNAs in the regulation of skin development and homeostasis has started to unfold within the last few years. Numerous evidence-based studies have shed light on some novel miRNAs that regulate gene expression in stem cell biology and developmental biology. To date, we have accumulated a significant amount of knowledge about the miRNAs’ involvement in specific steps of the organismal development, physiology, and pathogenesis of certain conditions. Nevertheless, the role of miRNAs in regulating skin development, maturation, functioning, and aging is not yet fully characterized; therefore, multiple studies are in progress to elucidate the related mechanisms. In this section, we will concentrate on the mechanisms of molecular regulation of the aging process maintained by miRNAs and their main skin targets (i.e., keratinocytes, dermal fibroblasts, melanocytes, etc.) (Figure 2).

### 8.1. MicroRNAs Influence Keratinocyte Aging

One of the first studies focused on senescence-associated skin cells revealed a set of miRNAs involved in the regulation of epidermal normal human keratinocytes (NHKs). Among 126 senescence-associated miRNAs specified in senescent NHKs, 9 miRNAs (7%) were down-regulated and 117 miRNAs (93%) were up-regulated. Shin et al. (2011) found significantly up-regulated miR-137 and miR-668 during the replicative senescence of three independent NHK cultures and organismal aging. The authors reported a strong direct correlation between the above-mentioned miRNAs and an elevation in senescence-associated β-galactosidase activity, along with an elevation in p16INK4A and p53 senescence markers of the ARF/p53 and p16INK4A/RB pathways, respectively [140]. In parallel, they considered senescence as a potent tumor-suppressive pathway based on the fact that miR-137 and miR-668 were down-regulated in many human head and neck squamous cell carcinoma cell lines. Apart from the inhibition of cancer cell growth, miR-137 is involved in the induction of the cell cycle G1 arrest by targeting CDK6 and Cdc42. miR-137 likely works through the suppression of either the Cdc42-PAK1-MLC or ERK signaling pathway and the proliferation/invasion of colorectal cancer cells by mimicking the effects of Cdc42 knockdown [122,123].

**Figure 3:**
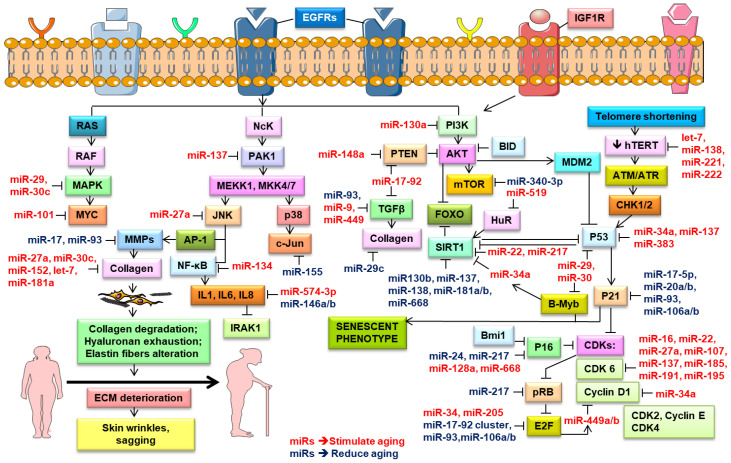
Role of microRNA in the regulation of the key molecular signaling pathways in the skin aging process. Schematic diagram shows the miRNAs that regulate cellular signaling mechanisms and potentially stimulate (depicted in red color) and reduce (represented in blue color) aging. Pointed arrow defines activation, while blunted – inhibition. This figure was created using images from Servier Medical Art Commons Attribution 3.0 Unported License (http://smart.servier.com). Servier Medical Art by Servier is licensed under a Creative Commons Attribution 3.0 Unported License.

Rivetti et al. (2012) identified up-regulated miR-138, miR-181a, miR-181b, and miR-130b in keratinocytes during replicative senescence [125]. They pointed out that these four miRNAs modulated the cell proliferation pathways by targeting p63 and Sirtuin 1 (SIRT1) mRNAs (Figure 3). SIRT1, an NAD-dependent histone deacetylase, modulated cellular differentiation, metabolism, immune and stress responses, and replicative senescence, and reduced p53-mediated apoptosis and FOXO-induced apoptosis [141]. *SIRT1* extended the lifespan in mammals [142], while its knockdown induced cellular senescence [125]. Aging is associated with a decrease in the SIRT1 levels and their protective effects, leading to numerous age-related diseases [17]. Down-regulated miR-138, miR-181a, miR-181b, and miR-130b as well as miR-137 and miR-668 were found in different cancers and were considered to be tumor-suppressor miRNAs [124,125,143]. Concomitantly, many research groups noted the role of miR-130b in targeting p63 mRNA. The *P63* gene was a transcription factor of the p53 family that played a vital part in the epithelial development. Based on in vitro and in vivo studies, *p63* was found to counteract cellular senescence and aging [9]. Besides, *p63* directly inhibited the expression of the senescence-inducing miRNAs (e.g., miR-130b, miRNA-138, miRNA-181a, and miR-181b) [125]. Moreover, the p63-mediated regulation of miRNA activity was demonstrated in tumor and metastasis suppression through the transcriptional regulation of Dicer and miR-130b [88].

Mancini et al. (2014) has broadly reviewed the role of senescence-associated miR-191 during keratinocyte senescence. MiR-191 is capable of blocking the G1–S phase transition through the control of cyclin-dependent protein kinase 6 (CDK6) gene expression. This blockage is manifested as a cell cycle arrest and a quiescent state that contributes to the development of senescence processes [9]. In parallel, miR-191 down-regulates CDK6 and SATB1 (special adenine- and thymine-rich binding protein 1) mRNA, accompanied by an increase in senescence-associated markers [144]. SATB1 acts as a docking site for chromatin remodeling enzymes; it also recruits corepressors (HDACs) or coactivators (HATs) directly to promoters [145]. miR-191 targets CDK9, NOTCH2, and RPS6KA3; it also represses the proliferation of primary human fibroblasts. Besides this, it plays a role in multiple cancer types, including gastric, colorectal, breast, thyroid, and hepatocellular carcinoma [146]. 

In addition to keratinocytes, the epidermis contains other cell types, such as melanocytes, Langerhans cells (LCs), Merkel cells, and stem cells [32] (Figure 1). 

### 8.2. The Role of microRNAs in the Age-Dependent Regulation of Immunomodulatory Skin Cells

Langerhans cells (LCs) are immature skin-residential dendritic cells (DCs) that recognize and present antigens. LCs, like all peripheral DCs, can traffic peripherally acquired antigens to regional lymph nodes, where they present antigens to naive and memory T cells and initiate adaptive immune responses or induce tolerance. These immune skin cells reside either in the epidermis or in the suprabasal layer of the stratified squamous epithelium of the upper part of the digestive system mucosae [147,148]. The epidermis is an epithelial layer that is composed primarily of keratinocytes, albeit LCs constitute 3–8% of the entire epidermal cellular population [149]. LCs are also involved in the pathogenesis of multiple cutaneous illnesses, including autoimmune diseases, allergy contact dermatitis, and cancer [148,149]. Considerable progress has been made in the characterization of various developmental processes in LCs [147], whereas the mechanisms involved in LC senescence and aging are still not clear.

The differential regulation of miRNAs in LCs with age contributes to immunosenescence by increasing the susceptibility to skin viral and fungal infections as well as to cutaneous cancer development. Several miRNAs are linked to LC development (miR-22 and miR-142 via the interferon regulatory factor 8 (IRF8), maturation, differentiation (miR-21, miR-34a, miR-99b, miR-223, and miR-511) and the immune function (miR-10, miR-21, miR-142-3p, miR-146a, and miR-155) [150]. Aging LCs are associated with the up-regulated expression of miR-709, miR-449, and miR-9 alongside with down-regulated miR-200c and miR-10a. miR-449 and miR-9 target the components of TGF-β signaling, such as TGFβ1, TGFβRI, TGFβRII, RUNX3, and C/EBP (CCAAT/enhancer-binding protein). Hence, the overexpressed miR-449 and miR-9 in aging LCs may down-regulate the TGF-β signaling pathway and also block the development of Langerhans cells [151]. LC homeostasis and development is also mediated by colony stimulating factor 1 (CSF1)/CSFR and IRF8 [147,152]. In mice, the overexpression of miR-709 and miR-449 in LCs down-regulates the expression of IRF8 and CSFR, causing a deficiency in the development of LCs [151]. Another study found that the transcription factor Gfi1 (growth factor independence 1) maintains LC homeostasis and is a potential target of miR-200 [153]. Interestingly, the down-regulation of miR-200 in aging LCs may directly up-regulate Gfi1, with a subsequent blockage of the LCs’ activity [151].

Besides this, the homeostasis and maturation of LCs are also dependent on the RANKL/RANK system, which is a putative target for miR-9 or miR-20 [151]. Hence, *RANKL* maintains lymphoid tissue formation and bone homeostasis. *RANKL* is also an apoptosis regulator gene, a binding partner of osteoprotegerin (OPG), a ligand for the receptor RANK that controls cell proliferation through cyclins D1, ID2, and ID4 [154,155]. The overexpression of miR-9 diminishes cellular migration and survival by repressing the expression of the proto-oncogene Cbl, which enhances the amount of pro-apoptotic protein Bim [156,157]. The miR-17-92 cluster encoding six miRNAs—miR-17, miR-18a, miR-19a, miR-20a, miR-19b-1, and miR-92a-1—is also highly expressed in skin-specific dendritic cells—i.e., the Langerhans cells [89]. Nevertheless, the miR-17-92 cluster has an unclear role in the process of survival, maturation, antigen presentation, and migration ability in these cells, presumably due to the functional redundancy [147,158]. Thereby, age-regulated miRNAs have the potential to deteriorate LC development, maturation, and function by targeting various signaling pathways.

### 8.3. The Age-Dependent microRNA Regulation of Various Processes in Melanocytes

Melanocytes are another network of epidermal cells that produce the pigment protecting from ultraviolet radiation [159]. Apart from the skin, these melanin-producing cells can be found in the hair follicles, eyes, inner ear, bones, heart, and brain. Melanins (eumelanin, pheomelanin, and neuromelanin) are pigment molecules that are most often synthesized by melanocytes (melanogenesis). In the skin, it subsequently distributes itself in the epidermal keratinocytes. Melanin absorbs light in the skin and hair, thus leading to photoreceptor shielding, thermoregulation, and photoprotection. Furthermore, it is a potent cation chelator and is able to act as a free radical sink, reducing the risk of folate depletion and dermal degradation, leading to premature aging and decreasing the odds of the development of malignant melanoma [160]. After the age of 30, melanocytes decrease in number by 8% to 20% per decade. The reduction in melanocyte numbers is smaller in sun-exposed areas. However, these cells are irregularly spaced and functionally compromised, producing an abnormal pigmentation upon sun exposure [37].

Interestingly, miRNAs and long non-coding RNAs were reported to play an essential role in melanogenesis-regulating molecules in melanocytes. The α-melanocyte-stimulating hormone (α-MSH), one of the leading factors in melanogenesis, was found to down-regulate the expression levels of miR-141-3p and miR-200a-3p. Conversely, the suppressed melanogenesis and tyrosinase activity were induced by the overexpression of miR-141-3p and miR-200a-3p in B16-4A5 cells, which is a melanoma-derived cell line. Furthermore, both miR-141-3p and miR-200a-3p directly targeted the microphthalmia-associated transcription factor (MITF), a key regulator of color genes coding for tyrosinase and hyaluronidase [66]. Tyrosinase, a melanocytic membrane-bound glycoprotein, is critical for melanin biosynthesis in the skin and hair. Hyaluronidase degrades subcutaneous hyaluronan, a central anti-aging component of the extracellular matrix, facilitating a mechanism for skin wrinkle formation [161]. MITF controls survival, proliferation, pigmentation, invasion, and oxygen stress in melanocytes by regulating the downstream gene expression. The up-regulation of miR-141-3p and miR-200a-3p decrease cutaneous pigmentation by targeting MITF [162]. There is also evidence that melanin production is controlled by miR-25, which suppresses MITF, whereas miR-21-5p regulates MITF indirectly via Sox-5 and miR-434-5p via tyrosinase and hyaluronidase targeting [161,163,164]. Besides this, the abatement of growth factors (e.g., SCF and α-MSH) may contribute to premature melanocyte cell senescence and the consequent clearance by the immune system [165]. Moreover, the inhibition of melanogenesis deteriorates melanocyte protective functions and might potentiate premature aging mechanisms. Indeed, senescent melanocytes induce paracrine telomere damage and senescence in the surrounding keratinocytes. This spontaneously affects the proliferative potential of keratinocytes, resulting in age-related epidermal atrophy [166].

Recent studies report that miRNAs might induce a senescence phenotype in melanocytes and inhibit melanoma tumor progression. The results of miRNA expression profiling analyses showed a significantly down-regulated miR-205 in melanoma compared with nevi; the miR-205 expression was correlated inversely with melanoma growth. The examination of miRNA target databases predicted E2F1 and E2F5 as plausible targets of miR-205 [167]. E2F1 is a member of the E2F family and regulates the G1/S transition phase in the cell cycle, which is tightly controlled by the retinoblastoma protein (Rb) (Figure 3). E2F1 is able to induce or inhibit apoptosis [168]. Furthermore, E2F1 potentiates cellular survival through the AKT pathway, which mediates cell growth, cell survival, and the inhibition of apoptosis [169]. Its increased expression prompts cells towards oncogenic transformation [170]. Another E2F family member, E2F5, similar to E2F1, is an oncogenic cell cycle regulator that is also found to be amplified in numerous tumors, including melanoma [171]. miR-205 in melanoma cell lines inversely correlates with the expression levels of E2F1 and E2F5. The increased expression of miR-205 reduces E2F-regulated AKT phosphorylation and suppresses the proliferative capacity of melanoma cells. The overexpression of miR-205 initiates a caspase cascade to induce apoptosis by suppressing the phosphorylation of caspase-9 and Bcl-2-associated death promoter (BAD), and concomitantly increasing the cleavage of caspase-3 and Poly (ADP-ribose) polymerase (PARP) with a subsequent release of cytochrome *c*. A continuous up-regulation of miR-205 suppresses melanoma cell proliferation, colony formation, and tumor cell growth in vivo and induces a senescence phenotype. The up-regulation of trimethyl-histone H3 (Lys-9), another marker of senescence at the chromatin level, is accompanied by the elevated expression of p16INK4A [45]. In turn, the up-regulation of p16INK4a activates Rb by suppressing its phosphorylation through the inhibition of CDK4/6 kinase activities and thus induces a senescence program. Altogether, these data indicate a tumor suppressor role of miR-205 in melanoma [167]. Another recent study suggests that the miR-29 family also prevents melanoma progression downstream of MAPK signaling by repressing the transcription factor MAFG (MAF bZIP transcription factor G) [172]; more studies are needed to substantiate these findings. 

### 8.4. The Role of microRNA Regulation in the Aging of Dermal Fibroblasts 

The dermal layer comprises large amounts of extracellular matrix (ECM), composed of proteins and specialized carbohydrates. Fibroblasts represent most of the dermal cells. In contrast, Schwann cells and nerve endings, endothelial cells organized in vessels, pericytes, mast cells, tissue macrophages, and other cells of the immune system are a minority. The dermis consists of the papillary (upper) and reticular (lower) layers. The papillary layer starts at the epidermal basement membrane and is more densely populated by fibroblasts than the reticular dermis, which contains thicker collagen fibers. However, the ECM becomes less organized due to the altered ECM protein turnover or/and post-translational modifications accumulated with age [159].

Fibroblasts produce and regenerate components of ECM; participate in wound healing, etc.; and, like other cells, deteriorate in functional capabilities with age. To date, we have accumulated a significant amount of evidence that miRNAs can also mediate the functions of fibroblasts. MiRNAs are linked to the reduced expression of transmembrane receptors (e.g., integrins) and components of the ECM (e.g., collagens, elastins) in senescent skin fibroblasts. Mancini et al. (2014) found that miR-152 and miR-181a induce senescence in proliferating human dermal fibroblasts. Indeed, miR-152 can significantly decrease dermal fibroblast adhesion through the down-regulation of integrin alpha 5 (ITGA5) by direct targeting [9]. Integrin-α5 participates in cell-surface-mediated signaling through the fibronectin receptor by enhancing cell adhesion and migration. Additionally, ITGA5 is one key functional miR-205 target in the re-epithelialization process in the epidermis. Similar to the effect of TGF-β1, the epidermal down-regulation of miR-205 promotes ITGA5 keratinocyte migration, whereas an increased miR-205 level is associated with delayed wound healing and chronic wound development [173]. Interestingly, the expression of collagen XVI (COL16A1) in senescent fibroblasts is down-regulated and directly targeted by the up-regulated miR-181a. COL16A is found in fibroblasts, keratinocytes, smooth muscle cells, and amnion; however, it is a minor component of the cutaneous ECM [174]. Collagen XVI provides the mechanical anchoring by connecting ECM proteins to cells; it is expressed in the dermal-epidermal junction zone of the papillary dermis [175]. It appears that both miR-152 and miR-181a may have an intricate role in the dermal ECM remodeling of aged skin [9]. Recently, more microRNAs as biomarkers of aging were identified in human dermal senescent fibroblasts that affected telomere maintenance and all phases of a cellular life cycle. Among the identified biomarkers were 15 up-regulated miRNAs, such as let-7d-5p (Table S3), let-7e-5p, miR-23a-3p, 34a-5p, miR-122-5p, miR-125a-3p, miR-125a-5p, miR-125b-5p, miR-181a-5p, miR-221-3p, miR-222-3p (Table S10), miR-503-5p, miR-574-3p (Table S1), miR-574-5p, and miR-4454; in fact, miR-221/222 were presented in both replicative and oxidative stress-induced senescence [85].

Martinez et al. (2011) demonstrated the up-regulation of the miR-29 and miR-30 microRNA families during induced and replicative fibroblast senescence, along with the activation of the Rb pathway. The binding of Rb-E2F complexes to the Myb-related protein B (B-Myb) promoter repressed B-Myb transcription and its expression, which could result in senescence. They found that the overexpressed miR-29a and miR-30 stimulated senescence directly suppressing the expression of the B-Myb transcription factor [176]. B-Myb is involved in cell cycle progression and cellular senescence by possessing both activator and repressor activities. The cyclin A/cyclin-dependent kinase 2 phosphorylated B-Myb during the S-phase of a cell cycle and was subsequently degraded during late G2 in a ubiquitin-dependent manner [177]. B-Myb was present in all mitotically cycling cells, thus activating the cell division cycle 2, cyclin D1 and insulin-like growth factor-binding protein 5 genes [178]. Interestingly, miR-29c was reduced in mice with isoproterenol-induced myocardial fibrogenesis. However, the increased expression of the anti-fibrotic factor miR-29c down-regulated its target genes, such as Collagen Type I Alpha 1 Chain (*COL1A1*), *COL1A2*, *COL3A1*, *COL5A1*, *fibrillin-1*, and *TGFβ1*, thus exerting protective effects against fibrosis and myocardial injury [179]. Conversely, the overexpression of B-Myb and factors mediating its activation strongly correlated with the down-regulated miR-29a and miR-30 and cancer progression, respectively [177]. miR-29a [180,181] and the entire miRNA-30 family (i.e., miR-30a (*p* = 0.024), miR-30b (*p* = 0.021), and miR-30c (*p* = 0.009)) [182] were found to be down-regulated in acute myeloid leukemia (AML).

In turn, the senescence process in dermal fibroblasts was also associated with down-regulated miRNAs, such as the miR-17-92 cluster and miR-106 [112]. The miR-17-92 cluster transcriptionally regulated genes involved in cell cycle control and tumor development, such as *BCL2L11 (BIM)*, *p63*, *p57*, *p27*, and *p21*. It potentiated the proliferation, and thereby was believed to counteract senescence [9]. Transcription factors E2F1 and E2F3 activated the miR-17-92 cluster transcription [183], while *p53* repressed it [184]. Hence, an age-dependent decrease in E2F1 and E2F3 led to a reduced expression of the miR-17-92 cluster, affecting the P53, ERB, and MAPK signaling pathways (Figure 3), and consequently accelerated apoptosis and inhibited growth [179]. Furthermore, the inhibition of miR-17-92 and miR-106b-25 in a c-MYC-dependent manner led to an increase in the BIM, PTEN, and P21 levels [185].

The data described here show that the expression of senescence-associated miRNAs affects the functioning of various genes in cutaneous cells and might promote or counteract cellular senescence. Moreover, many of these genes are expressed in other body tissues (e.g., *p63*, *SIRT1*) and are directly linked to organismal aging [22].

Table 3 summarizes the information about miRNAs that are differentially expressed in long-lived individuals and lists the top predicted targets of these miRNAs. A more detailed list of these targeted genes is presented in Table S1. All the possible targets of differentially expressed miRNAs are listed in Table S1–S10.

ADAM28, ADAM metallopeptidase domain 28; BMP2, bone morphogenetic protein 2; BNIP3L, BCL2 interacting protein 3 like; CASP3, caspase 3; CCND2, cyclin D2; CNR1, cannabinoid receptor 1 (CB1); CNRIP1 cannabinoid receptor interacting protein 1; COL1A2, collagen type I alpha 2 chain; CSF1, colony stimulating factor 1; E2F3, E2F transcription factor 3; EGFR, epidermal growth factor receptor; HA, hyaluronic acid; HAS2, hyaluronan synthase 2; IL6, interleukin 6; INF-α/β/ɣ, interferon-alpha/beta/gamma; JAK1, Janus kinase 1; KRT5, keratin 5; KRT10, keratin 10; LCs, Langerhans cells; MITF, melanocyte inducing transcription factor; MMP3, matrix metallopeptidase 3; NOTCH2NLA, notch 2 N-terminal like A; PAK5, p21 (RAC1) activated kinase 5; PIP_3,_ phosphatidylinositol-3,4,5-trisphosphate; pRB, retinoblastoma protein; PTEN, phosphatase and tensin homolog; RXRA, retinoid X receptor alpha; SIRT5, sirtuin 5; SIRT7, sirtuin 7; SMAD1, SMAD family member 1; SMAD4, SMAD family member 4; SPTBN1, spectrin beta, non-erythrocytic 1; TF, transcription factors; TGF-β, transforming growth factor-beta; USP45, ubiquitin specific peptidase 45.

Currently, various types of clinical trials directed towards studying aging and age-related pathologies are running worldwide. Table 4 summarizes ongoing clinical trials in which the prominent common feature is a microRNAs assessment in different tissues, including skin, and their role in the pathogenesis of aging-related processes; more detailed information about these clinical trials are published at https://clinicaltrials.gov/.

## 9. The Role of miRNAs in Dermal Photoaging

The continuous exogenous effect of solar ultraviolet (UV) exposure is one of the primary reasons for premature skin aging and is called photoaging. Daily, humans are typically exposed to the UV wavelengths of sunlight, which are primarily composed of UVA (90–95%) and UVB (5–10%). These wavelengths of light are absorbed by multiple cellular biomolecules, including genomic DNA [188]. The short-wave radiations (UVB under 300 nm and UVC) are the most dangerous. They can damage DNA because their wavelength coincides with the absorption spectra of DNA, RNA, and proteins [189]. DNA damage caused by UV exposure induces abundant bulky lesions, such as cyclobutane pyrimidine dimers (CPDs) and 6-4 photoproducts (6-4 PPs) [190]. CPDs formation occurs preferentially at thymine-containing dipyrimidine sites and likely also at methyl CpG-associated dipyrimidine sites, which include the TCG sequence [191]. It can lead directly or indirectly to DNA strand breaks that typically manifest in the form of mutations, neoplastic transformation, and premature cutaneous aging [192].

Recently, Zhang et al. (2020) demonstrated the UVB-induced up-regulation of miR-27a in keratinocytes [187]. They discovered that, following UVB irradiation, miR-27a facilitated the removal of CPDs and reduced cell apoptosis. Furthermore, miR-27a (Table S6) decreased the expression of *TARDBP* (target genes transactive response DNA-binding protein) and *APAF-1* (apoptotic protease activating factor-1) [187]. The overexpression of miR-1246 directly up-regulates UVB-induced apoptosis through the suppression of the Rhotekin 2 (*RTKN2*) expression [193]. An increase in the miR-26a in HaCaT cells promotes UVB-induced apoptosis by the histone methyltransferase enhancer zeste homolog 2 (EZH2), which it turn depends on the *MYC* expression level [187,194,195]. UVB irradiation also targets the 3′-UTR of EZH2 by the up-regulation of miR-101 and induces senescence in human fibroblasts. Nevertheless, both the overexpressed miR-101 and the down-regulated EZH2 may independently induce senescence in the absence of UVB exposure [196]. The UVB-stimulated up-regulation of miR-141 expression inversely correlates with PTEN (Phosphatase and tensin homologue deleted on chromosome 10) expression levels in HaCaT cells. In contrast, anti-miR-141 increased the PTEN expression and reduced apoptosis in the UVB-treated cells [197].

It was lately found that UVA- and UVB-exposure of keratinocytes in vitro induced hypermethylation and histone modifications, which silenced tumor suppressor genes (*Cip1/p21/p16INK4a*) relevant for photo-carcinogenesis. The DNA hypermethylation of the CDKN2A/B locus in dermal fibroblasts was also observed in gerontology donors [198]. A global genome analysis of DNA methylation patterns obtained from the sun-protected and sun-exposed skin areas of young and old individuals indicates that chronic insolation (i.e., extrinsic aging) results in global DNA hypomethylation, whereas the intrinsic aging of sun-protected skin may cause the widespread hypermethylation of CpG islands [6,199]. 

UV radiation types A and B are able to penetrate deep into the skin, harmfully affecting the epidermal and dermal layers. Despite the superficial location, epidermal keratinocytes are less sensitive to UV radiation than dermal fibroblasts [9]. Notably, UVA up-regulates c-Jun expression; the transcription of c-Jun is autoregulated by its product Jun, and c-Jun affects the collagen gene activity in human skin fibroblasts. MiR-155 is considered a direct modulator of c-Jun expression. UVA exposure down-regulates miR-155, leading to the up-regulation of c-Jun [200]. 

To date, several studies implicate miRNAs in premature cellular senescence caused by UVB irradiation. For example, the UVB-exposed cells have increased the expression of miR-34c-5p that targets the 3′-UTR of E2F3. Like other E2F family members, E2F3 participates in cell cycle progression, proliferation, and development. Additionally, it protects dermal fibroblasts from the UVB-induced premature senescence, because it regulates senescence-related genes (e.g., *p53* and *p21WAF-1*), and is also bound specifically to the retinoblastoma protein pRB in a cell-cycle dependent manner [201]. Recently, Blackstone et al. (2020) found a significant overexpression of the miR-34 family and a simultaneous increase in the Dicer1 expression along with the decreased fibroblast proliferation and reduced diameter of fibrils of dermal collagen caused by chronic UVB exposure in vivo. The disintegrated collagen structure entirely deteriorates the deposition of the extracellular matrix and alters the skin biomechanics, leading to skin weakness, stiffness, and reduced elasticity and pliability [202].

Differential miRNA expression profiles have been identified in photoaged primary human fibroblasts irradiated with long-wave UVA light [186]. They linked five up-regulated miRNAs (miR-30b, miR-30c, miR-148a (Table S9), miR-199a-5p, and miR-365) and seven down-regulated ones (miR-146a, miR-146b-5p, miR-181c, miR-218, miR-1246, miR-3613-3p, and miR-4281) in the photoaging process in dermal fibroblasts. The authors suggested that the predicted miRNA targets were associated with pathways in cancers, including the TGF-β pathway. They found that miR-146a targeted Smad4, TRAF6, IRAK1, and FADD; and they also noticed that the UVA-induced overexpression of miR-146a could moderately decrease the expression of Smad4 in fibroblasts via base pairing with Smad4 3′-UTR [186]. Curiously, another group of authors found that the overexpression of miR-146a antagonized the UVA-induced inhibition of proliferation and suppressed the up-regulation of aging-related genes (e.g., *p21WAF-1*, *p16*, and *p53*) through targeting Smad4 [203]. In another study, Bhaumik et al. (2009) showed the up-regulation of miRNAs 146a and 146b in response to the IL-1 receptor signaling as a part of a negative feedback loop that restrained the excessive senescence-associated secretory phenotype (SASP) activity. In particular, the ectopic expression of miR-146a/b in skin fibroblasts suppressed IL-6 and IL-8 secretion and down-regulated IRAK1 (Interleukin-1 receptor-associated kinase 1), a crucial component of the IL-1 receptor signal transduction pathway [102]. Even so, aging biology needs more evidence-based studies to support the role of miR-146 as an anti-aging photoprotective miRNA (Figure 3). 

In the recent study, using the microarray analysis, Srivastava et al. (2018) revealed the differentiated expression of miRNAs between the sun-protected and sun-exposed skin of young and aged groups [204]. In comparison with the sun-protected skin, in the sun-exposed skin the expression of miR-34a, miR-145, and miR-383 was increased, while that of miR-663b, miR-3648, and miR-6879 was decreased. The authors also found that miR-34a, miR-134, and miR-383 were differentially expressed in all three age groups studied. They explained the up-regulated levels of miR-34a and miR-383 in both chronological and photoaging. It was proposed that these miRNAs targeted the *p53*-gene network via a G1 phase arrest of the cell cycle and the suppression of cell-cycle regulator cyclin, such as D1 and Cdk4, resulting in senescence. During chronological and photoaging, the up-regulation of miR-134 was linked to the activation of NF-κB signaling alongside pro-inflammatory cytokines (e.g., IL-1β, TNF-α, and IL-8) and the inhibition of a longevity gene *SIRT1* [204].

Several studies reported the involvement of miRNAs in the modulation of autophagic pathways associated with aging. Indeed, the development of an aging phenotype was reciprocally related to the reduced autophagic activity [62]. Zhang et al. (2016) found that the PUVA (psoralen and ultraviolet A) and UVB-induced premature senescence was linked to the suppression of the autophagy signaling pathway via the overexpression of miR-23a. In this study, the authors elucidated that miR-23a inhibited the *AMBRA1* gene, [205] which is implicated in the formation of the autophagosome core complex at an early step of autophagy regulated by rapamycin complex 1 (mTORC1) [206]. Besides, in the model of the UVB stress-induced premature senescence (UVB-SIPS) of human fibroblasts, it was found that the overexpression of miR-23a-5p reduced cellular proliferation and accelerated a cell cycle arrest. At the same time, in UVB-SIPS fibroblasts it had an opposite effect on the photoprotective circulatory RNA_100797, which acted as a sponge of miR-23a-5p [207].

The progress made in recent years demonstrates beneficial results in the rejuvenation strategies on the models of SISP in fibroblasts. Thus, UVB-aged human dermal fibroblasts treated with exosomes from the induced pluripotent stem cells (iPSCs) showed the reduced cellular alterations and expression of matrix-degrading enzymes (MMP-1/3) that was directly involved in ECM degradation [208]. Interestingly, exosomes contain extracellular miRNAs, functional messenger RNAs, and some proteins generated in the host cells [209]. In the photoaged fibroblasts, the iPSCs-derived exosomes also restored the expression of collagen type I; they potentially might be used for future rejuvenation practices for skin aging treatment [208,210].

## 10. Conclusions

This review illustrates the role of microRNAs in skin development and functioning and describes the pathogenetic mechanisms of cutaneous aging. Numerous microRNAs are inversely expressed within different age groups, suggesting their role in the regulation of the age-related signaling pathways. Evidence of differentially regulated miRNAs during the chronological and photoaging of the skin was also presented. Although some studies have proposed miRNA senescence-regulated mechanisms in specific cutaneous cells, there is still controversy in the published results. The process of the regulation of senescence and aging in the skin by miRNAs is still not fully elucidated, which delays the development of antiaging therapies.

## Figures and Tables

**Table 1:** The differentially expressed microRNAs (miRNAs) in the skin of long-lived individuals and their role in normal skin development.

Long-Lived Individuals	Skin Development	References
**Up-regulated miRNAs in long-lived individuals**		
miR-574-3p	Differentiation of keratinocytes (inversely regulated by iASPP and p63)	[10,28,85,86,87,88]
miR-720	Differentiation of keratinocytes (inversely regulated by iASPP and p63)	[10,28,86,87,88]
**Down-regulated miRNAs in long-lived individuals**		
miR-17	Highly expressed in the Langerhans cells	[10,89]
miR-18a	Highly expressed in the Langerhans cells	[10,89]
miR-20a	Highly expressed in the Langerhans cells	[10,89]
miR-21	Up-regulated in hair development. Controls proliferation, differentiation, and epithelial-mesenchymal transition	[10,90,91,92]
miR-24	Regulates epidermal differentiation	[10,93,94]
miR-93	Highly expressed in the epidermis; regulates the development of the epidermis and hair follicle	[10,67]
miR-222	Up-regulation of miR-222 increases cell proliferation and melanogenesis via silencing *p27kip1* and *c-kit*, respectively	[10,95,96]

**Table 2:** The association of miRNAs differentially expressed in long-lived individuals with different pathological conditions.

Long-Lived Individuals	Diseases	References
**The up-regulated miRNAs in long-lived individuals**		
miR-30d	Down-regulated in cancers (e.g., chronic lymphoblastic leukemia)	[10,107]
miR-320d	Down-regulated in colon cancer stem cells	[10,105]
miR-339-5p	Down-regulated in breast cancer tissues	[10,106]
miR-423-5p	Up-regulated in short-lived twins	[10,76]^,^
**The down-regulated miRNAs in long-lived individuals**		
miR-17	Up-regulated in cancer	[10,21], [89,110,111,112]
miR-18a, miR-18b	Up-regulated in cancer Down-regulated in inflammation-associated conditions in centenarians	[10,21,103,110,113]
miR-20a	Up-regulated in cancer Down-regulated in inflammation-associated conditions in centenarians Increased levels in the plasma of patients with the complete remission of diffuse large B-cell lymphoma	[10,89,103,104,114]
miR-21	Up-regulated in numerous cancers (e.g., lung cancer, skin malignancies); psoriatic skin; fibrotic skin diseases; systemic scleroderma	[10,34], [115,116,117,118,119]
miR-93	Declined with age Up-regulated in cancer	[10,67,77,113,120]
miR-101	Up-regulated in Alzheimer’s disease and spinocerebellar ataxia type 1	[10,108]
miR-106a	Up-regulated in cancer	[10,21,111,112]
miR-106b	Up-regulated in cancer	[10,67,113,120]
miR-130a	Up-regulated in Alzheimer’s disease and spinocerebellar ataxia type 1 Up-regulated in rats’ brains with cerebral infarction with subsequent elevated neuronal apoptosis, and inhibited the PTEN/PI3K/Akt signaling pathway and anti-apoptotic genes.	[10,106,108,109,121]
miR-137	Down-regulated in different cancers (e.g., human head and neck squamous cell carcinoma cell lines)	[10,122,123,124,125]
miR-144	Up-regulated in Alzheimer’s disease and spinocerebellar ataxia type 1	[10,108]

**Table 3:** MicroRNA expression in the skin of long-lived individuals with a prediction of functional miRNA targets.

MicroRNAs in Long-Lived Individuals	Skin Aging	Predicted Gene Targets for microRNA*	Gene Function
**Up-regulated miRNAs in long-lived individuals**			
miR-574-3p	Up-regulated in senescent fibroblasts [10,85]	ADAM28	involved in cell-cell and cell-matrix interactions, muscle development, and neurogenesis
		RXRA	modulates retinoic acid-mediated gene activation; regulates cellular senescence; highly expressed in skin
		USP45	modulates the DNA repair ability of XPF-ERCC1 endonuclease; highly expressed in skin
miR-30c	Up-regulated in photoaged primary human fibroblasts irradiated UVA [10,182,186]	BNIP3L	binds to Bcl-2 pro-apoptotic protein; directly targets mitochondria; stimulates apoptosis: induces loss of membrane potential and the secretion of cytochrome c
		CNR1	known as CB1, G-protein-coupled receptor; inhibits adenylate cyclase activity in a dose-dependent, stereoselective and pertussis toxin-sensitive manner; abundantly present in the CNS and throughout the body
		SMAD1	modulates the signals of the bone morphogenetic proteins (BMPs), which participate in cell growth, morphogenesis, development, immune responses, and apoptosis; in the phosphorylated form, this protein generates a complex with SMAD4
**Down-regulated miRNAs in long-lived individuals**			
let-7d	Up-regulated in senescent fibroblasts [10,19,85]	COL1A2	a fibril-forming collagen present in connective tissues and ubiquitous in the dermis, corneas, bones, and tendons; gene mutations linked to Ehlers–Danlos syndrome type VIIB and osteogenesis imperfecta types I-IV
		HAS2	HA mediates space filling, cellular migration, wound healing, and tissue repair; supplies scaffolding for fibroblasts and new blood vessels; decreased expression is linked to skin aging, while the overexpression is linked to tumor metastasis
		KRT5	expressed in the basal layer of the epidermis with family member KRT14; involved in the differentiation of simple and stratified epithelial tissues; gene mutations associated with epidermolysis bullosa simplex
miR-17	Down-regulated in replicative senescence and organismal aging [10,89,112]	CNRIP1	interacts with the C-terminal tail of cannabinoid receptor 1
		PAK5	effector of Rac/Cdc42 GTPases that regulate cytoskeletal dynamics, cell cycle progression, proliferation, and survival signaling
		SIRT5	mediates mitochondrial enzymes activity in response to fasting and calorie restriction, cellular antioxidant defense
miR-20a	Down-regulated in skin fibroblasts replicative senescence and organismal aging [10,112]	BMP2	encodes a secreted ligand of the TGF-β superfamily of proteins and binds different TGF-β receptors; recruits and activates the SMAD family TFs; involved in bone, cartilage, and epidermal keratinocytes development
		JAK1	phosphorylates STAT proteins (signal transducers and activators of transcription); mediates INF-α/β and INF-ɣ signal transduction; associated with cutaneous aging and skin diseases such as vitiligo and psoriasis)
		KRT10	component of the epithelial cell cytoskeleton along with actin microfilaments and microtubules; involved in the renewal of the cutaneous barrier; gene mutations are associated with epidermolytic hyperkeratosis
miR-27a	Up-regulation in UVB-photoaged keratinocytes [187]	CNR1	known as CB1, G-protein-coupled receptor; inhibits adenylate cyclase activity in a dose-dependent, stereoselective, and pertussis toxin-sensitive manner; abundantly present in the CNS and throughout the body;
		CSF1	controls the production, differentiation, and function of macrophages and LCs
		MITF	mediates melanocyte development, survival, proliferation, pigmentation, invasion, and oxygen stress in melanocytes; gene mutations linked to auditory-pigmentary syndromes
miR-93	Highly expressed in the epidermis, declined with age [10,77]	E2F1	binds to retinoblastoma protein pRB; can modulate cell proliferation and p53-dependent/independent apoptosis
		MMP3	mediates the breakdown of extracellular matrix in normal physiological processes: embryonic development, reproduction, and tissue remodeling; wound repair; and pathological conditions, including cancerogenesis, metastasis, atherosclerosis, and arthritis. Plays a role in the degradation of collagens III, IV, IX, and X; fibronectin; cartilage proteoglycans; and laminin
		SMAD4	activated in TGF-β signaling; regulates the transcription of target genes; tumor suppressor; inhibits epithelial cell proliferation; ubiquitously expressed in skin
miR-106a	Down-regulated in replicative senescence and organismal aging [10,111,112]	CNRIP1	interacts with the C-terminal tail of cannabinoid receptor 1
		E2F3	involved in the cell cycle regulation via direct interaction with the pRB; protects dermal fibroblasts from UVB-induced premature senescence, as it regulates senescence-related genes (e.g., *p53* and *p21WAF-1*); gene alteration associated with multiple human cancers
		NOTCH2NLA	involved in cellular differentiation, alternative splicing; modulates brain neuronal development; interacts with neutrophil elastase; involved in hereditary neutropenia
miR-148a	Up-regulated in photoaged primary human fibroblasts irradiated with UVA [10,186]	CNR1	known as CB1, G-protein-coupled receptor; inhibits adenylate cyclase activity in a dose-dependent, stereoselective, and pertussis toxin-sensitive manner; abundantly present in the CNS and throughout the body
		PTEN	negatively regulates the intracellular levels of PIP_3_ in cells; facilitates energy metabolism in the mitochondria; acts as a tumor suppressor by negatively regulating the AKT/PKB signaling pathway; phosphatidylinositol 3-kinase/PTEN/AKT signaling pathway is involved in proliferation, migration, cell growth, cell survival, and tumorigenesis
		SIRT7	nuclear sirtuin that mediates cellular responses to energy demands; regulates telomere length and integrity via deacetylase activity, which also facilitates chromatin condensation and histone modification; modulates TGF-β1-induced proliferation and migration; like SIRT4, reduces fatty acid oxidation and insulin secretion
miR-222	Up-regulated during replicative senescence [10,19,85,95,96,111]	CASP3	modulates a central role in the execution phase of cell apoptosis; activates caspases 6, 7, and 9 and itself is processed by caspases 8, 9, and 10; associated with the cleavage of amyloid-beta 4A precursor protein, which is related to neuronal death in Alzheimer’s disease; involved in colon cancer cell migration, invasion, and metastasis
		CNR1	known as CB1, G-protein-coupled receptor; inhibits adenylate cyclase activity in a dose-dependent, stereoselective, and pertussis toxin-sensitive manner; abundantly present in the CNS and throughout the body
		SPTBN1	is an actin crosslinking and molecular scaffold protein that connects the plasma membrane to the actin cytoskeleton; mediates cell shape, the arrangement of transmembrane proteins, and the organization of organelles

*, data generated from the online database for the prediction of functional microRNA targets [83].Gene description based on the materials of https://www.ncbi.nlm.nih.gov/gene.

**Table 4:** Ongoing clinical trials associated with aging and age-related conditions which use miRNAs as a target or a marker.

Clinical Trials Identifier	Declared Study Description	Participants	Outcome Measures
NCT02953093 Study of Changes in Muscle and Fat Gene Transcription with Acarbose Treatment	The investigators are studying the effects of acarbose on muscle and adipose gene transcription in older adults—phase 2 of the clinical trial.	Males, 60–100 years old	Serum microRNA (time frame: 10 weeks): The difference in the miRNA expression level (with adjusted *p* <0.05) after 10 weeks of acarbose compared to 10 weeks of placebo.
NCT04113122 Senescence and the Early Ageing Phenotype After Chemotherapy for Testicular Cancer: the SEA-CAT Study	Cisplatin-combination chemotherapy causes DNA damage inevitably by platinum-DNA adduct formation of both tumor cells but also healthy cells. The investigators hypothesize that the increased burden of senescent cells during testicular cancer treatment induces SASP, resulting in a pro-inflammatory phenotype. Such a mechanism might trigger the development of late effects and an early aging phenotype after treatment for testicular cancer.	Males, 18–50 years old	Cellular senescence (time frame: 1 year): The change in the number of senescent cells in the skin and subcutaneous fat biopsy (defined as % of cells in which the nucleus is stained positive for P16, P21, and yH2Ax). Evaluation of the microRNA regulation of insulin signaling in adipose tissue: miR-103, miR-107, miR-29.
NCT01666340 Generation 100: How Exercise Affects Mortality and Morbidity in the Elderly: A Randomized Control Study	The investigators hypothesize that exercise will reduce the morbidity and mortality rates in an elderly population. The extent of reduction will be intensity-dependent.	Males and females, 70–76 years old	Epigenetics (time frame: 1 year follow up, 3 years follow up, 5 years follow up): assessment of transcriptomics (messenger RNAs and microRNAs) and proteomics arrays aimed at blood-borne factors induced by training.
CT03300388 Dysfunction of Adipose Tissue in Obesity, Inflammation and Aging: Mechanisms and Effects of Physical Exercise and Omega-3 Fatty Acids.	Dysfunction of adipose tissue in obesity, inflammation, and aging; mechanisms and effects of physical exercise and omega-3 fatty acids.	Post-menopausal women, 55–70 years old	Adipose tissue miRNA profiling (time frame: week 0 (baseline), week 16 (end of intervention)): Analysis of microRNA expression from subcutaneous abdominal adipose tissue biopsies by RNA-seq or GeneChip miRNA 4.0 Array (Affymetrix).
NCT03963050 Molecular and Functional Basis of Successful Aging and Frailty	This trial will aim to find evidence based on the behaviors and strategies that promote a healthy lifestyle and successful human aging during an investigation of frailty phenotypes (PF: physical or CF: cognitive).	Males and females, 80–90 years old	Expression of potential biomarkers (circulating microRNAs) (time frame: 3 years): assessment of the miRNA expression and involvement in skeletal muscle adaptation and repair.
NCT03052192 Biological Aging, Medication, Malnutrition and Inflammation Among Acutely Ill and Healthy Elderly.	The investigators will test a novel model for chronic inflammation and investigate the role of the NLRP3 inflammasome, NF-kB (nuclear factor kappa light chain enhancer of activated B cells), and miRNAs in biological aging and chronic inflammation in critical patients in intensive care departments to improve the current knowledge and possibilities for preventing chronic diseases and acute hospitalization.	Males and females, 20–110 years old	miRNA (time frame: from inclusion to 56 weeks after discharge): the levels of miRNA will be measured, and their association with NF-kB activity and biological aging will be investigated.
NCT04146818 Study of the Effects of Adapted Tango and Multidimensional Intervention in Prevention of Dementia in Aging: Developing Healthy Life Style Programs. (STRENGTH)	The STRENGTH project is a randomized controlled trial to assess the effects of 6 months of the multimodal intervention consisting of adapted Tango dancing together with music therapy, engagement in social activities, cognitive interference, and psycho-education on functional, biological, cognitive outcomes, and psycho-social aspects in 300 subjects with mild cognitive impairment.	Males and females, 60 years old and older	Change in genetic biomarkers (time frame: baseline, 6 months, 12 months, and 24 months): assessment of the cellular (leukocytes) microRNA of genes involved in the neurodegeneration process
NCT04005742 The Biomarkers Of Risk of Colorectal Cancer (BORICC) Follow-Up (BFU) Study	This longitudinal study concentrated on associations between aging and lifestyle factors and a panel of molecular biomarkers linked with colorectal cancer risk, which is the 3rd most common cancer worldwide.	Both sex (child, adult, older adult)	DNA methylation (% methylation) and microRNA expression in rectal mucosal biopsies (time frame: 12 years (on average): target gene and global methylation (LINE-1) in rectal mucosal biopsies

## Supplementary Materials

Supplementary materials can be found at https://www.mdpi.com/1422-0067/21/15/5281/s1.
