# Supplementary material for: The Role of microRNAs in Organismal and Skin Aging
Source: Int J Mol Sci. 2020 Jul 25;21(15):5281. doi: 10.3390/ijms21155281 (PMC7432402; doi:10.3390/ijms21155281)
Supplement: Supplementary file 1 [file ijms-21-05281-s001.zip › Appendix 1 hsa-miR-574-3p.docx]

**There are 22 predicted targets for hsa-miR-574-3p in miRDB**

| **Target Detail** | **Target Rank** | **Target Score** | **miRNA Name** | **Gene Symbol** | **Gene Description** |
| --- | --- | --- | --- | --- | --- |
| [Details](http://mirdb.org/cgi-bin/target_detail.cgi?targetID=1911073) | 1 | 92 | hsa-miR-574-3p | [RXRA](http://www.ncbi.nlm.nih.gov/entrez/query.fcgi?db=gene&cmd=Retrieve&dopt=full_report&list_uids=6256) | retinoid X receptor alpha |
| [Details](http://mirdb.org/cgi-bin/target_detail.cgi?targetID=1911069) | 2 | 87 | hsa-miR-574-3p | [TMPRSS11D](http://www.ncbi.nlm.nih.gov/entrez/query.fcgi?db=gene&cmd=Retrieve&dopt=full_report&list_uids=9407) | transmembrane serine protease 11D |
| [Details](http://mirdb.org/cgi-bin/target_detail.cgi?targetID=1911063) | 3 | 87 | hsa-miR-574-3p | [CCDC39](http://www.ncbi.nlm.nih.gov/entrez/query.fcgi?db=gene&cmd=Retrieve&dopt=full_report&list_uids=339829) | coiled-coil domain containing 39 |
| [Details](http://mirdb.org/cgi-bin/target_detail.cgi?targetID=1911056) | 4 | 81 | hsa-miR-574-3p | [ADAM28](http://www.ncbi.nlm.nih.gov/entrez/query.fcgi?db=gene&cmd=Retrieve&dopt=full_report&list_uids=10863) | ADAM metallopeptidase domain 28 |
| [Details](http://mirdb.org/cgi-bin/target_detail.cgi?targetID=1911067) | 5 | 81 | hsa-miR-574-3p | [USP45](http://www.ncbi.nlm.nih.gov/entrez/query.fcgi?db=gene&cmd=Retrieve&dopt=full_report&list_uids=85015) | ubiquitin specific peptidase 45 |
| [Details](http://mirdb.org/cgi-bin/target_detail.cgi?targetID=1911058) | 6 | 79 | hsa-miR-574-3p | [CLTC](http://www.ncbi.nlm.nih.gov/entrez/query.fcgi?db=gene&cmd=Retrieve&dopt=full_report&list_uids=1213) | clathrin heavy chain |
| [Details](http://mirdb.org/cgi-bin/target_detail.cgi?targetID=1911060) | 7 | 79 | hsa-miR-574-3p | [SERPINI2](http://www.ncbi.nlm.nih.gov/entrez/query.fcgi?db=gene&cmd=Retrieve&dopt=full_report&list_uids=5276) | serpin family I member 2 |
| [Details](http://mirdb.org/cgi-bin/target_detail.cgi?targetID=1911057) | 8 | 77 | hsa-miR-574-3p | [FAM19A5](http://www.ncbi.nlm.nih.gov/entrez/query.fcgi?db=gene&cmd=Retrieve&dopt=full_report&list_uids=25817) | family with sequence similarity 19 member A5, C-C motif chemokine like |
| [Details](http://mirdb.org/cgi-bin/target_detail.cgi?targetID=1911070) | 9 | 70 | hsa-miR-574-3p | [SNCB](http://www.ncbi.nlm.nih.gov/entrez/query.fcgi?db=gene&cmd=Retrieve&dopt=full_report&list_uids=6620) | synuclein beta |
| [Details](http://mirdb.org/cgi-bin/target_detail.cgi?targetID=1911075) | 10 | 69 | hsa-miR-574-3p | [FBXO34](http://www.ncbi.nlm.nih.gov/entrez/query.fcgi?db=gene&cmd=Retrieve&dopt=full_report&list_uids=55030) | F-box protein 34 |
| [Details](http://mirdb.org/cgi-bin/target_detail.cgi?targetID=1911077) | 11 | 62 | hsa-miR-574-3p | [CSE1L](http://www.ncbi.nlm.nih.gov/entrez/query.fcgi?db=gene&cmd=Retrieve&dopt=full_report&list_uids=1434) | chromosome segregation 1 like |
| [Details](http://mirdb.org/cgi-bin/target_detail.cgi?targetID=1911068) | 12 | 62 | hsa-miR-574-3p | [STRN3](http://www.ncbi.nlm.nih.gov/entrez/query.fcgi?db=gene&cmd=Retrieve&dopt=full_report&list_uids=29966) | striatin 3 |
| [Details](http://mirdb.org/cgi-bin/target_detail.cgi?targetID=1911064) | 13 | 61 | hsa-miR-574-3p | [CLRN3](http://www.ncbi.nlm.nih.gov/entrez/query.fcgi?db=gene&cmd=Retrieve&dopt=full_report&list_uids=119467) | clarin 3 |
| [Details](http://mirdb.org/cgi-bin/target_detail.cgi?targetID=1911062) | 14 | 57 | hsa-miR-574-3p | [MTM1](http://www.ncbi.nlm.nih.gov/entrez/query.fcgi?db=gene&cmd=Retrieve&dopt=full_report&list_uids=4534) | myotubularin 1 |
| [Details](http://mirdb.org/cgi-bin/target_detail.cgi?targetID=1911074) | 15 | 57 | hsa-miR-574-3p | [IL6](http://www.ncbi.nlm.nih.gov/entrez/query.fcgi?db=gene&cmd=Retrieve&dopt=full_report&list_uids=3569) | interleukin 6 |
| [Details](http://mirdb.org/cgi-bin/target_detail.cgi?targetID=1911066) | 16 | 55 | hsa-miR-574-3p | [BTG3](http://www.ncbi.nlm.nih.gov/entrez/query.fcgi?db=gene&cmd=Retrieve&dopt=full_report&list_uids=10950) | BTG anti-proliferation factor 3 |
| [Details](http://mirdb.org/cgi-bin/target_detail.cgi?targetID=1911061) | 17 | 53 | hsa-miR-574-3p | [TLNRD1](http://www.ncbi.nlm.nih.gov/entrez/query.fcgi?db=gene&cmd=Retrieve&dopt=full_report&list_uids=59274) | talin rod domain containing 1 |
| [Details](http://mirdb.org/cgi-bin/target_detail.cgi?targetID=1911071) | 18 | 52 | hsa-miR-574-3p | [ATP2A2](http://www.ncbi.nlm.nih.gov/entrez/query.fcgi?db=gene&cmd=Retrieve&dopt=full_report&list_uids=488) | ATPase sarcoplasmic/endoplasmic reticulum Ca2+ transporting 2 |
| [Details](http://mirdb.org/cgi-bin/target_detail.cgi?targetID=1911076) | 19 | 52 | hsa-miR-574-3p | [USP47](http://www.ncbi.nlm.nih.gov/entrez/query.fcgi?db=gene&cmd=Retrieve&dopt=full_report&list_uids=55031) | ubiquitin specific peptidase 47 |
| [Details](http://mirdb.org/cgi-bin/target_detail.cgi?targetID=1911072) | 20 | 51 | hsa-miR-574-3p | [NKG7](http://www.ncbi.nlm.nih.gov/entrez/query.fcgi?db=gene&cmd=Retrieve&dopt=full_report&list_uids=4818) | natural killer cell granule protein 7 |
| [Details](http://mirdb.org/cgi-bin/target_detail.cgi?targetID=1911065) | 21 | 51 | hsa-miR-574-3p | [BACE1](http://www.ncbi.nlm.nih.gov/entrez/query.fcgi?db=gene&cmd=Retrieve&dopt=full_report&list_uids=23621) | beta-secretase 1 |
| [Details](http://mirdb.org/cgi-bin/target_detail.cgi?targetID=1911059) | 22 | 50 | hsa-miR-574-3p | [SAMD4A](http://www.ncbi.nlm.nih.gov/entrez/query.fcgi?db=gene&cmd=Retrieve&dopt=full_report&list_uids=23034) | sterile alpha motif domain containing 4A |

Data generated from the online database for prediction of functional microRNA targets (Chen & Wang, 2020)

Chen, Y., & Wang, X. (2020). MiRDB: An online database for prediction of functional microRNA targets. *Nucleic Acids Research*, *48*(D1), D127–D131. https://doi.org/10.1093/nar/gkz757
