# Supplementary material for: The Role of microRNAs in Organismal and Skin Aging
Source: Int J Mol Sci. 2020 Jul 25;21(15):5281. doi: 10.3390/ijms21155281 (PMC7432402; doi:10.3390/ijms21155281)
Supplement: Supplementary file 1 [file ijms-21-05281-s001.zip › Appendix 10 hsa-miR-222-3p .docx]

**There are 619 predicted targets for hsa-miR-222-3p in miRDB**

| **Target Detail** | **Target Rank** | **Target Score** | **miRNA Name** | **Gene Symbol** | **Gene Description** |
| --- | --- | --- | --- | --- | --- |
| [Details](http://mirdb.org/cgi-bin/target_detail.cgi?targetID=2444086) | 1 | 100 | hsa-miR-222-3p | [GABRA1](http://www.ncbi.nlm.nih.gov/entrez/query.fcgi?db=gene&cmd=Retrieve&dopt=full_report&list_uids=2554) | gamma-aminobutyric acid type A receptor alpha1 subunit |
| [Details](http://mirdb.org/cgi-bin/target_detail.cgi?targetID=2444392) | 2 | 100 | hsa-miR-222-3p | [RIMS3](http://www.ncbi.nlm.nih.gov/entrez/query.fcgi?db=gene&cmd=Retrieve&dopt=full_report&list_uids=9783) | regulating synaptic membrane exocytosis 3 |
| [Details](http://mirdb.org/cgi-bin/target_detail.cgi?targetID=2444359) | 3 | 99 | hsa-miR-222-3p | [CDKN1B](http://www.ncbi.nlm.nih.gov/entrez/query.fcgi?db=gene&cmd=Retrieve&dopt=full_report&list_uids=1027) | cyclin dependent kinase inhibitor 1B |
| [Details](http://mirdb.org/cgi-bin/target_detail.cgi?targetID=2444424) | 4 | 98 | hsa-miR-222-3p | [HECTD2](http://www.ncbi.nlm.nih.gov/entrez/query.fcgi?db=gene&cmd=Retrieve&dopt=full_report&list_uids=143279) | HECT domain E3 ubiquitin protein ligase 2 |
| [Details](http://mirdb.org/cgi-bin/target_detail.cgi?targetID=2444546) | 5 | 98 | hsa-miR-222-3p | [PAIP1](http://www.ncbi.nlm.nih.gov/entrez/query.fcgi?db=gene&cmd=Retrieve&dopt=full_report&list_uids=10605) | poly(A) binding protein interacting protein 1 |
| [Details](http://mirdb.org/cgi-bin/target_detail.cgi?targetID=2444053) | 6 | 98 | hsa-miR-222-3p | [PANK3](http://www.ncbi.nlm.nih.gov/entrez/query.fcgi?db=gene&cmd=Retrieve&dopt=full_report&list_uids=79646) | pantothenate kinase 3 |
| [Details](http://mirdb.org/cgi-bin/target_detail.cgi?targetID=2444007) | 7 | 98 | hsa-miR-222-3p | [TCF12](http://www.ncbi.nlm.nih.gov/entrez/query.fcgi?db=gene&cmd=Retrieve&dopt=full_report&list_uids=6938) | transcription factor 12 |
| [Details](http://mirdb.org/cgi-bin/target_detail.cgi?targetID=2444482) | 8 | 97 | hsa-miR-222-3p | [RFX7](http://www.ncbi.nlm.nih.gov/entrez/query.fcgi?db=gene&cmd=Retrieve&dopt=full_report&list_uids=64864) | regulatory factor X7 |
| [Details](http://mirdb.org/cgi-bin/target_detail.cgi?targetID=2443942) | 9 | 97 | hsa-miR-222-3p | [HMBOX1](http://www.ncbi.nlm.nih.gov/entrez/query.fcgi?db=gene&cmd=Retrieve&dopt=full_report&list_uids=79618) | homeobox containing 1 |
| [Details](http://mirdb.org/cgi-bin/target_detail.cgi?targetID=2444239) | 10 | 97 | hsa-miR-222-3p | [RGS6](http://www.ncbi.nlm.nih.gov/entrez/query.fcgi?db=gene&cmd=Retrieve&dopt=full_report&list_uids=9628) | regulator of G protein signaling 6 |
| [Details](http://mirdb.org/cgi-bin/target_detail.cgi?targetID=2444334) | 11 | 96 | hsa-miR-222-3p | [EIF5A2](http://www.ncbi.nlm.nih.gov/entrez/query.fcgi?db=gene&cmd=Retrieve&dopt=full_report&list_uids=56648) | eukaryotic translation initiation factor 5A2 |
| [Details](http://mirdb.org/cgi-bin/target_detail.cgi?targetID=2444074) | 12 | 95 | hsa-miR-222-3p | [GRB10](http://www.ncbi.nlm.nih.gov/entrez/query.fcgi?db=gene&cmd=Retrieve&dopt=full_report&list_uids=2887) | growth factor receptor bound protein 10 |
| [Details](http://mirdb.org/cgi-bin/target_detail.cgi?targetID=2444132) | 13 | 95 | hsa-miR-222-3p | [KIT](http://www.ncbi.nlm.nih.gov/entrez/query.fcgi?db=gene&cmd=Retrieve&dopt=full_report&list_uids=3815) | KIT proto-oncogene receptor tyrosine kinase |
| [Details](http://mirdb.org/cgi-bin/target_detail.cgi?targetID=2444353) | 14 | 95 | hsa-miR-222-3p | [EML6](http://www.ncbi.nlm.nih.gov/entrez/query.fcgi?db=gene&cmd=Retrieve&dopt=full_report&list_uids=400954) | EMAP like 6 |
| [Details](http://mirdb.org/cgi-bin/target_detail.cgi?targetID=2444516) | 15 | 95 | hsa-miR-222-3p | [GNAI3](http://www.ncbi.nlm.nih.gov/entrez/query.fcgi?db=gene&cmd=Retrieve&dopt=full_report&list_uids=2773) | G protein subunit alpha i3 |
| [Details](http://mirdb.org/cgi-bin/target_detail.cgi?targetID=2444300) | 16 | 95 | hsa-miR-222-3p | [ARHGEF38](http://www.ncbi.nlm.nih.gov/entrez/query.fcgi?db=gene&cmd=Retrieve&dopt=full_report&list_uids=54848) | Rho guanine nucleotide exchange factor 38 |
| [Details](http://mirdb.org/cgi-bin/target_detail.cgi?targetID=2444502) | 17 | 95 | hsa-miR-222-3p | [CLVS2](http://www.ncbi.nlm.nih.gov/entrez/query.fcgi?db=gene&cmd=Retrieve&dopt=full_report&list_uids=134829) | clavesin 2 |
| [Details](http://mirdb.org/cgi-bin/target_detail.cgi?targetID=2444343) | 18 | 95 | hsa-miR-222-3p | [MIDN](http://www.ncbi.nlm.nih.gov/entrez/query.fcgi?db=gene&cmd=Retrieve&dopt=full_report&list_uids=90007) | midnolin |
| [Details](http://mirdb.org/cgi-bin/target_detail.cgi?targetID=2444320) | 19 | 94 | hsa-miR-222-3p | [GRIK1](http://www.ncbi.nlm.nih.gov/entrez/query.fcgi?db=gene&cmd=Retrieve&dopt=full_report&list_uids=2897) | glutamate ionotropic receptor kainate type subunit 1 |
| [Details](http://mirdb.org/cgi-bin/target_detail.cgi?targetID=2443962) | 20 | 94 | hsa-miR-222-3p | [VAPB](http://www.ncbi.nlm.nih.gov/entrez/query.fcgi?db=gene&cmd=Retrieve&dopt=full_report&list_uids=9217) | VAMP associated protein B and C |
| [Details](http://mirdb.org/cgi-bin/target_detail.cgi?targetID=2443974) | 21 | 94 | hsa-miR-222-3p | [RAB18](http://www.ncbi.nlm.nih.gov/entrez/query.fcgi?db=gene&cmd=Retrieve&dopt=full_report&list_uids=22931) | RAB18, member RAS oncogene family |
| [Details](http://mirdb.org/cgi-bin/target_detail.cgi?targetID=2444144) | 22 | 94 | hsa-miR-222-3p | [TGS1](http://www.ncbi.nlm.nih.gov/entrez/query.fcgi?db=gene&cmd=Retrieve&dopt=full_report&list_uids=96764) | trimethylguanosine synthase 1 |
| [Details](http://mirdb.org/cgi-bin/target_detail.cgi?targetID=2444149) | 23 | 94 | hsa-miR-222-3p | [FNIP2](http://www.ncbi.nlm.nih.gov/entrez/query.fcgi?db=gene&cmd=Retrieve&dopt=full_report&list_uids=57600) | folliculin interacting protein 2 |
| [Details](http://mirdb.org/cgi-bin/target_detail.cgi?targetID=2444362) | 24 | 94 | hsa-miR-222-3p | [DENND1B](http://www.ncbi.nlm.nih.gov/entrez/query.fcgi?db=gene&cmd=Retrieve&dopt=full_report&list_uids=163486) | DENN domain containing 1B |
| [Details](http://mirdb.org/cgi-bin/target_detail.cgi?targetID=2444093) | 25 | 94 | hsa-miR-222-3p | [MYLIP](http://www.ncbi.nlm.nih.gov/entrez/query.fcgi?db=gene&cmd=Retrieve&dopt=full_report&list_uids=29116) | myosin regulatory light chain interacting protein |
| [Details](http://mirdb.org/cgi-bin/target_detail.cgi?targetID=2444061) | 26 | 94 | hsa-miR-222-3p | [FNDC3A](http://www.ncbi.nlm.nih.gov/entrez/query.fcgi?db=gene&cmd=Retrieve&dopt=full_report&list_uids=22862) | fibronectin type III domain containing 3A |
| [Details](http://mirdb.org/cgi-bin/target_detail.cgi?targetID=2444549) | 27 | 94 | hsa-miR-222-3p | [ADAM22](http://www.ncbi.nlm.nih.gov/entrez/query.fcgi?db=gene&cmd=Retrieve&dopt=full_report&list_uids=53616) | ADAM metallopeptidase domain 22 |
| [Details](http://mirdb.org/cgi-bin/target_detail.cgi?targetID=2444554) | 28 | 93 | hsa-miR-222-3p | [C3orf70](http://www.ncbi.nlm.nih.gov/entrez/query.fcgi?db=gene&cmd=Retrieve&dopt=full_report&list_uids=285382) | chromosome 3 open reading frame 70 |
| [Details](http://mirdb.org/cgi-bin/target_detail.cgi?targetID=2444420) | 29 | 93 | hsa-miR-222-3p | [PCMTD1](http://www.ncbi.nlm.nih.gov/entrez/query.fcgi?db=gene&cmd=Retrieve&dopt=full_report&list_uids=115294) | protein-L-isoaspartate (D-aspartate) O-methyltransferase domain containing 1 |
| [Details](http://mirdb.org/cgi-bin/target_detail.cgi?targetID=2444461) | 30 | 93 | hsa-miR-222-3p | [WDR35](http://www.ncbi.nlm.nih.gov/entrez/query.fcgi?db=gene&cmd=Retrieve&dopt=full_report&list_uids=57539) | WD repeat domain 35 |
| [Details](http://mirdb.org/cgi-bin/target_detail.cgi?targetID=2444550) | 31 | 93 | hsa-miR-222-3p | [MIER3](http://www.ncbi.nlm.nih.gov/entrez/query.fcgi?db=gene&cmd=Retrieve&dopt=full_report&list_uids=166968) | MIER family member 3 |
| [Details](http://mirdb.org/cgi-bin/target_detail.cgi?targetID=2444067) | 32 | 93 | hsa-miR-222-3p | [TFG](http://www.ncbi.nlm.nih.gov/entrez/query.fcgi?db=gene&cmd=Retrieve&dopt=full_report&list_uids=10342) | TRK-fused gene |
| [Details](http://mirdb.org/cgi-bin/target_detail.cgi?targetID=2444161) | 33 | 93 | hsa-miR-222-3p | [GALNT3](http://www.ncbi.nlm.nih.gov/entrez/query.fcgi?db=gene&cmd=Retrieve&dopt=full_report&list_uids=2591) | polypeptide N-acetylgalactosaminyltransferase 3 |
| [Details](http://mirdb.org/cgi-bin/target_detail.cgi?targetID=2444416) | 34 | 93 | hsa-miR-222-3p | [NRK](http://www.ncbi.nlm.nih.gov/entrez/query.fcgi?db=gene&cmd=Retrieve&dopt=full_report&list_uids=203447) | Nik related kinase |
| [Details](http://mirdb.org/cgi-bin/target_detail.cgi?targetID=2444026) | 35 | 93 | hsa-miR-222-3p | [LOC100506388](http://www.ncbi.nlm.nih.gov/entrez/query.fcgi?db=gene&cmd=Retrieve&dopt=full_report&list_uids=100506388) | uncharacterized LOC100506388 |
| [Details](http://mirdb.org/cgi-bin/target_detail.cgi?targetID=2444163) | 36 | 93 | hsa-miR-222-3p | [ZNF91](http://www.ncbi.nlm.nih.gov/entrez/query.fcgi?db=gene&cmd=Retrieve&dopt=full_report&list_uids=7644) | zinc finger protein 91 |
| [Details](http://mirdb.org/cgi-bin/target_detail.cgi?targetID=2444194) | 37 | 92 | hsa-miR-222-3p | [DMRT3](http://www.ncbi.nlm.nih.gov/entrez/query.fcgi?db=gene&cmd=Retrieve&dopt=full_report&list_uids=58524) | doublesex and mab-3 related transcription factor 3 |
| [Details](http://mirdb.org/cgi-bin/target_detail.cgi?targetID=2444480) | 38 | 92 | hsa-miR-222-3p | [ERBB4](http://www.ncbi.nlm.nih.gov/entrez/query.fcgi?db=gene&cmd=Retrieve&dopt=full_report&list_uids=2066) | erb-b2 receptor tyrosine kinase 4 |
| [Details](http://mirdb.org/cgi-bin/target_detail.cgi?targetID=2444387) | 39 | 92 | hsa-miR-222-3p | [ARF4](http://www.ncbi.nlm.nih.gov/entrez/query.fcgi?db=gene&cmd=Retrieve&dopt=full_report&list_uids=378) | ADP ribosylation factor 4 |
| [Details](http://mirdb.org/cgi-bin/target_detail.cgi?targetID=2444522) | 40 | 92 | hsa-miR-222-3p | [CCN1](http://www.ncbi.nlm.nih.gov/entrez/query.fcgi?db=gene&cmd=Retrieve&dopt=full_report&list_uids=3491) | cellular communication network factor 1 |
| [Details](http://mirdb.org/cgi-bin/target_detail.cgi?targetID=2444280) | 41 | 92 | hsa-miR-222-3p | [RSBN1L](http://www.ncbi.nlm.nih.gov/entrez/query.fcgi?db=gene&cmd=Retrieve&dopt=full_report&list_uids=222194) | round spermatid basic protein 1 like |
| [Details](http://mirdb.org/cgi-bin/target_detail.cgi?targetID=2444544) | 42 | 92 | hsa-miR-222-3p | [RIT2](http://www.ncbi.nlm.nih.gov/entrez/query.fcgi?db=gene&cmd=Retrieve&dopt=full_report&list_uids=6014) | Ras like without CAAX 2 |
| [Details](http://mirdb.org/cgi-bin/target_detail.cgi?targetID=2444441) | 43 | 92 | hsa-miR-222-3p | [NAA25](http://www.ncbi.nlm.nih.gov/entrez/query.fcgi?db=gene&cmd=Retrieve&dopt=full_report&list_uids=80018) | N(alpha)-acetyltransferase 25, NatB auxiliary subunit |
| [Details](http://mirdb.org/cgi-bin/target_detail.cgi?targetID=2444555) | 44 | 92 | hsa-miR-222-3p | [ZFPM2](http://www.ncbi.nlm.nih.gov/entrez/query.fcgi?db=gene&cmd=Retrieve&dopt=full_report&list_uids=23414) | zinc finger protein, FOG family member 2 |
| [Details](http://mirdb.org/cgi-bin/target_detail.cgi?targetID=2444356) | 45 | 92 | hsa-miR-222-3p | [PPP3R1](http://www.ncbi.nlm.nih.gov/entrez/query.fcgi?db=gene&cmd=Retrieve&dopt=full_report&list_uids=5534) | protein phosphatase 3 regulatory subunit B, alpha |
| [Details](http://mirdb.org/cgi-bin/target_detail.cgi?targetID=2444452) | 46 | 92 | hsa-miR-222-3p | [CHSY1](http://www.ncbi.nlm.nih.gov/entrez/query.fcgi?db=gene&cmd=Retrieve&dopt=full_report&list_uids=22856) | chondroitin sulfate synthase 1 |
| [Details](http://mirdb.org/cgi-bin/target_detail.cgi?targetID=2444096) | 47 | 92 | hsa-miR-222-3p | [CDH2](http://www.ncbi.nlm.nih.gov/entrez/query.fcgi?db=gene&cmd=Retrieve&dopt=full_report&list_uids=1000) | cadherin 2 |
| [Details](http://mirdb.org/cgi-bin/target_detail.cgi?targetID=2444123) | 48 | 92 | hsa-miR-222-3p | [POGZ](http://www.ncbi.nlm.nih.gov/entrez/query.fcgi?db=gene&cmd=Retrieve&dopt=full_report&list_uids=23126) | pogo transposable element derived with ZNF domain |
| [Details](http://mirdb.org/cgi-bin/target_detail.cgi?targetID=2444022) | 49 | 92 | hsa-miR-222-3p | [AGTPBP1](http://www.ncbi.nlm.nih.gov/entrez/query.fcgi?db=gene&cmd=Retrieve&dopt=full_report&list_uids=23287) | ATP/GTP binding protein 1 |
| [Details](http://mirdb.org/cgi-bin/target_detail.cgi?targetID=2444005) | 50 | 92 | hsa-miR-222-3p | [DCUN1D1](http://www.ncbi.nlm.nih.gov/entrez/query.fcgi?db=gene&cmd=Retrieve&dopt=full_report&list_uids=54165) | defective in cullin neddylation 1 domain containing 1 |
| [Details](http://mirdb.org/cgi-bin/target_detail.cgi?targetID=2444222) | 51 | 92 | hsa-miR-222-3p | [SEC62](http://www.ncbi.nlm.nih.gov/entrez/query.fcgi?db=gene&cmd=Retrieve&dopt=full_report&list_uids=7095) | SEC62 homolog, preprotein translocation factor |
| [Details](http://mirdb.org/cgi-bin/target_detail.cgi?targetID=2444324) | 52 | 91 | hsa-miR-222-3p | [FOXN2](http://www.ncbi.nlm.nih.gov/entrez/query.fcgi?db=gene&cmd=Retrieve&dopt=full_report&list_uids=3344) | forkhead box N2 |
| [Details](http://mirdb.org/cgi-bin/target_detail.cgi?targetID=2444162) | 53 | 91 | hsa-miR-222-3p | [DCAF12](http://www.ncbi.nlm.nih.gov/entrez/query.fcgi?db=gene&cmd=Retrieve&dopt=full_report&list_uids=25853) | DDB1 and CUL4 associated factor 12 |
| [Details](http://mirdb.org/cgi-bin/target_detail.cgi?targetID=2444155) | 54 | 91 | hsa-miR-222-3p | [ETV3](http://www.ncbi.nlm.nih.gov/entrez/query.fcgi?db=gene&cmd=Retrieve&dopt=full_report&list_uids=2117) | ETS variant 3 |
| [Details](http://mirdb.org/cgi-bin/target_detail.cgi?targetID=2444011) | 55 | 91 | hsa-miR-222-3p | [ATXN1](http://www.ncbi.nlm.nih.gov/entrez/query.fcgi?db=gene&cmd=Retrieve&dopt=full_report&list_uids=6310) | ataxin 1 |
| [Details](http://mirdb.org/cgi-bin/target_detail.cgi?targetID=2444442) | 56 | 91 | hsa-miR-222-3p | [MRAP2](http://www.ncbi.nlm.nih.gov/entrez/query.fcgi?db=gene&cmd=Retrieve&dopt=full_report&list_uids=112609) | melanocortin 2 receptor accessory protein 2 |
| [Details](http://mirdb.org/cgi-bin/target_detail.cgi?targetID=2444044) | 57 | 91 | hsa-miR-222-3p | [AP3B2](http://www.ncbi.nlm.nih.gov/entrez/query.fcgi?db=gene&cmd=Retrieve&dopt=full_report&list_uids=8120) | adaptor related protein complex 3 subunit beta 2 |
| [Details](http://mirdb.org/cgi-bin/target_detail.cgi?targetID=2444098) | 58 | 91 | hsa-miR-222-3p | [CBWD5](http://www.ncbi.nlm.nih.gov/entrez/query.fcgi?db=gene&cmd=Retrieve&dopt=full_report&list_uids=220869) | COBW domain containing 5 |
| [Details](http://mirdb.org/cgi-bin/target_detail.cgi?targetID=2444027) | 59 | 91 | hsa-miR-222-3p | [KIF20A](http://www.ncbi.nlm.nih.gov/entrez/query.fcgi?db=gene&cmd=Retrieve&dopt=full_report&list_uids=10112) | kinesin family member 20A |
| [Details](http://mirdb.org/cgi-bin/target_detail.cgi?targetID=2444112) | 60 | 91 | hsa-miR-222-3p | [CASZ1](http://www.ncbi.nlm.nih.gov/entrez/query.fcgi?db=gene&cmd=Retrieve&dopt=full_report&list_uids=54897) | castor zinc finger 1 |
| [Details](http://mirdb.org/cgi-bin/target_detail.cgi?targetID=2444493) | 61 | 91 | hsa-miR-222-3p | [KIF16B](http://www.ncbi.nlm.nih.gov/entrez/query.fcgi?db=gene&cmd=Retrieve&dopt=full_report&list_uids=55614) | kinesin family member 16B |
| [Details](http://mirdb.org/cgi-bin/target_detail.cgi?targetID=2444145) | 62 | 91 | hsa-miR-222-3p | [TMCC1](http://www.ncbi.nlm.nih.gov/entrez/query.fcgi?db=gene&cmd=Retrieve&dopt=full_report&list_uids=23023) | transmembrane and coiled-coil domain family 1 |
| [Details](http://mirdb.org/cgi-bin/target_detail.cgi?targetID=2443952) | 63 | 91 | hsa-miR-222-3p | [CBWD6](http://www.ncbi.nlm.nih.gov/entrez/query.fcgi?db=gene&cmd=Retrieve&dopt=full_report&list_uids=644019) | COBW domain containing 6 |
| [Details](http://mirdb.org/cgi-bin/target_detail.cgi?targetID=2443978) | 64 | 91 | hsa-miR-222-3p | [VASH1](http://www.ncbi.nlm.nih.gov/entrez/query.fcgi?db=gene&cmd=Retrieve&dopt=full_report&list_uids=22846) | vasohibin 1 |
| [Details](http://mirdb.org/cgi-bin/target_detail.cgi?targetID=2444289) | 65 | 91 | hsa-miR-222-3p | [CBWD3](http://www.ncbi.nlm.nih.gov/entrez/query.fcgi?db=gene&cmd=Retrieve&dopt=full_report&list_uids=445571) | COBW domain containing 3 |
| [Details](http://mirdb.org/cgi-bin/target_detail.cgi?targetID=2443988) | 66 | 91 | hsa-miR-222-3p | [TP53BP2](http://www.ncbi.nlm.nih.gov/entrez/query.fcgi?db=gene&cmd=Retrieve&dopt=full_report&list_uids=7159) | tumor protein p53 binding protein 2 |
| [Details](http://mirdb.org/cgi-bin/target_detail.cgi?targetID=2444365) | 67 | 91 | hsa-miR-222-3p | [HNRNPH3](http://www.ncbi.nlm.nih.gov/entrez/query.fcgi?db=gene&cmd=Retrieve&dopt=full_report&list_uids=3189) | heterogeneous nuclear ribonucleoprotein H3 |
| [Details](http://mirdb.org/cgi-bin/target_detail.cgi?targetID=2443991) | 68 | 91 | hsa-miR-222-3p | [CBWD1](http://www.ncbi.nlm.nih.gov/entrez/query.fcgi?db=gene&cmd=Retrieve&dopt=full_report&list_uids=55871) | COBW domain containing 1 |
| [Details](http://mirdb.org/cgi-bin/target_detail.cgi?targetID=2444227) | 69 | 91 | hsa-miR-222-3p | [SUN2](http://www.ncbi.nlm.nih.gov/entrez/query.fcgi?db=gene&cmd=Retrieve&dopt=full_report&list_uids=25777) | Sad1 and UNC84 domain containing 2 |
| [Details](http://mirdb.org/cgi-bin/target_detail.cgi?targetID=2444312) | 70 | 91 | hsa-miR-222-3p | [FMR1](http://www.ncbi.nlm.nih.gov/entrez/query.fcgi?db=gene&cmd=Retrieve&dopt=full_report&list_uids=2332) | fragile X mental retardation 1 |
| [Details](http://mirdb.org/cgi-bin/target_detail.cgi?targetID=2444056) | 71 | 90 | hsa-miR-222-3p | [APOLD1](http://www.ncbi.nlm.nih.gov/entrez/query.fcgi?db=gene&cmd=Retrieve&dopt=full_report&list_uids=81575) | apolipoprotein L domain containing 1 |
| [Details](http://mirdb.org/cgi-bin/target_detail.cgi?targetID=2444419) | 72 | 90 | hsa-miR-222-3p | [FERMT2](http://www.ncbi.nlm.nih.gov/entrez/query.fcgi?db=gene&cmd=Retrieve&dopt=full_report&list_uids=10979) | fermitin family member 2 |
| [Details](http://mirdb.org/cgi-bin/target_detail.cgi?targetID=2444301) | 73 | 90 | hsa-miR-222-3p | [PHACTR4](http://www.ncbi.nlm.nih.gov/entrez/query.fcgi?db=gene&cmd=Retrieve&dopt=full_report&list_uids=65979) | phosphatase and actin regulator 4 |
| [Details](http://mirdb.org/cgi-bin/target_detail.cgi?targetID=2444247) | 74 | 90 | hsa-miR-222-3p | [PIK3R1](http://www.ncbi.nlm.nih.gov/entrez/query.fcgi?db=gene&cmd=Retrieve&dopt=full_report&list_uids=5295) | phosphoinositide-3-kinase regulatory subunit 1 |
| [Details](http://mirdb.org/cgi-bin/target_detail.cgi?targetID=2443957) | 75 | 90 | hsa-miR-222-3p | [MARK1](http://www.ncbi.nlm.nih.gov/entrez/query.fcgi?db=gene&cmd=Retrieve&dopt=full_report&list_uids=4139) | microtubule affinity regulating kinase 1 |
| [Details](http://mirdb.org/cgi-bin/target_detail.cgi?targetID=2444104) | 76 | 90 | hsa-miR-222-3p | [CBWD2](http://www.ncbi.nlm.nih.gov/entrez/query.fcgi?db=gene&cmd=Retrieve&dopt=full_report&list_uids=150472) | COBW domain containing 2 |
| [Details](http://mirdb.org/cgi-bin/target_detail.cgi?targetID=2444213) | 77 | 90 | hsa-miR-222-3p | [KLF7](http://www.ncbi.nlm.nih.gov/entrez/query.fcgi?db=gene&cmd=Retrieve&dopt=full_report&list_uids=8609) | Kruppel like factor 7 |
| [Details](http://mirdb.org/cgi-bin/target_detail.cgi?targetID=2444195) | 78 | 90 | hsa-miR-222-3p | [SYT10](http://www.ncbi.nlm.nih.gov/entrez/query.fcgi?db=gene&cmd=Retrieve&dopt=full_report&list_uids=341359) | synaptotagmin 10 |
| [Details](http://mirdb.org/cgi-bin/target_detail.cgi?targetID=2444339) | 79 | 90 | hsa-miR-222-3p | [LHFPL2](http://www.ncbi.nlm.nih.gov/entrez/query.fcgi?db=gene&cmd=Retrieve&dopt=full_report&list_uids=10184) | LHFPL tetraspan subfamily member 2 |
| [Details](http://mirdb.org/cgi-bin/target_detail.cgi?targetID=2444450) | 80 | 89 | hsa-miR-222-3p | [BRWD1](http://www.ncbi.nlm.nih.gov/entrez/query.fcgi?db=gene&cmd=Retrieve&dopt=full_report&list_uids=54014) | bromodomain and WD repeat domain containing 1 |
| [Details](http://mirdb.org/cgi-bin/target_detail.cgi?targetID=2444500) | 81 | 89 | hsa-miR-222-3p | [PPP2R2A](http://www.ncbi.nlm.nih.gov/entrez/query.fcgi?db=gene&cmd=Retrieve&dopt=full_report&list_uids=5520) | protein phosphatase 2 regulatory subunit Balpha |
| [Details](http://mirdb.org/cgi-bin/target_detail.cgi?targetID=2444088) | 82 | 89 | hsa-miR-222-3p | [CXCL12](http://www.ncbi.nlm.nih.gov/entrez/query.fcgi?db=gene&cmd=Retrieve&dopt=full_report&list_uids=6387) | C-X-C motif chemokine ligand 12 |
| [Details](http://mirdb.org/cgi-bin/target_detail.cgi?targetID=2443958) | 83 | 89 | hsa-miR-222-3p | [NXPH1](http://www.ncbi.nlm.nih.gov/entrez/query.fcgi?db=gene&cmd=Retrieve&dopt=full_report&list_uids=30010) | neurexophilin 1 |
| [Details](http://mirdb.org/cgi-bin/target_detail.cgi?targetID=2444418) | 84 | 89 | hsa-miR-222-3p | [CCDC18](http://www.ncbi.nlm.nih.gov/entrez/query.fcgi?db=gene&cmd=Retrieve&dopt=full_report&list_uids=343099) | coiled-coil domain containing 18 |
| [Details](http://mirdb.org/cgi-bin/target_detail.cgi?targetID=2443949) | 85 | 89 | hsa-miR-222-3p | [RFX8](http://www.ncbi.nlm.nih.gov/entrez/query.fcgi?db=gene&cmd=Retrieve&dopt=full_report&list_uids=731220) | RFX family member 8, lacking RFX DNA binding domain |
| [Details](http://mirdb.org/cgi-bin/target_detail.cgi?targetID=2444296) | 86 | 89 | hsa-miR-222-3p | [C6orf118](http://www.ncbi.nlm.nih.gov/entrez/query.fcgi?db=gene&cmd=Retrieve&dopt=full_report&list_uids=168090) | chromosome 6 open reading frame 118 |
| [Details](http://mirdb.org/cgi-bin/target_detail.cgi?targetID=2443996) | 87 | 89 | hsa-miR-222-3p | [MARF1](http://www.ncbi.nlm.nih.gov/entrez/query.fcgi?db=gene&cmd=Retrieve&dopt=full_report&list_uids=9665) | meiosis regulator and mRNA stability factor 1 |
| [Details](http://mirdb.org/cgi-bin/target_detail.cgi?targetID=2444408) | 88 | 89 | hsa-miR-222-3p | [BICDL1](http://www.ncbi.nlm.nih.gov/entrez/query.fcgi?db=gene&cmd=Retrieve&dopt=full_report&list_uids=92558) | BICD family like cargo adaptor 1 |
| [Details](http://mirdb.org/cgi-bin/target_detail.cgi?targetID=2444003) | 89 | 89 | hsa-miR-222-3p | [C6](http://www.ncbi.nlm.nih.gov/entrez/query.fcgi?db=gene&cmd=Retrieve&dopt=full_report&list_uids=729) | complement C6 |
| [Details](http://mirdb.org/cgi-bin/target_detail.cgi?targetID=2444060) | 90 | 88 | hsa-miR-222-3p | [HIPK1](http://www.ncbi.nlm.nih.gov/entrez/query.fcgi?db=gene&cmd=Retrieve&dopt=full_report&list_uids=204851) | homeodomain interacting protein kinase 1 |
| [Details](http://mirdb.org/cgi-bin/target_detail.cgi?targetID=2444066) | 91 | 88 | hsa-miR-222-3p | [PLCL2](http://www.ncbi.nlm.nih.gov/entrez/query.fcgi?db=gene&cmd=Retrieve&dopt=full_report&list_uids=23228) | phospholipase C like 2 |
| [Details](http://mirdb.org/cgi-bin/target_detail.cgi?targetID=2444283) | 92 | 88 | hsa-miR-222-3p | [WDR47](http://www.ncbi.nlm.nih.gov/entrez/query.fcgi?db=gene&cmd=Retrieve&dopt=full_report&list_uids=22911) | WD repeat domain 47 |
| [Details](http://mirdb.org/cgi-bin/target_detail.cgi?targetID=2444465) | 93 | 88 | hsa-miR-222-3p | [KDR](http://www.ncbi.nlm.nih.gov/entrez/query.fcgi?db=gene&cmd=Retrieve&dopt=full_report&list_uids=3791) | kinase insert domain receptor |
| [Details](http://mirdb.org/cgi-bin/target_detail.cgi?targetID=2444033) | 94 | 88 | hsa-miR-222-3p | [ZNF615](http://www.ncbi.nlm.nih.gov/entrez/query.fcgi?db=gene&cmd=Retrieve&dopt=full_report&list_uids=284370) | zinc finger protein 615 |
| [Details](http://mirdb.org/cgi-bin/target_detail.cgi?targetID=2444256) | 95 | 88 | hsa-miR-222-3p | [GABRG1](http://www.ncbi.nlm.nih.gov/entrez/query.fcgi?db=gene&cmd=Retrieve&dopt=full_report&list_uids=2565) | gamma-aminobutyric acid type A receptor gamma1 subunit |
| [Details](http://mirdb.org/cgi-bin/target_detail.cgi?targetID=2444120) | 96 | 88 | hsa-miR-222-3p | [FAM214A](http://www.ncbi.nlm.nih.gov/entrez/query.fcgi?db=gene&cmd=Retrieve&dopt=full_report&list_uids=56204) | family with sequence similarity 214 member A |
| [Details](http://mirdb.org/cgi-bin/target_detail.cgi?targetID=2444019) | 97 | 88 | hsa-miR-222-3p | [PIEZO2](http://www.ncbi.nlm.nih.gov/entrez/query.fcgi?db=gene&cmd=Retrieve&dopt=full_report&list_uids=63895) | piezo type mechanosensitive ion channel component 2 |
| [Details](http://mirdb.org/cgi-bin/target_detail.cgi?targetID=2443983) | 98 | 88 | hsa-miR-222-3p | [DPH6](http://www.ncbi.nlm.nih.gov/entrez/query.fcgi?db=gene&cmd=Retrieve&dopt=full_report&list_uids=89978) | diphthamine biosynthesis 6 |
| [Details](http://mirdb.org/cgi-bin/target_detail.cgi?targetID=2444364) | 99 | 88 | hsa-miR-222-3p | [GUCY1A2](http://www.ncbi.nlm.nih.gov/entrez/query.fcgi?db=gene&cmd=Retrieve&dopt=full_report&list_uids=2977) | guanylate cyclase 1 soluble subunit alpha 2 |
| [Details](http://mirdb.org/cgi-bin/target_detail.cgi?targetID=2444241) | 100 | 88 | hsa-miR-222-3p | [CDK19](http://www.ncbi.nlm.nih.gov/entrez/query.fcgi?db=gene&cmd=Retrieve&dopt=full_report&list_uids=23097) | cyclin dependent kinase 19 |
| [Details](http://mirdb.org/cgi-bin/target_detail.cgi?targetID=2444472) | 101 | 87 | hsa-miR-222-3p | [TUB](http://www.ncbi.nlm.nih.gov/entrez/query.fcgi?db=gene&cmd=Retrieve&dopt=full_report&list_uids=7275) | tubby bipartite transcription factor |
| [Details](http://mirdb.org/cgi-bin/target_detail.cgi?targetID=2444137) | 102 | 87 | hsa-miR-222-3p | [CTDSPL2](http://www.ncbi.nlm.nih.gov/entrez/query.fcgi?db=gene&cmd=Retrieve&dopt=full_report&list_uids=51496) | CTD small phosphatase like 2 |
| [Details](http://mirdb.org/cgi-bin/target_detail.cgi?targetID=2444370) | 103 | 87 | hsa-miR-222-3p | [USP27X](http://www.ncbi.nlm.nih.gov/entrez/query.fcgi?db=gene&cmd=Retrieve&dopt=full_report&list_uids=389856) | ubiquitin specific peptidase 27 X-linked |
| [Details](http://mirdb.org/cgi-bin/target_detail.cgi?targetID=2444020) | 104 | 87 | hsa-miR-222-3p | [SYBU](http://www.ncbi.nlm.nih.gov/entrez/query.fcgi?db=gene&cmd=Retrieve&dopt=full_report&list_uids=55638) | syntabulin |
| [Details](http://mirdb.org/cgi-bin/target_detail.cgi?targetID=2444444) | 105 | 87 | hsa-miR-222-3p | [SBK1](http://www.ncbi.nlm.nih.gov/entrez/query.fcgi?db=gene&cmd=Retrieve&dopt=full_report&list_uids=388228) | SH3 domain binding kinase 1 |
| [Details](http://mirdb.org/cgi-bin/target_detail.cgi?targetID=2444316) | 106 | 87 | hsa-miR-222-3p | [ASPA](http://www.ncbi.nlm.nih.gov/entrez/query.fcgi?db=gene&cmd=Retrieve&dopt=full_report&list_uids=443) | aspartoacylase |
| [Details](http://mirdb.org/cgi-bin/target_detail.cgi?targetID=2443965) | 107 | 87 | hsa-miR-222-3p | [ESR1](http://www.ncbi.nlm.nih.gov/entrez/query.fcgi?db=gene&cmd=Retrieve&dopt=full_report&list_uids=2099) | estrogen receptor 1 |
| [Details](http://mirdb.org/cgi-bin/target_detail.cgi?targetID=2444350) | 108 | 87 | hsa-miR-222-3p | [SESN3](http://www.ncbi.nlm.nih.gov/entrez/query.fcgi?db=gene&cmd=Retrieve&dopt=full_report&list_uids=143686) | sestrin 3 |
| [Details](http://mirdb.org/cgi-bin/target_detail.cgi?targetID=2444267) | 109 | 86 | hsa-miR-222-3p | [TSPAN13](http://www.ncbi.nlm.nih.gov/entrez/query.fcgi?db=gene&cmd=Retrieve&dopt=full_report&list_uids=27075) | tetraspanin 13 |
| [Details](http://mirdb.org/cgi-bin/target_detail.cgi?targetID=2444375) | 110 | 86 | hsa-miR-222-3p | [DDIT4](http://www.ncbi.nlm.nih.gov/entrez/query.fcgi?db=gene&cmd=Retrieve&dopt=full_report&list_uids=54541) | DNA damage inducible transcript 4 |
| [Details](http://mirdb.org/cgi-bin/target_detail.cgi?targetID=2444495) | 111 | 86 | hsa-miR-222-3p | [TLE3](http://www.ncbi.nlm.nih.gov/entrez/query.fcgi?db=gene&cmd=Retrieve&dopt=full_report&list_uids=7090) | TLE family member 3, transcriptional corepressor |
| [Details](http://mirdb.org/cgi-bin/target_detail.cgi?targetID=2443992) | 112 | 86 | hsa-miR-222-3p | [SLC4A7](http://www.ncbi.nlm.nih.gov/entrez/query.fcgi?db=gene&cmd=Retrieve&dopt=full_report&list_uids=9497) | solute carrier family 4 member 7 |
| [Details](http://mirdb.org/cgi-bin/target_detail.cgi?targetID=2444063) | 113 | 86 | hsa-miR-222-3p | [CLRN1](http://www.ncbi.nlm.nih.gov/entrez/query.fcgi?db=gene&cmd=Retrieve&dopt=full_report&list_uids=7401) | clarin 1 |
| [Details](http://mirdb.org/cgi-bin/target_detail.cgi?targetID=2444206) | 114 | 86 | hsa-miR-222-3p | [ANKRD12](http://www.ncbi.nlm.nih.gov/entrez/query.fcgi?db=gene&cmd=Retrieve&dopt=full_report&list_uids=23253) | ankyrin repeat domain 12 |
| [Details](http://mirdb.org/cgi-bin/target_detail.cgi?targetID=2444263) | 115 | 86 | hsa-miR-222-3p | [AQP3](http://www.ncbi.nlm.nih.gov/entrez/query.fcgi?db=gene&cmd=Retrieve&dopt=full_report&list_uids=360) | aquaporin 3 (Gill blood group) |
| [Details](http://mirdb.org/cgi-bin/target_detail.cgi?targetID=2444101) | 116 | 86 | hsa-miR-222-3p | [RFX3](http://www.ncbi.nlm.nih.gov/entrez/query.fcgi?db=gene&cmd=Retrieve&dopt=full_report&list_uids=5991) | regulatory factor X3 |
| [Details](http://mirdb.org/cgi-bin/target_detail.cgi?targetID=2444231) | 117 | 86 | hsa-miR-222-3p | [DPP8](http://www.ncbi.nlm.nih.gov/entrez/query.fcgi?db=gene&cmd=Retrieve&dopt=full_report&list_uids=54878) | dipeptidyl peptidase 8 |
| [Details](http://mirdb.org/cgi-bin/target_detail.cgi?targetID=2444099) | 118 | 86 | hsa-miR-222-3p | [EIF3J](http://www.ncbi.nlm.nih.gov/entrez/query.fcgi?db=gene&cmd=Retrieve&dopt=full_report&list_uids=8669) | eukaryotic translation initiation factor 3 subunit J |
| [Details](http://mirdb.org/cgi-bin/target_detail.cgi?targetID=2443956) | 119 | 86 | hsa-miR-222-3p | [ZMYM2](http://www.ncbi.nlm.nih.gov/entrez/query.fcgi?db=gene&cmd=Retrieve&dopt=full_report&list_uids=7750) | zinc finger MYM-type containing 2 |
| [Details](http://mirdb.org/cgi-bin/target_detail.cgi?targetID=2444065) | 120 | 85 | hsa-miR-222-3p | [BEND4](http://www.ncbi.nlm.nih.gov/entrez/query.fcgi?db=gene&cmd=Retrieve&dopt=full_report&list_uids=389206) | BEN domain containing 4 |
| [Details](http://mirdb.org/cgi-bin/target_detail.cgi?targetID=2444377) | 121 | 85 | hsa-miR-222-3p | [PGPEP1L](http://www.ncbi.nlm.nih.gov/entrez/query.fcgi?db=gene&cmd=Retrieve&dopt=full_report&list_uids=145814) | pyroglutamyl-peptidase I like |
| [Details](http://mirdb.org/cgi-bin/target_detail.cgi?targetID=2444535) | 122 | 85 | hsa-miR-222-3p | [LYPLA1](http://www.ncbi.nlm.nih.gov/entrez/query.fcgi?db=gene&cmd=Retrieve&dopt=full_report&list_uids=10434) | lysophospholipase 1 |
| [Details](http://mirdb.org/cgi-bin/target_detail.cgi?targetID=2444383) | 123 | 85 | hsa-miR-222-3p | [CLGN](http://www.ncbi.nlm.nih.gov/entrez/query.fcgi?db=gene&cmd=Retrieve&dopt=full_report&list_uids=1047) | calmegin |
| [Details](http://mirdb.org/cgi-bin/target_detail.cgi?targetID=2444082) | 124 | 85 | hsa-miR-222-3p | [PAF1](http://www.ncbi.nlm.nih.gov/entrez/query.fcgi?db=gene&cmd=Retrieve&dopt=full_report&list_uids=54623) | PAF1 homolog, Paf1/RNA polymerase II complex component |
| [Details](http://mirdb.org/cgi-bin/target_detail.cgi?targetID=2444201) | 125 | 85 | hsa-miR-222-3p | [MIA3](http://www.ncbi.nlm.nih.gov/entrez/query.fcgi?db=gene&cmd=Retrieve&dopt=full_report&list_uids=375056) | MIA SH3 domain ER export factor 3 |
| [Details](http://mirdb.org/cgi-bin/target_detail.cgi?targetID=2444069) | 126 | 85 | hsa-miR-222-3p | [KIAA1841](http://www.ncbi.nlm.nih.gov/entrez/query.fcgi?db=gene&cmd=Retrieve&dopt=full_report&list_uids=84542) | KIAA1841 |
| [Details](http://mirdb.org/cgi-bin/target_detail.cgi?targetID=2443998) | 127 | 85 | hsa-miR-222-3p | [ZFYVE16](http://www.ncbi.nlm.nih.gov/entrez/query.fcgi?db=gene&cmd=Retrieve&dopt=full_report&list_uids=9765) | zinc finger FYVE-type containing 16 |
| [Details](http://mirdb.org/cgi-bin/target_detail.cgi?targetID=2444034) | 128 | 85 | hsa-miR-222-3p | [RNPS1](http://www.ncbi.nlm.nih.gov/entrez/query.fcgi?db=gene&cmd=Retrieve&dopt=full_report&list_uids=10921) | RNA binding protein with serine rich domain 1 |
| [Details](http://mirdb.org/cgi-bin/target_detail.cgi?targetID=2444030) | 129 | 85 | hsa-miR-222-3p | [TRPC3](http://www.ncbi.nlm.nih.gov/entrez/query.fcgi?db=gene&cmd=Retrieve&dopt=full_report&list_uids=7222) | transient receptor potential cation channel subfamily C member 3 |
| [Details](http://mirdb.org/cgi-bin/target_detail.cgi?targetID=2444518) | 130 | 85 | hsa-miR-222-3p | [SEMA3C](http://www.ncbi.nlm.nih.gov/entrez/query.fcgi?db=gene&cmd=Retrieve&dopt=full_report&list_uids=10512) | semaphorin 3C |
| [Details](http://mirdb.org/cgi-bin/target_detail.cgi?targetID=2443994) | 131 | 85 | hsa-miR-222-3p | [IRX5](http://www.ncbi.nlm.nih.gov/entrez/query.fcgi?db=gene&cmd=Retrieve&dopt=full_report&list_uids=10265) | iroquois homeobox 5 |
| [Details](http://mirdb.org/cgi-bin/target_detail.cgi?targetID=2444094) | 132 | 84 | hsa-miR-222-3p | [ZNF385A](http://www.ncbi.nlm.nih.gov/entrez/query.fcgi?db=gene&cmd=Retrieve&dopt=full_report&list_uids=25946) | zinc finger protein 385A |
| [Details](http://mirdb.org/cgi-bin/target_detail.cgi?targetID=2444556) | 133 | 84 | hsa-miR-222-3p | [ZNF181](http://www.ncbi.nlm.nih.gov/entrez/query.fcgi?db=gene&cmd=Retrieve&dopt=full_report&list_uids=339318) | zinc finger protein 181 |
| [Details](http://mirdb.org/cgi-bin/target_detail.cgi?targetID=2444447) | 134 | 84 | hsa-miR-222-3p | [GDF9](http://www.ncbi.nlm.nih.gov/entrez/query.fcgi?db=gene&cmd=Retrieve&dopt=full_report&list_uids=2661) | growth differentiation factor 9 |
| [Details](http://mirdb.org/cgi-bin/target_detail.cgi?targetID=2443950) | 135 | 84 | hsa-miR-222-3p | [L3MBTL1](http://www.ncbi.nlm.nih.gov/entrez/query.fcgi?db=gene&cmd=Retrieve&dopt=full_report&list_uids=26013) | L3MBTL1, histone methyl-lysine binding protein |
| [Details](http://mirdb.org/cgi-bin/target_detail.cgi?targetID=2444294) | 136 | 84 | hsa-miR-222-3p | [AIDA](http://www.ncbi.nlm.nih.gov/entrez/query.fcgi?db=gene&cmd=Retrieve&dopt=full_report&list_uids=64853) | axin interactor, dorsalization associated |
| [Details](http://mirdb.org/cgi-bin/target_detail.cgi?targetID=2444409) | 137 | 84 | hsa-miR-222-3p | [NYAP2](http://www.ncbi.nlm.nih.gov/entrez/query.fcgi?db=gene&cmd=Retrieve&dopt=full_report&list_uids=57624) | neuronal tyrosine-phosphorylated phosphoinositide-3-kinase adaptor 2 |
| [Details](http://mirdb.org/cgi-bin/target_detail.cgi?targetID=2444266) | 138 | 84 | hsa-miR-222-3p | [UBE2J1](http://www.ncbi.nlm.nih.gov/entrez/query.fcgi?db=gene&cmd=Retrieve&dopt=full_report&list_uids=51465) | ubiquitin conjugating enzyme E2 J1 |
| [Details](http://mirdb.org/cgi-bin/target_detail.cgi?targetID=2443981) | 139 | 84 | hsa-miR-222-3p | [SNX4](http://www.ncbi.nlm.nih.gov/entrez/query.fcgi?db=gene&cmd=Retrieve&dopt=full_report&list_uids=8723) | sorting nexin 4 |
| [Details](http://mirdb.org/cgi-bin/target_detail.cgi?targetID=2444126) | 140 | 84 | hsa-miR-222-3p | [NIPAL4](http://www.ncbi.nlm.nih.gov/entrez/query.fcgi?db=gene&cmd=Retrieve&dopt=full_report&list_uids=348938) | NIPA like domain containing 4 |
| [Details](http://mirdb.org/cgi-bin/target_detail.cgi?targetID=2444023) | 141 | 84 | hsa-miR-222-3p | [GPBP1](http://www.ncbi.nlm.nih.gov/entrez/query.fcgi?db=gene&cmd=Retrieve&dopt=full_report&list_uids=65056) | GC-rich promoter binding protein 1 |
| [Details](http://mirdb.org/cgi-bin/target_detail.cgi?targetID=2444423) | 142 | 84 | hsa-miR-222-3p | [TSC22D3](http://www.ncbi.nlm.nih.gov/entrez/query.fcgi?db=gene&cmd=Retrieve&dopt=full_report&list_uids=1831) | TSC22 domain family member 3 |
| [Details](http://mirdb.org/cgi-bin/target_detail.cgi?targetID=2444018) | 143 | 84 | hsa-miR-222-3p | [BEAN1](http://www.ncbi.nlm.nih.gov/entrez/query.fcgi?db=gene&cmd=Retrieve&dopt=full_report&list_uids=146227) | brain expressed associated with NEDD4 1 |
| [Details](http://mirdb.org/cgi-bin/target_detail.cgi?targetID=2444029) | 144 | 84 | hsa-miR-222-3p | [FGF14](http://www.ncbi.nlm.nih.gov/entrez/query.fcgi?db=gene&cmd=Retrieve&dopt=full_report&list_uids=2259) | fibroblast growth factor 14 |
| [Details](http://mirdb.org/cgi-bin/target_detail.cgi?targetID=2444428) | 145 | 84 | hsa-miR-222-3p | [BCL2L11](http://www.ncbi.nlm.nih.gov/entrez/query.fcgi?db=gene&cmd=Retrieve&dopt=full_report&list_uids=10018) | BCL2 like 11 |
| [Details](http://mirdb.org/cgi-bin/target_detail.cgi?targetID=2444279) | 146 | 83 | hsa-miR-222-3p | [ATP1B1](http://www.ncbi.nlm.nih.gov/entrez/query.fcgi?db=gene&cmd=Retrieve&dopt=full_report&list_uids=481) | ATPase Na+/K+ transporting subunit beta 1 |
| [Details](http://mirdb.org/cgi-bin/target_detail.cgi?targetID=2444503) | 147 | 83 | hsa-miR-222-3p | [TUBA1A](http://www.ncbi.nlm.nih.gov/entrez/query.fcgi?db=gene&cmd=Retrieve&dopt=full_report&list_uids=7846) | tubulin alpha 1a |
| [Details](http://mirdb.org/cgi-bin/target_detail.cgi?targetID=2444150) | 148 | 83 | hsa-miR-222-3p | [FXN](http://www.ncbi.nlm.nih.gov/entrez/query.fcgi?db=gene&cmd=Retrieve&dopt=full_report&list_uids=2395) | frataxin |
| [Details](http://mirdb.org/cgi-bin/target_detail.cgi?targetID=2443948) | 149 | 83 | hsa-miR-222-3p | [POLR3E](http://www.ncbi.nlm.nih.gov/entrez/query.fcgi?db=gene&cmd=Retrieve&dopt=full_report&list_uids=55718) | RNA polymerase III subunit E |
| [Details](http://mirdb.org/cgi-bin/target_detail.cgi?targetID=2444536) | 150 | 83 | hsa-miR-222-3p | [RNF4](http://www.ncbi.nlm.nih.gov/entrez/query.fcgi?db=gene&cmd=Retrieve&dopt=full_report&list_uids=6047) | ring finger protein 4 |
| [Details](http://mirdb.org/cgi-bin/target_detail.cgi?targetID=2444203) | 151 | 83 | hsa-miR-222-3p | [BRWD3](http://www.ncbi.nlm.nih.gov/entrez/query.fcgi?db=gene&cmd=Retrieve&dopt=full_report&list_uids=254065) | bromodomain and WD repeat domain containing 3 |
| [Details](http://mirdb.org/cgi-bin/target_detail.cgi?targetID=2444354) | 152 | 83 | hsa-miR-222-3p | [DNAJC6](http://www.ncbi.nlm.nih.gov/entrez/query.fcgi?db=gene&cmd=Retrieve&dopt=full_report&list_uids=9829) | DnaJ heat shock protein family (Hsp40) member C6 |
| [Details](http://mirdb.org/cgi-bin/target_detail.cgi?targetID=2444273) | 153 | 83 | hsa-miR-222-3p | [IRF2](http://www.ncbi.nlm.nih.gov/entrez/query.fcgi?db=gene&cmd=Retrieve&dopt=full_report&list_uids=3660) | interferon regulatory factor 2 |
| [Details](http://mirdb.org/cgi-bin/target_detail.cgi?targetID=2444520) | 154 | 83 | hsa-miR-222-3p | [SNRNP48](http://www.ncbi.nlm.nih.gov/entrez/query.fcgi?db=gene&cmd=Retrieve&dopt=full_report&list_uids=154007) | small nuclear ribonucleoprotein U11/U12 subunit 48 |
| [Details](http://mirdb.org/cgi-bin/target_detail.cgi?targetID=2444537) | 155 | 82 | hsa-miR-222-3p | [PLXNC1](http://www.ncbi.nlm.nih.gov/entrez/query.fcgi?db=gene&cmd=Retrieve&dopt=full_report&list_uids=10154) | plexin C1 |
| [Details](http://mirdb.org/cgi-bin/target_detail.cgi?targetID=2444124) | 156 | 82 | hsa-miR-222-3p | [LBR](http://www.ncbi.nlm.nih.gov/entrez/query.fcgi?db=gene&cmd=Retrieve&dopt=full_report&list_uids=3930) | lamin B receptor |
| [Details](http://mirdb.org/cgi-bin/target_detail.cgi?targetID=2444199) | 157 | 82 | hsa-miR-222-3p | [RDX](http://www.ncbi.nlm.nih.gov/entrez/query.fcgi?db=gene&cmd=Retrieve&dopt=full_report&list_uids=5962) | radixin |
| [Details](http://mirdb.org/cgi-bin/target_detail.cgi?targetID=2444498) | 158 | 82 | hsa-miR-222-3p | [SYNCRIP](http://www.ncbi.nlm.nih.gov/entrez/query.fcgi?db=gene&cmd=Retrieve&dopt=full_report&list_uids=10492) | synaptotagmin binding cytoplasmic RNA interacting protein |
| [Details](http://mirdb.org/cgi-bin/target_detail.cgi?targetID=2444087) | 159 | 82 | hsa-miR-222-3p | [SHLD2](http://www.ncbi.nlm.nih.gov/entrez/query.fcgi?db=gene&cmd=Retrieve&dopt=full_report&list_uids=54537) | shieldin complex subunit 2 |
| [Details](http://mirdb.org/cgi-bin/target_detail.cgi?targetID=2444496) | 160 | 82 | hsa-miR-222-3p | [NAP1L5](http://www.ncbi.nlm.nih.gov/entrez/query.fcgi?db=gene&cmd=Retrieve&dopt=full_report&list_uids=266812) | nucleosome assembly protein 1 like 5 |
| [Details](http://mirdb.org/cgi-bin/target_detail.cgi?targetID=2444091) | 161 | 82 | hsa-miR-222-3p | [BBC3](http://www.ncbi.nlm.nih.gov/entrez/query.fcgi?db=gene&cmd=Retrieve&dopt=full_report&list_uids=27113) | BCL2 binding component 3 |
| [Details](http://mirdb.org/cgi-bin/target_detail.cgi?targetID=2443997) | 162 | 82 | hsa-miR-222-3p | [SLC2A13](http://www.ncbi.nlm.nih.gov/entrez/query.fcgi?db=gene&cmd=Retrieve&dopt=full_report&list_uids=114134) | solute carrier family 2 member 13 |
| [Details](http://mirdb.org/cgi-bin/target_detail.cgi?targetID=2444284) | 163 | 82 | hsa-miR-222-3p | [WNK3](http://www.ncbi.nlm.nih.gov/entrez/query.fcgi?db=gene&cmd=Retrieve&dopt=full_report&list_uids=65267) | WNK lysine deficient protein kinase 3 |
| [Details](http://mirdb.org/cgi-bin/target_detail.cgi?targetID=2444184) | 164 | 81 | hsa-miR-222-3p | [SVIP](http://www.ncbi.nlm.nih.gov/entrez/query.fcgi?db=gene&cmd=Retrieve&dopt=full_report&list_uids=258010) | small VCP interacting protein |
| [Details](http://mirdb.org/cgi-bin/target_detail.cgi?targetID=2444431) | 165 | 81 | hsa-miR-222-3p | [SMARCA5](http://www.ncbi.nlm.nih.gov/entrez/query.fcgi?db=gene&cmd=Retrieve&dopt=full_report&list_uids=8467) | SWI/SNF related, matrix associated, actin dependent regulator of chromatin, subfamily a, member 5 |
| [Details](http://mirdb.org/cgi-bin/target_detail.cgi?targetID=2444459) | 166 | 81 | hsa-miR-222-3p | [TDRP](http://www.ncbi.nlm.nih.gov/entrez/query.fcgi?db=gene&cmd=Retrieve&dopt=full_report&list_uids=157695) | testis development related protein |
| [Details](http://mirdb.org/cgi-bin/target_detail.cgi?targetID=2444415) | 167 | 81 | hsa-miR-222-3p | [INA](http://www.ncbi.nlm.nih.gov/entrez/query.fcgi?db=gene&cmd=Retrieve&dopt=full_report&list_uids=9118) | internexin neuronal intermediate filament protein alpha |
| [Details](http://mirdb.org/cgi-bin/target_detail.cgi?targetID=2444017) | 168 | 81 | hsa-miR-222-3p | [RBP2](http://www.ncbi.nlm.nih.gov/entrez/query.fcgi?db=gene&cmd=Retrieve&dopt=full_report&list_uids=5948) | retinol binding protein 2 |
| [Details](http://mirdb.org/cgi-bin/target_detail.cgi?targetID=2444446) | 169 | 81 | hsa-miR-222-3p | [LUZP2](http://www.ncbi.nlm.nih.gov/entrez/query.fcgi?db=gene&cmd=Retrieve&dopt=full_report&list_uids=338645) | leucine zipper protein 2 |
| [Details](http://mirdb.org/cgi-bin/target_detail.cgi?targetID=2444511) | 170 | 81 | hsa-miR-222-3p | [SNAP29](http://www.ncbi.nlm.nih.gov/entrez/query.fcgi?db=gene&cmd=Retrieve&dopt=full_report&list_uids=9342) | synaptosome associated protein 29 |
| [Details](http://mirdb.org/cgi-bin/target_detail.cgi?targetID=2444344) | 171 | 81 | hsa-miR-222-3p | [AKAP5](http://www.ncbi.nlm.nih.gov/entrez/query.fcgi?db=gene&cmd=Retrieve&dopt=full_report&list_uids=9495) | A-kinase anchoring protein 5 |
| [Details](http://mirdb.org/cgi-bin/target_detail.cgi?targetID=2444311) | 172 | 80 | hsa-miR-222-3p | [SEC24C](http://www.ncbi.nlm.nih.gov/entrez/query.fcgi?db=gene&cmd=Retrieve&dopt=full_report&list_uids=9632) | SEC24 homolog C, COPII coat complex component |
| [Details](http://mirdb.org/cgi-bin/target_detail.cgi?targetID=2444021) | 173 | 80 | hsa-miR-222-3p | [SLC6A4](http://www.ncbi.nlm.nih.gov/entrez/query.fcgi?db=gene&cmd=Retrieve&dopt=full_report&list_uids=6532) | solute carrier family 6 member 4 |
| [Details](http://mirdb.org/cgi-bin/target_detail.cgi?targetID=2444330) | 174 | 80 | hsa-miR-222-3p | [TNRC6C](http://www.ncbi.nlm.nih.gov/entrez/query.fcgi?db=gene&cmd=Retrieve&dopt=full_report&list_uids=57690) | trinucleotide repeat containing 6C |
| [Details](http://mirdb.org/cgi-bin/target_detail.cgi?targetID=2444318) | 175 | 80 | hsa-miR-222-3p | [CASR](http://www.ncbi.nlm.nih.gov/entrez/query.fcgi?db=gene&cmd=Retrieve&dopt=full_report&list_uids=846) | calcium sensing receptor |
| [Details](http://mirdb.org/cgi-bin/target_detail.cgi?targetID=2444100) | 176 | 80 | hsa-miR-222-3p | [RAB1A](http://www.ncbi.nlm.nih.gov/entrez/query.fcgi?db=gene&cmd=Retrieve&dopt=full_report&list_uids=5861) | RAB1A, member RAS oncogene family |
| [Details](http://mirdb.org/cgi-bin/target_detail.cgi?targetID=2443953) | 177 | 80 | hsa-miR-222-3p | [ZFP36L2](http://www.ncbi.nlm.nih.gov/entrez/query.fcgi?db=gene&cmd=Retrieve&dopt=full_report&list_uids=678) | ZFP36 ring finger protein like 2 |
| [Details](http://mirdb.org/cgi-bin/target_detail.cgi?targetID=2444189) | 178 | 80 | hsa-miR-222-3p | [PMEPA1](http://www.ncbi.nlm.nih.gov/entrez/query.fcgi?db=gene&cmd=Retrieve&dopt=full_report&list_uids=56937) | prostate transmembrane protein, androgen induced 1 |
| [Details](http://mirdb.org/cgi-bin/target_detail.cgi?targetID=2444509) | 179 | 80 | hsa-miR-222-3p | [ZNF624](http://www.ncbi.nlm.nih.gov/entrez/query.fcgi?db=gene&cmd=Retrieve&dopt=full_report&list_uids=57547) | zinc finger protein 624 |
| [Details](http://mirdb.org/cgi-bin/target_detail.cgi?targetID=2444319) | 180 | 80 | hsa-miR-222-3p | [HSPA8](http://www.ncbi.nlm.nih.gov/entrez/query.fcgi?db=gene&cmd=Retrieve&dopt=full_report&list_uids=3312) | heat shock protein family A (Hsp70) member 8 |
| [Details](http://mirdb.org/cgi-bin/target_detail.cgi?targetID=2444528) | 181 | 80 | hsa-miR-222-3p | [PRRC2B](http://www.ncbi.nlm.nih.gov/entrez/query.fcgi?db=gene&cmd=Retrieve&dopt=full_report&list_uids=84726) | proline rich coiled-coil 2B |
| [Details](http://mirdb.org/cgi-bin/target_detail.cgi?targetID=2444253) | 182 | 80 | hsa-miR-222-3p | [RALA](http://www.ncbi.nlm.nih.gov/entrez/query.fcgi?db=gene&cmd=Retrieve&dopt=full_report&list_uids=5898) | RAS like proto-oncogene A |
| [Details](http://mirdb.org/cgi-bin/target_detail.cgi?targetID=2444548) | 183 | 79 | hsa-miR-222-3p | [OSTM1](http://www.ncbi.nlm.nih.gov/entrez/query.fcgi?db=gene&cmd=Retrieve&dopt=full_report&list_uids=28962) | osteoclastogenesis associated transmembrane protein 1 |
| [Details](http://mirdb.org/cgi-bin/target_detail.cgi?targetID=2444131) | 184 | 79 | hsa-miR-222-3p | [NTF3](http://www.ncbi.nlm.nih.gov/entrez/query.fcgi?db=gene&cmd=Retrieve&dopt=full_report&list_uids=4908) | neurotrophin 3 |
| [Details](http://mirdb.org/cgi-bin/target_detail.cgi?targetID=2444505) | 185 | 79 | hsa-miR-222-3p | [CMTM4](http://www.ncbi.nlm.nih.gov/entrez/query.fcgi?db=gene&cmd=Retrieve&dopt=full_report&list_uids=146223) | CKLF like MARVEL transmembrane domain containing 4 |
| [Details](http://mirdb.org/cgi-bin/target_detail.cgi?targetID=2444322) | 186 | 79 | hsa-miR-222-3p | [NANOS1](http://www.ncbi.nlm.nih.gov/entrez/query.fcgi?db=gene&cmd=Retrieve&dopt=full_report&list_uids=340719) | nanos C2HC-type zinc finger 1 |
| [Details](http://mirdb.org/cgi-bin/target_detail.cgi?targetID=2444403) | 187 | 79 | hsa-miR-222-3p | [PARP9](http://www.ncbi.nlm.nih.gov/entrez/query.fcgi?db=gene&cmd=Retrieve&dopt=full_report&list_uids=83666) | poly(ADP-ribose) polymerase family member 9 |
| [Details](http://mirdb.org/cgi-bin/target_detail.cgi?targetID=2444202) | 188 | 79 | hsa-miR-222-3p | [AMMECR1](http://www.ncbi.nlm.nih.gov/entrez/query.fcgi?db=gene&cmd=Retrieve&dopt=full_report&list_uids=9949) | Alport syndrome, mental retardation, midface hypoplasia and elliptocytosis chromosomal region gene 1 |
| [Details](http://mirdb.org/cgi-bin/target_detail.cgi?targetID=2444278) | 189 | 79 | hsa-miR-222-3p | [NFYB](http://www.ncbi.nlm.nih.gov/entrez/query.fcgi?db=gene&cmd=Retrieve&dopt=full_report&list_uids=4801) | nuclear transcription factor Y subunit beta |
| [Details](http://mirdb.org/cgi-bin/target_detail.cgi?targetID=2444045) | 190 | 79 | hsa-miR-222-3p | [ANGPTL2](http://www.ncbi.nlm.nih.gov/entrez/query.fcgi?db=gene&cmd=Retrieve&dopt=full_report&list_uids=23452) | angiopoietin like 2 |
| [Details](http://mirdb.org/cgi-bin/target_detail.cgi?targetID=2444386) | 191 | 78 | hsa-miR-222-3p | [ZNF275](http://www.ncbi.nlm.nih.gov/entrez/query.fcgi?db=gene&cmd=Retrieve&dopt=full_report&list_uids=10838) | zinc finger protein 275 |
| [Details](http://mirdb.org/cgi-bin/target_detail.cgi?targetID=2444347) | 192 | 78 | hsa-miR-222-3p | [NFATC2](http://www.ncbi.nlm.nih.gov/entrez/query.fcgi?db=gene&cmd=Retrieve&dopt=full_report&list_uids=4773) | nuclear factor of activated T cells 2 |
| [Details](http://mirdb.org/cgi-bin/target_detail.cgi?targetID=2444243) | 193 | 78 | hsa-miR-222-3p | [DIRAS3](http://www.ncbi.nlm.nih.gov/entrez/query.fcgi?db=gene&cmd=Retrieve&dopt=full_report&list_uids=9077) | DIRAS family GTPase 3 |
| [Details](http://mirdb.org/cgi-bin/target_detail.cgi?targetID=2444543) | 194 | 78 | hsa-miR-222-3p | [NFATC3](http://www.ncbi.nlm.nih.gov/entrez/query.fcgi?db=gene&cmd=Retrieve&dopt=full_report&list_uids=4775) | nuclear factor of activated T cells 3 |
| [Details](http://mirdb.org/cgi-bin/target_detail.cgi?targetID=2444269) | 195 | 78 | hsa-miR-222-3p | [SOX10](http://www.ncbi.nlm.nih.gov/entrez/query.fcgi?db=gene&cmd=Retrieve&dopt=full_report&list_uids=6663) | SRY-box 10 |
| [Details](http://mirdb.org/cgi-bin/target_detail.cgi?targetID=2443968) | 196 | 78 | hsa-miR-222-3p | [LRFN2](http://www.ncbi.nlm.nih.gov/entrez/query.fcgi?db=gene&cmd=Retrieve&dopt=full_report&list_uids=57497) | leucine rich repeat and fibronectin type III domain containing 2 |
| [Details](http://mirdb.org/cgi-bin/target_detail.cgi?targetID=2444478) | 197 | 78 | hsa-miR-222-3p | [CREBL2](http://www.ncbi.nlm.nih.gov/entrez/query.fcgi?db=gene&cmd=Retrieve&dopt=full_report&list_uids=1389) | cAMP responsive element binding protein like 2 |
| [Details](http://mirdb.org/cgi-bin/target_detail.cgi?targetID=2444228) | 198 | 78 | hsa-miR-222-3p | [ADAM17](http://www.ncbi.nlm.nih.gov/entrez/query.fcgi?db=gene&cmd=Retrieve&dopt=full_report&list_uids=6868) | ADAM metallopeptidase domain 17 |
| [Details](http://mirdb.org/cgi-bin/target_detail.cgi?targetID=2444489) | 199 | 78 | hsa-miR-222-3p | [PTPN4](http://www.ncbi.nlm.nih.gov/entrez/query.fcgi?db=gene&cmd=Retrieve&dopt=full_report&list_uids=5775) | protein tyrosine phosphatase, non-receptor type 4 |
| [Details](http://mirdb.org/cgi-bin/target_detail.cgi?targetID=2444129) | 200 | 78 | hsa-miR-222-3p | [ANKRD52](http://www.ncbi.nlm.nih.gov/entrez/query.fcgi?db=gene&cmd=Retrieve&dopt=full_report&list_uids=283373) | ankyrin repeat domain 52 |
| [Details](http://mirdb.org/cgi-bin/target_detail.cgi?targetID=2444164) | 201 | 77 | hsa-miR-222-3p | [BMF](http://www.ncbi.nlm.nih.gov/entrez/query.fcgi?db=gene&cmd=Retrieve&dopt=full_report&list_uids=90427) | Bcl2 modifying factor |
| [Details](http://mirdb.org/cgi-bin/target_detail.cgi?targetID=2444476) | 202 | 77 | hsa-miR-222-3p | [ZBTB5](http://www.ncbi.nlm.nih.gov/entrez/query.fcgi?db=gene&cmd=Retrieve&dopt=full_report&list_uids=9925) | zinc finger and BTB domain containing 5 |
| [Details](http://mirdb.org/cgi-bin/target_detail.cgi?targetID=2444491) | 203 | 77 | hsa-miR-222-3p | [MYBL1](http://www.ncbi.nlm.nih.gov/entrez/query.fcgi?db=gene&cmd=Retrieve&dopt=full_report&list_uids=4603) | MYB proto-oncogene like 1 |
| [Details](http://mirdb.org/cgi-bin/target_detail.cgi?targetID=2444413) | 204 | 77 | hsa-miR-222-3p | [MAP4K5](http://www.ncbi.nlm.nih.gov/entrez/query.fcgi?db=gene&cmd=Retrieve&dopt=full_report&list_uids=11183) | mitogen-activated protein kinase kinase kinase kinase 5 |
| [Details](http://mirdb.org/cgi-bin/target_detail.cgi?targetID=2444527) | 205 | 77 | hsa-miR-222-3p | [KMT2A](http://www.ncbi.nlm.nih.gov/entrez/query.fcgi?db=gene&cmd=Retrieve&dopt=full_report&list_uids=4297) | lysine methyltransferase 2A |
| [Details](http://mirdb.org/cgi-bin/target_detail.cgi?targetID=2444219) | 206 | 77 | hsa-miR-222-3p | [ANXA3](http://www.ncbi.nlm.nih.gov/entrez/query.fcgi?db=gene&cmd=Retrieve&dopt=full_report&list_uids=306) | annexin A3 |
| [Details](http://mirdb.org/cgi-bin/target_detail.cgi?targetID=2444106) | 207 | 77 | hsa-miR-222-3p | [CUX2](http://www.ncbi.nlm.nih.gov/entrez/query.fcgi?db=gene&cmd=Retrieve&dopt=full_report&list_uids=23316) | cut like homeobox 2 |
| [Details](http://mirdb.org/cgi-bin/target_detail.cgi?targetID=2444183) | 208 | 77 | hsa-miR-222-3p | [ZFAND5](http://www.ncbi.nlm.nih.gov/entrez/query.fcgi?db=gene&cmd=Retrieve&dopt=full_report&list_uids=7763) | zinc finger AN1-type containing 5 |
| [Details](http://mirdb.org/cgi-bin/target_detail.cgi?targetID=2444281) | 209 | 77 | hsa-miR-222-3p | [EOGT](http://www.ncbi.nlm.nih.gov/entrez/query.fcgi?db=gene&cmd=Retrieve&dopt=full_report&list_uids=285203) | EGF domain specific O-linked N-acetylglucosamine transferase |
| [Details](http://mirdb.org/cgi-bin/target_detail.cgi?targetID=2444393) | 210 | 77 | hsa-miR-222-3p | [KCNH8](http://www.ncbi.nlm.nih.gov/entrez/query.fcgi?db=gene&cmd=Retrieve&dopt=full_report&list_uids=131096) | potassium voltage-gated channel subfamily H member 8 |
| [Details](http://mirdb.org/cgi-bin/target_detail.cgi?targetID=2444135) | 211 | 77 | hsa-miR-222-3p | [PLCXD3](http://www.ncbi.nlm.nih.gov/entrez/query.fcgi?db=gene&cmd=Retrieve&dopt=full_report&list_uids=345557) | phosphatidylinositol specific phospholipase C X domain containing 3 |
| [Details](http://mirdb.org/cgi-bin/target_detail.cgi?targetID=2444008) | 212 | 77 | hsa-miR-222-3p | [NCKAP5](http://www.ncbi.nlm.nih.gov/entrez/query.fcgi?db=gene&cmd=Retrieve&dopt=full_report&list_uids=344148) | NCK associated protein 5 |
| [Details](http://mirdb.org/cgi-bin/target_detail.cgi?targetID=2444258) | 213 | 77 | hsa-miR-222-3p | [SLC30A6](http://www.ncbi.nlm.nih.gov/entrez/query.fcgi?db=gene&cmd=Retrieve&dopt=full_report&list_uids=55676) | solute carrier family 30 member 6 |
| [Details](http://mirdb.org/cgi-bin/target_detail.cgi?targetID=2444174) | 214 | 77 | hsa-miR-222-3p | [NDST3](http://www.ncbi.nlm.nih.gov/entrez/query.fcgi?db=gene&cmd=Retrieve&dopt=full_report&list_uids=9348) | N-deacetylase and N-sulfotransferase 3 |
| [Details](http://mirdb.org/cgi-bin/target_detail.cgi?targetID=2444488) | 215 | 76 | hsa-miR-222-3p | [ZFP30](http://www.ncbi.nlm.nih.gov/entrez/query.fcgi?db=gene&cmd=Retrieve&dopt=full_report&list_uids=22835) | ZFP30 zinc finger protein |
| [Details](http://mirdb.org/cgi-bin/target_detail.cgi?targetID=2444542) | 216 | 76 | hsa-miR-222-3p | [CLDN11](http://www.ncbi.nlm.nih.gov/entrez/query.fcgi?db=gene&cmd=Retrieve&dopt=full_report&list_uids=5010) | claudin 11 |
| [Details](http://mirdb.org/cgi-bin/target_detail.cgi?targetID=2444406) | 217 | 76 | hsa-miR-222-3p | [ITGB3](http://www.ncbi.nlm.nih.gov/entrez/query.fcgi?db=gene&cmd=Retrieve&dopt=full_report&list_uids=3690) | integrin subunit beta 3 |
| [Details](http://mirdb.org/cgi-bin/target_detail.cgi?targetID=2444080) | 218 | 76 | hsa-miR-222-3p | [GALNT18](http://www.ncbi.nlm.nih.gov/entrez/query.fcgi?db=gene&cmd=Retrieve&dopt=full_report&list_uids=374378) | polypeptide N-acetylgalactosaminyltransferase 18 |
| [Details](http://mirdb.org/cgi-bin/target_detail.cgi?targetID=2444299) | 219 | 76 | hsa-miR-222-3p | [ZNF629](http://www.ncbi.nlm.nih.gov/entrez/query.fcgi?db=gene&cmd=Retrieve&dopt=full_report&list_uids=23361) | zinc finger protein 629 |
| [Details](http://mirdb.org/cgi-bin/target_detail.cgi?targetID=2444089) | 220 | 76 | hsa-miR-222-3p | [TRAF3IP2](http://www.ncbi.nlm.nih.gov/entrez/query.fcgi?db=gene&cmd=Retrieve&dopt=full_report&list_uids=10758) | TRAF3 interacting protein 2 |
| [Details](http://mirdb.org/cgi-bin/target_detail.cgi?targetID=2444307) | 221 | 76 | hsa-miR-222-3p | [SNCB](http://www.ncbi.nlm.nih.gov/entrez/query.fcgi?db=gene&cmd=Retrieve&dopt=full_report&list_uids=6620) | synuclein beta |
| [Details](http://mirdb.org/cgi-bin/target_detail.cgi?targetID=2443944) | 222 | 76 | hsa-miR-222-3p | [FRY](http://www.ncbi.nlm.nih.gov/entrez/query.fcgi?db=gene&cmd=Retrieve&dopt=full_report&list_uids=10129) | FRY microtubule binding protein |
| [Details](http://mirdb.org/cgi-bin/target_detail.cgi?targetID=2444462) | 223 | 76 | hsa-miR-222-3p | [MEGF9](http://www.ncbi.nlm.nih.gov/entrez/query.fcgi?db=gene&cmd=Retrieve&dopt=full_report&list_uids=1955) | multiple EGF like domains 9 |
| [Details](http://mirdb.org/cgi-bin/target_detail.cgi?targetID=2444058) | 224 | 76 | hsa-miR-222-3p | [PRICKLE2](http://www.ncbi.nlm.nih.gov/entrez/query.fcgi?db=gene&cmd=Retrieve&dopt=full_report&list_uids=166336) | prickle planar cell polarity protein 2 |
| [Details](http://mirdb.org/cgi-bin/target_detail.cgi?targetID=2444302) | 225 | 76 | hsa-miR-222-3p | [ATAD2B](http://www.ncbi.nlm.nih.gov/entrez/query.fcgi?db=gene&cmd=Retrieve&dopt=full_report&list_uids=54454) | ATPase family, AAA domain containing 2B |
| [Details](http://mirdb.org/cgi-bin/target_detail.cgi?targetID=2444107) | 226 | 76 | hsa-miR-222-3p | [CNR1](http://www.ncbi.nlm.nih.gov/entrez/query.fcgi?db=gene&cmd=Retrieve&dopt=full_report&list_uids=1268) | cannabinoid receptor 1 |
| [Details](http://mirdb.org/cgi-bin/target_detail.cgi?targetID=2443963) | 227 | 76 | hsa-miR-222-3p | [PPDPFL](http://www.ncbi.nlm.nih.gov/entrez/query.fcgi?db=gene&cmd=Retrieve&dopt=full_report&list_uids=492307) | pancreatic progenitor cell differentiation and proliferation factor like |
| [Details](http://mirdb.org/cgi-bin/target_detail.cgi?targetID=2444545) | 228 | 76 | hsa-miR-222-3p | [PRDM1](http://www.ncbi.nlm.nih.gov/entrez/query.fcgi?db=gene&cmd=Retrieve&dopt=full_report&list_uids=639) | PR/SET domain 1 |
| [Details](http://mirdb.org/cgi-bin/target_detail.cgi?targetID=2444233) | 229 | 76 | hsa-miR-222-3p | [PLPPR1](http://www.ncbi.nlm.nih.gov/entrez/query.fcgi?db=gene&cmd=Retrieve&dopt=full_report&list_uids=54886) | phospholipid phosphatase related 1 |
| [Details](http://mirdb.org/cgi-bin/target_detail.cgi?targetID=2444506) | 230 | 76 | hsa-miR-222-3p | [CPEB3](http://www.ncbi.nlm.nih.gov/entrez/query.fcgi?db=gene&cmd=Retrieve&dopt=full_report&list_uids=22849) | cytoplasmic polyadenylation element binding protein 3 |
| [Details](http://mirdb.org/cgi-bin/target_detail.cgi?targetID=2444207) | 231 | 75 | hsa-miR-222-3p | [XIRP2](http://www.ncbi.nlm.nih.gov/entrez/query.fcgi?db=gene&cmd=Retrieve&dopt=full_report&list_uids=129446) | xin actin binding repeat containing 2 |
| [Details](http://mirdb.org/cgi-bin/target_detail.cgi?targetID=2444372) | 232 | 75 | hsa-miR-222-3p | [PCDHA4](http://www.ncbi.nlm.nih.gov/entrez/query.fcgi?db=gene&cmd=Retrieve&dopt=full_report&list_uids=56144) | protocadherin alpha 4 |
| [Details](http://mirdb.org/cgi-bin/target_detail.cgi?targetID=2444191) | 233 | 75 | hsa-miR-222-3p | [PAIP2](http://www.ncbi.nlm.nih.gov/entrez/query.fcgi?db=gene&cmd=Retrieve&dopt=full_report&list_uids=51247) | poly(A) binding protein interacting protein 2 |
| [Details](http://mirdb.org/cgi-bin/target_detail.cgi?targetID=2443971) | 234 | 75 | hsa-miR-222-3p | [PCDHA8](http://www.ncbi.nlm.nih.gov/entrez/query.fcgi?db=gene&cmd=Retrieve&dopt=full_report&list_uids=56140) | protocadherin alpha 8 |
| [Details](http://mirdb.org/cgi-bin/target_detail.cgi?targetID=2444473) | 235 | 75 | hsa-miR-222-3p | [PCDHA2](http://www.ncbi.nlm.nih.gov/entrez/query.fcgi?db=gene&cmd=Retrieve&dopt=full_report&list_uids=56146) | protocadherin alpha 2 |
| [Details](http://mirdb.org/cgi-bin/target_detail.cgi?targetID=2444070) | 236 | 75 | hsa-miR-222-3p | [PCDHAC2](http://www.ncbi.nlm.nih.gov/entrez/query.fcgi?db=gene&cmd=Retrieve&dopt=full_report&list_uids=56134) | protocadherin alpha subfamily C, 2 |
| [Details](http://mirdb.org/cgi-bin/target_detail.cgi?targetID=2444212) | 237 | 75 | hsa-miR-222-3p | [FBXO47](http://www.ncbi.nlm.nih.gov/entrez/query.fcgi?db=gene&cmd=Retrieve&dopt=full_report&list_uids=494188) | F-box protein 47 |
| [Details](http://mirdb.org/cgi-bin/target_detail.cgi?targetID=2444467) | 238 | 75 | hsa-miR-222-3p | [PCDHA7](http://www.ncbi.nlm.nih.gov/entrez/query.fcgi?db=gene&cmd=Retrieve&dopt=full_report&list_uids=56141) | protocadherin alpha 7 |
| [Details](http://mirdb.org/cgi-bin/target_detail.cgi?targetID=2444217) | 239 | 75 | hsa-miR-222-3p | [PCDHA11](http://www.ncbi.nlm.nih.gov/entrez/query.fcgi?db=gene&cmd=Retrieve&dopt=full_report&list_uids=56138) | protocadherin alpha 11 |
| [Details](http://mirdb.org/cgi-bin/target_detail.cgi?targetID=2443972) | 240 | 75 | hsa-miR-222-3p | [PCDHAC1](http://www.ncbi.nlm.nih.gov/entrez/query.fcgi?db=gene&cmd=Retrieve&dopt=full_report&list_uids=56135) | protocadherin alpha subfamily C, 1 |
| [Details](http://mirdb.org/cgi-bin/target_detail.cgi?targetID=2444475) | 241 | 75 | hsa-miR-222-3p | [C6orf120](http://www.ncbi.nlm.nih.gov/entrez/query.fcgi?db=gene&cmd=Retrieve&dopt=full_report&list_uids=387263) | chromosome 6 open reading frame 120 |
| [Details](http://mirdb.org/cgi-bin/target_detail.cgi?targetID=2444073) | 242 | 75 | hsa-miR-222-3p | [PCDHA13](http://www.ncbi.nlm.nih.gov/entrez/query.fcgi?db=gene&cmd=Retrieve&dopt=full_report&list_uids=56136) | protocadherin alpha 13 |
| [Details](http://mirdb.org/cgi-bin/target_detail.cgi?targetID=2444188) | 243 | 75 | hsa-miR-222-3p | [PCDHA6](http://www.ncbi.nlm.nih.gov/entrez/query.fcgi?db=gene&cmd=Retrieve&dopt=full_report&list_uids=56142) | protocadherin alpha 6 |
| [Details](http://mirdb.org/cgi-bin/target_detail.cgi?targetID=2444282) | 244 | 75 | hsa-miR-222-3p | [ZNF704](http://www.ncbi.nlm.nih.gov/entrez/query.fcgi?db=gene&cmd=Retrieve&dopt=full_report&list_uids=619279) | zinc finger protein 704 |
| [Details](http://mirdb.org/cgi-bin/target_detail.cgi?targetID=2444376) | 245 | 75 | hsa-miR-222-3p | [C11orf87](http://www.ncbi.nlm.nih.gov/entrez/query.fcgi?db=gene&cmd=Retrieve&dopt=full_report&list_uids=399947) | chromosome 11 open reading frame 87 |
| [Details](http://mirdb.org/cgi-bin/target_detail.cgi?targetID=2443959) | 246 | 75 | hsa-miR-222-3p | [MAGI1](http://www.ncbi.nlm.nih.gov/entrez/query.fcgi?db=gene&cmd=Retrieve&dopt=full_report&list_uids=9223) | membrane associated guanylate kinase, WW and PDZ domain containing 1 |
| [Details](http://mirdb.org/cgi-bin/target_detail.cgi?targetID=2443954) | 247 | 75 | hsa-miR-222-3p | [ZKSCAN8](http://www.ncbi.nlm.nih.gov/entrez/query.fcgi?db=gene&cmd=Retrieve&dopt=full_report&list_uids=7745) | zinc finger with KRAB and SCAN domains 8 |
| [Details](http://mirdb.org/cgi-bin/target_detail.cgi?targetID=2444310) | 248 | 75 | hsa-miR-222-3p | [DCUN1D4](http://www.ncbi.nlm.nih.gov/entrez/query.fcgi?db=gene&cmd=Retrieve&dopt=full_report&list_uids=23142) | defective in cullin neddylation 1 domain containing 4 |
| [Details](http://mirdb.org/cgi-bin/target_detail.cgi?targetID=2444407) | 249 | 75 | hsa-miR-222-3p | [PCDHA1](http://www.ncbi.nlm.nih.gov/entrez/query.fcgi?db=gene&cmd=Retrieve&dopt=full_report&list_uids=56147) | protocadherin alpha 1 |
| [Details](http://mirdb.org/cgi-bin/target_detail.cgi?targetID=2444508) | 250 | 75 | hsa-miR-222-3p | [GBX2](http://www.ncbi.nlm.nih.gov/entrez/query.fcgi?db=gene&cmd=Retrieve&dopt=full_report&list_uids=2637) | gastrulation brain homeobox 2 |
| [Details](http://mirdb.org/cgi-bin/target_detail.cgi?targetID=2444002) | 251 | 75 | hsa-miR-222-3p | [PPARGC1A](http://www.ncbi.nlm.nih.gov/entrez/query.fcgi?db=gene&cmd=Retrieve&dopt=full_report&list_uids=10891) | PPARG coactivator 1 alpha |
| [Details](http://mirdb.org/cgi-bin/target_detail.cgi?targetID=2444147) | 252 | 75 | hsa-miR-222-3p | [PCDHA5](http://www.ncbi.nlm.nih.gov/entrez/query.fcgi?db=gene&cmd=Retrieve&dopt=full_report&list_uids=56143) | protocadherin alpha 5 |
| [Details](http://mirdb.org/cgi-bin/target_detail.cgi?targetID=2444513) | 253 | 75 | hsa-miR-222-3p | [PCDHA12](http://www.ncbi.nlm.nih.gov/entrez/query.fcgi?db=gene&cmd=Retrieve&dopt=full_report&list_uids=56137) | protocadherin alpha 12 |
| [Details](http://mirdb.org/cgi-bin/target_detail.cgi?targetID=2443970) | 254 | 75 | hsa-miR-222-3p | [CPNE8](http://www.ncbi.nlm.nih.gov/entrez/query.fcgi?db=gene&cmd=Retrieve&dopt=full_report&list_uids=144402) | copine 8 |
| [Details](http://mirdb.org/cgi-bin/target_detail.cgi?targetID=2444426) | 255 | 75 | hsa-miR-222-3p | [CBFB](http://www.ncbi.nlm.nih.gov/entrez/query.fcgi?db=gene&cmd=Retrieve&dopt=full_report&list_uids=865) | core-binding factor subunit beta |
| [Details](http://mirdb.org/cgi-bin/target_detail.cgi?targetID=2444246) | 256 | 75 | hsa-miR-222-3p | [PCDHA3](http://www.ncbi.nlm.nih.gov/entrez/query.fcgi?db=gene&cmd=Retrieve&dopt=full_report&list_uids=56145) | protocadherin alpha 3 |
| [Details](http://mirdb.org/cgi-bin/target_detail.cgi?targetID=2444552) | 257 | 75 | hsa-miR-222-3p | [DBT](http://www.ncbi.nlm.nih.gov/entrez/query.fcgi?db=gene&cmd=Retrieve&dopt=full_report&list_uids=1629) | dihydrolipoamide branched chain transacylase E2 |
| [Details](http://mirdb.org/cgi-bin/target_detail.cgi?targetID=2444140) | 258 | 75 | hsa-miR-222-3p | [PCDHA10](http://www.ncbi.nlm.nih.gov/entrez/query.fcgi?db=gene&cmd=Retrieve&dopt=full_report&list_uids=56139) | protocadherin alpha 10 |
| [Details](http://mirdb.org/cgi-bin/target_detail.cgi?targetID=2444121) | 259 | 74 | hsa-miR-222-3p | [SCD5](http://www.ncbi.nlm.nih.gov/entrez/query.fcgi?db=gene&cmd=Retrieve&dopt=full_report&list_uids=79966) | stearoyl-CoA desaturase 5 |
| [Details](http://mirdb.org/cgi-bin/target_detail.cgi?targetID=2444111) | 260 | 74 | hsa-miR-222-3p | [VGLL4](http://www.ncbi.nlm.nih.gov/entrez/query.fcgi?db=gene&cmd=Retrieve&dopt=full_report&list_uids=9686) | vestigial like family member 4 |
| [Details](http://mirdb.org/cgi-bin/target_detail.cgi?targetID=2444504) | 261 | 74 | hsa-miR-222-3p | [ABHD3](http://www.ncbi.nlm.nih.gov/entrez/query.fcgi?db=gene&cmd=Retrieve&dopt=full_report&list_uids=171586) | abhydrolase domain containing 3 |
| [Details](http://mirdb.org/cgi-bin/target_detail.cgi?targetID=2444105) | 262 | 74 | hsa-miR-222-3p | [SRD5A3](http://www.ncbi.nlm.nih.gov/entrez/query.fcgi?db=gene&cmd=Retrieve&dopt=full_report&list_uids=79644) | steroid 5 alpha-reductase 3 |
| [Details](http://mirdb.org/cgi-bin/target_detail.cgi?targetID=2444440) | 263 | 74 | hsa-miR-222-3p | [RORB](http://www.ncbi.nlm.nih.gov/entrez/query.fcgi?db=gene&cmd=Retrieve&dopt=full_report&list_uids=6096) | RAR related orphan receptor B |
| [Details](http://mirdb.org/cgi-bin/target_detail.cgi?targetID=2444455) | 264 | 74 | hsa-miR-222-3p | [RALGAPA1](http://www.ncbi.nlm.nih.gov/entrez/query.fcgi?db=gene&cmd=Retrieve&dopt=full_report&list_uids=253959) | Ral GTPase activating protein catalytic alpha subunit 1 |
| [Details](http://mirdb.org/cgi-bin/target_detail.cgi?targetID=2443973) | 265 | 73 | hsa-miR-222-3p | [MRPS7](http://www.ncbi.nlm.nih.gov/entrez/query.fcgi?db=gene&cmd=Retrieve&dopt=full_report&list_uids=51081) | mitochondrial ribosomal protein S7 |
| [Details](http://mirdb.org/cgi-bin/target_detail.cgi?targetID=2444308) | 266 | 73 | hsa-miR-222-3p | [ZNF93](http://www.ncbi.nlm.nih.gov/entrez/query.fcgi?db=gene&cmd=Retrieve&dopt=full_report&list_uids=81931) | zinc finger protein 93 |
| [Details](http://mirdb.org/cgi-bin/target_detail.cgi?targetID=2444230) | 267 | 73 | hsa-miR-222-3p | [TGOLN2](http://www.ncbi.nlm.nih.gov/entrez/query.fcgi?db=gene&cmd=Retrieve&dopt=full_report&list_uids=10618) | trans-golgi network protein 2 |
| [Details](http://mirdb.org/cgi-bin/target_detail.cgi?targetID=2444333) | 268 | 73 | hsa-miR-222-3p | [ABCA8](http://www.ncbi.nlm.nih.gov/entrez/query.fcgi?db=gene&cmd=Retrieve&dopt=full_report&list_uids=10351) | ATP binding cassette subfamily A member 8 |
| [Details](http://mirdb.org/cgi-bin/target_detail.cgi?targetID=2444336) | 269 | 73 | hsa-miR-222-3p | [SGIP1](http://www.ncbi.nlm.nih.gov/entrez/query.fcgi?db=gene&cmd=Retrieve&dopt=full_report&list_uids=84251) | SH3 domain GRB2 like endophilin interacting protein 1 |
| [Details](http://mirdb.org/cgi-bin/target_detail.cgi?targetID=2444224) | 270 | 73 | hsa-miR-222-3p | [CLDND1](http://www.ncbi.nlm.nih.gov/entrez/query.fcgi?db=gene&cmd=Retrieve&dopt=full_report&list_uids=56650) | claudin domain containing 1 |
| [Details](http://mirdb.org/cgi-bin/target_detail.cgi?targetID=2444251) | 271 | 73 | hsa-miR-222-3p | [SYCE2](http://www.ncbi.nlm.nih.gov/entrez/query.fcgi?db=gene&cmd=Retrieve&dopt=full_report&list_uids=256126) | synaptonemal complex central element protein 2 |
| [Details](http://mirdb.org/cgi-bin/target_detail.cgi?targetID=2444433) | 272 | 73 | hsa-miR-222-3p | [YWHAG](http://www.ncbi.nlm.nih.gov/entrez/query.fcgi?db=gene&cmd=Retrieve&dopt=full_report&list_uids=7532) | tyrosine 3-monooxygenase/tryptophan 5-monooxygenase activation protein gamma |
| [Details](http://mirdb.org/cgi-bin/target_detail.cgi?targetID=2444454) | 273 | 73 | hsa-miR-222-3p | [PDIK1L](http://www.ncbi.nlm.nih.gov/entrez/query.fcgi?db=gene&cmd=Retrieve&dopt=full_report&list_uids=149420) | PDLIM1 interacting kinase 1 like |
| [Details](http://mirdb.org/cgi-bin/target_detail.cgi?targetID=2444185) | 274 | 73 | hsa-miR-222-3p | [WSB2](http://www.ncbi.nlm.nih.gov/entrez/query.fcgi?db=gene&cmd=Retrieve&dopt=full_report&list_uids=55884) | WD repeat and SOCS box containing 2 |
| [Details](http://mirdb.org/cgi-bin/target_detail.cgi?targetID=2444411) | 275 | 73 | hsa-miR-222-3p | [KLC1](http://www.ncbi.nlm.nih.gov/entrez/query.fcgi?db=gene&cmd=Retrieve&dopt=full_report&list_uids=3831) | kinesin light chain 1 |
| [Details](http://mirdb.org/cgi-bin/target_detail.cgi?targetID=2444366) | 276 | 73 | hsa-miR-222-3p | [FANCD2](http://www.ncbi.nlm.nih.gov/entrez/query.fcgi?db=gene&cmd=Retrieve&dopt=full_report&list_uids=2177) | FA complementation group D2 |
| [Details](http://mirdb.org/cgi-bin/target_detail.cgi?targetID=2444068) | 277 | 73 | hsa-miR-222-3p | [SPART](http://www.ncbi.nlm.nih.gov/entrez/query.fcgi?db=gene&cmd=Retrieve&dopt=full_report&list_uids=23111) | spartin |
| [Details](http://mirdb.org/cgi-bin/target_detail.cgi?targetID=2444200) | 278 | 73 | hsa-miR-222-3p | [ARAP2](http://www.ncbi.nlm.nih.gov/entrez/query.fcgi?db=gene&cmd=Retrieve&dopt=full_report&list_uids=116984) | ArfGAP with RhoGAP domain, ankyrin repeat and PH domain 2 |
| [Details](http://mirdb.org/cgi-bin/target_detail.cgi?targetID=2444232) | 279 | 72 | hsa-miR-222-3p | [ZFHX3](http://www.ncbi.nlm.nih.gov/entrez/query.fcgi?db=gene&cmd=Retrieve&dopt=full_report&list_uids=463) | zinc finger homeobox 3 |
| [Details](http://mirdb.org/cgi-bin/target_detail.cgi?targetID=2444435) | 280 | 72 | hsa-miR-222-3p | [PLSCR4](http://www.ncbi.nlm.nih.gov/entrez/query.fcgi?db=gene&cmd=Retrieve&dopt=full_report&list_uids=57088) | phospholipid scramblase 4 |
| [Details](http://mirdb.org/cgi-bin/target_detail.cgi?targetID=2444240) | 281 | 72 | hsa-miR-222-3p | [AGPS](http://www.ncbi.nlm.nih.gov/entrez/query.fcgi?db=gene&cmd=Retrieve&dopt=full_report&list_uids=8540) | alkylglycerone phosphate synthase |
| [Details](http://mirdb.org/cgi-bin/target_detail.cgi?targetID=2444028) | 282 | 72 | hsa-miR-222-3p | [ONECUT2](http://www.ncbi.nlm.nih.gov/entrez/query.fcgi?db=gene&cmd=Retrieve&dopt=full_report&list_uids=9480) | one cut homeobox 2 |
| [Details](http://mirdb.org/cgi-bin/target_detail.cgi?targetID=2444138) | 283 | 72 | hsa-miR-222-3p | [PDS5A](http://www.ncbi.nlm.nih.gov/entrez/query.fcgi?db=gene&cmd=Retrieve&dopt=full_report&list_uids=23244) | PDS5 cohesin associated factor A |
| [Details](http://mirdb.org/cgi-bin/target_detail.cgi?targetID=2444154) | 284 | 72 | hsa-miR-222-3p | [CAMTA1](http://www.ncbi.nlm.nih.gov/entrez/query.fcgi?db=gene&cmd=Retrieve&dopt=full_report&list_uids=23261) | calmodulin binding transcription activator 1 |
| [Details](http://mirdb.org/cgi-bin/target_detail.cgi?targetID=2444136) | 285 | 72 | hsa-miR-222-3p | [TLNRD1](http://www.ncbi.nlm.nih.gov/entrez/query.fcgi?db=gene&cmd=Retrieve&dopt=full_report&list_uids=59274) | talin rod domain containing 1 |
| [Details](http://mirdb.org/cgi-bin/target_detail.cgi?targetID=2444425) | 286 | 72 | hsa-miR-222-3p | [MAN2A1](http://www.ncbi.nlm.nih.gov/entrez/query.fcgi?db=gene&cmd=Retrieve&dopt=full_report&list_uids=4124) | mannosidase alpha class 2A member 1 |
| [Details](http://mirdb.org/cgi-bin/target_detail.cgi?targetID=2444110) | 287 | 72 | hsa-miR-222-3p | [ZNF74](http://www.ncbi.nlm.nih.gov/entrez/query.fcgi?db=gene&cmd=Retrieve&dopt=full_report&list_uids=7625) | zinc finger protein 74 |
| [Details](http://mirdb.org/cgi-bin/target_detail.cgi?targetID=2444142) | 288 | 72 | hsa-miR-222-3p | [EVI2A](http://www.ncbi.nlm.nih.gov/entrez/query.fcgi?db=gene&cmd=Retrieve&dopt=full_report&list_uids=2123) | ecotropic viral integration site 2A |
| [Details](http://mirdb.org/cgi-bin/target_detail.cgi?targetID=2444434) | 289 | 72 | hsa-miR-222-3p | [DYNC1LI2](http://www.ncbi.nlm.nih.gov/entrez/query.fcgi?db=gene&cmd=Retrieve&dopt=full_report&list_uids=1783) | dynein cytoplasmic 1 light intermediate chain 2 |
| [Details](http://mirdb.org/cgi-bin/target_detail.cgi?targetID=2444470) | 290 | 72 | hsa-miR-222-3p | [GAB1](http://www.ncbi.nlm.nih.gov/entrez/query.fcgi?db=gene&cmd=Retrieve&dopt=full_report&list_uids=2549) | GRB2 associated binding protein 1 |
| [Details](http://mirdb.org/cgi-bin/target_detail.cgi?targetID=2443939) | 291 | 72 | hsa-miR-222-3p | [TBXT](http://www.ncbi.nlm.nih.gov/entrez/query.fcgi?db=gene&cmd=Retrieve&dopt=full_report&list_uids=6862) | T-box transcription factor T |
| [Details](http://mirdb.org/cgi-bin/target_detail.cgi?targetID=2444526) | 292 | 72 | hsa-miR-222-3p | [NIPBL](http://www.ncbi.nlm.nih.gov/entrez/query.fcgi?db=gene&cmd=Retrieve&dopt=full_report&list_uids=25836) | NIPBL, cohesin loading factor |
| [Details](http://mirdb.org/cgi-bin/target_detail.cgi?targetID=2444453) | 293 | 72 | hsa-miR-222-3p | [SERPINB2](http://www.ncbi.nlm.nih.gov/entrez/query.fcgi?db=gene&cmd=Retrieve&dopt=full_report&list_uids=5055) | serpin family B member 2 |
| [Details](http://mirdb.org/cgi-bin/target_detail.cgi?targetID=2444298) | 294 | 72 | hsa-miR-222-3p | [GTF2E1](http://www.ncbi.nlm.nih.gov/entrez/query.fcgi?db=gene&cmd=Retrieve&dopt=full_report&list_uids=2960) | general transcription factor IIE subunit 1 |
| [Details](http://mirdb.org/cgi-bin/target_detail.cgi?targetID=2444043) | 295 | 72 | hsa-miR-222-3p | [NOVA1](http://www.ncbi.nlm.nih.gov/entrez/query.fcgi?db=gene&cmd=Retrieve&dopt=full_report&list_uids=4857) | NOVA alternative splicing regulator 1 |
| [Details](http://mirdb.org/cgi-bin/target_detail.cgi?targetID=2443943) | 296 | 71 | hsa-miR-222-3p | [ZNF25](http://www.ncbi.nlm.nih.gov/entrez/query.fcgi?db=gene&cmd=Retrieve&dopt=full_report&list_uids=219749) | zinc finger protein 25 |
| [Details](http://mirdb.org/cgi-bin/target_detail.cgi?targetID=2444054) | 297 | 71 | hsa-miR-222-3p | [CYLD](http://www.ncbi.nlm.nih.gov/entrez/query.fcgi?db=gene&cmd=Retrieve&dopt=full_report&list_uids=1540) | CYLD lysine 63 deubiquitinase |
| [Details](http://mirdb.org/cgi-bin/target_detail.cgi?targetID=2444477) | 298 | 71 | hsa-miR-222-3p | [LRRCC1](http://www.ncbi.nlm.nih.gov/entrez/query.fcgi?db=gene&cmd=Retrieve&dopt=full_report&list_uids=85444) | leucine rich repeat and coiled-coil centrosomal protein 1 |
| [Details](http://mirdb.org/cgi-bin/target_detail.cgi?targetID=2444134) | 299 | 71 | hsa-miR-222-3p | [PDLIM2](http://www.ncbi.nlm.nih.gov/entrez/query.fcgi?db=gene&cmd=Retrieve&dopt=full_report&list_uids=64236) | PDZ and LIM domain 2 |
| [Details](http://mirdb.org/cgi-bin/target_detail.cgi?targetID=2444193) | 300 | 71 | hsa-miR-222-3p | [CDKN2AIP](http://www.ncbi.nlm.nih.gov/entrez/query.fcgi?db=gene&cmd=Retrieve&dopt=full_report&list_uids=55602) | CDKN2A interacting protein |
| [Details](http://mirdb.org/cgi-bin/target_detail.cgi?targetID=2444038) | 301 | 71 | hsa-miR-222-3p | [RBM24](http://www.ncbi.nlm.nih.gov/entrez/query.fcgi?db=gene&cmd=Retrieve&dopt=full_report&list_uids=221662) | RNA binding motif protein 24 |
| [Details](http://mirdb.org/cgi-bin/target_detail.cgi?targetID=2444016) | 302 | 71 | hsa-miR-222-3p | [ARHGEF7](http://www.ncbi.nlm.nih.gov/entrez/query.fcgi?db=gene&cmd=Retrieve&dopt=full_report&list_uids=8874) | Rho guanine nucleotide exchange factor 7 |
| [Details](http://mirdb.org/cgi-bin/target_detail.cgi?targetID=2444514) | 303 | 71 | hsa-miR-222-3p | [ENAH](http://www.ncbi.nlm.nih.gov/entrez/query.fcgi?db=gene&cmd=Retrieve&dopt=full_report&list_uids=55740) | ENAH, actin regulator |
| [Details](http://mirdb.org/cgi-bin/target_detail.cgi?targetID=2444405) | 304 | 71 | hsa-miR-222-3p | [ITPR2](http://www.ncbi.nlm.nih.gov/entrez/query.fcgi?db=gene&cmd=Retrieve&dopt=full_report&list_uids=3709) | inositol 1,4,5-trisphosphate receptor type 2 |
| [Details](http://mirdb.org/cgi-bin/target_detail.cgi?targetID=2444410) | 305 | 71 | hsa-miR-222-3p | [KPNA2](http://www.ncbi.nlm.nih.gov/entrez/query.fcgi?db=gene&cmd=Retrieve&dopt=full_report&list_uids=3838) | karyopherin subunit alpha 2 |
| [Details](http://mirdb.org/cgi-bin/target_detail.cgi?targetID=2444355) | 306 | 71 | hsa-miR-222-3p | [KIAA0586](http://www.ncbi.nlm.nih.gov/entrez/query.fcgi?db=gene&cmd=Retrieve&dopt=full_report&list_uids=9786) | KIAA0586 |
| [Details](http://mirdb.org/cgi-bin/target_detail.cgi?targetID=2444171) | 307 | 71 | hsa-miR-222-3p | [CREBZF](http://www.ncbi.nlm.nih.gov/entrez/query.fcgi?db=gene&cmd=Retrieve&dopt=full_report&list_uids=58487) | CREB/ATF bZIP transcription factor |
| [Details](http://mirdb.org/cgi-bin/target_detail.cgi?targetID=2444271) | 308 | 70 | hsa-miR-222-3p | [TRPS1](http://www.ncbi.nlm.nih.gov/entrez/query.fcgi?db=gene&cmd=Retrieve&dopt=full_report&list_uids=7227) | transcriptional repressor GATA binding 1 |
| [Details](http://mirdb.org/cgi-bin/target_detail.cgi?targetID=2443979) | 309 | 70 | hsa-miR-222-3p | [CLIC2](http://www.ncbi.nlm.nih.gov/entrez/query.fcgi?db=gene&cmd=Retrieve&dopt=full_report&list_uids=1193) | chloride intracellular channel 2 |
| [Details](http://mirdb.org/cgi-bin/target_detail.cgi?targetID=2444223) | 310 | 70 | hsa-miR-222-3p | [IL17RB](http://www.ncbi.nlm.nih.gov/entrez/query.fcgi?db=gene&cmd=Retrieve&dopt=full_report&list_uids=55540) | interleukin 17 receptor B |
| [Details](http://mirdb.org/cgi-bin/target_detail.cgi?targetID=2444235) | 311 | 70 | hsa-miR-222-3p | [TOX](http://www.ncbi.nlm.nih.gov/entrez/query.fcgi?db=gene&cmd=Retrieve&dopt=full_report&list_uids=9760) | thymocyte selection associated high mobility group box |
| [Details](http://mirdb.org/cgi-bin/target_detail.cgi?targetID=2444177) | 312 | 70 | hsa-miR-222-3p | [PRKAB2](http://www.ncbi.nlm.nih.gov/entrez/query.fcgi?db=gene&cmd=Retrieve&dopt=full_report&list_uids=5565) | protein kinase AMP-activated non-catalytic subunit beta 2 |
| [Details](http://mirdb.org/cgi-bin/target_detail.cgi?targetID=2444274) | 313 | 70 | hsa-miR-222-3p | [GJC3](http://www.ncbi.nlm.nih.gov/entrez/query.fcgi?db=gene&cmd=Retrieve&dopt=full_report&list_uids=349149) | gap junction protein gamma 3 |
| [Details](http://mirdb.org/cgi-bin/target_detail.cgi?targetID=2443995) | 314 | 70 | hsa-miR-222-3p | [SEC24B](http://www.ncbi.nlm.nih.gov/entrez/query.fcgi?db=gene&cmd=Retrieve&dopt=full_report&list_uids=10427) | SEC24 homolog B, COPII coat complex component |
| [Details](http://mirdb.org/cgi-bin/target_detail.cgi?targetID=2444210) | 315 | 70 | hsa-miR-222-3p | [NAP1L1](http://www.ncbi.nlm.nih.gov/entrez/query.fcgi?db=gene&cmd=Retrieve&dopt=full_report&list_uids=4673) | nucleosome assembly protein 1 like 1 |
| [Details](http://mirdb.org/cgi-bin/target_detail.cgi?targetID=2443975) | 316 | 70 | hsa-miR-222-3p | [LRRTM2](http://www.ncbi.nlm.nih.gov/entrez/query.fcgi?db=gene&cmd=Retrieve&dopt=full_report&list_uids=26045) | leucine rich repeat transmembrane neuronal 2 |
| [Details](http://mirdb.org/cgi-bin/target_detail.cgi?targetID=2444079) | 317 | 70 | hsa-miR-222-3p | [CYREN](http://www.ncbi.nlm.nih.gov/entrez/query.fcgi?db=gene&cmd=Retrieve&dopt=full_report&list_uids=78996) | cell cycle regulator of NHEJ |
| [Details](http://mirdb.org/cgi-bin/target_detail.cgi?targetID=2444102) | 318 | 70 | hsa-miR-222-3p | [SKP1](http://www.ncbi.nlm.nih.gov/entrez/query.fcgi?db=gene&cmd=Retrieve&dopt=full_report&list_uids=6500) | S-phase kinase associated protein 1 |
| [Details](http://mirdb.org/cgi-bin/target_detail.cgi?targetID=2443960) | 319 | 70 | hsa-miR-222-3p | [HNRNPA0](http://www.ncbi.nlm.nih.gov/entrez/query.fcgi?db=gene&cmd=Retrieve&dopt=full_report&list_uids=10949) | heterogeneous nuclear ribonucleoprotein A0 |
| [Details](http://mirdb.org/cgi-bin/target_detail.cgi?targetID=2444451) | 320 | 70 | hsa-miR-222-3p | [NSMCE4A](http://www.ncbi.nlm.nih.gov/entrez/query.fcgi?db=gene&cmd=Retrieve&dopt=full_report&list_uids=54780) | NSE4 homolog A, SMC5-SMC6 complex component |
| [Details](http://mirdb.org/cgi-bin/target_detail.cgi?targetID=2444412) | 321 | 70 | hsa-miR-222-3p | [CCP110](http://www.ncbi.nlm.nih.gov/entrez/query.fcgi?db=gene&cmd=Retrieve&dopt=full_report&list_uids=9738) | centriolar coiled-coil protein 110 |
| [Details](http://mirdb.org/cgi-bin/target_detail.cgi?targetID=2444092) | 322 | 70 | hsa-miR-222-3p | [SEMA6D](http://www.ncbi.nlm.nih.gov/entrez/query.fcgi?db=gene&cmd=Retrieve&dopt=full_report&list_uids=80031) | semaphorin 6D |
| [Details](http://mirdb.org/cgi-bin/target_detail.cgi?targetID=2444072) | 323 | 69 | hsa-miR-222-3p | [CHD7](http://www.ncbi.nlm.nih.gov/entrez/query.fcgi?db=gene&cmd=Retrieve&dopt=full_report&list_uids=55636) | chromodomain helicase DNA binding protein 7 |
| [Details](http://mirdb.org/cgi-bin/target_detail.cgi?targetID=2444521) | 324 | 69 | hsa-miR-222-3p | [CYP4X1](http://www.ncbi.nlm.nih.gov/entrez/query.fcgi?db=gene&cmd=Retrieve&dopt=full_report&list_uids=260293) | cytochrome P450 family 4 subfamily X member 1 |
| [Details](http://mirdb.org/cgi-bin/target_detail.cgi?targetID=2444146) | 325 | 69 | hsa-miR-222-3p | [IGF2BP2](http://www.ncbi.nlm.nih.gov/entrez/query.fcgi?db=gene&cmd=Retrieve&dopt=full_report&list_uids=10644) | insulin like growth factor 2 mRNA binding protein 2 |
| [Details](http://mirdb.org/cgi-bin/target_detail.cgi?targetID=2444236) | 326 | 69 | hsa-miR-222-3p | [KPNA1](http://www.ncbi.nlm.nih.gov/entrez/query.fcgi?db=gene&cmd=Retrieve&dopt=full_report&list_uids=3836) | karyopherin subunit alpha 1 |
| [Details](http://mirdb.org/cgi-bin/target_detail.cgi?targetID=2444275) | 327 | 69 | hsa-miR-222-3p | [ANKRD10](http://www.ncbi.nlm.nih.gov/entrez/query.fcgi?db=gene&cmd=Retrieve&dopt=full_report&list_uids=55608) | ankyrin repeat domain 10 |
| [Details](http://mirdb.org/cgi-bin/target_detail.cgi?targetID=2444013) | 328 | 69 | hsa-miR-222-3p | [SOCS3](http://www.ncbi.nlm.nih.gov/entrez/query.fcgi?db=gene&cmd=Retrieve&dopt=full_report&list_uids=9021) | suppressor of cytokine signaling 3 |
| [Details](http://mirdb.org/cgi-bin/target_detail.cgi?targetID=2444125) | 329 | 69 | hsa-miR-222-3p | [UGT2B15](http://www.ncbi.nlm.nih.gov/entrez/query.fcgi?db=gene&cmd=Retrieve&dopt=full_report&list_uids=7366) | UDP glucuronosyltransferase family 2 member B15 |
| [Details](http://mirdb.org/cgi-bin/target_detail.cgi?targetID=2444449) | 330 | 69 | hsa-miR-222-3p | [IFIT2](http://www.ncbi.nlm.nih.gov/entrez/query.fcgi?db=gene&cmd=Retrieve&dopt=full_report&list_uids=3433) | interferon induced protein with tetratricopeptide repeats 2 |
| [Details](http://mirdb.org/cgi-bin/target_detail.cgi?targetID=2444042) | 331 | 69 | hsa-miR-222-3p | [NLK](http://www.ncbi.nlm.nih.gov/entrez/query.fcgi?db=gene&cmd=Retrieve&dopt=full_report&list_uids=51701) | nemo like kinase |
| [Details](http://mirdb.org/cgi-bin/target_detail.cgi?targetID=2444165) | 332 | 69 | hsa-miR-222-3p | [MEX3A](http://www.ncbi.nlm.nih.gov/entrez/query.fcgi?db=gene&cmd=Retrieve&dopt=full_report&list_uids=92312) | mex-3 RNA binding family member A |
| [Details](http://mirdb.org/cgi-bin/target_detail.cgi?targetID=2444486) | 333 | 69 | hsa-miR-222-3p | [ANKS1B](http://www.ncbi.nlm.nih.gov/entrez/query.fcgi?db=gene&cmd=Retrieve&dopt=full_report&list_uids=56899) | ankyrin repeat and sterile alpha motif domain containing 1B |
| [Details](http://mirdb.org/cgi-bin/target_detail.cgi?targetID=2444466) | 334 | 69 | hsa-miR-222-3p | [HLTF](http://www.ncbi.nlm.nih.gov/entrez/query.fcgi?db=gene&cmd=Retrieve&dopt=full_report&list_uids=6596) | helicase like transcription factor |
| [Details](http://mirdb.org/cgi-bin/target_detail.cgi?targetID=2444178) | 335 | 69 | hsa-miR-222-3p | [YTHDF3](http://www.ncbi.nlm.nih.gov/entrez/query.fcgi?db=gene&cmd=Retrieve&dopt=full_report&list_uids=253943) | YTH N6-methyladenosine RNA binding protein 3 |
| [Details](http://mirdb.org/cgi-bin/target_detail.cgi?targetID=2444551) | 336 | 68 | hsa-miR-222-3p | [PTPRZ1](http://www.ncbi.nlm.nih.gov/entrez/query.fcgi?db=gene&cmd=Retrieve&dopt=full_report&list_uids=5803) | protein tyrosine phosphatase, receptor type Z1 |
| [Details](http://mirdb.org/cgi-bin/target_detail.cgi?targetID=2444321) | 337 | 68 | hsa-miR-222-3p | [ASB4](http://www.ncbi.nlm.nih.gov/entrez/query.fcgi?db=gene&cmd=Retrieve&dopt=full_report&list_uids=51666) | ankyrin repeat and SOCS box containing 4 |
| [Details](http://mirdb.org/cgi-bin/target_detail.cgi?targetID=2444507) | 338 | 68 | hsa-miR-222-3p | [KANK4](http://www.ncbi.nlm.nih.gov/entrez/query.fcgi?db=gene&cmd=Retrieve&dopt=full_report&list_uids=163782) | KN motif and ankyrin repeat domains 4 |
| [Details](http://mirdb.org/cgi-bin/target_detail.cgi?targetID=2444368) | 339 | 68 | hsa-miR-222-3p | [TRABD2B](http://www.ncbi.nlm.nih.gov/entrez/query.fcgi?db=gene&cmd=Retrieve&dopt=full_report&list_uids=388630) | TraB domain containing 2B |
| [Details](http://mirdb.org/cgi-bin/target_detail.cgi?targetID=2444463) | 340 | 68 | hsa-miR-222-3p | [CEP41](http://www.ncbi.nlm.nih.gov/entrez/query.fcgi?db=gene&cmd=Retrieve&dopt=full_report&list_uids=95681) | centrosomal protein 41 |
| [Details](http://mirdb.org/cgi-bin/target_detail.cgi?targetID=2444261) | 341 | 68 | hsa-miR-222-3p | [ZNF652](http://www.ncbi.nlm.nih.gov/entrez/query.fcgi?db=gene&cmd=Retrieve&dopt=full_report&list_uids=22834) | zinc finger protein 652 |
| [Details](http://mirdb.org/cgi-bin/target_detail.cgi?targetID=2444255) | 342 | 68 | hsa-miR-222-3p | [RUNX2](http://www.ncbi.nlm.nih.gov/entrez/query.fcgi?db=gene&cmd=Retrieve&dopt=full_report&list_uids=860) | runt related transcription factor 2 |
| [Details](http://mirdb.org/cgi-bin/target_detail.cgi?targetID=2444242) | 343 | 68 | hsa-miR-222-3p | [MPZL1](http://www.ncbi.nlm.nih.gov/entrez/query.fcgi?db=gene&cmd=Retrieve&dopt=full_report&list_uids=9019) | myelin protein zero like 1 |
| [Details](http://mirdb.org/cgi-bin/target_detail.cgi?targetID=2444402) | 344 | 68 | hsa-miR-222-3p | [PDCD10](http://www.ncbi.nlm.nih.gov/entrez/query.fcgi?db=gene&cmd=Retrieve&dopt=full_report&list_uids=11235) | programmed cell death 10 |
| [Details](http://mirdb.org/cgi-bin/target_detail.cgi?targetID=2444004) | 345 | 68 | hsa-miR-222-3p | [MASTL](http://www.ncbi.nlm.nih.gov/entrez/query.fcgi?db=gene&cmd=Retrieve&dopt=full_report&list_uids=84930) | microtubule associated serine/threonine kinase like |
| [Details](http://mirdb.org/cgi-bin/target_detail.cgi?targetID=2444229) | 346 | 68 | hsa-miR-222-3p | [FAM160B1](http://www.ncbi.nlm.nih.gov/entrez/query.fcgi?db=gene&cmd=Retrieve&dopt=full_report&list_uids=57700) | family with sequence similarity 160 member B1 |
| [Details](http://mirdb.org/cgi-bin/target_detail.cgi?targetID=2444057) | 347 | 68 | hsa-miR-222-3p | [NGRN](http://www.ncbi.nlm.nih.gov/entrez/query.fcgi?db=gene&cmd=Retrieve&dopt=full_report&list_uids=51335) | neugrin, neurite outgrowth associated |
| [Details](http://mirdb.org/cgi-bin/target_detail.cgi?targetID=2444009) | 348 | 68 | hsa-miR-222-3p | [COBLL1](http://www.ncbi.nlm.nih.gov/entrez/query.fcgi?db=gene&cmd=Retrieve&dopt=full_report&list_uids=22837) | cordon-bleu WH2 repeat protein like 1 |
| [Details](http://mirdb.org/cgi-bin/target_detail.cgi?targetID=2444226) | 349 | 68 | hsa-miR-222-3p | [FAT2](http://www.ncbi.nlm.nih.gov/entrez/query.fcgi?db=gene&cmd=Retrieve&dopt=full_report&list_uids=2196) | FAT atypical cadherin 2 |
| [Details](http://mirdb.org/cgi-bin/target_detail.cgi?targetID=2444143) | 350 | 68 | hsa-miR-222-3p | [PTBP3](http://www.ncbi.nlm.nih.gov/entrez/query.fcgi?db=gene&cmd=Retrieve&dopt=full_report&list_uids=9991) | polypyrimidine tract binding protein 3 |
| [Details](http://mirdb.org/cgi-bin/target_detail.cgi?targetID=2444037) | 351 | 68 | hsa-miR-222-3p | [TMEM132C](http://www.ncbi.nlm.nih.gov/entrez/query.fcgi?db=gene&cmd=Retrieve&dopt=full_report&list_uids=92293) | transmembrane protein 132C |
| [Details](http://mirdb.org/cgi-bin/target_detail.cgi?targetID=2444190) | 352 | 67 | hsa-miR-222-3p | [CEP70](http://www.ncbi.nlm.nih.gov/entrez/query.fcgi?db=gene&cmd=Retrieve&dopt=full_report&list_uids=80321) | centrosomal protein 70 |
| [Details](http://mirdb.org/cgi-bin/target_detail.cgi?targetID=2444075) | 353 | 67 | hsa-miR-222-3p | [NRG1](http://www.ncbi.nlm.nih.gov/entrez/query.fcgi?db=gene&cmd=Retrieve&dopt=full_report&list_uids=3084) | neuregulin 1 |
| [Details](http://mirdb.org/cgi-bin/target_detail.cgi?targetID=2444182) | 354 | 67 | hsa-miR-222-3p | [ACVR2B](http://www.ncbi.nlm.nih.gov/entrez/query.fcgi?db=gene&cmd=Retrieve&dopt=full_report&list_uids=93) | activin A receptor type 2B |
| [Details](http://mirdb.org/cgi-bin/target_detail.cgi?targetID=2443976) | 355 | 67 | hsa-miR-222-3p | [GNB1](http://www.ncbi.nlm.nih.gov/entrez/query.fcgi?db=gene&cmd=Retrieve&dopt=full_report&list_uids=2782) | G protein subunit beta 1 |
| [Details](http://mirdb.org/cgi-bin/target_detail.cgi?targetID=2444342) | 356 | 67 | hsa-miR-222-3p | [ERCC4](http://www.ncbi.nlm.nih.gov/entrez/query.fcgi?db=gene&cmd=Retrieve&dopt=full_report&list_uids=2072) | ERCC excision repair 4, endonuclease catalytic subunit |
| [Details](http://mirdb.org/cgi-bin/target_detail.cgi?targetID=2444117) | 357 | 67 | hsa-miR-222-3p | [CCSAP](http://www.ncbi.nlm.nih.gov/entrez/query.fcgi?db=gene&cmd=Retrieve&dopt=full_report&list_uids=126731) | centriole, cilia and spindle associated protein |
| [Details](http://mirdb.org/cgi-bin/target_detail.cgi?targetID=2444285) | 358 | 67 | hsa-miR-222-3p | [RASSF8](http://www.ncbi.nlm.nih.gov/entrez/query.fcgi?db=gene&cmd=Retrieve&dopt=full_report&list_uids=11228) | Ras association domain family member 8 |
| [Details](http://mirdb.org/cgi-bin/target_detail.cgi?targetID=2444398) | 359 | 67 | hsa-miR-222-3p | [CD164](http://www.ncbi.nlm.nih.gov/entrez/query.fcgi?db=gene&cmd=Retrieve&dopt=full_report&list_uids=8763) | CD164 molecule |
| [Details](http://mirdb.org/cgi-bin/target_detail.cgi?targetID=2444114) | 360 | 67 | hsa-miR-222-3p | [KHDRBS2](http://www.ncbi.nlm.nih.gov/entrez/query.fcgi?db=gene&cmd=Retrieve&dopt=full_report&list_uids=202559) | KH RNA binding domain containing, signal transduction associated 2 |
| [Details](http://mirdb.org/cgi-bin/target_detail.cgi?targetID=2444352) | 361 | 66 | hsa-miR-222-3p | [DLD](http://www.ncbi.nlm.nih.gov/entrez/query.fcgi?db=gene&cmd=Retrieve&dopt=full_report&list_uids=1738) | dihydrolipoamide dehydrogenase |
| [Details](http://mirdb.org/cgi-bin/target_detail.cgi?targetID=2444499) | 362 | 66 | hsa-miR-222-3p | [TYMSOS](http://www.ncbi.nlm.nih.gov/entrez/query.fcgi?db=gene&cmd=Retrieve&dopt=full_report&list_uids=494514) | TYMS opposite strand |
| [Details](http://mirdb.org/cgi-bin/target_detail.cgi?targetID=2444238) | 363 | 66 | hsa-miR-222-3p | [SLC4A4](http://www.ncbi.nlm.nih.gov/entrez/query.fcgi?db=gene&cmd=Retrieve&dopt=full_report&list_uids=8671) | solute carrier family 4 member 4 |
| [Details](http://mirdb.org/cgi-bin/target_detail.cgi?targetID=2444198) | 364 | 66 | hsa-miR-222-3p | [CFHR5](http://www.ncbi.nlm.nih.gov/entrez/query.fcgi?db=gene&cmd=Retrieve&dopt=full_report&list_uids=81494) | complement factor H related 5 |
| [Details](http://mirdb.org/cgi-bin/target_detail.cgi?targetID=2444430) | 365 | 66 | hsa-miR-222-3p | [CALM1](http://www.ncbi.nlm.nih.gov/entrez/query.fcgi?db=gene&cmd=Retrieve&dopt=full_report&list_uids=801) | calmodulin 1 |
| [Details](http://mirdb.org/cgi-bin/target_detail.cgi?targetID=2444010) | 366 | 66 | hsa-miR-222-3p | [SPTBN1](http://www.ncbi.nlm.nih.gov/entrez/query.fcgi?db=gene&cmd=Retrieve&dopt=full_report&list_uids=6711) | spectrin beta, non-erythrocytic 1 |
| [Details](http://mirdb.org/cgi-bin/target_detail.cgi?targetID=2444221) | 367 | 66 | hsa-miR-222-3p | [RNF20](http://www.ncbi.nlm.nih.gov/entrez/query.fcgi?db=gene&cmd=Retrieve&dopt=full_report&list_uids=56254) | ring finger protein 20 |
| [Details](http://mirdb.org/cgi-bin/target_detail.cgi?targetID=2444332) | 368 | 66 | hsa-miR-222-3p | [ETS1](http://www.ncbi.nlm.nih.gov/entrez/query.fcgi?db=gene&cmd=Retrieve&dopt=full_report&list_uids=2113) | ETS proto-oncogene 1, transcription factor |
| [Details](http://mirdb.org/cgi-bin/target_detail.cgi?targetID=2444225) | 369 | 66 | hsa-miR-222-3p | [ALDH16A1](http://www.ncbi.nlm.nih.gov/entrez/query.fcgi?db=gene&cmd=Retrieve&dopt=full_report&list_uids=126133) | aldehyde dehydrogenase 16 family member A1 |
| [Details](http://mirdb.org/cgi-bin/target_detail.cgi?targetID=2444286) | 370 | 66 | hsa-miR-222-3p | [CARF](http://www.ncbi.nlm.nih.gov/entrez/query.fcgi?db=gene&cmd=Retrieve&dopt=full_report&list_uids=79800) | calcium responsive transcription factor |
| [Details](http://mirdb.org/cgi-bin/target_detail.cgi?targetID=2444523) | 371 | 66 | hsa-miR-222-3p | [ANKHD1](http://www.ncbi.nlm.nih.gov/entrez/query.fcgi?db=gene&cmd=Retrieve&dopt=full_report&list_uids=54882) | ankyrin repeat and KH domain containing 1 |
| [Details](http://mirdb.org/cgi-bin/target_detail.cgi?targetID=2444438) | 372 | 66 | hsa-miR-222-3p | [STN1](http://www.ncbi.nlm.nih.gov/entrez/query.fcgi?db=gene&cmd=Retrieve&dopt=full_report&list_uids=79991) | STN1, CST complex subunit |
| [Details](http://mirdb.org/cgi-bin/target_detail.cgi?targetID=2444385) | 373 | 66 | hsa-miR-222-3p | [USP6NL](http://www.ncbi.nlm.nih.gov/entrez/query.fcgi?db=gene&cmd=Retrieve&dopt=full_report&list_uids=9712) | USP6 N-terminal like |
| [Details](http://mirdb.org/cgi-bin/target_detail.cgi?targetID=2444305) | 374 | 66 | hsa-miR-222-3p | [ANTXR2](http://www.ncbi.nlm.nih.gov/entrez/query.fcgi?db=gene&cmd=Retrieve&dopt=full_report&list_uids=118429) | ANTXR cell adhesion molecule 2 |
| [Details](http://mirdb.org/cgi-bin/target_detail.cgi?targetID=2443980) | 375 | 66 | hsa-miR-222-3p | [BCL2L14](http://www.ncbi.nlm.nih.gov/entrez/query.fcgi?db=gene&cmd=Retrieve&dopt=full_report&list_uids=79370) | BCL2 like 14 |
| [Details](http://mirdb.org/cgi-bin/target_detail.cgi?targetID=2444254) | 376 | 66 | hsa-miR-222-3p | [FBXO28](http://www.ncbi.nlm.nih.gov/entrez/query.fcgi?db=gene&cmd=Retrieve&dopt=full_report&list_uids=23219) | F-box protein 28 |
| [Details](http://mirdb.org/cgi-bin/target_detail.cgi?targetID=2444532) | 377 | 66 | hsa-miR-222-3p | [MON2](http://www.ncbi.nlm.nih.gov/entrez/query.fcgi?db=gene&cmd=Retrieve&dopt=full_report&list_uids=23041) | MON2 homolog, regulator of endosome-to-Golgi trafficking |
| [Details](http://mirdb.org/cgi-bin/target_detail.cgi?targetID=2444457) | 378 | 66 | hsa-miR-222-3p | [ZC3H13](http://www.ncbi.nlm.nih.gov/entrez/query.fcgi?db=gene&cmd=Retrieve&dopt=full_report&list_uids=23091) | zinc finger CCCH-type containing 13 |
| [Details](http://mirdb.org/cgi-bin/target_detail.cgi?targetID=2444108) | 379 | 66 | hsa-miR-222-3p | [ZNF3](http://www.ncbi.nlm.nih.gov/entrez/query.fcgi?db=gene&cmd=Retrieve&dopt=full_report&list_uids=7551) | zinc finger protein 3 |
| [Details](http://mirdb.org/cgi-bin/target_detail.cgi?targetID=2444297) | 380 | 65 | hsa-miR-222-3p | [KSR1](http://www.ncbi.nlm.nih.gov/entrez/query.fcgi?db=gene&cmd=Retrieve&dopt=full_report&list_uids=8844) | kinase suppressor of ras 1 |
| [Details](http://mirdb.org/cgi-bin/target_detail.cgi?targetID=2444468) | 381 | 65 | hsa-miR-222-3p | [CDON](http://www.ncbi.nlm.nih.gov/entrez/query.fcgi?db=gene&cmd=Retrieve&dopt=full_report&list_uids=50937) | cell adhesion associated, oncogene regulated |
| [Details](http://mirdb.org/cgi-bin/target_detail.cgi?targetID=2444208) | 382 | 65 | hsa-miR-222-3p | [TMEM132B](http://www.ncbi.nlm.nih.gov/entrez/query.fcgi?db=gene&cmd=Retrieve&dopt=full_report&list_uids=114795) | transmembrane protein 132B |
| [Details](http://mirdb.org/cgi-bin/target_detail.cgi?targetID=2444259) | 383 | 65 | hsa-miR-222-3p | [TCF7L2](http://www.ncbi.nlm.nih.gov/entrez/query.fcgi?db=gene&cmd=Retrieve&dopt=full_report&list_uids=6934) | transcription factor 7 like 2 |
| [Details](http://mirdb.org/cgi-bin/target_detail.cgi?targetID=2444292) | 384 | 65 | hsa-miR-222-3p | [PPP4R2](http://www.ncbi.nlm.nih.gov/entrez/query.fcgi?db=gene&cmd=Retrieve&dopt=full_report&list_uids=151987) | protein phosphatase 4 regulatory subunit 2 |
| [Details](http://mirdb.org/cgi-bin/target_detail.cgi?targetID=2444179) | 385 | 65 | hsa-miR-222-3p | [STK24](http://www.ncbi.nlm.nih.gov/entrez/query.fcgi?db=gene&cmd=Retrieve&dopt=full_report&list_uids=8428) | serine/threonine kinase 24 |
| [Details](http://mirdb.org/cgi-bin/target_detail.cgi?targetID=2444024) | 386 | 65 | hsa-miR-222-3p | [GOLGA1](http://www.ncbi.nlm.nih.gov/entrez/query.fcgi?db=gene&cmd=Retrieve&dopt=full_report&list_uids=2800) | golgin A1 |
| [Details](http://mirdb.org/cgi-bin/target_detail.cgi?targetID=2444422) | 387 | 65 | hsa-miR-222-3p | [PDCD6IP](http://www.ncbi.nlm.nih.gov/entrez/query.fcgi?db=gene&cmd=Retrieve&dopt=full_report&list_uids=10015) | programmed cell death 6 interacting protein |
| [Details](http://mirdb.org/cgi-bin/target_detail.cgi?targetID=2443967) | 388 | 65 | hsa-miR-222-3p | [C22orf39](http://www.ncbi.nlm.nih.gov/entrez/query.fcgi?db=gene&cmd=Retrieve&dopt=full_report&list_uids=128977) | chromosome 22 open reading frame 39 |
| [Details](http://mirdb.org/cgi-bin/target_detail.cgi?targetID=2444367) | 389 | 65 | hsa-miR-222-3p | [CACNB4](http://www.ncbi.nlm.nih.gov/entrez/query.fcgi?db=gene&cmd=Retrieve&dopt=full_report&list_uids=785) | calcium voltage-gated channel auxiliary subunit beta 4 |
| [Details](http://mirdb.org/cgi-bin/target_detail.cgi?targetID=2444524) | 390 | 65 | hsa-miR-222-3p | [DGKH](http://www.ncbi.nlm.nih.gov/entrez/query.fcgi?db=gene&cmd=Retrieve&dopt=full_report&list_uids=160851) | diacylglycerol kinase eta |
| [Details](http://mirdb.org/cgi-bin/target_detail.cgi?targetID=2444490) | 391 | 65 | hsa-miR-222-3p | [NUFIP2](http://www.ncbi.nlm.nih.gov/entrez/query.fcgi?db=gene&cmd=Retrieve&dopt=full_report&list_uids=57532) | nuclear FMR1 interacting protein 2 |
| [Details](http://mirdb.org/cgi-bin/target_detail.cgi?targetID=2443940) | 392 | 65 | hsa-miR-222-3p | [FAM222B](http://www.ncbi.nlm.nih.gov/entrez/query.fcgi?db=gene&cmd=Retrieve&dopt=full_report&list_uids=55731) | family with sequence similarity 222 member B |
| [Details](http://mirdb.org/cgi-bin/target_detail.cgi?targetID=2444538) | 393 | 65 | hsa-miR-222-3p | [AGFG1](http://www.ncbi.nlm.nih.gov/entrez/query.fcgi?db=gene&cmd=Retrieve&dopt=full_report&list_uids=3267) | ArfGAP with FG repeats 1 |
| [Details](http://mirdb.org/cgi-bin/target_detail.cgi?targetID=2444215) | 394 | 65 | hsa-miR-222-3p | [CCT5](http://www.ncbi.nlm.nih.gov/entrez/query.fcgi?db=gene&cmd=Retrieve&dopt=full_report&list_uids=22948) | chaperonin containing TCP1 subunit 5 |
| [Details](http://mirdb.org/cgi-bin/target_detail.cgi?targetID=2444048) | 395 | 64 | hsa-miR-222-3p | [PTPRR](http://www.ncbi.nlm.nih.gov/entrez/query.fcgi?db=gene&cmd=Retrieve&dopt=full_report&list_uids=5801) | protein tyrosine phosphatase, receptor type R |
| [Details](http://mirdb.org/cgi-bin/target_detail.cgi?targetID=2444015) | 396 | 64 | hsa-miR-222-3p | [CDKN2B](http://www.ncbi.nlm.nih.gov/entrez/query.fcgi?db=gene&cmd=Retrieve&dopt=full_report&list_uids=1030) | cyclin dependent kinase inhibitor 2B |
| [Details](http://mirdb.org/cgi-bin/target_detail.cgi?targetID=2444157) | 397 | 64 | hsa-miR-222-3p | [HDLBP](http://www.ncbi.nlm.nih.gov/entrez/query.fcgi?db=gene&cmd=Retrieve&dopt=full_report&list_uids=3069) | high density lipoprotein binding protein |
| [Details](http://mirdb.org/cgi-bin/target_detail.cgi?targetID=2444168) | 398 | 64 | hsa-miR-222-3p | [URI1](http://www.ncbi.nlm.nih.gov/entrez/query.fcgi?db=gene&cmd=Retrieve&dopt=full_report&list_uids=8725) | URI1, prefoldin like chaperone |
| [Details](http://mirdb.org/cgi-bin/target_detail.cgi?targetID=2444059) | 399 | 64 | hsa-miR-222-3p | [IGDCC4](http://www.ncbi.nlm.nih.gov/entrez/query.fcgi?db=gene&cmd=Retrieve&dopt=full_report&list_uids=57722) | immunoglobulin superfamily DCC subclass member 4 |
| [Details](http://mirdb.org/cgi-bin/target_detail.cgi?targetID=2444373) | 400 | 64 | hsa-miR-222-3p | [CFAP161](http://www.ncbi.nlm.nih.gov/entrez/query.fcgi?db=gene&cmd=Retrieve&dopt=full_report&list_uids=161502) | cilia and flagella associated protein 161 |
| [Details](http://mirdb.org/cgi-bin/target_detail.cgi?targetID=2444361) | 401 | 64 | hsa-miR-222-3p | [SPPL3](http://www.ncbi.nlm.nih.gov/entrez/query.fcgi?db=gene&cmd=Retrieve&dopt=full_report&list_uids=121665) | signal peptide peptidase like 3 |
| [Details](http://mirdb.org/cgi-bin/target_detail.cgi?targetID=2444363) | 402 | 64 | hsa-miR-222-3p | [EMX2](http://www.ncbi.nlm.nih.gov/entrez/query.fcgi?db=gene&cmd=Retrieve&dopt=full_report&list_uids=2018) | empty spiracles homeobox 2 |
| [Details](http://mirdb.org/cgi-bin/target_detail.cgi?targetID=2444196) | 403 | 64 | hsa-miR-222-3p | [PRDM11](http://www.ncbi.nlm.nih.gov/entrez/query.fcgi?db=gene&cmd=Retrieve&dopt=full_report&list_uids=56981) | PR/SET domain 11 |
| [Details](http://mirdb.org/cgi-bin/target_detail.cgi?targetID=2444432) | 404 | 64 | hsa-miR-222-3p | [SHANK2](http://www.ncbi.nlm.nih.gov/entrez/query.fcgi?db=gene&cmd=Retrieve&dopt=full_report&list_uids=22941) | SH3 and multiple ankyrin repeat domains 2 |
| [Details](http://mirdb.org/cgi-bin/target_detail.cgi?targetID=2444078) | 405 | 64 | hsa-miR-222-3p | [CRHBP](http://www.ncbi.nlm.nih.gov/entrez/query.fcgi?db=gene&cmd=Retrieve&dopt=full_report&list_uids=1393) | corticotropin releasing hormone binding protein |
| [Details](http://mirdb.org/cgi-bin/target_detail.cgi?targetID=2444323) | 406 | 64 | hsa-miR-222-3p | [LRRC19](http://www.ncbi.nlm.nih.gov/entrez/query.fcgi?db=gene&cmd=Retrieve&dopt=full_report&list_uids=64922) | leucine rich repeat containing 19 |
| [Details](http://mirdb.org/cgi-bin/target_detail.cgi?targetID=2444170) | 407 | 64 | hsa-miR-222-3p | [CD4](http://www.ncbi.nlm.nih.gov/entrez/query.fcgi?db=gene&cmd=Retrieve&dopt=full_report&list_uids=920) | CD4 molecule |
| [Details](http://mirdb.org/cgi-bin/target_detail.cgi?targetID=2443987) | 408 | 64 | hsa-miR-222-3p | [CDV3](http://www.ncbi.nlm.nih.gov/entrez/query.fcgi?db=gene&cmd=Retrieve&dopt=full_report&list_uids=55573) | CDV3 homolog |
| [Details](http://mirdb.org/cgi-bin/target_detail.cgi?targetID=2443977) | 409 | 64 | hsa-miR-222-3p | [CAMK1D](http://www.ncbi.nlm.nih.gov/entrez/query.fcgi?db=gene&cmd=Retrieve&dopt=full_report&list_uids=57118) | calcium/calmodulin dependent protein kinase ID |
| [Details](http://mirdb.org/cgi-bin/target_detail.cgi?targetID=2444260) | 410 | 64 | hsa-miR-222-3p | [CD47](http://www.ncbi.nlm.nih.gov/entrez/query.fcgi?db=gene&cmd=Retrieve&dopt=full_report&list_uids=961) | CD47 molecule |
| [Details](http://mirdb.org/cgi-bin/target_detail.cgi?targetID=2444317) | 411 | 64 | hsa-miR-222-3p | [TMEM25](http://www.ncbi.nlm.nih.gov/entrez/query.fcgi?db=gene&cmd=Retrieve&dopt=full_report&list_uids=84866) | transmembrane protein 25 |
| [Details](http://mirdb.org/cgi-bin/target_detail.cgi?targetID=2443969) | 412 | 63 | hsa-miR-222-3p | [STYX](http://www.ncbi.nlm.nih.gov/entrez/query.fcgi?db=gene&cmd=Retrieve&dopt=full_report&list_uids=6815) | serine/threonine/tyrosine interacting protein |
| [Details](http://mirdb.org/cgi-bin/target_detail.cgi?targetID=2444192) | 413 | 63 | hsa-miR-222-3p | [CRKL](http://www.ncbi.nlm.nih.gov/entrez/query.fcgi?db=gene&cmd=Retrieve&dopt=full_report&list_uids=1399) | CRK like proto-oncogene, adaptor protein |
| [Details](http://mirdb.org/cgi-bin/target_detail.cgi?targetID=2444262) | 414 | 63 | hsa-miR-222-3p | [GPD2](http://www.ncbi.nlm.nih.gov/entrez/query.fcgi?db=gene&cmd=Retrieve&dopt=full_report&list_uids=2820) | glycerol-3-phosphate dehydrogenase 2 |
| [Details](http://mirdb.org/cgi-bin/target_detail.cgi?targetID=2444384) | 415 | 63 | hsa-miR-222-3p | [ERMN](http://www.ncbi.nlm.nih.gov/entrez/query.fcgi?db=gene&cmd=Retrieve&dopt=full_report&list_uids=57471) | ermin |
| [Details](http://mirdb.org/cgi-bin/target_detail.cgi?targetID=2444304) | 416 | 63 | hsa-miR-222-3p | [HOXC10](http://www.ncbi.nlm.nih.gov/entrez/query.fcgi?db=gene&cmd=Retrieve&dopt=full_report&list_uids=3226) | homeobox C10 |
| [Details](http://mirdb.org/cgi-bin/target_detail.cgi?targetID=2444456) | 417 | 63 | hsa-miR-222-3p | [SOD2](http://www.ncbi.nlm.nih.gov/entrez/query.fcgi?db=gene&cmd=Retrieve&dopt=full_report&list_uids=6648) | superoxide dismutase 2 |
| [Details](http://mirdb.org/cgi-bin/target_detail.cgi?targetID=2444180) | 418 | 63 | hsa-miR-222-3p | [NBPF3](http://www.ncbi.nlm.nih.gov/entrez/query.fcgi?db=gene&cmd=Retrieve&dopt=full_report&list_uids=84224) | NBPF member 3 |
| [Details](http://mirdb.org/cgi-bin/target_detail.cgi?targetID=2444245) | 419 | 63 | hsa-miR-222-3p | [MGAT4A](http://www.ncbi.nlm.nih.gov/entrez/query.fcgi?db=gene&cmd=Retrieve&dopt=full_report&list_uids=11320) | alpha-1,3-mannosyl-glycoprotein 4-beta-N-acetylglucosaminyltransferase A |
| [Details](http://mirdb.org/cgi-bin/target_detail.cgi?targetID=2444379) | 420 | 63 | hsa-miR-222-3p | [ZFP90](http://www.ncbi.nlm.nih.gov/entrez/query.fcgi?db=gene&cmd=Retrieve&dopt=full_report&list_uids=146198) | ZFP90 zinc finger protein |
| [Details](http://mirdb.org/cgi-bin/target_detail.cgi?targetID=2444090) | 421 | 63 | hsa-miR-222-3p | [SEPT14](http://www.ncbi.nlm.nih.gov/entrez/query.fcgi?db=gene&cmd=Retrieve&dopt=full_report&list_uids=346288) | septin 14 |
| [Details](http://mirdb.org/cgi-bin/target_detail.cgi?targetID=2444388) | 422 | 63 | hsa-miR-222-3p | [RPS3](http://www.ncbi.nlm.nih.gov/entrez/query.fcgi?db=gene&cmd=Retrieve&dopt=full_report&list_uids=6188) | ribosomal protein S3 |
| [Details](http://mirdb.org/cgi-bin/target_detail.cgi?targetID=2444501) | 423 | 63 | hsa-miR-222-3p | [ZNF83](http://www.ncbi.nlm.nih.gov/entrez/query.fcgi?db=gene&cmd=Retrieve&dopt=full_report&list_uids=55769) | zinc finger protein 83 |
| [Details](http://mirdb.org/cgi-bin/target_detail.cgi?targetID=2444035) | 424 | 63 | hsa-miR-222-3p | [HIPK2](http://www.ncbi.nlm.nih.gov/entrez/query.fcgi?db=gene&cmd=Retrieve&dopt=full_report&list_uids=28996) | homeodomain interacting protein kinase 2 |
| [Details](http://mirdb.org/cgi-bin/target_detail.cgi?targetID=2444052) | 425 | 63 | hsa-miR-222-3p | [REV3L](http://www.ncbi.nlm.nih.gov/entrez/query.fcgi?db=gene&cmd=Retrieve&dopt=full_report&list_uids=5980) | REV3 like, DNA directed polymerase zeta catalytic subunit |
| [Details](http://mirdb.org/cgi-bin/target_detail.cgi?targetID=2444325) | 426 | 63 | hsa-miR-222-3p | [ST8SIA1](http://www.ncbi.nlm.nih.gov/entrez/query.fcgi?db=gene&cmd=Retrieve&dopt=full_report&list_uids=6489) | ST8 alpha-N-acetyl-neuraminide alpha-2,8-sialyltransferase 1 |
| [Details](http://mirdb.org/cgi-bin/target_detail.cgi?targetID=2444471) | 427 | 63 | hsa-miR-222-3p | [STOX2](http://www.ncbi.nlm.nih.gov/entrez/query.fcgi?db=gene&cmd=Retrieve&dopt=full_report&list_uids=56977) | storkhead box 2 |
| [Details](http://mirdb.org/cgi-bin/target_detail.cgi?targetID=2444040) | 428 | 62 | hsa-miR-222-3p | [PSAP](http://www.ncbi.nlm.nih.gov/entrez/query.fcgi?db=gene&cmd=Retrieve&dopt=full_report&list_uids=5660) | prosaposin |
| [Details](http://mirdb.org/cgi-bin/target_detail.cgi?targetID=2444211) | 429 | 62 | hsa-miR-222-3p | [C2CD4A](http://www.ncbi.nlm.nih.gov/entrez/query.fcgi?db=gene&cmd=Retrieve&dopt=full_report&list_uids=145741) | C2 calcium dependent domain containing 4A |
| [Details](http://mirdb.org/cgi-bin/target_detail.cgi?targetID=2444169) | 430 | 62 | hsa-miR-222-3p | [NIPA1](http://www.ncbi.nlm.nih.gov/entrez/query.fcgi?db=gene&cmd=Retrieve&dopt=full_report&list_uids=123606) | NIPA magnesium transporter 1 |
| [Details](http://mirdb.org/cgi-bin/target_detail.cgi?targetID=2444062) | 431 | 62 | hsa-miR-222-3p | [DLG2](http://www.ncbi.nlm.nih.gov/entrez/query.fcgi?db=gene&cmd=Retrieve&dopt=full_report&list_uids=1740) | discs large MAGUK scaffold protein 2 |
| [Details](http://mirdb.org/cgi-bin/target_detail.cgi?targetID=2444127) | 432 | 62 | hsa-miR-222-3p | [DIRAS2](http://www.ncbi.nlm.nih.gov/entrez/query.fcgi?db=gene&cmd=Retrieve&dopt=full_report&list_uids=54769) | DIRAS family GTPase 2 |
| [Details](http://mirdb.org/cgi-bin/target_detail.cgi?targetID=2444103) | 433 | 62 | hsa-miR-222-3p | [C18orf54](http://www.ncbi.nlm.nih.gov/entrez/query.fcgi?db=gene&cmd=Retrieve&dopt=full_report&list_uids=162681) | chromosome 18 open reading frame 54 |
| [Details](http://mirdb.org/cgi-bin/target_detail.cgi?targetID=2444494) | 434 | 62 | hsa-miR-222-3p | [CAVIN3](http://www.ncbi.nlm.nih.gov/entrez/query.fcgi?db=gene&cmd=Retrieve&dopt=full_report&list_uids=112464) | caveolae associated protein 3 |
| [Details](http://mirdb.org/cgi-bin/target_detail.cgi?targetID=2444443) | 435 | 62 | hsa-miR-222-3p | [LGI2](http://www.ncbi.nlm.nih.gov/entrez/query.fcgi?db=gene&cmd=Retrieve&dopt=full_report&list_uids=55203) | leucine rich repeat LGI family member 2 |
| [Details](http://mirdb.org/cgi-bin/target_detail.cgi?targetID=2444429) | 436 | 62 | hsa-miR-222-3p | [PLEKHA2](http://www.ncbi.nlm.nih.gov/entrez/query.fcgi?db=gene&cmd=Retrieve&dopt=full_report&list_uids=59339) | pleckstrin homology domain containing A2 |
| [Details](http://mirdb.org/cgi-bin/target_detail.cgi?targetID=2444345) | 437 | 62 | hsa-miR-222-3p | [KBTBD11](http://www.ncbi.nlm.nih.gov/entrez/query.fcgi?db=gene&cmd=Retrieve&dopt=full_report&list_uids=9920) | kelch repeat and BTB domain containing 11 |
| [Details](http://mirdb.org/cgi-bin/target_detail.cgi?targetID=2443961) | 438 | 62 | hsa-miR-222-3p | [GLMN](http://www.ncbi.nlm.nih.gov/entrez/query.fcgi?db=gene&cmd=Retrieve&dopt=full_report&list_uids=11146) | glomulin, FKBP associated protein |
| [Details](http://mirdb.org/cgi-bin/target_detail.cgi?targetID=2444540) | 439 | 62 | hsa-miR-222-3p | [STK38L](http://www.ncbi.nlm.nih.gov/entrez/query.fcgi?db=gene&cmd=Retrieve&dopt=full_report&list_uids=23012) | serine/threonine kinase 38 like |
| [Details](http://mirdb.org/cgi-bin/target_detail.cgi?targetID=2444335) | 440 | 62 | hsa-miR-222-3p | [SLC26A3](http://www.ncbi.nlm.nih.gov/entrez/query.fcgi?db=gene&cmd=Retrieve&dopt=full_report&list_uids=1811) | solute carrier family 26 member 3 |
| [Details](http://mirdb.org/cgi-bin/target_detail.cgi?targetID=2444303) | 441 | 62 | hsa-miR-222-3p | [CRX](http://www.ncbi.nlm.nih.gov/entrez/query.fcgi?db=gene&cmd=Retrieve&dopt=full_report&list_uids=1406) | cone-rod homeobox |
| [Details](http://mirdb.org/cgi-bin/target_detail.cgi?targetID=2444248) | 442 | 62 | hsa-miR-222-3p | [CCDC126](http://www.ncbi.nlm.nih.gov/entrez/query.fcgi?db=gene&cmd=Retrieve&dopt=full_report&list_uids=90693) | coiled-coil domain containing 126 |
| [Details](http://mirdb.org/cgi-bin/target_detail.cgi?targetID=2444197) | 443 | 62 | hsa-miR-222-3p | [PRDM2](http://www.ncbi.nlm.nih.gov/entrez/query.fcgi?db=gene&cmd=Retrieve&dopt=full_report&list_uids=7799) | PR/SET domain 2 |
| [Details](http://mirdb.org/cgi-bin/target_detail.cgi?targetID=2444510) | 444 | 61 | hsa-miR-222-3p | [TMEM237](http://www.ncbi.nlm.nih.gov/entrez/query.fcgi?db=gene&cmd=Retrieve&dopt=full_report&list_uids=65062) | transmembrane protein 237 |
| [Details](http://mirdb.org/cgi-bin/target_detail.cgi?targetID=2444257) | 445 | 61 | hsa-miR-222-3p | [MBD2](http://www.ncbi.nlm.nih.gov/entrez/query.fcgi?db=gene&cmd=Retrieve&dopt=full_report&list_uids=8932) | methyl-CpG binding domain protein 2 |
| [Details](http://mirdb.org/cgi-bin/target_detail.cgi?targetID=2444272) | 446 | 61 | hsa-miR-222-3p | [PCDHA9](http://www.ncbi.nlm.nih.gov/entrez/query.fcgi?db=gene&cmd=Retrieve&dopt=full_report&list_uids=9752) | protocadherin alpha 9 |
| [Details](http://mirdb.org/cgi-bin/target_detail.cgi?targetID=2444414) | 447 | 61 | hsa-miR-222-3p | [SLITRK5](http://www.ncbi.nlm.nih.gov/entrez/query.fcgi?db=gene&cmd=Retrieve&dopt=full_report&list_uids=26050) | SLIT and NTRK like family member 5 |
| [Details](http://mirdb.org/cgi-bin/target_detail.cgi?targetID=2444205) | 448 | 61 | hsa-miR-222-3p | [SLC10A7](http://www.ncbi.nlm.nih.gov/entrez/query.fcgi?db=gene&cmd=Retrieve&dopt=full_report&list_uids=84068) | solute carrier family 10 member 7 |
| [Details](http://mirdb.org/cgi-bin/target_detail.cgi?targetID=2444487) | 449 | 61 | hsa-miR-222-3p | [MSL2](http://www.ncbi.nlm.nih.gov/entrez/query.fcgi?db=gene&cmd=Retrieve&dopt=full_report&list_uids=55167) | MSL complex subunit 2 |
| [Details](http://mirdb.org/cgi-bin/target_detail.cgi?targetID=2444109) | 450 | 61 | hsa-miR-222-3p | [UBE2V1](http://www.ncbi.nlm.nih.gov/entrez/query.fcgi?db=gene&cmd=Retrieve&dopt=full_report&list_uids=7335) | ubiquitin conjugating enzyme E2 V1 |
| [Details](http://mirdb.org/cgi-bin/target_detail.cgi?targetID=2444277) | 451 | 61 | hsa-miR-222-3p | [THBS1](http://www.ncbi.nlm.nih.gov/entrez/query.fcgi?db=gene&cmd=Retrieve&dopt=full_report&list_uids=7057) | thrombospondin 1 |
| [Details](http://mirdb.org/cgi-bin/target_detail.cgi?targetID=2444047) | 452 | 61 | hsa-miR-222-3p | [GLS](http://www.ncbi.nlm.nih.gov/entrez/query.fcgi?db=gene&cmd=Retrieve&dopt=full_report&list_uids=2744) | glutaminase |
| [Details](http://mirdb.org/cgi-bin/target_detail.cgi?targetID=2444378) | 453 | 61 | hsa-miR-222-3p | [PLPP3](http://www.ncbi.nlm.nih.gov/entrez/query.fcgi?db=gene&cmd=Retrieve&dopt=full_report&list_uids=8613) | phospholipid phosphatase 3 |
| [Details](http://mirdb.org/cgi-bin/target_detail.cgi?targetID=2444380) | 454 | 61 | hsa-miR-222-3p | [PROM2](http://www.ncbi.nlm.nih.gov/entrez/query.fcgi?db=gene&cmd=Retrieve&dopt=full_report&list_uids=150696) | prominin 2 |
| [Details](http://mirdb.org/cgi-bin/target_detail.cgi?targetID=2444095) | 455 | 61 | hsa-miR-222-3p | [KCNQ3](http://www.ncbi.nlm.nih.gov/entrez/query.fcgi?db=gene&cmd=Retrieve&dopt=full_report&list_uids=3786) | potassium voltage-gated channel subfamily Q member 3 |
| [Details](http://mirdb.org/cgi-bin/target_detail.cgi?targetID=2444401) | 456 | 61 | hsa-miR-222-3p | [CCDC148](http://www.ncbi.nlm.nih.gov/entrez/query.fcgi?db=gene&cmd=Retrieve&dopt=full_report&list_uids=130940) | coiled-coil domain containing 148 |
| [Details](http://mirdb.org/cgi-bin/target_detail.cgi?targetID=2444209) | 457 | 61 | hsa-miR-222-3p | [ADIPOR1](http://www.ncbi.nlm.nih.gov/entrez/query.fcgi?db=gene&cmd=Retrieve&dopt=full_report&list_uids=51094) | adiponectin receptor 1 |
| [Details](http://mirdb.org/cgi-bin/target_detail.cgi?targetID=2444531) | 458 | 61 | hsa-miR-222-3p | [MAPK10](http://www.ncbi.nlm.nih.gov/entrez/query.fcgi?db=gene&cmd=Retrieve&dopt=full_report&list_uids=5602) | mitogen-activated protein kinase 10 |
| [Details](http://mirdb.org/cgi-bin/target_detail.cgi?targetID=2444249) | 459 | 61 | hsa-miR-222-3p | [TMEM167A](http://www.ncbi.nlm.nih.gov/entrez/query.fcgi?db=gene&cmd=Retrieve&dopt=full_report&list_uids=153339) | transmembrane protein 167A |
| [Details](http://mirdb.org/cgi-bin/target_detail.cgi?targetID=2444116) | 460 | 60 | hsa-miR-222-3p | [TMSB15B](http://www.ncbi.nlm.nih.gov/entrez/query.fcgi?db=gene&cmd=Retrieve&dopt=full_report&list_uids=286527) | thymosin beta 15B |
| [Details](http://mirdb.org/cgi-bin/target_detail.cgi?targetID=2443938) | 461 | 60 | hsa-miR-222-3p | [RECK](http://www.ncbi.nlm.nih.gov/entrez/query.fcgi?db=gene&cmd=Retrieve&dopt=full_report&list_uids=8434) | reversion inducing cysteine rich protein with kazal motifs |
| [Details](http://mirdb.org/cgi-bin/target_detail.cgi?targetID=2444176) | 462 | 60 | hsa-miR-222-3p | [SH3PXD2B](http://www.ncbi.nlm.nih.gov/entrez/query.fcgi?db=gene&cmd=Retrieve&dopt=full_report&list_uids=285590) | SH3 and PX domains 2B |
| [Details](http://mirdb.org/cgi-bin/target_detail.cgi?targetID=2444139) | 463 | 60 | hsa-miR-222-3p | [TBC1D22B](http://www.ncbi.nlm.nih.gov/entrez/query.fcgi?db=gene&cmd=Retrieve&dopt=full_report&list_uids=55633) | TBC1 domain family member 22B |
| [Details](http://mirdb.org/cgi-bin/target_detail.cgi?targetID=2443941) | 464 | 60 | hsa-miR-222-3p | [MRE11](http://www.ncbi.nlm.nih.gov/entrez/query.fcgi?db=gene&cmd=Retrieve&dopt=full_report&list_uids=4361) | MRE11 homolog, double strand break repair nuclease |
| [Details](http://mirdb.org/cgi-bin/target_detail.cgi?targetID=2444539) | 465 | 60 | hsa-miR-222-3p | [GPM6A](http://www.ncbi.nlm.nih.gov/entrez/query.fcgi?db=gene&cmd=Retrieve&dopt=full_report&list_uids=2823) | glycoprotein M6A |
| [Details](http://mirdb.org/cgi-bin/target_detail.cgi?targetID=2443951) | 466 | 60 | hsa-miR-222-3p | [DCAF7](http://www.ncbi.nlm.nih.gov/entrez/query.fcgi?db=gene&cmd=Retrieve&dopt=full_report&list_uids=10238) | DDB1 and CUL4 associated factor 7 |
| [Details](http://mirdb.org/cgi-bin/target_detail.cgi?targetID=2444439) | 467 | 60 | hsa-miR-222-3p | [ZNF547](http://www.ncbi.nlm.nih.gov/entrez/query.fcgi?db=gene&cmd=Retrieve&dopt=full_report&list_uids=284306) | zinc finger protein 547 |
| [Details](http://mirdb.org/cgi-bin/target_detail.cgi?targetID=2444448) | 468 | 60 | hsa-miR-222-3p | [BRD1](http://www.ncbi.nlm.nih.gov/entrez/query.fcgi?db=gene&cmd=Retrieve&dopt=full_report&list_uids=23774) | bromodomain containing 1 |
| [Details](http://mirdb.org/cgi-bin/target_detail.cgi?targetID=2443966) | 469 | 60 | hsa-miR-222-3p | [ECPAS](http://www.ncbi.nlm.nih.gov/entrez/query.fcgi?db=gene&cmd=Retrieve&dopt=full_report&list_uids=23392) | Ecm29 proteasome adaptor and scaffold |
| [Details](http://mirdb.org/cgi-bin/target_detail.cgi?targetID=2443993) | 470 | 60 | hsa-miR-222-3p | [GRM1](http://www.ncbi.nlm.nih.gov/entrez/query.fcgi?db=gene&cmd=Retrieve&dopt=full_report&list_uids=2911) | glutamate metabotropic receptor 1 |
| [Details](http://mirdb.org/cgi-bin/target_detail.cgi?targetID=2444012) | 471 | 60 | hsa-miR-222-3p | [LDHAL6B](http://www.ncbi.nlm.nih.gov/entrez/query.fcgi?db=gene&cmd=Retrieve&dopt=full_report&list_uids=92483) | lactate dehydrogenase A like 6B |
| [Details](http://mirdb.org/cgi-bin/target_detail.cgi?targetID=2444479) | 472 | 60 | hsa-miR-222-3p | [ANKIB1](http://www.ncbi.nlm.nih.gov/entrez/query.fcgi?db=gene&cmd=Retrieve&dopt=full_report&list_uids=54467) | ankyrin repeat and IBR domain containing 1 |
| [Details](http://mirdb.org/cgi-bin/target_detail.cgi?targetID=2444156) | 473 | 60 | hsa-miR-222-3p | [FBN2](http://www.ncbi.nlm.nih.gov/entrez/query.fcgi?db=gene&cmd=Retrieve&dopt=full_report&list_uids=2201) | fibrillin 2 |
| [Details](http://mirdb.org/cgi-bin/target_detail.cgi?targetID=2444270) | 474 | 60 | hsa-miR-222-3p | [SLC25A37](http://www.ncbi.nlm.nih.gov/entrez/query.fcgi?db=gene&cmd=Retrieve&dopt=full_report&list_uids=51312) | solute carrier family 25 member 37 |
| [Details](http://mirdb.org/cgi-bin/target_detail.cgi?targetID=2444327) | 475 | 60 | hsa-miR-222-3p | [MYO10](http://www.ncbi.nlm.nih.gov/entrez/query.fcgi?db=gene&cmd=Retrieve&dopt=full_report&list_uids=4651) | myosin X |
| [Details](http://mirdb.org/cgi-bin/target_detail.cgi?targetID=2444166) | 476 | 60 | hsa-miR-222-3p | [CSTF2T](http://www.ncbi.nlm.nih.gov/entrez/query.fcgi?db=gene&cmd=Retrieve&dopt=full_report&list_uids=23283) | cleavage stimulation factor subunit 2 tau variant |
| [Details](http://mirdb.org/cgi-bin/target_detail.cgi?targetID=2444309) | 477 | 59 | hsa-miR-222-3p | [CNPY2](http://www.ncbi.nlm.nih.gov/entrez/query.fcgi?db=gene&cmd=Retrieve&dopt=full_report&list_uids=10330) | canopy FGF signaling regulator 2 |
| [Details](http://mirdb.org/cgi-bin/target_detail.cgi?targetID=2444036) | 478 | 59 | hsa-miR-222-3p | [MRAP](http://www.ncbi.nlm.nih.gov/entrez/query.fcgi?db=gene&cmd=Retrieve&dopt=full_report&list_uids=56246) | melanocortin 2 receptor accessory protein |
| [Details](http://mirdb.org/cgi-bin/target_detail.cgi?targetID=2444032) | 479 | 59 | hsa-miR-222-3p | [PALM2](http://www.ncbi.nlm.nih.gov/entrez/query.fcgi?db=gene&cmd=Retrieve&dopt=full_report&list_uids=114299) | paralemmin 2 |
| [Details](http://mirdb.org/cgi-bin/target_detail.cgi?targetID=2444313) | 480 | 59 | hsa-miR-222-3p | [CDK8](http://www.ncbi.nlm.nih.gov/entrez/query.fcgi?db=gene&cmd=Retrieve&dopt=full_report&list_uids=1024) | cyclin dependent kinase 8 |
| [Details](http://mirdb.org/cgi-bin/target_detail.cgi?targetID=2443986) | 481 | 59 | hsa-miR-222-3p | [PRUNE1](http://www.ncbi.nlm.nih.gov/entrez/query.fcgi?db=gene&cmd=Retrieve&dopt=full_report&list_uids=58497) | prune exopolyphosphatase 1 |
| [Details](http://mirdb.org/cgi-bin/target_detail.cgi?targetID=2444512) | 482 | 59 | hsa-miR-222-3p | [ACADM](http://www.ncbi.nlm.nih.gov/entrez/query.fcgi?db=gene&cmd=Retrieve&dopt=full_report&list_uids=34) | acyl-CoA dehydrogenase medium chain |
| [Details](http://mirdb.org/cgi-bin/target_detail.cgi?targetID=2444220) | 483 | 59 | hsa-miR-222-3p | [ACTC1](http://www.ncbi.nlm.nih.gov/entrez/query.fcgi?db=gene&cmd=Retrieve&dopt=full_report&list_uids=70) | actin, alpha, cardiac muscle 1 |
| [Details](http://mirdb.org/cgi-bin/target_detail.cgi?targetID=2444214) | 484 | 59 | hsa-miR-222-3p | [MAGEL2](http://www.ncbi.nlm.nih.gov/entrez/query.fcgi?db=gene&cmd=Retrieve&dopt=full_report&list_uids=54551) | MAGE family member L2 |
| [Details](http://mirdb.org/cgi-bin/target_detail.cgi?targetID=2443945) | 485 | 59 | hsa-miR-222-3p | [IFRD1](http://www.ncbi.nlm.nih.gov/entrez/query.fcgi?db=gene&cmd=Retrieve&dopt=full_report&list_uids=3475) | interferon related developmental regulator 1 |
| [Details](http://mirdb.org/cgi-bin/target_detail.cgi?targetID=2444349) | 486 | 58 | hsa-miR-222-3p | [NDFIP1](http://www.ncbi.nlm.nih.gov/entrez/query.fcgi?db=gene&cmd=Retrieve&dopt=full_report&list_uids=80762) | Nedd4 family interacting protein 1 |
| [Details](http://mirdb.org/cgi-bin/target_detail.cgi?targetID=2444151) | 487 | 58 | hsa-miR-222-3p | [PTPRM](http://www.ncbi.nlm.nih.gov/entrez/query.fcgi?db=gene&cmd=Retrieve&dopt=full_report&list_uids=5797) | protein tyrosine phosphatase, receptor type M |
| [Details](http://mirdb.org/cgi-bin/target_detail.cgi?targetID=2444382) | 488 | 58 | hsa-miR-222-3p | [NDUFA1](http://www.ncbi.nlm.nih.gov/entrez/query.fcgi?db=gene&cmd=Retrieve&dopt=full_report&list_uids=4694) | NADH:ubiquinone oxidoreductase subunit A1 |
| [Details](http://mirdb.org/cgi-bin/target_detail.cgi?targetID=2444517) | 489 | 58 | hsa-miR-222-3p | [ENTPD7](http://www.ncbi.nlm.nih.gov/entrez/query.fcgi?db=gene&cmd=Retrieve&dopt=full_report&list_uids=57089) | ectonucleoside triphosphate diphosphohydrolase 7 |
| [Details](http://mirdb.org/cgi-bin/target_detail.cgi?targetID=2444437) | 490 | 58 | hsa-miR-222-3p | [PLXDC2](http://www.ncbi.nlm.nih.gov/entrez/query.fcgi?db=gene&cmd=Retrieve&dopt=full_report&list_uids=84898) | plexin domain containing 2 |
| [Details](http://mirdb.org/cgi-bin/target_detail.cgi?targetID=2443946) | 491 | 58 | hsa-miR-222-3p | [C16orf45](http://www.ncbi.nlm.nih.gov/entrez/query.fcgi?db=gene&cmd=Retrieve&dopt=full_report&list_uids=89927) | chromosome 16 open reading frame 45 |
| [Details](http://mirdb.org/cgi-bin/target_detail.cgi?targetID=2444314) | 492 | 58 | hsa-miR-222-3p | [AADAC](http://www.ncbi.nlm.nih.gov/entrez/query.fcgi?db=gene&cmd=Retrieve&dopt=full_report&list_uids=13) | arylacetamide deacetylase |
| [Details](http://mirdb.org/cgi-bin/target_detail.cgi?targetID=2444276) | 493 | 58 | hsa-miR-222-3p | [TMEM165](http://www.ncbi.nlm.nih.gov/entrez/query.fcgi?db=gene&cmd=Retrieve&dopt=full_report&list_uids=55858) | transmembrane protein 165 |
| [Details](http://mirdb.org/cgi-bin/target_detail.cgi?targetID=2444445) | 494 | 58 | hsa-miR-222-3p | [CTTN](http://www.ncbi.nlm.nih.gov/entrez/query.fcgi?db=gene&cmd=Retrieve&dopt=full_report&list_uids=2017) | cortactin |
| [Details](http://mirdb.org/cgi-bin/target_detail.cgi?targetID=2444119) | 495 | 58 | hsa-miR-222-3p | [RETREG1](http://www.ncbi.nlm.nih.gov/entrez/query.fcgi?db=gene&cmd=Retrieve&dopt=full_report&list_uids=54463) | reticulophagy regulator 1 |
| [Details](http://mirdb.org/cgi-bin/target_detail.cgi?targetID=2444049) | 496 | 58 | hsa-miR-222-3p | [HLA-F](http://www.ncbi.nlm.nih.gov/entrez/query.fcgi?db=gene&cmd=Retrieve&dopt=full_report&list_uids=3134) | major histocompatibility complex, class I, F |
| [Details](http://mirdb.org/cgi-bin/target_detail.cgi?targetID=2444064) | 497 | 57 | hsa-miR-222-3p | [HEXIM1](http://www.ncbi.nlm.nih.gov/entrez/query.fcgi?db=gene&cmd=Retrieve&dopt=full_report&list_uids=10614) | HEXIM P-TEFb complex subunit 1 |
| [Details](http://mirdb.org/cgi-bin/target_detail.cgi?targetID=2444328) | 498 | 57 | hsa-miR-222-3p | [HTT](http://www.ncbi.nlm.nih.gov/entrez/query.fcgi?db=gene&cmd=Retrieve&dopt=full_report&list_uids=3064) | huntingtin |
| [Details](http://mirdb.org/cgi-bin/target_detail.cgi?targetID=2444039) | 499 | 57 | hsa-miR-222-3p | [IPO5](http://www.ncbi.nlm.nih.gov/entrez/query.fcgi?db=gene&cmd=Retrieve&dopt=full_report&list_uids=3843) | importin 5 |
| [Details](http://mirdb.org/cgi-bin/target_detail.cgi?targetID=2444492) | 500 | 57 | hsa-miR-222-3p | [SOX11](http://www.ncbi.nlm.nih.gov/entrez/query.fcgi?db=gene&cmd=Retrieve&dopt=full_report&list_uids=6664) | SRY-box 11 |
| [Details](http://mirdb.org/cgi-bin/target_detail.cgi?targetID=2444458) | 501 | 57 | hsa-miR-222-3p | [SLC25A46](http://www.ncbi.nlm.nih.gov/entrez/query.fcgi?db=gene&cmd=Retrieve&dopt=full_report&list_uids=91137) | solute carrier family 25 member 46 |
| [Details](http://mirdb.org/cgi-bin/target_detail.cgi?targetID=2444357) | 502 | 57 | hsa-miR-222-3p | [SPATS2L](http://www.ncbi.nlm.nih.gov/entrez/query.fcgi?db=gene&cmd=Retrieve&dopt=full_report&list_uids=26010) | spermatogenesis associated serine rich 2 like |
| [Details](http://mirdb.org/cgi-bin/target_detail.cgi?targetID=2444084) | 503 | 57 | hsa-miR-222-3p | [KCNK2](http://www.ncbi.nlm.nih.gov/entrez/query.fcgi?db=gene&cmd=Retrieve&dopt=full_report&list_uids=3776) | potassium two pore domain channel subfamily K member 2 |
| [Details](http://mirdb.org/cgi-bin/target_detail.cgi?targetID=2444374) | 504 | 57 | hsa-miR-222-3p | [SATB1](http://www.ncbi.nlm.nih.gov/entrez/query.fcgi?db=gene&cmd=Retrieve&dopt=full_report&list_uids=6304) | SATB homeobox 1 |
| [Details](http://mirdb.org/cgi-bin/target_detail.cgi?targetID=2444050) | 505 | 57 | hsa-miR-222-3p | [CTCF](http://www.ncbi.nlm.nih.gov/entrez/query.fcgi?db=gene&cmd=Retrieve&dopt=full_report&list_uids=10664) | CCCTC-binding factor |
| [Details](http://mirdb.org/cgi-bin/target_detail.cgi?targetID=2443982) | 506 | 57 | hsa-miR-222-3p | [OSBPL3](http://www.ncbi.nlm.nih.gov/entrez/query.fcgi?db=gene&cmd=Retrieve&dopt=full_report&list_uids=26031) | oxysterol binding protein like 3 |
| [Details](http://mirdb.org/cgi-bin/target_detail.cgi?targetID=2444395) | 507 | 57 | hsa-miR-222-3p | [GEMIN2](http://www.ncbi.nlm.nih.gov/entrez/query.fcgi?db=gene&cmd=Retrieve&dopt=full_report&list_uids=8487) | gem nuclear organelle associated protein 2 |
| [Details](http://mirdb.org/cgi-bin/target_detail.cgi?targetID=2444290) | 508 | 57 | hsa-miR-222-3p | [SLC40A1](http://www.ncbi.nlm.nih.gov/entrez/query.fcgi?db=gene&cmd=Retrieve&dopt=full_report&list_uids=30061) | solute carrier family 40 member 1 |
| [Details](http://mirdb.org/cgi-bin/target_detail.cgi?targetID=2444244) | 509 | 57 | hsa-miR-222-3p | [LRRN1](http://www.ncbi.nlm.nih.gov/entrez/query.fcgi?db=gene&cmd=Retrieve&dopt=full_report&list_uids=57633) | leucine rich repeat neuronal 1 |
| [Details](http://mirdb.org/cgi-bin/target_detail.cgi?targetID=2444553) | 510 | 57 | hsa-miR-222-3p | [DEPDC4](http://www.ncbi.nlm.nih.gov/entrez/query.fcgi?db=gene&cmd=Retrieve&dopt=full_report&list_uids=120863) | DEP domain containing 4 |
| [Details](http://mirdb.org/cgi-bin/target_detail.cgi?targetID=2444071) | 511 | 57 | hsa-miR-222-3p | [OLA1](http://www.ncbi.nlm.nih.gov/entrez/query.fcgi?db=gene&cmd=Retrieve&dopt=full_report&list_uids=29789) | Obg like ATPase 1 |
| [Details](http://mirdb.org/cgi-bin/target_detail.cgi?targetID=2444252) | 512 | 57 | hsa-miR-222-3p | [FKTN](http://www.ncbi.nlm.nih.gov/entrez/query.fcgi?db=gene&cmd=Retrieve&dopt=full_report&list_uids=2218) | fukutin |
| [Details](http://mirdb.org/cgi-bin/target_detail.cgi?targetID=2444025) | 513 | 57 | hsa-miR-222-3p | [KLF3](http://www.ncbi.nlm.nih.gov/entrez/query.fcgi?db=gene&cmd=Retrieve&dopt=full_report&list_uids=51274) | Kruppel like factor 3 |
| [Details](http://mirdb.org/cgi-bin/target_detail.cgi?targetID=2444341) | 514 | 56 | hsa-miR-222-3p | [PRKAA2](http://www.ncbi.nlm.nih.gov/entrez/query.fcgi?db=gene&cmd=Retrieve&dopt=full_report&list_uids=5563) | protein kinase AMP-activated catalytic subunit alpha 2 |
| [Details](http://mirdb.org/cgi-bin/target_detail.cgi?targetID=2444306) | 515 | 56 | hsa-miR-222-3p | [PGGT1B](http://www.ncbi.nlm.nih.gov/entrez/query.fcgi?db=gene&cmd=Retrieve&dopt=full_report&list_uids=5229) | protein geranylgeranyltransferase type I subunit beta |
| [Details](http://mirdb.org/cgi-bin/target_detail.cgi?targetID=2444186) | 516 | 56 | hsa-miR-222-3p | [BBS4](http://www.ncbi.nlm.nih.gov/entrez/query.fcgi?db=gene&cmd=Retrieve&dopt=full_report&list_uids=585) | Bardet-Biedl syndrome 4 |
| [Details](http://mirdb.org/cgi-bin/target_detail.cgi?targetID=2444265) | 517 | 56 | hsa-miR-222-3p | [FIGNL2](http://www.ncbi.nlm.nih.gov/entrez/query.fcgi?db=gene&cmd=Retrieve&dopt=full_report&list_uids=401720) | fidgetin like 2 |
| [Details](http://mirdb.org/cgi-bin/target_detail.cgi?targetID=2443984) | 518 | 56 | hsa-miR-222-3p | [RREB1](http://www.ncbi.nlm.nih.gov/entrez/query.fcgi?db=gene&cmd=Retrieve&dopt=full_report&list_uids=6239) | ras responsive element binding protein 1 |
| [Details](http://mirdb.org/cgi-bin/target_detail.cgi?targetID=2444399) | 519 | 56 | hsa-miR-222-3p | [RBM18](http://www.ncbi.nlm.nih.gov/entrez/query.fcgi?db=gene&cmd=Retrieve&dopt=full_report&list_uids=92400) | RNA binding motif protein 18 |
| [Details](http://mirdb.org/cgi-bin/target_detail.cgi?targetID=2444326) | 520 | 56 | hsa-miR-222-3p | [ZNF518B](http://www.ncbi.nlm.nih.gov/entrez/query.fcgi?db=gene&cmd=Retrieve&dopt=full_report&list_uids=85460) | zinc finger protein 518B |
| [Details](http://mirdb.org/cgi-bin/target_detail.cgi?targetID=2443999) | 521 | 56 | hsa-miR-222-3p | [MAP3K10](http://www.ncbi.nlm.nih.gov/entrez/query.fcgi?db=gene&cmd=Retrieve&dopt=full_report&list_uids=4294) | mitogen-activated protein kinase kinase kinase 10 |
| [Details](http://mirdb.org/cgi-bin/target_detail.cgi?targetID=2444381) | 522 | 56 | hsa-miR-222-3p | [TDRKH](http://www.ncbi.nlm.nih.gov/entrez/query.fcgi?db=gene&cmd=Retrieve&dopt=full_report&list_uids=11022) | tudor and KH domain containing |
| [Details](http://mirdb.org/cgi-bin/target_detail.cgi?targetID=2444329) | 523 | 56 | hsa-miR-222-3p | [MAPK8](http://www.ncbi.nlm.nih.gov/entrez/query.fcgi?db=gene&cmd=Retrieve&dopt=full_report&list_uids=5599) | mitogen-activated protein kinase 8 |
| [Details](http://mirdb.org/cgi-bin/target_detail.cgi?targetID=2444160) | 524 | 56 | hsa-miR-222-3p | [GALC](http://www.ncbi.nlm.nih.gov/entrez/query.fcgi?db=gene&cmd=Retrieve&dopt=full_report&list_uids=2581) | galactosylceramidase |
| [Details](http://mirdb.org/cgi-bin/target_detail.cgi?targetID=2444436) | 525 | 56 | hsa-miR-222-3p | [FOS](http://www.ncbi.nlm.nih.gov/entrez/query.fcgi?db=gene&cmd=Retrieve&dopt=full_report&list_uids=2353) | Fos proto-oncogene, AP-1 transcription factor subunit |
| [Details](http://mirdb.org/cgi-bin/target_detail.cgi?targetID=2444083) | 526 | 56 | hsa-miR-222-3p | [SLITRK6](http://www.ncbi.nlm.nih.gov/entrez/query.fcgi?db=gene&cmd=Retrieve&dopt=full_report&list_uids=84189) | SLIT and NTRK like family member 6 |
| [Details](http://mirdb.org/cgi-bin/target_detail.cgi?targetID=2444293) | 527 | 56 | hsa-miR-222-3p | [MCMDC2](http://www.ncbi.nlm.nih.gov/entrez/query.fcgi?db=gene&cmd=Retrieve&dopt=full_report&list_uids=157777) | minichromosome maintenance domain containing 2 |
| [Details](http://mirdb.org/cgi-bin/target_detail.cgi?targetID=2444288) | 528 | 55 | hsa-miR-222-3p | [NAV3](http://www.ncbi.nlm.nih.gov/entrez/query.fcgi?db=gene&cmd=Retrieve&dopt=full_report&list_uids=89795) | neuron navigator 3 |
| [Details](http://mirdb.org/cgi-bin/target_detail.cgi?targetID=2444041) | 529 | 55 | hsa-miR-222-3p | [COX15](http://www.ncbi.nlm.nih.gov/entrez/query.fcgi?db=gene&cmd=Retrieve&dopt=full_report&list_uids=1355) | cytochrome c oxidase assembly homolog COX15 |
| [Details](http://mirdb.org/cgi-bin/target_detail.cgi?targetID=2444340) | 530 | 55 | hsa-miR-222-3p | [PAFAH1B2](http://www.ncbi.nlm.nih.gov/entrez/query.fcgi?db=gene&cmd=Retrieve&dopt=full_report&list_uids=5049) | platelet activating factor acetylhydrolase 1b catalytic subunit 2 |
| [Details](http://mirdb.org/cgi-bin/target_detail.cgi?targetID=2443947) | 531 | 55 | hsa-miR-222-3p | [MAT2A](http://www.ncbi.nlm.nih.gov/entrez/query.fcgi?db=gene&cmd=Retrieve&dopt=full_report&list_uids=4144) | methionine adenosyltransferase 2A |
| [Details](http://mirdb.org/cgi-bin/target_detail.cgi?targetID=2444237) | 532 | 55 | hsa-miR-222-3p | [ETS2](http://www.ncbi.nlm.nih.gov/entrez/query.fcgi?db=gene&cmd=Retrieve&dopt=full_report&list_uids=2114) | ETS proto-oncogene 2, transcription factor |
| [Details](http://mirdb.org/cgi-bin/target_detail.cgi?targetID=2444484) | 533 | 55 | hsa-miR-222-3p | [ADHFE1](http://www.ncbi.nlm.nih.gov/entrez/query.fcgi?db=gene&cmd=Retrieve&dopt=full_report&list_uids=137872) | alcohol dehydrogenase, iron containing 1 |
| [Details](http://mirdb.org/cgi-bin/target_detail.cgi?targetID=2444515) | 534 | 55 | hsa-miR-222-3p | [ATP2B3](http://www.ncbi.nlm.nih.gov/entrez/query.fcgi?db=gene&cmd=Retrieve&dopt=full_report&list_uids=492) | ATPase plasma membrane Ca2+ transporting 3 |
| [Details](http://mirdb.org/cgi-bin/target_detail.cgi?targetID=2444051) | 535 | 55 | hsa-miR-222-3p | [KBTBD8](http://www.ncbi.nlm.nih.gov/entrez/query.fcgi?db=gene&cmd=Retrieve&dopt=full_report&list_uids=84541) | kelch repeat and BTB domain containing 8 |
| [Details](http://mirdb.org/cgi-bin/target_detail.cgi?targetID=2444152) | 536 | 55 | hsa-miR-222-3p | [DDX42](http://www.ncbi.nlm.nih.gov/entrez/query.fcgi?db=gene&cmd=Retrieve&dopt=full_report&list_uids=11325) | DEAD-box helicase 42 |
| [Details](http://mirdb.org/cgi-bin/target_detail.cgi?targetID=2444141) | 537 | 55 | hsa-miR-222-3p | [ZNF595](http://www.ncbi.nlm.nih.gov/entrez/query.fcgi?db=gene&cmd=Retrieve&dopt=full_report&list_uids=152687) | zinc finger protein 595 |
| [Details](http://mirdb.org/cgi-bin/target_detail.cgi?targetID=2444159) | 538 | 55 | hsa-miR-222-3p | [ASB7](http://www.ncbi.nlm.nih.gov/entrez/query.fcgi?db=gene&cmd=Retrieve&dopt=full_report&list_uids=140460) | ankyrin repeat and SOCS box containing 7 |
| [Details](http://mirdb.org/cgi-bin/target_detail.cgi?targetID=2444148) | 539 | 55 | hsa-miR-222-3p | [RAB14](http://www.ncbi.nlm.nih.gov/entrez/query.fcgi?db=gene&cmd=Retrieve&dopt=full_report&list_uids=51552) | RAB14, member RAS oncogene family |
| [Details](http://mirdb.org/cgi-bin/target_detail.cgi?targetID=2444118) | 540 | 55 | hsa-miR-222-3p | [CXCL11](http://www.ncbi.nlm.nih.gov/entrez/query.fcgi?db=gene&cmd=Retrieve&dopt=full_report&list_uids=6373) | C-X-C motif chemokine ligand 11 |
| [Details](http://mirdb.org/cgi-bin/target_detail.cgi?targetID=2444175) | 541 | 55 | hsa-miR-222-3p | [PDZRN4](http://www.ncbi.nlm.nih.gov/entrez/query.fcgi?db=gene&cmd=Retrieve&dopt=full_report&list_uids=29951) | PDZ domain containing ring finger 4 |
| [Details](http://mirdb.org/cgi-bin/target_detail.cgi?targetID=2444158) | 542 | 55 | hsa-miR-222-3p | [GTF2A1](http://www.ncbi.nlm.nih.gov/entrez/query.fcgi?db=gene&cmd=Retrieve&dopt=full_report&list_uids=2957) | general transcription factor IIA subunit 1 |
| [Details](http://mirdb.org/cgi-bin/target_detail.cgi?targetID=2444360) | 543 | 55 | hsa-miR-222-3p | [TAOK1](http://www.ncbi.nlm.nih.gov/entrez/query.fcgi?db=gene&cmd=Retrieve&dopt=full_report&list_uids=57551) | TAO kinase 1 |
| [Details](http://mirdb.org/cgi-bin/target_detail.cgi?targetID=2444097) | 544 | 55 | hsa-miR-222-3p | [STMN1](http://www.ncbi.nlm.nih.gov/entrez/query.fcgi?db=gene&cmd=Retrieve&dopt=full_report&list_uids=3925) | stathmin 1 |
| [Details](http://mirdb.org/cgi-bin/target_detail.cgi?targetID=2444204) | 545 | 55 | hsa-miR-222-3p | [CASP3](http://www.ncbi.nlm.nih.gov/entrez/query.fcgi?db=gene&cmd=Retrieve&dopt=full_report&list_uids=836) | caspase 3 |
| [Details](http://mirdb.org/cgi-bin/target_detail.cgi?targetID=2444167) | 546 | 55 | hsa-miR-222-3p | [BCHE](http://www.ncbi.nlm.nih.gov/entrez/query.fcgi?db=gene&cmd=Retrieve&dopt=full_report&list_uids=590) | butyrylcholinesterase |
| [Details](http://mirdb.org/cgi-bin/target_detail.cgi?targetID=2444130) | 547 | 55 | hsa-miR-222-3p | [ARID1A](http://www.ncbi.nlm.nih.gov/entrez/query.fcgi?db=gene&cmd=Retrieve&dopt=full_report&list_uids=8289) | AT-rich interaction domain 1A |
| [Details](http://mirdb.org/cgi-bin/target_detail.cgi?targetID=2444525) | 548 | 54 | hsa-miR-222-3p | [KLHL18](http://www.ncbi.nlm.nih.gov/entrez/query.fcgi?db=gene&cmd=Retrieve&dopt=full_report&list_uids=23276) | kelch like family member 18 |
| [Details](http://mirdb.org/cgi-bin/target_detail.cgi?targetID=2444533) | 549 | 54 | hsa-miR-222-3p | [PITPNM2](http://www.ncbi.nlm.nih.gov/entrez/query.fcgi?db=gene&cmd=Retrieve&dopt=full_report&list_uids=57605) | phosphatidylinositol transfer protein membrane associated 2 |
| [Details](http://mirdb.org/cgi-bin/target_detail.cgi?targetID=2444530) | 550 | 54 | hsa-miR-222-3p | [VEZF1](http://www.ncbi.nlm.nih.gov/entrez/query.fcgi?db=gene&cmd=Retrieve&dopt=full_report&list_uids=7716) | vascular endothelial zinc finger 1 |
| [Details](http://mirdb.org/cgi-bin/target_detail.cgi?targetID=2444541) | 551 | 54 | hsa-miR-222-3p | [RNF44](http://www.ncbi.nlm.nih.gov/entrez/query.fcgi?db=gene&cmd=Retrieve&dopt=full_report&list_uids=22838) | ring finger protein 44 |
| [Details](http://mirdb.org/cgi-bin/target_detail.cgi?targetID=2444417) | 552 | 54 | hsa-miR-222-3p | [GTF2B](http://www.ncbi.nlm.nih.gov/entrez/query.fcgi?db=gene&cmd=Retrieve&dopt=full_report&list_uids=2959) | general transcription factor IIB |
| [Details](http://mirdb.org/cgi-bin/target_detail.cgi?targetID=2444264) | 553 | 54 | hsa-miR-222-3p | [SLC6A9](http://www.ncbi.nlm.nih.gov/entrez/query.fcgi?db=gene&cmd=Retrieve&dopt=full_report&list_uids=6536) | solute carrier family 6 member 9 |
| [Details](http://mirdb.org/cgi-bin/target_detail.cgi?targetID=2444346) | 554 | 54 | hsa-miR-222-3p | [MTMR6](http://www.ncbi.nlm.nih.gov/entrez/query.fcgi?db=gene&cmd=Retrieve&dopt=full_report&list_uids=9107) | myotubularin related protein 6 |
| [Details](http://mirdb.org/cgi-bin/target_detail.cgi?targetID=2443989) | 555 | 54 | hsa-miR-222-3p | [RAB3GAP2](http://www.ncbi.nlm.nih.gov/entrez/query.fcgi?db=gene&cmd=Retrieve&dopt=full_report&list_uids=25782) | RAB3 GTPase activating non-catalytic protein subunit 2 |
| [Details](http://mirdb.org/cgi-bin/target_detail.cgi?targetID=2444218) | 556 | 54 | hsa-miR-222-3p | [LIFR](http://www.ncbi.nlm.nih.gov/entrez/query.fcgi?db=gene&cmd=Retrieve&dopt=full_report&list_uids=3977) | LIF receptor alpha |
| [Details](http://mirdb.org/cgi-bin/target_detail.cgi?targetID=2444348) | 557 | 54 | hsa-miR-222-3p | [SLC45A3](http://www.ncbi.nlm.nih.gov/entrez/query.fcgi?db=gene&cmd=Retrieve&dopt=full_report&list_uids=85414) | solute carrier family 45 member 3 |
| [Details](http://mirdb.org/cgi-bin/target_detail.cgi?targetID=2443990) | 558 | 53 | hsa-miR-222-3p | [CHORDC1](http://www.ncbi.nlm.nih.gov/entrez/query.fcgi?db=gene&cmd=Retrieve&dopt=full_report&list_uids=26973) | cysteine and histidine rich domain containing 1 |
| [Details](http://mirdb.org/cgi-bin/target_detail.cgi?targetID=2444396) | 559 | 53 | hsa-miR-222-3p | [PRAMEF14](http://www.ncbi.nlm.nih.gov/entrez/query.fcgi?db=gene&cmd=Retrieve&dopt=full_report&list_uids=729528) | PRAME family member 14 |
| [Details](http://mirdb.org/cgi-bin/target_detail.cgi?targetID=2444113) | 560 | 53 | hsa-miR-222-3p | [USP49](http://www.ncbi.nlm.nih.gov/entrez/query.fcgi?db=gene&cmd=Retrieve&dopt=full_report&list_uids=25862) | ubiquitin specific peptidase 49 |
| [Details](http://mirdb.org/cgi-bin/target_detail.cgi?targetID=2444173) | 561 | 53 | hsa-miR-222-3p | [RANBP2](http://www.ncbi.nlm.nih.gov/entrez/query.fcgi?db=gene&cmd=Retrieve&dopt=full_report&list_uids=5903) | RAN binding protein 2 |
| [Details](http://mirdb.org/cgi-bin/target_detail.cgi?targetID=2444234) | 562 | 53 | hsa-miR-222-3p | [MPRIP](http://www.ncbi.nlm.nih.gov/entrez/query.fcgi?db=gene&cmd=Retrieve&dopt=full_report&list_uids=23164) | myosin phosphatase Rho interacting protein |
| [Details](http://mirdb.org/cgi-bin/target_detail.cgi?targetID=2444389) | 563 | 53 | hsa-miR-222-3p | [C12orf77](http://www.ncbi.nlm.nih.gov/entrez/query.fcgi?db=gene&cmd=Retrieve&dopt=full_report&list_uids=196415) | chromosome 12 open reading frame 77 |
| [Details](http://mirdb.org/cgi-bin/target_detail.cgi?targetID=2444390) | 564 | 53 | hsa-miR-222-3p | [KIAA1549L](http://www.ncbi.nlm.nih.gov/entrez/query.fcgi?db=gene&cmd=Retrieve&dopt=full_report&list_uids=25758) | KIAA1549 like |
| [Details](http://mirdb.org/cgi-bin/target_detail.cgi?targetID=2444331) | 565 | 53 | hsa-miR-222-3p | [PCDH11X](http://www.ncbi.nlm.nih.gov/entrez/query.fcgi?db=gene&cmd=Retrieve&dopt=full_report&list_uids=27328) | protocadherin 11 X-linked |
| [Details](http://mirdb.org/cgi-bin/target_detail.cgi?targetID=2444187) | 566 | 53 | hsa-miR-222-3p | [SLC16A6](http://www.ncbi.nlm.nih.gov/entrez/query.fcgi?db=gene&cmd=Retrieve&dopt=full_report&list_uids=9120) | solute carrier family 16 member 6 |
| [Details](http://mirdb.org/cgi-bin/target_detail.cgi?targetID=2444287) | 567 | 53 | hsa-miR-222-3p | [MED1](http://www.ncbi.nlm.nih.gov/entrez/query.fcgi?db=gene&cmd=Retrieve&dopt=full_report&list_uids=5469) | mediator complex subunit 1 |
| [Details](http://mirdb.org/cgi-bin/target_detail.cgi?targetID=2444460) | 568 | 53 | hsa-miR-222-3p | [CD8A](http://www.ncbi.nlm.nih.gov/entrez/query.fcgi?db=gene&cmd=Retrieve&dopt=full_report&list_uids=925) | CD8a molecule |
| [Details](http://mirdb.org/cgi-bin/target_detail.cgi?targetID=2444474) | 569 | 53 | hsa-miR-222-3p | [COL6A5](http://www.ncbi.nlm.nih.gov/entrez/query.fcgi?db=gene&cmd=Retrieve&dopt=full_report&list_uids=256076) | collagen type VI alpha 5 chain |
| [Details](http://mirdb.org/cgi-bin/target_detail.cgi?targetID=2444250) | 570 | 53 | hsa-miR-222-3p | [PRAMEF13](http://www.ncbi.nlm.nih.gov/entrez/query.fcgi?db=gene&cmd=Retrieve&dopt=full_report&list_uids=400736) | PRAME family member 13 |
| [Details](http://mirdb.org/cgi-bin/target_detail.cgi?targetID=2444128) | 571 | 53 | hsa-miR-222-3p | [KCNH1](http://www.ncbi.nlm.nih.gov/entrez/query.fcgi?db=gene&cmd=Retrieve&dopt=full_report&list_uids=3756) | potassium voltage-gated channel subfamily H member 1 |
| [Details](http://mirdb.org/cgi-bin/target_detail.cgi?targetID=2444497) | 572 | 53 | hsa-miR-222-3p | [PCDH11Y](http://www.ncbi.nlm.nih.gov/entrez/query.fcgi?db=gene&cmd=Retrieve&dopt=full_report&list_uids=83259) | protocadherin 11 Y-linked |
| [Details](http://mirdb.org/cgi-bin/target_detail.cgi?targetID=2444085) | 573 | 53 | hsa-miR-222-3p | [RGS8](http://www.ncbi.nlm.nih.gov/entrez/query.fcgi?db=gene&cmd=Retrieve&dopt=full_report&list_uids=85397) | regulator of G protein signaling 8 |
| [Details](http://mirdb.org/cgi-bin/target_detail.cgi?targetID=2443985) | 574 | 52 | hsa-miR-222-3p | [GPATCH2L](http://www.ncbi.nlm.nih.gov/entrez/query.fcgi?db=gene&cmd=Retrieve&dopt=full_report&list_uids=55668) | G-patch domain containing 2 like |
| [Details](http://mirdb.org/cgi-bin/target_detail.cgi?targetID=2444519) | 575 | 52 | hsa-miR-222-3p | [DDX58](http://www.ncbi.nlm.nih.gov/entrez/query.fcgi?db=gene&cmd=Retrieve&dopt=full_report&list_uids=23586) | DExD/H-box helicase 58 |
| [Details](http://mirdb.org/cgi-bin/target_detail.cgi?targetID=2444133) | 576 | 52 | hsa-miR-222-3p | [SNCA](http://www.ncbi.nlm.nih.gov/entrez/query.fcgi?db=gene&cmd=Retrieve&dopt=full_report&list_uids=6622) | synuclein alpha |
| [Details](http://mirdb.org/cgi-bin/target_detail.cgi?targetID=2444421) | 577 | 52 | hsa-miR-222-3p | [C4orf33](http://www.ncbi.nlm.nih.gov/entrez/query.fcgi?db=gene&cmd=Retrieve&dopt=full_report&list_uids=132321) | chromosome 4 open reading frame 33 |
| [Details](http://mirdb.org/cgi-bin/target_detail.cgi?targetID=2444397) | 578 | 52 | hsa-miR-222-3p | [DYRK1A](http://www.ncbi.nlm.nih.gov/entrez/query.fcgi?db=gene&cmd=Retrieve&dopt=full_report&list_uids=1859) | dual specificity tyrosine phosphorylation regulated kinase 1A |
| [Details](http://mirdb.org/cgi-bin/target_detail.cgi?targetID=2444295) | 579 | 52 | hsa-miR-222-3p | [IL1RAP](http://www.ncbi.nlm.nih.gov/entrez/query.fcgi?db=gene&cmd=Retrieve&dopt=full_report&list_uids=3556) | interleukin 1 receptor accessory protein |
| [Details](http://mirdb.org/cgi-bin/target_detail.cgi?targetID=2444400) | 580 | 52 | hsa-miR-222-3p | [PRSS37](http://www.ncbi.nlm.nih.gov/entrez/query.fcgi?db=gene&cmd=Retrieve&dopt=full_report&list_uids=136242) | serine protease 37 |
| [Details](http://mirdb.org/cgi-bin/target_detail.cgi?targetID=2444394) | 581 | 52 | hsa-miR-222-3p | [SLAIN2](http://www.ncbi.nlm.nih.gov/entrez/query.fcgi?db=gene&cmd=Retrieve&dopt=full_report&list_uids=57606) | SLAIN motif family member 2 |
| [Details](http://mirdb.org/cgi-bin/target_detail.cgi?targetID=2444369) | 582 | 52 | hsa-miR-222-3p | [TAF9B](http://www.ncbi.nlm.nih.gov/entrez/query.fcgi?db=gene&cmd=Retrieve&dopt=full_report&list_uids=51616) | TATA-box binding protein associated factor 9b |
| [Details](http://mirdb.org/cgi-bin/target_detail.cgi?targetID=2444006) | 583 | 52 | hsa-miR-222-3p | [LRP10](http://www.ncbi.nlm.nih.gov/entrez/query.fcgi?db=gene&cmd=Retrieve&dopt=full_report&list_uids=26020) | LDL receptor related protein 10 |
| [Details](http://mirdb.org/cgi-bin/target_detail.cgi?targetID=2444046) | 584 | 52 | hsa-miR-222-3p | [KCNA1](http://www.ncbi.nlm.nih.gov/entrez/query.fcgi?db=gene&cmd=Retrieve&dopt=full_report&list_uids=3736) | potassium voltage-gated channel subfamily A member 1 |
| [Details](http://mirdb.org/cgi-bin/target_detail.cgi?targetID=2444485) | 585 | 52 | hsa-miR-222-3p | [UBN2](http://www.ncbi.nlm.nih.gov/entrez/query.fcgi?db=gene&cmd=Retrieve&dopt=full_report&list_uids=254048) | ubinuclein 2 |
| [Details](http://mirdb.org/cgi-bin/target_detail.cgi?targetID=2444000) | 586 | 52 | hsa-miR-222-3p | [CYP1B1](http://www.ncbi.nlm.nih.gov/entrez/query.fcgi?db=gene&cmd=Retrieve&dopt=full_report&list_uids=1545) | cytochrome P450 family 1 subfamily B member 1 |
| [Details](http://mirdb.org/cgi-bin/target_detail.cgi?targetID=2444055) | 587 | 52 | hsa-miR-222-3p | [WDR77](http://www.ncbi.nlm.nih.gov/entrez/query.fcgi?db=gene&cmd=Retrieve&dopt=full_report&list_uids=79084) | WD repeat domain 77 |
| [Details](http://mirdb.org/cgi-bin/target_detail.cgi?targetID=2444371) | 588 | 52 | hsa-miR-222-3p | [BCL11B](http://www.ncbi.nlm.nih.gov/entrez/query.fcgi?db=gene&cmd=Retrieve&dopt=full_report&list_uids=64919) | BCL11B, BAF complex component |
| [Details](http://mirdb.org/cgi-bin/target_detail.cgi?targetID=2444469) | 589 | 51 | hsa-miR-222-3p | [ZBTB20](http://www.ncbi.nlm.nih.gov/entrez/query.fcgi?db=gene&cmd=Retrieve&dopt=full_report&list_uids=26137) | zinc finger and BTB domain containing 20 |
| [Details](http://mirdb.org/cgi-bin/target_detail.cgi?targetID=2444547) | 590 | 51 | hsa-miR-222-3p | [C16orf82](http://www.ncbi.nlm.nih.gov/entrez/query.fcgi?db=gene&cmd=Retrieve&dopt=full_report&list_uids=162083) | chromosome 16 open reading frame 82 |
| [Details](http://mirdb.org/cgi-bin/target_detail.cgi?targetID=2444391) | 591 | 51 | hsa-miR-222-3p | [KRT81](http://www.ncbi.nlm.nih.gov/entrez/query.fcgi?db=gene&cmd=Retrieve&dopt=full_report&list_uids=3887) | keratin 81 |
| [Details](http://mirdb.org/cgi-bin/target_detail.cgi?targetID=2444464) | 592 | 51 | hsa-miR-222-3p | [GNB3](http://www.ncbi.nlm.nih.gov/entrez/query.fcgi?db=gene&cmd=Retrieve&dopt=full_report&list_uids=2784) | G protein subunit beta 3 |
| [Details](http://mirdb.org/cgi-bin/target_detail.cgi?targetID=2444077) | 593 | 51 | hsa-miR-222-3p | [DICER1](http://www.ncbi.nlm.nih.gov/entrez/query.fcgi?db=gene&cmd=Retrieve&dopt=full_report&list_uids=23405) | dicer 1, ribonuclease III |
| [Details](http://mirdb.org/cgi-bin/target_detail.cgi?targetID=2444404) | 594 | 51 | hsa-miR-222-3p | [SLC25A21](http://www.ncbi.nlm.nih.gov/entrez/query.fcgi?db=gene&cmd=Retrieve&dopt=full_report&list_uids=89874) | solute carrier family 25 member 21 |
| [Details](http://mirdb.org/cgi-bin/target_detail.cgi?targetID=2444483) | 595 | 51 | hsa-miR-222-3p | [MAP4K4](http://www.ncbi.nlm.nih.gov/entrez/query.fcgi?db=gene&cmd=Retrieve&dopt=full_report&list_uids=9448) | mitogen-activated protein kinase kinase kinase kinase 4 |
| [Details](http://mirdb.org/cgi-bin/target_detail.cgi?targetID=2444338) | 596 | 51 | hsa-miR-222-3p | [ESYT1](http://www.ncbi.nlm.nih.gov/entrez/query.fcgi?db=gene&cmd=Retrieve&dopt=full_report&list_uids=23344) | extended synaptotagmin 1 |
| [Details](http://mirdb.org/cgi-bin/target_detail.cgi?targetID=2444358) | 597 | 51 | hsa-miR-222-3p | [CHFR](http://www.ncbi.nlm.nih.gov/entrez/query.fcgi?db=gene&cmd=Retrieve&dopt=full_report&list_uids=55743) | checkpoint with forkhead and ring finger domains |
| [Details](http://mirdb.org/cgi-bin/target_detail.cgi?targetID=2444351) | 598 | 51 | hsa-miR-222-3p | [MMD2](http://www.ncbi.nlm.nih.gov/entrez/query.fcgi?db=gene&cmd=Retrieve&dopt=full_report&list_uids=221938) | monocyte to macrophage differentiation associated 2 |
| [Details](http://mirdb.org/cgi-bin/target_detail.cgi?targetID=2444427) | 599 | 51 | hsa-miR-222-3p | [MYOD1](http://www.ncbi.nlm.nih.gov/entrez/query.fcgi?db=gene&cmd=Retrieve&dopt=full_report&list_uids=4654) | myogenic differentiation 1 |
| [Details](http://mirdb.org/cgi-bin/target_detail.cgi?targetID=2444153) | 600 | 51 | hsa-miR-222-3p | [NCAM1](http://www.ncbi.nlm.nih.gov/entrez/query.fcgi?db=gene&cmd=Retrieve&dopt=full_report&list_uids=4684) | neural cell adhesion molecule 1 |
| [Details](http://mirdb.org/cgi-bin/target_detail.cgi?targetID=2444001) | 601 | 51 | hsa-miR-222-3p | [ZBTB41](http://www.ncbi.nlm.nih.gov/entrez/query.fcgi?db=gene&cmd=Retrieve&dopt=full_report&list_uids=360023) | zinc finger and BTB domain containing 41 |
| [Details](http://mirdb.org/cgi-bin/target_detail.cgi?targetID=2444172) | 602 | 51 | hsa-miR-222-3p | [MDM2](http://www.ncbi.nlm.nih.gov/entrez/query.fcgi?db=gene&cmd=Retrieve&dopt=full_report&list_uids=4193) | MDM2 proto-oncogene |
| [Details](http://mirdb.org/cgi-bin/target_detail.cgi?targetID=2444315) | 603 | 50 | hsa-miR-222-3p | [ITPRID1](http://www.ncbi.nlm.nih.gov/entrez/query.fcgi?db=gene&cmd=Retrieve&dopt=full_report&list_uids=223075) | ITPR interacting domain containing 1 |
| [Details](http://mirdb.org/cgi-bin/target_detail.cgi?targetID=2444481) | 604 | 50 | hsa-miR-222-3p | [CNKSR3](http://www.ncbi.nlm.nih.gov/entrez/query.fcgi?db=gene&cmd=Retrieve&dopt=full_report&list_uids=154043) | CNKSR family member 3 |
| [Details](http://mirdb.org/cgi-bin/target_detail.cgi?targetID=2444115) | 605 | 50 | hsa-miR-222-3p | [CT45A10](http://www.ncbi.nlm.nih.gov/entrez/query.fcgi?db=gene&cmd=Retrieve&dopt=full_report&list_uids=102723631) | cancer/testis antigen family 45 member A10 |
| [Details](http://mirdb.org/cgi-bin/target_detail.cgi?targetID=2444268) | 606 | 50 | hsa-miR-222-3p | [GCNT1](http://www.ncbi.nlm.nih.gov/entrez/query.fcgi?db=gene&cmd=Retrieve&dopt=full_report&list_uids=2650) | glucosaminyl (N-acetyl) transferase 1 |
| [Details](http://mirdb.org/cgi-bin/target_detail.cgi?targetID=2443955) | 607 | 50 | hsa-miR-222-3p | [ITIH5](http://www.ncbi.nlm.nih.gov/entrez/query.fcgi?db=gene&cmd=Retrieve&dopt=full_report&list_uids=80760) | inter-alpha-trypsin inhibitor heavy chain family member 5 |
| [Details](http://mirdb.org/cgi-bin/target_detail.cgi?targetID=2444122) | 608 | 50 | hsa-miR-222-3p | [ERI2](http://www.ncbi.nlm.nih.gov/entrez/query.fcgi?db=gene&cmd=Retrieve&dopt=full_report&list_uids=112479) | ERI1 exoribonuclease family member 2 |
| [Details](http://mirdb.org/cgi-bin/target_detail.cgi?targetID=2443964) | 609 | 50 | hsa-miR-222-3p | [PKDCC](http://www.ncbi.nlm.nih.gov/entrez/query.fcgi?db=gene&cmd=Retrieve&dopt=full_report&list_uids=91461) | protein kinase domain containing, cytoplasmic |
| [Details](http://mirdb.org/cgi-bin/target_detail.cgi?targetID=2444534) | 610 | 50 | hsa-miR-222-3p | [A4GNT](http://www.ncbi.nlm.nih.gov/entrez/query.fcgi?db=gene&cmd=Retrieve&dopt=full_report&list_uids=51146) | alpha-1,4-N-acetylglucosaminyltransferase |
| [Details](http://mirdb.org/cgi-bin/target_detail.cgi?targetID=2444081) | 611 | 50 | hsa-miR-222-3p | [AJAP1](http://www.ncbi.nlm.nih.gov/entrez/query.fcgi?db=gene&cmd=Retrieve&dopt=full_report&list_uids=55966) | adherens junctions associated protein 1 |
| [Details](http://mirdb.org/cgi-bin/target_detail.cgi?targetID=2444031) | 612 | 50 | hsa-miR-222-3p | [CT45A5](http://www.ncbi.nlm.nih.gov/entrez/query.fcgi?db=gene&cmd=Retrieve&dopt=full_report&list_uids=441521) | cancer/testis antigen family 45 member A5 |
| [Details](http://mirdb.org/cgi-bin/target_detail.cgi?targetID=2444291) | 613 | 50 | hsa-miR-222-3p | [APAF1](http://www.ncbi.nlm.nih.gov/entrez/query.fcgi?db=gene&cmd=Retrieve&dopt=full_report&list_uids=317) | apoptotic peptidase activating factor 1 |
| [Details](http://mirdb.org/cgi-bin/target_detail.cgi?targetID=2444181) | 614 | 50 | hsa-miR-222-3p | [AGO4](http://www.ncbi.nlm.nih.gov/entrez/query.fcgi?db=gene&cmd=Retrieve&dopt=full_report&list_uids=192670) | argonaute RISC catalytic component 4 |
| [Details](http://mirdb.org/cgi-bin/target_detail.cgi?targetID=2444014) | 615 | 50 | hsa-miR-222-3p | [CAVIN4](http://www.ncbi.nlm.nih.gov/entrez/query.fcgi?db=gene&cmd=Retrieve&dopt=full_report&list_uids=347273) | caveolae associated protein 4 |
| [Details](http://mirdb.org/cgi-bin/target_detail.cgi?targetID=2444076) | 616 | 50 | hsa-miR-222-3p | [CLDN12](http://www.ncbi.nlm.nih.gov/entrez/query.fcgi?db=gene&cmd=Retrieve&dopt=full_report&list_uids=9069) | claudin 12 |
| [Details](http://mirdb.org/cgi-bin/target_detail.cgi?targetID=2444529) | 617 | 50 | hsa-miR-222-3p | [STAT2](http://www.ncbi.nlm.nih.gov/entrez/query.fcgi?db=gene&cmd=Retrieve&dopt=full_report&list_uids=6773) | signal transducer and activator of transcription 2 |
| [Details](http://mirdb.org/cgi-bin/target_detail.cgi?targetID=2444216) | 618 | 50 | hsa-miR-222-3p | [SAR1A](http://www.ncbi.nlm.nih.gov/entrez/query.fcgi?db=gene&cmd=Retrieve&dopt=full_report&list_uids=56681) | secretion associated Ras related GTPase 1A |
| [Details](http://mirdb.org/cgi-bin/target_detail.cgi?targetID=2444337) | 619 | 50 | hsa-miR-222-3p | [TRAM1](http://www.ncbi.nlm.nih.gov/entrez/query.fcgi?db=gene&cmd=Retrieve&dopt=full_report&list_uids=23471) | translocation associated membrane protein 1 |

Data generated from the online database for prediction of functional microRNA targets (Chen & Wang, 2020)

Chen, Y., & Wang, X. (2020). MiRDB: An online database for prediction of functional microRNA targets. *Nucleic Acids Research*, *48*(D1), D127–D131. https://doi.org/10.1093/nar/gkz757
