# Supplementary material for: The Role of microRNAs in Organismal and Skin Aging
Source: Int J Mol Sci. 2020 Jul 25;21(15):5281. doi: 10.3390/ijms21155281 (PMC7432402; doi:10.3390/ijms21155281)
Supplement: Supplementary file 1 [file ijms-21-05281-s001.zip › Appendix 2 hsa-miR-30c-5p .docx]

**There are 1545 predicted targets for hsa-miR-30c-5p in miRDB**

| **Target Detail** | **Target Rank** | **Target Score** | **miRNA Name** | **Gene Symbol** | **Gene Description** |
| --- | --- | --- | --- | --- | --- |
| [Details](http://mirdb.org/cgi-bin/target_detail.cgi?targetID=1439577) | 1 | 100 | hsa-miR-30c-5p | [TWF1](http://www.ncbi.nlm.nih.gov/entrez/query.fcgi?db=gene&cmd=Retrieve&dopt=full_report&list_uids=5756) | twinfilin actin binding protein 1 |
| [Details](http://mirdb.org/cgi-bin/target_detail.cgi?targetID=1439725) | 2 | 100 | hsa-miR-30c-5p | [B3GNT5](http://www.ncbi.nlm.nih.gov/entrez/query.fcgi?db=gene&cmd=Retrieve&dopt=full_report&list_uids=84002) | UDP-GlcNAc:betaGal beta-1,3-N-acetylglucosaminyltransferase 5 |
| [Details](http://mirdb.org/cgi-bin/target_detail.cgi?targetID=1439867) | 3 | 100 | hsa-miR-30c-5p | [EED](http://www.ncbi.nlm.nih.gov/entrez/query.fcgi?db=gene&cmd=Retrieve&dopt=full_report&list_uids=8726) | embryonic ectoderm development |
| [Details](http://mirdb.org/cgi-bin/target_detail.cgi?targetID=1440047) | 4 | 100 | hsa-miR-30c-5p | [WDR7](http://www.ncbi.nlm.nih.gov/entrez/query.fcgi?db=gene&cmd=Retrieve&dopt=full_report&list_uids=23335) | WD repeat domain 7 |
| [Details](http://mirdb.org/cgi-bin/target_detail.cgi?targetID=1440079) | 5 | 100 | hsa-miR-30c-5p | [SCN2A](http://www.ncbi.nlm.nih.gov/entrez/query.fcgi?db=gene&cmd=Retrieve&dopt=full_report&list_uids=6326) | sodium voltage-gated channel alpha subunit 2 |
| [Details](http://mirdb.org/cgi-bin/target_detail.cgi?targetID=1440144) | 6 | 100 | hsa-miR-30c-5p | [BRWD3](http://www.ncbi.nlm.nih.gov/entrez/query.fcgi?db=gene&cmd=Retrieve&dopt=full_report&list_uids=254065) | bromodomain and WD repeat domain containing 3 |
| [Details](http://mirdb.org/cgi-bin/target_detail.cgi?targetID=1440151) | 7 | 100 | hsa-miR-30c-5p | [PTGFRN](http://www.ncbi.nlm.nih.gov/entrez/query.fcgi?db=gene&cmd=Retrieve&dopt=full_report&list_uids=5738) | prostaglandin F2 receptor inhibitor |
| [Details](http://mirdb.org/cgi-bin/target_detail.cgi?targetID=1440227) | 8 | 100 | hsa-miR-30c-5p | [DCUN1D3](http://www.ncbi.nlm.nih.gov/entrez/query.fcgi?db=gene&cmd=Retrieve&dopt=full_report&list_uids=123879) | defective in cullin neddylation 1 domain containing 3 |
| [Details](http://mirdb.org/cgi-bin/target_detail.cgi?targetID=1440235) | 9 | 100 | hsa-miR-30c-5p | [NFAT5](http://www.ncbi.nlm.nih.gov/entrez/query.fcgi?db=gene&cmd=Retrieve&dopt=full_report&list_uids=10725) | nuclear factor of activated T cells 5 |
| [Details](http://mirdb.org/cgi-bin/target_detail.cgi?targetID=1440384) | 10 | 100 | hsa-miR-30c-5p | [KLHL20](http://www.ncbi.nlm.nih.gov/entrez/query.fcgi?db=gene&cmd=Retrieve&dopt=full_report&list_uids=27252) | kelch like family member 20 |
| [Details](http://mirdb.org/cgi-bin/target_detail.cgi?targetID=1440445) | 11 | 100 | hsa-miR-30c-5p | [PPARGC1B](http://www.ncbi.nlm.nih.gov/entrez/query.fcgi?db=gene&cmd=Retrieve&dopt=full_report&list_uids=133522) | PPARG coactivator 1 beta |
| [Details](http://mirdb.org/cgi-bin/target_detail.cgi?targetID=1440447) | 12 | 100 | hsa-miR-30c-5p | [MKRN3](http://www.ncbi.nlm.nih.gov/entrez/query.fcgi?db=gene&cmd=Retrieve&dopt=full_report&list_uids=7681) | makorin ring finger protein 3 |
| [Details](http://mirdb.org/cgi-bin/target_detail.cgi?targetID=1440482) | 13 | 100 | hsa-miR-30c-5p | [ANKRA2](http://www.ncbi.nlm.nih.gov/entrez/query.fcgi?db=gene&cmd=Retrieve&dopt=full_report&list_uids=57763) | ankyrin repeat family A member 2 |
| [Details](http://mirdb.org/cgi-bin/target_detail.cgi?targetID=1440523) | 14 | 100 | hsa-miR-30c-5p | [LIN28B](http://www.ncbi.nlm.nih.gov/entrez/query.fcgi?db=gene&cmd=Retrieve&dopt=full_report&list_uids=389421) | lin-28 homolog B |
| [Details](http://mirdb.org/cgi-bin/target_detail.cgi?targetID=1440794) | 15 | 100 | hsa-miR-30c-5p | [STOX2](http://www.ncbi.nlm.nih.gov/entrez/query.fcgi?db=gene&cmd=Retrieve&dopt=full_report&list_uids=56977) | storkhead box 2 |
| [Details](http://mirdb.org/cgi-bin/target_detail.cgi?targetID=1440913) | 16 | 100 | hsa-miR-30c-5p | [RORA](http://www.ncbi.nlm.nih.gov/entrez/query.fcgi?db=gene&cmd=Retrieve&dopt=full_report&list_uids=6095) | RAR related orphan receptor A |
| [Details](http://mirdb.org/cgi-bin/target_detail.cgi?targetID=1440933) | 17 | 100 | hsa-miR-30c-5p | [FZD3](http://www.ncbi.nlm.nih.gov/entrez/query.fcgi?db=gene&cmd=Retrieve&dopt=full_report&list_uids=7976) | frizzled class receptor 3 |
| [Details](http://mirdb.org/cgi-bin/target_detail.cgi?targetID=1440973) | 18 | 100 | hsa-miR-30c-5p | [CELSR3](http://www.ncbi.nlm.nih.gov/entrez/query.fcgi?db=gene&cmd=Retrieve&dopt=full_report&list_uids=1951) | cadherin EGF LAG seven-pass G-type receptor 3 |
| [Details](http://mirdb.org/cgi-bin/target_detail.cgi?targetID=1440985) | 19 | 100 | hsa-miR-30c-5p | [MIER3](http://www.ncbi.nlm.nih.gov/entrez/query.fcgi?db=gene&cmd=Retrieve&dopt=full_report&list_uids=166968) | MIER family member 3 |
| [Details](http://mirdb.org/cgi-bin/target_detail.cgi?targetID=1439471) | 20 | 99 | hsa-miR-30c-5p | [LCLAT1](http://www.ncbi.nlm.nih.gov/entrez/query.fcgi?db=gene&cmd=Retrieve&dopt=full_report&list_uids=253558) | lysocardiolipin acyltransferase 1 |
| [Details](http://mirdb.org/cgi-bin/target_detail.cgi?targetID=1439480) | 21 | 99 | hsa-miR-30c-5p | [XPO1](http://www.ncbi.nlm.nih.gov/entrez/query.fcgi?db=gene&cmd=Retrieve&dopt=full_report&list_uids=7514) | exportin 1 |
| [Details](http://mirdb.org/cgi-bin/target_detail.cgi?targetID=1439482) | 22 | 99 | hsa-miR-30c-5p | [POLR3E](http://www.ncbi.nlm.nih.gov/entrez/query.fcgi?db=gene&cmd=Retrieve&dopt=full_report&list_uids=55718) | RNA polymerase III subunit E |
| [Details](http://mirdb.org/cgi-bin/target_detail.cgi?targetID=1439497) | 23 | 99 | hsa-miR-30c-5p | [ALG10](http://www.ncbi.nlm.nih.gov/entrez/query.fcgi?db=gene&cmd=Retrieve&dopt=full_report&list_uids=84920) | ALG10, alpha-1,2-glucosyltransferase |
| [Details](http://mirdb.org/cgi-bin/target_detail.cgi?targetID=1439557) | 24 | 99 | hsa-miR-30c-5p | [CCDC97](http://www.ncbi.nlm.nih.gov/entrez/query.fcgi?db=gene&cmd=Retrieve&dopt=full_report&list_uids=90324) | coiled-coil domain containing 97 |
| [Details](http://mirdb.org/cgi-bin/target_detail.cgi?targetID=1439600) | 25 | 99 | hsa-miR-30c-5p | [PDE7A](http://www.ncbi.nlm.nih.gov/entrez/query.fcgi?db=gene&cmd=Retrieve&dopt=full_report&list_uids=5150) | phosphodiesterase 7A |
| [Details](http://mirdb.org/cgi-bin/target_detail.cgi?targetID=1439608) | 26 | 99 | hsa-miR-30c-5p | [ZBTB41](http://www.ncbi.nlm.nih.gov/entrez/query.fcgi?db=gene&cmd=Retrieve&dopt=full_report&list_uids=360023) | zinc finger and BTB domain containing 41 |
| [Details](http://mirdb.org/cgi-bin/target_detail.cgi?targetID=1439622) | 27 | 99 | hsa-miR-30c-5p | [RARG](http://www.ncbi.nlm.nih.gov/entrez/query.fcgi?db=gene&cmd=Retrieve&dopt=full_report&list_uids=5916) | retinoic acid receptor gamma |
| [Details](http://mirdb.org/cgi-bin/target_detail.cgi?targetID=1439735) | 28 | 99 | hsa-miR-30c-5p | [FNDC3A](http://www.ncbi.nlm.nih.gov/entrez/query.fcgi?db=gene&cmd=Retrieve&dopt=full_report&list_uids=22862) | fibronectin type III domain containing 3A |
| [Details](http://mirdb.org/cgi-bin/target_detail.cgi?targetID=1439932) | 29 | 99 | hsa-miR-30c-5p | [LHX8](http://www.ncbi.nlm.nih.gov/entrez/query.fcgi?db=gene&cmd=Retrieve&dopt=full_report&list_uids=431707) | LIM homeobox 8 |
| [Details](http://mirdb.org/cgi-bin/target_detail.cgi?targetID=1440024) | 30 | 99 | hsa-miR-30c-5p | [NCAM1](http://www.ncbi.nlm.nih.gov/entrez/query.fcgi?db=gene&cmd=Retrieve&dopt=full_report&list_uids=4684) | neural cell adhesion molecule 1 |
| [Details](http://mirdb.org/cgi-bin/target_detail.cgi?targetID=1440157) | 31 | 99 | hsa-miR-30c-5p | [TNRC6A](http://www.ncbi.nlm.nih.gov/entrez/query.fcgi?db=gene&cmd=Retrieve&dopt=full_report&list_uids=27327) | trinucleotide repeat containing 6A |
| [Details](http://mirdb.org/cgi-bin/target_detail.cgi?targetID=1440179) | 32 | 99 | hsa-miR-30c-5p | [USP37](http://www.ncbi.nlm.nih.gov/entrez/query.fcgi?db=gene&cmd=Retrieve&dopt=full_report&list_uids=57695) | ubiquitin specific peptidase 37 |
| [Details](http://mirdb.org/cgi-bin/target_detail.cgi?targetID=1440295) | 33 | 99 | hsa-miR-30c-5p | [SNX16](http://www.ncbi.nlm.nih.gov/entrez/query.fcgi?db=gene&cmd=Retrieve&dopt=full_report&list_uids=64089) | sorting nexin 16 |
| [Details](http://mirdb.org/cgi-bin/target_detail.cgi?targetID=1440323) | 34 | 99 | hsa-miR-30c-5p | [LRRC17](http://www.ncbi.nlm.nih.gov/entrez/query.fcgi?db=gene&cmd=Retrieve&dopt=full_report&list_uids=10234) | leucine rich repeat containing 17 |
| [Details](http://mirdb.org/cgi-bin/target_detail.cgi?targetID=1440379) | 35 | 99 | hsa-miR-30c-5p | [PIP4K2A](http://www.ncbi.nlm.nih.gov/entrez/query.fcgi?db=gene&cmd=Retrieve&dopt=full_report&list_uids=5305) | phosphatidylinositol-5-phosphate 4-kinase type 2 alpha |
| [Details](http://mirdb.org/cgi-bin/target_detail.cgi?targetID=1440405) | 36 | 99 | hsa-miR-30c-5p | [C9orf72](http://www.ncbi.nlm.nih.gov/entrez/query.fcgi?db=gene&cmd=Retrieve&dopt=full_report&list_uids=203228) | chromosome 9 open reading frame 72 |
| [Details](http://mirdb.org/cgi-bin/target_detail.cgi?targetID=1440465) | 37 | 99 | hsa-miR-30c-5p | [NT5E](http://www.ncbi.nlm.nih.gov/entrez/query.fcgi?db=gene&cmd=Retrieve&dopt=full_report&list_uids=4907) | 5'-nucleotidase ecto |
| [Details](http://mirdb.org/cgi-bin/target_detail.cgi?targetID=1440466) | 38 | 99 | hsa-miR-30c-5p | [MEIOB](http://www.ncbi.nlm.nih.gov/entrez/query.fcgi?db=gene&cmd=Retrieve&dopt=full_report&list_uids=254528) | meiosis specific with OB domains |
| [Details](http://mirdb.org/cgi-bin/target_detail.cgi?targetID=1440667) | 39 | 99 | hsa-miR-30c-5p | [SH3PXD2A](http://www.ncbi.nlm.nih.gov/entrez/query.fcgi?db=gene&cmd=Retrieve&dopt=full_report&list_uids=9644) | SH3 and PX domains 2A |
| [Details](http://mirdb.org/cgi-bin/target_detail.cgi?targetID=1440749) | 40 | 99 | hsa-miR-30c-5p | [BRWD1](http://www.ncbi.nlm.nih.gov/entrez/query.fcgi?db=gene&cmd=Retrieve&dopt=full_report&list_uids=54014) | bromodomain and WD repeat domain containing 1 |
| [Details](http://mirdb.org/cgi-bin/target_detail.cgi?targetID=1440764) | 41 | 99 | hsa-miR-30c-5p | [CCNE2](http://www.ncbi.nlm.nih.gov/entrez/query.fcgi?db=gene&cmd=Retrieve&dopt=full_report&list_uids=9134) | cyclin E2 |
| [Details](http://mirdb.org/cgi-bin/target_detail.cgi?targetID=1440791) | 42 | 99 | hsa-miR-30c-5p | [RFX6](http://www.ncbi.nlm.nih.gov/entrez/query.fcgi?db=gene&cmd=Retrieve&dopt=full_report&list_uids=222546) | regulatory factor X6 |
| [Details](http://mirdb.org/cgi-bin/target_detail.cgi?targetID=1440807) | 43 | 99 | hsa-miR-30c-5p | [COL25A1](http://www.ncbi.nlm.nih.gov/entrez/query.fcgi?db=gene&cmd=Retrieve&dopt=full_report&list_uids=84570) | collagen type XXV alpha 1 chain |
| [Details](http://mirdb.org/cgi-bin/target_detail.cgi?targetID=1440812) | 44 | 99 | hsa-miR-30c-5p | [RFX7](http://www.ncbi.nlm.nih.gov/entrez/query.fcgi?db=gene&cmd=Retrieve&dopt=full_report&list_uids=64864) | regulatory factor X7 |
| [Details](http://mirdb.org/cgi-bin/target_detail.cgi?targetID=1440828) | 45 | 99 | hsa-miR-30c-5p | [KLHL28](http://www.ncbi.nlm.nih.gov/entrez/query.fcgi?db=gene&cmd=Retrieve&dopt=full_report&list_uids=54813) | kelch like family member 28 |
| [Details](http://mirdb.org/cgi-bin/target_detail.cgi?targetID=1440853) | 46 | 99 | hsa-miR-30c-5p | [GMNC](http://www.ncbi.nlm.nih.gov/entrez/query.fcgi?db=gene&cmd=Retrieve&dopt=full_report&list_uids=647309) | geminin coiled-coil domain containing |
| [Details](http://mirdb.org/cgi-bin/target_detail.cgi?targetID=1440969) | 47 | 99 | hsa-miR-30c-5p | [PRDM1](http://www.ncbi.nlm.nih.gov/entrez/query.fcgi?db=gene&cmd=Retrieve&dopt=full_report&list_uids=639) | PR/SET domain 1 |
| [Details](http://mirdb.org/cgi-bin/target_detail.cgi?targetID=1439465) | 48 | 98 | hsa-miR-30c-5p | [BDP1](http://www.ncbi.nlm.nih.gov/entrez/query.fcgi?db=gene&cmd=Retrieve&dopt=full_report&list_uids=55814) | B double prime 1, subunit of RNA polymerase III transcription initiation factor IIIB |
| [Details](http://mirdb.org/cgi-bin/target_detail.cgi?targetID=1439525) | 49 | 98 | hsa-miR-30c-5p | [SCARA5](http://www.ncbi.nlm.nih.gov/entrez/query.fcgi?db=gene&cmd=Retrieve&dopt=full_report&list_uids=286133) | scavenger receptor class A member 5 |
| [Details](http://mirdb.org/cgi-bin/target_detail.cgi?targetID=1439540) | 50 | 98 | hsa-miR-30c-5p | [SCN9A](http://www.ncbi.nlm.nih.gov/entrez/query.fcgi?db=gene&cmd=Retrieve&dopt=full_report&list_uids=6335) | sodium voltage-gated channel alpha subunit 9 |
| [Details](http://mirdb.org/cgi-bin/target_detail.cgi?targetID=1439568) | 51 | 98 | hsa-miR-30c-5p | [ELL2](http://www.ncbi.nlm.nih.gov/entrez/query.fcgi?db=gene&cmd=Retrieve&dopt=full_report&list_uids=22936) | elongation factor for RNA polymerase II 2 |
| [Details](http://mirdb.org/cgi-bin/target_detail.cgi?targetID=1439587) | 52 | 98 | hsa-miR-30c-5p | [SCML1](http://www.ncbi.nlm.nih.gov/entrez/query.fcgi?db=gene&cmd=Retrieve&dopt=full_report&list_uids=6322) | Scm polycomb group protein like 1 |
| [Details](http://mirdb.org/cgi-bin/target_detail.cgi?targetID=1439612) | 53 | 98 | hsa-miR-30c-5p | [STXBP5](http://www.ncbi.nlm.nih.gov/entrez/query.fcgi?db=gene&cmd=Retrieve&dopt=full_report&list_uids=134957) | syntaxin binding protein 5 |
| [Details](http://mirdb.org/cgi-bin/target_detail.cgi?targetID=1439613) | 54 | 98 | hsa-miR-30c-5p | [LIMCH1](http://www.ncbi.nlm.nih.gov/entrez/query.fcgi?db=gene&cmd=Retrieve&dopt=full_report&list_uids=22998) | LIM and calponin homology domains 1 |
| [Details](http://mirdb.org/cgi-bin/target_detail.cgi?targetID=1439655) | 55 | 98 | hsa-miR-30c-5p | [MYH11](http://www.ncbi.nlm.nih.gov/entrez/query.fcgi?db=gene&cmd=Retrieve&dopt=full_report&list_uids=4629) | myosin heavy chain 11 |
| [Details](http://mirdb.org/cgi-bin/target_detail.cgi?targetID=1439673) | 56 | 98 | hsa-miR-30c-5p | [RTKN2](http://www.ncbi.nlm.nih.gov/entrez/query.fcgi?db=gene&cmd=Retrieve&dopt=full_report&list_uids=219790) | rhotekin 2 |
| [Details](http://mirdb.org/cgi-bin/target_detail.cgi?targetID=1439706) | 57 | 98 | hsa-miR-30c-5p | [ANO4](http://www.ncbi.nlm.nih.gov/entrez/query.fcgi?db=gene&cmd=Retrieve&dopt=full_report&list_uids=121601) | anoctamin 4 |
| [Details](http://mirdb.org/cgi-bin/target_detail.cgi?targetID=1439748) | 58 | 98 | hsa-miR-30c-5p | [GALNT7](http://www.ncbi.nlm.nih.gov/entrez/query.fcgi?db=gene&cmd=Retrieve&dopt=full_report&list_uids=51809) | polypeptide N-acetylgalactosaminyltransferase 7 |
| [Details](http://mirdb.org/cgi-bin/target_detail.cgi?targetID=1439778) | 59 | 98 | hsa-miR-30c-5p | [NEDD4](http://www.ncbi.nlm.nih.gov/entrez/query.fcgi?db=gene&cmd=Retrieve&dopt=full_report&list_uids=4734) | neural precursor cell expressed, developmentally down-regulated 4, E3 ubiquitin protein ligase |
| [Details](http://mirdb.org/cgi-bin/target_detail.cgi?targetID=1439795) | 60 | 98 | hsa-miR-30c-5p | [ROR1](http://www.ncbi.nlm.nih.gov/entrez/query.fcgi?db=gene&cmd=Retrieve&dopt=full_report&list_uids=4919) | receptor tyrosine kinase like orphan receptor 1 |
| [Details](http://mirdb.org/cgi-bin/target_detail.cgi?targetID=1439813) | 61 | 98 | hsa-miR-30c-5p | [YOD1](http://www.ncbi.nlm.nih.gov/entrez/query.fcgi?db=gene&cmd=Retrieve&dopt=full_report&list_uids=55432) | YOD1 deubiquitinase |
| [Details](http://mirdb.org/cgi-bin/target_detail.cgi?targetID=1439815) | 62 | 98 | hsa-miR-30c-5p | [CHD1](http://www.ncbi.nlm.nih.gov/entrez/query.fcgi?db=gene&cmd=Retrieve&dopt=full_report&list_uids=1105) | chromodomain helicase DNA binding protein 1 |
| [Details](http://mirdb.org/cgi-bin/target_detail.cgi?targetID=1439819) | 63 | 98 | hsa-miR-30c-5p | [RGS8](http://www.ncbi.nlm.nih.gov/entrez/query.fcgi?db=gene&cmd=Retrieve&dopt=full_report&list_uids=85397) | regulator of G protein signaling 8 |
| [Details](http://mirdb.org/cgi-bin/target_detail.cgi?targetID=1439873) | 64 | 98 | hsa-miR-30c-5p | [CYP24A1](http://www.ncbi.nlm.nih.gov/entrez/query.fcgi?db=gene&cmd=Retrieve&dopt=full_report&list_uids=1591) | cytochrome P450 family 24 subfamily A member 1 |
| [Details](http://mirdb.org/cgi-bin/target_detail.cgi?targetID=1439953) | 65 | 98 | hsa-miR-30c-5p | [LMBR1](http://www.ncbi.nlm.nih.gov/entrez/query.fcgi?db=gene&cmd=Retrieve&dopt=full_report&list_uids=64327) | limb development membrane protein 1 |
| [Details](http://mirdb.org/cgi-bin/target_detail.cgi?targetID=1439979) | 66 | 98 | hsa-miR-30c-5p | [RIMBP2](http://www.ncbi.nlm.nih.gov/entrez/query.fcgi?db=gene&cmd=Retrieve&dopt=full_report&list_uids=23504) | RIMS binding protein 2 |
| [Details](http://mirdb.org/cgi-bin/target_detail.cgi?targetID=1440282) | 67 | 98 | hsa-miR-30c-5p | [UBE2J1](http://www.ncbi.nlm.nih.gov/entrez/query.fcgi?db=gene&cmd=Retrieve&dopt=full_report&list_uids=51465) | ubiquitin conjugating enzyme E2 J1 |
| [Details](http://mirdb.org/cgi-bin/target_detail.cgi?targetID=1440300) | 68 | 98 | hsa-miR-30c-5p | [MAST4](http://www.ncbi.nlm.nih.gov/entrez/query.fcgi?db=gene&cmd=Retrieve&dopt=full_report&list_uids=375449) | microtubule associated serine/threonine kinase family member 4 |
| [Details](http://mirdb.org/cgi-bin/target_detail.cgi?targetID=1440303) | 69 | 98 | hsa-miR-30c-5p | [PEX5L](http://www.ncbi.nlm.nih.gov/entrez/query.fcgi?db=gene&cmd=Retrieve&dopt=full_report&list_uids=51555) | peroxisomal biogenesis factor 5 like |
| [Details](http://mirdb.org/cgi-bin/target_detail.cgi?targetID=1440340) | 70 | 98 | hsa-miR-30c-5p | [MTDH](http://www.ncbi.nlm.nih.gov/entrez/query.fcgi?db=gene&cmd=Retrieve&dopt=full_report&list_uids=92140) | metadherin |
| [Details](http://mirdb.org/cgi-bin/target_detail.cgi?targetID=1440372) | 71 | 98 | hsa-miR-30c-5p | [ZNRF1](http://www.ncbi.nlm.nih.gov/entrez/query.fcgi?db=gene&cmd=Retrieve&dopt=full_report&list_uids=84937) | zinc and ring finger 1 |
| [Details](http://mirdb.org/cgi-bin/target_detail.cgi?targetID=1440374) | 72 | 98 | hsa-miR-30c-5p | [GABRB1](http://www.ncbi.nlm.nih.gov/entrez/query.fcgi?db=gene&cmd=Retrieve&dopt=full_report&list_uids=2560) | gamma-aminobutyric acid type A receptor beta1 subunit |
| [Details](http://mirdb.org/cgi-bin/target_detail.cgi?targetID=1440415) | 73 | 98 | hsa-miR-30c-5p | [PLPP6](http://www.ncbi.nlm.nih.gov/entrez/query.fcgi?db=gene&cmd=Retrieve&dopt=full_report&list_uids=403313) | phospholipid phosphatase 6 |
| [Details](http://mirdb.org/cgi-bin/target_detail.cgi?targetID=1440448) | 74 | 98 | hsa-miR-30c-5p | [CHL1](http://www.ncbi.nlm.nih.gov/entrez/query.fcgi?db=gene&cmd=Retrieve&dopt=full_report&list_uids=10752) | cell adhesion molecule L1 like |
| [Details](http://mirdb.org/cgi-bin/target_detail.cgi?targetID=1440508) | 75 | 98 | hsa-miR-30c-5p | [SNX18](http://www.ncbi.nlm.nih.gov/entrez/query.fcgi?db=gene&cmd=Retrieve&dopt=full_report&list_uids=112574) | sorting nexin 18 |
| [Details](http://mirdb.org/cgi-bin/target_detail.cgi?targetID=1440567) | 76 | 98 | hsa-miR-30c-5p | [TMEM181](http://www.ncbi.nlm.nih.gov/entrez/query.fcgi?db=gene&cmd=Retrieve&dopt=full_report&list_uids=57583) | transmembrane protein 181 |
| [Details](http://mirdb.org/cgi-bin/target_detail.cgi?targetID=1440637) | 77 | 98 | hsa-miR-30c-5p | [PLPPR4](http://www.ncbi.nlm.nih.gov/entrez/query.fcgi?db=gene&cmd=Retrieve&dopt=full_report&list_uids=9890) | phospholipid phosphatase related 4 |
| [Details](http://mirdb.org/cgi-bin/target_detail.cgi?targetID=1440683) | 78 | 98 | hsa-miR-30c-5p | [LMBR1L](http://www.ncbi.nlm.nih.gov/entrez/query.fcgi?db=gene&cmd=Retrieve&dopt=full_report&list_uids=55716) | limb development membrane protein 1 like |
| [Details](http://mirdb.org/cgi-bin/target_detail.cgi?targetID=1440736) | 79 | 98 | hsa-miR-30c-5p | [PTPN13](http://www.ncbi.nlm.nih.gov/entrez/query.fcgi?db=gene&cmd=Retrieve&dopt=full_report&list_uids=5783) | protein tyrosine phosphatase, non-receptor type 13 |
| [Details](http://mirdb.org/cgi-bin/target_detail.cgi?targetID=1440745) | 80 | 98 | hsa-miR-30c-5p | [HCFC2](http://www.ncbi.nlm.nih.gov/entrez/query.fcgi?db=gene&cmd=Retrieve&dopt=full_report&list_uids=29915) | host cell factor C2 |
| [Details](http://mirdb.org/cgi-bin/target_detail.cgi?targetID=1440775) | 81 | 98 | hsa-miR-30c-5p | [HDAC9](http://www.ncbi.nlm.nih.gov/entrez/query.fcgi?db=gene&cmd=Retrieve&dopt=full_report&list_uids=9734) | histone deacetylase 9 |
| [Details](http://mirdb.org/cgi-bin/target_detail.cgi?targetID=1440815) | 82 | 98 | hsa-miR-30c-5p | [ADAMTS9](http://www.ncbi.nlm.nih.gov/entrez/query.fcgi?db=gene&cmd=Retrieve&dopt=full_report&list_uids=56999) | ADAM metallopeptidase with thrombospondin type 1 motif 9 |
| [Details](http://mirdb.org/cgi-bin/target_detail.cgi?targetID=1440819) | 83 | 98 | hsa-miR-30c-5p | [UBN2](http://www.ncbi.nlm.nih.gov/entrez/query.fcgi?db=gene&cmd=Retrieve&dopt=full_report&list_uids=254048) | ubinuclein 2 |
| [Details](http://mirdb.org/cgi-bin/target_detail.cgi?targetID=1440880) | 84 | 98 | hsa-miR-30c-5p | [ADRA2A](http://www.ncbi.nlm.nih.gov/entrez/query.fcgi?db=gene&cmd=Retrieve&dopt=full_report&list_uids=150) | adrenoceptor alpha 2A |
| [Details](http://mirdb.org/cgi-bin/target_detail.cgi?targetID=1440962) | 85 | 98 | hsa-miR-30c-5p | [DESI2](http://www.ncbi.nlm.nih.gov/entrez/query.fcgi?db=gene&cmd=Retrieve&dopt=full_report&list_uids=51029) | desumoylating isopeptidase 2 |
| [Details](http://mirdb.org/cgi-bin/target_detail.cgi?targetID=1440974) | 86 | 98 | hsa-miR-30c-5p | [PLAGL2](http://www.ncbi.nlm.nih.gov/entrez/query.fcgi?db=gene&cmd=Retrieve&dopt=full_report&list_uids=5326) | PLAG1 like zinc finger 2 |
| [Details](http://mirdb.org/cgi-bin/target_detail.cgi?targetID=1440997) | 87 | 98 | hsa-miR-30c-5p | [FRZB](http://www.ncbi.nlm.nih.gov/entrez/query.fcgi?db=gene&cmd=Retrieve&dopt=full_report&list_uids=2487) | frizzled related protein |
| [Details](http://mirdb.org/cgi-bin/target_detail.cgi?targetID=1439475) | 88 | 97 | hsa-miR-30c-5p | [SETD5](http://www.ncbi.nlm.nih.gov/entrez/query.fcgi?db=gene&cmd=Retrieve&dopt=full_report&list_uids=55209) | SET domain containing 5 |
| [Details](http://mirdb.org/cgi-bin/target_detail.cgi?targetID=1439523) | 89 | 97 | hsa-miR-30c-5p | [ZMYND8](http://www.ncbi.nlm.nih.gov/entrez/query.fcgi?db=gene&cmd=Retrieve&dopt=full_report&list_uids=23613) | zinc finger MYND-type containing 8 |
| [Details](http://mirdb.org/cgi-bin/target_detail.cgi?targetID=1439526) | 90 | 97 | hsa-miR-30c-5p | [TENT2](http://www.ncbi.nlm.nih.gov/entrez/query.fcgi?db=gene&cmd=Retrieve&dopt=full_report&list_uids=167153) | terminal nucleotidyltransferase 2 |
| [Details](http://mirdb.org/cgi-bin/target_detail.cgi?targetID=1439556) | 91 | 97 | hsa-miR-30c-5p | [SPOCK3](http://www.ncbi.nlm.nih.gov/entrez/query.fcgi?db=gene&cmd=Retrieve&dopt=full_report&list_uids=50859) | SPARC (osteonectin), cwcv and kazal like domains proteoglycan 3 |
| [Details](http://mirdb.org/cgi-bin/target_detail.cgi?targetID=1439637) | 92 | 97 | hsa-miR-30c-5p | [DCTN4](http://www.ncbi.nlm.nih.gov/entrez/query.fcgi?db=gene&cmd=Retrieve&dopt=full_report&list_uids=51164) | dynactin subunit 4 |
| [Details](http://mirdb.org/cgi-bin/target_detail.cgi?targetID=1439680) | 93 | 97 | hsa-miR-30c-5p | [FAP](http://www.ncbi.nlm.nih.gov/entrez/query.fcgi?db=gene&cmd=Retrieve&dopt=full_report&list_uids=2191) | fibroblast activation protein alpha |
| [Details](http://mirdb.org/cgi-bin/target_detail.cgi?targetID=1439797) | 94 | 97 | hsa-miR-30c-5p | [EML1](http://www.ncbi.nlm.nih.gov/entrez/query.fcgi?db=gene&cmd=Retrieve&dopt=full_report&list_uids=2009) | EMAP like 1 |
| [Details](http://mirdb.org/cgi-bin/target_detail.cgi?targetID=1439817) | 95 | 97 | hsa-miR-30c-5p | [ITPK1](http://www.ncbi.nlm.nih.gov/entrez/query.fcgi?db=gene&cmd=Retrieve&dopt=full_report&list_uids=3705) | inositol-tetrakisphosphate 1-kinase |
| [Details](http://mirdb.org/cgi-bin/target_detail.cgi?targetID=1439838) | 96 | 97 | hsa-miR-30c-5p | [RUNX1](http://www.ncbi.nlm.nih.gov/entrez/query.fcgi?db=gene&cmd=Retrieve&dopt=full_report&list_uids=861) | runt related transcription factor 1 |
| [Details](http://mirdb.org/cgi-bin/target_detail.cgi?targetID=1439842) | 97 | 97 | hsa-miR-30c-5p | [FOXG1](http://www.ncbi.nlm.nih.gov/entrez/query.fcgi?db=gene&cmd=Retrieve&dopt=full_report&list_uids=2290) | forkhead box G1 |
| [Details](http://mirdb.org/cgi-bin/target_detail.cgi?targetID=1439850) | 98 | 97 | hsa-miR-30c-5p | [STK39](http://www.ncbi.nlm.nih.gov/entrez/query.fcgi?db=gene&cmd=Retrieve&dopt=full_report&list_uids=27347) | serine/threonine kinase 39 |
| [Details](http://mirdb.org/cgi-bin/target_detail.cgi?targetID=1439882) | 99 | 97 | hsa-miR-30c-5p | [SLC35A3](http://www.ncbi.nlm.nih.gov/entrez/query.fcgi?db=gene&cmd=Retrieve&dopt=full_report&list_uids=23443) | solute carrier family 35 member A3 |
| [Details](http://mirdb.org/cgi-bin/target_detail.cgi?targetID=1439922) | 100 | 97 | hsa-miR-30c-5p | [KLF10](http://www.ncbi.nlm.nih.gov/entrez/query.fcgi?db=gene&cmd=Retrieve&dopt=full_report&list_uids=7071) | Kruppel like factor 10 |
| [Details](http://mirdb.org/cgi-bin/target_detail.cgi?targetID=1439939) | 101 | 97 | hsa-miR-30c-5p | [NFIB](http://www.ncbi.nlm.nih.gov/entrez/query.fcgi?db=gene&cmd=Retrieve&dopt=full_report&list_uids=4781) | nuclear factor I B |
| [Details](http://mirdb.org/cgi-bin/target_detail.cgi?targetID=1439942) | 102 | 97 | hsa-miR-30c-5p | [SPEN](http://www.ncbi.nlm.nih.gov/entrez/query.fcgi?db=gene&cmd=Retrieve&dopt=full_report&list_uids=23013) | spen family transcriptional repressor |
| [Details](http://mirdb.org/cgi-bin/target_detail.cgi?targetID=1439947) | 103 | 97 | hsa-miR-30c-5p | [TMEM170B](http://www.ncbi.nlm.nih.gov/entrez/query.fcgi?db=gene&cmd=Retrieve&dopt=full_report&list_uids=100113407) | transmembrane protein 170B |
| [Details](http://mirdb.org/cgi-bin/target_detail.cgi?targetID=1440021) | 104 | 97 | hsa-miR-30c-5p | [SH2B3](http://www.ncbi.nlm.nih.gov/entrez/query.fcgi?db=gene&cmd=Retrieve&dopt=full_report&list_uids=10019) | SH2B adaptor protein 3 |
| [Details](http://mirdb.org/cgi-bin/target_detail.cgi?targetID=1440030) | 105 | 97 | hsa-miR-30c-5p | [ADAMTS3](http://www.ncbi.nlm.nih.gov/entrez/query.fcgi?db=gene&cmd=Retrieve&dopt=full_report&list_uids=9508) | ADAM metallopeptidase with thrombospondin type 1 motif 3 |
| [Details](http://mirdb.org/cgi-bin/target_detail.cgi?targetID=1440036) | 106 | 97 | hsa-miR-30c-5p | [NECAP1](http://www.ncbi.nlm.nih.gov/entrez/query.fcgi?db=gene&cmd=Retrieve&dopt=full_report&list_uids=25977) | NECAP endocytosis associated 1 |
| [Details](http://mirdb.org/cgi-bin/target_detail.cgi?targetID=1440053) | 107 | 97 | hsa-miR-30c-5p | [PHTF2](http://www.ncbi.nlm.nih.gov/entrez/query.fcgi?db=gene&cmd=Retrieve&dopt=full_report&list_uids=57157) | putative homeodomain transcription factor 2 |
| [Details](http://mirdb.org/cgi-bin/target_detail.cgi?targetID=1440067) | 108 | 97 | hsa-miR-30c-5p | [DOLPP1](http://www.ncbi.nlm.nih.gov/entrez/query.fcgi?db=gene&cmd=Retrieve&dopt=full_report&list_uids=57171) | dolichyldiphosphatase 1 |
| [Details](http://mirdb.org/cgi-bin/target_detail.cgi?targetID=1440082) | 109 | 97 | hsa-miR-30c-5p | [SEC24A](http://www.ncbi.nlm.nih.gov/entrez/query.fcgi?db=gene&cmd=Retrieve&dopt=full_report&list_uids=10802) | SEC24 homolog A, COPII coat complex component |
| [Details](http://mirdb.org/cgi-bin/target_detail.cgi?targetID=1440098) | 110 | 97 | hsa-miR-30c-5p | [YTHDF3](http://www.ncbi.nlm.nih.gov/entrez/query.fcgi?db=gene&cmd=Retrieve&dopt=full_report&list_uids=253943) | YTH N6-methyladenosine RNA binding protein 3 |
| [Details](http://mirdb.org/cgi-bin/target_detail.cgi?targetID=1440159) | 111 | 97 | hsa-miR-30c-5p | [YPEL2](http://www.ncbi.nlm.nih.gov/entrez/query.fcgi?db=gene&cmd=Retrieve&dopt=full_report&list_uids=388403) | yippee like 2 |
| [Details](http://mirdb.org/cgi-bin/target_detail.cgi?targetID=1440193) | 112 | 97 | hsa-miR-30c-5p | [FAM160B1](http://www.ncbi.nlm.nih.gov/entrez/query.fcgi?db=gene&cmd=Retrieve&dopt=full_report&list_uids=57700) | family with sequence similarity 160 member B1 |
| [Details](http://mirdb.org/cgi-bin/target_detail.cgi?targetID=1440247) | 113 | 97 | hsa-miR-30c-5p | [TLL2](http://www.ncbi.nlm.nih.gov/entrez/query.fcgi?db=gene&cmd=Retrieve&dopt=full_report&list_uids=7093) | tolloid like 2 |
| [Details](http://mirdb.org/cgi-bin/target_detail.cgi?targetID=1440331) | 114 | 97 | hsa-miR-30c-5p | [E2F7](http://www.ncbi.nlm.nih.gov/entrez/query.fcgi?db=gene&cmd=Retrieve&dopt=full_report&list_uids=144455) | E2F transcription factor 7 |
| [Details](http://mirdb.org/cgi-bin/target_detail.cgi?targetID=1440334) | 115 | 97 | hsa-miR-30c-5p | [CARF](http://www.ncbi.nlm.nih.gov/entrez/query.fcgi?db=gene&cmd=Retrieve&dopt=full_report&list_uids=79800) | calcium responsive transcription factor |
| [Details](http://mirdb.org/cgi-bin/target_detail.cgi?targetID=1440354) | 116 | 97 | hsa-miR-30c-5p | [PPP1R2](http://www.ncbi.nlm.nih.gov/entrez/query.fcgi?db=gene&cmd=Retrieve&dopt=full_report&list_uids=5504) | protein phosphatase 1 regulatory inhibitor subunit 2 |
| [Details](http://mirdb.org/cgi-bin/target_detail.cgi?targetID=1440400) | 117 | 97 | hsa-miR-30c-5p | [SOX9](http://www.ncbi.nlm.nih.gov/entrez/query.fcgi?db=gene&cmd=Retrieve&dopt=full_report&list_uids=6662) | SRY-box 9 |
| [Details](http://mirdb.org/cgi-bin/target_detail.cgi?targetID=1440420) | 118 | 97 | hsa-miR-30c-5p | [SCN3A](http://www.ncbi.nlm.nih.gov/entrez/query.fcgi?db=gene&cmd=Retrieve&dopt=full_report&list_uids=6328) | sodium voltage-gated channel alpha subunit 3 |
| [Details](http://mirdb.org/cgi-bin/target_detail.cgi?targetID=1440453) | 119 | 97 | hsa-miR-30c-5p | [ATG12](http://www.ncbi.nlm.nih.gov/entrez/query.fcgi?db=gene&cmd=Retrieve&dopt=full_report&list_uids=9140) | autophagy related 12 |
| [Details](http://mirdb.org/cgi-bin/target_detail.cgi?targetID=1440519) | 120 | 97 | hsa-miR-30c-5p | [PPP3R1](http://www.ncbi.nlm.nih.gov/entrez/query.fcgi?db=gene&cmd=Retrieve&dopt=full_report&list_uids=5534) | protein phosphatase 3 regulatory subunit B, alpha |
| [Details](http://mirdb.org/cgi-bin/target_detail.cgi?targetID=1440584) | 121 | 97 | hsa-miR-30c-5p | [TNIK](http://www.ncbi.nlm.nih.gov/entrez/query.fcgi?db=gene&cmd=Retrieve&dopt=full_report&list_uids=23043) | TRAF2 and NCK interacting kinase |
| [Details](http://mirdb.org/cgi-bin/target_detail.cgi?targetID=1440595) | 122 | 97 | hsa-miR-30c-5p | [PTP4A1](http://www.ncbi.nlm.nih.gov/entrez/query.fcgi?db=gene&cmd=Retrieve&dopt=full_report&list_uids=7803) | protein tyrosine phosphatase type IVA, member 1 |
| [Details](http://mirdb.org/cgi-bin/target_detail.cgi?targetID=1440610) | 123 | 97 | hsa-miR-30c-5p | [REEP3](http://www.ncbi.nlm.nih.gov/entrez/query.fcgi?db=gene&cmd=Retrieve&dopt=full_report&list_uids=221035) | receptor accessory protein 3 |
| [Details](http://mirdb.org/cgi-bin/target_detail.cgi?targetID=1440681) | 124 | 97 | hsa-miR-30c-5p | [VIM](http://www.ncbi.nlm.nih.gov/entrez/query.fcgi?db=gene&cmd=Retrieve&dopt=full_report&list_uids=7431) | vimentin |
| [Details](http://mirdb.org/cgi-bin/target_detail.cgi?targetID=1440696) | 125 | 97 | hsa-miR-30c-5p | [EXTL2](http://www.ncbi.nlm.nih.gov/entrez/query.fcgi?db=gene&cmd=Retrieve&dopt=full_report&list_uids=2135) | exostosin like glycosyltransferase 2 |
| [Details](http://mirdb.org/cgi-bin/target_detail.cgi?targetID=1440746) | 126 | 97 | hsa-miR-30c-5p | [STK35](http://www.ncbi.nlm.nih.gov/entrez/query.fcgi?db=gene&cmd=Retrieve&dopt=full_report&list_uids=140901) | serine/threonine kinase 35 |
| [Details](http://mirdb.org/cgi-bin/target_detail.cgi?targetID=1440845) | 127 | 97 | hsa-miR-30c-5p | [ACTR3C](http://www.ncbi.nlm.nih.gov/entrez/query.fcgi?db=gene&cmd=Retrieve&dopt=full_report&list_uids=653857) | ARP3 actin related protein 3 homolog C |
| [Details](http://mirdb.org/cgi-bin/target_detail.cgi?targetID=1440852) | 128 | 97 | hsa-miR-30c-5p | [STIM2](http://www.ncbi.nlm.nih.gov/entrez/query.fcgi?db=gene&cmd=Retrieve&dopt=full_report&list_uids=57620) | stromal interaction molecule 2 |
| [Details](http://mirdb.org/cgi-bin/target_detail.cgi?targetID=1439454) | 129 | 96 | hsa-miR-30c-5p | [CCDC117](http://www.ncbi.nlm.nih.gov/entrez/query.fcgi?db=gene&cmd=Retrieve&dopt=full_report&list_uids=150275) | coiled-coil domain containing 117 |
| [Details](http://mirdb.org/cgi-bin/target_detail.cgi?targetID=1439459) | 130 | 96 | hsa-miR-30c-5p | [PNKD](http://www.ncbi.nlm.nih.gov/entrez/query.fcgi?db=gene&cmd=Retrieve&dopt=full_report&list_uids=25953) | PNKD, MBL domain containing |
| [Details](http://mirdb.org/cgi-bin/target_detail.cgi?targetID=1439573) | 131 | 96 | hsa-miR-30c-5p | [SAMD8](http://www.ncbi.nlm.nih.gov/entrez/query.fcgi?db=gene&cmd=Retrieve&dopt=full_report&list_uids=142891) | sterile alpha motif domain containing 8 |
| [Details](http://mirdb.org/cgi-bin/target_detail.cgi?targetID=1439611) | 132 | 96 | hsa-miR-30c-5p | [FBXO45](http://www.ncbi.nlm.nih.gov/entrez/query.fcgi?db=gene&cmd=Retrieve&dopt=full_report&list_uids=200933) | F-box protein 45 |
| [Details](http://mirdb.org/cgi-bin/target_detail.cgi?targetID=1439646) | 133 | 96 | hsa-miR-30c-5p | [GOLGA1](http://www.ncbi.nlm.nih.gov/entrez/query.fcgi?db=gene&cmd=Retrieve&dopt=full_report&list_uids=2800) | golgin A1 |
| [Details](http://mirdb.org/cgi-bin/target_detail.cgi?targetID=1439652) | 134 | 96 | hsa-miR-30c-5p | [PLEKHM3](http://www.ncbi.nlm.nih.gov/entrez/query.fcgi?db=gene&cmd=Retrieve&dopt=full_report&list_uids=389072) | pleckstrin homology domain containing M3 |
| [Details](http://mirdb.org/cgi-bin/target_detail.cgi?targetID=1439658) | 135 | 96 | hsa-miR-30c-5p | [AZIN1](http://www.ncbi.nlm.nih.gov/entrez/query.fcgi?db=gene&cmd=Retrieve&dopt=full_report&list_uids=51582) | antizyme inhibitor 1 |
| [Details](http://mirdb.org/cgi-bin/target_detail.cgi?targetID=1439686) | 136 | 96 | hsa-miR-30c-5p | [ZBTB11](http://www.ncbi.nlm.nih.gov/entrez/query.fcgi?db=gene&cmd=Retrieve&dopt=full_report&list_uids=27107) | zinc finger and BTB domain containing 11 |
| [Details](http://mirdb.org/cgi-bin/target_detail.cgi?targetID=1439854) | 137 | 96 | hsa-miR-30c-5p | [PHIP](http://www.ncbi.nlm.nih.gov/entrez/query.fcgi?db=gene&cmd=Retrieve&dopt=full_report&list_uids=55023) | pleckstrin homology domain interacting protein |
| [Details](http://mirdb.org/cgi-bin/target_detail.cgi?targetID=1439913) | 138 | 96 | hsa-miR-30c-5p | [DDAH1](http://www.ncbi.nlm.nih.gov/entrez/query.fcgi?db=gene&cmd=Retrieve&dopt=full_report&list_uids=23576) | dimethylarginine dimethylaminohydrolase 1 |
| [Details](http://mirdb.org/cgi-bin/target_detail.cgi?targetID=1439976) | 139 | 96 | hsa-miR-30c-5p | [SRSF7](http://www.ncbi.nlm.nih.gov/entrez/query.fcgi?db=gene&cmd=Retrieve&dopt=full_report&list_uids=6432) | serine and arginine rich splicing factor 7 |
| [Details](http://mirdb.org/cgi-bin/target_detail.cgi?targetID=1439998) | 140 | 96 | hsa-miR-30c-5p | [TBL1XR1](http://www.ncbi.nlm.nih.gov/entrez/query.fcgi?db=gene&cmd=Retrieve&dopt=full_report&list_uids=79718) | transducin beta like 1 X-linked receptor 1 |
| [Details](http://mirdb.org/cgi-bin/target_detail.cgi?targetID=1440044) | 141 | 96 | hsa-miR-30c-5p | [CHIC1](http://www.ncbi.nlm.nih.gov/entrez/query.fcgi?db=gene&cmd=Retrieve&dopt=full_report&list_uids=53344) | cysteine rich hydrophobic domain 1 |
| [Details](http://mirdb.org/cgi-bin/target_detail.cgi?targetID=1440211) | 142 | 96 | hsa-miR-30c-5p | [ITGA6](http://www.ncbi.nlm.nih.gov/entrez/query.fcgi?db=gene&cmd=Retrieve&dopt=full_report&list_uids=3655) | integrin subunit alpha 6 |
| [Details](http://mirdb.org/cgi-bin/target_detail.cgi?targetID=1440234) | 143 | 96 | hsa-miR-30c-5p | [SLC12A6](http://www.ncbi.nlm.nih.gov/entrez/query.fcgi?db=gene&cmd=Retrieve&dopt=full_report&list_uids=9990) | solute carrier family 12 member 6 |
| [Details](http://mirdb.org/cgi-bin/target_detail.cgi?targetID=1440258) | 144 | 96 | hsa-miR-30c-5p | [RUNX2](http://www.ncbi.nlm.nih.gov/entrez/query.fcgi?db=gene&cmd=Retrieve&dopt=full_report&list_uids=860) | runt related transcription factor 2 |
| [Details](http://mirdb.org/cgi-bin/target_detail.cgi?targetID=1440263) | 145 | 96 | hsa-miR-30c-5p | [OTUD6B](http://www.ncbi.nlm.nih.gov/entrez/query.fcgi?db=gene&cmd=Retrieve&dopt=full_report&list_uids=51633) | OTU domain containing 6B |
| [Details](http://mirdb.org/cgi-bin/target_detail.cgi?targetID=1440286) | 146 | 96 | hsa-miR-30c-5p | [CCDC43](http://www.ncbi.nlm.nih.gov/entrez/query.fcgi?db=gene&cmd=Retrieve&dopt=full_report&list_uids=124808) | coiled-coil domain containing 43 |
| [Details](http://mirdb.org/cgi-bin/target_detail.cgi?targetID=1440297) | 147 | 96 | hsa-miR-30c-5p | [SLC35C1](http://www.ncbi.nlm.nih.gov/entrez/query.fcgi?db=gene&cmd=Retrieve&dopt=full_report&list_uids=55343) | solute carrier family 35 member C1 |
| [Details](http://mirdb.org/cgi-bin/target_detail.cgi?targetID=1440335) | 148 | 96 | hsa-miR-30c-5p | [NAV3](http://www.ncbi.nlm.nih.gov/entrez/query.fcgi?db=gene&cmd=Retrieve&dopt=full_report&list_uids=89795) | neuron navigator 3 |
| [Details](http://mirdb.org/cgi-bin/target_detail.cgi?targetID=1440352) | 149 | 96 | hsa-miR-30c-5p | [ASB3](http://www.ncbi.nlm.nih.gov/entrez/query.fcgi?db=gene&cmd=Retrieve&dopt=full_report&list_uids=51130) | ankyrin repeat and SOCS box containing 3 |
| [Details](http://mirdb.org/cgi-bin/target_detail.cgi?targetID=1440367) | 150 | 96 | hsa-miR-30c-5p | [SOCS1](http://www.ncbi.nlm.nih.gov/entrez/query.fcgi?db=gene&cmd=Retrieve&dopt=full_report&list_uids=8651) | suppressor of cytokine signaling 1 |
| [Details](http://mirdb.org/cgi-bin/target_detail.cgi?targetID=1440382) | 151 | 96 | hsa-miR-30c-5p | [XPR1](http://www.ncbi.nlm.nih.gov/entrez/query.fcgi?db=gene&cmd=Retrieve&dopt=full_report&list_uids=9213) | xenotropic and polytropic retrovirus receptor 1 |
| [Details](http://mirdb.org/cgi-bin/target_detail.cgi?targetID=1440478) | 152 | 96 | hsa-miR-30c-5p | [PLEKHO2](http://www.ncbi.nlm.nih.gov/entrez/query.fcgi?db=gene&cmd=Retrieve&dopt=full_report&list_uids=80301) | pleckstrin homology domain containing O2 |
| [Details](http://mirdb.org/cgi-bin/target_detail.cgi?targetID=1440520) | 153 | 96 | hsa-miR-30c-5p | [CNKSR2](http://www.ncbi.nlm.nih.gov/entrez/query.fcgi?db=gene&cmd=Retrieve&dopt=full_report&list_uids=22866) | connector enhancer of kinase suppressor of Ras 2 |
| [Details](http://mirdb.org/cgi-bin/target_detail.cgi?targetID=1440528) | 154 | 96 | hsa-miR-30c-5p | [CCNT2](http://www.ncbi.nlm.nih.gov/entrez/query.fcgi?db=gene&cmd=Retrieve&dopt=full_report&list_uids=905) | cyclin T2 |
| [Details](http://mirdb.org/cgi-bin/target_detail.cgi?targetID=1440545) | 155 | 96 | hsa-miR-30c-5p | [S100PBP](http://www.ncbi.nlm.nih.gov/entrez/query.fcgi?db=gene&cmd=Retrieve&dopt=full_report&list_uids=64766) | S100P binding protein |
| [Details](http://mirdb.org/cgi-bin/target_detail.cgi?targetID=1440597) | 156 | 96 | hsa-miR-30c-5p | [ADAM19](http://www.ncbi.nlm.nih.gov/entrez/query.fcgi?db=gene&cmd=Retrieve&dopt=full_report&list_uids=8728) | ADAM metallopeptidase domain 19 |
| [Details](http://mirdb.org/cgi-bin/target_detail.cgi?targetID=1440650) | 157 | 96 | hsa-miR-30c-5p | [PAPOLA](http://www.ncbi.nlm.nih.gov/entrez/query.fcgi?db=gene&cmd=Retrieve&dopt=full_report&list_uids=10914) | poly(A) polymerase alpha |
| [Details](http://mirdb.org/cgi-bin/target_detail.cgi?targetID=1440656) | 158 | 96 | hsa-miR-30c-5p | [KLF12](http://www.ncbi.nlm.nih.gov/entrez/query.fcgi?db=gene&cmd=Retrieve&dopt=full_report&list_uids=11278) | Kruppel like factor 12 |
| [Details](http://mirdb.org/cgi-bin/target_detail.cgi?targetID=1440728) | 159 | 96 | hsa-miR-30c-5p | [EML4](http://www.ncbi.nlm.nih.gov/entrez/query.fcgi?db=gene&cmd=Retrieve&dopt=full_report&list_uids=27436) | EMAP like 4 |
| [Details](http://mirdb.org/cgi-bin/target_detail.cgi?targetID=1440773) | 160 | 96 | hsa-miR-30c-5p | [PON2](http://www.ncbi.nlm.nih.gov/entrez/query.fcgi?db=gene&cmd=Retrieve&dopt=full_report&list_uids=5445) | paraoxonase 2 |
| [Details](http://mirdb.org/cgi-bin/target_detail.cgi?targetID=1440830) | 161 | 96 | hsa-miR-30c-5p | [CFL2](http://www.ncbi.nlm.nih.gov/entrez/query.fcgi?db=gene&cmd=Retrieve&dopt=full_report&list_uids=1073) | cofilin 2 |
| [Details](http://mirdb.org/cgi-bin/target_detail.cgi?targetID=1440889) | 162 | 96 | hsa-miR-30c-5p | [CHST2](http://www.ncbi.nlm.nih.gov/entrez/query.fcgi?db=gene&cmd=Retrieve&dopt=full_report&list_uids=9435) | carbohydrate sulfotransferase 2 |
| [Details](http://mirdb.org/cgi-bin/target_detail.cgi?targetID=1440904) | 163 | 96 | hsa-miR-30c-5p | [ANKHD1](http://www.ncbi.nlm.nih.gov/entrez/query.fcgi?db=gene&cmd=Retrieve&dopt=full_report&list_uids=54882) | ankyrin repeat and KH domain containing 1 |
| [Details](http://mirdb.org/cgi-bin/target_detail.cgi?targetID=1440994) | 164 | 96 | hsa-miR-30c-5p | [CNOT9](http://www.ncbi.nlm.nih.gov/entrez/query.fcgi?db=gene&cmd=Retrieve&dopt=full_report&list_uids=9125) | CCR4-NOT transcription complex subunit 9 |
| [Details](http://mirdb.org/cgi-bin/target_detail.cgi?targetID=1439514) | 165 | 95 | hsa-miR-30c-5p | [RASA2](http://www.ncbi.nlm.nih.gov/entrez/query.fcgi?db=gene&cmd=Retrieve&dopt=full_report&list_uids=5922) | RAS p21 protein activator 2 |
| [Details](http://mirdb.org/cgi-bin/target_detail.cgi?targetID=1439516) | 166 | 95 | hsa-miR-30c-5p | [PFN2](http://www.ncbi.nlm.nih.gov/entrez/query.fcgi?db=gene&cmd=Retrieve&dopt=full_report&list_uids=5217) | profilin 2 |
| [Details](http://mirdb.org/cgi-bin/target_detail.cgi?targetID=1439527) | 167 | 95 | hsa-miR-30c-5p | [SEC22C](http://www.ncbi.nlm.nih.gov/entrez/query.fcgi?db=gene&cmd=Retrieve&dopt=full_report&list_uids=9117) | SEC22 homolog C, vesicle trafficking protein |
| [Details](http://mirdb.org/cgi-bin/target_detail.cgi?targetID=1439529) | 168 | 95 | hsa-miR-30c-5p | [TMEM56](http://www.ncbi.nlm.nih.gov/entrez/query.fcgi?db=gene&cmd=Retrieve&dopt=full_report&list_uids=148534) | transmembrane protein 56 |
| [Details](http://mirdb.org/cgi-bin/target_detail.cgi?targetID=1439630) | 169 | 95 | hsa-miR-30c-5p | [SOCS3](http://www.ncbi.nlm.nih.gov/entrez/query.fcgi?db=gene&cmd=Retrieve&dopt=full_report&list_uids=9021) | suppressor of cytokine signaling 3 |
| [Details](http://mirdb.org/cgi-bin/target_detail.cgi?targetID=1439645) | 170 | 95 | hsa-miR-30c-5p | [RRAD](http://www.ncbi.nlm.nih.gov/entrez/query.fcgi?db=gene&cmd=Retrieve&dopt=full_report&list_uids=6236) | RRAD, Ras related glycolysis inhibitor and calcium channel regulator |
| [Details](http://mirdb.org/cgi-bin/target_detail.cgi?targetID=1439703) | 171 | 95 | hsa-miR-30c-5p | [DLG5](http://www.ncbi.nlm.nih.gov/entrez/query.fcgi?db=gene&cmd=Retrieve&dopt=full_report&list_uids=9231) | discs large MAGUK scaffold protein 5 |
| [Details](http://mirdb.org/cgi-bin/target_detail.cgi?targetID=1439755) | 172 | 95 | hsa-miR-30c-5p | [ANKRD17](http://www.ncbi.nlm.nih.gov/entrez/query.fcgi?db=gene&cmd=Retrieve&dopt=full_report&list_uids=26057) | ankyrin repeat domain 17 |
| [Details](http://mirdb.org/cgi-bin/target_detail.cgi?targetID=1439935) | 173 | 95 | hsa-miR-30c-5p | [LPGAT1](http://www.ncbi.nlm.nih.gov/entrez/query.fcgi?db=gene&cmd=Retrieve&dopt=full_report&list_uids=9926) | lysophosphatidylglycerol acyltransferase 1 |
| [Details](http://mirdb.org/cgi-bin/target_detail.cgi?targetID=1439950) | 174 | 95 | hsa-miR-30c-5p | [MARCH6](http://www.ncbi.nlm.nih.gov/entrez/query.fcgi?db=gene&cmd=Retrieve&dopt=full_report&list_uids=10299) | membrane associated ring-CH-type finger 6 |
| [Details](http://mirdb.org/cgi-bin/target_detail.cgi?targetID=1439973) | 175 | 95 | hsa-miR-30c-5p | [SIX1](http://www.ncbi.nlm.nih.gov/entrez/query.fcgi?db=gene&cmd=Retrieve&dopt=full_report&list_uids=6495) | SIX homeobox 1 |
| [Details](http://mirdb.org/cgi-bin/target_detail.cgi?targetID=1440033) | 176 | 95 | hsa-miR-30c-5p | [FRMPD1](http://www.ncbi.nlm.nih.gov/entrez/query.fcgi?db=gene&cmd=Retrieve&dopt=full_report&list_uids=22844) | FERM and PDZ domain containing 1 |
| [Details](http://mirdb.org/cgi-bin/target_detail.cgi?targetID=1440037) | 177 | 95 | hsa-miR-30c-5p | [EEA1](http://www.ncbi.nlm.nih.gov/entrez/query.fcgi?db=gene&cmd=Retrieve&dopt=full_report&list_uids=8411) | early endosome antigen 1 |
| [Details](http://mirdb.org/cgi-bin/target_detail.cgi?targetID=1440048) | 178 | 95 | hsa-miR-30c-5p | [CLOCK](http://www.ncbi.nlm.nih.gov/entrez/query.fcgi?db=gene&cmd=Retrieve&dopt=full_report&list_uids=9575) | clock circadian regulator |
| [Details](http://mirdb.org/cgi-bin/target_detail.cgi?targetID=1440109) | 179 | 95 | hsa-miR-30c-5p | [TP53INP1](http://www.ncbi.nlm.nih.gov/entrez/query.fcgi?db=gene&cmd=Retrieve&dopt=full_report&list_uids=94241) | tumor protein p53 inducible nuclear protein 1 |
| [Details](http://mirdb.org/cgi-bin/target_detail.cgi?targetID=1440125) | 180 | 95 | hsa-miR-30c-5p | [SEC23A](http://www.ncbi.nlm.nih.gov/entrez/query.fcgi?db=gene&cmd=Retrieve&dopt=full_report&list_uids=10484) | Sec23 homolog A, coat complex II component |
| [Details](http://mirdb.org/cgi-bin/target_detail.cgi?targetID=1440150) | 181 | 95 | hsa-miR-30c-5p | [WDR82](http://www.ncbi.nlm.nih.gov/entrez/query.fcgi?db=gene&cmd=Retrieve&dopt=full_report&list_uids=80335) | WD repeat domain 82 |
| [Details](http://mirdb.org/cgi-bin/target_detail.cgi?targetID=1440175) | 182 | 95 | hsa-miR-30c-5p | [MLXIP](http://www.ncbi.nlm.nih.gov/entrez/query.fcgi?db=gene&cmd=Retrieve&dopt=full_report&list_uids=22877) | MLX interacting protein |
| [Details](http://mirdb.org/cgi-bin/target_detail.cgi?targetID=1440210) | 183 | 95 | hsa-miR-30c-5p | [SMAD1](http://www.ncbi.nlm.nih.gov/entrez/query.fcgi?db=gene&cmd=Retrieve&dopt=full_report&list_uids=4086) | SMAD family member 1 |
| [Details](http://mirdb.org/cgi-bin/target_detail.cgi?targetID=1440262) | 184 | 95 | hsa-miR-30c-5p | [COL13A1](http://www.ncbi.nlm.nih.gov/entrez/query.fcgi?db=gene&cmd=Retrieve&dopt=full_report&list_uids=1305) | collagen type XIII alpha 1 chain |
| [Details](http://mirdb.org/cgi-bin/target_detail.cgi?targetID=1440283) | 185 | 95 | hsa-miR-30c-5p | [KIAA0408](http://www.ncbi.nlm.nih.gov/entrez/query.fcgi?db=gene&cmd=Retrieve&dopt=full_report&list_uids=9729) | KIAA0408 |
| [Details](http://mirdb.org/cgi-bin/target_detail.cgi?targetID=1440392) | 186 | 95 | hsa-miR-30c-5p | [RAP2C](http://www.ncbi.nlm.nih.gov/entrez/query.fcgi?db=gene&cmd=Retrieve&dopt=full_report&list_uids=57826) | RAP2C, member of RAS oncogene family |
| [Details](http://mirdb.org/cgi-bin/target_detail.cgi?targetID=1440418) | 187 | 95 | hsa-miR-30c-5p | [ZCCHC2](http://www.ncbi.nlm.nih.gov/entrez/query.fcgi?db=gene&cmd=Retrieve&dopt=full_report&list_uids=54877) | zinc finger CCHC-type containing 2 |
| [Details](http://mirdb.org/cgi-bin/target_detail.cgi?targetID=1440425) | 188 | 95 | hsa-miR-30c-5p | [FKBP3](http://www.ncbi.nlm.nih.gov/entrez/query.fcgi?db=gene&cmd=Retrieve&dopt=full_report&list_uids=2287) | FKBP prolyl isomerase 3 |
| [Details](http://mirdb.org/cgi-bin/target_detail.cgi?targetID=1440525) | 189 | 95 | hsa-miR-30c-5p | [TBC1D10B](http://www.ncbi.nlm.nih.gov/entrez/query.fcgi?db=gene&cmd=Retrieve&dopt=full_report&list_uids=26000) | TBC1 domain family member 10B |
| [Details](http://mirdb.org/cgi-bin/target_detail.cgi?targetID=1440561) | 190 | 95 | hsa-miR-30c-5p | [PRUNE2](http://www.ncbi.nlm.nih.gov/entrez/query.fcgi?db=gene&cmd=Retrieve&dopt=full_report&list_uids=158471) | prune homolog 2 with BCH domain |
| [Details](http://mirdb.org/cgi-bin/target_detail.cgi?targetID=1440614) | 191 | 95 | hsa-miR-30c-5p | [STAC](http://www.ncbi.nlm.nih.gov/entrez/query.fcgi?db=gene&cmd=Retrieve&dopt=full_report&list_uids=6769) | SH3 and cysteine rich domain |
| [Details](http://mirdb.org/cgi-bin/target_detail.cgi?targetID=1440618) | 192 | 95 | hsa-miR-30c-5p | [STX2](http://www.ncbi.nlm.nih.gov/entrez/query.fcgi?db=gene&cmd=Retrieve&dopt=full_report&list_uids=2054) | syntaxin 2 |
| [Details](http://mirdb.org/cgi-bin/target_detail.cgi?targetID=1440748) | 193 | 95 | hsa-miR-30c-5p | [BRD1](http://www.ncbi.nlm.nih.gov/entrez/query.fcgi?db=gene&cmd=Retrieve&dopt=full_report&list_uids=23774) | bromodomain containing 1 |
| [Details](http://mirdb.org/cgi-bin/target_detail.cgi?targetID=1440781) | 194 | 95 | hsa-miR-30c-5p | [CALCR](http://www.ncbi.nlm.nih.gov/entrez/query.fcgi?db=gene&cmd=Retrieve&dopt=full_report&list_uids=799) | calcitonin receptor |
| [Details](http://mirdb.org/cgi-bin/target_detail.cgi?targetID=1440804) | 195 | 95 | hsa-miR-30c-5p | [MEOX2](http://www.ncbi.nlm.nih.gov/entrez/query.fcgi?db=gene&cmd=Retrieve&dopt=full_report&list_uids=4223) | mesenchyme homeobox 2 |
| [Details](http://mirdb.org/cgi-bin/target_detail.cgi?targetID=1440816) | 196 | 95 | hsa-miR-30c-5p | [TNRC6B](http://www.ncbi.nlm.nih.gov/entrez/query.fcgi?db=gene&cmd=Retrieve&dopt=full_report&list_uids=23112) | trinucleotide repeat containing 6B |
| [Details](http://mirdb.org/cgi-bin/target_detail.cgi?targetID=1440827) | 197 | 95 | hsa-miR-30c-5p | [NEURL1B](http://www.ncbi.nlm.nih.gov/entrez/query.fcgi?db=gene&cmd=Retrieve&dopt=full_report&list_uids=54492) | neuralized E3 ubiquitin protein ligase 1B |
| [Details](http://mirdb.org/cgi-bin/target_detail.cgi?targetID=1440871) | 198 | 95 | hsa-miR-30c-5p | [UBE2V2](http://www.ncbi.nlm.nih.gov/entrez/query.fcgi?db=gene&cmd=Retrieve&dopt=full_report&list_uids=7336) | ubiquitin conjugating enzyme E2 V2 |
| [Details](http://mirdb.org/cgi-bin/target_detail.cgi?targetID=1440911) | 199 | 95 | hsa-miR-30c-5p | [PCDH17](http://www.ncbi.nlm.nih.gov/entrez/query.fcgi?db=gene&cmd=Retrieve&dopt=full_report&list_uids=27253) | protocadherin 17 |
| [Details](http://mirdb.org/cgi-bin/target_detail.cgi?targetID=1439498) | 200 | 94 | hsa-miR-30c-5p | [RAB8A](http://www.ncbi.nlm.nih.gov/entrez/query.fcgi?db=gene&cmd=Retrieve&dopt=full_report&list_uids=4218) | RAB8A, member RAS oncogene family |
| [Details](http://mirdb.org/cgi-bin/target_detail.cgi?targetID=1439502) | 201 | 94 | hsa-miR-30c-5p | [MYO1H](http://www.ncbi.nlm.nih.gov/entrez/query.fcgi?db=gene&cmd=Retrieve&dopt=full_report&list_uids=283446) | myosin IH |
| [Details](http://mirdb.org/cgi-bin/target_detail.cgi?targetID=1439507) | 202 | 94 | hsa-miR-30c-5p | [KCTD8](http://www.ncbi.nlm.nih.gov/entrez/query.fcgi?db=gene&cmd=Retrieve&dopt=full_report&list_uids=386617) | potassium channel tetramerization domain containing 8 |
| [Details](http://mirdb.org/cgi-bin/target_detail.cgi?targetID=1439515) | 203 | 94 | hsa-miR-30c-5p | [PSMD7](http://www.ncbi.nlm.nih.gov/entrez/query.fcgi?db=gene&cmd=Retrieve&dopt=full_report&list_uids=5713) | proteasome 26S subunit, non-ATPase 7 |
| [Details](http://mirdb.org/cgi-bin/target_detail.cgi?targetID=1439552) | 204 | 94 | hsa-miR-30c-5p | [SH3RF1](http://www.ncbi.nlm.nih.gov/entrez/query.fcgi?db=gene&cmd=Retrieve&dopt=full_report&list_uids=57630) | SH3 domain containing ring finger 1 |
| [Details](http://mirdb.org/cgi-bin/target_detail.cgi?targetID=1439570) | 205 | 94 | hsa-miR-30c-5p | [RAPGEF4](http://www.ncbi.nlm.nih.gov/entrez/query.fcgi?db=gene&cmd=Retrieve&dopt=full_report&list_uids=11069) | Rap guanine nucleotide exchange factor 4 |
| [Details](http://mirdb.org/cgi-bin/target_detail.cgi?targetID=1439589) | 206 | 94 | hsa-miR-30c-5p | [RAB32](http://www.ncbi.nlm.nih.gov/entrez/query.fcgi?db=gene&cmd=Retrieve&dopt=full_report&list_uids=10981) | RAB32, member RAS oncogene family |
| [Details](http://mirdb.org/cgi-bin/target_detail.cgi?targetID=1439602) | 207 | 94 | hsa-miR-30c-5p | [MAB21L1](http://www.ncbi.nlm.nih.gov/entrez/query.fcgi?db=gene&cmd=Retrieve&dopt=full_report&list_uids=4081) | mab-21 like 1 |
| [Details](http://mirdb.org/cgi-bin/target_detail.cgi?targetID=1439606) | 208 | 94 | hsa-miR-30c-5p | [KMT2C](http://www.ncbi.nlm.nih.gov/entrez/query.fcgi?db=gene&cmd=Retrieve&dopt=full_report&list_uids=58508) | lysine methyltransferase 2C |
| [Details](http://mirdb.org/cgi-bin/target_detail.cgi?targetID=1439634) | 209 | 94 | hsa-miR-30c-5p | [HIVEP1](http://www.ncbi.nlm.nih.gov/entrez/query.fcgi?db=gene&cmd=Retrieve&dopt=full_report&list_uids=3096) | human immunodeficiency virus type I enhancer binding protein 1 |
| [Details](http://mirdb.org/cgi-bin/target_detail.cgi?targetID=1439639) | 210 | 94 | hsa-miR-30c-5p | [PGM1](http://www.ncbi.nlm.nih.gov/entrez/query.fcgi?db=gene&cmd=Retrieve&dopt=full_report&list_uids=5236) | phosphoglucomutase 1 |
| [Details](http://mirdb.org/cgi-bin/target_detail.cgi?targetID=1439643) | 211 | 94 | hsa-miR-30c-5p | [FLVCR2](http://www.ncbi.nlm.nih.gov/entrez/query.fcgi?db=gene&cmd=Retrieve&dopt=full_report&list_uids=55640) | feline leukemia virus subgroup C cellular receptor family member 2 |
| [Details](http://mirdb.org/cgi-bin/target_detail.cgi?targetID=1439667) | 212 | 94 | hsa-miR-30c-5p | [FAM43A](http://www.ncbi.nlm.nih.gov/entrez/query.fcgi?db=gene&cmd=Retrieve&dopt=full_report&list_uids=131583) | family with sequence similarity 43 member A |
| [Details](http://mirdb.org/cgi-bin/target_detail.cgi?targetID=1439743) | 213 | 94 | hsa-miR-30c-5p | [ADAMTS6](http://www.ncbi.nlm.nih.gov/entrez/query.fcgi?db=gene&cmd=Retrieve&dopt=full_report&list_uids=11174) | ADAM metallopeptidase with thrombospondin type 1 motif 6 |
| [Details](http://mirdb.org/cgi-bin/target_detail.cgi?targetID=1439803) | 214 | 94 | hsa-miR-30c-5p | [SCN1A](http://www.ncbi.nlm.nih.gov/entrez/query.fcgi?db=gene&cmd=Retrieve&dopt=full_report&list_uids=6323) | sodium voltage-gated channel alpha subunit 1 |
| [Details](http://mirdb.org/cgi-bin/target_detail.cgi?targetID=1439810) | 215 | 94 | hsa-miR-30c-5p | [VAT1L](http://www.ncbi.nlm.nih.gov/entrez/query.fcgi?db=gene&cmd=Retrieve&dopt=full_report&list_uids=57687) | vesicle amine transport 1 like |
| [Details](http://mirdb.org/cgi-bin/target_detail.cgi?targetID=1439814) | 216 | 94 | hsa-miR-30c-5p | [HNRNPUL2](http://www.ncbi.nlm.nih.gov/entrez/query.fcgi?db=gene&cmd=Retrieve&dopt=full_report&list_uids=221092) | heterogeneous nuclear ribonucleoprotein U like 2 |
| [Details](http://mirdb.org/cgi-bin/target_detail.cgi?targetID=1439827) | 217 | 94 | hsa-miR-30c-5p | [FRMD6](http://www.ncbi.nlm.nih.gov/entrez/query.fcgi?db=gene&cmd=Retrieve&dopt=full_report&list_uids=122786) | FERM domain containing 6 |
| [Details](http://mirdb.org/cgi-bin/target_detail.cgi?targetID=1439848) | 218 | 94 | hsa-miR-30c-5p | [UGT2A3](http://www.ncbi.nlm.nih.gov/entrez/query.fcgi?db=gene&cmd=Retrieve&dopt=full_report&list_uids=79799) | UDP glucuronosyltransferase family 2 member A3 |
| [Details](http://mirdb.org/cgi-bin/target_detail.cgi?targetID=1439851) | 219 | 94 | hsa-miR-30c-5p | [SCAF4](http://www.ncbi.nlm.nih.gov/entrez/query.fcgi?db=gene&cmd=Retrieve&dopt=full_report&list_uids=57466) | SR-related CTD associated factor 4 |
| [Details](http://mirdb.org/cgi-bin/target_detail.cgi?targetID=1439883) | 220 | 94 | hsa-miR-30c-5p | [SNX33](http://www.ncbi.nlm.nih.gov/entrez/query.fcgi?db=gene&cmd=Retrieve&dopt=full_report&list_uids=257364) | sorting nexin 33 |
| [Details](http://mirdb.org/cgi-bin/target_detail.cgi?targetID=1439952) | 221 | 94 | hsa-miR-30c-5p | [LARGE1](http://www.ncbi.nlm.nih.gov/entrez/query.fcgi?db=gene&cmd=Retrieve&dopt=full_report&list_uids=9215) | LARGE xylosyl- and glucuronyltransferase 1 |
| [Details](http://mirdb.org/cgi-bin/target_detail.cgi?targetID=1439969) | 222 | 94 | hsa-miR-30c-5p | [IL1RAPL2](http://www.ncbi.nlm.nih.gov/entrez/query.fcgi?db=gene&cmd=Retrieve&dopt=full_report&list_uids=26280) | interleukin 1 receptor accessory protein like 2 |
| [Details](http://mirdb.org/cgi-bin/target_detail.cgi?targetID=1440009) | 223 | 94 | hsa-miR-30c-5p | [FNIP2](http://www.ncbi.nlm.nih.gov/entrez/query.fcgi?db=gene&cmd=Retrieve&dopt=full_report&list_uids=57600) | folliculin interacting protein 2 |
| [Details](http://mirdb.org/cgi-bin/target_detail.cgi?targetID=1440064) | 224 | 94 | hsa-miR-30c-5p | [TMEM229A](http://www.ncbi.nlm.nih.gov/entrez/query.fcgi?db=gene&cmd=Retrieve&dopt=full_report&list_uids=730130) | transmembrane protein 229A |
| [Details](http://mirdb.org/cgi-bin/target_detail.cgi?targetID=1440084) | 225 | 94 | hsa-miR-30c-5p | [A1CF](http://www.ncbi.nlm.nih.gov/entrez/query.fcgi?db=gene&cmd=Retrieve&dopt=full_report&list_uids=29974) | APOBEC1 complementation factor |
| [Details](http://mirdb.org/cgi-bin/target_detail.cgi?targetID=1440094) | 226 | 94 | hsa-miR-30c-5p | [NR5A2](http://www.ncbi.nlm.nih.gov/entrez/query.fcgi?db=gene&cmd=Retrieve&dopt=full_report&list_uids=2494) | nuclear receptor subfamily 5 group A member 2 |
| [Details](http://mirdb.org/cgi-bin/target_detail.cgi?targetID=1440129) | 227 | 94 | hsa-miR-30c-5p | [ZBTB44](http://www.ncbi.nlm.nih.gov/entrez/query.fcgi?db=gene&cmd=Retrieve&dopt=full_report&list_uids=29068) | zinc finger and BTB domain containing 44 |
| [Details](http://mirdb.org/cgi-bin/target_detail.cgi?targetID=1440173) | 228 | 94 | hsa-miR-30c-5p | [R3HDM1](http://www.ncbi.nlm.nih.gov/entrez/query.fcgi?db=gene&cmd=Retrieve&dopt=full_report&list_uids=23518) | R3H domain containing 1 |
| [Details](http://mirdb.org/cgi-bin/target_detail.cgi?targetID=1440189) | 229 | 94 | hsa-miR-30c-5p | [OSBPL8](http://www.ncbi.nlm.nih.gov/entrez/query.fcgi?db=gene&cmd=Retrieve&dopt=full_report&list_uids=114882) | oxysterol binding protein like 8 |
| [Details](http://mirdb.org/cgi-bin/target_detail.cgi?targetID=1440200) | 230 | 94 | hsa-miR-30c-5p | [TEPSIN](http://www.ncbi.nlm.nih.gov/entrez/query.fcgi?db=gene&cmd=Retrieve&dopt=full_report&list_uids=146705) | TEPSIN, adaptor related protein complex 4 accessory protein |
| [Details](http://mirdb.org/cgi-bin/target_detail.cgi?targetID=1440209) | 231 | 94 | hsa-miR-30c-5p | [TMOD2](http://www.ncbi.nlm.nih.gov/entrez/query.fcgi?db=gene&cmd=Retrieve&dopt=full_report&list_uids=29767) | tropomodulin 2 |
| [Details](http://mirdb.org/cgi-bin/target_detail.cgi?targetID=1440293) | 232 | 94 | hsa-miR-30c-5p | [CD2AP](http://www.ncbi.nlm.nih.gov/entrez/query.fcgi?db=gene&cmd=Retrieve&dopt=full_report&list_uids=23607) | CD2 associated protein |
| [Details](http://mirdb.org/cgi-bin/target_detail.cgi?targetID=1440296) | 233 | 94 | hsa-miR-30c-5p | [BNIP3L](http://www.ncbi.nlm.nih.gov/entrez/query.fcgi?db=gene&cmd=Retrieve&dopt=full_report&list_uids=665) | BCL2 interacting protein 3 like |
| [Details](http://mirdb.org/cgi-bin/target_detail.cgi?targetID=1440301) | 234 | 94 | hsa-miR-30c-5p | [LGI1](http://www.ncbi.nlm.nih.gov/entrez/query.fcgi?db=gene&cmd=Retrieve&dopt=full_report&list_uids=9211) | leucine rich glioma inactivated 1 |
| [Details](http://mirdb.org/cgi-bin/target_detail.cgi?targetID=1440302) | 235 | 94 | hsa-miR-30c-5p | [RAP1B](http://www.ncbi.nlm.nih.gov/entrez/query.fcgi?db=gene&cmd=Retrieve&dopt=full_report&list_uids=5908) | RAP1B, member of RAS oncogene family |
| [Details](http://mirdb.org/cgi-bin/target_detail.cgi?targetID=1440318) | 236 | 94 | hsa-miR-30c-5p | [CAMK2D](http://www.ncbi.nlm.nih.gov/entrez/query.fcgi?db=gene&cmd=Retrieve&dopt=full_report&list_uids=817) | calcium/calmodulin dependent protein kinase II delta |
| [Details](http://mirdb.org/cgi-bin/target_detail.cgi?targetID=1440330) | 237 | 94 | hsa-miR-30c-5p | [CCNK](http://www.ncbi.nlm.nih.gov/entrez/query.fcgi?db=gene&cmd=Retrieve&dopt=full_report&list_uids=8812) | cyclin K |
| [Details](http://mirdb.org/cgi-bin/target_detail.cgi?targetID=1440341) | 238 | 94 | hsa-miR-30c-5p | [LRRC8C](http://www.ncbi.nlm.nih.gov/entrez/query.fcgi?db=gene&cmd=Retrieve&dopt=full_report&list_uids=84230) | leucine rich repeat containing 8 VRAC subunit C |
| [Details](http://mirdb.org/cgi-bin/target_detail.cgi?targetID=1440376) | 239 | 94 | hsa-miR-30c-5p | [PAWR](http://www.ncbi.nlm.nih.gov/entrez/query.fcgi?db=gene&cmd=Retrieve&dopt=full_report&list_uids=5074) | pro-apoptotic WT1 regulator |
| [Details](http://mirdb.org/cgi-bin/target_detail.cgi?targetID=1440398) | 240 | 94 | hsa-miR-30c-5p | [LPP](http://www.ncbi.nlm.nih.gov/entrez/query.fcgi?db=gene&cmd=Retrieve&dopt=full_report&list_uids=4026) | LIM domain containing preferred translocation partner in lipoma |
| [Details](http://mirdb.org/cgi-bin/target_detail.cgi?targetID=1440426) | 241 | 94 | hsa-miR-30c-5p | [INO80D](http://www.ncbi.nlm.nih.gov/entrez/query.fcgi?db=gene&cmd=Retrieve&dopt=full_report&list_uids=54891) | INO80 complex subunit D |
| [Details](http://mirdb.org/cgi-bin/target_detail.cgi?targetID=1440456) | 242 | 94 | hsa-miR-30c-5p | [P4HA2](http://www.ncbi.nlm.nih.gov/entrez/query.fcgi?db=gene&cmd=Retrieve&dopt=full_report&list_uids=8974) | prolyl 4-hydroxylase subunit alpha 2 |
| [Details](http://mirdb.org/cgi-bin/target_detail.cgi?targetID=1440485) | 243 | 94 | hsa-miR-30c-5p | [SHISA3](http://www.ncbi.nlm.nih.gov/entrez/query.fcgi?db=gene&cmd=Retrieve&dopt=full_report&list_uids=152573) | shisa family member 3 |
| [Details](http://mirdb.org/cgi-bin/target_detail.cgi?targetID=1440527) | 244 | 94 | hsa-miR-30c-5p | [TAOK1](http://www.ncbi.nlm.nih.gov/entrez/query.fcgi?db=gene&cmd=Retrieve&dopt=full_report&list_uids=57551) | TAO kinase 1 |
| [Details](http://mirdb.org/cgi-bin/target_detail.cgi?targetID=1440589) | 245 | 94 | hsa-miR-30c-5p | [PRLR](http://www.ncbi.nlm.nih.gov/entrez/query.fcgi?db=gene&cmd=Retrieve&dopt=full_report&list_uids=5618) | prolactin receptor |
| [Details](http://mirdb.org/cgi-bin/target_detail.cgi?targetID=1440634) | 246 | 94 | hsa-miR-30c-5p | [MZT1](http://www.ncbi.nlm.nih.gov/entrez/query.fcgi?db=gene&cmd=Retrieve&dopt=full_report&list_uids=440145) | mitotic spindle organizing protein 1 |
| [Details](http://mirdb.org/cgi-bin/target_detail.cgi?targetID=1440673) | 247 | 94 | hsa-miR-30c-5p | [ZPBP2](http://www.ncbi.nlm.nih.gov/entrez/query.fcgi?db=gene&cmd=Retrieve&dopt=full_report&list_uids=124626) | zona pellucida binding protein 2 |
| [Details](http://mirdb.org/cgi-bin/target_detail.cgi?targetID=1440674) | 248 | 94 | hsa-miR-30c-5p | [ZNF608](http://www.ncbi.nlm.nih.gov/entrez/query.fcgi?db=gene&cmd=Retrieve&dopt=full_report&list_uids=57507) | zinc finger protein 608 |
| [Details](http://mirdb.org/cgi-bin/target_detail.cgi?targetID=1440694) | 249 | 94 | hsa-miR-30c-5p | [MAP6](http://www.ncbi.nlm.nih.gov/entrez/query.fcgi?db=gene&cmd=Retrieve&dopt=full_report&list_uids=4135) | microtubule associated protein 6 |
| [Details](http://mirdb.org/cgi-bin/target_detail.cgi?targetID=1440709) | 250 | 94 | hsa-miR-30c-5p | [SEMA6B](http://www.ncbi.nlm.nih.gov/entrez/query.fcgi?db=gene&cmd=Retrieve&dopt=full_report&list_uids=10501) | semaphorin 6B |
| [Details](http://mirdb.org/cgi-bin/target_detail.cgi?targetID=1440759) | 251 | 94 | hsa-miR-30c-5p | [ERLIN1](http://www.ncbi.nlm.nih.gov/entrez/query.fcgi?db=gene&cmd=Retrieve&dopt=full_report&list_uids=10613) | ER lipid raft associated 1 |
| [Details](http://mirdb.org/cgi-bin/target_detail.cgi?targetID=1440878) | 252 | 94 | hsa-miR-30c-5p | [SACS](http://www.ncbi.nlm.nih.gov/entrez/query.fcgi?db=gene&cmd=Retrieve&dopt=full_report&list_uids=26278) | sacsin molecular chaperone |
| [Details](http://mirdb.org/cgi-bin/target_detail.cgi?targetID=1440887) | 253 | 94 | hsa-miR-30c-5p | [OTUD4](http://www.ncbi.nlm.nih.gov/entrez/query.fcgi?db=gene&cmd=Retrieve&dopt=full_report&list_uids=54726) | OTU deubiquitinase 4 |
| [Details](http://mirdb.org/cgi-bin/target_detail.cgi?targetID=1440968) | 254 | 94 | hsa-miR-30c-5p | [OXR1](http://www.ncbi.nlm.nih.gov/entrez/query.fcgi?db=gene&cmd=Retrieve&dopt=full_report&list_uids=55074) | oxidation resistance 1 |
| [Details](http://mirdb.org/cgi-bin/target_detail.cgi?targetID=1440987) | 255 | 94 | hsa-miR-30c-5p | [DSG2](http://www.ncbi.nlm.nih.gov/entrez/query.fcgi?db=gene&cmd=Retrieve&dopt=full_report&list_uids=1829) | desmoglein 2 |
| [Details](http://mirdb.org/cgi-bin/target_detail.cgi?targetID=1439466) | 256 | 93 | hsa-miR-30c-5p | [RAPH1](http://www.ncbi.nlm.nih.gov/entrez/query.fcgi?db=gene&cmd=Retrieve&dopt=full_report&list_uids=65059) | Ras association (RalGDS/AF-6) and pleckstrin homology domains 1 |
| [Details](http://mirdb.org/cgi-bin/target_detail.cgi?targetID=1439537) | 257 | 93 | hsa-miR-30c-5p | [CPNE8](http://www.ncbi.nlm.nih.gov/entrez/query.fcgi?db=gene&cmd=Retrieve&dopt=full_report&list_uids=144402) | copine 8 |
| [Details](http://mirdb.org/cgi-bin/target_detail.cgi?targetID=1439554) | 258 | 93 | hsa-miR-30c-5p | [HIC2](http://www.ncbi.nlm.nih.gov/entrez/query.fcgi?db=gene&cmd=Retrieve&dopt=full_report&list_uids=23119) | HIC ZBTB transcriptional repressor 2 |
| [Details](http://mirdb.org/cgi-bin/target_detail.cgi?targetID=1439582) | 259 | 93 | hsa-miR-30c-5p | [ZNF711](http://www.ncbi.nlm.nih.gov/entrez/query.fcgi?db=gene&cmd=Retrieve&dopt=full_report&list_uids=7552) | zinc finger protein 711 |
| [Details](http://mirdb.org/cgi-bin/target_detail.cgi?targetID=1439609) | 260 | 93 | hsa-miR-30c-5p | [PPARGC1A](http://www.ncbi.nlm.nih.gov/entrez/query.fcgi?db=gene&cmd=Retrieve&dopt=full_report&list_uids=10891) | PPARG coactivator 1 alpha |
| [Details](http://mirdb.org/cgi-bin/target_detail.cgi?targetID=1439676) | 261 | 93 | hsa-miR-30c-5p | [PALM2](http://www.ncbi.nlm.nih.gov/entrez/query.fcgi?db=gene&cmd=Retrieve&dopt=full_report&list_uids=114299) | paralemmin 2 |
| [Details](http://mirdb.org/cgi-bin/target_detail.cgi?targetID=1439747) | 262 | 93 | hsa-miR-30c-5p | [ME1](http://www.ncbi.nlm.nih.gov/entrez/query.fcgi?db=gene&cmd=Retrieve&dopt=full_report&list_uids=4199) | malic enzyme 1 |
| [Details](http://mirdb.org/cgi-bin/target_detail.cgi?targetID=1439756) | 263 | 93 | hsa-miR-30c-5p | [SCN8A](http://www.ncbi.nlm.nih.gov/entrez/query.fcgi?db=gene&cmd=Retrieve&dopt=full_report&list_uids=6334) | sodium voltage-gated channel alpha subunit 8 |
| [Details](http://mirdb.org/cgi-bin/target_detail.cgi?targetID=1439811) | 264 | 93 | hsa-miR-30c-5p | [CAND1](http://www.ncbi.nlm.nih.gov/entrez/query.fcgi?db=gene&cmd=Retrieve&dopt=full_report&list_uids=55832) | cullin associated and neddylation dissociated 1 |
| [Details](http://mirdb.org/cgi-bin/target_detail.cgi?targetID=1439823) | 265 | 93 | hsa-miR-30c-5p | [TTLL7](http://www.ncbi.nlm.nih.gov/entrez/query.fcgi?db=gene&cmd=Retrieve&dopt=full_report&list_uids=79739) | tubulin tyrosine ligase like 7 |
| [Details](http://mirdb.org/cgi-bin/target_detail.cgi?targetID=1439830) | 266 | 93 | hsa-miR-30c-5p | [MBNL3](http://www.ncbi.nlm.nih.gov/entrez/query.fcgi?db=gene&cmd=Retrieve&dopt=full_report&list_uids=55796) | muscleblind like splicing regulator 3 |
| [Details](http://mirdb.org/cgi-bin/target_detail.cgi?targetID=1439885) | 267 | 93 | hsa-miR-30c-5p | [DOK5](http://www.ncbi.nlm.nih.gov/entrez/query.fcgi?db=gene&cmd=Retrieve&dopt=full_report&list_uids=55816) | docking protein 5 |
| [Details](http://mirdb.org/cgi-bin/target_detail.cgi?targetID=1439893) | 268 | 93 | hsa-miR-30c-5p | [NADK](http://www.ncbi.nlm.nih.gov/entrez/query.fcgi?db=gene&cmd=Retrieve&dopt=full_report&list_uids=65220) | NAD kinase |
| [Details](http://mirdb.org/cgi-bin/target_detail.cgi?targetID=1439926) | 269 | 93 | hsa-miR-30c-5p | [PTPDC1](http://www.ncbi.nlm.nih.gov/entrez/query.fcgi?db=gene&cmd=Retrieve&dopt=full_report&list_uids=138639) | protein tyrosine phosphatase domain containing 1 |
| [Details](http://mirdb.org/cgi-bin/target_detail.cgi?targetID=1439951) | 270 | 93 | hsa-miR-30c-5p | [FAM210B](http://www.ncbi.nlm.nih.gov/entrez/query.fcgi?db=gene&cmd=Retrieve&dopt=full_report&list_uids=116151) | family with sequence similarity 210 member B |
| [Details](http://mirdb.org/cgi-bin/target_detail.cgi?targetID=1439967) | 271 | 93 | hsa-miR-30c-5p | [MAST3](http://www.ncbi.nlm.nih.gov/entrez/query.fcgi?db=gene&cmd=Retrieve&dopt=full_report&list_uids=23031) | microtubule associated serine/threonine kinase 3 |
| [Details](http://mirdb.org/cgi-bin/target_detail.cgi?targetID=1440006) | 272 | 93 | hsa-miR-30c-5p | [FBXO32](http://www.ncbi.nlm.nih.gov/entrez/query.fcgi?db=gene&cmd=Retrieve&dopt=full_report&list_uids=114907) | F-box protein 32 |
| [Details](http://mirdb.org/cgi-bin/target_detail.cgi?targetID=1440111) | 273 | 93 | hsa-miR-30c-5p | [RUNDC3B](http://www.ncbi.nlm.nih.gov/entrez/query.fcgi?db=gene&cmd=Retrieve&dopt=full_report&list_uids=154661) | RUN domain containing 3B |
| [Details](http://mirdb.org/cgi-bin/target_detail.cgi?targetID=1440118) | 274 | 93 | hsa-miR-30c-5p | [SPAST](http://www.ncbi.nlm.nih.gov/entrez/query.fcgi?db=gene&cmd=Retrieve&dopt=full_report&list_uids=6683) | spastin |
| [Details](http://mirdb.org/cgi-bin/target_detail.cgi?targetID=1440134) | 275 | 93 | hsa-miR-30c-5p | [SIX4](http://www.ncbi.nlm.nih.gov/entrez/query.fcgi?db=gene&cmd=Retrieve&dopt=full_report&list_uids=51804) | SIX homeobox 4 |
| [Details](http://mirdb.org/cgi-bin/target_detail.cgi?targetID=1440143) | 276 | 93 | hsa-miR-30c-5p | [SLC38A7](http://www.ncbi.nlm.nih.gov/entrez/query.fcgi?db=gene&cmd=Retrieve&dopt=full_report&list_uids=55238) | solute carrier family 38 member 7 |
| [Details](http://mirdb.org/cgi-bin/target_detail.cgi?targetID=1440207) | 277 | 93 | hsa-miR-30c-5p | [LRRC40](http://www.ncbi.nlm.nih.gov/entrez/query.fcgi?db=gene&cmd=Retrieve&dopt=full_report&list_uids=55631) | leucine rich repeat containing 40 |
| [Details](http://mirdb.org/cgi-bin/target_detail.cgi?targetID=1440223) | 278 | 93 | hsa-miR-30c-5p | [YPEL5](http://www.ncbi.nlm.nih.gov/entrez/query.fcgi?db=gene&cmd=Retrieve&dopt=full_report&list_uids=51646) | yippee like 5 |
| [Details](http://mirdb.org/cgi-bin/target_detail.cgi?targetID=1440232) | 279 | 93 | hsa-miR-30c-5p | [VPS26B](http://www.ncbi.nlm.nih.gov/entrez/query.fcgi?db=gene&cmd=Retrieve&dopt=full_report&list_uids=112936) | VPS26, retromer complex component B |
| [Details](http://mirdb.org/cgi-bin/target_detail.cgi?targetID=1440242) | 280 | 93 | hsa-miR-30c-5p | [PICALM](http://www.ncbi.nlm.nih.gov/entrez/query.fcgi?db=gene&cmd=Retrieve&dopt=full_report&list_uids=8301) | phosphatidylinositol binding clathrin assembly protein |
| [Details](http://mirdb.org/cgi-bin/target_detail.cgi?targetID=1440268) | 281 | 93 | hsa-miR-30c-5p | [FAM110B](http://www.ncbi.nlm.nih.gov/entrez/query.fcgi?db=gene&cmd=Retrieve&dopt=full_report&list_uids=90362) | family with sequence similarity 110 member B |
| [Details](http://mirdb.org/cgi-bin/target_detail.cgi?targetID=1440332) | 282 | 93 | hsa-miR-30c-5p | [TMEM87B](http://www.ncbi.nlm.nih.gov/entrez/query.fcgi?db=gene&cmd=Retrieve&dopt=full_report&list_uids=84910) | transmembrane protein 87B |
| [Details](http://mirdb.org/cgi-bin/target_detail.cgi?targetID=1440370) | 283 | 93 | hsa-miR-30c-5p | [NTNG1](http://www.ncbi.nlm.nih.gov/entrez/query.fcgi?db=gene&cmd=Retrieve&dopt=full_report&list_uids=22854) | netrin G1 |
| [Details](http://mirdb.org/cgi-bin/target_detail.cgi?targetID=1440386) | 284 | 93 | hsa-miR-30c-5p | [AFAP1L2](http://www.ncbi.nlm.nih.gov/entrez/query.fcgi?db=gene&cmd=Retrieve&dopt=full_report&list_uids=84632) | actin filament associated protein 1 like 2 |
| [Details](http://mirdb.org/cgi-bin/target_detail.cgi?targetID=1440407) | 285 | 93 | hsa-miR-30c-5p | [MYBL2](http://www.ncbi.nlm.nih.gov/entrez/query.fcgi?db=gene&cmd=Retrieve&dopt=full_report&list_uids=4605) | MYB proto-oncogene like 2 |
| [Details](http://mirdb.org/cgi-bin/target_detail.cgi?targetID=1440430) | 286 | 93 | hsa-miR-30c-5p | [MAN1A2](http://www.ncbi.nlm.nih.gov/entrez/query.fcgi?db=gene&cmd=Retrieve&dopt=full_report&list_uids=10905) | mannosidase alpha class 1A member 2 |
| [Details](http://mirdb.org/cgi-bin/target_detail.cgi?targetID=1440435) | 287 | 93 | hsa-miR-30c-5p | [MCF2L](http://www.ncbi.nlm.nih.gov/entrez/query.fcgi?db=gene&cmd=Retrieve&dopt=full_report&list_uids=23263) | MCF.2 cell line derived transforming sequence like |
| [Details](http://mirdb.org/cgi-bin/target_detail.cgi?targetID=1440483) | 288 | 93 | hsa-miR-30c-5p | [COL9A3](http://www.ncbi.nlm.nih.gov/entrez/query.fcgi?db=gene&cmd=Retrieve&dopt=full_report&list_uids=1299) | collagen type IX alpha 3 chain |
| [Details](http://mirdb.org/cgi-bin/target_detail.cgi?targetID=1440516) | 289 | 93 | hsa-miR-30c-5p | [RASA1](http://www.ncbi.nlm.nih.gov/entrez/query.fcgi?db=gene&cmd=Retrieve&dopt=full_report&list_uids=5921) | RAS p21 protein activator 1 |
| [Details](http://mirdb.org/cgi-bin/target_detail.cgi?targetID=1440529) | 290 | 93 | hsa-miR-30c-5p | [MAP3K21](http://www.ncbi.nlm.nih.gov/entrez/query.fcgi?db=gene&cmd=Retrieve&dopt=full_report&list_uids=84451) | mitogen-activated protein kinase kinase kinase 21 |
| [Details](http://mirdb.org/cgi-bin/target_detail.cgi?targetID=1440541) | 291 | 93 | hsa-miR-30c-5p | [DENND1B](http://www.ncbi.nlm.nih.gov/entrez/query.fcgi?db=gene&cmd=Retrieve&dopt=full_report&list_uids=163486) | DENN domain containing 1B |
| [Details](http://mirdb.org/cgi-bin/target_detail.cgi?targetID=1440548) | 292 | 93 | hsa-miR-30c-5p | [UBN1](http://www.ncbi.nlm.nih.gov/entrez/query.fcgi?db=gene&cmd=Retrieve&dopt=full_report&list_uids=29855) | ubinuclein 1 |
| [Details](http://mirdb.org/cgi-bin/target_detail.cgi?targetID=1440556) | 293 | 93 | hsa-miR-30c-5p | [BCL11B](http://www.ncbi.nlm.nih.gov/entrez/query.fcgi?db=gene&cmd=Retrieve&dopt=full_report&list_uids=64919) | BCL11B, BAF complex component |
| [Details](http://mirdb.org/cgi-bin/target_detail.cgi?targetID=1440578) | 294 | 93 | hsa-miR-30c-5p | [LYPLAL1](http://www.ncbi.nlm.nih.gov/entrez/query.fcgi?db=gene&cmd=Retrieve&dopt=full_report&list_uids=127018) | lysophospholipase like 1 |
| [Details](http://mirdb.org/cgi-bin/target_detail.cgi?targetID=1440605) | 295 | 93 | hsa-miR-30c-5p | [LRRK2](http://www.ncbi.nlm.nih.gov/entrez/query.fcgi?db=gene&cmd=Retrieve&dopt=full_report&list_uids=120892) | leucine rich repeat kinase 2 |
| [Details](http://mirdb.org/cgi-bin/target_detail.cgi?targetID=1440613) | 296 | 93 | hsa-miR-30c-5p | [RARRES1](http://www.ncbi.nlm.nih.gov/entrez/query.fcgi?db=gene&cmd=Retrieve&dopt=full_report&list_uids=5918) | retinoic acid receptor responder 1 |
| [Details](http://mirdb.org/cgi-bin/target_detail.cgi?targetID=1440619) | 297 | 93 | hsa-miR-30c-5p | [FAM91A1](http://www.ncbi.nlm.nih.gov/entrez/query.fcgi?db=gene&cmd=Retrieve&dopt=full_report&list_uids=157769) | family with sequence similarity 91 member A1 |
| [Details](http://mirdb.org/cgi-bin/target_detail.cgi?targetID=1440655) | 298 | 93 | hsa-miR-30c-5p | [WASHC4](http://www.ncbi.nlm.nih.gov/entrez/query.fcgi?db=gene&cmd=Retrieve&dopt=full_report&list_uids=23325) | WASH complex subunit 4 |
| [Details](http://mirdb.org/cgi-bin/target_detail.cgi?targetID=1440662) | 299 | 93 | hsa-miR-30c-5p | [LOC283710](http://www.ncbi.nlm.nih.gov/entrez/query.fcgi?db=gene&cmd=Retrieve&dopt=full_report&list_uids=283710) | uncharacterized LOC283710 |
| [Details](http://mirdb.org/cgi-bin/target_detail.cgi?targetID=1440687) | 300 | 93 | hsa-miR-30c-5p | [GRIA2](http://www.ncbi.nlm.nih.gov/entrez/query.fcgi?db=gene&cmd=Retrieve&dopt=full_report&list_uids=2891) | glutamate ionotropic receptor AMPA type subunit 2 |
| [Details](http://mirdb.org/cgi-bin/target_detail.cgi?targetID=1440725) | 301 | 93 | hsa-miR-30c-5p | [GATM](http://www.ncbi.nlm.nih.gov/entrez/query.fcgi?db=gene&cmd=Retrieve&dopt=full_report&list_uids=2628) | glycine amidinotransferase |
| [Details](http://mirdb.org/cgi-bin/target_detail.cgi?targetID=1440765) | 302 | 93 | hsa-miR-30c-5p | [DLL4](http://www.ncbi.nlm.nih.gov/entrez/query.fcgi?db=gene&cmd=Retrieve&dopt=full_report&list_uids=54567) | delta like canonical Notch ligand 4 |
| [Details](http://mirdb.org/cgi-bin/target_detail.cgi?targetID=1440789) | 303 | 93 | hsa-miR-30c-5p | [PIK3CD](http://www.ncbi.nlm.nih.gov/entrez/query.fcgi?db=gene&cmd=Retrieve&dopt=full_report&list_uids=5293) | phosphatidylinositol-4,5-bisphosphate 3-kinase catalytic subunit delta |
| [Details](http://mirdb.org/cgi-bin/target_detail.cgi?targetID=1440846) | 304 | 93 | hsa-miR-30c-5p | [GLCCI1](http://www.ncbi.nlm.nih.gov/entrez/query.fcgi?db=gene&cmd=Retrieve&dopt=full_report&list_uids=113263) | glucocorticoid induced 1 |
| [Details](http://mirdb.org/cgi-bin/target_detail.cgi?targetID=1440863) | 305 | 93 | hsa-miR-30c-5p | [PER2](http://www.ncbi.nlm.nih.gov/entrez/query.fcgi?db=gene&cmd=Retrieve&dopt=full_report&list_uids=8864) | period circadian regulator 2 |
| [Details](http://mirdb.org/cgi-bin/target_detail.cgi?targetID=1440941) | 306 | 93 | hsa-miR-30c-5p | [MAP3K2](http://www.ncbi.nlm.nih.gov/entrez/query.fcgi?db=gene&cmd=Retrieve&dopt=full_report&list_uids=10746) | mitogen-activated protein kinase kinase kinase 2 |
| [Details](http://mirdb.org/cgi-bin/target_detail.cgi?targetID=1439476) | 307 | 92 | hsa-miR-30c-5p | [FAM133A](http://www.ncbi.nlm.nih.gov/entrez/query.fcgi?db=gene&cmd=Retrieve&dopt=full_report&list_uids=286499) | family with sequence similarity 133 member A |
| [Details](http://mirdb.org/cgi-bin/target_detail.cgi?targetID=1439535) | 308 | 92 | hsa-miR-30c-5p | [LRFN2](http://www.ncbi.nlm.nih.gov/entrez/query.fcgi?db=gene&cmd=Retrieve&dopt=full_report&list_uids=57497) | leucine rich repeat and fibronectin type III domain containing 2 |
| [Details](http://mirdb.org/cgi-bin/target_detail.cgi?targetID=1439561) | 309 | 92 | hsa-miR-30c-5p | [ZNF518A](http://www.ncbi.nlm.nih.gov/entrez/query.fcgi?db=gene&cmd=Retrieve&dopt=full_report&list_uids=9849) | zinc finger protein 518A |
| [Details](http://mirdb.org/cgi-bin/target_detail.cgi?targetID=1439562) | 310 | 92 | hsa-miR-30c-5p | [LRRC8D](http://www.ncbi.nlm.nih.gov/entrez/query.fcgi?db=gene&cmd=Retrieve&dopt=full_report&list_uids=55144) | leucine rich repeat containing 8 VRAC subunit D |
| [Details](http://mirdb.org/cgi-bin/target_detail.cgi?targetID=1439632) | 311 | 92 | hsa-miR-30c-5p | [ARHGEF6](http://www.ncbi.nlm.nih.gov/entrez/query.fcgi?db=gene&cmd=Retrieve&dopt=full_report&list_uids=9459) | Rac/Cdc42 guanine nucleotide exchange factor 6 |
| [Details](http://mirdb.org/cgi-bin/target_detail.cgi?targetID=1439677) | 312 | 92 | hsa-miR-30c-5p | [CPSF6](http://www.ncbi.nlm.nih.gov/entrez/query.fcgi?db=gene&cmd=Retrieve&dopt=full_report&list_uids=11052) | cleavage and polyadenylation specific factor 6 |
| [Details](http://mirdb.org/cgi-bin/target_detail.cgi?targetID=1439681) | 313 | 92 | hsa-miR-30c-5p | [RAB38](http://www.ncbi.nlm.nih.gov/entrez/query.fcgi?db=gene&cmd=Retrieve&dopt=full_report&list_uids=23682) | RAB38, member RAS oncogene family |
| [Details](http://mirdb.org/cgi-bin/target_detail.cgi?targetID=1439693) | 314 | 92 | hsa-miR-30c-5p | [CALU](http://www.ncbi.nlm.nih.gov/entrez/query.fcgi?db=gene&cmd=Retrieve&dopt=full_report&list_uids=813) | calumenin |
| [Details](http://mirdb.org/cgi-bin/target_detail.cgi?targetID=1439695) | 315 | 92 | hsa-miR-30c-5p | [FAM126B](http://www.ncbi.nlm.nih.gov/entrez/query.fcgi?db=gene&cmd=Retrieve&dopt=full_report&list_uids=285172) | family with sequence similarity 126 member B |
| [Details](http://mirdb.org/cgi-bin/target_detail.cgi?targetID=1439720) | 316 | 92 | hsa-miR-30c-5p | [UNC5C](http://www.ncbi.nlm.nih.gov/entrez/query.fcgi?db=gene&cmd=Retrieve&dopt=full_report&list_uids=8633) | unc-5 netrin receptor C |
| [Details](http://mirdb.org/cgi-bin/target_detail.cgi?targetID=1439721) | 317 | 92 | hsa-miR-30c-5p | [MEX3B](http://www.ncbi.nlm.nih.gov/entrez/query.fcgi?db=gene&cmd=Retrieve&dopt=full_report&list_uids=84206) | mex-3 RNA binding family member B |
| [Details](http://mirdb.org/cgi-bin/target_detail.cgi?targetID=1439732) | 318 | 92 | hsa-miR-30c-5p | [MAP3K13](http://www.ncbi.nlm.nih.gov/entrez/query.fcgi?db=gene&cmd=Retrieve&dopt=full_report&list_uids=9175) | mitogen-activated protein kinase kinase kinase 13 |
| [Details](http://mirdb.org/cgi-bin/target_detail.cgi?targetID=1439745) | 319 | 92 | hsa-miR-30c-5p | [TTBK1](http://www.ncbi.nlm.nih.gov/entrez/query.fcgi?db=gene&cmd=Retrieve&dopt=full_report&list_uids=84630) | tau tubulin kinase 1 |
| [Details](http://mirdb.org/cgi-bin/target_detail.cgi?targetID=1439761) | 320 | 92 | hsa-miR-30c-5p | [RBM12](http://www.ncbi.nlm.nih.gov/entrez/query.fcgi?db=gene&cmd=Retrieve&dopt=full_report&list_uids=10137) | RNA binding motif protein 12 |
| [Details](http://mirdb.org/cgi-bin/target_detail.cgi?targetID=1439767) | 321 | 92 | hsa-miR-30c-5p | [DNMT3A](http://www.ncbi.nlm.nih.gov/entrez/query.fcgi?db=gene&cmd=Retrieve&dopt=full_report&list_uids=1788) | DNA methyltransferase 3 alpha |
| [Details](http://mirdb.org/cgi-bin/target_detail.cgi?targetID=1439798) | 322 | 92 | hsa-miR-30c-5p | [EDNRA](http://www.ncbi.nlm.nih.gov/entrez/query.fcgi?db=gene&cmd=Retrieve&dopt=full_report&list_uids=1909) | endothelin receptor type A |
| [Details](http://mirdb.org/cgi-bin/target_detail.cgi?targetID=1439889) | 323 | 92 | hsa-miR-30c-5p | [BNC1](http://www.ncbi.nlm.nih.gov/entrez/query.fcgi?db=gene&cmd=Retrieve&dopt=full_report&list_uids=646) | basonuclin 1 |
| [Details](http://mirdb.org/cgi-bin/target_detail.cgi?targetID=1439921) | 324 | 92 | hsa-miR-30c-5p | [GLDC](http://www.ncbi.nlm.nih.gov/entrez/query.fcgi?db=gene&cmd=Retrieve&dopt=full_report&list_uids=2731) | glycine decarboxylase |
| [Details](http://mirdb.org/cgi-bin/target_detail.cgi?targetID=1439983) | 325 | 92 | hsa-miR-30c-5p | [CBX2](http://www.ncbi.nlm.nih.gov/entrez/query.fcgi?db=gene&cmd=Retrieve&dopt=full_report&list_uids=84733) | chromobox 2 |
| [Details](http://mirdb.org/cgi-bin/target_detail.cgi?targetID=1440065) | 326 | 92 | hsa-miR-30c-5p | [STX16](http://www.ncbi.nlm.nih.gov/entrez/query.fcgi?db=gene&cmd=Retrieve&dopt=full_report&list_uids=8675) | syntaxin 16 |
| [Details](http://mirdb.org/cgi-bin/target_detail.cgi?targetID=1440115) | 327 | 92 | hsa-miR-30c-5p | [C4orf19](http://www.ncbi.nlm.nih.gov/entrez/query.fcgi?db=gene&cmd=Retrieve&dopt=full_report&list_uids=55286) | chromosome 4 open reading frame 19 |
| [Details](http://mirdb.org/cgi-bin/target_detail.cgi?targetID=1440178) | 328 | 92 | hsa-miR-30c-5p | [ACTC1](http://www.ncbi.nlm.nih.gov/entrez/query.fcgi?db=gene&cmd=Retrieve&dopt=full_report&list_uids=70) | actin, alpha, cardiac muscle 1 |
| [Details](http://mirdb.org/cgi-bin/target_detail.cgi?targetID=1440244) | 329 | 92 | hsa-miR-30c-5p | [JPH4](http://www.ncbi.nlm.nih.gov/entrez/query.fcgi?db=gene&cmd=Retrieve&dopt=full_report&list_uids=84502) | junctophilin 4 |
| [Details](http://mirdb.org/cgi-bin/target_detail.cgi?targetID=1440350) | 330 | 92 | hsa-miR-30c-5p | [ZFY](http://www.ncbi.nlm.nih.gov/entrez/query.fcgi?db=gene&cmd=Retrieve&dopt=full_report&list_uids=7544) | zinc finger protein Y-linked |
| [Details](http://mirdb.org/cgi-bin/target_detail.cgi?targetID=1440351) | 331 | 92 | hsa-miR-30c-5p | [PDSS1](http://www.ncbi.nlm.nih.gov/entrez/query.fcgi?db=gene&cmd=Retrieve&dopt=full_report&list_uids=23590) | decaprenyl diphosphate synthase subunit 1 |
| [Details](http://mirdb.org/cgi-bin/target_detail.cgi?targetID=1440389) | 332 | 92 | hsa-miR-30c-5p | [MYO5A](http://www.ncbi.nlm.nih.gov/entrez/query.fcgi?db=gene&cmd=Retrieve&dopt=full_report&list_uids=4644) | myosin VA |
| [Details](http://mirdb.org/cgi-bin/target_detail.cgi?targetID=1440459) | 333 | 92 | hsa-miR-30c-5p | [PRKAA2](http://www.ncbi.nlm.nih.gov/entrez/query.fcgi?db=gene&cmd=Retrieve&dopt=full_report&list_uids=5563) | protein kinase AMP-activated catalytic subunit alpha 2 |
| [Details](http://mirdb.org/cgi-bin/target_detail.cgi?targetID=1440640) | 334 | 92 | hsa-miR-30c-5p | [PHF13](http://www.ncbi.nlm.nih.gov/entrez/query.fcgi?db=gene&cmd=Retrieve&dopt=full_report&list_uids=148479) | PHD finger protein 13 |
| [Details](http://mirdb.org/cgi-bin/target_detail.cgi?targetID=1440671) | 335 | 92 | hsa-miR-30c-5p | [BAHD1](http://www.ncbi.nlm.nih.gov/entrez/query.fcgi?db=gene&cmd=Retrieve&dopt=full_report&list_uids=22893) | bromo adjacent homology domain containing 1 |
| [Details](http://mirdb.org/cgi-bin/target_detail.cgi?targetID=1440688) | 336 | 92 | hsa-miR-30c-5p | [SLC35F1](http://www.ncbi.nlm.nih.gov/entrez/query.fcgi?db=gene&cmd=Retrieve&dopt=full_report&list_uids=222553) | solute carrier family 35 member F1 |
| [Details](http://mirdb.org/cgi-bin/target_detail.cgi?targetID=1440711) | 337 | 92 | hsa-miR-30c-5p | [YY2](http://www.ncbi.nlm.nih.gov/entrez/query.fcgi?db=gene&cmd=Retrieve&dopt=full_report&list_uids=404281) | YY2 transcription factor |
| [Details](http://mirdb.org/cgi-bin/target_detail.cgi?targetID=1440727) | 338 | 92 | hsa-miR-30c-5p | [SETD7](http://www.ncbi.nlm.nih.gov/entrez/query.fcgi?db=gene&cmd=Retrieve&dopt=full_report&list_uids=80854) | SET domain containing 7, histone lysine methyltransferase |
| [Details](http://mirdb.org/cgi-bin/target_detail.cgi?targetID=1440756) | 339 | 92 | hsa-miR-30c-5p | [TENM3](http://www.ncbi.nlm.nih.gov/entrez/query.fcgi?db=gene&cmd=Retrieve&dopt=full_report&list_uids=55714) | teneurin transmembrane protein 3 |
| [Details](http://mirdb.org/cgi-bin/target_detail.cgi?targetID=1440782) | 340 | 92 | hsa-miR-30c-5p | [CEP41](http://www.ncbi.nlm.nih.gov/entrez/query.fcgi?db=gene&cmd=Retrieve&dopt=full_report&list_uids=95681) | centrosomal protein 41 |
| [Details](http://mirdb.org/cgi-bin/target_detail.cgi?targetID=1440822) | 341 | 92 | hsa-miR-30c-5p | [ZNF280B](http://www.ncbi.nlm.nih.gov/entrez/query.fcgi?db=gene&cmd=Retrieve&dopt=full_report&list_uids=140883) | zinc finger protein 280B |
| [Details](http://mirdb.org/cgi-bin/target_detail.cgi?targetID=1440841) | 342 | 92 | hsa-miR-30c-5p | [CBLB](http://www.ncbi.nlm.nih.gov/entrez/query.fcgi?db=gene&cmd=Retrieve&dopt=full_report&list_uids=868) | Cbl proto-oncogene B |
| [Details](http://mirdb.org/cgi-bin/target_detail.cgi?targetID=1440855) | 343 | 92 | hsa-miR-30c-5p | [CAMKK2](http://www.ncbi.nlm.nih.gov/entrez/query.fcgi?db=gene&cmd=Retrieve&dopt=full_report&list_uids=10645) | calcium/calmodulin dependent protein kinase kinase 2 |
| [Details](http://mirdb.org/cgi-bin/target_detail.cgi?targetID=1440862) | 344 | 92 | hsa-miR-30c-5p | [ACTR1A](http://www.ncbi.nlm.nih.gov/entrez/query.fcgi?db=gene&cmd=Retrieve&dopt=full_report&list_uids=10121) | ARP1 actin related protein 1 homolog A |
| [Details](http://mirdb.org/cgi-bin/target_detail.cgi?targetID=1440907) | 345 | 92 | hsa-miR-30c-5p | [DGKH](http://www.ncbi.nlm.nih.gov/entrez/query.fcgi?db=gene&cmd=Retrieve&dopt=full_report&list_uids=160851) | diacylglycerol kinase eta |
| [Details](http://mirdb.org/cgi-bin/target_detail.cgi?targetID=1440947) | 346 | 92 | hsa-miR-30c-5p | [TRIM13](http://www.ncbi.nlm.nih.gov/entrez/query.fcgi?db=gene&cmd=Retrieve&dopt=full_report&list_uids=10206) | tripartite motif containing 13 |
| [Details](http://mirdb.org/cgi-bin/target_detail.cgi?targetID=1440950) | 347 | 92 | hsa-miR-30c-5p | [RNF165](http://www.ncbi.nlm.nih.gov/entrez/query.fcgi?db=gene&cmd=Retrieve&dopt=full_report&list_uids=494470) | ring finger protein 165 |
| [Details](http://mirdb.org/cgi-bin/target_detail.cgi?targetID=1440961) | 348 | 92 | hsa-miR-30c-5p | [NRIP1](http://www.ncbi.nlm.nih.gov/entrez/query.fcgi?db=gene&cmd=Retrieve&dopt=full_report&list_uids=8204) | nuclear receptor interacting protein 1 |
| [Details](http://mirdb.org/cgi-bin/target_detail.cgi?targetID=1439469) | 349 | 91 | hsa-miR-30c-5p | [CACHD1](http://www.ncbi.nlm.nih.gov/entrez/query.fcgi?db=gene&cmd=Retrieve&dopt=full_report&list_uids=57685) | cache domain containing 1 |
| [Details](http://mirdb.org/cgi-bin/target_detail.cgi?targetID=1439517) | 350 | 91 | hsa-miR-30c-5p | [GALNT1](http://www.ncbi.nlm.nih.gov/entrez/query.fcgi?db=gene&cmd=Retrieve&dopt=full_report&list_uids=2589) | polypeptide N-acetylgalactosaminyltransferase 1 |
| [Details](http://mirdb.org/cgi-bin/target_detail.cgi?targetID=1439544) | 351 | 91 | hsa-miR-30c-5p | [SKP2](http://www.ncbi.nlm.nih.gov/entrez/query.fcgi?db=gene&cmd=Retrieve&dopt=full_report&list_uids=6502) | S-phase kinase associated protein 2 |
| [Details](http://mirdb.org/cgi-bin/target_detail.cgi?targetID=1439586) | 352 | 91 | hsa-miR-30c-5p | [FBXL20](http://www.ncbi.nlm.nih.gov/entrez/query.fcgi?db=gene&cmd=Retrieve&dopt=full_report&list_uids=84961) | F-box and leucine rich repeat protein 20 |
| [Details](http://mirdb.org/cgi-bin/target_detail.cgi?targetID=1439607) | 353 | 91 | hsa-miR-30c-5p | [CDCA7](http://www.ncbi.nlm.nih.gov/entrez/query.fcgi?db=gene&cmd=Retrieve&dopt=full_report&list_uids=83879) | cell division cycle associated 7 |
| [Details](http://mirdb.org/cgi-bin/target_detail.cgi?targetID=1439663) | 354 | 91 | hsa-miR-30c-5p | [PAAF1](http://www.ncbi.nlm.nih.gov/entrez/query.fcgi?db=gene&cmd=Retrieve&dopt=full_report&list_uids=80227) | proteasomal ATPase associated factor 1 |
| [Details](http://mirdb.org/cgi-bin/target_detail.cgi?targetID=1439679) | 355 | 91 | hsa-miR-30c-5p | [LOX](http://www.ncbi.nlm.nih.gov/entrez/query.fcgi?db=gene&cmd=Retrieve&dopt=full_report&list_uids=4015) | lysyl oxidase |
| [Details](http://mirdb.org/cgi-bin/target_detail.cgi?targetID=1439683) | 356 | 91 | hsa-miR-30c-5p | [HIPK2](http://www.ncbi.nlm.nih.gov/entrez/query.fcgi?db=gene&cmd=Retrieve&dopt=full_report&list_uids=28996) | homeodomain interacting protein kinase 2 |
| [Details](http://mirdb.org/cgi-bin/target_detail.cgi?targetID=1439760) | 357 | 91 | hsa-miR-30c-5p | [TSPAN2](http://www.ncbi.nlm.nih.gov/entrez/query.fcgi?db=gene&cmd=Retrieve&dopt=full_report&list_uids=10100) | tetraspanin 2 |
| [Details](http://mirdb.org/cgi-bin/target_detail.cgi?targetID=1439812) | 358 | 91 | hsa-miR-30c-5p | [RAI14](http://www.ncbi.nlm.nih.gov/entrez/query.fcgi?db=gene&cmd=Retrieve&dopt=full_report&list_uids=26064) | retinoic acid induced 14 |
| [Details](http://mirdb.org/cgi-bin/target_detail.cgi?targetID=1439834) | 359 | 91 | hsa-miR-30c-5p | [EPG5](http://www.ncbi.nlm.nih.gov/entrez/query.fcgi?db=gene&cmd=Retrieve&dopt=full_report&list_uids=57724) | ectopic P-granules autophagy protein 5 homolog |
| [Details](http://mirdb.org/cgi-bin/target_detail.cgi?targetID=1439928) | 360 | 91 | hsa-miR-30c-5p | [APBA1](http://www.ncbi.nlm.nih.gov/entrez/query.fcgi?db=gene&cmd=Retrieve&dopt=full_report&list_uids=320) | amyloid beta precursor protein binding family A member 1 |
| [Details](http://mirdb.org/cgi-bin/target_detail.cgi?targetID=1439985) | 361 | 91 | hsa-miR-30c-5p | [C7orf43](http://www.ncbi.nlm.nih.gov/entrez/query.fcgi?db=gene&cmd=Retrieve&dopt=full_report&list_uids=55262) | chromosome 7 open reading frame 43 |
| [Details](http://mirdb.org/cgi-bin/target_detail.cgi?targetID=1440000) | 362 | 91 | hsa-miR-30c-5p | [GRM3](http://www.ncbi.nlm.nih.gov/entrez/query.fcgi?db=gene&cmd=Retrieve&dopt=full_report&list_uids=2913) | glutamate metabotropic receptor 3 |
| [Details](http://mirdb.org/cgi-bin/target_detail.cgi?targetID=1440014) | 363 | 91 | hsa-miR-30c-5p | [MFSD6](http://www.ncbi.nlm.nih.gov/entrez/query.fcgi?db=gene&cmd=Retrieve&dopt=full_report&list_uids=54842) | major facilitator superfamily domain containing 6 |
| [Details](http://mirdb.org/cgi-bin/target_detail.cgi?targetID=1440049) | 364 | 91 | hsa-miR-30c-5p | [GNA13](http://www.ncbi.nlm.nih.gov/entrez/query.fcgi?db=gene&cmd=Retrieve&dopt=full_report&list_uids=10672) | G protein subunit alpha 13 |
| [Details](http://mirdb.org/cgi-bin/target_detail.cgi?targetID=1440107) | 365 | 91 | hsa-miR-30c-5p | [ZFAND5](http://www.ncbi.nlm.nih.gov/entrez/query.fcgi?db=gene&cmd=Retrieve&dopt=full_report&list_uids=7763) | zinc finger AN1-type containing 5 |
| [Details](http://mirdb.org/cgi-bin/target_detail.cgi?targetID=1440117) | 366 | 91 | hsa-miR-30c-5p | [FAM83F](http://www.ncbi.nlm.nih.gov/entrez/query.fcgi?db=gene&cmd=Retrieve&dopt=full_report&list_uids=113828) | family with sequence similarity 83 member F |
| [Details](http://mirdb.org/cgi-bin/target_detail.cgi?targetID=1440137) | 367 | 91 | hsa-miR-30c-5p | [MBTPS2](http://www.ncbi.nlm.nih.gov/entrez/query.fcgi?db=gene&cmd=Retrieve&dopt=full_report&list_uids=51360) | membrane bound transcription factor peptidase, site 2 |
| [Details](http://mirdb.org/cgi-bin/target_detail.cgi?targetID=1440142) | 368 | 91 | hsa-miR-30c-5p | [TOGARAM1](http://www.ncbi.nlm.nih.gov/entrez/query.fcgi?db=gene&cmd=Retrieve&dopt=full_report&list_uids=23116) | TOG array regulator of axonemal microtubules 1 |
| [Details](http://mirdb.org/cgi-bin/target_detail.cgi?targetID=1440165) | 369 | 91 | hsa-miR-30c-5p | [SEPT7](http://www.ncbi.nlm.nih.gov/entrez/query.fcgi?db=gene&cmd=Retrieve&dopt=full_report&list_uids=989) | septin 7 |
| [Details](http://mirdb.org/cgi-bin/target_detail.cgi?targetID=1440184) | 370 | 91 | hsa-miR-30c-5p | [SYPL1](http://www.ncbi.nlm.nih.gov/entrez/query.fcgi?db=gene&cmd=Retrieve&dopt=full_report&list_uids=6856) | synaptophysin like 1 |
| [Details](http://mirdb.org/cgi-bin/target_detail.cgi?targetID=1440198) | 371 | 91 | hsa-miR-30c-5p | [RAD23B](http://www.ncbi.nlm.nih.gov/entrez/query.fcgi?db=gene&cmd=Retrieve&dopt=full_report&list_uids=5887) | RAD23 homolog B, nucleotide excision repair protein |
| [Details](http://mirdb.org/cgi-bin/target_detail.cgi?targetID=1440213) | 372 | 91 | hsa-miR-30c-5p | [RAB23](http://www.ncbi.nlm.nih.gov/entrez/query.fcgi?db=gene&cmd=Retrieve&dopt=full_report&list_uids=51715) | RAB23, member RAS oncogene family |
| [Details](http://mirdb.org/cgi-bin/target_detail.cgi?targetID=1440310) | 373 | 91 | hsa-miR-30c-5p | [PDE4D](http://www.ncbi.nlm.nih.gov/entrez/query.fcgi?db=gene&cmd=Retrieve&dopt=full_report&list_uids=5144) | phosphodiesterase 4D |
| [Details](http://mirdb.org/cgi-bin/target_detail.cgi?targetID=1440329) | 374 | 91 | hsa-miR-30c-5p | [ABL1](http://www.ncbi.nlm.nih.gov/entrez/query.fcgi?db=gene&cmd=Retrieve&dopt=full_report&list_uids=25) | ABL proto-oncogene 1, non-receptor tyrosine kinase |
| [Details](http://mirdb.org/cgi-bin/target_detail.cgi?targetID=1440396) | 375 | 91 | hsa-miR-30c-5p | [ASB4](http://www.ncbi.nlm.nih.gov/entrez/query.fcgi?db=gene&cmd=Retrieve&dopt=full_report&list_uids=51666) | ankyrin repeat and SOCS box containing 4 |
| [Details](http://mirdb.org/cgi-bin/target_detail.cgi?targetID=1440513) | 376 | 91 | hsa-miR-30c-5p | [MMD](http://www.ncbi.nlm.nih.gov/entrez/query.fcgi?db=gene&cmd=Retrieve&dopt=full_report&list_uids=23531) | monocyte to macrophage differentiation associated |
| [Details](http://mirdb.org/cgi-bin/target_detail.cgi?targetID=1440559) | 377 | 91 | hsa-miR-30c-5p | [IP6K3](http://www.ncbi.nlm.nih.gov/entrez/query.fcgi?db=gene&cmd=Retrieve&dopt=full_report&list_uids=117283) | inositol hexakisphosphate kinase 3 |
| [Details](http://mirdb.org/cgi-bin/target_detail.cgi?targetID=1440562) | 378 | 91 | hsa-miR-30c-5p | [CAPZA1](http://www.ncbi.nlm.nih.gov/entrez/query.fcgi?db=gene&cmd=Retrieve&dopt=full_report&list_uids=829) | capping actin protein of muscle Z-line subunit alpha 1 |
| [Details](http://mirdb.org/cgi-bin/target_detail.cgi?targetID=1440580) | 379 | 91 | hsa-miR-30c-5p | [ZNF521](http://www.ncbi.nlm.nih.gov/entrez/query.fcgi?db=gene&cmd=Retrieve&dopt=full_report&list_uids=25925) | zinc finger protein 521 |
| [Details](http://mirdb.org/cgi-bin/target_detail.cgi?targetID=1440586) | 380 | 91 | hsa-miR-30c-5p | [PPP1R1C](http://www.ncbi.nlm.nih.gov/entrez/query.fcgi?db=gene&cmd=Retrieve&dopt=full_report&list_uids=151242) | protein phosphatase 1 regulatory inhibitor subunit 1C |
| [Details](http://mirdb.org/cgi-bin/target_detail.cgi?targetID=1440677) | 381 | 91 | hsa-miR-30c-5p | [VAT1](http://www.ncbi.nlm.nih.gov/entrez/query.fcgi?db=gene&cmd=Retrieve&dopt=full_report&list_uids=10493) | vesicle amine transport 1 |
| [Details](http://mirdb.org/cgi-bin/target_detail.cgi?targetID=1440680) | 382 | 91 | hsa-miR-30c-5p | [NDEL1](http://www.ncbi.nlm.nih.gov/entrez/query.fcgi?db=gene&cmd=Retrieve&dopt=full_report&list_uids=81565) | nudE neurodevelopment protein 1 like 1 |
| [Details](http://mirdb.org/cgi-bin/target_detail.cgi?targetID=1440698) | 383 | 91 | hsa-miR-30c-5p | [ZDHHC17](http://www.ncbi.nlm.nih.gov/entrez/query.fcgi?db=gene&cmd=Retrieve&dopt=full_report&list_uids=23390) | zinc finger DHHC-type containing 17 |
| [Details](http://mirdb.org/cgi-bin/target_detail.cgi?targetID=1440801) | 384 | 91 | hsa-miR-30c-5p | [RHOB](http://www.ncbi.nlm.nih.gov/entrez/query.fcgi?db=gene&cmd=Retrieve&dopt=full_report&list_uids=388) | ras homolog family member B |
| [Details](http://mirdb.org/cgi-bin/target_detail.cgi?targetID=1440856) | 385 | 91 | hsa-miR-30c-5p | [PAXBP1](http://www.ncbi.nlm.nih.gov/entrez/query.fcgi?db=gene&cmd=Retrieve&dopt=full_report&list_uids=94104) | PAX3 and PAX7 binding protein 1 |
| [Details](http://mirdb.org/cgi-bin/target_detail.cgi?targetID=1440876) | 386 | 91 | hsa-miR-30c-5p | [CSNK1A1](http://www.ncbi.nlm.nih.gov/entrez/query.fcgi?db=gene&cmd=Retrieve&dopt=full_report&list_uids=1452) | casein kinase 1 alpha 1 |
| [Details](http://mirdb.org/cgi-bin/target_detail.cgi?targetID=1440910) | 387 | 91 | hsa-miR-30c-5p | [MAP3K5](http://www.ncbi.nlm.nih.gov/entrez/query.fcgi?db=gene&cmd=Retrieve&dopt=full_report&list_uids=4217) | mitogen-activated protein kinase kinase kinase 5 |
| [Details](http://mirdb.org/cgi-bin/target_detail.cgi?targetID=1440946) | 388 | 91 | hsa-miR-30c-5p | [ARID4A](http://www.ncbi.nlm.nih.gov/entrez/query.fcgi?db=gene&cmd=Retrieve&dopt=full_report&list_uids=5926) | AT-rich interaction domain 4A |
| [Details](http://mirdb.org/cgi-bin/target_detail.cgi?targetID=1440979) | 389 | 91 | hsa-miR-30c-5p | [OSTM1](http://www.ncbi.nlm.nih.gov/entrez/query.fcgi?db=gene&cmd=Retrieve&dopt=full_report&list_uids=28962) | osteoclastogenesis associated transmembrane protein 1 |
| [Details](http://mirdb.org/cgi-bin/target_detail.cgi?targetID=1439461) | 390 | 90 | hsa-miR-30c-5p | [PAPOLB](http://www.ncbi.nlm.nih.gov/entrez/query.fcgi?db=gene&cmd=Retrieve&dopt=full_report&list_uids=56903) | poly(A) polymerase beta |
| [Details](http://mirdb.org/cgi-bin/target_detail.cgi?targetID=1439490) | 391 | 90 | hsa-miR-30c-5p | [ZCCHC3](http://www.ncbi.nlm.nih.gov/entrez/query.fcgi?db=gene&cmd=Retrieve&dopt=full_report&list_uids=85364) | zinc finger CCHC-type containing 3 |
| [Details](http://mirdb.org/cgi-bin/target_detail.cgi?targetID=1439510) | 392 | 90 | hsa-miR-30c-5p | [ADAM9](http://www.ncbi.nlm.nih.gov/entrez/query.fcgi?db=gene&cmd=Retrieve&dopt=full_report&list_uids=8754) | ADAM metallopeptidase domain 9 |
| [Details](http://mirdb.org/cgi-bin/target_detail.cgi?targetID=1439572) | 393 | 90 | hsa-miR-30c-5p | [NUP93](http://www.ncbi.nlm.nih.gov/entrez/query.fcgi?db=gene&cmd=Retrieve&dopt=full_report&list_uids=9688) | nucleoporin 93 |
| [Details](http://mirdb.org/cgi-bin/target_detail.cgi?targetID=1439588) | 394 | 90 | hsa-miR-30c-5p | [SLC25A36](http://www.ncbi.nlm.nih.gov/entrez/query.fcgi?db=gene&cmd=Retrieve&dopt=full_report&list_uids=55186) | solute carrier family 25 member 36 |
| [Details](http://mirdb.org/cgi-bin/target_detail.cgi?targetID=1439614) | 395 | 90 | hsa-miR-30c-5p | [MSI2](http://www.ncbi.nlm.nih.gov/entrez/query.fcgi?db=gene&cmd=Retrieve&dopt=full_report&list_uids=124540) | musashi RNA binding protein 2 |
| [Details](http://mirdb.org/cgi-bin/target_detail.cgi?targetID=1439615) | 396 | 90 | hsa-miR-30c-5p | [IQCB1](http://www.ncbi.nlm.nih.gov/entrez/query.fcgi?db=gene&cmd=Retrieve&dopt=full_report&list_uids=9657) | IQ motif containing B1 |
| [Details](http://mirdb.org/cgi-bin/target_detail.cgi?targetID=1439691) | 397 | 90 | hsa-miR-30c-5p | [PPP3CA](http://www.ncbi.nlm.nih.gov/entrez/query.fcgi?db=gene&cmd=Retrieve&dopt=full_report&list_uids=5530) | protein phosphatase 3 catalytic subunit alpha |
| [Details](http://mirdb.org/cgi-bin/target_detail.cgi?targetID=1439711) | 398 | 90 | hsa-miR-30c-5p | [FLVCR1](http://www.ncbi.nlm.nih.gov/entrez/query.fcgi?db=gene&cmd=Retrieve&dopt=full_report&list_uids=28982) | feline leukemia virus subgroup C cellular receptor 1 |
| [Details](http://mirdb.org/cgi-bin/target_detail.cgi?targetID=1439739) | 399 | 90 | hsa-miR-30c-5p | [PPP4R4](http://www.ncbi.nlm.nih.gov/entrez/query.fcgi?db=gene&cmd=Retrieve&dopt=full_report&list_uids=57718) | protein phosphatase 4 regulatory subunit 4 |
| [Details](http://mirdb.org/cgi-bin/target_detail.cgi?targetID=1439759) | 400 | 90 | hsa-miR-30c-5p | [MROH9](http://www.ncbi.nlm.nih.gov/entrez/query.fcgi?db=gene&cmd=Retrieve&dopt=full_report&list_uids=80133) | maestro heat like repeat family member 9 |
| [Details](http://mirdb.org/cgi-bin/target_detail.cgi?targetID=1439769) | 401 | 90 | hsa-miR-30c-5p | [CHD7](http://www.ncbi.nlm.nih.gov/entrez/query.fcgi?db=gene&cmd=Retrieve&dopt=full_report&list_uids=55636) | chromodomain helicase DNA binding protein 7 |
| [Details](http://mirdb.org/cgi-bin/target_detail.cgi?targetID=1439785) | 402 | 90 | hsa-miR-30c-5p | [TFDP1](http://www.ncbi.nlm.nih.gov/entrez/query.fcgi?db=gene&cmd=Retrieve&dopt=full_report&list_uids=7027) | transcription factor Dp-1 |
| [Details](http://mirdb.org/cgi-bin/target_detail.cgi?targetID=1439820) | 403 | 90 | hsa-miR-30c-5p | [TMEM87A](http://www.ncbi.nlm.nih.gov/entrez/query.fcgi?db=gene&cmd=Retrieve&dopt=full_report&list_uids=25963) | transmembrane protein 87A |
| [Details](http://mirdb.org/cgi-bin/target_detail.cgi?targetID=1439853) | 404 | 90 | hsa-miR-30c-5p | [SNX10](http://www.ncbi.nlm.nih.gov/entrez/query.fcgi?db=gene&cmd=Retrieve&dopt=full_report&list_uids=29887) | sorting nexin 10 |
| [Details](http://mirdb.org/cgi-bin/target_detail.cgi?targetID=1439869) | 405 | 90 | hsa-miR-30c-5p | [SAMD4A](http://www.ncbi.nlm.nih.gov/entrez/query.fcgi?db=gene&cmd=Retrieve&dopt=full_report&list_uids=23034) | sterile alpha motif domain containing 4A |
| [Details](http://mirdb.org/cgi-bin/target_detail.cgi?targetID=1439875) | 406 | 90 | hsa-miR-30c-5p | [ELAVL2](http://www.ncbi.nlm.nih.gov/entrez/query.fcgi?db=gene&cmd=Retrieve&dopt=full_report&list_uids=1993) | ELAV like RNA binding protein 2 |
| [Details](http://mirdb.org/cgi-bin/target_detail.cgi?targetID=1439881) | 407 | 90 | hsa-miR-30c-5p | [UBE2V1](http://www.ncbi.nlm.nih.gov/entrez/query.fcgi?db=gene&cmd=Retrieve&dopt=full_report&list_uids=7335) | ubiquitin conjugating enzyme E2 V1 |
| [Details](http://mirdb.org/cgi-bin/target_detail.cgi?targetID=1439898) | 408 | 90 | hsa-miR-30c-5p | [PRDM13](http://www.ncbi.nlm.nih.gov/entrez/query.fcgi?db=gene&cmd=Retrieve&dopt=full_report&list_uids=59336) | PR/SET domain 13 |
| [Details](http://mirdb.org/cgi-bin/target_detail.cgi?targetID=1439907) | 409 | 90 | hsa-miR-30c-5p | [RASD1](http://www.ncbi.nlm.nih.gov/entrez/query.fcgi?db=gene&cmd=Retrieve&dopt=full_report&list_uids=51655) | ras related dexamethasone induced 1 |
| [Details](http://mirdb.org/cgi-bin/target_detail.cgi?targetID=1439958) | 410 | 90 | hsa-miR-30c-5p | [YAF2](http://www.ncbi.nlm.nih.gov/entrez/query.fcgi?db=gene&cmd=Retrieve&dopt=full_report&list_uids=10138) | YY1 associated factor 2 |
| [Details](http://mirdb.org/cgi-bin/target_detail.cgi?targetID=1439971) | 411 | 90 | hsa-miR-30c-5p | [PPP1R12A](http://www.ncbi.nlm.nih.gov/entrez/query.fcgi?db=gene&cmd=Retrieve&dopt=full_report&list_uids=4659) | protein phosphatase 1 regulatory subunit 12A |
| [Details](http://mirdb.org/cgi-bin/target_detail.cgi?targetID=1439977) | 412 | 90 | hsa-miR-30c-5p | [EXOC6](http://www.ncbi.nlm.nih.gov/entrez/query.fcgi?db=gene&cmd=Retrieve&dopt=full_report&list_uids=54536) | exocyst complex component 6 |
| [Details](http://mirdb.org/cgi-bin/target_detail.cgi?targetID=1439986) | 413 | 90 | hsa-miR-30c-5p | [GJA1](http://www.ncbi.nlm.nih.gov/entrez/query.fcgi?db=gene&cmd=Retrieve&dopt=full_report&list_uids=2697) | gap junction protein alpha 1 |
| [Details](http://mirdb.org/cgi-bin/target_detail.cgi?targetID=1440092) | 414 | 90 | hsa-miR-30c-5p | [KLF9](http://www.ncbi.nlm.nih.gov/entrez/query.fcgi?db=gene&cmd=Retrieve&dopt=full_report&list_uids=687) | Kruppel like factor 9 |
| [Details](http://mirdb.org/cgi-bin/target_detail.cgi?targetID=1440112) | 415 | 90 | hsa-miR-30c-5p | [GNAI2](http://www.ncbi.nlm.nih.gov/entrez/query.fcgi?db=gene&cmd=Retrieve&dopt=full_report&list_uids=2771) | G protein subunit alpha i2 |
| [Details](http://mirdb.org/cgi-bin/target_detail.cgi?targetID=1440152) | 416 | 90 | hsa-miR-30c-5p | [STK17B](http://www.ncbi.nlm.nih.gov/entrez/query.fcgi?db=gene&cmd=Retrieve&dopt=full_report&list_uids=9262) | serine/threonine kinase 17b |
| [Details](http://mirdb.org/cgi-bin/target_detail.cgi?targetID=1440176) | 417 | 90 | hsa-miR-30c-5p | [OVOL1](http://www.ncbi.nlm.nih.gov/entrez/query.fcgi?db=gene&cmd=Retrieve&dopt=full_report&list_uids=5017) | ovo like transcriptional repressor 1 |
| [Details](http://mirdb.org/cgi-bin/target_detail.cgi?targetID=1440197) | 418 | 90 | hsa-miR-30c-5p | [GRM5](http://www.ncbi.nlm.nih.gov/entrez/query.fcgi?db=gene&cmd=Retrieve&dopt=full_report&list_uids=2915) | glutamate metabotropic receptor 5 |
| [Details](http://mirdb.org/cgi-bin/target_detail.cgi?targetID=1440201) | 419 | 90 | hsa-miR-30c-5p | [C10orf25](http://www.ncbi.nlm.nih.gov/entrez/query.fcgi?db=gene&cmd=Retrieve&dopt=full_report&list_uids=220979) | chromosome 10 open reading frame 25 |
| [Details](http://mirdb.org/cgi-bin/target_detail.cgi?targetID=1440276) | 420 | 90 | hsa-miR-30c-5p | [TENM1](http://www.ncbi.nlm.nih.gov/entrez/query.fcgi?db=gene&cmd=Retrieve&dopt=full_report&list_uids=10178) | teneurin transmembrane protein 1 |
| [Details](http://mirdb.org/cgi-bin/target_detail.cgi?targetID=1440381) | 421 | 90 | hsa-miR-30c-5p | [MFHAS1](http://www.ncbi.nlm.nih.gov/entrez/query.fcgi?db=gene&cmd=Retrieve&dopt=full_report&list_uids=9258) | malignant fibrous histiocytoma amplified sequence 1 |
| [Details](http://mirdb.org/cgi-bin/target_detail.cgi?targetID=1440421) | 422 | 90 | hsa-miR-30c-5p | [TM4SF1](http://www.ncbi.nlm.nih.gov/entrez/query.fcgi?db=gene&cmd=Retrieve&dopt=full_report&list_uids=4071) | transmembrane 4 L six family member 1 |
| [Details](http://mirdb.org/cgi-bin/target_detail.cgi?targetID=1440498) | 423 | 90 | hsa-miR-30c-5p | [NR6A1](http://www.ncbi.nlm.nih.gov/entrez/query.fcgi?db=gene&cmd=Retrieve&dopt=full_report&list_uids=2649) | nuclear receptor subfamily 6 group A member 1 |
| [Details](http://mirdb.org/cgi-bin/target_detail.cgi?targetID=1440515) | 424 | 90 | hsa-miR-30c-5p | [KDM3A](http://www.ncbi.nlm.nih.gov/entrez/query.fcgi?db=gene&cmd=Retrieve&dopt=full_report&list_uids=55818) | lysine demethylase 3A |
| [Details](http://mirdb.org/cgi-bin/target_detail.cgi?targetID=1440538) | 425 | 90 | hsa-miR-30c-5p | [STRIP1](http://www.ncbi.nlm.nih.gov/entrez/query.fcgi?db=gene&cmd=Retrieve&dopt=full_report&list_uids=85369) | striatin interacting protein 1 |
| [Details](http://mirdb.org/cgi-bin/target_detail.cgi?targetID=1440566) | 426 | 90 | hsa-miR-30c-5p | [EDEM3](http://www.ncbi.nlm.nih.gov/entrez/query.fcgi?db=gene&cmd=Retrieve&dopt=full_report&list_uids=80267) | ER degradation enhancing alpha-mannosidase like protein 3 |
| [Details](http://mirdb.org/cgi-bin/target_detail.cgi?targetID=1440576) | 427 | 90 | hsa-miR-30c-5p | [GFPT2](http://www.ncbi.nlm.nih.gov/entrez/query.fcgi?db=gene&cmd=Retrieve&dopt=full_report&list_uids=9945) | glutamine-fructose-6-phosphate transaminase 2 |
| [Details](http://mirdb.org/cgi-bin/target_detail.cgi?targetID=1440583) | 428 | 90 | hsa-miR-30c-5p | [LMLN](http://www.ncbi.nlm.nih.gov/entrez/query.fcgi?db=gene&cmd=Retrieve&dopt=full_report&list_uids=89782) | leishmanolysin like peptidase |
| [Details](http://mirdb.org/cgi-bin/target_detail.cgi?targetID=1440732) | 429 | 90 | hsa-miR-30c-5p | [SSH2](http://www.ncbi.nlm.nih.gov/entrez/query.fcgi?db=gene&cmd=Retrieve&dopt=full_report&list_uids=85464) | slingshot protein phosphatase 2 |
| [Details](http://mirdb.org/cgi-bin/target_detail.cgi?targetID=1440739) | 430 | 90 | hsa-miR-30c-5p | [HOXA1](http://www.ncbi.nlm.nih.gov/entrez/query.fcgi?db=gene&cmd=Retrieve&dopt=full_report&list_uids=3198) | homeobox A1 |
| [Details](http://mirdb.org/cgi-bin/target_detail.cgi?targetID=1440742) | 431 | 90 | hsa-miR-30c-5p | [NAA25](http://www.ncbi.nlm.nih.gov/entrez/query.fcgi?db=gene&cmd=Retrieve&dopt=full_report&list_uids=80018) | N(alpha)-acetyltransferase 25, NatB auxiliary subunit |
| [Details](http://mirdb.org/cgi-bin/target_detail.cgi?targetID=1440761) | 432 | 90 | hsa-miR-30c-5p | [FOXD1](http://www.ncbi.nlm.nih.gov/entrez/query.fcgi?db=gene&cmd=Retrieve&dopt=full_report&list_uids=2297) | forkhead box D1 |
| [Details](http://mirdb.org/cgi-bin/target_detail.cgi?targetID=1440763) | 433 | 90 | hsa-miR-30c-5p | [AGO3](http://www.ncbi.nlm.nih.gov/entrez/query.fcgi?db=gene&cmd=Retrieve&dopt=full_report&list_uids=192669) | argonaute RISC catalytic component 3 |
| [Details](http://mirdb.org/cgi-bin/target_detail.cgi?targetID=1440776) | 434 | 90 | hsa-miR-30c-5p | [KIAA2026](http://www.ncbi.nlm.nih.gov/entrez/query.fcgi?db=gene&cmd=Retrieve&dopt=full_report&list_uids=158358) | KIAA2026 |
| [Details](http://mirdb.org/cgi-bin/target_detail.cgi?targetID=1440780) | 435 | 90 | hsa-miR-30c-5p | [NAALADL2](http://www.ncbi.nlm.nih.gov/entrez/query.fcgi?db=gene&cmd=Retrieve&dopt=full_report&list_uids=254827) | N-acetylated alpha-linked acidic dipeptidase like 2 |
| [Details](http://mirdb.org/cgi-bin/target_detail.cgi?targetID=1440810) | 436 | 90 | hsa-miR-30c-5p | [SLC7A10](http://www.ncbi.nlm.nih.gov/entrez/query.fcgi?db=gene&cmd=Retrieve&dopt=full_report&list_uids=56301) | solute carrier family 7 member 10 |
| [Details](http://mirdb.org/cgi-bin/target_detail.cgi?targetID=1440826) | 437 | 90 | hsa-miR-30c-5p | [BCL9](http://www.ncbi.nlm.nih.gov/entrez/query.fcgi?db=gene&cmd=Retrieve&dopt=full_report&list_uids=607) | BCL9, transcription coactivator |
| [Details](http://mirdb.org/cgi-bin/target_detail.cgi?targetID=1440842) | 438 | 90 | hsa-miR-30c-5p | [IDH1](http://www.ncbi.nlm.nih.gov/entrez/query.fcgi?db=gene&cmd=Retrieve&dopt=full_report&list_uids=3417) | isocitrate dehydrogenase (NADP(+)) 1, cytosolic |
| [Details](http://mirdb.org/cgi-bin/target_detail.cgi?targetID=1440872) | 439 | 90 | hsa-miR-30c-5p | [CMTM4](http://www.ncbi.nlm.nih.gov/entrez/query.fcgi?db=gene&cmd=Retrieve&dopt=full_report&list_uids=146223) | CKLF like MARVEL transmembrane domain containing 4 |
| [Details](http://mirdb.org/cgi-bin/target_detail.cgi?targetID=1440916) | 440 | 90 | hsa-miR-30c-5p | [KMT2A](http://www.ncbi.nlm.nih.gov/entrez/query.fcgi?db=gene&cmd=Retrieve&dopt=full_report&list_uids=4297) | lysine methyltransferase 2A |
| [Details](http://mirdb.org/cgi-bin/target_detail.cgi?targetID=1440920) | 441 | 90 | hsa-miR-30c-5p | [RHEBL1](http://www.ncbi.nlm.nih.gov/entrez/query.fcgi?db=gene&cmd=Retrieve&dopt=full_report&list_uids=121268) | RHEB like 1 |
| [Details](http://mirdb.org/cgi-bin/target_detail.cgi?targetID=1439484) | 442 | 89 | hsa-miR-30c-5p | [CEP170](http://www.ncbi.nlm.nih.gov/entrez/query.fcgi?db=gene&cmd=Retrieve&dopt=full_report&list_uids=9859) | centrosomal protein 170 |
| [Details](http://mirdb.org/cgi-bin/target_detail.cgi?targetID=1439505) | 443 | 89 | hsa-miR-30c-5p | [MARK1](http://www.ncbi.nlm.nih.gov/entrez/query.fcgi?db=gene&cmd=Retrieve&dopt=full_report&list_uids=4139) | microtubule affinity regulating kinase 1 |
| [Details](http://mirdb.org/cgi-bin/target_detail.cgi?targetID=1439558) | 444 | 89 | hsa-miR-30c-5p | [KLF8](http://www.ncbi.nlm.nih.gov/entrez/query.fcgi?db=gene&cmd=Retrieve&dopt=full_report&list_uids=11279) | Kruppel like factor 8 |
| [Details](http://mirdb.org/cgi-bin/target_detail.cgi?targetID=1439596) | 445 | 89 | hsa-miR-30c-5p | [UBAC1](http://www.ncbi.nlm.nih.gov/entrez/query.fcgi?db=gene&cmd=Retrieve&dopt=full_report&list_uids=10422) | UBA domain containing 1 |
| [Details](http://mirdb.org/cgi-bin/target_detail.cgi?targetID=1439618) | 446 | 89 | hsa-miR-30c-5p | [PIP4K2B](http://www.ncbi.nlm.nih.gov/entrez/query.fcgi?db=gene&cmd=Retrieve&dopt=full_report&list_uids=8396) | phosphatidylinositol-5-phosphate 4-kinase type 2 beta |
| [Details](http://mirdb.org/cgi-bin/target_detail.cgi?targetID=1439670) | 447 | 89 | hsa-miR-30c-5p | [TDG](http://www.ncbi.nlm.nih.gov/entrez/query.fcgi?db=gene&cmd=Retrieve&dopt=full_report&list_uids=6996) | thymine DNA glycosylase |
| [Details](http://mirdb.org/cgi-bin/target_detail.cgi?targetID=1439740) | 448 | 89 | hsa-miR-30c-5p | [ACVR1](http://www.ncbi.nlm.nih.gov/entrez/query.fcgi?db=gene&cmd=Retrieve&dopt=full_report&list_uids=90) | activin A receptor type 1 |
| [Details](http://mirdb.org/cgi-bin/target_detail.cgi?targetID=1439833) | 449 | 89 | hsa-miR-30c-5p | [CDH20](http://www.ncbi.nlm.nih.gov/entrez/query.fcgi?db=gene&cmd=Retrieve&dopt=full_report&list_uids=28316) | cadherin 20 |
| [Details](http://mirdb.org/cgi-bin/target_detail.cgi?targetID=1439843) | 450 | 89 | hsa-miR-30c-5p | [CNST](http://www.ncbi.nlm.nih.gov/entrez/query.fcgi?db=gene&cmd=Retrieve&dopt=full_report&list_uids=163882) | consortin, connexin sorting protein |
| [Details](http://mirdb.org/cgi-bin/target_detail.cgi?targetID=1439903) | 451 | 89 | hsa-miR-30c-5p | [NHLH2](http://www.ncbi.nlm.nih.gov/entrez/query.fcgi?db=gene&cmd=Retrieve&dopt=full_report&list_uids=4808) | nescient helix-loop-helix 2 |
| [Details](http://mirdb.org/cgi-bin/target_detail.cgi?targetID=1439912) | 452 | 89 | hsa-miR-30c-5p | [BCL10](http://www.ncbi.nlm.nih.gov/entrez/query.fcgi?db=gene&cmd=Retrieve&dopt=full_report&list_uids=8915) | BCL10, immune signaling adaptor |
| [Details](http://mirdb.org/cgi-bin/target_detail.cgi?targetID=1439927) | 453 | 89 | hsa-miR-30c-5p | [DLGAP4](http://www.ncbi.nlm.nih.gov/entrez/query.fcgi?db=gene&cmd=Retrieve&dopt=full_report&list_uids=22839) | DLG associated protein 4 |
| [Details](http://mirdb.org/cgi-bin/target_detail.cgi?targetID=1439933) | 454 | 89 | hsa-miR-30c-5p | [OGA](http://www.ncbi.nlm.nih.gov/entrez/query.fcgi?db=gene&cmd=Retrieve&dopt=full_report&list_uids=10724) | O-GlcNAcase |
| [Details](http://mirdb.org/cgi-bin/target_detail.cgi?targetID=1439937) | 455 | 89 | hsa-miR-30c-5p | [ARID1A](http://www.ncbi.nlm.nih.gov/entrez/query.fcgi?db=gene&cmd=Retrieve&dopt=full_report&list_uids=8289) | AT-rich interaction domain 1A |
| [Details](http://mirdb.org/cgi-bin/target_detail.cgi?targetID=1440019) | 456 | 89 | hsa-miR-30c-5p | [CARS](http://www.ncbi.nlm.nih.gov/entrez/query.fcgi?db=gene&cmd=Retrieve&dopt=full_report&list_uids=833) | cysteinyl-tRNA synthetase |
| [Details](http://mirdb.org/cgi-bin/target_detail.cgi?targetID=1440023) | 457 | 89 | hsa-miR-30c-5p | [CACNB2](http://www.ncbi.nlm.nih.gov/entrez/query.fcgi?db=gene&cmd=Retrieve&dopt=full_report&list_uids=783) | calcium voltage-gated channel auxiliary subunit beta 2 |
| [Details](http://mirdb.org/cgi-bin/target_detail.cgi?targetID=1440040) | 458 | 89 | hsa-miR-30c-5p | [SYNGR3](http://www.ncbi.nlm.nih.gov/entrez/query.fcgi?db=gene&cmd=Retrieve&dopt=full_report&list_uids=9143) | synaptogyrin 3 |
| [Details](http://mirdb.org/cgi-bin/target_detail.cgi?targetID=1440073) | 459 | 89 | hsa-miR-30c-5p | [ABCC9](http://www.ncbi.nlm.nih.gov/entrez/query.fcgi?db=gene&cmd=Retrieve&dopt=full_report&list_uids=10060) | ATP binding cassette subfamily C member 9 |
| [Details](http://mirdb.org/cgi-bin/target_detail.cgi?targetID=1440086) | 460 | 89 | hsa-miR-30c-5p | [ZBTB6](http://www.ncbi.nlm.nih.gov/entrez/query.fcgi?db=gene&cmd=Retrieve&dopt=full_report&list_uids=10773) | zinc finger and BTB domain containing 6 |
| [Details](http://mirdb.org/cgi-bin/target_detail.cgi?targetID=1440093) | 461 | 89 | hsa-miR-30c-5p | [DLGAP1](http://www.ncbi.nlm.nih.gov/entrez/query.fcgi?db=gene&cmd=Retrieve&dopt=full_report&list_uids=9229) | DLG associated protein 1 |
| [Details](http://mirdb.org/cgi-bin/target_detail.cgi?targetID=1440191) | 462 | 89 | hsa-miR-30c-5p | [RAB4B](http://www.ncbi.nlm.nih.gov/entrez/query.fcgi?db=gene&cmd=Retrieve&dopt=full_report&list_uids=53916) | RAB4B, member RAS oncogene family |
| [Details](http://mirdb.org/cgi-bin/target_detail.cgi?targetID=1440272) | 463 | 89 | hsa-miR-30c-5p | [SNAI2](http://www.ncbi.nlm.nih.gov/entrez/query.fcgi?db=gene&cmd=Retrieve&dopt=full_report&list_uids=6591) | snail family transcriptional repressor 2 |
| [Details](http://mirdb.org/cgi-bin/target_detail.cgi?targetID=1440355) | 464 | 89 | hsa-miR-30c-5p | [KSR1](http://www.ncbi.nlm.nih.gov/entrez/query.fcgi?db=gene&cmd=Retrieve&dopt=full_report&list_uids=8844) | kinase suppressor of ras 1 |
| [Details](http://mirdb.org/cgi-bin/target_detail.cgi?targetID=1440449) | 465 | 89 | hsa-miR-30c-5p | [DDX59](http://www.ncbi.nlm.nih.gov/entrez/query.fcgi?db=gene&cmd=Retrieve&dopt=full_report&list_uids=83479) | DEAD-box helicase 59 |
| [Details](http://mirdb.org/cgi-bin/target_detail.cgi?targetID=1440464) | 466 | 89 | hsa-miR-30c-5p | [MAML1](http://www.ncbi.nlm.nih.gov/entrez/query.fcgi?db=gene&cmd=Retrieve&dopt=full_report&list_uids=9794) | mastermind like transcriptional coactivator 1 |
| [Details](http://mirdb.org/cgi-bin/target_detail.cgi?targetID=1440549) | 467 | 89 | hsa-miR-30c-5p | [BCOR](http://www.ncbi.nlm.nih.gov/entrez/query.fcgi?db=gene&cmd=Retrieve&dopt=full_report&list_uids=54880) | BCL6 corepressor |
| [Details](http://mirdb.org/cgi-bin/target_detail.cgi?targetID=1440560) | 468 | 89 | hsa-miR-30c-5p | [DDIT4](http://www.ncbi.nlm.nih.gov/entrez/query.fcgi?db=gene&cmd=Retrieve&dopt=full_report&list_uids=54541) | DNA damage inducible transcript 4 |
| [Details](http://mirdb.org/cgi-bin/target_detail.cgi?targetID=1440658) | 469 | 89 | hsa-miR-30c-5p | [ADGRA3](http://www.ncbi.nlm.nih.gov/entrez/query.fcgi?db=gene&cmd=Retrieve&dopt=full_report&list_uids=166647) | adhesion G protein-coupled receptor A3 |
| [Details](http://mirdb.org/cgi-bin/target_detail.cgi?targetID=1440686) | 470 | 89 | hsa-miR-30c-5p | [ESCO1](http://www.ncbi.nlm.nih.gov/entrez/query.fcgi?db=gene&cmd=Retrieve&dopt=full_report&list_uids=114799) | establishment of sister chromatid cohesion N-acetyltransferase 1 |
| [Details](http://mirdb.org/cgi-bin/target_detail.cgi?targetID=1440898) | 471 | 89 | hsa-miR-30c-5p | [RHD](http://www.ncbi.nlm.nih.gov/entrez/query.fcgi?db=gene&cmd=Retrieve&dopt=full_report&list_uids=6007) | Rh blood group D antigen |
| [Details](http://mirdb.org/cgi-bin/target_detail.cgi?targetID=1440919) | 472 | 89 | hsa-miR-30c-5p | [ATG5](http://www.ncbi.nlm.nih.gov/entrez/query.fcgi?db=gene&cmd=Retrieve&dopt=full_report&list_uids=9474) | autophagy related 5 |
| [Details](http://mirdb.org/cgi-bin/target_detail.cgi?targetID=1440976) | 473 | 89 | hsa-miR-30c-5p | [SLC38A2](http://www.ncbi.nlm.nih.gov/entrez/query.fcgi?db=gene&cmd=Retrieve&dopt=full_report&list_uids=54407) | solute carrier family 38 member 2 |
| [Details](http://mirdb.org/cgi-bin/target_detail.cgi?targetID=1440996) | 474 | 89 | hsa-miR-30c-5p | [MARCH4](http://www.ncbi.nlm.nih.gov/entrez/query.fcgi?db=gene&cmd=Retrieve&dopt=full_report&list_uids=57574) | membrane associated ring-CH-type finger 4 |
| [Details](http://mirdb.org/cgi-bin/target_detail.cgi?targetID=1439479) | 475 | 88 | hsa-miR-30c-5p | [MAT2A](http://www.ncbi.nlm.nih.gov/entrez/query.fcgi?db=gene&cmd=Retrieve&dopt=full_report&list_uids=4144) | methionine adenosyltransferase 2A |
| [Details](http://mirdb.org/cgi-bin/target_detail.cgi?targetID=1439492) | 476 | 88 | hsa-miR-30c-5p | [MED12L](http://www.ncbi.nlm.nih.gov/entrez/query.fcgi?db=gene&cmd=Retrieve&dopt=full_report&list_uids=116931) | mediator complex subunit 12 like |
| [Details](http://mirdb.org/cgi-bin/target_detail.cgi?targetID=1439522) | 477 | 88 | hsa-miR-30c-5p | [GALNT2](http://www.ncbi.nlm.nih.gov/entrez/query.fcgi?db=gene&cmd=Retrieve&dopt=full_report&list_uids=2590) | polypeptide N-acetylgalactosaminyltransferase 2 |
| [Details](http://mirdb.org/cgi-bin/target_detail.cgi?targetID=1439532) | 478 | 88 | hsa-miR-30c-5p | [PNPLA1](http://www.ncbi.nlm.nih.gov/entrez/query.fcgi?db=gene&cmd=Retrieve&dopt=full_report&list_uids=285848) | patatin like phospholipase domain containing 1 |
| [Details](http://mirdb.org/cgi-bin/target_detail.cgi?targetID=1439599) | 479 | 88 | hsa-miR-30c-5p | [ZNF644](http://www.ncbi.nlm.nih.gov/entrez/query.fcgi?db=gene&cmd=Retrieve&dopt=full_report&list_uids=84146) | zinc finger protein 644 |
| [Details](http://mirdb.org/cgi-bin/target_detail.cgi?targetID=1439629) | 480 | 88 | hsa-miR-30c-5p | [ATXN1](http://www.ncbi.nlm.nih.gov/entrez/query.fcgi?db=gene&cmd=Retrieve&dopt=full_report&list_uids=6310) | ataxin 1 |
| [Details](http://mirdb.org/cgi-bin/target_detail.cgi?targetID=1439675) | 481 | 88 | hsa-miR-30c-5p | [RAB27B](http://www.ncbi.nlm.nih.gov/entrez/query.fcgi?db=gene&cmd=Retrieve&dopt=full_report&list_uids=5874) | RAB27B, member RAS oncogene family |
| [Details](http://mirdb.org/cgi-bin/target_detail.cgi?targetID=1439766) | 482 | 88 | hsa-miR-30c-5p | [CSNK1G1](http://www.ncbi.nlm.nih.gov/entrez/query.fcgi?db=gene&cmd=Retrieve&dopt=full_report&list_uids=53944) | casein kinase 1 gamma 1 |
| [Details](http://mirdb.org/cgi-bin/target_detail.cgi?targetID=1439878) | 483 | 88 | hsa-miR-30c-5p | [JAKMIP2](http://www.ncbi.nlm.nih.gov/entrez/query.fcgi?db=gene&cmd=Retrieve&dopt=full_report&list_uids=9832) | janus kinase and microtubule interacting protein 2 |
| [Details](http://mirdb.org/cgi-bin/target_detail.cgi?targetID=1439899) | 484 | 88 | hsa-miR-30c-5p | [DSTYK](http://www.ncbi.nlm.nih.gov/entrez/query.fcgi?db=gene&cmd=Retrieve&dopt=full_report&list_uids=25778) | dual serine/threonine and tyrosine protein kinase |
| [Details](http://mirdb.org/cgi-bin/target_detail.cgi?targetID=1439919) | 485 | 88 | hsa-miR-30c-5p | [SRSF10](http://www.ncbi.nlm.nih.gov/entrez/query.fcgi?db=gene&cmd=Retrieve&dopt=full_report&list_uids=10772) | serine and arginine rich splicing factor 10 |
| [Details](http://mirdb.org/cgi-bin/target_detail.cgi?targetID=1439981) | 486 | 88 | hsa-miR-30c-5p | [TNXB](http://www.ncbi.nlm.nih.gov/entrez/query.fcgi?db=gene&cmd=Retrieve&dopt=full_report&list_uids=7148) | tenascin XB |
| [Details](http://mirdb.org/cgi-bin/target_detail.cgi?targetID=1440029) | 487 | 88 | hsa-miR-30c-5p | [TASP1](http://www.ncbi.nlm.nih.gov/entrez/query.fcgi?db=gene&cmd=Retrieve&dopt=full_report&list_uids=55617) | taspase 1 |
| [Details](http://mirdb.org/cgi-bin/target_detail.cgi?targetID=1440050) | 488 | 88 | hsa-miR-30c-5p | [TTC8](http://www.ncbi.nlm.nih.gov/entrez/query.fcgi?db=gene&cmd=Retrieve&dopt=full_report&list_uids=123016) | tetratricopeptide repeat domain 8 |
| [Details](http://mirdb.org/cgi-bin/target_detail.cgi?targetID=1440071) | 489 | 88 | hsa-miR-30c-5p | [PPP3CB](http://www.ncbi.nlm.nih.gov/entrez/query.fcgi?db=gene&cmd=Retrieve&dopt=full_report&list_uids=5532) | protein phosphatase 3 catalytic subunit beta |
| [Details](http://mirdb.org/cgi-bin/target_detail.cgi?targetID=1440077) | 490 | 88 | hsa-miR-30c-5p | [GOLGA4](http://www.ncbi.nlm.nih.gov/entrez/query.fcgi?db=gene&cmd=Retrieve&dopt=full_report&list_uids=2803) | golgin A4 |
| [Details](http://mirdb.org/cgi-bin/target_detail.cgi?targetID=1440090) | 491 | 88 | hsa-miR-30c-5p | [IRF4](http://www.ncbi.nlm.nih.gov/entrez/query.fcgi?db=gene&cmd=Retrieve&dopt=full_report&list_uids=3662) | interferon regulatory factor 4 |
| [Details](http://mirdb.org/cgi-bin/target_detail.cgi?targetID=1440105) | 492 | 88 | hsa-miR-30c-5p | [ZXDA](http://www.ncbi.nlm.nih.gov/entrez/query.fcgi?db=gene&cmd=Retrieve&dopt=full_report&list_uids=7789) | zinc finger X-linked duplicated A |
| [Details](http://mirdb.org/cgi-bin/target_detail.cgi?targetID=1440114) | 493 | 88 | hsa-miR-30c-5p | [SEPT8](http://www.ncbi.nlm.nih.gov/entrez/query.fcgi?db=gene&cmd=Retrieve&dopt=full_report&list_uids=23176) | septin 8 |
| [Details](http://mirdb.org/cgi-bin/target_detail.cgi?targetID=1440116) | 494 | 88 | hsa-miR-30c-5p | [NR4A2](http://www.ncbi.nlm.nih.gov/entrez/query.fcgi?db=gene&cmd=Retrieve&dopt=full_report&list_uids=4929) | nuclear receptor subfamily 4 group A member 2 |
| [Details](http://mirdb.org/cgi-bin/target_detail.cgi?targetID=1440120) | 495 | 88 | hsa-miR-30c-5p | [IRX4](http://www.ncbi.nlm.nih.gov/entrez/query.fcgi?db=gene&cmd=Retrieve&dopt=full_report&list_uids=50805) | iroquois homeobox 4 |
| [Details](http://mirdb.org/cgi-bin/target_detail.cgi?targetID=1440131) | 496 | 88 | hsa-miR-30c-5p | [CYB561](http://www.ncbi.nlm.nih.gov/entrez/query.fcgi?db=gene&cmd=Retrieve&dopt=full_report&list_uids=1534) | cytochrome b561 |
| [Details](http://mirdb.org/cgi-bin/target_detail.cgi?targetID=1440147) | 497 | 88 | hsa-miR-30c-5p | [USP48](http://www.ncbi.nlm.nih.gov/entrez/query.fcgi?db=gene&cmd=Retrieve&dopt=full_report&list_uids=84196) | ubiquitin specific peptidase 48 |
| [Details](http://mirdb.org/cgi-bin/target_detail.cgi?targetID=1440248) | 498 | 88 | hsa-miR-30c-5p | [GLI2](http://www.ncbi.nlm.nih.gov/entrez/query.fcgi?db=gene&cmd=Retrieve&dopt=full_report&list_uids=2736) | GLI family zinc finger 2 |
| [Details](http://mirdb.org/cgi-bin/target_detail.cgi?targetID=1440273) | 499 | 88 | hsa-miR-30c-5p | [IDE](http://www.ncbi.nlm.nih.gov/entrez/query.fcgi?db=gene&cmd=Retrieve&dopt=full_report&list_uids=3416) | insulin degrading enzyme |
| [Details](http://mirdb.org/cgi-bin/target_detail.cgi?targetID=1440285) | 500 | 88 | hsa-miR-30c-5p | [DOCK7](http://www.ncbi.nlm.nih.gov/entrez/query.fcgi?db=gene&cmd=Retrieve&dopt=full_report&list_uids=85440) | dedicator of cytokinesis 7 |
| [Details](http://mirdb.org/cgi-bin/target_detail.cgi?targetID=1440290) | 501 | 88 | hsa-miR-30c-5p | [UBE2I](http://www.ncbi.nlm.nih.gov/entrez/query.fcgi?db=gene&cmd=Retrieve&dopt=full_report&list_uids=7329) | ubiquitin conjugating enzyme E2 I |
| [Details](http://mirdb.org/cgi-bin/target_detail.cgi?targetID=1440328) | 502 | 88 | hsa-miR-30c-5p | [GIGYF2](http://www.ncbi.nlm.nih.gov/entrez/query.fcgi?db=gene&cmd=Retrieve&dopt=full_report&list_uids=26058) | GRB10 interacting GYF protein 2 |
| [Details](http://mirdb.org/cgi-bin/target_detail.cgi?targetID=1440417) | 503 | 88 | hsa-miR-30c-5p | [ATP2A2](http://www.ncbi.nlm.nih.gov/entrez/query.fcgi?db=gene&cmd=Retrieve&dopt=full_report&list_uids=488) | ATPase sarcoplasmic/endoplasmic reticulum Ca2+ transporting 2 |
| [Details](http://mirdb.org/cgi-bin/target_detail.cgi?targetID=1440429) | 504 | 88 | hsa-miR-30c-5p | [TNRC6C](http://www.ncbi.nlm.nih.gov/entrez/query.fcgi?db=gene&cmd=Retrieve&dopt=full_report&list_uids=57690) | trinucleotide repeat containing 6C |
| [Details](http://mirdb.org/cgi-bin/target_detail.cgi?targetID=1440439) | 505 | 88 | hsa-miR-30c-5p | [SNAI1](http://www.ncbi.nlm.nih.gov/entrez/query.fcgi?db=gene&cmd=Retrieve&dopt=full_report&list_uids=6615) | snail family transcriptional repressor 1 |
| [Details](http://mirdb.org/cgi-bin/target_detail.cgi?targetID=1440440) | 506 | 88 | hsa-miR-30c-5p | [EIF5A2](http://www.ncbi.nlm.nih.gov/entrez/query.fcgi?db=gene&cmd=Retrieve&dopt=full_report&list_uids=56648) | eukaryotic translation initiation factor 5A2 |
| [Details](http://mirdb.org/cgi-bin/target_detail.cgi?targetID=1440441) | 507 | 88 | hsa-miR-30c-5p | [CCDC6](http://www.ncbi.nlm.nih.gov/entrez/query.fcgi?db=gene&cmd=Retrieve&dopt=full_report&list_uids=8030) | coiled-coil domain containing 6 |
| [Details](http://mirdb.org/cgi-bin/target_detail.cgi?targetID=1440450) | 508 | 88 | hsa-miR-30c-5p | [PCGF5](http://www.ncbi.nlm.nih.gov/entrez/query.fcgi?db=gene&cmd=Retrieve&dopt=full_report&list_uids=84333) | polycomb group ring finger 5 |
| [Details](http://mirdb.org/cgi-bin/target_detail.cgi?targetID=1440477) | 509 | 88 | hsa-miR-30c-5p | [NFATC2](http://www.ncbi.nlm.nih.gov/entrez/query.fcgi?db=gene&cmd=Retrieve&dopt=full_report&list_uids=4773) | nuclear factor of activated T cells 2 |
| [Details](http://mirdb.org/cgi-bin/target_detail.cgi?targetID=1440488) | 510 | 88 | hsa-miR-30c-5p | [WDR64](http://www.ncbi.nlm.nih.gov/entrez/query.fcgi?db=gene&cmd=Retrieve&dopt=full_report&list_uids=128025) | WD repeat domain 64 |
| [Details](http://mirdb.org/cgi-bin/target_detail.cgi?targetID=1440547) | 511 | 88 | hsa-miR-30c-5p | [NF1](http://www.ncbi.nlm.nih.gov/entrez/query.fcgi?db=gene&cmd=Retrieve&dopt=full_report&list_uids=4763) | neurofibromin 1 |
| [Details](http://mirdb.org/cgi-bin/target_detail.cgi?targetID=1440557) | 512 | 88 | hsa-miR-30c-5p | [JDP2](http://www.ncbi.nlm.nih.gov/entrez/query.fcgi?db=gene&cmd=Retrieve&dopt=full_report&list_uids=122953) | Jun dimerization protein 2 |
| [Details](http://mirdb.org/cgi-bin/target_detail.cgi?targetID=1440648) | 513 | 88 | hsa-miR-30c-5p | [SLC25A21](http://www.ncbi.nlm.nih.gov/entrez/query.fcgi?db=gene&cmd=Retrieve&dopt=full_report&list_uids=89874) | solute carrier family 25 member 21 |
| [Details](http://mirdb.org/cgi-bin/target_detail.cgi?targetID=1440707) | 514 | 88 | hsa-miR-30c-5p | [CADPS](http://www.ncbi.nlm.nih.gov/entrez/query.fcgi?db=gene&cmd=Retrieve&dopt=full_report&list_uids=8618) | calcium dependent secretion activator |
| [Details](http://mirdb.org/cgi-bin/target_detail.cgi?targetID=1440737) | 515 | 88 | hsa-miR-30c-5p | [LIN28A](http://www.ncbi.nlm.nih.gov/entrez/query.fcgi?db=gene&cmd=Retrieve&dopt=full_report&list_uids=79727) | lin-28 homolog A |
| [Details](http://mirdb.org/cgi-bin/target_detail.cgi?targetID=1440774) | 516 | 88 | hsa-miR-30c-5p | [PROSER1](http://www.ncbi.nlm.nih.gov/entrez/query.fcgi?db=gene&cmd=Retrieve&dopt=full_report&list_uids=80209) | proline and serine rich 1 |
| [Details](http://mirdb.org/cgi-bin/target_detail.cgi?targetID=1440778) | 517 | 88 | hsa-miR-30c-5p | [ELOVL5](http://www.ncbi.nlm.nih.gov/entrez/query.fcgi?db=gene&cmd=Retrieve&dopt=full_report&list_uids=60481) | ELOVL fatty acid elongase 5 |
| [Details](http://mirdb.org/cgi-bin/target_detail.cgi?targetID=1440943) | 518 | 88 | hsa-miR-30c-5p | [DPYSL2](http://www.ncbi.nlm.nih.gov/entrez/query.fcgi?db=gene&cmd=Retrieve&dopt=full_report&list_uids=1808) | dihydropyrimidinase like 2 |
| [Details](http://mirdb.org/cgi-bin/target_detail.cgi?targetID=1440945) | 519 | 88 | hsa-miR-30c-5p | [JOSD1](http://www.ncbi.nlm.nih.gov/entrez/query.fcgi?db=gene&cmd=Retrieve&dopt=full_report&list_uids=9929) | Josephin domain containing 1 |
| [Details](http://mirdb.org/cgi-bin/target_detail.cgi?targetID=1440992) | 520 | 88 | hsa-miR-30c-5p | [NLGN1](http://www.ncbi.nlm.nih.gov/entrez/query.fcgi?db=gene&cmd=Retrieve&dopt=full_report&list_uids=22871) | neuroligin 1 |
| [Details](http://mirdb.org/cgi-bin/target_detail.cgi?targetID=1439477) | 521 | 87 | hsa-miR-30c-5p | [SNX8](http://www.ncbi.nlm.nih.gov/entrez/query.fcgi?db=gene&cmd=Retrieve&dopt=full_report&list_uids=29886) | sorting nexin 8 |
| [Details](http://mirdb.org/cgi-bin/target_detail.cgi?targetID=1439481) | 522 | 87 | hsa-miR-30c-5p | [SLC35F3](http://www.ncbi.nlm.nih.gov/entrez/query.fcgi?db=gene&cmd=Retrieve&dopt=full_report&list_uids=148641) | solute carrier family 35 member F3 |
| [Details](http://mirdb.org/cgi-bin/target_detail.cgi?targetID=1439520) | 523 | 87 | hsa-miR-30c-5p | [EFNA3](http://www.ncbi.nlm.nih.gov/entrez/query.fcgi?db=gene&cmd=Retrieve&dopt=full_report&list_uids=1944) | ephrin A3 |
| [Details](http://mirdb.org/cgi-bin/target_detail.cgi?targetID=1439533) | 524 | 87 | hsa-miR-30c-5p | [CHMP2B](http://www.ncbi.nlm.nih.gov/entrez/query.fcgi?db=gene&cmd=Retrieve&dopt=full_report&list_uids=25978) | charged multivesicular body protein 2B |
| [Details](http://mirdb.org/cgi-bin/target_detail.cgi?targetID=1439539) | 525 | 87 | hsa-miR-30c-5p | [PGM3](http://www.ncbi.nlm.nih.gov/entrez/query.fcgi?db=gene&cmd=Retrieve&dopt=full_report&list_uids=5238) | phosphoglucomutase 3 |
| [Details](http://mirdb.org/cgi-bin/target_detail.cgi?targetID=1439542) | 526 | 87 | hsa-miR-30c-5p | [RPRD1A](http://www.ncbi.nlm.nih.gov/entrez/query.fcgi?db=gene&cmd=Retrieve&dopt=full_report&list_uids=55197) | regulation of nuclear pre-mRNA domain containing 1A |
| [Details](http://mirdb.org/cgi-bin/target_detail.cgi?targetID=1439610) | 527 | 87 | hsa-miR-30c-5p | [GPT2](http://www.ncbi.nlm.nih.gov/entrez/query.fcgi?db=gene&cmd=Retrieve&dopt=full_report&list_uids=84706) | glutamic--pyruvic transaminase 2 |
| [Details](http://mirdb.org/cgi-bin/target_detail.cgi?targetID=1439619) | 528 | 87 | hsa-miR-30c-5p | [KIAA0355](http://www.ncbi.nlm.nih.gov/entrez/query.fcgi?db=gene&cmd=Retrieve&dopt=full_report&list_uids=9710) | KIAA0355 |
| [Details](http://mirdb.org/cgi-bin/target_detail.cgi?targetID=1439656) | 529 | 87 | hsa-miR-30c-5p | [CAMK2N2](http://www.ncbi.nlm.nih.gov/entrez/query.fcgi?db=gene&cmd=Retrieve&dopt=full_report&list_uids=94032) | calcium/calmodulin dependent protein kinase II inhibitor 2 |
| [Details](http://mirdb.org/cgi-bin/target_detail.cgi?targetID=1439672) | 530 | 87 | hsa-miR-30c-5p | [RNF220](http://www.ncbi.nlm.nih.gov/entrez/query.fcgi?db=gene&cmd=Retrieve&dopt=full_report&list_uids=55182) | ring finger protein 220 |
| [Details](http://mirdb.org/cgi-bin/target_detail.cgi?targetID=1439694) | 531 | 87 | hsa-miR-30c-5p | [GAREM1](http://www.ncbi.nlm.nih.gov/entrez/query.fcgi?db=gene&cmd=Retrieve&dopt=full_report&list_uids=64762) | GRB2 associated regulator of MAPK1 subtype 1 |
| [Details](http://mirdb.org/cgi-bin/target_detail.cgi?targetID=1439770) | 532 | 87 | hsa-miR-30c-5p | [TENT5A](http://www.ncbi.nlm.nih.gov/entrez/query.fcgi?db=gene&cmd=Retrieve&dopt=full_report&list_uids=55603) | terminal nucleotidyltransferase 5A |
| [Details](http://mirdb.org/cgi-bin/target_detail.cgi?targetID=1439782) | 533 | 87 | hsa-miR-30c-5p | [GRIN2A](http://www.ncbi.nlm.nih.gov/entrez/query.fcgi?db=gene&cmd=Retrieve&dopt=full_report&list_uids=2903) | glutamate ionotropic receptor NMDA type subunit 2A |
| [Details](http://mirdb.org/cgi-bin/target_detail.cgi?targetID=1439846) | 534 | 87 | hsa-miR-30c-5p | [ARID5B](http://www.ncbi.nlm.nih.gov/entrez/query.fcgi?db=gene&cmd=Retrieve&dopt=full_report&list_uids=84159) | AT-rich interaction domain 5B |
| [Details](http://mirdb.org/cgi-bin/target_detail.cgi?targetID=1439860) | 535 | 87 | hsa-miR-30c-5p | [CDC37L1](http://www.ncbi.nlm.nih.gov/entrez/query.fcgi?db=gene&cmd=Retrieve&dopt=full_report&list_uids=55664) | cell division cycle 37 like 1 |
| [Details](http://mirdb.org/cgi-bin/target_detail.cgi?targetID=1439902) | 536 | 87 | hsa-miR-30c-5p | [FAM214A](http://www.ncbi.nlm.nih.gov/entrez/query.fcgi?db=gene&cmd=Retrieve&dopt=full_report&list_uids=56204) | family with sequence similarity 214 member A |
| [Details](http://mirdb.org/cgi-bin/target_detail.cgi?targetID=1439918) | 537 | 87 | hsa-miR-30c-5p | [ZBTB18](http://www.ncbi.nlm.nih.gov/entrez/query.fcgi?db=gene&cmd=Retrieve&dopt=full_report&list_uids=10472) | zinc finger and BTB domain containing 18 |
| [Details](http://mirdb.org/cgi-bin/target_detail.cgi?targetID=1439948) | 538 | 87 | hsa-miR-30c-5p | [PPID](http://www.ncbi.nlm.nih.gov/entrez/query.fcgi?db=gene&cmd=Retrieve&dopt=full_report&list_uids=5481) | peptidylprolyl isomerase D |
| [Details](http://mirdb.org/cgi-bin/target_detail.cgi?targetID=1439982) | 539 | 87 | hsa-miR-30c-5p | [REEP1](http://www.ncbi.nlm.nih.gov/entrez/query.fcgi?db=gene&cmd=Retrieve&dopt=full_report&list_uids=65055) | receptor accessory protein 1 |
| [Details](http://mirdb.org/cgi-bin/target_detail.cgi?targetID=1440055) | 540 | 87 | hsa-miR-30c-5p | [NEUROD1](http://www.ncbi.nlm.nih.gov/entrez/query.fcgi?db=gene&cmd=Retrieve&dopt=full_report&list_uids=4760) | neuronal differentiation 1 |
| [Details](http://mirdb.org/cgi-bin/target_detail.cgi?targetID=1440177) | 541 | 87 | hsa-miR-30c-5p | [LIFR](http://www.ncbi.nlm.nih.gov/entrez/query.fcgi?db=gene&cmd=Retrieve&dopt=full_report&list_uids=3977) | LIF receptor alpha |
| [Details](http://mirdb.org/cgi-bin/target_detail.cgi?targetID=1440233) | 542 | 87 | hsa-miR-30c-5p | [CTHRC1](http://www.ncbi.nlm.nih.gov/entrez/query.fcgi?db=gene&cmd=Retrieve&dopt=full_report&list_uids=115908) | collagen triple helix repeat containing 1 |
| [Details](http://mirdb.org/cgi-bin/target_detail.cgi?targetID=1440266) | 543 | 87 | hsa-miR-30c-5p | [LYN](http://www.ncbi.nlm.nih.gov/entrez/query.fcgi?db=gene&cmd=Retrieve&dopt=full_report&list_uids=4067) | LYN proto-oncogene, Src family tyrosine kinase |
| [Details](http://mirdb.org/cgi-bin/target_detail.cgi?targetID=1440309) | 544 | 87 | hsa-miR-30c-5p | [IFNAR2](http://www.ncbi.nlm.nih.gov/entrez/query.fcgi?db=gene&cmd=Retrieve&dopt=full_report&list_uids=3455) | interferon alpha and beta receptor subunit 2 |
| [Details](http://mirdb.org/cgi-bin/target_detail.cgi?targetID=1440346) | 545 | 87 | hsa-miR-30c-5p | [NUCKS1](http://www.ncbi.nlm.nih.gov/entrez/query.fcgi?db=gene&cmd=Retrieve&dopt=full_report&list_uids=64710) | nuclear casein kinase and cyclin dependent kinase substrate 1 |
| [Details](http://mirdb.org/cgi-bin/target_detail.cgi?targetID=1440484) | 546 | 87 | hsa-miR-30c-5p | [SMAP1](http://www.ncbi.nlm.nih.gov/entrez/query.fcgi?db=gene&cmd=Retrieve&dopt=full_report&list_uids=60682) | small ArfGAP 1 |
| [Details](http://mirdb.org/cgi-bin/target_detail.cgi?targetID=1440499) | 547 | 87 | hsa-miR-30c-5p | [SNTB2](http://www.ncbi.nlm.nih.gov/entrez/query.fcgi?db=gene&cmd=Retrieve&dopt=full_report&list_uids=6645) | syntrophin beta 2 |
| [Details](http://mirdb.org/cgi-bin/target_detail.cgi?targetID=1440505) | 548 | 87 | hsa-miR-30c-5p | [SLC9A8](http://www.ncbi.nlm.nih.gov/entrez/query.fcgi?db=gene&cmd=Retrieve&dopt=full_report&list_uids=23315) | solute carrier family 9 member A8 |
| [Details](http://mirdb.org/cgi-bin/target_detail.cgi?targetID=1440518) | 549 | 87 | hsa-miR-30c-5p | [PGP](http://www.ncbi.nlm.nih.gov/entrez/query.fcgi?db=gene&cmd=Retrieve&dopt=full_report&list_uids=283871) | phosphoglycolate phosphatase |
| [Details](http://mirdb.org/cgi-bin/target_detail.cgi?targetID=1440585) | 550 | 87 | hsa-miR-30c-5p | [ST8SIA4](http://www.ncbi.nlm.nih.gov/entrez/query.fcgi?db=gene&cmd=Retrieve&dopt=full_report&list_uids=7903) | ST8 alpha-N-acetyl-neuraminide alpha-2,8-sialyltransferase 4 |
| [Details](http://mirdb.org/cgi-bin/target_detail.cgi?targetID=1440615) | 551 | 87 | hsa-miR-30c-5p | [IRS1](http://www.ncbi.nlm.nih.gov/entrez/query.fcgi?db=gene&cmd=Retrieve&dopt=full_report&list_uids=3667) | insulin receptor substrate 1 |
| [Details](http://mirdb.org/cgi-bin/target_detail.cgi?targetID=1440627) | 552 | 87 | hsa-miR-30c-5p | [SLCO6A1](http://www.ncbi.nlm.nih.gov/entrez/query.fcgi?db=gene&cmd=Retrieve&dopt=full_report&list_uids=133482) | solute carrier organic anion transporter family member 6A1 |
| [Details](http://mirdb.org/cgi-bin/target_detail.cgi?targetID=1440651) | 553 | 87 | hsa-miR-30c-5p | [ITGB3](http://www.ncbi.nlm.nih.gov/entrez/query.fcgi?db=gene&cmd=Retrieve&dopt=full_report&list_uids=3690) | integrin subunit beta 3 |
| [Details](http://mirdb.org/cgi-bin/target_detail.cgi?targetID=1440661) | 554 | 87 | hsa-miR-30c-5p | [LCORL](http://www.ncbi.nlm.nih.gov/entrez/query.fcgi?db=gene&cmd=Retrieve&dopt=full_report&list_uids=254251) | ligand dependent nuclear receptor corepressor like |
| [Details](http://mirdb.org/cgi-bin/target_detail.cgi?targetID=1440664) | 555 | 87 | hsa-miR-30c-5p | [LRP6](http://www.ncbi.nlm.nih.gov/entrez/query.fcgi?db=gene&cmd=Retrieve&dopt=full_report&list_uids=4040) | LDL receptor related protein 6 |
| [Details](http://mirdb.org/cgi-bin/target_detail.cgi?targetID=1440790) | 556 | 87 | hsa-miR-30c-5p | [TULP4](http://www.ncbi.nlm.nih.gov/entrez/query.fcgi?db=gene&cmd=Retrieve&dopt=full_report&list_uids=56995) | tubby like protein 4 |
| [Details](http://mirdb.org/cgi-bin/target_detail.cgi?targetID=1440802) | 557 | 87 | hsa-miR-30c-5p | [ANXA2R](http://www.ncbi.nlm.nih.gov/entrez/query.fcgi?db=gene&cmd=Retrieve&dopt=full_report&list_uids=389289) | annexin A2 receptor |
| [Details](http://mirdb.org/cgi-bin/target_detail.cgi?targetID=1440843) | 558 | 87 | hsa-miR-30c-5p | [EPB41](http://www.ncbi.nlm.nih.gov/entrez/query.fcgi?db=gene&cmd=Retrieve&dopt=full_report&list_uids=2035) | erythrocyte membrane protein band 4.1 |
| [Details](http://mirdb.org/cgi-bin/target_detail.cgi?targetID=1440891) | 559 | 87 | hsa-miR-30c-5p | [USO1](http://www.ncbi.nlm.nih.gov/entrez/query.fcgi?db=gene&cmd=Retrieve&dopt=full_report&list_uids=8615) | USO1 vesicle transport factor |
| [Details](http://mirdb.org/cgi-bin/target_detail.cgi?targetID=1440893) | 560 | 87 | hsa-miR-30c-5p | [SAP30BP](http://www.ncbi.nlm.nih.gov/entrez/query.fcgi?db=gene&cmd=Retrieve&dopt=full_report&list_uids=29115) | SAP30 binding protein |
| [Details](http://mirdb.org/cgi-bin/target_detail.cgi?targetID=1440909) | 561 | 87 | hsa-miR-30c-5p | [FGD6](http://www.ncbi.nlm.nih.gov/entrez/query.fcgi?db=gene&cmd=Retrieve&dopt=full_report&list_uids=55785) | FYVE, RhoGEF and PH domain containing 6 |
| [Details](http://mirdb.org/cgi-bin/target_detail.cgi?targetID=1439504) | 562 | 86 | hsa-miR-30c-5p | [TBC1D15](http://www.ncbi.nlm.nih.gov/entrez/query.fcgi?db=gene&cmd=Retrieve&dopt=full_report&list_uids=64786) | TBC1 domain family member 15 |
| [Details](http://mirdb.org/cgi-bin/target_detail.cgi?targetID=1439550) | 563 | 86 | hsa-miR-30c-5p | [AVL9](http://www.ncbi.nlm.nih.gov/entrez/query.fcgi?db=gene&cmd=Retrieve&dopt=full_report&list_uids=23080) | AVL9 cell migration associated |
| [Details](http://mirdb.org/cgi-bin/target_detail.cgi?targetID=1439627) | 564 | 86 | hsa-miR-30c-5p | [PPP1R18](http://www.ncbi.nlm.nih.gov/entrez/query.fcgi?db=gene&cmd=Retrieve&dopt=full_report&list_uids=170954) | protein phosphatase 1 regulatory subunit 18 |
| [Details](http://mirdb.org/cgi-bin/target_detail.cgi?targetID=1439669) | 565 | 86 | hsa-miR-30c-5p | [LHX1](http://www.ncbi.nlm.nih.gov/entrez/query.fcgi?db=gene&cmd=Retrieve&dopt=full_report&list_uids=3975) | LIM homeobox 1 |
| [Details](http://mirdb.org/cgi-bin/target_detail.cgi?targetID=1439716) | 566 | 86 | hsa-miR-30c-5p | [CFAP97](http://www.ncbi.nlm.nih.gov/entrez/query.fcgi?db=gene&cmd=Retrieve&dopt=full_report&list_uids=57587) | cilia and flagella associated protein 97 |
| [Details](http://mirdb.org/cgi-bin/target_detail.cgi?targetID=1439719) | 567 | 86 | hsa-miR-30c-5p | [KPNA6](http://www.ncbi.nlm.nih.gov/entrez/query.fcgi?db=gene&cmd=Retrieve&dopt=full_report&list_uids=23633) | karyopherin subunit alpha 6 |
| [Details](http://mirdb.org/cgi-bin/target_detail.cgi?targetID=1439723) | 568 | 86 | hsa-miR-30c-5p | [DPY19L1](http://www.ncbi.nlm.nih.gov/entrez/query.fcgi?db=gene&cmd=Retrieve&dopt=full_report&list_uids=23333) | dpy-19 like C-mannosyltransferase 1 |
| [Details](http://mirdb.org/cgi-bin/target_detail.cgi?targetID=1439726) | 569 | 86 | hsa-miR-30c-5p | [DNAJC13](http://www.ncbi.nlm.nih.gov/entrez/query.fcgi?db=gene&cmd=Retrieve&dopt=full_report&list_uids=23317) | DnaJ heat shock protein family (Hsp40) member C13 |
| [Details](http://mirdb.org/cgi-bin/target_detail.cgi?targetID=1439742) | 570 | 86 | hsa-miR-30c-5p | [CSAD](http://www.ncbi.nlm.nih.gov/entrez/query.fcgi?db=gene&cmd=Retrieve&dopt=full_report&list_uids=51380) | cysteine sulfinic acid decarboxylase |
| [Details](http://mirdb.org/cgi-bin/target_detail.cgi?targetID=1439794) | 571 | 86 | hsa-miR-30c-5p | [CAPN5](http://www.ncbi.nlm.nih.gov/entrez/query.fcgi?db=gene&cmd=Retrieve&dopt=full_report&list_uids=726) | calpain 5 |
| [Details](http://mirdb.org/cgi-bin/target_detail.cgi?targetID=1440001) | 572 | 86 | hsa-miR-30c-5p | [CSGALNACT1](http://www.ncbi.nlm.nih.gov/entrez/query.fcgi?db=gene&cmd=Retrieve&dopt=full_report&list_uids=55790) | chondroitin sulfate N-acetylgalactosaminyltransferase 1 |
| [Details](http://mirdb.org/cgi-bin/target_detail.cgi?targetID=1440069) | 573 | 86 | hsa-miR-30c-5p | [DPY19L3](http://www.ncbi.nlm.nih.gov/entrez/query.fcgi?db=gene&cmd=Retrieve&dopt=full_report&list_uids=147991) | dpy-19 like C-mannosyltransferase 3 |
| [Details](http://mirdb.org/cgi-bin/target_detail.cgi?targetID=1440101) | 574 | 86 | hsa-miR-30c-5p | [INPP4A](http://www.ncbi.nlm.nih.gov/entrez/query.fcgi?db=gene&cmd=Retrieve&dopt=full_report&list_uids=3631) | inositol polyphosphate-4-phosphatase type I A |
| [Details](http://mirdb.org/cgi-bin/target_detail.cgi?targetID=1440140) | 575 | 86 | hsa-miR-30c-5p | [MIA3](http://www.ncbi.nlm.nih.gov/entrez/query.fcgi?db=gene&cmd=Retrieve&dopt=full_report&list_uids=375056) | MIA SH3 domain ER export factor 3 |
| [Details](http://mirdb.org/cgi-bin/target_detail.cgi?targetID=1440154) | 576 | 86 | hsa-miR-30c-5p | [KCNA4](http://www.ncbi.nlm.nih.gov/entrez/query.fcgi?db=gene&cmd=Retrieve&dopt=full_report&list_uids=3739) | potassium voltage-gated channel subfamily A member 4 |
| [Details](http://mirdb.org/cgi-bin/target_detail.cgi?targetID=1440226) | 577 | 86 | hsa-miR-30c-5p | [ATRN](http://www.ncbi.nlm.nih.gov/entrez/query.fcgi?db=gene&cmd=Retrieve&dopt=full_report&list_uids=8455) | attractin |
| [Details](http://mirdb.org/cgi-bin/target_detail.cgi?targetID=1440269) | 578 | 86 | hsa-miR-30c-5p | [PPP1R9A](http://www.ncbi.nlm.nih.gov/entrez/query.fcgi?db=gene&cmd=Retrieve&dopt=full_report&list_uids=55607) | protein phosphatase 1 regulatory subunit 9A |
| [Details](http://mirdb.org/cgi-bin/target_detail.cgi?targetID=1440278) | 579 | 86 | hsa-miR-30c-5p | [TAB3](http://www.ncbi.nlm.nih.gov/entrez/query.fcgi?db=gene&cmd=Retrieve&dopt=full_report&list_uids=257397) | TGF-beta activated kinase 1 (MAP3K7) binding protein 3 |
| [Details](http://mirdb.org/cgi-bin/target_detail.cgi?targetID=1440284) | 580 | 86 | hsa-miR-30c-5p | [PCMTD2](http://www.ncbi.nlm.nih.gov/entrez/query.fcgi?db=gene&cmd=Retrieve&dopt=full_report&list_uids=55251) | protein-L-isoaspartate (D-aspartate) O-methyltransferase domain containing 2 |
| [Details](http://mirdb.org/cgi-bin/target_detail.cgi?targetID=1440342) | 581 | 86 | hsa-miR-30c-5p | [MRPL19](http://www.ncbi.nlm.nih.gov/entrez/query.fcgi?db=gene&cmd=Retrieve&dopt=full_report&list_uids=9801) | mitochondrial ribosomal protein L19 |
| [Details](http://mirdb.org/cgi-bin/target_detail.cgi?targetID=1440373) | 582 | 86 | hsa-miR-30c-5p | [CAMK2N1](http://www.ncbi.nlm.nih.gov/entrez/query.fcgi?db=gene&cmd=Retrieve&dopt=full_report&list_uids=55450) | calcium/calmodulin dependent protein kinase II inhibitor 1 |
| [Details](http://mirdb.org/cgi-bin/target_detail.cgi?targetID=1440427) | 583 | 86 | hsa-miR-30c-5p | [MBOAT1](http://www.ncbi.nlm.nih.gov/entrez/query.fcgi?db=gene&cmd=Retrieve&dopt=full_report&list_uids=154141) | membrane bound O-acyltransferase domain containing 1 |
| [Details](http://mirdb.org/cgi-bin/target_detail.cgi?targetID=1440434) | 584 | 86 | hsa-miR-30c-5p | [SUV39H2](http://www.ncbi.nlm.nih.gov/entrez/query.fcgi?db=gene&cmd=Retrieve&dopt=full_report&list_uids=79723) | suppressor of variegation 3-9 homolog 2 |
| [Details](http://mirdb.org/cgi-bin/target_detail.cgi?targetID=1440469) | 585 | 86 | hsa-miR-30c-5p | [KCTD7](http://www.ncbi.nlm.nih.gov/entrez/query.fcgi?db=gene&cmd=Retrieve&dopt=full_report&list_uids=154881) | potassium channel tetramerization domain containing 7 |
| [Details](http://mirdb.org/cgi-bin/target_detail.cgi?targetID=1440517) | 586 | 86 | hsa-miR-30c-5p | [LYRM7](http://www.ncbi.nlm.nih.gov/entrez/query.fcgi?db=gene&cmd=Retrieve&dopt=full_report&list_uids=90624) | LYR motif containing 7 |
| [Details](http://mirdb.org/cgi-bin/target_detail.cgi?targetID=1440581) | 587 | 86 | hsa-miR-30c-5p | [SCYL3](http://www.ncbi.nlm.nih.gov/entrez/query.fcgi?db=gene&cmd=Retrieve&dopt=full_report&list_uids=57147) | SCY1 like pseudokinase 3 |
| [Details](http://mirdb.org/cgi-bin/target_detail.cgi?targetID=1440593) | 588 | 86 | hsa-miR-30c-5p | [CUL2](http://www.ncbi.nlm.nih.gov/entrez/query.fcgi?db=gene&cmd=Retrieve&dopt=full_report&list_uids=8453) | cullin 2 |
| [Details](http://mirdb.org/cgi-bin/target_detail.cgi?targetID=1440608) | 589 | 86 | hsa-miR-30c-5p | [BMT2](http://www.ncbi.nlm.nih.gov/entrez/query.fcgi?db=gene&cmd=Retrieve&dopt=full_report&list_uids=154743) | base methyltransferase of 25S rRNA 2 homolog |
| [Details](http://mirdb.org/cgi-bin/target_detail.cgi?targetID=1440639) | 590 | 86 | hsa-miR-30c-5p | [PDCD10](http://www.ncbi.nlm.nih.gov/entrez/query.fcgi?db=gene&cmd=Retrieve&dopt=full_report&list_uids=11235) | programmed cell death 10 |
| [Details](http://mirdb.org/cgi-bin/target_detail.cgi?targetID=1440676) | 591 | 86 | hsa-miR-30c-5p | [WDR44](http://www.ncbi.nlm.nih.gov/entrez/query.fcgi?db=gene&cmd=Retrieve&dopt=full_report&list_uids=54521) | WD repeat domain 44 |
| [Details](http://mirdb.org/cgi-bin/target_detail.cgi?targetID=1440705) | 592 | 86 | hsa-miR-30c-5p | [CEP350](http://www.ncbi.nlm.nih.gov/entrez/query.fcgi?db=gene&cmd=Retrieve&dopt=full_report&list_uids=9857) | centrosomal protein 350 |
| [Details](http://mirdb.org/cgi-bin/target_detail.cgi?targetID=1440712) | 593 | 86 | hsa-miR-30c-5p | [PDXDC1](http://www.ncbi.nlm.nih.gov/entrez/query.fcgi?db=gene&cmd=Retrieve&dopt=full_report&list_uids=23042) | pyridoxal dependent decarboxylase domain containing 1 |
| [Details](http://mirdb.org/cgi-bin/target_detail.cgi?targetID=1440767) | 594 | 86 | hsa-miR-30c-5p | [C3orf14](http://www.ncbi.nlm.nih.gov/entrez/query.fcgi?db=gene&cmd=Retrieve&dopt=full_report&list_uids=57415) | chromosome 3 open reading frame 14 |
| [Details](http://mirdb.org/cgi-bin/target_detail.cgi?targetID=1440885) | 595 | 86 | hsa-miR-30c-5p | [TTLL2](http://www.ncbi.nlm.nih.gov/entrez/query.fcgi?db=gene&cmd=Retrieve&dopt=full_report&list_uids=83887) | tubulin tyrosine ligase like 2 |
| [Details](http://mirdb.org/cgi-bin/target_detail.cgi?targetID=1440917) | 596 | 86 | hsa-miR-30c-5p | [AP4E1](http://www.ncbi.nlm.nih.gov/entrez/query.fcgi?db=gene&cmd=Retrieve&dopt=full_report&list_uids=23431) | adaptor related protein complex 4 subunit epsilon 1 |
| [Details](http://mirdb.org/cgi-bin/target_detail.cgi?targetID=1440940) | 597 | 86 | hsa-miR-30c-5p | [EDC3](http://www.ncbi.nlm.nih.gov/entrez/query.fcgi?db=gene&cmd=Retrieve&dopt=full_report&list_uids=80153) | enhancer of mRNA decapping 3 |
| [Details](http://mirdb.org/cgi-bin/target_detail.cgi?targetID=1440960) | 598 | 86 | hsa-miR-30c-5p | [UBE3C](http://www.ncbi.nlm.nih.gov/entrez/query.fcgi?db=gene&cmd=Retrieve&dopt=full_report&list_uids=9690) | ubiquitin protein ligase E3C |
| [Details](http://mirdb.org/cgi-bin/target_detail.cgi?targetID=1440986) | 599 | 86 | hsa-miR-30c-5p | [MXRA5](http://www.ncbi.nlm.nih.gov/entrez/query.fcgi?db=gene&cmd=Retrieve&dopt=full_report&list_uids=25878) | matrix remodeling associated 5 |
| [Details](http://mirdb.org/cgi-bin/target_detail.cgi?targetID=1440990) | 600 | 86 | hsa-miR-30c-5p | [GCLC](http://www.ncbi.nlm.nih.gov/entrez/query.fcgi?db=gene&cmd=Retrieve&dopt=full_report&list_uids=2729) | glutamate-cysteine ligase catalytic subunit |
| [Details](http://mirdb.org/cgi-bin/target_detail.cgi?targetID=1439474) | 601 | 85 | hsa-miR-30c-5p | [TMEFF1](http://www.ncbi.nlm.nih.gov/entrez/query.fcgi?db=gene&cmd=Retrieve&dopt=full_report&list_uids=8577) | transmembrane protein with EGF like and two follistatin like domains 1 |
| [Details](http://mirdb.org/cgi-bin/target_detail.cgi?targetID=1439486) | 602 | 85 | hsa-miR-30c-5p | [PSD3](http://www.ncbi.nlm.nih.gov/entrez/query.fcgi?db=gene&cmd=Retrieve&dopt=full_report&list_uids=23362) | pleckstrin and Sec7 domain containing 3 |
| [Details](http://mirdb.org/cgi-bin/target_detail.cgi?targetID=1439509) | 603 | 85 | hsa-miR-30c-5p | [PI4K2B](http://www.ncbi.nlm.nih.gov/entrez/query.fcgi?db=gene&cmd=Retrieve&dopt=full_report&list_uids=55300) | phosphatidylinositol 4-kinase type 2 beta |
| [Details](http://mirdb.org/cgi-bin/target_detail.cgi?targetID=1439531) | 604 | 85 | hsa-miR-30c-5p | [LRRC8B](http://www.ncbi.nlm.nih.gov/entrez/query.fcgi?db=gene&cmd=Retrieve&dopt=full_report&list_uids=23507) | leucine rich repeat containing 8 VRAC subunit B |
| [Details](http://mirdb.org/cgi-bin/target_detail.cgi?targetID=1439584) | 605 | 85 | hsa-miR-30c-5p | [SLC4A7](http://www.ncbi.nlm.nih.gov/entrez/query.fcgi?db=gene&cmd=Retrieve&dopt=full_report&list_uids=9497) | solute carrier family 4 member 7 |
| [Details](http://mirdb.org/cgi-bin/target_detail.cgi?targetID=1439665) | 606 | 85 | hsa-miR-30c-5p | [GSKIP](http://www.ncbi.nlm.nih.gov/entrez/query.fcgi?db=gene&cmd=Retrieve&dopt=full_report&list_uids=51527) | GSK3B interacting protein |
| [Details](http://mirdb.org/cgi-bin/target_detail.cgi?targetID=1439698) | 607 | 85 | hsa-miR-30c-5p | [ADGRA2](http://www.ncbi.nlm.nih.gov/entrez/query.fcgi?db=gene&cmd=Retrieve&dopt=full_report&list_uids=25960) | adhesion G protein-coupled receptor A2 |
| [Details](http://mirdb.org/cgi-bin/target_detail.cgi?targetID=1439806) | 608 | 85 | hsa-miR-30c-5p | [CAMK4](http://www.ncbi.nlm.nih.gov/entrez/query.fcgi?db=gene&cmd=Retrieve&dopt=full_report&list_uids=814) | calcium/calmodulin dependent protein kinase IV |
| [Details](http://mirdb.org/cgi-bin/target_detail.cgi?targetID=1439821) | 609 | 85 | hsa-miR-30c-5p | [WIPF1](http://www.ncbi.nlm.nih.gov/entrez/query.fcgi?db=gene&cmd=Retrieve&dopt=full_report&list_uids=7456) | WAS/WASL interacting protein family member 1 |
| [Details](http://mirdb.org/cgi-bin/target_detail.cgi?targetID=1439892) | 610 | 85 | hsa-miR-30c-5p | [RNF122](http://www.ncbi.nlm.nih.gov/entrez/query.fcgi?db=gene&cmd=Retrieve&dopt=full_report&list_uids=79845) | ring finger protein 122 |
| [Details](http://mirdb.org/cgi-bin/target_detail.cgi?targetID=1439945) | 611 | 85 | hsa-miR-30c-5p | [TUT7](http://www.ncbi.nlm.nih.gov/entrez/query.fcgi?db=gene&cmd=Retrieve&dopt=full_report&list_uids=79670) | terminal uridylyl transferase 7 |
| [Details](http://mirdb.org/cgi-bin/target_detail.cgi?targetID=1439964) | 612 | 85 | hsa-miR-30c-5p | [DNAJC25-GNG10](http://www.ncbi.nlm.nih.gov/entrez/query.fcgi?db=gene&cmd=Retrieve&dopt=full_report&list_uids=552891) | DNAJC25-GNG10 readthrough |
| [Details](http://mirdb.org/cgi-bin/target_detail.cgi?targetID=1440003) | 613 | 85 | hsa-miR-30c-5p | [ITGA8](http://www.ncbi.nlm.nih.gov/entrez/query.fcgi?db=gene&cmd=Retrieve&dopt=full_report&list_uids=8516) | integrin subunit alpha 8 |
| [Details](http://mirdb.org/cgi-bin/target_detail.cgi?targetID=1440108) | 614 | 85 | hsa-miR-30c-5p | [GNG10](http://www.ncbi.nlm.nih.gov/entrez/query.fcgi?db=gene&cmd=Retrieve&dopt=full_report&list_uids=2790) | G protein subunit gamma 10 |
| [Details](http://mirdb.org/cgi-bin/target_detail.cgi?targetID=1440199) | 615 | 85 | hsa-miR-30c-5p | [ZFC3H1](http://www.ncbi.nlm.nih.gov/entrez/query.fcgi?db=gene&cmd=Retrieve&dopt=full_report&list_uids=196441) | zinc finger C3H1-type containing |
| [Details](http://mirdb.org/cgi-bin/target_detail.cgi?targetID=1440324) | 616 | 85 | hsa-miR-30c-5p | [CHST1](http://www.ncbi.nlm.nih.gov/entrez/query.fcgi?db=gene&cmd=Retrieve&dopt=full_report&list_uids=8534) | carbohydrate sulfotransferase 1 |
| [Details](http://mirdb.org/cgi-bin/target_detail.cgi?targetID=1440359) | 617 | 85 | hsa-miR-30c-5p | [DIPK2A](http://www.ncbi.nlm.nih.gov/entrez/query.fcgi?db=gene&cmd=Retrieve&dopt=full_report&list_uids=205428) | divergent protein kinase domain 2A |
| [Details](http://mirdb.org/cgi-bin/target_detail.cgi?targetID=1440452) | 618 | 85 | hsa-miR-30c-5p | [COL4A3BP](http://www.ncbi.nlm.nih.gov/entrez/query.fcgi?db=gene&cmd=Retrieve&dopt=full_report&list_uids=10087) | collagen type IV alpha 3 binding protein |
| [Details](http://mirdb.org/cgi-bin/target_detail.cgi?targetID=1440462) | 619 | 85 | hsa-miR-30c-5p | [NUS1](http://www.ncbi.nlm.nih.gov/entrez/query.fcgi?db=gene&cmd=Retrieve&dopt=full_report&list_uids=116150) | NUS1, dehydrodolichyl diphosphate synthase subunit |
| [Details](http://mirdb.org/cgi-bin/target_detail.cgi?targetID=1440503) | 620 | 85 | hsa-miR-30c-5p | [SMDT1](http://www.ncbi.nlm.nih.gov/entrez/query.fcgi?db=gene&cmd=Retrieve&dopt=full_report&list_uids=91689) | single-pass membrane protein with aspartate rich tail 1 |
| [Details](http://mirdb.org/cgi-bin/target_detail.cgi?targetID=1440510) | 621 | 85 | hsa-miR-30c-5p | [PRG4](http://www.ncbi.nlm.nih.gov/entrez/query.fcgi?db=gene&cmd=Retrieve&dopt=full_report&list_uids=10216) | proteoglycan 4 |
| [Details](http://mirdb.org/cgi-bin/target_detail.cgi?targetID=1440550) | 622 | 85 | hsa-miR-30c-5p | [BECN1](http://www.ncbi.nlm.nih.gov/entrez/query.fcgi?db=gene&cmd=Retrieve&dopt=full_report&list_uids=8678) | beclin 1 |
| [Details](http://mirdb.org/cgi-bin/target_detail.cgi?targetID=1440592) | 623 | 85 | hsa-miR-30c-5p | [ADRA1D](http://www.ncbi.nlm.nih.gov/entrez/query.fcgi?db=gene&cmd=Retrieve&dopt=full_report&list_uids=146) | adrenoceptor alpha 1D |
| [Details](http://mirdb.org/cgi-bin/target_detail.cgi?targetID=1440641) | 624 | 85 | hsa-miR-30c-5p | [SDAD1](http://www.ncbi.nlm.nih.gov/entrez/query.fcgi?db=gene&cmd=Retrieve&dopt=full_report&list_uids=55153) | SDA1 domain containing 1 |
| [Details](http://mirdb.org/cgi-bin/target_detail.cgi?targetID=1440770) | 625 | 85 | hsa-miR-30c-5p | [ALG10B](http://www.ncbi.nlm.nih.gov/entrez/query.fcgi?db=gene&cmd=Retrieve&dopt=full_report&list_uids=144245) | ALG10B, alpha-1,2-glucosyltransferase |
| [Details](http://mirdb.org/cgi-bin/target_detail.cgi?targetID=1440800) | 626 | 85 | hsa-miR-30c-5p | [IRF2BP2](http://www.ncbi.nlm.nih.gov/entrez/query.fcgi?db=gene&cmd=Retrieve&dopt=full_report&list_uids=359948) | interferon regulatory factor 2 binding protein 2 |
| [Details](http://mirdb.org/cgi-bin/target_detail.cgi?targetID=1440823) | 627 | 85 | hsa-miR-30c-5p | [WWP1](http://www.ncbi.nlm.nih.gov/entrez/query.fcgi?db=gene&cmd=Retrieve&dopt=full_report&list_uids=11059) | WW domain containing E3 ubiquitin protein ligase 1 |
| [Details](http://mirdb.org/cgi-bin/target_detail.cgi?targetID=1440884) | 628 | 85 | hsa-miR-30c-5p | [OMG](http://www.ncbi.nlm.nih.gov/entrez/query.fcgi?db=gene&cmd=Retrieve&dopt=full_report&list_uids=4974) | oligodendrocyte myelin glycoprotein |
| [Details](http://mirdb.org/cgi-bin/target_detail.cgi?targetID=1440980) | 629 | 85 | hsa-miR-30c-5p | [ADAM22](http://www.ncbi.nlm.nih.gov/entrez/query.fcgi?db=gene&cmd=Retrieve&dopt=full_report&list_uids=53616) | ADAM metallopeptidase domain 22 |
| [Details](http://mirdb.org/cgi-bin/target_detail.cgi?targetID=1439493) | 630 | 84 | hsa-miR-30c-5p | [IL36RN](http://www.ncbi.nlm.nih.gov/entrez/query.fcgi?db=gene&cmd=Retrieve&dopt=full_report&list_uids=26525) | interleukin 36 receptor antagonist |
| [Details](http://mirdb.org/cgi-bin/target_detail.cgi?targetID=1439565) | 631 | 84 | hsa-miR-30c-5p | [KIAA1211L](http://www.ncbi.nlm.nih.gov/entrez/query.fcgi?db=gene&cmd=Retrieve&dopt=full_report&list_uids=343990) | KIAA1211 like |
| [Details](http://mirdb.org/cgi-bin/target_detail.cgi?targetID=1439597) | 632 | 84 | hsa-miR-30c-5p | [KIF11](http://www.ncbi.nlm.nih.gov/entrez/query.fcgi?db=gene&cmd=Retrieve&dopt=full_report&list_uids=3832) | kinesin family member 11 |
| [Details](http://mirdb.org/cgi-bin/target_detail.cgi?targetID=1439604) | 633 | 84 | hsa-miR-30c-5p | [PDGFRB](http://www.ncbi.nlm.nih.gov/entrez/query.fcgi?db=gene&cmd=Retrieve&dopt=full_report&list_uids=5159) | platelet derived growth factor receptor beta |
| [Details](http://mirdb.org/cgi-bin/target_detail.cgi?targetID=1439623) | 634 | 84 | hsa-miR-30c-5p | [DMXL2](http://www.ncbi.nlm.nih.gov/entrez/query.fcgi?db=gene&cmd=Retrieve&dopt=full_report&list_uids=23312) | Dmx like 2 |
| [Details](http://mirdb.org/cgi-bin/target_detail.cgi?targetID=1439644) | 635 | 84 | hsa-miR-30c-5p | [CADM2](http://www.ncbi.nlm.nih.gov/entrez/query.fcgi?db=gene&cmd=Retrieve&dopt=full_report&list_uids=253559) | cell adhesion molecule 2 |
| [Details](http://mirdb.org/cgi-bin/target_detail.cgi?targetID=1439648) | 636 | 84 | hsa-miR-30c-5p | [PDS5B](http://www.ncbi.nlm.nih.gov/entrez/query.fcgi?db=gene&cmd=Retrieve&dopt=full_report&list_uids=23047) | PDS5 cohesin associated factor B |
| [Details](http://mirdb.org/cgi-bin/target_detail.cgi?targetID=1439661) | 637 | 84 | hsa-miR-30c-5p | [TAF4B](http://www.ncbi.nlm.nih.gov/entrez/query.fcgi?db=gene&cmd=Retrieve&dopt=full_report&list_uids=6875) | TATA-box binding protein associated factor 4b |
| [Details](http://mirdb.org/cgi-bin/target_detail.cgi?targetID=1439692) | 638 | 84 | hsa-miR-30c-5p | [RAPGEF2](http://www.ncbi.nlm.nih.gov/entrez/query.fcgi?db=gene&cmd=Retrieve&dopt=full_report&list_uids=9693) | Rap guanine nucleotide exchange factor 2 |
| [Details](http://mirdb.org/cgi-bin/target_detail.cgi?targetID=1439713) | 639 | 84 | hsa-miR-30c-5p | [PPTC7](http://www.ncbi.nlm.nih.gov/entrez/query.fcgi?db=gene&cmd=Retrieve&dopt=full_report&list_uids=160760) | PTC7 protein phosphatase homolog |
| [Details](http://mirdb.org/cgi-bin/target_detail.cgi?targetID=1439876) | 640 | 84 | hsa-miR-30c-5p | [NAP1L2](http://www.ncbi.nlm.nih.gov/entrez/query.fcgi?db=gene&cmd=Retrieve&dopt=full_report&list_uids=4674) | nucleosome assembly protein 1 like 2 |
| [Details](http://mirdb.org/cgi-bin/target_detail.cgi?targetID=1439924) | 641 | 84 | hsa-miR-30c-5p | [LRCH2](http://www.ncbi.nlm.nih.gov/entrez/query.fcgi?db=gene&cmd=Retrieve&dopt=full_report&list_uids=57631) | leucine rich repeats and calponin homology domain containing 2 |
| [Details](http://mirdb.org/cgi-bin/target_detail.cgi?targetID=1439959) | 642 | 84 | hsa-miR-30c-5p | [ERRFI1](http://www.ncbi.nlm.nih.gov/entrez/query.fcgi?db=gene&cmd=Retrieve&dopt=full_report&list_uids=54206) | ERBB receptor feedback inhibitor 1 |
| [Details](http://mirdb.org/cgi-bin/target_detail.cgi?targetID=1440004) | 643 | 84 | hsa-miR-30c-5p | [RAB2A](http://www.ncbi.nlm.nih.gov/entrez/query.fcgi?db=gene&cmd=Retrieve&dopt=full_report&list_uids=5862) | RAB2A, member RAS oncogene family |
| [Details](http://mirdb.org/cgi-bin/target_detail.cgi?targetID=1440013) | 644 | 84 | hsa-miR-30c-5p | [NKX2-2](http://www.ncbi.nlm.nih.gov/entrez/query.fcgi?db=gene&cmd=Retrieve&dopt=full_report&list_uids=4821) | NK2 homeobox 2 |
| [Details](http://mirdb.org/cgi-bin/target_detail.cgi?targetID=1440091) | 645 | 84 | hsa-miR-30c-5p | [SLC35B4](http://www.ncbi.nlm.nih.gov/entrez/query.fcgi?db=gene&cmd=Retrieve&dopt=full_report&list_uids=84912) | solute carrier family 35 member B4 |
| [Details](http://mirdb.org/cgi-bin/target_detail.cgi?targetID=1440100) | 646 | 84 | hsa-miR-30c-5p | [ELMOD2](http://www.ncbi.nlm.nih.gov/entrez/query.fcgi?db=gene&cmd=Retrieve&dopt=full_report&list_uids=255520) | ELMO domain containing 2 |
| [Details](http://mirdb.org/cgi-bin/target_detail.cgi?targetID=1440126) | 647 | 84 | hsa-miR-30c-5p | [KATNBL1](http://www.ncbi.nlm.nih.gov/entrez/query.fcgi?db=gene&cmd=Retrieve&dopt=full_report&list_uids=79768) | katanin regulatory subunit B1 like 1 |
| [Details](http://mirdb.org/cgi-bin/target_detail.cgi?targetID=1440130) | 648 | 84 | hsa-miR-30c-5p | [BRAP](http://www.ncbi.nlm.nih.gov/entrez/query.fcgi?db=gene&cmd=Retrieve&dopt=full_report&list_uids=8315) | BRCA1 associated protein |
| [Details](http://mirdb.org/cgi-bin/target_detail.cgi?targetID=1440162) | 649 | 84 | hsa-miR-30c-5p | [FBXL17](http://www.ncbi.nlm.nih.gov/entrez/query.fcgi?db=gene&cmd=Retrieve&dopt=full_report&list_uids=64839) | F-box and leucine rich repeat protein 17 |
| [Details](http://mirdb.org/cgi-bin/target_detail.cgi?targetID=1440183) | 650 | 84 | hsa-miR-30c-5p | [RRAS2](http://www.ncbi.nlm.nih.gov/entrez/query.fcgi?db=gene&cmd=Retrieve&dopt=full_report&list_uids=22800) | RAS related 2 |
| [Details](http://mirdb.org/cgi-bin/target_detail.cgi?targetID=1440356) | 651 | 84 | hsa-miR-30c-5p | [SLC30A4](http://www.ncbi.nlm.nih.gov/entrez/query.fcgi?db=gene&cmd=Retrieve&dopt=full_report&list_uids=7782) | solute carrier family 30 member 4 |
| [Details](http://mirdb.org/cgi-bin/target_detail.cgi?targetID=1440360) | 652 | 84 | hsa-miR-30c-5p | [SOX13](http://www.ncbi.nlm.nih.gov/entrez/query.fcgi?db=gene&cmd=Retrieve&dopt=full_report&list_uids=9580) | SRY-box 13 |
| [Details](http://mirdb.org/cgi-bin/target_detail.cgi?targetID=1440377) | 653 | 84 | hsa-miR-30c-5p | [SOGA1](http://www.ncbi.nlm.nih.gov/entrez/query.fcgi?db=gene&cmd=Retrieve&dopt=full_report&list_uids=140710) | suppressor of glucose, autophagy associated 1 |
| [Details](http://mirdb.org/cgi-bin/target_detail.cgi?targetID=1440401) | 654 | 84 | hsa-miR-30c-5p | [NHS](http://www.ncbi.nlm.nih.gov/entrez/query.fcgi?db=gene&cmd=Retrieve&dopt=full_report&list_uids=4810) | NHS actin remodeling regulator |
| [Details](http://mirdb.org/cgi-bin/target_detail.cgi?targetID=1440436) | 655 | 84 | hsa-miR-30c-5p | [NEFL](http://www.ncbi.nlm.nih.gov/entrez/query.fcgi?db=gene&cmd=Retrieve&dopt=full_report&list_uids=4747) | neurofilament light |
| [Details](http://mirdb.org/cgi-bin/target_detail.cgi?targetID=1440443) | 656 | 84 | hsa-miR-30c-5p | [RTN4R](http://www.ncbi.nlm.nih.gov/entrez/query.fcgi?db=gene&cmd=Retrieve&dopt=full_report&list_uids=65078) | reticulon 4 receptor |
| [Details](http://mirdb.org/cgi-bin/target_detail.cgi?targetID=1440444) | 657 | 84 | hsa-miR-30c-5p | [GNPDA1](http://www.ncbi.nlm.nih.gov/entrez/query.fcgi?db=gene&cmd=Retrieve&dopt=full_report&list_uids=10007) | glucosamine-6-phosphate deaminase 1 |
| [Details](http://mirdb.org/cgi-bin/target_detail.cgi?targetID=1440565) | 658 | 84 | hsa-miR-30c-5p | [FBXO34](http://www.ncbi.nlm.nih.gov/entrez/query.fcgi?db=gene&cmd=Retrieve&dopt=full_report&list_uids=55030) | F-box protein 34 |
| [Details](http://mirdb.org/cgi-bin/target_detail.cgi?targetID=1440612) | 659 | 84 | hsa-miR-30c-5p | [MARCH8](http://www.ncbi.nlm.nih.gov/entrez/query.fcgi?db=gene&cmd=Retrieve&dopt=full_report&list_uids=220972) | membrane associated ring-CH-type finger 8 |
| [Details](http://mirdb.org/cgi-bin/target_detail.cgi?targetID=1440644) | 660 | 84 | hsa-miR-30c-5p | [ERG](http://www.ncbi.nlm.nih.gov/entrez/query.fcgi?db=gene&cmd=Retrieve&dopt=full_report&list_uids=2078) | ETS transcription factor ERG |
| [Details](http://mirdb.org/cgi-bin/target_detail.cgi?targetID=1440808) | 661 | 84 | hsa-miR-30c-5p | [DACT1](http://www.ncbi.nlm.nih.gov/entrez/query.fcgi?db=gene&cmd=Retrieve&dopt=full_report&list_uids=51339) | dishevelled binding antagonist of beta catenin 1 |
| [Details](http://mirdb.org/cgi-bin/target_detail.cgi?targetID=1440838) | 662 | 84 | hsa-miR-30c-5p | [VKORC1L1](http://www.ncbi.nlm.nih.gov/entrez/query.fcgi?db=gene&cmd=Retrieve&dopt=full_report&list_uids=154807) | vitamin K epoxide reductase complex subunit 1 like 1 |
| [Details](http://mirdb.org/cgi-bin/target_detail.cgi?targetID=1440840) | 663 | 84 | hsa-miR-30c-5p | [NEFM](http://www.ncbi.nlm.nih.gov/entrez/query.fcgi?db=gene&cmd=Retrieve&dopt=full_report&list_uids=4741) | neurofilament medium |
| [Details](http://mirdb.org/cgi-bin/target_detail.cgi?targetID=1440844) | 664 | 84 | hsa-miR-30c-5p | [KIF16B](http://www.ncbi.nlm.nih.gov/entrez/query.fcgi?db=gene&cmd=Retrieve&dopt=full_report&list_uids=55614) | kinesin family member 16B |
| [Details](http://mirdb.org/cgi-bin/target_detail.cgi?targetID=1440870) | 665 | 84 | hsa-miR-30c-5p | [NRG3](http://www.ncbi.nlm.nih.gov/entrez/query.fcgi?db=gene&cmd=Retrieve&dopt=full_report&list_uids=10718) | neuregulin 3 |
| [Details](http://mirdb.org/cgi-bin/target_detail.cgi?targetID=1440900) | 666 | 84 | hsa-miR-30c-5p | [MEX3C](http://www.ncbi.nlm.nih.gov/entrez/query.fcgi?db=gene&cmd=Retrieve&dopt=full_report&list_uids=51320) | mex-3 RNA binding family member C |
| [Details](http://mirdb.org/cgi-bin/target_detail.cgi?targetID=1440923) | 667 | 84 | hsa-miR-30c-5p | [CCNJL](http://www.ncbi.nlm.nih.gov/entrez/query.fcgi?db=gene&cmd=Retrieve&dopt=full_report&list_uids=79616) | cyclin J like |
| [Details](http://mirdb.org/cgi-bin/target_detail.cgi?targetID=1440958) | 668 | 84 | hsa-miR-30c-5p | [NFATC3](http://www.ncbi.nlm.nih.gov/entrez/query.fcgi?db=gene&cmd=Retrieve&dopt=full_report&list_uids=4775) | nuclear factor of activated T cells 3 |
| [Details](http://mirdb.org/cgi-bin/target_detail.cgi?targetID=1439457) | 669 | 83 | hsa-miR-30c-5p | [TBC1D2B](http://www.ncbi.nlm.nih.gov/entrez/query.fcgi?db=gene&cmd=Retrieve&dopt=full_report&list_uids=23102) | TBC1 domain family member 2B |
| [Details](http://mirdb.org/cgi-bin/target_detail.cgi?targetID=1439512) | 670 | 83 | hsa-miR-30c-5p | [RTN4IP1](http://www.ncbi.nlm.nih.gov/entrez/query.fcgi?db=gene&cmd=Retrieve&dopt=full_report&list_uids=84816) | reticulon 4 interacting protein 1 |
| [Details](http://mirdb.org/cgi-bin/target_detail.cgi?targetID=1439601) | 671 | 83 | hsa-miR-30c-5p | [PAX3](http://www.ncbi.nlm.nih.gov/entrez/query.fcgi?db=gene&cmd=Retrieve&dopt=full_report&list_uids=5077) | paired box 3 |
| [Details](http://mirdb.org/cgi-bin/target_detail.cgi?targetID=1439621) | 672 | 83 | hsa-miR-30c-5p | [TSEN15](http://www.ncbi.nlm.nih.gov/entrez/query.fcgi?db=gene&cmd=Retrieve&dopt=full_report&list_uids=116461) | tRNA splicing endonuclease subunit 15 |
| [Details](http://mirdb.org/cgi-bin/target_detail.cgi?targetID=1439628) | 673 | 83 | hsa-miR-30c-5p | [STAG2](http://www.ncbi.nlm.nih.gov/entrez/query.fcgi?db=gene&cmd=Retrieve&dopt=full_report&list_uids=10735) | stromal antigen 2 |
| [Details](http://mirdb.org/cgi-bin/target_detail.cgi?targetID=1439636) | 674 | 83 | hsa-miR-30c-5p | [PIEZO2](http://www.ncbi.nlm.nih.gov/entrez/query.fcgi?db=gene&cmd=Retrieve&dopt=full_report&list_uids=63895) | piezo type mechanosensitive ion channel component 2 |
| [Details](http://mirdb.org/cgi-bin/target_detail.cgi?targetID=1439653) | 675 | 83 | hsa-miR-30c-5p | [ARMH3](http://www.ncbi.nlm.nih.gov/entrez/query.fcgi?db=gene&cmd=Retrieve&dopt=full_report&list_uids=79591) | armadillo-like helical domain containing 3 |
| [Details](http://mirdb.org/cgi-bin/target_detail.cgi?targetID=1439674) | 676 | 83 | hsa-miR-30c-5p | [ABHD10](http://www.ncbi.nlm.nih.gov/entrez/query.fcgi?db=gene&cmd=Retrieve&dopt=full_report&list_uids=55347) | abhydrolase domain containing 10 |
| [Details](http://mirdb.org/cgi-bin/target_detail.cgi?targetID=1439746) | 677 | 83 | hsa-miR-30c-5p | [ASB2](http://www.ncbi.nlm.nih.gov/entrez/query.fcgi?db=gene&cmd=Retrieve&dopt=full_report&list_uids=51676) | ankyrin repeat and SOCS box containing 2 |
| [Details](http://mirdb.org/cgi-bin/target_detail.cgi?targetID=1439757) | 678 | 83 | hsa-miR-30c-5p | [ADO](http://www.ncbi.nlm.nih.gov/entrez/query.fcgi?db=gene&cmd=Retrieve&dopt=full_report&list_uids=84890) | 2-aminoethanethiol dioxygenase |
| [Details](http://mirdb.org/cgi-bin/target_detail.cgi?targetID=1439764) | 679 | 83 | hsa-miR-30c-5p | [AFF4](http://www.ncbi.nlm.nih.gov/entrez/query.fcgi?db=gene&cmd=Retrieve&dopt=full_report&list_uids=27125) | AF4/FMR2 family member 4 |
| [Details](http://mirdb.org/cgi-bin/target_detail.cgi?targetID=1439773) | 680 | 83 | hsa-miR-30c-5p | [ZNF519](http://www.ncbi.nlm.nih.gov/entrez/query.fcgi?db=gene&cmd=Retrieve&dopt=full_report&list_uids=162655) | zinc finger protein 519 |
| [Details](http://mirdb.org/cgi-bin/target_detail.cgi?targetID=1439859) | 681 | 83 | hsa-miR-30c-5p | [GIGYF1](http://www.ncbi.nlm.nih.gov/entrez/query.fcgi?db=gene&cmd=Retrieve&dopt=full_report&list_uids=64599) | GRB10 interacting GYF protein 1 |
| [Details](http://mirdb.org/cgi-bin/target_detail.cgi?targetID=1439925) | 682 | 83 | hsa-miR-30c-5p | [CYP3A5](http://www.ncbi.nlm.nih.gov/entrez/query.fcgi?db=gene&cmd=Retrieve&dopt=full_report&list_uids=1577) | cytochrome P450 family 3 subfamily A member 5 |
| [Details](http://mirdb.org/cgi-bin/target_detail.cgi?targetID=1439949) | 683 | 83 | hsa-miR-30c-5p | [SOCS6](http://www.ncbi.nlm.nih.gov/entrez/query.fcgi?db=gene&cmd=Retrieve&dopt=full_report&list_uids=9306) | suppressor of cytokine signaling 6 |
| [Details](http://mirdb.org/cgi-bin/target_detail.cgi?targetID=1439992) | 684 | 83 | hsa-miR-30c-5p | [TMCC1](http://www.ncbi.nlm.nih.gov/entrez/query.fcgi?db=gene&cmd=Retrieve&dopt=full_report&list_uids=23023) | transmembrane and coiled-coil domain family 1 |
| [Details](http://mirdb.org/cgi-bin/target_detail.cgi?targetID=1440018) | 685 | 83 | hsa-miR-30c-5p | [ELOVL2](http://www.ncbi.nlm.nih.gov/entrez/query.fcgi?db=gene&cmd=Retrieve&dopt=full_report&list_uids=54898) | ELOVL fatty acid elongase 2 |
| [Details](http://mirdb.org/cgi-bin/target_detail.cgi?targetID=1440020) | 686 | 83 | hsa-miR-30c-5p | [LATS2](http://www.ncbi.nlm.nih.gov/entrez/query.fcgi?db=gene&cmd=Retrieve&dopt=full_report&list_uids=26524) | large tumor suppressor kinase 2 |
| [Details](http://mirdb.org/cgi-bin/target_detail.cgi?targetID=1440063) | 687 | 83 | hsa-miR-30c-5p | [SEC61A2](http://www.ncbi.nlm.nih.gov/entrez/query.fcgi?db=gene&cmd=Retrieve&dopt=full_report&list_uids=55176) | Sec61 translocon alpha 2 subunit |
| [Details](http://mirdb.org/cgi-bin/target_detail.cgi?targetID=1440133) | 688 | 83 | hsa-miR-30c-5p | [EEF1A1](http://www.ncbi.nlm.nih.gov/entrez/query.fcgi?db=gene&cmd=Retrieve&dopt=full_report&list_uids=1915) | eukaryotic translation elongation factor 1 alpha 1 |
| [Details](http://mirdb.org/cgi-bin/target_detail.cgi?targetID=1440149) | 689 | 83 | hsa-miR-30c-5p | [GPCPD1](http://www.ncbi.nlm.nih.gov/entrez/query.fcgi?db=gene&cmd=Retrieve&dopt=full_report&list_uids=56261) | glycerophosphocholine phosphodiesterase 1 |
| [Details](http://mirdb.org/cgi-bin/target_detail.cgi?targetID=1440160) | 690 | 83 | hsa-miR-30c-5p | [RALGDS](http://www.ncbi.nlm.nih.gov/entrez/query.fcgi?db=gene&cmd=Retrieve&dopt=full_report&list_uids=5900) | ral guanine nucleotide dissociation stimulator |
| [Details](http://mirdb.org/cgi-bin/target_detail.cgi?targetID=1440265) | 691 | 83 | hsa-miR-30c-5p | [SEMA3A](http://www.ncbi.nlm.nih.gov/entrez/query.fcgi?db=gene&cmd=Retrieve&dopt=full_report&list_uids=10371) | semaphorin 3A |
| [Details](http://mirdb.org/cgi-bin/target_detail.cgi?targetID=1440311) | 692 | 83 | hsa-miR-30c-5p | [CDK12](http://www.ncbi.nlm.nih.gov/entrez/query.fcgi?db=gene&cmd=Retrieve&dopt=full_report&list_uids=51755) | cyclin dependent kinase 12 |
| [Details](http://mirdb.org/cgi-bin/target_detail.cgi?targetID=1440416) | 693 | 83 | hsa-miR-30c-5p | [MAPK8](http://www.ncbi.nlm.nih.gov/entrez/query.fcgi?db=gene&cmd=Retrieve&dopt=full_report&list_uids=5599) | mitogen-activated protein kinase 8 |
| [Details](http://mirdb.org/cgi-bin/target_detail.cgi?targetID=1440543) | 694 | 83 | hsa-miR-30c-5p | [RALGPS1](http://www.ncbi.nlm.nih.gov/entrez/query.fcgi?db=gene&cmd=Retrieve&dopt=full_report&list_uids=9649) | Ral GEF with PH domain and SH3 binding motif 1 |
| [Details](http://mirdb.org/cgi-bin/target_detail.cgi?targetID=1440631) | 695 | 83 | hsa-miR-30c-5p | [HBS1L](http://www.ncbi.nlm.nih.gov/entrez/query.fcgi?db=gene&cmd=Retrieve&dopt=full_report&list_uids=10767) | HBS1 like translational GTPase |
| [Details](http://mirdb.org/cgi-bin/target_detail.cgi?targetID=1440691) | 696 | 83 | hsa-miR-30c-5p | [PRICKLE1](http://www.ncbi.nlm.nih.gov/entrez/query.fcgi?db=gene&cmd=Retrieve&dopt=full_report&list_uids=144165) | prickle planar cell polarity protein 1 |
| [Details](http://mirdb.org/cgi-bin/target_detail.cgi?targetID=1440701) | 697 | 83 | hsa-miR-30c-5p | [CBFB](http://www.ncbi.nlm.nih.gov/entrez/query.fcgi?db=gene&cmd=Retrieve&dopt=full_report&list_uids=865) | core-binding factor subunit beta |
| [Details](http://mirdb.org/cgi-bin/target_detail.cgi?targetID=1440723) | 698 | 83 | hsa-miR-30c-5p | [PLIN3](http://www.ncbi.nlm.nih.gov/entrez/query.fcgi?db=gene&cmd=Retrieve&dopt=full_report&list_uids=10226) | perilipin 3 |
| [Details](http://mirdb.org/cgi-bin/target_detail.cgi?targetID=1440796) | 699 | 83 | hsa-miR-30c-5p | [RAVER2](http://www.ncbi.nlm.nih.gov/entrez/query.fcgi?db=gene&cmd=Retrieve&dopt=full_report&list_uids=55225) | ribonucleoprotein, PTB binding 2 |
| [Details](http://mirdb.org/cgi-bin/target_detail.cgi?targetID=1440818) | 700 | 83 | hsa-miR-30c-5p | [FAM13C](http://www.ncbi.nlm.nih.gov/entrez/query.fcgi?db=gene&cmd=Retrieve&dopt=full_report&list_uids=220965) | family with sequence similarity 13 member C |
| [Details](http://mirdb.org/cgi-bin/target_detail.cgi?targetID=1440837) | 701 | 83 | hsa-miR-30c-5p | [FAM199X](http://www.ncbi.nlm.nih.gov/entrez/query.fcgi?db=gene&cmd=Retrieve&dopt=full_report&list_uids=139231) | family with sequence similarity 199, X-linked |
| [Details](http://mirdb.org/cgi-bin/target_detail.cgi?targetID=1440839) | 702 | 83 | hsa-miR-30c-5p | [NUFIP2](http://www.ncbi.nlm.nih.gov/entrez/query.fcgi?db=gene&cmd=Retrieve&dopt=full_report&list_uids=57532) | nuclear FMR1 interacting protein 2 |
| [Details](http://mirdb.org/cgi-bin/target_detail.cgi?targetID=1440874) | 703 | 83 | hsa-miR-30c-5p | [CPEB3](http://www.ncbi.nlm.nih.gov/entrez/query.fcgi?db=gene&cmd=Retrieve&dopt=full_report&list_uids=22849) | cytoplasmic polyadenylation element binding protein 3 |
| [Details](http://mirdb.org/cgi-bin/target_detail.cgi?targetID=1440918) | 704 | 83 | hsa-miR-30c-5p | [SP4](http://www.ncbi.nlm.nih.gov/entrez/query.fcgi?db=gene&cmd=Retrieve&dopt=full_report&list_uids=6671) | Sp4 transcription factor |
| [Details](http://mirdb.org/cgi-bin/target_detail.cgi?targetID=1440921) | 705 | 83 | hsa-miR-30c-5p | [FAM229B](http://www.ncbi.nlm.nih.gov/entrez/query.fcgi?db=gene&cmd=Retrieve&dopt=full_report&list_uids=619208) | family with sequence similarity 229 member B |
| [Details](http://mirdb.org/cgi-bin/target_detail.cgi?targetID=1440928) | 706 | 83 | hsa-miR-30c-5p | [MAP3K7](http://www.ncbi.nlm.nih.gov/entrez/query.fcgi?db=gene&cmd=Retrieve&dopt=full_report&list_uids=6885) | mitogen-activated protein kinase kinase kinase 7 |
| [Details](http://mirdb.org/cgi-bin/target_detail.cgi?targetID=1440964) | 707 | 83 | hsa-miR-30c-5p | [KXD1](http://www.ncbi.nlm.nih.gov/entrez/query.fcgi?db=gene&cmd=Retrieve&dopt=full_report&list_uids=79036) | KxDL motif containing 1 |
| [Details](http://mirdb.org/cgi-bin/target_detail.cgi?targetID=1440993) | 708 | 83 | hsa-miR-30c-5p | [PHACTR2](http://www.ncbi.nlm.nih.gov/entrez/query.fcgi?db=gene&cmd=Retrieve&dopt=full_report&list_uids=9749) | phosphatase and actin regulator 2 |
| [Details](http://mirdb.org/cgi-bin/target_detail.cgi?targetID=1439495) | 709 | 82 | hsa-miR-30c-5p | [FAM13A](http://www.ncbi.nlm.nih.gov/entrez/query.fcgi?db=gene&cmd=Retrieve&dopt=full_report&list_uids=10144) | family with sequence similarity 13 member A |
| [Details](http://mirdb.org/cgi-bin/target_detail.cgi?targetID=1439541) | 710 | 82 | hsa-miR-30c-5p | [ATP2B1](http://www.ncbi.nlm.nih.gov/entrez/query.fcgi?db=gene&cmd=Retrieve&dopt=full_report&list_uids=490) | ATPase plasma membrane Ca2+ transporting 1 |
| [Details](http://mirdb.org/cgi-bin/target_detail.cgi?targetID=1439585) | 711 | 82 | hsa-miR-30c-5p | [HACE1](http://www.ncbi.nlm.nih.gov/entrez/query.fcgi?db=gene&cmd=Retrieve&dopt=full_report&list_uids=57531) | HECT domain and ankyrin repeat containing E3 ubiquitin protein ligase 1 |
| [Details](http://mirdb.org/cgi-bin/target_detail.cgi?targetID=1439620) | 712 | 82 | hsa-miR-30c-5p | [DCUN1D1](http://www.ncbi.nlm.nih.gov/entrez/query.fcgi?db=gene&cmd=Retrieve&dopt=full_report&list_uids=54165) | defective in cullin neddylation 1 domain containing 1 |
| [Details](http://mirdb.org/cgi-bin/target_detail.cgi?targetID=1439625) | 713 | 82 | hsa-miR-30c-5p | [NDUFC2](http://www.ncbi.nlm.nih.gov/entrez/query.fcgi?db=gene&cmd=Retrieve&dopt=full_report&list_uids=4718) | NADH:ubiquinone oxidoreductase subunit C2 |
| [Details](http://mirdb.org/cgi-bin/target_detail.cgi?targetID=1439734) | 714 | 82 | hsa-miR-30c-5p | [ADAM12](http://www.ncbi.nlm.nih.gov/entrez/query.fcgi?db=gene&cmd=Retrieve&dopt=full_report&list_uids=8038) | ADAM metallopeptidase domain 12 |
| [Details](http://mirdb.org/cgi-bin/target_detail.cgi?targetID=1439737) | 715 | 82 | hsa-miR-30c-5p | [ATL2](http://www.ncbi.nlm.nih.gov/entrez/query.fcgi?db=gene&cmd=Retrieve&dopt=full_report&list_uids=64225) | atlastin GTPase 2 |
| [Details](http://mirdb.org/cgi-bin/target_detail.cgi?targetID=1439775) | 716 | 82 | hsa-miR-30c-5p | [GRB10](http://www.ncbi.nlm.nih.gov/entrez/query.fcgi?db=gene&cmd=Retrieve&dopt=full_report&list_uids=2887) | growth factor receptor bound protein 10 |
| [Details](http://mirdb.org/cgi-bin/target_detail.cgi?targetID=1439831) | 717 | 82 | hsa-miR-30c-5p | [ZNF286A](http://www.ncbi.nlm.nih.gov/entrez/query.fcgi?db=gene&cmd=Retrieve&dopt=full_report&list_uids=57335) | zinc finger protein 286A |
| [Details](http://mirdb.org/cgi-bin/target_detail.cgi?targetID=1439861) | 718 | 82 | hsa-miR-30c-5p | [RFX3](http://www.ncbi.nlm.nih.gov/entrez/query.fcgi?db=gene&cmd=Retrieve&dopt=full_report&list_uids=5991) | regulatory factor X3 |
| [Details](http://mirdb.org/cgi-bin/target_detail.cgi?targetID=1439896) | 719 | 82 | hsa-miR-30c-5p | [THAP12](http://www.ncbi.nlm.nih.gov/entrez/query.fcgi?db=gene&cmd=Retrieve&dopt=full_report&list_uids=5612) | THAP domain containing 12 |
| [Details](http://mirdb.org/cgi-bin/target_detail.cgi?targetID=1439965) | 720 | 82 | hsa-miR-30c-5p | [RAB15](http://www.ncbi.nlm.nih.gov/entrez/query.fcgi?db=gene&cmd=Retrieve&dopt=full_report&list_uids=376267) | RAB15, member RAS oncogene family |
| [Details](http://mirdb.org/cgi-bin/target_detail.cgi?targetID=1440042) | 721 | 82 | hsa-miR-30c-5p | [SHOC2](http://www.ncbi.nlm.nih.gov/entrez/query.fcgi?db=gene&cmd=Retrieve&dopt=full_report&list_uids=8036) | SHOC2, leucine rich repeat scaffold protein |
| [Details](http://mirdb.org/cgi-bin/target_detail.cgi?targetID=1440121) | 722 | 82 | hsa-miR-30c-5p | [CRKL](http://www.ncbi.nlm.nih.gov/entrez/query.fcgi?db=gene&cmd=Retrieve&dopt=full_report&list_uids=1399) | CRK like proto-oncogene, adaptor protein |
| [Details](http://mirdb.org/cgi-bin/target_detail.cgi?targetID=1440164) | 723 | 82 | hsa-miR-30c-5p | [IGF2R](http://www.ncbi.nlm.nih.gov/entrez/query.fcgi?db=gene&cmd=Retrieve&dopt=full_report&list_uids=3482) | insulin like growth factor 2 receptor |
| [Details](http://mirdb.org/cgi-bin/target_detail.cgi?targetID=1440182) | 724 | 82 | hsa-miR-30c-5p | [ATF1](http://www.ncbi.nlm.nih.gov/entrez/query.fcgi?db=gene&cmd=Retrieve&dopt=full_report&list_uids=466) | activating transcription factor 1 |
| [Details](http://mirdb.org/cgi-bin/target_detail.cgi?targetID=1440202) | 725 | 82 | hsa-miR-30c-5p | [BNC2](http://www.ncbi.nlm.nih.gov/entrez/query.fcgi?db=gene&cmd=Retrieve&dopt=full_report&list_uids=54796) | basonuclin 2 |
| [Details](http://mirdb.org/cgi-bin/target_detail.cgi?targetID=1440203) | 726 | 82 | hsa-miR-30c-5p | [MIER2](http://www.ncbi.nlm.nih.gov/entrez/query.fcgi?db=gene&cmd=Retrieve&dopt=full_report&list_uids=54531) | MIER family member 2 |
| [Details](http://mirdb.org/cgi-bin/target_detail.cgi?targetID=1440206) | 727 | 82 | hsa-miR-30c-5p | [WIPF3](http://www.ncbi.nlm.nih.gov/entrez/query.fcgi?db=gene&cmd=Retrieve&dopt=full_report&list_uids=644150) | WAS/WASL interacting protein family member 3 |
| [Details](http://mirdb.org/cgi-bin/target_detail.cgi?targetID=1440212) | 728 | 82 | hsa-miR-30c-5p | [TRIO](http://www.ncbi.nlm.nih.gov/entrez/query.fcgi?db=gene&cmd=Retrieve&dopt=full_report&list_uids=7204) | trio Rho guanine nucleotide exchange factor |
| [Details](http://mirdb.org/cgi-bin/target_detail.cgi?targetID=1440238) | 729 | 82 | hsa-miR-30c-5p | [GMEB2](http://www.ncbi.nlm.nih.gov/entrez/query.fcgi?db=gene&cmd=Retrieve&dopt=full_report&list_uids=26205) | glucocorticoid modulatory element binding protein 2 |
| [Details](http://mirdb.org/cgi-bin/target_detail.cgi?targetID=1440320) | 730 | 82 | hsa-miR-30c-5p | [CERS6](http://www.ncbi.nlm.nih.gov/entrez/query.fcgi?db=gene&cmd=Retrieve&dopt=full_report&list_uids=253782) | ceramide synthase 6 |
| [Details](http://mirdb.org/cgi-bin/target_detail.cgi?targetID=1440321) | 731 | 82 | hsa-miR-30c-5p | [ZNF704](http://www.ncbi.nlm.nih.gov/entrez/query.fcgi?db=gene&cmd=Retrieve&dopt=full_report&list_uids=619279) | zinc finger protein 704 |
| [Details](http://mirdb.org/cgi-bin/target_detail.cgi?targetID=1440410) | 732 | 82 | hsa-miR-30c-5p | [GPR75-ASB3](http://www.ncbi.nlm.nih.gov/entrez/query.fcgi?db=gene&cmd=Retrieve&dopt=full_report&list_uids=100302652) | GPR75-ASB3 readthrough |
| [Details](http://mirdb.org/cgi-bin/target_detail.cgi?targetID=1440414) | 733 | 82 | hsa-miR-30c-5p | [CNOT6](http://www.ncbi.nlm.nih.gov/entrez/query.fcgi?db=gene&cmd=Retrieve&dopt=full_report&list_uids=57472) | CCR4-NOT transcription complex subunit 6 |
| [Details](http://mirdb.org/cgi-bin/target_detail.cgi?targetID=1440542) | 734 | 82 | hsa-miR-30c-5p | [SRGAP3](http://www.ncbi.nlm.nih.gov/entrez/query.fcgi?db=gene&cmd=Retrieve&dopt=full_report&list_uids=9901) | SLIT-ROBO Rho GTPase activating protein 3 |
| [Details](http://mirdb.org/cgi-bin/target_detail.cgi?targetID=1440546) | 735 | 82 | hsa-miR-30c-5p | [MPZL3](http://www.ncbi.nlm.nih.gov/entrez/query.fcgi?db=gene&cmd=Retrieve&dopt=full_report&list_uids=196264) | myelin protein zero like 3 |
| [Details](http://mirdb.org/cgi-bin/target_detail.cgi?targetID=1440625) | 736 | 82 | hsa-miR-30c-5p | [SDK2](http://www.ncbi.nlm.nih.gov/entrez/query.fcgi?db=gene&cmd=Retrieve&dopt=full_report&list_uids=54549) | sidekick cell adhesion molecule 2 |
| [Details](http://mirdb.org/cgi-bin/target_detail.cgi?targetID=1440642) | 737 | 82 | hsa-miR-30c-5p | [PRPF40A](http://www.ncbi.nlm.nih.gov/entrez/query.fcgi?db=gene&cmd=Retrieve&dopt=full_report&list_uids=55660) | pre-mRNA processing factor 40 homolog A |
| [Details](http://mirdb.org/cgi-bin/target_detail.cgi?targetID=1440666) | 738 | 82 | hsa-miR-30c-5p | [GPR19](http://www.ncbi.nlm.nih.gov/entrez/query.fcgi?db=gene&cmd=Retrieve&dopt=full_report&list_uids=2842) | G protein-coupled receptor 19 |
| [Details](http://mirdb.org/cgi-bin/target_detail.cgi?targetID=1440668) | 739 | 82 | hsa-miR-30c-5p | [ZFAND1](http://www.ncbi.nlm.nih.gov/entrez/query.fcgi?db=gene&cmd=Retrieve&dopt=full_report&list_uids=79752) | zinc finger AN1-type containing 1 |
| [Details](http://mirdb.org/cgi-bin/target_detail.cgi?targetID=1440675) | 740 | 82 | hsa-miR-30c-5p | [SLC25A34](http://www.ncbi.nlm.nih.gov/entrez/query.fcgi?db=gene&cmd=Retrieve&dopt=full_report&list_uids=284723) | solute carrier family 25 member 34 |
| [Details](http://mirdb.org/cgi-bin/target_detail.cgi?targetID=1440704) | 741 | 82 | hsa-miR-30c-5p | [GDE1](http://www.ncbi.nlm.nih.gov/entrez/query.fcgi?db=gene&cmd=Retrieve&dopt=full_report&list_uids=51573) | glycerophosphodiester phosphodiesterase 1 |
| [Details](http://mirdb.org/cgi-bin/target_detail.cgi?targetID=1440715) | 742 | 82 | hsa-miR-30c-5p | [JADE3](http://www.ncbi.nlm.nih.gov/entrez/query.fcgi?db=gene&cmd=Retrieve&dopt=full_report&list_uids=9767) | jade family PHD finger 3 |
| [Details](http://mirdb.org/cgi-bin/target_detail.cgi?targetID=1440716) | 743 | 82 | hsa-miR-30c-5p | [BCL2L11](http://www.ncbi.nlm.nih.gov/entrez/query.fcgi?db=gene&cmd=Retrieve&dopt=full_report&list_uids=10018) | BCL2 like 11 |
| [Details](http://mirdb.org/cgi-bin/target_detail.cgi?targetID=1440750) | 744 | 82 | hsa-miR-30c-5p | [NCALD](http://www.ncbi.nlm.nih.gov/entrez/query.fcgi?db=gene&cmd=Retrieve&dopt=full_report&list_uids=83988) | neurocalcin delta |
| [Details](http://mirdb.org/cgi-bin/target_detail.cgi?targetID=1440783) | 745 | 82 | hsa-miR-30c-5p | [RNF157](http://www.ncbi.nlm.nih.gov/entrez/query.fcgi?db=gene&cmd=Retrieve&dopt=full_report&list_uids=114804) | ring finger protein 157 |
| [Details](http://mirdb.org/cgi-bin/target_detail.cgi?targetID=1440858) | 746 | 82 | hsa-miR-30c-5p | [ARAF](http://www.ncbi.nlm.nih.gov/entrez/query.fcgi?db=gene&cmd=Retrieve&dopt=full_report&list_uids=369) | A-Raf proto-oncogene, serine/threonine kinase |
| [Details](http://mirdb.org/cgi-bin/target_detail.cgi?targetID=1440895) | 747 | 82 | hsa-miR-30c-5p | [ZNF382](http://www.ncbi.nlm.nih.gov/entrez/query.fcgi?db=gene&cmd=Retrieve&dopt=full_report&list_uids=84911) | zinc finger protein 382 |
| [Details](http://mirdb.org/cgi-bin/target_detail.cgi?targetID=1440930) | 748 | 82 | hsa-miR-30c-5p | [TCIM](http://www.ncbi.nlm.nih.gov/entrez/query.fcgi?db=gene&cmd=Retrieve&dopt=full_report&list_uids=56892) | transcriptional and immune response regulator |
| [Details](http://mirdb.org/cgi-bin/target_detail.cgi?targetID=1440965) | 749 | 82 | hsa-miR-30c-5p | [ORC2](http://www.ncbi.nlm.nih.gov/entrez/query.fcgi?db=gene&cmd=Retrieve&dopt=full_report&list_uids=4999) | origin recognition complex subunit 2 |
| [Details](http://mirdb.org/cgi-bin/target_detail.cgi?targetID=1439649) | 750 | 81 | hsa-miR-30c-5p | [AVEN](http://www.ncbi.nlm.nih.gov/entrez/query.fcgi?db=gene&cmd=Retrieve&dopt=full_report&list_uids=57099) | apoptosis and caspase activation inhibitor |
| [Details](http://mirdb.org/cgi-bin/target_detail.cgi?targetID=1439659) | 751 | 81 | hsa-miR-30c-5p | [STT3B](http://www.ncbi.nlm.nih.gov/entrez/query.fcgi?db=gene&cmd=Retrieve&dopt=full_report&list_uids=201595) | STT3B, catalytic subunit of the oligosaccharyltransferase complex |
| [Details](http://mirdb.org/cgi-bin/target_detail.cgi?targetID=1439774) | 752 | 81 | hsa-miR-30c-5p | [GOLGA8A](http://www.ncbi.nlm.nih.gov/entrez/query.fcgi?db=gene&cmd=Retrieve&dopt=full_report&list_uids=23015) | golgin A8 family member A |
| [Details](http://mirdb.org/cgi-bin/target_detail.cgi?targetID=1439802) | 753 | 81 | hsa-miR-30c-5p | [ARHGAP29](http://www.ncbi.nlm.nih.gov/entrez/query.fcgi?db=gene&cmd=Retrieve&dopt=full_report&list_uids=9411) | Rho GTPase activating protein 29 |
| [Details](http://mirdb.org/cgi-bin/target_detail.cgi?targetID=1439909) | 754 | 81 | hsa-miR-30c-5p | [YTHDC1](http://www.ncbi.nlm.nih.gov/entrez/query.fcgi?db=gene&cmd=Retrieve&dopt=full_report&list_uids=91746) | YTH domain containing 1 |
| [Details](http://mirdb.org/cgi-bin/target_detail.cgi?targetID=1439929) | 755 | 81 | hsa-miR-30c-5p | [NSG1](http://www.ncbi.nlm.nih.gov/entrez/query.fcgi?db=gene&cmd=Retrieve&dopt=full_report&list_uids=27065) | neuronal vesicle trafficking associated 1 |
| [Details](http://mirdb.org/cgi-bin/target_detail.cgi?targetID=1440034) | 756 | 81 | hsa-miR-30c-5p | [GOLGA6C](http://www.ncbi.nlm.nih.gov/entrez/query.fcgi?db=gene&cmd=Retrieve&dopt=full_report&list_uids=653641) | golgin A6 family member C |
| [Details](http://mirdb.org/cgi-bin/target_detail.cgi?targetID=1440166) | 757 | 81 | hsa-miR-30c-5p | [SLC6A6](http://www.ncbi.nlm.nih.gov/entrez/query.fcgi?db=gene&cmd=Retrieve&dopt=full_report&list_uids=6533) | solute carrier family 6 member 6 |
| [Details](http://mirdb.org/cgi-bin/target_detail.cgi?targetID=1440216) | 758 | 81 | hsa-miR-30c-5p | [TOX](http://www.ncbi.nlm.nih.gov/entrez/query.fcgi?db=gene&cmd=Retrieve&dopt=full_report&list_uids=9760) | thymocyte selection associated high mobility group box |
| [Details](http://mirdb.org/cgi-bin/target_detail.cgi?targetID=1440228) | 759 | 81 | hsa-miR-30c-5p | [MAFG](http://www.ncbi.nlm.nih.gov/entrez/query.fcgi?db=gene&cmd=Retrieve&dopt=full_report&list_uids=4097) | MAF bZIP transcription factor G |
| [Details](http://mirdb.org/cgi-bin/target_detail.cgi?targetID=1440388) | 760 | 81 | hsa-miR-30c-5p | [TM4SF20](http://www.ncbi.nlm.nih.gov/entrez/query.fcgi?db=gene&cmd=Retrieve&dopt=full_report&list_uids=79853) | transmembrane 4 L six family member 20 |
| [Details](http://mirdb.org/cgi-bin/target_detail.cgi?targetID=1440428) | 761 | 81 | hsa-miR-30c-5p | [SLC5A3](http://www.ncbi.nlm.nih.gov/entrez/query.fcgi?db=gene&cmd=Retrieve&dopt=full_report&list_uids=6526) | solute carrier family 5 member 3 |
| [Details](http://mirdb.org/cgi-bin/target_detail.cgi?targetID=1440530) | 762 | 81 | hsa-miR-30c-5p | [ZEB2](http://www.ncbi.nlm.nih.gov/entrez/query.fcgi?db=gene&cmd=Retrieve&dopt=full_report&list_uids=9839) | zinc finger E-box binding homeobox 2 |
| [Details](http://mirdb.org/cgi-bin/target_detail.cgi?targetID=1440564) | 763 | 81 | hsa-miR-30c-5p | [VOPP1](http://www.ncbi.nlm.nih.gov/entrez/query.fcgi?db=gene&cmd=Retrieve&dopt=full_report&list_uids=81552) | VOPP1, WBP1/VOPP1 family member |
| [Details](http://mirdb.org/cgi-bin/target_detail.cgi?targetID=1440596) | 764 | 81 | hsa-miR-30c-5p | [ARHGAP26](http://www.ncbi.nlm.nih.gov/entrez/query.fcgi?db=gene&cmd=Retrieve&dopt=full_report&list_uids=23092) | Rho GTPase activating protein 26 |
| [Details](http://mirdb.org/cgi-bin/target_detail.cgi?targetID=1440685) | 765 | 81 | hsa-miR-30c-5p | [ZNF507](http://www.ncbi.nlm.nih.gov/entrez/query.fcgi?db=gene&cmd=Retrieve&dopt=full_report&list_uids=22847) | zinc finger protein 507 |
| [Details](http://mirdb.org/cgi-bin/target_detail.cgi?targetID=1440724) | 766 | 81 | hsa-miR-30c-5p | [CCDC120](http://www.ncbi.nlm.nih.gov/entrez/query.fcgi?db=gene&cmd=Retrieve&dopt=full_report&list_uids=90060) | coiled-coil domain containing 120 |
| [Details](http://mirdb.org/cgi-bin/target_detail.cgi?targetID=1440730) | 767 | 81 | hsa-miR-30c-5p | [CCNA1](http://www.ncbi.nlm.nih.gov/entrez/query.fcgi?db=gene&cmd=Retrieve&dopt=full_report&list_uids=8900) | cyclin A1 |
| [Details](http://mirdb.org/cgi-bin/target_detail.cgi?targetID=1440757) | 768 | 81 | hsa-miR-30c-5p | [PIGA](http://www.ncbi.nlm.nih.gov/entrez/query.fcgi?db=gene&cmd=Retrieve&dopt=full_report&list_uids=5277) | phosphatidylinositol glycan anchor biosynthesis class A |
| [Details](http://mirdb.org/cgi-bin/target_detail.cgi?targetID=1440779) | 769 | 81 | hsa-miR-30c-5p | [USP2](http://www.ncbi.nlm.nih.gov/entrez/query.fcgi?db=gene&cmd=Retrieve&dopt=full_report&list_uids=9099) | ubiquitin specific peptidase 2 |
| [Details](http://mirdb.org/cgi-bin/target_detail.cgi?targetID=1440795) | 770 | 81 | hsa-miR-30c-5p | [PLA2G12A](http://www.ncbi.nlm.nih.gov/entrez/query.fcgi?db=gene&cmd=Retrieve&dopt=full_report&list_uids=81579) | phospholipase A2 group XIIA |
| [Details](http://mirdb.org/cgi-bin/target_detail.cgi?targetID=1440814) | 771 | 81 | hsa-miR-30c-5p | [P4HA1](http://www.ncbi.nlm.nih.gov/entrez/query.fcgi?db=gene&cmd=Retrieve&dopt=full_report&list_uids=5033) | prolyl 4-hydroxylase subunit alpha 1 |
| [Details](http://mirdb.org/cgi-bin/target_detail.cgi?targetID=1440847) | 772 | 81 | hsa-miR-30c-5p | [TRPM7](http://www.ncbi.nlm.nih.gov/entrez/query.fcgi?db=gene&cmd=Retrieve&dopt=full_report&list_uids=54822) | transient receptor potential cation channel subfamily M member 7 |
| [Details](http://mirdb.org/cgi-bin/target_detail.cgi?targetID=1440868) | 773 | 81 | hsa-miR-30c-5p | [C14orf28](http://www.ncbi.nlm.nih.gov/entrez/query.fcgi?db=gene&cmd=Retrieve&dopt=full_report&list_uids=122525) | chromosome 14 open reading frame 28 |
| [Details](http://mirdb.org/cgi-bin/target_detail.cgi?targetID=1440934) | 774 | 81 | hsa-miR-30c-5p | [EPC2](http://www.ncbi.nlm.nih.gov/entrez/query.fcgi?db=gene&cmd=Retrieve&dopt=full_report&list_uids=26122) | enhancer of polycomb homolog 2 |
| [Details](http://mirdb.org/cgi-bin/target_detail.cgi?targetID=1439458) | 775 | 80 | hsa-miR-30c-5p | [SLC6A3](http://www.ncbi.nlm.nih.gov/entrez/query.fcgi?db=gene&cmd=Retrieve&dopt=full_report&list_uids=6531) | solute carrier family 6 member 3 |
| [Details](http://mirdb.org/cgi-bin/target_detail.cgi?targetID=1439464) | 776 | 80 | hsa-miR-30c-5p | [FIGN](http://www.ncbi.nlm.nih.gov/entrez/query.fcgi?db=gene&cmd=Retrieve&dopt=full_report&list_uids=55137) | fidgetin, microtubule severing factor |
| [Details](http://mirdb.org/cgi-bin/target_detail.cgi?targetID=1439603) | 777 | 80 | hsa-miR-30c-5p | [GOT2](http://www.ncbi.nlm.nih.gov/entrez/query.fcgi?db=gene&cmd=Retrieve&dopt=full_report&list_uids=2806) | glutamic-oxaloacetic transaminase 2 |
| [Details](http://mirdb.org/cgi-bin/target_detail.cgi?targetID=1439688) | 778 | 80 | hsa-miR-30c-5p | [DIO2](http://www.ncbi.nlm.nih.gov/entrez/query.fcgi?db=gene&cmd=Retrieve&dopt=full_report&list_uids=1734) | iodothyronine deiodinase 2 |
| [Details](http://mirdb.org/cgi-bin/target_detail.cgi?targetID=1439708) | 779 | 80 | hsa-miR-30c-5p | [CRB1](http://www.ncbi.nlm.nih.gov/entrez/query.fcgi?db=gene&cmd=Retrieve&dopt=full_report&list_uids=23418) | crumbs cell polarity complex component 1 |
| [Details](http://mirdb.org/cgi-bin/target_detail.cgi?targetID=1439710) | 780 | 80 | hsa-miR-30c-5p | [TMEM121](http://www.ncbi.nlm.nih.gov/entrez/query.fcgi?db=gene&cmd=Retrieve&dopt=full_report&list_uids=80757) | transmembrane protein 121 |
| [Details](http://mirdb.org/cgi-bin/target_detail.cgi?targetID=1439752) | 781 | 80 | hsa-miR-30c-5p | [SPART](http://www.ncbi.nlm.nih.gov/entrez/query.fcgi?db=gene&cmd=Retrieve&dopt=full_report&list_uids=23111) | spartin |
| [Details](http://mirdb.org/cgi-bin/target_detail.cgi?targetID=1439758) | 782 | 80 | hsa-miR-30c-5p | [NEDD4L](http://www.ncbi.nlm.nih.gov/entrez/query.fcgi?db=gene&cmd=Retrieve&dopt=full_report&list_uids=23327) | neural precursor cell expressed, developmentally down-regulated 4-like, E3 ubiquitin protein ligase |
| [Details](http://mirdb.org/cgi-bin/target_detail.cgi?targetID=1439901) | 783 | 80 | hsa-miR-30c-5p | [CTNND2](http://www.ncbi.nlm.nih.gov/entrez/query.fcgi?db=gene&cmd=Retrieve&dopt=full_report&list_uids=1501) | catenin delta 2 |
| [Details](http://mirdb.org/cgi-bin/target_detail.cgi?targetID=1439914) | 784 | 80 | hsa-miR-30c-5p | [ADGRL3](http://www.ncbi.nlm.nih.gov/entrez/query.fcgi?db=gene&cmd=Retrieve&dopt=full_report&list_uids=23284) | adhesion G protein-coupled receptor L3 |
| [Details](http://mirdb.org/cgi-bin/target_detail.cgi?targetID=1439941) | 785 | 80 | hsa-miR-30c-5p | [CECR2](http://www.ncbi.nlm.nih.gov/entrez/query.fcgi?db=gene&cmd=Retrieve&dopt=full_report&list_uids=27443) | CECR2, histone acetyl-lysine reader |
| [Details](http://mirdb.org/cgi-bin/target_detail.cgi?targetID=1440046) | 786 | 80 | hsa-miR-30c-5p | [NRXN3](http://www.ncbi.nlm.nih.gov/entrez/query.fcgi?db=gene&cmd=Retrieve&dopt=full_report&list_uids=9369) | neurexin 3 |
| [Details](http://mirdb.org/cgi-bin/target_detail.cgi?targetID=1440061) | 787 | 80 | hsa-miR-30c-5p | [DDHD2](http://www.ncbi.nlm.nih.gov/entrez/query.fcgi?db=gene&cmd=Retrieve&dopt=full_report&list_uids=23259) | DDHD domain containing 2 |
| [Details](http://mirdb.org/cgi-bin/target_detail.cgi?targetID=1440076) | 788 | 80 | hsa-miR-30c-5p | [KRAS](http://www.ncbi.nlm.nih.gov/entrez/query.fcgi?db=gene&cmd=Retrieve&dopt=full_report&list_uids=3845) | KRAS proto-oncogene, GTPase |
| [Details](http://mirdb.org/cgi-bin/target_detail.cgi?targetID=1440148) | 789 | 80 | hsa-miR-30c-5p | [SLC39A10](http://www.ncbi.nlm.nih.gov/entrez/query.fcgi?db=gene&cmd=Retrieve&dopt=full_report&list_uids=57181) | solute carrier family 39 member 10 |
| [Details](http://mirdb.org/cgi-bin/target_detail.cgi?targetID=1440185) | 790 | 80 | hsa-miR-30c-5p | [ERICH3](http://www.ncbi.nlm.nih.gov/entrez/query.fcgi?db=gene&cmd=Retrieve&dopt=full_report&list_uids=127254) | glutamate rich 3 |
| [Details](http://mirdb.org/cgi-bin/target_detail.cgi?targetID=1440225) | 791 | 80 | hsa-miR-30c-5p | [SCEL](http://www.ncbi.nlm.nih.gov/entrez/query.fcgi?db=gene&cmd=Retrieve&dopt=full_report&list_uids=8796) | sciellin |
| [Details](http://mirdb.org/cgi-bin/target_detail.cgi?targetID=1440267) | 792 | 80 | hsa-miR-30c-5p | [KCTD5](http://www.ncbi.nlm.nih.gov/entrez/query.fcgi?db=gene&cmd=Retrieve&dopt=full_report&list_uids=54442) | potassium channel tetramerization domain containing 5 |
| [Details](http://mirdb.org/cgi-bin/target_detail.cgi?targetID=1440322) | 793 | 80 | hsa-miR-30c-5p | [TRAF3](http://www.ncbi.nlm.nih.gov/entrez/query.fcgi?db=gene&cmd=Retrieve&dopt=full_report&list_uids=7187) | TNF receptor associated factor 3 |
| [Details](http://mirdb.org/cgi-bin/target_detail.cgi?targetID=1440378) | 794 | 80 | hsa-miR-30c-5p | [SBF1](http://www.ncbi.nlm.nih.gov/entrez/query.fcgi?db=gene&cmd=Retrieve&dopt=full_report&list_uids=6305) | SET binding factor 1 |
| [Details](http://mirdb.org/cgi-bin/target_detail.cgi?targetID=1440380) | 795 | 80 | hsa-miR-30c-5p | [FAM49A](http://www.ncbi.nlm.nih.gov/entrez/query.fcgi?db=gene&cmd=Retrieve&dopt=full_report&list_uids=81553) | family with sequence similarity 49 member A |
| [Details](http://mirdb.org/cgi-bin/target_detail.cgi?targetID=1440460) | 796 | 80 | hsa-miR-30c-5p | [ZBED1](http://www.ncbi.nlm.nih.gov/entrez/query.fcgi?db=gene&cmd=Retrieve&dopt=full_report&list_uids=9189) | zinc finger BED-type containing 1 |
| [Details](http://mirdb.org/cgi-bin/target_detail.cgi?targetID=1440479) | 797 | 80 | hsa-miR-30c-5p | [RASGEF1B](http://www.ncbi.nlm.nih.gov/entrez/query.fcgi?db=gene&cmd=Retrieve&dopt=full_report&list_uids=153020) | RasGEF domain family member 1B |
| [Details](http://mirdb.org/cgi-bin/target_detail.cgi?targetID=1440487) | 798 | 80 | hsa-miR-30c-5p | [NR3C1](http://www.ncbi.nlm.nih.gov/entrez/query.fcgi?db=gene&cmd=Retrieve&dopt=full_report&list_uids=2908) | nuclear receptor subfamily 3 group C member 1 |
| [Details](http://mirdb.org/cgi-bin/target_detail.cgi?targetID=1440536) | 799 | 80 | hsa-miR-30c-5p | [HTRA3](http://www.ncbi.nlm.nih.gov/entrez/query.fcgi?db=gene&cmd=Retrieve&dopt=full_report&list_uids=94031) | HtrA serine peptidase 3 |
| [Details](http://mirdb.org/cgi-bin/target_detail.cgi?targetID=1440579) | 800 | 80 | hsa-miR-30c-5p | [KCTD16](http://www.ncbi.nlm.nih.gov/entrez/query.fcgi?db=gene&cmd=Retrieve&dopt=full_report&list_uids=57528) | potassium channel tetramerization domain containing 16 |
| [Details](http://mirdb.org/cgi-bin/target_detail.cgi?targetID=1440601) | 801 | 80 | hsa-miR-30c-5p | [NAPG](http://www.ncbi.nlm.nih.gov/entrez/query.fcgi?db=gene&cmd=Retrieve&dopt=full_report&list_uids=8774) | NSF attachment protein gamma |
| [Details](http://mirdb.org/cgi-bin/target_detail.cgi?targetID=1440623) | 802 | 80 | hsa-miR-30c-5p | [KIAA1549](http://www.ncbi.nlm.nih.gov/entrez/query.fcgi?db=gene&cmd=Retrieve&dopt=full_report&list_uids=57670) | KIAA1549 |
| [Details](http://mirdb.org/cgi-bin/target_detail.cgi?targetID=1440679) | 803 | 80 | hsa-miR-30c-5p | [ZNF529](http://www.ncbi.nlm.nih.gov/entrez/query.fcgi?db=gene&cmd=Retrieve&dopt=full_report&list_uids=57711) | zinc finger protein 529 |
| [Details](http://mirdb.org/cgi-bin/target_detail.cgi?targetID=1440733) | 804 | 80 | hsa-miR-30c-5p | [ELAVL4](http://www.ncbi.nlm.nih.gov/entrez/query.fcgi?db=gene&cmd=Retrieve&dopt=full_report&list_uids=1996) | ELAV like RNA binding protein 4 |
| [Details](http://mirdb.org/cgi-bin/target_detail.cgi?targetID=1440758) | 805 | 80 | hsa-miR-30c-5p | [PCDH10](http://www.ncbi.nlm.nih.gov/entrez/query.fcgi?db=gene&cmd=Retrieve&dopt=full_report&list_uids=57575) | protocadherin 10 |
| [Details](http://mirdb.org/cgi-bin/target_detail.cgi?targetID=1440792) | 806 | 80 | hsa-miR-30c-5p | [KIAA1522](http://www.ncbi.nlm.nih.gov/entrez/query.fcgi?db=gene&cmd=Retrieve&dopt=full_report&list_uids=57648) | KIAA1522 |
| [Details](http://mirdb.org/cgi-bin/target_detail.cgi?targetID=1440813) | 807 | 80 | hsa-miR-30c-5p | [MAP4K4](http://www.ncbi.nlm.nih.gov/entrez/query.fcgi?db=gene&cmd=Retrieve&dopt=full_report&list_uids=9448) | mitogen-activated protein kinase kinase kinase kinase 4 |
| [Details](http://mirdb.org/cgi-bin/target_detail.cgi?targetID=1440861) | 808 | 80 | hsa-miR-30c-5p | [DGKD](http://www.ncbi.nlm.nih.gov/entrez/query.fcgi?db=gene&cmd=Retrieve&dopt=full_report&list_uids=8527) | diacylglycerol kinase delta |
| [Details](http://mirdb.org/cgi-bin/target_detail.cgi?targetID=1440877) | 809 | 80 | hsa-miR-30c-5p | [PTPN2](http://www.ncbi.nlm.nih.gov/entrez/query.fcgi?db=gene&cmd=Retrieve&dopt=full_report&list_uids=5771) | protein tyrosine phosphatase, non-receptor type 2 |
| [Details](http://mirdb.org/cgi-bin/target_detail.cgi?targetID=1440879) | 810 | 80 | hsa-miR-30c-5p | [CA10](http://www.ncbi.nlm.nih.gov/entrez/query.fcgi?db=gene&cmd=Retrieve&dopt=full_report&list_uids=56934) | carbonic anhydrase 10 |
| [Details](http://mirdb.org/cgi-bin/target_detail.cgi?targetID=1440888) | 811 | 80 | hsa-miR-30c-5p | [PAX9](http://www.ncbi.nlm.nih.gov/entrez/query.fcgi?db=gene&cmd=Retrieve&dopt=full_report&list_uids=5083) | paired box 9 |
| [Details](http://mirdb.org/cgi-bin/target_detail.cgi?targetID=1440901) | 812 | 80 | hsa-miR-30c-5p | [ZNF678](http://www.ncbi.nlm.nih.gov/entrez/query.fcgi?db=gene&cmd=Retrieve&dopt=full_report&list_uids=339500) | zinc finger protein 678 |
| [Details](http://mirdb.org/cgi-bin/target_detail.cgi?targetID=1440971) | 813 | 80 | hsa-miR-30c-5p | [ATAD5](http://www.ncbi.nlm.nih.gov/entrez/query.fcgi?db=gene&cmd=Retrieve&dopt=full_report&list_uids=79915) | ATPase family, AAA domain containing 5 |
| [Details](http://mirdb.org/cgi-bin/target_detail.cgi?targetID=1439463) | 814 | 79 | hsa-miR-30c-5p | [TNPO3](http://www.ncbi.nlm.nih.gov/entrez/query.fcgi?db=gene&cmd=Retrieve&dopt=full_report&list_uids=23534) | transportin 3 |
| [Details](http://mirdb.org/cgi-bin/target_detail.cgi?targetID=1439501) | 815 | 79 | hsa-miR-30c-5p | [METTL8](http://www.ncbi.nlm.nih.gov/entrez/query.fcgi?db=gene&cmd=Retrieve&dopt=full_report&list_uids=79828) | methyltransferase like 8 |
| [Details](http://mirdb.org/cgi-bin/target_detail.cgi?targetID=1439569) | 816 | 79 | hsa-miR-30c-5p | [STIMATE](http://www.ncbi.nlm.nih.gov/entrez/query.fcgi?db=gene&cmd=Retrieve&dopt=full_report&list_uids=375346) | STIM activating enhancer |
| [Details](http://mirdb.org/cgi-bin/target_detail.cgi?targetID=1439715) | 817 | 79 | hsa-miR-30c-5p | [ZCCHC24](http://www.ncbi.nlm.nih.gov/entrez/query.fcgi?db=gene&cmd=Retrieve&dopt=full_report&list_uids=219654) | zinc finger CCHC-type containing 24 |
| [Details](http://mirdb.org/cgi-bin/target_detail.cgi?targetID=1439788) | 818 | 79 | hsa-miR-30c-5p | [IER2](http://www.ncbi.nlm.nih.gov/entrez/query.fcgi?db=gene&cmd=Retrieve&dopt=full_report&list_uids=9592) | immediate early response 2 |
| [Details](http://mirdb.org/cgi-bin/target_detail.cgi?targetID=1439826) | 819 | 79 | hsa-miR-30c-5p | [AGO1](http://www.ncbi.nlm.nih.gov/entrez/query.fcgi?db=gene&cmd=Retrieve&dopt=full_report&list_uids=26523) | argonaute RISC catalytic component 1 |
| [Details](http://mirdb.org/cgi-bin/target_detail.cgi?targetID=1439866) | 820 | 79 | hsa-miR-30c-5p | [ZFP91](http://www.ncbi.nlm.nih.gov/entrez/query.fcgi?db=gene&cmd=Retrieve&dopt=full_report&list_uids=80829) | ZFP91 zinc finger protein |
| [Details](http://mirdb.org/cgi-bin/target_detail.cgi?targetID=1439879) | 821 | 79 | hsa-miR-30c-5p | [RASSF10](http://www.ncbi.nlm.nih.gov/entrez/query.fcgi?db=gene&cmd=Retrieve&dopt=full_report&list_uids=644943) | Ras association domain family member 10 |
| [Details](http://mirdb.org/cgi-bin/target_detail.cgi?targetID=1440089) | 822 | 79 | hsa-miR-30c-5p | [SLC7A6](http://www.ncbi.nlm.nih.gov/entrez/query.fcgi?db=gene&cmd=Retrieve&dopt=full_report&list_uids=9057) | solute carrier family 7 member 6 |
| [Details](http://mirdb.org/cgi-bin/target_detail.cgi?targetID=1440097) | 823 | 79 | hsa-miR-30c-5p | [MDM4](http://www.ncbi.nlm.nih.gov/entrez/query.fcgi?db=gene&cmd=Retrieve&dopt=full_report&list_uids=4194) | MDM4, p53 regulator |
| [Details](http://mirdb.org/cgi-bin/target_detail.cgi?targetID=1440249) | 824 | 79 | hsa-miR-30c-5p | [RPS6KA2](http://www.ncbi.nlm.nih.gov/entrez/query.fcgi?db=gene&cmd=Retrieve&dopt=full_report&list_uids=6196) | ribosomal protein S6 kinase A2 |
| [Details](http://mirdb.org/cgi-bin/target_detail.cgi?targetID=1440253) | 825 | 79 | hsa-miR-30c-5p | [USP45](http://www.ncbi.nlm.nih.gov/entrez/query.fcgi?db=gene&cmd=Retrieve&dopt=full_report&list_uids=85015) | ubiquitin specific peptidase 45 |
| [Details](http://mirdb.org/cgi-bin/target_detail.cgi?targetID=1440261) | 826 | 79 | hsa-miR-30c-5p | [IFNLR1](http://www.ncbi.nlm.nih.gov/entrez/query.fcgi?db=gene&cmd=Retrieve&dopt=full_report&list_uids=163702) | interferon lambda receptor 1 |
| [Details](http://mirdb.org/cgi-bin/target_detail.cgi?targetID=1440264) | 827 | 79 | hsa-miR-30c-5p | [ZNF652](http://www.ncbi.nlm.nih.gov/entrez/query.fcgi?db=gene&cmd=Retrieve&dopt=full_report&list_uids=22834) | zinc finger protein 652 |
| [Details](http://mirdb.org/cgi-bin/target_detail.cgi?targetID=1440308) | 828 | 79 | hsa-miR-30c-5p | [AUNIP](http://www.ncbi.nlm.nih.gov/entrez/query.fcgi?db=gene&cmd=Retrieve&dopt=full_report&list_uids=79000) | aurora kinase A and ninein interacting protein |
| [Details](http://mirdb.org/cgi-bin/target_detail.cgi?targetID=1440316) | 829 | 79 | hsa-miR-30c-5p | [HTR1F](http://www.ncbi.nlm.nih.gov/entrez/query.fcgi?db=gene&cmd=Retrieve&dopt=full_report&list_uids=3355) | 5-hydroxytryptamine receptor 1F |
| [Details](http://mirdb.org/cgi-bin/target_detail.cgi?targetID=1440391) | 830 | 79 | hsa-miR-30c-5p | [LEPR](http://www.ncbi.nlm.nih.gov/entrez/query.fcgi?db=gene&cmd=Retrieve&dopt=full_report&list_uids=3953) | leptin receptor |
| [Details](http://mirdb.org/cgi-bin/target_detail.cgi?targetID=1440512) | 831 | 79 | hsa-miR-30c-5p | [SIK3](http://www.ncbi.nlm.nih.gov/entrez/query.fcgi?db=gene&cmd=Retrieve&dopt=full_report&list_uids=23387) | SIK family kinase 3 |
| [Details](http://mirdb.org/cgi-bin/target_detail.cgi?targetID=1440551) | 832 | 79 | hsa-miR-30c-5p | [CACNB4](http://www.ncbi.nlm.nih.gov/entrez/query.fcgi?db=gene&cmd=Retrieve&dopt=full_report&list_uids=785) | calcium voltage-gated channel auxiliary subunit beta 4 |
| [Details](http://mirdb.org/cgi-bin/target_detail.cgi?targetID=1440616) | 833 | 79 | hsa-miR-30c-5p | [AP2A1](http://www.ncbi.nlm.nih.gov/entrez/query.fcgi?db=gene&cmd=Retrieve&dopt=full_report&list_uids=160) | adaptor related protein complex 2 subunit alpha 1 |
| [Details](http://mirdb.org/cgi-bin/target_detail.cgi?targetID=1440653) | 834 | 79 | hsa-miR-30c-5p | [GANC](http://www.ncbi.nlm.nih.gov/entrez/query.fcgi?db=gene&cmd=Retrieve&dopt=full_report&list_uids=2595) | glucosidase alpha, neutral C |
| [Details](http://mirdb.org/cgi-bin/target_detail.cgi?targetID=1440718) | 835 | 79 | hsa-miR-30c-5p | [RARB](http://www.ncbi.nlm.nih.gov/entrez/query.fcgi?db=gene&cmd=Retrieve&dopt=full_report&list_uids=5915) | retinoic acid receptor beta |
| [Details](http://mirdb.org/cgi-bin/target_detail.cgi?targetID=1440719) | 836 | 79 | hsa-miR-30c-5p | [ASCC3](http://www.ncbi.nlm.nih.gov/entrez/query.fcgi?db=gene&cmd=Retrieve&dopt=full_report&list_uids=10973) | activating signal cointegrator 1 complex subunit 3 |
| [Details](http://mirdb.org/cgi-bin/target_detail.cgi?targetID=1440752) | 837 | 79 | hsa-miR-30c-5p | [MAGI2](http://www.ncbi.nlm.nih.gov/entrez/query.fcgi?db=gene&cmd=Retrieve&dopt=full_report&list_uids=9863) | membrane associated guanylate kinase, WW and PDZ domain containing 2 |
| [Details](http://mirdb.org/cgi-bin/target_detail.cgi?targetID=1440772) | 838 | 79 | hsa-miR-30c-5p | [CDHR1](http://www.ncbi.nlm.nih.gov/entrez/query.fcgi?db=gene&cmd=Retrieve&dopt=full_report&list_uids=92211) | cadherin related family member 1 |
| [Details](http://mirdb.org/cgi-bin/target_detail.cgi?targetID=1440787) | 839 | 79 | hsa-miR-30c-5p | [RABGAP1L](http://www.ncbi.nlm.nih.gov/entrez/query.fcgi?db=gene&cmd=Retrieve&dopt=full_report&list_uids=9910) | RAB GTPase activating protein 1 like |
| [Details](http://mirdb.org/cgi-bin/target_detail.cgi?targetID=1440833) | 840 | 79 | hsa-miR-30c-5p | [PTPN4](http://www.ncbi.nlm.nih.gov/entrez/query.fcgi?db=gene&cmd=Retrieve&dopt=full_report&list_uids=5775) | protein tyrosine phosphatase, non-receptor type 4 |
| [Details](http://mirdb.org/cgi-bin/target_detail.cgi?targetID=1440970) | 841 | 79 | hsa-miR-30c-5p | [KCNMB2](http://www.ncbi.nlm.nih.gov/entrez/query.fcgi?db=gene&cmd=Retrieve&dopt=full_report&list_uids=10242) | potassium calcium-activated channel subfamily M regulatory beta subunit 2 |
| [Details](http://mirdb.org/cgi-bin/target_detail.cgi?targetID=1440984) | 842 | 79 | hsa-miR-30c-5p | [DBF4](http://www.ncbi.nlm.nih.gov/entrez/query.fcgi?db=gene&cmd=Retrieve&dopt=full_report&list_uids=10926) | DBF4 zinc finger |
| [Details](http://mirdb.org/cgi-bin/target_detail.cgi?targetID=1440988) | 843 | 79 | hsa-miR-30c-5p | [SAMHD1](http://www.ncbi.nlm.nih.gov/entrez/query.fcgi?db=gene&cmd=Retrieve&dopt=full_report&list_uids=25939) | SAM and HD domain containing deoxynucleoside triphosphate triphosphohydrolase 1 |
| [Details](http://mirdb.org/cgi-bin/target_detail.cgi?targetID=1439467) | 844 | 78 | hsa-miR-30c-5p | [ARID4B](http://www.ncbi.nlm.nih.gov/entrez/query.fcgi?db=gene&cmd=Retrieve&dopt=full_report&list_uids=51742) | AT-rich interaction domain 4B |
| [Details](http://mirdb.org/cgi-bin/target_detail.cgi?targetID=1439483) | 845 | 78 | hsa-miR-30c-5p | [HCN1](http://www.ncbi.nlm.nih.gov/entrez/query.fcgi?db=gene&cmd=Retrieve&dopt=full_report&list_uids=348980) | hyperpolarization activated cyclic nucleotide gated potassium channel 1 |
| [Details](http://mirdb.org/cgi-bin/target_detail.cgi?targetID=1439553) | 846 | 78 | hsa-miR-30c-5p | [TPP2](http://www.ncbi.nlm.nih.gov/entrez/query.fcgi?db=gene&cmd=Retrieve&dopt=full_report&list_uids=7174) | tripeptidyl peptidase 2 |
| [Details](http://mirdb.org/cgi-bin/target_detail.cgi?targetID=1439591) | 847 | 78 | hsa-miR-30c-5p | [SLC1A2](http://www.ncbi.nlm.nih.gov/entrez/query.fcgi?db=gene&cmd=Retrieve&dopt=full_report&list_uids=6506) | solute carrier family 1 member 2 |
| [Details](http://mirdb.org/cgi-bin/target_detail.cgi?targetID=1439593) | 848 | 78 | hsa-miR-30c-5p | [VAV3](http://www.ncbi.nlm.nih.gov/entrez/query.fcgi?db=gene&cmd=Retrieve&dopt=full_report&list_uids=10451) | vav guanine nucleotide exchange factor 3 |
| [Details](http://mirdb.org/cgi-bin/target_detail.cgi?targetID=1439594) | 849 | 78 | hsa-miR-30c-5p | [ALDH2](http://www.ncbi.nlm.nih.gov/entrez/query.fcgi?db=gene&cmd=Retrieve&dopt=full_report&list_uids=217) | aldehyde dehydrogenase 2 family member |
| [Details](http://mirdb.org/cgi-bin/target_detail.cgi?targetID=1439733) | 850 | 78 | hsa-miR-30c-5p | [SLC35F4](http://www.ncbi.nlm.nih.gov/entrez/query.fcgi?db=gene&cmd=Retrieve&dopt=full_report&list_uids=341880) | solute carrier family 35 member F4 |
| [Details](http://mirdb.org/cgi-bin/target_detail.cgi?targetID=1439751) | 851 | 78 | hsa-miR-30c-5p | [SSR3](http://www.ncbi.nlm.nih.gov/entrez/query.fcgi?db=gene&cmd=Retrieve&dopt=full_report&list_uids=6747) | signal sequence receptor subunit 3 |
| [Details](http://mirdb.org/cgi-bin/target_detail.cgi?targetID=1439792) | 852 | 78 | hsa-miR-30c-5p | [ITSN1](http://www.ncbi.nlm.nih.gov/entrez/query.fcgi?db=gene&cmd=Retrieve&dopt=full_report&list_uids=6453) | intersectin 1 |
| [Details](http://mirdb.org/cgi-bin/target_detail.cgi?targetID=1439923) | 853 | 78 | hsa-miR-30c-5p | [CLCF1](http://www.ncbi.nlm.nih.gov/entrez/query.fcgi?db=gene&cmd=Retrieve&dopt=full_report&list_uids=23529) | cardiotrophin like cytokine factor 1 |
| [Details](http://mirdb.org/cgi-bin/target_detail.cgi?targetID=1439963) | 854 | 78 | hsa-miR-30c-5p | [UGT8](http://www.ncbi.nlm.nih.gov/entrez/query.fcgi?db=gene&cmd=Retrieve&dopt=full_report&list_uids=7368) | UDP glycosyltransferase 8 |
| [Details](http://mirdb.org/cgi-bin/target_detail.cgi?targetID=1439995) | 855 | 78 | hsa-miR-30c-5p | [SEL1L3](http://www.ncbi.nlm.nih.gov/entrez/query.fcgi?db=gene&cmd=Retrieve&dopt=full_report&list_uids=23231) | SEL1L family member 3 |
| [Details](http://mirdb.org/cgi-bin/target_detail.cgi?targetID=1440017) | 856 | 78 | hsa-miR-30c-5p | [RELL1](http://www.ncbi.nlm.nih.gov/entrez/query.fcgi?db=gene&cmd=Retrieve&dopt=full_report&list_uids=768211) | RELT like 1 |
| [Details](http://mirdb.org/cgi-bin/target_detail.cgi?targetID=1440025) | 857 | 78 | hsa-miR-30c-5p | [MAP3K12](http://www.ncbi.nlm.nih.gov/entrez/query.fcgi?db=gene&cmd=Retrieve&dopt=full_report&list_uids=7786) | mitogen-activated protein kinase kinase kinase 12 |
| [Details](http://mirdb.org/cgi-bin/target_detail.cgi?targetID=1440056) | 858 | 78 | hsa-miR-30c-5p | [GALNT3](http://www.ncbi.nlm.nih.gov/entrez/query.fcgi?db=gene&cmd=Retrieve&dopt=full_report&list_uids=2591) | polypeptide N-acetylgalactosaminyltransferase 3 |
| [Details](http://mirdb.org/cgi-bin/target_detail.cgi?targetID=1440088) | 859 | 78 | hsa-miR-30c-5p | [AP3S1](http://www.ncbi.nlm.nih.gov/entrez/query.fcgi?db=gene&cmd=Retrieve&dopt=full_report&list_uids=1176) | adaptor related protein complex 3 subunit sigma 1 |
| [Details](http://mirdb.org/cgi-bin/target_detail.cgi?targetID=1440146) | 860 | 78 | hsa-miR-30c-5p | [PRRT2](http://www.ncbi.nlm.nih.gov/entrez/query.fcgi?db=gene&cmd=Retrieve&dopt=full_report&list_uids=112476) | proline rich transmembrane protein 2 |
| [Details](http://mirdb.org/cgi-bin/target_detail.cgi?targetID=1440180) | 861 | 78 | hsa-miR-30c-5p | [MPL](http://www.ncbi.nlm.nih.gov/entrez/query.fcgi?db=gene&cmd=Retrieve&dopt=full_report&list_uids=4352) | MPL proto-oncogene, thrombopoietin receptor |
| [Details](http://mirdb.org/cgi-bin/target_detail.cgi?targetID=1440294) | 862 | 78 | hsa-miR-30c-5p | [M1AP](http://www.ncbi.nlm.nih.gov/entrez/query.fcgi?db=gene&cmd=Retrieve&dopt=full_report&list_uids=130951) | meiosis 1 associated protein |
| [Details](http://mirdb.org/cgi-bin/target_detail.cgi?targetID=1440304) | 863 | 78 | hsa-miR-30c-5p | [GORASP2](http://www.ncbi.nlm.nih.gov/entrez/query.fcgi?db=gene&cmd=Retrieve&dopt=full_report&list_uids=26003) | golgi reassembly stacking protein 2 |
| [Details](http://mirdb.org/cgi-bin/target_detail.cgi?targetID=1440315) | 864 | 78 | hsa-miR-30c-5p | [HNRNPC](http://www.ncbi.nlm.nih.gov/entrez/query.fcgi?db=gene&cmd=Retrieve&dopt=full_report&list_uids=3183) | heterogeneous nuclear ribonucleoprotein C (C1/C2) |
| [Details](http://mirdb.org/cgi-bin/target_detail.cgi?targetID=1440366) | 865 | 78 | hsa-miR-30c-5p | [RRN3](http://www.ncbi.nlm.nih.gov/entrez/query.fcgi?db=gene&cmd=Retrieve&dopt=full_report&list_uids=54700) | RRN3 homolog, RNA polymerase I transcription factor |
| [Details](http://mirdb.org/cgi-bin/target_detail.cgi?targetID=1440390) | 866 | 78 | hsa-miR-30c-5p | [ATP2B2](http://www.ncbi.nlm.nih.gov/entrez/query.fcgi?db=gene&cmd=Retrieve&dopt=full_report&list_uids=491) | ATPase plasma membrane Ca2+ transporting 2 |
| [Details](http://mirdb.org/cgi-bin/target_detail.cgi?targetID=1440422) | 867 | 78 | hsa-miR-30c-5p | [SLC22A5](http://www.ncbi.nlm.nih.gov/entrez/query.fcgi?db=gene&cmd=Retrieve&dopt=full_report&list_uids=6584) | solute carrier family 22 member 5 |
| [Details](http://mirdb.org/cgi-bin/target_detail.cgi?targetID=1440451) | 868 | 78 | hsa-miR-30c-5p | [GCNT2](http://www.ncbi.nlm.nih.gov/entrez/query.fcgi?db=gene&cmd=Retrieve&dopt=full_report&list_uids=2651) | glucosaminyl (N-acetyl) transferase 2 (I blood group) |
| [Details](http://mirdb.org/cgi-bin/target_detail.cgi?targetID=1440486) | 869 | 78 | hsa-miR-30c-5p | [QKI](http://www.ncbi.nlm.nih.gov/entrez/query.fcgi?db=gene&cmd=Retrieve&dopt=full_report&list_uids=9444) | QKI, KH domain containing RNA binding |
| [Details](http://mirdb.org/cgi-bin/target_detail.cgi?targetID=1440502) | 870 | 78 | hsa-miR-30c-5p | [ZCRB1](http://www.ncbi.nlm.nih.gov/entrez/query.fcgi?db=gene&cmd=Retrieve&dopt=full_report&list_uids=85437) | zinc finger CCHC-type and RNA binding motif containing 1 |
| [Details](http://mirdb.org/cgi-bin/target_detail.cgi?targetID=1440570) | 871 | 78 | hsa-miR-30c-5p | [GLCE](http://www.ncbi.nlm.nih.gov/entrez/query.fcgi?db=gene&cmd=Retrieve&dopt=full_report&list_uids=26035) | glucuronic acid epimerase |
| [Details](http://mirdb.org/cgi-bin/target_detail.cgi?targetID=1440577) | 872 | 78 | hsa-miR-30c-5p | [CEP112](http://www.ncbi.nlm.nih.gov/entrez/query.fcgi?db=gene&cmd=Retrieve&dopt=full_report&list_uids=201134) | centrosomal protein 112 |
| [Details](http://mirdb.org/cgi-bin/target_detail.cgi?targetID=1440633) | 873 | 78 | hsa-miR-30c-5p | [MTTP](http://www.ncbi.nlm.nih.gov/entrez/query.fcgi?db=gene&cmd=Retrieve&dopt=full_report&list_uids=4547) | microsomal triglyceride transfer protein |
| [Details](http://mirdb.org/cgi-bin/target_detail.cgi?targetID=1440657) | 874 | 78 | hsa-miR-30c-5p | [ARL4A](http://www.ncbi.nlm.nih.gov/entrez/query.fcgi?db=gene&cmd=Retrieve&dopt=full_report&list_uids=10124) | ADP ribosylation factor like GTPase 4A |
| [Details](http://mirdb.org/cgi-bin/target_detail.cgi?targetID=1440672) | 875 | 78 | hsa-miR-30c-5p | [SEMA6A](http://www.ncbi.nlm.nih.gov/entrez/query.fcgi?db=gene&cmd=Retrieve&dopt=full_report&list_uids=57556) | semaphorin 6A |
| [Details](http://mirdb.org/cgi-bin/target_detail.cgi?targetID=1440851) | 876 | 78 | hsa-miR-30c-5p | [SSX2IP](http://www.ncbi.nlm.nih.gov/entrez/query.fcgi?db=gene&cmd=Retrieve&dopt=full_report&list_uids=117178) | SSX family member 2 interacting protein |
| [Details](http://mirdb.org/cgi-bin/target_detail.cgi?targetID=1440881) | 877 | 78 | hsa-miR-30c-5p | [MICAL2](http://www.ncbi.nlm.nih.gov/entrez/query.fcgi?db=gene&cmd=Retrieve&dopt=full_report&list_uids=9645) | microtubule associated monooxygenase, calponin and LIM domain containing 2 |
| [Details](http://mirdb.org/cgi-bin/target_detail.cgi?targetID=1440944) | 878 | 78 | hsa-miR-30c-5p | [ZBTB46](http://www.ncbi.nlm.nih.gov/entrez/query.fcgi?db=gene&cmd=Retrieve&dopt=full_report&list_uids=140685) | zinc finger and BTB domain containing 46 |
| [Details](http://mirdb.org/cgi-bin/target_detail.cgi?targetID=1439530) | 879 | 77 | hsa-miR-30c-5p | [ABCD2](http://www.ncbi.nlm.nih.gov/entrez/query.fcgi?db=gene&cmd=Retrieve&dopt=full_report&list_uids=225) | ATP binding cassette subfamily D member 2 |
| [Details](http://mirdb.org/cgi-bin/target_detail.cgi?targetID=1439560) | 880 | 77 | hsa-miR-30c-5p | [MMP21](http://www.ncbi.nlm.nih.gov/entrez/query.fcgi?db=gene&cmd=Retrieve&dopt=full_report&list_uids=118856) | matrix metallopeptidase 21 |
| [Details](http://mirdb.org/cgi-bin/target_detail.cgi?targetID=1439567) | 881 | 77 | hsa-miR-30c-5p | [SLC36A1](http://www.ncbi.nlm.nih.gov/entrez/query.fcgi?db=gene&cmd=Retrieve&dopt=full_report&list_uids=206358) | solute carrier family 36 member 1 |
| [Details](http://mirdb.org/cgi-bin/target_detail.cgi?targetID=1439750) | 882 | 77 | hsa-miR-30c-5p | [SKIL](http://www.ncbi.nlm.nih.gov/entrez/query.fcgi?db=gene&cmd=Retrieve&dopt=full_report&list_uids=6498) | SKI like proto-oncogene |
| [Details](http://mirdb.org/cgi-bin/target_detail.cgi?targetID=1439779) | 883 | 77 | hsa-miR-30c-5p | [SGK3](http://www.ncbi.nlm.nih.gov/entrez/query.fcgi?db=gene&cmd=Retrieve&dopt=full_report&list_uids=23678) | serum/glucocorticoid regulated kinase family member 3 |
| [Details](http://mirdb.org/cgi-bin/target_detail.cgi?targetID=1439836) | 884 | 77 | hsa-miR-30c-5p | [ZNF197](http://www.ncbi.nlm.nih.gov/entrez/query.fcgi?db=gene&cmd=Retrieve&dopt=full_report&list_uids=10168) | zinc finger protein 197 |
| [Details](http://mirdb.org/cgi-bin/target_detail.cgi?targetID=1439863) | 885 | 77 | hsa-miR-30c-5p | [NKTR](http://www.ncbi.nlm.nih.gov/entrez/query.fcgi?db=gene&cmd=Retrieve&dopt=full_report&list_uids=4820) | natural killer cell triggering receptor |
| [Details](http://mirdb.org/cgi-bin/target_detail.cgi?targetID=1439938) | 886 | 77 | hsa-miR-30c-5p | [FXR1](http://www.ncbi.nlm.nih.gov/entrez/query.fcgi?db=gene&cmd=Retrieve&dopt=full_report&list_uids=8087) | FMR1 autosomal homolog 1 |
| [Details](http://mirdb.org/cgi-bin/target_detail.cgi?targetID=1439978) | 887 | 77 | hsa-miR-30c-5p | [POP1](http://www.ncbi.nlm.nih.gov/entrez/query.fcgi?db=gene&cmd=Retrieve&dopt=full_report&list_uids=10940) | POP1 homolog, ribonuclease P/MRP subunit |
| [Details](http://mirdb.org/cgi-bin/target_detail.cgi?targetID=1439991) | 888 | 77 | hsa-miR-30c-5p | [KDELC2](http://www.ncbi.nlm.nih.gov/entrez/query.fcgi?db=gene&cmd=Retrieve&dopt=full_report&list_uids=143888) | KDEL motif containing 2 |
| [Details](http://mirdb.org/cgi-bin/target_detail.cgi?targetID=1440052) | 889 | 77 | hsa-miR-30c-5p | [MBNL1](http://www.ncbi.nlm.nih.gov/entrez/query.fcgi?db=gene&cmd=Retrieve&dopt=full_report&list_uids=4154) | muscleblind like splicing regulator 1 |
| [Details](http://mirdb.org/cgi-bin/target_detail.cgi?targetID=1440087) | 890 | 77 | hsa-miR-30c-5p | [SORCS3](http://www.ncbi.nlm.nih.gov/entrez/query.fcgi?db=gene&cmd=Retrieve&dopt=full_report&list_uids=22986) | sortilin related VPS10 domain containing receptor 3 |
| [Details](http://mirdb.org/cgi-bin/target_detail.cgi?targetID=1440181) | 891 | 77 | hsa-miR-30c-5p | [PARP16](http://www.ncbi.nlm.nih.gov/entrez/query.fcgi?db=gene&cmd=Retrieve&dopt=full_report&list_uids=54956) | poly(ADP-ribose) polymerase family member 16 |
| [Details](http://mirdb.org/cgi-bin/target_detail.cgi?targetID=1440192) | 892 | 77 | hsa-miR-30c-5p | [RUBCNL](http://www.ncbi.nlm.nih.gov/entrez/query.fcgi?db=gene&cmd=Retrieve&dopt=full_report&list_uids=80183) | rubicon like autophagy enhancer |
| [Details](http://mirdb.org/cgi-bin/target_detail.cgi?targetID=1440245) | 893 | 77 | hsa-miR-30c-5p | [ELFN2](http://www.ncbi.nlm.nih.gov/entrez/query.fcgi?db=gene&cmd=Retrieve&dopt=full_report&list_uids=114794) | extracellular leucine rich repeat and fibronectin type III domain containing 2 |
| [Details](http://mirdb.org/cgi-bin/target_detail.cgi?targetID=1440474) | 894 | 77 | hsa-miR-30c-5p | [FAM78B](http://www.ncbi.nlm.nih.gov/entrez/query.fcgi?db=gene&cmd=Retrieve&dopt=full_report&list_uids=149297) | family with sequence similarity 78 member B |
| [Details](http://mirdb.org/cgi-bin/target_detail.cgi?targetID=1440563) | 895 | 77 | hsa-miR-30c-5p | [FAM219B](http://www.ncbi.nlm.nih.gov/entrez/query.fcgi?db=gene&cmd=Retrieve&dopt=full_report&list_uids=57184) | family with sequence similarity 219 member B |
| [Details](http://mirdb.org/cgi-bin/target_detail.cgi?targetID=1440690) | 896 | 77 | hsa-miR-30c-5p | [NRK](http://www.ncbi.nlm.nih.gov/entrez/query.fcgi?db=gene&cmd=Retrieve&dopt=full_report&list_uids=203447) | Nik related kinase |
| [Details](http://mirdb.org/cgi-bin/target_detail.cgi?targetID=1440755) | 897 | 77 | hsa-miR-30c-5p | [ELL](http://www.ncbi.nlm.nih.gov/entrez/query.fcgi?db=gene&cmd=Retrieve&dopt=full_report&list_uids=8178) | elongation factor for RNA polymerase II |
| [Details](http://mirdb.org/cgi-bin/target_detail.cgi?targetID=1440769) | 898 | 77 | hsa-miR-30c-5p | [HNRNPA2B1](http://www.ncbi.nlm.nih.gov/entrez/query.fcgi?db=gene&cmd=Retrieve&dopt=full_report&list_uids=3181) | heterogeneous nuclear ribonucleoprotein A2/B1 |
| [Details](http://mirdb.org/cgi-bin/target_detail.cgi?targetID=1440771) | 899 | 77 | hsa-miR-30c-5p | [TDRP](http://www.ncbi.nlm.nih.gov/entrez/query.fcgi?db=gene&cmd=Retrieve&dopt=full_report&list_uids=157695) | testis development related protein |
| [Details](http://mirdb.org/cgi-bin/target_detail.cgi?targetID=1440836) | 900 | 77 | hsa-miR-30c-5p | [UQCRB](http://www.ncbi.nlm.nih.gov/entrez/query.fcgi?db=gene&cmd=Retrieve&dopt=full_report&list_uids=7381) | ubiquinol-cytochrome c reductase binding protein |
| [Details](http://mirdb.org/cgi-bin/target_detail.cgi?targetID=1440848) | 901 | 77 | hsa-miR-30c-5p | [COL8A1](http://www.ncbi.nlm.nih.gov/entrez/query.fcgi?db=gene&cmd=Retrieve&dopt=full_report&list_uids=1295) | collagen type VIII alpha 1 chain |
| [Details](http://mirdb.org/cgi-bin/target_detail.cgi?targetID=1440935) | 902 | 77 | hsa-miR-30c-5p | [HELZ](http://www.ncbi.nlm.nih.gov/entrez/query.fcgi?db=gene&cmd=Retrieve&dopt=full_report&list_uids=9931) | helicase with zinc finger |
| [Details](http://mirdb.org/cgi-bin/target_detail.cgi?targetID=1440942) | 903 | 77 | hsa-miR-30c-5p | [GRHL1](http://www.ncbi.nlm.nih.gov/entrez/query.fcgi?db=gene&cmd=Retrieve&dopt=full_report&list_uids=29841) | grainyhead like transcription factor 1 |
| [Details](http://mirdb.org/cgi-bin/target_detail.cgi?targetID=1440951) | 904 | 77 | hsa-miR-30c-5p | [STK38L](http://www.ncbi.nlm.nih.gov/entrez/query.fcgi?db=gene&cmd=Retrieve&dopt=full_report&list_uids=23012) | serine/threonine kinase 38 like |
| [Details](http://mirdb.org/cgi-bin/target_detail.cgi?targetID=1439534) | 905 | 76 | hsa-miR-30c-5p | [ZMAT1](http://www.ncbi.nlm.nih.gov/entrez/query.fcgi?db=gene&cmd=Retrieve&dopt=full_report&list_uids=84460) | zinc finger matrin-type 1 |
| [Details](http://mirdb.org/cgi-bin/target_detail.cgi?targetID=1439538) | 906 | 76 | hsa-miR-30c-5p | [OR11A1](http://www.ncbi.nlm.nih.gov/entrez/query.fcgi?db=gene&cmd=Retrieve&dopt=full_report&list_uids=26531) | olfactory receptor family 11 subfamily A member 1 |
| [Details](http://mirdb.org/cgi-bin/target_detail.cgi?targetID=1439576) | 907 | 76 | hsa-miR-30c-5p | [HNRNPA3](http://www.ncbi.nlm.nih.gov/entrez/query.fcgi?db=gene&cmd=Retrieve&dopt=full_report&list_uids=220988) | heterogeneous nuclear ribonucleoprotein A3 |
| [Details](http://mirdb.org/cgi-bin/target_detail.cgi?targetID=1439660) | 908 | 76 | hsa-miR-30c-5p | [SALL4](http://www.ncbi.nlm.nih.gov/entrez/query.fcgi?db=gene&cmd=Retrieve&dopt=full_report&list_uids=57167) | spalt like transcription factor 4 |
| [Details](http://mirdb.org/cgi-bin/target_detail.cgi?targetID=1439678) | 909 | 76 | hsa-miR-30c-5p | [RNF169](http://www.ncbi.nlm.nih.gov/entrez/query.fcgi?db=gene&cmd=Retrieve&dopt=full_report&list_uids=254225) | ring finger protein 169 |
| [Details](http://mirdb.org/cgi-bin/target_detail.cgi?targetID=1439789) | 910 | 76 | hsa-miR-30c-5p | [IKZF2](http://www.ncbi.nlm.nih.gov/entrez/query.fcgi?db=gene&cmd=Retrieve&dopt=full_report&list_uids=22807) | IKAROS family zinc finger 2 |
| [Details](http://mirdb.org/cgi-bin/target_detail.cgi?targetID=1439844) | 911 | 76 | hsa-miR-30c-5p | [GALR1](http://www.ncbi.nlm.nih.gov/entrez/query.fcgi?db=gene&cmd=Retrieve&dopt=full_report&list_uids=2587) | galanin receptor 1 |
| [Details](http://mirdb.org/cgi-bin/target_detail.cgi?targetID=1439904) | 912 | 76 | hsa-miR-30c-5p | [CPEB4](http://www.ncbi.nlm.nih.gov/entrez/query.fcgi?db=gene&cmd=Retrieve&dopt=full_report&list_uids=80315) | cytoplasmic polyadenylation element binding protein 4 |
| [Details](http://mirdb.org/cgi-bin/target_detail.cgi?targetID=1439999) | 913 | 76 | hsa-miR-30c-5p | [MIGA2](http://www.ncbi.nlm.nih.gov/entrez/query.fcgi?db=gene&cmd=Retrieve&dopt=full_report&list_uids=84895) | mitoguardin 2 |
| [Details](http://mirdb.org/cgi-bin/target_detail.cgi?targetID=1440043) | 914 | 76 | hsa-miR-30c-5p | [ZSCAN29](http://www.ncbi.nlm.nih.gov/entrez/query.fcgi?db=gene&cmd=Retrieve&dopt=full_report&list_uids=146050) | zinc finger and SCAN domain containing 29 |
| [Details](http://mirdb.org/cgi-bin/target_detail.cgi?targetID=1440167) | 915 | 76 | hsa-miR-30c-5p | [TENT4B](http://www.ncbi.nlm.nih.gov/entrez/query.fcgi?db=gene&cmd=Retrieve&dopt=full_report&list_uids=64282) | terminal nucleotidyltransferase 4B |
| [Details](http://mirdb.org/cgi-bin/target_detail.cgi?targetID=1440271) | 916 | 76 | hsa-miR-30c-5p | [BCL6](http://www.ncbi.nlm.nih.gov/entrez/query.fcgi?db=gene&cmd=Retrieve&dopt=full_report&list_uids=604) | BCL6, transcription repressor |
| [Details](http://mirdb.org/cgi-bin/target_detail.cgi?targetID=1440325) | 917 | 76 | hsa-miR-30c-5p | [WNK3](http://www.ncbi.nlm.nih.gov/entrez/query.fcgi?db=gene&cmd=Retrieve&dopt=full_report&list_uids=65267) | WNK lysine deficient protein kinase 3 |
| [Details](http://mirdb.org/cgi-bin/target_detail.cgi?targetID=1440471) | 918 | 76 | hsa-miR-30c-5p | [FAM131B](http://www.ncbi.nlm.nih.gov/entrez/query.fcgi?db=gene&cmd=Retrieve&dopt=full_report&list_uids=9715) | family with sequence similarity 131 member B |
| [Details](http://mirdb.org/cgi-bin/target_detail.cgi?targetID=1440670) | 919 | 76 | hsa-miR-30c-5p | [SSBP2](http://www.ncbi.nlm.nih.gov/entrez/query.fcgi?db=gene&cmd=Retrieve&dopt=full_report&list_uids=23635) | single stranded DNA binding protein 2 |
| [Details](http://mirdb.org/cgi-bin/target_detail.cgi?targetID=1440689) | 920 | 76 | hsa-miR-30c-5p | [HERC3](http://www.ncbi.nlm.nih.gov/entrez/query.fcgi?db=gene&cmd=Retrieve&dopt=full_report&list_uids=8916) | HECT and RLD domain containing E3 ubiquitin protein ligase 3 |
| [Details](http://mirdb.org/cgi-bin/target_detail.cgi?targetID=1440714) | 921 | 76 | hsa-miR-30c-5p | [ARPC5](http://www.ncbi.nlm.nih.gov/entrez/query.fcgi?db=gene&cmd=Retrieve&dopt=full_report&list_uids=10092) | actin related protein 2/3 complex subunit 5 |
| [Details](http://mirdb.org/cgi-bin/target_detail.cgi?targetID=1440741) | 922 | 76 | hsa-miR-30c-5p | [PABPC1L2A](http://www.ncbi.nlm.nih.gov/entrez/query.fcgi?db=gene&cmd=Retrieve&dopt=full_report&list_uids=340529) | poly(A) binding protein cytoplasmic 1 like 2A |
| [Details](http://mirdb.org/cgi-bin/target_detail.cgi?targetID=1440825) | 923 | 76 | hsa-miR-30c-5p | [AMOTL2](http://www.ncbi.nlm.nih.gov/entrez/query.fcgi?db=gene&cmd=Retrieve&dopt=full_report&list_uids=51421) | angiomotin like 2 |
| [Details](http://mirdb.org/cgi-bin/target_detail.cgi?targetID=1440905) | 924 | 76 | hsa-miR-30c-5p | [CYSLTR1](http://www.ncbi.nlm.nih.gov/entrez/query.fcgi?db=gene&cmd=Retrieve&dopt=full_report&list_uids=10800) | cysteinyl leukotriene receptor 1 |
| [Details](http://mirdb.org/cgi-bin/target_detail.cgi?targetID=1440906) | 925 | 76 | hsa-miR-30c-5p | [KCTD20](http://www.ncbi.nlm.nih.gov/entrez/query.fcgi?db=gene&cmd=Retrieve&dopt=full_report&list_uids=222658) | potassium channel tetramerization domain containing 20 |
| [Details](http://mirdb.org/cgi-bin/target_detail.cgi?targetID=1440915) | 926 | 76 | hsa-miR-30c-5p | [PABPC1L2B](http://www.ncbi.nlm.nih.gov/entrez/query.fcgi?db=gene&cmd=Retrieve&dopt=full_report&list_uids=645974) | poly(A) binding protein cytoplasmic 1 like 2B |
| [Details](http://mirdb.org/cgi-bin/target_detail.cgi?targetID=1440924) | 927 | 76 | hsa-miR-30c-5p | [SLC35A5](http://www.ncbi.nlm.nih.gov/entrez/query.fcgi?db=gene&cmd=Retrieve&dopt=full_report&list_uids=55032) | solute carrier family 35 member A5 |
| [Details](http://mirdb.org/cgi-bin/target_detail.cgi?targetID=1440952) | 928 | 76 | hsa-miR-30c-5p | [RNF44](http://www.ncbi.nlm.nih.gov/entrez/query.fcgi?db=gene&cmd=Retrieve&dopt=full_report&list_uids=22838) | ring finger protein 44 |
| [Details](http://mirdb.org/cgi-bin/target_detail.cgi?targetID=1440953) | 929 | 76 | hsa-miR-30c-5p | [TRMT5](http://www.ncbi.nlm.nih.gov/entrez/query.fcgi?db=gene&cmd=Retrieve&dopt=full_report&list_uids=57570) | tRNA methyltransferase 5 |
| [Details](http://mirdb.org/cgi-bin/target_detail.cgi?targetID=1439485) | 930 | 75 | hsa-miR-30c-5p | [PLAG1](http://www.ncbi.nlm.nih.gov/entrez/query.fcgi?db=gene&cmd=Retrieve&dopt=full_report&list_uids=5324) | PLAG1 zinc finger |
| [Details](http://mirdb.org/cgi-bin/target_detail.cgi?targetID=1439524) | 931 | 75 | hsa-miR-30c-5p | [BTBD10](http://www.ncbi.nlm.nih.gov/entrez/query.fcgi?db=gene&cmd=Retrieve&dopt=full_report&list_uids=84280) | BTB domain containing 10 |
| [Details](http://mirdb.org/cgi-bin/target_detail.cgi?targetID=1439575) | 932 | 75 | hsa-miR-30c-5p | [PLXNA2](http://www.ncbi.nlm.nih.gov/entrez/query.fcgi?db=gene&cmd=Retrieve&dopt=full_report&list_uids=5362) | plexin A2 |
| [Details](http://mirdb.org/cgi-bin/target_detail.cgi?targetID=1439631) | 933 | 75 | hsa-miR-30c-5p | [KPNA3](http://www.ncbi.nlm.nih.gov/entrez/query.fcgi?db=gene&cmd=Retrieve&dopt=full_report&list_uids=3839) | karyopherin subunit alpha 3 |
| [Details](http://mirdb.org/cgi-bin/target_detail.cgi?targetID=1439671) | 934 | 75 | hsa-miR-30c-5p | [FAM71F2](http://www.ncbi.nlm.nih.gov/entrez/query.fcgi?db=gene&cmd=Retrieve&dopt=full_report&list_uids=346653) | family with sequence similarity 71 member F2 |
| [Details](http://mirdb.org/cgi-bin/target_detail.cgi?targetID=1439712) | 935 | 75 | hsa-miR-30c-5p | [TET1](http://www.ncbi.nlm.nih.gov/entrez/query.fcgi?db=gene&cmd=Retrieve&dopt=full_report&list_uids=80312) | tet methylcytosine dioxygenase 1 |
| [Details](http://mirdb.org/cgi-bin/target_detail.cgi?targetID=1439954) | 936 | 75 | hsa-miR-30c-5p | [EYA2](http://www.ncbi.nlm.nih.gov/entrez/query.fcgi?db=gene&cmd=Retrieve&dopt=full_report&list_uids=2139) | EYA transcriptional coactivator and phosphatase 2 |
| [Details](http://mirdb.org/cgi-bin/target_detail.cgi?targetID=1439994) | 937 | 75 | hsa-miR-30c-5p | [WDR26](http://www.ncbi.nlm.nih.gov/entrez/query.fcgi?db=gene&cmd=Retrieve&dopt=full_report&list_uids=80232) | WD repeat domain 26 |
| [Details](http://mirdb.org/cgi-bin/target_detail.cgi?targetID=1440008) | 938 | 75 | hsa-miR-30c-5p | [PPFIA2](http://www.ncbi.nlm.nih.gov/entrez/query.fcgi?db=gene&cmd=Retrieve&dopt=full_report&list_uids=8499) | PTPRF interacting protein alpha 2 |
| [Details](http://mirdb.org/cgi-bin/target_detail.cgi?targetID=1440015) | 939 | 75 | hsa-miR-30c-5p | [PPP1R14C](http://www.ncbi.nlm.nih.gov/entrez/query.fcgi?db=gene&cmd=Retrieve&dopt=full_report&list_uids=81706) | protein phosphatase 1 regulatory inhibitor subunit 14C |
| [Details](http://mirdb.org/cgi-bin/target_detail.cgi?targetID=1440022) | 940 | 75 | hsa-miR-30c-5p | [DTD2](http://www.ncbi.nlm.nih.gov/entrez/query.fcgi?db=gene&cmd=Retrieve&dopt=full_report&list_uids=112487) | D-tyrosyl-tRNA deacylase 2 (putative) |
| [Details](http://mirdb.org/cgi-bin/target_detail.cgi?targetID=1440032) | 941 | 75 | hsa-miR-30c-5p | [TMEM121B](http://www.ncbi.nlm.nih.gov/entrez/query.fcgi?db=gene&cmd=Retrieve&dopt=full_report&list_uids=27439) | transmembrane protein 121B |
| [Details](http://mirdb.org/cgi-bin/target_detail.cgi?targetID=1440156) | 942 | 75 | hsa-miR-30c-5p | [HOXA13](http://www.ncbi.nlm.nih.gov/entrez/query.fcgi?db=gene&cmd=Retrieve&dopt=full_report&list_uids=3209) | homeobox A13 |
| [Details](http://mirdb.org/cgi-bin/target_detail.cgi?targetID=1440171) | 943 | 75 | hsa-miR-30c-5p | [AFF3](http://www.ncbi.nlm.nih.gov/entrez/query.fcgi?db=gene&cmd=Retrieve&dopt=full_report&list_uids=3899) | AF4/FMR2 family member 3 |
| [Details](http://mirdb.org/cgi-bin/target_detail.cgi?targetID=1440220) | 944 | 75 | hsa-miR-30c-5p | [LNPK](http://www.ncbi.nlm.nih.gov/entrez/query.fcgi?db=gene&cmd=Retrieve&dopt=full_report&list_uids=80856) | lunapark, ER junction formation factor |
| [Details](http://mirdb.org/cgi-bin/target_detail.cgi?targetID=1440277) | 945 | 75 | hsa-miR-30c-5p | [SLC6A9](http://www.ncbi.nlm.nih.gov/entrez/query.fcgi?db=gene&cmd=Retrieve&dopt=full_report&list_uids=6536) | solute carrier family 6 member 9 |
| [Details](http://mirdb.org/cgi-bin/target_detail.cgi?targetID=1440306) | 946 | 75 | hsa-miR-30c-5p | [PHF6](http://www.ncbi.nlm.nih.gov/entrez/query.fcgi?db=gene&cmd=Retrieve&dopt=full_report&list_uids=84295) | PHD finger protein 6 |
| [Details](http://mirdb.org/cgi-bin/target_detail.cgi?targetID=1440319) | 947 | 75 | hsa-miR-30c-5p | [EDAR](http://www.ncbi.nlm.nih.gov/entrez/query.fcgi?db=gene&cmd=Retrieve&dopt=full_report&list_uids=10913) | ectodysplasin A receptor |
| [Details](http://mirdb.org/cgi-bin/target_detail.cgi?targetID=1440327) | 948 | 75 | hsa-miR-30c-5p | [FAM217B](http://www.ncbi.nlm.nih.gov/entrez/query.fcgi?db=gene&cmd=Retrieve&dopt=full_report&list_uids=63939) | family with sequence similarity 217 member B |
| [Details](http://mirdb.org/cgi-bin/target_detail.cgi?targetID=1440534) | 949 | 75 | hsa-miR-30c-5p | [PIRT](http://www.ncbi.nlm.nih.gov/entrez/query.fcgi?db=gene&cmd=Retrieve&dopt=full_report&list_uids=644139) | phosphoinositide interacting regulator of transient receptor potential channels |
| [Details](http://mirdb.org/cgi-bin/target_detail.cgi?targetID=1440591) | 950 | 75 | hsa-miR-30c-5p | [PELO](http://www.ncbi.nlm.nih.gov/entrez/query.fcgi?db=gene&cmd=Retrieve&dopt=full_report&list_uids=53918) | pelota mRNA surveillance and ribosome rescue factor |
| [Details](http://mirdb.org/cgi-bin/target_detail.cgi?targetID=1440817) | 951 | 75 | hsa-miR-30c-5p | [SIKE1](http://www.ncbi.nlm.nih.gov/entrez/query.fcgi?db=gene&cmd=Retrieve&dopt=full_report&list_uids=80143) | suppressor of IKBKE 1 |
| [Details](http://mirdb.org/cgi-bin/target_detail.cgi?targetID=1440865) | 952 | 75 | hsa-miR-30c-5p | [TRMT10C](http://www.ncbi.nlm.nih.gov/entrez/query.fcgi?db=gene&cmd=Retrieve&dopt=full_report&list_uids=54931) | tRNA methyltransferase 10C, mitochondrial RNase P subunit |
| [Details](http://mirdb.org/cgi-bin/target_detail.cgi?targetID=1439511) | 953 | 74 | hsa-miR-30c-5p | [KLF11](http://www.ncbi.nlm.nih.gov/entrez/query.fcgi?db=gene&cmd=Retrieve&dopt=full_report&list_uids=8462) | Kruppel like factor 11 |
| [Details](http://mirdb.org/cgi-bin/target_detail.cgi?targetID=1439518) | 954 | 74 | hsa-miR-30c-5p | [SNX29](http://www.ncbi.nlm.nih.gov/entrez/query.fcgi?db=gene&cmd=Retrieve&dopt=full_report&list_uids=92017) | sorting nexin 29 |
| [Details](http://mirdb.org/cgi-bin/target_detail.cgi?targetID=1439911) | 955 | 74 | hsa-miR-30c-5p | [CD80](http://www.ncbi.nlm.nih.gov/entrez/query.fcgi?db=gene&cmd=Retrieve&dopt=full_report&list_uids=941) | CD80 molecule |
| [Details](http://mirdb.org/cgi-bin/target_detail.cgi?targetID=1439915) | 956 | 74 | hsa-miR-30c-5p | [GXYLT1](http://www.ncbi.nlm.nih.gov/entrez/query.fcgi?db=gene&cmd=Retrieve&dopt=full_report&list_uids=283464) | glucoside xylosyltransferase 1 |
| [Details](http://mirdb.org/cgi-bin/target_detail.cgi?targetID=1439956) | 957 | 74 | hsa-miR-30c-5p | [B4GALT5](http://www.ncbi.nlm.nih.gov/entrez/query.fcgi?db=gene&cmd=Retrieve&dopt=full_report&list_uids=9334) | beta-1,4-galactosyltransferase 5 |
| [Details](http://mirdb.org/cgi-bin/target_detail.cgi?targetID=1440045) | 958 | 74 | hsa-miR-30c-5p | [IRS2](http://www.ncbi.nlm.nih.gov/entrez/query.fcgi?db=gene&cmd=Retrieve&dopt=full_report&list_uids=8660) | insulin receptor substrate 2 |
| [Details](http://mirdb.org/cgi-bin/target_detail.cgi?targetID=1440158) | 959 | 74 | hsa-miR-30c-5p | [RBFOX1](http://www.ncbi.nlm.nih.gov/entrez/query.fcgi?db=gene&cmd=Retrieve&dopt=full_report&list_uids=54715) | RNA binding fox-1 homolog 1 |
| [Details](http://mirdb.org/cgi-bin/target_detail.cgi?targetID=1440205) | 960 | 74 | hsa-miR-30c-5p | [RAB22A](http://www.ncbi.nlm.nih.gov/entrez/query.fcgi?db=gene&cmd=Retrieve&dopt=full_report&list_uids=57403) | RAB22A, member RAS oncogene family |
| [Details](http://mirdb.org/cgi-bin/target_detail.cgi?targetID=1440279) | 961 | 74 | hsa-miR-30c-5p | [DET1](http://www.ncbi.nlm.nih.gov/entrez/query.fcgi?db=gene&cmd=Retrieve&dopt=full_report&list_uids=55070) | DET1, COP1 ubiquitin ligase partner |
| [Details](http://mirdb.org/cgi-bin/target_detail.cgi?targetID=1440385) | 962 | 74 | hsa-miR-30c-5p | [EPHB2](http://www.ncbi.nlm.nih.gov/entrez/query.fcgi?db=gene&cmd=Retrieve&dopt=full_report&list_uids=2048) | EPH receptor B2 |
| [Details](http://mirdb.org/cgi-bin/target_detail.cgi?targetID=1440468) | 963 | 74 | hsa-miR-30c-5p | [ABCG5](http://www.ncbi.nlm.nih.gov/entrez/query.fcgi?db=gene&cmd=Retrieve&dopt=full_report&list_uids=64240) | ATP binding cassette subfamily G member 5 |
| [Details](http://mirdb.org/cgi-bin/target_detail.cgi?targetID=1440496) | 964 | 74 | hsa-miR-30c-5p | [DLD](http://www.ncbi.nlm.nih.gov/entrez/query.fcgi?db=gene&cmd=Retrieve&dopt=full_report&list_uids=1738) | dihydrolipoamide dehydrogenase |
| [Details](http://mirdb.org/cgi-bin/target_detail.cgi?targetID=1440652) | 965 | 74 | hsa-miR-30c-5p | [TRPC5OS](http://www.ncbi.nlm.nih.gov/entrez/query.fcgi?db=gene&cmd=Retrieve&dopt=full_report&list_uids=100329135) | TRPC5 opposite strand |
| [Details](http://mirdb.org/cgi-bin/target_detail.cgi?targetID=1440695) | 966 | 74 | hsa-miR-30c-5p | [RFTN2](http://www.ncbi.nlm.nih.gov/entrez/query.fcgi?db=gene&cmd=Retrieve&dopt=full_report&list_uids=130132) | raftlin family member 2 |
| [Details](http://mirdb.org/cgi-bin/target_detail.cgi?targetID=1440854) | 967 | 74 | hsa-miR-30c-5p | [MID2](http://www.ncbi.nlm.nih.gov/entrez/query.fcgi?db=gene&cmd=Retrieve&dopt=full_report&list_uids=11043) | midline 2 |
| [Details](http://mirdb.org/cgi-bin/target_detail.cgi?targetID=1440908) | 968 | 74 | hsa-miR-30c-5p | [PLCB4](http://www.ncbi.nlm.nih.gov/entrez/query.fcgi?db=gene&cmd=Retrieve&dopt=full_report&list_uids=5332) | phospholipase C beta 4 |
| [Details](http://mirdb.org/cgi-bin/target_detail.cgi?targetID=1439503) | 969 | 73 | hsa-miR-30c-5p | [EIF5](http://www.ncbi.nlm.nih.gov/entrez/query.fcgi?db=gene&cmd=Retrieve&dopt=full_report&list_uids=1983) | eukaryotic translation initiation factor 5 |
| [Details](http://mirdb.org/cgi-bin/target_detail.cgi?targetID=1439545) | 970 | 73 | hsa-miR-30c-5p | [UCP3](http://www.ncbi.nlm.nih.gov/entrez/query.fcgi?db=gene&cmd=Retrieve&dopt=full_report&list_uids=7352) | uncoupling protein 3 |
| [Details](http://mirdb.org/cgi-bin/target_detail.cgi?targetID=1439590) | 971 | 73 | hsa-miR-30c-5p | [TTPA](http://www.ncbi.nlm.nih.gov/entrez/query.fcgi?db=gene&cmd=Retrieve&dopt=full_report&list_uids=7274) | alpha tocopherol transfer protein |
| [Details](http://mirdb.org/cgi-bin/target_detail.cgi?targetID=1439640) | 972 | 73 | hsa-miR-30c-5p | [RCOR1](http://www.ncbi.nlm.nih.gov/entrez/query.fcgi?db=gene&cmd=Retrieve&dopt=full_report&list_uids=23186) | REST corepressor 1 |
| [Details](http://mirdb.org/cgi-bin/target_detail.cgi?targetID=1439641) | 973 | 73 | hsa-miR-30c-5p | [SLF2](http://www.ncbi.nlm.nih.gov/entrez/query.fcgi?db=gene&cmd=Retrieve&dopt=full_report&list_uids=55719) | SMC5-SMC6 complex localization factor 2 |
| [Details](http://mirdb.org/cgi-bin/target_detail.cgi?targetID=1439662) | 974 | 73 | hsa-miR-30c-5p | [MBNL2](http://www.ncbi.nlm.nih.gov/entrez/query.fcgi?db=gene&cmd=Retrieve&dopt=full_report&list_uids=10150) | muscleblind like splicing regulator 2 |
| [Details](http://mirdb.org/cgi-bin/target_detail.cgi?targetID=1439829) | 975 | 73 | hsa-miR-30c-5p | [ICK](http://www.ncbi.nlm.nih.gov/entrez/query.fcgi?db=gene&cmd=Retrieve&dopt=full_report&list_uids=22858) | intestinal cell kinase |
| [Details](http://mirdb.org/cgi-bin/target_detail.cgi?targetID=1439835) | 976 | 73 | hsa-miR-30c-5p | [FMC1](http://www.ncbi.nlm.nih.gov/entrez/query.fcgi?db=gene&cmd=Retrieve&dopt=full_report&list_uids=154791) | formation of mitochondrial complex V assembly factor 1 homolog |
| [Details](http://mirdb.org/cgi-bin/target_detail.cgi?targetID=1439895) | 977 | 73 | hsa-miR-30c-5p | [KIF21B](http://www.ncbi.nlm.nih.gov/entrez/query.fcgi?db=gene&cmd=Retrieve&dopt=full_report&list_uids=23046) | kinesin family member 21B |
| [Details](http://mirdb.org/cgi-bin/target_detail.cgi?targetID=1439920) | 978 | 73 | hsa-miR-30c-5p | [METAP2](http://www.ncbi.nlm.nih.gov/entrez/query.fcgi?db=gene&cmd=Retrieve&dopt=full_report&list_uids=10988) | methionyl aminopeptidase 2 |
| [Details](http://mirdb.org/cgi-bin/target_detail.cgi?targetID=1440007) | 979 | 73 | hsa-miR-30c-5p | [BCL2L15](http://www.ncbi.nlm.nih.gov/entrez/query.fcgi?db=gene&cmd=Retrieve&dopt=full_report&list_uids=440603) | BCL2 like 15 |
| [Details](http://mirdb.org/cgi-bin/target_detail.cgi?targetID=1440010) | 980 | 73 | hsa-miR-30c-5p | [STC1](http://www.ncbi.nlm.nih.gov/entrez/query.fcgi?db=gene&cmd=Retrieve&dopt=full_report&list_uids=6781) | stanniocalcin 1 |
| [Details](http://mirdb.org/cgi-bin/target_detail.cgi?targetID=1440028) | 981 | 73 | hsa-miR-30c-5p | [VPS13C](http://www.ncbi.nlm.nih.gov/entrez/query.fcgi?db=gene&cmd=Retrieve&dopt=full_report&list_uids=54832) | vacuolar protein sorting 13 homolog C |
| [Details](http://mirdb.org/cgi-bin/target_detail.cgi?targetID=1440083) | 982 | 73 | hsa-miR-30c-5p | [RANBP2](http://www.ncbi.nlm.nih.gov/entrez/query.fcgi?db=gene&cmd=Retrieve&dopt=full_report&list_uids=5903) | RAN binding protein 2 |
| [Details](http://mirdb.org/cgi-bin/target_detail.cgi?targetID=1440186) | 983 | 73 | hsa-miR-30c-5p | [GCSAM](http://www.ncbi.nlm.nih.gov/entrez/query.fcgi?db=gene&cmd=Retrieve&dopt=full_report&list_uids=257144) | germinal center associated signaling and motility |
| [Details](http://mirdb.org/cgi-bin/target_detail.cgi?targetID=1440187) | 984 | 73 | hsa-miR-30c-5p | [NACC2](http://www.ncbi.nlm.nih.gov/entrez/query.fcgi?db=gene&cmd=Retrieve&dopt=full_report&list_uids=138151) | NACC family member 2 |
| [Details](http://mirdb.org/cgi-bin/target_detail.cgi?targetID=1440214) | 985 | 73 | hsa-miR-30c-5p | [CDC73](http://www.ncbi.nlm.nih.gov/entrez/query.fcgi?db=gene&cmd=Retrieve&dopt=full_report&list_uids=79577) | cell division cycle 73 |
| [Details](http://mirdb.org/cgi-bin/target_detail.cgi?targetID=1440217) | 986 | 73 | hsa-miR-30c-5p | [HEPHL1](http://www.ncbi.nlm.nih.gov/entrez/query.fcgi?db=gene&cmd=Retrieve&dopt=full_report&list_uids=341208) | hephaestin like 1 |
| [Details](http://mirdb.org/cgi-bin/target_detail.cgi?targetID=1440412) | 987 | 73 | hsa-miR-30c-5p | [SETD3](http://www.ncbi.nlm.nih.gov/entrez/query.fcgi?db=gene&cmd=Retrieve&dopt=full_report&list_uids=84193) | SET domain containing 3, actin histidine methyltransferase |
| [Details](http://mirdb.org/cgi-bin/target_detail.cgi?targetID=1440433) | 988 | 73 | hsa-miR-30c-5p | [PRTG](http://www.ncbi.nlm.nih.gov/entrez/query.fcgi?db=gene&cmd=Retrieve&dopt=full_report&list_uids=283659) | protogenin |
| [Details](http://mirdb.org/cgi-bin/target_detail.cgi?targetID=1440511) | 989 | 73 | hsa-miR-30c-5p | [DIPK1A](http://www.ncbi.nlm.nih.gov/entrez/query.fcgi?db=gene&cmd=Retrieve&dopt=full_report&list_uids=388650) | divergent protein kinase domain 1A |
| [Details](http://mirdb.org/cgi-bin/target_detail.cgi?targetID=1440617) | 990 | 73 | hsa-miR-30c-5p | [ALS2](http://www.ncbi.nlm.nih.gov/entrez/query.fcgi?db=gene&cmd=Retrieve&dopt=full_report&list_uids=57679) | ALS2, alsin Rho guanine nucleotide exchange factor |
| [Details](http://mirdb.org/cgi-bin/target_detail.cgi?targetID=1440649) | 991 | 73 | hsa-miR-30c-5p | [CELF4](http://www.ncbi.nlm.nih.gov/entrez/query.fcgi?db=gene&cmd=Retrieve&dopt=full_report&list_uids=56853) | CUGBP Elav-like family member 4 |
| [Details](http://mirdb.org/cgi-bin/target_detail.cgi?targetID=1440713) | 992 | 73 | hsa-miR-30c-5p | [SLC7A11](http://www.ncbi.nlm.nih.gov/entrez/query.fcgi?db=gene&cmd=Retrieve&dopt=full_report&list_uids=23657) | solute carrier family 7 member 11 |
| [Details](http://mirdb.org/cgi-bin/target_detail.cgi?targetID=1440766) | 993 | 73 | hsa-miR-30c-5p | [NT5DC3](http://www.ncbi.nlm.nih.gov/entrez/query.fcgi?db=gene&cmd=Retrieve&dopt=full_report&list_uids=51559) | 5'-nucleotidase domain containing 3 |
| [Details](http://mirdb.org/cgi-bin/target_detail.cgi?targetID=1440809) | 994 | 73 | hsa-miR-30c-5p | [ASXL3](http://www.ncbi.nlm.nih.gov/entrez/query.fcgi?db=gene&cmd=Retrieve&dopt=full_report&list_uids=80816) | ASXL transcriptional regulator 3 |
| [Details](http://mirdb.org/cgi-bin/target_detail.cgi?targetID=1440959) | 995 | 73 | hsa-miR-30c-5p | [TMEM170A](http://www.ncbi.nlm.nih.gov/entrez/query.fcgi?db=gene&cmd=Retrieve&dopt=full_report&list_uids=124491) | transmembrane protein 170A |
| [Details](http://mirdb.org/cgi-bin/target_detail.cgi?targetID=1439616) | 996 | 72 | hsa-miR-30c-5p | [PDE5A](http://www.ncbi.nlm.nih.gov/entrez/query.fcgi?db=gene&cmd=Retrieve&dopt=full_report&list_uids=8654) | phosphodiesterase 5A |
| [Details](http://mirdb.org/cgi-bin/target_detail.cgi?targetID=1439666) | 997 | 72 | hsa-miR-30c-5p | [C14orf39](http://www.ncbi.nlm.nih.gov/entrez/query.fcgi?db=gene&cmd=Retrieve&dopt=full_report&list_uids=317761) | chromosome 14 open reading frame 39 |
| [Details](http://mirdb.org/cgi-bin/target_detail.cgi?targetID=1439781) | 998 | 72 | hsa-miR-30c-5p | [URGCP](http://www.ncbi.nlm.nih.gov/entrez/query.fcgi?db=gene&cmd=Retrieve&dopt=full_report&list_uids=55665) | upregulator of cell proliferation |
| [Details](http://mirdb.org/cgi-bin/target_detail.cgi?targetID=1439847) | 999 | 72 | hsa-miR-30c-5p | [POLDIP2](http://www.ncbi.nlm.nih.gov/entrez/query.fcgi?db=gene&cmd=Retrieve&dopt=full_report&list_uids=26073) | DNA polymerase delta interacting protein 2 |
| [Details](http://mirdb.org/cgi-bin/target_detail.cgi?targetID=1439872) | 1000 | 72 | hsa-miR-30c-5p | [PPIP5K2](http://www.ncbi.nlm.nih.gov/entrez/query.fcgi?db=gene&cmd=Retrieve&dopt=full_report&list_uids=23262) | diphosphoinositol pentakisphosphate kinase 2 |
| [Details](http://mirdb.org/cgi-bin/target_detail.cgi?targetID=1439946) | 1001 | 72 | hsa-miR-30c-5p | [ZNF597](http://www.ncbi.nlm.nih.gov/entrez/query.fcgi?db=gene&cmd=Retrieve&dopt=full_report&list_uids=146434) | zinc finger protein 597 |
| [Details](http://mirdb.org/cgi-bin/target_detail.cgi?targetID=1439988) | 1002 | 72 | hsa-miR-30c-5p | [GID4](http://www.ncbi.nlm.nih.gov/entrez/query.fcgi?db=gene&cmd=Retrieve&dopt=full_report&list_uids=79018) | GID complex subunit 4 homolog |
| [Details](http://mirdb.org/cgi-bin/target_detail.cgi?targetID=1440054) | 1003 | 72 | hsa-miR-30c-5p | [GAS2L3](http://www.ncbi.nlm.nih.gov/entrez/query.fcgi?db=gene&cmd=Retrieve&dopt=full_report&list_uids=283431) | growth arrest specific 2 like 3 |
| [Details](http://mirdb.org/cgi-bin/target_detail.cgi?targetID=1440074) | 1004 | 72 | hsa-miR-30c-5p | [TVP23B](http://www.ncbi.nlm.nih.gov/entrez/query.fcgi?db=gene&cmd=Retrieve&dopt=full_report&list_uids=51030) | trans-golgi network vesicle protein 23 homolog B |
| [Details](http://mirdb.org/cgi-bin/target_detail.cgi?targetID=1440287) | 1005 | 72 | hsa-miR-30c-5p | [SH3TC2](http://www.ncbi.nlm.nih.gov/entrez/query.fcgi?db=gene&cmd=Retrieve&dopt=full_report&list_uids=79628) | SH3 domain and tetratricopeptide repeats 2 |
| [Details](http://mirdb.org/cgi-bin/target_detail.cgi?targetID=1440307) | 1006 | 72 | hsa-miR-30c-5p | [ZBTB34](http://www.ncbi.nlm.nih.gov/entrez/query.fcgi?db=gene&cmd=Retrieve&dopt=full_report&list_uids=403341) | zinc finger and BTB domain containing 34 |
| [Details](http://mirdb.org/cgi-bin/target_detail.cgi?targetID=1440409) | 1007 | 72 | hsa-miR-30c-5p | [UHRF1BP1](http://www.ncbi.nlm.nih.gov/entrez/query.fcgi?db=gene&cmd=Retrieve&dopt=full_report&list_uids=54887) | UHRF1 binding protein 1 |
| [Details](http://mirdb.org/cgi-bin/target_detail.cgi?targetID=1440526) | 1008 | 72 | hsa-miR-30c-5p | [ZCCHC14](http://www.ncbi.nlm.nih.gov/entrez/query.fcgi?db=gene&cmd=Retrieve&dopt=full_report&list_uids=23174) | zinc finger CCHC-type containing 14 |
| [Details](http://mirdb.org/cgi-bin/target_detail.cgi?targetID=1440572) | 1009 | 72 | hsa-miR-30c-5p | [SPTLC3](http://www.ncbi.nlm.nih.gov/entrez/query.fcgi?db=gene&cmd=Retrieve&dopt=full_report&list_uids=55304) | serine palmitoyltransferase long chain base subunit 3 |
| [Details](http://mirdb.org/cgi-bin/target_detail.cgi?targetID=1440611) | 1010 | 72 | hsa-miR-30c-5p | [HOXA11](http://www.ncbi.nlm.nih.gov/entrez/query.fcgi?db=gene&cmd=Retrieve&dopt=full_report&list_uids=3207) | homeobox A11 |
| [Details](http://mirdb.org/cgi-bin/target_detail.cgi?targetID=1440632) | 1011 | 72 | hsa-miR-30c-5p | [PKNOX2](http://www.ncbi.nlm.nih.gov/entrez/query.fcgi?db=gene&cmd=Retrieve&dopt=full_report&list_uids=63876) | PBX/knotted 1 homeobox 2 |
| [Details](http://mirdb.org/cgi-bin/target_detail.cgi?targetID=1440678) | 1012 | 72 | hsa-miR-30c-5p | [ESPN](http://www.ncbi.nlm.nih.gov/entrez/query.fcgi?db=gene&cmd=Retrieve&dopt=full_report&list_uids=83715) | espin |
| [Details](http://mirdb.org/cgi-bin/target_detail.cgi?targetID=1440721) | 1013 | 72 | hsa-miR-30c-5p | [KLF14](http://www.ncbi.nlm.nih.gov/entrez/query.fcgi?db=gene&cmd=Retrieve&dopt=full_report&list_uids=136259) | Kruppel like factor 14 |
| [Details](http://mirdb.org/cgi-bin/target_detail.cgi?targetID=1440735) | 1014 | 72 | hsa-miR-30c-5p | [B4GALT6](http://www.ncbi.nlm.nih.gov/entrez/query.fcgi?db=gene&cmd=Retrieve&dopt=full_report&list_uids=9331) | beta-1,4-galactosyltransferase 6 |
| [Details](http://mirdb.org/cgi-bin/target_detail.cgi?targetID=1440760) | 1015 | 72 | hsa-miR-30c-5p | [MSANTD3-TMEFF1](http://www.ncbi.nlm.nih.gov/entrez/query.fcgi?db=gene&cmd=Retrieve&dopt=full_report&list_uids=100526694) | MSANTD3-TMEFF1 readthrough |
| [Details](http://mirdb.org/cgi-bin/target_detail.cgi?targetID=1440793) | 1016 | 72 | hsa-miR-30c-5p | [HERC2](http://www.ncbi.nlm.nih.gov/entrez/query.fcgi?db=gene&cmd=Retrieve&dopt=full_report&list_uids=8924) | HECT and RLD domain containing E3 ubiquitin protein ligase 2 |
| [Details](http://mirdb.org/cgi-bin/target_detail.cgi?targetID=1440857) | 1017 | 72 | hsa-miR-30c-5p | [SORBS2](http://www.ncbi.nlm.nih.gov/entrez/query.fcgi?db=gene&cmd=Retrieve&dopt=full_report&list_uids=8470) | sorbin and SH3 domain containing 2 |
| [Details](http://mirdb.org/cgi-bin/target_detail.cgi?targetID=1440860) | 1018 | 72 | hsa-miR-30c-5p | [LPCAT2](http://www.ncbi.nlm.nih.gov/entrez/query.fcgi?db=gene&cmd=Retrieve&dopt=full_report&list_uids=54947) | lysophosphatidylcholine acyltransferase 2 |
| [Details](http://mirdb.org/cgi-bin/target_detail.cgi?targetID=1440949) | 1019 | 72 | hsa-miR-30c-5p | [ADGRF1](http://www.ncbi.nlm.nih.gov/entrez/query.fcgi?db=gene&cmd=Retrieve&dopt=full_report&list_uids=266977) | adhesion G protein-coupled receptor F1 |
| [Details](http://mirdb.org/cgi-bin/target_detail.cgi?targetID=1440967) | 1020 | 72 | hsa-miR-30c-5p | [INSYN2](http://www.ncbi.nlm.nih.gov/entrez/query.fcgi?db=gene&cmd=Retrieve&dopt=full_report&list_uids=642938) | inhibitory synaptic factor 2A |
| [Details](http://mirdb.org/cgi-bin/target_detail.cgi?targetID=1439488) | 1021 | 71 | hsa-miR-30c-5p | [HOXB8](http://www.ncbi.nlm.nih.gov/entrez/query.fcgi?db=gene&cmd=Retrieve&dopt=full_report&list_uids=3218) | homeobox B8 |
| [Details](http://mirdb.org/cgi-bin/target_detail.cgi?targetID=1439496) | 1022 | 71 | hsa-miR-30c-5p | [IL1A](http://www.ncbi.nlm.nih.gov/entrez/query.fcgi?db=gene&cmd=Retrieve&dopt=full_report&list_uids=3552) | interleukin 1 alpha |
| [Details](http://mirdb.org/cgi-bin/target_detail.cgi?targetID=1439657) | 1023 | 71 | hsa-miR-30c-5p | [LSM14B](http://www.ncbi.nlm.nih.gov/entrez/query.fcgi?db=gene&cmd=Retrieve&dopt=full_report&list_uids=149986) | LSM family member 14B |
| [Details](http://mirdb.org/cgi-bin/target_detail.cgi?targetID=1439840) | 1024 | 71 | hsa-miR-30c-5p | [SEMA6D](http://www.ncbi.nlm.nih.gov/entrez/query.fcgi?db=gene&cmd=Retrieve&dopt=full_report&list_uids=80031) | semaphorin 6D |
| [Details](http://mirdb.org/cgi-bin/target_detail.cgi?targetID=1439855) | 1025 | 71 | hsa-miR-30c-5p | [E2F3](http://www.ncbi.nlm.nih.gov/entrez/query.fcgi?db=gene&cmd=Retrieve&dopt=full_report&list_uids=1871) | E2F transcription factor 3 |
| [Details](http://mirdb.org/cgi-bin/target_detail.cgi?targetID=1439880) | 1026 | 71 | hsa-miR-30c-5p | [MICAL1](http://www.ncbi.nlm.nih.gov/entrez/query.fcgi?db=gene&cmd=Retrieve&dopt=full_report&list_uids=64780) | microtubule associated monooxygenase, calponin and LIM domain containing 1 |
| [Details](http://mirdb.org/cgi-bin/target_detail.cgi?targetID=1440237) | 1027 | 71 | hsa-miR-30c-5p | [CFDP1](http://www.ncbi.nlm.nih.gov/entrez/query.fcgi?db=gene&cmd=Retrieve&dopt=full_report&list_uids=10428) | craniofacial development protein 1 |
| [Details](http://mirdb.org/cgi-bin/target_detail.cgi?targetID=1440240) | 1028 | 71 | hsa-miR-30c-5p | [HECW1](http://www.ncbi.nlm.nih.gov/entrez/query.fcgi?db=gene&cmd=Retrieve&dopt=full_report&list_uids=23072) | HECT, C2 and WW domain containing E3 ubiquitin protein ligase 1 |
| [Details](http://mirdb.org/cgi-bin/target_detail.cgi?targetID=1440337) | 1029 | 71 | hsa-miR-30c-5p | [SOS1](http://www.ncbi.nlm.nih.gov/entrez/query.fcgi?db=gene&cmd=Retrieve&dopt=full_report&list_uids=6654) | SOS Ras/Rac guanine nucleotide exchange factor 1 |
| [Details](http://mirdb.org/cgi-bin/target_detail.cgi?targetID=1440344) | 1030 | 71 | hsa-miR-30c-5p | [RALGAPB](http://www.ncbi.nlm.nih.gov/entrez/query.fcgi?db=gene&cmd=Retrieve&dopt=full_report&list_uids=57148) | Ral GTPase activating protein non-catalytic beta subunit |
| [Details](http://mirdb.org/cgi-bin/target_detail.cgi?targetID=1440494) | 1031 | 71 | hsa-miR-30c-5p | [DACH2](http://www.ncbi.nlm.nih.gov/entrez/query.fcgi?db=gene&cmd=Retrieve&dopt=full_report&list_uids=117154) | dachshund family transcription factor 2 |
| [Details](http://mirdb.org/cgi-bin/target_detail.cgi?targetID=1440588) | 1032 | 71 | hsa-miR-30c-5p | [CDC7](http://www.ncbi.nlm.nih.gov/entrez/query.fcgi?db=gene&cmd=Retrieve&dopt=full_report&list_uids=8317) | cell division cycle 7 |
| [Details](http://mirdb.org/cgi-bin/target_detail.cgi?targetID=1440693) | 1033 | 71 | hsa-miR-30c-5p | [SEC23IP](http://www.ncbi.nlm.nih.gov/entrez/query.fcgi?db=gene&cmd=Retrieve&dopt=full_report&list_uids=11196) | SEC23 interacting protein |
| [Details](http://mirdb.org/cgi-bin/target_detail.cgi?targetID=1440717) | 1034 | 71 | hsa-miR-30c-5p | [KCTD3](http://www.ncbi.nlm.nih.gov/entrez/query.fcgi?db=gene&cmd=Retrieve&dopt=full_report&list_uids=51133) | potassium channel tetramerization domain containing 3 |
| [Details](http://mirdb.org/cgi-bin/target_detail.cgi?targetID=1440740) | 1035 | 71 | hsa-miR-30c-5p | [ZNF547](http://www.ncbi.nlm.nih.gov/entrez/query.fcgi?db=gene&cmd=Retrieve&dopt=full_report&list_uids=284306) | zinc finger protein 547 |
| [Details](http://mirdb.org/cgi-bin/target_detail.cgi?targetID=1440798) | 1036 | 71 | hsa-miR-30c-5p | [NAGPA](http://www.ncbi.nlm.nih.gov/entrez/query.fcgi?db=gene&cmd=Retrieve&dopt=full_report&list_uids=51172) | N-acetylglucosamine-1-phosphodiester alpha-N-acetylglucosaminidase |
| [Details](http://mirdb.org/cgi-bin/target_detail.cgi?targetID=1440824) | 1037 | 71 | hsa-miR-30c-5p | [SGCB](http://www.ncbi.nlm.nih.gov/entrez/query.fcgi?db=gene&cmd=Retrieve&dopt=full_report&list_uids=6443) | sarcoglycan beta |
| [Details](http://mirdb.org/cgi-bin/target_detail.cgi?targetID=1440850) | 1038 | 71 | hsa-miR-30c-5p | [PPFIA1](http://www.ncbi.nlm.nih.gov/entrez/query.fcgi?db=gene&cmd=Retrieve&dopt=full_report&list_uids=8500) | PTPRF interacting protein alpha 1 |
| [Details](http://mirdb.org/cgi-bin/target_detail.cgi?targetID=1439453) | 1039 | 70 | hsa-miR-30c-5p | [MYH10](http://www.ncbi.nlm.nih.gov/entrez/query.fcgi?db=gene&cmd=Retrieve&dopt=full_report&list_uids=4628) | myosin heavy chain 10 |
| [Details](http://mirdb.org/cgi-bin/target_detail.cgi?targetID=1439506) | 1040 | 70 | hsa-miR-30c-5p | [HOOK3](http://www.ncbi.nlm.nih.gov/entrez/query.fcgi?db=gene&cmd=Retrieve&dopt=full_report&list_uids=84376) | hook microtubule tethering protein 3 |
| [Details](http://mirdb.org/cgi-bin/target_detail.cgi?targetID=1439617) | 1041 | 70 | hsa-miR-30c-5p | [CCDC71L](http://www.ncbi.nlm.nih.gov/entrez/query.fcgi?db=gene&cmd=Retrieve&dopt=full_report&list_uids=168455) | coiled-coil domain containing 71 like |
| [Details](http://mirdb.org/cgi-bin/target_detail.cgi?targetID=1439638) | 1042 | 70 | hsa-miR-30c-5p | [AP1S3](http://www.ncbi.nlm.nih.gov/entrez/query.fcgi?db=gene&cmd=Retrieve&dopt=full_report&list_uids=130340) | adaptor related protein complex 1 subunit sigma 3 |
| [Details](http://mirdb.org/cgi-bin/target_detail.cgi?targetID=1439738) | 1043 | 70 | hsa-miR-30c-5p | [SPCS3](http://www.ncbi.nlm.nih.gov/entrez/query.fcgi?db=gene&cmd=Retrieve&dopt=full_report&list_uids=60559) | signal peptidase complex subunit 3 |
| [Details](http://mirdb.org/cgi-bin/target_detail.cgi?targetID=1439865) | 1044 | 70 | hsa-miR-30c-5p | [ZNF736](http://www.ncbi.nlm.nih.gov/entrez/query.fcgi?db=gene&cmd=Retrieve&dopt=full_report&list_uids=728927) | zinc finger protein 736 |
| [Details](http://mirdb.org/cgi-bin/target_detail.cgi?targetID=1439957) | 1045 | 70 | hsa-miR-30c-5p | [RPS6KA5](http://www.ncbi.nlm.nih.gov/entrez/query.fcgi?db=gene&cmd=Retrieve&dopt=full_report&list_uids=9252) | ribosomal protein S6 kinase A5 |
| [Details](http://mirdb.org/cgi-bin/target_detail.cgi?targetID=1440068) | 1046 | 70 | hsa-miR-30c-5p | [OSGEPL1](http://www.ncbi.nlm.nih.gov/entrez/query.fcgi?db=gene&cmd=Retrieve&dopt=full_report&list_uids=64172) | O-sialoglycoprotein endopeptidase like 1 |
| [Details](http://mirdb.org/cgi-bin/target_detail.cgi?targetID=1440070) | 1047 | 70 | hsa-miR-30c-5p | [FNDC3B](http://www.ncbi.nlm.nih.gov/entrez/query.fcgi?db=gene&cmd=Retrieve&dopt=full_report&list_uids=64778) | fibronectin type III domain containing 3B |
| [Details](http://mirdb.org/cgi-bin/target_detail.cgi?targetID=1440075) | 1048 | 70 | hsa-miR-30c-5p | [SLC41A2](http://www.ncbi.nlm.nih.gov/entrez/query.fcgi?db=gene&cmd=Retrieve&dopt=full_report&list_uids=84102) | solute carrier family 41 member 2 |
| [Details](http://mirdb.org/cgi-bin/target_detail.cgi?targetID=1440153) | 1049 | 70 | hsa-miR-30c-5p | [ZBTB39](http://www.ncbi.nlm.nih.gov/entrez/query.fcgi?db=gene&cmd=Retrieve&dopt=full_report&list_uids=9880) | zinc finger and BTB domain containing 39 |
| [Details](http://mirdb.org/cgi-bin/target_detail.cgi?targetID=1440163) | 1050 | 70 | hsa-miR-30c-5p | [NCEH1](http://www.ncbi.nlm.nih.gov/entrez/query.fcgi?db=gene&cmd=Retrieve&dopt=full_report&list_uids=57552) | neutral cholesterol ester hydrolase 1 |
| [Details](http://mirdb.org/cgi-bin/target_detail.cgi?targetID=1440254) | 1051 | 70 | hsa-miR-30c-5p | [TRIM9](http://www.ncbi.nlm.nih.gov/entrez/query.fcgi?db=gene&cmd=Retrieve&dopt=full_report&list_uids=114088) | tripartite motif containing 9 |
| [Details](http://mirdb.org/cgi-bin/target_detail.cgi?targetID=1440280) | 1052 | 70 | hsa-miR-30c-5p | [JUNB](http://www.ncbi.nlm.nih.gov/entrez/query.fcgi?db=gene&cmd=Retrieve&dopt=full_report&list_uids=3726) | JunB proto-oncogene, AP-1 transcription factor subunit |
| [Details](http://mirdb.org/cgi-bin/target_detail.cgi?targetID=1440292) | 1053 | 70 | hsa-miR-30c-5p | [TRPS1](http://www.ncbi.nlm.nih.gov/entrez/query.fcgi?db=gene&cmd=Retrieve&dopt=full_report&list_uids=7227) | transcriptional repressor GATA binding 1 |
| [Details](http://mirdb.org/cgi-bin/target_detail.cgi?targetID=1440305) | 1054 | 70 | hsa-miR-30c-5p | [ADRB2](http://www.ncbi.nlm.nih.gov/entrez/query.fcgi?db=gene&cmd=Retrieve&dopt=full_report&list_uids=154) | adrenoceptor beta 2 |
| [Details](http://mirdb.org/cgi-bin/target_detail.cgi?targetID=1440345) | 1055 | 70 | hsa-miR-30c-5p | [FYCO1](http://www.ncbi.nlm.nih.gov/entrez/query.fcgi?db=gene&cmd=Retrieve&dopt=full_report&list_uids=79443) | FYVE and coiled-coil domain containing 1 |
| [Details](http://mirdb.org/cgi-bin/target_detail.cgi?targetID=1440365) | 1056 | 70 | hsa-miR-30c-5p | [TNKS](http://www.ncbi.nlm.nih.gov/entrez/query.fcgi?db=gene&cmd=Retrieve&dopt=full_report&list_uids=8658) | tankyrase |
| [Details](http://mirdb.org/cgi-bin/target_detail.cgi?targetID=1440375) | 1057 | 70 | hsa-miR-30c-5p | [PGM2](http://www.ncbi.nlm.nih.gov/entrez/query.fcgi?db=gene&cmd=Retrieve&dopt=full_report&list_uids=55276) | phosphoglucomutase 2 |
| [Details](http://mirdb.org/cgi-bin/target_detail.cgi?targetID=1440402) | 1058 | 70 | hsa-miR-30c-5p | [SLC36A4](http://www.ncbi.nlm.nih.gov/entrez/query.fcgi?db=gene&cmd=Retrieve&dopt=full_report&list_uids=120103) | solute carrier family 36 member 4 |
| [Details](http://mirdb.org/cgi-bin/target_detail.cgi?targetID=1440455) | 1059 | 70 | hsa-miR-30c-5p | [LHFPL2](http://www.ncbi.nlm.nih.gov/entrez/query.fcgi?db=gene&cmd=Retrieve&dopt=full_report&list_uids=10184) | LHFPL tetraspan subfamily member 2 |
| [Details](http://mirdb.org/cgi-bin/target_detail.cgi?targetID=1440463) | 1060 | 70 | hsa-miR-30c-5p | [GTF2H1](http://www.ncbi.nlm.nih.gov/entrez/query.fcgi?db=gene&cmd=Retrieve&dopt=full_report&list_uids=2965) | general transcription factor IIH subunit 1 |
| [Details](http://mirdb.org/cgi-bin/target_detail.cgi?targetID=1440533) | 1061 | 70 | hsa-miR-30c-5p | [DCX](http://www.ncbi.nlm.nih.gov/entrez/query.fcgi?db=gene&cmd=Retrieve&dopt=full_report&list_uids=1641) | doublecortin |
| [Details](http://mirdb.org/cgi-bin/target_detail.cgi?targetID=1440606) | 1062 | 70 | hsa-miR-30c-5p | [NFIA](http://www.ncbi.nlm.nih.gov/entrez/query.fcgi?db=gene&cmd=Retrieve&dopt=full_report&list_uids=4774) | nuclear factor I A |
| [Details](http://mirdb.org/cgi-bin/target_detail.cgi?targetID=1440607) | 1063 | 70 | hsa-miR-30c-5p | [ARF4](http://www.ncbi.nlm.nih.gov/entrez/query.fcgi?db=gene&cmd=Retrieve&dopt=full_report&list_uids=378) | ADP ribosylation factor 4 |
| [Details](http://mirdb.org/cgi-bin/target_detail.cgi?targetID=1440626) | 1064 | 70 | hsa-miR-30c-5p | [GPR26](http://www.ncbi.nlm.nih.gov/entrez/query.fcgi?db=gene&cmd=Retrieve&dopt=full_report&list_uids=2849) | G protein-coupled receptor 26 |
| [Details](http://mirdb.org/cgi-bin/target_detail.cgi?targetID=1440820) | 1065 | 70 | hsa-miR-30c-5p | [BEST4](http://www.ncbi.nlm.nih.gov/entrez/query.fcgi?db=gene&cmd=Retrieve&dopt=full_report&list_uids=266675) | bestrophin 4 |
| [Details](http://mirdb.org/cgi-bin/target_detail.cgi?targetID=1440831) | 1066 | 70 | hsa-miR-30c-5p | [BAZ2B](http://www.ncbi.nlm.nih.gov/entrez/query.fcgi?db=gene&cmd=Retrieve&dopt=full_report&list_uids=29994) | bromodomain adjacent to zinc finger domain 2B |
| [Details](http://mirdb.org/cgi-bin/target_detail.cgi?targetID=1440883) | 1067 | 70 | hsa-miR-30c-5p | [TPM4](http://www.ncbi.nlm.nih.gov/entrez/query.fcgi?db=gene&cmd=Retrieve&dopt=full_report&list_uids=7171) | tropomyosin 4 |
| [Details](http://mirdb.org/cgi-bin/target_detail.cgi?targetID=1440927) | 1068 | 70 | hsa-miR-30c-5p | [GABRA5](http://www.ncbi.nlm.nih.gov/entrez/query.fcgi?db=gene&cmd=Retrieve&dopt=full_report&list_uids=2558) | gamma-aminobutyric acid type A receptor alpha5 subunit |
| [Details](http://mirdb.org/cgi-bin/target_detail.cgi?targetID=1440937) | 1069 | 70 | hsa-miR-30c-5p | [PLXNC1](http://www.ncbi.nlm.nih.gov/entrez/query.fcgi?db=gene&cmd=Retrieve&dopt=full_report&list_uids=10154) | plexin C1 |
| [Details](http://mirdb.org/cgi-bin/target_detail.cgi?targetID=1440981) | 1070 | 70 | hsa-miR-30c-5p | [IKZF1](http://www.ncbi.nlm.nih.gov/entrez/query.fcgi?db=gene&cmd=Retrieve&dopt=full_report&list_uids=10320) | IKAROS family zinc finger 1 |
| [Details](http://mirdb.org/cgi-bin/target_detail.cgi?targetID=1440983) | 1071 | 70 | hsa-miR-30c-5p | [VCPKMT](http://www.ncbi.nlm.nih.gov/entrez/query.fcgi?db=gene&cmd=Retrieve&dopt=full_report&list_uids=79609) | valosin containing protein lysine methyltransferase |
| [Details](http://mirdb.org/cgi-bin/target_detail.cgi?targetID=1439489) | 1072 | 69 | hsa-miR-30c-5p | [KRTAP1-5](http://www.ncbi.nlm.nih.gov/entrez/query.fcgi?db=gene&cmd=Retrieve&dopt=full_report&list_uids=83895) | keratin associated protein 1-5 |
| [Details](http://mirdb.org/cgi-bin/target_detail.cgi?targetID=1439513) | 1073 | 69 | hsa-miR-30c-5p | [LONRF1](http://www.ncbi.nlm.nih.gov/entrez/query.fcgi?db=gene&cmd=Retrieve&dopt=full_report&list_uids=91694) | LON peptidase N-terminal domain and ring finger 1 |
| [Details](http://mirdb.org/cgi-bin/target_detail.cgi?targetID=1439519) | 1074 | 69 | hsa-miR-30c-5p | [CCSER1](http://www.ncbi.nlm.nih.gov/entrez/query.fcgi?db=gene&cmd=Retrieve&dopt=full_report&list_uids=401145) | coiled-coil serine rich protein 1 |
| [Details](http://mirdb.org/cgi-bin/target_detail.cgi?targetID=1439543) | 1075 | 69 | hsa-miR-30c-5p | [SCML2](http://www.ncbi.nlm.nih.gov/entrez/query.fcgi?db=gene&cmd=Retrieve&dopt=full_report&list_uids=10389) | Scm polycomb group protein like 2 |
| [Details](http://mirdb.org/cgi-bin/target_detail.cgi?targetID=1439684) | 1076 | 69 | hsa-miR-30c-5p | [NCK2](http://www.ncbi.nlm.nih.gov/entrez/query.fcgi?db=gene&cmd=Retrieve&dopt=full_report&list_uids=8440) | NCK adaptor protein 2 |
| [Details](http://mirdb.org/cgi-bin/target_detail.cgi?targetID=1439689) | 1077 | 69 | hsa-miR-30c-5p | [IL21R](http://www.ncbi.nlm.nih.gov/entrez/query.fcgi?db=gene&cmd=Retrieve&dopt=full_report&list_uids=50615) | interleukin 21 receptor |
| [Details](http://mirdb.org/cgi-bin/target_detail.cgi?targetID=1439697) | 1078 | 69 | hsa-miR-30c-5p | [ARID3A](http://www.ncbi.nlm.nih.gov/entrez/query.fcgi?db=gene&cmd=Retrieve&dopt=full_report&list_uids=1820) | AT-rich interaction domain 3A |
| [Details](http://mirdb.org/cgi-bin/target_detail.cgi?targetID=1439700) | 1079 | 69 | hsa-miR-30c-5p | [SNAPIN](http://www.ncbi.nlm.nih.gov/entrez/query.fcgi?db=gene&cmd=Retrieve&dopt=full_report&list_uids=23557) | SNAP associated protein |
| [Details](http://mirdb.org/cgi-bin/target_detail.cgi?targetID=1439728) | 1080 | 69 | hsa-miR-30c-5p | [PCGF3](http://www.ncbi.nlm.nih.gov/entrez/query.fcgi?db=gene&cmd=Retrieve&dopt=full_report&list_uids=10336) | polycomb group ring finger 3 |
| [Details](http://mirdb.org/cgi-bin/target_detail.cgi?targetID=1439787) | 1081 | 69 | hsa-miR-30c-5p | [ACTBL2](http://www.ncbi.nlm.nih.gov/entrez/query.fcgi?db=gene&cmd=Retrieve&dopt=full_report&list_uids=345651) | actin, beta like 2 |
| [Details](http://mirdb.org/cgi-bin/target_detail.cgi?targetID=1439804) | 1082 | 69 | hsa-miR-30c-5p | [LRRFIP2](http://www.ncbi.nlm.nih.gov/entrez/query.fcgi?db=gene&cmd=Retrieve&dopt=full_report&list_uids=9209) | LRR binding FLII interacting protein 2 |
| [Details](http://mirdb.org/cgi-bin/target_detail.cgi?targetID=1439808) | 1083 | 69 | hsa-miR-30c-5p | [RGS22](http://www.ncbi.nlm.nih.gov/entrez/query.fcgi?db=gene&cmd=Retrieve&dopt=full_report&list_uids=26166) | regulator of G protein signaling 22 |
| [Details](http://mirdb.org/cgi-bin/target_detail.cgi?targetID=1439857) | 1084 | 69 | hsa-miR-30c-5p | [HDAC5](http://www.ncbi.nlm.nih.gov/entrez/query.fcgi?db=gene&cmd=Retrieve&dopt=full_report&list_uids=10014) | histone deacetylase 5 |
| [Details](http://mirdb.org/cgi-bin/target_detail.cgi?targetID=1439888) | 1085 | 69 | hsa-miR-30c-5p | [ICE2](http://www.ncbi.nlm.nih.gov/entrez/query.fcgi?db=gene&cmd=Retrieve&dopt=full_report&list_uids=79664) | interactor of little elongation complex ELL subunit 2 |
| [Details](http://mirdb.org/cgi-bin/target_detail.cgi?targetID=1439930) | 1086 | 69 | hsa-miR-30c-5p | [SLC30A2](http://www.ncbi.nlm.nih.gov/entrez/query.fcgi?db=gene&cmd=Retrieve&dopt=full_report&list_uids=7780) | solute carrier family 30 member 2 |
| [Details](http://mirdb.org/cgi-bin/target_detail.cgi?targetID=1439936) | 1087 | 69 | hsa-miR-30c-5p | [WDR1](http://www.ncbi.nlm.nih.gov/entrez/query.fcgi?db=gene&cmd=Retrieve&dopt=full_report&list_uids=9948) | WD repeat domain 1 |
| [Details](http://mirdb.org/cgi-bin/target_detail.cgi?targetID=1439974) | 1088 | 69 | hsa-miR-30c-5p | [CELF3](http://www.ncbi.nlm.nih.gov/entrez/query.fcgi?db=gene&cmd=Retrieve&dopt=full_report&list_uids=11189) | CUGBP Elav-like family member 3 |
| [Details](http://mirdb.org/cgi-bin/target_detail.cgi?targetID=1440231) | 1089 | 69 | hsa-miR-30c-5p | [AGPS](http://www.ncbi.nlm.nih.gov/entrez/query.fcgi?db=gene&cmd=Retrieve&dopt=full_report&list_uids=8540) | alkylglycerone phosphate synthase |
| [Details](http://mirdb.org/cgi-bin/target_detail.cgi?targetID=1440343) | 1090 | 69 | hsa-miR-30c-5p | [AIDA](http://www.ncbi.nlm.nih.gov/entrez/query.fcgi?db=gene&cmd=Retrieve&dopt=full_report&list_uids=64853) | axin interactor, dorsalization associated |
| [Details](http://mirdb.org/cgi-bin/target_detail.cgi?targetID=1440399) | 1091 | 69 | hsa-miR-30c-5p | [RPUSD4](http://www.ncbi.nlm.nih.gov/entrez/query.fcgi?db=gene&cmd=Retrieve&dopt=full_report&list_uids=84881) | RNA pseudouridine synthase D4 |
| [Details](http://mirdb.org/cgi-bin/target_detail.cgi?targetID=1440408) | 1092 | 69 | hsa-miR-30c-5p | [PTPN20](http://www.ncbi.nlm.nih.gov/entrez/query.fcgi?db=gene&cmd=Retrieve&dopt=full_report&list_uids=26095) | protein tyrosine phosphatase, non-receptor type 20 |
| [Details](http://mirdb.org/cgi-bin/target_detail.cgi?targetID=1440659) | 1093 | 69 | hsa-miR-30c-5p | [TASOR](http://www.ncbi.nlm.nih.gov/entrez/query.fcgi?db=gene&cmd=Retrieve&dopt=full_report&list_uids=23272) | transcription activation suppressor |
| [Details](http://mirdb.org/cgi-bin/target_detail.cgi?targetID=1440703) | 1094 | 69 | hsa-miR-30c-5p | [DENND4A](http://www.ncbi.nlm.nih.gov/entrez/query.fcgi?db=gene&cmd=Retrieve&dopt=full_report&list_uids=10260) | DENN domain containing 4A |
| [Details](http://mirdb.org/cgi-bin/target_detail.cgi?targetID=1440731) | 1095 | 69 | hsa-miR-30c-5p | [LCOR](http://www.ncbi.nlm.nih.gov/entrez/query.fcgi?db=gene&cmd=Retrieve&dopt=full_report&list_uids=84458) | ligand dependent nuclear receptor corepressor |
| [Details](http://mirdb.org/cgi-bin/target_detail.cgi?targetID=1440754) | 1096 | 69 | hsa-miR-30c-5p | [FAM81A](http://www.ncbi.nlm.nih.gov/entrez/query.fcgi?db=gene&cmd=Retrieve&dopt=full_report&list_uids=145773) | family with sequence similarity 81 member A |
| [Details](http://mirdb.org/cgi-bin/target_detail.cgi?targetID=1440799) | 1097 | 69 | hsa-miR-30c-5p | [BDNF](http://www.ncbi.nlm.nih.gov/entrez/query.fcgi?db=gene&cmd=Retrieve&dopt=full_report&list_uids=627) | brain derived neurotrophic factor |
| [Details](http://mirdb.org/cgi-bin/target_detail.cgi?targetID=1440859) | 1098 | 69 | hsa-miR-30c-5p | [SYNCRIP](http://www.ncbi.nlm.nih.gov/entrez/query.fcgi?db=gene&cmd=Retrieve&dopt=full_report&list_uids=10492) | synaptotagmin binding cytoplasmic RNA interacting protein |
| [Details](http://mirdb.org/cgi-bin/target_detail.cgi?targetID=1440864) | 1099 | 69 | hsa-miR-30c-5p | [EFR3A](http://www.ncbi.nlm.nih.gov/entrez/query.fcgi?db=gene&cmd=Retrieve&dopt=full_report&list_uids=23167) | EFR3 homolog A |
| [Details](http://mirdb.org/cgi-bin/target_detail.cgi?targetID=1440869) | 1100 | 69 | hsa-miR-30c-5p | [FRK](http://www.ncbi.nlm.nih.gov/entrez/query.fcgi?db=gene&cmd=Retrieve&dopt=full_report&list_uids=2444) | fyn related Src family tyrosine kinase |
| [Details](http://mirdb.org/cgi-bin/target_detail.cgi?targetID=1440954) | 1101 | 69 | hsa-miR-30c-5p | [LARP4](http://www.ncbi.nlm.nih.gov/entrez/query.fcgi?db=gene&cmd=Retrieve&dopt=full_report&list_uids=113251) | La ribonucleoprotein domain family member 4 |
| [Details](http://mirdb.org/cgi-bin/target_detail.cgi?targetID=1440975) | 1102 | 69 | hsa-miR-30c-5p | [PLEKHB2](http://www.ncbi.nlm.nih.gov/entrez/query.fcgi?db=gene&cmd=Retrieve&dopt=full_report&list_uids=55041) | pleckstrin homology domain containing B2 |
| [Details](http://mirdb.org/cgi-bin/target_detail.cgi?targetID=1439500) | 1103 | 68 | hsa-miR-30c-5p | [TMEM86A](http://www.ncbi.nlm.nih.gov/entrez/query.fcgi?db=gene&cmd=Retrieve&dopt=full_report&list_uids=144110) | transmembrane protein 86A |
| [Details](http://mirdb.org/cgi-bin/target_detail.cgi?targetID=1439682) | 1104 | 68 | hsa-miR-30c-5p | [IQCK](http://www.ncbi.nlm.nih.gov/entrez/query.fcgi?db=gene&cmd=Retrieve&dopt=full_report&list_uids=124152) | IQ motif containing K |
| [Details](http://mirdb.org/cgi-bin/target_detail.cgi?targetID=1439772) | 1105 | 68 | hsa-miR-30c-5p | [SOX12](http://www.ncbi.nlm.nih.gov/entrez/query.fcgi?db=gene&cmd=Retrieve&dopt=full_report&list_uids=6666) | SRY-box 12 |
| [Details](http://mirdb.org/cgi-bin/target_detail.cgi?targetID=1439801) | 1106 | 68 | hsa-miR-30c-5p | [MRTFB](http://www.ncbi.nlm.nih.gov/entrez/query.fcgi?db=gene&cmd=Retrieve&dopt=full_report&list_uids=57496) | myocardin related transcription factor B |
| [Details](http://mirdb.org/cgi-bin/target_detail.cgi?targetID=1440110) | 1107 | 68 | hsa-miR-30c-5p | [CARNMT1](http://www.ncbi.nlm.nih.gov/entrez/query.fcgi?db=gene&cmd=Retrieve&dopt=full_report&list_uids=138199) | carnosine N-methyltransferase 1 |
| [Details](http://mirdb.org/cgi-bin/target_detail.cgi?targetID=1440145) | 1108 | 68 | hsa-miR-30c-5p | [JARID2](http://www.ncbi.nlm.nih.gov/entrez/query.fcgi?db=gene&cmd=Retrieve&dopt=full_report&list_uids=3720) | jumonji and AT-rich interaction domain containing 2 |
| [Details](http://mirdb.org/cgi-bin/target_detail.cgi?targetID=1440236) | 1109 | 68 | hsa-miR-30c-5p | [MAP3K15](http://www.ncbi.nlm.nih.gov/entrez/query.fcgi?db=gene&cmd=Retrieve&dopt=full_report&list_uids=389840) | mitogen-activated protein kinase kinase kinase 15 |
| [Details](http://mirdb.org/cgi-bin/target_detail.cgi?targetID=1440362) | 1110 | 68 | hsa-miR-30c-5p | [ATAD2B](http://www.ncbi.nlm.nih.gov/entrez/query.fcgi?db=gene&cmd=Retrieve&dopt=full_report&list_uids=54454) | ATPase family, AAA domain containing 2B |
| [Details](http://mirdb.org/cgi-bin/target_detail.cgi?targetID=1440473) | 1111 | 68 | hsa-miR-30c-5p | [GRAMD2A](http://www.ncbi.nlm.nih.gov/entrez/query.fcgi?db=gene&cmd=Retrieve&dopt=full_report&list_uids=196996) | GRAM domain containing 2A |
| [Details](http://mirdb.org/cgi-bin/target_detail.cgi?targetID=1440509) | 1112 | 68 | hsa-miR-30c-5p | [TMED2](http://www.ncbi.nlm.nih.gov/entrez/query.fcgi?db=gene&cmd=Retrieve&dopt=full_report&list_uids=10959) | transmembrane p24 trafficking protein 2 |
| [Details](http://mirdb.org/cgi-bin/target_detail.cgi?targetID=1440532) | 1113 | 68 | hsa-miR-30c-5p | [GPR180](http://www.ncbi.nlm.nih.gov/entrez/query.fcgi?db=gene&cmd=Retrieve&dopt=full_report&list_uids=160897) | G protein-coupled receptor 180 |
| [Details](http://mirdb.org/cgi-bin/target_detail.cgi?targetID=1440594) | 1114 | 68 | hsa-miR-30c-5p | [ERMN](http://www.ncbi.nlm.nih.gov/entrez/query.fcgi?db=gene&cmd=Retrieve&dopt=full_report&list_uids=57471) | ermin |
| [Details](http://mirdb.org/cgi-bin/target_detail.cgi?targetID=1440598) | 1115 | 68 | hsa-miR-30c-5p | [SELENOS](http://www.ncbi.nlm.nih.gov/entrez/query.fcgi?db=gene&cmd=Retrieve&dopt=full_report&list_uids=55829) | selenoprotein S |
| [Details](http://mirdb.org/cgi-bin/target_detail.cgi?targetID=1440624) | 1116 | 68 | hsa-miR-30c-5p | [LYST](http://www.ncbi.nlm.nih.gov/entrez/query.fcgi?db=gene&cmd=Retrieve&dopt=full_report&list_uids=1130) | lysosomal trafficking regulator |
| [Details](http://mirdb.org/cgi-bin/target_detail.cgi?targetID=1440643) | 1117 | 68 | hsa-miR-30c-5p | [TRIM23](http://www.ncbi.nlm.nih.gov/entrez/query.fcgi?db=gene&cmd=Retrieve&dopt=full_report&list_uids=373) | tripartite motif containing 23 |
| [Details](http://mirdb.org/cgi-bin/target_detail.cgi?targetID=1440660) | 1118 | 68 | hsa-miR-30c-5p | [RRAGD](http://www.ncbi.nlm.nih.gov/entrez/query.fcgi?db=gene&cmd=Retrieve&dopt=full_report&list_uids=58528) | Ras related GTP binding D |
| [Details](http://mirdb.org/cgi-bin/target_detail.cgi?targetID=1440682) | 1119 | 68 | hsa-miR-30c-5p | [MAP3K19](http://www.ncbi.nlm.nih.gov/entrez/query.fcgi?db=gene&cmd=Retrieve&dopt=full_report&list_uids=80122) | mitogen-activated protein kinase kinase kinase 19 |
| [Details](http://mirdb.org/cgi-bin/target_detail.cgi?targetID=1440699) | 1120 | 68 | hsa-miR-30c-5p | [HECTD2](http://www.ncbi.nlm.nih.gov/entrez/query.fcgi?db=gene&cmd=Retrieve&dopt=full_report&list_uids=143279) | HECT domain E3 ubiquitin protein ligase 2 |
| [Details](http://mirdb.org/cgi-bin/target_detail.cgi?targetID=1440753) | 1121 | 68 | hsa-miR-30c-5p | [UBE2D3](http://www.ncbi.nlm.nih.gov/entrez/query.fcgi?db=gene&cmd=Retrieve&dopt=full_report&list_uids=7323) | ubiquitin conjugating enzyme E2 D3 |
| [Details](http://mirdb.org/cgi-bin/target_detail.cgi?targetID=1440811) | 1122 | 68 | hsa-miR-30c-5p | [MAVS](http://www.ncbi.nlm.nih.gov/entrez/query.fcgi?db=gene&cmd=Retrieve&dopt=full_report&list_uids=57506) | mitochondrial antiviral signaling protein |
| [Details](http://mirdb.org/cgi-bin/target_detail.cgi?targetID=1440926) | 1123 | 68 | hsa-miR-30c-5p | [PITPNM2](http://www.ncbi.nlm.nih.gov/entrez/query.fcgi?db=gene&cmd=Retrieve&dopt=full_report&list_uids=57605) | phosphatidylinositol transfer protein membrane associated 2 |
| [Details](http://mirdb.org/cgi-bin/target_detail.cgi?targetID=1440978) | 1124 | 68 | hsa-miR-30c-5p | [GLUD2](http://www.ncbi.nlm.nih.gov/entrez/query.fcgi?db=gene&cmd=Retrieve&dopt=full_report&list_uids=2747) | glutamate dehydrogenase 2 |
| [Details](http://mirdb.org/cgi-bin/target_detail.cgi?targetID=1439559) | 1125 | 67 | hsa-miR-30c-5p | [FAM124A](http://www.ncbi.nlm.nih.gov/entrez/query.fcgi?db=gene&cmd=Retrieve&dopt=full_report&list_uids=220108) | family with sequence similarity 124 member A |
| [Details](http://mirdb.org/cgi-bin/target_detail.cgi?targetID=1439816) | 1126 | 67 | hsa-miR-30c-5p | [PCDH20](http://www.ncbi.nlm.nih.gov/entrez/query.fcgi?db=gene&cmd=Retrieve&dopt=full_report&list_uids=64881) | protocadherin 20 |
| [Details](http://mirdb.org/cgi-bin/target_detail.cgi?targetID=1439818) | 1127 | 67 | hsa-miR-30c-5p | [KHNYN](http://www.ncbi.nlm.nih.gov/entrez/query.fcgi?db=gene&cmd=Retrieve&dopt=full_report&list_uids=23351) | KH and NYN domain containing |
| [Details](http://mirdb.org/cgi-bin/target_detail.cgi?targetID=1439884) | 1128 | 67 | hsa-miR-30c-5p | [RMND5A](http://www.ncbi.nlm.nih.gov/entrez/query.fcgi?db=gene&cmd=Retrieve&dopt=full_report&list_uids=64795) | required for meiotic nuclear division 5 homolog A |
| [Details](http://mirdb.org/cgi-bin/target_detail.cgi?targetID=1439886) | 1129 | 67 | hsa-miR-30c-5p | [SPIRE1](http://www.ncbi.nlm.nih.gov/entrez/query.fcgi?db=gene&cmd=Retrieve&dopt=full_report&list_uids=56907) | spire type actin nucleation factor 1 |
| [Details](http://mirdb.org/cgi-bin/target_detail.cgi?targetID=1440057) | 1130 | 67 | hsa-miR-30c-5p | [CAPN7](http://www.ncbi.nlm.nih.gov/entrez/query.fcgi?db=gene&cmd=Retrieve&dopt=full_report&list_uids=23473) | calpain 7 |
| [Details](http://mirdb.org/cgi-bin/target_detail.cgi?targetID=1440085) | 1131 | 67 | hsa-miR-30c-5p | [ZNF318](http://www.ncbi.nlm.nih.gov/entrez/query.fcgi?db=gene&cmd=Retrieve&dopt=full_report&list_uids=24149) | zinc finger protein 318 |
| [Details](http://mirdb.org/cgi-bin/target_detail.cgi?targetID=1440174) | 1132 | 67 | hsa-miR-30c-5p | [GOLGA8B](http://www.ncbi.nlm.nih.gov/entrez/query.fcgi?db=gene&cmd=Retrieve&dopt=full_report&list_uids=440270) | golgin A8 family member B |
| [Details](http://mirdb.org/cgi-bin/target_detail.cgi?targetID=1440281) | 1133 | 67 | hsa-miR-30c-5p | [SLC29A3](http://www.ncbi.nlm.nih.gov/entrez/query.fcgi?db=gene&cmd=Retrieve&dopt=full_report&list_uids=55315) | solute carrier family 29 member 3 |
| [Details](http://mirdb.org/cgi-bin/target_detail.cgi?targetID=1440317) | 1134 | 67 | hsa-miR-30c-5p | [ZNF20](http://www.ncbi.nlm.nih.gov/entrez/query.fcgi?db=gene&cmd=Retrieve&dopt=full_report&list_uids=7568) | zinc finger protein 20 |
| [Details](http://mirdb.org/cgi-bin/target_detail.cgi?targetID=1440411) | 1135 | 67 | hsa-miR-30c-5p | [EVI5](http://www.ncbi.nlm.nih.gov/entrez/query.fcgi?db=gene&cmd=Retrieve&dopt=full_report&list_uids=7813) | ecotropic viral integration site 5 |
| [Details](http://mirdb.org/cgi-bin/target_detail.cgi?targetID=1440481) | 1136 | 67 | hsa-miR-30c-5p | [PPIL3](http://www.ncbi.nlm.nih.gov/entrez/query.fcgi?db=gene&cmd=Retrieve&dopt=full_report&list_uids=53938) | peptidylprolyl isomerase like 3 |
| [Details](http://mirdb.org/cgi-bin/target_detail.cgi?targetID=1440700) | 1137 | 67 | hsa-miR-30c-5p | [PPP2R1B](http://www.ncbi.nlm.nih.gov/entrez/query.fcgi?db=gene&cmd=Retrieve&dopt=full_report&list_uids=5519) | protein phosphatase 2 scaffold subunit Abeta |
| [Details](http://mirdb.org/cgi-bin/target_detail.cgi?targetID=1440938) | 1138 | 67 | hsa-miR-30c-5p | [RCHY1](http://www.ncbi.nlm.nih.gov/entrez/query.fcgi?db=gene&cmd=Retrieve&dopt=full_report&list_uids=25898) | ring finger and CHY zinc finger domain containing 1 |
| [Details](http://mirdb.org/cgi-bin/target_detail.cgi?targetID=1440939) | 1139 | 67 | hsa-miR-30c-5p | [DIP2C](http://www.ncbi.nlm.nih.gov/entrez/query.fcgi?db=gene&cmd=Retrieve&dopt=full_report&list_uids=22982) | disco interacting protein 2 homolog C |
| [Details](http://mirdb.org/cgi-bin/target_detail.cgi?targetID=1439491) | 1140 | 66 | hsa-miR-30c-5p | [ZFP36L2](http://www.ncbi.nlm.nih.gov/entrez/query.fcgi?db=gene&cmd=Retrieve&dopt=full_report&list_uids=678) | ZFP36 ring finger protein like 2 |
| [Details](http://mirdb.org/cgi-bin/target_detail.cgi?targetID=1439521) | 1141 | 66 | hsa-miR-30c-5p | [PDK3](http://www.ncbi.nlm.nih.gov/entrez/query.fcgi?db=gene&cmd=Retrieve&dopt=full_report&list_uids=5165) | pyruvate dehydrogenase kinase 3 |
| [Details](http://mirdb.org/cgi-bin/target_detail.cgi?targetID=1439642) | 1142 | 66 | hsa-miR-30c-5p | [ASCC1](http://www.ncbi.nlm.nih.gov/entrez/query.fcgi?db=gene&cmd=Retrieve&dopt=full_report&list_uids=51008) | activating signal cointegrator 1 complex subunit 1 |
| [Details](http://mirdb.org/cgi-bin/target_detail.cgi?targetID=1439714) | 1143 | 66 | hsa-miR-30c-5p | [TTN](http://www.ncbi.nlm.nih.gov/entrez/query.fcgi?db=gene&cmd=Retrieve&dopt=full_report&list_uids=7273) | titin |
| [Details](http://mirdb.org/cgi-bin/target_detail.cgi?targetID=1439960) | 1144 | 66 | hsa-miR-30c-5p | [REV1](http://www.ncbi.nlm.nih.gov/entrez/query.fcgi?db=gene&cmd=Retrieve&dopt=full_report&list_uids=51455) | REV1, DNA directed polymerase |
| [Details](http://mirdb.org/cgi-bin/target_detail.cgi?targetID=1440188) | 1145 | 66 | hsa-miR-30c-5p | [ZFYVE26](http://www.ncbi.nlm.nih.gov/entrez/query.fcgi?db=gene&cmd=Retrieve&dopt=full_report&list_uids=23503) | zinc finger FYVE-type containing 26 |
| [Details](http://mirdb.org/cgi-bin/target_detail.cgi?targetID=1440204) | 1146 | 66 | hsa-miR-30c-5p | [INTS6](http://www.ncbi.nlm.nih.gov/entrez/query.fcgi?db=gene&cmd=Retrieve&dopt=full_report&list_uids=26512) | integrator complex subunit 6 |
| [Details](http://mirdb.org/cgi-bin/target_detail.cgi?targetID=1440224) | 1147 | 66 | hsa-miR-30c-5p | [G3BP2](http://www.ncbi.nlm.nih.gov/entrez/query.fcgi?db=gene&cmd=Retrieve&dopt=full_report&list_uids=9908) | G3BP stress granule assembly factor 2 |
| [Details](http://mirdb.org/cgi-bin/target_detail.cgi?targetID=1440252) | 1148 | 66 | hsa-miR-30c-5p | [ECT2L](http://www.ncbi.nlm.nih.gov/entrez/query.fcgi?db=gene&cmd=Retrieve&dopt=full_report&list_uids=345930) | epithelial cell transforming 2 like |
| [Details](http://mirdb.org/cgi-bin/target_detail.cgi?targetID=1440289) | 1149 | 66 | hsa-miR-30c-5p | [SSH1](http://www.ncbi.nlm.nih.gov/entrez/query.fcgi?db=gene&cmd=Retrieve&dopt=full_report&list_uids=54434) | slingshot protein phosphatase 1 |
| [Details](http://mirdb.org/cgi-bin/target_detail.cgi?targetID=1440470) | 1150 | 66 | hsa-miR-30c-5p | [VENTX](http://www.ncbi.nlm.nih.gov/entrez/query.fcgi?db=gene&cmd=Retrieve&dopt=full_report&list_uids=27287) | VENT homeobox |
| [Details](http://mirdb.org/cgi-bin/target_detail.cgi?targetID=1440500) | 1151 | 66 | hsa-miR-30c-5p | [FAM24A](http://www.ncbi.nlm.nih.gov/entrez/query.fcgi?db=gene&cmd=Retrieve&dopt=full_report&list_uids=118670) | family with sequence similarity 24 member A |
| [Details](http://mirdb.org/cgi-bin/target_detail.cgi?targetID=1440507) | 1152 | 66 | hsa-miR-30c-5p | [NAV1](http://www.ncbi.nlm.nih.gov/entrez/query.fcgi?db=gene&cmd=Retrieve&dopt=full_report&list_uids=89796) | neuron navigator 1 |
| [Details](http://mirdb.org/cgi-bin/target_detail.cgi?targetID=1440521) | 1153 | 66 | hsa-miR-30c-5p | [NRBP1](http://www.ncbi.nlm.nih.gov/entrez/query.fcgi?db=gene&cmd=Retrieve&dopt=full_report&list_uids=29959) | nuclear receptor binding protein 1 |
| [Details](http://mirdb.org/cgi-bin/target_detail.cgi?targetID=1440524) | 1154 | 66 | hsa-miR-30c-5p | [TBC1D30](http://www.ncbi.nlm.nih.gov/entrez/query.fcgi?db=gene&cmd=Retrieve&dopt=full_report&list_uids=23329) | TBC1 domain family member 30 |
| [Details](http://mirdb.org/cgi-bin/target_detail.cgi?targetID=1440571) | 1155 | 66 | hsa-miR-30c-5p | [ALPK3](http://www.ncbi.nlm.nih.gov/entrez/query.fcgi?db=gene&cmd=Retrieve&dopt=full_report&list_uids=57538) | alpha kinase 3 |
| [Details](http://mirdb.org/cgi-bin/target_detail.cgi?targetID=1440622) | 1156 | 66 | hsa-miR-30c-5p | [PEG10](http://www.ncbi.nlm.nih.gov/entrez/query.fcgi?db=gene&cmd=Retrieve&dopt=full_report&list_uids=23089) | paternally expressed 10 |
| [Details](http://mirdb.org/cgi-bin/target_detail.cgi?targetID=1440636) | 1157 | 66 | hsa-miR-30c-5p | [RAP2B](http://www.ncbi.nlm.nih.gov/entrez/query.fcgi?db=gene&cmd=Retrieve&dopt=full_report&list_uids=5912) | RAP2B, member of RAS oncogene family |
| [Details](http://mirdb.org/cgi-bin/target_detail.cgi?targetID=1440654) | 1158 | 66 | hsa-miR-30c-5p | [PELI1](http://www.ncbi.nlm.nih.gov/entrez/query.fcgi?db=gene&cmd=Retrieve&dopt=full_report&list_uids=57162) | pellino E3 ubiquitin protein ligase 1 |
| [Details](http://mirdb.org/cgi-bin/target_detail.cgi?targetID=1440663) | 1159 | 66 | hsa-miR-30c-5p | [ZNF746](http://www.ncbi.nlm.nih.gov/entrez/query.fcgi?db=gene&cmd=Retrieve&dopt=full_report&list_uids=155061) | zinc finger protein 746 |
| [Details](http://mirdb.org/cgi-bin/target_detail.cgi?targetID=1440785) | 1160 | 66 | hsa-miR-30c-5p | [GLUD1](http://www.ncbi.nlm.nih.gov/entrez/query.fcgi?db=gene&cmd=Retrieve&dopt=full_report&list_uids=2746) | glutamate dehydrogenase 1 |
| [Details](http://mirdb.org/cgi-bin/target_detail.cgi?targetID=1440866) | 1161 | 66 | hsa-miR-30c-5p | [GM2A](http://www.ncbi.nlm.nih.gov/entrez/query.fcgi?db=gene&cmd=Retrieve&dopt=full_report&list_uids=2760) | GM2 ganglioside activator |
| [Details](http://mirdb.org/cgi-bin/target_detail.cgi?targetID=1440936) | 1162 | 66 | hsa-miR-30c-5p | [MYD88](http://www.ncbi.nlm.nih.gov/entrez/query.fcgi?db=gene&cmd=Retrieve&dopt=full_report&list_uids=4615) | MYD88, innate immune signal transduction adaptor |
| [Details](http://mirdb.org/cgi-bin/target_detail.cgi?targetID=1439508) | 1163 | 65 | hsa-miR-30c-5p | [PBRM1](http://www.ncbi.nlm.nih.gov/entrez/query.fcgi?db=gene&cmd=Retrieve&dopt=full_report&list_uids=55193) | polybromo 1 |
| [Details](http://mirdb.org/cgi-bin/target_detail.cgi?targetID=1439654) | 1164 | 65 | hsa-miR-30c-5p | [CEP76](http://www.ncbi.nlm.nih.gov/entrez/query.fcgi?db=gene&cmd=Retrieve&dopt=full_report&list_uids=79959) | centrosomal protein 76 |
| [Details](http://mirdb.org/cgi-bin/target_detail.cgi?targetID=1439729) | 1165 | 65 | hsa-miR-30c-5p | [UBE2J2](http://www.ncbi.nlm.nih.gov/entrez/query.fcgi?db=gene&cmd=Retrieve&dopt=full_report&list_uids=118424) | ubiquitin conjugating enzyme E2 J2 |
| [Details](http://mirdb.org/cgi-bin/target_detail.cgi?targetID=1439744) | 1166 | 65 | hsa-miR-30c-5p | [PLXNA1](http://www.ncbi.nlm.nih.gov/entrez/query.fcgi?db=gene&cmd=Retrieve&dopt=full_report&list_uids=5361) | plexin A1 |
| [Details](http://mirdb.org/cgi-bin/target_detail.cgi?targetID=1439791) | 1167 | 65 | hsa-miR-30c-5p | [SH3KBP1](http://www.ncbi.nlm.nih.gov/entrez/query.fcgi?db=gene&cmd=Retrieve&dopt=full_report&list_uids=30011) | SH3 domain containing kinase binding protein 1 |
| [Details](http://mirdb.org/cgi-bin/target_detail.cgi?targetID=1439793) | 1168 | 65 | hsa-miR-30c-5p | [SLC38A1](http://www.ncbi.nlm.nih.gov/entrez/query.fcgi?db=gene&cmd=Retrieve&dopt=full_report&list_uids=81539) | solute carrier family 38 member 1 |
| [Details](http://mirdb.org/cgi-bin/target_detail.cgi?targetID=1439891) | 1169 | 65 | hsa-miR-30c-5p | [ANKRD20A1](http://www.ncbi.nlm.nih.gov/entrez/query.fcgi?db=gene&cmd=Retrieve&dopt=full_report&list_uids=84210) | ankyrin repeat domain 20 family member A1 |
| [Details](http://mirdb.org/cgi-bin/target_detail.cgi?targetID=1440012) | 1170 | 65 | hsa-miR-30c-5p | [RFX2](http://www.ncbi.nlm.nih.gov/entrez/query.fcgi?db=gene&cmd=Retrieve&dopt=full_report&list_uids=5990) | regulatory factor X2 |
| [Details](http://mirdb.org/cgi-bin/target_detail.cgi?targetID=1440155) | 1171 | 65 | hsa-miR-30c-5p | [TVP23C](http://www.ncbi.nlm.nih.gov/entrez/query.fcgi?db=gene&cmd=Retrieve&dopt=full_report&list_uids=201158) | trans-golgi network vesicle protein 23 homolog C |
| [Details](http://mirdb.org/cgi-bin/target_detail.cgi?targetID=1440169) | 1172 | 65 | hsa-miR-30c-5p | [ZSWIM5](http://www.ncbi.nlm.nih.gov/entrez/query.fcgi?db=gene&cmd=Retrieve&dopt=full_report&list_uids=57643) | zinc finger SWIM-type containing 5 |
| [Details](http://mirdb.org/cgi-bin/target_detail.cgi?targetID=1440239) | 1173 | 65 | hsa-miR-30c-5p | [NID1](http://www.ncbi.nlm.nih.gov/entrez/query.fcgi?db=gene&cmd=Retrieve&dopt=full_report&list_uids=4811) | nidogen 1 |
| [Details](http://mirdb.org/cgi-bin/target_detail.cgi?targetID=1440274) | 1174 | 65 | hsa-miR-30c-5p | [C8orf34](http://www.ncbi.nlm.nih.gov/entrez/query.fcgi?db=gene&cmd=Retrieve&dopt=full_report&list_uids=116328) | chromosome 8 open reading frame 34 |
| [Details](http://mirdb.org/cgi-bin/target_detail.cgi?targetID=1440348) | 1175 | 65 | hsa-miR-30c-5p | [VSTM4](http://www.ncbi.nlm.nih.gov/entrez/query.fcgi?db=gene&cmd=Retrieve&dopt=full_report&list_uids=196740) | V-set and transmembrane domain containing 4 |
| [Details](http://mirdb.org/cgi-bin/target_detail.cgi?targetID=1440497) | 1176 | 65 | hsa-miR-30c-5p | [SEH1L](http://www.ncbi.nlm.nih.gov/entrez/query.fcgi?db=gene&cmd=Retrieve&dopt=full_report&list_uids=81929) | SEH1 like nucleoporin |
| [Details](http://mirdb.org/cgi-bin/target_detail.cgi?targetID=1440540) | 1177 | 65 | hsa-miR-30c-5p | [THBS2](http://www.ncbi.nlm.nih.gov/entrez/query.fcgi?db=gene&cmd=Retrieve&dopt=full_report&list_uids=7058) | thrombospondin 2 |
| [Details](http://mirdb.org/cgi-bin/target_detail.cgi?targetID=1440558) | 1178 | 65 | hsa-miR-30c-5p | [SATB1](http://www.ncbi.nlm.nih.gov/entrez/query.fcgi?db=gene&cmd=Retrieve&dopt=full_report&list_uids=6304) | SATB homeobox 1 |
| [Details](http://mirdb.org/cgi-bin/target_detail.cgi?targetID=1440568) | 1179 | 65 | hsa-miR-30c-5p | [ANKRD20A4](http://www.ncbi.nlm.nih.gov/entrez/query.fcgi?db=gene&cmd=Retrieve&dopt=full_report&list_uids=728747) | ankyrin repeat domain 20 family member A4 |
| [Details](http://mirdb.org/cgi-bin/target_detail.cgi?targetID=1440569) | 1180 | 65 | hsa-miR-30c-5p | [LIN7C](http://www.ncbi.nlm.nih.gov/entrez/query.fcgi?db=gene&cmd=Retrieve&dopt=full_report&list_uids=55327) | lin-7 homolog C, crumbs cell polarity complex component |
| [Details](http://mirdb.org/cgi-bin/target_detail.cgi?targetID=1440575) | 1181 | 65 | hsa-miR-30c-5p | [FAM169B](http://www.ncbi.nlm.nih.gov/entrez/query.fcgi?db=gene&cmd=Retrieve&dopt=full_report&list_uids=283777) | family with sequence similarity 169 member B |
| [Details](http://mirdb.org/cgi-bin/target_detail.cgi?targetID=1440604) | 1182 | 65 | hsa-miR-30c-5p | [RNMT](http://www.ncbi.nlm.nih.gov/entrez/query.fcgi?db=gene&cmd=Retrieve&dopt=full_report&list_uids=8731) | RNA guanine-7 methyltransferase |
| [Details](http://mirdb.org/cgi-bin/target_detail.cgi?targetID=1440620) | 1183 | 65 | hsa-miR-30c-5p | [RPGRIP1L](http://www.ncbi.nlm.nih.gov/entrez/query.fcgi?db=gene&cmd=Retrieve&dopt=full_report&list_uids=23322) | RPGRIP1 like |
| [Details](http://mirdb.org/cgi-bin/target_detail.cgi?targetID=1440957) | 1184 | 65 | hsa-miR-30c-5p | [EAF1](http://www.ncbi.nlm.nih.gov/entrez/query.fcgi?db=gene&cmd=Retrieve&dopt=full_report&list_uids=85403) | ELL associated factor 1 |
| [Details](http://mirdb.org/cgi-bin/target_detail.cgi?targetID=1440963) | 1185 | 65 | hsa-miR-30c-5p | [ANKRD20A2](http://www.ncbi.nlm.nih.gov/entrez/query.fcgi?db=gene&cmd=Retrieve&dopt=full_report&list_uids=441430) | ankyrin repeat domain 20 family member A2 |
| [Details](http://mirdb.org/cgi-bin/target_detail.cgi?targetID=1440982) | 1186 | 65 | hsa-miR-30c-5p | [DGKZ](http://www.ncbi.nlm.nih.gov/entrez/query.fcgi?db=gene&cmd=Retrieve&dopt=full_report&list_uids=8525) | diacylglycerol kinase zeta |
| [Details](http://mirdb.org/cgi-bin/target_detail.cgi?targetID=1439551) | 1187 | 64 | hsa-miR-30c-5p | [USP22](http://www.ncbi.nlm.nih.gov/entrez/query.fcgi?db=gene&cmd=Retrieve&dopt=full_report&list_uids=23326) | ubiquitin specific peptidase 22 |
| [Details](http://mirdb.org/cgi-bin/target_detail.cgi?targetID=1439564) | 1188 | 64 | hsa-miR-30c-5p | [FAM155A](http://www.ncbi.nlm.nih.gov/entrez/query.fcgi?db=gene&cmd=Retrieve&dopt=full_report&list_uids=728215) | family with sequence similarity 155 member A |
| [Details](http://mirdb.org/cgi-bin/target_detail.cgi?targetID=1439605) | 1189 | 64 | hsa-miR-30c-5p | [WDR41](http://www.ncbi.nlm.nih.gov/entrez/query.fcgi?db=gene&cmd=Retrieve&dopt=full_report&list_uids=55255) | WD repeat domain 41 |
| [Details](http://mirdb.org/cgi-bin/target_detail.cgi?targetID=1439701) | 1190 | 64 | hsa-miR-30c-5p | [UNKL](http://www.ncbi.nlm.nih.gov/entrez/query.fcgi?db=gene&cmd=Retrieve&dopt=full_report&list_uids=64718) | unk like zinc finger |
| [Details](http://mirdb.org/cgi-bin/target_detail.cgi?targetID=1439707) | 1191 | 64 | hsa-miR-30c-5p | [PTPRK](http://www.ncbi.nlm.nih.gov/entrez/query.fcgi?db=gene&cmd=Retrieve&dopt=full_report&list_uids=5796) | protein tyrosine phosphatase, receptor type K |
| [Details](http://mirdb.org/cgi-bin/target_detail.cgi?targetID=1439730) | 1192 | 64 | hsa-miR-30c-5p | [ADAM28](http://www.ncbi.nlm.nih.gov/entrez/query.fcgi?db=gene&cmd=Retrieve&dopt=full_report&list_uids=10863) | ADAM metallopeptidase domain 28 |
| [Details](http://mirdb.org/cgi-bin/target_detail.cgi?targetID=1439741) | 1193 | 64 | hsa-miR-30c-5p | [CLRN1](http://www.ncbi.nlm.nih.gov/entrez/query.fcgi?db=gene&cmd=Retrieve&dopt=full_report&list_uids=7401) | clarin 1 |
| [Details](http://mirdb.org/cgi-bin/target_detail.cgi?targetID=1439753) | 1194 | 64 | hsa-miR-30c-5p | [MAPRE1](http://www.ncbi.nlm.nih.gov/entrez/query.fcgi?db=gene&cmd=Retrieve&dopt=full_report&list_uids=22919) | microtubule associated protein RP/EB family member 1 |
| [Details](http://mirdb.org/cgi-bin/target_detail.cgi?targetID=1439780) | 1195 | 64 | hsa-miR-30c-5p | [SLC6A15](http://www.ncbi.nlm.nih.gov/entrez/query.fcgi?db=gene&cmd=Retrieve&dopt=full_report&list_uids=55117) | solute carrier family 6 member 15 |
| [Details](http://mirdb.org/cgi-bin/target_detail.cgi?targetID=1439786) | 1196 | 64 | hsa-miR-30c-5p | [PGM2L1](http://www.ncbi.nlm.nih.gov/entrez/query.fcgi?db=gene&cmd=Retrieve&dopt=full_report&list_uids=283209) | phosphoglucomutase 2 like 1 |
| [Details](http://mirdb.org/cgi-bin/target_detail.cgi?targetID=1439837) | 1197 | 64 | hsa-miR-30c-5p | [PLCG1](http://www.ncbi.nlm.nih.gov/entrez/query.fcgi?db=gene&cmd=Retrieve&dopt=full_report&list_uids=5335) | phospholipase C gamma 1 |
| [Details](http://mirdb.org/cgi-bin/target_detail.cgi?targetID=1440027) | 1198 | 64 | hsa-miR-30c-5p | [MAN1B1](http://www.ncbi.nlm.nih.gov/entrez/query.fcgi?db=gene&cmd=Retrieve&dopt=full_report&list_uids=11253) | mannosidase alpha class 1B member 1 |
| [Details](http://mirdb.org/cgi-bin/target_detail.cgi?targetID=1440104) | 1199 | 64 | hsa-miR-30c-5p | [RNF213](http://www.ncbi.nlm.nih.gov/entrez/query.fcgi?db=gene&cmd=Retrieve&dopt=full_report&list_uids=57674) | ring finger protein 213 |
| [Details](http://mirdb.org/cgi-bin/target_detail.cgi?targetID=1440124) | 1200 | 64 | hsa-miR-30c-5p | [UNC5D](http://www.ncbi.nlm.nih.gov/entrez/query.fcgi?db=gene&cmd=Retrieve&dopt=full_report&list_uids=137970) | unc-5 netrin receptor D |
| [Details](http://mirdb.org/cgi-bin/target_detail.cgi?targetID=1440168) | 1201 | 64 | hsa-miR-30c-5p | [KIAA1328](http://www.ncbi.nlm.nih.gov/entrez/query.fcgi?db=gene&cmd=Retrieve&dopt=full_report&list_uids=57536) | KIAA1328 |
| [Details](http://mirdb.org/cgi-bin/target_detail.cgi?targetID=1440270) | 1202 | 64 | hsa-miR-30c-5p | [PAG1](http://www.ncbi.nlm.nih.gov/entrez/query.fcgi?db=gene&cmd=Retrieve&dopt=full_report&list_uids=55824) | phosphoprotein membrane anchor with glycosphingolipid microdomains 1 |
| [Details](http://mirdb.org/cgi-bin/target_detail.cgi?targetID=1440363) | 1203 | 64 | hsa-miR-30c-5p | [SNX30](http://www.ncbi.nlm.nih.gov/entrez/query.fcgi?db=gene&cmd=Retrieve&dopt=full_report&list_uids=401548) | sorting nexin family member 30 |
| [Details](http://mirdb.org/cgi-bin/target_detail.cgi?targetID=1440419) | 1204 | 64 | hsa-miR-30c-5p | [JAKMIP3](http://www.ncbi.nlm.nih.gov/entrez/query.fcgi?db=gene&cmd=Retrieve&dopt=full_report&list_uids=282973) | Janus kinase and microtubule interacting protein 3 |
| [Details](http://mirdb.org/cgi-bin/target_detail.cgi?targetID=1440424) | 1205 | 64 | hsa-miR-30c-5p | [NRBF2](http://www.ncbi.nlm.nih.gov/entrez/query.fcgi?db=gene&cmd=Retrieve&dopt=full_report&list_uids=29982) | nuclear receptor binding factor 2 |
| [Details](http://mirdb.org/cgi-bin/target_detail.cgi?targetID=1440553) | 1206 | 64 | hsa-miR-30c-5p | [DMTF1](http://www.ncbi.nlm.nih.gov/entrez/query.fcgi?db=gene&cmd=Retrieve&dopt=full_report&list_uids=9988) | cyclin D binding myb like transcription factor 1 |
| [Details](http://mirdb.org/cgi-bin/target_detail.cgi?targetID=1440555) | 1207 | 64 | hsa-miR-30c-5p | [CEP170B](http://www.ncbi.nlm.nih.gov/entrez/query.fcgi?db=gene&cmd=Retrieve&dopt=full_report&list_uids=283638) | centrosomal protein 170B |
| [Details](http://mirdb.org/cgi-bin/target_detail.cgi?targetID=1440629) | 1208 | 64 | hsa-miR-30c-5p | [DSP](http://www.ncbi.nlm.nih.gov/entrez/query.fcgi?db=gene&cmd=Retrieve&dopt=full_report&list_uids=1832) | desmoplakin |
| [Details](http://mirdb.org/cgi-bin/target_detail.cgi?targetID=1440697) | 1209 | 64 | hsa-miR-30c-5p | [TCTN3](http://www.ncbi.nlm.nih.gov/entrez/query.fcgi?db=gene&cmd=Retrieve&dopt=full_report&list_uids=26123) | tectonic family member 3 |
| [Details](http://mirdb.org/cgi-bin/target_detail.cgi?targetID=1440702) | 1210 | 64 | hsa-miR-30c-5p | [SON](http://www.ncbi.nlm.nih.gov/entrez/query.fcgi?db=gene&cmd=Retrieve&dopt=full_report&list_uids=6651) | SON DNA binding protein |
| [Details](http://mirdb.org/cgi-bin/target_detail.cgi?targetID=1440747) | 1211 | 64 | hsa-miR-30c-5p | [TFPI2](http://www.ncbi.nlm.nih.gov/entrez/query.fcgi?db=gene&cmd=Retrieve&dopt=full_report&list_uids=7980) | tissue factor pathway inhibitor 2 |
| [Details](http://mirdb.org/cgi-bin/target_detail.cgi?targetID=1440788) | 1212 | 64 | hsa-miR-30c-5p | [ZNF148](http://www.ncbi.nlm.nih.gov/entrez/query.fcgi?db=gene&cmd=Retrieve&dopt=full_report&list_uids=7707) | zinc finger protein 148 |
| [Details](http://mirdb.org/cgi-bin/target_detail.cgi?targetID=1440803) | 1213 | 64 | hsa-miR-30c-5p | [BID](http://www.ncbi.nlm.nih.gov/entrez/query.fcgi?db=gene&cmd=Retrieve&dopt=full_report&list_uids=637) | BH3 interacting domain death agonist |
| [Details](http://mirdb.org/cgi-bin/target_detail.cgi?targetID=1440899) | 1214 | 64 | hsa-miR-30c-5p | [NDUFA4](http://www.ncbi.nlm.nih.gov/entrez/query.fcgi?db=gene&cmd=Retrieve&dopt=full_report&list_uids=4697) | NDUFA4, mitochondrial complex associated |
| [Details](http://mirdb.org/cgi-bin/target_detail.cgi?targetID=1440903) | 1215 | 64 | hsa-miR-30c-5p | [UBE2R2](http://www.ncbi.nlm.nih.gov/entrez/query.fcgi?db=gene&cmd=Retrieve&dopt=full_report&list_uids=54926) | ubiquitin conjugating enzyme E2 R2 |
| [Details](http://mirdb.org/cgi-bin/target_detail.cgi?targetID=1440956) | 1216 | 64 | hsa-miR-30c-5p | [GALNT13](http://www.ncbi.nlm.nih.gov/entrez/query.fcgi?db=gene&cmd=Retrieve&dopt=full_report&list_uids=114805) | polypeptide N-acetylgalactosaminyltransferase 13 |
| [Details](http://mirdb.org/cgi-bin/target_detail.cgi?targetID=1440977) | 1217 | 64 | hsa-miR-30c-5p | [TRPA1](http://www.ncbi.nlm.nih.gov/entrez/query.fcgi?db=gene&cmd=Retrieve&dopt=full_report&list_uids=8989) | transient receptor potential cation channel subfamily A member 1 |
| [Details](http://mirdb.org/cgi-bin/target_detail.cgi?targetID=1439462) | 1218 | 63 | hsa-miR-30c-5p | [FMR1NB](http://www.ncbi.nlm.nih.gov/entrez/query.fcgi?db=gene&cmd=Retrieve&dopt=full_report&list_uids=158521) | FMR1 neighbor |
| [Details](http://mirdb.org/cgi-bin/target_detail.cgi?targetID=1439718) | 1219 | 63 | hsa-miR-30c-5p | [ZBTB7A](http://www.ncbi.nlm.nih.gov/entrez/query.fcgi?db=gene&cmd=Retrieve&dopt=full_report&list_uids=51341) | zinc finger and BTB domain containing 7A |
| [Details](http://mirdb.org/cgi-bin/target_detail.cgi?targetID=1439727) | 1220 | 63 | hsa-miR-30c-5p | [SIRT1](http://www.ncbi.nlm.nih.gov/entrez/query.fcgi?db=gene&cmd=Retrieve&dopt=full_report&list_uids=23411) | sirtuin 1 |
| [Details](http://mirdb.org/cgi-bin/target_detail.cgi?targetID=1439784) | 1221 | 63 | hsa-miR-30c-5p | [SLC25A40](http://www.ncbi.nlm.nih.gov/entrez/query.fcgi?db=gene&cmd=Retrieve&dopt=full_report&list_uids=55972) | solute carrier family 25 member 40 |
| [Details](http://mirdb.org/cgi-bin/target_detail.cgi?targetID=1439799) | 1222 | 63 | hsa-miR-30c-5p | [ABHD5](http://www.ncbi.nlm.nih.gov/entrez/query.fcgi?db=gene&cmd=Retrieve&dopt=full_report&list_uids=51099) | abhydrolase domain containing 5 |
| [Details](http://mirdb.org/cgi-bin/target_detail.cgi?targetID=1439864) | 1223 | 63 | hsa-miR-30c-5p | [MAU2](http://www.ncbi.nlm.nih.gov/entrez/query.fcgi?db=gene&cmd=Retrieve&dopt=full_report&list_uids=23383) | MAU2 sister chromatid cohesion factor |
| [Details](http://mirdb.org/cgi-bin/target_detail.cgi?targetID=1439908) | 1224 | 63 | hsa-miR-30c-5p | [NRP2](http://www.ncbi.nlm.nih.gov/entrez/query.fcgi?db=gene&cmd=Retrieve&dopt=full_report&list_uids=8828) | neuropilin 2 |
| [Details](http://mirdb.org/cgi-bin/target_detail.cgi?targetID=1439961) | 1225 | 63 | hsa-miR-30c-5p | [YES1](http://www.ncbi.nlm.nih.gov/entrez/query.fcgi?db=gene&cmd=Retrieve&dopt=full_report&list_uids=7525) | YES proto-oncogene 1, Src family tyrosine kinase |
| [Details](http://mirdb.org/cgi-bin/target_detail.cgi?targetID=1440096) | 1226 | 63 | hsa-miR-30c-5p | [SUN1](http://www.ncbi.nlm.nih.gov/entrez/query.fcgi?db=gene&cmd=Retrieve&dopt=full_report&list_uids=23353) | Sad1 and UNC84 domain containing 1 |
| [Details](http://mirdb.org/cgi-bin/target_detail.cgi?targetID=1440190) | 1227 | 63 | hsa-miR-30c-5p | [ZNF277](http://www.ncbi.nlm.nih.gov/entrez/query.fcgi?db=gene&cmd=Retrieve&dopt=full_report&list_uids=11179) | zinc finger protein 277 |
| [Details](http://mirdb.org/cgi-bin/target_detail.cgi?targetID=1440229) | 1228 | 63 | hsa-miR-30c-5p | [ENOX2](http://www.ncbi.nlm.nih.gov/entrez/query.fcgi?db=gene&cmd=Retrieve&dopt=full_report&list_uids=10495) | ecto-NOX disulfide-thiol exchanger 2 |
| [Details](http://mirdb.org/cgi-bin/target_detail.cgi?targetID=1440403) | 1229 | 63 | hsa-miR-30c-5p | [RSF1](http://www.ncbi.nlm.nih.gov/entrez/query.fcgi?db=gene&cmd=Retrieve&dopt=full_report&list_uids=51773) | remodeling and spacing factor 1 |
| [Details](http://mirdb.org/cgi-bin/target_detail.cgi?targetID=1440461) | 1230 | 63 | hsa-miR-30c-5p | [PTGER3](http://www.ncbi.nlm.nih.gov/entrez/query.fcgi?db=gene&cmd=Retrieve&dopt=full_report&list_uids=5733) | prostaglandin E receptor 3 |
| [Details](http://mirdb.org/cgi-bin/target_detail.cgi?targetID=1440506) | 1231 | 63 | hsa-miR-30c-5p | [CALB2](http://www.ncbi.nlm.nih.gov/entrez/query.fcgi?db=gene&cmd=Retrieve&dopt=full_report&list_uids=794) | calbindin 2 |
| [Details](http://mirdb.org/cgi-bin/target_detail.cgi?targetID=1440539) | 1232 | 63 | hsa-miR-30c-5p | [WDFY3](http://www.ncbi.nlm.nih.gov/entrez/query.fcgi?db=gene&cmd=Retrieve&dopt=full_report&list_uids=23001) | WD repeat and FYVE domain containing 3 |
| [Details](http://mirdb.org/cgi-bin/target_detail.cgi?targetID=1440554) | 1233 | 63 | hsa-miR-30c-5p | [MTF2](http://www.ncbi.nlm.nih.gov/entrez/query.fcgi?db=gene&cmd=Retrieve&dopt=full_report&list_uids=22823) | metal response element binding transcription factor 2 |
| [Details](http://mirdb.org/cgi-bin/target_detail.cgi?targetID=1440587) | 1234 | 63 | hsa-miR-30c-5p | [RGS1](http://www.ncbi.nlm.nih.gov/entrez/query.fcgi?db=gene&cmd=Retrieve&dopt=full_report&list_uids=5996) | regulator of G protein signaling 1 |
| [Details](http://mirdb.org/cgi-bin/target_detail.cgi?targetID=1439470) | 1235 | 62 | hsa-miR-30c-5p | [HMGB3](http://www.ncbi.nlm.nih.gov/entrez/query.fcgi?db=gene&cmd=Retrieve&dopt=full_report&list_uids=3149) | high mobility group box 3 |
| [Details](http://mirdb.org/cgi-bin/target_detail.cgi?targetID=1439574) | 1236 | 62 | hsa-miR-30c-5p | [TRDN](http://www.ncbi.nlm.nih.gov/entrez/query.fcgi?db=gene&cmd=Retrieve&dopt=full_report&list_uids=10345) | triadin |
| [Details](http://mirdb.org/cgi-bin/target_detail.cgi?targetID=1439581) | 1237 | 62 | hsa-miR-30c-5p | [TRIQK](http://www.ncbi.nlm.nih.gov/entrez/query.fcgi?db=gene&cmd=Retrieve&dopt=full_report&list_uids=286144) | triple QxxK/R motif containing |
| [Details](http://mirdb.org/cgi-bin/target_detail.cgi?targetID=1439651) | 1238 | 62 | hsa-miR-30c-5p | [TNIP1](http://www.ncbi.nlm.nih.gov/entrez/query.fcgi?db=gene&cmd=Retrieve&dopt=full_report&list_uids=10318) | TNFAIP3 interacting protein 1 |
| [Details](http://mirdb.org/cgi-bin/target_detail.cgi?targetID=1439664) | 1239 | 62 | hsa-miR-30c-5p | [NCOA3](http://www.ncbi.nlm.nih.gov/entrez/query.fcgi?db=gene&cmd=Retrieve&dopt=full_report&list_uids=8202) | nuclear receptor coactivator 3 |
| [Details](http://mirdb.org/cgi-bin/target_detail.cgi?targetID=1439796) | 1240 | 62 | hsa-miR-30c-5p | [SOX4](http://www.ncbi.nlm.nih.gov/entrez/query.fcgi?db=gene&cmd=Retrieve&dopt=full_report&list_uids=6659) | SRY-box 4 |
| [Details](http://mirdb.org/cgi-bin/target_detail.cgi?targetID=1439824) | 1241 | 62 | hsa-miR-30c-5p | [RAB10](http://www.ncbi.nlm.nih.gov/entrez/query.fcgi?db=gene&cmd=Retrieve&dopt=full_report&list_uids=10890) | RAB10, member RAS oncogene family |
| [Details](http://mirdb.org/cgi-bin/target_detail.cgi?targetID=1439825) | 1242 | 62 | hsa-miR-30c-5p | [GAS2](http://www.ncbi.nlm.nih.gov/entrez/query.fcgi?db=gene&cmd=Retrieve&dopt=full_report&list_uids=2620) | growth arrest specific 2 |
| [Details](http://mirdb.org/cgi-bin/target_detail.cgi?targetID=1439852) | 1243 | 62 | hsa-miR-30c-5p | [RASGEF1A](http://www.ncbi.nlm.nih.gov/entrez/query.fcgi?db=gene&cmd=Retrieve&dopt=full_report&list_uids=221002) | RasGEF domain family member 1A |
| [Details](http://mirdb.org/cgi-bin/target_detail.cgi?targetID=1439887) | 1244 | 62 | hsa-miR-30c-5p | [SOGA3](http://www.ncbi.nlm.nih.gov/entrez/query.fcgi?db=gene&cmd=Retrieve&dopt=full_report&list_uids=387104) | SOGA family member 3 |
| [Details](http://mirdb.org/cgi-bin/target_detail.cgi?targetID=1439900) | 1245 | 62 | hsa-miR-30c-5p | [FAHD1](http://www.ncbi.nlm.nih.gov/entrez/query.fcgi?db=gene&cmd=Retrieve&dopt=full_report&list_uids=81889) | fumarylacetoacetate hydrolase domain containing 1 |
| [Details](http://mirdb.org/cgi-bin/target_detail.cgi?targetID=1439984) | 1246 | 62 | hsa-miR-30c-5p | [ANKRD20A3](http://www.ncbi.nlm.nih.gov/entrez/query.fcgi?db=gene&cmd=Retrieve&dopt=full_report&list_uids=441425) | ankyrin repeat domain 20 family member A3 |
| [Details](http://mirdb.org/cgi-bin/target_detail.cgi?targetID=1439987) | 1247 | 62 | hsa-miR-30c-5p | [ABCB5](http://www.ncbi.nlm.nih.gov/entrez/query.fcgi?db=gene&cmd=Retrieve&dopt=full_report&list_uids=340273) | ATP binding cassette subfamily B member 5 |
| [Details](http://mirdb.org/cgi-bin/target_detail.cgi?targetID=1439989) | 1248 | 62 | hsa-miR-30c-5p | [RGS10](http://www.ncbi.nlm.nih.gov/entrez/query.fcgi?db=gene&cmd=Retrieve&dopt=full_report&list_uids=6001) | regulator of G protein signaling 10 |
| [Details](http://mirdb.org/cgi-bin/target_detail.cgi?targetID=1440011) | 1249 | 62 | hsa-miR-30c-5p | [UBE2F](http://www.ncbi.nlm.nih.gov/entrez/query.fcgi?db=gene&cmd=Retrieve&dopt=full_report&list_uids=140739) | ubiquitin conjugating enzyme E2 F (putative) |
| [Details](http://mirdb.org/cgi-bin/target_detail.cgi?targetID=1440058) | 1250 | 62 | hsa-miR-30c-5p | [AKAP10](http://www.ncbi.nlm.nih.gov/entrez/query.fcgi?db=gene&cmd=Retrieve&dopt=full_report&list_uids=11216) | A-kinase anchoring protein 10 |
| [Details](http://mirdb.org/cgi-bin/target_detail.cgi?targetID=1440170) | 1251 | 62 | hsa-miR-30c-5p | [PATL2](http://www.ncbi.nlm.nih.gov/entrez/query.fcgi?db=gene&cmd=Retrieve&dopt=full_report&list_uids=197135) | PAT1 homolog 2 |
| [Details](http://mirdb.org/cgi-bin/target_detail.cgi?targetID=1440288) | 1252 | 62 | hsa-miR-30c-5p | [PNISR](http://www.ncbi.nlm.nih.gov/entrez/query.fcgi?db=gene&cmd=Retrieve&dopt=full_report&list_uids=25957) | PNN interacting serine and arginine rich protein |
| [Details](http://mirdb.org/cgi-bin/target_detail.cgi?targetID=1440457) | 1253 | 62 | hsa-miR-30c-5p | [PAFAH1B2](http://www.ncbi.nlm.nih.gov/entrez/query.fcgi?db=gene&cmd=Retrieve&dopt=full_report&list_uids=5049) | platelet activating factor acetylhydrolase 1b catalytic subunit 2 |
| [Details](http://mirdb.org/cgi-bin/target_detail.cgi?targetID=1440609) | 1254 | 62 | hsa-miR-30c-5p | [CELF5](http://www.ncbi.nlm.nih.gov/entrez/query.fcgi?db=gene&cmd=Retrieve&dopt=full_report&list_uids=60680) | CUGBP Elav-like family member 5 |
| [Details](http://mirdb.org/cgi-bin/target_detail.cgi?targetID=1440706) | 1255 | 62 | hsa-miR-30c-5p | [MFSD4B](http://www.ncbi.nlm.nih.gov/entrez/query.fcgi?db=gene&cmd=Retrieve&dopt=full_report&list_uids=91749) | major facilitator superfamily domain containing 4B |
| [Details](http://mirdb.org/cgi-bin/target_detail.cgi?targetID=1440821) | 1256 | 62 | hsa-miR-30c-5p | [CALD1](http://www.ncbi.nlm.nih.gov/entrez/query.fcgi?db=gene&cmd=Retrieve&dopt=full_report&list_uids=800) | caldesmon 1 |
| [Details](http://mirdb.org/cgi-bin/target_detail.cgi?targetID=1440834) | 1257 | 62 | hsa-miR-30c-5p | [FOXA1](http://www.ncbi.nlm.nih.gov/entrez/query.fcgi?db=gene&cmd=Retrieve&dopt=full_report&list_uids=3169) | forkhead box A1 |
| [Details](http://mirdb.org/cgi-bin/target_detail.cgi?targetID=1440882) | 1258 | 62 | hsa-miR-30c-5p | [TNFRSF10B](http://www.ncbi.nlm.nih.gov/entrez/query.fcgi?db=gene&cmd=Retrieve&dopt=full_report&list_uids=8795) | TNF receptor superfamily member 10b |
| [Details](http://mirdb.org/cgi-bin/target_detail.cgi?targetID=1439460) | 1259 | 61 | hsa-miR-30c-5p | [NSL1](http://www.ncbi.nlm.nih.gov/entrez/query.fcgi?db=gene&cmd=Retrieve&dopt=full_report&list_uids=25936) | NSL1, MIS12 kinetochore complex component |
| [Details](http://mirdb.org/cgi-bin/target_detail.cgi?targetID=1439473) | 1260 | 61 | hsa-miR-30c-5p | [KIAA1147](http://www.ncbi.nlm.nih.gov/entrez/query.fcgi?db=gene&cmd=Retrieve&dopt=full_report&list_uids=57189) | KIAA1147 |
| [Details](http://mirdb.org/cgi-bin/target_detail.cgi?targetID=1439547) | 1261 | 61 | hsa-miR-30c-5p | [OSBPL3](http://www.ncbi.nlm.nih.gov/entrez/query.fcgi?db=gene&cmd=Retrieve&dopt=full_report&list_uids=26031) | oxysterol binding protein like 3 |
| [Details](http://mirdb.org/cgi-bin/target_detail.cgi?targetID=1439626) | 1262 | 61 | hsa-miR-30c-5p | [DIXDC1](http://www.ncbi.nlm.nih.gov/entrez/query.fcgi?db=gene&cmd=Retrieve&dopt=full_report&list_uids=85458) | DIX domain containing 1 |
| [Details](http://mirdb.org/cgi-bin/target_detail.cgi?targetID=1439704) | 1263 | 61 | hsa-miR-30c-5p | [ABL2](http://www.ncbi.nlm.nih.gov/entrez/query.fcgi?db=gene&cmd=Retrieve&dopt=full_report&list_uids=27) | ABL proto-oncogene 2, non-receptor tyrosine kinase |
| [Details](http://mirdb.org/cgi-bin/target_detail.cgi?targetID=1439731) | 1264 | 61 | hsa-miR-30c-5p | [SMIM11B](http://www.ncbi.nlm.nih.gov/entrez/query.fcgi?db=gene&cmd=Retrieve&dopt=full_report&list_uids=102723553) | small integral membrane protein 11B |
| [Details](http://mirdb.org/cgi-bin/target_detail.cgi?targetID=1439771) | 1265 | 61 | hsa-miR-30c-5p | [NAP1L3](http://www.ncbi.nlm.nih.gov/entrez/query.fcgi?db=gene&cmd=Retrieve&dopt=full_report&list_uids=4675) | nucleosome assembly protein 1 like 3 |
| [Details](http://mirdb.org/cgi-bin/target_detail.cgi?targetID=1439807) | 1266 | 61 | hsa-miR-30c-5p | [LAMC3](http://www.ncbi.nlm.nih.gov/entrez/query.fcgi?db=gene&cmd=Retrieve&dopt=full_report&list_uids=10319) | laminin subunit gamma 3 |
| [Details](http://mirdb.org/cgi-bin/target_detail.cgi?targetID=1439845) | 1267 | 61 | hsa-miR-30c-5p | [ALG6](http://www.ncbi.nlm.nih.gov/entrez/query.fcgi?db=gene&cmd=Retrieve&dopt=full_report&list_uids=29929) | ALG6, alpha-1,3-glucosyltransferase |
| [Details](http://mirdb.org/cgi-bin/target_detail.cgi?targetID=1439975) | 1268 | 61 | hsa-miR-30c-5p | [SLC35D3](http://www.ncbi.nlm.nih.gov/entrez/query.fcgi?db=gene&cmd=Retrieve&dopt=full_report&list_uids=340146) | solute carrier family 35 member D3 |
| [Details](http://mirdb.org/cgi-bin/target_detail.cgi?targetID=1439993) | 1269 | 61 | hsa-miR-30c-5p | [TRIP12](http://www.ncbi.nlm.nih.gov/entrez/query.fcgi?db=gene&cmd=Retrieve&dopt=full_report&list_uids=9320) | thyroid hormone receptor interactor 12 |
| [Details](http://mirdb.org/cgi-bin/target_detail.cgi?targetID=1440127) | 1270 | 61 | hsa-miR-30c-5p | [IFFO2](http://www.ncbi.nlm.nih.gov/entrez/query.fcgi?db=gene&cmd=Retrieve&dopt=full_report&list_uids=126917) | intermediate filament family orphan 2 |
| [Details](http://mirdb.org/cgi-bin/target_detail.cgi?targetID=1440141) | 1271 | 61 | hsa-miR-30c-5p | [CTTNBP2NL](http://www.ncbi.nlm.nih.gov/entrez/query.fcgi?db=gene&cmd=Retrieve&dopt=full_report&list_uids=55917) | CTTNBP2 N-terminal like |
| [Details](http://mirdb.org/cgi-bin/target_detail.cgi?targetID=1440221) | 1272 | 61 | hsa-miR-30c-5p | [STX17](http://www.ncbi.nlm.nih.gov/entrez/query.fcgi?db=gene&cmd=Retrieve&dopt=full_report&list_uids=55014) | syntaxin 17 |
| [Details](http://mirdb.org/cgi-bin/target_detail.cgi?targetID=1440259) | 1273 | 61 | hsa-miR-30c-5p | [ZBTB38](http://www.ncbi.nlm.nih.gov/entrez/query.fcgi?db=gene&cmd=Retrieve&dopt=full_report&list_uids=253461) | zinc finger and BTB domain containing 38 |
| [Details](http://mirdb.org/cgi-bin/target_detail.cgi?targetID=1440312) | 1274 | 61 | hsa-miR-30c-5p | [SAP30](http://www.ncbi.nlm.nih.gov/entrez/query.fcgi?db=gene&cmd=Retrieve&dopt=full_report&list_uids=8819) | Sin3A associated protein 30 |
| [Details](http://mirdb.org/cgi-bin/target_detail.cgi?targetID=1440467) | 1275 | 61 | hsa-miR-30c-5p | [DSCC1](http://www.ncbi.nlm.nih.gov/entrez/query.fcgi?db=gene&cmd=Retrieve&dopt=full_report&list_uids=79075) | DNA replication and sister chromatid cohesion 1 |
| [Details](http://mirdb.org/cgi-bin/target_detail.cgi?targetID=1440490) | 1276 | 61 | hsa-miR-30c-5p | [POLR3G](http://www.ncbi.nlm.nih.gov/entrez/query.fcgi?db=gene&cmd=Retrieve&dopt=full_report&list_uids=10622) | RNA polymerase III subunit G |
| [Details](http://mirdb.org/cgi-bin/target_detail.cgi?targetID=1440582) | 1277 | 61 | hsa-miR-30c-5p | [MFAP3](http://www.ncbi.nlm.nih.gov/entrez/query.fcgi?db=gene&cmd=Retrieve&dopt=full_report&list_uids=4238) | microfibril associated protein 3 |
| [Details](http://mirdb.org/cgi-bin/target_detail.cgi?targetID=1440692) | 1278 | 61 | hsa-miR-30c-5p | [ILDR2](http://www.ncbi.nlm.nih.gov/entrez/query.fcgi?db=gene&cmd=Retrieve&dopt=full_report&list_uids=387597) | immunoglobulin like domain containing receptor 2 |
| [Details](http://mirdb.org/cgi-bin/target_detail.cgi?targetID=1440722) | 1279 | 61 | hsa-miR-30c-5p | [ZDHHC20](http://www.ncbi.nlm.nih.gov/entrez/query.fcgi?db=gene&cmd=Retrieve&dopt=full_report&list_uids=253832) | zinc finger DHHC-type containing 20 |
| [Details](http://mirdb.org/cgi-bin/target_detail.cgi?targetID=1440729) | 1280 | 61 | hsa-miR-30c-5p | [PHF20](http://www.ncbi.nlm.nih.gov/entrez/query.fcgi?db=gene&cmd=Retrieve&dopt=full_report&list_uids=51230) | PHD finger protein 20 |
| [Details](http://mirdb.org/cgi-bin/target_detail.cgi?targetID=1440734) | 1281 | 61 | hsa-miR-30c-5p | [SMIM11A](http://www.ncbi.nlm.nih.gov/entrez/query.fcgi?db=gene&cmd=Retrieve&dopt=full_report&list_uids=54065) | small integral membrane protein 11A |
| [Details](http://mirdb.org/cgi-bin/target_detail.cgi?targetID=1440743) | 1282 | 61 | hsa-miR-30c-5p | [MTX3](http://www.ncbi.nlm.nih.gov/entrez/query.fcgi?db=gene&cmd=Retrieve&dopt=full_report&list_uids=345778) | metaxin 3 |
| [Details](http://mirdb.org/cgi-bin/target_detail.cgi?targetID=1440867) | 1283 | 61 | hsa-miR-30c-5p | [WDR36](http://www.ncbi.nlm.nih.gov/entrez/query.fcgi?db=gene&cmd=Retrieve&dopt=full_report&list_uids=134430) | WD repeat domain 36 |
| [Details](http://mirdb.org/cgi-bin/target_detail.cgi?targetID=1440890) | 1284 | 61 | hsa-miR-30c-5p | [ATRNL1](http://www.ncbi.nlm.nih.gov/entrez/query.fcgi?db=gene&cmd=Retrieve&dopt=full_report&list_uids=26033) | attractin like 1 |
| [Details](http://mirdb.org/cgi-bin/target_detail.cgi?targetID=1440892) | 1285 | 61 | hsa-miR-30c-5p | [GLP1R](http://www.ncbi.nlm.nih.gov/entrez/query.fcgi?db=gene&cmd=Retrieve&dopt=full_report&list_uids=2740) | glucagon like peptide 1 receptor |
| [Details](http://mirdb.org/cgi-bin/target_detail.cgi?targetID=1440932) | 1286 | 61 | hsa-miR-30c-5p | [ERMAP](http://www.ncbi.nlm.nih.gov/entrez/query.fcgi?db=gene&cmd=Retrieve&dopt=full_report&list_uids=114625) | erythroblast membrane associated protein (Scianna blood group) |
| [Details](http://mirdb.org/cgi-bin/target_detail.cgi?targetID=1440991) | 1287 | 61 | hsa-miR-30c-5p | [DEPDC4](http://www.ncbi.nlm.nih.gov/entrez/query.fcgi?db=gene&cmd=Retrieve&dopt=full_report&list_uids=120863) | DEP domain containing 4 |
| [Details](http://mirdb.org/cgi-bin/target_detail.cgi?targetID=1439472) | 1288 | 60 | hsa-miR-30c-5p | [RCOR3](http://www.ncbi.nlm.nih.gov/entrez/query.fcgi?db=gene&cmd=Retrieve&dopt=full_report&list_uids=55758) | REST corepressor 3 |
| [Details](http://mirdb.org/cgi-bin/target_detail.cgi?targetID=1439528) | 1289 | 60 | hsa-miR-30c-5p | [EEPD1](http://www.ncbi.nlm.nih.gov/entrez/query.fcgi?db=gene&cmd=Retrieve&dopt=full_report&list_uids=80820) | endonuclease/exonuclease/phosphatase family domain containing 1 |
| [Details](http://mirdb.org/cgi-bin/target_detail.cgi?targetID=1439633) | 1290 | 60 | hsa-miR-30c-5p | [HAO1](http://www.ncbi.nlm.nih.gov/entrez/query.fcgi?db=gene&cmd=Retrieve&dopt=full_report&list_uids=54363) | hydroxyacid oxidase 1 |
| [Details](http://mirdb.org/cgi-bin/target_detail.cgi?targetID=1439702) | 1291 | 60 | hsa-miR-30c-5p | [NOVA1](http://www.ncbi.nlm.nih.gov/entrez/query.fcgi?db=gene&cmd=Retrieve&dopt=full_report&list_uids=4857) | NOVA alternative splicing regulator 1 |
| [Details](http://mirdb.org/cgi-bin/target_detail.cgi?targetID=1439800) | 1292 | 60 | hsa-miR-30c-5p | [GATA6](http://www.ncbi.nlm.nih.gov/entrez/query.fcgi?db=gene&cmd=Retrieve&dopt=full_report&list_uids=2627) | GATA binding protein 6 |
| [Details](http://mirdb.org/cgi-bin/target_detail.cgi?targetID=1439805) | 1293 | 60 | hsa-miR-30c-5p | [SEC24D](http://www.ncbi.nlm.nih.gov/entrez/query.fcgi?db=gene&cmd=Retrieve&dopt=full_report&list_uids=9871) | SEC24 homolog D, COPII coat complex component |
| [Details](http://mirdb.org/cgi-bin/target_detail.cgi?targetID=1439839) | 1294 | 60 | hsa-miR-30c-5p | [BACH1](http://www.ncbi.nlm.nih.gov/entrez/query.fcgi?db=gene&cmd=Retrieve&dopt=full_report&list_uids=571) | BTB domain and CNC homolog 1 |
| [Details](http://mirdb.org/cgi-bin/target_detail.cgi?targetID=1439841) | 1295 | 60 | hsa-miR-30c-5p | [YIPF6](http://www.ncbi.nlm.nih.gov/entrez/query.fcgi?db=gene&cmd=Retrieve&dopt=full_report&list_uids=286451) | Yip1 domain family member 6 |
| [Details](http://mirdb.org/cgi-bin/target_detail.cgi?targetID=1439868) | 1296 | 60 | hsa-miR-30c-5p | [NEIL2](http://www.ncbi.nlm.nih.gov/entrez/query.fcgi?db=gene&cmd=Retrieve&dopt=full_report&list_uids=252969) | nei like DNA glycosylase 2 |
| [Details](http://mirdb.org/cgi-bin/target_detail.cgi?targetID=1439894) | 1297 | 60 | hsa-miR-30c-5p | [DYNLT3](http://www.ncbi.nlm.nih.gov/entrez/query.fcgi?db=gene&cmd=Retrieve&dopt=full_report&list_uids=6990) | dynein light chain Tctex-type 3 |
| [Details](http://mirdb.org/cgi-bin/target_detail.cgi?targetID=1439955) | 1298 | 60 | hsa-miR-30c-5p | [DMD](http://www.ncbi.nlm.nih.gov/entrez/query.fcgi?db=gene&cmd=Retrieve&dopt=full_report&list_uids=1756) | dystrophin |
| [Details](http://mirdb.org/cgi-bin/target_detail.cgi?targetID=1440072) | 1299 | 60 | hsa-miR-30c-5p | [KIAA1958](http://www.ncbi.nlm.nih.gov/entrez/query.fcgi?db=gene&cmd=Retrieve&dopt=full_report&list_uids=158405) | KIAA1958 |
| [Details](http://mirdb.org/cgi-bin/target_detail.cgi?targetID=1440139) | 1300 | 60 | hsa-miR-30c-5p | [HYKK](http://www.ncbi.nlm.nih.gov/entrez/query.fcgi?db=gene&cmd=Retrieve&dopt=full_report&list_uids=123688) | hydroxylysine kinase |
| [Details](http://mirdb.org/cgi-bin/target_detail.cgi?targetID=1440208) | 1301 | 60 | hsa-miR-30c-5p | [ADRB1](http://www.ncbi.nlm.nih.gov/entrez/query.fcgi?db=gene&cmd=Retrieve&dopt=full_report&list_uids=153) | adrenoceptor beta 1 |
| [Details](http://mirdb.org/cgi-bin/target_detail.cgi?targetID=1440219) | 1302 | 60 | hsa-miR-30c-5p | [C11orf21](http://www.ncbi.nlm.nih.gov/entrez/query.fcgi?db=gene&cmd=Retrieve&dopt=full_report&list_uids=29125) | chromosome 11 open reading frame 21 |
| [Details](http://mirdb.org/cgi-bin/target_detail.cgi?targetID=1440222) | 1303 | 60 | hsa-miR-30c-5p | [USP46](http://www.ncbi.nlm.nih.gov/entrez/query.fcgi?db=gene&cmd=Retrieve&dopt=full_report&list_uids=64854) | ubiquitin specific peptidase 46 |
| [Details](http://mirdb.org/cgi-bin/target_detail.cgi?targetID=1440573) | 1304 | 60 | hsa-miR-30c-5p | [PNPT1](http://www.ncbi.nlm.nih.gov/entrez/query.fcgi?db=gene&cmd=Retrieve&dopt=full_report&list_uids=87178) | polyribonucleotide nucleotidyltransferase 1 |
| [Details](http://mirdb.org/cgi-bin/target_detail.cgi?targetID=1440621) | 1305 | 60 | hsa-miR-30c-5p | [MTA1](http://www.ncbi.nlm.nih.gov/entrez/query.fcgi?db=gene&cmd=Retrieve&dopt=full_report&list_uids=9112) | metastasis associated 1 |
| [Details](http://mirdb.org/cgi-bin/target_detail.cgi?targetID=1440628) | 1306 | 60 | hsa-miR-30c-5p | [ACTN1](http://www.ncbi.nlm.nih.gov/entrez/query.fcgi?db=gene&cmd=Retrieve&dopt=full_report&list_uids=87) | actinin alpha 1 |
| [Details](http://mirdb.org/cgi-bin/target_detail.cgi?targetID=1440744) | 1307 | 60 | hsa-miR-30c-5p | [SBK1](http://www.ncbi.nlm.nih.gov/entrez/query.fcgi?db=gene&cmd=Retrieve&dopt=full_report&list_uids=388228) | SH3 domain binding kinase 1 |
| [Details](http://mirdb.org/cgi-bin/target_detail.cgi?targetID=1440768) | 1308 | 60 | hsa-miR-30c-5p | [KCTD9](http://www.ncbi.nlm.nih.gov/entrez/query.fcgi?db=gene&cmd=Retrieve&dopt=full_report&list_uids=54793) | potassium channel tetramerization domain containing 9 |
| [Details](http://mirdb.org/cgi-bin/target_detail.cgi?targetID=1440835) | 1309 | 60 | hsa-miR-30c-5p | [AAK1](http://www.ncbi.nlm.nih.gov/entrez/query.fcgi?db=gene&cmd=Retrieve&dopt=full_report&list_uids=22848) | AP2 associated kinase 1 |
| [Details](http://mirdb.org/cgi-bin/target_detail.cgi?targetID=1440914) | 1310 | 60 | hsa-miR-30c-5p | [ZNF827](http://www.ncbi.nlm.nih.gov/entrez/query.fcgi?db=gene&cmd=Retrieve&dopt=full_report&list_uids=152485) | zinc finger protein 827 |
| [Details](http://mirdb.org/cgi-bin/target_detail.cgi?targetID=1439499) | 1311 | 59 | hsa-miR-30c-5p | [CACNA1C](http://www.ncbi.nlm.nih.gov/entrez/query.fcgi?db=gene&cmd=Retrieve&dopt=full_report&list_uids=775) | calcium voltage-gated channel subunit alpha1 C |
| [Details](http://mirdb.org/cgi-bin/target_detail.cgi?targetID=1439546) | 1312 | 59 | hsa-miR-30c-5p | [LRRTM2](http://www.ncbi.nlm.nih.gov/entrez/query.fcgi?db=gene&cmd=Retrieve&dopt=full_report&list_uids=26045) | leucine rich repeat transmembrane neuronal 2 |
| [Details](http://mirdb.org/cgi-bin/target_detail.cgi?targetID=1439578) | 1313 | 59 | hsa-miR-30c-5p | [CCN3](http://www.ncbi.nlm.nih.gov/entrez/query.fcgi?db=gene&cmd=Retrieve&dopt=full_report&list_uids=4856) | cellular communication network factor 3 |
| [Details](http://mirdb.org/cgi-bin/target_detail.cgi?targetID=1439580) | 1314 | 59 | hsa-miR-30c-5p | [CHORDC1](http://www.ncbi.nlm.nih.gov/entrez/query.fcgi?db=gene&cmd=Retrieve&dopt=full_report&list_uids=26973) | cysteine and histidine rich domain containing 1 |
| [Details](http://mirdb.org/cgi-bin/target_detail.cgi?targetID=1439595) | 1315 | 59 | hsa-miR-30c-5p | [RAB3D](http://www.ncbi.nlm.nih.gov/entrez/query.fcgi?db=gene&cmd=Retrieve&dopt=full_report&list_uids=9545) | RAB3D, member RAS oncogene family |
| [Details](http://mirdb.org/cgi-bin/target_detail.cgi?targetID=1439705) | 1316 | 59 | hsa-miR-30c-5p | [TCAF2](http://www.ncbi.nlm.nih.gov/entrez/query.fcgi?db=gene&cmd=Retrieve&dopt=full_report&list_uids=285966) | TRPM8 channel associated factor 2 |
| [Details](http://mirdb.org/cgi-bin/target_detail.cgi?targetID=1439862) | 1317 | 59 | hsa-miR-30c-5p | [CCDC14](http://www.ncbi.nlm.nih.gov/entrez/query.fcgi?db=gene&cmd=Retrieve&dopt=full_report&list_uids=64770) | coiled-coil domain containing 14 |
| [Details](http://mirdb.org/cgi-bin/target_detail.cgi?targetID=1439870) | 1318 | 59 | hsa-miR-30c-5p | [SMIM14](http://www.ncbi.nlm.nih.gov/entrez/query.fcgi?db=gene&cmd=Retrieve&dopt=full_report&list_uids=201895) | small integral membrane protein 14 |
| [Details](http://mirdb.org/cgi-bin/target_detail.cgi?targetID=1440031) | 1319 | 59 | hsa-miR-30c-5p | [SLAIN1](http://www.ncbi.nlm.nih.gov/entrez/query.fcgi?db=gene&cmd=Retrieve&dopt=full_report&list_uids=122060) | SLAIN motif family member 1 |
| [Details](http://mirdb.org/cgi-bin/target_detail.cgi?targetID=1440062) | 1320 | 59 | hsa-miR-30c-5p | [MICOS10](http://www.ncbi.nlm.nih.gov/entrez/query.fcgi?db=gene&cmd=Retrieve&dopt=full_report&list_uids=440574) | mitochondrial contact site and cristae organizing system subunit 10 |
| [Details](http://mirdb.org/cgi-bin/target_detail.cgi?targetID=1440102) | 1321 | 59 | hsa-miR-30c-5p | [RIMS1](http://www.ncbi.nlm.nih.gov/entrez/query.fcgi?db=gene&cmd=Retrieve&dopt=full_report&list_uids=22999) | regulating synaptic membrane exocytosis 1 |
| [Details](http://mirdb.org/cgi-bin/target_detail.cgi?targetID=1440243) | 1322 | 59 | hsa-miR-30c-5p | [NSD1](http://www.ncbi.nlm.nih.gov/entrez/query.fcgi?db=gene&cmd=Retrieve&dopt=full_report&list_uids=64324) | nuclear receptor binding SET domain protein 1 |
| [Details](http://mirdb.org/cgi-bin/target_detail.cgi?targetID=1440299) | 1323 | 59 | hsa-miR-30c-5p | [GCG](http://www.ncbi.nlm.nih.gov/entrez/query.fcgi?db=gene&cmd=Retrieve&dopt=full_report&list_uids=2641) | glucagon |
| [Details](http://mirdb.org/cgi-bin/target_detail.cgi?targetID=1440368) | 1324 | 59 | hsa-miR-30c-5p | [MAP7D3](http://www.ncbi.nlm.nih.gov/entrez/query.fcgi?db=gene&cmd=Retrieve&dopt=full_report&list_uids=79649) | MAP7 domain containing 3 |
| [Details](http://mirdb.org/cgi-bin/target_detail.cgi?targetID=1440493) | 1325 | 59 | hsa-miR-30c-5p | [ATP8A1](http://www.ncbi.nlm.nih.gov/entrez/query.fcgi?db=gene&cmd=Retrieve&dopt=full_report&list_uids=10396) | ATPase phospholipid transporting 8A1 |
| [Details](http://mirdb.org/cgi-bin/target_detail.cgi?targetID=1440535) | 1326 | 59 | hsa-miR-30c-5p | [CCNY](http://www.ncbi.nlm.nih.gov/entrez/query.fcgi?db=gene&cmd=Retrieve&dopt=full_report&list_uids=219771) | cyclin Y |
| [Details](http://mirdb.org/cgi-bin/target_detail.cgi?targetID=1440537) | 1327 | 59 | hsa-miR-30c-5p | [YBX3](http://www.ncbi.nlm.nih.gov/entrez/query.fcgi?db=gene&cmd=Retrieve&dopt=full_report&list_uids=8531) | Y-box binding protein 3 |
| [Details](http://mirdb.org/cgi-bin/target_detail.cgi?targetID=1440544) | 1328 | 59 | hsa-miR-30c-5p | [CPEB2](http://www.ncbi.nlm.nih.gov/entrez/query.fcgi?db=gene&cmd=Retrieve&dopt=full_report&list_uids=132864) | cytoplasmic polyadenylation element binding protein 2 |
| [Details](http://mirdb.org/cgi-bin/target_detail.cgi?targetID=1440647) | 1329 | 59 | hsa-miR-30c-5p | [PIP5K1B](http://www.ncbi.nlm.nih.gov/entrez/query.fcgi?db=gene&cmd=Retrieve&dopt=full_report&list_uids=8395) | phosphatidylinositol-4-phosphate 5-kinase type 1 beta |
| [Details](http://mirdb.org/cgi-bin/target_detail.cgi?targetID=1440832) | 1330 | 59 | hsa-miR-30c-5p | [PITX1](http://www.ncbi.nlm.nih.gov/entrez/query.fcgi?db=gene&cmd=Retrieve&dopt=full_report&list_uids=5307) | paired like homeodomain 1 |
| [Details](http://mirdb.org/cgi-bin/target_detail.cgi?targetID=1440875) | 1331 | 59 | hsa-miR-30c-5p | [JAG2](http://www.ncbi.nlm.nih.gov/entrez/query.fcgi?db=gene&cmd=Retrieve&dopt=full_report&list_uids=3714) | jagged 2 |
| [Details](http://mirdb.org/cgi-bin/target_detail.cgi?targetID=1440894) | 1332 | 59 | hsa-miR-30c-5p | [SERPINE1](http://www.ncbi.nlm.nih.gov/entrez/query.fcgi?db=gene&cmd=Retrieve&dopt=full_report&list_uids=5054) | serpin family E member 1 |
| [Details](http://mirdb.org/cgi-bin/target_detail.cgi?targetID=1440902) | 1333 | 59 | hsa-miR-30c-5p | [ASAP1](http://www.ncbi.nlm.nih.gov/entrez/query.fcgi?db=gene&cmd=Retrieve&dopt=full_report&list_uids=50807) | ArfGAP with SH3 domain, ankyrin repeat and PH domain 1 |
| [Details](http://mirdb.org/cgi-bin/target_detail.cgi?targetID=1439487) | 1334 | 58 | hsa-miR-30c-5p | [ATG2B](http://www.ncbi.nlm.nih.gov/entrez/query.fcgi?db=gene&cmd=Retrieve&dopt=full_report&list_uids=55102) | autophagy related 2B |
| [Details](http://mirdb.org/cgi-bin/target_detail.cgi?targetID=1439555) | 1335 | 58 | hsa-miR-30c-5p | [GATA5](http://www.ncbi.nlm.nih.gov/entrez/query.fcgi?db=gene&cmd=Retrieve&dopt=full_report&list_uids=140628) | GATA binding protein 5 |
| [Details](http://mirdb.org/cgi-bin/target_detail.cgi?targetID=1439571) | 1336 | 58 | hsa-miR-30c-5p | [GPATCH2L](http://www.ncbi.nlm.nih.gov/entrez/query.fcgi?db=gene&cmd=Retrieve&dopt=full_report&list_uids=55668) | G-patch domain containing 2 like |
| [Details](http://mirdb.org/cgi-bin/target_detail.cgi?targetID=1439579) | 1337 | 58 | hsa-miR-30c-5p | [ACAP2](http://www.ncbi.nlm.nih.gov/entrez/query.fcgi?db=gene&cmd=Retrieve&dopt=full_report&list_uids=23527) | ArfGAP with coiled-coil, ankyrin repeat and PH domains 2 |
| [Details](http://mirdb.org/cgi-bin/target_detail.cgi?targetID=1439583) | 1338 | 58 | hsa-miR-30c-5p | [BAZ1A](http://www.ncbi.nlm.nih.gov/entrez/query.fcgi?db=gene&cmd=Retrieve&dopt=full_report&list_uids=11177) | bromodomain adjacent to zinc finger domain 1A |
| [Details](http://mirdb.org/cgi-bin/target_detail.cgi?targetID=1439849) | 1339 | 58 | hsa-miR-30c-5p | [GNAQ](http://www.ncbi.nlm.nih.gov/entrez/query.fcgi?db=gene&cmd=Retrieve&dopt=full_report&list_uids=2776) | G protein subunit alpha q |
| [Details](http://mirdb.org/cgi-bin/target_detail.cgi?targetID=1439944) | 1340 | 58 | hsa-miR-30c-5p | [ZNF100](http://www.ncbi.nlm.nih.gov/entrez/query.fcgi?db=gene&cmd=Retrieve&dopt=full_report&list_uids=163227) | zinc finger protein 100 |
| [Details](http://mirdb.org/cgi-bin/target_detail.cgi?targetID=1439962) | 1341 | 58 | hsa-miR-30c-5p | [CCDC80](http://www.ncbi.nlm.nih.gov/entrez/query.fcgi?db=gene&cmd=Retrieve&dopt=full_report&list_uids=151887) | coiled-coil domain containing 80 |
| [Details](http://mirdb.org/cgi-bin/target_detail.cgi?targetID=1440066) | 1342 | 58 | hsa-miR-30c-5p | [LHX9](http://www.ncbi.nlm.nih.gov/entrez/query.fcgi?db=gene&cmd=Retrieve&dopt=full_report&list_uids=56956) | LIM homeobox 9 |
| [Details](http://mirdb.org/cgi-bin/target_detail.cgi?targetID=1440113) | 1343 | 58 | hsa-miR-30c-5p | [TSPAN33](http://www.ncbi.nlm.nih.gov/entrez/query.fcgi?db=gene&cmd=Retrieve&dopt=full_report&list_uids=340348) | tetraspanin 33 |
| [Details](http://mirdb.org/cgi-bin/target_detail.cgi?targetID=1440119) | 1344 | 58 | hsa-miR-30c-5p | [MOCS3](http://www.ncbi.nlm.nih.gov/entrez/query.fcgi?db=gene&cmd=Retrieve&dopt=full_report&list_uids=27304) | molybdenum cofactor synthesis 3 |
| [Details](http://mirdb.org/cgi-bin/target_detail.cgi?targetID=1440128) | 1345 | 58 | hsa-miR-30c-5p | [STAT1](http://www.ncbi.nlm.nih.gov/entrez/query.fcgi?db=gene&cmd=Retrieve&dopt=full_report&list_uids=6772) | signal transducer and activator of transcription 1 |
| [Details](http://mirdb.org/cgi-bin/target_detail.cgi?targetID=1440256) | 1346 | 58 | hsa-miR-30c-5p | [USP15](http://www.ncbi.nlm.nih.gov/entrez/query.fcgi?db=gene&cmd=Retrieve&dopt=full_report&list_uids=9958) | ubiquitin specific peptidase 15 |
| [Details](http://mirdb.org/cgi-bin/target_detail.cgi?targetID=1440338) | 1347 | 58 | hsa-miR-30c-5p | [MCC](http://www.ncbi.nlm.nih.gov/entrez/query.fcgi?db=gene&cmd=Retrieve&dopt=full_report&list_uids=4163) | MCC, WNT signaling pathway regulator |
| [Details](http://mirdb.org/cgi-bin/target_detail.cgi?targetID=1440635) | 1348 | 58 | hsa-miR-30c-5p | [CCDC148](http://www.ncbi.nlm.nih.gov/entrez/query.fcgi?db=gene&cmd=Retrieve&dopt=full_report&list_uids=130940) | coiled-coil domain containing 148 |
| [Details](http://mirdb.org/cgi-bin/target_detail.cgi?targetID=1440829) | 1349 | 58 | hsa-miR-30c-5p | [PHETA1](http://www.ncbi.nlm.nih.gov/entrez/query.fcgi?db=gene&cmd=Retrieve&dopt=full_report&list_uids=144717) | PH domain containing endocytic trafficking adaptor 1 |
| [Details](http://mirdb.org/cgi-bin/target_detail.cgi?targetID=1440886) | 1350 | 58 | hsa-miR-30c-5p | [CLIP4](http://www.ncbi.nlm.nih.gov/entrez/query.fcgi?db=gene&cmd=Retrieve&dopt=full_report&list_uids=79745) | CAP-Gly domain containing linker protein family member 4 |
| [Details](http://mirdb.org/cgi-bin/target_detail.cgi?targetID=1439455) | 1351 | 57 | hsa-miR-30c-5p | [TGM5](http://www.ncbi.nlm.nih.gov/entrez/query.fcgi?db=gene&cmd=Retrieve&dopt=full_report&list_uids=9333) | transglutaminase 5 |
| [Details](http://mirdb.org/cgi-bin/target_detail.cgi?targetID=1439592) | 1352 | 57 | hsa-miR-30c-5p | [GKN2](http://www.ncbi.nlm.nih.gov/entrez/query.fcgi?db=gene&cmd=Retrieve&dopt=full_report&list_uids=200504) | gastrokine 2 |
| [Details](http://mirdb.org/cgi-bin/target_detail.cgi?targetID=1439724) | 1353 | 57 | hsa-miR-30c-5p | [C8orf44-SGK3](http://www.ncbi.nlm.nih.gov/entrez/query.fcgi?db=gene&cmd=Retrieve&dopt=full_report&list_uids=100533105) | C8orf44-SGK3 readthrough |
| [Details](http://mirdb.org/cgi-bin/target_detail.cgi?targetID=1439768) | 1354 | 57 | hsa-miR-30c-5p | [SF3B1](http://www.ncbi.nlm.nih.gov/entrez/query.fcgi?db=gene&cmd=Retrieve&dopt=full_report&list_uids=23451) | splicing factor 3b subunit 1 |
| [Details](http://mirdb.org/cgi-bin/target_detail.cgi?targetID=1439809) | 1355 | 57 | hsa-miR-30c-5p | [N4BP2](http://www.ncbi.nlm.nih.gov/entrez/query.fcgi?db=gene&cmd=Retrieve&dopt=full_report&list_uids=55728) | NEDD4 binding protein 2 |
| [Details](http://mirdb.org/cgi-bin/target_detail.cgi?targetID=1439822) | 1356 | 57 | hsa-miR-30c-5p | [ATP8B2](http://www.ncbi.nlm.nih.gov/entrez/query.fcgi?db=gene&cmd=Retrieve&dopt=full_report&list_uids=57198) | ATPase phospholipid transporting 8B2 |
| [Details](http://mirdb.org/cgi-bin/target_detail.cgi?targetID=1439871) | 1357 | 57 | hsa-miR-30c-5p | [PGBD2](http://www.ncbi.nlm.nih.gov/entrez/query.fcgi?db=gene&cmd=Retrieve&dopt=full_report&list_uids=267002) | piggyBac transposable element derived 2 |
| [Details](http://mirdb.org/cgi-bin/target_detail.cgi?targetID=1439905) | 1358 | 57 | hsa-miR-30c-5p | [AGO2](http://www.ncbi.nlm.nih.gov/entrez/query.fcgi?db=gene&cmd=Retrieve&dopt=full_report&list_uids=27161) | argonaute RISC catalytic component 2 |
| [Details](http://mirdb.org/cgi-bin/target_detail.cgi?targetID=1439966) | 1359 | 57 | hsa-miR-30c-5p | [PPM1E](http://www.ncbi.nlm.nih.gov/entrez/query.fcgi?db=gene&cmd=Retrieve&dopt=full_report&list_uids=22843) | protein phosphatase, Mg2+/Mn2+ dependent 1E |
| [Details](http://mirdb.org/cgi-bin/target_detail.cgi?targetID=1440123) | 1360 | 57 | hsa-miR-30c-5p | [NAA35](http://www.ncbi.nlm.nih.gov/entrez/query.fcgi?db=gene&cmd=Retrieve&dopt=full_report&list_uids=60560) | N(alpha)-acetyltransferase 35, NatC auxiliary subunit |
| [Details](http://mirdb.org/cgi-bin/target_detail.cgi?targetID=1440218) | 1361 | 57 | hsa-miR-30c-5p | [JAM2](http://www.ncbi.nlm.nih.gov/entrez/query.fcgi?db=gene&cmd=Retrieve&dopt=full_report&list_uids=58494) | junctional adhesion molecule 2 |
| [Details](http://mirdb.org/cgi-bin/target_detail.cgi?targetID=1440246) | 1362 | 57 | hsa-miR-30c-5p | [TRO](http://www.ncbi.nlm.nih.gov/entrez/query.fcgi?db=gene&cmd=Retrieve&dopt=full_report&list_uids=7216) | trophinin |
| [Details](http://mirdb.org/cgi-bin/target_detail.cgi?targetID=1440250) | 1363 | 57 | hsa-miR-30c-5p | [ZNF510](http://www.ncbi.nlm.nih.gov/entrez/query.fcgi?db=gene&cmd=Retrieve&dopt=full_report&list_uids=22869) | zinc finger protein 510 |
| [Details](http://mirdb.org/cgi-bin/target_detail.cgi?targetID=1440255) | 1364 | 57 | hsa-miR-30c-5p | [FBXO28](http://www.ncbi.nlm.nih.gov/entrez/query.fcgi?db=gene&cmd=Retrieve&dopt=full_report&list_uids=23219) | F-box protein 28 |
| [Details](http://mirdb.org/cgi-bin/target_detail.cgi?targetID=1440298) | 1365 | 57 | hsa-miR-30c-5p | [GZF1](http://www.ncbi.nlm.nih.gov/entrez/query.fcgi?db=gene&cmd=Retrieve&dopt=full_report&list_uids=64412) | GDNF inducible zinc finger protein 1 |
| [Details](http://mirdb.org/cgi-bin/target_detail.cgi?targetID=1440339) | 1366 | 57 | hsa-miR-30c-5p | [CCL19](http://www.ncbi.nlm.nih.gov/entrez/query.fcgi?db=gene&cmd=Retrieve&dopt=full_report&list_uids=6363) | C-C motif chemokine ligand 19 |
| [Details](http://mirdb.org/cgi-bin/target_detail.cgi?targetID=1440357) | 1367 | 57 | hsa-miR-30c-5p | [PLIN1](http://www.ncbi.nlm.nih.gov/entrez/query.fcgi?db=gene&cmd=Retrieve&dopt=full_report&list_uids=5346) | perilipin 1 |
| [Details](http://mirdb.org/cgi-bin/target_detail.cgi?targetID=1440394) | 1368 | 57 | hsa-miR-30c-5p | [TLL1](http://www.ncbi.nlm.nih.gov/entrez/query.fcgi?db=gene&cmd=Retrieve&dopt=full_report&list_uids=7092) | tolloid like 1 |
| [Details](http://mirdb.org/cgi-bin/target_detail.cgi?targetID=1440431) | 1369 | 57 | hsa-miR-30c-5p | [COLCA1](http://www.ncbi.nlm.nih.gov/entrez/query.fcgi?db=gene&cmd=Retrieve&dopt=full_report&list_uids=399948) | colorectal cancer associated 1 |
| [Details](http://mirdb.org/cgi-bin/target_detail.cgi?targetID=1440492) | 1370 | 57 | hsa-miR-30c-5p | [FAM126A](http://www.ncbi.nlm.nih.gov/entrez/query.fcgi?db=gene&cmd=Retrieve&dopt=full_report&list_uids=84668) | family with sequence similarity 126 member A |
| [Details](http://mirdb.org/cgi-bin/target_detail.cgi?targetID=1440603) | 1371 | 57 | hsa-miR-30c-5p | [CEACAM1](http://www.ncbi.nlm.nih.gov/entrez/query.fcgi?db=gene&cmd=Retrieve&dopt=full_report&list_uids=634) | carcinoembryonic antigen related cell adhesion molecule 1 |
| [Details](http://mirdb.org/cgi-bin/target_detail.cgi?targetID=1440726) | 1372 | 57 | hsa-miR-30c-5p | [FOXJ2](http://www.ncbi.nlm.nih.gov/entrez/query.fcgi?db=gene&cmd=Retrieve&dopt=full_report&list_uids=55810) | forkhead box J2 |
| [Details](http://mirdb.org/cgi-bin/target_detail.cgi?targetID=1440777) | 1373 | 57 | hsa-miR-30c-5p | [PKNOX1](http://www.ncbi.nlm.nih.gov/entrez/query.fcgi?db=gene&cmd=Retrieve&dopt=full_report&list_uids=5316) | PBX/knotted 1 homeobox 1 |
| [Details](http://mirdb.org/cgi-bin/target_detail.cgi?targetID=1440966) | 1374 | 57 | hsa-miR-30c-5p | [RANBP10](http://www.ncbi.nlm.nih.gov/entrez/query.fcgi?db=gene&cmd=Retrieve&dopt=full_report&list_uids=57610) | RAN binding protein 10 |
| [Details](http://mirdb.org/cgi-bin/target_detail.cgi?targetID=1439494) | 1375 | 56 | hsa-miR-30c-5p | [ANKRD22](http://www.ncbi.nlm.nih.gov/entrez/query.fcgi?db=gene&cmd=Retrieve&dopt=full_report&list_uids=118932) | ankyrin repeat domain 22 |
| [Details](http://mirdb.org/cgi-bin/target_detail.cgi?targetID=1439549) | 1376 | 56 | hsa-miR-30c-5p | [SLC25A14](http://www.ncbi.nlm.nih.gov/entrez/query.fcgi?db=gene&cmd=Retrieve&dopt=full_report&list_uids=9016) | solute carrier family 25 member 14 |
| [Details](http://mirdb.org/cgi-bin/target_detail.cgi?targetID=1439668) | 1377 | 56 | hsa-miR-30c-5p | [MYSM1](http://www.ncbi.nlm.nih.gov/entrez/query.fcgi?db=gene&cmd=Retrieve&dopt=full_report&list_uids=114803) | Myb like, SWIRM and MPN domains 1 |
| [Details](http://mirdb.org/cgi-bin/target_detail.cgi?targetID=1439699) | 1378 | 56 | hsa-miR-30c-5p | [GNAO1](http://www.ncbi.nlm.nih.gov/entrez/query.fcgi?db=gene&cmd=Retrieve&dopt=full_report&list_uids=2775) | G protein subunit alpha o1 |
| [Details](http://mirdb.org/cgi-bin/target_detail.cgi?targetID=1439874) | 1379 | 56 | hsa-miR-30c-5p | [TEX2](http://www.ncbi.nlm.nih.gov/entrez/query.fcgi?db=gene&cmd=Retrieve&dopt=full_report&list_uids=55852) | testis expressed 2 |
| [Details](http://mirdb.org/cgi-bin/target_detail.cgi?targetID=1439877) | 1380 | 56 | hsa-miR-30c-5p | [CNR1](http://www.ncbi.nlm.nih.gov/entrez/query.fcgi?db=gene&cmd=Retrieve&dopt=full_report&list_uids=1268) | cannabinoid receptor 1 |
| [Details](http://mirdb.org/cgi-bin/target_detail.cgi?targetID=1439910) | 1381 | 56 | hsa-miR-30c-5p | [MTHFSD](http://www.ncbi.nlm.nih.gov/entrez/query.fcgi?db=gene&cmd=Retrieve&dopt=full_report&list_uids=64779) | methenyltetrahydrofolate synthetase domain containing |
| [Details](http://mirdb.org/cgi-bin/target_detail.cgi?targetID=1439916) | 1382 | 56 | hsa-miR-30c-5p | [ADAM10](http://www.ncbi.nlm.nih.gov/entrez/query.fcgi?db=gene&cmd=Retrieve&dopt=full_report&list_uids=102) | ADAM metallopeptidase domain 10 |
| [Details](http://mirdb.org/cgi-bin/target_detail.cgi?targetID=1439917) | 1383 | 56 | hsa-miR-30c-5p | [ZNF200](http://www.ncbi.nlm.nih.gov/entrez/query.fcgi?db=gene&cmd=Retrieve&dopt=full_report&list_uids=7752) | zinc finger protein 200 |
| [Details](http://mirdb.org/cgi-bin/target_detail.cgi?targetID=1439931) | 1384 | 56 | hsa-miR-30c-5p | [EPC1](http://www.ncbi.nlm.nih.gov/entrez/query.fcgi?db=gene&cmd=Retrieve&dopt=full_report&list_uids=80314) | enhancer of polycomb homolog 1 |
| [Details](http://mirdb.org/cgi-bin/target_detail.cgi?targetID=1439934) | 1385 | 56 | hsa-miR-30c-5p | [KCNJ12](http://www.ncbi.nlm.nih.gov/entrez/query.fcgi?db=gene&cmd=Retrieve&dopt=full_report&list_uids=3768) | potassium voltage-gated channel subfamily J member 12 |
| [Details](http://mirdb.org/cgi-bin/target_detail.cgi?targetID=1439943) | 1386 | 56 | hsa-miR-30c-5p | [SCRN3](http://www.ncbi.nlm.nih.gov/entrez/query.fcgi?db=gene&cmd=Retrieve&dopt=full_report&list_uids=79634) | secernin 3 |
| [Details](http://mirdb.org/cgi-bin/target_detail.cgi?targetID=1440080) | 1387 | 56 | hsa-miR-30c-5p | [MINPP1](http://www.ncbi.nlm.nih.gov/entrez/query.fcgi?db=gene&cmd=Retrieve&dopt=full_report&list_uids=9562) | multiple inositol-polyphosphate phosphatase 1 |
| [Details](http://mirdb.org/cgi-bin/target_detail.cgi?targetID=1440095) | 1388 | 56 | hsa-miR-30c-5p | [L3MBTL4](http://www.ncbi.nlm.nih.gov/entrez/query.fcgi?db=gene&cmd=Retrieve&dopt=full_report&list_uids=91133) | L3MBTL4, histone methyl-lysine binding protein |
| [Details](http://mirdb.org/cgi-bin/target_detail.cgi?targetID=1440195) | 1389 | 56 | hsa-miR-30c-5p | [TMEM35A](http://www.ncbi.nlm.nih.gov/entrez/query.fcgi?db=gene&cmd=Retrieve&dopt=full_report&list_uids=59353) | transmembrane protein 35A |
| [Details](http://mirdb.org/cgi-bin/target_detail.cgi?targetID=1440291) | 1390 | 56 | hsa-miR-30c-5p | [SLC25A37](http://www.ncbi.nlm.nih.gov/entrez/query.fcgi?db=gene&cmd=Retrieve&dopt=full_report&list_uids=51312) | solute carrier family 25 member 37 |
| [Details](http://mirdb.org/cgi-bin/target_detail.cgi?targetID=1440333) | 1391 | 56 | hsa-miR-30c-5p | [NEBL](http://www.ncbi.nlm.nih.gov/entrez/query.fcgi?db=gene&cmd=Retrieve&dopt=full_report&list_uids=10529) | nebulette |
| [Details](http://mirdb.org/cgi-bin/target_detail.cgi?targetID=1440349) | 1392 | 56 | hsa-miR-30c-5p | [GUCY2C](http://www.ncbi.nlm.nih.gov/entrez/query.fcgi?db=gene&cmd=Retrieve&dopt=full_report&list_uids=2984) | guanylate cyclase 2C |
| [Details](http://mirdb.org/cgi-bin/target_detail.cgi?targetID=1440387) | 1393 | 56 | hsa-miR-30c-5p | [CENPQ](http://www.ncbi.nlm.nih.gov/entrez/query.fcgi?db=gene&cmd=Retrieve&dopt=full_report&list_uids=55166) | centromere protein Q |
| [Details](http://mirdb.org/cgi-bin/target_detail.cgi?targetID=1440406) | 1394 | 56 | hsa-miR-30c-5p | [ZNF585A](http://www.ncbi.nlm.nih.gov/entrez/query.fcgi?db=gene&cmd=Retrieve&dopt=full_report&list_uids=199704) | zinc finger protein 585A |
| [Details](http://mirdb.org/cgi-bin/target_detail.cgi?targetID=1440432) | 1395 | 56 | hsa-miR-30c-5p | [P2RY1](http://www.ncbi.nlm.nih.gov/entrez/query.fcgi?db=gene&cmd=Retrieve&dopt=full_report&list_uids=5028) | purinergic receptor P2Y1 |
| [Details](http://mirdb.org/cgi-bin/target_detail.cgi?targetID=1440504) | 1396 | 56 | hsa-miR-30c-5p | [PAQR9](http://www.ncbi.nlm.nih.gov/entrez/query.fcgi?db=gene&cmd=Retrieve&dopt=full_report&list_uids=344838) | progestin and adipoQ receptor family member 9 |
| [Details](http://mirdb.org/cgi-bin/target_detail.cgi?targetID=1440552) | 1397 | 56 | hsa-miR-30c-5p | [STX12](http://www.ncbi.nlm.nih.gov/entrez/query.fcgi?db=gene&cmd=Retrieve&dopt=full_report&list_uids=23673) | syntaxin 12 |
| [Details](http://mirdb.org/cgi-bin/target_detail.cgi?targetID=1440602) | 1398 | 56 | hsa-miR-30c-5p | [UPRT](http://www.ncbi.nlm.nih.gov/entrez/query.fcgi?db=gene&cmd=Retrieve&dopt=full_report&list_uids=139596) | uracil phosphoribosyltransferase homolog |
| [Details](http://mirdb.org/cgi-bin/target_detail.cgi?targetID=1440638) | 1399 | 56 | hsa-miR-30c-5p | [ZFX](http://www.ncbi.nlm.nih.gov/entrez/query.fcgi?db=gene&cmd=Retrieve&dopt=full_report&list_uids=7543) | zinc finger protein X-linked |
| [Details](http://mirdb.org/cgi-bin/target_detail.cgi?targetID=1440708) | 1400 | 56 | hsa-miR-30c-5p | [USP47](http://www.ncbi.nlm.nih.gov/entrez/query.fcgi?db=gene&cmd=Retrieve&dopt=full_report&list_uids=55031) | ubiquitin specific peptidase 47 |
| [Details](http://mirdb.org/cgi-bin/target_detail.cgi?targetID=1440922) | 1401 | 56 | hsa-miR-30c-5p | [MRI1](http://www.ncbi.nlm.nih.gov/entrez/query.fcgi?db=gene&cmd=Retrieve&dopt=full_report&list_uids=84245) | methylthioribose-1-phosphate isomerase 1 |
| [Details](http://mirdb.org/cgi-bin/target_detail.cgi?targetID=1439456) | 1402 | 55 | hsa-miR-30c-5p | [RAB41](http://www.ncbi.nlm.nih.gov/entrez/query.fcgi?db=gene&cmd=Retrieve&dopt=full_report&list_uids=347517) | RAB41, member RAS oncogene family |
| [Details](http://mirdb.org/cgi-bin/target_detail.cgi?targetID=1439468) | 1403 | 55 | hsa-miR-30c-5p | [VPS41](http://www.ncbi.nlm.nih.gov/entrez/query.fcgi?db=gene&cmd=Retrieve&dopt=full_report&list_uids=27072) | VPS41, HOPS complex subunit |
| [Details](http://mirdb.org/cgi-bin/target_detail.cgi?targetID=1439687) | 1404 | 55 | hsa-miR-30c-5p | [NOL4L](http://www.ncbi.nlm.nih.gov/entrez/query.fcgi?db=gene&cmd=Retrieve&dopt=full_report&list_uids=140688) | nucleolar protein 4 like |
| [Details](http://mirdb.org/cgi-bin/target_detail.cgi?targetID=1439717) | 1405 | 55 | hsa-miR-30c-5p | [MIB1](http://www.ncbi.nlm.nih.gov/entrez/query.fcgi?db=gene&cmd=Retrieve&dopt=full_report&list_uids=57534) | mindbomb E3 ubiquitin protein ligase 1 |
| [Details](http://mirdb.org/cgi-bin/target_detail.cgi?targetID=1439765) | 1406 | 55 | hsa-miR-30c-5p | [TUSC3](http://www.ncbi.nlm.nih.gov/entrez/query.fcgi?db=gene&cmd=Retrieve&dopt=full_report&list_uids=7991) | tumor suppressor candidate 3 |
| [Details](http://mirdb.org/cgi-bin/target_detail.cgi?targetID=1439890) | 1407 | 55 | hsa-miR-30c-5p | [NEGR1](http://www.ncbi.nlm.nih.gov/entrez/query.fcgi?db=gene&cmd=Retrieve&dopt=full_report&list_uids=257194) | neuronal growth regulator 1 |
| [Details](http://mirdb.org/cgi-bin/target_detail.cgi?targetID=1439980) | 1408 | 55 | hsa-miR-30c-5p | [SNX27](http://www.ncbi.nlm.nih.gov/entrez/query.fcgi?db=gene&cmd=Retrieve&dopt=full_report&list_uids=81609) | sorting nexin family member 27 |
| [Details](http://mirdb.org/cgi-bin/target_detail.cgi?targetID=1440099) | 1409 | 55 | hsa-miR-30c-5p | [IKBIP](http://www.ncbi.nlm.nih.gov/entrez/query.fcgi?db=gene&cmd=Retrieve&dopt=full_report&list_uids=121457) | IKBKB interacting protein |
| [Details](http://mirdb.org/cgi-bin/target_detail.cgi?targetID=1440122) | 1410 | 55 | hsa-miR-30c-5p | [IFIT1](http://www.ncbi.nlm.nih.gov/entrez/query.fcgi?db=gene&cmd=Retrieve&dopt=full_report&list_uids=3434) | interferon induced protein with tetratricopeptide repeats 1 |
| [Details](http://mirdb.org/cgi-bin/target_detail.cgi?targetID=1440313) | 1411 | 55 | hsa-miR-30c-5p | [MSANTD4](http://www.ncbi.nlm.nih.gov/entrez/query.fcgi?db=gene&cmd=Retrieve&dopt=full_report&list_uids=84437) | Myb/SANT DNA binding domain containing 4 with coiled-coils |
| [Details](http://mirdb.org/cgi-bin/target_detail.cgi?targetID=1440383) | 1412 | 55 | hsa-miR-30c-5p | [ARL10](http://www.ncbi.nlm.nih.gov/entrez/query.fcgi?db=gene&cmd=Retrieve&dopt=full_report&list_uids=285598) | ADP ribosylation factor like GTPase 10 |
| [Details](http://mirdb.org/cgi-bin/target_detail.cgi?targetID=1440476) | 1413 | 55 | hsa-miR-30c-5p | [TIMP2](http://www.ncbi.nlm.nih.gov/entrez/query.fcgi?db=gene&cmd=Retrieve&dopt=full_report&list_uids=7077) | TIMP metallopeptidase inhibitor 2 |
| [Details](http://mirdb.org/cgi-bin/target_detail.cgi?targetID=1440522) | 1414 | 55 | hsa-miR-30c-5p | [AMOTL1](http://www.ncbi.nlm.nih.gov/entrez/query.fcgi?db=gene&cmd=Retrieve&dopt=full_report&list_uids=154810) | angiomotin like 1 |
| [Details](http://mirdb.org/cgi-bin/target_detail.cgi?targetID=1440600) | 1415 | 55 | hsa-miR-30c-5p | [TXNDC5](http://www.ncbi.nlm.nih.gov/entrez/query.fcgi?db=gene&cmd=Retrieve&dopt=full_report&list_uids=81567) | thioredoxin domain containing 5 |
| [Details](http://mirdb.org/cgi-bin/target_detail.cgi?targetID=1440669) | 1416 | 55 | hsa-miR-30c-5p | [SLC28A3](http://www.ncbi.nlm.nih.gov/entrez/query.fcgi?db=gene&cmd=Retrieve&dopt=full_report&list_uids=64078) | solute carrier family 28 member 3 |
| [Details](http://mirdb.org/cgi-bin/target_detail.cgi?targetID=1440806) | 1417 | 55 | hsa-miR-30c-5p | [ABCA12](http://www.ncbi.nlm.nih.gov/entrez/query.fcgi?db=gene&cmd=Retrieve&dopt=full_report&list_uids=26154) | ATP binding cassette subfamily A member 12 |
| [Details](http://mirdb.org/cgi-bin/target_detail.cgi?targetID=1440931) | 1418 | 55 | hsa-miR-30c-5p | [NOTCH1](http://www.ncbi.nlm.nih.gov/entrez/query.fcgi?db=gene&cmd=Retrieve&dopt=full_report&list_uids=4851) | notch 1 |
| [Details](http://mirdb.org/cgi-bin/target_detail.cgi?targetID=1440989) | 1419 | 55 | hsa-miR-30c-5p | [TCP11L1](http://www.ncbi.nlm.nih.gov/entrez/query.fcgi?db=gene&cmd=Retrieve&dopt=full_report&list_uids=55346) | t-complex 11 like 1 |
| [Details](http://mirdb.org/cgi-bin/target_detail.cgi?targetID=1439536) | 1420 | 54 | hsa-miR-30c-5p | [ZDHHC21](http://www.ncbi.nlm.nih.gov/entrez/query.fcgi?db=gene&cmd=Retrieve&dopt=full_report&list_uids=340481) | zinc finger DHHC-type containing 21 |
| [Details](http://mirdb.org/cgi-bin/target_detail.cgi?targetID=1439635) | 1421 | 54 | hsa-miR-30c-5p | [BEAN1](http://www.ncbi.nlm.nih.gov/entrez/query.fcgi?db=gene&cmd=Retrieve&dopt=full_report&list_uids=146227) | brain expressed associated with NEDD4 1 |
| [Details](http://mirdb.org/cgi-bin/target_detail.cgi?targetID=1439647) | 1422 | 54 | hsa-miR-30c-5p | [MIS12](http://www.ncbi.nlm.nih.gov/entrez/query.fcgi?db=gene&cmd=Retrieve&dopt=full_report&list_uids=79003) | MIS12, kinetochore complex component |
| [Details](http://mirdb.org/cgi-bin/target_detail.cgi?targetID=1439650) | 1423 | 54 | hsa-miR-30c-5p | [MYOZ2](http://www.ncbi.nlm.nih.gov/entrez/query.fcgi?db=gene&cmd=Retrieve&dopt=full_report&list_uids=51778) | myozenin 2 |
| [Details](http://mirdb.org/cgi-bin/target_detail.cgi?targetID=1439696) | 1424 | 54 | hsa-miR-30c-5p | [HTR4](http://www.ncbi.nlm.nih.gov/entrez/query.fcgi?db=gene&cmd=Retrieve&dopt=full_report&list_uids=3360) | 5-hydroxytryptamine receptor 4 |
| [Details](http://mirdb.org/cgi-bin/target_detail.cgi?targetID=1439736) | 1425 | 54 | hsa-miR-30c-5p | [FUCA1](http://www.ncbi.nlm.nih.gov/entrez/query.fcgi?db=gene&cmd=Retrieve&dopt=full_report&list_uids=2517) | alpha-L-fucosidase 1 |
| [Details](http://mirdb.org/cgi-bin/target_detail.cgi?targetID=1439972) | 1426 | 54 | hsa-miR-30c-5p | [CHKA](http://www.ncbi.nlm.nih.gov/entrez/query.fcgi?db=gene&cmd=Retrieve&dopt=full_report&list_uids=1119) | choline kinase alpha |
| [Details](http://mirdb.org/cgi-bin/target_detail.cgi?targetID=1439996) | 1427 | 54 | hsa-miR-30c-5p | [FOXP4](http://www.ncbi.nlm.nih.gov/entrez/query.fcgi?db=gene&cmd=Retrieve&dopt=full_report&list_uids=116113) | forkhead box P4 |
| [Details](http://mirdb.org/cgi-bin/target_detail.cgi?targetID=1440005) | 1428 | 54 | hsa-miR-30c-5p | [EPDR1](http://www.ncbi.nlm.nih.gov/entrez/query.fcgi?db=gene&cmd=Retrieve&dopt=full_report&list_uids=54749) | ependymin related 1 |
| [Details](http://mirdb.org/cgi-bin/target_detail.cgi?targetID=1440039) | 1429 | 54 | hsa-miR-30c-5p | [EPB41L3](http://www.ncbi.nlm.nih.gov/entrez/query.fcgi?db=gene&cmd=Retrieve&dopt=full_report&list_uids=23136) | erythrocyte membrane protein band 4.1 like 3 |
| [Details](http://mirdb.org/cgi-bin/target_detail.cgi?targetID=1440041) | 1430 | 54 | hsa-miR-30c-5p | [RBM15](http://www.ncbi.nlm.nih.gov/entrez/query.fcgi?db=gene&cmd=Retrieve&dopt=full_report&list_uids=64783) | RNA binding motif protein 15 |
| [Details](http://mirdb.org/cgi-bin/target_detail.cgi?targetID=1440051) | 1431 | 54 | hsa-miR-30c-5p | [ZNF264](http://www.ncbi.nlm.nih.gov/entrez/query.fcgi?db=gene&cmd=Retrieve&dopt=full_report&list_uids=9422) | zinc finger protein 264 |
| [Details](http://mirdb.org/cgi-bin/target_detail.cgi?targetID=1440132) | 1432 | 54 | hsa-miR-30c-5p | [SLC35G2](http://www.ncbi.nlm.nih.gov/entrez/query.fcgi?db=gene&cmd=Retrieve&dopt=full_report&list_uids=80723) | solute carrier family 35 member G2 |
| [Details](http://mirdb.org/cgi-bin/target_detail.cgi?targetID=1440138) | 1433 | 54 | hsa-miR-30c-5p | [ARAP2](http://www.ncbi.nlm.nih.gov/entrez/query.fcgi?db=gene&cmd=Retrieve&dopt=full_report&list_uids=116984) | ArfGAP with RhoGAP domain, ankyrin repeat and PH domain 2 |
| [Details](http://mirdb.org/cgi-bin/target_detail.cgi?targetID=1440172) | 1434 | 54 | hsa-miR-30c-5p | [SRPRA](http://www.ncbi.nlm.nih.gov/entrez/query.fcgi?db=gene&cmd=Retrieve&dopt=full_report&list_uids=6734) | SRP receptor subunit alpha |
| [Details](http://mirdb.org/cgi-bin/target_detail.cgi?targetID=1440251) | 1435 | 54 | hsa-miR-30c-5p | [C21orf91](http://www.ncbi.nlm.nih.gov/entrez/query.fcgi?db=gene&cmd=Retrieve&dopt=full_report&list_uids=54149) | chromosome 21 open reading frame 91 |
| [Details](http://mirdb.org/cgi-bin/target_detail.cgi?targetID=1440437) | 1436 | 54 | hsa-miR-30c-5p | [ZNF566](http://www.ncbi.nlm.nih.gov/entrez/query.fcgi?db=gene&cmd=Retrieve&dopt=full_report&list_uids=84924) | zinc finger protein 566 |
| [Details](http://mirdb.org/cgi-bin/target_detail.cgi?targetID=1440454) | 1437 | 54 | hsa-miR-30c-5p | [ANLN](http://www.ncbi.nlm.nih.gov/entrez/query.fcgi?db=gene&cmd=Retrieve&dopt=full_report&list_uids=54443) | anillin actin binding protein |
| [Details](http://mirdb.org/cgi-bin/target_detail.cgi?targetID=1440458) | 1438 | 54 | hsa-miR-30c-5p | [RNF168](http://www.ncbi.nlm.nih.gov/entrez/query.fcgi?db=gene&cmd=Retrieve&dopt=full_report&list_uids=165918) | ring finger protein 168 |
| [Details](http://mirdb.org/cgi-bin/target_detail.cgi?targetID=1440531) | 1439 | 54 | hsa-miR-30c-5p | [ARHGEF3](http://www.ncbi.nlm.nih.gov/entrez/query.fcgi?db=gene&cmd=Retrieve&dopt=full_report&list_uids=50650) | Rho guanine nucleotide exchange factor 3 |
| [Details](http://mirdb.org/cgi-bin/target_detail.cgi?targetID=1440645) | 1440 | 54 | hsa-miR-30c-5p | [PROM1](http://www.ncbi.nlm.nih.gov/entrez/query.fcgi?db=gene&cmd=Retrieve&dopt=full_report&list_uids=8842) | prominin 1 |
| [Details](http://mirdb.org/cgi-bin/target_detail.cgi?targetID=1440684) | 1441 | 54 | hsa-miR-30c-5p | [SLC22A23](http://www.ncbi.nlm.nih.gov/entrez/query.fcgi?db=gene&cmd=Retrieve&dopt=full_report&list_uids=63027) | solute carrier family 22 member 23 |
| [Details](http://mirdb.org/cgi-bin/target_detail.cgi?targetID=1440738) | 1442 | 54 | hsa-miR-30c-5p | [AKAIN1](http://www.ncbi.nlm.nih.gov/entrez/query.fcgi?db=gene&cmd=Retrieve&dopt=full_report&list_uids=642597) | A-kinase anchor inhibitor 1 |
| [Details](http://mirdb.org/cgi-bin/target_detail.cgi?targetID=1440912) | 1443 | 54 | hsa-miR-30c-5p | [FAM104A](http://www.ncbi.nlm.nih.gov/entrez/query.fcgi?db=gene&cmd=Retrieve&dopt=full_report&list_uids=84923) | family with sequence similarity 104 member A |
| [Details](http://mirdb.org/cgi-bin/target_detail.cgi?targetID=1439548) | 1444 | 53 | hsa-miR-30c-5p | [SUCLG2](http://www.ncbi.nlm.nih.gov/entrez/query.fcgi?db=gene&cmd=Retrieve&dopt=full_report&list_uids=8801) | succinate-CoA ligase GDP-forming beta subunit |
| [Details](http://mirdb.org/cgi-bin/target_detail.cgi?targetID=1439566) | 1445 | 53 | hsa-miR-30c-5p | [RCBTB1](http://www.ncbi.nlm.nih.gov/entrez/query.fcgi?db=gene&cmd=Retrieve&dopt=full_report&list_uids=55213) | RCC1 and BTB domain containing protein 1 |
| [Details](http://mirdb.org/cgi-bin/target_detail.cgi?targetID=1439709) | 1446 | 53 | hsa-miR-30c-5p | [CHD9](http://www.ncbi.nlm.nih.gov/entrez/query.fcgi?db=gene&cmd=Retrieve&dopt=full_report&list_uids=80205) | chromodomain helicase DNA binding protein 9 |
| [Details](http://mirdb.org/cgi-bin/target_detail.cgi?targetID=1439722) | 1447 | 53 | hsa-miR-30c-5p | [TBPL1](http://www.ncbi.nlm.nih.gov/entrez/query.fcgi?db=gene&cmd=Retrieve&dopt=full_report&list_uids=9519) | TATA-box binding protein like 1 |
| [Details](http://mirdb.org/cgi-bin/target_detail.cgi?targetID=1439777) | 1448 | 53 | hsa-miR-30c-5p | [LHFPL3](http://www.ncbi.nlm.nih.gov/entrez/query.fcgi?db=gene&cmd=Retrieve&dopt=full_report&list_uids=375612) | LHFPL tetraspan subfamily member 3 |
| [Details](http://mirdb.org/cgi-bin/target_detail.cgi?targetID=1439828) | 1449 | 53 | hsa-miR-30c-5p | [ZNF354C](http://www.ncbi.nlm.nih.gov/entrez/query.fcgi?db=gene&cmd=Retrieve&dopt=full_report&list_uids=30832) | zinc finger protein 354C |
| [Details](http://mirdb.org/cgi-bin/target_detail.cgi?targetID=1439990) | 1450 | 53 | hsa-miR-30c-5p | [PTBP3](http://www.ncbi.nlm.nih.gov/entrez/query.fcgi?db=gene&cmd=Retrieve&dopt=full_report&list_uids=9991) | polypyrimidine tract binding protein 3 |
| [Details](http://mirdb.org/cgi-bin/target_detail.cgi?targetID=1440002) | 1451 | 53 | hsa-miR-30c-5p | [RAB14](http://www.ncbi.nlm.nih.gov/entrez/query.fcgi?db=gene&cmd=Retrieve&dopt=full_report&list_uids=51552) | RAB14, member RAS oncogene family |
| [Details](http://mirdb.org/cgi-bin/target_detail.cgi?targetID=1440026) | 1452 | 53 | hsa-miR-30c-5p | [CAMTA1](http://www.ncbi.nlm.nih.gov/entrez/query.fcgi?db=gene&cmd=Retrieve&dopt=full_report&list_uids=23261) | calmodulin binding transcription activator 1 |
| [Details](http://mirdb.org/cgi-bin/target_detail.cgi?targetID=1440060) | 1453 | 53 | hsa-miR-30c-5p | [PIK3R2](http://www.ncbi.nlm.nih.gov/entrez/query.fcgi?db=gene&cmd=Retrieve&dopt=full_report&list_uids=5296) | phosphoinositide-3-kinase regulatory subunit 2 |
| [Details](http://mirdb.org/cgi-bin/target_detail.cgi?targetID=1440078) | 1454 | 53 | hsa-miR-30c-5p | [SLC46A2](http://www.ncbi.nlm.nih.gov/entrez/query.fcgi?db=gene&cmd=Retrieve&dopt=full_report&list_uids=57864) | solute carrier family 46 member 2 |
| [Details](http://mirdb.org/cgi-bin/target_detail.cgi?targetID=1440161) | 1455 | 53 | hsa-miR-30c-5p | [TIA1](http://www.ncbi.nlm.nih.gov/entrez/query.fcgi?db=gene&cmd=Retrieve&dopt=full_report&list_uids=7072) | TIA1 cytotoxic granule associated RNA binding protein |
| [Details](http://mirdb.org/cgi-bin/target_detail.cgi?targetID=1440215) | 1456 | 53 | hsa-miR-30c-5p | [FBXL14](http://www.ncbi.nlm.nih.gov/entrez/query.fcgi?db=gene&cmd=Retrieve&dopt=full_report&list_uids=144699) | F-box and leucine rich repeat protein 14 |
| [Details](http://mirdb.org/cgi-bin/target_detail.cgi?targetID=1440230) | 1457 | 53 | hsa-miR-30c-5p | [RGS6](http://www.ncbi.nlm.nih.gov/entrez/query.fcgi?db=gene&cmd=Retrieve&dopt=full_report&list_uids=9628) | regulator of G protein signaling 6 |
| [Details](http://mirdb.org/cgi-bin/target_detail.cgi?targetID=1440241) | 1458 | 53 | hsa-miR-30c-5p | [ITGA4](http://www.ncbi.nlm.nih.gov/entrez/query.fcgi?db=gene&cmd=Retrieve&dopt=full_report&list_uids=3676) | integrin subunit alpha 4 |
| [Details](http://mirdb.org/cgi-bin/target_detail.cgi?targetID=1440260) | 1459 | 53 | hsa-miR-30c-5p | [QRFPR](http://www.ncbi.nlm.nih.gov/entrez/query.fcgi?db=gene&cmd=Retrieve&dopt=full_report&list_uids=84109) | pyroglutamylated RFamide peptide receptor |
| [Details](http://mirdb.org/cgi-bin/target_detail.cgi?targetID=1440314) | 1460 | 53 | hsa-miR-30c-5p | [DOCK4](http://www.ncbi.nlm.nih.gov/entrez/query.fcgi?db=gene&cmd=Retrieve&dopt=full_report&list_uids=9732) | dedicator of cytokinesis 4 |
| [Details](http://mirdb.org/cgi-bin/target_detail.cgi?targetID=1440371) | 1461 | 53 | hsa-miR-30c-5p | [PGGT1B](http://www.ncbi.nlm.nih.gov/entrez/query.fcgi?db=gene&cmd=Retrieve&dopt=full_report&list_uids=5229) | protein geranylgeranyltransferase type I subunit beta |
| [Details](http://mirdb.org/cgi-bin/target_detail.cgi?targetID=1440397) | 1462 | 53 | hsa-miR-30c-5p | [VIP](http://www.ncbi.nlm.nih.gov/entrez/query.fcgi?db=gene&cmd=Retrieve&dopt=full_report&list_uids=7432) | vasoactive intestinal peptide |
| [Details](http://mirdb.org/cgi-bin/target_detail.cgi?targetID=1440413) | 1463 | 53 | hsa-miR-30c-5p | [AR](http://www.ncbi.nlm.nih.gov/entrez/query.fcgi?db=gene&cmd=Retrieve&dopt=full_report&list_uids=367) | androgen receptor |
| [Details](http://mirdb.org/cgi-bin/target_detail.cgi?targetID=1440480) | 1464 | 53 | hsa-miR-30c-5p | [FCN1](http://www.ncbi.nlm.nih.gov/entrez/query.fcgi?db=gene&cmd=Retrieve&dopt=full_report&list_uids=2219) | ficolin 1 |
| [Details](http://mirdb.org/cgi-bin/target_detail.cgi?targetID=1440514) | 1465 | 53 | hsa-miR-30c-5p | [AMER1](http://www.ncbi.nlm.nih.gov/entrez/query.fcgi?db=gene&cmd=Retrieve&dopt=full_report&list_uids=139285) | APC membrane recruitment protein 1 |
| [Details](http://mirdb.org/cgi-bin/target_detail.cgi?targetID=1440590) | 1466 | 53 | hsa-miR-30c-5p | [TANK](http://www.ncbi.nlm.nih.gov/entrez/query.fcgi?db=gene&cmd=Retrieve&dopt=full_report&list_uids=10010) | TRAF family member associated NFKB activator |
| [Details](http://mirdb.org/cgi-bin/target_detail.cgi?targetID=1440646) | 1467 | 53 | hsa-miR-30c-5p | [CRISPLD1](http://www.ncbi.nlm.nih.gov/entrez/query.fcgi?db=gene&cmd=Retrieve&dopt=full_report&list_uids=83690) | cysteine rich secretory protein LCCL domain containing 1 |
| [Details](http://mirdb.org/cgi-bin/target_detail.cgi?targetID=1440665) | 1468 | 53 | hsa-miR-30c-5p | [IL25](http://www.ncbi.nlm.nih.gov/entrez/query.fcgi?db=gene&cmd=Retrieve&dopt=full_report&list_uids=64806) | interleukin 25 |
| [Details](http://mirdb.org/cgi-bin/target_detail.cgi?targetID=1440751) | 1469 | 53 | hsa-miR-30c-5p | [MEX3D](http://www.ncbi.nlm.nih.gov/entrez/query.fcgi?db=gene&cmd=Retrieve&dopt=full_report&list_uids=399664) | mex-3 RNA binding family member D |
| [Details](http://mirdb.org/cgi-bin/target_detail.cgi?targetID=1440762) | 1470 | 53 | hsa-miR-30c-5p | [TBL1X](http://www.ncbi.nlm.nih.gov/entrez/query.fcgi?db=gene&cmd=Retrieve&dopt=full_report&list_uids=6907) | transducin beta like 1 X-linked |
| [Details](http://mirdb.org/cgi-bin/target_detail.cgi?targetID=1440797) | 1471 | 53 | hsa-miR-30c-5p | [RTCB](http://www.ncbi.nlm.nih.gov/entrez/query.fcgi?db=gene&cmd=Retrieve&dopt=full_report&list_uids=51493) | RNA 2',3'-cyclic phosphate and 5'-OH ligase |
| [Details](http://mirdb.org/cgi-bin/target_detail.cgi?targetID=1440805) | 1472 | 53 | hsa-miR-30c-5p | [RAB21](http://www.ncbi.nlm.nih.gov/entrez/query.fcgi?db=gene&cmd=Retrieve&dopt=full_report&list_uids=23011) | RAB21, member RAS oncogene family |
| [Details](http://mirdb.org/cgi-bin/target_detail.cgi?targetID=1440873) | 1473 | 53 | hsa-miR-30c-5p | [INTS2](http://www.ncbi.nlm.nih.gov/entrez/query.fcgi?db=gene&cmd=Retrieve&dopt=full_report&list_uids=57508) | integrator complex subunit 2 |
| [Details](http://mirdb.org/cgi-bin/target_detail.cgi?targetID=1440925) | 1474 | 53 | hsa-miR-30c-5p | [RAB11A](http://www.ncbi.nlm.nih.gov/entrez/query.fcgi?db=gene&cmd=Retrieve&dopt=full_report&list_uids=8766) | RAB11A, member RAS oncogene family |
| [Details](http://mirdb.org/cgi-bin/target_detail.cgi?targetID=1440995) | 1475 | 53 | hsa-miR-30c-5p | [C7orf31](http://www.ncbi.nlm.nih.gov/entrez/query.fcgi?db=gene&cmd=Retrieve&dopt=full_report&list_uids=136895) | chromosome 7 open reading frame 31 |
| [Details](http://mirdb.org/cgi-bin/target_detail.cgi?targetID=1439563) | 1476 | 52 | hsa-miR-30c-5p | [CACNA1D](http://www.ncbi.nlm.nih.gov/entrez/query.fcgi?db=gene&cmd=Retrieve&dopt=full_report&list_uids=776) | calcium voltage-gated channel subunit alpha1 D |
| [Details](http://mirdb.org/cgi-bin/target_detail.cgi?targetID=1439598) | 1477 | 52 | hsa-miR-30c-5p | [SMAP2](http://www.ncbi.nlm.nih.gov/entrez/query.fcgi?db=gene&cmd=Retrieve&dopt=full_report&list_uids=64744) | small ArfGAP2 |
| [Details](http://mirdb.org/cgi-bin/target_detail.cgi?targetID=1439624) | 1478 | 52 | hsa-miR-30c-5p | [NBPF1](http://www.ncbi.nlm.nih.gov/entrez/query.fcgi?db=gene&cmd=Retrieve&dopt=full_report&list_uids=55672) | NBPF member 1 |
| [Details](http://mirdb.org/cgi-bin/target_detail.cgi?targetID=1439690) | 1479 | 52 | hsa-miR-30c-5p | [NEUROD6](http://www.ncbi.nlm.nih.gov/entrez/query.fcgi?db=gene&cmd=Retrieve&dopt=full_report&list_uids=63974) | neuronal differentiation 6 |
| [Details](http://mirdb.org/cgi-bin/target_detail.cgi?targetID=1439749) | 1480 | 52 | hsa-miR-30c-5p | [ZNF491](http://www.ncbi.nlm.nih.gov/entrez/query.fcgi?db=gene&cmd=Retrieve&dopt=full_report&list_uids=126069) | zinc finger protein 491 |
| [Details](http://mirdb.org/cgi-bin/target_detail.cgi?targetID=1439754) | 1481 | 52 | hsa-miR-30c-5p | [CDK17](http://www.ncbi.nlm.nih.gov/entrez/query.fcgi?db=gene&cmd=Retrieve&dopt=full_report&list_uids=5128) | cyclin dependent kinase 17 |
| [Details](http://mirdb.org/cgi-bin/target_detail.cgi?targetID=1439776) | 1482 | 52 | hsa-miR-30c-5p | [SLC16A14](http://www.ncbi.nlm.nih.gov/entrez/query.fcgi?db=gene&cmd=Retrieve&dopt=full_report&list_uids=151473) | solute carrier family 16 member 14 |
| [Details](http://mirdb.org/cgi-bin/target_detail.cgi?targetID=1439783) | 1483 | 52 | hsa-miR-30c-5p | [USP44](http://www.ncbi.nlm.nih.gov/entrez/query.fcgi?db=gene&cmd=Retrieve&dopt=full_report&list_uids=84101) | ubiquitin specific peptidase 44 |
| [Details](http://mirdb.org/cgi-bin/target_detail.cgi?targetID=1439856) | 1484 | 52 | hsa-miR-30c-5p | [GORAB](http://www.ncbi.nlm.nih.gov/entrez/query.fcgi?db=gene&cmd=Retrieve&dopt=full_report&list_uids=92344) | golgin, RAB6 interacting |
| [Details](http://mirdb.org/cgi-bin/target_detail.cgi?targetID=1439940) | 1485 | 52 | hsa-miR-30c-5p | [CAT](http://www.ncbi.nlm.nih.gov/entrez/query.fcgi?db=gene&cmd=Retrieve&dopt=full_report&list_uids=847) | catalase |
| [Details](http://mirdb.org/cgi-bin/target_detail.cgi?targetID=1439997) | 1486 | 52 | hsa-miR-30c-5p | [COPS2](http://www.ncbi.nlm.nih.gov/entrez/query.fcgi?db=gene&cmd=Retrieve&dopt=full_report&list_uids=9318) | COP9 signalosome subunit 2 |
| [Details](http://mirdb.org/cgi-bin/target_detail.cgi?targetID=1440194) | 1487 | 52 | hsa-miR-30c-5p | [GOLGA6L4](http://www.ncbi.nlm.nih.gov/entrez/query.fcgi?db=gene&cmd=Retrieve&dopt=full_report&list_uids=643707) | golgin A6 family-like 4 |
| [Details](http://mirdb.org/cgi-bin/target_detail.cgi?targetID=1440196) | 1488 | 52 | hsa-miR-30c-5p | [ABHD6](http://www.ncbi.nlm.nih.gov/entrez/query.fcgi?db=gene&cmd=Retrieve&dopt=full_report&list_uids=57406) | abhydrolase domain containing 6 |
| [Details](http://mirdb.org/cgi-bin/target_detail.cgi?targetID=1440257) | 1489 | 52 | hsa-miR-30c-5p | [ERC2](http://www.ncbi.nlm.nih.gov/entrez/query.fcgi?db=gene&cmd=Retrieve&dopt=full_report&list_uids=26059) | ELKS/RAB6-interacting/CAST family member 2 |
| [Details](http://mirdb.org/cgi-bin/target_detail.cgi?targetID=1440336) | 1490 | 52 | hsa-miR-30c-5p | [ANO5](http://www.ncbi.nlm.nih.gov/entrez/query.fcgi?db=gene&cmd=Retrieve&dopt=full_report&list_uids=203859) | anoctamin 5 |
| [Details](http://mirdb.org/cgi-bin/target_detail.cgi?targetID=1440364) | 1491 | 52 | hsa-miR-30c-5p | [ZNF805](http://www.ncbi.nlm.nih.gov/entrez/query.fcgi?db=gene&cmd=Retrieve&dopt=full_report&list_uids=390980) | zinc finger protein 805 |
| [Details](http://mirdb.org/cgi-bin/target_detail.cgi?targetID=1440369) | 1492 | 52 | hsa-miR-30c-5p | [EPB41L4B](http://www.ncbi.nlm.nih.gov/entrez/query.fcgi?db=gene&cmd=Retrieve&dopt=full_report&list_uids=54566) | erythrocyte membrane protein band 4.1 like 4B |
| [Details](http://mirdb.org/cgi-bin/target_detail.cgi?targetID=1440404) | 1493 | 52 | hsa-miR-30c-5p | [ST8SIA1](http://www.ncbi.nlm.nih.gov/entrez/query.fcgi?db=gene&cmd=Retrieve&dopt=full_report&list_uids=6489) | ST8 alpha-N-acetyl-neuraminide alpha-2,8-sialyltransferase 1 |
| [Details](http://mirdb.org/cgi-bin/target_detail.cgi?targetID=1440442) | 1494 | 52 | hsa-miR-30c-5p | [RGL1](http://www.ncbi.nlm.nih.gov/entrez/query.fcgi?db=gene&cmd=Retrieve&dopt=full_report&list_uids=23179) | ral guanine nucleotide dissociation stimulator like 1 |
| [Details](http://mirdb.org/cgi-bin/target_detail.cgi?targetID=1440446) | 1495 | 52 | hsa-miR-30c-5p | [GOLGA6L10](http://www.ncbi.nlm.nih.gov/entrez/query.fcgi?db=gene&cmd=Retrieve&dopt=full_report&list_uids=647042) | golgin A6 family-like 10 |
| [Details](http://mirdb.org/cgi-bin/target_detail.cgi?targetID=1440495) | 1496 | 52 | hsa-miR-30c-5p | [EYS](http://www.ncbi.nlm.nih.gov/entrez/query.fcgi?db=gene&cmd=Retrieve&dopt=full_report&list_uids=346007) | eyes shut homolog |
| [Details](http://mirdb.org/cgi-bin/target_detail.cgi?targetID=1440784) | 1497 | 52 | hsa-miR-30c-5p | [DOC2A](http://www.ncbi.nlm.nih.gov/entrez/query.fcgi?db=gene&cmd=Retrieve&dopt=full_report&list_uids=8448) | double C2 domain alpha |
| [Details](http://mirdb.org/cgi-bin/target_detail.cgi?targetID=1440786) | 1498 | 52 | hsa-miR-30c-5p | [ARFIP1](http://www.ncbi.nlm.nih.gov/entrez/query.fcgi?db=gene&cmd=Retrieve&dopt=full_report&list_uids=27236) | ADP ribosylation factor interacting protein 1 |
| [Details](http://mirdb.org/cgi-bin/target_detail.cgi?targetID=1440896) | 1499 | 52 | hsa-miR-30c-5p | [FRMD4A](http://www.ncbi.nlm.nih.gov/entrez/query.fcgi?db=gene&cmd=Retrieve&dopt=full_report&list_uids=55691) | FERM domain containing 4A |
| [Details](http://mirdb.org/cgi-bin/target_detail.cgi?targetID=1439685) | 1500 | 51 | hsa-miR-30c-5p | [TICAM1](http://www.ncbi.nlm.nih.gov/entrez/query.fcgi?db=gene&cmd=Retrieve&dopt=full_report&list_uids=148022) | toll like receptor adaptor molecule 1 |
| [Details](http://mirdb.org/cgi-bin/target_detail.cgi?targetID=1439790) | 1501 | 51 | hsa-miR-30c-5p | [CEP85L](http://www.ncbi.nlm.nih.gov/entrez/query.fcgi?db=gene&cmd=Retrieve&dopt=full_report&list_uids=387119) | centrosomal protein 85 like |
| [Details](http://mirdb.org/cgi-bin/target_detail.cgi?targetID=1439832) | 1502 | 51 | hsa-miR-30c-5p | [MPP7](http://www.ncbi.nlm.nih.gov/entrez/query.fcgi?db=gene&cmd=Retrieve&dopt=full_report&list_uids=143098) | membrane palmitoylated protein 7 |
| [Details](http://mirdb.org/cgi-bin/target_detail.cgi?targetID=1439858) | 1503 | 51 | hsa-miR-30c-5p | [EBF2](http://www.ncbi.nlm.nih.gov/entrez/query.fcgi?db=gene&cmd=Retrieve&dopt=full_report&list_uids=64641) | EBF transcription factor 2 |
| [Details](http://mirdb.org/cgi-bin/target_detail.cgi?targetID=1439897) | 1504 | 51 | hsa-miR-30c-5p | [NIF3L1](http://www.ncbi.nlm.nih.gov/entrez/query.fcgi?db=gene&cmd=Retrieve&dopt=full_report&list_uids=60491) | NGG1 interacting factor 3 like 1 |
| [Details](http://mirdb.org/cgi-bin/target_detail.cgi?targetID=1439968) | 1505 | 51 | hsa-miR-30c-5p | [PITPNB](http://www.ncbi.nlm.nih.gov/entrez/query.fcgi?db=gene&cmd=Retrieve&dopt=full_report&list_uids=23760) | phosphatidylinositol transfer protein beta |
| [Details](http://mirdb.org/cgi-bin/target_detail.cgi?targetID=1440035) | 1506 | 51 | hsa-miR-30c-5p | [PDK4](http://www.ncbi.nlm.nih.gov/entrez/query.fcgi?db=gene&cmd=Retrieve&dopt=full_report&list_uids=5166) | pyruvate dehydrogenase kinase 4 |
| [Details](http://mirdb.org/cgi-bin/target_detail.cgi?targetID=1440038) | 1507 | 51 | hsa-miR-30c-5p | [TNFSF9](http://www.ncbi.nlm.nih.gov/entrez/query.fcgi?db=gene&cmd=Retrieve&dopt=full_report&list_uids=8744) | TNF superfamily member 9 |
| [Details](http://mirdb.org/cgi-bin/target_detail.cgi?targetID=1440059) | 1508 | 51 | hsa-miR-30c-5p | [ZFP1](http://www.ncbi.nlm.nih.gov/entrez/query.fcgi?db=gene&cmd=Retrieve&dopt=full_report&list_uids=162239) | ZFP1 zinc finger protein |
| [Details](http://mirdb.org/cgi-bin/target_detail.cgi?targetID=1440081) | 1509 | 51 | hsa-miR-30c-5p | [CTH](http://www.ncbi.nlm.nih.gov/entrez/query.fcgi?db=gene&cmd=Retrieve&dopt=full_report&list_uids=1491) | cystathionine gamma-lyase |
| [Details](http://mirdb.org/cgi-bin/target_detail.cgi?targetID=1440103) | 1510 | 51 | hsa-miR-30c-5p | [NBPF3](http://www.ncbi.nlm.nih.gov/entrez/query.fcgi?db=gene&cmd=Retrieve&dopt=full_report&list_uids=84224) | NBPF member 3 |
| [Details](http://mirdb.org/cgi-bin/target_detail.cgi?targetID=1440136) | 1511 | 51 | hsa-miR-30c-5p | [NTM](http://www.ncbi.nlm.nih.gov/entrez/query.fcgi?db=gene&cmd=Retrieve&dopt=full_report&list_uids=50863) | neurotrimin |
| [Details](http://mirdb.org/cgi-bin/target_detail.cgi?targetID=1440275) | 1512 | 51 | hsa-miR-30c-5p | [TMEM263](http://www.ncbi.nlm.nih.gov/entrez/query.fcgi?db=gene&cmd=Retrieve&dopt=full_report&list_uids=90488) | transmembrane protein 263 |
| [Details](http://mirdb.org/cgi-bin/target_detail.cgi?targetID=1440347) | 1513 | 51 | hsa-miR-30c-5p | [ZNF286B](http://www.ncbi.nlm.nih.gov/entrez/query.fcgi?db=gene&cmd=Retrieve&dopt=full_report&list_uids=729288) | zinc finger protein 286B |
| [Details](http://mirdb.org/cgi-bin/target_detail.cgi?targetID=1440353) | 1514 | 51 | hsa-miR-30c-5p | [SV2B](http://www.ncbi.nlm.nih.gov/entrez/query.fcgi?db=gene&cmd=Retrieve&dopt=full_report&list_uids=9899) | synaptic vesicle glycoprotein 2B |
| [Details](http://mirdb.org/cgi-bin/target_detail.cgi?targetID=1440358) | 1515 | 51 | hsa-miR-30c-5p | [SPA17](http://www.ncbi.nlm.nih.gov/entrez/query.fcgi?db=gene&cmd=Retrieve&dopt=full_report&list_uids=53340) | sperm autoantigenic protein 17 |
| [Details](http://mirdb.org/cgi-bin/target_detail.cgi?targetID=1440395) | 1516 | 51 | hsa-miR-30c-5p | [DNAJC3](http://www.ncbi.nlm.nih.gov/entrez/query.fcgi?db=gene&cmd=Retrieve&dopt=full_report&list_uids=5611) | DnaJ heat shock protein family (Hsp40) member C3 |
| [Details](http://mirdb.org/cgi-bin/target_detail.cgi?targetID=1440489) | 1517 | 51 | hsa-miR-30c-5p | [PIK3CB](http://www.ncbi.nlm.nih.gov/entrez/query.fcgi?db=gene&cmd=Retrieve&dopt=full_report&list_uids=5291) | phosphatidylinositol-4,5-bisphosphate 3-kinase catalytic subunit beta |
| [Details](http://mirdb.org/cgi-bin/target_detail.cgi?targetID=1440491) | 1518 | 51 | hsa-miR-30c-5p | [ZRANB3](http://www.ncbi.nlm.nih.gov/entrez/query.fcgi?db=gene&cmd=Retrieve&dopt=full_report&list_uids=84083) | zinc finger RANBP2-type containing 3 |
| [Details](http://mirdb.org/cgi-bin/target_detail.cgi?targetID=1440501) | 1519 | 51 | hsa-miR-30c-5p | [SFXN1](http://www.ncbi.nlm.nih.gov/entrez/query.fcgi?db=gene&cmd=Retrieve&dopt=full_report&list_uids=94081) | sideroflexin 1 |
| [Details](http://mirdb.org/cgi-bin/target_detail.cgi?targetID=1440630) | 1520 | 51 | hsa-miR-30c-5p | [CATSPERG](http://www.ncbi.nlm.nih.gov/entrez/query.fcgi?db=gene&cmd=Retrieve&dopt=full_report&list_uids=57828) | cation channel sperm associated auxiliary subunit gamma |
| [Details](http://mirdb.org/cgi-bin/target_detail.cgi?targetID=1440720) | 1521 | 51 | hsa-miR-30c-5p | [AP1AR](http://www.ncbi.nlm.nih.gov/entrez/query.fcgi?db=gene&cmd=Retrieve&dopt=full_report&list_uids=55435) | adaptor related protein complex 1 associated regulatory protein |
| [Details](http://mirdb.org/cgi-bin/target_detail.cgi?targetID=1440955) | 1522 | 51 | hsa-miR-30c-5p | [ELOVL7](http://www.ncbi.nlm.nih.gov/entrez/query.fcgi?db=gene&cmd=Retrieve&dopt=full_report&list_uids=79993) | ELOVL fatty acid elongase 7 |
| [Details](http://mirdb.org/cgi-bin/target_detail.cgi?targetID=1439478) | 1523 | 50 | hsa-miR-30c-5p | [MAGI3](http://www.ncbi.nlm.nih.gov/entrez/query.fcgi?db=gene&cmd=Retrieve&dopt=full_report&list_uids=260425) | membrane associated guanylate kinase, WW and PDZ domain containing 3 |
| [Details](http://mirdb.org/cgi-bin/target_detail.cgi?targetID=1439762) | 1524 | 50 | hsa-miR-30c-5p | [TUBGCP3](http://www.ncbi.nlm.nih.gov/entrez/query.fcgi?db=gene&cmd=Retrieve&dopt=full_report&list_uids=10426) | tubulin gamma complex associated protein 3 |
| [Details](http://mirdb.org/cgi-bin/target_detail.cgi?targetID=1439763) | 1525 | 50 | hsa-miR-30c-5p | [KIAA1841](http://www.ncbi.nlm.nih.gov/entrez/query.fcgi?db=gene&cmd=Retrieve&dopt=full_report&list_uids=84542) | KIAA1841 |
| [Details](http://mirdb.org/cgi-bin/target_detail.cgi?targetID=1439906) | 1526 | 50 | hsa-miR-30c-5p | [ERI2](http://www.ncbi.nlm.nih.gov/entrez/query.fcgi?db=gene&cmd=Retrieve&dopt=full_report&list_uids=112479) | ERI1 exoribonuclease family member 2 |
| [Details](http://mirdb.org/cgi-bin/target_detail.cgi?targetID=1439970) | 1527 | 50 | hsa-miR-30c-5p | [CCL16](http://www.ncbi.nlm.nih.gov/entrez/query.fcgi?db=gene&cmd=Retrieve&dopt=full_report&list_uids=6360) | C-C motif chemokine ligand 16 |
| [Details](http://mirdb.org/cgi-bin/target_detail.cgi?targetID=1440016) | 1528 | 50 | hsa-miR-30c-5p | [CRK](http://www.ncbi.nlm.nih.gov/entrez/query.fcgi?db=gene&cmd=Retrieve&dopt=full_report&list_uids=1398) | CRK proto-oncogene, adaptor protein |
| [Details](http://mirdb.org/cgi-bin/target_detail.cgi?targetID=1440106) | 1529 | 50 | hsa-miR-30c-5p | [AGO4](http://www.ncbi.nlm.nih.gov/entrez/query.fcgi?db=gene&cmd=Retrieve&dopt=full_report&list_uids=192670) | argonaute RISC catalytic component 4 |
| [Details](http://mirdb.org/cgi-bin/target_detail.cgi?targetID=1440135) | 1530 | 50 | hsa-miR-30c-5p | [CYYR1](http://www.ncbi.nlm.nih.gov/entrez/query.fcgi?db=gene&cmd=Retrieve&dopt=full_report&list_uids=116159) | cysteine and tyrosine rich 1 |
| [Details](http://mirdb.org/cgi-bin/target_detail.cgi?targetID=1440326) | 1531 | 50 | hsa-miR-30c-5p | [MECP2](http://www.ncbi.nlm.nih.gov/entrez/query.fcgi?db=gene&cmd=Retrieve&dopt=full_report&list_uids=4204) | methyl-CpG binding protein 2 |
| [Details](http://mirdb.org/cgi-bin/target_detail.cgi?targetID=1440361) | 1532 | 50 | hsa-miR-30c-5p | [MGAT2](http://www.ncbi.nlm.nih.gov/entrez/query.fcgi?db=gene&cmd=Retrieve&dopt=full_report&list_uids=4247) | mannosyl (alpha-1,6-)-glycoprotein beta-1,2-N-acetylglucosaminyltransferase |
| [Details](http://mirdb.org/cgi-bin/target_detail.cgi?targetID=1440393) | 1533 | 50 | hsa-miR-30c-5p | [SMARCD2](http://www.ncbi.nlm.nih.gov/entrez/query.fcgi?db=gene&cmd=Retrieve&dopt=full_report&list_uids=6603) | SWI/SNF related, matrix associated, actin dependent regulator of chromatin, subfamily d, member 2 |
| [Details](http://mirdb.org/cgi-bin/target_detail.cgi?targetID=1440423) | 1534 | 50 | hsa-miR-30c-5p | [ENPP4](http://www.ncbi.nlm.nih.gov/entrez/query.fcgi?db=gene&cmd=Retrieve&dopt=full_report&list_uids=22875) | ectonucleotide pyrophosphatase/phosphodiesterase 4 |
| [Details](http://mirdb.org/cgi-bin/target_detail.cgi?targetID=1440438) | 1535 | 50 | hsa-miR-30c-5p | [MAP3K1](http://www.ncbi.nlm.nih.gov/entrez/query.fcgi?db=gene&cmd=Retrieve&dopt=full_report&list_uids=4214) | mitogen-activated protein kinase kinase kinase 1 |
| [Details](http://mirdb.org/cgi-bin/target_detail.cgi?targetID=1440472) | 1536 | 50 | hsa-miR-30c-5p | [C15orf40](http://www.ncbi.nlm.nih.gov/entrez/query.fcgi?db=gene&cmd=Retrieve&dopt=full_report&list_uids=123207) | chromosome 15 open reading frame 40 |
| [Details](http://mirdb.org/cgi-bin/target_detail.cgi?targetID=1440475) | 1537 | 50 | hsa-miR-30c-5p | [POU4F2](http://www.ncbi.nlm.nih.gov/entrez/query.fcgi?db=gene&cmd=Retrieve&dopt=full_report&list_uids=5458) | POU class 4 homeobox 2 |
| [Details](http://mirdb.org/cgi-bin/target_detail.cgi?targetID=1440574) | 1538 | 50 | hsa-miR-30c-5p | [ST6GAL2](http://www.ncbi.nlm.nih.gov/entrez/query.fcgi?db=gene&cmd=Retrieve&dopt=full_report&list_uids=84620) | ST6 beta-galactoside alpha-2,6-sialyltransferase 2 |
| [Details](http://mirdb.org/cgi-bin/target_detail.cgi?targetID=1440599) | 1539 | 50 | hsa-miR-30c-5p | [ZNF275](http://www.ncbi.nlm.nih.gov/entrez/query.fcgi?db=gene&cmd=Retrieve&dopt=full_report&list_uids=10838) | zinc finger protein 275 |
| [Details](http://mirdb.org/cgi-bin/target_detail.cgi?targetID=1440710) | 1540 | 50 | hsa-miR-30c-5p | [GRK5](http://www.ncbi.nlm.nih.gov/entrez/query.fcgi?db=gene&cmd=Retrieve&dopt=full_report&list_uids=2869) | G protein-coupled receptor kinase 5 |
| [Details](http://mirdb.org/cgi-bin/target_detail.cgi?targetID=1440849) | 1541 | 50 | hsa-miR-30c-5p | [B4GALT4](http://www.ncbi.nlm.nih.gov/entrez/query.fcgi?db=gene&cmd=Retrieve&dopt=full_report&list_uids=8702) | beta-1,4-galactosyltransferase 4 |
| [Details](http://mirdb.org/cgi-bin/target_detail.cgi?targetID=1440897) | 1542 | 50 | hsa-miR-30c-5p | [FAM240A](http://www.ncbi.nlm.nih.gov/entrez/query.fcgi?db=gene&cmd=Retrieve&dopt=full_report&list_uids=100132146) | family with sequence similarity 240 member A |
| [Details](http://mirdb.org/cgi-bin/target_detail.cgi?targetID=1440929) | 1543 | 50 | hsa-miR-30c-5p | [SEPT3](http://www.ncbi.nlm.nih.gov/entrez/query.fcgi?db=gene&cmd=Retrieve&dopt=full_report&list_uids=55964) | septin 3 |
| [Details](http://mirdb.org/cgi-bin/target_detail.cgi?targetID=1440948) | 1544 | 50 | hsa-miR-30c-5p | [SMIM15](http://www.ncbi.nlm.nih.gov/entrez/query.fcgi?db=gene&cmd=Retrieve&dopt=full_report&list_uids=643155) | small integral membrane protein 15 |
| [Details](http://mirdb.org/cgi-bin/target_detail.cgi?targetID=1440972) | 1545 | 50 | hsa-miR-30c-5p | [EXOC4](http://www.ncbi.nlm.nih.gov/entrez/query.fcgi?db=gene&cmd=Retrieve&dopt=full_report&list_uids=60412) | exocyst complex component 4 |

Data generated from the online database for prediction of functional microRNA targets (Chen & Wang, 2020)

Chen, Y., & Wang, X. (2020). MiRDB: An online database for prediction of functional microRNA targets. *Nucleic Acids Research*, *48*(D1), D127–D131. https://doi.org/10.1093/nar/gkz757
