# Supplementary material for: The Role of microRNAs in Organismal and Skin Aging
Source: Int J Mol Sci. 2020 Jul 25;21(15):5281. doi: 10.3390/ijms21155281 (PMC7432402; doi:10.3390/ijms21155281)
Supplement: Supplementary file 1 [file ijms-21-05281-s001.zip › Appendix 3 hsa-let-7d-5p .docx]

**There are 923 predicted targets for hsa-let-7d-5p in miRDB**

| **Target Detail** | **Target Rank** | **Target Score** | **miRNA Name** | **Gene Symbol** | **Gene Description** |
| --- | --- | --- | --- | --- | --- |
| [Details](http://mirdb.org/cgi-bin/target_detail.cgi?targetID=1232152) | 1 | 100 | hsa-let-7d-5p | [LIN28B](http://www.ncbi.nlm.nih.gov/entrez/query.fcgi?db=gene&cmd=Retrieve&dopt=full_report&list_uids=389421) | lin-28 homolog B |
| [Details](http://mirdb.org/cgi-bin/target_detail.cgi?targetID=1231484) | 2 | 100 | hsa-let-7d-5p | [HMGA2](http://www.ncbi.nlm.nih.gov/entrez/query.fcgi?db=gene&cmd=Retrieve&dopt=full_report&list_uids=8091) | high mobility group AT-hook 2 |
| [Details](http://mirdb.org/cgi-bin/target_detail.cgi?targetID=1232338) | 3 | 100 | hsa-let-7d-5p | [TRIM71](http://www.ncbi.nlm.nih.gov/entrez/query.fcgi?db=gene&cmd=Retrieve&dopt=full_report&list_uids=131405) | tripartite motif containing 71 |
| [Details](http://mirdb.org/cgi-bin/target_detail.cgi?targetID=1232106) | 4 | 100 | hsa-let-7d-5p | [PRTG](http://www.ncbi.nlm.nih.gov/entrez/query.fcgi?db=gene&cmd=Retrieve&dopt=full_report&list_uids=283659) | protogenin |
| [Details](http://mirdb.org/cgi-bin/target_detail.cgi?targetID=1231474) | 5 | 100 | hsa-let-7d-5p | [STARD13](http://www.ncbi.nlm.nih.gov/entrez/query.fcgi?db=gene&cmd=Retrieve&dopt=full_report&list_uids=90627) | StAR related lipid transfer domain containing 13 |
| [Details](http://mirdb.org/cgi-bin/target_detail.cgi?targetID=1231662) | 6 | 100 | hsa-let-7d-5p | [IGF2BP1](http://www.ncbi.nlm.nih.gov/entrez/query.fcgi?db=gene&cmd=Retrieve&dopt=full_report&list_uids=10642) | insulin like growth factor 2 mRNA binding protein 1 |
| [Details](http://mirdb.org/cgi-bin/target_detail.cgi?targetID=1231503) | 7 | 99 | hsa-let-7d-5p | [IGDCC3](http://www.ncbi.nlm.nih.gov/entrez/query.fcgi?db=gene&cmd=Retrieve&dopt=full_report&list_uids=9543) | immunoglobulin superfamily DCC subclass member 3 |
| [Details](http://mirdb.org/cgi-bin/target_detail.cgi?targetID=1231965) | 8 | 99 | hsa-let-7d-5p | [NPHP3](http://www.ncbi.nlm.nih.gov/entrez/query.fcgi?db=gene&cmd=Retrieve&dopt=full_report&list_uids=27031) | nephrocystin 3 |
| [Details](http://mirdb.org/cgi-bin/target_detail.cgi?targetID=1232011) | 9 | 99 | hsa-let-7d-5p | [FIGNL2](http://www.ncbi.nlm.nih.gov/entrez/query.fcgi?db=gene&cmd=Retrieve&dopt=full_report&list_uids=401720) | fidgetin like 2 |
| [Details](http://mirdb.org/cgi-bin/target_detail.cgi?targetID=1231521) | 10 | 99 | hsa-let-7d-5p | [SMARCAD1](http://www.ncbi.nlm.nih.gov/entrez/query.fcgi?db=gene&cmd=Retrieve&dopt=full_report&list_uids=56916) | SWI/SNF-related, matrix-associated actin-dependent regulator of chromatin, subfamily a, containing DEAD/H box 1 |
| [Details](http://mirdb.org/cgi-bin/target_detail.cgi?targetID=1232357) | 11 | 99 | hsa-let-7d-5p | [FRMD4B](http://www.ncbi.nlm.nih.gov/entrez/query.fcgi?db=gene&cmd=Retrieve&dopt=full_report&list_uids=23150) | FERM domain containing 4B |
| [Details](http://mirdb.org/cgi-bin/target_detail.cgi?targetID=1231477) | 12 | 99 | hsa-let-7d-5p | [FIGN](http://www.ncbi.nlm.nih.gov/entrez/query.fcgi?db=gene&cmd=Retrieve&dopt=full_report&list_uids=55137) | fidgetin, microtubule severing factor |
| [Details](http://mirdb.org/cgi-bin/target_detail.cgi?targetID=1232335) | 13 | 99 | hsa-let-7d-5p | [C14orf28](http://www.ncbi.nlm.nih.gov/entrez/query.fcgi?db=gene&cmd=Retrieve&dopt=full_report&list_uids=122525) | chromosome 14 open reading frame 28 |
| [Details](http://mirdb.org/cgi-bin/target_detail.cgi?targetID=1232142) | 14 | 99 | hsa-let-7d-5p | [NR6A1](http://www.ncbi.nlm.nih.gov/entrez/query.fcgi?db=gene&cmd=Retrieve&dopt=full_report&list_uids=2649) | nuclear receptor subfamily 6 group A member 1 |
| [Details](http://mirdb.org/cgi-bin/target_detail.cgi?targetID=1232315) | 15 | 99 | hsa-let-7d-5p | [ARID3B](http://www.ncbi.nlm.nih.gov/entrez/query.fcgi?db=gene&cmd=Retrieve&dopt=full_report&list_uids=10620) | AT-rich interaction domain 3B |
| [Details](http://mirdb.org/cgi-bin/target_detail.cgi?targetID=1232250) | 16 | 98 | hsa-let-7d-5p | [CCND2](http://www.ncbi.nlm.nih.gov/entrez/query.fcgi?db=gene&cmd=Retrieve&dopt=full_report&list_uids=894) | cyclin D2 |
| [Details](http://mirdb.org/cgi-bin/target_detail.cgi?targetID=1231936) | 17 | 98 | hsa-let-7d-5p | [NAP1L1](http://www.ncbi.nlm.nih.gov/entrez/query.fcgi?db=gene&cmd=Retrieve&dopt=full_report&list_uids=4673) | nucleosome assembly protein 1 like 1 |
| [Details](http://mirdb.org/cgi-bin/target_detail.cgi?targetID=1232256) | 18 | 98 | hsa-let-7d-5p | [GATM](http://www.ncbi.nlm.nih.gov/entrez/query.fcgi?db=gene&cmd=Retrieve&dopt=full_report&list_uids=2628) | glycine amidinotransferase |
| [Details](http://mirdb.org/cgi-bin/target_detail.cgi?targetID=1231551) | 19 | 98 | hsa-let-7d-5p | [PTAFR](http://www.ncbi.nlm.nih.gov/entrez/query.fcgi?db=gene&cmd=Retrieve&dopt=full_report&list_uids=5724) | platelet activating factor receptor |
| [Details](http://mirdb.org/cgi-bin/target_detail.cgi?targetID=1232134) | 20 | 97 | hsa-let-7d-5p | [PGRMC1](http://www.ncbi.nlm.nih.gov/entrez/query.fcgi?db=gene&cmd=Retrieve&dopt=full_report&list_uids=10857) | progesterone receptor membrane component 1 |
| [Details](http://mirdb.org/cgi-bin/target_detail.cgi?targetID=1231528) | 21 | 97 | hsa-let-7d-5p | [HIC2](http://www.ncbi.nlm.nih.gov/entrez/query.fcgi?db=gene&cmd=Retrieve&dopt=full_report&list_uids=23119) | HIC ZBTB transcriptional repressor 2 |
| [Details](http://mirdb.org/cgi-bin/target_detail.cgi?targetID=1232099) | 22 | 97 | hsa-let-7d-5p | [LRIG3](http://www.ncbi.nlm.nih.gov/entrez/query.fcgi?db=gene&cmd=Retrieve&dopt=full_report&list_uids=121227) | leucine rich repeats and immunoglobulin like domains 3 |
| [Details](http://mirdb.org/cgi-bin/target_detail.cgi?targetID=1231920) | 23 | 97 | hsa-let-7d-5p | [SLC10A7](http://www.ncbi.nlm.nih.gov/entrez/query.fcgi?db=gene&cmd=Retrieve&dopt=full_report&list_uids=84068) | solute carrier family 10 member 7 |
| [Details](http://mirdb.org/cgi-bin/target_detail.cgi?targetID=1231628) | 24 | 97 | hsa-let-7d-5p | [MIB1](http://www.ncbi.nlm.nih.gov/entrez/query.fcgi?db=gene&cmd=Retrieve&dopt=full_report&list_uids=57534) | mindbomb E3 ubiquitin protein ligase 1 |
| [Details](http://mirdb.org/cgi-bin/target_detail.cgi?targetID=1231565) | 25 | 97 | hsa-let-7d-5p | [NME6](http://www.ncbi.nlm.nih.gov/entrez/query.fcgi?db=gene&cmd=Retrieve&dopt=full_report&list_uids=10201) | NME/NM23 nucleoside diphosphate kinase 6 |
| [Details](http://mirdb.org/cgi-bin/target_detail.cgi?targetID=1232028) | 26 | 97 | hsa-let-7d-5p | [SFMBT1](http://www.ncbi.nlm.nih.gov/entrez/query.fcgi?db=gene&cmd=Retrieve&dopt=full_report&list_uids=51460) | Scm like with four mbt domains 1 |
| [Details](http://mirdb.org/cgi-bin/target_detail.cgi?targetID=1231621) | 27 | 97 | hsa-let-7d-5p | [ADAMTS15](http://www.ncbi.nlm.nih.gov/entrez/query.fcgi?db=gene&cmd=Retrieve&dopt=full_report&list_uids=170689) | ADAM metallopeptidase with thrombospondin type 1 motif 15 |
| [Details](http://mirdb.org/cgi-bin/target_detail.cgi?targetID=1231580) | 28 | 97 | hsa-let-7d-5p | [SLF2](http://www.ncbi.nlm.nih.gov/entrez/query.fcgi?db=gene&cmd=Retrieve&dopt=full_report&list_uids=55719) | SMC5-SMC6 complex localization factor 2 |
| [Details](http://mirdb.org/cgi-bin/target_detail.cgi?targetID=1232208) | 29 | 97 | hsa-let-7d-5p | [CBX5](http://www.ncbi.nlm.nih.gov/entrez/query.fcgi?db=gene&cmd=Retrieve&dopt=full_report&list_uids=23468) | chromobox 5 |
| [Details](http://mirdb.org/cgi-bin/target_detail.cgi?targetID=1231593) | 30 | 97 | hsa-let-7d-5p | [SALL4](http://www.ncbi.nlm.nih.gov/entrez/query.fcgi?db=gene&cmd=Retrieve&dopt=full_report&list_uids=57167) | spalt like transcription factor 4 |
| [Details](http://mirdb.org/cgi-bin/target_detail.cgi?targetID=1232293) | 31 | 97 | hsa-let-7d-5p | [ZNF512B](http://www.ncbi.nlm.nih.gov/entrez/query.fcgi?db=gene&cmd=Retrieve&dopt=full_report&list_uids=57473) | zinc finger protein 512B |
| [Details](http://mirdb.org/cgi-bin/target_detail.cgi?targetID=1231541) | 32 | 96 | hsa-let-7d-5p | [UTRN](http://www.ncbi.nlm.nih.gov/entrez/query.fcgi?db=gene&cmd=Retrieve&dopt=full_report&list_uids=7402) | utrophin |
| [Details](http://mirdb.org/cgi-bin/target_detail.cgi?targetID=1232296) | 33 | 96 | hsa-let-7d-5p | [ZBTB5](http://www.ncbi.nlm.nih.gov/entrez/query.fcgi?db=gene&cmd=Retrieve&dopt=full_report&list_uids=9925) | zinc finger and BTB domain containing 5 |
| [Details](http://mirdb.org/cgi-bin/target_detail.cgi?targetID=1231572) | 34 | 96 | hsa-let-7d-5p | [GNG5](http://www.ncbi.nlm.nih.gov/entrez/query.fcgi?db=gene&cmd=Retrieve&dopt=full_report&list_uids=2787) | G protein subunit gamma 5 |
| [Details](http://mirdb.org/cgi-bin/target_detail.cgi?targetID=1231741) | 35 | 96 | hsa-let-7d-5p | [PAPPA](http://www.ncbi.nlm.nih.gov/entrez/query.fcgi?db=gene&cmd=Retrieve&dopt=full_report&list_uids=5069) | pappalysin 1 |
| [Details](http://mirdb.org/cgi-bin/target_detail.cgi?targetID=1231679) | 36 | 96 | hsa-let-7d-5p | [USP44](http://www.ncbi.nlm.nih.gov/entrez/query.fcgi?db=gene&cmd=Retrieve&dopt=full_report&list_uids=84101) | ubiquitin specific peptidase 44 |
| [Details](http://mirdb.org/cgi-bin/target_detail.cgi?targetID=1232220) | 37 | 96 | hsa-let-7d-5p | [CCNJ](http://www.ncbi.nlm.nih.gov/entrez/query.fcgi?db=gene&cmd=Retrieve&dopt=full_report&list_uids=54619) | cyclin J |
| [Details](http://mirdb.org/cgi-bin/target_detail.cgi?targetID=1231469) | 38 | 96 | hsa-let-7d-5p | [NRAS](http://www.ncbi.nlm.nih.gov/entrez/query.fcgi?db=gene&cmd=Retrieve&dopt=full_report&list_uids=4893) | NRAS proto-oncogene, GTPase |
| [Details](http://mirdb.org/cgi-bin/target_detail.cgi?targetID=1232370) | 39 | 96 | hsa-let-7d-5p | [FZD3](http://www.ncbi.nlm.nih.gov/entrez/query.fcgi?db=gene&cmd=Retrieve&dopt=full_report&list_uids=7976) | frizzled class receptor 3 |
| [Details](http://mirdb.org/cgi-bin/target_detail.cgi?targetID=1231798) | 40 | 96 | hsa-let-7d-5p | [CDC34](http://www.ncbi.nlm.nih.gov/entrez/query.fcgi?db=gene&cmd=Retrieve&dopt=full_report&list_uids=997) | cell division cycle 34 |
| [Details](http://mirdb.org/cgi-bin/target_detail.cgi?targetID=1231490) | 41 | 96 | hsa-let-7d-5p | [ZNF710](http://www.ncbi.nlm.nih.gov/entrez/query.fcgi?db=gene&cmd=Retrieve&dopt=full_report&list_uids=374655) | zinc finger protein 710 |
| [Details](http://mirdb.org/cgi-bin/target_detail.cgi?targetID=1231779) | 42 | 96 | hsa-let-7d-5p | [LPGAT1](http://www.ncbi.nlm.nih.gov/entrez/query.fcgi?db=gene&cmd=Retrieve&dopt=full_report&list_uids=9926) | lysophosphatidylglycerol acyltransferase 1 |
| [Details](http://mirdb.org/cgi-bin/target_detail.cgi?targetID=1231901) | 43 | 95 | hsa-let-7d-5p | [ADAMTS8](http://www.ncbi.nlm.nih.gov/entrez/query.fcgi?db=gene&cmd=Retrieve&dopt=full_report&list_uids=11095) | ADAM metallopeptidase with thrombospondin type 1 motif 8 |
| [Details](http://mirdb.org/cgi-bin/target_detail.cgi?targetID=1231531) | 44 | 95 | hsa-let-7d-5p | [C8orf58](http://www.ncbi.nlm.nih.gov/entrez/query.fcgi?db=gene&cmd=Retrieve&dopt=full_report&list_uids=541565) | chromosome 8 open reading frame 58 |
| [Details](http://mirdb.org/cgi-bin/target_detail.cgi?targetID=1231857) | 45 | 95 | hsa-let-7d-5p | [IGF1R](http://www.ncbi.nlm.nih.gov/entrez/query.fcgi?db=gene&cmd=Retrieve&dopt=full_report&list_uids=3480) | insulin like growth factor 1 receptor |
| [Details](http://mirdb.org/cgi-bin/target_detail.cgi?targetID=1231893) | 46 | 95 | hsa-let-7d-5p | [AGO4](http://www.ncbi.nlm.nih.gov/entrez/query.fcgi?db=gene&cmd=Retrieve&dopt=full_report&list_uids=192670) | argonaute RISC catalytic component 4 |
| [Details](http://mirdb.org/cgi-bin/target_detail.cgi?targetID=1231710) | 47 | 95 | hsa-let-7d-5p | [BACH1](http://www.ncbi.nlm.nih.gov/entrez/query.fcgi?db=gene&cmd=Retrieve&dopt=full_report&list_uids=571) | BTB domain and CNC homolog 1 |
| [Details](http://mirdb.org/cgi-bin/target_detail.cgi?targetID=1231894) | 48 | 95 | hsa-let-7d-5p | [GNPTAB](http://www.ncbi.nlm.nih.gov/entrez/query.fcgi?db=gene&cmd=Retrieve&dopt=full_report&list_uids=79158) | N-acetylglucosamine-1-phosphate transferase subunits alpha and beta |
| [Details](http://mirdb.org/cgi-bin/target_detail.cgi?targetID=1232249) | 49 | 95 | hsa-let-7d-5p | [PIK3IP1](http://www.ncbi.nlm.nih.gov/entrez/query.fcgi?db=gene&cmd=Retrieve&dopt=full_report&list_uids=113791) | phosphoinositide-3-kinase interacting protein 1 |
| [Details](http://mirdb.org/cgi-bin/target_detail.cgi?targetID=1232034) | 50 | 95 | hsa-let-7d-5p | [THRSP](http://www.ncbi.nlm.nih.gov/entrez/query.fcgi?db=gene&cmd=Retrieve&dopt=full_report&list_uids=7069) | thyroid hormone responsive |
| [Details](http://mirdb.org/cgi-bin/target_detail.cgi?targetID=1232352) | 51 | 95 | hsa-let-7d-5p | [COL3A1](http://www.ncbi.nlm.nih.gov/entrez/query.fcgi?db=gene&cmd=Retrieve&dopt=full_report&list_uids=1281) | collagen type III alpha 1 chain |
| [Details](http://mirdb.org/cgi-bin/target_detail.cgi?targetID=1232355) | 52 | 95 | hsa-let-7d-5p | [BIN3](http://www.ncbi.nlm.nih.gov/entrez/query.fcgi?db=gene&cmd=Retrieve&dopt=full_report&list_uids=55909) | bridging integrator 3 |
| [Details](http://mirdb.org/cgi-bin/target_detail.cgi?targetID=1231937) | 53 | 95 | hsa-let-7d-5p | [ZSWIM5](http://www.ncbi.nlm.nih.gov/entrez/query.fcgi?db=gene&cmd=Retrieve&dopt=full_report&list_uids=57643) | zinc finger SWIM-type containing 5 |
| [Details](http://mirdb.org/cgi-bin/target_detail.cgi?targetID=1231651) | 54 | 95 | hsa-let-7d-5p | [SKIL](http://www.ncbi.nlm.nih.gov/entrez/query.fcgi?db=gene&cmd=Retrieve&dopt=full_report&list_uids=6498) | SKI like proto-oncogene |
| [Details](http://mirdb.org/cgi-bin/target_detail.cgi?targetID=1231618) | 55 | 95 | hsa-let-7d-5p | [PPP1R15B](http://www.ncbi.nlm.nih.gov/entrez/query.fcgi?db=gene&cmd=Retrieve&dopt=full_report&list_uids=84919) | protein phosphatase 1 regulatory subunit 15B |
| [Details](http://mirdb.org/cgi-bin/target_detail.cgi?targetID=1232356) | 56 | 95 | hsa-let-7d-5p | [STX3](http://www.ncbi.nlm.nih.gov/entrez/query.fcgi?db=gene&cmd=Retrieve&dopt=full_report&list_uids=6809) | syntaxin 3 |
| [Details](http://mirdb.org/cgi-bin/target_detail.cgi?targetID=1231990) | 57 | 94 | hsa-let-7d-5p | [DLST](http://www.ncbi.nlm.nih.gov/entrez/query.fcgi?db=gene&cmd=Retrieve&dopt=full_report&list_uids=1743) | dihydrolipoamide S-succinyltransferase |
| [Details](http://mirdb.org/cgi-bin/target_detail.cgi?targetID=1231546) | 58 | 94 | hsa-let-7d-5p | [ZNF644](http://www.ncbi.nlm.nih.gov/entrez/query.fcgi?db=gene&cmd=Retrieve&dopt=full_report&list_uids=84146) | zinc finger protein 644 |
| [Details](http://mirdb.org/cgi-bin/target_detail.cgi?targetID=1231808) | 59 | 94 | hsa-let-7d-5p | [E2F5](http://www.ncbi.nlm.nih.gov/entrez/query.fcgi?db=gene&cmd=Retrieve&dopt=full_report&list_uids=1875) | E2F transcription factor 5 |
| [Details](http://mirdb.org/cgi-bin/target_detail.cgi?targetID=1232027) | 60 | 94 | hsa-let-7d-5p | [ADRB2](http://www.ncbi.nlm.nih.gov/entrez/query.fcgi?db=gene&cmd=Retrieve&dopt=full_report&list_uids=154) | adrenoceptor beta 2 |
| [Details](http://mirdb.org/cgi-bin/target_detail.cgi?targetID=1231725) | 61 | 94 | hsa-let-7d-5p | [STK40](http://www.ncbi.nlm.nih.gov/entrez/query.fcgi?db=gene&cmd=Retrieve&dopt=full_report&list_uids=83931) | serine/threonine kinase 40 |
| [Details](http://mirdb.org/cgi-bin/target_detail.cgi?targetID=1231730) | 62 | 94 | hsa-let-7d-5p | [PXT1](http://www.ncbi.nlm.nih.gov/entrez/query.fcgi?db=gene&cmd=Retrieve&dopt=full_report&list_uids=222659) | peroxisomal testis enriched protein 1 |
| [Details](http://mirdb.org/cgi-bin/target_detail.cgi?targetID=1231573) | 63 | 94 | hsa-let-7d-5p | [SMIM3](http://www.ncbi.nlm.nih.gov/entrez/query.fcgi?db=gene&cmd=Retrieve&dopt=full_report&list_uids=85027) | small integral membrane protein 3 |
| [Details](http://mirdb.org/cgi-bin/target_detail.cgi?targetID=1231692) | 64 | 94 | hsa-let-7d-5p | [YOD1](http://www.ncbi.nlm.nih.gov/entrez/query.fcgi?db=gene&cmd=Retrieve&dopt=full_report&list_uids=55432) | YOD1 deubiquitinase |
| [Details](http://mirdb.org/cgi-bin/target_detail.cgi?targetID=1231839) | 65 | 94 | hsa-let-7d-5p | [COIL](http://www.ncbi.nlm.nih.gov/entrez/query.fcgi?db=gene&cmd=Retrieve&dopt=full_report&list_uids=8161) | coilin |
| [Details](http://mirdb.org/cgi-bin/target_detail.cgi?targetID=1232244) | 66 | 94 | hsa-let-7d-5p | [CLCN5](http://www.ncbi.nlm.nih.gov/entrez/query.fcgi?db=gene&cmd=Retrieve&dopt=full_report&list_uids=1184) | chloride voltage-gated channel 5 |
| [Details](http://mirdb.org/cgi-bin/target_detail.cgi?targetID=1232303) | 67 | 94 | hsa-let-7d-5p | [SMC1A](http://www.ncbi.nlm.nih.gov/entrez/query.fcgi?db=gene&cmd=Retrieve&dopt=full_report&list_uids=8243) | structural maintenance of chromosomes 1A |
| [Details](http://mirdb.org/cgi-bin/target_detail.cgi?targetID=1231585) | 68 | 94 | hsa-let-7d-5p | [TGFBR3](http://www.ncbi.nlm.nih.gov/entrez/query.fcgi?db=gene&cmd=Retrieve&dopt=full_report&list_uids=7049) | transforming growth factor beta receptor 3 |
| [Details](http://mirdb.org/cgi-bin/target_detail.cgi?targetID=1231680) | 69 | 94 | hsa-let-7d-5p | [CLDN12](http://www.ncbi.nlm.nih.gov/entrez/query.fcgi?db=gene&cmd=Retrieve&dopt=full_report&list_uids=9069) | claudin 12 |
| [Details](http://mirdb.org/cgi-bin/target_detail.cgi?targetID=1232135) | 70 | 94 | hsa-let-7d-5p | [DLC1](http://www.ncbi.nlm.nih.gov/entrez/query.fcgi?db=gene&cmd=Retrieve&dopt=full_report&list_uids=10395) | DLC1 Rho GTPase activating protein |
| [Details](http://mirdb.org/cgi-bin/target_detail.cgi?targetID=1231766) | 71 | 94 | hsa-let-7d-5p | [GXYLT1](http://www.ncbi.nlm.nih.gov/entrez/query.fcgi?db=gene&cmd=Retrieve&dopt=full_report&list_uids=283464) | glucoside xylosyltransferase 1 |
| [Details](http://mirdb.org/cgi-bin/target_detail.cgi?targetID=1232147) | 72 | 93 | hsa-let-7d-5p | [SLC16A9](http://www.ncbi.nlm.nih.gov/entrez/query.fcgi?db=gene&cmd=Retrieve&dopt=full_report&list_uids=220963) | solute carrier family 16 member 9 |
| [Details](http://mirdb.org/cgi-bin/target_detail.cgi?targetID=1231992) | 73 | 93 | hsa-let-7d-5p | [TMPRSS2](http://www.ncbi.nlm.nih.gov/entrez/query.fcgi?db=gene&cmd=Retrieve&dopt=full_report&list_uids=7113) | transmembrane serine protease 2 |
| [Details](http://mirdb.org/cgi-bin/target_detail.cgi?targetID=1231578) | 74 | 93 | hsa-let-7d-5p | [ATP8B4](http://www.ncbi.nlm.nih.gov/entrez/query.fcgi?db=gene&cmd=Retrieve&dopt=full_report&list_uids=79895) | ATPase phospholipid transporting 8B4 (putative) |
| [Details](http://mirdb.org/cgi-bin/target_detail.cgi?targetID=1232014) | 75 | 93 | hsa-let-7d-5p | [ADRB3](http://www.ncbi.nlm.nih.gov/entrez/query.fcgi?db=gene&cmd=Retrieve&dopt=full_report&list_uids=155) | adrenoceptor beta 3 |
| [Details](http://mirdb.org/cgi-bin/target_detail.cgi?targetID=1232221) | 76 | 93 | hsa-let-7d-5p | [TET3](http://www.ncbi.nlm.nih.gov/entrez/query.fcgi?db=gene&cmd=Retrieve&dopt=full_report&list_uids=200424) | tet methylcytosine dioxygenase 3 |
| [Details](http://mirdb.org/cgi-bin/target_detail.cgi?targetID=1231748) | 77 | 93 | hsa-let-7d-5p | [MAP4K3](http://www.ncbi.nlm.nih.gov/entrez/query.fcgi?db=gene&cmd=Retrieve&dopt=full_report&list_uids=8491) | mitogen-activated protein kinase kinase kinase kinase 3 |
| [Details](http://mirdb.org/cgi-bin/target_detail.cgi?targetID=1231790) | 78 | 93 | hsa-let-7d-5p | [DMD](http://www.ncbi.nlm.nih.gov/entrez/query.fcgi?db=gene&cmd=Retrieve&dopt=full_report&list_uids=1756) | dystrophin |
| [Details](http://mirdb.org/cgi-bin/target_detail.cgi?targetID=1232054) | 79 | 93 | hsa-let-7d-5p | [C5orf51](http://www.ncbi.nlm.nih.gov/entrez/query.fcgi?db=gene&cmd=Retrieve&dopt=full_report&list_uids=285636) | chromosome 5 open reading frame 51 |
| [Details](http://mirdb.org/cgi-bin/target_detail.cgi?targetID=1231767) | 80 | 93 | hsa-let-7d-5p | [NIPAL4](http://www.ncbi.nlm.nih.gov/entrez/query.fcgi?db=gene&cmd=Retrieve&dopt=full_report&list_uids=348938) | NIPA like domain containing 4 |
| [Details](http://mirdb.org/cgi-bin/target_detail.cgi?targetID=1231659) | 81 | 93 | hsa-let-7d-5p | [IGF2BP3](http://www.ncbi.nlm.nih.gov/entrez/query.fcgi?db=gene&cmd=Retrieve&dopt=full_report&list_uids=10643) | insulin like growth factor 2 mRNA binding protein 3 |
| [Details](http://mirdb.org/cgi-bin/target_detail.cgi?targetID=1231481) | 82 | 93 | hsa-let-7d-5p | [AHCTF1](http://www.ncbi.nlm.nih.gov/entrez/query.fcgi?db=gene&cmd=Retrieve&dopt=full_report&list_uids=25909) | AT-hook containing transcription factor 1 |
| [Details](http://mirdb.org/cgi-bin/target_detail.cgi?targetID=1231751) | 83 | 92 | hsa-let-7d-5p | [FAM122A](http://www.ncbi.nlm.nih.gov/entrez/query.fcgi?db=gene&cmd=Retrieve&dopt=full_report&list_uids=116224) | family with sequence similarity 122A |
| [Details](http://mirdb.org/cgi-bin/target_detail.cgi?targetID=1232189) | 84 | 92 | hsa-let-7d-5p | [HAND1](http://www.ncbi.nlm.nih.gov/entrez/query.fcgi?db=gene&cmd=Retrieve&dopt=full_report&list_uids=9421) | heart and neural crest derivatives expressed 1 |
| [Details](http://mirdb.org/cgi-bin/target_detail.cgi?targetID=1232308) | 85 | 92 | hsa-let-7d-5p | [ZNF280B](http://www.ncbi.nlm.nih.gov/entrez/query.fcgi?db=gene&cmd=Retrieve&dopt=full_report&list_uids=140883) | zinc finger protein 280B |
| [Details](http://mirdb.org/cgi-bin/target_detail.cgi?targetID=1231935) | 86 | 92 | hsa-let-7d-5p | [PEX11B](http://www.ncbi.nlm.nih.gov/entrez/query.fcgi?db=gene&cmd=Retrieve&dopt=full_report&list_uids=8799) | peroxisomal biogenesis factor 11 beta |
| [Details](http://mirdb.org/cgi-bin/target_detail.cgi?targetID=1232289) | 87 | 92 | hsa-let-7d-5p | [FAM189A1](http://www.ncbi.nlm.nih.gov/entrez/query.fcgi?db=gene&cmd=Retrieve&dopt=full_report&list_uids=23359) | family with sequence similarity 189 member A1 |
| [Details](http://mirdb.org/cgi-bin/target_detail.cgi?targetID=1231970) | 88 | 92 | hsa-let-7d-5p | [RUFY3](http://www.ncbi.nlm.nih.gov/entrez/query.fcgi?db=gene&cmd=Retrieve&dopt=full_report&list_uids=22902) | RUN and FYVE domain containing 3 |
| [Details](http://mirdb.org/cgi-bin/target_detail.cgi?targetID=1231610) | 89 | 92 | hsa-let-7d-5p | [WNT9B](http://www.ncbi.nlm.nih.gov/entrez/query.fcgi?db=gene&cmd=Retrieve&dopt=full_report&list_uids=7484) | Wnt family member 9B |
| [Details](http://mirdb.org/cgi-bin/target_detail.cgi?targetID=1232124) | 90 | 92 | hsa-let-7d-5p | [COL4A2](http://www.ncbi.nlm.nih.gov/entrez/query.fcgi?db=gene&cmd=Retrieve&dopt=full_report&list_uids=1284) | collagen type IV alpha 2 chain |
| [Details](http://mirdb.org/cgi-bin/target_detail.cgi?targetID=1231827) | 91 | 92 | hsa-let-7d-5p | [SENP2](http://www.ncbi.nlm.nih.gov/entrez/query.fcgi?db=gene&cmd=Retrieve&dopt=full_report&list_uids=59343) | SUMO specific peptidase 2 |
| [Details](http://mirdb.org/cgi-bin/target_detail.cgi?targetID=1231714) | 92 | 92 | hsa-let-7d-5p | [USP38](http://www.ncbi.nlm.nih.gov/entrez/query.fcgi?db=gene&cmd=Retrieve&dopt=full_report&list_uids=84640) | ubiquitin specific peptidase 38 |
| [Details](http://mirdb.org/cgi-bin/target_detail.cgi?targetID=1231655) | 93 | 92 | hsa-let-7d-5p | [PTPRD](http://www.ncbi.nlm.nih.gov/entrez/query.fcgi?db=gene&cmd=Retrieve&dopt=full_report&list_uids=5789) | protein tyrosine phosphatase, receptor type D |
| [Details](http://mirdb.org/cgi-bin/target_detail.cgi?targetID=1231629) | 94 | 92 | hsa-let-7d-5p | [DNA2](http://www.ncbi.nlm.nih.gov/entrez/query.fcgi?db=gene&cmd=Retrieve&dopt=full_report&list_uids=1763) | DNA replication helicase/nuclease 2 |
| [Details](http://mirdb.org/cgi-bin/target_detail.cgi?targetID=1231968) | 95 | 92 | hsa-let-7d-5p | [MARS2](http://www.ncbi.nlm.nih.gov/entrez/query.fcgi?db=gene&cmd=Retrieve&dopt=full_report&list_uids=92935) | methionyl-tRNA synthetase 2, mitochondrial |
| [Details](http://mirdb.org/cgi-bin/target_detail.cgi?targetID=1232254) | 96 | 92 | hsa-let-7d-5p | [BZW1](http://www.ncbi.nlm.nih.gov/entrez/query.fcgi?db=gene&cmd=Retrieve&dopt=full_report&list_uids=9689) | basic leucine zipper and W2 domains 1 |
| [Details](http://mirdb.org/cgi-bin/target_detail.cgi?targetID=1232255) | 97 | 92 | hsa-let-7d-5p | [CLP1](http://www.ncbi.nlm.nih.gov/entrez/query.fcgi?db=gene&cmd=Retrieve&dopt=full_report&list_uids=10978) | cleavage and polyadenylation factor I subunit 1 |
| [Details](http://mirdb.org/cgi-bin/target_detail.cgi?targetID=1231918) | 98 | 92 | hsa-let-7d-5p | [TSEN34](http://www.ncbi.nlm.nih.gov/entrez/query.fcgi?db=gene&cmd=Retrieve&dopt=full_report&list_uids=79042) | tRNA splicing endonuclease subunit 34 |
| [Details](http://mirdb.org/cgi-bin/target_detail.cgi?targetID=1231596) | 99 | 91 | hsa-let-7d-5p | [ONECUT2](http://www.ncbi.nlm.nih.gov/entrez/query.fcgi?db=gene&cmd=Retrieve&dopt=full_report&list_uids=9480) | one cut homeobox 2 |
| [Details](http://mirdb.org/cgi-bin/target_detail.cgi?targetID=1232205) | 100 | 91 | hsa-let-7d-5p | [SRGAP1](http://www.ncbi.nlm.nih.gov/entrez/query.fcgi?db=gene&cmd=Retrieve&dopt=full_report&list_uids=57522) | SLIT-ROBO Rho GTPase activating protein 1 |
| [Details](http://mirdb.org/cgi-bin/target_detail.cgi?targetID=1232281) | 101 | 91 | hsa-let-7d-5p | [HAS2](http://www.ncbi.nlm.nih.gov/entrez/query.fcgi?db=gene&cmd=Retrieve&dopt=full_report&list_uids=3037) | hyaluronan synthase 2 |
| [Details](http://mirdb.org/cgi-bin/target_detail.cgi?targetID=1231889) | 102 | 91 | hsa-let-7d-5p | [CEP135](http://www.ncbi.nlm.nih.gov/entrez/query.fcgi?db=gene&cmd=Retrieve&dopt=full_report&list_uids=9662) | centrosomal protein 135 |
| [Details](http://mirdb.org/cgi-bin/target_detail.cgi?targetID=1232064) | 103 | 91 | hsa-let-7d-5p | [ARHGEF38](http://www.ncbi.nlm.nih.gov/entrez/query.fcgi?db=gene&cmd=Retrieve&dopt=full_report&list_uids=54848) | Rho guanine nucleotide exchange factor 38 |
| [Details](http://mirdb.org/cgi-bin/target_detail.cgi?targetID=1232372) | 104 | 91 | hsa-let-7d-5p | [PLXNC1](http://www.ncbi.nlm.nih.gov/entrez/query.fcgi?db=gene&cmd=Retrieve&dopt=full_report&list_uids=10154) | plexin C1 |
| [Details](http://mirdb.org/cgi-bin/target_detail.cgi?targetID=1232022) | 105 | 91 | hsa-let-7d-5p | [TMPPE](http://www.ncbi.nlm.nih.gov/entrez/query.fcgi?db=gene&cmd=Retrieve&dopt=full_report&list_uids=643853) | transmembrane protein with metallophosphoesterase domain |
| [Details](http://mirdb.org/cgi-bin/target_detail.cgi?targetID=1231523) | 106 | 91 | hsa-let-7d-5p | [SLC35D2](http://www.ncbi.nlm.nih.gov/entrez/query.fcgi?db=gene&cmd=Retrieve&dopt=full_report&list_uids=11046) | solute carrier family 35 member D2 |
| [Details](http://mirdb.org/cgi-bin/target_detail.cgi?targetID=1232162) | 107 | 91 | hsa-let-7d-5p | [EDN1](http://www.ncbi.nlm.nih.gov/entrez/query.fcgi?db=gene&cmd=Retrieve&dopt=full_report&list_uids=1906) | endothelin 1 |
| [Details](http://mirdb.org/cgi-bin/target_detail.cgi?targetID=1231637) | 108 | 91 | hsa-let-7d-5p | [PCGF3](http://www.ncbi.nlm.nih.gov/entrez/query.fcgi?db=gene&cmd=Retrieve&dopt=full_report&list_uids=10336) | polycomb group ring finger 3 |
| [Details](http://mirdb.org/cgi-bin/target_detail.cgi?targetID=1231809) | 109 | 91 | hsa-let-7d-5p | [PBX1](http://www.ncbi.nlm.nih.gov/entrez/query.fcgi?db=gene&cmd=Retrieve&dopt=full_report&list_uids=5087) | PBX homeobox 1 |
| [Details](http://mirdb.org/cgi-bin/target_detail.cgi?targetID=1232222) | 110 | 91 | hsa-let-7d-5p | [ITGB3](http://www.ncbi.nlm.nih.gov/entrez/query.fcgi?db=gene&cmd=Retrieve&dopt=full_report&list_uids=3690) | integrin subunit beta 3 |
| [Details](http://mirdb.org/cgi-bin/target_detail.cgi?targetID=1232003) | 111 | 91 | hsa-let-7d-5p | [TMEM65](http://www.ncbi.nlm.nih.gov/entrez/query.fcgi?db=gene&cmd=Retrieve&dopt=full_report&list_uids=157378) | transmembrane protein 65 |
| [Details](http://mirdb.org/cgi-bin/target_detail.cgi?targetID=1232266) | 112 | 91 | hsa-let-7d-5p | [CEMIP2](http://www.ncbi.nlm.nih.gov/entrez/query.fcgi?db=gene&cmd=Retrieve&dopt=full_report&list_uids=23670) | cell migration inducing hyaluronidase 2 |
| [Details](http://mirdb.org/cgi-bin/target_detail.cgi?targetID=1231505) | 113 | 91 | hsa-let-7d-5p | [ZNF322](http://www.ncbi.nlm.nih.gov/entrez/query.fcgi?db=gene&cmd=Retrieve&dopt=full_report&list_uids=79692) | zinc finger protein 322 |
| [Details](http://mirdb.org/cgi-bin/target_detail.cgi?targetID=1231946) | 114 | 91 | hsa-let-7d-5p | [ZFYVE26](http://www.ncbi.nlm.nih.gov/entrez/query.fcgi?db=gene&cmd=Retrieve&dopt=full_report&list_uids=23503) | zinc finger FYVE-type containing 26 |
| [Details](http://mirdb.org/cgi-bin/target_detail.cgi?targetID=1231664) | 115 | 91 | hsa-let-7d-5p | [PLEKHA8](http://www.ncbi.nlm.nih.gov/entrez/query.fcgi?db=gene&cmd=Retrieve&dopt=full_report&list_uids=84725) | pleckstrin homology domain containing A8 |
| [Details](http://mirdb.org/cgi-bin/target_detail.cgi?targetID=1231653) | 116 | 90 | hsa-let-7d-5p | [DDI2](http://www.ncbi.nlm.nih.gov/entrez/query.fcgi?db=gene&cmd=Retrieve&dopt=full_report&list_uids=84301) | DNA damage inducible 1 homolog 2 |
| [Details](http://mirdb.org/cgi-bin/target_detail.cgi?targetID=1231897) | 117 | 90 | hsa-let-7d-5p | [CARNMT1](http://www.ncbi.nlm.nih.gov/entrez/query.fcgi?db=gene&cmd=Retrieve&dopt=full_report&list_uids=138199) | carnosine N-methyltransferase 1 |
| [Details](http://mirdb.org/cgi-bin/target_detail.cgi?targetID=1231627) | 118 | 90 | hsa-let-7d-5p | [RGS16](http://www.ncbi.nlm.nih.gov/entrez/query.fcgi?db=gene&cmd=Retrieve&dopt=full_report&list_uids=6004) | regulator of G protein signaling 16 |
| [Details](http://mirdb.org/cgi-bin/target_detail.cgi?targetID=1232112) | 119 | 90 | hsa-let-7d-5p | [MAPK6](http://www.ncbi.nlm.nih.gov/entrez/query.fcgi?db=gene&cmd=Retrieve&dopt=full_report&list_uids=5597) | mitogen-activated protein kinase 6 |
| [Details](http://mirdb.org/cgi-bin/target_detail.cgi?targetID=1232024) | 120 | 90 | hsa-let-7d-5p | [GAS7](http://www.ncbi.nlm.nih.gov/entrez/query.fcgi?db=gene&cmd=Retrieve&dopt=full_report&list_uids=8522) | growth arrest specific 7 |
| [Details](http://mirdb.org/cgi-bin/target_detail.cgi?targetID=1231884) | 121 | 90 | hsa-let-7d-5p | [MDM4](http://www.ncbi.nlm.nih.gov/entrez/query.fcgi?db=gene&cmd=Retrieve&dopt=full_report&list_uids=4194) | MDM4, p53 regulator |
| [Details](http://mirdb.org/cgi-bin/target_detail.cgi?targetID=1231898) | 122 | 90 | hsa-let-7d-5p | [FGF11](http://www.ncbi.nlm.nih.gov/entrez/query.fcgi?db=gene&cmd=Retrieve&dopt=full_report&list_uids=2256) | fibroblast growth factor 11 |
| [Details](http://mirdb.org/cgi-bin/target_detail.cgi?targetID=1231524) | 123 | 90 | hsa-let-7d-5p | [GDF6](http://www.ncbi.nlm.nih.gov/entrez/query.fcgi?db=gene&cmd=Retrieve&dopt=full_report&list_uids=392255) | growth differentiation factor 6 |
| [Details](http://mirdb.org/cgi-bin/target_detail.cgi?targetID=1231844) | 124 | 90 | hsa-let-7d-5p | [GALC](http://www.ncbi.nlm.nih.gov/entrez/query.fcgi?db=gene&cmd=Retrieve&dopt=full_report&list_uids=2581) | galactosylceramidase |
| [Details](http://mirdb.org/cgi-bin/target_detail.cgi?targetID=1231993) | 125 | 90 | hsa-let-7d-5p | [FRAS1](http://www.ncbi.nlm.nih.gov/entrez/query.fcgi?db=gene&cmd=Retrieve&dopt=full_report&list_uids=80144) | Fraser extracellular matrix complex subunit 1 |
| [Details](http://mirdb.org/cgi-bin/target_detail.cgi?targetID=1232084) | 126 | 90 | hsa-let-7d-5p | [E2F6](http://www.ncbi.nlm.nih.gov/entrez/query.fcgi?db=gene&cmd=Retrieve&dopt=full_report&list_uids=1876) | E2F transcription factor 6 |
| [Details](http://mirdb.org/cgi-bin/target_detail.cgi?targetID=1231912) | 127 | 90 | hsa-let-7d-5p | [TTLL4](http://www.ncbi.nlm.nih.gov/entrez/query.fcgi?db=gene&cmd=Retrieve&dopt=full_report&list_uids=9654) | tubulin tyrosine ligase like 4 |
| [Details](http://mirdb.org/cgi-bin/target_detail.cgi?targetID=1231771) | 128 | 90 | hsa-let-7d-5p | [ACVR1C](http://www.ncbi.nlm.nih.gov/entrez/query.fcgi?db=gene&cmd=Retrieve&dopt=full_report&list_uids=130399) | activin A receptor type 1C |
| [Details](http://mirdb.org/cgi-bin/target_detail.cgi?targetID=1232322) | 129 | 90 | hsa-let-7d-5p | [SLC5A9](http://www.ncbi.nlm.nih.gov/entrez/query.fcgi?db=gene&cmd=Retrieve&dopt=full_report&list_uids=200010) | solute carrier family 5 member 9 |
| [Details](http://mirdb.org/cgi-bin/target_detail.cgi?targetID=1232323) | 130 | 90 | hsa-let-7d-5p | [AMT](http://www.ncbi.nlm.nih.gov/entrez/query.fcgi?db=gene&cmd=Retrieve&dopt=full_report&list_uids=275) | aminomethyltransferase |
| [Details](http://mirdb.org/cgi-bin/target_detail.cgi?targetID=1232172) | 131 | 90 | hsa-let-7d-5p | [LIMD2](http://www.ncbi.nlm.nih.gov/entrez/query.fcgi?db=gene&cmd=Retrieve&dopt=full_report&list_uids=80774) | LIM domain containing 2 |
| [Details](http://mirdb.org/cgi-bin/target_detail.cgi?targetID=1231922) | 132 | 90 | hsa-let-7d-5p | [RASGRP1](http://www.ncbi.nlm.nih.gov/entrez/query.fcgi?db=gene&cmd=Retrieve&dopt=full_report&list_uids=10125) | RAS guanyl releasing protein 1 |
| [Details](http://mirdb.org/cgi-bin/target_detail.cgi?targetID=1231826) | 133 | 90 | hsa-let-7d-5p | [CPEB1](http://www.ncbi.nlm.nih.gov/entrez/query.fcgi?db=gene&cmd=Retrieve&dopt=full_report&list_uids=64506) | cytoplasmic polyadenylation element binding protein 1 |
| [Details](http://mirdb.org/cgi-bin/target_detail.cgi?targetID=1231932) | 134 | 90 | hsa-let-7d-5p | [FNIP1](http://www.ncbi.nlm.nih.gov/entrez/query.fcgi?db=gene&cmd=Retrieve&dopt=full_report&list_uids=96459) | folliculin interacting protein 1 |
| [Details](http://mirdb.org/cgi-bin/target_detail.cgi?targetID=1232344) | 135 | 90 | hsa-let-7d-5p | [NHLRC3](http://www.ncbi.nlm.nih.gov/entrez/query.fcgi?db=gene&cmd=Retrieve&dopt=full_report&list_uids=387921) | NHL repeat containing 3 |
| [Details](http://mirdb.org/cgi-bin/target_detail.cgi?targetID=1231851) | 136 | 90 | hsa-let-7d-5p | [XRN1](http://www.ncbi.nlm.nih.gov/entrez/query.fcgi?db=gene&cmd=Retrieve&dopt=full_report&list_uids=54464) | 5'-3' exoribonuclease 1 |
| [Details](http://mirdb.org/cgi-bin/target_detail.cgi?targetID=1231899) | 137 | 90 | hsa-let-7d-5p | [PBX2](http://www.ncbi.nlm.nih.gov/entrez/query.fcgi?db=gene&cmd=Retrieve&dopt=full_report&list_uids=5089) | PBX homeobox 2 |
| [Details](http://mirdb.org/cgi-bin/target_detail.cgi?targetID=1231579) | 138 | 90 | hsa-let-7d-5p | [DTX4](http://www.ncbi.nlm.nih.gov/entrez/query.fcgi?db=gene&cmd=Retrieve&dopt=full_report&list_uids=23220) | deltex E3 ubiquitin ligase 4 |
| [Details](http://mirdb.org/cgi-bin/target_detail.cgi?targetID=1231964) | 139 | 90 | hsa-let-7d-5p | [HIP1](http://www.ncbi.nlm.nih.gov/entrez/query.fcgi?db=gene&cmd=Retrieve&dopt=full_report&list_uids=3092) | huntingtin interacting protein 1 |
| [Details](http://mirdb.org/cgi-bin/target_detail.cgi?targetID=1232122) | 140 | 90 | hsa-let-7d-5p | [TRIM67](http://www.ncbi.nlm.nih.gov/entrez/query.fcgi?db=gene&cmd=Retrieve&dopt=full_report&list_uids=440730) | tripartite motif containing 67 |
| [Details](http://mirdb.org/cgi-bin/target_detail.cgi?targetID=1232141) | 141 | 90 | hsa-let-7d-5p | [LAMP2](http://www.ncbi.nlm.nih.gov/entrez/query.fcgi?db=gene&cmd=Retrieve&dopt=full_report&list_uids=3920) | lysosomal associated membrane protein 2 |
| [Details](http://mirdb.org/cgi-bin/target_detail.cgi?targetID=1231532) | 142 | 89 | hsa-let-7d-5p | [STIMATE](http://www.ncbi.nlm.nih.gov/entrez/query.fcgi?db=gene&cmd=Retrieve&dopt=full_report&list_uids=375346) | STIM activating enhancer |
| [Details](http://mirdb.org/cgi-bin/target_detail.cgi?targetID=1232331) | 143 | 89 | hsa-let-7d-5p | [RBFOX2](http://www.ncbi.nlm.nih.gov/entrez/query.fcgi?db=gene&cmd=Retrieve&dopt=full_report&list_uids=23543) | RNA binding fox-1 homolog 2 |
| [Details](http://mirdb.org/cgi-bin/target_detail.cgi?targetID=1231986) | 144 | 89 | hsa-let-7d-5p | [TMEM167A](http://www.ncbi.nlm.nih.gov/entrez/query.fcgi?db=gene&cmd=Retrieve&dopt=full_report&list_uids=153339) | transmembrane protein 167A |
| [Details](http://mirdb.org/cgi-bin/target_detail.cgi?targetID=1231787) | 145 | 89 | hsa-let-7d-5p | [ERCC6](http://www.ncbi.nlm.nih.gov/entrez/query.fcgi?db=gene&cmd=Retrieve&dopt=full_report&list_uids=2074) | ERCC excision repair 6, chromatin remodeling factor |
| [Details](http://mirdb.org/cgi-bin/target_detail.cgi?targetID=1231677) | 146 | 89 | hsa-let-7d-5p | [CNOT6L](http://www.ncbi.nlm.nih.gov/entrez/query.fcgi?db=gene&cmd=Retrieve&dopt=full_report&list_uids=246175) | CCR4-NOT transcription complex subunit 6 like |
| [Details](http://mirdb.org/cgi-bin/target_detail.cgi?targetID=1231944) | 147 | 89 | hsa-let-7d-5p | [RNF20](http://www.ncbi.nlm.nih.gov/entrez/query.fcgi?db=gene&cmd=Retrieve&dopt=full_report&list_uids=56254) | ring finger protein 20 |
| [Details](http://mirdb.org/cgi-bin/target_detail.cgi?targetID=1232204) | 148 | 89 | hsa-let-7d-5p | [CD59](http://www.ncbi.nlm.nih.gov/entrez/query.fcgi?db=gene&cmd=Retrieve&dopt=full_report&list_uids=966) | CD59 molecule (CD59 blood group) |
| [Details](http://mirdb.org/cgi-bin/target_detail.cgi?targetID=1231803) | 149 | 89 | hsa-let-7d-5p | [ZBP1](http://www.ncbi.nlm.nih.gov/entrez/query.fcgi?db=gene&cmd=Retrieve&dopt=full_report&list_uids=81030) | Z-DNA binding protein 1 |
| [Details](http://mirdb.org/cgi-bin/target_detail.cgi?targetID=1231525) | 150 | 89 | hsa-let-7d-5p | [OSBPL3](http://www.ncbi.nlm.nih.gov/entrez/query.fcgi?db=gene&cmd=Retrieve&dopt=full_report&list_uids=26031) | oxysterol binding protein like 3 |
| [Details](http://mirdb.org/cgi-bin/target_detail.cgi?targetID=1232389) | 151 | 89 | hsa-let-7d-5p | [DTX2](http://www.ncbi.nlm.nih.gov/entrez/query.fcgi?db=gene&cmd=Retrieve&dopt=full_report&list_uids=113878) | deltex E3 ubiquitin ligase 2 |
| [Details](http://mirdb.org/cgi-bin/target_detail.cgi?targetID=1232260) | 152 | 89 | hsa-let-7d-5p | [PBX3](http://www.ncbi.nlm.nih.gov/entrez/query.fcgi?db=gene&cmd=Retrieve&dopt=full_report&list_uids=5090) | PBX homeobox 3 |
| [Details](http://mirdb.org/cgi-bin/target_detail.cgi?targetID=1232078) | 153 | 89 | hsa-let-7d-5p | [PPP1R16B](http://www.ncbi.nlm.nih.gov/entrez/query.fcgi?db=gene&cmd=Retrieve&dopt=full_report&list_uids=26051) | protein phosphatase 1 regulatory subunit 16B |
| [Details](http://mirdb.org/cgi-bin/target_detail.cgi?targetID=1231506) | 154 | 89 | hsa-let-7d-5p | [GALNT2](http://www.ncbi.nlm.nih.gov/entrez/query.fcgi?db=gene&cmd=Retrieve&dopt=full_report&list_uids=2590) | polypeptide N-acetylgalactosaminyltransferase 2 |
| [Details](http://mirdb.org/cgi-bin/target_detail.cgi?targetID=1231605) | 155 | 89 | hsa-let-7d-5p | [FZD4](http://www.ncbi.nlm.nih.gov/entrez/query.fcgi?db=gene&cmd=Retrieve&dopt=full_report&list_uids=8322) | frizzled class receptor 4 |
| [Details](http://mirdb.org/cgi-bin/target_detail.cgi?targetID=1232207) | 156 | 89 | hsa-let-7d-5p | [COL1A2](http://www.ncbi.nlm.nih.gov/entrez/query.fcgi?db=gene&cmd=Retrieve&dopt=full_report&list_uids=1278) | collagen type I alpha 2 chain |
| [Details](http://mirdb.org/cgi-bin/target_detail.cgi?targetID=1232219) | 157 | 89 | hsa-let-7d-5p | [USP24](http://www.ncbi.nlm.nih.gov/entrez/query.fcgi?db=gene&cmd=Retrieve&dopt=full_report&list_uids=23358) | ubiquitin specific peptidase 24 |
| [Details](http://mirdb.org/cgi-bin/target_detail.cgi?targetID=1231584) | 158 | 89 | hsa-let-7d-5p | [CPA4](http://www.ncbi.nlm.nih.gov/entrez/query.fcgi?db=gene&cmd=Retrieve&dopt=full_report&list_uids=51200) | carboxypeptidase A4 |
| [Details](http://mirdb.org/cgi-bin/target_detail.cgi?targetID=1232325) | 159 | 89 | hsa-let-7d-5p | [SOCS4](http://www.ncbi.nlm.nih.gov/entrez/query.fcgi?db=gene&cmd=Retrieve&dopt=full_report&list_uids=122809) | suppressor of cytokine signaling 4 |
| [Details](http://mirdb.org/cgi-bin/target_detail.cgi?targetID=1232137) | 160 | 89 | hsa-let-7d-5p | [SLC20A1](http://www.ncbi.nlm.nih.gov/entrez/query.fcgi?db=gene&cmd=Retrieve&dopt=full_report&list_uids=6574) | solute carrier family 20 member 1 |
| [Details](http://mirdb.org/cgi-bin/target_detail.cgi?targetID=1231828) | 161 | 89 | hsa-let-7d-5p | [HDLBP](http://www.ncbi.nlm.nih.gov/entrez/query.fcgi?db=gene&cmd=Retrieve&dopt=full_report&list_uids=3069) | high density lipoprotein binding protein |
| [Details](http://mirdb.org/cgi-bin/target_detail.cgi?targetID=1232070) | 162 | 89 | hsa-let-7d-5p | [TGFBR1](http://www.ncbi.nlm.nih.gov/entrez/query.fcgi?db=gene&cmd=Retrieve&dopt=full_report&list_uids=7046) | transforming growth factor beta receptor 1 |
| [Details](http://mirdb.org/cgi-bin/target_detail.cgi?targetID=1231750) | 163 | 88 | hsa-let-7d-5p | [THOC2](http://www.ncbi.nlm.nih.gov/entrez/query.fcgi?db=gene&cmd=Retrieve&dopt=full_report&list_uids=57187) | THO complex 2 |
| [Details](http://mirdb.org/cgi-bin/target_detail.cgi?targetID=1231758) | 164 | 88 | hsa-let-7d-5p | [SPRYD4](http://www.ncbi.nlm.nih.gov/entrez/query.fcgi?db=gene&cmd=Retrieve&dopt=full_report&list_uids=283377) | SPRY domain containing 4 |
| [Details](http://mirdb.org/cgi-bin/target_detail.cgi?targetID=1232232) | 165 | 88 | hsa-let-7d-5p | [AP1S1](http://www.ncbi.nlm.nih.gov/entrez/query.fcgi?db=gene&cmd=Retrieve&dopt=full_report&list_uids=1174) | adaptor related protein complex 1 subunit sigma 1 |
| [Details](http://mirdb.org/cgi-bin/target_detail.cgi?targetID=1231674) | 166 | 88 | hsa-let-7d-5p | [ELP1](http://www.ncbi.nlm.nih.gov/entrez/query.fcgi?db=gene&cmd=Retrieve&dopt=full_report&list_uids=8518) | elongator complex protein 1 |
| [Details](http://mirdb.org/cgi-bin/target_detail.cgi?targetID=1231658) | 167 | 88 | hsa-let-7d-5p | [HSPA14](http://www.ncbi.nlm.nih.gov/entrez/query.fcgi?db=gene&cmd=Retrieve&dopt=full_report&list_uids=51182) | heat shock protein family A (Hsp70) member 14 |
| [Details](http://mirdb.org/cgi-bin/target_detail.cgi?targetID=1231807) | 168 | 88 | hsa-let-7d-5p | [INSR](http://www.ncbi.nlm.nih.gov/entrez/query.fcgi?db=gene&cmd=Retrieve&dopt=full_report&list_uids=3643) | insulin receptor |
| [Details](http://mirdb.org/cgi-bin/target_detail.cgi?targetID=1231717) | 169 | 88 | hsa-let-7d-5p | [ERO1A](http://www.ncbi.nlm.nih.gov/entrez/query.fcgi?db=gene&cmd=Retrieve&dopt=full_report&list_uids=30001) | endoplasmic reticulum oxidoreductase 1 alpha |
| [Details](http://mirdb.org/cgi-bin/target_detail.cgi?targetID=1231842) | 170 | 88 | hsa-let-7d-5p | [SCN4B](http://www.ncbi.nlm.nih.gov/entrez/query.fcgi?db=gene&cmd=Retrieve&dopt=full_report&list_uids=6330) | sodium voltage-gated channel beta subunit 4 |
| [Details](http://mirdb.org/cgi-bin/target_detail.cgi?targetID=1232165) | 171 | 88 | hsa-let-7d-5p | [CPEB2](http://www.ncbi.nlm.nih.gov/entrez/query.fcgi?db=gene&cmd=Retrieve&dopt=full_report&list_uids=132864) | cytoplasmic polyadenylation element binding protein 2 |
| [Details](http://mirdb.org/cgi-bin/target_detail.cgi?targetID=1232214) | 172 | 88 | hsa-let-7d-5p | [GPR26](http://www.ncbi.nlm.nih.gov/entrez/query.fcgi?db=gene&cmd=Retrieve&dopt=full_report&list_uids=2849) | G protein-coupled receptor 26 |
| [Details](http://mirdb.org/cgi-bin/target_detail.cgi?targetID=1232111) | 173 | 88 | hsa-let-7d-5p | [PXDN](http://www.ncbi.nlm.nih.gov/entrez/query.fcgi?db=gene&cmd=Retrieve&dopt=full_report&list_uids=7837) | peroxidasin |
| [Details](http://mirdb.org/cgi-bin/target_detail.cgi?targetID=1232211) | 174 | 88 | hsa-let-7d-5p | [CD164](http://www.ncbi.nlm.nih.gov/entrez/query.fcgi?db=gene&cmd=Retrieve&dopt=full_report&list_uids=8763) | CD164 molecule |
| [Details](http://mirdb.org/cgi-bin/target_detail.cgi?targetID=1231978) | 175 | 88 | hsa-let-7d-5p | [HDX](http://www.ncbi.nlm.nih.gov/entrez/query.fcgi?db=gene&cmd=Retrieve&dopt=full_report&list_uids=139324) | highly divergent homeobox |
| [Details](http://mirdb.org/cgi-bin/target_detail.cgi?targetID=1231529) | 176 | 88 | hsa-let-7d-5p | [SLC31A2](http://www.ncbi.nlm.nih.gov/entrez/query.fcgi?db=gene&cmd=Retrieve&dopt=full_report&list_uids=1318) | solute carrier family 31 member 2 |
| [Details](http://mirdb.org/cgi-bin/target_detail.cgi?targetID=1231972) | 177 | 88 | hsa-let-7d-5p | [RGS6](http://www.ncbi.nlm.nih.gov/entrez/query.fcgi?db=gene&cmd=Retrieve&dopt=full_report&list_uids=9628) | regulator of G protein signaling 6 |
| [Details](http://mirdb.org/cgi-bin/target_detail.cgi?targetID=1231881) | 178 | 88 | hsa-let-7d-5p | [KLHDC8B](http://www.ncbi.nlm.nih.gov/entrez/query.fcgi?db=gene&cmd=Retrieve&dopt=full_report&list_uids=200942) | kelch domain containing 8B |
| [Details](http://mirdb.org/cgi-bin/target_detail.cgi?targetID=1231631) | 179 | 88 | hsa-let-7d-5p | [FAM135A](http://www.ncbi.nlm.nih.gov/entrez/query.fcgi?db=gene&cmd=Retrieve&dopt=full_report&list_uids=57579) | family with sequence similarity 135 member A |
| [Details](http://mirdb.org/cgi-bin/target_detail.cgi?targetID=1232019) | 180 | 88 | hsa-let-7d-5p | [PALD1](http://www.ncbi.nlm.nih.gov/entrez/query.fcgi?db=gene&cmd=Retrieve&dopt=full_report&list_uids=27143) | phosphatase domain containing paladin 1 |
| [Details](http://mirdb.org/cgi-bin/target_detail.cgi?targetID=1232367) | 181 | 88 | hsa-let-7d-5p | [KCTD21](http://www.ncbi.nlm.nih.gov/entrez/query.fcgi?db=gene&cmd=Retrieve&dopt=full_report&list_uids=283219) | potassium channel tetramerization domain containing 21 |
| [Details](http://mirdb.org/cgi-bin/target_detail.cgi?targetID=1231492) | 182 | 88 | hsa-let-7d-5p | [MRS2](http://www.ncbi.nlm.nih.gov/entrez/query.fcgi?db=gene&cmd=Retrieve&dopt=full_report&list_uids=57380) | magnesium transporter MRS2 |
| [Details](http://mirdb.org/cgi-bin/target_detail.cgi?targetID=1232033) | 183 | 88 | hsa-let-7d-5p | [COL4A1](http://www.ncbi.nlm.nih.gov/entrez/query.fcgi?db=gene&cmd=Retrieve&dopt=full_report&list_uids=1282) | collagen type IV alpha 1 chain |
| [Details](http://mirdb.org/cgi-bin/target_detail.cgi?targetID=1232353) | 184 | 88 | hsa-let-7d-5p | [ASAP1](http://www.ncbi.nlm.nih.gov/entrez/query.fcgi?db=gene&cmd=Retrieve&dopt=full_report&list_uids=50807) | ArfGAP with SH3 domain, ankyrin repeat and PH domain 1 |
| [Details](http://mirdb.org/cgi-bin/target_detail.cgi?targetID=1231958) | 185 | 88 | hsa-let-7d-5p | [TMOD2](http://www.ncbi.nlm.nih.gov/entrez/query.fcgi?db=gene&cmd=Retrieve&dopt=full_report&list_uids=29767) | tropomodulin 2 |
| [Details](http://mirdb.org/cgi-bin/target_detail.cgi?targetID=1231956) | 186 | 88 | hsa-let-7d-5p | [ACTA1](http://www.ncbi.nlm.nih.gov/entrez/query.fcgi?db=gene&cmd=Retrieve&dopt=full_report&list_uids=58) | actin, alpha 1, skeletal muscle |
| [Details](http://mirdb.org/cgi-bin/target_detail.cgi?targetID=1231871) | 187 | 88 | hsa-let-7d-5p | [DPP6](http://www.ncbi.nlm.nih.gov/entrez/query.fcgi?db=gene&cmd=Retrieve&dopt=full_report&list_uids=1804) | dipeptidyl peptidase like 6 |
| [Details](http://mirdb.org/cgi-bin/target_detail.cgi?targetID=1232100) | 188 | 87 | hsa-let-7d-5p | [NKAPD1](http://www.ncbi.nlm.nih.gov/entrez/query.fcgi?db=gene&cmd=Retrieve&dopt=full_report&list_uids=55216) | NKAP domain containing 1 |
| [Details](http://mirdb.org/cgi-bin/target_detail.cgi?targetID=1231752) | 189 | 87 | hsa-let-7d-5p | [COL4A6](http://www.ncbi.nlm.nih.gov/entrez/query.fcgi?db=gene&cmd=Retrieve&dopt=full_report&list_uids=1288) | collagen type IV alpha 6 chain |
| [Details](http://mirdb.org/cgi-bin/target_detail.cgi?targetID=1231645) | 190 | 87 | hsa-let-7d-5p | [FNDC3A](http://www.ncbi.nlm.nih.gov/entrez/query.fcgi?db=gene&cmd=Retrieve&dopt=full_report&list_uids=22862) | fibronectin type III domain containing 3A |
| [Details](http://mirdb.org/cgi-bin/target_detail.cgi?targetID=1231549) | 191 | 87 | hsa-let-7d-5p | [DDX19B](http://www.ncbi.nlm.nih.gov/entrez/query.fcgi?db=gene&cmd=Retrieve&dopt=full_report&list_uids=11269) | DEAD-box helicase 19B |
| [Details](http://mirdb.org/cgi-bin/target_detail.cgi?targetID=1231663) | 192 | 87 | hsa-let-7d-5p | [B3GNT7](http://www.ncbi.nlm.nih.gov/entrez/query.fcgi?db=gene&cmd=Retrieve&dopt=full_report&list_uids=93010) | UDP-GlcNAc:betaGal beta-1,3-N-acetylglucosaminyltransferase 7 |
| [Details](http://mirdb.org/cgi-bin/target_detail.cgi?targetID=1231550) | 193 | 87 | hsa-let-7d-5p | [KCNC2](http://www.ncbi.nlm.nih.gov/entrez/query.fcgi?db=gene&cmd=Retrieve&dopt=full_report&list_uids=3747) | potassium voltage-gated channel subfamily C member 2 |
| [Details](http://mirdb.org/cgi-bin/target_detail.cgi?targetID=1232093) | 194 | 87 | hsa-let-7d-5p | [PRSS22](http://www.ncbi.nlm.nih.gov/entrez/query.fcgi?db=gene&cmd=Retrieve&dopt=full_report&list_uids=64063) | serine protease 22 |
| [Details](http://mirdb.org/cgi-bin/target_detail.cgi?targetID=1231599) | 195 | 87 | hsa-let-7d-5p | [BEGAIN](http://www.ncbi.nlm.nih.gov/entrez/query.fcgi?db=gene&cmd=Retrieve&dopt=full_report&list_uids=57596) | brain enriched guanylate kinase associated |
| [Details](http://mirdb.org/cgi-bin/target_detail.cgi?targetID=1232294) | 196 | 87 | hsa-let-7d-5p | [OSMR](http://www.ncbi.nlm.nih.gov/entrez/query.fcgi?db=gene&cmd=Retrieve&dopt=full_report&list_uids=9180) | oncostatin M receptor |
| [Details](http://mirdb.org/cgi-bin/target_detail.cgi?targetID=1231761) | 197 | 87 | hsa-let-7d-5p | [LBR](http://www.ncbi.nlm.nih.gov/entrez/query.fcgi?db=gene&cmd=Retrieve&dopt=full_report&list_uids=3930) | lamin B receptor |
| [Details](http://mirdb.org/cgi-bin/target_detail.cgi?targetID=1232080) | 198 | 87 | hsa-let-7d-5p | [PDPR](http://www.ncbi.nlm.nih.gov/entrez/query.fcgi?db=gene&cmd=Retrieve&dopt=full_report&list_uids=55066) | pyruvate dehydrogenase phosphatase regulatory subunit |
| [Details](http://mirdb.org/cgi-bin/target_detail.cgi?targetID=1232198) | 199 | 87 | hsa-let-7d-5p | [ZNF275](http://www.ncbi.nlm.nih.gov/entrez/query.fcgi?db=gene&cmd=Retrieve&dopt=full_report&list_uids=10838) | zinc finger protein 275 |
| [Details](http://mirdb.org/cgi-bin/target_detail.cgi?targetID=1232105) | 200 | 87 | hsa-let-7d-5p | [COL27A1](http://www.ncbi.nlm.nih.gov/entrez/query.fcgi?db=gene&cmd=Retrieve&dopt=full_report&list_uids=85301) | collagen type XXVII alpha 1 chain |
| [Details](http://mirdb.org/cgi-bin/target_detail.cgi?targetID=1231941) | 201 | 87 | hsa-let-7d-5p | [PLXND1](http://www.ncbi.nlm.nih.gov/entrez/query.fcgi?db=gene&cmd=Retrieve&dopt=full_report&list_uids=23129) | plexin D1 |
| [Details](http://mirdb.org/cgi-bin/target_detail.cgi?targetID=1231566) | 202 | 87 | hsa-let-7d-5p | [IQCB1](http://www.ncbi.nlm.nih.gov/entrez/query.fcgi?db=gene&cmd=Retrieve&dopt=full_report&list_uids=9657) | IQ motif containing B1 |
| [Details](http://mirdb.org/cgi-bin/target_detail.cgi?targetID=1232239) | 203 | 87 | hsa-let-7d-5p | [FBXL12](http://www.ncbi.nlm.nih.gov/entrez/query.fcgi?db=gene&cmd=Retrieve&dopt=full_report&list_uids=54850) | F-box and leucine rich repeat protein 12 |
| [Details](http://mirdb.org/cgi-bin/target_detail.cgi?targetID=1231475) | 204 | 87 | hsa-let-7d-5p | [MAP3K9](http://www.ncbi.nlm.nih.gov/entrez/query.fcgi?db=gene&cmd=Retrieve&dopt=full_report&list_uids=4293) | mitogen-activated protein kinase kinase kinase 9 |
| [Details](http://mirdb.org/cgi-bin/target_detail.cgi?targetID=1232241) | 205 | 87 | hsa-let-7d-5p | [HECTD2](http://www.ncbi.nlm.nih.gov/entrez/query.fcgi?db=gene&cmd=Retrieve&dopt=full_report&list_uids=143279) | HECT domain E3 ubiquitin protein ligase 2 |
| [Details](http://mirdb.org/cgi-bin/target_detail.cgi?targetID=1231961) | 206 | 87 | hsa-let-7d-5p | [KCTD17](http://www.ncbi.nlm.nih.gov/entrez/query.fcgi?db=gene&cmd=Retrieve&dopt=full_report&list_uids=79734) | potassium channel tetramerization domain containing 17 |
| [Details](http://mirdb.org/cgi-bin/target_detail.cgi?targetID=1231757) | 207 | 87 | hsa-let-7d-5p | [ZNF583](http://www.ncbi.nlm.nih.gov/entrez/query.fcgi?db=gene&cmd=Retrieve&dopt=full_report&list_uids=147949) | zinc finger protein 583 |
| [Details](http://mirdb.org/cgi-bin/target_detail.cgi?targetID=1231722) | 208 | 86 | hsa-let-7d-5p | [RAB11FIP4](http://www.ncbi.nlm.nih.gov/entrez/query.fcgi?db=gene&cmd=Retrieve&dopt=full_report&list_uids=84440) | RAB11 family interacting protein 4 |
| [Details](http://mirdb.org/cgi-bin/target_detail.cgi?targetID=1232329) | 209 | 86 | hsa-let-7d-5p | [PLA2G3](http://www.ncbi.nlm.nih.gov/entrez/query.fcgi?db=gene&cmd=Retrieve&dopt=full_report&list_uids=50487) | phospholipase A2 group III |
| [Details](http://mirdb.org/cgi-bin/target_detail.cgi?targetID=1232327) | 210 | 86 | hsa-let-7d-5p | [MED8](http://www.ncbi.nlm.nih.gov/entrez/query.fcgi?db=gene&cmd=Retrieve&dopt=full_report&list_uids=112950) | mediator complex subunit 8 |
| [Details](http://mirdb.org/cgi-bin/target_detail.cgi?targetID=1232085) | 211 | 86 | hsa-let-7d-5p | [GCNT4](http://www.ncbi.nlm.nih.gov/entrez/query.fcgi?db=gene&cmd=Retrieve&dopt=full_report&list_uids=51301) | glucosaminyl (N-acetyl) transferase 4 |
| [Details](http://mirdb.org/cgi-bin/target_detail.cgi?targetID=1232174) | 212 | 86 | hsa-let-7d-5p | [LRIG2](http://www.ncbi.nlm.nih.gov/entrez/query.fcgi?db=gene&cmd=Retrieve&dopt=full_report&list_uids=9860) | leucine rich repeats and immunoglobulin like domains 2 |
| [Details](http://mirdb.org/cgi-bin/target_detail.cgi?targetID=1231915) | 213 | 86 | hsa-let-7d-5p | [CASP3](http://www.ncbi.nlm.nih.gov/entrez/query.fcgi?db=gene&cmd=Retrieve&dopt=full_report&list_uids=836) | caspase 3 |
| [Details](http://mirdb.org/cgi-bin/target_detail.cgi?targetID=1232173) | 214 | 86 | hsa-let-7d-5p | [DNAJC1](http://www.ncbi.nlm.nih.gov/entrez/query.fcgi?db=gene&cmd=Retrieve&dopt=full_report&list_uids=64215) | DnaJ heat shock protein family (Hsp40) member C1 |
| [Details](http://mirdb.org/cgi-bin/target_detail.cgi?targetID=1231543) | 215 | 86 | hsa-let-7d-5p | [SLC5A6](http://www.ncbi.nlm.nih.gov/entrez/query.fcgi?db=gene&cmd=Retrieve&dopt=full_report&list_uids=8884) | solute carrier family 5 member 6 |
| [Details](http://mirdb.org/cgi-bin/target_detail.cgi?targetID=1232362) | 216 | 86 | hsa-let-7d-5p | [FAM104A](http://www.ncbi.nlm.nih.gov/entrez/query.fcgi?db=gene&cmd=Retrieve&dopt=full_report&list_uids=84923) | family with sequence similarity 104 member A |
| [Details](http://mirdb.org/cgi-bin/target_detail.cgi?targetID=1231502) | 217 | 86 | hsa-let-7d-5p | [MYCN](http://www.ncbi.nlm.nih.gov/entrez/query.fcgi?db=gene&cmd=Retrieve&dopt=full_report&list_uids=4613) | MYCN proto-oncogene, bHLH transcription factor |
| [Details](http://mirdb.org/cgi-bin/target_detail.cgi?targetID=1231951) | 218 | 86 | hsa-let-7d-5p | [IL13](http://www.ncbi.nlm.nih.gov/entrez/query.fcgi?db=gene&cmd=Retrieve&dopt=full_report&list_uids=3596) | interleukin 13 |
| [Details](http://mirdb.org/cgi-bin/target_detail.cgi?targetID=1232140) | 219 | 86 | hsa-let-7d-5p | [XKR8](http://www.ncbi.nlm.nih.gov/entrez/query.fcgi?db=gene&cmd=Retrieve&dopt=full_report&list_uids=55113) | XK related 8 |
| [Details](http://mirdb.org/cgi-bin/target_detail.cgi?targetID=1232349) | 220 | 86 | hsa-let-7d-5p | [ENTPD7](http://www.ncbi.nlm.nih.gov/entrez/query.fcgi?db=gene&cmd=Retrieve&dopt=full_report&list_uids=57089) | ectonucleoside triphosphate diphosphohydrolase 7 |
| [Details](http://mirdb.org/cgi-bin/target_detail.cgi?targetID=1232117) | 221 | 86 | hsa-let-7d-5p | [OPA3](http://www.ncbi.nlm.nih.gov/entrez/query.fcgi?db=gene&cmd=Retrieve&dopt=full_report&list_uids=80207) | OPA3, outer mitochondrial membrane lipid metabolism regulator |
| [Details](http://mirdb.org/cgi-bin/target_detail.cgi?targetID=1232119) | 222 | 86 | hsa-let-7d-5p | [ERCC4](http://www.ncbi.nlm.nih.gov/entrez/query.fcgi?db=gene&cmd=Retrieve&dopt=full_report&list_uids=2072) | ERCC excision repair 4, endonuclease catalytic subunit |
| [Details](http://mirdb.org/cgi-bin/target_detail.cgi?targetID=1231744) | 223 | 86 | hsa-let-7d-5p | [GAN](http://www.ncbi.nlm.nih.gov/entrez/query.fcgi?db=gene&cmd=Retrieve&dopt=full_report&list_uids=8139) | gigaxonin |
| [Details](http://mirdb.org/cgi-bin/target_detail.cgi?targetID=1232156) | 224 | 86 | hsa-let-7d-5p | [DHX57](http://www.ncbi.nlm.nih.gov/entrez/query.fcgi?db=gene&cmd=Retrieve&dopt=full_report&list_uids=90957) | DExH-box helicase 57 |
| [Details](http://mirdb.org/cgi-bin/target_detail.cgi?targetID=1231745) | 225 | 86 | hsa-let-7d-5p | [SIGLEC5](http://www.ncbi.nlm.nih.gov/entrez/query.fcgi?db=gene&cmd=Retrieve&dopt=full_report&list_uids=8778) | sialic acid binding Ig like lectin 5 |
| [Details](http://mirdb.org/cgi-bin/target_detail.cgi?targetID=1231947) | 226 | 86 | hsa-let-7d-5p | [ZBTB8B](http://www.ncbi.nlm.nih.gov/entrez/query.fcgi?db=gene&cmd=Retrieve&dopt=full_report&list_uids=728116) | zinc finger and BTB domain containing 8B |
| [Details](http://mirdb.org/cgi-bin/target_detail.cgi?targetID=1231650) | 227 | 86 | hsa-let-7d-5p | [ELF4](http://www.ncbi.nlm.nih.gov/entrez/query.fcgi?db=gene&cmd=Retrieve&dopt=full_report&list_uids=2000) | E74 like ETS transcription factor 4 |
| [Details](http://mirdb.org/cgi-bin/target_detail.cgi?targetID=1232253) | 228 | 86 | hsa-let-7d-5p | [CERCAM](http://www.ncbi.nlm.nih.gov/entrez/query.fcgi?db=gene&cmd=Retrieve&dopt=full_report&list_uids=51148) | cerebral endothelial cell adhesion molecule |
| [Details](http://mirdb.org/cgi-bin/target_detail.cgi?targetID=1232297) | 229 | 86 | hsa-let-7d-5p | [ZNF516](http://www.ncbi.nlm.nih.gov/entrez/query.fcgi?db=gene&cmd=Retrieve&dopt=full_report&list_uids=9658) | zinc finger protein 516 |
| [Details](http://mirdb.org/cgi-bin/target_detail.cgi?targetID=1232274) | 230 | 86 | hsa-let-7d-5p | [CEP120](http://www.ncbi.nlm.nih.gov/entrez/query.fcgi?db=gene&cmd=Retrieve&dopt=full_report&list_uids=153241) | centrosomal protein 120 |
| [Details](http://mirdb.org/cgi-bin/target_detail.cgi?targetID=1231643) | 231 | 86 | hsa-let-7d-5p | [IGDCC4](http://www.ncbi.nlm.nih.gov/entrez/query.fcgi?db=gene&cmd=Retrieve&dopt=full_report&list_uids=57722) | immunoglobulin superfamily DCC subclass member 4 |
| [Details](http://mirdb.org/cgi-bin/target_detail.cgi?targetID=1231656) | 232 | 86 | hsa-let-7d-5p | [SLC25A27](http://www.ncbi.nlm.nih.gov/entrez/query.fcgi?db=gene&cmd=Retrieve&dopt=full_report&list_uids=9481) | solute carrier family 25 member 27 |
| [Details](http://mirdb.org/cgi-bin/target_detail.cgi?targetID=1232039) | 233 | 86 | hsa-let-7d-5p | [EFHD2](http://www.ncbi.nlm.nih.gov/entrez/query.fcgi?db=gene&cmd=Retrieve&dopt=full_report&list_uids=79180) | EF-hand domain family member D2 |
| [Details](http://mirdb.org/cgi-bin/target_detail.cgi?targetID=1232007) | 234 | 86 | hsa-let-7d-5p | [HIF1AN](http://www.ncbi.nlm.nih.gov/entrez/query.fcgi?db=gene&cmd=Retrieve&dopt=full_report&list_uids=55662) | hypoxia inducible factor 1 subunit alpha inhibitor |
| [Details](http://mirdb.org/cgi-bin/target_detail.cgi?targetID=1232263) | 235 | 86 | hsa-let-7d-5p | [LIN28A](http://www.ncbi.nlm.nih.gov/entrez/query.fcgi?db=gene&cmd=Retrieve&dopt=full_report&list_uids=79727) | lin-28 homolog A |
| [Details](http://mirdb.org/cgi-bin/target_detail.cgi?targetID=1232264) | 236 | 85 | hsa-let-7d-5p | [HOXA1](http://www.ncbi.nlm.nih.gov/entrez/query.fcgi?db=gene&cmd=Retrieve&dopt=full_report&list_uids=3198) | homeobox A1 |
| [Details](http://mirdb.org/cgi-bin/target_detail.cgi?targetID=1231814) | 237 | 85 | hsa-let-7d-5p | [GFM2](http://www.ncbi.nlm.nih.gov/entrez/query.fcgi?db=gene&cmd=Retrieve&dopt=full_report&list_uids=84340) | G elongation factor mitochondrial 2 |
| [Details](http://mirdb.org/cgi-bin/target_detail.cgi?targetID=1231738) | 238 | 85 | hsa-let-7d-5p | [PLEKHG6](http://www.ncbi.nlm.nih.gov/entrez/query.fcgi?db=gene&cmd=Retrieve&dopt=full_report&list_uids=55200) | pleckstrin homology and RhoGEF domain containing G6 |
| [Details](http://mirdb.org/cgi-bin/target_detail.cgi?targetID=1232115) | 239 | 85 | hsa-let-7d-5p | [COL4A3BP](http://www.ncbi.nlm.nih.gov/entrez/query.fcgi?db=gene&cmd=Retrieve&dopt=full_report&list_uids=10087) | collagen type IV alpha 3 binding protein |
| [Details](http://mirdb.org/cgi-bin/target_detail.cgi?targetID=1232129) | 240 | 85 | hsa-let-7d-5p | [ANKRA2](http://www.ncbi.nlm.nih.gov/entrez/query.fcgi?db=gene&cmd=Retrieve&dopt=full_report&list_uids=57763) | ankyrin repeat family A member 2 |
| [Details](http://mirdb.org/cgi-bin/target_detail.cgi?targetID=1231820) | 241 | 85 | hsa-let-7d-5p | [ARHGAP28](http://www.ncbi.nlm.nih.gov/entrez/query.fcgi?db=gene&cmd=Retrieve&dopt=full_report&list_uids=79822) | Rho GTPase activating protein 28 |
| [Details](http://mirdb.org/cgi-bin/target_detail.cgi?targetID=1231924) | 242 | 85 | hsa-let-7d-5p | [GPCPD1](http://www.ncbi.nlm.nih.gov/entrez/query.fcgi?db=gene&cmd=Retrieve&dopt=full_report&list_uids=56261) | glycerophosphocholine phosphodiesterase 1 |
| [Details](http://mirdb.org/cgi-bin/target_detail.cgi?targetID=1231583) | 243 | 85 | hsa-let-7d-5p | [CADM2](http://www.ncbi.nlm.nih.gov/entrez/query.fcgi?db=gene&cmd=Retrieve&dopt=full_report&list_uids=253559) | cell adhesion molecule 2 |
| [Details](http://mirdb.org/cgi-bin/target_detail.cgi?targetID=1232104) | 244 | 85 | hsa-let-7d-5p | [PLPP5](http://www.ncbi.nlm.nih.gov/entrez/query.fcgi?db=gene&cmd=Retrieve&dopt=full_report&list_uids=84513) | phospholipid phosphatase 5 |
| [Details](http://mirdb.org/cgi-bin/target_detail.cgi?targetID=1231933) | 245 | 85 | hsa-let-7d-5p | [SLC38A9](http://www.ncbi.nlm.nih.gov/entrez/query.fcgi?db=gene&cmd=Retrieve&dopt=full_report&list_uids=153129) | solute carrier family 38 member 9 |
| [Details](http://mirdb.org/cgi-bin/target_detail.cgi?targetID=1231648) | 246 | 85 | hsa-let-7d-5p | [BEND4](http://www.ncbi.nlm.nih.gov/entrez/query.fcgi?db=gene&cmd=Retrieve&dopt=full_report&list_uids=389206) | BEN domain containing 4 |
| [Details](http://mirdb.org/cgi-bin/target_detail.cgi?targetID=1232176) | 247 | 85 | hsa-let-7d-5p | [ZNF784](http://www.ncbi.nlm.nih.gov/entrez/query.fcgi?db=gene&cmd=Retrieve&dopt=full_report&list_uids=147808) | zinc finger protein 784 |
| [Details](http://mirdb.org/cgi-bin/target_detail.cgi?targetID=1231749) | 248 | 85 | hsa-let-7d-5p | [SDK1](http://www.ncbi.nlm.nih.gov/entrez/query.fcgi?db=gene&cmd=Retrieve&dopt=full_report&list_uids=221935) | sidekick cell adhesion molecule 1 |
| [Details](http://mirdb.org/cgi-bin/target_detail.cgi?targetID=1231953) | 249 | 85 | hsa-let-7d-5p | [EEF2K](http://www.ncbi.nlm.nih.gov/entrez/query.fcgi?db=gene&cmd=Retrieve&dopt=full_report&list_uids=29904) | eukaryotic elongation factor 2 kinase |
| [Details](http://mirdb.org/cgi-bin/target_detail.cgi?targetID=1232181) | 250 | 85 | hsa-let-7d-5p | [DNAJA2](http://www.ncbi.nlm.nih.gov/entrez/query.fcgi?db=gene&cmd=Retrieve&dopt=full_report&list_uids=10294) | DnaJ heat shock protein family (Hsp40) member A2 |
| [Details](http://mirdb.org/cgi-bin/target_detail.cgi?targetID=1231483) | 251 | 85 | hsa-let-7d-5p | [GJC1](http://www.ncbi.nlm.nih.gov/entrez/query.fcgi?db=gene&cmd=Retrieve&dopt=full_report&list_uids=10052) | gap junction protein gamma 1 |
| [Details](http://mirdb.org/cgi-bin/target_detail.cgi?targetID=1232167) | 252 | 85 | hsa-let-7d-5p | [DCAF15](http://www.ncbi.nlm.nih.gov/entrez/query.fcgi?db=gene&cmd=Retrieve&dopt=full_report&list_uids=90379) | DDB1 and CUL4 associated factor 15 |
| [Details](http://mirdb.org/cgi-bin/target_detail.cgi?targetID=1231665) | 253 | 84 | hsa-let-7d-5p | [PCDH19](http://www.ncbi.nlm.nih.gov/entrez/query.fcgi?db=gene&cmd=Retrieve&dopt=full_report&list_uids=57526) | protocadherin 19 |
| [Details](http://mirdb.org/cgi-bin/target_detail.cgi?targetID=1231929) | 254 | 84 | hsa-let-7d-5p | [CLDN16](http://www.ncbi.nlm.nih.gov/entrez/query.fcgi?db=gene&cmd=Retrieve&dopt=full_report&list_uids=10686) | claudin 16 |
| [Details](http://mirdb.org/cgi-bin/target_detail.cgi?targetID=1232334) | 255 | 84 | hsa-let-7d-5p | [CHD4](http://www.ncbi.nlm.nih.gov/entrez/query.fcgi?db=gene&cmd=Retrieve&dopt=full_report&list_uids=1108) | chromodomain helicase DNA binding protein 4 |
| [Details](http://mirdb.org/cgi-bin/target_detail.cgi?targetID=1232097) | 256 | 84 | hsa-let-7d-5p | [PLEKHO1](http://www.ncbi.nlm.nih.gov/entrez/query.fcgi?db=gene&cmd=Retrieve&dopt=full_report&list_uids=51177) | pleckstrin homology domain containing O1 |
| [Details](http://mirdb.org/cgi-bin/target_detail.cgi?targetID=1232144) | 257 | 84 | hsa-let-7d-5p | [MASP1](http://www.ncbi.nlm.nih.gov/entrez/query.fcgi?db=gene&cmd=Retrieve&dopt=full_report&list_uids=5648) | mannan binding lectin serine peptidase 1 |
| [Details](http://mirdb.org/cgi-bin/target_detail.cgi?targetID=1232342) | 258 | 84 | hsa-let-7d-5p | [DDX19A](http://www.ncbi.nlm.nih.gov/entrez/query.fcgi?db=gene&cmd=Retrieve&dopt=full_report&list_uids=55308) | DEAD-box helicase 19A |
| [Details](http://mirdb.org/cgi-bin/target_detail.cgi?targetID=1232216) | 259 | 84 | hsa-let-7d-5p | [RAB8B](http://www.ncbi.nlm.nih.gov/entrez/query.fcgi?db=gene&cmd=Retrieve&dopt=full_report&list_uids=51762) | RAB8B, member RAS oncogene family |
| [Details](http://mirdb.org/cgi-bin/target_detail.cgi?targetID=1231568) | 260 | 84 | hsa-let-7d-5p | [AGAP1](http://www.ncbi.nlm.nih.gov/entrez/query.fcgi?db=gene&cmd=Retrieve&dopt=full_report&list_uids=116987) | ArfGAP with GTPase domain, ankyrin repeat and PH domain 1 |
| [Details](http://mirdb.org/cgi-bin/target_detail.cgi?targetID=1232345) | 261 | 84 | hsa-let-7d-5p | [PARPBP](http://www.ncbi.nlm.nih.gov/entrez/query.fcgi?db=gene&cmd=Retrieve&dopt=full_report&list_uids=55010) | PARP1 binding protein |
| [Details](http://mirdb.org/cgi-bin/target_detail.cgi?targetID=1232045) | 262 | 84 | hsa-let-7d-5p | [GPATCH2](http://www.ncbi.nlm.nih.gov/entrez/query.fcgi?db=gene&cmd=Retrieve&dopt=full_report&list_uids=55105) | G-patch domain containing 2 |
| [Details](http://mirdb.org/cgi-bin/target_detail.cgi?targetID=1231909) | 263 | 84 | hsa-let-7d-5p | [RDX](http://www.ncbi.nlm.nih.gov/entrez/query.fcgi?db=gene&cmd=Retrieve&dopt=full_report&list_uids=5962) | radixin |
| [Details](http://mirdb.org/cgi-bin/target_detail.cgi?targetID=1232242) | 264 | 84 | hsa-let-7d-5p | [POLL](http://www.ncbi.nlm.nih.gov/entrez/query.fcgi?db=gene&cmd=Retrieve&dopt=full_report&list_uids=27343) | DNA polymerase lambda |
| [Details](http://mirdb.org/cgi-bin/target_detail.cgi?targetID=1231867) | 265 | 84 | hsa-let-7d-5p | [TSPEAR](http://www.ncbi.nlm.nih.gov/entrez/query.fcgi?db=gene&cmd=Retrieve&dopt=full_report&list_uids=54084) | thrombospondin type laminin G domain and EAR repeats |
| [Details](http://mirdb.org/cgi-bin/target_detail.cgi?targetID=1231561) | 266 | 84 | hsa-let-7d-5p | [IMPG2](http://www.ncbi.nlm.nih.gov/entrez/query.fcgi?db=gene&cmd=Retrieve&dopt=full_report&list_uids=50939) | interphotoreceptor matrix proteoglycan 2 |
| [Details](http://mirdb.org/cgi-bin/target_detail.cgi?targetID=1232089) | 267 | 84 | hsa-let-7d-5p | [PLPP6](http://www.ncbi.nlm.nih.gov/entrez/query.fcgi?db=gene&cmd=Retrieve&dopt=full_report&list_uids=403313) | phospholipid phosphatase 6 |
| [Details](http://mirdb.org/cgi-bin/target_detail.cgi?targetID=1232090) | 268 | 84 | hsa-let-7d-5p | [MAPK8](http://www.ncbi.nlm.nih.gov/entrez/query.fcgi?db=gene&cmd=Retrieve&dopt=full_report&list_uids=5599) | mitogen-activated protein kinase 8 |
| [Details](http://mirdb.org/cgi-bin/target_detail.cgi?targetID=1232125) | 269 | 84 | hsa-let-7d-5p | [UGCG](http://www.ncbi.nlm.nih.gov/entrez/query.fcgi?db=gene&cmd=Retrieve&dopt=full_report&list_uids=7357) | UDP-glucose ceramide glucosyltransferase |
| [Details](http://mirdb.org/cgi-bin/target_detail.cgi?targetID=1232299) | 270 | 84 | hsa-let-7d-5p | [FAXC](http://www.ncbi.nlm.nih.gov/entrez/query.fcgi?db=gene&cmd=Retrieve&dopt=full_report&list_uids=84553) | failed axon connections homolog |
| [Details](http://mirdb.org/cgi-bin/target_detail.cgi?targetID=1232040) | 271 | 84 | hsa-let-7d-5p | [ABT1](http://www.ncbi.nlm.nih.gov/entrez/query.fcgi?db=gene&cmd=Retrieve&dopt=full_report&list_uids=29777) | activator of basal transcription 1 |
| [Details](http://mirdb.org/cgi-bin/target_detail.cgi?targetID=1231498) | 272 | 84 | hsa-let-7d-5p | [LIPH](http://www.ncbi.nlm.nih.gov/entrez/query.fcgi?db=gene&cmd=Retrieve&dopt=full_report&list_uids=200879) | lipase H |
| [Details](http://mirdb.org/cgi-bin/target_detail.cgi?targetID=1231783) | 273 | 84 | hsa-let-7d-5p | [RALB](http://www.ncbi.nlm.nih.gov/entrez/query.fcgi?db=gene&cmd=Retrieve&dopt=full_report&list_uids=5899) | RAS like proto-oncogene B |
| [Details](http://mirdb.org/cgi-bin/target_detail.cgi?targetID=1232171) | 274 | 84 | hsa-let-7d-5p | [TAF9B](http://www.ncbi.nlm.nih.gov/entrez/query.fcgi?db=gene&cmd=Retrieve&dopt=full_report&list_uids=51616) | TATA-box binding protein associated factor 9b |
| [Details](http://mirdb.org/cgi-bin/target_detail.cgi?targetID=1232126) | 275 | 83 | hsa-let-7d-5p | [STRBP](http://www.ncbi.nlm.nih.gov/entrez/query.fcgi?db=gene&cmd=Retrieve&dopt=full_report&list_uids=55342) | spermatid perinuclear RNA binding protein |
| [Details](http://mirdb.org/cgi-bin/target_detail.cgi?targetID=1232382) | 276 | 83 | hsa-let-7d-5p | [PLAGL2](http://www.ncbi.nlm.nih.gov/entrez/query.fcgi?db=gene&cmd=Retrieve&dopt=full_report&list_uids=5326) | PLAG1 like zinc finger 2 |
| [Details](http://mirdb.org/cgi-bin/target_detail.cgi?targetID=1231877) | 277 | 83 | hsa-let-7d-5p | [STARD9](http://www.ncbi.nlm.nih.gov/entrez/query.fcgi?db=gene&cmd=Retrieve&dopt=full_report&list_uids=57519) | StAR related lipid transfer domain containing 9 |
| [Details](http://mirdb.org/cgi-bin/target_detail.cgi?targetID=1231515) | 278 | 83 | hsa-let-7d-5p | [UHRF2](http://www.ncbi.nlm.nih.gov/entrez/query.fcgi?db=gene&cmd=Retrieve&dopt=full_report&list_uids=115426) | ubiquitin like with PHD and ring finger domains 2 |
| [Details](http://mirdb.org/cgi-bin/target_detail.cgi?targetID=1231668) | 279 | 83 | hsa-let-7d-5p | [TBKBP1](http://www.ncbi.nlm.nih.gov/entrez/query.fcgi?db=gene&cmd=Retrieve&dopt=full_report&list_uids=9755) | TBK1 binding protein 1 |
| [Details](http://mirdb.org/cgi-bin/target_detail.cgi?targetID=1231487) | 280 | 83 | hsa-let-7d-5p | [POLR3D](http://www.ncbi.nlm.nih.gov/entrez/query.fcgi?db=gene&cmd=Retrieve&dopt=full_report&list_uids=661) | RNA polymerase III subunit D |
| [Details](http://mirdb.org/cgi-bin/target_detail.cgi?targetID=1231493) | 281 | 83 | hsa-let-7d-5p | [AEN](http://www.ncbi.nlm.nih.gov/entrez/query.fcgi?db=gene&cmd=Retrieve&dopt=full_report&list_uids=64782) | apoptosis enhancing nuclease |
| [Details](http://mirdb.org/cgi-bin/target_detail.cgi?targetID=1231577) | 282 | 83 | hsa-let-7d-5p | [SALL3](http://www.ncbi.nlm.nih.gov/entrez/query.fcgi?db=gene&cmd=Retrieve&dopt=full_report&list_uids=27164) | spalt like transcription factor 3 |
| [Details](http://mirdb.org/cgi-bin/target_detail.cgi?targetID=1232005) | 283 | 83 | hsa-let-7d-5p | [DVL3](http://www.ncbi.nlm.nih.gov/entrez/query.fcgi?db=gene&cmd=Retrieve&dopt=full_report&list_uids=1857) | dishevelled segment polarity protein 3 |
| [Details](http://mirdb.org/cgi-bin/target_detail.cgi?targetID=1232178) | 284 | 83 | hsa-let-7d-5p | [EDEM3](http://www.ncbi.nlm.nih.gov/entrez/query.fcgi?db=gene&cmd=Retrieve&dopt=full_report&list_uids=80267) | ER degradation enhancing alpha-mannosidase like protein 3 |
| [Details](http://mirdb.org/cgi-bin/target_detail.cgi?targetID=1231724) | 285 | 83 | hsa-let-7d-5p | [RICTOR](http://www.ncbi.nlm.nih.gov/entrez/query.fcgi?db=gene&cmd=Retrieve&dopt=full_report&list_uids=253260) | RPTOR independent companion of MTOR complex 2 |
| [Details](http://mirdb.org/cgi-bin/target_detail.cgi?targetID=1232212) | 286 | 83 | hsa-let-7d-5p | [PEG10](http://www.ncbi.nlm.nih.gov/entrez/query.fcgi?db=gene&cmd=Retrieve&dopt=full_report&list_uids=23089) | paternally expressed 10 |
| [Details](http://mirdb.org/cgi-bin/target_detail.cgi?targetID=1232187) | 287 | 83 | hsa-let-7d-5p | [C19orf47](http://www.ncbi.nlm.nih.gov/entrez/query.fcgi?db=gene&cmd=Retrieve&dopt=full_report&list_uids=126526) | chromosome 19 open reading frame 47 |
| [Details](http://mirdb.org/cgi-bin/target_detail.cgi?targetID=1231831) | 288 | 83 | hsa-let-7d-5p | [EEA1](http://www.ncbi.nlm.nih.gov/entrez/query.fcgi?db=gene&cmd=Retrieve&dopt=full_report&list_uids=8411) | early endosome antigen 1 |
| [Details](http://mirdb.org/cgi-bin/target_detail.cgi?targetID=1231706) | 289 | 83 | hsa-let-7d-5p | [GTF2I](http://www.ncbi.nlm.nih.gov/entrez/query.fcgi?db=gene&cmd=Retrieve&dopt=full_report&list_uids=2969) | general transcription factor IIi |
| [Details](http://mirdb.org/cgi-bin/target_detail.cgi?targetID=1231895) | 290 | 83 | hsa-let-7d-5p | [KCNJ11](http://www.ncbi.nlm.nih.gov/entrez/query.fcgi?db=gene&cmd=Retrieve&dopt=full_report&list_uids=3767) | potassium voltage-gated channel subfamily J member 11 |
| [Details](http://mirdb.org/cgi-bin/target_detail.cgi?targetID=1232004) | 291 | 83 | hsa-let-7d-5p | [LEPROTL1](http://www.ncbi.nlm.nih.gov/entrez/query.fcgi?db=gene&cmd=Retrieve&dopt=full_report&list_uids=23484) | leptin receptor overlapping transcript like 1 |
| [Details](http://mirdb.org/cgi-bin/target_detail.cgi?targetID=1231734) | 292 | 83 | hsa-let-7d-5p | [CDKN1A](http://www.ncbi.nlm.nih.gov/entrez/query.fcgi?db=gene&cmd=Retrieve&dopt=full_report&list_uids=1026) | cyclin dependent kinase inhibitor 1A |
| [Details](http://mirdb.org/cgi-bin/target_detail.cgi?targetID=1232194) | 293 | 83 | hsa-let-7d-5p | [AMOT](http://www.ncbi.nlm.nih.gov/entrez/query.fcgi?db=gene&cmd=Retrieve&dopt=full_report&list_uids=154796) | angiomotin |
| [Details](http://mirdb.org/cgi-bin/target_detail.cgi?targetID=1232271) | 294 | 83 | hsa-let-7d-5p | [APBB3](http://www.ncbi.nlm.nih.gov/entrez/query.fcgi?db=gene&cmd=Retrieve&dopt=full_report&list_uids=10307) | amyloid beta precursor protein binding family B member 3 |
| [Details](http://mirdb.org/cgi-bin/target_detail.cgi?targetID=1232021) | 295 | 83 | hsa-let-7d-5p | [SNX16](http://www.ncbi.nlm.nih.gov/entrez/query.fcgi?db=gene&cmd=Retrieve&dopt=full_report&list_uids=64089) | sorting nexin 16 |
| [Details](http://mirdb.org/cgi-bin/target_detail.cgi?targetID=1232075) | 296 | 83 | hsa-let-7d-5p | [ACER2](http://www.ncbi.nlm.nih.gov/entrez/query.fcgi?db=gene&cmd=Retrieve&dopt=full_report&list_uids=340485) | alkaline ceramidase 2 |
| [Details](http://mirdb.org/cgi-bin/target_detail.cgi?targetID=1231921) | 297 | 83 | hsa-let-7d-5p | [SLC2A12](http://www.ncbi.nlm.nih.gov/entrez/query.fcgi?db=gene&cmd=Retrieve&dopt=full_report&list_uids=154091) | solute carrier family 2 member 12 |
| [Details](http://mirdb.org/cgi-bin/target_detail.cgi?targetID=1231821) | 298 | 83 | hsa-let-7d-5p | [E2F2](http://www.ncbi.nlm.nih.gov/entrez/query.fcgi?db=gene&cmd=Retrieve&dopt=full_report&list_uids=1870) | E2F transcription factor 2 |
| [Details](http://mirdb.org/cgi-bin/target_detail.cgi?targetID=1231530) | 299 | 83 | hsa-let-7d-5p | [KLF8](http://www.ncbi.nlm.nih.gov/entrez/query.fcgi?db=gene&cmd=Retrieve&dopt=full_report&list_uids=11279) | Kruppel like factor 8 |
| [Details](http://mirdb.org/cgi-bin/target_detail.cgi?targetID=1232094) | 300 | 83 | hsa-let-7d-5p | [ABCB9](http://www.ncbi.nlm.nih.gov/entrez/query.fcgi?db=gene&cmd=Retrieve&dopt=full_report&list_uids=23457) | ATP binding cassette subfamily B member 9 |
| [Details](http://mirdb.org/cgi-bin/target_detail.cgi?targetID=1231830) | 301 | 82 | hsa-let-7d-5p | [TNFSF9](http://www.ncbi.nlm.nih.gov/entrez/query.fcgi?db=gene&cmd=Retrieve&dopt=full_report&list_uids=8744) | TNF superfamily member 9 |
| [Details](http://mirdb.org/cgi-bin/target_detail.cgi?targetID=1231943) | 302 | 82 | hsa-let-7d-5p | [SEMA4C](http://www.ncbi.nlm.nih.gov/entrez/query.fcgi?db=gene&cmd=Retrieve&dopt=full_report&list_uids=54910) | semaphorin 4C |
| [Details](http://mirdb.org/cgi-bin/target_detail.cgi?targetID=1231562) | 303 | 82 | hsa-let-7d-5p | [STXBP5](http://www.ncbi.nlm.nih.gov/entrez/query.fcgi?db=gene&cmd=Retrieve&dopt=full_report&list_uids=134957) | syntaxin binding protein 5 |
| [Details](http://mirdb.org/cgi-bin/target_detail.cgi?targetID=1232110) | 304 | 82 | hsa-let-7d-5p | [NYNRIN](http://www.ncbi.nlm.nih.gov/entrez/query.fcgi?db=gene&cmd=Retrieve&dopt=full_report&list_uids=57523) | NYN domain and retroviral integrase containing |
| [Details](http://mirdb.org/cgi-bin/target_detail.cgi?targetID=1232088) | 305 | 82 | hsa-let-7d-5p | [B4GAT1](http://www.ncbi.nlm.nih.gov/entrez/query.fcgi?db=gene&cmd=Retrieve&dopt=full_report&list_uids=11041) | beta-1,4-glucuronyltransferase 1 |
| [Details](http://mirdb.org/cgi-bin/target_detail.cgi?targetID=1231646) | 306 | 82 | hsa-let-7d-5p | [ATL2](http://www.ncbi.nlm.nih.gov/entrez/query.fcgi?db=gene&cmd=Retrieve&dopt=full_report&list_uids=64225) | atlastin GTPase 2 |
| [Details](http://mirdb.org/cgi-bin/target_detail.cgi?targetID=1231994) | 307 | 82 | hsa-let-7d-5p | [MBD2](http://www.ncbi.nlm.nih.gov/entrez/query.fcgi?db=gene&cmd=Retrieve&dopt=full_report&list_uids=8932) | methyl-CpG binding domain protein 2 |
| [Details](http://mirdb.org/cgi-bin/target_detail.cgi?targetID=1231792) | 308 | 82 | hsa-let-7d-5p | [NME4](http://www.ncbi.nlm.nih.gov/entrez/query.fcgi?db=gene&cmd=Retrieve&dopt=full_report&list_uids=4833) | NME/NM23 nucleoside diphosphate kinase 4 |
| [Details](http://mirdb.org/cgi-bin/target_detail.cgi?targetID=1231829) | 309 | 82 | hsa-let-7d-5p | [TMEM121B](http://www.ncbi.nlm.nih.gov/entrez/query.fcgi?db=gene&cmd=Retrieve&dopt=full_report&list_uids=27439) | transmembrane protein 121B |
| [Details](http://mirdb.org/cgi-bin/target_detail.cgi?targetID=1231919) | 310 | 82 | hsa-let-7d-5p | [LINGO1](http://www.ncbi.nlm.nih.gov/entrez/query.fcgi?db=gene&cmd=Retrieve&dopt=full_report&list_uids=84894) | leucine rich repeat and Ig domain containing 1 |
| [Details](http://mirdb.org/cgi-bin/target_detail.cgi?targetID=1232074) | 311 | 82 | hsa-let-7d-5p | [CCL7](http://www.ncbi.nlm.nih.gov/entrez/query.fcgi?db=gene&cmd=Retrieve&dopt=full_report&list_uids=6354) | C-C motif chemokine ligand 7 |
| [Details](http://mirdb.org/cgi-bin/target_detail.cgi?targetID=1232155) | 312 | 82 | hsa-let-7d-5p | [C15orf39](http://www.ncbi.nlm.nih.gov/entrez/query.fcgi?db=gene&cmd=Retrieve&dopt=full_report&list_uids=56905) | chromosome 15 open reading frame 39 |
| [Details](http://mirdb.org/cgi-bin/target_detail.cgi?targetID=1231619) | 313 | 82 | hsa-let-7d-5p | [HOXD1](http://www.ncbi.nlm.nih.gov/entrez/query.fcgi?db=gene&cmd=Retrieve&dopt=full_report&list_uids=3231) | homeobox D1 |
| [Details](http://mirdb.org/cgi-bin/target_detail.cgi?targetID=1232158) | 314 | 82 | hsa-let-7d-5p | [DIP2A](http://www.ncbi.nlm.nih.gov/entrez/query.fcgi?db=gene&cmd=Retrieve&dopt=full_report&list_uids=23181) | disco interacting protein 2 homolog A |
| [Details](http://mirdb.org/cgi-bin/target_detail.cgi?targetID=1231813) | 315 | 82 | hsa-let-7d-5p | [IGF2BP2](http://www.ncbi.nlm.nih.gov/entrez/query.fcgi?db=gene&cmd=Retrieve&dopt=full_report&list_uids=10644) | insulin like growth factor 2 mRNA binding protein 2 |
| [Details](http://mirdb.org/cgi-bin/target_detail.cgi?targetID=1232029) | 316 | 82 | hsa-let-7d-5p | [NEK3](http://www.ncbi.nlm.nih.gov/entrez/query.fcgi?db=gene&cmd=Retrieve&dopt=full_report&list_uids=4752) | NIMA related kinase 3 |
| [Details](http://mirdb.org/cgi-bin/target_detail.cgi?targetID=1231878) | 317 | 82 | hsa-let-7d-5p | [KLF9](http://www.ncbi.nlm.nih.gov/entrez/query.fcgi?db=gene&cmd=Retrieve&dopt=full_report&list_uids=687) | Kruppel like factor 9 |
| [Details](http://mirdb.org/cgi-bin/target_detail.cgi?targetID=1231510) | 318 | 82 | hsa-let-7d-5p | [SENP5](http://www.ncbi.nlm.nih.gov/entrez/query.fcgi?db=gene&cmd=Retrieve&dopt=full_report&list_uids=205564) | SUMO specific peptidase 5 |
| [Details](http://mirdb.org/cgi-bin/target_detail.cgi?targetID=1232280) | 319 | 82 | hsa-let-7d-5p | [MMS22L](http://www.ncbi.nlm.nih.gov/entrez/query.fcgi?db=gene&cmd=Retrieve&dopt=full_report&list_uids=253714) | MMS22 like, DNA repair protein |
| [Details](http://mirdb.org/cgi-bin/target_detail.cgi?targetID=1232050) | 320 | 82 | hsa-let-7d-5p | [MTDH](http://www.ncbi.nlm.nih.gov/entrez/query.fcgi?db=gene&cmd=Retrieve&dopt=full_report&list_uids=92140) | metadherin |
| [Details](http://mirdb.org/cgi-bin/target_detail.cgi?targetID=1231501) | 321 | 82 | hsa-let-7d-5p | [GALNT1](http://www.ncbi.nlm.nih.gov/entrez/query.fcgi?db=gene&cmd=Retrieve&dopt=full_report&list_uids=2589) | polypeptide N-acetylgalactosaminyltransferase 1 |
| [Details](http://mirdb.org/cgi-bin/target_detail.cgi?targetID=1232283) | 322 | 82 | hsa-let-7d-5p | [RFX6](http://www.ncbi.nlm.nih.gov/entrez/query.fcgi?db=gene&cmd=Retrieve&dopt=full_report&list_uids=222546) | regulatory factor X6 |
| [Details](http://mirdb.org/cgi-bin/target_detail.cgi?targetID=1232041) | 323 | 82 | hsa-let-7d-5p | [TMC7](http://www.ncbi.nlm.nih.gov/entrez/query.fcgi?db=gene&cmd=Retrieve&dopt=full_report&list_uids=79905) | transmembrane channel like 7 |
| [Details](http://mirdb.org/cgi-bin/target_detail.cgi?targetID=1232149) | 324 | 82 | hsa-let-7d-5p | [KDM3A](http://www.ncbi.nlm.nih.gov/entrez/query.fcgi?db=gene&cmd=Retrieve&dopt=full_report&list_uids=55818) | lysine demethylase 3A |
| [Details](http://mirdb.org/cgi-bin/target_detail.cgi?targetID=1232095) | 325 | 81 | hsa-let-7d-5p | [ATP2A2](http://www.ncbi.nlm.nih.gov/entrez/query.fcgi?db=gene&cmd=Retrieve&dopt=full_report&list_uids=488) | ATPase sarcoplasmic/endoplasmic reticulum Ca2+ transporting 2 |
| [Details](http://mirdb.org/cgi-bin/target_detail.cgi?targetID=1231817) | 326 | 81 | hsa-let-7d-5p | [FNIP2](http://www.ncbi.nlm.nih.gov/entrez/query.fcgi?db=gene&cmd=Retrieve&dopt=full_report&list_uids=57600) | folliculin interacting protein 2 |
| [Details](http://mirdb.org/cgi-bin/target_detail.cgi?targetID=1232081) | 327 | 81 | hsa-let-7d-5p | [GYG2](http://www.ncbi.nlm.nih.gov/entrez/query.fcgi?db=gene&cmd=Retrieve&dopt=full_report&list_uids=8908) | glycogenin 2 |
| [Details](http://mirdb.org/cgi-bin/target_detail.cgi?targetID=1231704) | 328 | 81 | hsa-let-7d-5p | [PDE12](http://www.ncbi.nlm.nih.gov/entrez/query.fcgi?db=gene&cmd=Retrieve&dopt=full_report&list_uids=201626) | phosphodiesterase 12 |
| [Details](http://mirdb.org/cgi-bin/target_detail.cgi?targetID=1232268) | 329 | 81 | hsa-let-7d-5p | [PIGA](http://www.ncbi.nlm.nih.gov/entrez/query.fcgi?db=gene&cmd=Retrieve&dopt=full_report&list_uids=5277) | phosphatidylinositol glycan anchor biosynthesis class A |
| [Details](http://mirdb.org/cgi-bin/target_detail.cgi?targetID=1231760) | 330 | 81 | hsa-let-7d-5p | [POGZ](http://www.ncbi.nlm.nih.gov/entrez/query.fcgi?db=gene&cmd=Retrieve&dopt=full_report&list_uids=23126) | pogo transposable element derived with ZNF domain |
| [Details](http://mirdb.org/cgi-bin/target_detail.cgi?targetID=1231520) | 331 | 81 | hsa-let-7d-5p | [DUSP1](http://www.ncbi.nlm.nih.gov/entrez/query.fcgi?db=gene&cmd=Retrieve&dopt=full_report&list_uids=1843) | dual specificity phosphatase 1 |
| [Details](http://mirdb.org/cgi-bin/target_detail.cgi?targetID=1231558) | 332 | 81 | hsa-let-7d-5p | [VIRMA](http://www.ncbi.nlm.nih.gov/entrez/query.fcgi?db=gene&cmd=Retrieve&dopt=full_report&list_uids=25962) | vir like m6A methyltransferase associated |
| [Details](http://mirdb.org/cgi-bin/target_detail.cgi?targetID=1231607) | 333 | 81 | hsa-let-7d-5p | [KLHL31](http://www.ncbi.nlm.nih.gov/entrez/query.fcgi?db=gene&cmd=Retrieve&dopt=full_report&list_uids=401265) | kelch like family member 31 |
| [Details](http://mirdb.org/cgi-bin/target_detail.cgi?targetID=1231726) | 334 | 81 | hsa-let-7d-5p | [CRTAM](http://www.ncbi.nlm.nih.gov/entrez/query.fcgi?db=gene&cmd=Retrieve&dopt=full_report&list_uids=56253) | cytotoxic and regulatory T cell molecule |
| [Details](http://mirdb.org/cgi-bin/target_detail.cgi?targetID=1231719) | 335 | 81 | hsa-let-7d-5p | [XK](http://www.ncbi.nlm.nih.gov/entrez/query.fcgi?db=gene&cmd=Retrieve&dopt=full_report&list_uids=7504) | X-linked Kx blood group |
| [Details](http://mirdb.org/cgi-bin/target_detail.cgi?targetID=1232292) | 336 | 81 | hsa-let-7d-5p | [CDC25A](http://www.ncbi.nlm.nih.gov/entrez/query.fcgi?db=gene&cmd=Retrieve&dopt=full_report&list_uids=993) | cell division cycle 25A |
| [Details](http://mirdb.org/cgi-bin/target_detail.cgi?targetID=1231623) | 337 | 81 | hsa-let-7d-5p | [ABL2](http://www.ncbi.nlm.nih.gov/entrez/query.fcgi?db=gene&cmd=Retrieve&dopt=full_report&list_uids=27) | ABL proto-oncogene 2, non-receptor tyrosine kinase |
| [Details](http://mirdb.org/cgi-bin/target_detail.cgi?targetID=1231835) | 338 | 81 | hsa-let-7d-5p | [IRS2](http://www.ncbi.nlm.nih.gov/entrez/query.fcgi?db=gene&cmd=Retrieve&dopt=full_report&list_uids=8660) | insulin receptor substrate 2 |
| [Details](http://mirdb.org/cgi-bin/target_detail.cgi?targetID=1232196) | 339 | 81 | hsa-let-7d-5p | [FASLG](http://www.ncbi.nlm.nih.gov/entrez/query.fcgi?db=gene&cmd=Retrieve&dopt=full_report&list_uids=356) | Fas ligand |
| [Details](http://mirdb.org/cgi-bin/target_detail.cgi?targetID=1231794) | 340 | 81 | hsa-let-7d-5p | [NGF](http://www.ncbi.nlm.nih.gov/entrez/query.fcgi?db=gene&cmd=Retrieve&dopt=full_report&list_uids=4803) | nerve growth factor |
| [Details](http://mirdb.org/cgi-bin/target_detail.cgi?targetID=1232192) | 341 | 81 | hsa-let-7d-5p | [AKAP6](http://www.ncbi.nlm.nih.gov/entrez/query.fcgi?db=gene&cmd=Retrieve&dopt=full_report&list_uids=9472) | A-kinase anchoring protein 6 |
| [Details](http://mirdb.org/cgi-bin/target_detail.cgi?targetID=1232305) | 342 | 80 | hsa-let-7d-5p | [SESTD1](http://www.ncbi.nlm.nih.gov/entrez/query.fcgi?db=gene&cmd=Retrieve&dopt=full_report&list_uids=91404) | SEC14 and spectrin domain containing 1 |
| [Details](http://mirdb.org/cgi-bin/target_detail.cgi?targetID=1231611) | 343 | 80 | hsa-let-7d-5p | [HTR1E](http://www.ncbi.nlm.nih.gov/entrez/query.fcgi?db=gene&cmd=Retrieve&dopt=full_report&list_uids=3354) | 5-hydroxytryptamine receptor 1E |
| [Details](http://mirdb.org/cgi-bin/target_detail.cgi?targetID=1232209) | 344 | 80 | hsa-let-7d-5p | [TRIM41](http://www.ncbi.nlm.nih.gov/entrez/query.fcgi?db=gene&cmd=Retrieve&dopt=full_report&list_uids=90933) | tripartite motif containing 41 |
| [Details](http://mirdb.org/cgi-bin/target_detail.cgi?targetID=1231847) | 345 | 80 | hsa-let-7d-5p | [TXLNA](http://www.ncbi.nlm.nih.gov/entrez/query.fcgi?db=gene&cmd=Retrieve&dopt=full_report&list_uids=200081) | taxilin alpha |
| [Details](http://mirdb.org/cgi-bin/target_detail.cgi?targetID=1232363) | 346 | 80 | hsa-let-7d-5p | [PRRX1](http://www.ncbi.nlm.nih.gov/entrez/query.fcgi?db=gene&cmd=Retrieve&dopt=full_report&list_uids=5396) | paired related homeobox 1 |
| [Details](http://mirdb.org/cgi-bin/target_detail.cgi?targetID=1232130) | 347 | 80 | hsa-let-7d-5p | [SIGLEC14](http://www.ncbi.nlm.nih.gov/entrez/query.fcgi?db=gene&cmd=Retrieve&dopt=full_report&list_uids=100049587) | sialic acid binding Ig like lectin 14 |
| [Details](http://mirdb.org/cgi-bin/target_detail.cgi?targetID=1232321) | 348 | 80 | hsa-let-7d-5p | [ABCC5](http://www.ncbi.nlm.nih.gov/entrez/query.fcgi?db=gene&cmd=Retrieve&dopt=full_report&list_uids=10057) | ATP binding cassette subfamily C member 5 |
| [Details](http://mirdb.org/cgi-bin/target_detail.cgi?targetID=1231780) | 349 | 80 | hsa-let-7d-5p | [HOOK1](http://www.ncbi.nlm.nih.gov/entrez/query.fcgi?db=gene&cmd=Retrieve&dopt=full_report&list_uids=51361) | hook microtubule tethering protein 1 |
| [Details](http://mirdb.org/cgi-bin/target_detail.cgi?targetID=1231630) | 350 | 80 | hsa-let-7d-5p | [EIF4G2](http://www.ncbi.nlm.nih.gov/entrez/query.fcgi?db=gene&cmd=Retrieve&dopt=full_report&list_uids=1982) | eukaryotic translation initiation factor 4 gamma 2 |
| [Details](http://mirdb.org/cgi-bin/target_detail.cgi?targetID=1232031) | 351 | 80 | hsa-let-7d-5p | [RSPO2](http://www.ncbi.nlm.nih.gov/entrez/query.fcgi?db=gene&cmd=Retrieve&dopt=full_report&list_uids=340419) | R-spondin 2 |
| [Details](http://mirdb.org/cgi-bin/target_detail.cgi?targetID=1231594) | 352 | 80 | hsa-let-7d-5p | [ARMT1](http://www.ncbi.nlm.nih.gov/entrez/query.fcgi?db=gene&cmd=Retrieve&dopt=full_report&list_uids=79624) | acidic residue methyltransferase 1 |
| [Details](http://mirdb.org/cgi-bin/target_detail.cgi?targetID=1232374) | 353 | 80 | hsa-let-7d-5p | [RNF165](http://www.ncbi.nlm.nih.gov/entrez/query.fcgi?db=gene&cmd=Retrieve&dopt=full_report&list_uids=494470) | ring finger protein 165 |
| [Details](http://mirdb.org/cgi-bin/target_detail.cgi?targetID=1231500) | 354 | 80 | hsa-let-7d-5p | [SUB1](http://www.ncbi.nlm.nih.gov/entrez/query.fcgi?db=gene&cmd=Retrieve&dopt=full_report&list_uids=10923) | SUB1 homolog, transcriptional regulator |
| [Details](http://mirdb.org/cgi-bin/target_detail.cgi?targetID=1231793) | 355 | 80 | hsa-let-7d-5p | [GABBR2](http://www.ncbi.nlm.nih.gov/entrez/query.fcgi?db=gene&cmd=Retrieve&dopt=full_report&list_uids=9568) | gamma-aminobutyric acid type B receptor subunit 2 |
| [Details](http://mirdb.org/cgi-bin/target_detail.cgi?targetID=1231974) | 356 | 80 | hsa-let-7d-5p | [ARL5A](http://www.ncbi.nlm.nih.gov/entrez/query.fcgi?db=gene&cmd=Retrieve&dopt=full_report&list_uids=26225) | ADP ribosylation factor like GTPase 5A |
| [Details](http://mirdb.org/cgi-bin/target_detail.cgi?targetID=1232067) | 357 | 80 | hsa-let-7d-5p | [SNX30](http://www.ncbi.nlm.nih.gov/entrez/query.fcgi?db=gene&cmd=Retrieve&dopt=full_report&list_uids=401548) | sorting nexin family member 30 |
| [Details](http://mirdb.org/cgi-bin/target_detail.cgi?targetID=1231538) | 358 | 80 | hsa-let-7d-5p | [TRANK1](http://www.ncbi.nlm.nih.gov/entrez/query.fcgi?db=gene&cmd=Retrieve&dopt=full_report&list_uids=9881) | tetratricopeptide repeat and ankyrin repeat containing 1 |
| [Details](http://mirdb.org/cgi-bin/target_detail.cgi?targetID=1232082) | 359 | 79 | hsa-let-7d-5p | [GRPEL2](http://www.ncbi.nlm.nih.gov/entrez/query.fcgi?db=gene&cmd=Retrieve&dopt=full_report&list_uids=134266) | GrpE like 2, mitochondrial |
| [Details](http://mirdb.org/cgi-bin/target_detail.cgi?targetID=1231873) | 360 | 79 | hsa-let-7d-5p | [RANBP2](http://www.ncbi.nlm.nih.gov/entrez/query.fcgi?db=gene&cmd=Retrieve&dopt=full_report&list_uids=5903) | RAN binding protein 2 |
| [Details](http://mirdb.org/cgi-bin/target_detail.cgi?targetID=1231850) | 361 | 79 | hsa-let-7d-5p | [TECPR2](http://www.ncbi.nlm.nih.gov/entrez/query.fcgi?db=gene&cmd=Retrieve&dopt=full_report&list_uids=9895) | tectonin beta-propeller repeat containing 2 |
| [Details](http://mirdb.org/cgi-bin/target_detail.cgi?targetID=1232269) | 362 | 79 | hsa-let-7d-5p | [DPH3](http://www.ncbi.nlm.nih.gov/entrez/query.fcgi?db=gene&cmd=Retrieve&dopt=full_report&list_uids=285381) | diphthamide biosynthesis 3 |
| [Details](http://mirdb.org/cgi-bin/target_detail.cgi?targetID=1232052) | 363 | 79 | hsa-let-7d-5p | [GOLT1B](http://www.ncbi.nlm.nih.gov/entrez/query.fcgi?db=gene&cmd=Retrieve&dopt=full_report&list_uids=51026) | golgi transport 1B |
| [Details](http://mirdb.org/cgi-bin/target_detail.cgi?targetID=1232273) | 364 | 79 | hsa-let-7d-5p | [FAM214B](http://www.ncbi.nlm.nih.gov/entrez/query.fcgi?db=gene&cmd=Retrieve&dopt=full_report&list_uids=80256) | family with sequence similarity 214 member B |
| [Details](http://mirdb.org/cgi-bin/target_detail.cgi?targetID=1231856) | 365 | 79 | hsa-let-7d-5p | [KIAA1958](http://www.ncbi.nlm.nih.gov/entrez/query.fcgi?db=gene&cmd=Retrieve&dopt=full_report&list_uids=158405) | KIAA1958 |
| [Details](http://mirdb.org/cgi-bin/target_detail.cgi?targetID=1231682) | 366 | 79 | hsa-let-7d-5p | [PGM2L1](http://www.ncbi.nlm.nih.gov/entrez/query.fcgi?db=gene&cmd=Retrieve&dopt=full_report&list_uids=283209) | phosphoglucomutase 2 like 1 |
| [Details](http://mirdb.org/cgi-bin/target_detail.cgi?targetID=1231615) | 367 | 79 | hsa-let-7d-5p | [ARID3A](http://www.ncbi.nlm.nih.gov/entrez/query.fcgi?db=gene&cmd=Retrieve&dopt=full_report&list_uids=1820) | AT-rich interaction domain 3A |
| [Details](http://mirdb.org/cgi-bin/target_detail.cgi?targetID=1231590) | 368 | 79 | hsa-let-7d-5p | [MEF2C](http://www.ncbi.nlm.nih.gov/entrez/query.fcgi?db=gene&cmd=Retrieve&dopt=full_report&list_uids=4208) | myocyte enhancer factor 2C |
| [Details](http://mirdb.org/cgi-bin/target_detail.cgi?targetID=1231753) | 369 | 79 | hsa-let-7d-5p | [PITPNM3](http://www.ncbi.nlm.nih.gov/entrez/query.fcgi?db=gene&cmd=Retrieve&dopt=full_report&list_uids=83394) | PITPNM family member 3 |
| [Details](http://mirdb.org/cgi-bin/target_detail.cgi?targetID=1232359) | 370 | 79 | hsa-let-7d-5p | [FGD6](http://www.ncbi.nlm.nih.gov/entrez/query.fcgi?db=gene&cmd=Retrieve&dopt=full_report&list_uids=55785) | FYVE, RhoGEF and PH domain containing 6 |
| [Details](http://mirdb.org/cgi-bin/target_detail.cgi?targetID=1232191) | 371 | 79 | hsa-let-7d-5p | [CLASP2](http://www.ncbi.nlm.nih.gov/entrez/query.fcgi?db=gene&cmd=Retrieve&dopt=full_report&list_uids=23122) | cytoplasmic linker associated protein 2 |
| [Details](http://mirdb.org/cgi-bin/target_detail.cgi?targetID=1232333) | 372 | 79 | hsa-let-7d-5p | [DUSP22](http://www.ncbi.nlm.nih.gov/entrez/query.fcgi?db=gene&cmd=Retrieve&dopt=full_report&list_uids=56940) | dual specificity phosphatase 22 |
| [Details](http://mirdb.org/cgi-bin/target_detail.cgi?targetID=1231540) | 373 | 79 | hsa-let-7d-5p | [RAB3GAP2](http://www.ncbi.nlm.nih.gov/entrez/query.fcgi?db=gene&cmd=Retrieve&dopt=full_report&list_uids=25782) | RAB3 GTPase activating non-catalytic protein subunit 2 |
| [Details](http://mirdb.org/cgi-bin/target_detail.cgi?targetID=1231991) | 374 | 79 | hsa-let-7d-5p | [HOXA9](http://www.ncbi.nlm.nih.gov/entrez/query.fcgi?db=gene&cmd=Retrieve&dopt=full_report&list_uids=3205) | homeobox A9 |
| [Details](http://mirdb.org/cgi-bin/target_detail.cgi?targetID=1232157) | 375 | 79 | hsa-let-7d-5p | [CCNT2](http://www.ncbi.nlm.nih.gov/entrez/query.fcgi?db=gene&cmd=Retrieve&dopt=full_report&list_uids=905) | cyclin T2 |
| [Details](http://mirdb.org/cgi-bin/target_detail.cgi?targetID=1232376) | 376 | 79 | hsa-let-7d-5p | [ACSL6](http://www.ncbi.nlm.nih.gov/entrez/query.fcgi?db=gene&cmd=Retrieve&dopt=full_report&list_uids=23305) | acyl-CoA synthetase long chain family member 6 |
| [Details](http://mirdb.org/cgi-bin/target_detail.cgi?targetID=1232168) | 377 | 79 | hsa-let-7d-5p | [CACNB4](http://www.ncbi.nlm.nih.gov/entrez/query.fcgi?db=gene&cmd=Retrieve&dopt=full_report&list_uids=785) | calcium voltage-gated channel auxiliary subunit beta 4 |
| [Details](http://mirdb.org/cgi-bin/target_detail.cgi?targetID=1231939) | 378 | 79 | hsa-let-7d-5p | [CRCT1](http://www.ncbi.nlm.nih.gov/entrez/query.fcgi?db=gene&cmd=Retrieve&dopt=full_report&list_uids=54544) | cysteine rich C-terminal 1 |
| [Details](http://mirdb.org/cgi-bin/target_detail.cgi?targetID=1231954) | 379 | 79 | hsa-let-7d-5p | [INTS6L](http://www.ncbi.nlm.nih.gov/entrez/query.fcgi?db=gene&cmd=Retrieve&dopt=full_report&list_uids=203522) | integrator complex subunit 6 like |
| [Details](http://mirdb.org/cgi-bin/target_detail.cgi?targetID=1231563) | 380 | 79 | hsa-let-7d-5p | [SCD](http://www.ncbi.nlm.nih.gov/entrez/query.fcgi?db=gene&cmd=Retrieve&dopt=full_report&list_uids=6319) | stearoyl-CoA desaturase |
| [Details](http://mirdb.org/cgi-bin/target_detail.cgi?targetID=1232175) | 381 | 79 | hsa-let-7d-5p | [STARD3NL](http://www.ncbi.nlm.nih.gov/entrez/query.fcgi?db=gene&cmd=Retrieve&dopt=full_report&list_uids=83930) | STARD3 N-terminal like |
| [Details](http://mirdb.org/cgi-bin/target_detail.cgi?targetID=1232193) | 382 | 79 | hsa-let-7d-5p | [PRLR](http://www.ncbi.nlm.nih.gov/entrez/query.fcgi?db=gene&cmd=Retrieve&dopt=full_report&list_uids=5618) | prolactin receptor |
| [Details](http://mirdb.org/cgi-bin/target_detail.cgi?targetID=1232381) | 383 | 79 | hsa-let-7d-5p | [SLC25A24](http://www.ncbi.nlm.nih.gov/entrez/query.fcgi?db=gene&cmd=Retrieve&dopt=full_report&list_uids=29957) | solute carrier family 25 member 24 |
| [Details](http://mirdb.org/cgi-bin/target_detail.cgi?targetID=1232182) | 384 | 79 | hsa-let-7d-5p | [PARD6B](http://www.ncbi.nlm.nih.gov/entrez/query.fcgi?db=gene&cmd=Retrieve&dopt=full_report&list_uids=84612) | par-6 family cell polarity regulator beta |
| [Details](http://mirdb.org/cgi-bin/target_detail.cgi?targetID=1232251) | 385 | 78 | hsa-let-7d-5p | [USP32](http://www.ncbi.nlm.nih.gov/entrez/query.fcgi?db=gene&cmd=Retrieve&dopt=full_report&list_uids=84669) | ubiquitin specific peptidase 32 |
| [Details](http://mirdb.org/cgi-bin/target_detail.cgi?targetID=1232259) | 386 | 78 | hsa-let-7d-5p | [SMARCC1](http://www.ncbi.nlm.nih.gov/entrez/query.fcgi?db=gene&cmd=Retrieve&dopt=full_report&list_uids=6599) | SWI/SNF related, matrix associated, actin dependent regulator of chromatin subfamily c member 1 |
| [Details](http://mirdb.org/cgi-bin/target_detail.cgi?targetID=1231479) | 387 | 78 | hsa-let-7d-5p | [ZNF587](http://www.ncbi.nlm.nih.gov/entrez/query.fcgi?db=gene&cmd=Retrieve&dopt=full_report&list_uids=84914) | zinc finger protein 587 |
| [Details](http://mirdb.org/cgi-bin/target_detail.cgi?targetID=1232224) | 388 | 78 | hsa-let-7d-5p | [BSN](http://www.ncbi.nlm.nih.gov/entrez/query.fcgi?db=gene&cmd=Retrieve&dopt=full_report&list_uids=8927) | bassoon presynaptic cytomatrix protein |
| [Details](http://mirdb.org/cgi-bin/target_detail.cgi?targetID=1231513) | 389 | 78 | hsa-let-7d-5p | [PDP2](http://www.ncbi.nlm.nih.gov/entrez/query.fcgi?db=gene&cmd=Retrieve&dopt=full_report&list_uids=57546) | pyruvate dehyrogenase phosphatase catalytic subunit 2 |
| [Details](http://mirdb.org/cgi-bin/target_detail.cgi?targetID=1231571) | 390 | 78 | hsa-let-7d-5p | [ACVR2A](http://www.ncbi.nlm.nih.gov/entrez/query.fcgi?db=gene&cmd=Retrieve&dopt=full_report&list_uids=92) | activin A receptor type 2A |
| [Details](http://mirdb.org/cgi-bin/target_detail.cgi?targetID=1231907) | 391 | 78 | hsa-let-7d-5p | [LRFN4](http://www.ncbi.nlm.nih.gov/entrez/query.fcgi?db=gene&cmd=Retrieve&dopt=full_report&list_uids=78999) | leucine rich repeat and fibronectin type III domain containing 4 |
| [Details](http://mirdb.org/cgi-bin/target_detail.cgi?targetID=1231914) | 392 | 78 | hsa-let-7d-5p | [ARG2](http://www.ncbi.nlm.nih.gov/entrez/query.fcgi?db=gene&cmd=Retrieve&dopt=full_report&list_uids=384) | arginase 2 |
| [Details](http://mirdb.org/cgi-bin/target_detail.cgi?targetID=1232020) | 393 | 78 | hsa-let-7d-5p | [IGF1](http://www.ncbi.nlm.nih.gov/entrez/query.fcgi?db=gene&cmd=Retrieve&dopt=full_report&list_uids=3479) | insulin like growth factor 1 |
| [Details](http://mirdb.org/cgi-bin/target_detail.cgi?targetID=1232316) | 394 | 78 | hsa-let-7d-5p | [HMGA1](http://www.ncbi.nlm.nih.gov/entrez/query.fcgi?db=gene&cmd=Retrieve&dopt=full_report&list_uids=3159) | high mobility group AT-hook 1 |
| [Details](http://mirdb.org/cgi-bin/target_detail.cgi?targetID=1232330) | 395 | 78 | hsa-let-7d-5p | [SYNCRIP](http://www.ncbi.nlm.nih.gov/entrez/query.fcgi?db=gene&cmd=Retrieve&dopt=full_report&list_uids=10492) | synaptotagmin binding cytoplasmic RNA interacting protein |
| [Details](http://mirdb.org/cgi-bin/target_detail.cgi?targetID=1232301) | 396 | 78 | hsa-let-7d-5p | [DDTL](http://www.ncbi.nlm.nih.gov/entrez/query.fcgi?db=gene&cmd=Retrieve&dopt=full_report&list_uids=100037417) | D-dopachrome tautomerase like |
| [Details](http://mirdb.org/cgi-bin/target_detail.cgi?targetID=1232371) | 397 | 78 | hsa-let-7d-5p | [NAT8L](http://www.ncbi.nlm.nih.gov/entrez/query.fcgi?db=gene&cmd=Retrieve&dopt=full_report&list_uids=339983) | N-acetyltransferase 8 like |
| [Details](http://mirdb.org/cgi-bin/target_detail.cgi?targetID=1232248) | 398 | 78 | hsa-let-7d-5p | [MTUS1](http://www.ncbi.nlm.nih.gov/entrez/query.fcgi?db=gene&cmd=Retrieve&dopt=full_report&list_uids=57509) | microtubule associated scaffold protein 1 |
| [Details](http://mirdb.org/cgi-bin/target_detail.cgi?targetID=1231791) | 399 | 78 | hsa-let-7d-5p | [AFF2](http://www.ncbi.nlm.nih.gov/entrez/query.fcgi?db=gene&cmd=Retrieve&dopt=full_report&list_uids=2334) | AF4/FMR2 family member 2 |
| [Details](http://mirdb.org/cgi-bin/target_detail.cgi?targetID=1231718) | 400 | 78 | hsa-let-7d-5p | [ARRDC4](http://www.ncbi.nlm.nih.gov/entrez/query.fcgi?db=gene&cmd=Retrieve&dopt=full_report&list_uids=91947) | arrestin domain containing 4 |
| [Details](http://mirdb.org/cgi-bin/target_detail.cgi?targetID=1231701) | 401 | 78 | hsa-let-7d-5p | [POGLUT1](http://www.ncbi.nlm.nih.gov/entrez/query.fcgi?db=gene&cmd=Retrieve&dopt=full_report&list_uids=56983) | protein O-glucosyltransferase 1 |
| [Details](http://mirdb.org/cgi-bin/target_detail.cgi?targetID=1232170) | 402 | 78 | hsa-let-7d-5p | [TXLNG](http://www.ncbi.nlm.nih.gov/entrez/query.fcgi?db=gene&cmd=Retrieve&dopt=full_report&list_uids=55787) | taxilin gamma |
| [Details](http://mirdb.org/cgi-bin/target_detail.cgi?targetID=1231818) | 403 | 78 | hsa-let-7d-5p | [MEIS2](http://www.ncbi.nlm.nih.gov/entrez/query.fcgi?db=gene&cmd=Retrieve&dopt=full_report&list_uids=4212) | Meis homeobox 2 |
| [Details](http://mirdb.org/cgi-bin/target_detail.cgi?targetID=1232139) | 404 | 78 | hsa-let-7d-5p | [LIPT2](http://www.ncbi.nlm.nih.gov/entrez/query.fcgi?db=gene&cmd=Retrieve&dopt=full_report&list_uids=387787) | lipoyl(octanoyl) transferase 2 |
| [Details](http://mirdb.org/cgi-bin/target_detail.cgi?targetID=1232102) | 405 | 78 | hsa-let-7d-5p | [WDR37](http://www.ncbi.nlm.nih.gov/entrez/query.fcgi?db=gene&cmd=Retrieve&dopt=full_report&list_uids=22884) | WD repeat domain 37 |
| [Details](http://mirdb.org/cgi-bin/target_detail.cgi?targetID=1231773) | 406 | 78 | hsa-let-7d-5p | [MFSD4A](http://www.ncbi.nlm.nih.gov/entrez/query.fcgi?db=gene&cmd=Retrieve&dopt=full_report&list_uids=148808) | major facilitator superfamily domain containing 4A |
| [Details](http://mirdb.org/cgi-bin/target_detail.cgi?targetID=1232030) | 407 | 77 | hsa-let-7d-5p | [MSN](http://www.ncbi.nlm.nih.gov/entrez/query.fcgi?db=gene&cmd=Retrieve&dopt=full_report&list_uids=4478) | moesin |
| [Details](http://mirdb.org/cgi-bin/target_detail.cgi?targetID=1231948) | 408 | 77 | hsa-let-7d-5p | [ZBTB10](http://www.ncbi.nlm.nih.gov/entrez/query.fcgi?db=gene&cmd=Retrieve&dopt=full_report&list_uids=65986) | zinc finger and BTB domain containing 10 |
| [Details](http://mirdb.org/cgi-bin/target_detail.cgi?targetID=1231691) | 409 | 77 | hsa-let-7d-5p | [TSC1](http://www.ncbi.nlm.nih.gov/entrez/query.fcgi?db=gene&cmd=Retrieve&dopt=full_report&list_uids=7248) | TSC complex subunit 1 |
| [Details](http://mirdb.org/cgi-bin/target_detail.cgi?targetID=1232326) | 410 | 77 | hsa-let-7d-5p | [IFI44L](http://www.ncbi.nlm.nih.gov/entrez/query.fcgi?db=gene&cmd=Retrieve&dopt=full_report&list_uids=10964) | interferon induced protein 44 like |
| [Details](http://mirdb.org/cgi-bin/target_detail.cgi?targetID=1231552) | 411 | 77 | hsa-let-7d-5p | [C20orf194](http://www.ncbi.nlm.nih.gov/entrez/query.fcgi?db=gene&cmd=Retrieve&dopt=full_report&list_uids=25943) | chromosome 20 open reading frame 194 |
| [Details](http://mirdb.org/cgi-bin/target_detail.cgi?targetID=1232163) | 412 | 77 | hsa-let-7d-5p | [PLD3](http://www.ncbi.nlm.nih.gov/entrez/query.fcgi?db=gene&cmd=Retrieve&dopt=full_report&list_uids=23646) | phospholipase D family member 3 |
| [Details](http://mirdb.org/cgi-bin/target_detail.cgi?targetID=1232312) | 413 | 77 | hsa-let-7d-5p | [KIAA0930](http://www.ncbi.nlm.nih.gov/entrez/query.fcgi?db=gene&cmd=Retrieve&dopt=full_report&list_uids=23313) | KIAA0930 |
| [Details](http://mirdb.org/cgi-bin/target_detail.cgi?targetID=1231785) | 414 | 77 | hsa-let-7d-5p | [C6orf203](http://www.ncbi.nlm.nih.gov/entrez/query.fcgi?db=gene&cmd=Retrieve&dopt=full_report&list_uids=51250) | chromosome 6 open reading frame 203 |
| [Details](http://mirdb.org/cgi-bin/target_detail.cgi?targetID=1231998) | 415 | 77 | hsa-let-7d-5p | [ZNF652](http://www.ncbi.nlm.nih.gov/entrez/query.fcgi?db=gene&cmd=Retrieve&dopt=full_report&list_uids=22834) | zinc finger protein 652 |
| [Details](http://mirdb.org/cgi-bin/target_detail.cgi?targetID=1232375) | 416 | 77 | hsa-let-7d-5p | [RNF44](http://www.ncbi.nlm.nih.gov/entrez/query.fcgi?db=gene&cmd=Retrieve&dopt=full_report&list_uids=22838) | ring finger protein 44 |
| [Details](http://mirdb.org/cgi-bin/target_detail.cgi?targetID=1231908) | 417 | 77 | hsa-let-7d-5p | [GGA3](http://www.ncbi.nlm.nih.gov/entrez/query.fcgi?db=gene&cmd=Retrieve&dopt=full_report&list_uids=23163) | golgi associated, gamma adaptin ear containing, ARF binding protein 3 |
| [Details](http://mirdb.org/cgi-bin/target_detail.cgi?targetID=1232304) | 418 | 77 | hsa-let-7d-5p | [RBMS1](http://www.ncbi.nlm.nih.gov/entrez/query.fcgi?db=gene&cmd=Retrieve&dopt=full_report&list_uids=5937) | RNA binding motif single stranded interacting protein 1 |
| [Details](http://mirdb.org/cgi-bin/target_detail.cgi?targetID=1232186) | 419 | 77 | hsa-let-7d-5p | [COL5A2](http://www.ncbi.nlm.nih.gov/entrez/query.fcgi?db=gene&cmd=Retrieve&dopt=full_report&list_uids=1290) | collagen type V alpha 2 chain |
| [Details](http://mirdb.org/cgi-bin/target_detail.cgi?targetID=1231925) | 420 | 77 | hsa-let-7d-5p | [ZBTB39](http://www.ncbi.nlm.nih.gov/entrez/query.fcgi?db=gene&cmd=Retrieve&dopt=full_report&list_uids=9880) | zinc finger and BTB domain containing 39 |
| [Details](http://mirdb.org/cgi-bin/target_detail.cgi?targetID=1232148) | 421 | 77 | hsa-let-7d-5p | [SIGMAR1](http://www.ncbi.nlm.nih.gov/entrez/query.fcgi?db=gene&cmd=Retrieve&dopt=full_report&list_uids=10280) | sigma non-opioid intracellular receptor 1 |
| [Details](http://mirdb.org/cgi-bin/target_detail.cgi?targetID=1232059) | 422 | 77 | hsa-let-7d-5p | [SLC30A4](http://www.ncbi.nlm.nih.gov/entrez/query.fcgi?db=gene&cmd=Retrieve&dopt=full_report&list_uids=7782) | solute carrier family 30 member 4 |
| [Details](http://mirdb.org/cgi-bin/target_detail.cgi?targetID=1232369) | 423 | 77 | hsa-let-7d-5p | [PARP8](http://www.ncbi.nlm.nih.gov/entrez/query.fcgi?db=gene&cmd=Retrieve&dopt=full_report&list_uids=79668) | poly(ADP-ribose) polymerase family member 8 |
| [Details](http://mirdb.org/cgi-bin/target_detail.cgi?targetID=1231876) | 424 | 77 | hsa-let-7d-5p | [CCR7](http://www.ncbi.nlm.nih.gov/entrez/query.fcgi?db=gene&cmd=Retrieve&dopt=full_report&list_uids=1236) | C-C motif chemokine receptor 7 |
| [Details](http://mirdb.org/cgi-bin/target_detail.cgi?targetID=1232131) | 425 | 77 | hsa-let-7d-5p | [SMAP1](http://www.ncbi.nlm.nih.gov/entrez/query.fcgi?db=gene&cmd=Retrieve&dopt=full_report&list_uids=60682) | small ArfGAP 1 |
| [Details](http://mirdb.org/cgi-bin/target_detail.cgi?targetID=1232234) | 426 | 77 | hsa-let-7d-5p | [SLC22A23](http://www.ncbi.nlm.nih.gov/entrez/query.fcgi?db=gene&cmd=Retrieve&dopt=full_report&list_uids=63027) | solute carrier family 22 member 23 |
| [Details](http://mirdb.org/cgi-bin/target_detail.cgi?targetID=1232123) | 427 | 77 | hsa-let-7d-5p | [ESR2](http://www.ncbi.nlm.nih.gov/entrez/query.fcgi?db=gene&cmd=Retrieve&dopt=full_report&list_uids=2100) | estrogen receptor 2 |
| [Details](http://mirdb.org/cgi-bin/target_detail.cgi?targetID=1231526) | 428 | 77 | hsa-let-7d-5p | [SUCLG2](http://www.ncbi.nlm.nih.gov/entrez/query.fcgi?db=gene&cmd=Retrieve&dopt=full_report&list_uids=8801) | succinate-CoA ligase GDP-forming beta subunit |
| [Details](http://mirdb.org/cgi-bin/target_detail.cgi?targetID=1232023) | 429 | 77 | hsa-let-7d-5p | [PRPF38B](http://www.ncbi.nlm.nih.gov/entrez/query.fcgi?db=gene&cmd=Retrieve&dopt=full_report&list_uids=55119) | pre-mRNA processing factor 38B |
| [Details](http://mirdb.org/cgi-bin/target_detail.cgi?targetID=1232373) | 430 | 77 | hsa-let-7d-5p | [MAP3K2](http://www.ncbi.nlm.nih.gov/entrez/query.fcgi?db=gene&cmd=Retrieve&dopt=full_report&list_uids=10746) | mitogen-activated protein kinase kinase kinase 2 |
| [Details](http://mirdb.org/cgi-bin/target_detail.cgi?targetID=1232339) | 431 | 77 | hsa-let-7d-5p | [CPEB3](http://www.ncbi.nlm.nih.gov/entrez/query.fcgi?db=gene&cmd=Retrieve&dopt=full_report&list_uids=22849) | cytoplasmic polyadenylation element binding protein 3 |
| [Details](http://mirdb.org/cgi-bin/target_detail.cgi?targetID=1232300) | 432 | 77 | hsa-let-7d-5p | [MAP4K4](http://www.ncbi.nlm.nih.gov/entrez/query.fcgi?db=gene&cmd=Retrieve&dopt=full_report&list_uids=9448) | mitogen-activated protein kinase kinase kinase kinase 4 |
| [Details](http://mirdb.org/cgi-bin/target_detail.cgi?targetID=1232365) | 433 | 76 | hsa-let-7d-5p | [C18orf21](http://www.ncbi.nlm.nih.gov/entrez/query.fcgi?db=gene&cmd=Retrieve&dopt=full_report&list_uids=83608) | chromosome 18 open reading frame 21 |
| [Details](http://mirdb.org/cgi-bin/target_detail.cgi?targetID=1232121) | 434 | 76 | hsa-let-7d-5p | [TRMT13](http://www.ncbi.nlm.nih.gov/entrez/query.fcgi?db=gene&cmd=Retrieve&dopt=full_report&list_uids=54482) | tRNA methyltransferase 13 homolog |
| [Details](http://mirdb.org/cgi-bin/target_detail.cgi?targetID=1232307) | 435 | 76 | hsa-let-7d-5p | [TMED5](http://www.ncbi.nlm.nih.gov/entrez/query.fcgi?db=gene&cmd=Retrieve&dopt=full_report&list_uids=50999) | transmembrane p24 trafficking protein 5 |
| [Details](http://mirdb.org/cgi-bin/target_detail.cgi?targetID=1231518) | 436 | 76 | hsa-let-7d-5p | [FOXP2](http://www.ncbi.nlm.nih.gov/entrez/query.fcgi?db=gene&cmd=Retrieve&dopt=full_report&list_uids=93986) | forkhead box P2 |
| [Details](http://mirdb.org/cgi-bin/target_detail.cgi?targetID=1231711) | 437 | 76 | hsa-let-7d-5p | [SCN5A](http://www.ncbi.nlm.nih.gov/entrez/query.fcgi?db=gene&cmd=Retrieve&dopt=full_report&list_uids=6331) | sodium voltage-gated channel alpha subunit 5 |
| [Details](http://mirdb.org/cgi-bin/target_detail.cgi?targetID=1231634) | 438 | 76 | hsa-let-7d-5p | [PRKAR2A](http://www.ncbi.nlm.nih.gov/entrez/query.fcgi?db=gene&cmd=Retrieve&dopt=full_report&list_uids=5576) | protein kinase cAMP-dependent type II regulatory subunit alpha |
| [Details](http://mirdb.org/cgi-bin/target_detail.cgi?targetID=1232286) | 439 | 76 | hsa-let-7d-5p | [ZNF689](http://www.ncbi.nlm.nih.gov/entrez/query.fcgi?db=gene&cmd=Retrieve&dopt=full_report&list_uids=115509) | zinc finger protein 689 |
| [Details](http://mirdb.org/cgi-bin/target_detail.cgi?targetID=1232233) | 440 | 76 | hsa-let-7d-5p | [GALNT15](http://www.ncbi.nlm.nih.gov/entrez/query.fcgi?db=gene&cmd=Retrieve&dopt=full_report&list_uids=117248) | polypeptide N-acetylgalactosaminyltransferase 15 |
| [Details](http://mirdb.org/cgi-bin/target_detail.cgi?targetID=1231591) | 441 | 76 | hsa-let-7d-5p | [SLK](http://www.ncbi.nlm.nih.gov/entrez/query.fcgi?db=gene&cmd=Retrieve&dopt=full_report&list_uids=9748) | STE20 like kinase |
| [Details](http://mirdb.org/cgi-bin/target_detail.cgi?targetID=1231684) | 442 | 76 | hsa-let-7d-5p | [IKZF2](http://www.ncbi.nlm.nih.gov/entrez/query.fcgi?db=gene&cmd=Retrieve&dopt=full_report&list_uids=22807) | IKAROS family zinc finger 2 |
| [Details](http://mirdb.org/cgi-bin/target_detail.cgi?targetID=1232243) | 443 | 76 | hsa-let-7d-5p | [ARHGEF15](http://www.ncbi.nlm.nih.gov/entrez/query.fcgi?db=gene&cmd=Retrieve&dopt=full_report&list_uids=22899) | Rho guanine nucleotide exchange factor 15 |
| [Details](http://mirdb.org/cgi-bin/target_detail.cgi?targetID=1232116) | 444 | 76 | hsa-let-7d-5p | [P4HA2](http://www.ncbi.nlm.nih.gov/entrez/query.fcgi?db=gene&cmd=Retrieve&dopt=full_report&list_uids=8974) | prolyl 4-hydroxylase subunit alpha 2 |
| [Details](http://mirdb.org/cgi-bin/target_detail.cgi?targetID=1231778) | 445 | 75 | hsa-let-7d-5p | [AMER3](http://www.ncbi.nlm.nih.gov/entrez/query.fcgi?db=gene&cmd=Retrieve&dopt=full_report&list_uids=205147) | APC membrane recruitment protein 3 |
| [Details](http://mirdb.org/cgi-bin/target_detail.cgi?targetID=1231660) | 446 | 75 | hsa-let-7d-5p | [PALM3](http://www.ncbi.nlm.nih.gov/entrez/query.fcgi?db=gene&cmd=Retrieve&dopt=full_report&list_uids=342979) | paralemmin 3 |
| [Details](http://mirdb.org/cgi-bin/target_detail.cgi?targetID=1231942) | 447 | 75 | hsa-let-7d-5p | [ARPP19](http://www.ncbi.nlm.nih.gov/entrez/query.fcgi?db=gene&cmd=Retrieve&dopt=full_report&list_uids=10776) | cAMP regulated phosphoprotein 19 |
| [Details](http://mirdb.org/cgi-bin/target_detail.cgi?targetID=1232225) | 448 | 75 | hsa-let-7d-5p | [LCORL](http://www.ncbi.nlm.nih.gov/entrez/query.fcgi?db=gene&cmd=Retrieve&dopt=full_report&list_uids=254251) | ligand dependent nuclear receptor corepressor like |
| [Details](http://mirdb.org/cgi-bin/target_detail.cgi?targetID=1232101) | 449 | 75 | hsa-let-7d-5p | [NLRP2B](http://www.ncbi.nlm.nih.gov/entrez/query.fcgi?db=gene&cmd=Retrieve&dopt=full_report&list_uids=286430) | NLR family pyrin domain containing 2B |
| [Details](http://mirdb.org/cgi-bin/target_detail.cgi?targetID=1231689) | 450 | 75 | hsa-let-7d-5p | [PHOSPHO2-KLHL23](http://www.ncbi.nlm.nih.gov/entrez/query.fcgi?db=gene&cmd=Retrieve&dopt=full_report&list_uids=100526832) | PHOSPHO2-KLHL23 readthrough |
| [Details](http://mirdb.org/cgi-bin/target_detail.cgi?targetID=1231853) | 451 | 75 | hsa-let-7d-5p | [FNDC3B](http://www.ncbi.nlm.nih.gov/entrez/query.fcgi?db=gene&cmd=Retrieve&dopt=full_report&list_uids=64778) | fibronectin type III domain containing 3B |
| [Details](http://mirdb.org/cgi-bin/target_detail.cgi?targetID=1231620) | 452 | 75 | hsa-let-7d-5p | [EPHA4](http://www.ncbi.nlm.nih.gov/entrez/query.fcgi?db=gene&cmd=Retrieve&dopt=full_report&list_uids=2043) | EPH receptor A4 |
| [Details](http://mirdb.org/cgi-bin/target_detail.cgi?targetID=1232279) | 453 | 75 | hsa-let-7d-5p | [RBMS2](http://www.ncbi.nlm.nih.gov/entrez/query.fcgi?db=gene&cmd=Retrieve&dopt=full_report&list_uids=5939) | RNA binding motif single stranded interacting protein 2 |
| [Details](http://mirdb.org/cgi-bin/target_detail.cgi?targetID=1232385) | 454 | 75 | hsa-let-7d-5p | [ZNF10](http://www.ncbi.nlm.nih.gov/entrez/query.fcgi?db=gene&cmd=Retrieve&dopt=full_report&list_uids=7556) | zinc finger protein 10 |
| [Details](http://mirdb.org/cgi-bin/target_detail.cgi?targetID=1231868) | 455 | 75 | hsa-let-7d-5p | [XYLT1](http://www.ncbi.nlm.nih.gov/entrez/query.fcgi?db=gene&cmd=Retrieve&dopt=full_report&list_uids=64131) | xylosyltransferase 1 |
| [Details](http://mirdb.org/cgi-bin/target_detail.cgi?targetID=1232201) | 456 | 75 | hsa-let-7d-5p | [KLHL23](http://www.ncbi.nlm.nih.gov/entrez/query.fcgi?db=gene&cmd=Retrieve&dopt=full_report&list_uids=151230) | kelch like family member 23 |
| [Details](http://mirdb.org/cgi-bin/target_detail.cgi?targetID=1231642) | 457 | 75 | hsa-let-7d-5p | [TSC22D2](http://www.ncbi.nlm.nih.gov/entrez/query.fcgi?db=gene&cmd=Retrieve&dopt=full_report&list_uids=9819) | TSC22 domain family member 2 |
| [Details](http://mirdb.org/cgi-bin/target_detail.cgi?targetID=1232109) | 458 | 75 | hsa-let-7d-5p | [MAP3K1](http://www.ncbi.nlm.nih.gov/entrez/query.fcgi?db=gene&cmd=Retrieve&dopt=full_report&list_uids=4214) | mitogen-activated protein kinase kinase kinase 1 |
| [Details](http://mirdb.org/cgi-bin/target_detail.cgi?targetID=1232151) | 459 | 74 | hsa-let-7d-5p | [KLHL6](http://www.ncbi.nlm.nih.gov/entrez/query.fcgi?db=gene&cmd=Retrieve&dopt=full_report&list_uids=89857) | kelch like family member 6 |
| [Details](http://mirdb.org/cgi-bin/target_detail.cgi?targetID=1231957) | 460 | 74 | hsa-let-7d-5p | [ADRB1](http://www.ncbi.nlm.nih.gov/entrez/query.fcgi?db=gene&cmd=Retrieve&dopt=full_report&list_uids=153) | adrenoceptor beta 1 |
| [Details](http://mirdb.org/cgi-bin/target_detail.cgi?targetID=1232060) | 461 | 74 | hsa-let-7d-5p | [SDR42E1](http://www.ncbi.nlm.nih.gov/entrez/query.fcgi?db=gene&cmd=Retrieve&dopt=full_report&list_uids=93517) | short chain dehydrogenase/reductase family 42E, member 1 |
| [Details](http://mirdb.org/cgi-bin/target_detail.cgi?targetID=1232118) | 462 | 74 | hsa-let-7d-5p | [PRKAA2](http://www.ncbi.nlm.nih.gov/entrez/query.fcgi?db=gene&cmd=Retrieve&dopt=full_report&list_uids=5563) | protein kinase AMP-activated catalytic subunit alpha 2 |
| [Details](http://mirdb.org/cgi-bin/target_detail.cgi?targetID=1231478) | 463 | 74 | hsa-let-7d-5p | [SCN11A](http://www.ncbi.nlm.nih.gov/entrez/query.fcgi?db=gene&cmd=Retrieve&dopt=full_report&list_uids=11280) | sodium voltage-gated channel alpha subunit 11 |
| [Details](http://mirdb.org/cgi-bin/target_detail.cgi?targetID=1232306) | 464 | 74 | hsa-let-7d-5p | [MED28](http://www.ncbi.nlm.nih.gov/entrez/query.fcgi?db=gene&cmd=Retrieve&dopt=full_report&list_uids=80306) | mediator complex subunit 28 |
| [Details](http://mirdb.org/cgi-bin/target_detail.cgi?targetID=1231872) | 465 | 74 | hsa-let-7d-5p | [DCUN1D2](http://www.ncbi.nlm.nih.gov/entrez/query.fcgi?db=gene&cmd=Retrieve&dopt=full_report&list_uids=55208) | defective in cullin neddylation 1 domain containing 2 |
| [Details](http://mirdb.org/cgi-bin/target_detail.cgi?targetID=1231654) | 466 | 74 | hsa-let-7d-5p | [NKD1](http://www.ncbi.nlm.nih.gov/entrez/query.fcgi?db=gene&cmd=Retrieve&dopt=full_report&list_uids=85407) | NKD1, WNT signaling pathway inhibitor |
| [Details](http://mirdb.org/cgi-bin/target_detail.cgi?targetID=1232035) | 467 | 74 | hsa-let-7d-5p | [EOGT](http://www.ncbi.nlm.nih.gov/entrez/query.fcgi?db=gene&cmd=Retrieve&dopt=full_report&list_uids=285203) | EGF domain specific O-linked N-acetylglucosamine transferase |
| [Details](http://mirdb.org/cgi-bin/target_detail.cgi?targetID=1231833) | 468 | 74 | hsa-let-7d-5p | [SMUG1](http://www.ncbi.nlm.nih.gov/entrez/query.fcgi?db=gene&cmd=Retrieve&dopt=full_report&list_uids=23583) | single-strand-selective monofunctional uracil-DNA glycosylase 1 |
| [Details](http://mirdb.org/cgi-bin/target_detail.cgi?targetID=1231542) | 469 | 74 | hsa-let-7d-5p | [NPEPL1](http://www.ncbi.nlm.nih.gov/entrez/query.fcgi?db=gene&cmd=Retrieve&dopt=full_report&list_uids=79716) | aminopeptidase like 1 |
| [Details](http://mirdb.org/cgi-bin/target_detail.cgi?targetID=1231544) | 470 | 74 | hsa-let-7d-5p | [VAV3](http://www.ncbi.nlm.nih.gov/entrez/query.fcgi?db=gene&cmd=Retrieve&dopt=full_report&list_uids=10451) | vav guanine nucleotide exchange factor 3 |
| [Details](http://mirdb.org/cgi-bin/target_detail.cgi?targetID=1231892) | 471 | 74 | hsa-let-7d-5p | [SEMA3F](http://www.ncbi.nlm.nih.gov/entrez/query.fcgi?db=gene&cmd=Retrieve&dopt=full_report&list_uids=6405) | semaphorin 3F |
| [Details](http://mirdb.org/cgi-bin/target_detail.cgi?targetID=1231736) | 472 | 74 | hsa-let-7d-5p | [ERVH48-1](http://www.ncbi.nlm.nih.gov/entrez/query.fcgi?db=gene&cmd=Retrieve&dopt=full_report&list_uids=90625) | endogenous retrovirus group 48 member 1 |
| [Details](http://mirdb.org/cgi-bin/target_detail.cgi?targetID=1231731) | 473 | 74 | hsa-let-7d-5p | [SRD5A3](http://www.ncbi.nlm.nih.gov/entrez/query.fcgi?db=gene&cmd=Retrieve&dopt=full_report&list_uids=79644) | steroid 5 alpha-reductase 3 |
| [Details](http://mirdb.org/cgi-bin/target_detail.cgi?targetID=1231694) | 474 | 74 | hsa-let-7d-5p | [PCDH20](http://www.ncbi.nlm.nih.gov/entrez/query.fcgi?db=gene&cmd=Retrieve&dopt=full_report&list_uids=64881) | protocadherin 20 |
| [Details](http://mirdb.org/cgi-bin/target_detail.cgi?targetID=1231913) | 475 | 74 | hsa-let-7d-5p | [BRWD3](http://www.ncbi.nlm.nih.gov/entrez/query.fcgi?db=gene&cmd=Retrieve&dopt=full_report&list_uids=254065) | bromodomain and WD repeat domain containing 3 |
| [Details](http://mirdb.org/cgi-bin/target_detail.cgi?targetID=1231567) | 476 | 74 | hsa-let-7d-5p | [CCDC71L](http://www.ncbi.nlm.nih.gov/entrez/query.fcgi?db=gene&cmd=Retrieve&dopt=full_report&list_uids=168455) | coiled-coil domain containing 71 like |
| [Details](http://mirdb.org/cgi-bin/target_detail.cgi?targetID=1231900) | 477 | 74 | hsa-let-7d-5p | [SERF2](http://www.ncbi.nlm.nih.gov/entrez/query.fcgi?db=gene&cmd=Retrieve&dopt=full_report&list_uids=10169) | small EDRK-rich factor 2 |
| [Details](http://mirdb.org/cgi-bin/target_detail.cgi?targetID=1232098) | 478 | 74 | hsa-let-7d-5p | [IL10](http://www.ncbi.nlm.nih.gov/entrez/query.fcgi?db=gene&cmd=Retrieve&dopt=full_report&list_uids=3586) | interleukin 10 |
| [Details](http://mirdb.org/cgi-bin/target_detail.cgi?targetID=1231846) | 479 | 74 | hsa-let-7d-5p | [CNTRL](http://www.ncbi.nlm.nih.gov/entrez/query.fcgi?db=gene&cmd=Retrieve&dopt=full_report&list_uids=11064) | centriolin |
| [Details](http://mirdb.org/cgi-bin/target_detail.cgi?targetID=1231608) | 480 | 74 | hsa-let-7d-5p | [NOL4L](http://www.ncbi.nlm.nih.gov/entrez/query.fcgi?db=gene&cmd=Retrieve&dopt=full_report&list_uids=140688) | nucleolar protein 4 like |
| [Details](http://mirdb.org/cgi-bin/target_detail.cgi?targetID=1232145) | 481 | 74 | hsa-let-7d-5p | [MYORG](http://www.ncbi.nlm.nih.gov/entrez/query.fcgi?db=gene&cmd=Retrieve&dopt=full_report&list_uids=57462) | myogenesis regulating glycosidase (putative) |
| [Details](http://mirdb.org/cgi-bin/target_detail.cgi?targetID=1231886) | 482 | 74 | hsa-let-7d-5p | [TMEM234](http://www.ncbi.nlm.nih.gov/entrez/query.fcgi?db=gene&cmd=Retrieve&dopt=full_report&list_uids=56063) | transmembrane protein 234 |
| [Details](http://mirdb.org/cgi-bin/target_detail.cgi?targetID=1231971) | 483 | 74 | hsa-let-7d-5p | [DCUN1D3](http://www.ncbi.nlm.nih.gov/entrez/query.fcgi?db=gene&cmd=Retrieve&dopt=full_report&list_uids=123879) | defective in cullin neddylation 1 domain containing 3 |
| [Details](http://mirdb.org/cgi-bin/target_detail.cgi?targetID=1231522) | 484 | 74 | hsa-let-7d-5p | [CD200R1](http://www.ncbi.nlm.nih.gov/entrez/query.fcgi?db=gene&cmd=Retrieve&dopt=full_report&list_uids=131450) | CD200 receptor 1 |
| [Details](http://mirdb.org/cgi-bin/target_detail.cgi?targetID=1232217) | 485 | 73 | hsa-let-7d-5p | [TNFRSF1B](http://www.ncbi.nlm.nih.gov/entrez/query.fcgi?db=gene&cmd=Retrieve&dopt=full_report&list_uids=7133) | TNF receptor superfamily member 1B |
| [Details](http://mirdb.org/cgi-bin/target_detail.cgi?targetID=1231969) | 486 | 73 | hsa-let-7d-5p | [SEMA4G](http://www.ncbi.nlm.nih.gov/entrez/query.fcgi?db=gene&cmd=Retrieve&dopt=full_report&list_uids=57715) | semaphorin 4G |
| [Details](http://mirdb.org/cgi-bin/target_detail.cgi?targetID=1232235) | 487 | 73 | hsa-let-7d-5p | [PARM1](http://www.ncbi.nlm.nih.gov/entrez/query.fcgi?db=gene&cmd=Retrieve&dopt=full_report&list_uids=25849) | prostate androgen-regulated mucin-like protein 1 |
| [Details](http://mirdb.org/cgi-bin/target_detail.cgi?targetID=1232210) | 488 | 73 | hsa-let-7d-5p | [BCAT1](http://www.ncbi.nlm.nih.gov/entrez/query.fcgi?db=gene&cmd=Retrieve&dopt=full_report&list_uids=586) | branched chain amino acid transaminase 1 |
| [Details](http://mirdb.org/cgi-bin/target_detail.cgi?targetID=1232348) | 489 | 73 | hsa-let-7d-5p | [GPAT4](http://www.ncbi.nlm.nih.gov/entrez/query.fcgi?db=gene&cmd=Retrieve&dopt=full_report&list_uids=137964) | glycerol-3-phosphate acyltransferase 4 |
| [Details](http://mirdb.org/cgi-bin/target_detail.cgi?targetID=1231815) | 490 | 73 | hsa-let-7d-5p | [PQLC2](http://www.ncbi.nlm.nih.gov/entrez/query.fcgi?db=gene&cmd=Retrieve&dopt=full_report&list_uids=54896) | PQ loop repeat containing 2 |
| [Details](http://mirdb.org/cgi-bin/target_detail.cgi?targetID=1231602) | 491 | 73 | hsa-let-7d-5p | [NEMP1](http://www.ncbi.nlm.nih.gov/entrez/query.fcgi?db=gene&cmd=Retrieve&dopt=full_report&list_uids=23306) | nuclear envelope integral membrane protein 1 |
| [Details](http://mirdb.org/cgi-bin/target_detail.cgi?targetID=1231874) | 492 | 73 | hsa-let-7d-5p | [A1CF](http://www.ncbi.nlm.nih.gov/entrez/query.fcgi?db=gene&cmd=Retrieve&dopt=full_report&list_uids=29974) | APOBEC1 complementation factor |
| [Details](http://mirdb.org/cgi-bin/target_detail.cgi?targetID=1231849) | 493 | 73 | hsa-let-7d-5p | [KATNAL1](http://www.ncbi.nlm.nih.gov/entrez/query.fcgi?db=gene&cmd=Retrieve&dopt=full_report&list_uids=84056) | katanin catalytic subunit A1 like 1 |
| [Details](http://mirdb.org/cgi-bin/target_detail.cgi?targetID=1231862) | 494 | 73 | hsa-let-7d-5p | [TREML1](http://www.ncbi.nlm.nih.gov/entrez/query.fcgi?db=gene&cmd=Retrieve&dopt=full_report&list_uids=340205) | triggering receptor expressed on myeloid cells like 1 |
| [Details](http://mirdb.org/cgi-bin/target_detail.cgi?targetID=1231772) | 495 | 73 | hsa-let-7d-5p | [VSNL1](http://www.ncbi.nlm.nih.gov/entrez/query.fcgi?db=gene&cmd=Retrieve&dopt=full_report&list_uids=7447) | visinin like 1 |
| [Details](http://mirdb.org/cgi-bin/target_detail.cgi?targetID=1231598) | 496 | 73 | hsa-let-7d-5p | [ABHD17C](http://www.ncbi.nlm.nih.gov/entrez/query.fcgi?db=gene&cmd=Retrieve&dopt=full_report&list_uids=58489) | abhydrolase domain containing 17C |
| [Details](http://mirdb.org/cgi-bin/target_detail.cgi?targetID=1231600) | 497 | 73 | hsa-let-7d-5p | [RMI2](http://www.ncbi.nlm.nih.gov/entrez/query.fcgi?db=gene&cmd=Retrieve&dopt=full_report&list_uids=116028) | RecQ mediated genome instability 2 |
| [Details](http://mirdb.org/cgi-bin/target_detail.cgi?targetID=1231723) | 498 | 73 | hsa-let-7d-5p | [RCN1](http://www.ncbi.nlm.nih.gov/entrez/query.fcgi?db=gene&cmd=Retrieve&dopt=full_report&list_uids=5954) | reticulocalbin 1 |
| [Details](http://mirdb.org/cgi-bin/target_detail.cgi?targetID=1231917) | 499 | 73 | hsa-let-7d-5p | [TRPM6](http://www.ncbi.nlm.nih.gov/entrez/query.fcgi?db=gene&cmd=Retrieve&dopt=full_report&list_uids=140803) | transient receptor potential cation channel subfamily M member 6 |
| [Details](http://mirdb.org/cgi-bin/target_detail.cgi?targetID=1231720) | 500 | 73 | hsa-let-7d-5p | [AMMECR1L](http://www.ncbi.nlm.nih.gov/entrez/query.fcgi?db=gene&cmd=Retrieve&dopt=full_report&list_uids=83607) | AMMECR1 like |
| [Details](http://mirdb.org/cgi-bin/target_detail.cgi?targetID=1231489) | 501 | 73 | hsa-let-7d-5p | [LTN1](http://www.ncbi.nlm.nih.gov/entrez/query.fcgi?db=gene&cmd=Retrieve&dopt=full_report&list_uids=26046) | listerin E3 ubiquitin protein ligase 1 |
| [Details](http://mirdb.org/cgi-bin/target_detail.cgi?targetID=1231609) | 502 | 72 | hsa-let-7d-5p | [TRIB1](http://www.ncbi.nlm.nih.gov/entrez/query.fcgi?db=gene&cmd=Retrieve&dopt=full_report&list_uids=10221) | tribbles pseudokinase 1 |
| [Details](http://mirdb.org/cgi-bin/target_detail.cgi?targetID=1232287) | 503 | 72 | hsa-let-7d-5p | [STOX2](http://www.ncbi.nlm.nih.gov/entrez/query.fcgi?db=gene&cmd=Retrieve&dopt=full_report&list_uids=56977) | storkhead box 2 |
| [Details](http://mirdb.org/cgi-bin/target_detail.cgi?targetID=1232328) | 504 | 72 | hsa-let-7d-5p | [SECISBP2L](http://www.ncbi.nlm.nih.gov/entrez/query.fcgi?db=gene&cmd=Retrieve&dopt=full_report&list_uids=9728) | SECIS binding protein 2 like |
| [Details](http://mirdb.org/cgi-bin/target_detail.cgi?targetID=1231485) | 505 | 72 | hsa-let-7d-5p | [ABHD14B](http://www.ncbi.nlm.nih.gov/entrez/query.fcgi?db=gene&cmd=Retrieve&dopt=full_report&list_uids=84836) | abhydrolase domain containing 14B |
| [Details](http://mirdb.org/cgi-bin/target_detail.cgi?targetID=1231471) | 506 | 72 | hsa-let-7d-5p | [PNKD](http://www.ncbi.nlm.nih.gov/entrez/query.fcgi?db=gene&cmd=Retrieve&dopt=full_report&list_uids=25953) | PNKD, MBL domain containing |
| [Details](http://mirdb.org/cgi-bin/target_detail.cgi?targetID=1231861) | 507 | 72 | hsa-let-7d-5p | [DPP3](http://www.ncbi.nlm.nih.gov/entrez/query.fcgi?db=gene&cmd=Retrieve&dopt=full_report&list_uids=10072) | dipeptidyl peptidase 3 |
| [Details](http://mirdb.org/cgi-bin/target_detail.cgi?targetID=1231537) | 508 | 72 | hsa-let-7d-5p | [MSR1](http://www.ncbi.nlm.nih.gov/entrez/query.fcgi?db=gene&cmd=Retrieve&dopt=full_report&list_uids=4481) | macrophage scavenger receptor 1 |
| [Details](http://mirdb.org/cgi-bin/target_detail.cgi?targetID=1231891) | 509 | 72 | hsa-let-7d-5p | [CUX1](http://www.ncbi.nlm.nih.gov/entrez/query.fcgi?db=gene&cmd=Retrieve&dopt=full_report&list_uids=1523) | cut like homeobox 1 |
| [Details](http://mirdb.org/cgi-bin/target_detail.cgi?targetID=1232310) | 510 | 72 | hsa-let-7d-5p | [MOB4](http://www.ncbi.nlm.nih.gov/entrez/query.fcgi?db=gene&cmd=Retrieve&dopt=full_report&list_uids=25843) | MOB family member 4, phocein |
| [Details](http://mirdb.org/cgi-bin/target_detail.cgi?targetID=1231536) | 511 | 72 | hsa-let-7d-5p | [CDV3](http://www.ncbi.nlm.nih.gov/entrez/query.fcgi?db=gene&cmd=Retrieve&dopt=full_report&list_uids=55573) | CDV3 homolog |
| [Details](http://mirdb.org/cgi-bin/target_detail.cgi?targetID=1231683) | 512 | 72 | hsa-let-7d-5p | [SEC14L1](http://www.ncbi.nlm.nih.gov/entrez/query.fcgi?db=gene&cmd=Retrieve&dopt=full_report&list_uids=6397) | SEC14 like lipid binding 1 |
| [Details](http://mirdb.org/cgi-bin/target_detail.cgi?targetID=1231497) | 513 | 72 | hsa-let-7d-5p | [ZCCHC9](http://www.ncbi.nlm.nih.gov/entrez/query.fcgi?db=gene&cmd=Retrieve&dopt=full_report&list_uids=84240) | zinc finger CCHC-type containing 9 |
| [Details](http://mirdb.org/cgi-bin/target_detail.cgi?targetID=1232012) | 514 | 72 | hsa-let-7d-5p | [PLXNA4](http://www.ncbi.nlm.nih.gov/entrez/query.fcgi?db=gene&cmd=Retrieve&dopt=full_report&list_uids=91584) | plexin A4 |
| [Details](http://mirdb.org/cgi-bin/target_detail.cgi?targetID=1231837) | 515 | 72 | hsa-let-7d-5p | [ZNF697](http://www.ncbi.nlm.nih.gov/entrez/query.fcgi?db=gene&cmd=Retrieve&dopt=full_report&list_uids=90874) | zinc finger protein 697 |
| [Details](http://mirdb.org/cgi-bin/target_detail.cgi?targetID=1232278) | 516 | 72 | hsa-let-7d-5p | [SLC25A18](http://www.ncbi.nlm.nih.gov/entrez/query.fcgi?db=gene&cmd=Retrieve&dopt=full_report&list_uids=83733) | solute carrier family 25 member 18 |
| [Details](http://mirdb.org/cgi-bin/target_detail.cgi?targetID=1231576) | 517 | 72 | hsa-let-7d-5p | [TTL](http://www.ncbi.nlm.nih.gov/entrez/query.fcgi?db=gene&cmd=Retrieve&dopt=full_report&list_uids=150465) | tubulin tyrosine ligase |
| [Details](http://mirdb.org/cgi-bin/target_detail.cgi?targetID=1231647) | 518 | 72 | hsa-let-7d-5p | [ADAMTS6](http://www.ncbi.nlm.nih.gov/entrez/query.fcgi?db=gene&cmd=Retrieve&dopt=full_report&list_uids=11174) | ADAM metallopeptidase with thrombospondin type 1 motif 6 |
| [Details](http://mirdb.org/cgi-bin/target_detail.cgi?targetID=1231589) | 519 | 71 | hsa-let-7d-5p | [GRID2IP](http://www.ncbi.nlm.nih.gov/entrez/query.fcgi?db=gene&cmd=Retrieve&dopt=full_report&list_uids=392862) | Grid2 interacting protein |
| [Details](http://mirdb.org/cgi-bin/target_detail.cgi?targetID=1231592) | 520 | 71 | hsa-let-7d-5p | [EIF2S2](http://www.ncbi.nlm.nih.gov/entrez/query.fcgi?db=gene&cmd=Retrieve&dopt=full_report&list_uids=8894) | eukaryotic translation initiation factor 2 subunit beta |
| [Details](http://mirdb.org/cgi-bin/target_detail.cgi?targetID=1231681) | 521 | 71 | hsa-let-7d-5p | [DICER1](http://www.ncbi.nlm.nih.gov/entrez/query.fcgi?db=gene&cmd=Retrieve&dopt=full_report&list_uids=23405) | dicer 1, ribonuclease III |
| [Details](http://mirdb.org/cgi-bin/target_detail.cgi?targetID=1232183) | 522 | 71 | hsa-let-7d-5p | [KCTD16](http://www.ncbi.nlm.nih.gov/entrez/query.fcgi?db=gene&cmd=Retrieve&dopt=full_report&list_uids=57528) | potassium channel tetramerization domain containing 16 |
| [Details](http://mirdb.org/cgi-bin/target_detail.cgi?targetID=1231624) | 523 | 71 | hsa-let-7d-5p | [GDPD1](http://www.ncbi.nlm.nih.gov/entrez/query.fcgi?db=gene&cmd=Retrieve&dopt=full_report&list_uids=284161) | glycerophosphodiester phosphodiesterase domain containing 1 |
| [Details](http://mirdb.org/cgi-bin/target_detail.cgi?targetID=1231801) | 524 | 71 | hsa-let-7d-5p | [UTY](http://www.ncbi.nlm.nih.gov/entrez/query.fcgi?db=gene&cmd=Retrieve&dopt=full_report&list_uids=7404) | ubiquitously transcribed tetratricopeptide repeat containing, Y-linked |
| [Details](http://mirdb.org/cgi-bin/target_detail.cgi?targetID=1232009) | 525 | 71 | hsa-let-7d-5p | [KLHL13](http://www.ncbi.nlm.nih.gov/entrez/query.fcgi?db=gene&cmd=Retrieve&dopt=full_report&list_uids=90293) | kelch like family member 13 |
| [Details](http://mirdb.org/cgi-bin/target_detail.cgi?targetID=1232018) | 526 | 71 | hsa-let-7d-5p | [SSH1](http://www.ncbi.nlm.nih.gov/entrez/query.fcgi?db=gene&cmd=Retrieve&dopt=full_report&list_uids=54434) | slingshot protein phosphatase 1 |
| [Details](http://mirdb.org/cgi-bin/target_detail.cgi?targetID=1231754) | 527 | 71 | hsa-let-7d-5p | [KIF21B](http://www.ncbi.nlm.nih.gov/entrez/query.fcgi?db=gene&cmd=Retrieve&dopt=full_report&list_uids=23046) | kinesin family member 21B |
| [Details](http://mirdb.org/cgi-bin/target_detail.cgi?targetID=1231495) | 528 | 71 | hsa-let-7d-5p | [FBXO30](http://www.ncbi.nlm.nih.gov/entrez/query.fcgi?db=gene&cmd=Retrieve&dopt=full_report&list_uids=84085) | F-box protein 30 |
| [Details](http://mirdb.org/cgi-bin/target_detail.cgi?targetID=1232113) | 529 | 71 | hsa-let-7d-5p | [PPARGC1B](http://www.ncbi.nlm.nih.gov/entrez/query.fcgi?db=gene&cmd=Retrieve&dopt=full_report&list_uids=133522) | PPARG coactivator 1 beta |
| [Details](http://mirdb.org/cgi-bin/target_detail.cgi?targetID=1231635) | 530 | 71 | hsa-let-7d-5p | [PLEKHH1](http://www.ncbi.nlm.nih.gov/entrez/query.fcgi?db=gene&cmd=Retrieve&dopt=full_report&list_uids=57475) | pleckstrin homology, MyTH4 and FERM domain containing H1 |
| [Details](http://mirdb.org/cgi-bin/target_detail.cgi?targetID=1232114) | 531 | 71 | hsa-let-7d-5p | [PDGFB](http://www.ncbi.nlm.nih.gov/entrez/query.fcgi?db=gene&cmd=Retrieve&dopt=full_report&list_uids=5155) | platelet derived growth factor subunit B |
| [Details](http://mirdb.org/cgi-bin/target_detail.cgi?targetID=1231795) | 532 | 71 | hsa-let-7d-5p | [CGNL1](http://www.ncbi.nlm.nih.gov/entrez/query.fcgi?db=gene&cmd=Retrieve&dopt=full_report&list_uids=84952) | cingulin like 1 |
| [Details](http://mirdb.org/cgi-bin/target_detail.cgi?targetID=1232341) | 533 | 71 | hsa-let-7d-5p | [DDX31](http://www.ncbi.nlm.nih.gov/entrez/query.fcgi?db=gene&cmd=Retrieve&dopt=full_report&list_uids=64794) | DEAD-box helicase 31 |
| [Details](http://mirdb.org/cgi-bin/target_detail.cgi?targetID=1231788) | 534 | 71 | hsa-let-7d-5p | [TSPAN18](http://www.ncbi.nlm.nih.gov/entrez/query.fcgi?db=gene&cmd=Retrieve&dopt=full_report&list_uids=90139) | tetraspanin 18 |
| [Details](http://mirdb.org/cgi-bin/target_detail.cgi?targetID=1231928) | 535 | 71 | hsa-let-7d-5p | [YPEL2](http://www.ncbi.nlm.nih.gov/entrez/query.fcgi?db=gene&cmd=Retrieve&dopt=full_report&list_uids=388403) | yippee like 2 |
| [Details](http://mirdb.org/cgi-bin/target_detail.cgi?targetID=1231981) | 536 | 71 | hsa-let-7d-5p | [MGAT4A](http://www.ncbi.nlm.nih.gov/entrez/query.fcgi?db=gene&cmd=Retrieve&dopt=full_report&list_uids=11320) | alpha-1,3-mannosyl-glycoprotein 4-beta-N-acetylglucosaminyltransferase A |
| [Details](http://mirdb.org/cgi-bin/target_detail.cgi?targetID=1231985) | 537 | 71 | hsa-let-7d-5p | [DMP1](http://www.ncbi.nlm.nih.gov/entrez/query.fcgi?db=gene&cmd=Retrieve&dopt=full_report&list_uids=1758) | dentin matrix acidic phosphoprotein 1 |
| [Details](http://mirdb.org/cgi-bin/target_detail.cgi?targetID=1232332) | 538 | 71 | hsa-let-7d-5p | [PPP2R2A](http://www.ncbi.nlm.nih.gov/entrez/query.fcgi?db=gene&cmd=Retrieve&dopt=full_report&list_uids=5520) | protein phosphatase 2 regulatory subunit Balpha |
| [Details](http://mirdb.org/cgi-bin/target_detail.cgi?targetID=1231666) | 539 | 71 | hsa-let-7d-5p | [GRAMD2B](http://www.ncbi.nlm.nih.gov/entrez/query.fcgi?db=gene&cmd=Retrieve&dopt=full_report&list_uids=65983) | GRAM domain containing 2B |
| [Details](http://mirdb.org/cgi-bin/target_detail.cgi?targetID=1231888) | 540 | 71 | hsa-let-7d-5p | [KCNQ4](http://www.ncbi.nlm.nih.gov/entrez/query.fcgi?db=gene&cmd=Retrieve&dopt=full_report&list_uids=9132) | potassium voltage-gated channel subfamily Q member 4 |
| [Details](http://mirdb.org/cgi-bin/target_detail.cgi?targetID=1231560) | 541 | 71 | hsa-let-7d-5p | [FBXO45](http://www.ncbi.nlm.nih.gov/entrez/query.fcgi?db=gene&cmd=Retrieve&dopt=full_report&list_uids=200933) | F-box protein 45 |
| [Details](http://mirdb.org/cgi-bin/target_detail.cgi?targetID=1231810) | 542 | 71 | hsa-let-7d-5p | [MXD1](http://www.ncbi.nlm.nih.gov/entrez/query.fcgi?db=gene&cmd=Retrieve&dopt=full_report&list_uids=4084) | MAX dimerization protein 1 |
| [Details](http://mirdb.org/cgi-bin/target_detail.cgi?targetID=1231883) | 543 | 70 | hsa-let-7d-5p | [FMO4](http://www.ncbi.nlm.nih.gov/entrez/query.fcgi?db=gene&cmd=Retrieve&dopt=full_report&list_uids=2329) | flavin containing monooxygenase 4 |
| [Details](http://mirdb.org/cgi-bin/target_detail.cgi?targetID=1232245) | 544 | 70 | hsa-let-7d-5p | [PRDM5](http://www.ncbi.nlm.nih.gov/entrez/query.fcgi?db=gene&cmd=Retrieve&dopt=full_report&list_uids=11107) | PR/SET domain 5 |
| [Details](http://mirdb.org/cgi-bin/target_detail.cgi?targetID=1231632) | 545 | 70 | hsa-let-7d-5p | [CYP4F2](http://www.ncbi.nlm.nih.gov/entrez/query.fcgi?db=gene&cmd=Retrieve&dopt=full_report&list_uids=8529) | cytochrome P450 family 4 subfamily F member 2 |
| [Details](http://mirdb.org/cgi-bin/target_detail.cgi?targetID=1231695) | 546 | 70 | hsa-let-7d-5p | [MIEF1](http://www.ncbi.nlm.nih.gov/entrez/query.fcgi?db=gene&cmd=Retrieve&dopt=full_report&list_uids=54471) | mitochondrial elongation factor 1 |
| [Details](http://mirdb.org/cgi-bin/target_detail.cgi?targetID=1232053) | 547 | 70 | hsa-let-7d-5p | [MYRIP](http://www.ncbi.nlm.nih.gov/entrez/query.fcgi?db=gene&cmd=Retrieve&dopt=full_report&list_uids=25924) | myosin VIIA and Rab interacting protein |
| [Details](http://mirdb.org/cgi-bin/target_detail.cgi?targetID=1231910) | 548 | 70 | hsa-let-7d-5p | [PACS2](http://www.ncbi.nlm.nih.gov/entrez/query.fcgi?db=gene&cmd=Retrieve&dopt=full_report&list_uids=23241) | phosphofurin acidic cluster sorting protein 2 |
| [Details](http://mirdb.org/cgi-bin/target_detail.cgi?targetID=1231802) | 549 | 70 | hsa-let-7d-5p | [RAB15](http://www.ncbi.nlm.nih.gov/entrez/query.fcgi?db=gene&cmd=Retrieve&dopt=full_report&list_uids=376267) | RAB15, member RAS oncogene family |
| [Details](http://mirdb.org/cgi-bin/target_detail.cgi?targetID=1231508) | 550 | 70 | hsa-let-7d-5p | [ZDHHC23](http://www.ncbi.nlm.nih.gov/entrez/query.fcgi?db=gene&cmd=Retrieve&dopt=full_report&list_uids=254887) | zinc finger DHHC-type containing 23 |
| [Details](http://mirdb.org/cgi-bin/target_detail.cgi?targetID=1231756) | 551 | 70 | hsa-let-7d-5p | [C2](http://www.ncbi.nlm.nih.gov/entrez/query.fcgi?db=gene&cmd=Retrieve&dopt=full_report&list_uids=717) | complement C2 |
| [Details](http://mirdb.org/cgi-bin/target_detail.cgi?targetID=1231765) | 552 | 70 | hsa-let-7d-5p | [ADGRL3](http://www.ncbi.nlm.nih.gov/entrez/query.fcgi?db=gene&cmd=Retrieve&dopt=full_report&list_uids=23284) | adhesion G protein-coupled receptor L3 |
| [Details](http://mirdb.org/cgi-bin/target_detail.cgi?targetID=1231553) | 553 | 70 | hsa-let-7d-5p | [RPUSD2](http://www.ncbi.nlm.nih.gov/entrez/query.fcgi?db=gene&cmd=Retrieve&dopt=full_report&list_uids=27079) | RNA pseudouridine synthase domain containing 2 |
| [Details](http://mirdb.org/cgi-bin/target_detail.cgi?targetID=1231848) | 554 | 69 | hsa-let-7d-5p | [USP12](http://www.ncbi.nlm.nih.gov/entrez/query.fcgi?db=gene&cmd=Retrieve&dopt=full_report&list_uids=219333) | ubiquitin specific peptidase 12 |
| [Details](http://mirdb.org/cgi-bin/target_detail.cgi?targetID=1232065) | 555 | 69 | hsa-let-7d-5p | [SOX13](http://www.ncbi.nlm.nih.gov/entrez/query.fcgi?db=gene&cmd=Retrieve&dopt=full_report&list_uids=9580) | SRY-box 13 |
| [Details](http://mirdb.org/cgi-bin/target_detail.cgi?targetID=1231759) | 556 | 69 | hsa-let-7d-5p | [ANKFY1](http://www.ncbi.nlm.nih.gov/entrez/query.fcgi?db=gene&cmd=Retrieve&dopt=full_report&list_uids=51479) | ankyrin repeat and FYVE domain containing 1 |
| [Details](http://mirdb.org/cgi-bin/target_detail.cgi?targetID=1232063) | 557 | 69 | hsa-let-7d-5p | [LRTOMT](http://www.ncbi.nlm.nih.gov/entrez/query.fcgi?db=gene&cmd=Retrieve&dopt=full_report&list_uids=220074) | leucine rich transmembrane and O-methyltransferase domain containing |
| [Details](http://mirdb.org/cgi-bin/target_detail.cgi?targetID=1232391) | 558 | 69 | hsa-let-7d-5p | [ZNF341](http://www.ncbi.nlm.nih.gov/entrez/query.fcgi?db=gene&cmd=Retrieve&dopt=full_report&list_uids=84905) | zinc finger protein 341 |
| [Details](http://mirdb.org/cgi-bin/target_detail.cgi?targetID=1232038) | 559 | 69 | hsa-let-7d-5p | [MECP2](http://www.ncbi.nlm.nih.gov/entrez/query.fcgi?db=gene&cmd=Retrieve&dopt=full_report&list_uids=4204) | methyl-CpG binding protein 2 |
| [Details](http://mirdb.org/cgi-bin/target_detail.cgi?targetID=1231700) | 560 | 69 | hsa-let-7d-5p | [CANT1](http://www.ncbi.nlm.nih.gov/entrez/query.fcgi?db=gene&cmd=Retrieve&dopt=full_report&list_uids=124583) | calcium activated nucleotidase 1 |
| [Details](http://mirdb.org/cgi-bin/target_detail.cgi?targetID=1232337) | 561 | 69 | hsa-let-7d-5p | [INTS2](http://www.ncbi.nlm.nih.gov/entrez/query.fcgi?db=gene&cmd=Retrieve&dopt=full_report&list_uids=57508) | integrator complex subunit 2 |
| [Details](http://mirdb.org/cgi-bin/target_detail.cgi?targetID=1232340) | 562 | 69 | hsa-let-7d-5p | [SLC1A4](http://www.ncbi.nlm.nih.gov/entrez/query.fcgi?db=gene&cmd=Retrieve&dopt=full_report&list_uids=6509) | solute carrier family 1 member 4 |
| [Details](http://mirdb.org/cgi-bin/target_detail.cgi?targetID=1231811) | 563 | 69 | hsa-let-7d-5p | [CBX2](http://www.ncbi.nlm.nih.gov/entrez/query.fcgi?db=gene&cmd=Retrieve&dopt=full_report&list_uids=84733) | chromobox 2 |
| [Details](http://mirdb.org/cgi-bin/target_detail.cgi?targetID=1231905) | 564 | 69 | hsa-let-7d-5p | [KATNBL1](http://www.ncbi.nlm.nih.gov/entrez/query.fcgi?db=gene&cmd=Retrieve&dopt=full_report&list_uids=79768) | katanin regulatory subunit B1 like 1 |
| [Details](http://mirdb.org/cgi-bin/target_detail.cgi?targetID=1231527) | 565 | 69 | hsa-let-7d-5p | [ERGIC1](http://www.ncbi.nlm.nih.gov/entrez/query.fcgi?db=gene&cmd=Retrieve&dopt=full_report&list_uids=57222) | endoplasmic reticulum-golgi intermediate compartment 1 |
| [Details](http://mirdb.org/cgi-bin/target_detail.cgi?targetID=1232010) | 566 | 69 | hsa-let-7d-5p | [SLC12A9](http://www.ncbi.nlm.nih.gov/entrez/query.fcgi?db=gene&cmd=Retrieve&dopt=full_report&list_uids=56996) | solute carrier family 12 member 9 |
| [Details](http://mirdb.org/cgi-bin/target_detail.cgi?targetID=1231698) | 567 | 68 | hsa-let-7d-5p | [SOWAHA](http://www.ncbi.nlm.nih.gov/entrez/query.fcgi?db=gene&cmd=Retrieve&dopt=full_report&list_uids=134548) | sosondowah ankyrin repeat domain family member A |
| [Details](http://mirdb.org/cgi-bin/target_detail.cgi?targetID=1232138) | 568 | 68 | hsa-let-7d-5p | [SESN3](http://www.ncbi.nlm.nih.gov/entrez/query.fcgi?db=gene&cmd=Retrieve&dopt=full_report&list_uids=143686) | sestrin 3 |
| [Details](http://mirdb.org/cgi-bin/target_detail.cgi?targetID=1232008) | 569 | 68 | hsa-let-7d-5p | [TXNDC8](http://www.ncbi.nlm.nih.gov/entrez/query.fcgi?db=gene&cmd=Retrieve&dopt=full_report&list_uids=255220) | thioredoxin domain containing 8 |
| [Details](http://mirdb.org/cgi-bin/target_detail.cgi?targetID=1231564) | 570 | 68 | hsa-let-7d-5p | [MSI2](http://www.ncbi.nlm.nih.gov/entrez/query.fcgi?db=gene&cmd=Retrieve&dopt=full_report&list_uids=124540) | musashi RNA binding protein 2 |
| [Details](http://mirdb.org/cgi-bin/target_detail.cgi?targetID=1231746) | 571 | 68 | hsa-let-7d-5p | [USP49](http://www.ncbi.nlm.nih.gov/entrez/query.fcgi?db=gene&cmd=Retrieve&dopt=full_report&list_uids=25862) | ubiquitin specific peptidase 49 |
| [Details](http://mirdb.org/cgi-bin/target_detail.cgi?targetID=1231860) | 572 | 68 | hsa-let-7d-5p | [RNF170](http://www.ncbi.nlm.nih.gov/entrez/query.fcgi?db=gene&cmd=Retrieve&dopt=full_report&list_uids=81790) | ring finger protein 170 |
| [Details](http://mirdb.org/cgi-bin/target_detail.cgi?targetID=1231729) | 573 | 68 | hsa-let-7d-5p | [ITGB8](http://www.ncbi.nlm.nih.gov/entrez/query.fcgi?db=gene&cmd=Retrieve&dopt=full_report&list_uids=3696) | integrin subunit beta 8 |
| [Details](http://mirdb.org/cgi-bin/target_detail.cgi?targetID=1232025) | 574 | 68 | hsa-let-7d-5p | [OLR1](http://www.ncbi.nlm.nih.gov/entrez/query.fcgi?db=gene&cmd=Retrieve&dopt=full_report&list_uids=4973) | oxidized low density lipoprotein receptor 1 |
| [Details](http://mirdb.org/cgi-bin/target_detail.cgi?targetID=1232347) | 575 | 68 | hsa-let-7d-5p | [AURKB](http://www.ncbi.nlm.nih.gov/entrez/query.fcgi?db=gene&cmd=Retrieve&dopt=full_report&list_uids=9212) | aurora kinase B |
| [Details](http://mirdb.org/cgi-bin/target_detail.cgi?targetID=1232108) | 576 | 68 | hsa-let-7d-5p | [AK3](http://www.ncbi.nlm.nih.gov/entrez/query.fcgi?db=gene&cmd=Retrieve&dopt=full_report&list_uids=50808) | adenylate kinase 3 |
| [Details](http://mirdb.org/cgi-bin/target_detail.cgi?targetID=1232229) | 577 | 68 | hsa-let-7d-5p | [ARMC8](http://www.ncbi.nlm.nih.gov/entrez/query.fcgi?db=gene&cmd=Retrieve&dopt=full_report&list_uids=25852) | armadillo repeat containing 8 |
| [Details](http://mirdb.org/cgi-bin/target_detail.cgi?targetID=1231959) | 578 | 67 | hsa-let-7d-5p | [HIF3A](http://www.ncbi.nlm.nih.gov/entrez/query.fcgi?db=gene&cmd=Retrieve&dopt=full_report&list_uids=64344) | hypoxia inducible factor 3 subunit alpha |
| [Details](http://mirdb.org/cgi-bin/target_detail.cgi?targetID=1231834) | 579 | 67 | hsa-let-7d-5p | [CHIC1](http://www.ncbi.nlm.nih.gov/entrez/query.fcgi?db=gene&cmd=Retrieve&dopt=full_report&list_uids=53344) | cysteine rich hydrophobic domain 1 |
| [Details](http://mirdb.org/cgi-bin/target_detail.cgi?targetID=1232146) | 580 | 67 | hsa-let-7d-5p | [SLC31A1](http://www.ncbi.nlm.nih.gov/entrez/query.fcgi?db=gene&cmd=Retrieve&dopt=full_report&list_uids=1317) | solute carrier family 31 member 1 |
| [Details](http://mirdb.org/cgi-bin/target_detail.cgi?targetID=1232360) | 581 | 67 | hsa-let-7d-5p | [EPHA3](http://www.ncbi.nlm.nih.gov/entrez/query.fcgi?db=gene&cmd=Retrieve&dopt=full_report&list_uids=2042) | EPH receptor A3 |
| [Details](http://mirdb.org/cgi-bin/target_detail.cgi?targetID=1231840) | 582 | 67 | hsa-let-7d-5p | [CLDN1](http://www.ncbi.nlm.nih.gov/entrez/query.fcgi?db=gene&cmd=Retrieve&dopt=full_report&list_uids=9076) | claudin 1 |
| [Details](http://mirdb.org/cgi-bin/target_detail.cgi?targetID=1231796) | 583 | 67 | hsa-let-7d-5p | [SPPL2B](http://www.ncbi.nlm.nih.gov/entrez/query.fcgi?db=gene&cmd=Retrieve&dopt=full_report&list_uids=56928) | signal peptide peptidase like 2B |
| [Details](http://mirdb.org/cgi-bin/target_detail.cgi?targetID=1232069) | 584 | 67 | hsa-let-7d-5p | [SAMD12](http://www.ncbi.nlm.nih.gov/entrez/query.fcgi?db=gene&cmd=Retrieve&dopt=full_report&list_uids=401474) | sterile alpha motif domain containing 12 |
| [Details](http://mirdb.org/cgi-bin/target_detail.cgi?targetID=1231775) | 585 | 67 | hsa-let-7d-5p | [APBA1](http://www.ncbi.nlm.nih.gov/entrez/query.fcgi?db=gene&cmd=Retrieve&dopt=full_report&list_uids=320) | amyloid beta precursor protein binding family A member 1 |
| [Details](http://mirdb.org/cgi-bin/target_detail.cgi?targetID=1232079) | 586 | 67 | hsa-let-7d-5p | [SREBF2](http://www.ncbi.nlm.nih.gov/entrez/query.fcgi?db=gene&cmd=Retrieve&dopt=full_report&list_uids=6721) | sterol regulatory element binding transcription factor 2 |
| [Details](http://mirdb.org/cgi-bin/target_detail.cgi?targetID=1231975) | 587 | 67 | hsa-let-7d-5p | [ZC3H3](http://www.ncbi.nlm.nih.gov/entrez/query.fcgi?db=gene&cmd=Retrieve&dopt=full_report&list_uids=23144) | zinc finger CCCH-type containing 3 |
| [Details](http://mirdb.org/cgi-bin/target_detail.cgi?targetID=1231721) | 588 | 67 | hsa-let-7d-5p | [GALE](http://www.ncbi.nlm.nih.gov/entrez/query.fcgi?db=gene&cmd=Retrieve&dopt=full_report&list_uids=2582) | UDP-galactose-4-epimerase |
| [Details](http://mirdb.org/cgi-bin/target_detail.cgi?targetID=1231636) | 589 | 67 | hsa-let-7d-5p | [SLC9A9](http://www.ncbi.nlm.nih.gov/entrez/query.fcgi?db=gene&cmd=Retrieve&dopt=full_report&list_uids=285195) | solute carrier family 9 member A9 |
| [Details](http://mirdb.org/cgi-bin/target_detail.cgi?targetID=1232290) | 590 | 67 | hsa-let-7d-5p | [MAPK9](http://www.ncbi.nlm.nih.gov/entrez/query.fcgi?db=gene&cmd=Retrieve&dopt=full_report&list_uids=5601) | mitogen-activated protein kinase 9 |
| [Details](http://mirdb.org/cgi-bin/target_detail.cgi?targetID=1232227) | 591 | 67 | hsa-let-7d-5p | [C1GALT1](http://www.ncbi.nlm.nih.gov/entrez/query.fcgi?db=gene&cmd=Retrieve&dopt=full_report&list_uids=56913) | core 1 synthase, glycoprotein-N-acetylgalactosamine 3-beta-galactosyltransferase 1 |
| [Details](http://mirdb.org/cgi-bin/target_detail.cgi?targetID=1231866) | 592 | 66 | hsa-let-7d-5p | [CCDC141](http://www.ncbi.nlm.nih.gov/entrez/query.fcgi?db=gene&cmd=Retrieve&dopt=full_report&list_uids=285025) | coiled-coil domain containing 141 |
| [Details](http://mirdb.org/cgi-bin/target_detail.cgi?targetID=1231669) | 593 | 66 | hsa-let-7d-5p | [SPRYD7](http://www.ncbi.nlm.nih.gov/entrez/query.fcgi?db=gene&cmd=Retrieve&dopt=full_report&list_uids=57213) | SPRY domain containing 7 |
| [Details](http://mirdb.org/cgi-bin/target_detail.cgi?targetID=1231582) | 594 | 66 | hsa-let-7d-5p | [LSM11](http://www.ncbi.nlm.nih.gov/entrez/query.fcgi?db=gene&cmd=Retrieve&dopt=full_report&list_uids=134353) | LSM11, U7 small nuclear RNA associated |
| [Details](http://mirdb.org/cgi-bin/target_detail.cgi?targetID=1231612) | 595 | 66 | hsa-let-7d-5p | [CALU](http://www.ncbi.nlm.nih.gov/entrez/query.fcgi?db=gene&cmd=Retrieve&dopt=full_report&list_uids=813) | calumenin |
| [Details](http://mirdb.org/cgi-bin/target_detail.cgi?targetID=1232161) | 596 | 66 | hsa-let-7d-5p | [WDFY3](http://www.ncbi.nlm.nih.gov/entrez/query.fcgi?db=gene&cmd=Retrieve&dopt=full_report&list_uids=23001) | WD repeat and FYVE domain containing 3 |
| [Details](http://mirdb.org/cgi-bin/target_detail.cgi?targetID=1231509) | 597 | 66 | hsa-let-7d-5p | [BCAP29](http://www.ncbi.nlm.nih.gov/entrez/query.fcgi?db=gene&cmd=Retrieve&dopt=full_report&list_uids=55973) | B cell receptor associated protein 29 |
| [Details](http://mirdb.org/cgi-bin/target_detail.cgi?targetID=1231644) | 598 | 66 | hsa-let-7d-5p | [NUGGC](http://www.ncbi.nlm.nih.gov/entrez/query.fcgi?db=gene&cmd=Retrieve&dopt=full_report&list_uids=389643) | nuclear GTPase, germinal center associated |
| [Details](http://mirdb.org/cgi-bin/target_detail.cgi?targetID=1231667) | 599 | 66 | hsa-let-7d-5p | [PKD1L2](http://www.ncbi.nlm.nih.gov/entrez/query.fcgi?db=gene&cmd=Retrieve&dopt=full_report&list_uids=114780) | polycystin 1 like 2 (gene/pseudogene) |
| [Details](http://mirdb.org/cgi-bin/target_detail.cgi?targetID=1231949) | 600 | 66 | hsa-let-7d-5p | [GOLGA6L4](http://www.ncbi.nlm.nih.gov/entrez/query.fcgi?db=gene&cmd=Retrieve&dopt=full_report&list_uids=643707) | golgin A6 family-like 4 |
| [Details](http://mirdb.org/cgi-bin/target_detail.cgi?targetID=1231887) | 601 | 66 | hsa-let-7d-5p | [STK24](http://www.ncbi.nlm.nih.gov/entrez/query.fcgi?db=gene&cmd=Retrieve&dopt=full_report&list_uids=8428) | serine/threonine kinase 24 |
| [Details](http://mirdb.org/cgi-bin/target_detail.cgi?targetID=1231832) | 602 | 66 | hsa-let-7d-5p | [CEP63](http://www.ncbi.nlm.nih.gov/entrez/query.fcgi?db=gene&cmd=Retrieve&dopt=full_report&list_uids=80254) | centrosomal protein 63 |
| [Details](http://mirdb.org/cgi-bin/target_detail.cgi?targetID=1231699) | 603 | 66 | hsa-let-7d-5p | [TAB2](http://www.ncbi.nlm.nih.gov/entrez/query.fcgi?db=gene&cmd=Retrieve&dopt=full_report&list_uids=23118) | TGF-beta activated kinase 1 (MAP3K7) binding protein 2 |
| [Details](http://mirdb.org/cgi-bin/target_detail.cgi?targetID=1231499) | 604 | 66 | hsa-let-7d-5p | [ETNK2](http://www.ncbi.nlm.nih.gov/entrez/query.fcgi?db=gene&cmd=Retrieve&dopt=full_report&list_uids=55224) | ethanolamine kinase 2 |
| [Details](http://mirdb.org/cgi-bin/target_detail.cgi?targetID=1231740) | 605 | 66 | hsa-let-7d-5p | [SH3RF3](http://www.ncbi.nlm.nih.gov/entrez/query.fcgi?db=gene&cmd=Retrieve&dopt=full_report&list_uids=344558) | SH3 domain containing ring finger 3 |
| [Details](http://mirdb.org/cgi-bin/target_detail.cgi?targetID=1231569) | 606 | 66 | hsa-let-7d-5p | [PARS2](http://www.ncbi.nlm.nih.gov/entrez/query.fcgi?db=gene&cmd=Retrieve&dopt=full_report&list_uids=25973) | prolyl-tRNA synthetase 2, mitochondrial |
| [Details](http://mirdb.org/cgi-bin/target_detail.cgi?targetID=1231904) | 607 | 66 | hsa-let-7d-5p | [ZNF641](http://www.ncbi.nlm.nih.gov/entrez/query.fcgi?db=gene&cmd=Retrieve&dopt=full_report&list_uids=121274) | zinc finger protein 641 |
| [Details](http://mirdb.org/cgi-bin/target_detail.cgi?targetID=1232311) | 608 | 66 | hsa-let-7d-5p | [ZNF512](http://www.ncbi.nlm.nih.gov/entrez/query.fcgi?db=gene&cmd=Retrieve&dopt=full_report&list_uids=84450) | zinc finger protein 512 |
| [Details](http://mirdb.org/cgi-bin/target_detail.cgi?targetID=1231555) | 609 | 66 | hsa-let-7d-5p | [WDR41](http://www.ncbi.nlm.nih.gov/entrez/query.fcgi?db=gene&cmd=Retrieve&dopt=full_report&list_uids=55255) | WD repeat domain 41 |
| [Details](http://mirdb.org/cgi-bin/target_detail.cgi?targetID=1231545) | 610 | 66 | hsa-let-7d-5p | [NDST2](http://www.ncbi.nlm.nih.gov/entrez/query.fcgi?db=gene&cmd=Retrieve&dopt=full_report&list_uids=8509) | N-deacetylase and N-sulfotransferase 2 |
| [Details](http://mirdb.org/cgi-bin/target_detail.cgi?targetID=1232188) | 611 | 66 | hsa-let-7d-5p | [LMLN](http://www.ncbi.nlm.nih.gov/entrez/query.fcgi?db=gene&cmd=Retrieve&dopt=full_report&list_uids=89782) | leishmanolysin like peptidase |
| [Details](http://mirdb.org/cgi-bin/target_detail.cgi?targetID=1232066) | 612 | 65 | hsa-let-7d-5p | [ATAD2B](http://www.ncbi.nlm.nih.gov/entrez/query.fcgi?db=gene&cmd=Retrieve&dopt=full_report&list_uids=54454) | ATPase family, AAA domain containing 2B |
| [Details](http://mirdb.org/cgi-bin/target_detail.cgi?targetID=1231574) | 613 | 65 | hsa-let-7d-5p | [SURF4](http://www.ncbi.nlm.nih.gov/entrez/query.fcgi?db=gene&cmd=Retrieve&dopt=full_report&list_uids=6836) | surfeit 4 |
| [Details](http://mirdb.org/cgi-bin/target_detail.cgi?targetID=1231782) | 614 | 65 | hsa-let-7d-5p | [STRN](http://www.ncbi.nlm.nih.gov/entrez/query.fcgi?db=gene&cmd=Retrieve&dopt=full_report&list_uids=6801) | striatin |
| [Details](http://mirdb.org/cgi-bin/target_detail.cgi?targetID=1231539) | 615 | 65 | hsa-let-7d-5p | [SMCR8](http://www.ncbi.nlm.nih.gov/entrez/query.fcgi?db=gene&cmd=Retrieve&dopt=full_report&list_uids=140775) | Smith-Magenis syndrome chromosome region, candidate 8 |
| [Details](http://mirdb.org/cgi-bin/target_detail.cgi?targetID=1232133) | 616 | 65 | hsa-let-7d-5p | [TGDS](http://www.ncbi.nlm.nih.gov/entrez/query.fcgi?db=gene&cmd=Retrieve&dopt=full_report&list_uids=23483) | TDP-glucose 4,6-dehydratase |
| [Details](http://mirdb.org/cgi-bin/target_detail.cgi?targetID=1231482) | 617 | 65 | hsa-let-7d-5p | [POLR2D](http://www.ncbi.nlm.nih.gov/entrez/query.fcgi?db=gene&cmd=Retrieve&dopt=full_report&list_uids=5433) | RNA polymerase II subunit D |
| [Details](http://mirdb.org/cgi-bin/target_detail.cgi?targetID=1231686) | 618 | 65 | hsa-let-7d-5p | [SPATA2](http://www.ncbi.nlm.nih.gov/entrez/query.fcgi?db=gene&cmd=Retrieve&dopt=full_report&list_uids=9825) | spermatogenesis associated 2 |
| [Details](http://mirdb.org/cgi-bin/target_detail.cgi?targetID=1232318) | 619 | 65 | hsa-let-7d-5p | [KRTAP5-8](http://www.ncbi.nlm.nih.gov/entrez/query.fcgi?db=gene&cmd=Retrieve&dopt=full_report&list_uids=57830) | keratin associated protein 5-8 |
| [Details](http://mirdb.org/cgi-bin/target_detail.cgi?targetID=1231606) | 620 | 65 | hsa-let-7d-5p | [PLD5](http://www.ncbi.nlm.nih.gov/entrez/query.fcgi?db=gene&cmd=Retrieve&dopt=full_report&list_uids=200150) | phospholipase D family member 5 |
| [Details](http://mirdb.org/cgi-bin/target_detail.cgi?targetID=1231763) | 621 | 65 | hsa-let-7d-5p | [CRB2](http://www.ncbi.nlm.nih.gov/entrez/query.fcgi?db=gene&cmd=Retrieve&dopt=full_report&list_uids=286204) | crumbs cell polarity complex component 2 |
| [Details](http://mirdb.org/cgi-bin/target_detail.cgi?targetID=1232272) | 622 | 65 | hsa-let-7d-5p | [AGO3](http://www.ncbi.nlm.nih.gov/entrez/query.fcgi?db=gene&cmd=Retrieve&dopt=full_report&list_uids=192669) | argonaute RISC catalytic component 3 |
| [Details](http://mirdb.org/cgi-bin/target_detail.cgi?targetID=1231797) | 623 | 65 | hsa-let-7d-5p | [ADAMTS5](http://www.ncbi.nlm.nih.gov/entrez/query.fcgi?db=gene&cmd=Retrieve&dopt=full_report&list_uids=11096) | ADAM metallopeptidase with thrombospondin type 1 motif 5 |
| [Details](http://mirdb.org/cgi-bin/target_detail.cgi?targetID=1232213) | 624 | 65 | hsa-let-7d-5p | [KIAA1549](http://www.ncbi.nlm.nih.gov/entrez/query.fcgi?db=gene&cmd=Retrieve&dopt=full_report&list_uids=57670) | KIAA1549 |
| [Details](http://mirdb.org/cgi-bin/target_detail.cgi?targetID=1231988) | 625 | 65 | hsa-let-7d-5p | [TMEM143](http://www.ncbi.nlm.nih.gov/entrez/query.fcgi?db=gene&cmd=Retrieve&dopt=full_report&list_uids=55260) | transmembrane protein 143 |
| [Details](http://mirdb.org/cgi-bin/target_detail.cgi?targetID=1231890) | 626 | 65 | hsa-let-7d-5p | [MEIS3](http://www.ncbi.nlm.nih.gov/entrez/query.fcgi?db=gene&cmd=Retrieve&dopt=full_report&list_uids=56917) | Meis homeobox 3 |
| [Details](http://mirdb.org/cgi-bin/target_detail.cgi?targetID=1231963) | 627 | 65 | hsa-let-7d-5p | [STX17](http://www.ncbi.nlm.nih.gov/entrez/query.fcgi?db=gene&cmd=Retrieve&dopt=full_report&list_uids=55014) | syntaxin 17 |
| [Details](http://mirdb.org/cgi-bin/target_detail.cgi?targetID=1231976) | 628 | 65 | hsa-let-7d-5p | [KMT2D](http://www.ncbi.nlm.nih.gov/entrez/query.fcgi?db=gene&cmd=Retrieve&dopt=full_report&list_uids=8085) | lysine methyltransferase 2D |
| [Details](http://mirdb.org/cgi-bin/target_detail.cgi?targetID=1231841) | 629 | 65 | hsa-let-7d-5p | [FARP1](http://www.ncbi.nlm.nih.gov/entrez/query.fcgi?db=gene&cmd=Retrieve&dopt=full_report&list_uids=10160) | FERM, ARH/RhoGEF and pleckstrin domain protein 1 |
| [Details](http://mirdb.org/cgi-bin/target_detail.cgi?targetID=1232228) | 630 | 64 | hsa-let-7d-5p | [LIMK2](http://www.ncbi.nlm.nih.gov/entrez/query.fcgi?db=gene&cmd=Retrieve&dopt=full_report&list_uids=3985) | LIM domain kinase 2 |
| [Details](http://mirdb.org/cgi-bin/target_detail.cgi?targetID=1231967) | 631 | 64 | hsa-let-7d-5p | [KPNA1](http://www.ncbi.nlm.nih.gov/entrez/query.fcgi?db=gene&cmd=Retrieve&dopt=full_report&list_uids=3836) | karyopherin subunit alpha 1 |
| [Details](http://mirdb.org/cgi-bin/target_detail.cgi?targetID=1231984) | 632 | 64 | hsa-let-7d-5p | [SYT7](http://www.ncbi.nlm.nih.gov/entrez/query.fcgi?db=gene&cmd=Retrieve&dopt=full_report&list_uids=9066) | synaptotagmin 7 |
| [Details](http://mirdb.org/cgi-bin/target_detail.cgi?targetID=1231945) | 633 | 64 | hsa-let-7d-5p | [RAB40C](http://www.ncbi.nlm.nih.gov/entrez/query.fcgi?db=gene&cmd=Retrieve&dopt=full_report&list_uids=57799) | RAB40C, member RAS oncogene family |
| [Details](http://mirdb.org/cgi-bin/target_detail.cgi?targetID=1231977) | 634 | 64 | hsa-let-7d-5p | [NRARP](http://www.ncbi.nlm.nih.gov/entrez/query.fcgi?db=gene&cmd=Retrieve&dopt=full_report&list_uids=441478) | NOTCH regulated ankyrin repeat protein |
| [Details](http://mirdb.org/cgi-bin/target_detail.cgi?targetID=1232336) | 635 | 64 | hsa-let-7d-5p | [LHCGR](http://www.ncbi.nlm.nih.gov/entrez/query.fcgi?db=gene&cmd=Retrieve&dopt=full_report&list_uids=3973) | luteinizing hormone/choriogonadotropin receptor |
| [Details](http://mirdb.org/cgi-bin/target_detail.cgi?targetID=1231762) | 636 | 64 | hsa-let-7d-5p | [UBE2G2](http://www.ncbi.nlm.nih.gov/entrez/query.fcgi?db=gene&cmd=Retrieve&dopt=full_report&list_uids=7327) | ubiquitin conjugating enzyme E2 G2 |
| [Details](http://mirdb.org/cgi-bin/target_detail.cgi?targetID=1232068) | 637 | 64 | hsa-let-7d-5p | [SOCS1](http://www.ncbi.nlm.nih.gov/entrez/query.fcgi?db=gene&cmd=Retrieve&dopt=full_report&list_uids=8651) | suppressor of cytokine signaling 1 |
| [Details](http://mirdb.org/cgi-bin/target_detail.cgi?targetID=1231517) | 638 | 64 | hsa-let-7d-5p | [RBM19](http://www.ncbi.nlm.nih.gov/entrez/query.fcgi?db=gene&cmd=Retrieve&dopt=full_report&list_uids=9904) | RNA binding motif protein 19 |
| [Details](http://mirdb.org/cgi-bin/target_detail.cgi?targetID=1231934) | 639 | 64 | hsa-let-7d-5p | [TUSC2](http://www.ncbi.nlm.nih.gov/entrez/query.fcgi?db=gene&cmd=Retrieve&dopt=full_report&list_uids=11334) | tumor suppressor 2, mitochondrial calcium regulator |
| [Details](http://mirdb.org/cgi-bin/target_detail.cgi?targetID=1232107) | 640 | 64 | hsa-let-7d-5p | [ZNF566](http://www.ncbi.nlm.nih.gov/entrez/query.fcgi?db=gene&cmd=Retrieve&dopt=full_report&list_uids=84924) | zinc finger protein 566 |
| [Details](http://mirdb.org/cgi-bin/target_detail.cgi?targetID=1232247) | 641 | 64 | hsa-let-7d-5p | [LIMD1](http://www.ncbi.nlm.nih.gov/entrez/query.fcgi?db=gene&cmd=Retrieve&dopt=full_report&list_uids=8994) | LIM domains containing 1 |
| [Details](http://mirdb.org/cgi-bin/target_detail.cgi?targetID=1231805) | 642 | 64 | hsa-let-7d-5p | [ZNF354A](http://www.ncbi.nlm.nih.gov/entrez/query.fcgi?db=gene&cmd=Retrieve&dopt=full_report&list_uids=6940) | zinc finger protein 354A |
| [Details](http://mirdb.org/cgi-bin/target_detail.cgi?targetID=1231713) | 643 | 64 | hsa-let-7d-5p | [AIFM1](http://www.ncbi.nlm.nih.gov/entrez/query.fcgi?db=gene&cmd=Retrieve&dopt=full_report&list_uids=9131) | apoptosis inducing factor mitochondria associated 1 |
| [Details](http://mirdb.org/cgi-bin/target_detail.cgi?targetID=1232000) | 644 | 63 | hsa-let-7d-5p | [TMX4](http://www.ncbi.nlm.nih.gov/entrez/query.fcgi?db=gene&cmd=Retrieve&dopt=full_report&list_uids=56255) | thioredoxin related transmembrane protein 4 |
| [Details](http://mirdb.org/cgi-bin/target_detail.cgi?targetID=1232291) | 645 | 63 | hsa-let-7d-5p | [LOXL4](http://www.ncbi.nlm.nih.gov/entrez/query.fcgi?db=gene&cmd=Retrieve&dopt=full_report&list_uids=84171) | lysyl oxidase like 4 |
| [Details](http://mirdb.org/cgi-bin/target_detail.cgi?targetID=1231597) | 646 | 63 | hsa-let-7d-5p | [MFSD8](http://www.ncbi.nlm.nih.gov/entrez/query.fcgi?db=gene&cmd=Retrieve&dopt=full_report&list_uids=256471) | major facilitator superfamily domain containing 8 |
| [Details](http://mirdb.org/cgi-bin/target_detail.cgi?targetID=1232002) | 647 | 63 | hsa-let-7d-5p | [PAG1](http://www.ncbi.nlm.nih.gov/entrez/query.fcgi?db=gene&cmd=Retrieve&dopt=full_report&list_uids=55824) | phosphoprotein membrane anchor with glycosphingolipid microdomains 1 |
| [Details](http://mirdb.org/cgi-bin/target_detail.cgi?targetID=1231693) | 648 | 63 | hsa-let-7d-5p | [PUDP](http://www.ncbi.nlm.nih.gov/entrez/query.fcgi?db=gene&cmd=Retrieve&dopt=full_report&list_uids=8226) | pseudouridine 5'-phosphatase |
| [Details](http://mirdb.org/cgi-bin/target_detail.cgi?targetID=1231622) | 649 | 63 | hsa-let-7d-5p | [NOVA1](http://www.ncbi.nlm.nih.gov/entrez/query.fcgi?db=gene&cmd=Retrieve&dopt=full_report&list_uids=4857) | NOVA alternative splicing regulator 1 |
| [Details](http://mirdb.org/cgi-bin/target_detail.cgi?targetID=1231859) | 650 | 63 | hsa-let-7d-5p | [CHSY3](http://www.ncbi.nlm.nih.gov/entrez/query.fcgi?db=gene&cmd=Retrieve&dopt=full_report&list_uids=337876) | chondroitin sulfate synthase 3 |
| [Details](http://mirdb.org/cgi-bin/target_detail.cgi?targetID=1232159) | 651 | 63 | hsa-let-7d-5p | [TEAD3](http://www.ncbi.nlm.nih.gov/entrez/query.fcgi?db=gene&cmd=Retrieve&dopt=full_report&list_uids=7005) | TEA domain transcription factor 3 |
| [Details](http://mirdb.org/cgi-bin/target_detail.cgi?targetID=1232046) | 652 | 63 | hsa-let-7d-5p | [ZNF763](http://www.ncbi.nlm.nih.gov/entrez/query.fcgi?db=gene&cmd=Retrieve&dopt=full_report&list_uids=284390) | zinc finger protein 763 |
| [Details](http://mirdb.org/cgi-bin/target_detail.cgi?targetID=1231999) | 653 | 63 | hsa-let-7d-5p | [GPX7](http://www.ncbi.nlm.nih.gov/entrez/query.fcgi?db=gene&cmd=Retrieve&dopt=full_report&list_uids=2882) | glutathione peroxidase 7 |
| [Details](http://mirdb.org/cgi-bin/target_detail.cgi?targetID=1231926) | 654 | 63 | hsa-let-7d-5p | [RBFOX1](http://www.ncbi.nlm.nih.gov/entrez/query.fcgi?db=gene&cmd=Retrieve&dopt=full_report&list_uids=54715) | RNA binding fox-1 homolog 1 |
| [Details](http://mirdb.org/cgi-bin/target_detail.cgi?targetID=1232324) | 655 | 63 | hsa-let-7d-5p | [EPB41](http://www.ncbi.nlm.nih.gov/entrez/query.fcgi?db=gene&cmd=Retrieve&dopt=full_report&list_uids=2035) | erythrocyte membrane protein band 4.1 |
| [Details](http://mirdb.org/cgi-bin/target_detail.cgi?targetID=1232319) | 656 | 63 | hsa-let-7d-5p | [LBH](http://www.ncbi.nlm.nih.gov/entrez/query.fcgi?db=gene&cmd=Retrieve&dopt=full_report&list_uids=81606) | limb bud and heart development |
| [Details](http://mirdb.org/cgi-bin/target_detail.cgi?targetID=1231557) | 657 | 63 | hsa-let-7d-5p | [CIAO2A](http://www.ncbi.nlm.nih.gov/entrez/query.fcgi?db=gene&cmd=Retrieve&dopt=full_report&list_uids=84191) | cytosolic iron-sulfur assembly component 2A |
| [Details](http://mirdb.org/cgi-bin/target_detail.cgi?targetID=1231633) | 658 | 63 | hsa-let-7d-5p | [SNAP23](http://www.ncbi.nlm.nih.gov/entrez/query.fcgi?db=gene&cmd=Retrieve&dopt=full_report&list_uids=8773) | synaptosome associated protein 23 |
| [Details](http://mirdb.org/cgi-bin/target_detail.cgi?targetID=1232092) | 659 | 63 | hsa-let-7d-5p | [ETNK1](http://www.ncbi.nlm.nih.gov/entrez/query.fcgi?db=gene&cmd=Retrieve&dopt=full_report&list_uids=55500) | ethanolamine kinase 1 |
| [Details](http://mirdb.org/cgi-bin/target_detail.cgi?targetID=1232377) | 660 | 63 | hsa-let-7d-5p | [ATG10](http://www.ncbi.nlm.nih.gov/entrez/query.fcgi?db=gene&cmd=Retrieve&dopt=full_report&list_uids=83734) | autophagy related 10 |
| [Details](http://mirdb.org/cgi-bin/target_detail.cgi?targetID=1232383) | 661 | 63 | hsa-let-7d-5p | [SP8](http://www.ncbi.nlm.nih.gov/entrez/query.fcgi?db=gene&cmd=Retrieve&dopt=full_report&list_uids=221833) | Sp8 transcription factor |
| [Details](http://mirdb.org/cgi-bin/target_detail.cgi?targetID=1232361) | 662 | 63 | hsa-let-7d-5p | [ELOVL4](http://www.ncbi.nlm.nih.gov/entrez/query.fcgi?db=gene&cmd=Retrieve&dopt=full_report&list_uids=6785) | ELOVL fatty acid elongase 4 |
| [Details](http://mirdb.org/cgi-bin/target_detail.cgi?targetID=1231494) | 663 | 63 | hsa-let-7d-5p | [GLRX](http://www.ncbi.nlm.nih.gov/entrez/query.fcgi?db=gene&cmd=Retrieve&dopt=full_report&list_uids=2745) | glutaredoxin |
| [Details](http://mirdb.org/cgi-bin/target_detail.cgi?targetID=1231697) | 664 | 62 | hsa-let-7d-5p | [STEAP3](http://www.ncbi.nlm.nih.gov/entrez/query.fcgi?db=gene&cmd=Retrieve&dopt=full_report&list_uids=55240) | STEAP3 metalloreductase |
| [Details](http://mirdb.org/cgi-bin/target_detail.cgi?targetID=1232032) | 665 | 62 | hsa-let-7d-5p | [COL14A1](http://www.ncbi.nlm.nih.gov/entrez/query.fcgi?db=gene&cmd=Retrieve&dopt=full_report&list_uids=7373) | collagen type XIV alpha 1 chain |
| [Details](http://mirdb.org/cgi-bin/target_detail.cgi?targetID=1232354) | 666 | 62 | hsa-let-7d-5p | [ZNF792](http://www.ncbi.nlm.nih.gov/entrez/query.fcgi?db=gene&cmd=Retrieve&dopt=full_report&list_uids=126375) | zinc finger protein 792 |
| [Details](http://mirdb.org/cgi-bin/target_detail.cgi?targetID=1232037) | 667 | 62 | hsa-let-7d-5p | [WNK3](http://www.ncbi.nlm.nih.gov/entrez/query.fcgi?db=gene&cmd=Retrieve&dopt=full_report&list_uids=65267) | WNK lysine deficient protein kinase 3 |
| [Details](http://mirdb.org/cgi-bin/target_detail.cgi?targetID=1232015) | 668 | 62 | hsa-let-7d-5p | [POLR3A](http://www.ncbi.nlm.nih.gov/entrez/query.fcgi?db=gene&cmd=Retrieve&dopt=full_report&list_uids=11128) | RNA polymerase III subunit A |
| [Details](http://mirdb.org/cgi-bin/target_detail.cgi?targetID=1232223) | 669 | 62 | hsa-let-7d-5p | [THRB](http://www.ncbi.nlm.nih.gov/entrez/query.fcgi?db=gene&cmd=Retrieve&dopt=full_report&list_uids=7068) | thyroid hormone receptor beta |
| [Details](http://mirdb.org/cgi-bin/target_detail.cgi?targetID=1231822) | 670 | 62 | hsa-let-7d-5p | [OTOF](http://www.ncbi.nlm.nih.gov/entrez/query.fcgi?db=gene&cmd=Retrieve&dopt=full_report&list_uids=9381) | otoferlin |
| [Details](http://mirdb.org/cgi-bin/target_detail.cgi?targetID=1232071) | 671 | 62 | hsa-let-7d-5p | [UGGT1](http://www.ncbi.nlm.nih.gov/entrez/query.fcgi?db=gene&cmd=Retrieve&dopt=full_report&list_uids=56886) | UDP-glucose glycoprotein glucosyltransferase 1 |
| [Details](http://mirdb.org/cgi-bin/target_detail.cgi?targetID=1232026) | 672 | 62 | hsa-let-7d-5p | [THBS1](http://www.ncbi.nlm.nih.gov/entrez/query.fcgi?db=gene&cmd=Retrieve&dopt=full_report&list_uids=7057) | thrombospondin 1 |
| [Details](http://mirdb.org/cgi-bin/target_detail.cgi?targetID=1232043) | 673 | 62 | hsa-let-7d-5p | [MYO1F](http://www.ncbi.nlm.nih.gov/entrez/query.fcgi?db=gene&cmd=Retrieve&dopt=full_report&list_uids=4542) | myosin IF |
| [Details](http://mirdb.org/cgi-bin/target_detail.cgi?targetID=1231512) | 674 | 62 | hsa-let-7d-5p | [LRRC8B](http://www.ncbi.nlm.nih.gov/entrez/query.fcgi?db=gene&cmd=Retrieve&dopt=full_report&list_uids=23507) | leucine rich repeat containing 8 VRAC subunit B |
| [Details](http://mirdb.org/cgi-bin/target_detail.cgi?targetID=1232016) | 675 | 62 | hsa-let-7d-5p | [BBX](http://www.ncbi.nlm.nih.gov/entrez/query.fcgi?db=gene&cmd=Retrieve&dopt=full_report&list_uids=56987) | BBX, HMG-box containing |
| [Details](http://mirdb.org/cgi-bin/target_detail.cgi?targetID=1231769) | 676 | 62 | hsa-let-7d-5p | [ZNF200](http://www.ncbi.nlm.nih.gov/entrez/query.fcgi?db=gene&cmd=Retrieve&dopt=full_report&list_uids=7752) | zinc finger protein 200 |
| [Details](http://mirdb.org/cgi-bin/target_detail.cgi?targetID=1231737) | 677 | 61 | hsa-let-7d-5p | [APH1A](http://www.ncbi.nlm.nih.gov/entrez/query.fcgi?db=gene&cmd=Retrieve&dopt=full_report&list_uids=51107) | aph-1 homolog A, gamma-secretase subunit |
| [Details](http://mirdb.org/cgi-bin/target_detail.cgi?targetID=1231930) | 678 | 61 | hsa-let-7d-5p | [CBFA2T3](http://www.ncbi.nlm.nih.gov/entrez/query.fcgi?db=gene&cmd=Retrieve&dopt=full_report&list_uids=863) | CBFA2/RUNX1 translocation partner 3 |
| [Details](http://mirdb.org/cgi-bin/target_detail.cgi?targetID=1232298) | 679 | 61 | hsa-let-7d-5p | [ARL4D](http://www.ncbi.nlm.nih.gov/entrez/query.fcgi?db=gene&cmd=Retrieve&dopt=full_report&list_uids=379) | ADP ribosylation factor like GTPase 4D |
| [Details](http://mirdb.org/cgi-bin/target_detail.cgi?targetID=1231676) | 680 | 61 | hsa-let-7d-5p | [SYT11](http://www.ncbi.nlm.nih.gov/entrez/query.fcgi?db=gene&cmd=Retrieve&dopt=full_report&list_uids=23208) | synaptotagmin 11 |
| [Details](http://mirdb.org/cgi-bin/target_detail.cgi?targetID=1231688) | 681 | 61 | hsa-let-7d-5p | [CSRNP3](http://www.ncbi.nlm.nih.gov/entrez/query.fcgi?db=gene&cmd=Retrieve&dopt=full_report&list_uids=80034) | cysteine and serine rich nuclear protein 3 |
| [Details](http://mirdb.org/cgi-bin/target_detail.cgi?targetID=1232001) | 682 | 61 | hsa-let-7d-5p | [NTRK3](http://www.ncbi.nlm.nih.gov/entrez/query.fcgi?db=gene&cmd=Retrieve&dopt=full_report&list_uids=4916) | neurotrophic receptor tyrosine kinase 3 |
| [Details](http://mirdb.org/cgi-bin/target_detail.cgi?targetID=1232364) | 683 | 61 | hsa-let-7d-5p | [ANKRD49](http://www.ncbi.nlm.nih.gov/entrez/query.fcgi?db=gene&cmd=Retrieve&dopt=full_report&list_uids=54851) | ankyrin repeat domain 49 |
| [Details](http://mirdb.org/cgi-bin/target_detail.cgi?targetID=1231950) | 684 | 61 | hsa-let-7d-5p | [FAM120C](http://www.ncbi.nlm.nih.gov/entrez/query.fcgi?db=gene&cmd=Retrieve&dopt=full_report&list_uids=54954) | family with sequence similarity 120C |
| [Details](http://mirdb.org/cgi-bin/target_detail.cgi?targetID=1231588) | 685 | 61 | hsa-let-7d-5p | [ESRP2](http://www.ncbi.nlm.nih.gov/entrez/query.fcgi?db=gene&cmd=Retrieve&dopt=full_report&list_uids=80004) | epithelial splicing regulatory protein 2 |
| [Details](http://mirdb.org/cgi-bin/target_detail.cgi?targetID=1231864) | 686 | 61 | hsa-let-7d-5p | [ARHGAP12](http://www.ncbi.nlm.nih.gov/entrez/query.fcgi?db=gene&cmd=Retrieve&dopt=full_report&list_uids=94134) | Rho GTPase activating protein 12 |
| [Details](http://mirdb.org/cgi-bin/target_detail.cgi?targetID=1231705) | 687 | 61 | hsa-let-7d-5p | [CRY2](http://www.ncbi.nlm.nih.gov/entrez/query.fcgi?db=gene&cmd=Retrieve&dopt=full_report&list_uids=1408) | cryptochrome circadian regulator 2 |
| [Details](http://mirdb.org/cgi-bin/target_detail.cgi?targetID=1231685) | 688 | 61 | hsa-let-7d-5p | [RRM2](http://www.ncbi.nlm.nih.gov/entrez/query.fcgi?db=gene&cmd=Retrieve&dopt=full_report&list_uids=6241) | ribonucleotide reductase regulatory subunit M2 |
| [Details](http://mirdb.org/cgi-bin/target_detail.cgi?targetID=1231673) | 689 | 61 | hsa-let-7d-5p | [SLC16A14](http://www.ncbi.nlm.nih.gov/entrez/query.fcgi?db=gene&cmd=Retrieve&dopt=full_report&list_uids=151473) | solute carrier family 16 member 14 |
| [Details](http://mirdb.org/cgi-bin/target_detail.cgi?targetID=1232343) | 690 | 61 | hsa-let-7d-5p | [GIPC1](http://www.ncbi.nlm.nih.gov/entrez/query.fcgi?db=gene&cmd=Retrieve&dopt=full_report&list_uids=10755) | GIPC PDZ domain containing family member 1 |
| [Details](http://mirdb.org/cgi-bin/target_detail.cgi?targetID=1231774) | 691 | 61 | hsa-let-7d-5p | [DLGAP4](http://www.ncbi.nlm.nih.gov/entrez/query.fcgi?db=gene&cmd=Retrieve&dopt=full_report&list_uids=22839) | DLG associated protein 4 |
| [Details](http://mirdb.org/cgi-bin/target_detail.cgi?targetID=1231613) | 692 | 61 | hsa-let-7d-5p | [GAREM1](http://www.ncbi.nlm.nih.gov/entrez/query.fcgi?db=gene&cmd=Retrieve&dopt=full_report&list_uids=64762) | GRB2 associated regulator of MAPK1 subtype 1 |
| [Details](http://mirdb.org/cgi-bin/target_detail.cgi?targetID=1231715) | 693 | 61 | hsa-let-7d-5p | [CPSF4](http://www.ncbi.nlm.nih.gov/entrez/query.fcgi?db=gene&cmd=Retrieve&dopt=full_report&list_uids=10898) | cleavage and polyadenylation specific factor 4 |
| [Details](http://mirdb.org/cgi-bin/target_detail.cgi?targetID=1231516) | 694 | 61 | hsa-let-7d-5p | [CDCA8](http://www.ncbi.nlm.nih.gov/entrez/query.fcgi?db=gene&cmd=Retrieve&dopt=full_report&list_uids=55143) | cell division cycle associated 8 |
| [Details](http://mirdb.org/cgi-bin/target_detail.cgi?targetID=1232036) | 695 | 61 | hsa-let-7d-5p | [ABHD18](http://www.ncbi.nlm.nih.gov/entrez/query.fcgi?db=gene&cmd=Retrieve&dopt=full_report&list_uids=80167) | abhydrolase domain containing 18 |
| [Details](http://mirdb.org/cgi-bin/target_detail.cgi?targetID=1231743) | 696 | 61 | hsa-let-7d-5p | [SOGA3](http://www.ncbi.nlm.nih.gov/entrez/query.fcgi?db=gene&cmd=Retrieve&dopt=full_report&list_uids=387104) | SOGA family member 3 |
| [Details](http://mirdb.org/cgi-bin/target_detail.cgi?targetID=1232132) | 697 | 61 | hsa-let-7d-5p | [BZW2](http://www.ncbi.nlm.nih.gov/entrez/query.fcgi?db=gene&cmd=Retrieve&dopt=full_report&list_uids=28969) | basic leucine zipper and W2 domains 2 |
| [Details](http://mirdb.org/cgi-bin/target_detail.cgi?targetID=1232236) | 698 | 61 | hsa-let-7d-5p | [IL22RA1](http://www.ncbi.nlm.nih.gov/entrez/query.fcgi?db=gene&cmd=Retrieve&dopt=full_report&list_uids=58985) | interleukin 22 receptor subunit alpha 1 |
| [Details](http://mirdb.org/cgi-bin/target_detail.cgi?targetID=1231955) | 699 | 61 | hsa-let-7d-5p | [TEPSIN](http://www.ncbi.nlm.nih.gov/entrez/query.fcgi?db=gene&cmd=Retrieve&dopt=full_report&list_uids=146705) | TEPSIN, adaptor related protein complex 4 accessory protein |
| [Details](http://mirdb.org/cgi-bin/target_detail.cgi?targetID=1232257) | 700 | 61 | hsa-let-7d-5p | [GAB2](http://www.ncbi.nlm.nih.gov/entrez/query.fcgi?db=gene&cmd=Retrieve&dopt=full_report&list_uids=9846) | GRB2 associated binding protein 2 |
| [Details](http://mirdb.org/cgi-bin/target_detail.cgi?targetID=1232237) | 701 | 60 | hsa-let-7d-5p | [PKN2](http://www.ncbi.nlm.nih.gov/entrez/query.fcgi?db=gene&cmd=Retrieve&dopt=full_report&list_uids=5586) | protein kinase N2 |
| [Details](http://mirdb.org/cgi-bin/target_detail.cgi?targetID=1231690) | 702 | 60 | hsa-let-7d-5p | [SLC7A14](http://www.ncbi.nlm.nih.gov/entrez/query.fcgi?db=gene&cmd=Retrieve&dopt=full_report&list_uids=57709) | solute carrier family 7 member 14 |
| [Details](http://mirdb.org/cgi-bin/target_detail.cgi?targetID=1232190) | 703 | 60 | hsa-let-7d-5p | [DUSP16](http://www.ncbi.nlm.nih.gov/entrez/query.fcgi?db=gene&cmd=Retrieve&dopt=full_report&list_uids=80824) | dual specificity phosphatase 16 |
| [Details](http://mirdb.org/cgi-bin/target_detail.cgi?targetID=1231777) | 704 | 60 | hsa-let-7d-5p | [ANKRD52](http://www.ncbi.nlm.nih.gov/entrez/query.fcgi?db=gene&cmd=Retrieve&dopt=full_report&list_uids=283373) | ankyrin repeat domain 52 |
| [Details](http://mirdb.org/cgi-bin/target_detail.cgi?targetID=1231514) | 705 | 60 | hsa-let-7d-5p | [RNF217](http://www.ncbi.nlm.nih.gov/entrez/query.fcgi?db=gene&cmd=Retrieve&dopt=full_report&list_uids=154214) | ring finger protein 217 |
| [Details](http://mirdb.org/cgi-bin/target_detail.cgi?targetID=1231739) | 706 | 60 | hsa-let-7d-5p | [TET2](http://www.ncbi.nlm.nih.gov/entrez/query.fcgi?db=gene&cmd=Retrieve&dopt=full_report&list_uids=54790) | tet methylcytosine dioxygenase 2 |
| [Details](http://mirdb.org/cgi-bin/target_detail.cgi?targetID=1232096) | 707 | 60 | hsa-let-7d-5p | [RUNX1T1](http://www.ncbi.nlm.nih.gov/entrez/query.fcgi?db=gene&cmd=Retrieve&dopt=full_report&list_uids=862) | RUNX1 translocation partner 1 |
| [Details](http://mirdb.org/cgi-bin/target_detail.cgi?targetID=1231789) | 708 | 60 | hsa-let-7d-5p | [STON2](http://www.ncbi.nlm.nih.gov/entrez/query.fcgi?db=gene&cmd=Retrieve&dopt=full_report&list_uids=85439) | stonin 2 |
| [Details](http://mirdb.org/cgi-bin/target_detail.cgi?targetID=1231982) | 709 | 60 | hsa-let-7d-5p | [C15orf41](http://www.ncbi.nlm.nih.gov/entrez/query.fcgi?db=gene&cmd=Retrieve&dopt=full_report&list_uids=84529) | chromosome 15 open reading frame 41 |
| [Details](http://mirdb.org/cgi-bin/target_detail.cgi?targetID=1232061) | 710 | 60 | hsa-let-7d-5p | [VPS33A](http://www.ncbi.nlm.nih.gov/entrez/query.fcgi?db=gene&cmd=Retrieve&dopt=full_report&list_uids=65082) | VPS33A, CORVET/HOPS core subunit |
| [Details](http://mirdb.org/cgi-bin/target_detail.cgi?targetID=1232262) | 711 | 60 | hsa-let-7d-5p | [BRD3](http://www.ncbi.nlm.nih.gov/entrez/query.fcgi?db=gene&cmd=Retrieve&dopt=full_report&list_uids=8019) | bromodomain containing 3 |
| [Details](http://mirdb.org/cgi-bin/target_detail.cgi?targetID=1231980) | 712 | 60 | hsa-let-7d-5p | [HABP4](http://www.ncbi.nlm.nih.gov/entrez/query.fcgi?db=gene&cmd=Retrieve&dopt=full_report&list_uids=22927) | hyaluronan binding protein 4 |
| [Details](http://mirdb.org/cgi-bin/target_detail.cgi?targetID=1231639) | 713 | 59 | hsa-let-7d-5p | [BTG2](http://www.ncbi.nlm.nih.gov/entrez/query.fcgi?db=gene&cmd=Retrieve&dopt=full_report&list_uids=7832) | BTG anti-proliferation factor 2 |
| [Details](http://mirdb.org/cgi-bin/target_detail.cgi?targetID=1231640) | 714 | 59 | hsa-let-7d-5p | [KCNC1](http://www.ncbi.nlm.nih.gov/entrez/query.fcgi?db=gene&cmd=Retrieve&dopt=full_report&list_uids=3746) | potassium voltage-gated channel subfamily C member 1 |
| [Details](http://mirdb.org/cgi-bin/target_detail.cgi?targetID=1231896) | 715 | 59 | hsa-let-7d-5p | [FAM118A](http://www.ncbi.nlm.nih.gov/entrez/query.fcgi?db=gene&cmd=Retrieve&dopt=full_report&list_uids=55007) | family with sequence similarity 118 member A |
| [Details](http://mirdb.org/cgi-bin/target_detail.cgi?targetID=1231604) | 716 | 59 | hsa-let-7d-5p | [ATP6V1G1](http://www.ncbi.nlm.nih.gov/entrez/query.fcgi?db=gene&cmd=Retrieve&dopt=full_report&list_uids=9550) | ATPase H+ transporting V1 subunit G1 |
| [Details](http://mirdb.org/cgi-bin/target_detail.cgi?targetID=1231869) | 717 | 59 | hsa-let-7d-5p | [TPP1](http://www.ncbi.nlm.nih.gov/entrez/query.fcgi?db=gene&cmd=Retrieve&dopt=full_report&list_uids=1200) | tripeptidyl peptidase 1 |
| [Details](http://mirdb.org/cgi-bin/target_detail.cgi?targetID=1232200) | 718 | 59 | hsa-let-7d-5p | [RNMT](http://www.ncbi.nlm.nih.gov/entrez/query.fcgi?db=gene&cmd=Retrieve&dopt=full_report&list_uids=8731) | RNA guanine-7 methyltransferase |
| [Details](http://mirdb.org/cgi-bin/target_detail.cgi?targetID=1231661) | 719 | 59 | hsa-let-7d-5p | [TSPAN2](http://www.ncbi.nlm.nih.gov/entrez/query.fcgi?db=gene&cmd=Retrieve&dopt=full_report&list_uids=10100) | tetraspanin 2 |
| [Details](http://mirdb.org/cgi-bin/target_detail.cgi?targetID=1232006) | 720 | 59 | hsa-let-7d-5p | [OLFM4](http://www.ncbi.nlm.nih.gov/entrez/query.fcgi?db=gene&cmd=Retrieve&dopt=full_report&list_uids=10562) | olfactomedin 4 |
| [Details](http://mirdb.org/cgi-bin/target_detail.cgi?targetID=1231671) | 721 | 59 | hsa-let-7d-5p | [CPED1](http://www.ncbi.nlm.nih.gov/entrez/query.fcgi?db=gene&cmd=Retrieve&dopt=full_report&list_uids=79974) | cadherin like and PC-esterase domain containing 1 |
| [Details](http://mirdb.org/cgi-bin/target_detail.cgi?targetID=1231556) | 722 | 59 | hsa-let-7d-5p | [ZCCHC10](http://www.ncbi.nlm.nih.gov/entrez/query.fcgi?db=gene&cmd=Retrieve&dopt=full_report&list_uids=54819) | zinc finger CCHC-type containing 10 |
| [Details](http://mirdb.org/cgi-bin/target_detail.cgi?targetID=1232358) | 723 | 59 | hsa-let-7d-5p | [PLCB4](http://www.ncbi.nlm.nih.gov/entrez/query.fcgi?db=gene&cmd=Retrieve&dopt=full_report&list_uids=5332) | phospholipase C beta 4 |
| [Details](http://mirdb.org/cgi-bin/target_detail.cgi?targetID=1232055) | 724 | 59 | hsa-let-7d-5p | [RTL9](http://www.ncbi.nlm.nih.gov/entrez/query.fcgi?db=gene&cmd=Retrieve&dopt=full_report&list_uids=57529) | retrotransposon Gag like 9 |
| [Details](http://mirdb.org/cgi-bin/target_detail.cgi?targetID=1231534) | 725 | 59 | hsa-let-7d-5p | [DYNC2LI1](http://www.ncbi.nlm.nih.gov/entrez/query.fcgi?db=gene&cmd=Retrieve&dopt=full_report&list_uids=51626) | dynein cytoplasmic 2 light intermediate chain 1 |
| [Details](http://mirdb.org/cgi-bin/target_detail.cgi?targetID=1231799) | 726 | 59 | hsa-let-7d-5p | [PLCXD3](http://www.ncbi.nlm.nih.gov/entrez/query.fcgi?db=gene&cmd=Retrieve&dopt=full_report&list_uids=345557) | phosphatidylinositol specific phospholipase C X domain containing 3 |
| [Details](http://mirdb.org/cgi-bin/target_detail.cgi?targetID=1231504) | 727 | 59 | hsa-let-7d-5p | [ANKRD46](http://www.ncbi.nlm.nih.gov/entrez/query.fcgi?db=gene&cmd=Retrieve&dopt=full_report&list_uids=157567) | ankyrin repeat domain 46 |
| [Details](http://mirdb.org/cgi-bin/target_detail.cgi?targetID=1232295) | 728 | 59 | hsa-let-7d-5p | [RPS6KA3](http://www.ncbi.nlm.nih.gov/entrez/query.fcgi?db=gene&cmd=Retrieve&dopt=full_report&list_uids=6197) | ribosomal protein S6 kinase A3 |
| [Details](http://mirdb.org/cgi-bin/target_detail.cgi?targetID=1232049) | 729 | 59 | hsa-let-7d-5p | [SLC52A3](http://www.ncbi.nlm.nih.gov/entrez/query.fcgi?db=gene&cmd=Retrieve&dopt=full_report&list_uids=113278) | solute carrier family 52 member 3 |
| [Details](http://mirdb.org/cgi-bin/target_detail.cgi?targetID=1232127) | 730 | 59 | hsa-let-7d-5p | [LYVE1](http://www.ncbi.nlm.nih.gov/entrez/query.fcgi?db=gene&cmd=Retrieve&dopt=full_report&list_uids=10894) | lymphatic vessel endothelial hyaluronan receptor 1 |
| [Details](http://mirdb.org/cgi-bin/target_detail.cgi?targetID=1231987) | 731 | 58 | hsa-let-7d-5p | [GPATCH3](http://www.ncbi.nlm.nih.gov/entrez/query.fcgi?db=gene&cmd=Retrieve&dopt=full_report&list_uids=63906) | G-patch domain containing 3 |
| [Details](http://mirdb.org/cgi-bin/target_detail.cgi?targetID=1232202) | 732 | 58 | hsa-let-7d-5p | [TRABD](http://www.ncbi.nlm.nih.gov/entrez/query.fcgi?db=gene&cmd=Retrieve&dopt=full_report&list_uids=80305) | TraB domain containing |
| [Details](http://mirdb.org/cgi-bin/target_detail.cgi?targetID=1231708) | 733 | 58 | hsa-let-7d-5p | [ARHGAP8](http://www.ncbi.nlm.nih.gov/entrez/query.fcgi?db=gene&cmd=Retrieve&dopt=full_report&list_uids=23779) | Rho GTPase activating protein 8 |
| [Details](http://mirdb.org/cgi-bin/target_detail.cgi?targetID=1231823) | 734 | 58 | hsa-let-7d-5p | [FCAR](http://www.ncbi.nlm.nih.gov/entrez/query.fcgi?db=gene&cmd=Retrieve&dopt=full_report&list_uids=2204) | Fc fragment of IgA receptor |
| [Details](http://mirdb.org/cgi-bin/target_detail.cgi?targetID=1231547) | 735 | 58 | hsa-let-7d-5p | [CPM](http://www.ncbi.nlm.nih.gov/entrez/query.fcgi?db=gene&cmd=Retrieve&dopt=full_report&list_uids=1368) | carboxypeptidase M |
| [Details](http://mirdb.org/cgi-bin/target_detail.cgi?targetID=1232230) | 736 | 58 | hsa-let-7d-5p | [DOCK1](http://www.ncbi.nlm.nih.gov/entrez/query.fcgi?db=gene&cmd=Retrieve&dopt=full_report&list_uids=1793) | dedicator of cytokinesis 1 |
| [Details](http://mirdb.org/cgi-bin/target_detail.cgi?targetID=1232154) | 737 | 58 | hsa-let-7d-5p | [SNN](http://www.ncbi.nlm.nih.gov/entrez/query.fcgi?db=gene&cmd=Retrieve&dopt=full_report&list_uids=8303) | stannin |
| [Details](http://mirdb.org/cgi-bin/target_detail.cgi?targetID=1232077) | 738 | 58 | hsa-let-7d-5p | [DOCK3](http://www.ncbi.nlm.nih.gov/entrez/query.fcgi?db=gene&cmd=Retrieve&dopt=full_report&list_uids=1795) | dedicator of cytokinesis 3 |
| [Details](http://mirdb.org/cgi-bin/target_detail.cgi?targetID=1232150) | 739 | 58 | hsa-let-7d-5p | [ELK4](http://www.ncbi.nlm.nih.gov/entrez/query.fcgi?db=gene&cmd=Retrieve&dopt=full_report&list_uids=2005) | ELK4, ETS transcription factor |
| [Details](http://mirdb.org/cgi-bin/target_detail.cgi?targetID=1231824) | 740 | 58 | hsa-let-7d-5p | [SH2B3](http://www.ncbi.nlm.nih.gov/entrez/query.fcgi?db=gene&cmd=Retrieve&dopt=full_report&list_uids=10019) | SH2B adaptor protein 3 |
| [Details](http://mirdb.org/cgi-bin/target_detail.cgi?targetID=1231702) | 741 | 58 | hsa-let-7d-5p | [AGO1](http://www.ncbi.nlm.nih.gov/entrez/query.fcgi?db=gene&cmd=Retrieve&dopt=full_report&list_uids=26523) | argonaute RISC catalytic component 1 |
| [Details](http://mirdb.org/cgi-bin/target_detail.cgi?targetID=1231473) | 742 | 58 | hsa-let-7d-5p | [VASH2](http://www.ncbi.nlm.nih.gov/entrez/query.fcgi?db=gene&cmd=Retrieve&dopt=full_report&list_uids=79805) | vasohibin 2 |
| [Details](http://mirdb.org/cgi-bin/target_detail.cgi?targetID=1231614) | 743 | 58 | hsa-let-7d-5p | [HTR4](http://www.ncbi.nlm.nih.gov/entrez/query.fcgi?db=gene&cmd=Retrieve&dopt=full_report&list_uids=3360) | 5-hydroxytryptamine receptor 4 |
| [Details](http://mirdb.org/cgi-bin/target_detail.cgi?targetID=1231603) | 744 | 58 | hsa-let-7d-5p | [SBNO1](http://www.ncbi.nlm.nih.gov/entrez/query.fcgi?db=gene&cmd=Retrieve&dopt=full_report&list_uids=55206) | strawberry notch homolog 1 |
| [Details](http://mirdb.org/cgi-bin/target_detail.cgi?targetID=1231816) | 745 | 58 | hsa-let-7d-5p | [FBXO32](http://www.ncbi.nlm.nih.gov/entrez/query.fcgi?db=gene&cmd=Retrieve&dopt=full_report&list_uids=114907) | F-box protein 32 |
| [Details](http://mirdb.org/cgi-bin/target_detail.cgi?targetID=1231885) | 746 | 58 | hsa-let-7d-5p | [YTHDF3](http://www.ncbi.nlm.nih.gov/entrez/query.fcgi?db=gene&cmd=Retrieve&dopt=full_report&list_uids=253943) | YTH N6-methyladenosine RNA binding protein 3 |
| [Details](http://mirdb.org/cgi-bin/target_detail.cgi?targetID=1232285) | 747 | 58 | hsa-let-7d-5p | [STAG3](http://www.ncbi.nlm.nih.gov/entrez/query.fcgi?db=gene&cmd=Retrieve&dopt=full_report&list_uids=10734) | stromal antigen 3 |
| [Details](http://mirdb.org/cgi-bin/target_detail.cgi?targetID=1232143) | 748 | 58 | hsa-let-7d-5p | [FAM122C](http://www.ncbi.nlm.nih.gov/entrez/query.fcgi?db=gene&cmd=Retrieve&dopt=full_report&list_uids=159091) | family with sequence similarity 122C |
| [Details](http://mirdb.org/cgi-bin/target_detail.cgi?targetID=1232072) | 749 | 58 | hsa-let-7d-5p | [PLEKHG7](http://www.ncbi.nlm.nih.gov/entrez/query.fcgi?db=gene&cmd=Retrieve&dopt=full_report&list_uids=440107) | pleckstrin homology and RhoGEF domain containing G7 |
| [Details](http://mirdb.org/cgi-bin/target_detail.cgi?targetID=1231879) | 750 | 58 | hsa-let-7d-5p | [SKIDA1](http://www.ncbi.nlm.nih.gov/entrez/query.fcgi?db=gene&cmd=Retrieve&dopt=full_report&list_uids=387640) | SKI/DACH domain containing 1 |
| [Details](http://mirdb.org/cgi-bin/target_detail.cgi?targetID=1231923) | 751 | 58 | hsa-let-7d-5p | [DYRK2](http://www.ncbi.nlm.nih.gov/entrez/query.fcgi?db=gene&cmd=Retrieve&dopt=full_report&list_uids=8445) | dual specificity tyrosine phosphorylation regulated kinase 2 |
| [Details](http://mirdb.org/cgi-bin/target_detail.cgi?targetID=1232258) | 752 | 57 | hsa-let-7d-5p | [COL1A1](http://www.ncbi.nlm.nih.gov/entrez/query.fcgi?db=gene&cmd=Retrieve&dopt=full_report&list_uids=1277) | collagen type I alpha 1 chain |
| [Details](http://mirdb.org/cgi-bin/target_detail.cgi?targetID=1231940) | 753 | 57 | hsa-let-7d-5p | [GALNT4](http://www.ncbi.nlm.nih.gov/entrez/query.fcgi?db=gene&cmd=Retrieve&dopt=full_report&list_uids=8693) | polypeptide N-acetylgalactosaminyltransferase 4 |
| [Details](http://mirdb.org/cgi-bin/target_detail.cgi?targetID=1231875) | 754 | 57 | hsa-let-7d-5p | [ZNF318](http://www.ncbi.nlm.nih.gov/entrez/query.fcgi?db=gene&cmd=Retrieve&dopt=full_report&list_uids=24149) | zinc finger protein 318 |
| [Details](http://mirdb.org/cgi-bin/target_detail.cgi?targetID=1231535) | 755 | 57 | hsa-let-7d-5p | [PPT2](http://www.ncbi.nlm.nih.gov/entrez/query.fcgi?db=gene&cmd=Retrieve&dopt=full_report&list_uids=9374) | palmitoyl-protein thioesterase 2 |
| [Details](http://mirdb.org/cgi-bin/target_detail.cgi?targetID=1231519) | 756 | 57 | hsa-let-7d-5p | [RNF5](http://www.ncbi.nlm.nih.gov/entrez/query.fcgi?db=gene&cmd=Retrieve&dopt=full_report&list_uids=6048) | ring finger protein 5 |
| [Details](http://mirdb.org/cgi-bin/target_detail.cgi?targetID=1232197) | 757 | 57 | hsa-let-7d-5p | [ATP6V1C1](http://www.ncbi.nlm.nih.gov/entrez/query.fcgi?db=gene&cmd=Retrieve&dopt=full_report&list_uids=528) | ATPase H+ transporting V1 subunit C1 |
| [Details](http://mirdb.org/cgi-bin/target_detail.cgi?targetID=1232350) | 758 | 57 | hsa-let-7d-5p | [POC1B-GALNT4](http://www.ncbi.nlm.nih.gov/entrez/query.fcgi?db=gene&cmd=Retrieve&dopt=full_report&list_uids=100528030) | POC1B-GALNT4 readthrough |
| [Details](http://mirdb.org/cgi-bin/target_detail.cgi?targetID=1232386) | 759 | 57 | hsa-let-7d-5p | [TMEM41A](http://www.ncbi.nlm.nih.gov/entrez/query.fcgi?db=gene&cmd=Retrieve&dopt=full_report&list_uids=90407) | transmembrane protein 41A |
| [Details](http://mirdb.org/cgi-bin/target_detail.cgi?targetID=1231995) | 760 | 57 | hsa-let-7d-5p | [TMEM178B](http://www.ncbi.nlm.nih.gov/entrez/query.fcgi?db=gene&cmd=Retrieve&dopt=full_report&list_uids=100507421) | transmembrane protein 178B |
| [Details](http://mirdb.org/cgi-bin/target_detail.cgi?targetID=1231488) | 761 | 57 | hsa-let-7d-5p | [GHR](http://www.ncbi.nlm.nih.gov/entrez/query.fcgi?db=gene&cmd=Retrieve&dopt=full_report&list_uids=2690) | growth hormone receptor |
| [Details](http://mirdb.org/cgi-bin/target_detail.cgi?targetID=1231989) | 762 | 57 | hsa-let-7d-5p | [GPR156](http://www.ncbi.nlm.nih.gov/entrez/query.fcgi?db=gene&cmd=Retrieve&dopt=full_report&list_uids=165829) | G protein-coupled receptor 156 |
| [Details](http://mirdb.org/cgi-bin/target_detail.cgi?targetID=1232282) | 763 | 57 | hsa-let-7d-5p | [SMIM13](http://www.ncbi.nlm.nih.gov/entrez/query.fcgi?db=gene&cmd=Retrieve&dopt=full_report&list_uids=221710) | small integral membrane protein 13 |
| [Details](http://mirdb.org/cgi-bin/target_detail.cgi?targetID=1231800) | 764 | 57 | hsa-let-7d-5p | [ERP29](http://www.ncbi.nlm.nih.gov/entrez/query.fcgi?db=gene&cmd=Retrieve&dopt=full_report&list_uids=10961) | endoplasmic reticulum protein 29 |
| [Details](http://mirdb.org/cgi-bin/target_detail.cgi?targetID=1231716) | 765 | 57 | hsa-let-7d-5p | [SFT2D3](http://www.ncbi.nlm.nih.gov/entrez/query.fcgi?db=gene&cmd=Retrieve&dopt=full_report&list_uids=84826) | SFT2 domain containing 3 |
| [Details](http://mirdb.org/cgi-bin/target_detail.cgi?targetID=1231843) | 766 | 57 | hsa-let-7d-5p | [BCL7A](http://www.ncbi.nlm.nih.gov/entrez/query.fcgi?db=gene&cmd=Retrieve&dopt=full_report&list_uids=605) | BCL7A, BAF complex component |
| [Details](http://mirdb.org/cgi-bin/target_detail.cgi?targetID=1232218) | 767 | 57 | hsa-let-7d-5p | [DKK3](http://www.ncbi.nlm.nih.gov/entrez/query.fcgi?db=gene&cmd=Retrieve&dopt=full_report&list_uids=27122) | dickkopf WNT signaling pathway inhibitor 3 |
| [Details](http://mirdb.org/cgi-bin/target_detail.cgi?targetID=1232058) | 768 | 57 | hsa-let-7d-5p | [ZNF268](http://www.ncbi.nlm.nih.gov/entrez/query.fcgi?db=gene&cmd=Retrieve&dopt=full_report&list_uids=10795) | zinc finger protein 268 |
| [Details](http://mirdb.org/cgi-bin/target_detail.cgi?targetID=1231742) | 769 | 57 | hsa-let-7d-5p | [ZNF215](http://www.ncbi.nlm.nih.gov/entrez/query.fcgi?db=gene&cmd=Retrieve&dopt=full_report&list_uids=7762) | zinc finger protein 215 |
| [Details](http://mirdb.org/cgi-bin/target_detail.cgi?targetID=1232184) | 770 | 57 | hsa-let-7d-5p | [SCYL3](http://www.ncbi.nlm.nih.gov/entrez/query.fcgi?db=gene&cmd=Retrieve&dopt=full_report&list_uids=57147) | SCY1 like pseudokinase 3 |
| [Details](http://mirdb.org/cgi-bin/target_detail.cgi?targetID=1231601) | 771 | 56 | hsa-let-7d-5p | [CCND1](http://www.ncbi.nlm.nih.gov/entrez/query.fcgi?db=gene&cmd=Retrieve&dopt=full_report&list_uids=595) | cyclin D1 |
| [Details](http://mirdb.org/cgi-bin/target_detail.cgi?targetID=1231626) | 772 | 56 | hsa-let-7d-5p | [GABRA6](http://www.ncbi.nlm.nih.gov/entrez/query.fcgi?db=gene&cmd=Retrieve&dopt=full_report&list_uids=2559) | gamma-aminobutyric acid type A receptor alpha6 subunit |
| [Details](http://mirdb.org/cgi-bin/target_detail.cgi?targetID=1231625) | 773 | 56 | hsa-let-7d-5p | [ACTR10](http://www.ncbi.nlm.nih.gov/entrez/query.fcgi?db=gene&cmd=Retrieve&dopt=full_report&list_uids=55860) | actin related protein 10 homolog |
| [Details](http://mirdb.org/cgi-bin/target_detail.cgi?targetID=1232317) | 774 | 56 | hsa-let-7d-5p | [AAK1](http://www.ncbi.nlm.nih.gov/entrez/query.fcgi?db=gene&cmd=Retrieve&dopt=full_report&list_uids=22848) | AP2 associated kinase 1 |
| [Details](http://mirdb.org/cgi-bin/target_detail.cgi?targetID=1232270) | 775 | 56 | hsa-let-7d-5p | [POTEM](http://www.ncbi.nlm.nih.gov/entrez/query.fcgi?db=gene&cmd=Retrieve&dopt=full_report&list_uids=641455) | POTE ankyrin domain family member M |
| [Details](http://mirdb.org/cgi-bin/target_detail.cgi?targetID=1231559) | 776 | 56 | hsa-let-7d-5p | [FRMD5](http://www.ncbi.nlm.nih.gov/entrez/query.fcgi?db=gene&cmd=Retrieve&dopt=full_report&list_uids=84978) | FERM domain containing 5 |
| [Details](http://mirdb.org/cgi-bin/target_detail.cgi?targetID=1232309) | 777 | 56 | hsa-let-7d-5p | [SOX6](http://www.ncbi.nlm.nih.gov/entrez/query.fcgi?db=gene&cmd=Retrieve&dopt=full_report&list_uids=55553) | SRY-box 6 |
| [Details](http://mirdb.org/cgi-bin/target_detail.cgi?targetID=1232284) | 778 | 56 | hsa-let-7d-5p | [ICMT](http://www.ncbi.nlm.nih.gov/entrez/query.fcgi?db=gene&cmd=Retrieve&dopt=full_report&list_uids=23463) | isoprenylcysteine carboxyl methyltransferase |
| [Details](http://mirdb.org/cgi-bin/target_detail.cgi?targetID=1232314) | 779 | 56 | hsa-let-7d-5p | [CFL2](http://www.ncbi.nlm.nih.gov/entrez/query.fcgi?db=gene&cmd=Retrieve&dopt=full_report&list_uids=1073) | cofilin 2 |
| [Details](http://mirdb.org/cgi-bin/target_detail.cgi?targetID=1231678) | 780 | 56 | hsa-let-7d-5p | [CACNA1E](http://www.ncbi.nlm.nih.gov/entrez/query.fcgi?db=gene&cmd=Retrieve&dopt=full_report&list_uids=777) | calcium voltage-gated channel subunit alpha1 E |
| [Details](http://mirdb.org/cgi-bin/target_detail.cgi?targetID=1232073) | 781 | 56 | hsa-let-7d-5p | [MARCH9](http://www.ncbi.nlm.nih.gov/entrez/query.fcgi?db=gene&cmd=Retrieve&dopt=full_report&list_uids=92979) | membrane associated ring-CH-type finger 9 |
| [Details](http://mirdb.org/cgi-bin/target_detail.cgi?targetID=1232195) | 782 | 56 | hsa-let-7d-5p | [NAA30](http://www.ncbi.nlm.nih.gov/entrez/query.fcgi?db=gene&cmd=Retrieve&dopt=full_report&list_uids=122830) | N(alpha)-acetyltransferase 30, NatC catalytic subunit |
| [Details](http://mirdb.org/cgi-bin/target_detail.cgi?targetID=1232226) | 783 | 56 | hsa-let-7d-5p | [ARID1B](http://www.ncbi.nlm.nih.gov/entrez/query.fcgi?db=gene&cmd=Retrieve&dopt=full_report&list_uids=57492) | AT-rich interaction domain 1B |
| [Details](http://mirdb.org/cgi-bin/target_detail.cgi?targetID=1231781) | 784 | 56 | hsa-let-7d-5p | [SPEG](http://www.ncbi.nlm.nih.gov/entrez/query.fcgi?db=gene&cmd=Retrieve&dopt=full_report&list_uids=10290) | striated muscle enriched protein kinase |
| [Details](http://mirdb.org/cgi-bin/target_detail.cgi?targetID=1231687) | 785 | 56 | hsa-let-7d-5p | [CEP164](http://www.ncbi.nlm.nih.gov/entrez/query.fcgi?db=gene&cmd=Retrieve&dopt=full_report&list_uids=22897) | centrosomal protein 164 |
| [Details](http://mirdb.org/cgi-bin/target_detail.cgi?targetID=1232091) | 786 | 56 | hsa-let-7d-5p | [TBX5](http://www.ncbi.nlm.nih.gov/entrez/query.fcgi?db=gene&cmd=Retrieve&dopt=full_report&list_uids=6910) | T-box 5 |
| [Details](http://mirdb.org/cgi-bin/target_detail.cgi?targetID=1231784) | 787 | 56 | hsa-let-7d-5p | [TTC14](http://www.ncbi.nlm.nih.gov/entrez/query.fcgi?db=gene&cmd=Retrieve&dopt=full_report&list_uids=151613) | tetratricopeptide repeat domain 14 |
| [Details](http://mirdb.org/cgi-bin/target_detail.cgi?targetID=1232153) | 788 | 56 | hsa-let-7d-5p | [ZNF311](http://www.ncbi.nlm.nih.gov/entrez/query.fcgi?db=gene&cmd=Retrieve&dopt=full_report&list_uids=282890) | zinc finger protein 311 |
| [Details](http://mirdb.org/cgi-bin/target_detail.cgi?targetID=1231675) | 789 | 56 | hsa-let-7d-5p | [FREM2](http://www.ncbi.nlm.nih.gov/entrez/query.fcgi?db=gene&cmd=Retrieve&dopt=full_report&list_uids=341640) | FRAS1 related extracellular matrix protein 2 |
| [Details](http://mirdb.org/cgi-bin/target_detail.cgi?targetID=1232056) | 790 | 56 | hsa-let-7d-5p | [MAN2A2](http://www.ncbi.nlm.nih.gov/entrez/query.fcgi?db=gene&cmd=Retrieve&dopt=full_report&list_uids=4122) | mannosidase alpha class 2A member 2 |
| [Details](http://mirdb.org/cgi-bin/target_detail.cgi?targetID=1231854) | 791 | 56 | hsa-let-7d-5p | [FKRP](http://www.ncbi.nlm.nih.gov/entrez/query.fcgi?db=gene&cmd=Retrieve&dopt=full_report&list_uids=79147) | fukutin related protein |
| [Details](http://mirdb.org/cgi-bin/target_detail.cgi?targetID=1232177) | 792 | 56 | hsa-let-7d-5p | [DMRT2](http://www.ncbi.nlm.nih.gov/entrez/query.fcgi?db=gene&cmd=Retrieve&dopt=full_report&list_uids=10655) | doublesex and mab-3 related transcription factor 2 |
| [Details](http://mirdb.org/cgi-bin/target_detail.cgi?targetID=1231735) | 793 | 56 | hsa-let-7d-5p | [AGBL1](http://www.ncbi.nlm.nih.gov/entrez/query.fcgi?db=gene&cmd=Retrieve&dopt=full_report&list_uids=123624) | ATP/GTP binding protein like 1 |
| [Details](http://mirdb.org/cgi-bin/target_detail.cgi?targetID=1231938) | 794 | 55 | hsa-let-7d-5p | [ANKRD28](http://www.ncbi.nlm.nih.gov/entrez/query.fcgi?db=gene&cmd=Retrieve&dopt=full_report&list_uids=23243) | ankyrin repeat domain 28 |
| [Details](http://mirdb.org/cgi-bin/target_detail.cgi?targetID=1231865) | 795 | 55 | hsa-let-7d-5p | [NIPA1](http://www.ncbi.nlm.nih.gov/entrez/query.fcgi?db=gene&cmd=Retrieve&dopt=full_report&list_uids=123606) | NIPA magnesium transporter 1 |
| [Details](http://mirdb.org/cgi-bin/target_detail.cgi?targetID=1232013) | 796 | 55 | hsa-let-7d-5p | [NKIRAS2](http://www.ncbi.nlm.nih.gov/entrez/query.fcgi?db=gene&cmd=Retrieve&dopt=full_report&list_uids=28511) | NFKB inhibitor interacting Ras like 2 |
| [Details](http://mirdb.org/cgi-bin/target_detail.cgi?targetID=1232083) | 797 | 55 | hsa-let-7d-5p | [ST8SIA1](http://www.ncbi.nlm.nih.gov/entrez/query.fcgi?db=gene&cmd=Retrieve&dopt=full_report&list_uids=6489) | ST8 alpha-N-acetyl-neuraminide alpha-2,8-sialyltransferase 1 |
| [Details](http://mirdb.org/cgi-bin/target_detail.cgi?targetID=1232042) | 798 | 55 | hsa-let-7d-5p | [RAMAC](http://www.ncbi.nlm.nih.gov/entrez/query.fcgi?db=gene&cmd=Retrieve&dopt=full_report&list_uids=83640) | RNA guanine-7 methyltransferase activating subunit |
| [Details](http://mirdb.org/cgi-bin/target_detail.cgi?targetID=1231595) | 799 | 55 | hsa-let-7d-5p | [OPRM1](http://www.ncbi.nlm.nih.gov/entrez/query.fcgi?db=gene&cmd=Retrieve&dopt=full_report&list_uids=4988) | opioid receptor mu 1 |
| [Details](http://mirdb.org/cgi-bin/target_detail.cgi?targetID=1231707) | 800 | 55 | hsa-let-7d-5p | [MSANTD2](http://www.ncbi.nlm.nih.gov/entrez/query.fcgi?db=gene&cmd=Retrieve&dopt=full_report&list_uids=79684) | Myb/SANT DNA binding domain containing 2 |
| [Details](http://mirdb.org/cgi-bin/target_detail.cgi?targetID=1231776) | 801 | 55 | hsa-let-7d-5p | [DAPK1](http://www.ncbi.nlm.nih.gov/entrez/query.fcgi?db=gene&cmd=Retrieve&dopt=full_report&list_uids=1612) | death associated protein kinase 1 |
| [Details](http://mirdb.org/cgi-bin/target_detail.cgi?targetID=1232128) | 802 | 55 | hsa-let-7d-5p | [SREK1IP1](http://www.ncbi.nlm.nih.gov/entrez/query.fcgi?db=gene&cmd=Retrieve&dopt=full_report&list_uids=285672) | SREK1 interacting protein 1 |
| [Details](http://mirdb.org/cgi-bin/target_detail.cgi?targetID=1231997) | 803 | 55 | hsa-let-7d-5p | [IFNLR1](http://www.ncbi.nlm.nih.gov/entrez/query.fcgi?db=gene&cmd=Retrieve&dopt=full_report&list_uids=163702) | interferon lambda receptor 1 |
| [Details](http://mirdb.org/cgi-bin/target_detail.cgi?targetID=1232346) | 804 | 55 | hsa-let-7d-5p | [DUSP7](http://www.ncbi.nlm.nih.gov/entrez/query.fcgi?db=gene&cmd=Retrieve&dopt=full_report&list_uids=1849) | dual specificity phosphatase 7 |
| [Details](http://mirdb.org/cgi-bin/target_detail.cgi?targetID=1231755) | 805 | 55 | hsa-let-7d-5p | [PSORS1C2](http://www.ncbi.nlm.nih.gov/entrez/query.fcgi?db=gene&cmd=Retrieve&dopt=full_report&list_uids=170680) | psoriasis susceptibility 1 candidate 2 |
| [Details](http://mirdb.org/cgi-bin/target_detail.cgi?targetID=1231727) | 806 | 55 | hsa-let-7d-5p | [DOK3](http://www.ncbi.nlm.nih.gov/entrez/query.fcgi?db=gene&cmd=Retrieve&dopt=full_report&list_uids=79930) | docking protein 3 |
| [Details](http://mirdb.org/cgi-bin/target_detail.cgi?targetID=1232275) | 807 | 55 | hsa-let-7d-5p | [EPPIN-WFDC6](http://www.ncbi.nlm.nih.gov/entrez/query.fcgi?db=gene&cmd=Retrieve&dopt=full_report&list_uids=100526773) | EPPIN-WFDC6 readthrough |
| [Details](http://mirdb.org/cgi-bin/target_detail.cgi?targetID=1232265) | 808 | 55 | hsa-let-7d-5p | [GDAP2](http://www.ncbi.nlm.nih.gov/entrez/query.fcgi?db=gene&cmd=Retrieve&dopt=full_report&list_uids=54834) | ganglioside induced differentiation associated protein 2 |
| [Details](http://mirdb.org/cgi-bin/target_detail.cgi?targetID=1232203) | 809 | 55 | hsa-let-7d-5p | [BTBD9](http://www.ncbi.nlm.nih.gov/entrez/query.fcgi?db=gene&cmd=Retrieve&dopt=full_report&list_uids=114781) | BTB domain containing 9 |
| [Details](http://mirdb.org/cgi-bin/target_detail.cgi?targetID=1231617) | 810 | 55 | hsa-let-7d-5p | [ARMC9](http://www.ncbi.nlm.nih.gov/entrez/query.fcgi?db=gene&cmd=Retrieve&dopt=full_report&list_uids=80210) | armadillo repeat containing 9 |
| [Details](http://mirdb.org/cgi-bin/target_detail.cgi?targetID=1231870) | 811 | 55 | hsa-let-7d-5p | [PLCG2](http://www.ncbi.nlm.nih.gov/entrez/query.fcgi?db=gene&cmd=Retrieve&dopt=full_report&list_uids=5336) | phospholipase C gamma 2 |
| [Details](http://mirdb.org/cgi-bin/target_detail.cgi?targetID=1231641) | 812 | 55 | hsa-let-7d-5p | [ADAM28](http://www.ncbi.nlm.nih.gov/entrez/query.fcgi?db=gene&cmd=Retrieve&dopt=full_report&list_uids=10863) | ADAM metallopeptidase domain 28 |
| [Details](http://mirdb.org/cgi-bin/target_detail.cgi?targetID=1232390) | 813 | 55 | hsa-let-7d-5p | [GJB4](http://www.ncbi.nlm.nih.gov/entrez/query.fcgi?db=gene&cmd=Retrieve&dopt=full_report&list_uids=127534) | gap junction protein beta 4 |
| [Details](http://mirdb.org/cgi-bin/target_detail.cgi?targetID=1231836) | 814 | 55 | hsa-let-7d-5p | [EHF](http://www.ncbi.nlm.nih.gov/entrez/query.fcgi?db=gene&cmd=Retrieve&dopt=full_report&list_uids=26298) | ETS homologous factor |
| [Details](http://mirdb.org/cgi-bin/target_detail.cgi?targetID=1231786) | 815 | 55 | hsa-let-7d-5p | [EGLN2](http://www.ncbi.nlm.nih.gov/entrez/query.fcgi?db=gene&cmd=Retrieve&dopt=full_report&list_uids=112398) | egl-9 family hypoxia inducible factor 2 |
| [Details](http://mirdb.org/cgi-bin/target_detail.cgi?targetID=1231812) | 816 | 55 | hsa-let-7d-5p | [POU2F1](http://www.ncbi.nlm.nih.gov/entrez/query.fcgi?db=gene&cmd=Retrieve&dopt=full_report&list_uids=5451) | POU class 2 homeobox 1 |
| [Details](http://mirdb.org/cgi-bin/target_detail.cgi?targetID=1231966) | 817 | 55 | hsa-let-7d-5p | [SLC45A4](http://www.ncbi.nlm.nih.gov/entrez/query.fcgi?db=gene&cmd=Retrieve&dopt=full_report&list_uids=57210) | solute carrier family 45 member 4 |
| [Details](http://mirdb.org/cgi-bin/target_detail.cgi?targetID=1231470) | 818 | 55 | hsa-let-7d-5p | [VGLL3](http://www.ncbi.nlm.nih.gov/entrez/query.fcgi?db=gene&cmd=Retrieve&dopt=full_report&list_uids=389136) | vestigial like family member 3 |
| [Details](http://mirdb.org/cgi-bin/target_detail.cgi?targetID=1231709) | 819 | 54 | hsa-let-7d-5p | [ZNF197](http://www.ncbi.nlm.nih.gov/entrez/query.fcgi?db=gene&cmd=Retrieve&dopt=full_report&list_uids=10168) | zinc finger protein 197 |
| [Details](http://mirdb.org/cgi-bin/target_detail.cgi?targetID=1231863) | 820 | 54 | hsa-let-7d-5p | [PPP1R12B](http://www.ncbi.nlm.nih.gov/entrez/query.fcgi?db=gene&cmd=Retrieve&dopt=full_report&list_uids=4660) | protein phosphatase 1 regulatory subunit 12B |
| [Details](http://mirdb.org/cgi-bin/target_detail.cgi?targetID=1231852) | 821 | 54 | hsa-let-7d-5p | [MEX3A](http://www.ncbi.nlm.nih.gov/entrez/query.fcgi?db=gene&cmd=Retrieve&dopt=full_report&list_uids=92312) | mex-3 RNA binding family member A |
| [Details](http://mirdb.org/cgi-bin/target_detail.cgi?targetID=1232048) | 822 | 54 | hsa-let-7d-5p | [GPR132](http://www.ncbi.nlm.nih.gov/entrez/query.fcgi?db=gene&cmd=Retrieve&dopt=full_report&list_uids=29933) | G protein-coupled receptor 132 |
| [Details](http://mirdb.org/cgi-bin/target_detail.cgi?targetID=1232252) | 823 | 54 | hsa-let-7d-5p | [HS2ST1](http://www.ncbi.nlm.nih.gov/entrez/query.fcgi?db=gene&cmd=Retrieve&dopt=full_report&list_uids=9653) | heparan sulfate 2-O-sulfotransferase 1 |
| [Details](http://mirdb.org/cgi-bin/target_detail.cgi?targetID=1231747) | 824 | 54 | hsa-let-7d-5p | [LDB1](http://www.ncbi.nlm.nih.gov/entrez/query.fcgi?db=gene&cmd=Retrieve&dopt=full_report&list_uids=8861) | LIM domain binding 1 |
| [Details](http://mirdb.org/cgi-bin/target_detail.cgi?targetID=1231979) | 825 | 54 | hsa-let-7d-5p | [CTHRC1](http://www.ncbi.nlm.nih.gov/entrez/query.fcgi?db=gene&cmd=Retrieve&dopt=full_report&list_uids=115908) | collagen triple helix repeat containing 1 |
| [Details](http://mirdb.org/cgi-bin/target_detail.cgi?targetID=1231880) | 826 | 54 | hsa-let-7d-5p | [PPARA](http://www.ncbi.nlm.nih.gov/entrez/query.fcgi?db=gene&cmd=Retrieve&dopt=full_report&list_uids=5465) | peroxisome proliferator activated receptor alpha |
| [Details](http://mirdb.org/cgi-bin/target_detail.cgi?targetID=1231804) | 827 | 54 | hsa-let-7d-5p | [OSM](http://www.ncbi.nlm.nih.gov/entrez/query.fcgi?db=gene&cmd=Retrieve&dopt=full_report&list_uids=5008) | oncostatin M |
| [Details](http://mirdb.org/cgi-bin/target_detail.cgi?targetID=1231511) | 828 | 54 | hsa-let-7d-5p | [SULF2](http://www.ncbi.nlm.nih.gov/entrez/query.fcgi?db=gene&cmd=Retrieve&dopt=full_report&list_uids=55959) | sulfatase 2 |
| [Details](http://mirdb.org/cgi-bin/target_detail.cgi?targetID=1232277) | 829 | 54 | hsa-let-7d-5p | [TRIB2](http://www.ncbi.nlm.nih.gov/entrez/query.fcgi?db=gene&cmd=Retrieve&dopt=full_report&list_uids=28951) | tribbles pseudokinase 2 |
| [Details](http://mirdb.org/cgi-bin/target_detail.cgi?targetID=1231902) | 830 | 54 | hsa-let-7d-5p | [YY1](http://www.ncbi.nlm.nih.gov/entrez/query.fcgi?db=gene&cmd=Retrieve&dopt=full_report&list_uids=7528) | YY1 transcription factor |
| [Details](http://mirdb.org/cgi-bin/target_detail.cgi?targetID=1231670) | 831 | 54 | hsa-let-7d-5p | [SOCS7](http://www.ncbi.nlm.nih.gov/entrez/query.fcgi?db=gene&cmd=Retrieve&dopt=full_report&list_uids=30837) | suppressor of cytokine signaling 7 |
| [Details](http://mirdb.org/cgi-bin/target_detail.cgi?targetID=1231554) | 832 | 54 | hsa-let-7d-5p | [BTBD3](http://www.ncbi.nlm.nih.gov/entrez/query.fcgi?db=gene&cmd=Retrieve&dopt=full_report&list_uids=22903) | BTB domain containing 3 |
| [Details](http://mirdb.org/cgi-bin/target_detail.cgi?targetID=1232087) | 833 | 54 | hsa-let-7d-5p | [CNOT6](http://www.ncbi.nlm.nih.gov/entrez/query.fcgi?db=gene&cmd=Retrieve&dopt=full_report&list_uids=57472) | CCR4-NOT transcription complex subunit 6 |
| [Details](http://mirdb.org/cgi-bin/target_detail.cgi?targetID=1231712) | 834 | 54 | hsa-let-7d-5p | [SNAI3](http://www.ncbi.nlm.nih.gov/entrez/query.fcgi?db=gene&cmd=Retrieve&dopt=full_report&list_uids=333929) | snail family transcriptional repressor 3 |
| [Details](http://mirdb.org/cgi-bin/target_detail.cgi?targetID=1231732) | 835 | 54 | hsa-let-7d-5p | [ATP2B4](http://www.ncbi.nlm.nih.gov/entrez/query.fcgi?db=gene&cmd=Retrieve&dopt=full_report&list_uids=493) | ATPase plasma membrane Ca2+ transporting 4 |
| [Details](http://mirdb.org/cgi-bin/target_detail.cgi?targetID=1232199) | 836 | 54 | hsa-let-7d-5p | [MUC4](http://www.ncbi.nlm.nih.gov/entrez/query.fcgi?db=gene&cmd=Retrieve&dopt=full_report&list_uids=4585) | mucin 4, cell surface associated |
| [Details](http://mirdb.org/cgi-bin/target_detail.cgi?targetID=1231703) | 837 | 54 | hsa-let-7d-5p | [UGT3A1](http://www.ncbi.nlm.nih.gov/entrez/query.fcgi?db=gene&cmd=Retrieve&dopt=full_report&list_uids=133688) | UDP glycosyltransferase family 3 member A1 |
| [Details](http://mirdb.org/cgi-bin/target_detail.cgi?targetID=1231491) | 838 | 54 | hsa-let-7d-5p | [RORC](http://www.ncbi.nlm.nih.gov/entrez/query.fcgi?db=gene&cmd=Retrieve&dopt=full_report&list_uids=6097) | RAR related orphan receptor C |
| [Details](http://mirdb.org/cgi-bin/target_detail.cgi?targetID=1232166) | 839 | 53 | hsa-let-7d-5p | [TRHDE](http://www.ncbi.nlm.nih.gov/entrez/query.fcgi?db=gene&cmd=Retrieve&dopt=full_report&list_uids=29953) | thyrotropin releasing hormone degrading enzyme |
| [Details](http://mirdb.org/cgi-bin/target_detail.cgi?targetID=1232164) | 840 | 53 | hsa-let-7d-5p | [IL6](http://www.ncbi.nlm.nih.gov/entrez/query.fcgi?db=gene&cmd=Retrieve&dopt=full_report&list_uids=3569) | interleukin 6 |
| [Details](http://mirdb.org/cgi-bin/target_detail.cgi?targetID=1231838) | 841 | 53 | hsa-let-7d-5p | [CLOCK](http://www.ncbi.nlm.nih.gov/entrez/query.fcgi?db=gene&cmd=Retrieve&dopt=full_report&list_uids=9575) | clock circadian regulator |
| [Details](http://mirdb.org/cgi-bin/target_detail.cgi?targetID=1232320) | 842 | 53 | hsa-let-7d-5p | [NEFM](http://www.ncbi.nlm.nih.gov/entrez/query.fcgi?db=gene&cmd=Retrieve&dopt=full_report&list_uids=4741) | neurofilament medium |
| [Details](http://mirdb.org/cgi-bin/target_detail.cgi?targetID=1231931) | 843 | 53 | hsa-let-7d-5p | [CTPS2](http://www.ncbi.nlm.nih.gov/entrez/query.fcgi?db=gene&cmd=Retrieve&dopt=full_report&list_uids=56474) | CTP synthase 2 |
| [Details](http://mirdb.org/cgi-bin/target_detail.cgi?targetID=1231819) | 844 | 53 | hsa-let-7d-5p | [CRK](http://www.ncbi.nlm.nih.gov/entrez/query.fcgi?db=gene&cmd=Retrieve&dopt=full_report&list_uids=1398) | CRK proto-oncogene, adaptor protein |
| [Details](http://mirdb.org/cgi-bin/target_detail.cgi?targetID=1232379) | 845 | 53 | hsa-let-7d-5p | [CACNA1I](http://www.ncbi.nlm.nih.gov/entrez/query.fcgi?db=gene&cmd=Retrieve&dopt=full_report&list_uids=8911) | calcium voltage-gated channel subunit alpha1 I |
| [Details](http://mirdb.org/cgi-bin/target_detail.cgi?targetID=1231845) | 846 | 53 | hsa-let-7d-5p | [IFT80](http://www.ncbi.nlm.nih.gov/entrez/query.fcgi?db=gene&cmd=Retrieve&dopt=full_report&list_uids=57560) | intraflagellar transport 80 |
| [Details](http://mirdb.org/cgi-bin/target_detail.cgi?targetID=1231960) | 847 | 53 | hsa-let-7d-5p | [GOLGA6L9](http://www.ncbi.nlm.nih.gov/entrez/query.fcgi?db=gene&cmd=Retrieve&dopt=full_report&list_uids=440295) | golgin A6 family-like 9 |
| [Details](http://mirdb.org/cgi-bin/target_detail.cgi?targetID=1232076) | 848 | 53 | hsa-let-7d-5p | [CCDC93](http://www.ncbi.nlm.nih.gov/entrez/query.fcgi?db=gene&cmd=Retrieve&dopt=full_report&list_uids=54520) | coiled-coil domain containing 93 |
| [Details](http://mirdb.org/cgi-bin/target_detail.cgi?targetID=1232120) | 849 | 53 | hsa-let-7d-5p | [KIAA0895](http://www.ncbi.nlm.nih.gov/entrez/query.fcgi?db=gene&cmd=Retrieve&dopt=full_report&list_uids=23366) | KIAA0895 |
| [Details](http://mirdb.org/cgi-bin/target_detail.cgi?targetID=1232366) | 850 | 53 | hsa-let-7d-5p | [MYO5B](http://www.ncbi.nlm.nih.gov/entrez/query.fcgi?db=gene&cmd=Retrieve&dopt=full_report&list_uids=4645) | myosin VB |
| [Details](http://mirdb.org/cgi-bin/target_detail.cgi?targetID=1232231) | 851 | 53 | hsa-let-7d-5p | [TP53](http://www.ncbi.nlm.nih.gov/entrez/query.fcgi?db=gene&cmd=Retrieve&dopt=full_report&list_uids=7157) | tumor protein p53 |
| [Details](http://mirdb.org/cgi-bin/target_detail.cgi?targetID=1231616) | 852 | 53 | hsa-let-7d-5p | [LMX1A](http://www.ncbi.nlm.nih.gov/entrez/query.fcgi?db=gene&cmd=Retrieve&dopt=full_report&list_uids=4009) | LIM homeobox transcription factor 1 alpha |
| [Details](http://mirdb.org/cgi-bin/target_detail.cgi?targetID=1231649) | 853 | 53 | hsa-let-7d-5p | [RRP1B](http://www.ncbi.nlm.nih.gov/entrez/query.fcgi?db=gene&cmd=Retrieve&dopt=full_report&list_uids=23076) | ribosomal RNA processing 1B |
| [Details](http://mirdb.org/cgi-bin/target_detail.cgi?targetID=1231533) | 854 | 53 | hsa-let-7d-5p | [RDH10](http://www.ncbi.nlm.nih.gov/entrez/query.fcgi?db=gene&cmd=Retrieve&dopt=full_report&list_uids=157506) | retinol dehydrogenase 10 |
| [Details](http://mirdb.org/cgi-bin/target_detail.cgi?targetID=1231587) | 855 | 53 | hsa-let-7d-5p | [ODF2L](http://www.ncbi.nlm.nih.gov/entrez/query.fcgi?db=gene&cmd=Retrieve&dopt=full_report&list_uids=57489) | outer dense fiber of sperm tails 2 like |
| [Details](http://mirdb.org/cgi-bin/target_detail.cgi?targetID=1232017) | 856 | 53 | hsa-let-7d-5p | [P2RX1](http://www.ncbi.nlm.nih.gov/entrez/query.fcgi?db=gene&cmd=Retrieve&dopt=full_report&list_uids=5023) | purinergic receptor P2X 1 |
| [Details](http://mirdb.org/cgi-bin/target_detail.cgi?targetID=1231882) | 857 | 53 | hsa-let-7d-5p | [ZBTB8OS](http://www.ncbi.nlm.nih.gov/entrez/query.fcgi?db=gene&cmd=Retrieve&dopt=full_report&list_uids=339487) | zinc finger and BTB domain containing 8 opposite strand |
| [Details](http://mirdb.org/cgi-bin/target_detail.cgi?targetID=1231906) | 858 | 53 | hsa-let-7d-5p | [PRDM2](http://www.ncbi.nlm.nih.gov/entrez/query.fcgi?db=gene&cmd=Retrieve&dopt=full_report&list_uids=7799) | PR/SET domain 2 |
| [Details](http://mirdb.org/cgi-bin/target_detail.cgi?targetID=1232044) | 859 | 53 | hsa-let-7d-5p | [GVQW2](http://www.ncbi.nlm.nih.gov/entrez/query.fcgi?db=gene&cmd=Retrieve&dopt=full_report&list_uids=100507462) | GVQW motif containing 2 |
| [Details](http://mirdb.org/cgi-bin/target_detail.cgi?targetID=1232267) | 860 | 52 | hsa-let-7d-5p | [BRWD1](http://www.ncbi.nlm.nih.gov/entrez/query.fcgi?db=gene&cmd=Retrieve&dopt=full_report&list_uids=54014) | bromodomain and WD repeat domain containing 1 |
| [Details](http://mirdb.org/cgi-bin/target_detail.cgi?targetID=1231581) | 861 | 52 | hsa-let-7d-5p | [KLK10](http://www.ncbi.nlm.nih.gov/entrez/query.fcgi?db=gene&cmd=Retrieve&dopt=full_report&list_uids=5655) | kallikrein related peptidase 10 |
| [Details](http://mirdb.org/cgi-bin/target_detail.cgi?targetID=1231586) | 862 | 52 | hsa-let-7d-5p | [HSPE1-MOB4](http://www.ncbi.nlm.nih.gov/entrez/query.fcgi?db=gene&cmd=Retrieve&dopt=full_report&list_uids=100529241) | HSPE1-MOB4 readthrough |
| [Details](http://mirdb.org/cgi-bin/target_detail.cgi?targetID=1231768) | 863 | 52 | hsa-let-7d-5p | [PIK3CA](http://www.ncbi.nlm.nih.gov/entrez/query.fcgi?db=gene&cmd=Retrieve&dopt=full_report&list_uids=5290) | phosphatidylinositol-4,5-bisphosphate 3-kinase catalytic subunit alpha |
| [Details](http://mirdb.org/cgi-bin/target_detail.cgi?targetID=1231575) | 864 | 52 | hsa-let-7d-5p | [DARS2](http://www.ncbi.nlm.nih.gov/entrez/query.fcgi?db=gene&cmd=Retrieve&dopt=full_report&list_uids=55157) | aspartyl-tRNA synthetase 2, mitochondrial |
| [Details](http://mirdb.org/cgi-bin/target_detail.cgi?targetID=1231638) | 865 | 52 | hsa-let-7d-5p | [KRT5](http://www.ncbi.nlm.nih.gov/entrez/query.fcgi?db=gene&cmd=Retrieve&dopt=full_report&list_uids=3852) | keratin 5 |
| [Details](http://mirdb.org/cgi-bin/target_detail.cgi?targetID=1232276) | 866 | 52 | hsa-let-7d-5p | [SERPINB9](http://www.ncbi.nlm.nih.gov/entrez/query.fcgi?db=gene&cmd=Retrieve&dopt=full_report&list_uids=5272) | serpin family B member 9 |
| [Details](http://mirdb.org/cgi-bin/target_detail.cgi?targetID=1231480) | 867 | 52 | hsa-let-7d-5p | [WIPI2](http://www.ncbi.nlm.nih.gov/entrez/query.fcgi?db=gene&cmd=Retrieve&dopt=full_report&list_uids=26100) | WD repeat domain, phosphoinositide interacting 2 |
| [Details](http://mirdb.org/cgi-bin/target_detail.cgi?targetID=1232062) | 868 | 52 | hsa-let-7d-5p | [MDFI](http://www.ncbi.nlm.nih.gov/entrez/query.fcgi?db=gene&cmd=Retrieve&dopt=full_report&list_uids=4188) | MyoD family inhibitor |
| [Details](http://mirdb.org/cgi-bin/target_detail.cgi?targetID=1232103) | 869 | 52 | hsa-let-7d-5p | [ICOS](http://www.ncbi.nlm.nih.gov/entrez/query.fcgi?db=gene&cmd=Retrieve&dopt=full_report&list_uids=29851) | inducible T cell costimulator |
| [Details](http://mirdb.org/cgi-bin/target_detail.cgi?targetID=1232169) | 870 | 52 | hsa-let-7d-5p | [KREMEN1](http://www.ncbi.nlm.nih.gov/entrez/query.fcgi?db=gene&cmd=Retrieve&dopt=full_report&list_uids=83999) | kringle containing transmembrane protein 1 |
| [Details](http://mirdb.org/cgi-bin/target_detail.cgi?targetID=1232051) | 871 | 52 | hsa-let-7d-5p | [CYB561D1](http://www.ncbi.nlm.nih.gov/entrez/query.fcgi?db=gene&cmd=Retrieve&dopt=full_report&list_uids=284613) | cytochrome b561 family member D1 |
| [Details](http://mirdb.org/cgi-bin/target_detail.cgi?targetID=1232388) | 872 | 52 | hsa-let-7d-5p | [CYTH3](http://www.ncbi.nlm.nih.gov/entrez/query.fcgi?db=gene&cmd=Retrieve&dopt=full_report&list_uids=9265) | cytohesin 3 |
| [Details](http://mirdb.org/cgi-bin/target_detail.cgi?targetID=1232387) | 873 | 52 | hsa-let-7d-5p | [ECHDC1](http://www.ncbi.nlm.nih.gov/entrez/query.fcgi?db=gene&cmd=Retrieve&dopt=full_report&list_uids=55862) | ethylmalonyl-CoA decarboxylase 1 |
| [Details](http://mirdb.org/cgi-bin/target_detail.cgi?targetID=1231927) | 874 | 52 | hsa-let-7d-5p | [GPAT3](http://www.ncbi.nlm.nih.gov/entrez/query.fcgi?db=gene&cmd=Retrieve&dopt=full_report&list_uids=84803) | glycerol-3-phosphate acyltransferase 3 |
| [Details](http://mirdb.org/cgi-bin/target_detail.cgi?targetID=1231548) | 875 | 52 | hsa-let-7d-5p | [PAX3](http://www.ncbi.nlm.nih.gov/entrez/query.fcgi?db=gene&cmd=Retrieve&dopt=full_report&list_uids=5077) | paired box 3 |
| [Details](http://mirdb.org/cgi-bin/target_detail.cgi?targetID=1232179) | 876 | 52 | hsa-let-7d-5p | [SEC31B](http://www.ncbi.nlm.nih.gov/entrez/query.fcgi?db=gene&cmd=Retrieve&dopt=full_report&list_uids=25956) | SEC31 homolog B, COPII coat complex component |
| [Details](http://mirdb.org/cgi-bin/target_detail.cgi?targetID=1232160) | 877 | 52 | hsa-let-7d-5p | [CCNY](http://www.ncbi.nlm.nih.gov/entrez/query.fcgi?db=gene&cmd=Retrieve&dopt=full_report&list_uids=219771) | cyclin Y |
| [Details](http://mirdb.org/cgi-bin/target_detail.cgi?targetID=1232378) | 878 | 52 | hsa-let-7d-5p | [RFX5](http://www.ncbi.nlm.nih.gov/entrez/query.fcgi?db=gene&cmd=Retrieve&dopt=full_report&list_uids=5993) | regulatory factor X5 |
| [Details](http://mirdb.org/cgi-bin/target_detail.cgi?targetID=1231657) | 879 | 52 | hsa-let-7d-5p | [B3GAT3](http://www.ncbi.nlm.nih.gov/entrez/query.fcgi?db=gene&cmd=Retrieve&dopt=full_report&list_uids=26229) | beta-1,3-glucuronyltransferase 3 |
| [Details](http://mirdb.org/cgi-bin/target_detail.cgi?targetID=1232240) | 880 | 51 | hsa-let-7d-5p | [TEX261](http://www.ncbi.nlm.nih.gov/entrez/query.fcgi?db=gene&cmd=Retrieve&dopt=full_report&list_uids=113419) | testis expressed 261 |
| [Details](http://mirdb.org/cgi-bin/target_detail.cgi?targetID=1231903) | 881 | 51 | hsa-let-7d-5p | [SYT2](http://www.ncbi.nlm.nih.gov/entrez/query.fcgi?db=gene&cmd=Retrieve&dopt=full_report&list_uids=127833) | synaptotagmin 2 |
| [Details](http://mirdb.org/cgi-bin/target_detail.cgi?targetID=1232215) | 882 | 51 | hsa-let-7d-5p | [RAG1](http://www.ncbi.nlm.nih.gov/entrez/query.fcgi?db=gene&cmd=Retrieve&dopt=full_report&list_uids=5896) | recombination activating 1 |
| [Details](http://mirdb.org/cgi-bin/target_detail.cgi?targetID=1231672) | 883 | 51 | hsa-let-7d-5p | [POLQ](http://www.ncbi.nlm.nih.gov/entrez/query.fcgi?db=gene&cmd=Retrieve&dopt=full_report&list_uids=10721) | DNA polymerase theta |
| [Details](http://mirdb.org/cgi-bin/target_detail.cgi?targetID=1231496) | 884 | 51 | hsa-let-7d-5p | [CASKIN1](http://www.ncbi.nlm.nih.gov/entrez/query.fcgi?db=gene&cmd=Retrieve&dopt=full_report&list_uids=57524) | CASK interacting protein 1 |
| [Details](http://mirdb.org/cgi-bin/target_detail.cgi?targetID=1232086) | 885 | 51 | hsa-let-7d-5p | [PTAR1](http://www.ncbi.nlm.nih.gov/entrez/query.fcgi?db=gene&cmd=Retrieve&dopt=full_report&list_uids=375743) | protein prenyltransferase alpha subunit repeat containing 1 |
| [Details](http://mirdb.org/cgi-bin/target_detail.cgi?targetID=1232261) | 886 | 51 | hsa-let-7d-5p | [BTN2A1](http://www.ncbi.nlm.nih.gov/entrez/query.fcgi?db=gene&cmd=Retrieve&dopt=full_report&list_uids=11120) | butyrophilin subfamily 2 member A1 |
| [Details](http://mirdb.org/cgi-bin/target_detail.cgi?targetID=1232238) | 887 | 51 | hsa-let-7d-5p | [NRK](http://www.ncbi.nlm.nih.gov/entrez/query.fcgi?db=gene&cmd=Retrieve&dopt=full_report&list_uids=203447) | Nik related kinase |
| [Details](http://mirdb.org/cgi-bin/target_detail.cgi?targetID=1232368) | 888 | 51 | hsa-let-7d-5p | [MON2](http://www.ncbi.nlm.nih.gov/entrez/query.fcgi?db=gene&cmd=Retrieve&dopt=full_report&list_uids=23041) | MON2 homolog, regulator of endosome-to-Golgi trafficking |
| [Details](http://mirdb.org/cgi-bin/target_detail.cgi?targetID=1232384) | 889 | 51 | hsa-let-7d-5p | [MMP1](http://www.ncbi.nlm.nih.gov/entrez/query.fcgi?db=gene&cmd=Retrieve&dopt=full_report&list_uids=4312) | matrix metallopeptidase 1 |
| [Details](http://mirdb.org/cgi-bin/target_detail.cgi?targetID=1232206) | 890 | 51 | hsa-let-7d-5p | [DYRK1A](http://www.ncbi.nlm.nih.gov/entrez/query.fcgi?db=gene&cmd=Retrieve&dopt=full_report&list_uids=1859) | dual specificity tyrosine phosphorylation regulated kinase 1A |
| [Details](http://mirdb.org/cgi-bin/target_detail.cgi?targetID=1232246) | 891 | 51 | hsa-let-7d-5p | [USP47](http://www.ncbi.nlm.nih.gov/entrez/query.fcgi?db=gene&cmd=Retrieve&dopt=full_report&list_uids=55031) | ubiquitin specific peptidase 47 |
| [Details](http://mirdb.org/cgi-bin/target_detail.cgi?targetID=1231570) | 892 | 51 | hsa-let-7d-5p | [CAP1](http://www.ncbi.nlm.nih.gov/entrez/query.fcgi?db=gene&cmd=Retrieve&dopt=full_report&list_uids=10487) | cyclase associated actin cytoskeleton regulatory protein 1 |
| [Details](http://mirdb.org/cgi-bin/target_detail.cgi?targetID=1231858) | 893 | 51 | hsa-let-7d-5p | [SEMA4F](http://www.ncbi.nlm.nih.gov/entrez/query.fcgi?db=gene&cmd=Retrieve&dopt=full_report&list_uids=10505) | ssemaphorin 4F |
| [Details](http://mirdb.org/cgi-bin/target_detail.cgi?targetID=1232180) | 894 | 51 | hsa-let-7d-5p | [KCNJ3](http://www.ncbi.nlm.nih.gov/entrez/query.fcgi?db=gene&cmd=Retrieve&dopt=full_report&list_uids=3760) | potassium voltage-gated channel subfamily J member 3 |
| [Details](http://mirdb.org/cgi-bin/target_detail.cgi?targetID=1232302) | 895 | 51 | hsa-let-7d-5p | [SPTBN4](http://www.ncbi.nlm.nih.gov/entrez/query.fcgi?db=gene&cmd=Retrieve&dopt=full_report&list_uids=57731) | spectrin beta, non-erythrocytic 4 |
| [Details](http://mirdb.org/cgi-bin/target_detail.cgi?targetID=1231983) | 896 | 51 | hsa-let-7d-5p | [NSD1](http://www.ncbi.nlm.nih.gov/entrez/query.fcgi?db=gene&cmd=Retrieve&dopt=full_report&list_uids=64324) | nuclear receptor binding SET domain protein 1 |
| [Details](http://mirdb.org/cgi-bin/target_detail.cgi?targetID=1231855) | 897 | 51 | hsa-let-7d-5p | [RB1](http://www.ncbi.nlm.nih.gov/entrez/query.fcgi?db=gene&cmd=Retrieve&dopt=full_report&list_uids=5925) | RB transcriptional corepressor 1 |
| [Details](http://mirdb.org/cgi-bin/target_detail.cgi?targetID=1231764) | 898 | 51 | hsa-let-7d-5p | [USP6](http://www.ncbi.nlm.nih.gov/entrez/query.fcgi?db=gene&cmd=Retrieve&dopt=full_report&list_uids=9098) | ubiquitin specific peptidase 6 |
| [Details](http://mirdb.org/cgi-bin/target_detail.cgi?targetID=1232313) | 899 | 51 | hsa-let-7d-5p | [UBE2K](http://www.ncbi.nlm.nih.gov/entrez/query.fcgi?db=gene&cmd=Retrieve&dopt=full_report&list_uids=3093) | ubiquitin conjugating enzyme E2 K |
| [Details](http://mirdb.org/cgi-bin/target_detail.cgi?targetID=1231507) | 900 | 51 | hsa-let-7d-5p | [EPPIN](http://www.ncbi.nlm.nih.gov/entrez/query.fcgi?db=gene&cmd=Retrieve&dopt=full_report&list_uids=57119) | epididymal peptidase inhibitor |
| [Details](http://mirdb.org/cgi-bin/target_detail.cgi?targetID=1231770) | 901 | 51 | hsa-let-7d-5p | [MYCBP](http://www.ncbi.nlm.nih.gov/entrez/query.fcgi?db=gene&cmd=Retrieve&dopt=full_report&list_uids=26292) | MYC binding protein |
| [Details](http://mirdb.org/cgi-bin/target_detail.cgi?targetID=1232047) | 902 | 51 | hsa-let-7d-5p | [ESPL1](http://www.ncbi.nlm.nih.gov/entrez/query.fcgi?db=gene&cmd=Retrieve&dopt=full_report&list_uids=9700) | extra spindle pole bodies like 1, separase |
| [Details](http://mirdb.org/cgi-bin/target_detail.cgi?targetID=1231825) | 903 | 51 | hsa-let-7d-5p | [ENAM](http://www.ncbi.nlm.nih.gov/entrez/query.fcgi?db=gene&cmd=Retrieve&dopt=full_report&list_uids=10117) | enamelin |
| [Details](http://mirdb.org/cgi-bin/target_detail.cgi?targetID=1232185) | 904 | 51 | hsa-let-7d-5p | [MAP3K3](http://www.ncbi.nlm.nih.gov/entrez/query.fcgi?db=gene&cmd=Retrieve&dopt=full_report&list_uids=4215) | mitogen-activated protein kinase kinase kinase 3 |
| [Details](http://mirdb.org/cgi-bin/target_detail.cgi?targetID=1232288) | 905 | 51 | hsa-let-7d-5p | [GRIN3A](http://www.ncbi.nlm.nih.gov/entrez/query.fcgi?db=gene&cmd=Retrieve&dopt=full_report&list_uids=116443) | glutamate ionotropic receptor NMDA type subunit 3A |
| [Details](http://mirdb.org/cgi-bin/target_detail.cgi?targetID=1231696) | 906 | 51 | hsa-let-7d-5p | [KHNYN](http://www.ncbi.nlm.nih.gov/entrez/query.fcgi?db=gene&cmd=Retrieve&dopt=full_report&list_uids=23351) | KH and NYN domain containing |
| [Details](http://mirdb.org/cgi-bin/target_detail.cgi?targetID=1231728) | 907 | 50 | hsa-let-7d-5p | [RNF38](http://www.ncbi.nlm.nih.gov/entrez/query.fcgi?db=gene&cmd=Retrieve&dopt=full_report&list_uids=152006) | ring finger protein 38 |
| [Details](http://mirdb.org/cgi-bin/target_detail.cgi?targetID=1231472) | 908 | 50 | hsa-let-7d-5p | [SMAD2](http://www.ncbi.nlm.nih.gov/entrez/query.fcgi?db=gene&cmd=Retrieve&dopt=full_report&list_uids=4087) | SMAD family member 2 |
| [Details](http://mirdb.org/cgi-bin/target_detail.cgi?targetID=1231911) | 909 | 50 | hsa-let-7d-5p | [MBTPS2](http://www.ncbi.nlm.nih.gov/entrez/query.fcgi?db=gene&cmd=Retrieve&dopt=full_report&list_uids=51360) | membrane bound transcription factor peptidase, site 2 |
| [Details](http://mirdb.org/cgi-bin/target_detail.cgi?targetID=1231916) | 910 | 50 | hsa-let-7d-5p | [ZNF799](http://www.ncbi.nlm.nih.gov/entrez/query.fcgi?db=gene&cmd=Retrieve&dopt=full_report&list_uids=90576) | zinc finger protein 799 |
| [Details](http://mirdb.org/cgi-bin/target_detail.cgi?targetID=1231733) | 911 | 50 | hsa-let-7d-5p | [SLC8A2](http://www.ncbi.nlm.nih.gov/entrez/query.fcgi?db=gene&cmd=Retrieve&dopt=full_report&list_uids=6543) | solute carrier family 8 member A2 |
| [Details](http://mirdb.org/cgi-bin/target_detail.cgi?targetID=1231652) | 912 | 50 | hsa-let-7d-5p | [FAM180A](http://www.ncbi.nlm.nih.gov/entrez/query.fcgi?db=gene&cmd=Retrieve&dopt=full_report&list_uids=389558) | family with sequence similarity 180 member A |
| [Details](http://mirdb.org/cgi-bin/target_detail.cgi?targetID=1232380) | 913 | 50 | hsa-let-7d-5p | [PRDM1](http://www.ncbi.nlm.nih.gov/entrez/query.fcgi?db=gene&cmd=Retrieve&dopt=full_report&list_uids=639) | PR/SET domain 1 |
| [Details](http://mirdb.org/cgi-bin/target_detail.cgi?targetID=1232351) | 914 | 50 | hsa-let-7d-5p | [ADIPOR2](http://www.ncbi.nlm.nih.gov/entrez/query.fcgi?db=gene&cmd=Retrieve&dopt=full_report&list_uids=79602) | adiponectin receptor 2 |
| [Details](http://mirdb.org/cgi-bin/target_detail.cgi?targetID=1232136) | 915 | 50 | hsa-let-7d-5p | [RASA4](http://www.ncbi.nlm.nih.gov/entrez/query.fcgi?db=gene&cmd=Retrieve&dopt=full_report&list_uids=10156) | RAS p21 protein activator 4 |
| [Details](http://mirdb.org/cgi-bin/target_detail.cgi?targetID=1231996) | 916 | 50 | hsa-let-7d-5p | [WARS2](http://www.ncbi.nlm.nih.gov/entrez/query.fcgi?db=gene&cmd=Retrieve&dopt=full_report&list_uids=10352) | tryptophanyl tRNA synthetase 2, mitochondrial |
| [Details](http://mirdb.org/cgi-bin/target_detail.cgi?targetID=1231973) | 917 | 50 | hsa-let-7d-5p | [MGLL](http://www.ncbi.nlm.nih.gov/entrez/query.fcgi?db=gene&cmd=Retrieve&dopt=full_report&list_uids=11343) | monoglyceride lipase |
| [Details](http://mirdb.org/cgi-bin/target_detail.cgi?targetID=1231806) | 918 | 50 | hsa-let-7d-5p | [SYT1](http://www.ncbi.nlm.nih.gov/entrez/query.fcgi?db=gene&cmd=Retrieve&dopt=full_report&list_uids=6857) | synaptotagmin 1 |
| [Details](http://mirdb.org/cgi-bin/target_detail.cgi?targetID=1231486) | 919 | 50 | hsa-let-7d-5p | [TUT4](http://www.ncbi.nlm.nih.gov/entrez/query.fcgi?db=gene&cmd=Retrieve&dopt=full_report&list_uids=23318) | terminal uridylyl transferase 4 |
| [Details](http://mirdb.org/cgi-bin/target_detail.cgi?targetID=1231952) | 920 | 50 | hsa-let-7d-5p | [FBXO22](http://www.ncbi.nlm.nih.gov/entrez/query.fcgi?db=gene&cmd=Retrieve&dopt=full_report&list_uids=26263) | F-box protein 22 |
| [Details](http://mirdb.org/cgi-bin/target_detail.cgi?targetID=1232057) | 921 | 50 | hsa-let-7d-5p | [ZNF443](http://www.ncbi.nlm.nih.gov/entrez/query.fcgi?db=gene&cmd=Retrieve&dopt=full_report&list_uids=10224) | zinc finger protein 443 |
| [Details](http://mirdb.org/cgi-bin/target_detail.cgi?targetID=1231476) | 922 | 50 | hsa-let-7d-5p | [PANX2](http://www.ncbi.nlm.nih.gov/entrez/query.fcgi?db=gene&cmd=Retrieve&dopt=full_report&list_uids=56666) | pannexin 2 |
| [Details](http://mirdb.org/cgi-bin/target_detail.cgi?targetID=1231962) | 923 | 50 | hsa-let-7d-5p | [CHRD](http://www.ncbi.nlm.nih.gov/entrez/query.fcgi?db=gene&cmd=Retrieve&dopt=full_report&list_uids=8646) | chordin |

Data generated from the online database for prediction of functional microRNA targets (Chen & Wang, 2020)

Chen, Y., & Wang, X. (2020). MiRDB: An online database for prediction of functional microRNA targets. *Nucleic Acids Research*, *48*(D1), D127–D131. https://doi.org/10.1093/nar/gkz757
