# Supplementary material for: The Role of microRNAs in Organismal and Skin Aging
Source: Int J Mol Sci. 2020 Jul 25;21(15):5281. doi: 10.3390/ijms21155281 (PMC7432402; doi:10.3390/ijms21155281)
Supplement: Supplementary file 1 [file ijms-21-05281-s001.zip › Appendix 6 hsa-miR-27a-3p .docx]

**There are 1497 predicted targets for hsa-miR-27a-3p in miRDB**

| **Target Detail** | **Target Rank** | **Target Score** | **miRNA Name** | **Gene Symbol** | **Gene Description** |
| --- | --- | --- | --- | --- | --- |
| [Details](http://mirdb.org/cgi-bin/target_detail.cgi?targetID=3072768) | 1 | 100 | hsa-miR-27a-3p | [AFF4](http://www.ncbi.nlm.nih.gov/entrez/query.fcgi?db=gene&cmd=Retrieve&dopt=full_report&list_uids=27125) | AF4/FMR2 family member 4 |
| [Details](http://mirdb.org/cgi-bin/target_detail.cgi?targetID=3072941) | 2 | 100 | hsa-miR-27a-3p | [GXYLT1](http://www.ncbi.nlm.nih.gov/entrez/query.fcgi?db=gene&cmd=Retrieve&dopt=full_report&list_uids=283464) | glucoside xylosyltransferase 1 |
| [Details](http://mirdb.org/cgi-bin/target_detail.cgi?targetID=3073265) | 3 | 100 | hsa-miR-27a-3p | [ARFGEF1](http://www.ncbi.nlm.nih.gov/entrez/query.fcgi?db=gene&cmd=Retrieve&dopt=full_report&list_uids=10565) | ADP ribosylation factor guanine nucleotide exchange factor 1 |
| [Details](http://mirdb.org/cgi-bin/target_detail.cgi?targetID=3073312) | 4 | 100 | hsa-miR-27a-3p | [GCC2](http://www.ncbi.nlm.nih.gov/entrez/query.fcgi?db=gene&cmd=Retrieve&dopt=full_report&list_uids=9648) | GRIP and coiled-coil domain containing 2 |
| [Details](http://mirdb.org/cgi-bin/target_detail.cgi?targetID=3073378) | 5 | 100 | hsa-miR-27a-3p | [DCUN1D4](http://www.ncbi.nlm.nih.gov/entrez/query.fcgi?db=gene&cmd=Retrieve&dopt=full_report&list_uids=23142) | defective in cullin neddylation 1 domain containing 4 |
| [Details](http://mirdb.org/cgi-bin/target_detail.cgi?targetID=3073493) | 6 | 100 | hsa-miR-27a-3p | [PLK2](http://www.ncbi.nlm.nih.gov/entrez/query.fcgi?db=gene&cmd=Retrieve&dopt=full_report&list_uids=10769) | polo like kinase 2 |
| [Details](http://mirdb.org/cgi-bin/target_detail.cgi?targetID=3073522) | 7 | 100 | hsa-miR-27a-3p | [TNPO1](http://www.ncbi.nlm.nih.gov/entrez/query.fcgi?db=gene&cmd=Retrieve&dopt=full_report&list_uids=3842) | transportin 1 |
| [Details](http://mirdb.org/cgi-bin/target_detail.cgi?targetID=3073684) | 8 | 100 | hsa-miR-27a-3p | [TRPV3](http://www.ncbi.nlm.nih.gov/entrez/query.fcgi?db=gene&cmd=Retrieve&dopt=full_report&list_uids=162514) | transient receptor potential cation channel subfamily V member 3 |
| [Details](http://mirdb.org/cgi-bin/target_detail.cgi?targetID=3073764) | 9 | 100 | hsa-miR-27a-3p | [GAB1](http://www.ncbi.nlm.nih.gov/entrez/query.fcgi?db=gene&cmd=Retrieve&dopt=full_report&list_uids=2549) | GRB2 associated binding protein 1 |
| [Details](http://mirdb.org/cgi-bin/target_detail.cgi?targetID=3072557) | 10 | 99 | hsa-miR-27a-3p | [GRIA4](http://www.ncbi.nlm.nih.gov/entrez/query.fcgi?db=gene&cmd=Retrieve&dopt=full_report&list_uids=2893) | glutamate ionotropic receptor AMPA type subunit 4 |
| [Details](http://mirdb.org/cgi-bin/target_detail.cgi?targetID=3072572) | 11 | 99 | hsa-miR-27a-3p | [BEX3](http://www.ncbi.nlm.nih.gov/entrez/query.fcgi?db=gene&cmd=Retrieve&dopt=full_report&list_uids=27018) | brain expressed X-linked 3 |
| [Details](http://mirdb.org/cgi-bin/target_detail.cgi?targetID=3072573) | 12 | 99 | hsa-miR-27a-3p | [MBTD1](http://www.ncbi.nlm.nih.gov/entrez/query.fcgi?db=gene&cmd=Retrieve&dopt=full_report&list_uids=54799) | mbt domain containing 1 |
| [Details](http://mirdb.org/cgi-bin/target_detail.cgi?targetID=3072643) | 13 | 99 | hsa-miR-27a-3p | [PDS5B](http://www.ncbi.nlm.nih.gov/entrez/query.fcgi?db=gene&cmd=Retrieve&dopt=full_report&list_uids=23047) | PDS5 cohesin associated factor B |
| [Details](http://mirdb.org/cgi-bin/target_detail.cgi?targetID=3072793) | 14 | 99 | hsa-miR-27a-3p | [AKIRIN1](http://www.ncbi.nlm.nih.gov/entrez/query.fcgi?db=gene&cmd=Retrieve&dopt=full_report&list_uids=79647) | akirin 1 |
| [Details](http://mirdb.org/cgi-bin/target_detail.cgi?targetID=3072979) | 15 | 99 | hsa-miR-27a-3p | [RPS6KA5](http://www.ncbi.nlm.nih.gov/entrez/query.fcgi?db=gene&cmd=Retrieve&dopt=full_report&list_uids=9252) | ribosomal protein S6 kinase A5 |
| [Details](http://mirdb.org/cgi-bin/target_detail.cgi?targetID=3073123) | 16 | 99 | hsa-miR-27a-3p | [RGPD6](http://www.ncbi.nlm.nih.gov/entrez/query.fcgi?db=gene&cmd=Retrieve&dopt=full_report&list_uids=729540) | RANBP2-like and GRIP domain containing 6 |
| [Details](http://mirdb.org/cgi-bin/target_detail.cgi?targetID=3073279) | 17 | 99 | hsa-miR-27a-3p | [PHLPP2](http://www.ncbi.nlm.nih.gov/entrez/query.fcgi?db=gene&cmd=Retrieve&dopt=full_report&list_uids=23035) | PH domain and leucine rich repeat protein phosphatase 2 |
| [Details](http://mirdb.org/cgi-bin/target_detail.cgi?targetID=3073289) | 18 | 99 | hsa-miR-27a-3p | [TAB3](http://www.ncbi.nlm.nih.gov/entrez/query.fcgi?db=gene&cmd=Retrieve&dopt=full_report&list_uids=257397) | TGF-beta activated kinase 1 (MAP3K7) binding protein 3 |
| [Details](http://mirdb.org/cgi-bin/target_detail.cgi?targetID=3073338) | 19 | 99 | hsa-miR-27a-3p | [SOS1](http://www.ncbi.nlm.nih.gov/entrez/query.fcgi?db=gene&cmd=Retrieve&dopt=full_report&list_uids=6654) | SOS Ras/Rac guanine nucleotide exchange factor 1 |
| [Details](http://mirdb.org/cgi-bin/target_detail.cgi?targetID=3073369) | 20 | 99 | hsa-miR-27a-3p | [FBXW7](http://www.ncbi.nlm.nih.gov/entrez/query.fcgi?db=gene&cmd=Retrieve&dopt=full_report&list_uids=55294) | F-box and WD repeat domain containing 7 |
| [Details](http://mirdb.org/cgi-bin/target_detail.cgi?targetID=3073412) | 21 | 99 | hsa-miR-27a-3p | [CDS1](http://www.ncbi.nlm.nih.gov/entrez/query.fcgi?db=gene&cmd=Retrieve&dopt=full_report&list_uids=1040) | CDP-diacylglycerol synthase 1 |
| [Details](http://mirdb.org/cgi-bin/target_detail.cgi?targetID=3073627) | 22 | 99 | hsa-miR-27a-3p | [RGPD5](http://www.ncbi.nlm.nih.gov/entrez/query.fcgi?db=gene&cmd=Retrieve&dopt=full_report&list_uids=84220) | RANBP2-like and GRIP domain containing 5 |
| [Details](http://mirdb.org/cgi-bin/target_detail.cgi?targetID=3073654) | 23 | 99 | hsa-miR-27a-3p | [SEMA6A](http://www.ncbi.nlm.nih.gov/entrez/query.fcgi?db=gene&cmd=Retrieve&dopt=full_report&list_uids=57556) | semaphorin 6A |
| [Details](http://mirdb.org/cgi-bin/target_detail.cgi?targetID=3073666) | 24 | 99 | hsa-miR-27a-3p | [NRK](http://www.ncbi.nlm.nih.gov/entrez/query.fcgi?db=gene&cmd=Retrieve&dopt=full_report&list_uids=203447) | Nik related kinase |
| [Details](http://mirdb.org/cgi-bin/target_detail.cgi?targetID=3072475) | 25 | 98 | hsa-miR-27a-3p | [USP42](http://www.ncbi.nlm.nih.gov/entrez/query.fcgi?db=gene&cmd=Retrieve&dopt=full_report&list_uids=84132) | ubiquitin specific peptidase 42 |
| [Details](http://mirdb.org/cgi-bin/target_detail.cgi?targetID=3072669) | 26 | 98 | hsa-miR-27a-3p | [ABHD17C](http://www.ncbi.nlm.nih.gov/entrez/query.fcgi?db=gene&cmd=Retrieve&dopt=full_report&list_uids=58489) | abhydrolase domain containing 17C |
| [Details](http://mirdb.org/cgi-bin/target_detail.cgi?targetID=3072693) | 27 | 98 | hsa-miR-27a-3p | [GPAM](http://www.ncbi.nlm.nih.gov/entrez/query.fcgi?db=gene&cmd=Retrieve&dopt=full_report&list_uids=57678) | glycerol-3-phosphate acyltransferase, mitochondrial |
| [Details](http://mirdb.org/cgi-bin/target_detail.cgi?targetID=3072755) | 28 | 98 | hsa-miR-27a-3p | [ARHGEF26](http://www.ncbi.nlm.nih.gov/entrez/query.fcgi?db=gene&cmd=Retrieve&dopt=full_report&list_uids=26084) | Rho guanine nucleotide exchange factor 26 |
| [Details](http://mirdb.org/cgi-bin/target_detail.cgi?targetID=3072827) | 29 | 98 | hsa-miR-27a-3p | [RGPD4](http://www.ncbi.nlm.nih.gov/entrez/query.fcgi?db=gene&cmd=Retrieve&dopt=full_report&list_uids=285190) | RANBP2-like and GRIP domain containing 4 |
| [Details](http://mirdb.org/cgi-bin/target_detail.cgi?targetID=3072832) | 30 | 98 | hsa-miR-27a-3p | [KCNK2](http://www.ncbi.nlm.nih.gov/entrez/query.fcgi?db=gene&cmd=Retrieve&dopt=full_report&list_uids=3776) | potassium two pore domain channel subfamily K member 2 |
| [Details](http://mirdb.org/cgi-bin/target_detail.cgi?targetID=3072839) | 31 | 98 | hsa-miR-27a-3p | [EYA1](http://www.ncbi.nlm.nih.gov/entrez/query.fcgi?db=gene&cmd=Retrieve&dopt=full_report&list_uids=2138) | EYA transcriptional coactivator and phosphatase 1 |
| [Details](http://mirdb.org/cgi-bin/target_detail.cgi?targetID=3072921) | 32 | 98 | hsa-miR-27a-3p | [RGPD8](http://www.ncbi.nlm.nih.gov/entrez/query.fcgi?db=gene&cmd=Retrieve&dopt=full_report&list_uids=727851) | RANBP2-like and GRIP domain containing 8 |
| [Details](http://mirdb.org/cgi-bin/target_detail.cgi?targetID=3073316) | 33 | 98 | hsa-miR-27a-3p | [ZBTB34](http://www.ncbi.nlm.nih.gov/entrez/query.fcgi?db=gene&cmd=Retrieve&dopt=full_report&list_uids=403341) | zinc finger and BTB domain containing 34 |
| [Details](http://mirdb.org/cgi-bin/target_detail.cgi?targetID=3073362) | 34 | 98 | hsa-miR-27a-3p | [KIAA1109](http://www.ncbi.nlm.nih.gov/entrez/query.fcgi?db=gene&cmd=Retrieve&dopt=full_report&list_uids=84162) | KIAA1109 |
| [Details](http://mirdb.org/cgi-bin/target_detail.cgi?targetID=3073598) | 35 | 98 | hsa-miR-27a-3p | [ST6GALNAC3](http://www.ncbi.nlm.nih.gov/entrez/query.fcgi?db=gene&cmd=Retrieve&dopt=full_report&list_uids=256435) | ST6 N-acetylgalactosaminide alpha-2,6-sialyltransferase 3 |
| [Details](http://mirdb.org/cgi-bin/target_detail.cgi?targetID=3073754) | 36 | 98 | hsa-miR-27a-3p | [EYA4](http://www.ncbi.nlm.nih.gov/entrez/query.fcgi?db=gene&cmd=Retrieve&dopt=full_report&list_uids=2070) | EYA transcriptional coactivator and phosphatase 4 |
| [Details](http://mirdb.org/cgi-bin/target_detail.cgi?targetID=3073792) | 37 | 98 | hsa-miR-27a-3p | [PLEKHJ1](http://www.ncbi.nlm.nih.gov/entrez/query.fcgi?db=gene&cmd=Retrieve&dopt=full_report&list_uids=55111) | pleckstrin homology domain containing J1 |
| [Details](http://mirdb.org/cgi-bin/target_detail.cgi?targetID=3072427) | 38 | 97 | hsa-miR-27a-3p | [SBF2](http://www.ncbi.nlm.nih.gov/entrez/query.fcgi?db=gene&cmd=Retrieve&dopt=full_report&list_uids=81846) | SET binding factor 2 |
| [Details](http://mirdb.org/cgi-bin/target_detail.cgi?targetID=3072599) | 39 | 97 | hsa-miR-27a-3p | [PRR3](http://www.ncbi.nlm.nih.gov/entrez/query.fcgi?db=gene&cmd=Retrieve&dopt=full_report&list_uids=80742) | proline rich 3 |
| [Details](http://mirdb.org/cgi-bin/target_detail.cgi?targetID=3072660) | 40 | 97 | hsa-miR-27a-3p | [ONECUT2](http://www.ncbi.nlm.nih.gov/entrez/query.fcgi?db=gene&cmd=Retrieve&dopt=full_report&list_uids=9480) | one cut homeobox 2 |
| [Details](http://mirdb.org/cgi-bin/target_detail.cgi?targetID=3072687) | 41 | 97 | hsa-miR-27a-3p | [TMBIM6](http://www.ncbi.nlm.nih.gov/entrez/query.fcgi?db=gene&cmd=Retrieve&dopt=full_report&list_uids=7009) | transmembrane BAX inhibitor motif containing 6 |
| [Details](http://mirdb.org/cgi-bin/target_detail.cgi?targetID=3072713) | 42 | 97 | hsa-miR-27a-3p | [FOXA3](http://www.ncbi.nlm.nih.gov/entrez/query.fcgi?db=gene&cmd=Retrieve&dopt=full_report&list_uids=3171) | forkhead box A3 |
| [Details](http://mirdb.org/cgi-bin/target_detail.cgi?targetID=3072731) | 43 | 97 | hsa-miR-27a-3p | [BTG2](http://www.ncbi.nlm.nih.gov/entrez/query.fcgi?db=gene&cmd=Retrieve&dopt=full_report&list_uids=7832) | BTG anti-proliferation factor 2 |
| [Details](http://mirdb.org/cgi-bin/target_detail.cgi?targetID=3072743) | 44 | 97 | hsa-miR-27a-3p | [ADAMTSL3](http://www.ncbi.nlm.nih.gov/entrez/query.fcgi?db=gene&cmd=Retrieve&dopt=full_report&list_uids=57188) | ADAMTS like 3 |
| [Details](http://mirdb.org/cgi-bin/target_detail.cgi?targetID=3072859) | 45 | 97 | hsa-miR-27a-3p | [RUNX1](http://www.ncbi.nlm.nih.gov/entrez/query.fcgi?db=gene&cmd=Retrieve&dopt=full_report&list_uids=861) | runt related transcription factor 1 |
| [Details](http://mirdb.org/cgi-bin/target_detail.cgi?targetID=3072950) | 46 | 97 | hsa-miR-27a-3p | [ACVR1C](http://www.ncbi.nlm.nih.gov/entrez/query.fcgi?db=gene&cmd=Retrieve&dopt=full_report&list_uids=130399) | activin A receptor type 1C |
| [Details](http://mirdb.org/cgi-bin/target_detail.cgi?targetID=3072970) | 47 | 97 | hsa-miR-27a-3p | [TMEM170B](http://www.ncbi.nlm.nih.gov/entrez/query.fcgi?db=gene&cmd=Retrieve&dopt=full_report&list_uids=100113407) | transmembrane protein 170B |
| [Details](http://mirdb.org/cgi-bin/target_detail.cgi?targetID=3073013) | 48 | 97 | hsa-miR-27a-3p | [TMCC1](http://www.ncbi.nlm.nih.gov/entrez/query.fcgi?db=gene&cmd=Retrieve&dopt=full_report&list_uids=23023) | transmembrane and coiled-coil domain family 1 |
| [Details](http://mirdb.org/cgi-bin/target_detail.cgi?targetID=3073056) | 49 | 97 | hsa-miR-27a-3p | [EHF](http://www.ncbi.nlm.nih.gov/entrez/query.fcgi?db=gene&cmd=Retrieve&dopt=full_report&list_uids=26298) | ETS homologous factor |
| [Details](http://mirdb.org/cgi-bin/target_detail.cgi?targetID=3073162) | 50 | 97 | hsa-miR-27a-3p | [GRIN2D](http://www.ncbi.nlm.nih.gov/entrez/query.fcgi?db=gene&cmd=Retrieve&dopt=full_report&list_uids=2906) | glutamate ionotropic receptor NMDA type subunit 2D |
| [Details](http://mirdb.org/cgi-bin/target_detail.cgi?targetID=3073235) | 51 | 97 | hsa-miR-27a-3p | [USP46](http://www.ncbi.nlm.nih.gov/entrez/query.fcgi?db=gene&cmd=Retrieve&dopt=full_report&list_uids=64854) | ubiquitin specific peptidase 46 |
| [Details](http://mirdb.org/cgi-bin/target_detail.cgi?targetID=3073293) | 52 | 97 | hsa-miR-27a-3p | [C2CD2](http://www.ncbi.nlm.nih.gov/entrez/query.fcgi?db=gene&cmd=Retrieve&dopt=full_report&list_uids=25966) | C2 calcium dependent domain containing 2 |
| [Details](http://mirdb.org/cgi-bin/target_detail.cgi?targetID=3073328) | 53 | 97 | hsa-miR-27a-3p | [WNK3](http://www.ncbi.nlm.nih.gov/entrez/query.fcgi?db=gene&cmd=Retrieve&dopt=full_report&list_uids=65267) | WNK lysine deficient protein kinase 3 |
| [Details](http://mirdb.org/cgi-bin/target_detail.cgi?targetID=3073505) | 54 | 97 | hsa-miR-27a-3p | [MMD](http://www.ncbi.nlm.nih.gov/entrez/query.fcgi?db=gene&cmd=Retrieve&dopt=full_report&list_uids=23531) | monocyte to macrophage differentiation associated |
| [Details](http://mirdb.org/cgi-bin/target_detail.cgi?targetID=3073577) | 55 | 97 | hsa-miR-27a-3p | [HOXA5](http://www.ncbi.nlm.nih.gov/entrez/query.fcgi?db=gene&cmd=Retrieve&dopt=full_report&list_uids=3202) | homeobox A5 |
| [Details](http://mirdb.org/cgi-bin/target_detail.cgi?targetID=3073689) | 56 | 97 | hsa-miR-27a-3p | [SLC7A11](http://www.ncbi.nlm.nih.gov/entrez/query.fcgi?db=gene&cmd=Retrieve&dopt=full_report&list_uids=23657) | solute carrier family 7 member 11 |
| [Details](http://mirdb.org/cgi-bin/target_detail.cgi?targetID=3073779) | 57 | 97 | hsa-miR-27a-3p | [RNF139](http://www.ncbi.nlm.nih.gov/entrez/query.fcgi?db=gene&cmd=Retrieve&dopt=full_report&list_uids=11236) | ring finger protein 139 |
| [Details](http://mirdb.org/cgi-bin/target_detail.cgi?targetID=3073802) | 58 | 97 | hsa-miR-27a-3p | [GATC](http://www.ncbi.nlm.nih.gov/entrez/query.fcgi?db=gene&cmd=Retrieve&dopt=full_report&list_uids=283459) | glutamyl-tRNA amidotransferase subunit C |
| [Details](http://mirdb.org/cgi-bin/target_detail.cgi?targetID=3073851) | 59 | 97 | hsa-miR-27a-3p | [DNAJC27](http://www.ncbi.nlm.nih.gov/entrez/query.fcgi?db=gene&cmd=Retrieve&dopt=full_report&list_uids=51277) | DnaJ heat shock protein family (Hsp40) member C27 |
| [Details](http://mirdb.org/cgi-bin/target_detail.cgi?targetID=3073876) | 60 | 97 | hsa-miR-27a-3p | [KDM7A](http://www.ncbi.nlm.nih.gov/entrez/query.fcgi?db=gene&cmd=Retrieve&dopt=full_report&list_uids=80853) | lysine demethylase 7A |
| [Details](http://mirdb.org/cgi-bin/target_detail.cgi?targetID=3072438) | 61 | 96 | hsa-miR-27a-3p | [PNKD](http://www.ncbi.nlm.nih.gov/entrez/query.fcgi?db=gene&cmd=Retrieve&dopt=full_report&list_uids=25953) | PNKD, MBL domain containing |
| [Details](http://mirdb.org/cgi-bin/target_detail.cgi?targetID=3072485) | 62 | 96 | hsa-miR-27a-3p | [KCTD8](http://www.ncbi.nlm.nih.gov/entrez/query.fcgi?db=gene&cmd=Retrieve&dopt=full_report&list_uids=386617) | potassium channel tetramerization domain containing 8 |
| [Details](http://mirdb.org/cgi-bin/target_detail.cgi?targetID=3072562) | 63 | 96 | hsa-miR-27a-3p | [SLITRK1](http://www.ncbi.nlm.nih.gov/entrez/query.fcgi?db=gene&cmd=Retrieve&dopt=full_report&list_uids=114798) | SLIT and NTRK like family member 1 |
| [Details](http://mirdb.org/cgi-bin/target_detail.cgi?targetID=3072614) | 64 | 96 | hsa-miR-27a-3p | [PDIA5](http://www.ncbi.nlm.nih.gov/entrez/query.fcgi?db=gene&cmd=Retrieve&dopt=full_report&list_uids=10954) | protein disulfide isomerase family A member 5 |
| [Details](http://mirdb.org/cgi-bin/target_detail.cgi?targetID=3072624) | 65 | 96 | hsa-miR-27a-3p | [ABCA1](http://www.ncbi.nlm.nih.gov/entrez/query.fcgi?db=gene&cmd=Retrieve&dopt=full_report&list_uids=19) | ATP binding cassette subfamily A member 1 |
| [Details](http://mirdb.org/cgi-bin/target_detail.cgi?targetID=3072637) | 66 | 96 | hsa-miR-27a-3p | [EDRF1](http://www.ncbi.nlm.nih.gov/entrez/query.fcgi?db=gene&cmd=Retrieve&dopt=full_report&list_uids=26098) | erythroid differentiation regulatory factor 1 |
| [Details](http://mirdb.org/cgi-bin/target_detail.cgi?targetID=3072642) | 67 | 96 | hsa-miR-27a-3p | [KLF3](http://www.ncbi.nlm.nih.gov/entrez/query.fcgi?db=gene&cmd=Retrieve&dopt=full_report&list_uids=51274) | Kruppel like factor 3 |
| [Details](http://mirdb.org/cgi-bin/target_detail.cgi?targetID=3072648) | 68 | 96 | hsa-miR-27a-3p | [SMAD9](http://www.ncbi.nlm.nih.gov/entrez/query.fcgi?db=gene&cmd=Retrieve&dopt=full_report&list_uids=4093) | SMAD family member 9 |
| [Details](http://mirdb.org/cgi-bin/target_detail.cgi?targetID=3072680) | 69 | 96 | hsa-miR-27a-3p | [TFAP2B](http://www.ncbi.nlm.nih.gov/entrez/query.fcgi?db=gene&cmd=Retrieve&dopt=full_report&list_uids=7021) | transcription factor AP-2 beta |
| [Details](http://mirdb.org/cgi-bin/target_detail.cgi?targetID=3072683) | 70 | 96 | hsa-miR-27a-3p | [HIPK2](http://www.ncbi.nlm.nih.gov/entrez/query.fcgi?db=gene&cmd=Retrieve&dopt=full_report&list_uids=28996) | homeodomain interacting protein kinase 2 |
| [Details](http://mirdb.org/cgi-bin/target_detail.cgi?targetID=3072694) | 71 | 96 | hsa-miR-27a-3p | [UNKL](http://www.ncbi.nlm.nih.gov/entrez/query.fcgi?db=gene&cmd=Retrieve&dopt=full_report&list_uids=64718) | unk like zinc finger |
| [Details](http://mirdb.org/cgi-bin/target_detail.cgi?targetID=3072727) | 72 | 96 | hsa-miR-27a-3p | [PLEKHH1](http://www.ncbi.nlm.nih.gov/entrez/query.fcgi?db=gene&cmd=Retrieve&dopt=full_report&list_uids=57475) | pleckstrin homology, MyTH4 and FERM domain containing H1 |
| [Details](http://mirdb.org/cgi-bin/target_detail.cgi?targetID=3072752) | 73 | 96 | hsa-miR-27a-3p | [PLCL2](http://www.ncbi.nlm.nih.gov/entrez/query.fcgi?db=gene&cmd=Retrieve&dopt=full_report&list_uids=23228) | phospholipase C like 2 |
| [Details](http://mirdb.org/cgi-bin/target_detail.cgi?targetID=3072808) | 74 | 96 | hsa-miR-27a-3p | [ROR1](http://www.ncbi.nlm.nih.gov/entrez/query.fcgi?db=gene&cmd=Retrieve&dopt=full_report&list_uids=4919) | receptor tyrosine kinase like orphan receptor 1 |
| [Details](http://mirdb.org/cgi-bin/target_detail.cgi?targetID=3072853) | 75 | 96 | hsa-miR-27a-3p | [SEMA7A](http://www.ncbi.nlm.nih.gov/entrez/query.fcgi?db=gene&cmd=Retrieve&dopt=full_report&list_uids=8482) | semaphorin 7A (John Milton Hagen blood group) |
| [Details](http://mirdb.org/cgi-bin/target_detail.cgi?targetID=3072919) | 76 | 96 | hsa-miR-27a-3p | [CKAP4](http://www.ncbi.nlm.nih.gov/entrez/query.fcgi?db=gene&cmd=Retrieve&dopt=full_report&list_uids=10970) | cytoskeleton associated protein 4 |
| [Details](http://mirdb.org/cgi-bin/target_detail.cgi?targetID=3072952) | 77 | 96 | hsa-miR-27a-3p | [VAV2](http://www.ncbi.nlm.nih.gov/entrez/query.fcgi?db=gene&cmd=Retrieve&dopt=full_report&list_uids=7410) | vav guanine nucleotide exchange factor 2 |
| [Details](http://mirdb.org/cgi-bin/target_detail.cgi?targetID=3073101) | 78 | 96 | hsa-miR-27a-3p | [CDR2](http://www.ncbi.nlm.nih.gov/entrez/query.fcgi?db=gene&cmd=Retrieve&dopt=full_report&list_uids=1039) | cerebellar degeneration related protein 2 |
| [Details](http://mirdb.org/cgi-bin/target_detail.cgi?targetID=3073136) | 79 | 96 | hsa-miR-27a-3p | [FBXO10](http://www.ncbi.nlm.nih.gov/entrez/query.fcgi?db=gene&cmd=Retrieve&dopt=full_report&list_uids=26267) | F-box protein 10 |
| [Details](http://mirdb.org/cgi-bin/target_detail.cgi?targetID=3073195) | 80 | 96 | hsa-miR-27a-3p | [LIFR](http://www.ncbi.nlm.nih.gov/entrez/query.fcgi?db=gene&cmd=Retrieve&dopt=full_report&list_uids=3977) | LIF receptor alpha |
| [Details](http://mirdb.org/cgi-bin/target_detail.cgi?targetID=3073220) | 81 | 96 | hsa-miR-27a-3p | [ABHD6](http://www.ncbi.nlm.nih.gov/entrez/query.fcgi?db=gene&cmd=Retrieve&dopt=full_report&list_uids=57406) | abhydrolase domain containing 6 |
| [Details](http://mirdb.org/cgi-bin/target_detail.cgi?targetID=3073222) | 82 | 96 | hsa-miR-27a-3p | [ZFHX3](http://www.ncbi.nlm.nih.gov/entrez/query.fcgi?db=gene&cmd=Retrieve&dopt=full_report&list_uids=463) | zinc finger homeobox 3 |
| [Details](http://mirdb.org/cgi-bin/target_detail.cgi?targetID=3073253) | 83 | 96 | hsa-miR-27a-3p | [ADORA2B](http://www.ncbi.nlm.nih.gov/entrez/query.fcgi?db=gene&cmd=Retrieve&dopt=full_report&list_uids=136) | adenosine A2b receptor |
| [Details](http://mirdb.org/cgi-bin/target_detail.cgi?targetID=3073254) | 84 | 96 | hsa-miR-27a-3p | [GOLM1](http://www.ncbi.nlm.nih.gov/entrez/query.fcgi?db=gene&cmd=Retrieve&dopt=full_report&list_uids=51280) | golgi membrane protein 1 |
| [Details](http://mirdb.org/cgi-bin/target_detail.cgi?targetID=3073329) | 85 | 96 | hsa-miR-27a-3p | [EPS8](http://www.ncbi.nlm.nih.gov/entrez/query.fcgi?db=gene&cmd=Retrieve&dopt=full_report&list_uids=2059) | epidermal growth factor receptor pathway substrate 8 |
| [Details](http://mirdb.org/cgi-bin/target_detail.cgi?targetID=3073334) | 86 | 96 | hsa-miR-27a-3p | [CCNK](http://www.ncbi.nlm.nih.gov/entrez/query.fcgi?db=gene&cmd=Retrieve&dopt=full_report&list_uids=8812) | cyclin K |
| [Details](http://mirdb.org/cgi-bin/target_detail.cgi?targetID=3073461) | 87 | 96 | hsa-miR-27a-3p | [UGCG](http://www.ncbi.nlm.nih.gov/entrez/query.fcgi?db=gene&cmd=Retrieve&dopt=full_report&list_uids=7357) | UDP-glucose ceramide glucosyltransferase |
| [Details](http://mirdb.org/cgi-bin/target_detail.cgi?targetID=3073575) | 88 | 96 | hsa-miR-27a-3p | [IPMK](http://www.ncbi.nlm.nih.gov/entrez/query.fcgi?db=gene&cmd=Retrieve&dopt=full_report&list_uids=253430) | inositol polyphosphate multikinase |
| [Details](http://mirdb.org/cgi-bin/target_detail.cgi?targetID=3073591) | 89 | 96 | hsa-miR-27a-3p | [HIVEP3](http://www.ncbi.nlm.nih.gov/entrez/query.fcgi?db=gene&cmd=Retrieve&dopt=full_report&list_uids=59269) | human immunodeficiency virus type I enhancer binding protein 3 |
| [Details](http://mirdb.org/cgi-bin/target_detail.cgi?targetID=3073608) | 90 | 96 | hsa-miR-27a-3p | [PEG10](http://www.ncbi.nlm.nih.gov/entrez/query.fcgi?db=gene&cmd=Retrieve&dopt=full_report&list_uids=23089) | paternally expressed 10 |
| [Details](http://mirdb.org/cgi-bin/target_detail.cgi?targetID=3073610) | 91 | 96 | hsa-miR-27a-3p | [SZRD1](http://www.ncbi.nlm.nih.gov/entrez/query.fcgi?db=gene&cmd=Retrieve&dopt=full_report&list_uids=26099) | SUZ RNA binding domain containing 1 |
| [Details](http://mirdb.org/cgi-bin/target_detail.cgi?targetID=3073663) | 92 | 96 | hsa-miR-27a-3p | [SLC35F1](http://www.ncbi.nlm.nih.gov/entrez/query.fcgi?db=gene&cmd=Retrieve&dopt=full_report&list_uids=222553) | solute carrier family 35 member F1 |
| [Details](http://mirdb.org/cgi-bin/target_detail.cgi?targetID=3073713) | 93 | 96 | hsa-miR-27a-3p | [ZHX1](http://www.ncbi.nlm.nih.gov/entrez/query.fcgi?db=gene&cmd=Retrieve&dopt=full_report&list_uids=11244) | zinc fingers and homeoboxes 1 |
| [Details](http://mirdb.org/cgi-bin/target_detail.cgi?targetID=3073770) | 94 | 96 | hsa-miR-27a-3p | [GNS](http://www.ncbi.nlm.nih.gov/entrez/query.fcgi?db=gene&cmd=Retrieve&dopt=full_report&list_uids=2799) | glucosamine (N-acetyl)-6-sulfatase |
| [Details](http://mirdb.org/cgi-bin/target_detail.cgi?targetID=3072484) | 95 | 95 | hsa-miR-27a-3p | [MARK1](http://www.ncbi.nlm.nih.gov/entrez/query.fcgi?db=gene&cmd=Retrieve&dopt=full_report&list_uids=4139) | microtubule affinity regulating kinase 1 |
| [Details](http://mirdb.org/cgi-bin/target_detail.cgi?targetID=3072536) | 96 | 95 | hsa-miR-27a-3p | [ZNF800](http://www.ncbi.nlm.nih.gov/entrez/query.fcgi?db=gene&cmd=Retrieve&dopt=full_report&list_uids=168850) | zinc finger protein 800 |
| [Details](http://mirdb.org/cgi-bin/target_detail.cgi?targetID=3072552) | 97 | 95 | hsa-miR-27a-3p | [RREB1](http://www.ncbi.nlm.nih.gov/entrez/query.fcgi?db=gene&cmd=Retrieve&dopt=full_report&list_uids=6239) | ras responsive element binding protein 1 |
| [Details](http://mirdb.org/cgi-bin/target_detail.cgi?targetID=3072570) | 98 | 95 | hsa-miR-27a-3p | [PDE7B](http://www.ncbi.nlm.nih.gov/entrez/query.fcgi?db=gene&cmd=Retrieve&dopt=full_report&list_uids=27115) | phosphodiesterase 7B |
| [Details](http://mirdb.org/cgi-bin/target_detail.cgi?targetID=3072586) | 99 | 95 | hsa-miR-27a-3p | [C20orf194](http://www.ncbi.nlm.nih.gov/entrez/query.fcgi?db=gene&cmd=Retrieve&dopt=full_report&list_uids=25943) | chromosome 20 open reading frame 194 |
| [Details](http://mirdb.org/cgi-bin/target_detail.cgi?targetID=3072605) | 100 | 95 | hsa-miR-27a-3p | [CREBRF](http://www.ncbi.nlm.nih.gov/entrez/query.fcgi?db=gene&cmd=Retrieve&dopt=full_report&list_uids=153222) | CREB3 regulatory factor |
| [Details](http://mirdb.org/cgi-bin/target_detail.cgi?targetID=3072729) | 101 | 95 | hsa-miR-27a-3p | [DNAJC13](http://www.ncbi.nlm.nih.gov/entrez/query.fcgi?db=gene&cmd=Retrieve&dopt=full_report&list_uids=23317) | DnaJ heat shock protein family (Hsp40) member C13 |
| [Details](http://mirdb.org/cgi-bin/target_detail.cgi?targetID=3072737) | 102 | 95 | hsa-miR-27a-3p | [TSC22D2](http://www.ncbi.nlm.nih.gov/entrez/query.fcgi?db=gene&cmd=Retrieve&dopt=full_report&list_uids=9819) | TSC22 domain family member 2 |
| [Details](http://mirdb.org/cgi-bin/target_detail.cgi?targetID=3072771) | 103 | 95 | hsa-miR-27a-3p | [GSPT1](http://www.ncbi.nlm.nih.gov/entrez/query.fcgi?db=gene&cmd=Retrieve&dopt=full_report&list_uids=2935) | G1 to S phase transition 1 |
| [Details](http://mirdb.org/cgi-bin/target_detail.cgi?targetID=3072789) | 104 | 95 | hsa-miR-27a-3p | [B4GALT3](http://www.ncbi.nlm.nih.gov/entrez/query.fcgi?db=gene&cmd=Retrieve&dopt=full_report&list_uids=8703) | beta-1,4-galactosyltransferase 3 |
| [Details](http://mirdb.org/cgi-bin/target_detail.cgi?targetID=3072843) | 105 | 95 | hsa-miR-27a-3p | [CCNG1](http://www.ncbi.nlm.nih.gov/entrez/query.fcgi?db=gene&cmd=Retrieve&dopt=full_report&list_uids=900) | cyclin G1 |
| [Details](http://mirdb.org/cgi-bin/target_detail.cgi?targetID=3072849) | 106 | 95 | hsa-miR-27a-3p | [NR2F6](http://www.ncbi.nlm.nih.gov/entrez/query.fcgi?db=gene&cmd=Retrieve&dopt=full_report&list_uids=2063) | nuclear receptor subfamily 2 group F member 6 |
| [Details](http://mirdb.org/cgi-bin/target_detail.cgi?targetID=3072924) | 107 | 95 | hsa-miR-27a-3p | [ITSN2](http://www.ncbi.nlm.nih.gov/entrez/query.fcgi?db=gene&cmd=Retrieve&dopt=full_report&list_uids=50618) | intersectin 2 |
| [Details](http://mirdb.org/cgi-bin/target_detail.cgi?targetID=3073048) | 108 | 95 | hsa-miR-27a-3p | [CSRP2](http://www.ncbi.nlm.nih.gov/entrez/query.fcgi?db=gene&cmd=Retrieve&dopt=full_report&list_uids=1466) | cysteine and glycine rich protein 2 |
| [Details](http://mirdb.org/cgi-bin/target_detail.cgi?targetID=3073051) | 109 | 95 | hsa-miR-27a-3p | [NECAP1](http://www.ncbi.nlm.nih.gov/entrez/query.fcgi?db=gene&cmd=Retrieve&dopt=full_report&list_uids=25977) | NECAP endocytosis associated 1 |
| [Details](http://mirdb.org/cgi-bin/target_detail.cgi?targetID=3073112) | 110 | 95 | hsa-miR-27a-3p | [ID4](http://www.ncbi.nlm.nih.gov/entrez/query.fcgi?db=gene&cmd=Retrieve&dopt=full_report&list_uids=3400) | inhibitor of DNA binding 4, HLH protein |
| [Details](http://mirdb.org/cgi-bin/target_detail.cgi?targetID=3073150) | 111 | 95 | hsa-miR-27a-3p | [RO60](http://www.ncbi.nlm.nih.gov/entrez/query.fcgi?db=gene&cmd=Retrieve&dopt=full_report&list_uids=6738) | Ro60, Y RNA binding protein |
| [Details](http://mirdb.org/cgi-bin/target_detail.cgi?targetID=3073155) | 112 | 95 | hsa-miR-27a-3p | [RALGAPA2](http://www.ncbi.nlm.nih.gov/entrez/query.fcgi?db=gene&cmd=Retrieve&dopt=full_report&list_uids=57186) | Ral GTPase activating protein catalytic alpha subunit 2 |
| [Details](http://mirdb.org/cgi-bin/target_detail.cgi?targetID=3073208) | 113 | 95 | hsa-miR-27a-3p | [MAP1B](http://www.ncbi.nlm.nih.gov/entrez/query.fcgi?db=gene&cmd=Retrieve&dopt=full_report&list_uids=4131) | microtubule associated protein 1B |
| [Details](http://mirdb.org/cgi-bin/target_detail.cgi?targetID=3073221) | 114 | 95 | hsa-miR-27a-3p | [GRM5](http://www.ncbi.nlm.nih.gov/entrez/query.fcgi?db=gene&cmd=Retrieve&dopt=full_report&list_uids=2915) | glutamate metabotropic receptor 5 |
| [Details](http://mirdb.org/cgi-bin/target_detail.cgi?targetID=3073238) | 115 | 95 | hsa-miR-27a-3p | [ST3GAL6](http://www.ncbi.nlm.nih.gov/entrez/query.fcgi?db=gene&cmd=Retrieve&dopt=full_report&list_uids=10402) | ST3 beta-galactoside alpha-2,3-sialyltransferase 6 |
| [Details](http://mirdb.org/cgi-bin/target_detail.cgi?targetID=3073245) | 116 | 95 | hsa-miR-27a-3p | [ASPH](http://www.ncbi.nlm.nih.gov/entrez/query.fcgi?db=gene&cmd=Retrieve&dopt=full_report&list_uids=444) | aspartate beta-hydroxylase |
| [Details](http://mirdb.org/cgi-bin/target_detail.cgi?targetID=3073281) | 117 | 95 | hsa-miR-27a-3p | [BRPF3](http://www.ncbi.nlm.nih.gov/entrez/query.fcgi?db=gene&cmd=Retrieve&dopt=full_report&list_uids=27154) | bromodomain and PHD finger containing 3 |
| [Details](http://mirdb.org/cgi-bin/target_detail.cgi?targetID=3073299) | 118 | 95 | hsa-miR-27a-3p | [SSH1](http://www.ncbi.nlm.nih.gov/entrez/query.fcgi?db=gene&cmd=Retrieve&dopt=full_report&list_uids=54434) | slingshot protein phosphatase 1 |
| [Details](http://mirdb.org/cgi-bin/target_detail.cgi?targetID=3073302) | 119 | 95 | hsa-miR-27a-3p | [STYK1](http://www.ncbi.nlm.nih.gov/entrez/query.fcgi?db=gene&cmd=Retrieve&dopt=full_report&list_uids=55359) | serine/threonine/tyrosine kinase 1 |
| [Details](http://mirdb.org/cgi-bin/target_detail.cgi?targetID=3073508) | 120 | 95 | hsa-miR-27a-3p | [KDM3A](http://www.ncbi.nlm.nih.gov/entrez/query.fcgi?db=gene&cmd=Retrieve&dopt=full_report&list_uids=55818) | lysine demethylase 3A |
| [Details](http://mirdb.org/cgi-bin/target_detail.cgi?targetID=3073544) | 121 | 95 | hsa-miR-27a-3p | [COG7](http://www.ncbi.nlm.nih.gov/entrez/query.fcgi?db=gene&cmd=Retrieve&dopt=full_report&list_uids=91949) | component of oligomeric golgi complex 7 |
| [Details](http://mirdb.org/cgi-bin/target_detail.cgi?targetID=3073549) | 122 | 95 | hsa-miR-27a-3p | [EDEM3](http://www.ncbi.nlm.nih.gov/entrez/query.fcgi?db=gene&cmd=Retrieve&dopt=full_report&list_uids=80267) | ER degradation enhancing alpha-mannosidase like protein 3 |
| [Details](http://mirdb.org/cgi-bin/target_detail.cgi?targetID=3073775) | 123 | 95 | hsa-miR-27a-3p | [SLC25A25](http://www.ncbi.nlm.nih.gov/entrez/query.fcgi?db=gene&cmd=Retrieve&dopt=full_report&list_uids=114789) | solute carrier family 25 member 25 |
| [Details](http://mirdb.org/cgi-bin/target_detail.cgi?targetID=3073889) | 124 | 95 | hsa-miR-27a-3p | [SNRNP27](http://www.ncbi.nlm.nih.gov/entrez/query.fcgi?db=gene&cmd=Retrieve&dopt=full_report&list_uids=11017) | small nuclear ribonucleoprotein U4/U6.U5 subunit 27 |
| [Details](http://mirdb.org/cgi-bin/target_detail.cgi?targetID=3072538) | 125 | 94 | hsa-miR-27a-3p | [DOT1L](http://www.ncbi.nlm.nih.gov/entrez/query.fcgi?db=gene&cmd=Retrieve&dopt=full_report&list_uids=84444) | DOT1 like histone lysine methyltransferase |
| [Details](http://mirdb.org/cgi-bin/target_detail.cgi?targetID=3072549) | 126 | 94 | hsa-miR-27a-3p | [KIAA1211L](http://www.ncbi.nlm.nih.gov/entrez/query.fcgi?db=gene&cmd=Retrieve&dopt=full_report&list_uids=343990) | KIAA1211 like |
| [Details](http://mirdb.org/cgi-bin/target_detail.cgi?targetID=3072672) | 127 | 94 | hsa-miR-27a-3p | [BICC1](http://www.ncbi.nlm.nih.gov/entrez/query.fcgi?db=gene&cmd=Retrieve&dopt=full_report&list_uids=80114) | BicC family RNA binding protein 1 |
| [Details](http://mirdb.org/cgi-bin/target_detail.cgi?targetID=3072681) | 128 | 94 | hsa-miR-27a-3p | [INSM2](http://www.ncbi.nlm.nih.gov/entrez/query.fcgi?db=gene&cmd=Retrieve&dopt=full_report&list_uids=84684) | INSM transcriptional repressor 2 |
| [Details](http://mirdb.org/cgi-bin/target_detail.cgi?targetID=3072711) | 129 | 94 | hsa-miR-27a-3p | [ZCCHC24](http://www.ncbi.nlm.nih.gov/entrez/query.fcgi?db=gene&cmd=Retrieve&dopt=full_report&list_uids=219654) | zinc finger CCHC-type containing 24 |
| [Details](http://mirdb.org/cgi-bin/target_detail.cgi?targetID=3072754) | 130 | 94 | hsa-miR-27a-3p | [GALNT7](http://www.ncbi.nlm.nih.gov/entrez/query.fcgi?db=gene&cmd=Retrieve&dopt=full_report&list_uids=51809) | polypeptide N-acetylgalactosaminyltransferase 7 |
| [Details](http://mirdb.org/cgi-bin/target_detail.cgi?targetID=3072764) | 131 | 94 | hsa-miR-27a-3p | [B3GNT7](http://www.ncbi.nlm.nih.gov/entrez/query.fcgi?db=gene&cmd=Retrieve&dopt=full_report&list_uids=93010) | UDP-GlcNAc:betaGal beta-1,3-N-acetylglucosaminyltransferase 7 |
| [Details](http://mirdb.org/cgi-bin/target_detail.cgi?targetID=3072880) | 132 | 94 | hsa-miR-27a-3p | [APPBP2](http://www.ncbi.nlm.nih.gov/entrez/query.fcgi?db=gene&cmd=Retrieve&dopt=full_report&list_uids=10513) | amyloid beta precursor protein binding protein 2 |
| [Details](http://mirdb.org/cgi-bin/target_detail.cgi?targetID=3072905) | 133 | 94 | hsa-miR-27a-3p | [PPARG](http://www.ncbi.nlm.nih.gov/entrez/query.fcgi?db=gene&cmd=Retrieve&dopt=full_report&list_uids=5468) | peroxisome proliferator activated receptor gamma |
| [Details](http://mirdb.org/cgi-bin/target_detail.cgi?targetID=3072906) | 134 | 94 | hsa-miR-27a-3p | [UBE2V1](http://www.ncbi.nlm.nih.gov/entrez/query.fcgi?db=gene&cmd=Retrieve&dopt=full_report&list_uids=7335) | ubiquitin conjugating enzyme E2 V1 |
| [Details](http://mirdb.org/cgi-bin/target_detail.cgi?targetID=3072912) | 135 | 94 | hsa-miR-27a-3p | [RMND5A](http://www.ncbi.nlm.nih.gov/entrez/query.fcgi?db=gene&cmd=Retrieve&dopt=full_report&list_uids=64795) | required for meiotic nuclear division 5 homolog A |
| [Details](http://mirdb.org/cgi-bin/target_detail.cgi?targetID=3072927) | 136 | 94 | hsa-miR-27a-3p | [SHE](http://www.ncbi.nlm.nih.gov/entrez/query.fcgi?db=gene&cmd=Retrieve&dopt=full_report&list_uids=126669) | Src homology 2 domain containing E |
| [Details](http://mirdb.org/cgi-bin/target_detail.cgi?targetID=3072944) | 137 | 94 | hsa-miR-27a-3p | [NIPAL4](http://www.ncbi.nlm.nih.gov/entrez/query.fcgi?db=gene&cmd=Retrieve&dopt=full_report&list_uids=348938) | NIPA like domain containing 4 |
| [Details](http://mirdb.org/cgi-bin/target_detail.cgi?targetID=3072972) | 138 | 94 | hsa-miR-27a-3p | [ARF3](http://www.ncbi.nlm.nih.gov/entrez/query.fcgi?db=gene&cmd=Retrieve&dopt=full_report&list_uids=377) | ADP ribosylation factor 3 |
| [Details](http://mirdb.org/cgi-bin/target_detail.cgi?targetID=3073094) | 139 | 94 | hsa-miR-27a-3p | [CTH](http://www.ncbi.nlm.nih.gov/entrez/query.fcgi?db=gene&cmd=Retrieve&dopt=full_report&list_uids=1491) | cystathionine gamma-lyase |
| [Details](http://mirdb.org/cgi-bin/target_detail.cgi?targetID=3073172) | 140 | 94 | hsa-miR-27a-3p | [KCNA4](http://www.ncbi.nlm.nih.gov/entrez/query.fcgi?db=gene&cmd=Retrieve&dopt=full_report&list_uids=3739) | potassium voltage-gated channel subfamily A member 4 |
| [Details](http://mirdb.org/cgi-bin/target_detail.cgi?targetID=3073256) | 141 | 94 | hsa-miR-27a-3p | [ENDOU](http://www.ncbi.nlm.nih.gov/entrez/query.fcgi?db=gene&cmd=Retrieve&dopt=full_report&list_uids=8909) | endonuclease, poly(U) specific |
| [Details](http://mirdb.org/cgi-bin/target_detail.cgi?targetID=3073296) | 142 | 94 | hsa-miR-27a-3p | [PCNX1](http://www.ncbi.nlm.nih.gov/entrez/query.fcgi?db=gene&cmd=Retrieve&dopt=full_report&list_uids=22990) | pecanex 1 |
| [Details](http://mirdb.org/cgi-bin/target_detail.cgi?targetID=3073310) | 143 | 94 | hsa-miR-27a-3p | [TNRC18](http://www.ncbi.nlm.nih.gov/entrez/query.fcgi?db=gene&cmd=Retrieve&dopt=full_report&list_uids=84629) | trinucleotide repeat containing 18 |
| [Details](http://mirdb.org/cgi-bin/target_detail.cgi?targetID=3073415) | 144 | 94 | hsa-miR-27a-3p | [INO80D](http://www.ncbi.nlm.nih.gov/entrez/query.fcgi?db=gene&cmd=Retrieve&dopt=full_report&list_uids=54891) | INO80 complex subunit D |
| [Details](http://mirdb.org/cgi-bin/target_detail.cgi?targetID=3073449) | 145 | 94 | hsa-miR-27a-3p | [PTGER3](http://www.ncbi.nlm.nih.gov/entrez/query.fcgi?db=gene&cmd=Retrieve&dopt=full_report&list_uids=5733) | prostaglandin E receptor 3 |
| [Details](http://mirdb.org/cgi-bin/target_detail.cgi?targetID=3073494) | 146 | 94 | hsa-miR-27a-3p | [KITLG](http://www.ncbi.nlm.nih.gov/entrez/query.fcgi?db=gene&cmd=Retrieve&dopt=full_report&list_uids=4254) | KIT ligand |
| [Details](http://mirdb.org/cgi-bin/target_detail.cgi?targetID=3073640) | 147 | 94 | hsa-miR-27a-3p | [USP25](http://www.ncbi.nlm.nih.gov/entrez/query.fcgi?db=gene&cmd=Retrieve&dopt=full_report&list_uids=29761) | ubiquitin specific peptidase 25 |
| [Details](http://mirdb.org/cgi-bin/target_detail.cgi?targetID=3073667) | 148 | 94 | hsa-miR-27a-3p | [ZSCAN26](http://www.ncbi.nlm.nih.gov/entrez/query.fcgi?db=gene&cmd=Retrieve&dopt=full_report&list_uids=7741) | zinc finger and SCAN domain containing 26 |
| [Details](http://mirdb.org/cgi-bin/target_detail.cgi?targetID=3073750) | 149 | 94 | hsa-miR-27a-3p | [NPAS3](http://www.ncbi.nlm.nih.gov/entrez/query.fcgi?db=gene&cmd=Retrieve&dopt=full_report&list_uids=64067) | neuronal PAS domain protein 3 |
| [Details](http://mirdb.org/cgi-bin/target_detail.cgi?targetID=3073769) | 150 | 94 | hsa-miR-27a-3p | [SLC39A11](http://www.ncbi.nlm.nih.gov/entrez/query.fcgi?db=gene&cmd=Retrieve&dopt=full_report&list_uids=201266) | solute carrier family 39 member 11 |
| [Details](http://mirdb.org/cgi-bin/target_detail.cgi?targetID=3073870) | 151 | 94 | hsa-miR-27a-3p | [GRIA3](http://www.ncbi.nlm.nih.gov/entrez/query.fcgi?db=gene&cmd=Retrieve&dopt=full_report&list_uids=2892) | glutamate ionotropic receptor AMPA type subunit 3 |
| [Details](http://mirdb.org/cgi-bin/target_detail.cgi?targetID=3073872) | 152 | 94 | hsa-miR-27a-3p | [PCDH9](http://www.ncbi.nlm.nih.gov/entrez/query.fcgi?db=gene&cmd=Retrieve&dopt=full_report&list_uids=5101) | protocadherin 9 |
| [Details](http://mirdb.org/cgi-bin/target_detail.cgi?targetID=3073887) | 153 | 94 | hsa-miR-27a-3p | [AGFG1](http://www.ncbi.nlm.nih.gov/entrez/query.fcgi?db=gene&cmd=Retrieve&dopt=full_report&list_uids=3267) | ArfGAP with FG repeats 1 |
| [Details](http://mirdb.org/cgi-bin/target_detail.cgi?targetID=3073909) | 154 | 94 | hsa-miR-27a-3p | [VSIG10](http://www.ncbi.nlm.nih.gov/entrez/query.fcgi?db=gene&cmd=Retrieve&dopt=full_report&list_uids=54621) | V-set and immunoglobulin domain containing 10 |
| [Details](http://mirdb.org/cgi-bin/target_detail.cgi?targetID=3073914) | 155 | 94 | hsa-miR-27a-3p | [MIER3](http://www.ncbi.nlm.nih.gov/entrez/query.fcgi?db=gene&cmd=Retrieve&dopt=full_report&list_uids=166968) | MIER family member 3 |
| [Details](http://mirdb.org/cgi-bin/target_detail.cgi?targetID=3072492) | 156 | 93 | hsa-miR-27a-3p | [C1orf52](http://www.ncbi.nlm.nih.gov/entrez/query.fcgi?db=gene&cmd=Retrieve&dopt=full_report&list_uids=148423) | chromosome 1 open reading frame 52 |
| [Details](http://mirdb.org/cgi-bin/target_detail.cgi?targetID=3072594) | 157 | 93 | hsa-miR-27a-3p | [ADAMTS10](http://www.ncbi.nlm.nih.gov/entrez/query.fcgi?db=gene&cmd=Retrieve&dopt=full_report&list_uids=81794) | ADAM metallopeptidase with thrombospondin type 1 motif 10 |
| [Details](http://mirdb.org/cgi-bin/target_detail.cgi?targetID=3072652) | 158 | 93 | hsa-miR-27a-3p | [SLC6A1](http://www.ncbi.nlm.nih.gov/entrez/query.fcgi?db=gene&cmd=Retrieve&dopt=full_report&list_uids=6529) | solute carrier family 6 member 1 |
| [Details](http://mirdb.org/cgi-bin/target_detail.cgi?targetID=3072658) | 159 | 93 | hsa-miR-27a-3p | [HMGCR](http://www.ncbi.nlm.nih.gov/entrez/query.fcgi?db=gene&cmd=Retrieve&dopt=full_report&list_uids=3156) | 3-hydroxy-3-methylglutaryl-CoA reductase |
| [Details](http://mirdb.org/cgi-bin/target_detail.cgi?targetID=3072677) | 160 | 93 | hsa-miR-27a-3p | [PALM2](http://www.ncbi.nlm.nih.gov/entrez/query.fcgi?db=gene&cmd=Retrieve&dopt=full_report&list_uids=114299) | paralemmin 2 |
| [Details](http://mirdb.org/cgi-bin/target_detail.cgi?targetID=3072691) | 161 | 93 | hsa-miR-27a-3p | [GAREM1](http://www.ncbi.nlm.nih.gov/entrez/query.fcgi?db=gene&cmd=Retrieve&dopt=full_report&list_uids=64762) | GRB2 associated regulator of MAPK1 subtype 1 |
| [Details](http://mirdb.org/cgi-bin/target_detail.cgi?targetID=3072773) | 162 | 93 | hsa-miR-27a-3p | [HBEGF](http://www.ncbi.nlm.nih.gov/entrez/query.fcgi?db=gene&cmd=Retrieve&dopt=full_report&list_uids=1839) | heparin binding EGF like growth factor |
| [Details](http://mirdb.org/cgi-bin/target_detail.cgi?targetID=3072879) | 163 | 93 | hsa-miR-27a-3p | [REPS1](http://www.ncbi.nlm.nih.gov/entrez/query.fcgi?db=gene&cmd=Retrieve&dopt=full_report&list_uids=85021) | RALBP1 associated Eps domain containing 1 |
| [Details](http://mirdb.org/cgi-bin/target_detail.cgi?targetID=3073077) | 164 | 93 | hsa-miR-27a-3p | [EPB41L4A](http://www.ncbi.nlm.nih.gov/entrez/query.fcgi?db=gene&cmd=Retrieve&dopt=full_report&list_uids=64097) | erythrocyte membrane protein band 4.1 like 4A |
| [Details](http://mirdb.org/cgi-bin/target_detail.cgi?targetID=3073156) | 165 | 93 | hsa-miR-27a-3p | [SFRP1](http://www.ncbi.nlm.nih.gov/entrez/query.fcgi?db=gene&cmd=Retrieve&dopt=full_report&list_uids=6422) | secreted frizzled related protein 1 |
| [Details](http://mirdb.org/cgi-bin/target_detail.cgi?targetID=3073157) | 166 | 93 | hsa-miR-27a-3p | [CREB1](http://www.ncbi.nlm.nih.gov/entrez/query.fcgi?db=gene&cmd=Retrieve&dopt=full_report&list_uids=1385) | cAMP responsive element binding protein 1 |
| [Details](http://mirdb.org/cgi-bin/target_detail.cgi?targetID=3073209) | 167 | 93 | hsa-miR-27a-3p | [TPR](http://www.ncbi.nlm.nih.gov/entrez/query.fcgi?db=gene&cmd=Retrieve&dopt=full_report&list_uids=7175) | translocated promoter region, nuclear basket protein |
| [Details](http://mirdb.org/cgi-bin/target_detail.cgi?targetID=3073262) | 168 | 93 | hsa-miR-27a-3p | [ELFN2](http://www.ncbi.nlm.nih.gov/entrez/query.fcgi?db=gene&cmd=Retrieve&dopt=full_report&list_uids=114794) | extracellular leucine rich repeat and fibronectin type III domain containing 2 |
| [Details](http://mirdb.org/cgi-bin/target_detail.cgi?targetID=3073268) | 169 | 93 | hsa-miR-27a-3p | [FAM184A](http://www.ncbi.nlm.nih.gov/entrez/query.fcgi?db=gene&cmd=Retrieve&dopt=full_report&list_uids=79632) | family with sequence similarity 184 member A |
| [Details](http://mirdb.org/cgi-bin/target_detail.cgi?targetID=3073364) | 170 | 93 | hsa-miR-27a-3p | [ATAD2B](http://www.ncbi.nlm.nih.gov/entrez/query.fcgi?db=gene&cmd=Retrieve&dopt=full_report&list_uids=54454) | ATPase family, AAA domain containing 2B |
| [Details](http://mirdb.org/cgi-bin/target_detail.cgi?targetID=3073375) | 171 | 93 | hsa-miR-27a-3p | [STAB2](http://www.ncbi.nlm.nih.gov/entrez/query.fcgi?db=gene&cmd=Retrieve&dopt=full_report&list_uids=55576) | stabilin 2 |
| [Details](http://mirdb.org/cgi-bin/target_detail.cgi?targetID=3073382) | 172 | 93 | hsa-miR-27a-3p | [MTURN](http://www.ncbi.nlm.nih.gov/entrez/query.fcgi?db=gene&cmd=Retrieve&dopt=full_report&list_uids=222166) | maturin, neural progenitor differentiation regulator homolog |
| [Details](http://mirdb.org/cgi-bin/target_detail.cgi?targetID=3073495) | 173 | 93 | hsa-miR-27a-3p | [PAQR9](http://www.ncbi.nlm.nih.gov/entrez/query.fcgi?db=gene&cmd=Retrieve&dopt=full_report&list_uids=344838) | progestin and adipoQ receptor family member 9 |
| [Details](http://mirdb.org/cgi-bin/target_detail.cgi?targetID=3073504) | 174 | 93 | hsa-miR-27a-3p | [DIPK1A](http://www.ncbi.nlm.nih.gov/entrez/query.fcgi?db=gene&cmd=Retrieve&dopt=full_report&list_uids=388650) | divergent protein kinase domain 1A |
| [Details](http://mirdb.org/cgi-bin/target_detail.cgi?targetID=3073547) | 175 | 93 | hsa-miR-27a-3p | [MKNK2](http://www.ncbi.nlm.nih.gov/entrez/query.fcgi?db=gene&cmd=Retrieve&dopt=full_report&list_uids=2872) | MAP kinase interacting serine/threonine kinase 2 |
| [Details](http://mirdb.org/cgi-bin/target_detail.cgi?targetID=3073548) | 176 | 93 | hsa-miR-27a-3p | [FBXO34](http://www.ncbi.nlm.nih.gov/entrez/query.fcgi?db=gene&cmd=Retrieve&dopt=full_report&list_uids=55030) | F-box protein 34 |
| [Details](http://mirdb.org/cgi-bin/target_detail.cgi?targetID=3073560) | 177 | 93 | hsa-miR-27a-3p | [PARD6B](http://www.ncbi.nlm.nih.gov/entrez/query.fcgi?db=gene&cmd=Retrieve&dopt=full_report&list_uids=84612) | par-6 family cell polarity regulator beta |
| [Details](http://mirdb.org/cgi-bin/target_detail.cgi?targetID=3073576) | 178 | 93 | hsa-miR-27a-3p | [ARHGAP32](http://www.ncbi.nlm.nih.gov/entrez/query.fcgi?db=gene&cmd=Retrieve&dopt=full_report&list_uids=9743) | Rho GTPase activating protein 32 |
| [Details](http://mirdb.org/cgi-bin/target_detail.cgi?targetID=3073597) | 179 | 93 | hsa-miR-27a-3p | [STAG1](http://www.ncbi.nlm.nih.gov/entrez/query.fcgi?db=gene&cmd=Retrieve&dopt=full_report&list_uids=10274) | stromal antigen 1 |
| [Details](http://mirdb.org/cgi-bin/target_detail.cgi?targetID=3073607) | 180 | 93 | hsa-miR-27a-3p | [NABP1](http://www.ncbi.nlm.nih.gov/entrez/query.fcgi?db=gene&cmd=Retrieve&dopt=full_report&list_uids=64859) | nucleic acid binding protein 1 |
| [Details](http://mirdb.org/cgi-bin/target_detail.cgi?targetID=3073614) | 181 | 93 | hsa-miR-27a-3p | [RPS6KB1](http://www.ncbi.nlm.nih.gov/entrez/query.fcgi?db=gene&cmd=Retrieve&dopt=full_report&list_uids=6198) | ribosomal protein S6 kinase B1 |
| [Details](http://mirdb.org/cgi-bin/target_detail.cgi?targetID=3073623) | 182 | 93 | hsa-miR-27a-3p | [TRIM23](http://www.ncbi.nlm.nih.gov/entrez/query.fcgi?db=gene&cmd=Retrieve&dopt=full_report&list_uids=373) | tripartite motif containing 23 |
| [Details](http://mirdb.org/cgi-bin/target_detail.cgi?targetID=3073744) | 183 | 93 | hsa-miR-27a-3p | [RCAN2](http://www.ncbi.nlm.nih.gov/entrez/query.fcgi?db=gene&cmd=Retrieve&dopt=full_report&list_uids=10231) | regulator of calcineurin 2 |
| [Details](http://mirdb.org/cgi-bin/target_detail.cgi?targetID=3073746) | 184 | 93 | hsa-miR-27a-3p | [ACTA2](http://www.ncbi.nlm.nih.gov/entrez/query.fcgi?db=gene&cmd=Retrieve&dopt=full_report&list_uids=59) | actin, alpha 2, smooth muscle, aorta |
| [Details](http://mirdb.org/cgi-bin/target_detail.cgi?targetID=3073798) | 185 | 93 | hsa-miR-27a-3p | [HOXA10](http://www.ncbi.nlm.nih.gov/entrez/query.fcgi?db=gene&cmd=Retrieve&dopt=full_report&list_uids=3206) | homeobox A10 |
| [Details](http://mirdb.org/cgi-bin/target_detail.cgi?targetID=3073867) | 186 | 93 | hsa-miR-27a-3p | [MATN3](http://www.ncbi.nlm.nih.gov/entrez/query.fcgi?db=gene&cmd=Retrieve&dopt=full_report&list_uids=4148) | matrilin 3 |
| [Details](http://mirdb.org/cgi-bin/target_detail.cgi?targetID=3073918) | 187 | 93 | hsa-miR-27a-3p | [TEAD1](http://www.ncbi.nlm.nih.gov/entrez/query.fcgi?db=gene&cmd=Retrieve&dopt=full_report&list_uids=7003) | TEA domain transcription factor 1 |
| [Details](http://mirdb.org/cgi-bin/target_detail.cgi?targetID=3072446) | 188 | 92 | hsa-miR-27a-3p | [RAPH1](http://www.ncbi.nlm.nih.gov/entrez/query.fcgi?db=gene&cmd=Retrieve&dopt=full_report&list_uids=65059) | Ras association (RalGDS/AF-6) and pleckstrin homology domains 1 |
| [Details](http://mirdb.org/cgi-bin/target_detail.cgi?targetID=3072490) | 189 | 92 | hsa-miR-27a-3p | [NPEPPS](http://www.ncbi.nlm.nih.gov/entrez/query.fcgi?db=gene&cmd=Retrieve&dopt=full_report&list_uids=9520) | aminopeptidase puromycin sensitive |
| [Details](http://mirdb.org/cgi-bin/target_detail.cgi?targetID=3072501) | 190 | 92 | hsa-miR-27a-3p | [LPAR6](http://www.ncbi.nlm.nih.gov/entrez/query.fcgi?db=gene&cmd=Retrieve&dopt=full_report&list_uids=10161) | lysophosphatidic acid receptor 6 |
| [Details](http://mirdb.org/cgi-bin/target_detail.cgi?targetID=3072510) | 191 | 92 | hsa-miR-27a-3p | [CLCN3](http://www.ncbi.nlm.nih.gov/entrez/query.fcgi?db=gene&cmd=Retrieve&dopt=full_report&list_uids=1182) | chloride voltage-gated channel 3 |
| [Details](http://mirdb.org/cgi-bin/target_detail.cgi?targetID=3072568) | 192 | 92 | hsa-miR-27a-3p | [PANK1](http://www.ncbi.nlm.nih.gov/entrez/query.fcgi?db=gene&cmd=Retrieve&dopt=full_report&list_uids=53354) | pantothenate kinase 1 |
| [Details](http://mirdb.org/cgi-bin/target_detail.cgi?targetID=3072601) | 193 | 92 | hsa-miR-27a-3p | [RNF141](http://www.ncbi.nlm.nih.gov/entrez/query.fcgi?db=gene&cmd=Retrieve&dopt=full_report&list_uids=50862) | ring finger protein 141 |
| [Details](http://mirdb.org/cgi-bin/target_detail.cgi?targetID=3072619) | 194 | 92 | hsa-miR-27a-3p | [TLK2](http://www.ncbi.nlm.nih.gov/entrez/query.fcgi?db=gene&cmd=Retrieve&dopt=full_report&list_uids=11011) | tousled like kinase 2 |
| [Details](http://mirdb.org/cgi-bin/target_detail.cgi?targetID=3072732) | 195 | 92 | hsa-miR-27a-3p | [YWHAQ](http://www.ncbi.nlm.nih.gov/entrez/query.fcgi?db=gene&cmd=Retrieve&dopt=full_report&list_uids=10971) | tyrosine 3-monooxygenase/tryptophan 5-monooxygenase activation protein theta |
| [Details](http://mirdb.org/cgi-bin/target_detail.cgi?targetID=3072763) | 196 | 92 | hsa-miR-27a-3p | [LPCAT1](http://www.ncbi.nlm.nih.gov/entrez/query.fcgi?db=gene&cmd=Retrieve&dopt=full_report&list_uids=79888) | lysophosphatidylcholine acyltransferase 1 |
| [Details](http://mirdb.org/cgi-bin/target_detail.cgi?targetID=3072772) | 197 | 92 | hsa-miR-27a-3p | [MAP2K4](http://www.ncbi.nlm.nih.gov/entrez/query.fcgi?db=gene&cmd=Retrieve&dopt=full_report&list_uids=6416) | mitogen-activated protein kinase kinase 4 |
| [Details](http://mirdb.org/cgi-bin/target_detail.cgi?targetID=3072871) | 198 | 92 | hsa-miR-27a-3p | [ING5](http://www.ncbi.nlm.nih.gov/entrez/query.fcgi?db=gene&cmd=Retrieve&dopt=full_report&list_uids=84289) | inhibitor of growth family member 5 |
| [Details](http://mirdb.org/cgi-bin/target_detail.cgi?targetID=3072898) | 199 | 92 | hsa-miR-27a-3p | [AK4](http://www.ncbi.nlm.nih.gov/entrez/query.fcgi?db=gene&cmd=Retrieve&dopt=full_report&list_uids=205) | adenylate kinase 4 |
| [Details](http://mirdb.org/cgi-bin/target_detail.cgi?targetID=3072913) | 200 | 92 | hsa-miR-27a-3p | [NEMP2](http://www.ncbi.nlm.nih.gov/entrez/query.fcgi?db=gene&cmd=Retrieve&dopt=full_report&list_uids=100131211) | nuclear envelope integral membrane protein 2 |
| [Details](http://mirdb.org/cgi-bin/target_detail.cgi?targetID=3073054) | 201 | 92 | hsa-miR-27a-3p | [CACNG2](http://www.ncbi.nlm.nih.gov/entrez/query.fcgi?db=gene&cmd=Retrieve&dopt=full_report&list_uids=10369) | calcium voltage-gated channel auxiliary subunit gamma 2 |
| [Details](http://mirdb.org/cgi-bin/target_detail.cgi?targetID=3073106) | 202 | 92 | hsa-miR-27a-3p | [NR5A2](http://www.ncbi.nlm.nih.gov/entrez/query.fcgi?db=gene&cmd=Retrieve&dopt=full_report&list_uids=2494) | nuclear receptor subfamily 5 group A member 2 |
| [Details](http://mirdb.org/cgi-bin/target_detail.cgi?targetID=3073119) | 203 | 92 | hsa-miR-27a-3p | [CEP135](http://www.ncbi.nlm.nih.gov/entrez/query.fcgi?db=gene&cmd=Retrieve&dopt=full_report&list_uids=9662) | centrosomal protein 135 |
| [Details](http://mirdb.org/cgi-bin/target_detail.cgi?targetID=3073159) | 204 | 92 | hsa-miR-27a-3p | [DTNA](http://www.ncbi.nlm.nih.gov/entrez/query.fcgi?db=gene&cmd=Retrieve&dopt=full_report&list_uids=1837) | dystrobrevin alpha |
| [Details](http://mirdb.org/cgi-bin/target_detail.cgi?targetID=3073163) | 205 | 92 | hsa-miR-27a-3p | [BRWD3](http://www.ncbi.nlm.nih.gov/entrez/query.fcgi?db=gene&cmd=Retrieve&dopt=full_report&list_uids=254065) | bromodomain and WD repeat domain containing 3 |
| [Details](http://mirdb.org/cgi-bin/target_detail.cgi?targetID=3073234) | 206 | 92 | hsa-miR-27a-3p | [SNAP25](http://www.ncbi.nlm.nih.gov/entrez/query.fcgi?db=gene&cmd=Retrieve&dopt=full_report&list_uids=6616) | synaptosome associated protein 25 |
| [Details](http://mirdb.org/cgi-bin/target_detail.cgi?targetID=3073243) | 207 | 92 | hsa-miR-27a-3p | [MYT1](http://www.ncbi.nlm.nih.gov/entrez/query.fcgi?db=gene&cmd=Retrieve&dopt=full_report&list_uids=4661) | myelin transcription factor 1 |
| [Details](http://mirdb.org/cgi-bin/target_detail.cgi?targetID=3073300) | 208 | 92 | hsa-miR-27a-3p | [LPIN1](http://www.ncbi.nlm.nih.gov/entrez/query.fcgi?db=gene&cmd=Retrieve&dopt=full_report&list_uids=23175) | lipin 1 |
| [Details](http://mirdb.org/cgi-bin/target_detail.cgi?targetID=3073336) | 209 | 92 | hsa-miR-27a-3p | [E2F7](http://www.ncbi.nlm.nih.gov/entrez/query.fcgi?db=gene&cmd=Retrieve&dopt=full_report&list_uids=144455) | E2F transcription factor 7 |
| [Details](http://mirdb.org/cgi-bin/target_detail.cgi?targetID=3073340) | 210 | 92 | hsa-miR-27a-3p | [PPP4R2](http://www.ncbi.nlm.nih.gov/entrez/query.fcgi?db=gene&cmd=Retrieve&dopt=full_report&list_uids=151987) | protein phosphatase 4 regulatory subunit 2 |
| [Details](http://mirdb.org/cgi-bin/target_detail.cgi?targetID=3073451) | 211 | 92 | hsa-miR-27a-3p | [PDE10A](http://www.ncbi.nlm.nih.gov/entrez/query.fcgi?db=gene&cmd=Retrieve&dopt=full_report&list_uids=10846) | phosphodiesterase 10A |
| [Details](http://mirdb.org/cgi-bin/target_detail.cgi?targetID=3073552) | 212 | 92 | hsa-miR-27a-3p | [PLPP3](http://www.ncbi.nlm.nih.gov/entrez/query.fcgi?db=gene&cmd=Retrieve&dopt=full_report&list_uids=8613) | phospholipid phosphatase 3 |
| [Details](http://mirdb.org/cgi-bin/target_detail.cgi?targetID=3073677) | 213 | 92 | hsa-miR-27a-3p | [HAPLN1](http://www.ncbi.nlm.nih.gov/entrez/query.fcgi?db=gene&cmd=Retrieve&dopt=full_report&list_uids=1404) | hyaluronan and proteoglycan link protein 1 |
| [Details](http://mirdb.org/cgi-bin/target_detail.cgi?targetID=3073710) | 214 | 92 | hsa-miR-27a-3p | [CLK2](http://www.ncbi.nlm.nih.gov/entrez/query.fcgi?db=gene&cmd=Retrieve&dopt=full_report&list_uids=1196) | CDC like kinase 2 |
| [Details](http://mirdb.org/cgi-bin/target_detail.cgi?targetID=3073791) | 215 | 92 | hsa-miR-27a-3p | [TNRC6B](http://www.ncbi.nlm.nih.gov/entrez/query.fcgi?db=gene&cmd=Retrieve&dopt=full_report&list_uids=23112) | trinucleotide repeat containing 6B |
| [Details](http://mirdb.org/cgi-bin/target_detail.cgi?targetID=3073815) | 216 | 92 | hsa-miR-27a-3p | [SOX11](http://www.ncbi.nlm.nih.gov/entrez/query.fcgi?db=gene&cmd=Retrieve&dopt=full_report&list_uids=6664) | SRY-box 11 |
| [Details](http://mirdb.org/cgi-bin/target_detail.cgi?targetID=3073838) | 217 | 92 | hsa-miR-27a-3p | [CA10](http://www.ncbi.nlm.nih.gov/entrez/query.fcgi?db=gene&cmd=Retrieve&dopt=full_report&list_uids=56934) | carbonic anhydrase 10 |
| [Details](http://mirdb.org/cgi-bin/target_detail.cgi?targetID=3073860) | 218 | 92 | hsa-miR-27a-3p | [SCAF11](http://www.ncbi.nlm.nih.gov/entrez/query.fcgi?db=gene&cmd=Retrieve&dopt=full_report&list_uids=9169) | SR-related CTD associated factor 11 |
| [Details](http://mirdb.org/cgi-bin/target_detail.cgi?targetID=3073880) | 219 | 92 | hsa-miR-27a-3p | [SRGAP2](http://www.ncbi.nlm.nih.gov/entrez/query.fcgi?db=gene&cmd=Retrieve&dopt=full_report&list_uids=23380) | SLIT-ROBO Rho GTPase activating protein 2 |
| [Details](http://mirdb.org/cgi-bin/target_detail.cgi?targetID=3073922) | 220 | 92 | hsa-miR-27a-3p | [MARC1](http://www.ncbi.nlm.nih.gov/entrez/query.fcgi?db=gene&cmd=Retrieve&dopt=full_report&list_uids=64757) | mitochondrial amidoxime reducing component 1 |
| [Details](http://mirdb.org/cgi-bin/target_detail.cgi?targetID=3072472) | 221 | 91 | hsa-miR-27a-3p | [MED12L](http://www.ncbi.nlm.nih.gov/entrez/query.fcgi?db=gene&cmd=Retrieve&dopt=full_report&list_uids=116931) | mediator complex subunit 12 like |
| [Details](http://mirdb.org/cgi-bin/target_detail.cgi?targetID=3072489) | 222 | 91 | hsa-miR-27a-3p | [LONRF1](http://www.ncbi.nlm.nih.gov/entrez/query.fcgi?db=gene&cmd=Retrieve&dopt=full_report&list_uids=91694) | LON peptidase N-terminal domain and ring finger 1 |
| [Details](http://mirdb.org/cgi-bin/target_detail.cgi?targetID=3072558) | 223 | 91 | hsa-miR-27a-3p | [DYNC2LI1](http://www.ncbi.nlm.nih.gov/entrez/query.fcgi?db=gene&cmd=Retrieve&dopt=full_report&list_uids=51626) | dynein cytoplasmic 2 light intermediate chain 1 |
| [Details](http://mirdb.org/cgi-bin/target_detail.cgi?targetID=3072563) | 224 | 91 | hsa-miR-27a-3p | [MTMR4](http://www.ncbi.nlm.nih.gov/entrez/query.fcgi?db=gene&cmd=Retrieve&dopt=full_report&list_uids=9110) | myotubularin related protein 4 |
| [Details](http://mirdb.org/cgi-bin/target_detail.cgi?targetID=3072592) | 225 | 91 | hsa-miR-27a-3p | [KMT2C](http://www.ncbi.nlm.nih.gov/entrez/query.fcgi?db=gene&cmd=Retrieve&dopt=full_report&list_uids=58508) | lysine methyltransferase 2C |
| [Details](http://mirdb.org/cgi-bin/target_detail.cgi?targetID=3072639) | 226 | 91 | hsa-miR-27a-3p | [GOLGA1](http://www.ncbi.nlm.nih.gov/entrez/query.fcgi?db=gene&cmd=Retrieve&dopt=full_report&list_uids=2800) | golgin A1 |
| [Details](http://mirdb.org/cgi-bin/target_detail.cgi?targetID=3072640) | 227 | 91 | hsa-miR-27a-3p | [CIPC](http://www.ncbi.nlm.nih.gov/entrez/query.fcgi?db=gene&cmd=Retrieve&dopt=full_report&list_uids=85457) | CLOCK interacting pacemaker |
| [Details](http://mirdb.org/cgi-bin/target_detail.cgi?targetID=3072766) | 228 | 91 | hsa-miR-27a-3p | [FAM133B](http://www.ncbi.nlm.nih.gov/entrez/query.fcgi?db=gene&cmd=Retrieve&dopt=full_report&list_uids=257415) | family with sequence similarity 133 member B |
| [Details](http://mirdb.org/cgi-bin/target_detail.cgi?targetID=3072803) | 229 | 91 | hsa-miR-27a-3p | [IKZF2](http://www.ncbi.nlm.nih.gov/entrez/query.fcgi?db=gene&cmd=Retrieve&dopt=full_report&list_uids=22807) | IKAROS family zinc finger 2 |
| [Details](http://mirdb.org/cgi-bin/target_detail.cgi?targetID=3072821) | 230 | 91 | hsa-miR-27a-3p | [NGFR](http://www.ncbi.nlm.nih.gov/entrez/query.fcgi?db=gene&cmd=Retrieve&dopt=full_report&list_uids=4804) | nerve growth factor receptor |
| [Details](http://mirdb.org/cgi-bin/target_detail.cgi?targetID=3072834) | 231 | 91 | hsa-miR-27a-3p | [RGS8](http://www.ncbi.nlm.nih.gov/entrez/query.fcgi?db=gene&cmd=Retrieve&dopt=full_report&list_uids=85397) | regulator of G protein signaling 8 |
| [Details](http://mirdb.org/cgi-bin/target_detail.cgi?targetID=3072882) | 232 | 91 | hsa-miR-27a-3p | [SNX10](http://www.ncbi.nlm.nih.gov/entrez/query.fcgi?db=gene&cmd=Retrieve&dopt=full_report&list_uids=29887) | sorting nexin 10 |
| [Details](http://mirdb.org/cgi-bin/target_detail.cgi?targetID=3072945) | 233 | 91 | hsa-miR-27a-3p | [PIK3CA](http://www.ncbi.nlm.nih.gov/entrez/query.fcgi?db=gene&cmd=Retrieve&dopt=full_report&list_uids=5290) | phosphatidylinositol-4,5-bisphosphate 3-kinase catalytic subunit alpha |
| [Details](http://mirdb.org/cgi-bin/target_detail.cgi?targetID=3072954) | 234 | 91 | hsa-miR-27a-3p | [PSEN1](http://www.ncbi.nlm.nih.gov/entrez/query.fcgi?db=gene&cmd=Retrieve&dopt=full_report&list_uids=5663) | presenilin 1 |
| [Details](http://mirdb.org/cgi-bin/target_detail.cgi?targetID=3072975) | 235 | 91 | hsa-miR-27a-3p | [OAF](http://www.ncbi.nlm.nih.gov/entrez/query.fcgi?db=gene&cmd=Retrieve&dopt=full_report&list_uids=220323) | out at first homolog |
| [Details](http://mirdb.org/cgi-bin/target_detail.cgi?targetID=3073067) | 236 | 91 | hsa-miR-27a-3p | [GALNT3](http://www.ncbi.nlm.nih.gov/entrez/query.fcgi?db=gene&cmd=Retrieve&dopt=full_report&list_uids=2591) | polypeptide N-acetylgalactosaminyltransferase 3 |
| [Details](http://mirdb.org/cgi-bin/target_detail.cgi?targetID=3073076) | 237 | 91 | hsa-miR-27a-3p | [PLCH1](http://www.ncbi.nlm.nih.gov/entrez/query.fcgi?db=gene&cmd=Retrieve&dopt=full_report&list_uids=23007) | phospholipase C eta 1 |
| [Details](http://mirdb.org/cgi-bin/target_detail.cgi?targetID=3073129) | 238 | 91 | hsa-miR-27a-3p | [NDUFS4](http://www.ncbi.nlm.nih.gov/entrez/query.fcgi?db=gene&cmd=Retrieve&dopt=full_report&list_uids=4724) | NADH:ubiquinone oxidoreductase subunit S4 |
| [Details](http://mirdb.org/cgi-bin/target_detail.cgi?targetID=3073130) | 239 | 91 | hsa-miR-27a-3p | [SORL1](http://www.ncbi.nlm.nih.gov/entrez/query.fcgi?db=gene&cmd=Retrieve&dopt=full_report&list_uids=6653) | sortilin related receptor 1 |
| [Details](http://mirdb.org/cgi-bin/target_detail.cgi?targetID=3073137) | 240 | 91 | hsa-miR-27a-3p | [MOCS3](http://www.ncbi.nlm.nih.gov/entrez/query.fcgi?db=gene&cmd=Retrieve&dopt=full_report&list_uids=27304) | molybdenum cofactor synthesis 3 |
| [Details](http://mirdb.org/cgi-bin/target_detail.cgi?targetID=3073160) | 241 | 91 | hsa-miR-27a-3p | [HIC1](http://www.ncbi.nlm.nih.gov/entrez/query.fcgi?db=gene&cmd=Retrieve&dopt=full_report&list_uids=3090) | HIC ZBTB transcriptional repressor 1 |
| [Details](http://mirdb.org/cgi-bin/target_detail.cgi?targetID=3073282) | 242 | 91 | hsa-miR-27a-3p | [GPD2](http://www.ncbi.nlm.nih.gov/entrez/query.fcgi?db=gene&cmd=Retrieve&dopt=full_report&list_uids=2820) | glycerol-3-phosphate dehydrogenase 2 |
| [Details](http://mirdb.org/cgi-bin/target_detail.cgi?targetID=3073297) | 243 | 91 | hsa-miR-27a-3p | [PKIA](http://www.ncbi.nlm.nih.gov/entrez/query.fcgi?db=gene&cmd=Retrieve&dopt=full_report&list_uids=5569) | cAMP-dependent protein kinase inhibitor alpha |
| [Details](http://mirdb.org/cgi-bin/target_detail.cgi?targetID=3073357) | 244 | 91 | hsa-miR-27a-3p | [SUCO](http://www.ncbi.nlm.nih.gov/entrez/query.fcgi?db=gene&cmd=Retrieve&dopt=full_report&list_uids=51430) | SUN domain containing ossification factor |
| [Details](http://mirdb.org/cgi-bin/target_detail.cgi?targetID=3073379) | 245 | 91 | hsa-miR-27a-3p | [SOGA1](http://www.ncbi.nlm.nih.gov/entrez/query.fcgi?db=gene&cmd=Retrieve&dopt=full_report&list_uids=140710) | suppressor of glucose, autophagy associated 1 |
| [Details](http://mirdb.org/cgi-bin/target_detail.cgi?targetID=3073491) | 246 | 91 | hsa-miR-27a-3p | [SMOC2](http://www.ncbi.nlm.nih.gov/entrez/query.fcgi?db=gene&cmd=Retrieve&dopt=full_report&list_uids=64094) | SPARC related modular calcium binding 2 |
| [Details](http://mirdb.org/cgi-bin/target_detail.cgi?targetID=3073524) | 247 | 91 | hsa-miR-27a-3p | [ARL4C](http://www.ncbi.nlm.nih.gov/entrez/query.fcgi?db=gene&cmd=Retrieve&dopt=full_report&list_uids=10123) | ADP ribosylation factor like GTPase 4C |
| [Details](http://mirdb.org/cgi-bin/target_detail.cgi?targetID=3073536) | 248 | 91 | hsa-miR-27a-3p | [TXLNG](http://www.ncbi.nlm.nih.gov/entrez/query.fcgi?db=gene&cmd=Retrieve&dopt=full_report&list_uids=55787) | taxilin gamma |
| [Details](http://mirdb.org/cgi-bin/target_detail.cgi?targetID=3073550) | 249 | 91 | hsa-miR-27a-3p | [EGFR](http://www.ncbi.nlm.nih.gov/entrez/query.fcgi?db=gene&cmd=Retrieve&dopt=full_report&list_uids=1956) | epidermal growth factor receptor |
| [Details](http://mirdb.org/cgi-bin/target_detail.cgi?targetID=3073564) | 250 | 91 | hsa-miR-27a-3p | [FRS3](http://www.ncbi.nlm.nih.gov/entrez/query.fcgi?db=gene&cmd=Retrieve&dopt=full_report&list_uids=10817) | fibroblast growth factor receptor substrate 3 |
| [Details](http://mirdb.org/cgi-bin/target_detail.cgi?targetID=3073583) | 251 | 91 | hsa-miR-27a-3p | [TMEM189-UBE2V1](http://www.ncbi.nlm.nih.gov/entrez/query.fcgi?db=gene&cmd=Retrieve&dopt=full_report&list_uids=387522) | TMEM189-UBE2V1 readthrough |
| [Details](http://mirdb.org/cgi-bin/target_detail.cgi?targetID=3073590) | 252 | 91 | hsa-miR-27a-3p | [NXT2](http://www.ncbi.nlm.nih.gov/entrez/query.fcgi?db=gene&cmd=Retrieve&dopt=full_report&list_uids=55916) | nuclear transport factor 2 like export factor 2 |
| [Details](http://mirdb.org/cgi-bin/target_detail.cgi?targetID=3073599) | 253 | 91 | hsa-miR-27a-3p | [STAC](http://www.ncbi.nlm.nih.gov/entrez/query.fcgi?db=gene&cmd=Retrieve&dopt=full_report&list_uids=6769) | SH3 and cysteine rich domain |
| [Details](http://mirdb.org/cgi-bin/target_detail.cgi?targetID=3073662) | 254 | 91 | hsa-miR-27a-3p | [SLC22A23](http://www.ncbi.nlm.nih.gov/entrez/query.fcgi?db=gene&cmd=Retrieve&dopt=full_report&list_uids=63027) | solute carrier family 22 member 23 |
| [Details](http://mirdb.org/cgi-bin/target_detail.cgi?targetID=3073708) | 255 | 91 | hsa-miR-27a-3p | [LCOR](http://www.ncbi.nlm.nih.gov/entrez/query.fcgi?db=gene&cmd=Retrieve&dopt=full_report&list_uids=84458) | ligand dependent nuclear receptor corepressor |
| [Details](http://mirdb.org/cgi-bin/target_detail.cgi?targetID=3073730) | 256 | 91 | hsa-miR-27a-3p | [NPTN](http://www.ncbi.nlm.nih.gov/entrez/query.fcgi?db=gene&cmd=Retrieve&dopt=full_report&list_uids=27020) | neuroplastin |
| [Details](http://mirdb.org/cgi-bin/target_detail.cgi?targetID=3073734) | 257 | 91 | hsa-miR-27a-3p | [RELN](http://www.ncbi.nlm.nih.gov/entrez/query.fcgi?db=gene&cmd=Retrieve&dopt=full_report&list_uids=5649) | reelin |
| [Details](http://mirdb.org/cgi-bin/target_detail.cgi?targetID=3073759) | 258 | 91 | hsa-miR-27a-3p | [KLHL29](http://www.ncbi.nlm.nih.gov/entrez/query.fcgi?db=gene&cmd=Retrieve&dopt=full_report&list_uids=114818) | kelch like family member 29 |
| [Details](http://mirdb.org/cgi-bin/target_detail.cgi?targetID=3073783) | 259 | 91 | hsa-miR-27a-3p | [COLEC10](http://www.ncbi.nlm.nih.gov/entrez/query.fcgi?db=gene&cmd=Retrieve&dopt=full_report&list_uids=10584) | collectin subfamily member 10 |
| [Details](http://mirdb.org/cgi-bin/target_detail.cgi?targetID=3073790) | 260 | 91 | hsa-miR-27a-3p | [PTGDR](http://www.ncbi.nlm.nih.gov/entrez/query.fcgi?db=gene&cmd=Retrieve&dopt=full_report&list_uids=5729) | prostaglandin D2 receptor |
| [Details](http://mirdb.org/cgi-bin/target_detail.cgi?targetID=3073800) | 261 | 91 | hsa-miR-27a-3p | [ANK1](http://www.ncbi.nlm.nih.gov/entrez/query.fcgi?db=gene&cmd=Retrieve&dopt=full_report&list_uids=286) | ankyrin 1 |
| [Details](http://mirdb.org/cgi-bin/target_detail.cgi?targetID=3073843) | 262 | 91 | hsa-miR-27a-3p | [PAX9](http://www.ncbi.nlm.nih.gov/entrez/query.fcgi?db=gene&cmd=Retrieve&dopt=full_report&list_uids=5083) | paired box 9 |
| [Details](http://mirdb.org/cgi-bin/target_detail.cgi?targetID=3073907) | 263 | 91 | hsa-miR-27a-3p | [ZMYM4](http://www.ncbi.nlm.nih.gov/entrez/query.fcgi?db=gene&cmd=Retrieve&dopt=full_report&list_uids=9202) | zinc finger MYM-type containing 4 |
| [Details](http://mirdb.org/cgi-bin/target_detail.cgi?targetID=3072455) | 264 | 90 | hsa-miR-27a-3p | [SETD5](http://www.ncbi.nlm.nih.gov/entrez/query.fcgi?db=gene&cmd=Retrieve&dopt=full_report&list_uids=55209) | SET domain containing 5 |
| [Details](http://mirdb.org/cgi-bin/target_detail.cgi?targetID=3072466) | 265 | 90 | hsa-miR-27a-3p | [CCM2](http://www.ncbi.nlm.nih.gov/entrez/query.fcgi?db=gene&cmd=Retrieve&dopt=full_report&list_uids=83605) | CCM2 scaffold protein |
| [Details](http://mirdb.org/cgi-bin/target_detail.cgi?targetID=3072500) | 266 | 90 | hsa-miR-27a-3p | [COMMD3-BMI1](http://www.ncbi.nlm.nih.gov/entrez/query.fcgi?db=gene&cmd=Retrieve&dopt=full_report&list_uids=100532731) | COMMD3-BMI1 readthrough |
| [Details](http://mirdb.org/cgi-bin/target_detail.cgi?targetID=3072523) | 267 | 90 | hsa-miR-27a-3p | [ATP2B1](http://www.ncbi.nlm.nih.gov/entrez/query.fcgi?db=gene&cmd=Retrieve&dopt=full_report&list_uids=490) | ATPase plasma membrane Ca2+ transporting 1 |
| [Details](http://mirdb.org/cgi-bin/target_detail.cgi?targetID=3072555) | 268 | 90 | hsa-miR-27a-3p | [USF3](http://www.ncbi.nlm.nih.gov/entrez/query.fcgi?db=gene&cmd=Retrieve&dopt=full_report&list_uids=205717) | upstream transcription factor family member 3 |
| [Details](http://mirdb.org/cgi-bin/target_detail.cgi?targetID=3072603) | 269 | 90 | hsa-miR-27a-3p | [OTULIN](http://www.ncbi.nlm.nih.gov/entrez/query.fcgi?db=gene&cmd=Retrieve&dopt=full_report&list_uids=90268) | OTU deubiquitinase with linear linkage specificity |
| [Details](http://mirdb.org/cgi-bin/target_detail.cgi?targetID=3072679) | 270 | 90 | hsa-miR-27a-3p | [FZD4](http://www.ncbi.nlm.nih.gov/entrez/query.fcgi?db=gene&cmd=Retrieve&dopt=full_report&list_uids=8322) | frizzled class receptor 4 |
| [Details](http://mirdb.org/cgi-bin/target_detail.cgi?targetID=3072742) | 271 | 90 | hsa-miR-27a-3p | [HSD17B12](http://www.ncbi.nlm.nih.gov/entrez/query.fcgi?db=gene&cmd=Retrieve&dopt=full_report&list_uids=51144) | hydroxysteroid 17-beta dehydrogenase 12 |
| [Details](http://mirdb.org/cgi-bin/target_detail.cgi?targetID=3072759) | 272 | 90 | hsa-miR-27a-3p | [HOXC6](http://www.ncbi.nlm.nih.gov/entrez/query.fcgi?db=gene&cmd=Retrieve&dopt=full_report&list_uids=3223) | homeobox C6 |
| [Details](http://mirdb.org/cgi-bin/target_detail.cgi?targetID=3072782) | 273 | 90 | hsa-miR-27a-3p | [CAPN15](http://www.ncbi.nlm.nih.gov/entrez/query.fcgi?db=gene&cmd=Retrieve&dopt=full_report&list_uids=6650) | calpain 15 |
| [Details](http://mirdb.org/cgi-bin/target_detail.cgi?targetID=3072798) | 274 | 90 | hsa-miR-27a-3p | [NEURL4](http://www.ncbi.nlm.nih.gov/entrez/query.fcgi?db=gene&cmd=Retrieve&dopt=full_report&list_uids=84461) | neuralized E3 ubiquitin protein ligase 4 |
| [Details](http://mirdb.org/cgi-bin/target_detail.cgi?targetID=3072805) | 275 | 90 | hsa-miR-27a-3p | [TRIM50](http://www.ncbi.nlm.nih.gov/entrez/query.fcgi?db=gene&cmd=Retrieve&dopt=full_report&list_uids=135892) | tripartite motif containing 50 |
| [Details](http://mirdb.org/cgi-bin/target_detail.cgi?targetID=3072812) | 276 | 90 | hsa-miR-27a-3p | [GATA6](http://www.ncbi.nlm.nih.gov/entrez/query.fcgi?db=gene&cmd=Retrieve&dopt=full_report&list_uids=2627) | GATA binding protein 6 |
| [Details](http://mirdb.org/cgi-bin/target_detail.cgi?targetID=3072835) | 277 | 90 | hsa-miR-27a-3p | [RARA](http://www.ncbi.nlm.nih.gov/entrez/query.fcgi?db=gene&cmd=Retrieve&dopt=full_report&list_uids=5914) | retinoic acid receptor alpha |
| [Details](http://mirdb.org/cgi-bin/target_detail.cgi?targetID=3072838) | 278 | 90 | hsa-miR-27a-3p | [FRMD6](http://www.ncbi.nlm.nih.gov/entrez/query.fcgi?db=gene&cmd=Retrieve&dopt=full_report&list_uids=122786) | FERM domain containing 6 |
| [Details](http://mirdb.org/cgi-bin/target_detail.cgi?targetID=3072851) | 279 | 90 | hsa-miR-27a-3p | [ZNF329](http://www.ncbi.nlm.nih.gov/entrez/query.fcgi?db=gene&cmd=Retrieve&dopt=full_report&list_uids=79673) | zinc finger protein 329 |
| [Details](http://mirdb.org/cgi-bin/target_detail.cgi?targetID=3072997) | 280 | 90 | hsa-miR-27a-3p | [SIX1](http://www.ncbi.nlm.nih.gov/entrez/query.fcgi?db=gene&cmd=Retrieve&dopt=full_report&list_uids=6495) | SIX homeobox 1 |
| [Details](http://mirdb.org/cgi-bin/target_detail.cgi?targetID=3073007) | 281 | 90 | hsa-miR-27a-3p | [AQP11](http://www.ncbi.nlm.nih.gov/entrez/query.fcgi?db=gene&cmd=Retrieve&dopt=full_report&list_uids=282679) | aquaporin 11 |
| [Details](http://mirdb.org/cgi-bin/target_detail.cgi?targetID=3073021) | 282 | 90 | hsa-miR-27a-3p | [PSPC1](http://www.ncbi.nlm.nih.gov/entrez/query.fcgi?db=gene&cmd=Retrieve&dopt=full_report&list_uids=55269) | paraspeckle component 1 |
| [Details](http://mirdb.org/cgi-bin/target_detail.cgi?targetID=3073030) | 283 | 90 | hsa-miR-27a-3p | [NEK6](http://www.ncbi.nlm.nih.gov/entrez/query.fcgi?db=gene&cmd=Retrieve&dopt=full_report&list_uids=10783) | NIMA related kinase 6 |
| [Details](http://mirdb.org/cgi-bin/target_detail.cgi?targetID=3073062) | 284 | 90 | hsa-miR-27a-3p | [PDPK1](http://www.ncbi.nlm.nih.gov/entrez/query.fcgi?db=gene&cmd=Retrieve&dopt=full_report&list_uids=5170) | 3-phosphoinositide dependent protein kinase 1 |
| [Details](http://mirdb.org/cgi-bin/target_detail.cgi?targetID=3073132) | 285 | 90 | hsa-miR-27a-3p | [COLGALT2](http://www.ncbi.nlm.nih.gov/entrez/query.fcgi?db=gene&cmd=Retrieve&dopt=full_report&list_uids=23127) | collagen beta(1-O)galactosyltransferase 2 |
| [Details](http://mirdb.org/cgi-bin/target_detail.cgi?targetID=3073174) | 286 | 90 | hsa-miR-27a-3p | [SLC9B1](http://www.ncbi.nlm.nih.gov/entrez/query.fcgi?db=gene&cmd=Retrieve&dopt=full_report&list_uids=150159) | solute carrier family 9 member B1 |
| [Details](http://mirdb.org/cgi-bin/target_detail.cgi?targetID=3073193) | 287 | 90 | hsa-miR-27a-3p | [PLXND1](http://www.ncbi.nlm.nih.gov/entrez/query.fcgi?db=gene&cmd=Retrieve&dopt=full_report&list_uids=23129) | plexin D1 |
| [Details](http://mirdb.org/cgi-bin/target_detail.cgi?targetID=3073206) | 288 | 90 | hsa-miR-27a-3p | [PATZ1](http://www.ncbi.nlm.nih.gov/entrez/query.fcgi?db=gene&cmd=Retrieve&dopt=full_report&list_uids=23598) | POZ/BTB and AT hook containing zinc finger 1 |
| [Details](http://mirdb.org/cgi-bin/target_detail.cgi?targetID=3073226) | 289 | 90 | hsa-miR-27a-3p | [PLPPR1](http://www.ncbi.nlm.nih.gov/entrez/query.fcgi?db=gene&cmd=Retrieve&dopt=full_report&list_uids=54886) | phospholipid phosphatase related 1 |
| [Details](http://mirdb.org/cgi-bin/target_detail.cgi?targetID=3073229) | 290 | 90 | hsa-miR-27a-3p | [MED14](http://www.ncbi.nlm.nih.gov/entrez/query.fcgi?db=gene&cmd=Retrieve&dopt=full_report&list_uids=9282) | mediator complex subunit 14 |
| [Details](http://mirdb.org/cgi-bin/target_detail.cgi?targetID=3073322) | 291 | 90 | hsa-miR-27a-3p | [RSBN1L](http://www.ncbi.nlm.nih.gov/entrez/query.fcgi?db=gene&cmd=Retrieve&dopt=full_report&list_uids=222194) | round spermatid basic protein 1 like |
| [Details](http://mirdb.org/cgi-bin/target_detail.cgi?targetID=3073349) | 292 | 90 | hsa-miR-27a-3p | [ZNF268](http://www.ncbi.nlm.nih.gov/entrez/query.fcgi?db=gene&cmd=Retrieve&dopt=full_report&list_uids=10795) | zinc finger protein 268 |
| [Details](http://mirdb.org/cgi-bin/target_detail.cgi?targetID=3073391) | 293 | 90 | hsa-miR-27a-3p | [RSPO3](http://www.ncbi.nlm.nih.gov/entrez/query.fcgi?db=gene&cmd=Retrieve&dopt=full_report&list_uids=84870) | R-spondin 3 |
| [Details](http://mirdb.org/cgi-bin/target_detail.cgi?targetID=3073413) | 294 | 90 | hsa-miR-27a-3p | [NRBF2](http://www.ncbi.nlm.nih.gov/entrez/query.fcgi?db=gene&cmd=Retrieve&dopt=full_report&list_uids=29982) | nuclear receptor binding factor 2 |
| [Details](http://mirdb.org/cgi-bin/target_detail.cgi?targetID=3073476) | 295 | 90 | hsa-miR-27a-3p | [LITAF](http://www.ncbi.nlm.nih.gov/entrez/query.fcgi?db=gene&cmd=Retrieve&dopt=full_report&list_uids=9516) | lipopolysaccharide induced TNF factor |
| [Details](http://mirdb.org/cgi-bin/target_detail.cgi?targetID=3073584) | 296 | 90 | hsa-miR-27a-3p | [TRAPPC8](http://www.ncbi.nlm.nih.gov/entrez/query.fcgi?db=gene&cmd=Retrieve&dopt=full_report&list_uids=22878) | trafficking protein particle complex 8 |
| [Details](http://mirdb.org/cgi-bin/target_detail.cgi?targetID=3073605) | 297 | 90 | hsa-miR-27a-3p | [RPGRIP1L](http://www.ncbi.nlm.nih.gov/entrez/query.fcgi?db=gene&cmd=Retrieve&dopt=full_report&list_uids=23322) | RPGRIP1 like |
| [Details](http://mirdb.org/cgi-bin/target_detail.cgi?targetID=3073675) | 298 | 90 | hsa-miR-27a-3p | [ZDHHC17](http://www.ncbi.nlm.nih.gov/entrez/query.fcgi?db=gene&cmd=Retrieve&dopt=full_report&list_uids=23390) | zinc finger DHHC-type containing 17 |
| [Details](http://mirdb.org/cgi-bin/target_detail.cgi?targetID=3073724) | 299 | 90 | hsa-miR-27a-3p | [NCALD](http://www.ncbi.nlm.nih.gov/entrez/query.fcgi?db=gene&cmd=Retrieve&dopt=full_report&list_uids=83988) | neurocalcin delta |
| [Details](http://mirdb.org/cgi-bin/target_detail.cgi?targetID=3073787) | 300 | 90 | hsa-miR-27a-3p | [PF4V1](http://www.ncbi.nlm.nih.gov/entrez/query.fcgi?db=gene&cmd=Retrieve&dopt=full_report&list_uids=5197) | platelet factor 4 variant 1 |
| [Details](http://mirdb.org/cgi-bin/target_detail.cgi?targetID=3073818) | 301 | 90 | hsa-miR-27a-3p | [EPB41](http://www.ncbi.nlm.nih.gov/entrez/query.fcgi?db=gene&cmd=Retrieve&dopt=full_report&list_uids=2035) | erythrocyte membrane protein band 4.1 |
| [Details](http://mirdb.org/cgi-bin/target_detail.cgi?targetID=3073839) | 302 | 90 | hsa-miR-27a-3p | [KPNB1](http://www.ncbi.nlm.nih.gov/entrez/query.fcgi?db=gene&cmd=Retrieve&dopt=full_report&list_uids=3837) | karyopherin subunit beta 1 |
| [Details](http://mirdb.org/cgi-bin/target_detail.cgi?targetID=3073859) | 303 | 90 | hsa-miR-27a-3p | [TMEM9B](http://www.ncbi.nlm.nih.gov/entrez/query.fcgi?db=gene&cmd=Retrieve&dopt=full_report&list_uids=56674) | TMEM9 domain family member B |
| [Details](http://mirdb.org/cgi-bin/target_detail.cgi?targetID=3073868) | 304 | 90 | hsa-miR-27a-3p | [MKLN1](http://www.ncbi.nlm.nih.gov/entrez/query.fcgi?db=gene&cmd=Retrieve&dopt=full_report&list_uids=4289) | muskelin 1 |
| [Details](http://mirdb.org/cgi-bin/target_detail.cgi?targetID=3073878) | 305 | 90 | hsa-miR-27a-3p | [TCIM](http://www.ncbi.nlm.nih.gov/entrez/query.fcgi?db=gene&cmd=Retrieve&dopt=full_report&list_uids=56892) | transcriptional and immune response regulator |
| [Details](http://mirdb.org/cgi-bin/target_detail.cgi?targetID=3073915) | 306 | 90 | hsa-miR-27a-3p | [CDH11](http://www.ncbi.nlm.nih.gov/entrez/query.fcgi?db=gene&cmd=Retrieve&dopt=full_report&list_uids=1009) | cadherin 11 |
| [Details](http://mirdb.org/cgi-bin/target_detail.cgi?targetID=3072486) | 307 | 89 | hsa-miR-27a-3p | [ZNF80](http://www.ncbi.nlm.nih.gov/entrez/query.fcgi?db=gene&cmd=Retrieve&dopt=full_report&list_uids=7634) | zinc finger protein 80 |
| [Details](http://mirdb.org/cgi-bin/target_detail.cgi?targetID=3072539) | 308 | 89 | hsa-miR-27a-3p | [SLC24A1](http://www.ncbi.nlm.nih.gov/entrez/query.fcgi?db=gene&cmd=Retrieve&dopt=full_report&list_uids=9187) | solute carrier family 24 member 1 |
| [Details](http://mirdb.org/cgi-bin/target_detail.cgi?targetID=3072543) | 309 | 89 | hsa-miR-27a-3p | [ZMAT3](http://www.ncbi.nlm.nih.gov/entrez/query.fcgi?db=gene&cmd=Retrieve&dopt=full_report&list_uids=64393) | zinc finger matrin-type 3 |
| [Details](http://mirdb.org/cgi-bin/target_detail.cgi?targetID=3072547) | 310 | 89 | hsa-miR-27a-3p | [PRKG1](http://www.ncbi.nlm.nih.gov/entrez/query.fcgi?db=gene&cmd=Retrieve&dopt=full_report&list_uids=5592) | protein kinase cGMP-dependent 1 |
| [Details](http://mirdb.org/cgi-bin/target_detail.cgi?targetID=3072559) | 311 | 89 | hsa-miR-27a-3p | [CDK18](http://www.ncbi.nlm.nih.gov/entrez/query.fcgi?db=gene&cmd=Retrieve&dopt=full_report&list_uids=5129) | cyclin dependent kinase 18 |
| [Details](http://mirdb.org/cgi-bin/target_detail.cgi?targetID=3072575) | 312 | 89 | hsa-miR-27a-3p | [INPP1](http://www.ncbi.nlm.nih.gov/entrez/query.fcgi?db=gene&cmd=Retrieve&dopt=full_report&list_uids=3628) | inositol polyphosphate-1-phosphatase |
| [Details](http://mirdb.org/cgi-bin/target_detail.cgi?targetID=3072581) | 313 | 89 | hsa-miR-27a-3p | [DDX5](http://www.ncbi.nlm.nih.gov/entrez/query.fcgi?db=gene&cmd=Retrieve&dopt=full_report&list_uids=1655) | DEAD-box helicase 5 |
| [Details](http://mirdb.org/cgi-bin/target_detail.cgi?targetID=3072589) | 314 | 89 | hsa-miR-27a-3p | [BEST1](http://www.ncbi.nlm.nih.gov/entrez/query.fcgi?db=gene&cmd=Retrieve&dopt=full_report&list_uids=7439) | bestrophin 1 |
| [Details](http://mirdb.org/cgi-bin/target_detail.cgi?targetID=3072654) | 315 | 89 | hsa-miR-27a-3p | [GNG12](http://www.ncbi.nlm.nih.gov/entrez/query.fcgi?db=gene&cmd=Retrieve&dopt=full_report&list_uids=55970) | G protein subunit gamma 12 |
| [Details](http://mirdb.org/cgi-bin/target_detail.cgi?targetID=3072702) | 316 | 89 | hsa-miR-27a-3p | [ABL2](http://www.ncbi.nlm.nih.gov/entrez/query.fcgi?db=gene&cmd=Retrieve&dopt=full_report&list_uids=27) | ABL proto-oncogene 2, non-receptor tyrosine kinase |
| [Details](http://mirdb.org/cgi-bin/target_detail.cgi?targetID=3072715) | 317 | 89 | hsa-miR-27a-3p | [ASAH1](http://www.ncbi.nlm.nih.gov/entrez/query.fcgi?db=gene&cmd=Retrieve&dopt=full_report&list_uids=427) | N-acylsphingosine amidohydrolase 1 |
| [Details](http://mirdb.org/cgi-bin/target_detail.cgi?targetID=3072736) | 318 | 89 | hsa-miR-27a-3p | [TMA7](http://www.ncbi.nlm.nih.gov/entrez/query.fcgi?db=gene&cmd=Retrieve&dopt=full_report&list_uids=51372) | translation machinery associated 7 homolog |
| [Details](http://mirdb.org/cgi-bin/target_detail.cgi?targetID=3072837) | 319 | 89 | hsa-miR-27a-3p | [POGLUT1](http://www.ncbi.nlm.nih.gov/entrez/query.fcgi?db=gene&cmd=Retrieve&dopt=full_report&list_uids=56983) | protein O-glucosyltransferase 1 |
| [Details](http://mirdb.org/cgi-bin/target_detail.cgi?targetID=3072886) | 320 | 89 | hsa-miR-27a-3p | [TOR2A](http://www.ncbi.nlm.nih.gov/entrez/query.fcgi?db=gene&cmd=Retrieve&dopt=full_report&list_uids=27433) | torsin family 2 member A |
| [Details](http://mirdb.org/cgi-bin/target_detail.cgi?targetID=3073029) | 321 | 89 | hsa-miR-27a-3p | [ITGA8](http://www.ncbi.nlm.nih.gov/entrez/query.fcgi?db=gene&cmd=Retrieve&dopt=full_report&list_uids=8516) | integrin subunit alpha 8 |
| [Details](http://mirdb.org/cgi-bin/target_detail.cgi?targetID=3073100) | 322 | 89 | hsa-miR-27a-3p | [OTX2](http://www.ncbi.nlm.nih.gov/entrez/query.fcgi?db=gene&cmd=Retrieve&dopt=full_report&list_uids=5015) | orthodenticle homeobox 2 |
| [Details](http://mirdb.org/cgi-bin/target_detail.cgi?targetID=3073111) | 323 | 89 | hsa-miR-27a-3p | [GATA3](http://www.ncbi.nlm.nih.gov/entrez/query.fcgi?db=gene&cmd=Retrieve&dopt=full_report&list_uids=2625) | GATA binding protein 3 |
| [Details](http://mirdb.org/cgi-bin/target_detail.cgi?targetID=3073197) | 324 | 89 | hsa-miR-27a-3p | [ARX](http://www.ncbi.nlm.nih.gov/entrez/query.fcgi?db=gene&cmd=Retrieve&dopt=full_report&list_uids=170302) | aristaless related homeobox |
| [Details](http://mirdb.org/cgi-bin/target_detail.cgi?targetID=3073237) | 325 | 89 | hsa-miR-27a-3p | [RUFY3](http://www.ncbi.nlm.nih.gov/entrez/query.fcgi?db=gene&cmd=Retrieve&dopt=full_report&list_uids=22902) | RUN and FYVE domain containing 3 |
| [Details](http://mirdb.org/cgi-bin/target_detail.cgi?targetID=3073248) | 326 | 89 | hsa-miR-27a-3p | [NRARP](http://www.ncbi.nlm.nih.gov/entrez/query.fcgi?db=gene&cmd=Retrieve&dopt=full_report&list_uids=441478) | NOTCH regulated ankyrin repeat protein |
| [Details](http://mirdb.org/cgi-bin/target_detail.cgi?targetID=3073373) | 327 | 89 | hsa-miR-27a-3p | [PDHX](http://www.ncbi.nlm.nih.gov/entrez/query.fcgi?db=gene&cmd=Retrieve&dopt=full_report&list_uids=8050) | pyruvate dehydrogenase complex component X |
| [Details](http://mirdb.org/cgi-bin/target_detail.cgi?targetID=3073374) | 328 | 89 | hsa-miR-27a-3p | [RBPMS2](http://www.ncbi.nlm.nih.gov/entrez/query.fcgi?db=gene&cmd=Retrieve&dopt=full_report&list_uids=348093) | RNA binding protein, mRNA processing factor 2 |
| [Details](http://mirdb.org/cgi-bin/target_detail.cgi?targetID=3073388) | 329 | 89 | hsa-miR-27a-3p | [TOM1L1](http://www.ncbi.nlm.nih.gov/entrez/query.fcgi?db=gene&cmd=Retrieve&dopt=full_report&list_uids=10040) | target of myb1 like 1 membrane trafficking protein |
| [Details](http://mirdb.org/cgi-bin/target_detail.cgi?targetID=3073435) | 330 | 89 | hsa-miR-27a-3p | [CACNA2D3](http://www.ncbi.nlm.nih.gov/entrez/query.fcgi?db=gene&cmd=Retrieve&dopt=full_report&list_uids=55799) | calcium voltage-gated channel auxiliary subunit alpha2delta 3 |
| [Details](http://mirdb.org/cgi-bin/target_detail.cgi?targetID=3073445) | 331 | 89 | hsa-miR-27a-3p | [WSB1](http://www.ncbi.nlm.nih.gov/entrez/query.fcgi?db=gene&cmd=Retrieve&dopt=full_report&list_uids=26118) | WD repeat and SOCS box containing 1 |
| [Details](http://mirdb.org/cgi-bin/target_detail.cgi?targetID=3073533) | 332 | 89 | hsa-miR-27a-3p | [MAPK14](http://www.ncbi.nlm.nih.gov/entrez/query.fcgi?db=gene&cmd=Retrieve&dopt=full_report&list_uids=1432) | mitogen-activated protein kinase 14 |
| [Details](http://mirdb.org/cgi-bin/target_detail.cgi?targetID=3073540) | 333 | 89 | hsa-miR-27a-3p | [GLRA2](http://www.ncbi.nlm.nih.gov/entrez/query.fcgi?db=gene&cmd=Retrieve&dopt=full_report&list_uids=2742) | glycine receptor alpha 2 |
| [Details](http://mirdb.org/cgi-bin/target_detail.cgi?targetID=3073557) | 334 | 89 | hsa-miR-27a-3p | [GFPT2](http://www.ncbi.nlm.nih.gov/entrez/query.fcgi?db=gene&cmd=Retrieve&dopt=full_report&list_uids=9945) | glutamine-fructose-6-phosphate transaminase 2 |
| [Details](http://mirdb.org/cgi-bin/target_detail.cgi?targetID=3073630) | 335 | 89 | hsa-miR-27a-3p | [CCNJ](http://www.ncbi.nlm.nih.gov/entrez/query.fcgi?db=gene&cmd=Retrieve&dopt=full_report&list_uids=54619) | cyclin J |
| [Details](http://mirdb.org/cgi-bin/target_detail.cgi?targetID=3073683) | 336 | 89 | hsa-miR-27a-3p | [MAP3K4](http://www.ncbi.nlm.nih.gov/entrez/query.fcgi?db=gene&cmd=Retrieve&dopt=full_report&list_uids=4216) | mitogen-activated protein kinase kinase kinase 4 |
| [Details](http://mirdb.org/cgi-bin/target_detail.cgi?targetID=3073694) | 337 | 89 | hsa-miR-27a-3p | [DKK2](http://www.ncbi.nlm.nih.gov/entrez/query.fcgi?db=gene&cmd=Retrieve&dopt=full_report&list_uids=27123) | dickkopf WNT signaling pathway inhibitor 2 |
| [Details](http://mirdb.org/cgi-bin/target_detail.cgi?targetID=3073757) | 338 | 89 | hsa-miR-27a-3p | [RASSF3](http://www.ncbi.nlm.nih.gov/entrez/query.fcgi?db=gene&cmd=Retrieve&dopt=full_report&list_uids=283349) | Ras association domain family member 3 |
| [Details](http://mirdb.org/cgi-bin/target_detail.cgi?targetID=3073763) | 339 | 89 | hsa-miR-27a-3p | [ZBTB20](http://www.ncbi.nlm.nih.gov/entrez/query.fcgi?db=gene&cmd=Retrieve&dopt=full_report&list_uids=26137) | zinc finger and BTB domain containing 20 |
| [Details](http://mirdb.org/cgi-bin/target_detail.cgi?targetID=3073767) | 340 | 89 | hsa-miR-27a-3p | [EN2](http://www.ncbi.nlm.nih.gov/entrez/query.fcgi?db=gene&cmd=Retrieve&dopt=full_report&list_uids=2020) | engrailed homeobox 2 |
| [Details](http://mirdb.org/cgi-bin/target_detail.cgi?targetID=3073805) | 341 | 89 | hsa-miR-27a-3p | [RNGTT](http://www.ncbi.nlm.nih.gov/entrez/query.fcgi?db=gene&cmd=Retrieve&dopt=full_report&list_uids=8732) | RNA guanylyltransferase and 5'-phosphatase |
| [Details](http://mirdb.org/cgi-bin/target_detail.cgi?targetID=3073808) | 342 | 89 | hsa-miR-27a-3p | [NEURL1B](http://www.ncbi.nlm.nih.gov/entrez/query.fcgi?db=gene&cmd=Retrieve&dopt=full_report&list_uids=54492) | neuralized E3 ubiquitin protein ligase 1B |
| [Details](http://mirdb.org/cgi-bin/target_detail.cgi?targetID=3073848) | 343 | 89 | hsa-miR-27a-3p | [MS4A7](http://www.ncbi.nlm.nih.gov/entrez/query.fcgi?db=gene&cmd=Retrieve&dopt=full_report&list_uids=58475) | membrane spanning 4-domains A7 |
| [Details](http://mirdb.org/cgi-bin/target_detail.cgi?targetID=3073921) | 344 | 89 | hsa-miR-27a-3p | [GRB2](http://www.ncbi.nlm.nih.gov/entrez/query.fcgi?db=gene&cmd=Retrieve&dopt=full_report&list_uids=2885) | growth factor receptor bound protein 2 |
| [Details](http://mirdb.org/cgi-bin/target_detail.cgi?targetID=3072587) | 345 | 88 | hsa-miR-27a-3p | [SLC30A7](http://www.ncbi.nlm.nih.gov/entrez/query.fcgi?db=gene&cmd=Retrieve&dopt=full_report&list_uids=148867) | solute carrier family 30 member 7 |
| [Details](http://mirdb.org/cgi-bin/target_detail.cgi?targetID=3072641) | 346 | 88 | hsa-miR-27a-3p | [TGFBR3](http://www.ncbi.nlm.nih.gov/entrez/query.fcgi?db=gene&cmd=Retrieve&dopt=full_report&list_uids=7049) | transforming growth factor beta receptor 3 |
| [Details](http://mirdb.org/cgi-bin/target_detail.cgi?targetID=3072678) | 347 | 88 | hsa-miR-27a-3p | [LOX](http://www.ncbi.nlm.nih.gov/entrez/query.fcgi?db=gene&cmd=Retrieve&dopt=full_report&list_uids=4015) | lysyl oxidase |
| [Details](http://mirdb.org/cgi-bin/target_detail.cgi?targetID=3072692) | 348 | 88 | hsa-miR-27a-3p | [PDE3B](http://www.ncbi.nlm.nih.gov/entrez/query.fcgi?db=gene&cmd=Retrieve&dopt=full_report&list_uids=5140) | phosphodiesterase 3B |
| [Details](http://mirdb.org/cgi-bin/target_detail.cgi?targetID=3072822) | 349 | 88 | hsa-miR-27a-3p | [CAND1](http://www.ncbi.nlm.nih.gov/entrez/query.fcgi?db=gene&cmd=Retrieve&dopt=full_report&list_uids=55832) | cullin associated and neddylation dissociated 1 |
| [Details](http://mirdb.org/cgi-bin/target_detail.cgi?targetID=3072854) | 350 | 88 | hsa-miR-27a-3p | [GPR6](http://www.ncbi.nlm.nih.gov/entrez/query.fcgi?db=gene&cmd=Retrieve&dopt=full_report&list_uids=2830) | G protein-coupled receptor 6 |
| [Details](http://mirdb.org/cgi-bin/target_detail.cgi?targetID=3072904) | 351 | 88 | hsa-miR-27a-3p | [TMEM91](http://www.ncbi.nlm.nih.gov/entrez/query.fcgi?db=gene&cmd=Retrieve&dopt=full_report&list_uids=641649) | transmembrane protein 91 |
| [Details](http://mirdb.org/cgi-bin/target_detail.cgi?targetID=3072907) | 352 | 88 | hsa-miR-27a-3p | [NKAIN1](http://www.ncbi.nlm.nih.gov/entrez/query.fcgi?db=gene&cmd=Retrieve&dopt=full_report&list_uids=79570) | sodium/potassium transporting ATPase interacting 1 |
| [Details](http://mirdb.org/cgi-bin/target_detail.cgi?targetID=3072908) | 353 | 88 | hsa-miR-27a-3p | [NAV2](http://www.ncbi.nlm.nih.gov/entrez/query.fcgi?db=gene&cmd=Retrieve&dopt=full_report&list_uids=89797) | neuron navigator 2 |
| [Details](http://mirdb.org/cgi-bin/target_detail.cgi?targetID=3072932) | 354 | 88 | hsa-miR-27a-3p | [NHLH2](http://www.ncbi.nlm.nih.gov/entrez/query.fcgi?db=gene&cmd=Retrieve&dopt=full_report&list_uids=4808) | nescient helix-loop-helix 2 |
| [Details](http://mirdb.org/cgi-bin/target_detail.cgi?targetID=3072936) | 355 | 88 | hsa-miR-27a-3p | [NRP2](http://www.ncbi.nlm.nih.gov/entrez/query.fcgi?db=gene&cmd=Retrieve&dopt=full_report&list_uids=8828) | neuropilin 2 |
| [Details](http://mirdb.org/cgi-bin/target_detail.cgi?targetID=3072958) | 356 | 88 | hsa-miR-27a-3p | [OGA](http://www.ncbi.nlm.nih.gov/entrez/query.fcgi?db=gene&cmd=Retrieve&dopt=full_report&list_uids=10724) | O-GlcNAcase |
| [Details](http://mirdb.org/cgi-bin/target_detail.cgi?targetID=3072981) | 357 | 88 | hsa-miR-27a-3p | [RNF8](http://www.ncbi.nlm.nih.gov/entrez/query.fcgi?db=gene&cmd=Retrieve&dopt=full_report&list_uids=9025) | ring finger protein 8 |
| [Details](http://mirdb.org/cgi-bin/target_detail.cgi?targetID=3073012) | 358 | 88 | hsa-miR-27a-3p | [ALDH5A1](http://www.ncbi.nlm.nih.gov/entrez/query.fcgi?db=gene&cmd=Retrieve&dopt=full_report&list_uids=7915) | aldehyde dehydrogenase 5 family member A1 |
| [Details](http://mirdb.org/cgi-bin/target_detail.cgi?targetID=3073022) | 359 | 88 | hsa-miR-27a-3p | [ATP11C](http://www.ncbi.nlm.nih.gov/entrez/query.fcgi?db=gene&cmd=Retrieve&dopt=full_report&list_uids=286410) | ATPase phospholipid transporting 11C |
| [Details](http://mirdb.org/cgi-bin/target_detail.cgi?targetID=3073039) | 360 | 88 | hsa-miR-27a-3p | [SHC4](http://www.ncbi.nlm.nih.gov/entrez/query.fcgi?db=gene&cmd=Retrieve&dopt=full_report&list_uids=399694) | SHC adaptor protein 4 |
| [Details](http://mirdb.org/cgi-bin/target_detail.cgi?targetID=3073040) | 361 | 88 | hsa-miR-27a-3p | [NCAM1](http://www.ncbi.nlm.nih.gov/entrez/query.fcgi?db=gene&cmd=Retrieve&dopt=full_report&list_uids=4684) | neural cell adhesion molecule 1 |
| [Details](http://mirdb.org/cgi-bin/target_detail.cgi?targetID=3073110) | 362 | 88 | hsa-miR-27a-3p | [MDM4](http://www.ncbi.nlm.nih.gov/entrez/query.fcgi?db=gene&cmd=Retrieve&dopt=full_report&list_uids=4194) | MDM4, p53 regulator |
| [Details](http://mirdb.org/cgi-bin/target_detail.cgi?targetID=3073121) | 363 | 88 | hsa-miR-27a-3p | [PRKX](http://www.ncbi.nlm.nih.gov/entrez/query.fcgi?db=gene&cmd=Retrieve&dopt=full_report&list_uids=5613) | protein kinase X-linked |
| [Details](http://mirdb.org/cgi-bin/target_detail.cgi?targetID=3073189) | 364 | 88 | hsa-miR-27a-3p | [TERB2](http://www.ncbi.nlm.nih.gov/entrez/query.fcgi?db=gene&cmd=Retrieve&dopt=full_report&list_uids=145645) | telomere repeat binding bouquet formation protein 2 |
| [Details](http://mirdb.org/cgi-bin/target_detail.cgi?targetID=3073205) | 365 | 88 | hsa-miR-27a-3p | [ERICH3](http://www.ncbi.nlm.nih.gov/entrez/query.fcgi?db=gene&cmd=Retrieve&dopt=full_report&list_uids=127254) | glutamate rich 3 |
| [Details](http://mirdb.org/cgi-bin/target_detail.cgi?targetID=3073225) | 366 | 88 | hsa-miR-27a-3p | [MIER2](http://www.ncbi.nlm.nih.gov/entrez/query.fcgi?db=gene&cmd=Retrieve&dopt=full_report&list_uids=54531) | MIER family member 2 |
| [Details](http://mirdb.org/cgi-bin/target_detail.cgi?targetID=3073261) | 367 | 88 | hsa-miR-27a-3p | [NSD1](http://www.ncbi.nlm.nih.gov/entrez/query.fcgi?db=gene&cmd=Retrieve&dopt=full_report&list_uids=64324) | nuclear receptor binding SET domain protein 1 |
| [Details](http://mirdb.org/cgi-bin/target_detail.cgi?targetID=3073295) | 368 | 88 | hsa-miR-27a-3p | [NR2F2](http://www.ncbi.nlm.nih.gov/entrez/query.fcgi?db=gene&cmd=Retrieve&dopt=full_report&list_uids=7026) | nuclear receptor subfamily 2 group F member 2 |
| [Details](http://mirdb.org/cgi-bin/target_detail.cgi?targetID=3073321) | 369 | 88 | hsa-miR-27a-3p | [CMKLR1](http://www.ncbi.nlm.nih.gov/entrez/query.fcgi?db=gene&cmd=Retrieve&dopt=full_report&list_uids=1240) | chemerin chemokine-like receptor 1 |
| [Details](http://mirdb.org/cgi-bin/target_detail.cgi?targetID=3073410) | 370 | 88 | hsa-miR-27a-3p | [ABCB9](http://www.ncbi.nlm.nih.gov/entrez/query.fcgi?db=gene&cmd=Retrieve&dopt=full_report&list_uids=23457) | ATP binding cassette subfamily B member 9 |
| [Details](http://mirdb.org/cgi-bin/target_detail.cgi?targetID=3073459) | 371 | 88 | hsa-miR-27a-3p | [CDC42EP3](http://www.ncbi.nlm.nih.gov/entrez/query.fcgi?db=gene&cmd=Retrieve&dopt=full_report&list_uids=10602) | CDC42 effector protein 3 |
| [Details](http://mirdb.org/cgi-bin/target_detail.cgi?targetID=3073496) | 372 | 88 | hsa-miR-27a-3p | [EFNB2](http://www.ncbi.nlm.nih.gov/entrez/query.fcgi?db=gene&cmd=Retrieve&dopt=full_report&list_uids=1948) | ephrin B2 |
| [Details](http://mirdb.org/cgi-bin/target_detail.cgi?targetID=3073545) | 373 | 88 | hsa-miR-27a-3p | [CDIP1](http://www.ncbi.nlm.nih.gov/entrez/query.fcgi?db=gene&cmd=Retrieve&dopt=full_report&list_uids=29965) | cell death inducing p53 target 1 |
| [Details](http://mirdb.org/cgi-bin/target_detail.cgi?targetID=3073572) | 374 | 88 | hsa-miR-27a-3p | [NXF1](http://www.ncbi.nlm.nih.gov/entrez/query.fcgi?db=gene&cmd=Retrieve&dopt=full_report&list_uids=10482) | nuclear RNA export factor 1 |
| [Details](http://mirdb.org/cgi-bin/target_detail.cgi?targetID=3073624) | 375 | 88 | hsa-miR-27a-3p | [ERG](http://www.ncbi.nlm.nih.gov/entrez/query.fcgi?db=gene&cmd=Retrieve&dopt=full_report&list_uids=2078) | ETS transcription factor ERG |
| [Details](http://mirdb.org/cgi-bin/target_detail.cgi?targetID=3073661) | 376 | 88 | hsa-miR-27a-3p | [INA](http://www.ncbi.nlm.nih.gov/entrez/query.fcgi?db=gene&cmd=Retrieve&dopt=full_report&list_uids=9118) | internexin neuronal intermediate filament protein alpha |
| [Details](http://mirdb.org/cgi-bin/target_detail.cgi?targetID=3073698) | 377 | 88 | hsa-miR-27a-3p | [NCOA5](http://www.ncbi.nlm.nih.gov/entrez/query.fcgi?db=gene&cmd=Retrieve&dopt=full_report&list_uids=57727) | nuclear receptor coactivator 5 |
| [Details](http://mirdb.org/cgi-bin/target_detail.cgi?targetID=3073803) | 378 | 88 | hsa-miR-27a-3p | [CALD1](http://www.ncbi.nlm.nih.gov/entrez/query.fcgi?db=gene&cmd=Retrieve&dopt=full_report&list_uids=800) | caldesmon 1 |
| [Details](http://mirdb.org/cgi-bin/target_detail.cgi?targetID=3073886) | 379 | 88 | hsa-miR-27a-3p | [ABHD17B](http://www.ncbi.nlm.nih.gov/entrez/query.fcgi?db=gene&cmd=Retrieve&dopt=full_report&list_uids=51104) | abhydrolase domain containing 17B |
| [Details](http://mirdb.org/cgi-bin/target_detail.cgi?targetID=3073919) | 380 | 88 | hsa-miR-27a-3p | [DEPDC4](http://www.ncbi.nlm.nih.gov/entrez/query.fcgi?db=gene&cmd=Retrieve&dopt=full_report&list_uids=120863) | DEP domain containing 4 |
| [Details](http://mirdb.org/cgi-bin/target_detail.cgi?targetID=3073923) | 381 | 88 | hsa-miR-27a-3p | [GEM](http://www.ncbi.nlm.nih.gov/entrez/query.fcgi?db=gene&cmd=Retrieve&dopt=full_report&list_uids=2669) | GTP binding protein overexpressed in skeletal muscle |
| [Details](http://mirdb.org/cgi-bin/target_detail.cgi?targetID=3072479) | 382 | 87 | hsa-miR-27a-3p | [AMPD3](http://www.ncbi.nlm.nih.gov/entrez/query.fcgi?db=gene&cmd=Retrieve&dopt=full_report&list_uids=272) | adenosine monophosphate deaminase 3 |
| [Details](http://mirdb.org/cgi-bin/target_detail.cgi?targetID=3072522) | 383 | 87 | hsa-miR-27a-3p | [POU2F3](http://www.ncbi.nlm.nih.gov/entrez/query.fcgi?db=gene&cmd=Retrieve&dopt=full_report&list_uids=25833) | POU class 2 homeobox 3 |
| [Details](http://mirdb.org/cgi-bin/target_detail.cgi?targetID=3072535) | 384 | 87 | hsa-miR-27a-3p | [TAF5L](http://www.ncbi.nlm.nih.gov/entrez/query.fcgi?db=gene&cmd=Retrieve&dopt=full_report&list_uids=27097) | TATA-box binding protein associated factor 5 like |
| [Details](http://mirdb.org/cgi-bin/target_detail.cgi?targetID=3072638) | 385 | 87 | hsa-miR-27a-3p | [SYNJ1](http://www.ncbi.nlm.nih.gov/entrez/query.fcgi?db=gene&cmd=Retrieve&dopt=full_report&list_uids=8867) | synaptojanin 1 |
| [Details](http://mirdb.org/cgi-bin/target_detail.cgi?targetID=3072740) | 386 | 87 | hsa-miR-27a-3p | [SS18L1](http://www.ncbi.nlm.nih.gov/entrez/query.fcgi?db=gene&cmd=Retrieve&dopt=full_report&list_uids=26039) | SS18L1, nBAF chromatin remodeling complex subunit |
| [Details](http://mirdb.org/cgi-bin/target_detail.cgi?targetID=3072741) | 387 | 87 | hsa-miR-27a-3p | [SLC35F4](http://www.ncbi.nlm.nih.gov/entrez/query.fcgi?db=gene&cmd=Retrieve&dopt=full_report&list_uids=341880) | solute carrier family 35 member F4 |
| [Details](http://mirdb.org/cgi-bin/target_detail.cgi?targetID=3072806) | 388 | 87 | hsa-miR-27a-3p | [SLIT2](http://www.ncbi.nlm.nih.gov/entrez/query.fcgi?db=gene&cmd=Retrieve&dopt=full_report&list_uids=9353) | slit guidance ligand 2 |
| [Details](http://mirdb.org/cgi-bin/target_detail.cgi?targetID=3072819) | 389 | 87 | hsa-miR-27a-3p | [GLTP](http://www.ncbi.nlm.nih.gov/entrez/query.fcgi?db=gene&cmd=Retrieve&dopt=full_report&list_uids=51228) | glycolipid transfer protein |
| [Details](http://mirdb.org/cgi-bin/target_detail.cgi?targetID=3072870) | 390 | 87 | hsa-miR-27a-3p | [ARRDC4](http://www.ncbi.nlm.nih.gov/entrez/query.fcgi?db=gene&cmd=Retrieve&dopt=full_report&list_uids=91947) | arrestin domain containing 4 |
| [Details](http://mirdb.org/cgi-bin/target_detail.cgi?targetID=3072884) | 391 | 87 | hsa-miR-27a-3p | [NECAB1](http://www.ncbi.nlm.nih.gov/entrez/query.fcgi?db=gene&cmd=Retrieve&dopt=full_report&list_uids=64168) | N-terminal EF-hand calcium binding protein 1 |
| [Details](http://mirdb.org/cgi-bin/target_detail.cgi?targetID=3072923) | 392 | 87 | hsa-miR-27a-3p | [UNC13C](http://www.ncbi.nlm.nih.gov/entrez/query.fcgi?db=gene&cmd=Retrieve&dopt=full_report&list_uids=440279) | unc-13 homolog C |
| [Details](http://mirdb.org/cgi-bin/target_detail.cgi?targetID=3073023) | 393 | 87 | hsa-miR-27a-3p | [MIGA2](http://www.ncbi.nlm.nih.gov/entrez/query.fcgi?db=gene&cmd=Retrieve&dopt=full_report&list_uids=84895) | mitoguardin 2 |
| [Details](http://mirdb.org/cgi-bin/target_detail.cgi?targetID=3073041) | 394 | 87 | hsa-miR-27a-3p | [CAMTA1](http://www.ncbi.nlm.nih.gov/entrez/query.fcgi?db=gene&cmd=Retrieve&dopt=full_report&list_uids=23261) | calmodulin binding transcription activator 1 |
| [Details](http://mirdb.org/cgi-bin/target_detail.cgi?targetID=3073143) | 395 | 87 | hsa-miR-27a-3p | [PAIP2](http://www.ncbi.nlm.nih.gov/entrez/query.fcgi?db=gene&cmd=Retrieve&dopt=full_report&list_uids=51247) | poly(A) binding protein interacting protein 2 |
| [Details](http://mirdb.org/cgi-bin/target_detail.cgi?targetID=3073191) | 396 | 87 | hsa-miR-27a-3p | [APOOL](http://www.ncbi.nlm.nih.gov/entrez/query.fcgi?db=gene&cmd=Retrieve&dopt=full_report&list_uids=139322) | apolipoprotein O like |
| [Details](http://mirdb.org/cgi-bin/target_detail.cgi?targetID=3073219) | 397 | 87 | hsa-miR-27a-3p | [SYNRG](http://www.ncbi.nlm.nih.gov/entrez/query.fcgi?db=gene&cmd=Retrieve&dopt=full_report&list_uids=11276) | synergin gamma |
| [Details](http://mirdb.org/cgi-bin/target_detail.cgi?targetID=3073275) | 398 | 87 | hsa-miR-27a-3p | [PF4](http://www.ncbi.nlm.nih.gov/entrez/query.fcgi?db=gene&cmd=Retrieve&dopt=full_report&list_uids=5196) | platelet factor 4 |
| [Details](http://mirdb.org/cgi-bin/target_detail.cgi?targetID=3073284) | 399 | 87 | hsa-miR-27a-3p | [ATP1A2](http://www.ncbi.nlm.nih.gov/entrez/query.fcgi?db=gene&cmd=Retrieve&dopt=full_report&list_uids=477) | ATPase Na+/K+ transporting subunit alpha 2 |
| [Details](http://mirdb.org/cgi-bin/target_detail.cgi?targetID=3073341) | 400 | 87 | hsa-miR-27a-3p | [CCNC](http://www.ncbi.nlm.nih.gov/entrez/query.fcgi?db=gene&cmd=Retrieve&dopt=full_report&list_uids=892) | cyclin C |
| [Details](http://mirdb.org/cgi-bin/target_detail.cgi?targetID=3073354) | 401 | 87 | hsa-miR-27a-3p | [TAPT1](http://www.ncbi.nlm.nih.gov/entrez/query.fcgi?db=gene&cmd=Retrieve&dopt=full_report&list_uids=202018) | transmembrane anterior posterior transformation 1 |
| [Details](http://mirdb.org/cgi-bin/target_detail.cgi?targetID=3073392) | 402 | 87 | hsa-miR-27a-3p | [KCTD14](http://www.ncbi.nlm.nih.gov/entrez/query.fcgi?db=gene&cmd=Retrieve&dopt=full_report&list_uids=65987) | potassium channel tetramerization domain containing 14 |
| [Details](http://mirdb.org/cgi-bin/target_detail.cgi?targetID=3073437) | 403 | 87 | hsa-miR-27a-3p | [WIPF2](http://www.ncbi.nlm.nih.gov/entrez/query.fcgi?db=gene&cmd=Retrieve&dopt=full_report&list_uids=147179) | WAS/WASL interacting protein family member 2 |
| [Details](http://mirdb.org/cgi-bin/target_detail.cgi?targetID=3073453) | 404 | 87 | hsa-miR-27a-3p | [SPATA13](http://www.ncbi.nlm.nih.gov/entrez/query.fcgi?db=gene&cmd=Retrieve&dopt=full_report&list_uids=221178) | spermatogenesis associated 13 |
| [Details](http://mirdb.org/cgi-bin/target_detail.cgi?targetID=3073484) | 405 | 87 | hsa-miR-27a-3p | [LIPT2](http://www.ncbi.nlm.nih.gov/entrez/query.fcgi?db=gene&cmd=Retrieve&dopt=full_report&list_uids=387787) | lipoyl(octanoyl) transferase 2 |
| [Details](http://mirdb.org/cgi-bin/target_detail.cgi?targetID=3073527) | 406 | 87 | hsa-miR-27a-3p | [CCNY](http://www.ncbi.nlm.nih.gov/entrez/query.fcgi?db=gene&cmd=Retrieve&dopt=full_report&list_uids=219771) | cyclin Y |
| [Details](http://mirdb.org/cgi-bin/target_detail.cgi?targetID=3073542) | 407 | 87 | hsa-miR-27a-3p | [FN1](http://www.ncbi.nlm.nih.gov/entrez/query.fcgi?db=gene&cmd=Retrieve&dopt=full_report&list_uids=2335) | fibronectin 1 |
| [Details](http://mirdb.org/cgi-bin/target_detail.cgi?targetID=3073561) | 408 | 87 | hsa-miR-27a-3p | [RPN2](http://www.ncbi.nlm.nih.gov/entrez/query.fcgi?db=gene&cmd=Retrieve&dopt=full_report&list_uids=6185) | ribophorin II |
| [Details](http://mirdb.org/cgi-bin/target_detail.cgi?targetID=3073644) | 409 | 87 | hsa-miR-27a-3p | [RC3H1](http://www.ncbi.nlm.nih.gov/entrez/query.fcgi?db=gene&cmd=Retrieve&dopt=full_report&list_uids=149041) | ring finger and CCCH-type domains 1 |
| [Details](http://mirdb.org/cgi-bin/target_detail.cgi?targetID=3073762) | 410 | 87 | hsa-miR-27a-3p | [LIMK1](http://www.ncbi.nlm.nih.gov/entrez/query.fcgi?db=gene&cmd=Retrieve&dopt=full_report&list_uids=3984) | LIM domain kinase 1 |
| [Details](http://mirdb.org/cgi-bin/target_detail.cgi?targetID=3073810) | 411 | 87 | hsa-miR-27a-3p | [SLCO5A1](http://www.ncbi.nlm.nih.gov/entrez/query.fcgi?db=gene&cmd=Retrieve&dopt=full_report&list_uids=81796) | solute carrier organic anion transporter family member 5A1 |
| [Details](http://mirdb.org/cgi-bin/target_detail.cgi?targetID=3073829) | 412 | 87 | hsa-miR-27a-3p | [PCLO](http://www.ncbi.nlm.nih.gov/entrez/query.fcgi?db=gene&cmd=Retrieve&dopt=full_report&list_uids=27445) | piccolo presynaptic cytomatrix protein |
| [Details](http://mirdb.org/cgi-bin/target_detail.cgi?targetID=3073896) | 413 | 87 | hsa-miR-27a-3p | [TMEM170A](http://www.ncbi.nlm.nih.gov/entrez/query.fcgi?db=gene&cmd=Retrieve&dopt=full_report&list_uids=124491) | transmembrane protein 170A |
| [Details](http://mirdb.org/cgi-bin/target_detail.cgi?targetID=3072483) | 414 | 86 | hsa-miR-27a-3p | [RYBP](http://www.ncbi.nlm.nih.gov/entrez/query.fcgi?db=gene&cmd=Retrieve&dopt=full_report&list_uids=23429) | RING1 and YY1 binding protein |
| [Details](http://mirdb.org/cgi-bin/target_detail.cgi?targetID=3072524) | 415 | 86 | hsa-miR-27a-3p | [FBLN5](http://www.ncbi.nlm.nih.gov/entrez/query.fcgi?db=gene&cmd=Retrieve&dopt=full_report&list_uids=10516) | fibulin 5 |
| [Details](http://mirdb.org/cgi-bin/target_detail.cgi?targetID=3072526) | 416 | 86 | hsa-miR-27a-3p | [SRL](http://www.ncbi.nlm.nih.gov/entrez/query.fcgi?db=gene&cmd=Retrieve&dopt=full_report&list_uids=6345) | sarcalumenin |
| [Details](http://mirdb.org/cgi-bin/target_detail.cgi?targetID=3072579) | 417 | 86 | hsa-miR-27a-3p | [VAV3](http://www.ncbi.nlm.nih.gov/entrez/query.fcgi?db=gene&cmd=Retrieve&dopt=full_report&list_uids=10451) | vav guanine nucleotide exchange factor 3 |
| [Details](http://mirdb.org/cgi-bin/target_detail.cgi?targetID=3072609) | 418 | 86 | hsa-miR-27a-3p | [AFAP1](http://www.ncbi.nlm.nih.gov/entrez/query.fcgi?db=gene&cmd=Retrieve&dopt=full_report&list_uids=60312) | actin filament associated protein 1 |
| [Details](http://mirdb.org/cgi-bin/target_detail.cgi?targetID=3072621) | 419 | 86 | hsa-miR-27a-3p | [KPNA3](http://www.ncbi.nlm.nih.gov/entrez/query.fcgi?db=gene&cmd=Retrieve&dopt=full_report&list_uids=3839) | karyopherin subunit alpha 3 |
| [Details](http://mirdb.org/cgi-bin/target_detail.cgi?targetID=3072625) | 420 | 86 | hsa-miR-27a-3p | [ARHGEF7](http://www.ncbi.nlm.nih.gov/entrez/query.fcgi?db=gene&cmd=Retrieve&dopt=full_report&list_uids=8874) | Rho guanine nucleotide exchange factor 7 |
| [Details](http://mirdb.org/cgi-bin/target_detail.cgi?targetID=3072632) | 421 | 86 | hsa-miR-27a-3p | [WDCP](http://www.ncbi.nlm.nih.gov/entrez/query.fcgi?db=gene&cmd=Retrieve&dopt=full_report&list_uids=80304) | WD repeat and coiled coil containing |
| [Details](http://mirdb.org/cgi-bin/target_detail.cgi?targetID=3072695) | 422 | 86 | hsa-miR-27a-3p | [AGRN](http://www.ncbi.nlm.nih.gov/entrez/query.fcgi?db=gene&cmd=Retrieve&dopt=full_report&list_uids=375790) | agrin |
| [Details](http://mirdb.org/cgi-bin/target_detail.cgi?targetID=3072747) | 423 | 86 | hsa-miR-27a-3p | [C11orf58](http://www.ncbi.nlm.nih.gov/entrez/query.fcgi?db=gene&cmd=Retrieve&dopt=full_report&list_uids=10944) | chromosome 11 open reading frame 58 |
| [Details](http://mirdb.org/cgi-bin/target_detail.cgi?targetID=3072751) | 424 | 86 | hsa-miR-27a-3p | [BEND4](http://www.ncbi.nlm.nih.gov/entrez/query.fcgi?db=gene&cmd=Retrieve&dopt=full_report&list_uids=389206) | BEN domain containing 4 |
| [Details](http://mirdb.org/cgi-bin/target_detail.cgi?targetID=3072833) | 425 | 86 | hsa-miR-27a-3p | [SOWAHA](http://www.ncbi.nlm.nih.gov/entrez/query.fcgi?db=gene&cmd=Retrieve&dopt=full_report&list_uids=134548) | sosondowah ankyrin repeat domain family member A |
| [Details](http://mirdb.org/cgi-bin/target_detail.cgi?targetID=3072844) | 426 | 86 | hsa-miR-27a-3p | [ZNF708](http://www.ncbi.nlm.nih.gov/entrez/query.fcgi?db=gene&cmd=Retrieve&dopt=full_report&list_uids=7562) | zinc finger protein 708 |
| [Details](http://mirdb.org/cgi-bin/target_detail.cgi?targetID=3072850) | 427 | 86 | hsa-miR-27a-3p | [AKIRIN2](http://www.ncbi.nlm.nih.gov/entrez/query.fcgi?db=gene&cmd=Retrieve&dopt=full_report&list_uids=55122) | akirin 2 |
| [Details](http://mirdb.org/cgi-bin/target_detail.cgi?targetID=3072909) | 428 | 86 | hsa-miR-27a-3p | [SLC24A4](http://www.ncbi.nlm.nih.gov/entrez/query.fcgi?db=gene&cmd=Retrieve&dopt=full_report&list_uids=123041) | solute carrier family 24 member 4 |
| [Details](http://mirdb.org/cgi-bin/target_detail.cgi?targetID=3072961) | 429 | 86 | hsa-miR-27a-3p | [SLC13A3](http://www.ncbi.nlm.nih.gov/entrez/query.fcgi?db=gene&cmd=Retrieve&dopt=full_report&list_uids=64849) | solute carrier family 13 member 3 |
| [Details](http://mirdb.org/cgi-bin/target_detail.cgi?targetID=3072969) | 430 | 86 | hsa-miR-27a-3p | [ST14](http://www.ncbi.nlm.nih.gov/entrez/query.fcgi?db=gene&cmd=Retrieve&dopt=full_report&list_uids=6768) | suppression of tumorigenicity 14 |
| [Details](http://mirdb.org/cgi-bin/target_detail.cgi?targetID=3073006) | 431 | 86 | hsa-miR-27a-3p | [INSR](http://www.ncbi.nlm.nih.gov/entrez/query.fcgi?db=gene&cmd=Retrieve&dopt=full_report&list_uids=3643) | insulin receptor |
| [Details](http://mirdb.org/cgi-bin/target_detail.cgi?targetID=3073016) | 432 | 86 | hsa-miR-27a-3p | [FOXP4](http://www.ncbi.nlm.nih.gov/entrez/query.fcgi?db=gene&cmd=Retrieve&dopt=full_report&list_uids=116113) | forkhead box P4 |
| [Details](http://mirdb.org/cgi-bin/target_detail.cgi?targetID=3073050) | 433 | 86 | hsa-miR-27a-3p | [PDK4](http://www.ncbi.nlm.nih.gov/entrez/query.fcgi?db=gene&cmd=Retrieve&dopt=full_report&list_uids=5166) | pyruvate dehydrogenase kinase 4 |
| [Details](http://mirdb.org/cgi-bin/target_detail.cgi?targetID=3073068) | 434 | 86 | hsa-miR-27a-3p | [BTG1](http://www.ncbi.nlm.nih.gov/entrez/query.fcgi?db=gene&cmd=Retrieve&dopt=full_report&list_uids=694) | BTG anti-proliferation factor 1 |
| [Details](http://mirdb.org/cgi-bin/target_detail.cgi?targetID=3073084) | 435 | 86 | hsa-miR-27a-3p | [BMP3](http://www.ncbi.nlm.nih.gov/entrez/query.fcgi?db=gene&cmd=Retrieve&dopt=full_report&list_uids=651) | bone morphogenetic protein 3 |
| [Details](http://mirdb.org/cgi-bin/target_detail.cgi?targetID=3073170) | 436 | 86 | hsa-miR-27a-3p | [NCOA7](http://www.ncbi.nlm.nih.gov/entrez/query.fcgi?db=gene&cmd=Retrieve&dopt=full_report&list_uids=135112) | nuclear receptor coactivator 7 |
| [Details](http://mirdb.org/cgi-bin/target_detail.cgi?targetID=3073199) | 437 | 86 | hsa-miR-27a-3p | [SEC62](http://www.ncbi.nlm.nih.gov/entrez/query.fcgi?db=gene&cmd=Retrieve&dopt=full_report&list_uids=7095) | SEC62 homolog, preprotein translocation factor |
| [Details](http://mirdb.org/cgi-bin/target_detail.cgi?targetID=3073231) | 438 | 86 | hsa-miR-27a-3p | [FCRL2](http://www.ncbi.nlm.nih.gov/entrez/query.fcgi?db=gene&cmd=Retrieve&dopt=full_report&list_uids=79368) | Fc receptor like 2 |
| [Details](http://mirdb.org/cgi-bin/target_detail.cgi?targetID=3073298) | 439 | 86 | hsa-miR-27a-3p | [PNISR](http://www.ncbi.nlm.nih.gov/entrez/query.fcgi?db=gene&cmd=Retrieve&dopt=full_report&list_uids=25957) | PNN interacting serine and arginine rich protein |
| [Details](http://mirdb.org/cgi-bin/target_detail.cgi?targetID=3073304) | 440 | 86 | hsa-miR-27a-3p | [FOSB](http://www.ncbi.nlm.nih.gov/entrez/query.fcgi?db=gene&cmd=Retrieve&dopt=full_report&list_uids=2354) | FosB proto-oncogene, AP-1 transcription factor subunit |
| [Details](http://mirdb.org/cgi-bin/target_detail.cgi?targetID=3073318) | 441 | 86 | hsa-miR-27a-3p | [FAM78A](http://www.ncbi.nlm.nih.gov/entrez/query.fcgi?db=gene&cmd=Retrieve&dopt=full_report&list_uids=286336) | family with sequence similarity 78 member A |
| [Details](http://mirdb.org/cgi-bin/target_detail.cgi?targetID=3073417) | 442 | 86 | hsa-miR-27a-3p | [ANKS1A](http://www.ncbi.nlm.nih.gov/entrez/query.fcgi?db=gene&cmd=Retrieve&dopt=full_report&list_uids=23294) | ankyrin repeat and sterile alpha motif domain containing 1A |
| [Details](http://mirdb.org/cgi-bin/target_detail.cgi?targetID=3073419) | 443 | 86 | hsa-miR-27a-3p | [WDR37](http://www.ncbi.nlm.nih.gov/entrez/query.fcgi?db=gene&cmd=Retrieve&dopt=full_report&list_uids=22884) | WD repeat domain 37 |
| [Details](http://mirdb.org/cgi-bin/target_detail.cgi?targetID=3073450) | 444 | 86 | hsa-miR-27a-3p | [GATA2](http://www.ncbi.nlm.nih.gov/entrez/query.fcgi?db=gene&cmd=Retrieve&dopt=full_report&list_uids=2624) | GATA binding protein 2 |
| [Details](http://mirdb.org/cgi-bin/target_detail.cgi?targetID=3073465) | 445 | 86 | hsa-miR-27a-3p | [PTCH1](http://www.ncbi.nlm.nih.gov/entrez/query.fcgi?db=gene&cmd=Retrieve&dopt=full_report&list_uids=5727) | patched 1 |
| [Details](http://mirdb.org/cgi-bin/target_detail.cgi?targetID=3073471) | 446 | 86 | hsa-miR-27a-3p | [GATAD2A](http://www.ncbi.nlm.nih.gov/entrez/query.fcgi?db=gene&cmd=Retrieve&dopt=full_report&list_uids=54815) | GATA zinc finger domain containing 2A |
| [Details](http://mirdb.org/cgi-bin/target_detail.cgi?targetID=3073483) | 447 | 86 | hsa-miR-27a-3p | [ZFP36](http://www.ncbi.nlm.nih.gov/entrez/query.fcgi?db=gene&cmd=Retrieve&dopt=full_report&list_uids=7538) | ZFP36 ring finger protein |
| [Details](http://mirdb.org/cgi-bin/target_detail.cgi?targetID=3073487) | 448 | 86 | hsa-miR-27a-3p | [SEH1L](http://www.ncbi.nlm.nih.gov/entrez/query.fcgi?db=gene&cmd=Retrieve&dopt=full_report&list_uids=81929) | SEH1 like nucleoporin |
| [Details](http://mirdb.org/cgi-bin/target_detail.cgi?targetID=3073498) | 449 | 86 | hsa-miR-27a-3p | [HSPH1](http://www.ncbi.nlm.nih.gov/entrez/query.fcgi?db=gene&cmd=Retrieve&dopt=full_report&list_uids=10808) | heat shock protein family H (Hsp110) member 1 |
| [Details](http://mirdb.org/cgi-bin/target_detail.cgi?targetID=3073501) | 450 | 86 | hsa-miR-27a-3p | [SLC9A4](http://www.ncbi.nlm.nih.gov/entrez/query.fcgi?db=gene&cmd=Retrieve&dopt=full_report&list_uids=389015) | solute carrier family 9 member A4 |
| [Details](http://mirdb.org/cgi-bin/target_detail.cgi?targetID=3073520) | 451 | 86 | hsa-miR-27a-3p | [CCNT2](http://www.ncbi.nlm.nih.gov/entrez/query.fcgi?db=gene&cmd=Retrieve&dopt=full_report&list_uids=905) | cyclin T2 |
| [Details](http://mirdb.org/cgi-bin/target_detail.cgi?targetID=3073555) | 452 | 86 | hsa-miR-27a-3p | [KCNJ3](http://www.ncbi.nlm.nih.gov/entrez/query.fcgi?db=gene&cmd=Retrieve&dopt=full_report&list_uids=3760) | potassium voltage-gated channel subfamily J member 3 |
| [Details](http://mirdb.org/cgi-bin/target_detail.cgi?targetID=3073574) | 453 | 86 | hsa-miR-27a-3p | [PRLR](http://www.ncbi.nlm.nih.gov/entrez/query.fcgi?db=gene&cmd=Retrieve&dopt=full_report&list_uids=5618) | prolactin receptor |
| [Details](http://mirdb.org/cgi-bin/target_detail.cgi?targetID=3073595) | 454 | 86 | hsa-miR-27a-3p | [PAIP2B](http://www.ncbi.nlm.nih.gov/entrez/query.fcgi?db=gene&cmd=Retrieve&dopt=full_report&list_uids=400961) | poly(A) binding protein interacting protein 2B |
| [Details](http://mirdb.org/cgi-bin/target_detail.cgi?targetID=3073635) | 455 | 86 | hsa-miR-27a-3p | [THRB](http://www.ncbi.nlm.nih.gov/entrez/query.fcgi?db=gene&cmd=Retrieve&dopt=full_report&list_uids=7068) | thyroid hormone receptor beta |
| [Details](http://mirdb.org/cgi-bin/target_detail.cgi?targetID=3073645) | 456 | 86 | hsa-miR-27a-3p | [TRIL](http://www.ncbi.nlm.nih.gov/entrez/query.fcgi?db=gene&cmd=Retrieve&dopt=full_report&list_uids=9865) | TLR4 interactor with leucine rich repeats |
| [Details](http://mirdb.org/cgi-bin/target_detail.cgi?targetID=3073660) | 457 | 86 | hsa-miR-27a-3p | [MRPS14](http://www.ncbi.nlm.nih.gov/entrez/query.fcgi?db=gene&cmd=Retrieve&dopt=full_report&list_uids=63931) | mitochondrial ribosomal protein S14 |
| [Details](http://mirdb.org/cgi-bin/target_detail.cgi?targetID=3073703) | 458 | 86 | hsa-miR-27a-3p | [SATB2](http://www.ncbi.nlm.nih.gov/entrez/query.fcgi?db=gene&cmd=Retrieve&dopt=full_report&list_uids=23314) | SATB homeobox 2 |
| [Details](http://mirdb.org/cgi-bin/target_detail.cgi?targetID=3073729) | 459 | 86 | hsa-miR-27a-3p | [FCRL1](http://www.ncbi.nlm.nih.gov/entrez/query.fcgi?db=gene&cmd=Retrieve&dopt=full_report&list_uids=115350) | Fc receptor like 1 |
| [Details](http://mirdb.org/cgi-bin/target_detail.cgi?targetID=3073761) | 460 | 86 | hsa-miR-27a-3p | [HSDL1](http://www.ncbi.nlm.nih.gov/entrez/query.fcgi?db=gene&cmd=Retrieve&dopt=full_report&list_uids=83693) | hydroxysteroid dehydrogenase like 1 |
| [Details](http://mirdb.org/cgi-bin/target_detail.cgi?targetID=3073826) | 461 | 86 | hsa-miR-27a-3p | [CCN4](http://www.ncbi.nlm.nih.gov/entrez/query.fcgi?db=gene&cmd=Retrieve&dopt=full_report&list_uids=8840) | cellular communication network factor 4 |
| [Details](http://mirdb.org/cgi-bin/target_detail.cgi?targetID=3072460) | 462 | 85 | hsa-miR-27a-3p | [MAGI3](http://www.ncbi.nlm.nih.gov/entrez/query.fcgi?db=gene&cmd=Retrieve&dopt=full_report&list_uids=260425) | membrane associated guanylate kinase, WW and PDZ domain containing 3 |
| [Details](http://mirdb.org/cgi-bin/target_detail.cgi?targetID=3072461) | 463 | 85 | hsa-miR-27a-3p | [XPO1](http://www.ncbi.nlm.nih.gov/entrez/query.fcgi?db=gene&cmd=Retrieve&dopt=full_report&list_uids=7514) | exportin 1 |
| [Details](http://mirdb.org/cgi-bin/target_detail.cgi?targetID=3072598) | 464 | 85 | hsa-miR-27a-3p | [GPT2](http://www.ncbi.nlm.nih.gov/entrez/query.fcgi?db=gene&cmd=Retrieve&dopt=full_report&list_uids=84706) | glutamic--pyruvic transaminase 2 |
| [Details](http://mirdb.org/cgi-bin/target_detail.cgi?targetID=3072704) | 465 | 85 | hsa-miR-27a-3p | [COL19A1](http://www.ncbi.nlm.nih.gov/entrez/query.fcgi?db=gene&cmd=Retrieve&dopt=full_report&list_uids=1310) | collagen type XIX alpha 1 chain |
| [Details](http://mirdb.org/cgi-bin/target_detail.cgi?targetID=3072707) | 466 | 85 | hsa-miR-27a-3p | [TET1](http://www.ncbi.nlm.nih.gov/entrez/query.fcgi?db=gene&cmd=Retrieve&dopt=full_report&list_uids=80312) | tet methylcytosine dioxygenase 1 |
| [Details](http://mirdb.org/cgi-bin/target_detail.cgi?targetID=3072723) | 467 | 85 | hsa-miR-27a-3p | [CSF1](http://www.ncbi.nlm.nih.gov/entrez/query.fcgi?db=gene&cmd=Retrieve&dopt=full_report&list_uids=1435) | colony stimulating factor 1 |
| [Details](http://mirdb.org/cgi-bin/target_detail.cgi?targetID=3072779) | 468 | 85 | hsa-miR-27a-3p | [CHD7](http://www.ncbi.nlm.nih.gov/entrez/query.fcgi?db=gene&cmd=Retrieve&dopt=full_report&list_uids=55636) | chromodomain helicase DNA binding protein 7 |
| [Details](http://mirdb.org/cgi-bin/target_detail.cgi?targetID=3072788) | 469 | 85 | hsa-miR-27a-3p | [MDN1](http://www.ncbi.nlm.nih.gov/entrez/query.fcgi?db=gene&cmd=Retrieve&dopt=full_report&list_uids=23195) | midasin AAA ATPase 1 |
| [Details](http://mirdb.org/cgi-bin/target_detail.cgi?targetID=3072824) | 470 | 85 | hsa-miR-27a-3p | [TSC1](http://www.ncbi.nlm.nih.gov/entrez/query.fcgi?db=gene&cmd=Retrieve&dopt=full_report&list_uids=7248) | TSC complex subunit 1 |
| [Details](http://mirdb.org/cgi-bin/target_detail.cgi?targetID=3072826) | 471 | 85 | hsa-miR-27a-3p | [PLEKHA5](http://www.ncbi.nlm.nih.gov/entrez/query.fcgi?db=gene&cmd=Retrieve&dopt=full_report&list_uids=54477) | pleckstrin homology domain containing A5 |
| [Details](http://mirdb.org/cgi-bin/target_detail.cgi?targetID=3072873) | 472 | 85 | hsa-miR-27a-3p | [STK39](http://www.ncbi.nlm.nih.gov/entrez/query.fcgi?db=gene&cmd=Retrieve&dopt=full_report&list_uids=27347) | serine/threonine kinase 39 |
| [Details](http://mirdb.org/cgi-bin/target_detail.cgi?targetID=3072939) | 473 | 85 | hsa-miR-27a-3p | [ITGA5](http://www.ncbi.nlm.nih.gov/entrez/query.fcgi?db=gene&cmd=Retrieve&dopt=full_report&list_uids=3678) | integrin subunit alpha 5 |
| [Details](http://mirdb.org/cgi-bin/target_detail.cgi?targetID=3072967) | 474 | 85 | hsa-miR-27a-3p | [SLFN5](http://www.ncbi.nlm.nih.gov/entrez/query.fcgi?db=gene&cmd=Retrieve&dopt=full_report&list_uids=162394) | schlafen family member 5 |
| [Details](http://mirdb.org/cgi-bin/target_detail.cgi?targetID=3073075) | 475 | 85 | hsa-miR-27a-3p | [CCDC28B](http://www.ncbi.nlm.nih.gov/entrez/query.fcgi?db=gene&cmd=Retrieve&dopt=full_report&list_uids=79140) | coiled-coil domain containing 28B |
| [Details](http://mirdb.org/cgi-bin/target_detail.cgi?targetID=3073200) | 476 | 85 | hsa-miR-27a-3p | [CLSTN2](http://www.ncbi.nlm.nih.gov/entrez/query.fcgi?db=gene&cmd=Retrieve&dopt=full_report&list_uids=64084) | calsyntenin 2 |
| [Details](http://mirdb.org/cgi-bin/target_detail.cgi?targetID=3073201) | 477 | 85 | hsa-miR-27a-3p | [SRP19](http://www.ncbi.nlm.nih.gov/entrez/query.fcgi?db=gene&cmd=Retrieve&dopt=full_report&list_uids=6728) | signal recognition particle 19 |
| [Details](http://mirdb.org/cgi-bin/target_detail.cgi?targetID=3073273) | 478 | 85 | hsa-miR-27a-3p | [FOXO1](http://www.ncbi.nlm.nih.gov/entrez/query.fcgi?db=gene&cmd=Retrieve&dopt=full_report&list_uids=2308) | forkhead box O1 |
| [Details](http://mirdb.org/cgi-bin/target_detail.cgi?targetID=3073277) | 479 | 85 | hsa-miR-27a-3p | [USP31](http://www.ncbi.nlm.nih.gov/entrez/query.fcgi?db=gene&cmd=Retrieve&dopt=full_report&list_uids=57478) | ubiquitin specific peptidase 31 |
| [Details](http://mirdb.org/cgi-bin/target_detail.cgi?targetID=3073319) | 480 | 85 | hsa-miR-27a-3p | [CYP39A1](http://www.ncbi.nlm.nih.gov/entrez/query.fcgi?db=gene&cmd=Retrieve&dopt=full_report&list_uids=51302) | cytochrome P450 family 39 subfamily A member 1 |
| [Details](http://mirdb.org/cgi-bin/target_detail.cgi?targetID=3073428) | 481 | 85 | hsa-miR-27a-3p | [HEG1](http://www.ncbi.nlm.nih.gov/entrez/query.fcgi?db=gene&cmd=Retrieve&dopt=full_report&list_uids=57493) | heart development protein with EGF like domains 1 |
| [Details](http://mirdb.org/cgi-bin/target_detail.cgi?targetID=3073440) | 482 | 85 | hsa-miR-27a-3p | [RAB11FIP1](http://www.ncbi.nlm.nih.gov/entrez/query.fcgi?db=gene&cmd=Retrieve&dopt=full_report&list_uids=80223) | RAB11 family interacting protein 1 |
| [Details](http://mirdb.org/cgi-bin/target_detail.cgi?targetID=3073478) | 483 | 85 | hsa-miR-27a-3p | [LYSMD3](http://www.ncbi.nlm.nih.gov/entrez/query.fcgi?db=gene&cmd=Retrieve&dopt=full_report&list_uids=116068) | LysM domain containing 3 |
| [Details](http://mirdb.org/cgi-bin/target_detail.cgi?targetID=3073513) | 484 | 85 | hsa-miR-27a-3p | [SNN](http://www.ncbi.nlm.nih.gov/entrez/query.fcgi?db=gene&cmd=Retrieve&dopt=full_report&list_uids=8303) | stannin |
| [Details](http://mirdb.org/cgi-bin/target_detail.cgi?targetID=3073529) | 485 | 85 | hsa-miR-27a-3p | [KHSRP](http://www.ncbi.nlm.nih.gov/entrez/query.fcgi?db=gene&cmd=Retrieve&dopt=full_report&list_uids=8570) | KH-type splicing regulatory protein |
| [Details](http://mirdb.org/cgi-bin/target_detail.cgi?targetID=3073537) | 486 | 85 | hsa-miR-27a-3p | [SLC25A16](http://www.ncbi.nlm.nih.gov/entrez/query.fcgi?db=gene&cmd=Retrieve&dopt=full_report&list_uids=8034) | solute carrier family 25 member 16 |
| [Details](http://mirdb.org/cgi-bin/target_detail.cgi?targetID=3073612) | 487 | 85 | hsa-miR-27a-3p | [COL21A1](http://www.ncbi.nlm.nih.gov/entrez/query.fcgi?db=gene&cmd=Retrieve&dopt=full_report&list_uids=81578) | collagen type XXI alpha 1 chain |
| [Details](http://mirdb.org/cgi-bin/target_detail.cgi?targetID=3073672) | 488 | 85 | hsa-miR-27a-3p | [CTU1](http://www.ncbi.nlm.nih.gov/entrez/query.fcgi?db=gene&cmd=Retrieve&dopt=full_report&list_uids=90353) | cytosolic thiouridylase subunit 1 |
| [Details](http://mirdb.org/cgi-bin/target_detail.cgi?targetID=3073736) | 489 | 85 | hsa-miR-27a-3p | [RIPOR2](http://www.ncbi.nlm.nih.gov/entrez/query.fcgi?db=gene&cmd=Retrieve&dopt=full_report&list_uids=9750) | RHO family interacting cell polarization regulator 2 |
| [Details](http://mirdb.org/cgi-bin/target_detail.cgi?targetID=3073745) | 490 | 85 | hsa-miR-27a-3p | [KMT5A](http://www.ncbi.nlm.nih.gov/entrez/query.fcgi?db=gene&cmd=Retrieve&dopt=full_report&list_uids=387893) | lysine methyltransferase 5A |
| [Details](http://mirdb.org/cgi-bin/target_detail.cgi?targetID=3073811) | 491 | 85 | hsa-miR-27a-3p | [TRAF3IP3](http://www.ncbi.nlm.nih.gov/entrez/query.fcgi?db=gene&cmd=Retrieve&dopt=full_report&list_uids=80342) | TRAF3 interacting protein 3 |
| [Details](http://mirdb.org/cgi-bin/target_detail.cgi?targetID=3073819) | 492 | 85 | hsa-miR-27a-3p | [MSL1](http://www.ncbi.nlm.nih.gov/entrez/query.fcgi?db=gene&cmd=Retrieve&dopt=full_report&list_uids=339287) | MSL complex subunit 1 |
| [Details](http://mirdb.org/cgi-bin/target_detail.cgi?targetID=3073856) | 493 | 85 | hsa-miR-27a-3p | [NDUFC2-KCTD14](http://www.ncbi.nlm.nih.gov/entrez/query.fcgi?db=gene&cmd=Retrieve&dopt=full_report&list_uids=100532726) | NDUFC2-KCTD14 readthrough |
| [Details](http://mirdb.org/cgi-bin/target_detail.cgi?targetID=3073875) | 494 | 85 | hsa-miR-27a-3p | [FOXO3](http://www.ncbi.nlm.nih.gov/entrez/query.fcgi?db=gene&cmd=Retrieve&dopt=full_report&list_uids=2309) | forkhead box O3 |
| [Details](http://mirdb.org/cgi-bin/target_detail.cgi?targetID=3073879) | 495 | 85 | hsa-miR-27a-3p | [SPRY2](http://www.ncbi.nlm.nih.gov/entrez/query.fcgi?db=gene&cmd=Retrieve&dopt=full_report&list_uids=10253) | sprouty RTK signaling antagonist 2 |
| [Details](http://mirdb.org/cgi-bin/target_detail.cgi?targetID=3072465) | 496 | 84 | hsa-miR-27a-3p | [PLAG1](http://www.ncbi.nlm.nih.gov/entrez/query.fcgi?db=gene&cmd=Retrieve&dopt=full_report&list_uids=5324) | PLAG1 zinc finger |
| [Details](http://mirdb.org/cgi-bin/target_detail.cgi?targetID=3072480) | 497 | 84 | hsa-miR-27a-3p | [NUP210](http://www.ncbi.nlm.nih.gov/entrez/query.fcgi?db=gene&cmd=Retrieve&dopt=full_report&list_uids=23225) | nucleoporin 210 |
| [Details](http://mirdb.org/cgi-bin/target_detail.cgi?targetID=3072496) | 498 | 84 | hsa-miR-27a-3p | [ADCY6](http://www.ncbi.nlm.nih.gov/entrez/query.fcgi?db=gene&cmd=Retrieve&dopt=full_report&list_uids=112) | adenylate cyclase 6 |
| [Details](http://mirdb.org/cgi-bin/target_detail.cgi?targetID=3072531) | 499 | 84 | hsa-miR-27a-3p | [CAMK1D](http://www.ncbi.nlm.nih.gov/entrez/query.fcgi?db=gene&cmd=Retrieve&dopt=full_report&list_uids=57118) | calcium/calmodulin dependent protein kinase ID |
| [Details](http://mirdb.org/cgi-bin/target_detail.cgi?targetID=3072689) | 500 | 84 | hsa-miR-27a-3p | [RAPGEF2](http://www.ncbi.nlm.nih.gov/entrez/query.fcgi?db=gene&cmd=Retrieve&dopt=full_report&list_uids=9693) | Rap guanine nucleotide exchange factor 2 |
| [Details](http://mirdb.org/cgi-bin/target_detail.cgi?targetID=3072712) | 501 | 84 | hsa-miR-27a-3p | [PHB](http://www.ncbi.nlm.nih.gov/entrez/query.fcgi?db=gene&cmd=Retrieve&dopt=full_report&list_uids=5245) | prohibitin |
| [Details](http://mirdb.org/cgi-bin/target_detail.cgi?targetID=3072719) | 502 | 84 | hsa-miR-27a-3p | [KIAA0319L](http://www.ncbi.nlm.nih.gov/entrez/query.fcgi?db=gene&cmd=Retrieve&dopt=full_report&list_uids=79932) | KIAA0319 like |
| [Details](http://mirdb.org/cgi-bin/target_detail.cgi?targetID=3072792) | 503 | 84 | hsa-miR-27a-3p | [NEDD4](http://www.ncbi.nlm.nih.gov/entrez/query.fcgi?db=gene&cmd=Retrieve&dopt=full_report&list_uids=4734) | neural precursor cell expressed, developmentally down-regulated 4, E3 ubiquitin protein ligase |
| [Details](http://mirdb.org/cgi-bin/target_detail.cgi?targetID=3072861) | 504 | 84 | hsa-miR-27a-3p | [SEMA6D](http://www.ncbi.nlm.nih.gov/entrez/query.fcgi?db=gene&cmd=Retrieve&dopt=full_report&list_uids=80031) | semaphorin 6D |
| [Details](http://mirdb.org/cgi-bin/target_detail.cgi?targetID=3072900) | 505 | 84 | hsa-miR-27a-3p | [ELAVL2](http://www.ncbi.nlm.nih.gov/entrez/query.fcgi?db=gene&cmd=Retrieve&dopt=full_report&list_uids=1993) | ELAV like RNA binding protein 2 |
| [Details](http://mirdb.org/cgi-bin/target_detail.cgi?targetID=3073004) | 506 | 84 | hsa-miR-27a-3p | [STMN2](http://www.ncbi.nlm.nih.gov/entrez/query.fcgi?db=gene&cmd=Retrieve&dopt=full_report&list_uids=11075) | stathmin 2 |
| [Details](http://mirdb.org/cgi-bin/target_detail.cgi?targetID=3073057) | 507 | 84 | hsa-miR-27a-3p | [RGL2](http://www.ncbi.nlm.nih.gov/entrez/query.fcgi?db=gene&cmd=Retrieve&dopt=full_report&list_uids=5863) | ral guanine nucleotide dissociation stimulator like 2 |
| [Details](http://mirdb.org/cgi-bin/target_detail.cgi?targetID=3073114) | 508 | 84 | hsa-miR-27a-3p | [UBR5](http://www.ncbi.nlm.nih.gov/entrez/query.fcgi?db=gene&cmd=Retrieve&dopt=full_report&list_uids=51366) | ubiquitin protein ligase E3 component n-recognin 5 |
| [Details](http://mirdb.org/cgi-bin/target_detail.cgi?targetID=3073144) | 509 | 84 | hsa-miR-27a-3p | [UNC5D](http://www.ncbi.nlm.nih.gov/entrez/query.fcgi?db=gene&cmd=Retrieve&dopt=full_report&list_uids=137970) | unc-5 netrin receptor D |
| [Details](http://mirdb.org/cgi-bin/target_detail.cgi?targetID=3073185) | 510 | 84 | hsa-miR-27a-3p | [PSMA1](http://www.ncbi.nlm.nih.gov/entrez/query.fcgi?db=gene&cmd=Retrieve&dopt=full_report&list_uids=5682) | proteasome subunit alpha 1 |
| [Details](http://mirdb.org/cgi-bin/target_detail.cgi?targetID=3073188) | 511 | 84 | hsa-miR-27a-3p | [ZNF426](http://www.ncbi.nlm.nih.gov/entrez/query.fcgi?db=gene&cmd=Retrieve&dopt=full_report&list_uids=79088) | zinc finger protein 426 |
| [Details](http://mirdb.org/cgi-bin/target_detail.cgi?targetID=3073276) | 512 | 84 | hsa-miR-27a-3p | [HDHD2](http://www.ncbi.nlm.nih.gov/entrez/query.fcgi?db=gene&cmd=Retrieve&dopt=full_report&list_uids=84064) | haloacid dehalogenase like hydrolase domain containing 2 |
| [Details](http://mirdb.org/cgi-bin/target_detail.cgi?targetID=3073331) | 513 | 84 | hsa-miR-27a-3p | [FAM217B](http://www.ncbi.nlm.nih.gov/entrez/query.fcgi?db=gene&cmd=Retrieve&dopt=full_report&list_uids=63939) | family with sequence similarity 217 member B |
| [Details](http://mirdb.org/cgi-bin/target_detail.cgi?targetID=3073425) | 514 | 84 | hsa-miR-27a-3p | [KCNK5](http://www.ncbi.nlm.nih.gov/entrez/query.fcgi?db=gene&cmd=Retrieve&dopt=full_report&list_uids=8645) | potassium two pore domain channel subfamily K member 5 |
| [Details](http://mirdb.org/cgi-bin/target_detail.cgi?targetID=3073480) | 515 | 84 | hsa-miR-27a-3p | [FAM126A](http://www.ncbi.nlm.nih.gov/entrez/query.fcgi?db=gene&cmd=Retrieve&dopt=full_report&list_uids=84668) | family with sequence similarity 126 member A |
| [Details](http://mirdb.org/cgi-bin/target_detail.cgi?targetID=3073539) | 516 | 84 | hsa-miR-27a-3p | [PRMT8](http://www.ncbi.nlm.nih.gov/entrez/query.fcgi?db=gene&cmd=Retrieve&dopt=full_report&list_uids=56341) | protein arginine methyltransferase 8 |
| [Details](http://mirdb.org/cgi-bin/target_detail.cgi?targetID=3073618) | 517 | 84 | hsa-miR-27a-3p | [AHSA2P](http://www.ncbi.nlm.nih.gov/entrez/query.fcgi?db=gene&cmd=Retrieve&dopt=full_report&list_uids=130872) | activator of HSP90 ATPase homolog 2, pseudogene |
| [Details](http://mirdb.org/cgi-bin/target_detail.cgi?targetID=3073641) | 518 | 84 | hsa-miR-27a-3p | [ADGRA3](http://www.ncbi.nlm.nih.gov/entrez/query.fcgi?db=gene&cmd=Retrieve&dopt=full_report&list_uids=166647) | adhesion G protein-coupled receptor A3 |
| [Details](http://mirdb.org/cgi-bin/target_detail.cgi?targetID=3073650) | 519 | 84 | hsa-miR-27a-3p | [ARMC8](http://www.ncbi.nlm.nih.gov/entrez/query.fcgi?db=gene&cmd=Retrieve&dopt=full_report&list_uids=25852) | armadillo repeat containing 8 |
| [Details](http://mirdb.org/cgi-bin/target_detail.cgi?targetID=3073702) | 520 | 84 | hsa-miR-27a-3p | [ALG9](http://www.ncbi.nlm.nih.gov/entrez/query.fcgi?db=gene&cmd=Retrieve&dopt=full_report&list_uids=79796) | ALG9, alpha-1,2-mannosyltransferase |
| [Details](http://mirdb.org/cgi-bin/target_detail.cgi?targetID=3073738) | 521 | 84 | hsa-miR-27a-3p | [DLL4](http://www.ncbi.nlm.nih.gov/entrez/query.fcgi?db=gene&cmd=Retrieve&dopt=full_report&list_uids=54567) | delta like canonical Notch ligand 4 |
| [Details](http://mirdb.org/cgi-bin/target_detail.cgi?targetID=3073817) | 522 | 84 | hsa-miR-27a-3p | [CBLB](http://www.ncbi.nlm.nih.gov/entrez/query.fcgi?db=gene&cmd=Retrieve&dopt=full_report&list_uids=868) | Cbl proto-oncogene B |
| [Details](http://mirdb.org/cgi-bin/target_detail.cgi?targetID=3073891) | 523 | 84 | hsa-miR-27a-3p | [FGF7](http://www.ncbi.nlm.nih.gov/entrez/query.fcgi?db=gene&cmd=Retrieve&dopt=full_report&list_uids=2252) | fibroblast growth factor 7 |
| [Details](http://mirdb.org/cgi-bin/target_detail.cgi?targetID=3072457) | 524 | 83 | hsa-miR-27a-3p | [CNN3](http://www.ncbi.nlm.nih.gov/entrez/query.fcgi?db=gene&cmd=Retrieve&dopt=full_report&list_uids=1266) | calponin 3 |
| [Details](http://mirdb.org/cgi-bin/target_detail.cgi?targetID=3072499) | 525 | 83 | hsa-miR-27a-3p | [AMD1](http://www.ncbi.nlm.nih.gov/entrez/query.fcgi?db=gene&cmd=Retrieve&dopt=full_report&list_uids=262) | adenosylmethionine decarboxylase 1 |
| [Details](http://mirdb.org/cgi-bin/target_detail.cgi?targetID=3072518) | 526 | 83 | hsa-miR-27a-3p | [CELF2](http://www.ncbi.nlm.nih.gov/entrez/query.fcgi?db=gene&cmd=Retrieve&dopt=full_report&list_uids=10659) | CUGBP Elav-like family member 2 |
| [Details](http://mirdb.org/cgi-bin/target_detail.cgi?targetID=3072525) | 527 | 83 | hsa-miR-27a-3p | [INPP5J](http://www.ncbi.nlm.nih.gov/entrez/query.fcgi?db=gene&cmd=Retrieve&dopt=full_report&list_uids=27124) | inositol polyphosphate-5-phosphatase J |
| [Details](http://mirdb.org/cgi-bin/target_detail.cgi?targetID=3072613) | 528 | 83 | hsa-miR-27a-3p | [CEMIP](http://www.ncbi.nlm.nih.gov/entrez/query.fcgi?db=gene&cmd=Retrieve&dopt=full_report&list_uids=57214) | cell migration inducing hyaluronidase 1 |
| [Details](http://mirdb.org/cgi-bin/target_detail.cgi?targetID=3072684) | 529 | 83 | hsa-miR-27a-3p | [KLHL31](http://www.ncbi.nlm.nih.gov/entrez/query.fcgi?db=gene&cmd=Retrieve&dopt=full_report&list_uids=401265) | kelch like family member 31 |
| [Details](http://mirdb.org/cgi-bin/target_detail.cgi?targetID=3072700) | 530 | 83 | hsa-miR-27a-3p | [NOVA1](http://www.ncbi.nlm.nih.gov/entrez/query.fcgi?db=gene&cmd=Retrieve&dopt=full_report&list_uids=4857) | NOVA alternative splicing regulator 1 |
| [Details](http://mirdb.org/cgi-bin/target_detail.cgi?targetID=3072738) | 531 | 83 | hsa-miR-27a-3p | [PNPLA7](http://www.ncbi.nlm.nih.gov/entrez/query.fcgi?db=gene&cmd=Retrieve&dopt=full_report&list_uids=375775) | patatin like phospholipase domain containing 7 |
| [Details](http://mirdb.org/cgi-bin/target_detail.cgi?targetID=3072778) | 532 | 83 | hsa-miR-27a-3p | [TSPYL1](http://www.ncbi.nlm.nih.gov/entrez/query.fcgi?db=gene&cmd=Retrieve&dopt=full_report&list_uids=7259) | TSPY like 1 |
| [Details](http://mirdb.org/cgi-bin/target_detail.cgi?targetID=3072791) | 533 | 83 | hsa-miR-27a-3p | [POU3F2](http://www.ncbi.nlm.nih.gov/entrez/query.fcgi?db=gene&cmd=Retrieve&dopt=full_report&list_uids=5454) | POU class 3 homeobox 2 |
| [Details](http://mirdb.org/cgi-bin/target_detail.cgi?targetID=3072816) | 534 | 83 | hsa-miR-27a-3p | [CPPED1](http://www.ncbi.nlm.nih.gov/entrez/query.fcgi?db=gene&cmd=Retrieve&dopt=full_report&list_uids=55313) | calcineurin like phosphoesterase domain containing 1 |
| [Details](http://mirdb.org/cgi-bin/target_detail.cgi?targetID=3072856) | 535 | 83 | hsa-miR-27a-3p | [RAP2A](http://www.ncbi.nlm.nih.gov/entrez/query.fcgi?db=gene&cmd=Retrieve&dopt=full_report&list_uids=5911) | RAP2A, member of RAS oncogene family |
| [Details](http://mirdb.org/cgi-bin/target_detail.cgi?targetID=3072910) | 536 | 83 | hsa-miR-27a-3p | [SLC35A3](http://www.ncbi.nlm.nih.gov/entrez/query.fcgi?db=gene&cmd=Retrieve&dopt=full_report&list_uids=23443) | solute carrier family 35 member A3 |
| [Details](http://mirdb.org/cgi-bin/target_detail.cgi?targetID=3072996) | 537 | 83 | hsa-miR-27a-3p | [CHKA](http://www.ncbi.nlm.nih.gov/entrez/query.fcgi?db=gene&cmd=Retrieve&dopt=full_report&list_uids=1119) | choline kinase alpha |
| [Details](http://mirdb.org/cgi-bin/target_detail.cgi?targetID=3073069) | 538 | 83 | hsa-miR-27a-3p | [DYNLL2](http://www.ncbi.nlm.nih.gov/entrez/query.fcgi?db=gene&cmd=Retrieve&dopt=full_report&list_uids=140735) | dynein light chain LC8-type 2 |
| [Details](http://mirdb.org/cgi-bin/target_detail.cgi?targetID=3073073) | 539 | 83 | hsa-miR-27a-3p | [STX16](http://www.ncbi.nlm.nih.gov/entrez/query.fcgi?db=gene&cmd=Retrieve&dopt=full_report&list_uids=8675) | syntaxin 16 |
| [Details](http://mirdb.org/cgi-bin/target_detail.cgi?targetID=3073171) | 540 | 83 | hsa-miR-27a-3p | [ZBTB39](http://www.ncbi.nlm.nih.gov/entrez/query.fcgi?db=gene&cmd=Retrieve&dopt=full_report&list_uids=9880) | zinc finger and BTB domain containing 39 |
| [Details](http://mirdb.org/cgi-bin/target_detail.cgi?targetID=3073252) | 541 | 83 | hsa-miR-27a-3p | [SH3GL3](http://www.ncbi.nlm.nih.gov/entrez/query.fcgi?db=gene&cmd=Retrieve&dopt=full_report&list_uids=6457) | SH3 domain containing GRB2 like 3, endophilin A3 |
| [Details](http://mirdb.org/cgi-bin/target_detail.cgi?targetID=3073291) | 542 | 83 | hsa-miR-27a-3p | [WBP1L](http://www.ncbi.nlm.nih.gov/entrez/query.fcgi?db=gene&cmd=Retrieve&dopt=full_report&list_uids=54838) | WW domain binding protein 1 like |
| [Details](http://mirdb.org/cgi-bin/target_detail.cgi?targetID=3073314) | 543 | 83 | hsa-miR-27a-3p | [SOX7](http://www.ncbi.nlm.nih.gov/entrez/query.fcgi?db=gene&cmd=Retrieve&dopt=full_report&list_uids=83595) | SRY-box 7 |
| [Details](http://mirdb.org/cgi-bin/target_detail.cgi?targetID=3073492) | 544 | 83 | hsa-miR-27a-3p | [COPZ1](http://www.ncbi.nlm.nih.gov/entrez/query.fcgi?db=gene&cmd=Retrieve&dopt=full_report&list_uids=22818) | coatomer protein complex subunit zeta 1 |
| [Details](http://mirdb.org/cgi-bin/target_detail.cgi?targetID=3073601) | 545 | 83 | hsa-miR-27a-3p | [DYRK1A](http://www.ncbi.nlm.nih.gov/entrez/query.fcgi?db=gene&cmd=Retrieve&dopt=full_report&list_uids=1859) | dual specificity tyrosine phosphorylation regulated kinase 1A |
| [Details](http://mirdb.org/cgi-bin/target_detail.cgi?targetID=3073625) | 546 | 83 | hsa-miR-27a-3p | [MAP2K7](http://www.ncbi.nlm.nih.gov/entrez/query.fcgi?db=gene&cmd=Retrieve&dopt=full_report&list_uids=5609) | mitogen-activated protein kinase kinase 7 |
| [Details](http://mirdb.org/cgi-bin/target_detail.cgi?targetID=3073655) | 547 | 83 | hsa-miR-27a-3p | [ZNF608](http://www.ncbi.nlm.nih.gov/entrez/query.fcgi?db=gene&cmd=Retrieve&dopt=full_report&list_uids=57507) | zinc finger protein 608 |
| [Details](http://mirdb.org/cgi-bin/target_detail.cgi?targetID=3073697) | 548 | 83 | hsa-miR-27a-3p | [PELI2](http://www.ncbi.nlm.nih.gov/entrez/query.fcgi?db=gene&cmd=Retrieve&dopt=full_report&list_uids=57161) | pellino E3 ubiquitin protein ligase family member 2 |
| [Details](http://mirdb.org/cgi-bin/target_detail.cgi?targetID=3073747) | 549 | 83 | hsa-miR-27a-3p | [HSPD1](http://www.ncbi.nlm.nih.gov/entrez/query.fcgi?db=gene&cmd=Retrieve&dopt=full_report&list_uids=3329) | heat shock protein family D (Hsp60) member 1 |
| [Details](http://mirdb.org/cgi-bin/target_detail.cgi?targetID=3072428) | 550 | 82 | hsa-miR-27a-3p | [MYH10](http://www.ncbi.nlm.nih.gov/entrez/query.fcgi?db=gene&cmd=Retrieve&dopt=full_report&list_uids=4628) | myosin heavy chain 10 |
| [Details](http://mirdb.org/cgi-bin/target_detail.cgi?targetID=3072786) | 551 | 82 | hsa-miR-27a-3p | [PRKCB](http://www.ncbi.nlm.nih.gov/entrez/query.fcgi?db=gene&cmd=Retrieve&dopt=full_report&list_uids=5579) | protein kinase C beta |
| [Details](http://mirdb.org/cgi-bin/target_detail.cgi?targetID=3072817) | 552 | 82 | hsa-miR-27a-3p | [SCN1A](http://www.ncbi.nlm.nih.gov/entrez/query.fcgi?db=gene&cmd=Retrieve&dopt=full_report&list_uids=6323) | sodium voltage-gated channel alpha subunit 1 |
| [Details](http://mirdb.org/cgi-bin/target_detail.cgi?targetID=3072829) | 553 | 82 | hsa-miR-27a-3p | [KMT5B](http://www.ncbi.nlm.nih.gov/entrez/query.fcgi?db=gene&cmd=Retrieve&dopt=full_report&list_uids=51111) | lysine methyltransferase 5B |
| [Details](http://mirdb.org/cgi-bin/target_detail.cgi?targetID=3072887) | 554 | 82 | hsa-miR-27a-3p | [ZFAND3](http://www.ncbi.nlm.nih.gov/entrez/query.fcgi?db=gene&cmd=Retrieve&dopt=full_report&list_uids=60685) | zinc finger AN1-type containing 3 |
| [Details](http://mirdb.org/cgi-bin/target_detail.cgi?targetID=3072890) | 555 | 82 | hsa-miR-27a-3p | [RNF38](http://www.ncbi.nlm.nih.gov/entrez/query.fcgi?db=gene&cmd=Retrieve&dopt=full_report&list_uids=152006) | ring finger protein 38 |
| [Details](http://mirdb.org/cgi-bin/target_detail.cgi?targetID=3073045) | 556 | 82 | hsa-miR-27a-3p | [SPTLC2](http://www.ncbi.nlm.nih.gov/entrez/query.fcgi?db=gene&cmd=Retrieve&dopt=full_report&list_uids=9517) | serine palmitoyltransferase long chain base subunit 2 |
| [Details](http://mirdb.org/cgi-bin/target_detail.cgi?targetID=3073082) | 557 | 82 | hsa-miR-27a-3p | [BNIP3](http://www.ncbi.nlm.nih.gov/entrez/query.fcgi?db=gene&cmd=Retrieve&dopt=full_report&list_uids=664) | BCL2 interacting protein 3 |
| [Details](http://mirdb.org/cgi-bin/target_detail.cgi?targetID=3073089) | 558 | 82 | hsa-miR-27a-3p | [SLC7A2](http://www.ncbi.nlm.nih.gov/entrez/query.fcgi?db=gene&cmd=Retrieve&dopt=full_report&list_uids=6542) | solute carrier family 7 member 2 |
| [Details](http://mirdb.org/cgi-bin/target_detail.cgi?targetID=3073103) | 559 | 82 | hsa-miR-27a-3p | [IRF4](http://www.ncbi.nlm.nih.gov/entrez/query.fcgi?db=gene&cmd=Retrieve&dopt=full_report&list_uids=3662) | interferon regulatory factor 4 |
| [Details](http://mirdb.org/cgi-bin/target_detail.cgi?targetID=3073211) | 560 | 82 | hsa-miR-27a-3p | [YWHAB](http://www.ncbi.nlm.nih.gov/entrez/query.fcgi?db=gene&cmd=Retrieve&dopt=full_report&list_uids=7529) | tyrosine 3-monooxygenase/tryptophan 5-monooxygenase activation protein beta |
| [Details](http://mirdb.org/cgi-bin/target_detail.cgi?targetID=3073233) | 561 | 82 | hsa-miR-27a-3p | [HIP1](http://www.ncbi.nlm.nih.gov/entrez/query.fcgi?db=gene&cmd=Retrieve&dopt=full_report&list_uids=3092) | huntingtin interacting protein 1 |
| [Details](http://mirdb.org/cgi-bin/target_detail.cgi?targetID=3073301) | 562 | 82 | hsa-miR-27a-3p | [SLC9A7](http://www.ncbi.nlm.nih.gov/entrez/query.fcgi?db=gene&cmd=Retrieve&dopt=full_report&list_uids=84679) | solute carrier family 9 member A7 |
| [Details](http://mirdb.org/cgi-bin/target_detail.cgi?targetID=3073311) | 563 | 82 | hsa-miR-27a-3p | [FAM171A1](http://www.ncbi.nlm.nih.gov/entrez/query.fcgi?db=gene&cmd=Retrieve&dopt=full_report&list_uids=221061) | family with sequence similarity 171 member A1 |
| [Details](http://mirdb.org/cgi-bin/target_detail.cgi?targetID=3073430) | 564 | 82 | hsa-miR-27a-3p | [SGIP1](http://www.ncbi.nlm.nih.gov/entrez/query.fcgi?db=gene&cmd=Retrieve&dopt=full_report&list_uids=84251) | SH3 domain GRB2 like endophilin interacting protein 1 |
| [Details](http://mirdb.org/cgi-bin/target_detail.cgi?targetID=3073475) | 565 | 82 | hsa-miR-27a-3p | [NR3C1](http://www.ncbi.nlm.nih.gov/entrez/query.fcgi?db=gene&cmd=Retrieve&dopt=full_report&list_uids=2908) | nuclear receptor subfamily 3 group C member 1 |
| [Details](http://mirdb.org/cgi-bin/target_detail.cgi?targetID=3073602) | 566 | 82 | hsa-miR-27a-3p | [KCNN3](http://www.ncbi.nlm.nih.gov/entrez/query.fcgi?db=gene&cmd=Retrieve&dopt=full_report&list_uids=3782) | potassium calcium-activated channel subfamily N member 3 |
| [Details](http://mirdb.org/cgi-bin/target_detail.cgi?targetID=3073696) | 567 | 82 | hsa-miR-27a-3p | [CDH5](http://www.ncbi.nlm.nih.gov/entrez/query.fcgi?db=gene&cmd=Retrieve&dopt=full_report&list_uids=1003) | cadherin 5 |
| [Details](http://mirdb.org/cgi-bin/target_detail.cgi?targetID=3073699) | 568 | 82 | hsa-miR-27a-3p | [HS2ST1](http://www.ncbi.nlm.nih.gov/entrez/query.fcgi?db=gene&cmd=Retrieve&dopt=full_report&list_uids=9653) | heparan sulfate 2-O-sulfotransferase 1 |
| [Details](http://mirdb.org/cgi-bin/target_detail.cgi?targetID=3073716) | 569 | 82 | hsa-miR-27a-3p | [TMPRSS7](http://www.ncbi.nlm.nih.gov/entrez/query.fcgi?db=gene&cmd=Retrieve&dopt=full_report&list_uids=344805) | transmembrane serine protease 7 |
| [Details](http://mirdb.org/cgi-bin/target_detail.cgi?targetID=3073727) | 570 | 82 | hsa-miR-27a-3p | [NR2C2](http://www.ncbi.nlm.nih.gov/entrez/query.fcgi?db=gene&cmd=Retrieve&dopt=full_report&list_uids=7182) | nuclear receptor subfamily 2 group C member 2 |
| [Details](http://mirdb.org/cgi-bin/target_detail.cgi?targetID=3073743) | 571 | 82 | hsa-miR-27a-3p | [NETO1](http://www.ncbi.nlm.nih.gov/entrez/query.fcgi?db=gene&cmd=Retrieve&dopt=full_report&list_uids=81832) | neuropilin and tolloid like 1 |
| [Details](http://mirdb.org/cgi-bin/target_detail.cgi?targetID=3073813) | 572 | 82 | hsa-miR-27a-3p | [NREP](http://www.ncbi.nlm.nih.gov/entrez/query.fcgi?db=gene&cmd=Retrieve&dopt=full_report&list_uids=9315) | neuronal regeneration related protein |
| [Details](http://mirdb.org/cgi-bin/target_detail.cgi?targetID=3073832) | 573 | 82 | hsa-miR-27a-3p | [TTC39A](http://www.ncbi.nlm.nih.gov/entrez/query.fcgi?db=gene&cmd=Retrieve&dopt=full_report&list_uids=22996) | tetratricopeptide repeat domain 39A |
| [Details](http://mirdb.org/cgi-bin/target_detail.cgi?targetID=3073854) | 574 | 82 | hsa-miR-27a-3p | [ADAMTSL1](http://www.ncbi.nlm.nih.gov/entrez/query.fcgi?db=gene&cmd=Retrieve&dopt=full_report&list_uids=92949) | ADAMTS like 1 |
| [Details](http://mirdb.org/cgi-bin/target_detail.cgi?targetID=3073890) | 575 | 82 | hsa-miR-27a-3p | [LARP4](http://www.ncbi.nlm.nih.gov/entrez/query.fcgi?db=gene&cmd=Retrieve&dopt=full_report&list_uids=113251) | La ribonucleoprotein domain family member 4 |
| [Details](http://mirdb.org/cgi-bin/target_detail.cgi?targetID=3072442) | 576 | 81 | hsa-miR-27a-3p | [KAT2B](http://www.ncbi.nlm.nih.gov/entrez/query.fcgi?db=gene&cmd=Retrieve&dopt=full_report&list_uids=8850) | lysine acetyltransferase 2B |
| [Details](http://mirdb.org/cgi-bin/target_detail.cgi?targetID=3072464) | 577 | 81 | hsa-miR-27a-3p | [DCAF7](http://www.ncbi.nlm.nih.gov/entrez/query.fcgi?db=gene&cmd=Retrieve&dopt=full_report&list_uids=10238) | DDB1 and CUL4 associated factor 7 |
| [Details](http://mirdb.org/cgi-bin/target_detail.cgi?targetID=3072551) | 578 | 81 | hsa-miR-27a-3p | [RCBTB1](http://www.ncbi.nlm.nih.gov/entrez/query.fcgi?db=gene&cmd=Retrieve&dopt=full_report&list_uids=55213) | RCC1 and BTB domain containing protein 1 |
| [Details](http://mirdb.org/cgi-bin/target_detail.cgi?targetID=3072574) | 579 | 81 | hsa-miR-27a-3p | [UBE2N](http://www.ncbi.nlm.nih.gov/entrez/query.fcgi?db=gene&cmd=Retrieve&dopt=full_report&list_uids=7334) | ubiquitin conjugating enzyme E2 N |
| [Details](http://mirdb.org/cgi-bin/target_detail.cgi?targetID=3072597) | 580 | 81 | hsa-miR-27a-3p | [FBXO45](http://www.ncbi.nlm.nih.gov/entrez/query.fcgi?db=gene&cmd=Retrieve&dopt=full_report&list_uids=200933) | F-box protein 45 |
| [Details](http://mirdb.org/cgi-bin/target_detail.cgi?targetID=3072630) | 581 | 81 | hsa-miR-27a-3p | [PEAK1](http://www.ncbi.nlm.nih.gov/entrez/query.fcgi?db=gene&cmd=Retrieve&dopt=full_report&list_uids=79834) | pseudopodium enriched atypical kinase 1 |
| [Details](http://mirdb.org/cgi-bin/target_detail.cgi?targetID=3072635) | 582 | 81 | hsa-miR-27a-3p | [ZNF705E](http://www.ncbi.nlm.nih.gov/entrez/query.fcgi?db=gene&cmd=Retrieve&dopt=full_report&list_uids=100131539) | zinc finger protein 705E |
| [Details](http://mirdb.org/cgi-bin/target_detail.cgi?targetID=3072655) | 583 | 81 | hsa-miR-27a-3p | [SLC5A7](http://www.ncbi.nlm.nih.gov/entrez/query.fcgi?db=gene&cmd=Retrieve&dopt=full_report&list_uids=60482) | solute carrier family 5 member 7 |
| [Details](http://mirdb.org/cgi-bin/target_detail.cgi?targetID=3072675) | 584 | 81 | hsa-miR-27a-3p | [FAM76B](http://www.ncbi.nlm.nih.gov/entrez/query.fcgi?db=gene&cmd=Retrieve&dopt=full_report&list_uids=143684) | family with sequence similarity 76 member B |
| [Details](http://mirdb.org/cgi-bin/target_detail.cgi?targetID=3072721) | 585 | 81 | hsa-miR-27a-3p | [KBTBD8](http://www.ncbi.nlm.nih.gov/entrez/query.fcgi?db=gene&cmd=Retrieve&dopt=full_report&list_uids=84541) | kelch repeat and BTB domain containing 8 |
| [Details](http://mirdb.org/cgi-bin/target_detail.cgi?targetID=3072780) | 586 | 81 | hsa-miR-27a-3p | [TMEM184A](http://www.ncbi.nlm.nih.gov/entrez/query.fcgi?db=gene&cmd=Retrieve&dopt=full_report&list_uids=202915) | transmembrane protein 184A |
| [Details](http://mirdb.org/cgi-bin/target_detail.cgi?targetID=3072864) | 587 | 81 | hsa-miR-27a-3p | [FLRT3](http://www.ncbi.nlm.nih.gov/entrez/query.fcgi?db=gene&cmd=Retrieve&dopt=full_report&list_uids=23767) | fibronectin leucine rich transmembrane protein 3 |
| [Details](http://mirdb.org/cgi-bin/target_detail.cgi?targetID=3072865) | 588 | 81 | hsa-miR-27a-3p | [SIK1](http://www.ncbi.nlm.nih.gov/entrez/query.fcgi?db=gene&cmd=Retrieve&dopt=full_report&list_uids=150094) | salt inducible kinase 1 |
| [Details](http://mirdb.org/cgi-bin/target_detail.cgi?targetID=3072928) | 589 | 81 | hsa-miR-27a-3p | [BNIP2](http://www.ncbi.nlm.nih.gov/entrez/query.fcgi?db=gene&cmd=Retrieve&dopt=full_report&list_uids=663) | BCL2 interacting protein 2 |
| [Details](http://mirdb.org/cgi-bin/target_detail.cgi?targetID=3072931) | 590 | 81 | hsa-miR-27a-3p | [SMAD4](http://www.ncbi.nlm.nih.gov/entrez/query.fcgi?db=gene&cmd=Retrieve&dopt=full_report&list_uids=4089) | SMAD family member 4 |
| [Details](http://mirdb.org/cgi-bin/target_detail.cgi?targetID=3072943) | 591 | 81 | hsa-miR-27a-3p | [GRAMD1B](http://www.ncbi.nlm.nih.gov/entrez/query.fcgi?db=gene&cmd=Retrieve&dopt=full_report&list_uids=57476) | GRAM domain containing 1B |
| [Details](http://mirdb.org/cgi-bin/target_detail.cgi?targetID=3072964) | 592 | 81 | hsa-miR-27a-3p | [CECR2](http://www.ncbi.nlm.nih.gov/entrez/query.fcgi?db=gene&cmd=Retrieve&dopt=full_report&list_uids=27443) | CECR2, histone acetyl-lysine reader |
| [Details](http://mirdb.org/cgi-bin/target_detail.cgi?targetID=3073011) | 593 | 81 | hsa-miR-27a-3p | [ECE2](http://www.ncbi.nlm.nih.gov/entrez/query.fcgi?db=gene&cmd=Retrieve&dopt=full_report&list_uids=9718) | endothelin converting enzyme 2 |
| [Details](http://mirdb.org/cgi-bin/target_detail.cgi?targetID=3073066) | 594 | 81 | hsa-miR-27a-3p | [HYOU1](http://www.ncbi.nlm.nih.gov/entrez/query.fcgi?db=gene&cmd=Retrieve&dopt=full_report&list_uids=10525) | hypoxia up-regulated 1 |
| [Details](http://mirdb.org/cgi-bin/target_detail.cgi?targetID=3073072) | 595 | 81 | hsa-miR-27a-3p | [DCAF12](http://www.ncbi.nlm.nih.gov/entrez/query.fcgi?db=gene&cmd=Retrieve&dopt=full_report&list_uids=25853) | DDB1 and CUL4 associated factor 12 |
| [Details](http://mirdb.org/cgi-bin/target_detail.cgi?targetID=3073087) | 596 | 81 | hsa-miR-27a-3p | [SCAI](http://www.ncbi.nlm.nih.gov/entrez/query.fcgi?db=gene&cmd=Retrieve&dopt=full_report&list_uids=286205) | suppressor of cancer cell invasion |
| [Details](http://mirdb.org/cgi-bin/target_detail.cgi?targetID=3073180) | 597 | 81 | hsa-miR-27a-3p | [SIK1B](http://www.ncbi.nlm.nih.gov/entrez/query.fcgi?db=gene&cmd=Retrieve&dopt=full_report&list_uids=102724428) | salt inducible kinase 1B (putative) |
| [Details](http://mirdb.org/cgi-bin/target_detail.cgi?targetID=3073255) | 598 | 81 | hsa-miR-27a-3p | [MGAT4A](http://www.ncbi.nlm.nih.gov/entrez/query.fcgi?db=gene&cmd=Retrieve&dopt=full_report&list_uids=11320) | alpha-1,3-mannosyl-glycoprotein 4-beta-N-acetylglucosaminyltransferase A |
| [Details](http://mirdb.org/cgi-bin/target_detail.cgi?targetID=3073326) | 599 | 81 | hsa-miR-27a-3p | [TRAF3](http://www.ncbi.nlm.nih.gov/entrez/query.fcgi?db=gene&cmd=Retrieve&dopt=full_report&list_uids=7187) | TNF receptor associated factor 3 |
| [Details](http://mirdb.org/cgi-bin/target_detail.cgi?targetID=3073400) | 600 | 81 | hsa-miR-27a-3p | [SERBP1](http://www.ncbi.nlm.nih.gov/entrez/query.fcgi?db=gene&cmd=Retrieve&dopt=full_report&list_uids=26135) | SERPINE1 mRNA binding protein 1 |
| [Details](http://mirdb.org/cgi-bin/target_detail.cgi?targetID=3073402) | 601 | 81 | hsa-miR-27a-3p | [UHRF1BP1](http://www.ncbi.nlm.nih.gov/entrez/query.fcgi?db=gene&cmd=Retrieve&dopt=full_report&list_uids=54887) | UHRF1 binding protein 1 |
| [Details](http://mirdb.org/cgi-bin/target_detail.cgi?targetID=3073427) | 602 | 81 | hsa-miR-27a-3p | [EIF5A2](http://www.ncbi.nlm.nih.gov/entrez/query.fcgi?db=gene&cmd=Retrieve&dopt=full_report&list_uids=56648) | eukaryotic translation initiation factor 5A2 |
| [Details](http://mirdb.org/cgi-bin/target_detail.cgi?targetID=3073436) | 603 | 81 | hsa-miR-27a-3p | [CHL1](http://www.ncbi.nlm.nih.gov/entrez/query.fcgi?db=gene&cmd=Retrieve&dopt=full_report&list_uids=10752) | cell adhesion molecule L1 like |
| [Details](http://mirdb.org/cgi-bin/target_detail.cgi?targetID=3073472) | 604 | 81 | hsa-miR-27a-3p | [QKI](http://www.ncbi.nlm.nih.gov/entrez/query.fcgi?db=gene&cmd=Retrieve&dopt=full_report&list_uids=9444) | QKI, KH domain containing RNA binding |
| [Details](http://mirdb.org/cgi-bin/target_detail.cgi?targetID=3073488) | 605 | 81 | hsa-miR-27a-3p | [EEF1AKMT4-ECE2](http://www.ncbi.nlm.nih.gov/entrez/query.fcgi?db=gene&cmd=Retrieve&dopt=full_report&list_uids=110599583) | EEF1AKMT4-ECE2 readthrough |
| [Details](http://mirdb.org/cgi-bin/target_detail.cgi?targetID=3073506) | 606 | 81 | hsa-miR-27a-3p | [ZNF84](http://www.ncbi.nlm.nih.gov/entrez/query.fcgi?db=gene&cmd=Retrieve&dopt=full_report&list_uids=7637) | zinc finger protein 84 |
| [Details](http://mirdb.org/cgi-bin/target_detail.cgi?targetID=3073510) | 607 | 81 | hsa-miR-27a-3p | [TBR1](http://www.ncbi.nlm.nih.gov/entrez/query.fcgi?db=gene&cmd=Retrieve&dopt=full_report&list_uids=10716) | T-box, brain 1 |
| [Details](http://mirdb.org/cgi-bin/target_detail.cgi?targetID=3073530) | 608 | 81 | hsa-miR-27a-3p | [NF1](http://www.ncbi.nlm.nih.gov/entrez/query.fcgi?db=gene&cmd=Retrieve&dopt=full_report&list_uids=4763) | neurofibromin 1 |
| [Details](http://mirdb.org/cgi-bin/target_detail.cgi?targetID=3073771) | 609 | 81 | hsa-miR-27a-3p | [ZIC5](http://www.ncbi.nlm.nih.gov/entrez/query.fcgi?db=gene&cmd=Retrieve&dopt=full_report&list_uids=85416) | Zic family member 5 |
| [Details](http://mirdb.org/cgi-bin/target_detail.cgi?targetID=3073837) | 610 | 81 | hsa-miR-27a-3p | [FUBP3](http://www.ncbi.nlm.nih.gov/entrez/query.fcgi?db=gene&cmd=Retrieve&dopt=full_report&list_uids=8939) | far upstream element binding protein 3 |
| [Details](http://mirdb.org/cgi-bin/target_detail.cgi?targetID=3072452) | 611 | 80 | hsa-miR-27a-3p | [RCOR3](http://www.ncbi.nlm.nih.gov/entrez/query.fcgi?db=gene&cmd=Retrieve&dopt=full_report&list_uids=55758) | REST corepressor 3 |
| [Details](http://mirdb.org/cgi-bin/target_detail.cgi?targetID=3072495) | 612 | 80 | hsa-miR-27a-3p | [VAPB](http://www.ncbi.nlm.nih.gov/entrez/query.fcgi?db=gene&cmd=Retrieve&dopt=full_report&list_uids=9217) | VAMP associated protein B and C |
| [Details](http://mirdb.org/cgi-bin/target_detail.cgi?targetID=3072590) | 613 | 80 | hsa-miR-27a-3p | [ZBTB42](http://www.ncbi.nlm.nih.gov/entrez/query.fcgi?db=gene&cmd=Retrieve&dopt=full_report&list_uids=100128927) | zinc finger and BTB domain containing 42 |
| [Details](http://mirdb.org/cgi-bin/target_detail.cgi?targetID=3072653) | 614 | 80 | hsa-miR-27a-3p | [LONP2](http://www.ncbi.nlm.nih.gov/entrez/query.fcgi?db=gene&cmd=Retrieve&dopt=full_report&list_uids=83752) | lon peptidase 2, peroxisomal |
| [Details](http://mirdb.org/cgi-bin/target_detail.cgi?targetID=3072753) | 615 | 80 | hsa-miR-27a-3p | [TMEM68](http://www.ncbi.nlm.nih.gov/entrez/query.fcgi?db=gene&cmd=Retrieve&dopt=full_report&list_uids=137695) | transmembrane protein 68 |
| [Details](http://mirdb.org/cgi-bin/target_detail.cgi?targetID=3072776) | 616 | 80 | hsa-miR-27a-3p | [RNF182](http://www.ncbi.nlm.nih.gov/entrez/query.fcgi?db=gene&cmd=Retrieve&dopt=full_report&list_uids=221687) | ring finger protein 182 |
| [Details](http://mirdb.org/cgi-bin/target_detail.cgi?targetID=3072836) | 617 | 80 | hsa-miR-27a-3p | [SFSWAP](http://www.ncbi.nlm.nih.gov/entrez/query.fcgi?db=gene&cmd=Retrieve&dopt=full_report&list_uids=6433) | splicing factor SWAP |
| [Details](http://mirdb.org/cgi-bin/target_detail.cgi?targetID=3072855) | 618 | 80 | hsa-miR-27a-3p | [ISL1](http://www.ncbi.nlm.nih.gov/entrez/query.fcgi?db=gene&cmd=Retrieve&dopt=full_report&list_uids=3670) | ISL LIM homeobox 1 |
| [Details](http://mirdb.org/cgi-bin/target_detail.cgi?targetID=3072935) | 619 | 80 | hsa-miR-27a-3p | [PPP1CC](http://www.ncbi.nlm.nih.gov/entrez/query.fcgi?db=gene&cmd=Retrieve&dopt=full_report&list_uids=5501) | protein phosphatase 1 catalytic subunit gamma |
| [Details](http://mirdb.org/cgi-bin/target_detail.cgi?targetID=3072946) | 620 | 80 | hsa-miR-27a-3p | [CHD2](http://www.ncbi.nlm.nih.gov/entrez/query.fcgi?db=gene&cmd=Retrieve&dopt=full_report&list_uids=1106) | chromodomain helicase DNA binding protein 2 |
| [Details](http://mirdb.org/cgi-bin/target_detail.cgi?targetID=3073113) | 621 | 80 | hsa-miR-27a-3p | [SMAD5](http://www.ncbi.nlm.nih.gov/entrez/query.fcgi?db=gene&cmd=Retrieve&dopt=full_report&list_uids=4090) | SMAD family member 5 |
| [Details](http://mirdb.org/cgi-bin/target_detail.cgi?targetID=3073181) | 622 | 80 | hsa-miR-27a-3p | [CBFA2T3](http://www.ncbi.nlm.nih.gov/entrez/query.fcgi?db=gene&cmd=Retrieve&dopt=full_report&list_uids=863) | CBFA2/RUNX1 translocation partner 3 |
| [Details](http://mirdb.org/cgi-bin/target_detail.cgi?targetID=3073182) | 623 | 80 | hsa-miR-27a-3p | [TICRR](http://www.ncbi.nlm.nih.gov/entrez/query.fcgi?db=gene&cmd=Retrieve&dopt=full_report&list_uids=90381) | TOPBP1 interacting checkpoint and replication regulator |
| [Details](http://mirdb.org/cgi-bin/target_detail.cgi?targetID=3073257) | 624 | 80 | hsa-miR-27a-3p | [FAM102A](http://www.ncbi.nlm.nih.gov/entrez/query.fcgi?db=gene&cmd=Retrieve&dopt=full_report&list_uids=399665) | family with sequence similarity 102 member A |
| [Details](http://mirdb.org/cgi-bin/target_detail.cgi?targetID=3073285) | 625 | 80 | hsa-miR-27a-3p | [PPIF](http://www.ncbi.nlm.nih.gov/entrez/query.fcgi?db=gene&cmd=Retrieve&dopt=full_report&list_uids=10105) | peptidylprolyl isomerase F |
| [Details](http://mirdb.org/cgi-bin/target_detail.cgi?targetID=3073323) | 626 | 80 | hsa-miR-27a-3p | [EDAR](http://www.ncbi.nlm.nih.gov/entrez/query.fcgi?db=gene&cmd=Retrieve&dopt=full_report&list_uids=10913) | ectodysplasin A receptor |
| [Details](http://mirdb.org/cgi-bin/target_detail.cgi?targetID=3073429) | 627 | 80 | hsa-miR-27a-3p | [NR1D2](http://www.ncbi.nlm.nih.gov/entrez/query.fcgi?db=gene&cmd=Retrieve&dopt=full_report&list_uids=9975) | nuclear receptor subfamily 1 group D member 2 |
| [Details](http://mirdb.org/cgi-bin/target_detail.cgi?targetID=3073444) | 628 | 80 | hsa-miR-27a-3p | [PRKAA2](http://www.ncbi.nlm.nih.gov/entrez/query.fcgi?db=gene&cmd=Retrieve&dopt=full_report&list_uids=5563) | protein kinase AMP-activated catalytic subunit alpha 2 |
| [Details](http://mirdb.org/cgi-bin/target_detail.cgi?targetID=3073682) | 629 | 80 | hsa-miR-27a-3p | [CLCN5](http://www.ncbi.nlm.nih.gov/entrez/query.fcgi?db=gene&cmd=Retrieve&dopt=full_report&list_uids=1184) | chloride voltage-gated channel 5 |
| [Details](http://mirdb.org/cgi-bin/target_detail.cgi?targetID=3073781) | 630 | 80 | hsa-miR-27a-3p | [TRERF1](http://www.ncbi.nlm.nih.gov/entrez/query.fcgi?db=gene&cmd=Retrieve&dopt=full_report&list_uids=55809) | transcriptional regulating factor 1 |
| [Details](http://mirdb.org/cgi-bin/target_detail.cgi?targetID=3073804) | 631 | 80 | hsa-miR-27a-3p | [DCP1A](http://www.ncbi.nlm.nih.gov/entrez/query.fcgi?db=gene&cmd=Retrieve&dopt=full_report&list_uids=55802) | decapping mRNA 1A |
| [Details](http://mirdb.org/cgi-bin/target_detail.cgi?targetID=3073814) | 632 | 80 | hsa-miR-27a-3p | [KCMF1](http://www.ncbi.nlm.nih.gov/entrez/query.fcgi?db=gene&cmd=Retrieve&dopt=full_report&list_uids=56888) | potassium channel modulatory factor 1 |
| [Details](http://mirdb.org/cgi-bin/target_detail.cgi?targetID=3073820) | 633 | 80 | hsa-miR-27a-3p | [FAM206A](http://www.ncbi.nlm.nih.gov/entrez/query.fcgi?db=gene&cmd=Retrieve&dopt=full_report&list_uids=54942) | family with sequence similarity 206 member A |
| [Details](http://mirdb.org/cgi-bin/target_detail.cgi?targetID=3073863) | 634 | 80 | hsa-miR-27a-3p | [FGD6](http://www.ncbi.nlm.nih.gov/entrez/query.fcgi?db=gene&cmd=Retrieve&dopt=full_report&list_uids=55785) | FYVE, RhoGEF and PH domain containing 6 |
| [Details](http://mirdb.org/cgi-bin/target_detail.cgi?targetID=3072441) | 635 | 79 | hsa-miR-27a-3p | [ZNF75A](http://www.ncbi.nlm.nih.gov/entrez/query.fcgi?db=gene&cmd=Retrieve&dopt=full_report&list_uids=7627) | zinc finger protein 75a |
| [Details](http://mirdb.org/cgi-bin/target_detail.cgi?targetID=3072494) | 636 | 79 | hsa-miR-27a-3p | [GALNT1](http://www.ncbi.nlm.nih.gov/entrez/query.fcgi?db=gene&cmd=Retrieve&dopt=full_report&list_uids=2589) | polypeptide N-acetylgalactosaminyltransferase 1 |
| [Details](http://mirdb.org/cgi-bin/target_detail.cgi?targetID=3072502) | 637 | 79 | hsa-miR-27a-3p | [PDZK1IP1](http://www.ncbi.nlm.nih.gov/entrez/query.fcgi?db=gene&cmd=Retrieve&dopt=full_report&list_uids=10158) | PDZK1 interacting protein 1 |
| [Details](http://mirdb.org/cgi-bin/target_detail.cgi?targetID=3072617) | 638 | 79 | hsa-miR-27a-3p | [PTPRT](http://www.ncbi.nlm.nih.gov/entrez/query.fcgi?db=gene&cmd=Retrieve&dopt=full_report&list_uids=11122) | protein tyrosine phosphatase, receptor type T |
| [Details](http://mirdb.org/cgi-bin/target_detail.cgi?targetID=3072720) | 639 | 79 | hsa-miR-27a-3p | [TMTC4](http://www.ncbi.nlm.nih.gov/entrez/query.fcgi?db=gene&cmd=Retrieve&dopt=full_report&list_uids=84899) | transmembrane and tetratricopeptide repeat containing 4 |
| [Details](http://mirdb.org/cgi-bin/target_detail.cgi?targetID=3072888) | 640 | 79 | hsa-miR-27a-3p | [ARHGAP21](http://www.ncbi.nlm.nih.gov/entrez/query.fcgi?db=gene&cmd=Retrieve&dopt=full_report&list_uids=57584) | Rho GTPase activating protein 21 |
| [Details](http://mirdb.org/cgi-bin/target_detail.cgi?targetID=3072901) | 641 | 79 | hsa-miR-27a-3p | [CNR1](http://www.ncbi.nlm.nih.gov/entrez/query.fcgi?db=gene&cmd=Retrieve&dopt=full_report&list_uids=1268) | cannabinoid receptor 1 |
| [Details](http://mirdb.org/cgi-bin/target_detail.cgi?targetID=3072917) | 642 | 79 | hsa-miR-27a-3p | [ARL6IP1](http://www.ncbi.nlm.nih.gov/entrez/query.fcgi?db=gene&cmd=Retrieve&dopt=full_report&list_uids=23204) | ADP ribosylation factor like GTPase 6 interacting protein 1 |
| [Details](http://mirdb.org/cgi-bin/target_detail.cgi?targetID=3073046) | 643 | 79 | hsa-miR-27a-3p | [GTF2A1](http://www.ncbi.nlm.nih.gov/entrez/query.fcgi?db=gene&cmd=Retrieve&dopt=full_report&list_uids=2957) | general transcription factor IIA subunit 1 |
| [Details](http://mirdb.org/cgi-bin/target_detail.cgi?targetID=3073058) | 644 | 79 | hsa-miR-27a-3p | [CLOCK](http://www.ncbi.nlm.nih.gov/entrez/query.fcgi?db=gene&cmd=Retrieve&dopt=full_report&list_uids=9575) | clock circadian regulator |
| [Details](http://mirdb.org/cgi-bin/target_detail.cgi?targetID=3073095) | 645 | 79 | hsa-miR-27a-3p | [SEC24A](http://www.ncbi.nlm.nih.gov/entrez/query.fcgi?db=gene&cmd=Retrieve&dopt=full_report&list_uids=10802) | SEC24 homolog A, COPII coat complex component |
| [Details](http://mirdb.org/cgi-bin/target_detail.cgi?targetID=3073105) | 646 | 79 | hsa-miR-27a-3p | [PPARA](http://www.ncbi.nlm.nih.gov/entrez/query.fcgi?db=gene&cmd=Retrieve&dopt=full_report&list_uids=5465) | peroxisome proliferator activated receptor alpha |
| [Details](http://mirdb.org/cgi-bin/target_detail.cgi?targetID=3073403) | 647 | 79 | hsa-miR-27a-3p | [DCP2](http://www.ncbi.nlm.nih.gov/entrez/query.fcgi?db=gene&cmd=Retrieve&dopt=full_report&list_uids=167227) | decapping mRNA 2 |
| [Details](http://mirdb.org/cgi-bin/target_detail.cgi?targetID=3073434) | 648 | 79 | hsa-miR-27a-3p | [RXRA](http://www.ncbi.nlm.nih.gov/entrez/query.fcgi?db=gene&cmd=Retrieve&dopt=full_report&list_uids=6256) | retinoid X receptor alpha |
| [Details](http://mirdb.org/cgi-bin/target_detail.cgi?targetID=3073490) | 649 | 79 | hsa-miR-27a-3p | [NR6A1](http://www.ncbi.nlm.nih.gov/entrez/query.fcgi?db=gene&cmd=Retrieve&dopt=full_report&list_uids=2649) | nuclear receptor subfamily 6 group A member 1 |
| [Details](http://mirdb.org/cgi-bin/target_detail.cgi?targetID=3073546) | 650 | 79 | hsa-miR-27a-3p | [CAPZA1](http://www.ncbi.nlm.nih.gov/entrez/query.fcgi?db=gene&cmd=Retrieve&dopt=full_report&list_uids=829) | capping actin protein of muscle Z-line subunit alpha 1 |
| [Details](http://mirdb.org/cgi-bin/target_detail.cgi?targetID=3073562) | 651 | 79 | hsa-miR-27a-3p | [MET](http://www.ncbi.nlm.nih.gov/entrez/query.fcgi?db=gene&cmd=Retrieve&dopt=full_report&list_uids=4233) | MET proto-oncogene, receptor tyrosine kinase |
| [Details](http://mirdb.org/cgi-bin/target_detail.cgi?targetID=3073586) | 652 | 79 | hsa-miR-27a-3p | [NFIA](http://www.ncbi.nlm.nih.gov/entrez/query.fcgi?db=gene&cmd=Retrieve&dopt=full_report&list_uids=4774) | nuclear factor I A |
| [Details](http://mirdb.org/cgi-bin/target_detail.cgi?targetID=3073656) | 653 | 79 | hsa-miR-27a-3p | [CABLES2](http://www.ncbi.nlm.nih.gov/entrez/query.fcgi?db=gene&cmd=Retrieve&dopt=full_report&list_uids=81928) | Cdk5 and Abl enzyme substrate 2 |
| [Details](http://mirdb.org/cgi-bin/target_detail.cgi?targetID=3073719) | 654 | 79 | hsa-miR-27a-3p | [SESN2](http://www.ncbi.nlm.nih.gov/entrez/query.fcgi?db=gene&cmd=Retrieve&dopt=full_report&list_uids=83667) | sestrin 2 |
| [Details](http://mirdb.org/cgi-bin/target_detail.cgi?targetID=3073765) | 655 | 79 | hsa-miR-27a-3p | [NCBP3](http://www.ncbi.nlm.nih.gov/entrez/query.fcgi?db=gene&cmd=Retrieve&dopt=full_report&list_uids=55421) | nuclear cap binding subunit 3 |
| [Details](http://mirdb.org/cgi-bin/target_detail.cgi?targetID=3073796) | 656 | 79 | hsa-miR-27a-3p | [NRCAM](http://www.ncbi.nlm.nih.gov/entrez/query.fcgi?db=gene&cmd=Retrieve&dopt=full_report&list_uids=4897) | neuronal cell adhesion molecule |
| [Details](http://mirdb.org/cgi-bin/target_detail.cgi?targetID=3073852) | 657 | 79 | hsa-miR-27a-3p | [TXN2](http://www.ncbi.nlm.nih.gov/entrez/query.fcgi?db=gene&cmd=Retrieve&dopt=full_report&list_uids=25828) | thioredoxin 2 |
| [Details](http://mirdb.org/cgi-bin/target_detail.cgi?targetID=3073862) | 658 | 79 | hsa-miR-27a-3p | [ACLY](http://www.ncbi.nlm.nih.gov/entrez/query.fcgi?db=gene&cmd=Retrieve&dopt=full_report&list_uids=47) | ATP citrate lyase |
| [Details](http://mirdb.org/cgi-bin/target_detail.cgi?targetID=3073920) | 659 | 79 | hsa-miR-27a-3p | [IFNAR1](http://www.ncbi.nlm.nih.gov/entrez/query.fcgi?db=gene&cmd=Retrieve&dopt=full_report&list_uids=3454) | interferon alpha and beta receptor subunit 1 |
| [Details](http://mirdb.org/cgi-bin/target_detail.cgi?targetID=3072456) | 660 | 78 | hsa-miR-27a-3p | [VDAC3](http://www.ncbi.nlm.nih.gov/entrez/query.fcgi?db=gene&cmd=Retrieve&dopt=full_report&list_uids=7419) | voltage dependent anion channel 3 |
| [Details](http://mirdb.org/cgi-bin/target_detail.cgi?targetID=3072469) | 661 | 78 | hsa-miR-27a-3p | [HMGCS1](http://www.ncbi.nlm.nih.gov/entrez/query.fcgi?db=gene&cmd=Retrieve&dopt=full_report&list_uids=3157) | 3-hydroxy-3-methylglutaryl-CoA synthase 1 |
| [Details](http://mirdb.org/cgi-bin/target_detail.cgi?targetID=3072701) | 662 | 78 | hsa-miR-27a-3p | [NMB](http://www.ncbi.nlm.nih.gov/entrez/query.fcgi?db=gene&cmd=Retrieve&dopt=full_report&list_uids=4828) | neuromedin B |
| [Details](http://mirdb.org/cgi-bin/target_detail.cgi?targetID=3072728) | 663 | 78 | hsa-miR-27a-3p | [CDK14](http://www.ncbi.nlm.nih.gov/entrez/query.fcgi?db=gene&cmd=Retrieve&dopt=full_report&list_uids=5218) | cyclin dependent kinase 14 |
| [Details](http://mirdb.org/cgi-bin/target_detail.cgi?targetID=3072831) | 664 | 78 | hsa-miR-27a-3p | [FRYL](http://www.ncbi.nlm.nih.gov/entrez/query.fcgi?db=gene&cmd=Retrieve&dopt=full_report&list_uids=285527) | FRY like transcription coactivator |
| [Details](http://mirdb.org/cgi-bin/target_detail.cgi?targetID=3072895) | 665 | 78 | hsa-miR-27a-3p | [B9D1](http://www.ncbi.nlm.nih.gov/entrez/query.fcgi?db=gene&cmd=Retrieve&dopt=full_report&list_uids=27077) | B9 domain containing 1 |
| [Details](http://mirdb.org/cgi-bin/target_detail.cgi?targetID=3073035) | 666 | 78 | hsa-miR-27a-3p | [MFSD6](http://www.ncbi.nlm.nih.gov/entrez/query.fcgi?db=gene&cmd=Retrieve&dopt=full_report&list_uids=54842) | major facilitator superfamily domain containing 6 |
| [Details](http://mirdb.org/cgi-bin/target_detail.cgi?targetID=3073092) | 667 | 78 | hsa-miR-27a-3p | [MINPP1](http://www.ncbi.nlm.nih.gov/entrez/query.fcgi?db=gene&cmd=Retrieve&dopt=full_report&list_uids=9562) | multiple inositol-polyphosphate phosphatase 1 |
| [Details](http://mirdb.org/cgi-bin/target_detail.cgi?targetID=3073097) | 668 | 78 | hsa-miR-27a-3p | [BTBD11](http://www.ncbi.nlm.nih.gov/entrez/query.fcgi?db=gene&cmd=Retrieve&dopt=full_report&list_uids=121551) | BTB domain containing 11 |
| [Details](http://mirdb.org/cgi-bin/target_detail.cgi?targetID=3073146) | 669 | 78 | hsa-miR-27a-3p | [DMRT3](http://www.ncbi.nlm.nih.gov/entrez/query.fcgi?db=gene&cmd=Retrieve&dopt=full_report&list_uids=58524) | doublesex and mab-3 related transcription factor 3 |
| [Details](http://mirdb.org/cgi-bin/target_detail.cgi?targetID=3073151) | 670 | 78 | hsa-miR-27a-3p | [ZBTB44](http://www.ncbi.nlm.nih.gov/entrez/query.fcgi?db=gene&cmd=Retrieve&dopt=full_report&list_uids=29068) | zinc finger and BTB domain containing 44 |
| [Details](http://mirdb.org/cgi-bin/target_detail.cgi?targetID=3073175) | 671 | 78 | hsa-miR-27a-3p | [HOXA13](http://www.ncbi.nlm.nih.gov/entrez/query.fcgi?db=gene&cmd=Retrieve&dopt=full_report&list_uids=3209) | homeobox A13 |
| [Details](http://mirdb.org/cgi-bin/target_detail.cgi?targetID=3073270) | 672 | 78 | hsa-miR-27a-3p | [AEBP2](http://www.ncbi.nlm.nih.gov/entrez/query.fcgi?db=gene&cmd=Retrieve&dopt=full_report&list_uids=121536) | AE binding protein 2 |
| [Details](http://mirdb.org/cgi-bin/target_detail.cgi?targetID=3073313) | 673 | 78 | hsa-miR-27a-3p | [RAP1B](http://www.ncbi.nlm.nih.gov/entrez/query.fcgi?db=gene&cmd=Retrieve&dopt=full_report&list_uids=5908) | RAP1B, member of RAS oncogene family |
| [Details](http://mirdb.org/cgi-bin/target_detail.cgi?targetID=3073325) | 674 | 78 | hsa-miR-27a-3p | [CCDC149](http://www.ncbi.nlm.nih.gov/entrez/query.fcgi?db=gene&cmd=Retrieve&dopt=full_report&list_uids=91050) | coiled-coil domain containing 149 |
| [Details](http://mirdb.org/cgi-bin/target_detail.cgi?targetID=3073342) | 675 | 78 | hsa-miR-27a-3p | [AIDA](http://www.ncbi.nlm.nih.gov/entrez/query.fcgi?db=gene&cmd=Retrieve&dopt=full_report&list_uids=64853) | axin interactor, dorsalization associated |
| [Details](http://mirdb.org/cgi-bin/target_detail.cgi?targetID=3073381) | 676 | 78 | hsa-miR-27a-3p | [EPHB2](http://www.ncbi.nlm.nih.gov/entrez/query.fcgi?db=gene&cmd=Retrieve&dopt=full_report&list_uids=2048) | EPH receptor B2 |
| [Details](http://mirdb.org/cgi-bin/target_detail.cgi?targetID=3073396) | 677 | 78 | hsa-miR-27a-3p | [NHS](http://www.ncbi.nlm.nih.gov/entrez/query.fcgi?db=gene&cmd=Retrieve&dopt=full_report&list_uids=4810) | NHS actin remodeling regulator |
| [Details](http://mirdb.org/cgi-bin/target_detail.cgi?targetID=3073423) | 678 | 78 | hsa-miR-27a-3p | [PRTG](http://www.ncbi.nlm.nih.gov/entrez/query.fcgi?db=gene&cmd=Retrieve&dopt=full_report&list_uids=283659) | protogenin |
| [Details](http://mirdb.org/cgi-bin/target_detail.cgi?targetID=3073426) | 679 | 78 | hsa-miR-27a-3p | [ZNF230](http://www.ncbi.nlm.nih.gov/entrez/query.fcgi?db=gene&cmd=Retrieve&dopt=full_report&list_uids=7773) | zinc finger protein 230 |
| [Details](http://mirdb.org/cgi-bin/target_detail.cgi?targetID=3073512) | 680 | 78 | hsa-miR-27a-3p | [LIN28B](http://www.ncbi.nlm.nih.gov/entrez/query.fcgi?db=gene&cmd=Retrieve&dopt=full_report&list_uids=389421) | lin-28 homolog B |
| [Details](http://mirdb.org/cgi-bin/target_detail.cgi?targetID=3073518) | 681 | 78 | hsa-miR-27a-3p | [TAOK1](http://www.ncbi.nlm.nih.gov/entrez/query.fcgi?db=gene&cmd=Retrieve&dopt=full_report&list_uids=57551) | TAO kinase 1 |
| [Details](http://mirdb.org/cgi-bin/target_detail.cgi?targetID=3073578) | 682 | 78 | hsa-miR-27a-3p | [AP1G1](http://www.ncbi.nlm.nih.gov/entrez/query.fcgi?db=gene&cmd=Retrieve&dopt=full_report&list_uids=164) | adaptor related protein complex 1 subunit gamma 1 |
| [Details](http://mirdb.org/cgi-bin/target_detail.cgi?targetID=3073596) | 683 | 78 | hsa-miR-27a-3p | [FAM84B](http://www.ncbi.nlm.nih.gov/entrez/query.fcgi?db=gene&cmd=Retrieve&dopt=full_report&list_uids=157638) | family with sequence similarity 84 member B |
| [Details](http://mirdb.org/cgi-bin/target_detail.cgi?targetID=3073653) | 684 | 78 | hsa-miR-27a-3p | [SSBP2](http://www.ncbi.nlm.nih.gov/entrez/query.fcgi?db=gene&cmd=Retrieve&dopt=full_report&list_uids=23635) | single stranded DNA binding protein 2 |
| [Details](http://mirdb.org/cgi-bin/target_detail.cgi?targetID=3073670) | 685 | 78 | hsa-miR-27a-3p | [CSRNP1](http://www.ncbi.nlm.nih.gov/entrez/query.fcgi?db=gene&cmd=Retrieve&dopt=full_report&list_uids=64651) | cysteine and serine rich nuclear protein 1 |
| [Details](http://mirdb.org/cgi-bin/target_detail.cgi?targetID=3073681) | 686 | 78 | hsa-miR-27a-3p | [CBFB](http://www.ncbi.nlm.nih.gov/entrez/query.fcgi?db=gene&cmd=Retrieve&dopt=full_report&list_uids=865) | core-binding factor subunit beta |
| [Details](http://mirdb.org/cgi-bin/target_detail.cgi?targetID=3073855) | 687 | 78 | hsa-miR-27a-3p | [SMPD3](http://www.ncbi.nlm.nih.gov/entrez/query.fcgi?db=gene&cmd=Retrieve&dopt=full_report&list_uids=55512) | sphingomyelin phosphodiesterase 3 |
| [Details](http://mirdb.org/cgi-bin/target_detail.cgi?targetID=3072453) | 688 | 77 | hsa-miR-27a-3p | [KIAA1147](http://www.ncbi.nlm.nih.gov/entrez/query.fcgi?db=gene&cmd=Retrieve&dopt=full_report&list_uids=57189) | KIAA1147 |
| [Details](http://mirdb.org/cgi-bin/target_detail.cgi?targetID=3072481) | 689 | 77 | hsa-miR-27a-3p | [EIF5](http://www.ncbi.nlm.nih.gov/entrez/query.fcgi?db=gene&cmd=Retrieve&dopt=full_report&list_uids=1983) | eukaryotic translation initiation factor 5 |
| [Details](http://mirdb.org/cgi-bin/target_detail.cgi?targetID=3072540) | 690 | 77 | hsa-miR-27a-3p | [SH3RF1](http://www.ncbi.nlm.nih.gov/entrez/query.fcgi?db=gene&cmd=Retrieve&dopt=full_report&list_uids=57630) | SH3 domain containing ring finger 1 |
| [Details](http://mirdb.org/cgi-bin/target_detail.cgi?targetID=3072550) | 691 | 77 | hsa-miR-27a-3p | [GFPT1](http://www.ncbi.nlm.nih.gov/entrez/query.fcgi?db=gene&cmd=Retrieve&dopt=full_report&list_uids=2673) | glutamine--fructose-6-phosphate transaminase 1 |
| [Details](http://mirdb.org/cgi-bin/target_detail.cgi?targetID=3072615) | 692 | 77 | hsa-miR-27a-3p | [SEC22A](http://www.ncbi.nlm.nih.gov/entrez/query.fcgi?db=gene&cmd=Retrieve&dopt=full_report&list_uids=26984) | SEC22 homolog A, vesicle trafficking protein |
| [Details](http://mirdb.org/cgi-bin/target_detail.cgi?targetID=3072797) | 693 | 77 | hsa-miR-27a-3p | [RHOT1](http://www.ncbi.nlm.nih.gov/entrez/query.fcgi?db=gene&cmd=Retrieve&dopt=full_report&list_uids=55288) | ras homolog family member T1 |
| [Details](http://mirdb.org/cgi-bin/target_detail.cgi?targetID=3072866) | 694 | 77 | hsa-miR-27a-3p | [DGKG](http://www.ncbi.nlm.nih.gov/entrez/query.fcgi?db=gene&cmd=Retrieve&dopt=full_report&list_uids=1608) | diacylglycerol kinase gamma |
| [Details](http://mirdb.org/cgi-bin/target_detail.cgi?targetID=3072934) | 695 | 77 | hsa-miR-27a-3p | [LBR](http://www.ncbi.nlm.nih.gov/entrez/query.fcgi?db=gene&cmd=Retrieve&dopt=full_report&list_uids=3930) | lamin B receptor |
| [Details](http://mirdb.org/cgi-bin/target_detail.cgi?targetID=3072937) | 696 | 77 | hsa-miR-27a-3p | [SPATA16](http://www.ncbi.nlm.nih.gov/entrez/query.fcgi?db=gene&cmd=Retrieve&dopt=full_report&list_uids=83893) | spermatogenesis associated 16 |
| [Details](http://mirdb.org/cgi-bin/target_detail.cgi?targetID=3073026) | 697 | 77 | hsa-miR-27a-3p | [RAB14](http://www.ncbi.nlm.nih.gov/entrez/query.fcgi?db=gene&cmd=Retrieve&dopt=full_report&list_uids=51552) | RAB14, member RAS oncogene family |
| [Details](http://mirdb.org/cgi-bin/target_detail.cgi?targetID=3073061) | 698 | 77 | hsa-miR-27a-3p | [FAM98A](http://www.ncbi.nlm.nih.gov/entrez/query.fcgi?db=gene&cmd=Retrieve&dopt=full_report&list_uids=25940) | family with sequence similarity 98 member A |
| [Details](http://mirdb.org/cgi-bin/target_detail.cgi?targetID=3073079) | 699 | 77 | hsa-miR-27a-3p | [MOSMO](http://www.ncbi.nlm.nih.gov/entrez/query.fcgi?db=gene&cmd=Retrieve&dopt=full_report&list_uids=730094) | modulator of smoothened |
| [Details](http://mirdb.org/cgi-bin/target_detail.cgi?targetID=3073115) | 700 | 77 | hsa-miR-27a-3p | [INPP4A](http://www.ncbi.nlm.nih.gov/entrez/query.fcgi?db=gene&cmd=Retrieve&dopt=full_report&list_uids=3631) | inositol polyphosphate-4-phosphatase type I A |
| [Details](http://mirdb.org/cgi-bin/target_detail.cgi?targetID=3073126) | 701 | 77 | hsa-miR-27a-3p | [CDC42BPB](http://www.ncbi.nlm.nih.gov/entrez/query.fcgi?db=gene&cmd=Retrieve&dopt=full_report&list_uids=9578) | CDC42 binding protein kinase beta |
| [Details](http://mirdb.org/cgi-bin/target_detail.cgi?targetID=3073133) | 702 | 77 | hsa-miR-27a-3p | [SEPT8](http://www.ncbi.nlm.nih.gov/entrez/query.fcgi?db=gene&cmd=Retrieve&dopt=full_report&list_uids=23176) | septin 8 |
| [Details](http://mirdb.org/cgi-bin/target_detail.cgi?targetID=3073145) | 703 | 77 | hsa-miR-27a-3p | [MPP6](http://www.ncbi.nlm.nih.gov/entrez/query.fcgi?db=gene&cmd=Retrieve&dopt=full_report&list_uids=51678) | membrane palmitoylated protein 6 |
| [Details](http://mirdb.org/cgi-bin/target_detail.cgi?targetID=3073158) | 704 | 77 | hsa-miR-27a-3p | [MAP3K14](http://www.ncbi.nlm.nih.gov/entrez/query.fcgi?db=gene&cmd=Retrieve&dopt=full_report&list_uids=9020) | mitogen-activated protein kinase kinase kinase 14 |
| [Details](http://mirdb.org/cgi-bin/target_detail.cgi?targetID=3073196) | 705 | 77 | hsa-miR-27a-3p | [CNTLN](http://www.ncbi.nlm.nih.gov/entrez/query.fcgi?db=gene&cmd=Retrieve&dopt=full_report&list_uids=54875) | centlein |
| [Details](http://mirdb.org/cgi-bin/target_detail.cgi?targetID=3073251) | 706 | 77 | hsa-miR-27a-3p | [HDX](http://www.ncbi.nlm.nih.gov/entrez/query.fcgi?db=gene&cmd=Retrieve&dopt=full_report&list_uids=139324) | highly divergent homeobox |
| [Details](http://mirdb.org/cgi-bin/target_detail.cgi?targetID=3073307) | 707 | 77 | hsa-miR-27a-3p | [BMI1](http://www.ncbi.nlm.nih.gov/entrez/query.fcgi?db=gene&cmd=Retrieve&dopt=full_report&list_uids=648) | BMI1 proto-oncogene, polycomb ring finger |
| [Details](http://mirdb.org/cgi-bin/target_detail.cgi?targetID=3073330) | 708 | 77 | hsa-miR-27a-3p | [EFHD2](http://www.ncbi.nlm.nih.gov/entrez/query.fcgi?db=gene&cmd=Retrieve&dopt=full_report&list_uids=79180) | EF-hand domain family member D2 |
| [Details](http://mirdb.org/cgi-bin/target_detail.cgi?targetID=3073343) | 709 | 77 | hsa-miR-27a-3p | [ASH2L](http://www.ncbi.nlm.nih.gov/entrez/query.fcgi?db=gene&cmd=Retrieve&dopt=full_report&list_uids=9070) | ASH2 like, histone lysine methyltransferase complex subunit |
| [Details](http://mirdb.org/cgi-bin/target_detail.cgi?targetID=3073358) | 710 | 77 | hsa-miR-27a-3p | [MDFI](http://www.ncbi.nlm.nih.gov/entrez/query.fcgi?db=gene&cmd=Retrieve&dopt=full_report&list_uids=4188) | MyoD family inhibitor |
| [Details](http://mirdb.org/cgi-bin/target_detail.cgi?targetID=3073420) | 711 | 77 | hsa-miR-27a-3p | [ICOS](http://www.ncbi.nlm.nih.gov/entrez/query.fcgi?db=gene&cmd=Retrieve&dopt=full_report&list_uids=29851) | inducible T cell costimulator |
| [Details](http://mirdb.org/cgi-bin/target_detail.cgi?targetID=3073443) | 712 | 77 | hsa-miR-27a-3p | [DPY19L4](http://www.ncbi.nlm.nih.gov/entrez/query.fcgi?db=gene&cmd=Retrieve&dopt=full_report&list_uids=286148) | dpy-19 like 4 |
| [Details](http://mirdb.org/cgi-bin/target_detail.cgi?targetID=3073463) | 713 | 77 | hsa-miR-27a-3p | [STRBP](http://www.ncbi.nlm.nih.gov/entrez/query.fcgi?db=gene&cmd=Retrieve&dopt=full_report&list_uids=55342) | spermatid perinuclear RNA binding protein |
| [Details](http://mirdb.org/cgi-bin/target_detail.cgi?targetID=3073516) | 714 | 77 | hsa-miR-27a-3p | [MEPCE](http://www.ncbi.nlm.nih.gov/entrez/query.fcgi?db=gene&cmd=Retrieve&dopt=full_report&list_uids=56257) | methylphosphate capping enzyme |
| [Details](http://mirdb.org/cgi-bin/target_detail.cgi?targetID=3073526) | 715 | 77 | hsa-miR-27a-3p | [DCX](http://www.ncbi.nlm.nih.gov/entrez/query.fcgi?db=gene&cmd=Retrieve&dopt=full_report&list_uids=1641) | doublecortin |
| [Details](http://mirdb.org/cgi-bin/target_detail.cgi?targetID=3073558) | 716 | 77 | hsa-miR-27a-3p | [GNPNAT1](http://www.ncbi.nlm.nih.gov/entrez/query.fcgi?db=gene&cmd=Retrieve&dopt=full_report&list_uids=64841) | glucosamine-phosphate N-acetyltransferase 1 |
| [Details](http://mirdb.org/cgi-bin/target_detail.cgi?targetID=3073581) | 717 | 77 | hsa-miR-27a-3p | [SLC16A10](http://www.ncbi.nlm.nih.gov/entrez/query.fcgi?db=gene&cmd=Retrieve&dopt=full_report&list_uids=117247) | solute carrier family 16 member 10 |
| [Details](http://mirdb.org/cgi-bin/target_detail.cgi?targetID=3073657) | 718 | 77 | hsa-miR-27a-3p | [ZNF780B](http://www.ncbi.nlm.nih.gov/entrez/query.fcgi?db=gene&cmd=Retrieve&dopt=full_report&list_uids=163131) | zinc finger protein 780B |
| [Details](http://mirdb.org/cgi-bin/target_detail.cgi?targetID=3073709) | 719 | 77 | hsa-miR-27a-3p | [FEM1B](http://www.ncbi.nlm.nih.gov/entrez/query.fcgi?db=gene&cmd=Retrieve&dopt=full_report&list_uids=10116) | fem-1 homolog B |
| [Details](http://mirdb.org/cgi-bin/target_detail.cgi?targetID=3073766) | 720 | 77 | hsa-miR-27a-3p | [STOX2](http://www.ncbi.nlm.nih.gov/entrez/query.fcgi?db=gene&cmd=Retrieve&dopt=full_report&list_uids=56977) | storkhead box 2 |
| [Details](http://mirdb.org/cgi-bin/target_detail.cgi?targetID=3073812) | 721 | 77 | hsa-miR-27a-3p | [FAM199X](http://www.ncbi.nlm.nih.gov/entrez/query.fcgi?db=gene&cmd=Retrieve&dopt=full_report&list_uids=139231) | family with sequence similarity 199, X-linked |
| [Details](http://mirdb.org/cgi-bin/target_detail.cgi?targetID=3073842) | 722 | 77 | hsa-miR-27a-3p | [TMEM248](http://www.ncbi.nlm.nih.gov/entrez/query.fcgi?db=gene&cmd=Retrieve&dopt=full_report&list_uids=55069) | transmembrane protein 248 |
| [Details](http://mirdb.org/cgi-bin/target_detail.cgi?targetID=3072444) | 723 | 76 | hsa-miR-27a-3p | [AGGF1](http://www.ncbi.nlm.nih.gov/entrez/query.fcgi?db=gene&cmd=Retrieve&dopt=full_report&list_uids=55109) | angiogenic factor with G-patch and FHA domains 1 |
| [Details](http://mirdb.org/cgi-bin/target_detail.cgi?targetID=3072462) | 724 | 76 | hsa-miR-27a-3p | [SLC35F3](http://www.ncbi.nlm.nih.gov/entrez/query.fcgi?db=gene&cmd=Retrieve&dopt=full_report&list_uids=148641) | solute carrier family 35 member F3 |
| [Details](http://mirdb.org/cgi-bin/target_detail.cgi?targetID=3072504) | 725 | 76 | hsa-miR-27a-3p | [ANKRD40](http://www.ncbi.nlm.nih.gov/entrez/query.fcgi?db=gene&cmd=Retrieve&dopt=full_report&list_uids=91369) | ankyrin repeat domain 40 |
| [Details](http://mirdb.org/cgi-bin/target_detail.cgi?targetID=3072571) | 726 | 76 | hsa-miR-27a-3p | [SERP1](http://www.ncbi.nlm.nih.gov/entrez/query.fcgi?db=gene&cmd=Retrieve&dopt=full_report&list_uids=27230) | stress associated endoplasmic reticulum protein 1 |
| [Details](http://mirdb.org/cgi-bin/target_detail.cgi?targetID=3072607) | 727 | 76 | hsa-miR-27a-3p | [RSAD2](http://www.ncbi.nlm.nih.gov/entrez/query.fcgi?db=gene&cmd=Retrieve&dopt=full_report&list_uids=91543) | radical S-adenosyl methionine domain containing 2 |
| [Details](http://mirdb.org/cgi-bin/target_detail.cgi?targetID=3072733) | 728 | 76 | hsa-miR-27a-3p | [ITGA2](http://www.ncbi.nlm.nih.gov/entrez/query.fcgi?db=gene&cmd=Retrieve&dopt=full_report&list_uids=3673) | integrin subunit alpha 2 |
| [Details](http://mirdb.org/cgi-bin/target_detail.cgi?targetID=3072761) | 729 | 76 | hsa-miR-27a-3p | [IGF2BP3](http://www.ncbi.nlm.nih.gov/entrez/query.fcgi?db=gene&cmd=Retrieve&dopt=full_report&list_uids=10643) | insulin like growth factor 2 mRNA binding protein 3 |
| [Details](http://mirdb.org/cgi-bin/target_detail.cgi?targetID=3072799) | 730 | 76 | hsa-miR-27a-3p | [PGM2L1](http://www.ncbi.nlm.nih.gov/entrez/query.fcgi?db=gene&cmd=Retrieve&dopt=full_report&list_uids=283209) | phosphoglucomutase 2 like 1 |
| [Details](http://mirdb.org/cgi-bin/target_detail.cgi?targetID=3072802) | 731 | 76 | hsa-miR-27a-3p | [ABAT](http://www.ncbi.nlm.nih.gov/entrez/query.fcgi?db=gene&cmd=Retrieve&dopt=full_report&list_uids=18) | 4-aminobutyrate aminotransferase |
| [Details](http://mirdb.org/cgi-bin/target_detail.cgi?targetID=3072823) | 732 | 76 | hsa-miR-27a-3p | [VAT1L](http://www.ncbi.nlm.nih.gov/entrez/query.fcgi?db=gene&cmd=Retrieve&dopt=full_report&list_uids=57687) | vesicle amine transport 1 like |
| [Details](http://mirdb.org/cgi-bin/target_detail.cgi?targetID=3072828) | 733 | 76 | hsa-miR-27a-3p | [VPS13A](http://www.ncbi.nlm.nih.gov/entrez/query.fcgi?db=gene&cmd=Retrieve&dopt=full_report&list_uids=23230) | vacuolar protein sorting 13 homolog A |
| [Details](http://mirdb.org/cgi-bin/target_detail.cgi?targetID=3072830) | 734 | 76 | hsa-miR-27a-3p | [BRSK1](http://www.ncbi.nlm.nih.gov/entrez/query.fcgi?db=gene&cmd=Retrieve&dopt=full_report&list_uids=84446) | BR serine/threonine kinase 1 |
| [Details](http://mirdb.org/cgi-bin/target_detail.cgi?targetID=3072918) | 735 | 76 | hsa-miR-27a-3p | [WNT3A](http://www.ncbi.nlm.nih.gov/entrez/query.fcgi?db=gene&cmd=Retrieve&dopt=full_report&list_uids=89780) | Wnt family member 3A |
| [Details](http://mirdb.org/cgi-bin/target_detail.cgi?targetID=3072965) | 736 | 76 | hsa-miR-27a-3p | [C1orf21](http://www.ncbi.nlm.nih.gov/entrez/query.fcgi?db=gene&cmd=Retrieve&dopt=full_report&list_uids=81563) | chromosome 1 open reading frame 21 |
| [Details](http://mirdb.org/cgi-bin/target_detail.cgi?targetID=3072976) | 737 | 76 | hsa-miR-27a-3p | [LCN8](http://www.ncbi.nlm.nih.gov/entrez/query.fcgi?db=gene&cmd=Retrieve&dopt=full_report&list_uids=138307) | lipocalin 8 |
| [Details](http://mirdb.org/cgi-bin/target_detail.cgi?targetID=3073063) | 738 | 76 | hsa-miR-27a-3p | [BCL7A](http://www.ncbi.nlm.nih.gov/entrez/query.fcgi?db=gene&cmd=Retrieve&dopt=full_report&list_uids=605) | BCL7A, BAF complex component |
| [Details](http://mirdb.org/cgi-bin/target_detail.cgi?targetID=3073140) | 739 | 76 | hsa-miR-27a-3p | [PDGFRA](http://www.ncbi.nlm.nih.gov/entrez/query.fcgi?db=gene&cmd=Retrieve&dopt=full_report&list_uids=5156) | platelet derived growth factor receptor alpha |
| [Details](http://mirdb.org/cgi-bin/target_detail.cgi?targetID=3073152) | 740 | 76 | hsa-miR-27a-3p | [AGPAT3](http://www.ncbi.nlm.nih.gov/entrez/query.fcgi?db=gene&cmd=Retrieve&dopt=full_report&list_uids=56894) | 1-acylglycerol-3-phosphate O-acyltransferase 3 |
| [Details](http://mirdb.org/cgi-bin/target_detail.cgi?targetID=3073168) | 741 | 76 | hsa-miR-27a-3p | [EBF3](http://www.ncbi.nlm.nih.gov/entrez/query.fcgi?db=gene&cmd=Retrieve&dopt=full_report&list_uids=253738) | EBF transcription factor 3 |
| [Details](http://mirdb.org/cgi-bin/target_detail.cgi?targetID=3073356) | 742 | 76 | hsa-miR-27a-3p | [HOXC11](http://www.ncbi.nlm.nih.gov/entrez/query.fcgi?db=gene&cmd=Retrieve&dopt=full_report&list_uids=3227) | homeobox C11 |
| [Details](http://mirdb.org/cgi-bin/target_detail.cgi?targetID=3073521) | 743 | 76 | hsa-miR-27a-3p | [ZEB2](http://www.ncbi.nlm.nih.gov/entrez/query.fcgi?db=gene&cmd=Retrieve&dopt=full_report&list_uids=9839) | zinc finger E-box binding homeobox 2 |
| [Details](http://mirdb.org/cgi-bin/target_detail.cgi?targetID=3073616) | 744 | 76 | hsa-miR-27a-3p | [FAM193B](http://www.ncbi.nlm.nih.gov/entrez/query.fcgi?db=gene&cmd=Retrieve&dopt=full_report&list_uids=54540) | family with sequence similarity 193 member B |
| [Details](http://mirdb.org/cgi-bin/target_detail.cgi?targetID=3073617) | 745 | 76 | hsa-miR-27a-3p | [RAP2B](http://www.ncbi.nlm.nih.gov/entrez/query.fcgi?db=gene&cmd=Retrieve&dopt=full_report&list_uids=5912) | RAP2B, member of RAS oncogene family |
| [Details](http://mirdb.org/cgi-bin/target_detail.cgi?targetID=3073638) | 746 | 76 | hsa-miR-27a-3p | [CXADR](http://www.ncbi.nlm.nih.gov/entrez/query.fcgi?db=gene&cmd=Retrieve&dopt=full_report&list_uids=1525) | CXADR, Ig-like cell adhesion molecule |
| [Details](http://mirdb.org/cgi-bin/target_detail.cgi?targetID=3073758) | 747 | 76 | hsa-miR-27a-3p | [ZNF148](http://www.ncbi.nlm.nih.gov/entrez/query.fcgi?db=gene&cmd=Retrieve&dopt=full_report&list_uids=7707) | zinc finger protein 148 |
| [Details](http://mirdb.org/cgi-bin/target_detail.cgi?targetID=3073799) | 748 | 76 | hsa-miR-27a-3p | [UBN2](http://www.ncbi.nlm.nih.gov/entrez/query.fcgi?db=gene&cmd=Retrieve&dopt=full_report&list_uids=254048) | ubinuclein 2 |
| [Details](http://mirdb.org/cgi-bin/target_detail.cgi?targetID=3073835) | 749 | 76 | hsa-miR-27a-3p | [CPEB3](http://www.ncbi.nlm.nih.gov/entrez/query.fcgi?db=gene&cmd=Retrieve&dopt=full_report&list_uids=22849) | cytoplasmic polyadenylation element binding protein 3 |
| [Details](http://mirdb.org/cgi-bin/target_detail.cgi?targetID=3073911) | 750 | 76 | hsa-miR-27a-3p | [IKZF1](http://www.ncbi.nlm.nih.gov/entrez/query.fcgi?db=gene&cmd=Retrieve&dopt=full_report&list_uids=10320) | IKAROS family zinc finger 1 |
| [Details](http://mirdb.org/cgi-bin/target_detail.cgi?targetID=3073912) | 751 | 76 | hsa-miR-27a-3p | [ANKRD36B](http://www.ncbi.nlm.nih.gov/entrez/query.fcgi?db=gene&cmd=Retrieve&dopt=full_report&list_uids=57730) | ankyrin repeat domain 36B |
| [Details](http://mirdb.org/cgi-bin/target_detail.cgi?targetID=3072468) | 752 | 75 | hsa-miR-27a-3p | [RBBP5](http://www.ncbi.nlm.nih.gov/entrez/query.fcgi?db=gene&cmd=Retrieve&dopt=full_report&list_uids=5929) | RB binding protein 5, histone lysine methyltransferase complex subunit |
| [Details](http://mirdb.org/cgi-bin/target_detail.cgi?targetID=3072748) | 753 | 75 | hsa-miR-27a-3p | [TBC1D4](http://www.ncbi.nlm.nih.gov/entrez/query.fcgi?db=gene&cmd=Retrieve&dopt=full_report&list_uids=9882) | TBC1 domain family member 4 |
| [Details](http://mirdb.org/cgi-bin/target_detail.cgi?targetID=3072784) | 754 | 75 | hsa-miR-27a-3p | [PTCHD1](http://www.ncbi.nlm.nih.gov/entrez/query.fcgi?db=gene&cmd=Retrieve&dopt=full_report&list_uids=139411) | patched domain containing 1 |
| [Details](http://mirdb.org/cgi-bin/target_detail.cgi?targetID=3072809) | 755 | 75 | hsa-miR-27a-3p | [EML1](http://www.ncbi.nlm.nih.gov/entrez/query.fcgi?db=gene&cmd=Retrieve&dopt=full_report&list_uids=2009) | EMAP like 1 |
| [Details](http://mirdb.org/cgi-bin/target_detail.cgi?targetID=3072810) | 756 | 75 | hsa-miR-27a-3p | [ABHD5](http://www.ncbi.nlm.nih.gov/entrez/query.fcgi?db=gene&cmd=Retrieve&dopt=full_report&list_uids=51099) | abhydrolase domain containing 5 |
| [Details](http://mirdb.org/cgi-bin/target_detail.cgi?targetID=3072814) | 757 | 75 | hsa-miR-27a-3p | [ALKAL1](http://www.ncbi.nlm.nih.gov/entrez/query.fcgi?db=gene&cmd=Retrieve&dopt=full_report&list_uids=389658) | ALK and LTK ligand 1 |
| [Details](http://mirdb.org/cgi-bin/target_detail.cgi?targetID=3072978) | 758 | 75 | hsa-miR-27a-3p | [BEND3](http://www.ncbi.nlm.nih.gov/entrez/query.fcgi?db=gene&cmd=Retrieve&dopt=full_report&list_uids=57673) | BEN domain containing 3 |
| [Details](http://mirdb.org/cgi-bin/target_detail.cgi?targetID=3072991) | 759 | 75 | hsa-miR-27a-3p | [CAB39](http://www.ncbi.nlm.nih.gov/entrez/query.fcgi?db=gene&cmd=Retrieve&dopt=full_report&list_uids=51719) | calcium binding protein 39 |
| [Details](http://mirdb.org/cgi-bin/target_detail.cgi?targetID=3072994) | 760 | 75 | hsa-miR-27a-3p | [STXBP6](http://www.ncbi.nlm.nih.gov/entrez/query.fcgi?db=gene&cmd=Retrieve&dopt=full_report&list_uids=29091) | syntaxin binding protein 6 |
| [Details](http://mirdb.org/cgi-bin/target_detail.cgi?targetID=3073014) | 761 | 75 | hsa-miR-27a-3p | [ENOX1](http://www.ncbi.nlm.nih.gov/entrez/query.fcgi?db=gene&cmd=Retrieve&dopt=full_report&list_uids=55068) | ecto-NOX disulfide-thiol exchanger 1 |
| [Details](http://mirdb.org/cgi-bin/target_detail.cgi?targetID=3073187) | 762 | 75 | hsa-miR-27a-3p | [C2CD4A](http://www.ncbi.nlm.nih.gov/entrez/query.fcgi?db=gene&cmd=Retrieve&dopt=full_report&list_uids=145741) | C2 calcium dependent domain containing 4A |
| [Details](http://mirdb.org/cgi-bin/target_detail.cgi?targetID=3073264) | 763 | 75 | hsa-miR-27a-3p | [PRR9](http://www.ncbi.nlm.nih.gov/entrez/query.fcgi?db=gene&cmd=Retrieve&dopt=full_report&list_uids=574414) | proline rich 9 |
| [Details](http://mirdb.org/cgi-bin/target_detail.cgi?targetID=3073337) | 764 | 75 | hsa-miR-27a-3p | [NEBL](http://www.ncbi.nlm.nih.gov/entrez/query.fcgi?db=gene&cmd=Retrieve&dopt=full_report&list_uids=10529) | nebulette |
| [Details](http://mirdb.org/cgi-bin/target_detail.cgi?targetID=3073348) | 765 | 75 | hsa-miR-27a-3p | [LRBA](http://www.ncbi.nlm.nih.gov/entrez/query.fcgi?db=gene&cmd=Retrieve&dopt=full_report&list_uids=987) | LPS responsive beige-like anchor protein |
| [Details](http://mirdb.org/cgi-bin/target_detail.cgi?targetID=3073398) | 766 | 75 | hsa-miR-27a-3p | [PRPSAP2](http://www.ncbi.nlm.nih.gov/entrez/query.fcgi?db=gene&cmd=Retrieve&dopt=full_report&list_uids=5636) | phosphoribosyl pyrophosphate synthetase associated protein 2 |
| [Details](http://mirdb.org/cgi-bin/target_detail.cgi?targetID=3073568) | 767 | 75 | hsa-miR-27a-3p | [LMLN](http://www.ncbi.nlm.nih.gov/entrez/query.fcgi?db=gene&cmd=Retrieve&dopt=full_report&list_uids=89782) | leishmanolysin like peptidase |
| [Details](http://mirdb.org/cgi-bin/target_detail.cgi?targetID=3073582) | 768 | 75 | hsa-miR-27a-3p | [ADAM19](http://www.ncbi.nlm.nih.gov/entrez/query.fcgi?db=gene&cmd=Retrieve&dopt=full_report&list_uids=8728) | ADAM metallopeptidase domain 19 |
| [Details](http://mirdb.org/cgi-bin/target_detail.cgi?targetID=3073622) | 769 | 75 | hsa-miR-27a-3p | [VEGFC](http://www.ncbi.nlm.nih.gov/entrez/query.fcgi?db=gene&cmd=Retrieve&dopt=full_report&list_uids=7424) | vascular endothelial growth factor C |
| [Details](http://mirdb.org/cgi-bin/target_detail.cgi?targetID=3073737) | 770 | 75 | hsa-miR-27a-3p | [KIF3A](http://www.ncbi.nlm.nih.gov/entrez/query.fcgi?db=gene&cmd=Retrieve&dopt=full_report&list_uids=11127) | kinesin family member 3A |
| [Details](http://mirdb.org/cgi-bin/target_detail.cgi?targetID=3073755) | 771 | 75 | hsa-miR-27a-3p | [PCDH7](http://www.ncbi.nlm.nih.gov/entrez/query.fcgi?db=gene&cmd=Retrieve&dopt=full_report&list_uids=5099) | protocadherin 7 |
| [Details](http://mirdb.org/cgi-bin/target_detail.cgi?targetID=3073809) | 772 | 75 | hsa-miR-27a-3p | [BAZ2B](http://www.ncbi.nlm.nih.gov/entrez/query.fcgi?db=gene&cmd=Retrieve&dopt=full_report&list_uids=29994) | bromodomain adjacent to zinc finger domain 2B |
| [Details](http://mirdb.org/cgi-bin/target_detail.cgi?targetID=3072509) | 773 | 74 | hsa-miR-27a-3p | [TMEM56](http://www.ncbi.nlm.nih.gov/entrez/query.fcgi?db=gene&cmd=Retrieve&dopt=full_report&list_uids=148534) | transmembrane protein 56 |
| [Details](http://mirdb.org/cgi-bin/target_detail.cgi?targetID=3072608) | 774 | 74 | hsa-miR-27a-3p | [NFIL3](http://www.ncbi.nlm.nih.gov/entrez/query.fcgi?db=gene&cmd=Retrieve&dopt=full_report&list_uids=4783) | nuclear factor, interleukin 3 regulated |
| [Details](http://mirdb.org/cgi-bin/target_detail.cgi?targetID=3072627) | 775 | 74 | hsa-miR-27a-3p | [USP51](http://www.ncbi.nlm.nih.gov/entrez/query.fcgi?db=gene&cmd=Retrieve&dopt=full_report&list_uids=158880) | ubiquitin specific peptidase 51 |
| [Details](http://mirdb.org/cgi-bin/target_detail.cgi?targetID=3072745) | 776 | 74 | hsa-miR-27a-3p | [TMEM50A](http://www.ncbi.nlm.nih.gov/entrez/query.fcgi?db=gene&cmd=Retrieve&dopt=full_report&list_uids=23585) | transmembrane protein 50A |
| [Details](http://mirdb.org/cgi-bin/target_detail.cgi?targetID=3072783) | 777 | 74 | hsa-miR-27a-3p | [EPB41L1](http://www.ncbi.nlm.nih.gov/entrez/query.fcgi?db=gene&cmd=Retrieve&dopt=full_report&list_uids=2036) | erythrocyte membrane protein band 4.1 like 1 |
| [Details](http://mirdb.org/cgi-bin/target_detail.cgi?targetID=3072862) | 778 | 74 | hsa-miR-27a-3p | [COX6C](http://www.ncbi.nlm.nih.gov/entrez/query.fcgi?db=gene&cmd=Retrieve&dopt=full_report&list_uids=1345) | cytochrome c oxidase subunit 6C |
| [Details](http://mirdb.org/cgi-bin/target_detail.cgi?targetID=3072894) | 779 | 74 | hsa-miR-27a-3p | [COLQ](http://www.ncbi.nlm.nih.gov/entrez/query.fcgi?db=gene&cmd=Retrieve&dopt=full_report&list_uids=8292) | collagen like tail subunit of asymmetric acetylcholinesterase |
| [Details](http://mirdb.org/cgi-bin/target_detail.cgi?targetID=3072942) | 780 | 74 | hsa-miR-27a-3p | [VANGL1](http://www.ncbi.nlm.nih.gov/entrez/query.fcgi?db=gene&cmd=Retrieve&dopt=full_report&list_uids=81839) | VANGL planar cell polarity protein 1 |
| [Details](http://mirdb.org/cgi-bin/target_detail.cgi?targetID=3072948) | 781 | 74 | hsa-miR-27a-3p | [ZBTB18](http://www.ncbi.nlm.nih.gov/entrez/query.fcgi?db=gene&cmd=Retrieve&dopt=full_report&list_uids=10472) | zinc finger and BTB domain containing 18 |
| [Details](http://mirdb.org/cgi-bin/target_detail.cgi?targetID=3073017) | 782 | 74 | hsa-miR-27a-3p | [LHFPL6](http://www.ncbi.nlm.nih.gov/entrez/query.fcgi?db=gene&cmd=Retrieve&dopt=full_report&list_uids=10186) | LHFPL tetraspan subfamily member 6 |
| [Details](http://mirdb.org/cgi-bin/target_detail.cgi?targetID=3073018) | 783 | 74 | hsa-miR-27a-3p | [DIS3](http://www.ncbi.nlm.nih.gov/entrez/query.fcgi?db=gene&cmd=Retrieve&dopt=full_report&list_uids=22894) | DIS3 homolog, exosome endoribonuclease and 3'-5' exoribonuclease |
| [Details](http://mirdb.org/cgi-bin/target_detail.cgi?targetID=3073060) | 784 | 74 | hsa-miR-27a-3p | [NEO1](http://www.ncbi.nlm.nih.gov/entrez/query.fcgi?db=gene&cmd=Retrieve&dopt=full_report&list_uids=4756) | neogenin 1 |
| [Details](http://mirdb.org/cgi-bin/target_detail.cgi?targetID=3073071) | 785 | 74 | hsa-miR-27a-3p | [ZFP1](http://www.ncbi.nlm.nih.gov/entrez/query.fcgi?db=gene&cmd=Retrieve&dopt=full_report&list_uids=162239) | ZFP1 zinc finger protein |
| [Details](http://mirdb.org/cgi-bin/target_detail.cgi?targetID=3073164) | 786 | 74 | hsa-miR-27a-3p | [NFE2L2](http://www.ncbi.nlm.nih.gov/entrez/query.fcgi?db=gene&cmd=Retrieve&dopt=full_report&list_uids=4780) | nuclear factor, erythroid 2 like 2 |
| [Details](http://mirdb.org/cgi-bin/target_detail.cgi?targetID=3073173) | 787 | 74 | hsa-miR-27a-3p | [APBA2](http://www.ncbi.nlm.nih.gov/entrez/query.fcgi?db=gene&cmd=Retrieve&dopt=full_report&list_uids=321) | amyloid beta precursor protein binding family A member 2 |
| [Details](http://mirdb.org/cgi-bin/target_detail.cgi?targetID=3073202) | 788 | 74 | hsa-miR-27a-3p | [ADORA1](http://www.ncbi.nlm.nih.gov/entrez/query.fcgi?db=gene&cmd=Retrieve&dopt=full_report&list_uids=134) | adenosine A1 receptor |
| [Details](http://mirdb.org/cgi-bin/target_detail.cgi?targetID=3073215) | 789 | 74 | hsa-miR-27a-3p | [CNOT1](http://www.ncbi.nlm.nih.gov/entrez/query.fcgi?db=gene&cmd=Retrieve&dopt=full_report&list_uids=23019) | CCR4-NOT transcription complex subunit 1 |
| [Details](http://mirdb.org/cgi-bin/target_detail.cgi?targetID=3073223) | 790 | 74 | hsa-miR-27a-3p | [IKZF5](http://www.ncbi.nlm.nih.gov/entrez/query.fcgi?db=gene&cmd=Retrieve&dopt=full_report&list_uids=64376) | IKAROS family zinc finger 5 |
| [Details](http://mirdb.org/cgi-bin/target_detail.cgi?targetID=3073246) | 791 | 74 | hsa-miR-27a-3p | [SNX12](http://www.ncbi.nlm.nih.gov/entrez/query.fcgi?db=gene&cmd=Retrieve&dopt=full_report&list_uids=29934) | sorting nexin 12 |
| [Details](http://mirdb.org/cgi-bin/target_detail.cgi?targetID=3073327) | 792 | 74 | hsa-miR-27a-3p | [CHST1](http://www.ncbi.nlm.nih.gov/entrez/query.fcgi?db=gene&cmd=Retrieve&dopt=full_report&list_uids=8534) | carbohydrate sulfotransferase 1 |
| [Details](http://mirdb.org/cgi-bin/target_detail.cgi?targetID=3073353) | 793 | 74 | hsa-miR-27a-3p | [DUSP5](http://www.ncbi.nlm.nih.gov/entrez/query.fcgi?db=gene&cmd=Retrieve&dopt=full_report&list_uids=1847) | dual specificity phosphatase 5 |
| [Details](http://mirdb.org/cgi-bin/target_detail.cgi?targetID=3073360) | 794 | 74 | hsa-miR-27a-3p | [JMJD1C](http://www.ncbi.nlm.nih.gov/entrez/query.fcgi?db=gene&cmd=Retrieve&dopt=full_report&list_uids=221037) | jumonji domain containing 1C |
| [Details](http://mirdb.org/cgi-bin/target_detail.cgi?targetID=3073414) | 795 | 74 | hsa-miR-27a-3p | [PDE8B](http://www.ncbi.nlm.nih.gov/entrez/query.fcgi?db=gene&cmd=Retrieve&dopt=full_report&list_uids=8622) | phosphodiesterase 8B |
| [Details](http://mirdb.org/cgi-bin/target_detail.cgi?targetID=3073468) | 796 | 74 | hsa-miR-27a-3p | [NFASC](http://www.ncbi.nlm.nih.gov/entrez/query.fcgi?db=gene&cmd=Retrieve&dopt=full_report&list_uids=23114) | neurofascin |
| [Details](http://mirdb.org/cgi-bin/target_detail.cgi?targetID=3073514) | 797 | 74 | hsa-miR-27a-3p | [CNOT7](http://www.ncbi.nlm.nih.gov/entrez/query.fcgi?db=gene&cmd=Retrieve&dopt=full_report&list_uids=29883) | CCR4-NOT transcription complex subunit 7 |
| [Details](http://mirdb.org/cgi-bin/target_detail.cgi?targetID=3073567) | 798 | 74 | hsa-miR-27a-3p | [FAM172A](http://www.ncbi.nlm.nih.gov/entrez/query.fcgi?db=gene&cmd=Retrieve&dopt=full_report&list_uids=83989) | family with sequence similarity 172 member A |
| [Details](http://mirdb.org/cgi-bin/target_detail.cgi?targetID=3073628) | 799 | 74 | hsa-miR-27a-3p | [PIP5K1B](http://www.ncbi.nlm.nih.gov/entrez/query.fcgi?db=gene&cmd=Retrieve&dopt=full_report&list_uids=8395) | phosphatidylinositol-4-phosphate 5-kinase type 1 beta |
| [Details](http://mirdb.org/cgi-bin/target_detail.cgi?targetID=3073643) | 800 | 74 | hsa-miR-27a-3p | [ADGRL2](http://www.ncbi.nlm.nih.gov/entrez/query.fcgi?db=gene&cmd=Retrieve&dopt=full_report&list_uids=23266) | adhesion G protein-coupled receptor L2 |
| [Details](http://mirdb.org/cgi-bin/target_detail.cgi?targetID=3073671) | 801 | 74 | hsa-miR-27a-3p | [SH3BGRL2](http://www.ncbi.nlm.nih.gov/entrez/query.fcgi?db=gene&cmd=Retrieve&dopt=full_report&list_uids=83699) | SH3 domain binding glutamate rich protein like 2 |
| [Details](http://mirdb.org/cgi-bin/target_detail.cgi?targetID=3073732) | 802 | 74 | hsa-miR-27a-3p | [MCPH1](http://www.ncbi.nlm.nih.gov/entrez/query.fcgi?db=gene&cmd=Retrieve&dopt=full_report&list_uids=79648) | microcephalin 1 |
| [Details](http://mirdb.org/cgi-bin/target_detail.cgi?targetID=3073824) | 803 | 74 | hsa-miR-27a-3p | [MYOCD](http://www.ncbi.nlm.nih.gov/entrez/query.fcgi?db=gene&cmd=Retrieve&dopt=full_report&list_uids=93649) | myocardin |
| [Details](http://mirdb.org/cgi-bin/target_detail.cgi?targetID=3072473) | 804 | 73 | hsa-miR-27a-3p | [LZTS3](http://www.ncbi.nlm.nih.gov/entrez/query.fcgi?db=gene&cmd=Retrieve&dopt=full_report&list_uids=9762) | leucine zipper tumor suppressor family member 3 |
| [Details](http://mirdb.org/cgi-bin/target_detail.cgi?targetID=3072519) | 805 | 73 | hsa-miR-27a-3p | [FOXP2](http://www.ncbi.nlm.nih.gov/entrez/query.fcgi?db=gene&cmd=Retrieve&dopt=full_report&list_uids=93986) | forkhead box P2 |
| [Details](http://mirdb.org/cgi-bin/target_detail.cgi?targetID=3072569) | 806 | 73 | hsa-miR-27a-3p | [ATF3](http://www.ncbi.nlm.nih.gov/entrez/query.fcgi?db=gene&cmd=Retrieve&dopt=full_report&list_uids=467) | activating transcription factor 3 |
| [Details](http://mirdb.org/cgi-bin/target_detail.cgi?targetID=3072600) | 807 | 73 | hsa-miR-27a-3p | [STXBP5](http://www.ncbi.nlm.nih.gov/entrez/query.fcgi?db=gene&cmd=Retrieve&dopt=full_report&list_uids=134957) | syntaxin binding protein 5 |
| [Details](http://mirdb.org/cgi-bin/target_detail.cgi?targetID=3072606) | 808 | 73 | hsa-miR-27a-3p | [SLC46A3](http://www.ncbi.nlm.nih.gov/entrez/query.fcgi?db=gene&cmd=Retrieve&dopt=full_report&list_uids=283537) | solute carrier family 46 member 3 |
| [Details](http://mirdb.org/cgi-bin/target_detail.cgi?targetID=3072650) | 809 | 73 | hsa-miR-27a-3p | [HINT3](http://www.ncbi.nlm.nih.gov/entrez/query.fcgi?db=gene&cmd=Retrieve&dopt=full_report&list_uids=135114) | histidine triad nucleotide binding protein 3 |
| [Details](http://mirdb.org/cgi-bin/target_detail.cgi?targetID=3072651) | 810 | 73 | hsa-miR-27a-3p | [PLEKHM3](http://www.ncbi.nlm.nih.gov/entrez/query.fcgi?db=gene&cmd=Retrieve&dopt=full_report&list_uids=389072) | pleckstrin homology domain containing M3 |
| [Details](http://mirdb.org/cgi-bin/target_detail.cgi?targetID=3072667) | 811 | 73 | hsa-miR-27a-3p | [ATP6V1A](http://www.ncbi.nlm.nih.gov/entrez/query.fcgi?db=gene&cmd=Retrieve&dopt=full_report&list_uids=523) | ATPase H+ transporting V1 subunit A |
| [Details](http://mirdb.org/cgi-bin/target_detail.cgi?targetID=3072690) | 812 | 73 | hsa-miR-27a-3p | [CALU](http://www.ncbi.nlm.nih.gov/entrez/query.fcgi?db=gene&cmd=Retrieve&dopt=full_report&list_uids=813) | calumenin |
| [Details](http://mirdb.org/cgi-bin/target_detail.cgi?targetID=3072697) | 813 | 73 | hsa-miR-27a-3p | [EPHA4](http://www.ncbi.nlm.nih.gov/entrez/query.fcgi?db=gene&cmd=Retrieve&dopt=full_report&list_uids=2043) | EPH receptor A4 |
| [Details](http://mirdb.org/cgi-bin/target_detail.cgi?targetID=3072725) | 814 | 73 | hsa-miR-27a-3p | [ENPEP](http://www.ncbi.nlm.nih.gov/entrez/query.fcgi?db=gene&cmd=Retrieve&dopt=full_report&list_uids=2028) | glutamyl aminopeptidase |
| [Details](http://mirdb.org/cgi-bin/target_detail.cgi?targetID=3072820) | 815 | 73 | hsa-miR-27a-3p | [DCHS2](http://www.ncbi.nlm.nih.gov/entrez/query.fcgi?db=gene&cmd=Retrieve&dopt=full_report&list_uids=54798) | dachsous cadherin-related 2 |
| [Details](http://mirdb.org/cgi-bin/target_detail.cgi?targetID=3072966) | 816 | 73 | hsa-miR-27a-3p | [ZNF100](http://www.ncbi.nlm.nih.gov/entrez/query.fcgi?db=gene&cmd=Retrieve&dopt=full_report&list_uids=163227) | zinc finger protein 100 |
| [Details](http://mirdb.org/cgi-bin/target_detail.cgi?targetID=3073070) | 817 | 73 | hsa-miR-27a-3p | [POLR2F](http://www.ncbi.nlm.nih.gov/entrez/query.fcgi?db=gene&cmd=Retrieve&dopt=full_report&list_uids=5435) | RNA polymerase II subunit F |
| [Details](http://mirdb.org/cgi-bin/target_detail.cgi?targetID=3073124) | 818 | 73 | hsa-miR-27a-3p | [SGPP1](http://www.ncbi.nlm.nih.gov/entrez/query.fcgi?db=gene&cmd=Retrieve&dopt=full_report&list_uids=81537) | sphingosine-1-phosphate phosphatase 1 |
| [Details](http://mirdb.org/cgi-bin/target_detail.cgi?targetID=3073153) | 819 | 73 | hsa-miR-27a-3p | [GABRR2](http://www.ncbi.nlm.nih.gov/entrez/query.fcgi?db=gene&cmd=Retrieve&dopt=full_report&list_uids=2570) | gamma-aminobutyric acid type A receptor rho2 subunit |
| [Details](http://mirdb.org/cgi-bin/target_detail.cgi?targetID=3073178) | 820 | 73 | hsa-miR-27a-3p | [MED13L](http://www.ncbi.nlm.nih.gov/entrez/query.fcgi?db=gene&cmd=Retrieve&dopt=full_report&list_uids=23389) | mediator complex subunit 13 like |
| [Details](http://mirdb.org/cgi-bin/target_detail.cgi?targetID=3073184) | 821 | 73 | hsa-miR-27a-3p | [ZNF189](http://www.ncbi.nlm.nih.gov/entrez/query.fcgi?db=gene&cmd=Retrieve&dopt=full_report&list_uids=7743) | zinc finger protein 189 |
| [Details](http://mirdb.org/cgi-bin/target_detail.cgi?targetID=3073213) | 822 | 73 | hsa-miR-27a-3p | [ZBTB10](http://www.ncbi.nlm.nih.gov/entrez/query.fcgi?db=gene&cmd=Retrieve&dopt=full_report&list_uids=65986) | zinc finger and BTB domain containing 10 |
| [Details](http://mirdb.org/cgi-bin/target_detail.cgi?targetID=3073292) | 823 | 73 | hsa-miR-27a-3p | [RETREG3](http://www.ncbi.nlm.nih.gov/entrez/query.fcgi?db=gene&cmd=Retrieve&dopt=full_report&list_uids=162427) | reticulophagy regulator family member 3 |
| [Details](http://mirdb.org/cgi-bin/target_detail.cgi?targetID=3073393) | 824 | 73 | hsa-miR-27a-3p | [VIP](http://www.ncbi.nlm.nih.gov/entrez/query.fcgi?db=gene&cmd=Retrieve&dopt=full_report&list_uids=7432) | vasoactive intestinal peptide |
| [Details](http://mirdb.org/cgi-bin/target_detail.cgi?targetID=3073481) | 825 | 73 | hsa-miR-27a-3p | [C5orf63](http://www.ncbi.nlm.nih.gov/entrez/query.fcgi?db=gene&cmd=Retrieve&dopt=full_report&list_uids=401207) | chromosome 5 open reading frame 63 |
| [Details](http://mirdb.org/cgi-bin/target_detail.cgi?targetID=3073509) | 826 | 73 | hsa-miR-27a-3p | [GCH1](http://www.ncbi.nlm.nih.gov/entrez/query.fcgi?db=gene&cmd=Retrieve&dopt=full_report&list_uids=2643) | GTP cyclohydrolase 1 |
| [Details](http://mirdb.org/cgi-bin/target_detail.cgi?targetID=3073532) | 827 | 73 | hsa-miR-27a-3p | [BCOR](http://www.ncbi.nlm.nih.gov/entrez/query.fcgi?db=gene&cmd=Retrieve&dopt=full_report&list_uids=54880) | BCL6 corepressor |
| [Details](http://mirdb.org/cgi-bin/target_detail.cgi?targetID=3073592) | 828 | 73 | hsa-miR-27a-3p | [FAM189B](http://www.ncbi.nlm.nih.gov/entrez/query.fcgi?db=gene&cmd=Retrieve&dopt=full_report&list_uids=10712) | family with sequence similarity 189 member B |
| [Details](http://mirdb.org/cgi-bin/target_detail.cgi?targetID=3073600) | 829 | 73 | hsa-miR-27a-3p | [IRS1](http://www.ncbi.nlm.nih.gov/entrez/query.fcgi?db=gene&cmd=Retrieve&dopt=full_report&list_uids=3667) | insulin receptor substrate 1 |
| [Details](http://mirdb.org/cgi-bin/target_detail.cgi?targetID=3073649) | 830 | 73 | hsa-miR-27a-3p | [LIMK2](http://www.ncbi.nlm.nih.gov/entrez/query.fcgi?db=gene&cmd=Retrieve&dopt=full_report&list_uids=3985) | LIM domain kinase 2 |
| [Details](http://mirdb.org/cgi-bin/target_detail.cgi?targetID=3073676) | 831 | 73 | hsa-miR-27a-3p | [RS1](http://www.ncbi.nlm.nih.gov/entrez/query.fcgi?db=gene&cmd=Retrieve&dopt=full_report&list_uids=6247) | retinoschisin 1 |
| [Details](http://mirdb.org/cgi-bin/target_detail.cgi?targetID=3073785) | 832 | 73 | hsa-miR-27a-3p | [ABCA12](http://www.ncbi.nlm.nih.gov/entrez/query.fcgi?db=gene&cmd=Retrieve&dopt=full_report&list_uids=26154) | ATP binding cassette subfamily A member 12 |
| [Details](http://mirdb.org/cgi-bin/target_detail.cgi?targetID=3073894) | 833 | 73 | hsa-miR-27a-3p | [ALG8](http://www.ncbi.nlm.nih.gov/entrez/query.fcgi?db=gene&cmd=Retrieve&dopt=full_report&list_uids=79053) | ALG8, alpha-1,3-glucosyltransferase |
| [Details](http://mirdb.org/cgi-bin/target_detail.cgi?targetID=3073917) | 834 | 73 | hsa-miR-27a-3p | [HMGN1](http://www.ncbi.nlm.nih.gov/entrez/query.fcgi?db=gene&cmd=Retrieve&dopt=full_report&list_uids=3150) | high mobility group nucleosome binding domain 1 |
| [Details](http://mirdb.org/cgi-bin/target_detail.cgi?targetID=3072544) | 835 | 72 | hsa-miR-27a-3p | [IFITM10](http://www.ncbi.nlm.nih.gov/entrez/query.fcgi?db=gene&cmd=Retrieve&dopt=full_report&list_uids=402778) | interferon induced transmembrane protein 10 |
| [Details](http://mirdb.org/cgi-bin/target_detail.cgi?targetID=3072548) | 836 | 72 | hsa-miR-27a-3p | [WDR43](http://www.ncbi.nlm.nih.gov/entrez/query.fcgi?db=gene&cmd=Retrieve&dopt=full_report&list_uids=23160) | WD repeat domain 43 |
| [Details](http://mirdb.org/cgi-bin/target_detail.cgi?targetID=3072644) | 837 | 72 | hsa-miR-27a-3p | [NUP153](http://www.ncbi.nlm.nih.gov/entrez/query.fcgi?db=gene&cmd=Retrieve&dopt=full_report&list_uids=9972) | nucleoporin 153 |
| [Details](http://mirdb.org/cgi-bin/target_detail.cgi?targetID=3072698) | 838 | 72 | hsa-miR-27a-3p | [NLK](http://www.ncbi.nlm.nih.gov/entrez/query.fcgi?db=gene&cmd=Retrieve&dopt=full_report&list_uids=51701) | nemo like kinase |
| [Details](http://mirdb.org/cgi-bin/target_detail.cgi?targetID=3072716) | 839 | 72 | hsa-miR-27a-3p | [DNA2](http://www.ncbi.nlm.nih.gov/entrez/query.fcgi?db=gene&cmd=Retrieve&dopt=full_report&list_uids=1763) | DNA replication helicase/nuclease 2 |
| [Details](http://mirdb.org/cgi-bin/target_detail.cgi?targetID=3072885) | 840 | 72 | hsa-miR-27a-3p | [SMARCA1](http://www.ncbi.nlm.nih.gov/entrez/query.fcgi?db=gene&cmd=Retrieve&dopt=full_report&list_uids=6594) | SWI/SNF related, matrix associated, actin dependent regulator of chromatin, subfamily a, member 1 |
| [Details](http://mirdb.org/cgi-bin/target_detail.cgi?targetID=3072893) | 841 | 72 | hsa-miR-27a-3p | [JMJD8](http://www.ncbi.nlm.nih.gov/entrez/query.fcgi?db=gene&cmd=Retrieve&dopt=full_report&list_uids=339123) | jumonji domain containing 8 |
| [Details](http://mirdb.org/cgi-bin/target_detail.cgi?targetID=3072955) | 842 | 72 | hsa-miR-27a-3p | [EPC1](http://www.ncbi.nlm.nih.gov/entrez/query.fcgi?db=gene&cmd=Retrieve&dopt=full_report&list_uids=80314) | enhancer of polycomb homolog 1 |
| [Details](http://mirdb.org/cgi-bin/target_detail.cgi?targetID=3072980) | 843 | 72 | hsa-miR-27a-3p | [DEPDC1B](http://www.ncbi.nlm.nih.gov/entrez/query.fcgi?db=gene&cmd=Retrieve&dopt=full_report&list_uids=55789) | DEP domain containing 1B |
| [Details](http://mirdb.org/cgi-bin/target_detail.cgi?targetID=3072990) | 844 | 72 | hsa-miR-27a-3p | [SREK1](http://www.ncbi.nlm.nih.gov/entrez/query.fcgi?db=gene&cmd=Retrieve&dopt=full_report&list_uids=140890) | splicing regulatory glutamic acid and lysine rich protein 1 |
| [Details](http://mirdb.org/cgi-bin/target_detail.cgi?targetID=3073025) | 845 | 72 | hsa-miR-27a-3p | [SLC18B1](http://www.ncbi.nlm.nih.gov/entrez/query.fcgi?db=gene&cmd=Retrieve&dopt=full_report&list_uids=116843) | solute carrier family 18 member B1 |
| [Details](http://mirdb.org/cgi-bin/target_detail.cgi?targetID=3073139) | 846 | 72 | hsa-miR-27a-3p | [SLC25A44](http://www.ncbi.nlm.nih.gov/entrez/query.fcgi?db=gene&cmd=Retrieve&dopt=full_report&list_uids=9673) | solute carrier family 25 member 44 |
| [Details](http://mirdb.org/cgi-bin/target_detail.cgi?targetID=3073161) | 847 | 72 | hsa-miR-27a-3p | [ATP5MC3](http://www.ncbi.nlm.nih.gov/entrez/query.fcgi?db=gene&cmd=Retrieve&dopt=full_report&list_uids=518) | ATP synthase membrane subunit c locus 3 |
| [Details](http://mirdb.org/cgi-bin/target_detail.cgi?targetID=3073167) | 848 | 72 | hsa-miR-27a-3p | [ARNT2](http://www.ncbi.nlm.nih.gov/entrez/query.fcgi?db=gene&cmd=Retrieve&dopt=full_report&list_uids=9915) | aryl hydrocarbon receptor nuclear translocator 2 |
| [Details](http://mirdb.org/cgi-bin/target_detail.cgi?targetID=3073179) | 849 | 72 | hsa-miR-27a-3p | [ADGRG6](http://www.ncbi.nlm.nih.gov/entrez/query.fcgi?db=gene&cmd=Retrieve&dopt=full_report&list_uids=57211) | adhesion G protein-coupled receptor G6 |
| [Details](http://mirdb.org/cgi-bin/target_detail.cgi?targetID=3073203) | 850 | 72 | hsa-miR-27a-3p | [EHD3](http://www.ncbi.nlm.nih.gov/entrez/query.fcgi?db=gene&cmd=Retrieve&dopt=full_report&list_uids=30845) | EH domain containing 3 |
| [Details](http://mirdb.org/cgi-bin/target_detail.cgi?targetID=3073218) | 851 | 72 | hsa-miR-27a-3p | [BCAN](http://www.ncbi.nlm.nih.gov/entrez/query.fcgi?db=gene&cmd=Retrieve&dopt=full_report&list_uids=63827) | brevican |
| [Details](http://mirdb.org/cgi-bin/target_detail.cgi?targetID=3073278) | 852 | 72 | hsa-miR-27a-3p | [PRR14L](http://www.ncbi.nlm.nih.gov/entrez/query.fcgi?db=gene&cmd=Retrieve&dopt=full_report&list_uids=253143) | proline rich 14 like |
| [Details](http://mirdb.org/cgi-bin/target_detail.cgi?targetID=3073283) | 853 | 72 | hsa-miR-27a-3p | [TARDBP](http://www.ncbi.nlm.nih.gov/entrez/query.fcgi?db=gene&cmd=Retrieve&dopt=full_report&list_uids=23435) | TAR DNA binding protein |
| [Details](http://mirdb.org/cgi-bin/target_detail.cgi?targetID=3073406) | 854 | 72 | hsa-miR-27a-3p | [PLPP6](http://www.ncbi.nlm.nih.gov/entrez/query.fcgi?db=gene&cmd=Retrieve&dopt=full_report&list_uids=403313) | phospholipid phosphatase 6 |
| [Details](http://mirdb.org/cgi-bin/target_detail.cgi?targetID=3073409) | 855 | 72 | hsa-miR-27a-3p | [NRXN1](http://www.ncbi.nlm.nih.gov/entrez/query.fcgi?db=gene&cmd=Retrieve&dopt=full_report&list_uids=9378) | neurexin 1 |
| [Details](http://mirdb.org/cgi-bin/target_detail.cgi?targetID=3073455) | 856 | 72 | hsa-miR-27a-3p | [CBX1](http://www.ncbi.nlm.nih.gov/entrez/query.fcgi?db=gene&cmd=Retrieve&dopt=full_report&list_uids=10951) | chromobox 1 |
| [Details](http://mirdb.org/cgi-bin/target_detail.cgi?targetID=3073457) | 857 | 72 | hsa-miR-27a-3p | [BRSK2](http://www.ncbi.nlm.nih.gov/entrez/query.fcgi?db=gene&cmd=Retrieve&dopt=full_report&list_uids=9024) | BR serine/threonine kinase 2 |
| [Details](http://mirdb.org/cgi-bin/target_detail.cgi?targetID=3073479) | 858 | 72 | hsa-miR-27a-3p | [DNAJC12](http://www.ncbi.nlm.nih.gov/entrez/query.fcgi?db=gene&cmd=Retrieve&dopt=full_report&list_uids=56521) | DnaJ heat shock protein family (Hsp40) member C12 |
| [Details](http://mirdb.org/cgi-bin/target_detail.cgi?targetID=3073648) | 859 | 72 | hsa-miR-27a-3p | [YPEL1](http://www.ncbi.nlm.nih.gov/entrez/query.fcgi?db=gene&cmd=Retrieve&dopt=full_report&list_uids=29799) | yippee like 1 |
| [Details](http://mirdb.org/cgi-bin/target_detail.cgi?targetID=3073652) | 860 | 72 | hsa-miR-27a-3p | [B4GALNT4](http://www.ncbi.nlm.nih.gov/entrez/query.fcgi?db=gene&cmd=Retrieve&dopt=full_report&list_uids=338707) | beta-1,4-N-acetyl-galactosaminyltransferase 4 |
| [Details](http://mirdb.org/cgi-bin/target_detail.cgi?targetID=3073720) | 861 | 72 | hsa-miR-27a-3p | [TFPI](http://www.ncbi.nlm.nih.gov/entrez/query.fcgi?db=gene&cmd=Retrieve&dopt=full_report&list_uids=7035) | tissue factor pathway inhibitor |
| [Details](http://mirdb.org/cgi-bin/target_detail.cgi?targetID=3073772) | 862 | 72 | hsa-miR-27a-3p | [PDPN](http://www.ncbi.nlm.nih.gov/entrez/query.fcgi?db=gene&cmd=Retrieve&dopt=full_report&list_uids=10630) | podoplanin |
| [Details](http://mirdb.org/cgi-bin/target_detail.cgi?targetID=3073801) | 863 | 72 | hsa-miR-27a-3p | [TMED5](http://www.ncbi.nlm.nih.gov/entrez/query.fcgi?db=gene&cmd=Retrieve&dopt=full_report&list_uids=50999) | transmembrane p24 trafficking protein 5 |
| [Details](http://mirdb.org/cgi-bin/target_detail.cgi?targetID=3073816) | 864 | 72 | hsa-miR-27a-3p | [ZNF124](http://www.ncbi.nlm.nih.gov/entrez/query.fcgi?db=gene&cmd=Retrieve&dopt=full_report&list_uids=7678) | zinc finger protein 124 |
| [Details](http://mirdb.org/cgi-bin/target_detail.cgi?targetID=3073874) | 865 | 72 | hsa-miR-27a-3p | [MXI1](http://www.ncbi.nlm.nih.gov/entrez/query.fcgi?db=gene&cmd=Retrieve&dopt=full_report&list_uids=4601) | MAX interactor 1, dimerization protein |
| [Details](http://mirdb.org/cgi-bin/target_detail.cgi?targetID=3073904) | 866 | 72 | hsa-miR-27a-3p | [PLAGL2](http://www.ncbi.nlm.nih.gov/entrez/query.fcgi?db=gene&cmd=Retrieve&dopt=full_report&list_uids=5326) | PLAG1 like zinc finger 2 |
| [Details](http://mirdb.org/cgi-bin/target_detail.cgi?targetID=3072493) | 867 | 71 | hsa-miR-27a-3p | [SUB1](http://www.ncbi.nlm.nih.gov/entrez/query.fcgi?db=gene&cmd=Retrieve&dopt=full_report&list_uids=10923) | SUB1 homolog, transcriptional regulator |
| [Details](http://mirdb.org/cgi-bin/target_detail.cgi?targetID=3072527) | 868 | 71 | hsa-miR-27a-3p | [MAGEF1](http://www.ncbi.nlm.nih.gov/entrez/query.fcgi?db=gene&cmd=Retrieve&dopt=full_report&list_uids=64110) | MAGE family member F1 |
| [Details](http://mirdb.org/cgi-bin/target_detail.cgi?targetID=3072576) | 869 | 71 | hsa-miR-27a-3p | [UMAD1](http://www.ncbi.nlm.nih.gov/entrez/query.fcgi?db=gene&cmd=Retrieve&dopt=full_report&list_uids=729852) | UBAP1-MVB12-associated (UMA) domain containing 1 |
| [Details](http://mirdb.org/cgi-bin/target_detail.cgi?targetID=3072633) | 870 | 71 | hsa-miR-27a-3p | [GRAMD1C](http://www.ncbi.nlm.nih.gov/entrez/query.fcgi?db=gene&cmd=Retrieve&dopt=full_report&list_uids=54762) | GRAM domain containing 1C |
| [Details](http://mirdb.org/cgi-bin/target_detail.cgi?targetID=3072696) | 871 | 71 | hsa-miR-27a-3p | [THOC1](http://www.ncbi.nlm.nih.gov/entrez/query.fcgi?db=gene&cmd=Retrieve&dopt=full_report&list_uids=9984) | THO complex 1 |
| [Details](http://mirdb.org/cgi-bin/target_detail.cgi?targetID=3072730) | 872 | 71 | hsa-miR-27a-3p | [CHML](http://www.ncbi.nlm.nih.gov/entrez/query.fcgi?db=gene&cmd=Retrieve&dopt=full_report&list_uids=1122) | CHM like, Rab escort protein 2 |
| [Details](http://mirdb.org/cgi-bin/target_detail.cgi?targetID=3072770) | 873 | 71 | hsa-miR-27a-3p | [CSNK1G1](http://www.ncbi.nlm.nih.gov/entrez/query.fcgi?db=gene&cmd=Retrieve&dopt=full_report&list_uids=53944) | casein kinase 1 gamma 1 |
| [Details](http://mirdb.org/cgi-bin/target_detail.cgi?targetID=3072774) | 874 | 71 | hsa-miR-27a-3p | [RNF144A](http://www.ncbi.nlm.nih.gov/entrez/query.fcgi?db=gene&cmd=Retrieve&dopt=full_report&list_uids=9781) | ring finger protein 144A |
| [Details](http://mirdb.org/cgi-bin/target_detail.cgi?targetID=3072796) | 875 | 71 | hsa-miR-27a-3p | [OSBPL10](http://www.ncbi.nlm.nih.gov/entrez/query.fcgi?db=gene&cmd=Retrieve&dopt=full_report&list_uids=114884) | oxysterol binding protein like 10 |
| [Details](http://mirdb.org/cgi-bin/target_detail.cgi?targetID=3072889) | 876 | 71 | hsa-miR-27a-3p | [RFX3](http://www.ncbi.nlm.nih.gov/entrez/query.fcgi?db=gene&cmd=Retrieve&dopt=full_report&list_uids=5991) | regulatory factor X3 |
| [Details](http://mirdb.org/cgi-bin/target_detail.cgi?targetID=3072960) | 877 | 71 | hsa-miR-27a-3p | [AKR7A2](http://www.ncbi.nlm.nih.gov/entrez/query.fcgi?db=gene&cmd=Retrieve&dopt=full_report&list_uids=8574) | aldo-keto reductase family 7 member A2 |
| [Details](http://mirdb.org/cgi-bin/target_detail.cgi?targetID=3072963) | 878 | 71 | hsa-miR-27a-3p | [MTSS1L](http://www.ncbi.nlm.nih.gov/entrez/query.fcgi?db=gene&cmd=Retrieve&dopt=full_report&list_uids=92154) | MTSS1L, I-BAR domain containing |
| [Details](http://mirdb.org/cgi-bin/target_detail.cgi?targetID=3073028) | 879 | 71 | hsa-miR-27a-3p | [ADD1](http://www.ncbi.nlm.nih.gov/entrez/query.fcgi?db=gene&cmd=Retrieve&dopt=full_report&list_uids=118) | adducin 1 |
| [Details](http://mirdb.org/cgi-bin/target_detail.cgi?targetID=3073031) | 880 | 71 | hsa-miR-27a-3p | [CABP1](http://www.ncbi.nlm.nih.gov/entrez/query.fcgi?db=gene&cmd=Retrieve&dopt=full_report&list_uids=9478) | calcium binding protein 1 |
| [Details](http://mirdb.org/cgi-bin/target_detail.cgi?targetID=3073127) | 881 | 71 | hsa-miR-27a-3p | [HIVEP2](http://www.ncbi.nlm.nih.gov/entrez/query.fcgi?db=gene&cmd=Retrieve&dopt=full_report&list_uids=3097) | human immunodeficiency virus type I enhancer binding protein 2 |
| [Details](http://mirdb.org/cgi-bin/target_detail.cgi?targetID=3073190) | 882 | 71 | hsa-miR-27a-3p | [PTGER2](http://www.ncbi.nlm.nih.gov/entrez/query.fcgi?db=gene&cmd=Retrieve&dopt=full_report&list_uids=5732) | prostaglandin E receptor 2 |
| [Details](http://mirdb.org/cgi-bin/target_detail.cgi?targetID=3073244) | 883 | 71 | hsa-miR-27a-3p | [AGPS](http://www.ncbi.nlm.nih.gov/entrez/query.fcgi?db=gene&cmd=Retrieve&dopt=full_report&list_uids=8540) | alkylglycerone phosphate synthase |
| [Details](http://mirdb.org/cgi-bin/target_detail.cgi?targetID=3073309) | 884 | 71 | hsa-miR-27a-3p | [LYPD3](http://www.ncbi.nlm.nih.gov/entrez/query.fcgi?db=gene&cmd=Retrieve&dopt=full_report&list_uids=27076) | LY6/PLAUR domain containing 3 |
| [Details](http://mirdb.org/cgi-bin/target_detail.cgi?targetID=3073332) | 885 | 71 | hsa-miR-27a-3p | [RCAN1](http://www.ncbi.nlm.nih.gov/entrez/query.fcgi?db=gene&cmd=Retrieve&dopt=full_report&list_uids=1827) | regulator of calcineurin 1 |
| [Details](http://mirdb.org/cgi-bin/target_detail.cgi?targetID=3073387) | 886 | 71 | hsa-miR-27a-3p | [PTHLH](http://www.ncbi.nlm.nih.gov/entrez/query.fcgi?db=gene&cmd=Retrieve&dopt=full_report&list_uids=5744) | parathyroid hormone like hormone |
| [Details](http://mirdb.org/cgi-bin/target_detail.cgi?targetID=3073580) | 887 | 71 | hsa-miR-27a-3p | [MMP16](http://www.ncbi.nlm.nih.gov/entrez/query.fcgi?db=gene&cmd=Retrieve&dopt=full_report&list_uids=4325) | matrix metallopeptidase 16 |
| [Details](http://mirdb.org/cgi-bin/target_detail.cgi?targetID=3073585) | 888 | 71 | hsa-miR-27a-3p | [SRRM2](http://www.ncbi.nlm.nih.gov/entrez/query.fcgi?db=gene&cmd=Retrieve&dopt=full_report&list_uids=23524) | serine/arginine repetitive matrix 2 |
| [Details](http://mirdb.org/cgi-bin/target_detail.cgi?targetID=3073594) | 889 | 71 | hsa-miR-27a-3p | [GSE1](http://www.ncbi.nlm.nih.gov/entrez/query.fcgi?db=gene&cmd=Retrieve&dopt=full_report&list_uids=23199) | Gse1 coiled-coil protein |
| [Details](http://mirdb.org/cgi-bin/target_detail.cgi?targetID=3073695) | 890 | 71 | hsa-miR-27a-3p | [HCRTR1](http://www.ncbi.nlm.nih.gov/entrez/query.fcgi?db=gene&cmd=Retrieve&dopt=full_report&list_uids=3061) | hypocretin receptor 1 |
| [Details](http://mirdb.org/cgi-bin/target_detail.cgi?targetID=3073704) | 891 | 71 | hsa-miR-27a-3p | [ZBTB25](http://www.ncbi.nlm.nih.gov/entrez/query.fcgi?db=gene&cmd=Retrieve&dopt=full_report&list_uids=7597) | zinc finger and BTB domain containing 25 |
| [Details](http://mirdb.org/cgi-bin/target_detail.cgi?targetID=3073760) | 892 | 71 | hsa-miR-27a-3p | [CAP2](http://www.ncbi.nlm.nih.gov/entrez/query.fcgi?db=gene&cmd=Retrieve&dopt=full_report&list_uids=10486) | cyclase associated actin cytoskeleton regulatory protein 2 |
| [Details](http://mirdb.org/cgi-bin/target_detail.cgi?targetID=3073830) | 893 | 71 | hsa-miR-27a-3p | [FBXO46](http://www.ncbi.nlm.nih.gov/entrez/query.fcgi?db=gene&cmd=Retrieve&dopt=full_report&list_uids=23403) | F-box protein 46 |
| [Details](http://mirdb.org/cgi-bin/target_detail.cgi?targetID=3073898) | 894 | 71 | hsa-miR-27a-3p | [NRIP1](http://www.ncbi.nlm.nih.gov/entrez/query.fcgi?db=gene&cmd=Retrieve&dopt=full_report&list_uids=8204) | nuclear receptor interacting protein 1 |
| [Details](http://mirdb.org/cgi-bin/target_detail.cgi?targetID=3072471) | 895 | 70 | hsa-miR-27a-3p | [ZFP36L2](http://www.ncbi.nlm.nih.gov/entrez/query.fcgi?db=gene&cmd=Retrieve&dopt=full_report&list_uids=678) | ZFP36 ring finger protein like 2 |
| [Details](http://mirdb.org/cgi-bin/target_detail.cgi?targetID=3072585) | 896 | 70 | hsa-miR-27a-3p | [KCNC2](http://www.ncbi.nlm.nih.gov/entrez/query.fcgi?db=gene&cmd=Retrieve&dopt=full_report&list_uids=3747) | potassium voltage-gated channel subfamily C member 2 |
| [Details](http://mirdb.org/cgi-bin/target_detail.cgi?targetID=3072671) | 897 | 70 | hsa-miR-27a-3p | [GRSF1](http://www.ncbi.nlm.nih.gov/entrez/query.fcgi?db=gene&cmd=Retrieve&dopt=full_report&list_uids=2926) | G-rich RNA sequence binding factor 1 |
| [Details](http://mirdb.org/cgi-bin/target_detail.cgi?targetID=3072674) | 898 | 70 | hsa-miR-27a-3p | [RTKN2](http://www.ncbi.nlm.nih.gov/entrez/query.fcgi?db=gene&cmd=Retrieve&dopt=full_report&list_uids=219790) | rhotekin 2 |
| [Details](http://mirdb.org/cgi-bin/target_detail.cgi?targetID=3072708) | 899 | 70 | hsa-miR-27a-3p | [GNB4](http://www.ncbi.nlm.nih.gov/entrez/query.fcgi?db=gene&cmd=Retrieve&dopt=full_report&list_uids=59345) | G protein subunit beta 4 |
| [Details](http://mirdb.org/cgi-bin/target_detail.cgi?targetID=3072877) | 900 | 70 | hsa-miR-27a-3p | [DAZAP2](http://www.ncbi.nlm.nih.gov/entrez/query.fcgi?db=gene&cmd=Retrieve&dopt=full_report&list_uids=9802) | DAZ associated protein 2 |
| [Details](http://mirdb.org/cgi-bin/target_detail.cgi?targetID=3072925) | 901 | 70 | hsa-miR-27a-3p | [URB1](http://www.ncbi.nlm.nih.gov/entrez/query.fcgi?db=gene&cmd=Retrieve&dopt=full_report&list_uids=9875) | URB1 ribosome biogenesis homolog |
| [Details](http://mirdb.org/cgi-bin/target_detail.cgi?targetID=3072983) | 902 | 70 | hsa-miR-27a-3p | [ADAMTS5](http://www.ncbi.nlm.nih.gov/entrez/query.fcgi?db=gene&cmd=Retrieve&dopt=full_report&list_uids=11096) | ADAM metallopeptidase with thrombospondin type 1 motif 5 |
| [Details](http://mirdb.org/cgi-bin/target_detail.cgi?targetID=3073104) | 903 | 70 | hsa-miR-27a-3p | [SFXN2](http://www.ncbi.nlm.nih.gov/entrez/query.fcgi?db=gene&cmd=Retrieve&dopt=full_report&list_uids=118980) | sideroflexin 2 |
| [Details](http://mirdb.org/cgi-bin/target_detail.cgi?targetID=3073207) | 904 | 70 | hsa-miR-27a-3p | [POM121C](http://www.ncbi.nlm.nih.gov/entrez/query.fcgi?db=gene&cmd=Retrieve&dopt=full_report&list_uids=100101267) | POM121 transmembrane nucleoporin C |
| [Details](http://mirdb.org/cgi-bin/target_detail.cgi?targetID=3073535) | 905 | 70 | hsa-miR-27a-3p | [LDLRAD4](http://www.ncbi.nlm.nih.gov/entrez/query.fcgi?db=gene&cmd=Retrieve&dopt=full_report&list_uids=753) | low density lipoprotein receptor class A domain containing 4 |
| [Details](http://mirdb.org/cgi-bin/target_detail.cgi?targetID=3073566) | 906 | 70 | hsa-miR-27a-3p | [GCA](http://www.ncbi.nlm.nih.gov/entrez/query.fcgi?db=gene&cmd=Retrieve&dopt=full_report&list_uids=25801) | grancalcin |
| [Details](http://mirdb.org/cgi-bin/target_detail.cgi?targetID=3073569) | 907 | 70 | hsa-miR-27a-3p | [CCDC175](http://www.ncbi.nlm.nih.gov/entrez/query.fcgi?db=gene&cmd=Retrieve&dopt=full_report&list_uids=729665) | coiled-coil domain containing 175 |
| [Details](http://mirdb.org/cgi-bin/target_detail.cgi?targetID=3073687) | 908 | 70 | hsa-miR-27a-3p | [GSPT2](http://www.ncbi.nlm.nih.gov/entrez/query.fcgi?db=gene&cmd=Retrieve&dopt=full_report&list_uids=23708) | G1 to S phase transition 2 |
| [Details](http://mirdb.org/cgi-bin/target_detail.cgi?targetID=3073717) | 909 | 70 | hsa-miR-27a-3p | [TCEA1](http://www.ncbi.nlm.nih.gov/entrez/query.fcgi?db=gene&cmd=Retrieve&dopt=full_report&list_uids=6917) | transcription elongation factor A1 |
| [Details](http://mirdb.org/cgi-bin/target_detail.cgi?targetID=3073768) | 910 | 70 | hsa-miR-27a-3p | [GRIN3A](http://www.ncbi.nlm.nih.gov/entrez/query.fcgi?db=gene&cmd=Retrieve&dopt=full_report&list_uids=116443) | glutamate ionotropic receptor NMDA type subunit 3A |
| [Details](http://mirdb.org/cgi-bin/target_detail.cgi?targetID=3073777) | 911 | 70 | hsa-miR-27a-3p | [C6orf120](http://www.ncbi.nlm.nih.gov/entrez/query.fcgi?db=gene&cmd=Retrieve&dopt=full_report&list_uids=387263) | chromosome 6 open reading frame 120 |
| [Details](http://mirdb.org/cgi-bin/target_detail.cgi?targetID=3073831) | 912 | 70 | hsa-miR-27a-3p | [SRSF1](http://www.ncbi.nlm.nih.gov/entrez/query.fcgi?db=gene&cmd=Retrieve&dopt=full_report&list_uids=6426) | serine and arginine rich splicing factor 1 |
| [Details](http://mirdb.org/cgi-bin/target_detail.cgi?targetID=3073845) | 913 | 70 | hsa-miR-27a-3p | [CHST2](http://www.ncbi.nlm.nih.gov/entrez/query.fcgi?db=gene&cmd=Retrieve&dopt=full_report&list_uids=9435) | carbohydrate sulfotransferase 2 |
| [Details](http://mirdb.org/cgi-bin/target_detail.cgi?targetID=3073905) | 914 | 70 | hsa-miR-27a-3p | [NME9](http://www.ncbi.nlm.nih.gov/entrez/query.fcgi?db=gene&cmd=Retrieve&dopt=full_report&list_uids=347736) | NME/NM23 family member 9 |
| [Details](http://mirdb.org/cgi-bin/target_detail.cgi?targetID=3072448) | 915 | 69 | hsa-miR-27a-3p | [ZNF705A](http://www.ncbi.nlm.nih.gov/entrez/query.fcgi?db=gene&cmd=Retrieve&dopt=full_report&list_uids=440077) | zinc finger protein 705A |
| [Details](http://mirdb.org/cgi-bin/target_detail.cgi?targetID=3072578) | 916 | 69 | hsa-miR-27a-3p | [SLC1A2](http://www.ncbi.nlm.nih.gov/entrez/query.fcgi?db=gene&cmd=Retrieve&dopt=full_report&list_uids=6506) | solute carrier family 1 member 2 |
| [Details](http://mirdb.org/cgi-bin/target_detail.cgi?targetID=3072591) | 917 | 69 | hsa-miR-27a-3p | [CYP1B1](http://www.ncbi.nlm.nih.gov/entrez/query.fcgi?db=gene&cmd=Retrieve&dopt=full_report&list_uids=1545) | cytochrome P450 family 1 subfamily B member 1 |
| [Details](http://mirdb.org/cgi-bin/target_detail.cgi?targetID=3072595) | 918 | 69 | hsa-miR-27a-3p | [FASTKD5](http://www.ncbi.nlm.nih.gov/entrez/query.fcgi?db=gene&cmd=Retrieve&dopt=full_report&list_uids=60493) | FAST kinase domains 5 |
| [Details](http://mirdb.org/cgi-bin/target_detail.cgi?targetID=3072735) | 919 | 69 | hsa-miR-27a-3p | [HORMAD2](http://www.ncbi.nlm.nih.gov/entrez/query.fcgi?db=gene&cmd=Retrieve&dopt=full_report&list_uids=150280) | HORMA domain containing 2 |
| [Details](http://mirdb.org/cgi-bin/target_detail.cgi?targetID=3072860) | 920 | 69 | hsa-miR-27a-3p | [CRTC1](http://www.ncbi.nlm.nih.gov/entrez/query.fcgi?db=gene&cmd=Retrieve&dopt=full_report&list_uids=23373) | CREB regulated transcription coactivator 1 |
| [Details](http://mirdb.org/cgi-bin/target_detail.cgi?targetID=3072959) | 921 | 69 | hsa-miR-27a-3p | [EDIL3](http://www.ncbi.nlm.nih.gov/entrez/query.fcgi?db=gene&cmd=Retrieve&dopt=full_report&list_uids=10085) | EGF like repeats and discoidin domains 3 |
| [Details](http://mirdb.org/cgi-bin/target_detail.cgi?targetID=3073053) | 922 | 69 | hsa-miR-27a-3p | [ATF2](http://www.ncbi.nlm.nih.gov/entrez/query.fcgi?db=gene&cmd=Retrieve&dopt=full_report&list_uids=1386) | activating transcription factor 2 |
| [Details](http://mirdb.org/cgi-bin/target_detail.cgi?targetID=3073083) | 923 | 69 | hsa-miR-27a-3p | [KRAS](http://www.ncbi.nlm.nih.gov/entrez/query.fcgi?db=gene&cmd=Retrieve&dopt=full_report&list_uids=3845) | KRAS proto-oncogene, GTPase |
| [Details](http://mirdb.org/cgi-bin/target_detail.cgi?targetID=3073093) | 924 | 69 | hsa-miR-27a-3p | [SCN2A](http://www.ncbi.nlm.nih.gov/entrez/query.fcgi?db=gene&cmd=Retrieve&dopt=full_report&list_uids=6326) | sodium voltage-gated channel alpha subunit 2 |
| [Details](http://mirdb.org/cgi-bin/target_detail.cgi?targetID=3073107) | 925 | 69 | hsa-miR-27a-3p | [PLD6](http://www.ncbi.nlm.nih.gov/entrez/query.fcgi?db=gene&cmd=Retrieve&dopt=full_report&list_uids=201164) | phospholipase D family member 6 |
| [Details](http://mirdb.org/cgi-bin/target_detail.cgi?targetID=3073117) | 926 | 69 | hsa-miR-27a-3p | [KRTAP13-2](http://www.ncbi.nlm.nih.gov/entrez/query.fcgi?db=gene&cmd=Retrieve&dopt=full_report&list_uids=337959) | keratin associated protein 13-2 |
| [Details](http://mirdb.org/cgi-bin/target_detail.cgi?targetID=3073118) | 927 | 69 | hsa-miR-27a-3p | [CDC25B](http://www.ncbi.nlm.nih.gov/entrez/query.fcgi?db=gene&cmd=Retrieve&dopt=full_report&list_uids=994) | cell division cycle 25B |
| [Details](http://mirdb.org/cgi-bin/target_detail.cgi?targetID=3073198) | 928 | 69 | hsa-miR-27a-3p | [SEMA4C](http://www.ncbi.nlm.nih.gov/entrez/query.fcgi?db=gene&cmd=Retrieve&dopt=full_report&list_uids=54910) | semaphorin 4C |
| [Details](http://mirdb.org/cgi-bin/target_detail.cgi?targetID=3073204) | 929 | 69 | hsa-miR-27a-3p | [HRASLS5](http://www.ncbi.nlm.nih.gov/entrez/query.fcgi?db=gene&cmd=Retrieve&dopt=full_report&list_uids=117245) | HRAS like suppressor family member 5 |
| [Details](http://mirdb.org/cgi-bin/target_detail.cgi?targetID=3073228) | 930 | 69 | hsa-miR-27a-3p | [GSK3B](http://www.ncbi.nlm.nih.gov/entrez/query.fcgi?db=gene&cmd=Retrieve&dopt=full_report&list_uids=2932) | glycogen synthase kinase 3 beta |
| [Details](http://mirdb.org/cgi-bin/target_detail.cgi?targetID=3073249) | 931 | 69 | hsa-miR-27a-3p | [VPS26B](http://www.ncbi.nlm.nih.gov/entrez/query.fcgi?db=gene&cmd=Retrieve&dopt=full_report&list_uids=112936) | VPS26, retromer complex component B |
| [Details](http://mirdb.org/cgi-bin/target_detail.cgi?targetID=3073263) | 932 | 69 | hsa-miR-27a-3p | [KLHDC3](http://www.ncbi.nlm.nih.gov/entrez/query.fcgi?db=gene&cmd=Retrieve&dopt=full_report&list_uids=116138) | kelch domain containing 3 |
| [Details](http://mirdb.org/cgi-bin/target_detail.cgi?targetID=3073306) | 933 | 69 | hsa-miR-27a-3p | [CCNT1](http://www.ncbi.nlm.nih.gov/entrez/query.fcgi?db=gene&cmd=Retrieve&dopt=full_report&list_uids=904) | cyclin T1 |
| [Details](http://mirdb.org/cgi-bin/target_detail.cgi?targetID=3073462) | 934 | 69 | hsa-miR-27a-3p | [HCN4](http://www.ncbi.nlm.nih.gov/entrez/query.fcgi?db=gene&cmd=Retrieve&dopt=full_report&list_uids=10021) | hyperpolarization activated cyclic nucleotide gated potassium channel 4 |
| [Details](http://mirdb.org/cgi-bin/target_detail.cgi?targetID=3073474) | 935 | 69 | hsa-miR-27a-3p | [GOSR2](http://www.ncbi.nlm.nih.gov/entrez/query.fcgi?db=gene&cmd=Retrieve&dopt=full_report&list_uids=9570) | golgi SNAP receptor complex member 2 |
| [Details](http://mirdb.org/cgi-bin/target_detail.cgi?targetID=3073486) | 936 | 69 | hsa-miR-27a-3p | [DPYD](http://www.ncbi.nlm.nih.gov/entrez/query.fcgi?db=gene&cmd=Retrieve&dopt=full_report&list_uids=1806) | dihydropyrimidine dehydrogenase |
| [Details](http://mirdb.org/cgi-bin/target_detail.cgi?targetID=3073503) | 937 | 69 | hsa-miR-27a-3p | [PIKFYVE](http://www.ncbi.nlm.nih.gov/entrez/query.fcgi?db=gene&cmd=Retrieve&dopt=full_report&list_uids=200576) | phosphoinositide kinase, FYVE-type zinc finger containing |
| [Details](http://mirdb.org/cgi-bin/target_detail.cgi?targetID=3073538) | 938 | 69 | hsa-miR-27a-3p | [TRABD2B](http://www.ncbi.nlm.nih.gov/entrez/query.fcgi?db=gene&cmd=Retrieve&dopt=full_report&list_uids=388630) | TraB domain containing 2B |
| [Details](http://mirdb.org/cgi-bin/target_detail.cgi?targetID=3073543) | 939 | 69 | hsa-miR-27a-3p | [JDP2](http://www.ncbi.nlm.nih.gov/entrez/query.fcgi?db=gene&cmd=Retrieve&dopt=full_report&list_uids=122953) | Jun dimerization protein 2 |
| [Details](http://mirdb.org/cgi-bin/target_detail.cgi?targetID=3073685) | 940 | 69 | hsa-miR-27a-3p | [DLGAP3](http://www.ncbi.nlm.nih.gov/entrez/query.fcgi?db=gene&cmd=Retrieve&dopt=full_report&list_uids=58512) | DLG associated protein 3 |
| [Details](http://mirdb.org/cgi-bin/target_detail.cgi?targetID=3073690) | 941 | 69 | hsa-miR-27a-3p | [PRR16](http://www.ncbi.nlm.nih.gov/entrez/query.fcgi?db=gene&cmd=Retrieve&dopt=full_report&list_uids=51334) | proline rich 16 |
| [Details](http://mirdb.org/cgi-bin/target_detail.cgi?targetID=3073881) | 942 | 69 | hsa-miR-27a-3p | [SYNPO2](http://www.ncbi.nlm.nih.gov/entrez/query.fcgi?db=gene&cmd=Retrieve&dopt=full_report&list_uids=171024) | synaptopodin 2 |
| [Details](http://mirdb.org/cgi-bin/target_detail.cgi?targetID=3073885) | 943 | 69 | hsa-miR-27a-3p | [NFKBID](http://www.ncbi.nlm.nih.gov/entrez/query.fcgi?db=gene&cmd=Retrieve&dopt=full_report&list_uids=84807) | NFKB inhibitor delta |
| [Details](http://mirdb.org/cgi-bin/target_detail.cgi?targetID=3072516) | 944 | 68 | hsa-miR-27a-3p | [SELENON](http://www.ncbi.nlm.nih.gov/entrez/query.fcgi?db=gene&cmd=Retrieve&dopt=full_report&list_uids=57190) | selenoprotein N |
| [Details](http://mirdb.org/cgi-bin/target_detail.cgi?targetID=3072533) | 945 | 68 | hsa-miR-27a-3p | [PAK6](http://www.ncbi.nlm.nih.gov/entrez/query.fcgi?db=gene&cmd=Retrieve&dopt=full_report&list_uids=56924) | p21 (RAC1) activated kinase 6 |
| [Details](http://mirdb.org/cgi-bin/target_detail.cgi?targetID=3072553) | 946 | 68 | hsa-miR-27a-3p | [ELL2](http://www.ncbi.nlm.nih.gov/entrez/query.fcgi?db=gene&cmd=Retrieve&dopt=full_report&list_uids=22936) | elongation factor for RNA polymerase II 2 |
| [Details](http://mirdb.org/cgi-bin/target_detail.cgi?targetID=3072604) | 947 | 68 | hsa-miR-27a-3p | [MSI2](http://www.ncbi.nlm.nih.gov/entrez/query.fcgi?db=gene&cmd=Retrieve&dopt=full_report&list_uids=124540) | musashi RNA binding protein 2 |
| [Details](http://mirdb.org/cgi-bin/target_detail.cgi?targetID=3072645) | 948 | 68 | hsa-miR-27a-3p | [SENP1](http://www.ncbi.nlm.nih.gov/entrez/query.fcgi?db=gene&cmd=Retrieve&dopt=full_report&list_uids=29843) | SUMO specific peptidase 1 |
| [Details](http://mirdb.org/cgi-bin/target_detail.cgi?targetID=3072790) | 949 | 68 | hsa-miR-27a-3p | [CSNK1D](http://www.ncbi.nlm.nih.gov/entrez/query.fcgi?db=gene&cmd=Retrieve&dopt=full_report&list_uids=1453) | casein kinase 1 delta |
| [Details](http://mirdb.org/cgi-bin/target_detail.cgi?targetID=3072857) | 950 | 68 | hsa-miR-27a-3p | [GTF2I](http://www.ncbi.nlm.nih.gov/entrez/query.fcgi?db=gene&cmd=Retrieve&dopt=full_report&list_uids=2969) | general transcription factor IIi |
| [Details](http://mirdb.org/cgi-bin/target_detail.cgi?targetID=3072922) | 951 | 68 | hsa-miR-27a-3p | [SMIM17](http://www.ncbi.nlm.nih.gov/entrez/query.fcgi?db=gene&cmd=Retrieve&dopt=full_report&list_uids=147670) | small integral membrane protein 17 |
| [Details](http://mirdb.org/cgi-bin/target_detail.cgi?targetID=3073015) | 952 | 68 | hsa-miR-27a-3p | [YPEL3](http://www.ncbi.nlm.nih.gov/entrez/query.fcgi?db=gene&cmd=Retrieve&dopt=full_report&list_uids=83719) | yippee like 3 |
| [Details](http://mirdb.org/cgi-bin/target_detail.cgi?targetID=3073036) | 953 | 68 | hsa-miR-27a-3p | [C14orf93](http://www.ncbi.nlm.nih.gov/entrez/query.fcgi?db=gene&cmd=Retrieve&dopt=full_report&list_uids=60686) | chromosome 14 open reading frame 93 |
| [Details](http://mirdb.org/cgi-bin/target_detail.cgi?targetID=3073052) | 954 | 68 | hsa-miR-27a-3p | [ASB7](http://www.ncbi.nlm.nih.gov/entrez/query.fcgi?db=gene&cmd=Retrieve&dopt=full_report&list_uids=140460) | ankyrin repeat and SOCS box containing 7 |
| [Details](http://mirdb.org/cgi-bin/target_detail.cgi?targetID=3073116) | 955 | 68 | hsa-miR-27a-3p | [FAM200B](http://www.ncbi.nlm.nih.gov/entrez/query.fcgi?db=gene&cmd=Retrieve&dopt=full_report&list_uids=285550) | family with sequence similarity 200 member B |
| [Details](http://mirdb.org/cgi-bin/target_detail.cgi?targetID=3073125) | 956 | 68 | hsa-miR-27a-3p | [ZNF81](http://www.ncbi.nlm.nih.gov/entrez/query.fcgi?db=gene&cmd=Retrieve&dopt=full_report&list_uids=347344) | zinc finger protein 81 |
| [Details](http://mirdb.org/cgi-bin/target_detail.cgi?targetID=3073166) | 957 | 68 | hsa-miR-27a-3p | [LONRF2](http://www.ncbi.nlm.nih.gov/entrez/query.fcgi?db=gene&cmd=Retrieve&dopt=full_report&list_uids=164832) | LON peptidase N-terminal domain and ring finger 2 |
| [Details](http://mirdb.org/cgi-bin/target_detail.cgi?targetID=3073280) | 958 | 68 | hsa-miR-27a-3p | [ARID2](http://www.ncbi.nlm.nih.gov/entrez/query.fcgi?db=gene&cmd=Retrieve&dopt=full_report&list_uids=196528) | AT-rich interaction domain 2 |
| [Details](http://mirdb.org/cgi-bin/target_detail.cgi?targetID=3073370) | 959 | 68 | hsa-miR-27a-3p | [GRP](http://www.ncbi.nlm.nih.gov/entrez/query.fcgi?db=gene&cmd=Retrieve&dopt=full_report&list_uids=2922) | gastrin releasing peptide |
| [Details](http://mirdb.org/cgi-bin/target_detail.cgi?targetID=3073404) | 960 | 68 | hsa-miR-27a-3p | [PTAR1](http://www.ncbi.nlm.nih.gov/entrez/query.fcgi?db=gene&cmd=Retrieve&dopt=full_report&list_uids=375743) | protein prenyltransferase alpha subunit repeat containing 1 |
| [Details](http://mirdb.org/cgi-bin/target_detail.cgi?targetID=3073418) | 961 | 68 | hsa-miR-27a-3p | [SLC5A3](http://www.ncbi.nlm.nih.gov/entrez/query.fcgi?db=gene&cmd=Retrieve&dopt=full_report&list_uids=6526) | solute carrier family 5 member 3 |
| [Details](http://mirdb.org/cgi-bin/target_detail.cgi?targetID=3073517) | 962 | 68 | hsa-miR-27a-3p | [RFC3](http://www.ncbi.nlm.nih.gov/entrez/query.fcgi?db=gene&cmd=Retrieve&dopt=full_report&list_uids=5983) | replication factor C subunit 3 |
| [Details](http://mirdb.org/cgi-bin/target_detail.cgi?targetID=3073559) | 963 | 68 | hsa-miR-27a-3p | [UBE2W](http://www.ncbi.nlm.nih.gov/entrez/query.fcgi?db=gene&cmd=Retrieve&dopt=full_report&list_uids=55284) | ubiquitin conjugating enzyme E2 W |
| [Details](http://mirdb.org/cgi-bin/target_detail.cgi?targetID=3073579) | 964 | 68 | hsa-miR-27a-3p | [CDC14A](http://www.ncbi.nlm.nih.gov/entrez/query.fcgi?db=gene&cmd=Retrieve&dopt=full_report&list_uids=8556) | cell division cycle 14A |
| [Details](http://mirdb.org/cgi-bin/target_detail.cgi?targetID=3073633) | 965 | 68 | hsa-miR-27a-3p | [IL6ST](http://www.ncbi.nlm.nih.gov/entrez/query.fcgi?db=gene&cmd=Retrieve&dopt=full_report&list_uids=3572) | interleukin 6 signal transducer |
| [Details](http://mirdb.org/cgi-bin/target_detail.cgi?targetID=3073673) | 966 | 68 | hsa-miR-27a-3p | [RBMXL1](http://www.ncbi.nlm.nih.gov/entrez/query.fcgi?db=gene&cmd=Retrieve&dopt=full_report&list_uids=494115) | RBMX like 1 |
| [Details](http://mirdb.org/cgi-bin/target_detail.cgi?targetID=3073679) | 967 | 68 | hsa-miR-27a-3p | [GLYATL1](http://www.ncbi.nlm.nih.gov/entrez/query.fcgi?db=gene&cmd=Retrieve&dopt=full_report&list_uids=92292) | glycine-N-acyltransferase like 1 |
| [Details](http://mirdb.org/cgi-bin/target_detail.cgi?targetID=3073752) | 968 | 68 | hsa-miR-27a-3p | [WDR35](http://www.ncbi.nlm.nih.gov/entrez/query.fcgi?db=gene&cmd=Retrieve&dopt=full_report&list_uids=57539) | WD repeat domain 35 |
| [Details](http://mirdb.org/cgi-bin/target_detail.cgi?targetID=3073756) | 969 | 68 | hsa-miR-27a-3p | [WEE1](http://www.ncbi.nlm.nih.gov/entrez/query.fcgi?db=gene&cmd=Retrieve&dopt=full_report&list_uids=7465) | WEE1 G2 checkpoint kinase |
| [Details](http://mirdb.org/cgi-bin/target_detail.cgi?targetID=3073782) | 970 | 68 | hsa-miR-27a-3p | [GALNT12](http://www.ncbi.nlm.nih.gov/entrez/query.fcgi?db=gene&cmd=Retrieve&dopt=full_report&list_uids=79695) | polypeptide N-acetylgalactosaminyltransferase 12 |
| [Details](http://mirdb.org/cgi-bin/target_detail.cgi?targetID=3073888) | 971 | 68 | hsa-miR-27a-3p | [TDP1](http://www.ncbi.nlm.nih.gov/entrez/query.fcgi?db=gene&cmd=Retrieve&dopt=full_report&list_uids=55775) | tyrosyl-DNA phosphodiesterase 1 |
| [Details](http://mirdb.org/cgi-bin/target_detail.cgi?targetID=3072430) | 972 | 67 | hsa-miR-27a-3p | [ATP13A3](http://www.ncbi.nlm.nih.gov/entrez/query.fcgi?db=gene&cmd=Retrieve&dopt=full_report&list_uids=79572) | ATPase 13A3 |
| [Details](http://mirdb.org/cgi-bin/target_detail.cgi?targetID=3072507) | 973 | 67 | hsa-miR-27a-3p | [EEPD1](http://www.ncbi.nlm.nih.gov/entrez/query.fcgi?db=gene&cmd=Retrieve&dopt=full_report&list_uids=80820) | endonuclease/exonuclease/phosphatase family domain containing 1 |
| [Details](http://mirdb.org/cgi-bin/target_detail.cgi?targetID=3072647) | 974 | 67 | hsa-miR-27a-3p | [ACACA](http://www.ncbi.nlm.nih.gov/entrez/query.fcgi?db=gene&cmd=Retrieve&dopt=full_report&list_uids=31) | acetyl-CoA carboxylase alpha |
| [Details](http://mirdb.org/cgi-bin/target_detail.cgi?targetID=3072714) | 975 | 67 | hsa-miR-27a-3p | [CTCF](http://www.ncbi.nlm.nih.gov/entrez/query.fcgi?db=gene&cmd=Retrieve&dopt=full_report&list_uids=10664) | CCCTC-binding factor |
| [Details](http://mirdb.org/cgi-bin/target_detail.cgi?targetID=3072757) | 976 | 67 | hsa-miR-27a-3p | [ANKRD17](http://www.ncbi.nlm.nih.gov/entrez/query.fcgi?db=gene&cmd=Retrieve&dopt=full_report&list_uids=26057) | ankyrin repeat domain 17 |
| [Details](http://mirdb.org/cgi-bin/target_detail.cgi?targetID=3072869) | 977 | 67 | hsa-miR-27a-3p | [ARID5B](http://www.ncbi.nlm.nih.gov/entrez/query.fcgi?db=gene&cmd=Retrieve&dopt=full_report&list_uids=84159) | AT-rich interaction domain 5B |
| [Details](http://mirdb.org/cgi-bin/target_detail.cgi?targetID=3072940) | 978 | 67 | hsa-miR-27a-3p | [DDAH1](http://www.ncbi.nlm.nih.gov/entrez/query.fcgi?db=gene&cmd=Retrieve&dopt=full_report&list_uids=23576) | dimethylarginine dimethylaminohydrolase 1 |
| [Details](http://mirdb.org/cgi-bin/target_detail.cgi?targetID=3072973) | 979 | 67 | hsa-miR-27a-3p | [BCL3](http://www.ncbi.nlm.nih.gov/entrez/query.fcgi?db=gene&cmd=Retrieve&dopt=full_report&list_uids=602) | BCL3, transcription coactivator |
| [Details](http://mirdb.org/cgi-bin/target_detail.cgi?targetID=3073074) | 980 | 67 | hsa-miR-27a-3p | [MED13](http://www.ncbi.nlm.nih.gov/entrez/query.fcgi?db=gene&cmd=Retrieve&dopt=full_report&list_uids=9969) | mediator complex subunit 13 |
| [Details](http://mirdb.org/cgi-bin/target_detail.cgi?targetID=3073147) | 981 | 67 | hsa-miR-27a-3p | [PRDM11](http://www.ncbi.nlm.nih.gov/entrez/query.fcgi?db=gene&cmd=Retrieve&dopt=full_report&list_uids=56981) | PR/SET domain 11 |
| [Details](http://mirdb.org/cgi-bin/target_detail.cgi?targetID=3073365) | 982 | 67 | hsa-miR-27a-3p | [UAP1](http://www.ncbi.nlm.nih.gov/entrez/query.fcgi?db=gene&cmd=Retrieve&dopt=full_report&list_uids=6675) | UDP-N-acetylglucosamine pyrophosphorylase 1 |
| [Details](http://mirdb.org/cgi-bin/target_detail.cgi?targetID=3073401) | 983 | 67 | hsa-miR-27a-3p | [E2F6](http://www.ncbi.nlm.nih.gov/entrez/query.fcgi?db=gene&cmd=Retrieve&dopt=full_report&list_uids=1876) | E2F transcription factor 6 |
| [Details](http://mirdb.org/cgi-bin/target_detail.cgi?targetID=3073432) | 984 | 67 | hsa-miR-27a-3p | [TRIM2](http://www.ncbi.nlm.nih.gov/entrez/query.fcgi?db=gene&cmd=Retrieve&dopt=full_report&list_uids=23321) | tripartite motif containing 2 |
| [Details](http://mirdb.org/cgi-bin/target_detail.cgi?targetID=3073515) | 985 | 67 | hsa-miR-27a-3p | [PPM1B](http://www.ncbi.nlm.nih.gov/entrez/query.fcgi?db=gene&cmd=Retrieve&dopt=full_report&list_uids=5495) | protein phosphatase, Mg2+/Mn2+ dependent 1B |
| [Details](http://mirdb.org/cgi-bin/target_detail.cgi?targetID=3073668) | 986 | 67 | hsa-miR-27a-3p | [CASC3](http://www.ncbi.nlm.nih.gov/entrez/query.fcgi?db=gene&cmd=Retrieve&dopt=full_report&list_uids=22794) | CASC3, exon junction complex subunit |
| [Details](http://mirdb.org/cgi-bin/target_detail.cgi?targetID=3073706) | 987 | 67 | hsa-miR-27a-3p | [KCTD4](http://www.ncbi.nlm.nih.gov/entrez/query.fcgi?db=gene&cmd=Retrieve&dopt=full_report&list_uids=386618) | potassium channel tetramerization domain containing 4 |
| [Details](http://mirdb.org/cgi-bin/target_detail.cgi?targetID=3073739) | 988 | 67 | hsa-miR-27a-3p | [IGLON5](http://www.ncbi.nlm.nih.gov/entrez/query.fcgi?db=gene&cmd=Retrieve&dopt=full_report&list_uids=402665) | IgLON family member 5 |
| [Details](http://mirdb.org/cgi-bin/target_detail.cgi?targetID=3073778) | 989 | 67 | hsa-miR-27a-3p | [ZNF783](http://www.ncbi.nlm.nih.gov/entrez/query.fcgi?db=gene&cmd=Retrieve&dopt=full_report&list_uids=100289678) | zinc finger family member 783 |
| [Details](http://mirdb.org/cgi-bin/target_detail.cgi?targetID=3073828) | 990 | 67 | hsa-miR-27a-3p | [SUMO2](http://www.ncbi.nlm.nih.gov/entrez/query.fcgi?db=gene&cmd=Retrieve&dopt=full_report&list_uids=6613) | small ubiquitin-like modifier 2 |
| [Details](http://mirdb.org/cgi-bin/target_detail.cgi?targetID=3073893) | 991 | 67 | hsa-miR-27a-3p | [SHB](http://www.ncbi.nlm.nih.gov/entrez/query.fcgi?db=gene&cmd=Retrieve&dopt=full_report&list_uids=6461) | SH2 domain containing adaptor protein B |
| [Details](http://mirdb.org/cgi-bin/target_detail.cgi?targetID=3072431) | 992 | 66 | hsa-miR-27a-3p | [PPM1K](http://www.ncbi.nlm.nih.gov/entrez/query.fcgi?db=gene&cmd=Retrieve&dopt=full_report&list_uids=152926) | protein phosphatase, Mg2+/Mn2+ dependent 1K |
| [Details](http://mirdb.org/cgi-bin/target_detail.cgi?targetID=3072487) | 993 | 66 | hsa-miR-27a-3p | [MRPS25](http://www.ncbi.nlm.nih.gov/entrez/query.fcgi?db=gene&cmd=Retrieve&dopt=full_report&list_uids=64432) | mitochondrial ribosomal protein S25 |
| [Details](http://mirdb.org/cgi-bin/target_detail.cgi?targetID=3072512) | 994 | 66 | hsa-miR-27a-3p | [LRRC8B](http://www.ncbi.nlm.nih.gov/entrez/query.fcgi?db=gene&cmd=Retrieve&dopt=full_report&list_uids=23507) | leucine rich repeat containing 8 VRAC subunit B |
| [Details](http://mirdb.org/cgi-bin/target_detail.cgi?targetID=3072513) | 995 | 66 | hsa-miR-27a-3p | [CHMP2B](http://www.ncbi.nlm.nih.gov/entrez/query.fcgi?db=gene&cmd=Retrieve&dopt=full_report&list_uids=25978) | charged multivesicular body protein 2B |
| [Details](http://mirdb.org/cgi-bin/target_detail.cgi?targetID=3072612) | 996 | 66 | hsa-miR-27a-3p | [NCKAP5](http://www.ncbi.nlm.nih.gov/entrez/query.fcgi?db=gene&cmd=Retrieve&dopt=full_report&list_uids=344148) | NCK associated protein 5 |
| [Details](http://mirdb.org/cgi-bin/target_detail.cgi?targetID=3072646) | 997 | 66 | hsa-miR-27a-3p | [N4BP2L1](http://www.ncbi.nlm.nih.gov/entrez/query.fcgi?db=gene&cmd=Retrieve&dopt=full_report&list_uids=90634) | NEDD4 binding protein 2 like 1 |
| [Details](http://mirdb.org/cgi-bin/target_detail.cgi?targetID=3072676) | 998 | 66 | hsa-miR-27a-3p | [CLEC1A](http://www.ncbi.nlm.nih.gov/entrez/query.fcgi?db=gene&cmd=Retrieve&dopt=full_report&list_uids=51267) | C-type lectin domain family 1 member A |
| [Details](http://mirdb.org/cgi-bin/target_detail.cgi?targetID=3072951) | 999 | 66 | hsa-miR-27a-3p | [LHX3](http://www.ncbi.nlm.nih.gov/entrez/query.fcgi?db=gene&cmd=Retrieve&dopt=full_report&list_uids=8022) | LIM homeobox 3 |
| [Details](http://mirdb.org/cgi-bin/target_detail.cgi?targetID=3072962) | 1000 | 66 | hsa-miR-27a-3p | [NFIB](http://www.ncbi.nlm.nih.gov/entrez/query.fcgi?db=gene&cmd=Retrieve&dopt=full_report&list_uids=4781) | nuclear factor I B |
| [Details](http://mirdb.org/cgi-bin/target_detail.cgi?targetID=3072971) | 1001 | 66 | hsa-miR-27a-3p | [LPIN2](http://www.ncbi.nlm.nih.gov/entrez/query.fcgi?db=gene&cmd=Retrieve&dopt=full_report&list_uids=9663) | lipin 2 |
| [Details](http://mirdb.org/cgi-bin/target_detail.cgi?targetID=3072984) | 1002 | 66 | hsa-miR-27a-3p | [UGT8](http://www.ncbi.nlm.nih.gov/entrez/query.fcgi?db=gene&cmd=Retrieve&dopt=full_report&list_uids=7368) | UDP glycosyltransferase 8 |
| [Details](http://mirdb.org/cgi-bin/target_detail.cgi?targetID=3072998) | 1003 | 66 | hsa-miR-27a-3p | [SLC12A2](http://www.ncbi.nlm.nih.gov/entrez/query.fcgi?db=gene&cmd=Retrieve&dopt=full_report&list_uids=6558) | solute carrier family 12 member 2 |
| [Details](http://mirdb.org/cgi-bin/target_detail.cgi?targetID=3073020) | 1004 | 66 | hsa-miR-27a-3p | [MICAL3](http://www.ncbi.nlm.nih.gov/entrez/query.fcgi?db=gene&cmd=Retrieve&dopt=full_report&list_uids=57553) | microtubule associated monooxygenase, calponin and LIM domain containing 3 |
| [Details](http://mirdb.org/cgi-bin/target_detail.cgi?targetID=3073034) | 1005 | 66 | hsa-miR-27a-3p | [UBE2F](http://www.ncbi.nlm.nih.gov/entrez/query.fcgi?db=gene&cmd=Retrieve&dopt=full_report&list_uids=140739) | ubiquitin conjugating enzyme E2 F (putative) |
| [Details](http://mirdb.org/cgi-bin/target_detail.cgi?targetID=3073065) | 1006 | 66 | hsa-miR-27a-3p | [C18orf65](http://www.ncbi.nlm.nih.gov/entrez/query.fcgi?db=gene&cmd=Retrieve&dopt=full_report&list_uids=400658) | chromosome 18 open reading frame 65 |
| [Details](http://mirdb.org/cgi-bin/target_detail.cgi?targetID=3073081) | 1007 | 66 | hsa-miR-27a-3p | [CNTNAP2](http://www.ncbi.nlm.nih.gov/entrez/query.fcgi?db=gene&cmd=Retrieve&dopt=full_report&list_uids=26047) | contactin associated protein like 2 |
| [Details](http://mirdb.org/cgi-bin/target_detail.cgi?targetID=3073109) | 1008 | 66 | hsa-miR-27a-3p | [TTC39B](http://www.ncbi.nlm.nih.gov/entrez/query.fcgi?db=gene&cmd=Retrieve&dopt=full_report&list_uids=158219) | tetratricopeptide repeat domain 39B |
| [Details](http://mirdb.org/cgi-bin/target_detail.cgi?targetID=3073367) | 1009 | 66 | hsa-miR-27a-3p | [ARSJ](http://www.ncbi.nlm.nih.gov/entrez/query.fcgi?db=gene&cmd=Retrieve&dopt=full_report&list_uids=79642) | arylsulfatase family member J |
| [Details](http://mirdb.org/cgi-bin/target_detail.cgi?targetID=3073422) | 1010 | 66 | hsa-miR-27a-3p | [ADD3](http://www.ncbi.nlm.nih.gov/entrez/query.fcgi?db=gene&cmd=Retrieve&dopt=full_report&list_uids=120) | adducin 3 |
| [Details](http://mirdb.org/cgi-bin/target_detail.cgi?targetID=3073571) | 1011 | 66 | hsa-miR-27a-3p | [CDS2](http://www.ncbi.nlm.nih.gov/entrez/query.fcgi?db=gene&cmd=Retrieve&dopt=full_report&list_uids=8760) | CDP-diacylglycerol synthase 2 |
| [Details](http://mirdb.org/cgi-bin/target_detail.cgi?targetID=3073621) | 1012 | 66 | hsa-miR-27a-3p | [EMCN](http://www.ncbi.nlm.nih.gov/entrez/query.fcgi?db=gene&cmd=Retrieve&dopt=full_report&list_uids=51705) | endomucin |
| [Details](http://mirdb.org/cgi-bin/target_detail.cgi?targetID=3073631) | 1013 | 66 | hsa-miR-27a-3p | [TET3](http://www.ncbi.nlm.nih.gov/entrez/query.fcgi?db=gene&cmd=Retrieve&dopt=full_report&list_uids=200424) | tet methylcytosine dioxygenase 3 |
| [Details](http://mirdb.org/cgi-bin/target_detail.cgi?targetID=3073678) | 1014 | 66 | hsa-miR-27a-3p | [MAN2A1](http://www.ncbi.nlm.nih.gov/entrez/query.fcgi?db=gene&cmd=Retrieve&dopt=full_report&list_uids=4124) | mannosidase alpha class 2A member 1 |
| [Details](http://mirdb.org/cgi-bin/target_detail.cgi?targetID=3073680) | 1015 | 66 | hsa-miR-27a-3p | [CDYL](http://www.ncbi.nlm.nih.gov/entrez/query.fcgi?db=gene&cmd=Retrieve&dopt=full_report&list_uids=9425) | chromodomain Y like |
| [Details](http://mirdb.org/cgi-bin/target_detail.cgi?targetID=3073789) | 1016 | 66 | hsa-miR-27a-3p | [IQCH](http://www.ncbi.nlm.nih.gov/entrez/query.fcgi?db=gene&cmd=Retrieve&dopt=full_report&list_uids=64799) | IQ motif containing H |
| [Details](http://mirdb.org/cgi-bin/target_detail.cgi?targetID=3073892) | 1017 | 66 | hsa-miR-27a-3p | [HLA-DRA](http://www.ncbi.nlm.nih.gov/entrez/query.fcgi?db=gene&cmd=Retrieve&dopt=full_report&list_uids=3122) | major histocompatibility complex, class II, DR alpha |
| [Details](http://mirdb.org/cgi-bin/target_detail.cgi?targetID=3073903) | 1018 | 66 | hsa-miR-27a-3p | [CHERP](http://www.ncbi.nlm.nih.gov/entrez/query.fcgi?db=gene&cmd=Retrieve&dopt=full_report&list_uids=10523) | calcium homeostasis endoplasmic reticulum protein |
| [Details](http://mirdb.org/cgi-bin/target_detail.cgi?targetID=3073916) | 1019 | 66 | hsa-miR-27a-3p | [UTP23](http://www.ncbi.nlm.nih.gov/entrez/query.fcgi?db=gene&cmd=Retrieve&dopt=full_report&list_uids=84294) | UTP23, small subunit processome component |
| [Details](http://mirdb.org/cgi-bin/target_detail.cgi?targetID=3072437) | 1020 | 65 | hsa-miR-27a-3p | [HNRNPF](http://www.ncbi.nlm.nih.gov/entrez/query.fcgi?db=gene&cmd=Retrieve&dopt=full_report&list_uids=3185) | heterogeneous nuclear ribonucleoprotein F |
| [Details](http://mirdb.org/cgi-bin/target_detail.cgi?targetID=3072478) | 1021 | 65 | hsa-miR-27a-3p | [ALG10](http://www.ncbi.nlm.nih.gov/entrez/query.fcgi?db=gene&cmd=Retrieve&dopt=full_report&list_uids=84920) | ALG10, alpha-1,2-glucosyltransferase |
| [Details](http://mirdb.org/cgi-bin/target_detail.cgi?targetID=3072511) | 1022 | 65 | hsa-miR-27a-3p | [SV2C](http://www.ncbi.nlm.nih.gov/entrez/query.fcgi?db=gene&cmd=Retrieve&dopt=full_report&list_uids=22987) | synaptic vesicle glycoprotein 2C |
| [Details](http://mirdb.org/cgi-bin/target_detail.cgi?targetID=3072528) | 1023 | 65 | hsa-miR-27a-3p | [HIPK3](http://www.ncbi.nlm.nih.gov/entrez/query.fcgi?db=gene&cmd=Retrieve&dopt=full_report&list_uids=10114) | homeodomain interacting protein kinase 3 |
| [Details](http://mirdb.org/cgi-bin/target_detail.cgi?targetID=3072554) | 1024 | 65 | hsa-miR-27a-3p | [STIMATE](http://www.ncbi.nlm.nih.gov/entrez/query.fcgi?db=gene&cmd=Retrieve&dopt=full_report&list_uids=375346) | STIM activating enhancer |
| [Details](http://mirdb.org/cgi-bin/target_detail.cgi?targetID=3072564) | 1025 | 65 | hsa-miR-27a-3p | [FSIP1](http://www.ncbi.nlm.nih.gov/entrez/query.fcgi?db=gene&cmd=Retrieve&dopt=full_report&list_uids=161835) | fibrous sheath interacting protein 1 |
| [Details](http://mirdb.org/cgi-bin/target_detail.cgi?targetID=3072623) | 1026 | 65 | hsa-miR-27a-3p | [RGPD3](http://www.ncbi.nlm.nih.gov/entrez/query.fcgi?db=gene&cmd=Retrieve&dopt=full_report&list_uids=653489) | RANBP2-like and GRIP domain containing 3 |
| [Details](http://mirdb.org/cgi-bin/target_detail.cgi?targetID=3072762) | 1027 | 65 | hsa-miR-27a-3p | [FBXO33](http://www.ncbi.nlm.nih.gov/entrez/query.fcgi?db=gene&cmd=Retrieve&dopt=full_report&list_uids=254170) | F-box protein 33 |
| [Details](http://mirdb.org/cgi-bin/target_detail.cgi?targetID=3072804) | 1028 | 65 | hsa-miR-27a-3p | [CEP85L](http://www.ncbi.nlm.nih.gov/entrez/query.fcgi?db=gene&cmd=Retrieve&dopt=full_report&list_uids=387119) | centrosomal protein 85 like |
| [Details](http://mirdb.org/cgi-bin/target_detail.cgi?targetID=3072846) | 1029 | 65 | hsa-miR-27a-3p | [MPP7](http://www.ncbi.nlm.nih.gov/entrez/query.fcgi?db=gene&cmd=Retrieve&dopt=full_report&list_uids=143098) | membrane palmitoylated protein 7 |
| [Details](http://mirdb.org/cgi-bin/target_detail.cgi?targetID=3072956) | 1030 | 65 | hsa-miR-27a-3p | [RPN1](http://www.ncbi.nlm.nih.gov/entrez/query.fcgi?db=gene&cmd=Retrieve&dopt=full_report&list_uids=6184) | ribophorin I |
| [Details](http://mirdb.org/cgi-bin/target_detail.cgi?targetID=3073096) | 1031 | 65 | hsa-miR-27a-3p | [ARHGAP5](http://www.ncbi.nlm.nih.gov/entrez/query.fcgi?db=gene&cmd=Retrieve&dopt=full_report&list_uids=394) | Rho GTPase activating protein 5 |
| [Details](http://mirdb.org/cgi-bin/target_detail.cgi?targetID=3073148) | 1032 | 65 | hsa-miR-27a-3p | [ZNF106](http://www.ncbi.nlm.nih.gov/entrez/query.fcgi?db=gene&cmd=Retrieve&dopt=full_report&list_uids=64397) | zinc finger protein 106 |
| [Details](http://mirdb.org/cgi-bin/target_detail.cgi?targetID=3073154) | 1033 | 65 | hsa-miR-27a-3p | [RGS17](http://www.ncbi.nlm.nih.gov/entrez/query.fcgi?db=gene&cmd=Retrieve&dopt=full_report&list_uids=26575) | regulator of G protein signaling 17 |
| [Details](http://mirdb.org/cgi-bin/target_detail.cgi?targetID=3073176) | 1034 | 65 | hsa-miR-27a-3p | [ANKRD36C](http://www.ncbi.nlm.nih.gov/entrez/query.fcgi?db=gene&cmd=Retrieve&dopt=full_report&list_uids=400986) | ankyrin repeat domain 36C |
| [Details](http://mirdb.org/cgi-bin/target_detail.cgi?targetID=3073224) | 1035 | 65 | hsa-miR-27a-3p | [PAQR5](http://www.ncbi.nlm.nih.gov/entrez/query.fcgi?db=gene&cmd=Retrieve&dopt=full_report&list_uids=54852) | progestin and adipoQ receptor family member 5 |
| [Details](http://mirdb.org/cgi-bin/target_detail.cgi?targetID=3073242) | 1036 | 65 | hsa-miR-27a-3p | [SEPT11](http://www.ncbi.nlm.nih.gov/entrez/query.fcgi?db=gene&cmd=Retrieve&dopt=full_report&list_uids=55752) | septin 11 |
| [Details](http://mirdb.org/cgi-bin/target_detail.cgi?targetID=3073250) | 1037 | 65 | hsa-miR-27a-3p | [ARL13B](http://www.ncbi.nlm.nih.gov/entrez/query.fcgi?db=gene&cmd=Retrieve&dopt=full_report&list_uids=200894) | ADP ribosylation factor like GTPase 13B |
| [Details](http://mirdb.org/cgi-bin/target_detail.cgi?targetID=3073287) | 1038 | 65 | hsa-miR-27a-3p | [HGF](http://www.ncbi.nlm.nih.gov/entrez/query.fcgi?db=gene&cmd=Retrieve&dopt=full_report&list_uids=3082) | hepatocyte growth factor |
| [Details](http://mirdb.org/cgi-bin/target_detail.cgi?targetID=3073303) | 1039 | 65 | hsa-miR-27a-3p | [CD2AP](http://www.ncbi.nlm.nih.gov/entrez/query.fcgi?db=gene&cmd=Retrieve&dopt=full_report&list_uids=23607) | CD2 associated protein |
| [Details](http://mirdb.org/cgi-bin/target_detail.cgi?targetID=3073339) | 1040 | 65 | hsa-miR-27a-3p | [TMEM126B](http://www.ncbi.nlm.nih.gov/entrez/query.fcgi?db=gene&cmd=Retrieve&dopt=full_report&list_uids=55863) | transmembrane protein 126B |
| [Details](http://mirdb.org/cgi-bin/target_detail.cgi?targetID=3073366) | 1041 | 65 | hsa-miR-27a-3p | [SNX30](http://www.ncbi.nlm.nih.gov/entrez/query.fcgi?db=gene&cmd=Retrieve&dopt=full_report&list_uids=401548) | sorting nexin family member 30 |
| [Details](http://mirdb.org/cgi-bin/target_detail.cgi?targetID=3073407) | 1042 | 65 | hsa-miR-27a-3p | [RAD54L2](http://www.ncbi.nlm.nih.gov/entrez/query.fcgi?db=gene&cmd=Retrieve&dopt=full_report&list_uids=23132) | RAD54 like 2 |
| [Details](http://mirdb.org/cgi-bin/target_detail.cgi?targetID=3073464) | 1043 | 65 | hsa-miR-27a-3p | [GYS1](http://www.ncbi.nlm.nih.gov/entrez/query.fcgi?db=gene&cmd=Retrieve&dopt=full_report&list_uids=2997) | glycogen synthase 1 |
| [Details](http://mirdb.org/cgi-bin/target_detail.cgi?targetID=3073742) | 1044 | 65 | hsa-miR-27a-3p | [PM20D1](http://www.ncbi.nlm.nih.gov/entrez/query.fcgi?db=gene&cmd=Retrieve&dopt=full_report&list_uids=148811) | peptidase M20 domain containing 1 |
| [Details](http://mirdb.org/cgi-bin/target_detail.cgi?targetID=3073786) | 1045 | 65 | hsa-miR-27a-3p | [ARL4D](http://www.ncbi.nlm.nih.gov/entrez/query.fcgi?db=gene&cmd=Retrieve&dopt=full_report&list_uids=379) | ADP ribosylation factor like GTPase 4D |
| [Details](http://mirdb.org/cgi-bin/target_detail.cgi?targetID=3073864) | 1046 | 65 | hsa-miR-27a-3p | [SYS1](http://www.ncbi.nlm.nih.gov/entrez/query.fcgi?db=gene&cmd=Retrieve&dopt=full_report&list_uids=90196) | SYS1, golgi trafficking protein |
| [Details](http://mirdb.org/cgi-bin/target_detail.cgi?targetID=3073869) | 1047 | 65 | hsa-miR-27a-3p | [SFXN4](http://www.ncbi.nlm.nih.gov/entrez/query.fcgi?db=gene&cmd=Retrieve&dopt=full_report&list_uids=119559) | sideroflexin 4 |
| [Details](http://mirdb.org/cgi-bin/target_detail.cgi?targetID=3073871) | 1048 | 65 | hsa-miR-27a-3p | [MAGEC1](http://www.ncbi.nlm.nih.gov/entrez/query.fcgi?db=gene&cmd=Retrieve&dopt=full_report&list_uids=9947) | MAGE family member C1 |
| [Details](http://mirdb.org/cgi-bin/target_detail.cgi?targetID=3072521) | 1049 | 64 | hsa-miR-27a-3p | [SCN9A](http://www.ncbi.nlm.nih.gov/entrez/query.fcgi?db=gene&cmd=Retrieve&dopt=full_report&list_uids=6335) | sodium voltage-gated channel alpha subunit 9 |
| [Details](http://mirdb.org/cgi-bin/target_detail.cgi?targetID=3072596) | 1050 | 64 | hsa-miR-27a-3p | [SP6](http://www.ncbi.nlm.nih.gov/entrez/query.fcgi?db=gene&cmd=Retrieve&dopt=full_report&list_uids=80320) | Sp6 transcription factor |
| [Details](http://mirdb.org/cgi-bin/target_detail.cgi?targetID=3072602) | 1051 | 64 | hsa-miR-27a-3p | [CSMD1](http://www.ncbi.nlm.nih.gov/entrez/query.fcgi?db=gene&cmd=Retrieve&dopt=full_report&list_uids=64478) | CUB and Sushi multiple domains 1 |
| [Details](http://mirdb.org/cgi-bin/target_detail.cgi?targetID=3072610) | 1052 | 64 | hsa-miR-27a-3p | [CLEC12A](http://www.ncbi.nlm.nih.gov/entrez/query.fcgi?db=gene&cmd=Retrieve&dopt=full_report&list_uids=160364) | C-type lectin domain family 12 member A |
| [Details](http://mirdb.org/cgi-bin/target_detail.cgi?targetID=3072611) | 1053 | 64 | hsa-miR-27a-3p | [DMXL2](http://www.ncbi.nlm.nih.gov/entrez/query.fcgi?db=gene&cmd=Retrieve&dopt=full_report&list_uids=23312) | Dmx like 2 |
| [Details](http://mirdb.org/cgi-bin/target_detail.cgi?targetID=3072661) | 1054 | 64 | hsa-miR-27a-3p | [NCOA3](http://www.ncbi.nlm.nih.gov/entrez/query.fcgi?db=gene&cmd=Retrieve&dopt=full_report&list_uids=8202) | nuclear receptor coactivator 3 |
| [Details](http://mirdb.org/cgi-bin/target_detail.cgi?targetID=3072685) | 1055 | 64 | hsa-miR-27a-3p | [NEUROD6](http://www.ncbi.nlm.nih.gov/entrez/query.fcgi?db=gene&cmd=Retrieve&dopt=full_report&list_uids=63974) | neuronal differentiation 6 |
| [Details](http://mirdb.org/cgi-bin/target_detail.cgi?targetID=3072710) | 1056 | 64 | hsa-miR-27a-3p | [TMUB1](http://www.ncbi.nlm.nih.gov/entrez/query.fcgi?db=gene&cmd=Retrieve&dopt=full_report&list_uids=83590) | transmembrane and ubiquitin like domain containing 1 |
| [Details](http://mirdb.org/cgi-bin/target_detail.cgi?targetID=3072756) | 1057 | 64 | hsa-miR-27a-3p | [DHTKD1](http://www.ncbi.nlm.nih.gov/entrez/query.fcgi?db=gene&cmd=Retrieve&dopt=full_report&list_uids=55526) | dehydrogenase E1 and transketolase domain containing 1 |
| [Details](http://mirdb.org/cgi-bin/target_detail.cgi?targetID=3072758) | 1058 | 64 | hsa-miR-27a-3p | [BAG2](http://www.ncbi.nlm.nih.gov/entrez/query.fcgi?db=gene&cmd=Retrieve&dopt=full_report&list_uids=9532) | BCL2 associated athanogene 2 |
| [Details](http://mirdb.org/cgi-bin/target_detail.cgi?targetID=3072765) | 1059 | 64 | hsa-miR-27a-3p | [SLC4A8](http://www.ncbi.nlm.nih.gov/entrez/query.fcgi?db=gene&cmd=Retrieve&dopt=full_report&list_uids=9498) | solute carrier family 4 member 8 |
| [Details](http://mirdb.org/cgi-bin/target_detail.cgi?targetID=3072785) | 1060 | 64 | hsa-miR-27a-3p | [NSA2](http://www.ncbi.nlm.nih.gov/entrez/query.fcgi?db=gene&cmd=Retrieve&dopt=full_report&list_uids=10412) | NSA2, ribosome biogenesis homolog |
| [Details](http://mirdb.org/cgi-bin/target_detail.cgi?targetID=3072795) | 1061 | 64 | hsa-miR-27a-3p | [GRIN2A](http://www.ncbi.nlm.nih.gov/entrez/query.fcgi?db=gene&cmd=Retrieve&dopt=full_report&list_uids=2903) | glutamate ionotropic receptor NMDA type subunit 2A |
| [Details](http://mirdb.org/cgi-bin/target_detail.cgi?targetID=3072852) | 1062 | 64 | hsa-miR-27a-3p | [CCNYL1](http://www.ncbi.nlm.nih.gov/entrez/query.fcgi?db=gene&cmd=Retrieve&dopt=full_report&list_uids=151195) | cyclin Y like 1 |
| [Details](http://mirdb.org/cgi-bin/target_detail.cgi?targetID=3072891) | 1063 | 64 | hsa-miR-27a-3p | [RRP12](http://www.ncbi.nlm.nih.gov/entrez/query.fcgi?db=gene&cmd=Retrieve&dopt=full_report&list_uids=23223) | ribosomal RNA processing 12 homolog |
| [Details](http://mirdb.org/cgi-bin/target_detail.cgi?targetID=3072911) | 1064 | 64 | hsa-miR-27a-3p | [CCR9](http://www.ncbi.nlm.nih.gov/entrez/query.fcgi?db=gene&cmd=Retrieve&dopt=full_report&list_uids=10803) | C-C motif chemokine receptor 9 |
| [Details](http://mirdb.org/cgi-bin/target_detail.cgi?targetID=3072986) | 1065 | 64 | hsa-miR-27a-3p | [PPM1E](http://www.ncbi.nlm.nih.gov/entrez/query.fcgi?db=gene&cmd=Retrieve&dopt=full_report&list_uids=22843) | protein phosphatase, Mg2+/Mn2+ dependent 1E |
| [Details](http://mirdb.org/cgi-bin/target_detail.cgi?targetID=3072989) | 1066 | 64 | hsa-miR-27a-3p | [TLNRD1](http://www.ncbi.nlm.nih.gov/entrez/query.fcgi?db=gene&cmd=Retrieve&dopt=full_report&list_uids=59274) | talin rod domain containing 1 |
| [Details](http://mirdb.org/cgi-bin/target_detail.cgi?targetID=3073003) | 1067 | 64 | hsa-miR-27a-3p | [SYT1](http://www.ncbi.nlm.nih.gov/entrez/query.fcgi?db=gene&cmd=Retrieve&dopt=full_report&list_uids=6857) | synaptotagmin 1 |
| [Details](http://mirdb.org/cgi-bin/target_detail.cgi?targetID=3073019) | 1068 | 64 | hsa-miR-27a-3p | [ERI3](http://www.ncbi.nlm.nih.gov/entrez/query.fcgi?db=gene&cmd=Retrieve&dopt=full_report&list_uids=79033) | ERI1 exoribonuclease family member 3 |
| [Details](http://mirdb.org/cgi-bin/target_detail.cgi?targetID=3073043) | 1069 | 64 | hsa-miR-27a-3p | [ZNF431](http://www.ncbi.nlm.nih.gov/entrez/query.fcgi?db=gene&cmd=Retrieve&dopt=full_report&list_uids=170959) | zinc finger protein 431 |
| [Details](http://mirdb.org/cgi-bin/target_detail.cgi?targetID=3073044) | 1070 | 64 | hsa-miR-27a-3p | [CALCRL](http://www.ncbi.nlm.nih.gov/entrez/query.fcgi?db=gene&cmd=Retrieve&dopt=full_report&list_uids=10203) | calcitonin receptor like receptor |
| [Details](http://mirdb.org/cgi-bin/target_detail.cgi?targetID=3073059) | 1071 | 64 | hsa-miR-27a-3p | [GNA13](http://www.ncbi.nlm.nih.gov/entrez/query.fcgi?db=gene&cmd=Retrieve&dopt=full_report&list_uids=10672) | G protein subunit alpha 13 |
| [Details](http://mirdb.org/cgi-bin/target_detail.cgi?targetID=3073080) | 1072 | 64 | hsa-miR-27a-3p | [ZFP36L1](http://www.ncbi.nlm.nih.gov/entrez/query.fcgi?db=gene&cmd=Retrieve&dopt=full_report&list_uids=677) | ZFP36 ring finger protein like 1 |
| [Details](http://mirdb.org/cgi-bin/target_detail.cgi?targetID=3073099) | 1073 | 64 | hsa-miR-27a-3p | [FAM222A](http://www.ncbi.nlm.nih.gov/entrez/query.fcgi?db=gene&cmd=Retrieve&dopt=full_report&list_uids=84915) | family with sequence similarity 222 member A |
| [Details](http://mirdb.org/cgi-bin/target_detail.cgi?targetID=3073241) | 1074 | 64 | hsa-miR-27a-3p | [RGS6](http://www.ncbi.nlm.nih.gov/entrez/query.fcgi?db=gene&cmd=Retrieve&dopt=full_report&list_uids=9628) | regulator of G protein signaling 6 |
| [Details](http://mirdb.org/cgi-bin/target_detail.cgi?targetID=3073267) | 1075 | 64 | hsa-miR-27a-3p | [ZNF510](http://www.ncbi.nlm.nih.gov/entrez/query.fcgi?db=gene&cmd=Retrieve&dopt=full_report&list_uids=22869) | zinc finger protein 510 |
| [Details](http://mirdb.org/cgi-bin/target_detail.cgi?targetID=3073286) | 1076 | 64 | hsa-miR-27a-3p | [PTGER4](http://www.ncbi.nlm.nih.gov/entrez/query.fcgi?db=gene&cmd=Retrieve&dopt=full_report&list_uids=5734) | prostaglandin E receptor 4 |
| [Details](http://mirdb.org/cgi-bin/target_detail.cgi?targetID=3073390) | 1077 | 64 | hsa-miR-27a-3p | [TMEM25](http://www.ncbi.nlm.nih.gov/entrez/query.fcgi?db=gene&cmd=Retrieve&dopt=full_report&list_uids=84866) | transmembrane protein 25 |
| [Details](http://mirdb.org/cgi-bin/target_detail.cgi?targetID=3073405) | 1078 | 64 | hsa-miR-27a-3p | [MAL2](http://www.ncbi.nlm.nih.gov/entrez/query.fcgi?db=gene&cmd=Retrieve&dopt=full_report&list_uids=114569) | mal, T cell differentiation protein 2 (gene/pseudogene) |
| [Details](http://mirdb.org/cgi-bin/target_detail.cgi?targetID=3073482) | 1079 | 64 | hsa-miR-27a-3p | [TMEM41B](http://www.ncbi.nlm.nih.gov/entrez/query.fcgi?db=gene&cmd=Retrieve&dopt=full_report&list_uids=440026) | transmembrane protein 41B |
| [Details](http://mirdb.org/cgi-bin/target_detail.cgi?targetID=3073589) | 1080 | 64 | hsa-miR-27a-3p | [RNF19B](http://www.ncbi.nlm.nih.gov/entrez/query.fcgi?db=gene&cmd=Retrieve&dopt=full_report&list_uids=127544) | ring finger protein 19B |
| [Details](http://mirdb.org/cgi-bin/target_detail.cgi?targetID=3073626) | 1081 | 64 | hsa-miR-27a-3p | [CACNA1A](http://www.ncbi.nlm.nih.gov/entrez/query.fcgi?db=gene&cmd=Retrieve&dopt=full_report&list_uids=773) | calcium voltage-gated channel subunit alpha1 A |
| [Details](http://mirdb.org/cgi-bin/target_detail.cgi?targetID=3073647) | 1082 | 64 | hsa-miR-27a-3p | [LRP6](http://www.ncbi.nlm.nih.gov/entrez/query.fcgi?db=gene&cmd=Retrieve&dopt=full_report&list_uids=4040) | LDL receptor related protein 6 |
| [Details](http://mirdb.org/cgi-bin/target_detail.cgi?targetID=3072459) | 1083 | 63 | hsa-miR-27a-3p | [MICU3](http://www.ncbi.nlm.nih.gov/entrez/query.fcgi?db=gene&cmd=Retrieve&dopt=full_report&list_uids=286097) | mitochondrial calcium uptake family member 3 |
| [Details](http://mirdb.org/cgi-bin/target_detail.cgi?targetID=3072565) | 1084 | 63 | hsa-miR-27a-3p | [PRR11](http://www.ncbi.nlm.nih.gov/entrez/query.fcgi?db=gene&cmd=Retrieve&dopt=full_report&list_uids=55771) | proline rich 11 |
| [Details](http://mirdb.org/cgi-bin/target_detail.cgi?targetID=3072582) | 1085 | 63 | hsa-miR-27a-3p | [EFNA2](http://www.ncbi.nlm.nih.gov/entrez/query.fcgi?db=gene&cmd=Retrieve&dopt=full_report&list_uids=1943) | ephrin A2 |
| [Details](http://mirdb.org/cgi-bin/target_detail.cgi?targetID=3072618) | 1086 | 63 | hsa-miR-27a-3p | [ACVR2A](http://www.ncbi.nlm.nih.gov/entrez/query.fcgi?db=gene&cmd=Retrieve&dopt=full_report&list_uids=92) | activin A receptor type 2A |
| [Details](http://mirdb.org/cgi-bin/target_detail.cgi?targetID=3072634) | 1087 | 63 | hsa-miR-27a-3p | [SLC16A12](http://www.ncbi.nlm.nih.gov/entrez/query.fcgi?db=gene&cmd=Retrieve&dopt=full_report&list_uids=387700) | solute carrier family 16 member 12 |
| [Details](http://mirdb.org/cgi-bin/target_detail.cgi?targetID=3072746) | 1088 | 63 | hsa-miR-27a-3p | [FNDC3A](http://www.ncbi.nlm.nih.gov/entrez/query.fcgi?db=gene&cmd=Retrieve&dopt=full_report&list_uids=22862) | fibronectin type III domain containing 3A |
| [Details](http://mirdb.org/cgi-bin/target_detail.cgi?targetID=3072825) | 1089 | 63 | hsa-miR-27a-3p | [PVR](http://www.ncbi.nlm.nih.gov/entrez/query.fcgi?db=gene&cmd=Retrieve&dopt=full_report&list_uids=5817) | poliovirus receptor |
| [Details](http://mirdb.org/cgi-bin/target_detail.cgi?targetID=3072872) | 1090 | 63 | hsa-miR-27a-3p | [OLFM3](http://www.ncbi.nlm.nih.gov/entrez/query.fcgi?db=gene&cmd=Retrieve&dopt=full_report&list_uids=118427) | olfactomedin 3 |
| [Details](http://mirdb.org/cgi-bin/target_detail.cgi?targetID=3072875) | 1091 | 63 | hsa-miR-27a-3p | [ANK3](http://www.ncbi.nlm.nih.gov/entrez/query.fcgi?db=gene&cmd=Retrieve&dopt=full_report&list_uids=288) | ankyrin 3 |
| [Details](http://mirdb.org/cgi-bin/target_detail.cgi?targetID=3072896) | 1092 | 63 | hsa-miR-27a-3p | [LSM12](http://www.ncbi.nlm.nih.gov/entrez/query.fcgi?db=gene&cmd=Retrieve&dopt=full_report&list_uids=124801) | LSM12 homolog |
| [Details](http://mirdb.org/cgi-bin/target_detail.cgi?targetID=3073091) | 1093 | 63 | hsa-miR-27a-3p | [UBE2G1](http://www.ncbi.nlm.nih.gov/entrez/query.fcgi?db=gene&cmd=Retrieve&dopt=full_report&list_uids=7326) | ubiquitin conjugating enzyme E2 G1 |
| [Details](http://mirdb.org/cgi-bin/target_detail.cgi?targetID=3073098) | 1094 | 63 | hsa-miR-27a-3p | [NDST3](http://www.ncbi.nlm.nih.gov/entrez/query.fcgi?db=gene&cmd=Retrieve&dopt=full_report&list_uids=9348) | N-deacetylase and N-sulfotransferase 3 |
| [Details](http://mirdb.org/cgi-bin/target_detail.cgi?targetID=3073131) | 1095 | 63 | hsa-miR-27a-3p | [TBC1D8B](http://www.ncbi.nlm.nih.gov/entrez/query.fcgi?db=gene&cmd=Retrieve&dopt=full_report&list_uids=54885) | TBC1 domain family member 8B |
| [Details](http://mirdb.org/cgi-bin/target_detail.cgi?targetID=3073135) | 1096 | 63 | hsa-miR-27a-3p | [MMGT1](http://www.ncbi.nlm.nih.gov/entrez/query.fcgi?db=gene&cmd=Retrieve&dopt=full_report&list_uids=93380) | membrane magnesium transporter 1 |
| [Details](http://mirdb.org/cgi-bin/target_detail.cgi?targetID=3073227) | 1097 | 63 | hsa-miR-27a-3p | [RALYL](http://www.ncbi.nlm.nih.gov/entrez/query.fcgi?db=gene&cmd=Retrieve&dopt=full_report&list_uids=138046) | RALY RNA binding protein like |
| [Details](http://mirdb.org/cgi-bin/target_detail.cgi?targetID=3073345) | 1098 | 63 | hsa-miR-27a-3p | [PGR](http://www.ncbi.nlm.nih.gov/entrez/query.fcgi?db=gene&cmd=Retrieve&dopt=full_report&list_uids=5241) | progesterone receptor |
| [Details](http://mirdb.org/cgi-bin/target_detail.cgi?targetID=3073359) | 1099 | 63 | hsa-miR-27a-3p | [GREM1](http://www.ncbi.nlm.nih.gov/entrez/query.fcgi?db=gene&cmd=Retrieve&dopt=full_report&list_uids=26585) | gremlin 1, DAN family BMP antagonist |
| [Details](http://mirdb.org/cgi-bin/target_detail.cgi?targetID=3073376) | 1100 | 63 | hsa-miR-27a-3p | [AP3D1](http://www.ncbi.nlm.nih.gov/entrez/query.fcgi?db=gene&cmd=Retrieve&dopt=full_report&list_uids=8943) | adaptor related protein complex 3 subunit delta 1 |
| [Details](http://mirdb.org/cgi-bin/target_detail.cgi?targetID=3073424) | 1101 | 63 | hsa-miR-27a-3p | [GABRP](http://www.ncbi.nlm.nih.gov/entrez/query.fcgi?db=gene&cmd=Retrieve&dopt=full_report&list_uids=2568) | gamma-aminobutyric acid type A receptor pi subunit |
| [Details](http://mirdb.org/cgi-bin/target_detail.cgi?targetID=3073466) | 1102 | 63 | hsa-miR-27a-3p | [FBXO36](http://www.ncbi.nlm.nih.gov/entrez/query.fcgi?db=gene&cmd=Retrieve&dopt=full_report&list_uids=130888) | F-box protein 36 |
| [Details](http://mirdb.org/cgi-bin/target_detail.cgi?targetID=3073615) | 1103 | 63 | hsa-miR-27a-3p | [PKNOX2](http://www.ncbi.nlm.nih.gov/entrez/query.fcgi?db=gene&cmd=Retrieve&dopt=full_report&list_uids=63876) | PBX/knotted 1 homeobox 2 |
| [Details](http://mirdb.org/cgi-bin/target_detail.cgi?targetID=3073629) | 1104 | 63 | hsa-miR-27a-3p | [FBLN2](http://www.ncbi.nlm.nih.gov/entrez/query.fcgi?db=gene&cmd=Retrieve&dopt=full_report&list_uids=2199) | fibulin 2 |
| [Details](http://mirdb.org/cgi-bin/target_detail.cgi?targetID=3073795) | 1105 | 63 | hsa-miR-27a-3p | [ID2](http://www.ncbi.nlm.nih.gov/entrez/query.fcgi?db=gene&cmd=Retrieve&dopt=full_report&list_uids=3398) | inhibitor of DNA binding 2 |
| [Details](http://mirdb.org/cgi-bin/target_detail.cgi?targetID=3073844) | 1106 | 63 | hsa-miR-27a-3p | [ENAH](http://www.ncbi.nlm.nih.gov/entrez/query.fcgi?db=gene&cmd=Retrieve&dopt=full_report&list_uids=55740) | ENAH, actin regulator |
| [Details](http://mirdb.org/cgi-bin/target_detail.cgi?targetID=3072433) | 1107 | 62 | hsa-miR-27a-3p | [NRAS](http://www.ncbi.nlm.nih.gov/entrez/query.fcgi?db=gene&cmd=Retrieve&dopt=full_report&list_uids=4893) | NRAS proto-oncogene, GTPase |
| [Details](http://mirdb.org/cgi-bin/target_detail.cgi?targetID=3072450) | 1108 | 62 | hsa-miR-27a-3p | [STARD7](http://www.ncbi.nlm.nih.gov/entrez/query.fcgi?db=gene&cmd=Retrieve&dopt=full_report&list_uids=56910) | StAR related lipid transfer domain containing 7 |
| [Details](http://mirdb.org/cgi-bin/target_detail.cgi?targetID=3072467) | 1109 | 62 | hsa-miR-27a-3p | [HOXB8](http://www.ncbi.nlm.nih.gov/entrez/query.fcgi?db=gene&cmd=Retrieve&dopt=full_report&list_uids=3218) | homeobox B8 |
| [Details](http://mirdb.org/cgi-bin/target_detail.cgi?targetID=3072515) | 1110 | 62 | hsa-miR-27a-3p | [PTPRJ](http://www.ncbi.nlm.nih.gov/entrez/query.fcgi?db=gene&cmd=Retrieve&dopt=full_report&list_uids=5795) | protein tyrosine phosphatase, receptor type J |
| [Details](http://mirdb.org/cgi-bin/target_detail.cgi?targetID=3072529) | 1111 | 62 | hsa-miR-27a-3p | [CUBN](http://www.ncbi.nlm.nih.gov/entrez/query.fcgi?db=gene&cmd=Retrieve&dopt=full_report&list_uids=8029) | cubilin |
| [Details](http://mirdb.org/cgi-bin/target_detail.cgi?targetID=3072537) | 1112 | 62 | hsa-miR-27a-3p | [RASEF](http://www.ncbi.nlm.nih.gov/entrez/query.fcgi?db=gene&cmd=Retrieve&dopt=full_report&list_uids=158158) | RAS and EF-hand domain containing |
| [Details](http://mirdb.org/cgi-bin/target_detail.cgi?targetID=3072561) | 1113 | 62 | hsa-miR-27a-3p | [ZNF619](http://www.ncbi.nlm.nih.gov/entrez/query.fcgi?db=gene&cmd=Retrieve&dopt=full_report&list_uids=285267) | zinc finger protein 619 |
| [Details](http://mirdb.org/cgi-bin/target_detail.cgi?targetID=3072566) | 1114 | 62 | hsa-miR-27a-3p | [COL5A1](http://www.ncbi.nlm.nih.gov/entrez/query.fcgi?db=gene&cmd=Retrieve&dopt=full_report&list_uids=1289) | collagen type V alpha 1 chain |
| [Details](http://mirdb.org/cgi-bin/target_detail.cgi?targetID=3072593) | 1115 | 62 | hsa-miR-27a-3p | [ZBTB41](http://www.ncbi.nlm.nih.gov/entrez/query.fcgi?db=gene&cmd=Retrieve&dopt=full_report&list_uids=360023) | zinc finger and BTB domain containing 41 |
| [Details](http://mirdb.org/cgi-bin/target_detail.cgi?targetID=3072666) | 1116 | 62 | hsa-miR-27a-3p | [MYSM1](http://www.ncbi.nlm.nih.gov/entrez/query.fcgi?db=gene&cmd=Retrieve&dopt=full_report&list_uids=114803) | Myb like, SWIRM and MPN domains 1 |
| [Details](http://mirdb.org/cgi-bin/target_detail.cgi?targetID=3072682) | 1117 | 62 | hsa-miR-27a-3p | [RNF6](http://www.ncbi.nlm.nih.gov/entrez/query.fcgi?db=gene&cmd=Retrieve&dopt=full_report&list_uids=6049) | ring finger protein 6 |
| [Details](http://mirdb.org/cgi-bin/target_detail.cgi?targetID=3072717) | 1118 | 62 | hsa-miR-27a-3p | [SLC27A4](http://www.ncbi.nlm.nih.gov/entrez/query.fcgi?db=gene&cmd=Retrieve&dopt=full_report&list_uids=10999) | solute carrier family 27 member 4 |
| [Details](http://mirdb.org/cgi-bin/target_detail.cgi?targetID=3072726) | 1119 | 62 | hsa-miR-27a-3p | [CASC10](http://www.ncbi.nlm.nih.gov/entrez/query.fcgi?db=gene&cmd=Retrieve&dopt=full_report&list_uids=399726) | cancer susceptibility 10 |
| [Details](http://mirdb.org/cgi-bin/target_detail.cgi?targetID=3072750) | 1120 | 62 | hsa-miR-27a-3p | [SIN3A](http://www.ncbi.nlm.nih.gov/entrez/query.fcgi?db=gene&cmd=Retrieve&dopt=full_report&list_uids=25942) | SIN3 transcription regulator family member A |
| [Details](http://mirdb.org/cgi-bin/target_detail.cgi?targetID=3072807) | 1121 | 62 | hsa-miR-27a-3p | [ADRA1A](http://www.ncbi.nlm.nih.gov/entrez/query.fcgi?db=gene&cmd=Retrieve&dopt=full_report&list_uids=148) | adrenoceptor alpha 1A |
| [Details](http://mirdb.org/cgi-bin/target_detail.cgi?targetID=3072818) | 1122 | 62 | hsa-miR-27a-3p | [ZNF140](http://www.ncbi.nlm.nih.gov/entrez/query.fcgi?db=gene&cmd=Retrieve&dopt=full_report&list_uids=7699) | zinc finger protein 140 |
| [Details](http://mirdb.org/cgi-bin/target_detail.cgi?targetID=3072914) | 1123 | 62 | hsa-miR-27a-3p | [PAPPA](http://www.ncbi.nlm.nih.gov/entrez/query.fcgi?db=gene&cmd=Retrieve&dopt=full_report&list_uids=5069) | pappalysin 1 |
| [Details](http://mirdb.org/cgi-bin/target_detail.cgi?targetID=3072933) | 1124 | 62 | hsa-miR-27a-3p | [AGO2](http://www.ncbi.nlm.nih.gov/entrez/query.fcgi?db=gene&cmd=Retrieve&dopt=full_report&list_uids=27161) | argonaute RISC catalytic component 2 |
| [Details](http://mirdb.org/cgi-bin/target_detail.cgi?targetID=3072947) | 1125 | 62 | hsa-miR-27a-3p | [SLC26A2](http://www.ncbi.nlm.nih.gov/entrez/query.fcgi?db=gene&cmd=Retrieve&dopt=full_report&list_uids=1836) | solute carrier family 26 member 2 |
| [Details](http://mirdb.org/cgi-bin/target_detail.cgi?targetID=3072953) | 1126 | 62 | hsa-miR-27a-3p | [NSG1](http://www.ncbi.nlm.nih.gov/entrez/query.fcgi?db=gene&cmd=Retrieve&dopt=full_report&list_uids=27065) | neuronal vesicle trafficking associated 1 |
| [Details](http://mirdb.org/cgi-bin/target_detail.cgi?targetID=3072982) | 1127 | 62 | hsa-miR-27a-3p | [ZNF609](http://www.ncbi.nlm.nih.gov/entrez/query.fcgi?db=gene&cmd=Retrieve&dopt=full_report&list_uids=23060) | zinc finger protein 609 |
| [Details](http://mirdb.org/cgi-bin/target_detail.cgi?targetID=3073086) | 1128 | 62 | hsa-miR-27a-3p | [ARHGAP12](http://www.ncbi.nlm.nih.gov/entrez/query.fcgi?db=gene&cmd=Retrieve&dopt=full_report&list_uids=94134) | Rho GTPase activating protein 12 |
| [Details](http://mirdb.org/cgi-bin/target_detail.cgi?targetID=3073165) | 1129 | 62 | hsa-miR-27a-3p | [AARD](http://www.ncbi.nlm.nih.gov/entrez/query.fcgi?db=gene&cmd=Retrieve&dopt=full_report&list_uids=441376) | alanine and arginine rich domain containing protein |
| [Details](http://mirdb.org/cgi-bin/target_detail.cgi?targetID=3073169) | 1130 | 62 | hsa-miR-27a-3p | [CAPRIN2](http://www.ncbi.nlm.nih.gov/entrez/query.fcgi?db=gene&cmd=Retrieve&dopt=full_report&list_uids=65981) | caprin family member 2 |
| [Details](http://mirdb.org/cgi-bin/target_detail.cgi?targetID=3073177) | 1131 | 62 | hsa-miR-27a-3p | [YPEL2](http://www.ncbi.nlm.nih.gov/entrez/query.fcgi?db=gene&cmd=Retrieve&dopt=full_report&list_uids=388403) | yippee like 2 |
| [Details](http://mirdb.org/cgi-bin/target_detail.cgi?targetID=3073192) | 1132 | 62 | hsa-miR-27a-3p | [DOK6](http://www.ncbi.nlm.nih.gov/entrez/query.fcgi?db=gene&cmd=Retrieve&dopt=full_report&list_uids=220164) | docking protein 6 |
| [Details](http://mirdb.org/cgi-bin/target_detail.cgi?targetID=3073269) | 1133 | 62 | hsa-miR-27a-3p | [ERCC8](http://www.ncbi.nlm.nih.gov/entrez/query.fcgi?db=gene&cmd=Retrieve&dopt=full_report&list_uids=1161) | ERCC excision repair 8, CSA ubiquitin ligase complex subunit |
| [Details](http://mirdb.org/cgi-bin/target_detail.cgi?targetID=3073274) | 1134 | 62 | hsa-miR-27a-3p | [RNF180](http://www.ncbi.nlm.nih.gov/entrez/query.fcgi?db=gene&cmd=Retrieve&dopt=full_report&list_uids=285671) | ring finger protein 180 |
| [Details](http://mirdb.org/cgi-bin/target_detail.cgi?targetID=3073421) | 1135 | 62 | hsa-miR-27a-3p | [COL27A1](http://www.ncbi.nlm.nih.gov/entrez/query.fcgi?db=gene&cmd=Retrieve&dopt=full_report&list_uids=85301) | collagen type XXVII alpha 1 chain |
| [Details](http://mirdb.org/cgi-bin/target_detail.cgi?targetID=3073454) | 1136 | 62 | hsa-miR-27a-3p | [INIP](http://www.ncbi.nlm.nih.gov/entrez/query.fcgi?db=gene&cmd=Retrieve&dopt=full_report&list_uids=58493) | INTS3 and NABP interacting protein |
| [Details](http://mirdb.org/cgi-bin/target_detail.cgi?targetID=3073485) | 1137 | 62 | hsa-miR-27a-3p | [AOX1](http://www.ncbi.nlm.nih.gov/entrez/query.fcgi?db=gene&cmd=Retrieve&dopt=full_report&list_uids=316) | aldehyde oxidase 1 |
| [Details](http://mirdb.org/cgi-bin/target_detail.cgi?targetID=3073497) | 1138 | 62 | hsa-miR-27a-3p | [KLF2](http://www.ncbi.nlm.nih.gov/entrez/query.fcgi?db=gene&cmd=Retrieve&dopt=full_report&list_uids=10365) | Kruppel like factor 2 |
| [Details](http://mirdb.org/cgi-bin/target_detail.cgi?targetID=3073511) | 1139 | 62 | hsa-miR-27a-3p | [AMOTL1](http://www.ncbi.nlm.nih.gov/entrez/query.fcgi?db=gene&cmd=Retrieve&dopt=full_report&list_uids=154810) | angiomotin like 1 |
| [Details](http://mirdb.org/cgi-bin/target_detail.cgi?targetID=3073665) | 1140 | 62 | hsa-miR-27a-3p | [HERC3](http://www.ncbi.nlm.nih.gov/entrez/query.fcgi?db=gene&cmd=Retrieve&dopt=full_report&list_uids=8916) | HECT and RLD domain containing E3 ubiquitin protein ligase 3 |
| [Details](http://mirdb.org/cgi-bin/target_detail.cgi?targetID=3073707) | 1141 | 62 | hsa-miR-27a-3p | [BMPR1A](http://www.ncbi.nlm.nih.gov/entrez/query.fcgi?db=gene&cmd=Retrieve&dopt=full_report&list_uids=657) | bone morphogenetic protein receptor type 1A |
| [Details](http://mirdb.org/cgi-bin/target_detail.cgi?targetID=3073715) | 1142 | 62 | hsa-miR-27a-3p | [ARL2BP](http://www.ncbi.nlm.nih.gov/entrez/query.fcgi?db=gene&cmd=Retrieve&dopt=full_report&list_uids=23568) | ADP ribosylation factor like GTPase 2 binding protein |
| [Details](http://mirdb.org/cgi-bin/target_detail.cgi?targetID=3073723) | 1143 | 62 | hsa-miR-27a-3p | [BRWD1](http://www.ncbi.nlm.nih.gov/entrez/query.fcgi?db=gene&cmd=Retrieve&dopt=full_report&list_uids=54014) | bromodomain and WD repeat domain containing 1 |
| [Details](http://mirdb.org/cgi-bin/target_detail.cgi?targetID=3073740) | 1144 | 62 | hsa-miR-27a-3p | [PPP6C](http://www.ncbi.nlm.nih.gov/entrez/query.fcgi?db=gene&cmd=Retrieve&dopt=full_report&list_uids=5537) | protein phosphatase 6 catalytic subunit |
| [Details](http://mirdb.org/cgi-bin/target_detail.cgi?targetID=3073741) | 1145 | 62 | hsa-miR-27a-3p | [SHPK](http://www.ncbi.nlm.nih.gov/entrez/query.fcgi?db=gene&cmd=Retrieve&dopt=full_report&list_uids=23729) | sedoheptulokinase |
| [Details](http://mirdb.org/cgi-bin/target_detail.cgi?targetID=3073794) | 1146 | 62 | hsa-miR-27a-3p | [MED28](http://www.ncbi.nlm.nih.gov/entrez/query.fcgi?db=gene&cmd=Retrieve&dopt=full_report&list_uids=80306) | mediator complex subunit 28 |
| [Details](http://mirdb.org/cgi-bin/target_detail.cgi?targetID=3072435) | 1147 | 61 | hsa-miR-27a-3p | [CNTNAP4](http://www.ncbi.nlm.nih.gov/entrez/query.fcgi?db=gene&cmd=Retrieve&dopt=full_report&list_uids=85445) | contactin associated protein like 4 |
| [Details](http://mirdb.org/cgi-bin/target_detail.cgi?targetID=3072476) | 1148 | 61 | hsa-miR-27a-3p | [GLRX](http://www.ncbi.nlm.nih.gov/entrez/query.fcgi?db=gene&cmd=Retrieve&dopt=full_report&list_uids=2745) | glutaredoxin |
| [Details](http://mirdb.org/cgi-bin/target_detail.cgi?targetID=3072482) | 1149 | 61 | hsa-miR-27a-3p | [PGBD5](http://www.ncbi.nlm.nih.gov/entrez/query.fcgi?db=gene&cmd=Retrieve&dopt=full_report&list_uids=79605) | piggyBac transposable element derived 5 |
| [Details](http://mirdb.org/cgi-bin/target_detail.cgi?targetID=3072668) | 1150 | 61 | hsa-miR-27a-3p | [ACTR8](http://www.ncbi.nlm.nih.gov/entrez/query.fcgi?db=gene&cmd=Retrieve&dopt=full_report&list_uids=93973) | ARP8 actin related protein 8 homolog |
| [Details](http://mirdb.org/cgi-bin/target_detail.cgi?targetID=3072767) | 1151 | 61 | hsa-miR-27a-3p | [RIT1](http://www.ncbi.nlm.nih.gov/entrez/query.fcgi?db=gene&cmd=Retrieve&dopt=full_report&list_uids=6016) | Ras like without CAAX 1 |
| [Details](http://mirdb.org/cgi-bin/target_detail.cgi?targetID=3072787) | 1152 | 61 | hsa-miR-27a-3p | [ASIC1](http://www.ncbi.nlm.nih.gov/entrez/query.fcgi?db=gene&cmd=Retrieve&dopt=full_report&list_uids=41) | acid sensing ion channel subunit 1 |
| [Details](http://mirdb.org/cgi-bin/target_detail.cgi?targetID=3072988) | 1153 | 61 | hsa-miR-27a-3p | [RAB3C](http://www.ncbi.nlm.nih.gov/entrez/query.fcgi?db=gene&cmd=Retrieve&dopt=full_report&list_uids=115827) | RAB3C, member RAS oncogene family |
| [Details](http://mirdb.org/cgi-bin/target_detail.cgi?targetID=3072993) | 1154 | 61 | hsa-miR-27a-3p | [GORASP1](http://www.ncbi.nlm.nih.gov/entrez/query.fcgi?db=gene&cmd=Retrieve&dopt=full_report&list_uids=64689) | golgi reassembly stacking protein 1 |
| [Details](http://mirdb.org/cgi-bin/target_detail.cgi?targetID=3073005) | 1155 | 61 | hsa-miR-27a-3p | [POP1](http://www.ncbi.nlm.nih.gov/entrez/query.fcgi?db=gene&cmd=Retrieve&dopt=full_report&list_uids=10940) | POP1 homolog, ribonuclease P/MRP subunit |
| [Details](http://mirdb.org/cgi-bin/target_detail.cgi?targetID=3073088) | 1156 | 61 | hsa-miR-27a-3p | [KCNJ1](http://www.ncbi.nlm.nih.gov/entrez/query.fcgi?db=gene&cmd=Retrieve&dopt=full_report&list_uids=3758) | potassium voltage-gated channel subfamily J member 1 |
| [Details](http://mirdb.org/cgi-bin/target_detail.cgi?targetID=3073355) | 1157 | 61 | hsa-miR-27a-3p | [EI24](http://www.ncbi.nlm.nih.gov/entrez/query.fcgi?db=gene&cmd=Retrieve&dopt=full_report&list_uids=9538) | EI24, autophagy associated transmembrane protein |
| [Details](http://mirdb.org/cgi-bin/target_detail.cgi?targetID=3073416) | 1158 | 61 | hsa-miR-27a-3p | [NKAPD1](http://www.ncbi.nlm.nih.gov/entrez/query.fcgi?db=gene&cmd=Retrieve&dopt=full_report&list_uids=55216) | NKAP domain containing 1 |
| [Details](http://mirdb.org/cgi-bin/target_detail.cgi?targetID=3073439) | 1159 | 61 | hsa-miR-27a-3p | [GCNT2](http://www.ncbi.nlm.nih.gov/entrez/query.fcgi?db=gene&cmd=Retrieve&dopt=full_report&list_uids=2651) | glucosaminyl (N-acetyl) transferase 2 (I blood group) |
| [Details](http://mirdb.org/cgi-bin/target_detail.cgi?targetID=3073467) | 1160 | 61 | hsa-miR-27a-3p | [NCOA1](http://www.ncbi.nlm.nih.gov/entrez/query.fcgi?db=gene&cmd=Retrieve&dopt=full_report&list_uids=8648) | nuclear receptor coactivator 1 |
| [Details](http://mirdb.org/cgi-bin/target_detail.cgi?targetID=3073473) | 1161 | 61 | hsa-miR-27a-3p | [CD44](http://www.ncbi.nlm.nih.gov/entrez/query.fcgi?db=gene&cmd=Retrieve&dopt=full_report&list_uids=960) | CD44 molecule (Indian blood group) |
| [Details](http://mirdb.org/cgi-bin/target_detail.cgi?targetID=3073499) | 1162 | 61 | hsa-miR-27a-3p | [CEP57L1](http://www.ncbi.nlm.nih.gov/entrez/query.fcgi?db=gene&cmd=Retrieve&dopt=full_report&list_uids=285753) | centrosomal protein 57 like 1 |
| [Details](http://mirdb.org/cgi-bin/target_detail.cgi?targetID=3073541) | 1163 | 61 | hsa-miR-27a-3p | [FGL2](http://www.ncbi.nlm.nih.gov/entrez/query.fcgi?db=gene&cmd=Retrieve&dopt=full_report&list_uids=10875) | fibrinogen like 2 |
| [Details](http://mirdb.org/cgi-bin/target_detail.cgi?targetID=3073573) | 1164 | 61 | hsa-miR-27a-3p | [PRKCH](http://www.ncbi.nlm.nih.gov/entrez/query.fcgi?db=gene&cmd=Retrieve&dopt=full_report&list_uids=5583) | protein kinase C eta |
| [Details](http://mirdb.org/cgi-bin/target_detail.cgi?targetID=3073609) | 1165 | 61 | hsa-miR-27a-3p | [KIAA1549](http://www.ncbi.nlm.nih.gov/entrez/query.fcgi?db=gene&cmd=Retrieve&dopt=full_report&list_uids=57670) | KIAA1549 |
| [Details](http://mirdb.org/cgi-bin/target_detail.cgi?targetID=3073619) | 1166 | 61 | hsa-miR-27a-3p | [ZFX](http://www.ncbi.nlm.nih.gov/entrez/query.fcgi?db=gene&cmd=Retrieve&dopt=full_report&list_uids=7543) | zinc finger protein X-linked |
| [Details](http://mirdb.org/cgi-bin/target_detail.cgi?targetID=3073908) | 1167 | 61 | hsa-miR-27a-3p | [DAP3](http://www.ncbi.nlm.nih.gov/entrez/query.fcgi?db=gene&cmd=Retrieve&dopt=full_report&list_uids=7818) | death associated protein 3 |
| [Details](http://mirdb.org/cgi-bin/target_detail.cgi?targetID=3072445) | 1168 | 60 | hsa-miR-27a-3p | [TCAIM](http://www.ncbi.nlm.nih.gov/entrez/query.fcgi?db=gene&cmd=Retrieve&dopt=full_report&list_uids=285343) | T cell activation inhibitor, mitochondrial |
| [Details](http://mirdb.org/cgi-bin/target_detail.cgi?targetID=3072451) | 1169 | 60 | hsa-miR-27a-3p | [N4BP1](http://www.ncbi.nlm.nih.gov/entrez/query.fcgi?db=gene&cmd=Retrieve&dopt=full_report&list_uids=9683) | NEDD4 binding protein 1 |
| [Details](http://mirdb.org/cgi-bin/target_detail.cgi?targetID=3072454) | 1170 | 60 | hsa-miR-27a-3p | [ABCD3](http://www.ncbi.nlm.nih.gov/entrez/query.fcgi?db=gene&cmd=Retrieve&dopt=full_report&list_uids=5825) | ATP binding cassette subfamily D member 3 |
| [Details](http://mirdb.org/cgi-bin/target_detail.cgi?targetID=3072488) | 1171 | 60 | hsa-miR-27a-3p | [NXPH1](http://www.ncbi.nlm.nih.gov/entrez/query.fcgi?db=gene&cmd=Retrieve&dopt=full_report&list_uids=30010) | neurexophilin 1 |
| [Details](http://mirdb.org/cgi-bin/target_detail.cgi?targetID=3072520) | 1172 | 60 | hsa-miR-27a-3p | [CPNE8](http://www.ncbi.nlm.nih.gov/entrez/query.fcgi?db=gene&cmd=Retrieve&dopt=full_report&list_uids=144402) | copine 8 |
| [Details](http://mirdb.org/cgi-bin/target_detail.cgi?targetID=3072744) | 1173 | 60 | hsa-miR-27a-3p | [ADAM12](http://www.ncbi.nlm.nih.gov/entrez/query.fcgi?db=gene&cmd=Retrieve&dopt=full_report&list_uids=8038) | ADAM metallopeptidase domain 12 |
| [Details](http://mirdb.org/cgi-bin/target_detail.cgi?targetID=3072760) | 1174 | 60 | hsa-miR-27a-3p | [JMY](http://www.ncbi.nlm.nih.gov/entrez/query.fcgi?db=gene&cmd=Retrieve&dopt=full_report&list_uids=133746) | junction mediating and regulatory protein, p53 cofactor |
| [Details](http://mirdb.org/cgi-bin/target_detail.cgi?targetID=3072840) | 1175 | 60 | hsa-miR-27a-3p | [MR1](http://www.ncbi.nlm.nih.gov/entrez/query.fcgi?db=gene&cmd=Retrieve&dopt=full_report&list_uids=3140) | major histocompatibility complex, class I-related |
| [Details](http://mirdb.org/cgi-bin/target_detail.cgi?targetID=3072848) | 1176 | 60 | hsa-miR-27a-3p | [HSBP1L1](http://www.ncbi.nlm.nih.gov/entrez/query.fcgi?db=gene&cmd=Retrieve&dopt=full_report&list_uids=440498) | heat shock factor binding protein 1 like 1 |
| [Details](http://mirdb.org/cgi-bin/target_detail.cgi?targetID=3072874) | 1177 | 60 | hsa-miR-27a-3p | [SCAMP1](http://www.ncbi.nlm.nih.gov/entrez/query.fcgi?db=gene&cmd=Retrieve&dopt=full_report&list_uids=9522) | secretory carrier membrane protein 1 |
| [Details](http://mirdb.org/cgi-bin/target_detail.cgi?targetID=3072915) | 1178 | 60 | hsa-miR-27a-3p | [SDC2](http://www.ncbi.nlm.nih.gov/entrez/query.fcgi?db=gene&cmd=Retrieve&dopt=full_report&list_uids=6383) | syndecan 2 |
| [Details](http://mirdb.org/cgi-bin/target_detail.cgi?targetID=3072992) | 1179 | 60 | hsa-miR-27a-3p | [GNL3L](http://www.ncbi.nlm.nih.gov/entrez/query.fcgi?db=gene&cmd=Retrieve&dopt=full_report&list_uids=54552) | G protein nucleolar 3 like |
| [Details](http://mirdb.org/cgi-bin/target_detail.cgi?targetID=3073001) | 1180 | 60 | hsa-miR-27a-3p | [ZC3H12D](http://www.ncbi.nlm.nih.gov/entrez/query.fcgi?db=gene&cmd=Retrieve&dopt=full_report&list_uids=340152) | zinc finger CCCH-type containing 12D |
| [Details](http://mirdb.org/cgi-bin/target_detail.cgi?targetID=3073078) | 1181 | 60 | hsa-miR-27a-3p | [DPY19L3](http://www.ncbi.nlm.nih.gov/entrez/query.fcgi?db=gene&cmd=Retrieve&dopt=full_report&list_uids=147991) | dpy-19 like C-mannosyltransferase 3 |
| [Details](http://mirdb.org/cgi-bin/target_detail.cgi?targetID=3073149) | 1182 | 60 | hsa-miR-27a-3p | [NOLC1](http://www.ncbi.nlm.nih.gov/entrez/query.fcgi?db=gene&cmd=Retrieve&dopt=full_report&list_uids=9221) | nucleolar and coiled-body phosphoprotein 1 |
| [Details](http://mirdb.org/cgi-bin/target_detail.cgi?targetID=3073186) | 1183 | 60 | hsa-miR-27a-3p | [SLC6A6](http://www.ncbi.nlm.nih.gov/entrez/query.fcgi?db=gene&cmd=Retrieve&dopt=full_report&list_uids=6533) | solute carrier family 6 member 6 |
| [Details](http://mirdb.org/cgi-bin/target_detail.cgi?targetID=3073217) | 1184 | 60 | hsa-miR-27a-3p | [TGOLN2](http://www.ncbi.nlm.nih.gov/entrez/query.fcgi?db=gene&cmd=Retrieve&dopt=full_report&list_uids=10618) | trans-golgi network protein 2 |
| [Details](http://mirdb.org/cgi-bin/target_detail.cgi?targetID=3073230) | 1185 | 60 | hsa-miR-27a-3p | [SMAD1](http://www.ncbi.nlm.nih.gov/entrez/query.fcgi?db=gene&cmd=Retrieve&dopt=full_report&list_uids=4086) | SMAD family member 1 |
| [Details](http://mirdb.org/cgi-bin/target_detail.cgi?targetID=3073272) | 1186 | 60 | hsa-miR-27a-3p | [ERC2](http://www.ncbi.nlm.nih.gov/entrez/query.fcgi?db=gene&cmd=Retrieve&dopt=full_report&list_uids=26059) | ELKS/RAB6-interacting/CAST family member 2 |
| [Details](http://mirdb.org/cgi-bin/target_detail.cgi?targetID=3073333) | 1187 | 60 | hsa-miR-27a-3p | [GIGYF2](http://www.ncbi.nlm.nih.gov/entrez/query.fcgi?db=gene&cmd=Retrieve&dopt=full_report&list_uids=26058) | GRB10 interacting GYF protein 2 |
| [Details](http://mirdb.org/cgi-bin/target_detail.cgi?targetID=3073352) | 1188 | 60 | hsa-miR-27a-3p | [CALM3](http://www.ncbi.nlm.nih.gov/entrez/query.fcgi?db=gene&cmd=Retrieve&dopt=full_report&list_uids=808) | calmodulin 3 |
| [Details](http://mirdb.org/cgi-bin/target_detail.cgi?targetID=3073368) | 1189 | 60 | hsa-miR-27a-3p | [MAP7D3](http://www.ncbi.nlm.nih.gov/entrez/query.fcgi?db=gene&cmd=Retrieve&dopt=full_report&list_uids=79649) | MAP7 domain containing 3 |
| [Details](http://mirdb.org/cgi-bin/target_detail.cgi?targetID=3073674) | 1190 | 60 | hsa-miR-27a-3p | [ARHGEF9](http://www.ncbi.nlm.nih.gov/entrez/query.fcgi?db=gene&cmd=Retrieve&dopt=full_report&list_uids=23229) | Cdc42 guanine nucleotide exchange factor 9 |
| [Details](http://mirdb.org/cgi-bin/target_detail.cgi?targetID=3073692) | 1191 | 60 | hsa-miR-27a-3p | [ZNF350](http://www.ncbi.nlm.nih.gov/entrez/query.fcgi?db=gene&cmd=Retrieve&dopt=full_report&list_uids=59348) | zinc finger protein 350 |
| [Details](http://mirdb.org/cgi-bin/target_detail.cgi?targetID=3073712) | 1192 | 60 | hsa-miR-27a-3p | [SUSD5](http://www.ncbi.nlm.nih.gov/entrez/query.fcgi?db=gene&cmd=Retrieve&dopt=full_report&list_uids=26032) | sushi domain containing 5 |
| [Details](http://mirdb.org/cgi-bin/target_detail.cgi?targetID=3073728) | 1193 | 60 | hsa-miR-27a-3p | [CAVIN2](http://www.ncbi.nlm.nih.gov/entrez/query.fcgi?db=gene&cmd=Retrieve&dopt=full_report&list_uids=8436) | caveolae associated protein 2 |
| [Details](http://mirdb.org/cgi-bin/target_detail.cgi?targetID=3073847) | 1194 | 60 | hsa-miR-27a-3p | [GLP1R](http://www.ncbi.nlm.nih.gov/entrez/query.fcgi?db=gene&cmd=Retrieve&dopt=full_report&list_uids=2740) | glucagon like peptide 1 receptor |
| [Details](http://mirdb.org/cgi-bin/target_detail.cgi?targetID=3073913) | 1195 | 60 | hsa-miR-27a-3p | [CIAO1](http://www.ncbi.nlm.nih.gov/entrez/query.fcgi?db=gene&cmd=Retrieve&dopt=full_report&list_uids=9391) | cytosolic iron-sulfur assembly component 1 |
| [Details](http://mirdb.org/cgi-bin/target_detail.cgi?targetID=3072432) | 1196 | 59 | hsa-miR-27a-3p | [HMBOX1](http://www.ncbi.nlm.nih.gov/entrez/query.fcgi?db=gene&cmd=Retrieve&dopt=full_report&list_uids=79618) | homeobox containing 1 |
| [Details](http://mirdb.org/cgi-bin/target_detail.cgi?targetID=3072436) | 1197 | 59 | hsa-miR-27a-3p | [DCC](http://www.ncbi.nlm.nih.gov/entrez/query.fcgi?db=gene&cmd=Retrieve&dopt=full_report&list_uids=1630) | DCC netrin 1 receptor |
| [Details](http://mirdb.org/cgi-bin/target_detail.cgi?targetID=3072447) | 1198 | 59 | hsa-miR-27a-3p | [CEPT1](http://www.ncbi.nlm.nih.gov/entrez/query.fcgi?db=gene&cmd=Retrieve&dopt=full_report&list_uids=10390) | choline/ethanolamine phosphotransferase 1 |
| [Details](http://mirdb.org/cgi-bin/target_detail.cgi?targetID=3072514) | 1199 | 59 | hsa-miR-27a-3p | [PRR27](http://www.ncbi.nlm.nih.gov/entrez/query.fcgi?db=gene&cmd=Retrieve&dopt=full_report&list_uids=401137) | proline rich 27 |
| [Details](http://mirdb.org/cgi-bin/target_detail.cgi?targetID=3072567) | 1200 | 59 | hsa-miR-27a-3p | [PTGFR](http://www.ncbi.nlm.nih.gov/entrez/query.fcgi?db=gene&cmd=Retrieve&dopt=full_report&list_uids=5737) | prostaglandin F receptor |
| [Details](http://mirdb.org/cgi-bin/target_detail.cgi?targetID=3072626) | 1201 | 59 | hsa-miR-27a-3p | [RAD9B](http://www.ncbi.nlm.nih.gov/entrez/query.fcgi?db=gene&cmd=Retrieve&dopt=full_report&list_uids=144715) | RAD9 checkpoint clamp component B |
| [Details](http://mirdb.org/cgi-bin/target_detail.cgi?targetID=3072628) | 1202 | 59 | hsa-miR-27a-3p | [PHF24](http://www.ncbi.nlm.nih.gov/entrez/query.fcgi?db=gene&cmd=Retrieve&dopt=full_report&list_uids=23349) | PHD finger protein 24 |
| [Details](http://mirdb.org/cgi-bin/target_detail.cgi?targetID=3072656) | 1203 | 59 | hsa-miR-27a-3p | [WNK1](http://www.ncbi.nlm.nih.gov/entrez/query.fcgi?db=gene&cmd=Retrieve&dopt=full_report&list_uids=65125) | WNK lysine deficient protein kinase 1 |
| [Details](http://mirdb.org/cgi-bin/target_detail.cgi?targetID=3072686) | 1204 | 59 | hsa-miR-27a-3p | [TMSB10](http://www.ncbi.nlm.nih.gov/entrez/query.fcgi?db=gene&cmd=Retrieve&dopt=full_report&list_uids=9168) | thymosin beta 10 |
| [Details](http://mirdb.org/cgi-bin/target_detail.cgi?targetID=3072794) | 1205 | 59 | hsa-miR-27a-3p | [URGCP](http://www.ncbi.nlm.nih.gov/entrez/query.fcgi?db=gene&cmd=Retrieve&dopt=full_report&list_uids=55665) | upregulator of cell proliferation |
| [Details](http://mirdb.org/cgi-bin/target_detail.cgi?targetID=3072841) | 1206 | 59 | hsa-miR-27a-3p | [MLPH](http://www.ncbi.nlm.nih.gov/entrez/query.fcgi?db=gene&cmd=Retrieve&dopt=full_report&list_uids=79083) | melanophilin |
| [Details](http://mirdb.org/cgi-bin/target_detail.cgi?targetID=3072858) | 1207 | 59 | hsa-miR-27a-3p | [BBC3](http://www.ncbi.nlm.nih.gov/entrez/query.fcgi?db=gene&cmd=Retrieve&dopt=full_report&list_uids=27113) | BCL2 binding component 3 |
| [Details](http://mirdb.org/cgi-bin/target_detail.cgi?targetID=3072929) | 1208 | 59 | hsa-miR-27a-3p | [EFNA5](http://www.ncbi.nlm.nih.gov/entrez/query.fcgi?db=gene&cmd=Retrieve&dopt=full_report&list_uids=1946) | ephrin A5 |
| [Details](http://mirdb.org/cgi-bin/target_detail.cgi?targetID=3073033) | 1209 | 59 | hsa-miR-27a-3p | [CDK6](http://www.ncbi.nlm.nih.gov/entrez/query.fcgi?db=gene&cmd=Retrieve&dopt=full_report&list_uids=1021) | cyclin dependent kinase 6 |
| [Details](http://mirdb.org/cgi-bin/target_detail.cgi?targetID=3073128) | 1210 | 59 | hsa-miR-27a-3p | [AK6](http://www.ncbi.nlm.nih.gov/entrez/query.fcgi?db=gene&cmd=Retrieve&dopt=full_report&list_uids=102157402) | adenylate kinase 6 |
| [Details](http://mirdb.org/cgi-bin/target_detail.cgi?targetID=3073134) | 1211 | 59 | hsa-miR-27a-3p | [CCDC85A](http://www.ncbi.nlm.nih.gov/entrez/query.fcgi?db=gene&cmd=Retrieve&dopt=full_report&list_uids=114800) | coiled-coil domain containing 85A |
| [Details](http://mirdb.org/cgi-bin/target_detail.cgi?targetID=3073142) | 1212 | 59 | hsa-miR-27a-3p | [DBF4B](http://www.ncbi.nlm.nih.gov/entrez/query.fcgi?db=gene&cmd=Retrieve&dopt=full_report&list_uids=80174) | DBF4 zinc finger B |
| [Details](http://mirdb.org/cgi-bin/target_detail.cgi?targetID=3073294) | 1213 | 59 | hsa-miR-27a-3p | [SH3TC2](http://www.ncbi.nlm.nih.gov/entrez/query.fcgi?db=gene&cmd=Retrieve&dopt=full_report&list_uids=79628) | SH3 domain and tetratricopeptide repeats 2 |
| [Details](http://mirdb.org/cgi-bin/target_detail.cgi?targetID=3073308) | 1214 | 59 | hsa-miR-27a-3p | [SHANK3](http://www.ncbi.nlm.nih.gov/entrez/query.fcgi?db=gene&cmd=Retrieve&dopt=full_report&list_uids=85358) | SH3 and multiple ankyrin repeat domains 3 |
| [Details](http://mirdb.org/cgi-bin/target_detail.cgi?targetID=3073384) | 1215 | 59 | hsa-miR-27a-3p | [CDK8](http://www.ncbi.nlm.nih.gov/entrez/query.fcgi?db=gene&cmd=Retrieve&dopt=full_report&list_uids=1024) | cyclin dependent kinase 8 |
| [Details](http://mirdb.org/cgi-bin/target_detail.cgi?targetID=3073394) | 1216 | 59 | hsa-miR-27a-3p | [FAM169A](http://www.ncbi.nlm.nih.gov/entrez/query.fcgi?db=gene&cmd=Retrieve&dopt=full_report&list_uids=26049) | family with sequence similarity 169 member A |
| [Details](http://mirdb.org/cgi-bin/target_detail.cgi?targetID=3073507) | 1217 | 59 | hsa-miR-27a-3p | [ZNF568](http://www.ncbi.nlm.nih.gov/entrez/query.fcgi?db=gene&cmd=Retrieve&dopt=full_report&list_uids=374900) | zinc finger protein 568 |
| [Details](http://mirdb.org/cgi-bin/target_detail.cgi?targetID=3073551) | 1218 | 59 | hsa-miR-27a-3p | [FECH](http://www.ncbi.nlm.nih.gov/entrez/query.fcgi?db=gene&cmd=Retrieve&dopt=full_report&list_uids=2235) | ferrochelatase |
| [Details](http://mirdb.org/cgi-bin/target_detail.cgi?targetID=3073570) | 1219 | 59 | hsa-miR-27a-3p | [TNIK](http://www.ncbi.nlm.nih.gov/entrez/query.fcgi?db=gene&cmd=Retrieve&dopt=full_report&list_uids=23043) | TRAF2 and NCK interacting kinase |
| [Details](http://mirdb.org/cgi-bin/target_detail.cgi?targetID=3073634) | 1220 | 59 | hsa-miR-27a-3p | [MITF](http://www.ncbi.nlm.nih.gov/entrez/query.fcgi?db=gene&cmd=Retrieve&dopt=full_report&list_uids=4286) | melanocyte inducing transcription factor |
| [Details](http://mirdb.org/cgi-bin/target_detail.cgi?targetID=3073693) | 1221 | 59 | hsa-miR-27a-3p | [SLFN11](http://www.ncbi.nlm.nih.gov/entrez/query.fcgi?db=gene&cmd=Retrieve&dopt=full_report&list_uids=91607) | schlafen family member 11 |
| [Details](http://mirdb.org/cgi-bin/target_detail.cgi?targetID=3073722) | 1222 | 59 | hsa-miR-27a-3p | [HSPA1L](http://www.ncbi.nlm.nih.gov/entrez/query.fcgi?db=gene&cmd=Retrieve&dopt=full_report&list_uids=3305) | heat shock protein family A (Hsp70) member 1 like |
| [Details](http://mirdb.org/cgi-bin/target_detail.cgi?targetID=3073784) | 1223 | 59 | hsa-miR-27a-3p | [ANKIB1](http://www.ncbi.nlm.nih.gov/entrez/query.fcgi?db=gene&cmd=Retrieve&dopt=full_report&list_uids=54467) | ankyrin repeat and IBR domain containing 1 |
| [Details](http://mirdb.org/cgi-bin/target_detail.cgi?targetID=3073834) | 1224 | 59 | hsa-miR-27a-3p | [MACC1](http://www.ncbi.nlm.nih.gov/entrez/query.fcgi?db=gene&cmd=Retrieve&dopt=full_report&list_uids=346389) | MET transcriptional regulator MACC1 |
| [Details](http://mirdb.org/cgi-bin/target_detail.cgi?targetID=3073836) | 1225 | 59 | hsa-miR-27a-3p | [ZNF346](http://www.ncbi.nlm.nih.gov/entrez/query.fcgi?db=gene&cmd=Retrieve&dopt=full_report&list_uids=23567) | zinc finger protein 346 |
| [Details](http://mirdb.org/cgi-bin/target_detail.cgi?targetID=3072429) | 1226 | 58 | hsa-miR-27a-3p | [FAM222B](http://www.ncbi.nlm.nih.gov/entrez/query.fcgi?db=gene&cmd=Retrieve&dopt=full_report&list_uids=55731) | family with sequence similarity 222 member B |
| [Details](http://mirdb.org/cgi-bin/target_detail.cgi?targetID=3072491) | 1227 | 58 | hsa-miR-27a-3p | [CFAP126](http://www.ncbi.nlm.nih.gov/entrez/query.fcgi?db=gene&cmd=Retrieve&dopt=full_report&list_uids=257177) | cilia and flagella associated protein 126 |
| [Details](http://mirdb.org/cgi-bin/target_detail.cgi?targetID=3072508) | 1228 | 58 | hsa-miR-27a-3p | [SPIDR](http://www.ncbi.nlm.nih.gov/entrez/query.fcgi?db=gene&cmd=Retrieve&dopt=full_report&list_uids=23514) | scaffold protein involved in DNA repair |
| [Details](http://mirdb.org/cgi-bin/target_detail.cgi?targetID=3072530) | 1229 | 58 | hsa-miR-27a-3p | [ZNF831](http://www.ncbi.nlm.nih.gov/entrez/query.fcgi?db=gene&cmd=Retrieve&dopt=full_report&list_uids=128611) | zinc finger protein 831 |
| [Details](http://mirdb.org/cgi-bin/target_detail.cgi?targetID=3072532) | 1230 | 58 | hsa-miR-27a-3p | [DAAM1](http://www.ncbi.nlm.nih.gov/entrez/query.fcgi?db=gene&cmd=Retrieve&dopt=full_report&list_uids=23002) | dishevelled associated activator of morphogenesis 1 |
| [Details](http://mirdb.org/cgi-bin/target_detail.cgi?targetID=3072534) | 1231 | 58 | hsa-miR-27a-3p | [GDF6](http://www.ncbi.nlm.nih.gov/entrez/query.fcgi?db=gene&cmd=Retrieve&dopt=full_report&list_uids=392255) | growth differentiation factor 6 |
| [Details](http://mirdb.org/cgi-bin/target_detail.cgi?targetID=3072541) | 1232 | 58 | hsa-miR-27a-3p | [SH2D3C](http://www.ncbi.nlm.nih.gov/entrez/query.fcgi?db=gene&cmd=Retrieve&dopt=full_report&list_uids=10044) | SH2 domain containing 3C |
| [Details](http://mirdb.org/cgi-bin/target_detail.cgi?targetID=3072542) | 1233 | 58 | hsa-miR-27a-3p | [SPRY4](http://www.ncbi.nlm.nih.gov/entrez/query.fcgi?db=gene&cmd=Retrieve&dopt=full_report&list_uids=81848) | sprouty RTK signaling antagonist 4 |
| [Details](http://mirdb.org/cgi-bin/target_detail.cgi?targetID=3072580) | 1234 | 58 | hsa-miR-27a-3p | [CCDC71](http://www.ncbi.nlm.nih.gov/entrez/query.fcgi?db=gene&cmd=Retrieve&dopt=full_report&list_uids=64925) | coiled-coil domain containing 71 |
| [Details](http://mirdb.org/cgi-bin/target_detail.cgi?targetID=3072588) | 1235 | 58 | hsa-miR-27a-3p | [ARHGEF37](http://www.ncbi.nlm.nih.gov/entrez/query.fcgi?db=gene&cmd=Retrieve&dopt=full_report&list_uids=389337) | Rho guanine nucleotide exchange factor 37 |
| [Details](http://mirdb.org/cgi-bin/target_detail.cgi?targetID=3072620) | 1236 | 58 | hsa-miR-27a-3p | [UBFD1](http://www.ncbi.nlm.nih.gov/entrez/query.fcgi?db=gene&cmd=Retrieve&dopt=full_report&list_uids=56061) | ubiquitin family domain containing 1 |
| [Details](http://mirdb.org/cgi-bin/target_detail.cgi?targetID=3072664) | 1237 | 58 | hsa-miR-27a-3p | [CD28](http://www.ncbi.nlm.nih.gov/entrez/query.fcgi?db=gene&cmd=Retrieve&dopt=full_report&list_uids=940) | CD28 molecule |
| [Details](http://mirdb.org/cgi-bin/target_detail.cgi?targetID=3072777) | 1238 | 58 | hsa-miR-27a-3p | [BBS7](http://www.ncbi.nlm.nih.gov/entrez/query.fcgi?db=gene&cmd=Retrieve&dopt=full_report&list_uids=55212) | Bardet-Biedl syndrome 7 |
| [Details](http://mirdb.org/cgi-bin/target_detail.cgi?targetID=3072902) | 1239 | 58 | hsa-miR-27a-3p | [BOD1L1](http://www.ncbi.nlm.nih.gov/entrez/query.fcgi?db=gene&cmd=Retrieve&dopt=full_report&list_uids=259282) | biorientation of chromosomes in cell division 1 like 1 |
| [Details](http://mirdb.org/cgi-bin/target_detail.cgi?targetID=3072930) | 1240 | 58 | hsa-miR-27a-3p | [MBOAT2](http://www.ncbi.nlm.nih.gov/entrez/query.fcgi?db=gene&cmd=Retrieve&dopt=full_report&list_uids=129642) | membrane bound O-acyltransferase domain containing 2 |
| [Details](http://mirdb.org/cgi-bin/target_detail.cgi?targetID=3072995) | 1241 | 58 | hsa-miR-27a-3p | [C19orf25](http://www.ncbi.nlm.nih.gov/entrez/query.fcgi?db=gene&cmd=Retrieve&dopt=full_report&list_uids=148223) | chromosome 19 open reading frame 25 |
| [Details](http://mirdb.org/cgi-bin/target_detail.cgi?targetID=3073009) | 1242 | 58 | hsa-miR-27a-3p | [ERO1B](http://www.ncbi.nlm.nih.gov/entrez/query.fcgi?db=gene&cmd=Retrieve&dopt=full_report&list_uids=56605) | endoplasmic reticulum oxidoreductase 1 beta |
| [Details](http://mirdb.org/cgi-bin/target_detail.cgi?targetID=3073010) | 1243 | 58 | hsa-miR-27a-3p | [ABCB5](http://www.ncbi.nlm.nih.gov/entrez/query.fcgi?db=gene&cmd=Retrieve&dopt=full_report&list_uids=340273) | ATP binding cassette subfamily B member 5 |
| [Details](http://mirdb.org/cgi-bin/target_detail.cgi?targetID=3073038) | 1244 | 58 | hsa-miR-27a-3p | [LATS2](http://www.ncbi.nlm.nih.gov/entrez/query.fcgi?db=gene&cmd=Retrieve&dopt=full_report&list_uids=26524) | large tumor suppressor kinase 2 |
| [Details](http://mirdb.org/cgi-bin/target_detail.cgi?targetID=3073324) | 1245 | 58 | hsa-miR-27a-3p | [CERS6](http://www.ncbi.nlm.nih.gov/entrez/query.fcgi?db=gene&cmd=Retrieve&dopt=full_report&list_uids=253782) | ceramide synthase 6 |
| [Details](http://mirdb.org/cgi-bin/target_detail.cgi?targetID=3073380) | 1246 | 58 | hsa-miR-27a-3p | [MFHAS1](http://www.ncbi.nlm.nih.gov/entrez/query.fcgi?db=gene&cmd=Retrieve&dopt=full_report&list_uids=9258) | malignant fibrous histiocytoma amplified sequence 1 |
| [Details](http://mirdb.org/cgi-bin/target_detail.cgi?targetID=3073389) | 1247 | 58 | hsa-miR-27a-3p | [SLC46A1](http://www.ncbi.nlm.nih.gov/entrez/query.fcgi?db=gene&cmd=Retrieve&dopt=full_report&list_uids=113235) | solute carrier family 46 member 1 |
| [Details](http://mirdb.org/cgi-bin/target_detail.cgi?targetID=3073395) | 1248 | 58 | hsa-miR-27a-3p | [PPIL6](http://www.ncbi.nlm.nih.gov/entrez/query.fcgi?db=gene&cmd=Retrieve&dopt=full_report&list_uids=285755) | peptidylprolyl isomerase like 6 |
| [Details](http://mirdb.org/cgi-bin/target_detail.cgi?targetID=3073411) | 1249 | 58 | hsa-miR-27a-3p | [TXNRD3](http://www.ncbi.nlm.nih.gov/entrez/query.fcgi?db=gene&cmd=Retrieve&dopt=full_report&list_uids=114112) | thioredoxin reductase 3 |
| [Details](http://mirdb.org/cgi-bin/target_detail.cgi?targetID=3073441) | 1250 | 58 | hsa-miR-27a-3p | [SUSD6](http://www.ncbi.nlm.nih.gov/entrez/query.fcgi?db=gene&cmd=Retrieve&dopt=full_report&list_uids=9766) | sushi domain containing 6 |
| [Details](http://mirdb.org/cgi-bin/target_detail.cgi?targetID=3073565) | 1251 | 58 | hsa-miR-27a-3p | [DPP10](http://www.ncbi.nlm.nih.gov/entrez/query.fcgi?db=gene&cmd=Retrieve&dopt=full_report&list_uids=57628) | dipeptidyl peptidase like 10 |
| [Details](http://mirdb.org/cgi-bin/target_detail.cgi?targetID=3073646) | 1252 | 58 | hsa-miR-27a-3p | [ZNF746](http://www.ncbi.nlm.nih.gov/entrez/query.fcgi?db=gene&cmd=Retrieve&dopt=full_report&list_uids=155061) | zinc finger protein 746 |
| [Details](http://mirdb.org/cgi-bin/target_detail.cgi?targetID=3073733) | 1253 | 58 | hsa-miR-27a-3p | [PCDH10](http://www.ncbi.nlm.nih.gov/entrez/query.fcgi?db=gene&cmd=Retrieve&dopt=full_report&list_uids=57575) | protocadherin 10 |
| [Details](http://mirdb.org/cgi-bin/target_detail.cgi?targetID=3073865) | 1254 | 58 | hsa-miR-27a-3p | [FAM104A](http://www.ncbi.nlm.nih.gov/entrez/query.fcgi?db=gene&cmd=Retrieve&dopt=full_report&list_uids=84923) | family with sequence similarity 104 member A |
| [Details](http://mirdb.org/cgi-bin/target_detail.cgi?targetID=3073866) | 1255 | 58 | hsa-miR-27a-3p | [RORA](http://www.ncbi.nlm.nih.gov/entrez/query.fcgi?db=gene&cmd=Retrieve&dopt=full_report&list_uids=6095) | RAR related orphan receptor A |
| [Details](http://mirdb.org/cgi-bin/target_detail.cgi?targetID=3073877) | 1256 | 58 | hsa-miR-27a-3p | [PITPNM2](http://www.ncbi.nlm.nih.gov/entrez/query.fcgi?db=gene&cmd=Retrieve&dopt=full_report&list_uids=57605) | phosphatidylinositol transfer protein membrane associated 2 |
| [Details](http://mirdb.org/cgi-bin/target_detail.cgi?targetID=3073899) | 1257 | 58 | hsa-miR-27a-3p | [MSTN](http://www.ncbi.nlm.nih.gov/entrez/query.fcgi?db=gene&cmd=Retrieve&dopt=full_report&list_uids=2660) | myostatin |
| [Details](http://mirdb.org/cgi-bin/target_detail.cgi?targetID=3072449) | 1258 | 57 | hsa-miR-27a-3p | [FNBP1](http://www.ncbi.nlm.nih.gov/entrez/query.fcgi?db=gene&cmd=Retrieve&dopt=full_report&list_uids=23048) | formin binding protein 1 |
| [Details](http://mirdb.org/cgi-bin/target_detail.cgi?targetID=3072458) | 1259 | 57 | hsa-miR-27a-3p | [PTPN9](http://www.ncbi.nlm.nih.gov/entrez/query.fcgi?db=gene&cmd=Retrieve&dopt=full_report&list_uids=5780) | protein tyrosine phosphatase, non-receptor type 9 |
| [Details](http://mirdb.org/cgi-bin/target_detail.cgi?targetID=3072498) | 1260 | 57 | hsa-miR-27a-3p | [NTN1](http://www.ncbi.nlm.nih.gov/entrez/query.fcgi?db=gene&cmd=Retrieve&dopt=full_report&list_uids=9423) | netrin 1 |
| [Details](http://mirdb.org/cgi-bin/target_detail.cgi?targetID=3072718) | 1261 | 57 | hsa-miR-27a-3p | [EDNRB](http://www.ncbi.nlm.nih.gov/entrez/query.fcgi?db=gene&cmd=Retrieve&dopt=full_report&list_uids=1910) | endothelin receptor type B |
| [Details](http://mirdb.org/cgi-bin/target_detail.cgi?targetID=3072724) | 1262 | 57 | hsa-miR-27a-3p | [CLN8](http://www.ncbi.nlm.nih.gov/entrez/query.fcgi?db=gene&cmd=Retrieve&dopt=full_report&list_uids=2055) | CLN8, transmembrane ER and ERGIC protein |
| [Details](http://mirdb.org/cgi-bin/target_detail.cgi?targetID=3072749) | 1263 | 57 | hsa-miR-27a-3p | [ADAMTS6](http://www.ncbi.nlm.nih.gov/entrez/query.fcgi?db=gene&cmd=Retrieve&dopt=full_report&list_uids=11174) | ADAM metallopeptidase with thrombospondin type 1 motif 6 |
| [Details](http://mirdb.org/cgi-bin/target_detail.cgi?targetID=3072811) | 1264 | 57 | hsa-miR-27a-3p | [EDNRA](http://www.ncbi.nlm.nih.gov/entrez/query.fcgi?db=gene&cmd=Retrieve&dopt=full_report&list_uids=1909) | endothelin receptor type A |
| [Details](http://mirdb.org/cgi-bin/target_detail.cgi?targetID=3072883) | 1265 | 57 | hsa-miR-27a-3p | [CRISP2](http://www.ncbi.nlm.nih.gov/entrez/query.fcgi?db=gene&cmd=Retrieve&dopt=full_report&list_uids=7180) | cysteine rich secretory protein 2 |
| [Details](http://mirdb.org/cgi-bin/target_detail.cgi?targetID=3072903) | 1266 | 57 | hsa-miR-27a-3p | [MAB21L4](http://www.ncbi.nlm.nih.gov/entrez/query.fcgi?db=gene&cmd=Retrieve&dopt=full_report&list_uids=79919) | mab-21 like 4 |
| [Details](http://mirdb.org/cgi-bin/target_detail.cgi?targetID=3072985) | 1267 | 57 | hsa-miR-27a-3p | [F3](http://www.ncbi.nlm.nih.gov/entrez/query.fcgi?db=gene&cmd=Retrieve&dopt=full_report&list_uids=2152) | coagulation factor III, tissue factor |
| [Details](http://mirdb.org/cgi-bin/target_detail.cgi?targetID=3073037) | 1268 | 57 | hsa-miR-27a-3p | [SSRP1](http://www.ncbi.nlm.nih.gov/entrez/query.fcgi?db=gene&cmd=Retrieve&dopt=full_report&list_uids=6749) | structure specific recognition protein 1 |
| [Details](http://mirdb.org/cgi-bin/target_detail.cgi?targetID=3073122) | 1269 | 57 | hsa-miR-27a-3p | [FAM177B](http://www.ncbi.nlm.nih.gov/entrez/query.fcgi?db=gene&cmd=Retrieve&dopt=full_report&list_uids=400823) | family with sequence similarity 177 member B |
| [Details](http://mirdb.org/cgi-bin/target_detail.cgi?targetID=3073214) | 1270 | 57 | hsa-miR-27a-3p | [FAM160B1](http://www.ncbi.nlm.nih.gov/entrez/query.fcgi?db=gene&cmd=Retrieve&dopt=full_report&list_uids=57700) | family with sequence similarity 160 member B1 |
| [Details](http://mirdb.org/cgi-bin/target_detail.cgi?targetID=3073232) | 1271 | 57 | hsa-miR-27a-3p | [SLC6A17](http://www.ncbi.nlm.nih.gov/entrez/query.fcgi?db=gene&cmd=Retrieve&dopt=full_report&list_uids=388662) | solute carrier family 6 member 17 |
| [Details](http://mirdb.org/cgi-bin/target_detail.cgi?targetID=3073259) | 1272 | 57 | hsa-miR-27a-3p | [PIK3R1](http://www.ncbi.nlm.nih.gov/entrez/query.fcgi?db=gene&cmd=Retrieve&dopt=full_report&list_uids=5295) | phosphoinositide-3-kinase regulatory subunit 1 |
| [Details](http://mirdb.org/cgi-bin/target_detail.cgi?targetID=3073397) | 1273 | 57 | hsa-miR-27a-3p | [SLC36A4](http://www.ncbi.nlm.nih.gov/entrez/query.fcgi?db=gene&cmd=Retrieve&dopt=full_report&list_uids=120103) | solute carrier family 36 member 4 |
| [Details](http://mirdb.org/cgi-bin/target_detail.cgi?targetID=3073447) | 1274 | 57 | hsa-miR-27a-3p | [NUS1](http://www.ncbi.nlm.nih.gov/entrez/query.fcgi?db=gene&cmd=Retrieve&dopt=full_report&list_uids=116150) | NUS1, dehydrodolichyl diphosphate synthase subunit |
| [Details](http://mirdb.org/cgi-bin/target_detail.cgi?targetID=3073534) | 1275 | 57 | hsa-miR-27a-3p | [AKAP7](http://www.ncbi.nlm.nih.gov/entrez/query.fcgi?db=gene&cmd=Retrieve&dopt=full_report&list_uids=9465) | A-kinase anchoring protein 7 |
| [Details](http://mirdb.org/cgi-bin/target_detail.cgi?targetID=3073563) | 1276 | 57 | hsa-miR-27a-3p | [TBC1D7-LOC100130357](http://www.ncbi.nlm.nih.gov/entrez/query.fcgi?db=gene&cmd=Retrieve&dopt=full_report&list_uids=107080638) | TBC1D7-LOC100130357 readthrough |
| [Details](http://mirdb.org/cgi-bin/target_detail.cgi?targetID=3073658) | 1277 | 57 | hsa-miR-27a-3p | [SERTAD3](http://www.ncbi.nlm.nih.gov/entrez/query.fcgi?db=gene&cmd=Retrieve&dopt=full_report&list_uids=29946) | SERTA domain containing 3 |
| [Details](http://mirdb.org/cgi-bin/target_detail.cgi?targetID=3073735) | 1278 | 57 | hsa-miR-27a-3p | [HEATR5A](http://www.ncbi.nlm.nih.gov/entrez/query.fcgi?db=gene&cmd=Retrieve&dopt=full_report&list_uids=25938) | HEAT repeat containing 5A |
| [Details](http://mirdb.org/cgi-bin/target_detail.cgi?targetID=3073776) | 1279 | 57 | hsa-miR-27a-3p | [PRDM16](http://www.ncbi.nlm.nih.gov/entrez/query.fcgi?db=gene&cmd=Retrieve&dopt=full_report&list_uids=63976) | PR/SET domain 16 |
| [Details](http://mirdb.org/cgi-bin/target_detail.cgi?targetID=3073788) | 1280 | 57 | hsa-miR-27a-3p | [LGALS8](http://www.ncbi.nlm.nih.gov/entrez/query.fcgi?db=gene&cmd=Retrieve&dopt=full_report&list_uids=3964) | galectin 8 |
| [Details](http://mirdb.org/cgi-bin/target_detail.cgi?targetID=3073827) | 1281 | 57 | hsa-miR-27a-3p | [MFSD2A](http://www.ncbi.nlm.nih.gov/entrez/query.fcgi?db=gene&cmd=Retrieve&dopt=full_report&list_uids=84879) | major facilitator superfamily domain containing 2A |
| [Details](http://mirdb.org/cgi-bin/target_detail.cgi?targetID=3073840) | 1282 | 57 | hsa-miR-27a-3p | [MICAL2](http://www.ncbi.nlm.nih.gov/entrez/query.fcgi?db=gene&cmd=Retrieve&dopt=full_report&list_uids=9645) | microtubule associated monooxygenase, calponin and LIM domain containing 2 |
| [Details](http://mirdb.org/cgi-bin/target_detail.cgi?targetID=3073861) | 1283 | 57 | hsa-miR-27a-3p | [ATG2A](http://www.ncbi.nlm.nih.gov/entrez/query.fcgi?db=gene&cmd=Retrieve&dopt=full_report&list_uids=23130) | autophagy related 2A |
| [Details](http://mirdb.org/cgi-bin/target_detail.cgi?targetID=3073897) | 1284 | 57 | hsa-miR-27a-3p | [LSAMP](http://www.ncbi.nlm.nih.gov/entrez/query.fcgi?db=gene&cmd=Retrieve&dopt=full_report&list_uids=4045) | limbic system associated membrane protein |
| [Details](http://mirdb.org/cgi-bin/target_detail.cgi?targetID=3073901) | 1285 | 57 | hsa-miR-27a-3p | [CREB3L2](http://www.ncbi.nlm.nih.gov/entrez/query.fcgi?db=gene&cmd=Retrieve&dopt=full_report&list_uids=64764) | cAMP responsive element binding protein 3 like 2 |
| [Details](http://mirdb.org/cgi-bin/target_detail.cgi?targetID=3072463) | 1286 | 56 | hsa-miR-27a-3p | [LRRC27](http://www.ncbi.nlm.nih.gov/entrez/query.fcgi?db=gene&cmd=Retrieve&dopt=full_report&list_uids=80313) | leucine rich repeat containing 27 |
| [Details](http://mirdb.org/cgi-bin/target_detail.cgi?targetID=3072503) | 1287 | 56 | hsa-miR-27a-3p | [PLD1](http://www.ncbi.nlm.nih.gov/entrez/query.fcgi?db=gene&cmd=Retrieve&dopt=full_report&list_uids=5337) | phospholipase D1 |
| [Details](http://mirdb.org/cgi-bin/target_detail.cgi?targetID=3072517) | 1288 | 56 | hsa-miR-27a-3p | [ZC3H6](http://www.ncbi.nlm.nih.gov/entrez/query.fcgi?db=gene&cmd=Retrieve&dopt=full_report&list_uids=376940) | zinc finger CCCH-type containing 6 |
| [Details](http://mirdb.org/cgi-bin/target_detail.cgi?targetID=3072616) | 1289 | 56 | hsa-miR-27a-3p | [EXOSC1](http://www.ncbi.nlm.nih.gov/entrez/query.fcgi?db=gene&cmd=Retrieve&dopt=full_report&list_uids=51013) | exosome component 1 |
| [Details](http://mirdb.org/cgi-bin/target_detail.cgi?targetID=3072622) | 1290 | 56 | hsa-miR-27a-3p | [ARNTL](http://www.ncbi.nlm.nih.gov/entrez/query.fcgi?db=gene&cmd=Retrieve&dopt=full_report&list_uids=406) | aryl hydrocarbon receptor nuclear translocator like |
| [Details](http://mirdb.org/cgi-bin/target_detail.cgi?targetID=3072665) | 1291 | 56 | hsa-miR-27a-3p | [EGLN1](http://www.ncbi.nlm.nih.gov/entrez/query.fcgi?db=gene&cmd=Retrieve&dopt=full_report&list_uids=54583) | egl-9 family hypoxia inducible factor 1 |
| [Details](http://mirdb.org/cgi-bin/target_detail.cgi?targetID=3072688) | 1292 | 56 | hsa-miR-27a-3p | [TCEANC2](http://www.ncbi.nlm.nih.gov/entrez/query.fcgi?db=gene&cmd=Retrieve&dopt=full_report&list_uids=127428) | transcription elongation factor A N-terminal and central domain containing 2 |
| [Details](http://mirdb.org/cgi-bin/target_detail.cgi?targetID=3072703) | 1293 | 56 | hsa-miR-27a-3p | [KCNA1](http://www.ncbi.nlm.nih.gov/entrez/query.fcgi?db=gene&cmd=Retrieve&dopt=full_report&list_uids=3736) | potassium voltage-gated channel subfamily A member 1 |
| [Details](http://mirdb.org/cgi-bin/target_detail.cgi?targetID=3072705) | 1294 | 56 | hsa-miR-27a-3p | [LIN7A](http://www.ncbi.nlm.nih.gov/entrez/query.fcgi?db=gene&cmd=Retrieve&dopt=full_report&list_uids=8825) | lin-7 homolog A, crumbs cell polarity complex component |
| [Details](http://mirdb.org/cgi-bin/target_detail.cgi?targetID=3072801) | 1295 | 56 | hsa-miR-27a-3p | [STX11](http://www.ncbi.nlm.nih.gov/entrez/query.fcgi?db=gene&cmd=Retrieve&dopt=full_report&list_uids=8676) | syntaxin 11 |
| [Details](http://mirdb.org/cgi-bin/target_detail.cgi?targetID=3072847) | 1296 | 56 | hsa-miR-27a-3p | [SOX8](http://www.ncbi.nlm.nih.gov/entrez/query.fcgi?db=gene&cmd=Retrieve&dopt=full_report&list_uids=30812) | SRY-box 8 |
| [Details](http://mirdb.org/cgi-bin/target_detail.cgi?targetID=3072920) | 1297 | 56 | hsa-miR-27a-3p | [USP49](http://www.ncbi.nlm.nih.gov/entrez/query.fcgi?db=gene&cmd=Retrieve&dopt=full_report&list_uids=25862) | ubiquitin specific peptidase 49 |
| [Details](http://mirdb.org/cgi-bin/target_detail.cgi?targetID=3072957) | 1298 | 56 | hsa-miR-27a-3p | [LHX8](http://www.ncbi.nlm.nih.gov/entrez/query.fcgi?db=gene&cmd=Retrieve&dopt=full_report&list_uids=431707) | LIM homeobox 8 |
| [Details](http://mirdb.org/cgi-bin/target_detail.cgi?targetID=3073008) | 1299 | 56 | hsa-miR-27a-3p | [ST20-MTHFS](http://www.ncbi.nlm.nih.gov/entrez/query.fcgi?db=gene&cmd=Retrieve&dopt=full_report&list_uids=100528021) | ST20-MTHFS readthrough |
| [Details](http://mirdb.org/cgi-bin/target_detail.cgi?targetID=3073024) | 1300 | 56 | hsa-miR-27a-3p | [VPS37B](http://www.ncbi.nlm.nih.gov/entrez/query.fcgi?db=gene&cmd=Retrieve&dopt=full_report&list_uids=79720) | VPS37B, ESCRT-I subunit |
| [Details](http://mirdb.org/cgi-bin/target_detail.cgi?targetID=3073047) | 1301 | 56 | hsa-miR-27a-3p | [TMEM121B](http://www.ncbi.nlm.nih.gov/entrez/query.fcgi?db=gene&cmd=Retrieve&dopt=full_report&list_uids=27439) | transmembrane protein 121B |
| [Details](http://mirdb.org/cgi-bin/target_detail.cgi?targetID=3073064) | 1302 | 56 | hsa-miR-27a-3p | [FMN1](http://www.ncbi.nlm.nih.gov/entrez/query.fcgi?db=gene&cmd=Retrieve&dopt=full_report&list_uids=342184) | formin 1 |
| [Details](http://mirdb.org/cgi-bin/target_detail.cgi?targetID=3073102) | 1303 | 56 | hsa-miR-27a-3p | [CCDC92](http://www.ncbi.nlm.nih.gov/entrez/query.fcgi?db=gene&cmd=Retrieve&dopt=full_report&list_uids=80212) | coiled-coil domain containing 92 |
| [Details](http://mirdb.org/cgi-bin/target_detail.cgi?targetID=3073120) | 1304 | 56 | hsa-miR-27a-3p | [KLHL4](http://www.ncbi.nlm.nih.gov/entrez/query.fcgi?db=gene&cmd=Retrieve&dopt=full_report&list_uids=56062) | kelch like family member 4 |
| [Details](http://mirdb.org/cgi-bin/target_detail.cgi?targetID=3073210) | 1305 | 56 | hsa-miR-27a-3p | [GCSAM](http://www.ncbi.nlm.nih.gov/entrez/query.fcgi?db=gene&cmd=Retrieve&dopt=full_report&list_uids=257144) | germinal center associated signaling and motility |
| [Details](http://mirdb.org/cgi-bin/target_detail.cgi?targetID=3073240) | 1306 | 56 | hsa-miR-27a-3p | [ENPP5](http://www.ncbi.nlm.nih.gov/entrez/query.fcgi?db=gene&cmd=Retrieve&dopt=full_report&list_uids=59084) | ectonucleotide pyrophosphatase/phosphodiesterase 5 (putative) |
| [Details](http://mirdb.org/cgi-bin/target_detail.cgi?targetID=3073315) | 1307 | 56 | hsa-miR-27a-3p | [GTF3C4](http://www.ncbi.nlm.nih.gov/entrez/query.fcgi?db=gene&cmd=Retrieve&dopt=full_report&list_uids=9329) | general transcription factor IIIC subunit 4 |
| [Details](http://mirdb.org/cgi-bin/target_detail.cgi?targetID=3073317) | 1308 | 56 | hsa-miR-27a-3p | [MSN](http://www.ncbi.nlm.nih.gov/entrez/query.fcgi?db=gene&cmd=Retrieve&dopt=full_report&list_uids=4478) | moesin |
| [Details](http://mirdb.org/cgi-bin/target_detail.cgi?targetID=3073320) | 1309 | 56 | hsa-miR-27a-3p | [UBE2D1](http://www.ncbi.nlm.nih.gov/entrez/query.fcgi?db=gene&cmd=Retrieve&dopt=full_report&list_uids=7321) | ubiquitin conjugating enzyme E2 D1 |
| [Details](http://mirdb.org/cgi-bin/target_detail.cgi?targetID=3073363) | 1310 | 56 | hsa-miR-27a-3p | [ARHGEF38](http://www.ncbi.nlm.nih.gov/entrez/query.fcgi?db=gene&cmd=Retrieve&dopt=full_report&list_uids=54848) | Rho guanine nucleotide exchange factor 38 |
| [Details](http://mirdb.org/cgi-bin/target_detail.cgi?targetID=3073386) | 1311 | 56 | hsa-miR-27a-3p | [MT1G](http://www.ncbi.nlm.nih.gov/entrez/query.fcgi?db=gene&cmd=Retrieve&dopt=full_report&list_uids=4495) | metallothionein 1G |
| [Details](http://mirdb.org/cgi-bin/target_detail.cgi?targetID=3073431) | 1312 | 56 | hsa-miR-27a-3p | [KDM4C](http://www.ncbi.nlm.nih.gov/entrez/query.fcgi?db=gene&cmd=Retrieve&dopt=full_report&list_uids=23081) | lysine demethylase 4C |
| [Details](http://mirdb.org/cgi-bin/target_detail.cgi?targetID=3073446) | 1313 | 56 | hsa-miR-27a-3p | [PGAP1](http://www.ncbi.nlm.nih.gov/entrez/query.fcgi?db=gene&cmd=Retrieve&dopt=full_report&list_uids=80055) | post-GPI attachment to proteins 1 |
| [Details](http://mirdb.org/cgi-bin/target_detail.cgi?targetID=3073528) | 1314 | 56 | hsa-miR-27a-3p | [DENND1B](http://www.ncbi.nlm.nih.gov/entrez/query.fcgi?db=gene&cmd=Retrieve&dopt=full_report&list_uids=163486) | DENN domain containing 1B |
| [Details](http://mirdb.org/cgi-bin/target_detail.cgi?targetID=3073556) | 1315 | 56 | hsa-miR-27a-3p | [SLC49A4](http://www.ncbi.nlm.nih.gov/entrez/query.fcgi?db=gene&cmd=Retrieve&dopt=full_report&list_uids=84925) | solute carrier family 49 member 4 |
| [Details](http://mirdb.org/cgi-bin/target_detail.cgi?targetID=3073588) | 1316 | 56 | hsa-miR-27a-3p | [ZNF334](http://www.ncbi.nlm.nih.gov/entrez/query.fcgi?db=gene&cmd=Retrieve&dopt=full_report&list_uids=55713) | zinc finger protein 334 |
| [Details](http://mirdb.org/cgi-bin/target_detail.cgi?targetID=3073637) | 1317 | 56 | hsa-miR-27a-3p | [SESN1](http://www.ncbi.nlm.nih.gov/entrez/query.fcgi?db=gene&cmd=Retrieve&dopt=full_report&list_uids=27244) | sestrin 1 |
| [Details](http://mirdb.org/cgi-bin/target_detail.cgi?targetID=3073686) | 1318 | 56 | hsa-miR-27a-3p | [RFXAP](http://www.ncbi.nlm.nih.gov/entrez/query.fcgi?db=gene&cmd=Retrieve&dopt=full_report&list_uids=5994) | regulatory factor X associated protein |
| [Details](http://mirdb.org/cgi-bin/target_detail.cgi?targetID=3073718) | 1319 | 56 | hsa-miR-27a-3p | [TFPI2](http://www.ncbi.nlm.nih.gov/entrez/query.fcgi?db=gene&cmd=Retrieve&dopt=full_report&list_uids=7980) | tissue factor pathway inhibitor 2 |
| [Details](http://mirdb.org/cgi-bin/target_detail.cgi?targetID=3073726) | 1320 | 56 | hsa-miR-27a-3p | [CSMD3](http://www.ncbi.nlm.nih.gov/entrez/query.fcgi?db=gene&cmd=Retrieve&dopt=full_report&list_uids=114788) | CUB and Sushi multiple domains 3 |
| [Details](http://mirdb.org/cgi-bin/target_detail.cgi?targetID=3073748) | 1321 | 56 | hsa-miR-27a-3p | [MAIP1](http://www.ncbi.nlm.nih.gov/entrez/query.fcgi?db=gene&cmd=Retrieve&dopt=full_report&list_uids=79568) | matrix AAA peptidase interacting protein 1 |
| [Details](http://mirdb.org/cgi-bin/target_detail.cgi?targetID=3073749) | 1322 | 56 | hsa-miR-27a-3p | [SPTY2D1](http://www.ncbi.nlm.nih.gov/entrez/query.fcgi?db=gene&cmd=Retrieve&dopt=full_report&list_uids=144108) | SPT2 chromatin protein domain containing 1 |
| [Details](http://mirdb.org/cgi-bin/target_detail.cgi?targetID=3073751) | 1323 | 56 | hsa-miR-27a-3p | [MTHFS](http://www.ncbi.nlm.nih.gov/entrez/query.fcgi?db=gene&cmd=Retrieve&dopt=full_report&list_uids=10588) | methenyltetrahydrofolate synthetase |
| [Details](http://mirdb.org/cgi-bin/target_detail.cgi?targetID=3073774) | 1324 | 56 | hsa-miR-27a-3p | [MT1H](http://www.ncbi.nlm.nih.gov/entrez/query.fcgi?db=gene&cmd=Retrieve&dopt=full_report&list_uids=4496) | metallothionein 1H |
| [Details](http://mirdb.org/cgi-bin/target_detail.cgi?targetID=3073793) | 1325 | 56 | hsa-miR-27a-3p | [SGMS1](http://www.ncbi.nlm.nih.gov/entrez/query.fcgi?db=gene&cmd=Retrieve&dopt=full_report&list_uids=259230) | sphingomyelin synthase 1 |
| [Details](http://mirdb.org/cgi-bin/target_detail.cgi?targetID=3073849) | 1326 | 56 | hsa-miR-27a-3p | [SAP30BP](http://www.ncbi.nlm.nih.gov/entrez/query.fcgi?db=gene&cmd=Retrieve&dopt=full_report&list_uids=29115) | SAP30 binding protein |
| [Details](http://mirdb.org/cgi-bin/target_detail.cgi?targetID=3073857) | 1327 | 56 | hsa-miR-27a-3p | [ZNF678](http://www.ncbi.nlm.nih.gov/entrez/query.fcgi?db=gene&cmd=Retrieve&dopt=full_report&list_uids=339500) | zinc finger protein 678 |
| [Details](http://mirdb.org/cgi-bin/target_detail.cgi?targetID=3073895) | 1328 | 56 | hsa-miR-27a-3p | [SIPA1L3](http://www.ncbi.nlm.nih.gov/entrez/query.fcgi?db=gene&cmd=Retrieve&dopt=full_report&list_uids=23094) | signal induced proliferation associated 1 like 3 |
| [Details](http://mirdb.org/cgi-bin/target_detail.cgi?targetID=3072657) | 1329 | 55 | hsa-miR-27a-3p | [EIF2S2](http://www.ncbi.nlm.nih.gov/entrez/query.fcgi?db=gene&cmd=Retrieve&dopt=full_report&list_uids=8894) | eukaryotic translation initiation factor 2 subunit beta |
| [Details](http://mirdb.org/cgi-bin/target_detail.cgi?targetID=3072659) | 1330 | 55 | hsa-miR-27a-3p | [ZMYND11](http://www.ncbi.nlm.nih.gov/entrez/query.fcgi?db=gene&cmd=Retrieve&dopt=full_report&list_uids=10771) | zinc finger MYND-type containing 11 |
| [Details](http://mirdb.org/cgi-bin/target_detail.cgi?targetID=3072662) | 1331 | 55 | hsa-miR-27a-3p | [SEMA4D](http://www.ncbi.nlm.nih.gov/entrez/query.fcgi?db=gene&cmd=Retrieve&dopt=full_report&list_uids=10507) | semaphorin 4D |
| [Details](http://mirdb.org/cgi-bin/target_detail.cgi?targetID=3072709) | 1332 | 55 | hsa-miR-27a-3p | [RAD51B](http://www.ncbi.nlm.nih.gov/entrez/query.fcgi?db=gene&cmd=Retrieve&dopt=full_report&list_uids=5890) | RAD51 paralog B |
| [Details](http://mirdb.org/cgi-bin/target_detail.cgi?targetID=3072734) | 1333 | 55 | hsa-miR-27a-3p | [CNTN1](http://www.ncbi.nlm.nih.gov/entrez/query.fcgi?db=gene&cmd=Retrieve&dopt=full_report&list_uids=1272) | contactin 1 |
| [Details](http://mirdb.org/cgi-bin/target_detail.cgi?targetID=3072739) | 1334 | 55 | hsa-miR-27a-3p | [PRICKLE2](http://www.ncbi.nlm.nih.gov/entrez/query.fcgi?db=gene&cmd=Retrieve&dopt=full_report&list_uids=166336) | prickle planar cell polarity protein 2 |
| [Details](http://mirdb.org/cgi-bin/target_detail.cgi?targetID=3072813) | 1335 | 55 | hsa-miR-27a-3p | [SPATA2](http://www.ncbi.nlm.nih.gov/entrez/query.fcgi?db=gene&cmd=Retrieve&dopt=full_report&list_uids=9825) | spermatogenesis associated 2 |
| [Details](http://mirdb.org/cgi-bin/target_detail.cgi?targetID=3072876) | 1336 | 55 | hsa-miR-27a-3p | [APOO](http://www.ncbi.nlm.nih.gov/entrez/query.fcgi?db=gene&cmd=Retrieve&dopt=full_report&list_uids=79135) | apolipoprotein O |
| [Details](http://mirdb.org/cgi-bin/target_detail.cgi?targetID=3072897) | 1337 | 55 | hsa-miR-27a-3p | [ZNF470](http://www.ncbi.nlm.nih.gov/entrez/query.fcgi?db=gene&cmd=Retrieve&dopt=full_report&list_uids=388566) | zinc finger protein 470 |
| [Details](http://mirdb.org/cgi-bin/target_detail.cgi?targetID=3072938) | 1338 | 55 | hsa-miR-27a-3p | [KCNJ16](http://www.ncbi.nlm.nih.gov/entrez/query.fcgi?db=gene&cmd=Retrieve&dopt=full_report&list_uids=3773) | potassium voltage-gated channel subfamily J member 16 |
| [Details](http://mirdb.org/cgi-bin/target_detail.cgi?targetID=3072949) | 1339 | 55 | hsa-miR-27a-3p | [LIF](http://www.ncbi.nlm.nih.gov/entrez/query.fcgi?db=gene&cmd=Retrieve&dopt=full_report&list_uids=3976) | LIF, interleukin 6 family cytokine |
| [Details](http://mirdb.org/cgi-bin/target_detail.cgi?targetID=3072977) | 1340 | 55 | hsa-miR-27a-3p | [ALCAM](http://www.ncbi.nlm.nih.gov/entrez/query.fcgi?db=gene&cmd=Retrieve&dopt=full_report&list_uids=214) | activated leukocyte cell adhesion molecule |
| [Details](http://mirdb.org/cgi-bin/target_detail.cgi?targetID=3072999) | 1341 | 55 | hsa-miR-27a-3p | [STX5](http://www.ncbi.nlm.nih.gov/entrez/query.fcgi?db=gene&cmd=Retrieve&dopt=full_report&list_uids=6811) | syntaxin 5 |
| [Details](http://mirdb.org/cgi-bin/target_detail.cgi?targetID=3073002) | 1342 | 55 | hsa-miR-27a-3p | [CORIN](http://www.ncbi.nlm.nih.gov/entrez/query.fcgi?db=gene&cmd=Retrieve&dopt=full_report&list_uids=10699) | corin, serine peptidase |
| [Details](http://mirdb.org/cgi-bin/target_detail.cgi?targetID=3073032) | 1343 | 55 | hsa-miR-27a-3p | [STOX1](http://www.ncbi.nlm.nih.gov/entrez/query.fcgi?db=gene&cmd=Retrieve&dopt=full_report&list_uids=219736) | storkhead box 1 |
| [Details](http://mirdb.org/cgi-bin/target_detail.cgi?targetID=3073055) | 1344 | 55 | hsa-miR-27a-3p | [PSRC1](http://www.ncbi.nlm.nih.gov/entrez/query.fcgi?db=gene&cmd=Retrieve&dopt=full_report&list_uids=84722) | proline and serine rich coiled-coil 1 |
| [Details](http://mirdb.org/cgi-bin/target_detail.cgi?targetID=3073090) | 1345 | 55 | hsa-miR-27a-3p | [SLC17A6](http://www.ncbi.nlm.nih.gov/entrez/query.fcgi?db=gene&cmd=Retrieve&dopt=full_report&list_uids=57084) | solute carrier family 17 member 6 |
| [Details](http://mirdb.org/cgi-bin/target_detail.cgi?targetID=3073194) | 1346 | 55 | hsa-miR-27a-3p | [RCL1](http://www.ncbi.nlm.nih.gov/entrez/query.fcgi?db=gene&cmd=Retrieve&dopt=full_report&list_uids=10171) | RNA terminal phosphate cyclase like 1 |
| [Details](http://mirdb.org/cgi-bin/target_detail.cgi?targetID=3073212) | 1347 | 55 | hsa-miR-27a-3p | [RUBCNL](http://www.ncbi.nlm.nih.gov/entrez/query.fcgi?db=gene&cmd=Retrieve&dopt=full_report&list_uids=80183) | rubicon like autophagy enhancer |
| [Details](http://mirdb.org/cgi-bin/target_detail.cgi?targetID=3073239) | 1348 | 55 | hsa-miR-27a-3p | [ENOX2](http://www.ncbi.nlm.nih.gov/entrez/query.fcgi?db=gene&cmd=Retrieve&dopt=full_report&list_uids=10495) | ecto-NOX disulfide-thiol exchanger 2 |
| [Details](http://mirdb.org/cgi-bin/target_detail.cgi?targetID=3073247) | 1349 | 55 | hsa-miR-27a-3p | [VPS53](http://www.ncbi.nlm.nih.gov/entrez/query.fcgi?db=gene&cmd=Retrieve&dopt=full_report&list_uids=55275) | VPS53, GARP complex subunit |
| [Details](http://mirdb.org/cgi-bin/target_detail.cgi?targetID=3073344) | 1350 | 55 | hsa-miR-27a-3p | [GOLT1B](http://www.ncbi.nlm.nih.gov/entrez/query.fcgi?db=gene&cmd=Retrieve&dopt=full_report&list_uids=51026) | golgi transport 1B |
| [Details](http://mirdb.org/cgi-bin/target_detail.cgi?targetID=3073346) | 1351 | 55 | hsa-miR-27a-3p | [ITCH](http://www.ncbi.nlm.nih.gov/entrez/query.fcgi?db=gene&cmd=Retrieve&dopt=full_report&list_uids=83737) | itchy E3 ubiquitin protein ligase |
| [Details](http://mirdb.org/cgi-bin/target_detail.cgi?targetID=3073371) | 1352 | 55 | hsa-miR-27a-3p | [ANTXR2](http://www.ncbi.nlm.nih.gov/entrez/query.fcgi?db=gene&cmd=Retrieve&dopt=full_report&list_uids=118429) | ANTXR cell adhesion molecule 2 |
| [Details](http://mirdb.org/cgi-bin/target_detail.cgi?targetID=3073456) | 1353 | 55 | hsa-miR-27a-3p | [OGFRL1](http://www.ncbi.nlm.nih.gov/entrez/query.fcgi?db=gene&cmd=Retrieve&dopt=full_report&list_uids=79627) | opioid growth factor receptor like 1 |
| [Details](http://mirdb.org/cgi-bin/target_detail.cgi?targetID=3073519) | 1354 | 55 | hsa-miR-27a-3p | [STK10](http://www.ncbi.nlm.nih.gov/entrez/query.fcgi?db=gene&cmd=Retrieve&dopt=full_report&list_uids=6793) | serine/threonine kinase 10 |
| [Details](http://mirdb.org/cgi-bin/target_detail.cgi?targetID=3073659) | 1355 | 55 | hsa-miR-27a-3p | [VANGL2](http://www.ncbi.nlm.nih.gov/entrez/query.fcgi?db=gene&cmd=Retrieve&dopt=full_report&list_uids=57216) | VANGL planar cell polarity protein 2 |
| [Details](http://mirdb.org/cgi-bin/target_detail.cgi?targetID=3073664) | 1356 | 55 | hsa-miR-27a-3p | [PKN2](http://www.ncbi.nlm.nih.gov/entrez/query.fcgi?db=gene&cmd=Retrieve&dopt=full_report&list_uids=5586) | protein kinase N2 |
| [Details](http://mirdb.org/cgi-bin/target_detail.cgi?targetID=3073721) | 1357 | 55 | hsa-miR-27a-3p | [YAP1](http://www.ncbi.nlm.nih.gov/entrez/query.fcgi?db=gene&cmd=Retrieve&dopt=full_report&list_uids=10413) | Yes associated protein 1 |
| [Details](http://mirdb.org/cgi-bin/target_detail.cgi?targetID=3073797) | 1358 | 55 | hsa-miR-27a-3p | [POM121](http://www.ncbi.nlm.nih.gov/entrez/query.fcgi?db=gene&cmd=Retrieve&dopt=full_report&list_uids=9883) | POM121 transmembrane nucleoporin |
| [Details](http://mirdb.org/cgi-bin/target_detail.cgi?targetID=3073825) | 1359 | 55 | hsa-miR-27a-3p | [PAXBP1](http://www.ncbi.nlm.nih.gov/entrez/query.fcgi?db=gene&cmd=Retrieve&dopt=full_report&list_uids=94104) | PAX3 and PAX7 binding protein 1 |
| [Details](http://mirdb.org/cgi-bin/target_detail.cgi?targetID=3073846) | 1360 | 55 | hsa-miR-27a-3p | [PARPBP](http://www.ncbi.nlm.nih.gov/entrez/query.fcgi?db=gene&cmd=Retrieve&dopt=full_report&list_uids=55010) | PARP1 binding protein |
| [Details](http://mirdb.org/cgi-bin/target_detail.cgi?targetID=3073902) | 1361 | 55 | hsa-miR-27a-3p | [ASB11](http://www.ncbi.nlm.nih.gov/entrez/query.fcgi?db=gene&cmd=Retrieve&dopt=full_report&list_uids=140456) | ankyrin repeat and SOCS box containing 11 |
| [Details](http://mirdb.org/cgi-bin/target_detail.cgi?targetID=3072470) | 1362 | 54 | hsa-miR-27a-3p | [FCHSD2](http://www.ncbi.nlm.nih.gov/entrez/query.fcgi?db=gene&cmd=Retrieve&dopt=full_report&list_uids=9873) | FCH and double SH3 domains 2 |
| [Details](http://mirdb.org/cgi-bin/target_detail.cgi?targetID=3072477) | 1363 | 54 | hsa-miR-27a-3p | [FBXO30](http://www.ncbi.nlm.nih.gov/entrez/query.fcgi?db=gene&cmd=Retrieve&dopt=full_report&list_uids=84085) | F-box protein 30 |
| [Details](http://mirdb.org/cgi-bin/target_detail.cgi?targetID=3072497) | 1364 | 54 | hsa-miR-27a-3p | [RBM3](http://www.ncbi.nlm.nih.gov/entrez/query.fcgi?db=gene&cmd=Retrieve&dopt=full_report&list_uids=5935) | RNA binding motif protein 3 |
| [Details](http://mirdb.org/cgi-bin/target_detail.cgi?targetID=3072583) | 1365 | 54 | hsa-miR-27a-3p | [UFL1](http://www.ncbi.nlm.nih.gov/entrez/query.fcgi?db=gene&cmd=Retrieve&dopt=full_report&list_uids=23376) | UFM1 specific ligase 1 |
| [Details](http://mirdb.org/cgi-bin/target_detail.cgi?targetID=3072670) | 1366 | 54 | hsa-miR-27a-3p | [NEMP1](http://www.ncbi.nlm.nih.gov/entrez/query.fcgi?db=gene&cmd=Retrieve&dopt=full_report&list_uids=23306) | nuclear envelope integral membrane protein 1 |
| [Details](http://mirdb.org/cgi-bin/target_detail.cgi?targetID=3072775) | 1367 | 54 | hsa-miR-27a-3p | [TSTD3](http://www.ncbi.nlm.nih.gov/entrez/query.fcgi?db=gene&cmd=Retrieve&dopt=full_report&list_uids=100130890) | thiosulfate sulfurtransferase like domain containing 3 |
| [Details](http://mirdb.org/cgi-bin/target_detail.cgi?targetID=3072867) | 1368 | 54 | hsa-miR-27a-3p | [ZNF385A](http://www.ncbi.nlm.nih.gov/entrez/query.fcgi?db=gene&cmd=Retrieve&dopt=full_report&list_uids=25946) | zinc finger protein 385A |
| [Details](http://mirdb.org/cgi-bin/target_detail.cgi?targetID=3072868) | 1369 | 54 | hsa-miR-27a-3p | [KCNQ3](http://www.ncbi.nlm.nih.gov/entrez/query.fcgi?db=gene&cmd=Retrieve&dopt=full_report&list_uids=3786) | potassium voltage-gated channel subfamily Q member 3 |
| [Details](http://mirdb.org/cgi-bin/target_detail.cgi?targetID=3073042) | 1370 | 54 | hsa-miR-27a-3p | [MAP3K12](http://www.ncbi.nlm.nih.gov/entrez/query.fcgi?db=gene&cmd=Retrieve&dopt=full_report&list_uids=7786) | mitogen-activated protein kinase kinase kinase 12 |
| [Details](http://mirdb.org/cgi-bin/target_detail.cgi?targetID=3073049) | 1371 | 54 | hsa-miR-27a-3p | [GOLGA6C](http://www.ncbi.nlm.nih.gov/entrez/query.fcgi?db=gene&cmd=Retrieve&dopt=full_report&list_uids=653641) | golgin A6 family member C |
| [Details](http://mirdb.org/cgi-bin/target_detail.cgi?targetID=3073260) | 1372 | 54 | hsa-miR-27a-3p | [RAB3IP](http://www.ncbi.nlm.nih.gov/entrez/query.fcgi?db=gene&cmd=Retrieve&dopt=full_report&list_uids=117177) | RAB3A interacting protein |
| [Details](http://mirdb.org/cgi-bin/target_detail.cgi?targetID=3073271) | 1373 | 54 | hsa-miR-27a-3p | [USP21](http://www.ncbi.nlm.nih.gov/entrez/query.fcgi?db=gene&cmd=Retrieve&dopt=full_report&list_uids=27005) | ubiquitin specific peptidase 21 |
| [Details](http://mirdb.org/cgi-bin/target_detail.cgi?targetID=3073290) | 1374 | 54 | hsa-miR-27a-3p | [DNAJB9](http://www.ncbi.nlm.nih.gov/entrez/query.fcgi?db=gene&cmd=Retrieve&dopt=full_report&list_uids=4189) | DnaJ heat shock protein family (Hsp40) member B9 |
| [Details](http://mirdb.org/cgi-bin/target_detail.cgi?targetID=3073361) | 1375 | 54 | hsa-miR-27a-3p | [PDLIM4](http://www.ncbi.nlm.nih.gov/entrez/query.fcgi?db=gene&cmd=Retrieve&dopt=full_report&list_uids=8572) | PDZ and LIM domain 4 |
| [Details](http://mirdb.org/cgi-bin/target_detail.cgi?targetID=3073377) | 1376 | 54 | hsa-miR-27a-3p | [TGFBR1](http://www.ncbi.nlm.nih.gov/entrez/query.fcgi?db=gene&cmd=Retrieve&dopt=full_report&list_uids=7046) | transforming growth factor beta receptor 1 |
| [Details](http://mirdb.org/cgi-bin/target_detail.cgi?targetID=3073385) | 1377 | 54 | hsa-miR-27a-3p | [HNF4G](http://www.ncbi.nlm.nih.gov/entrez/query.fcgi?db=gene&cmd=Retrieve&dopt=full_report&list_uids=3174) | hepatocyte nuclear factor 4 gamma |
| [Details](http://mirdb.org/cgi-bin/target_detail.cgi?targetID=3073408) | 1378 | 54 | hsa-miR-27a-3p | [TBX5](http://www.ncbi.nlm.nih.gov/entrez/query.fcgi?db=gene&cmd=Retrieve&dopt=full_report&list_uids=6910) | T-box 5 |
| [Details](http://mirdb.org/cgi-bin/target_detail.cgi?targetID=3073438) | 1379 | 54 | hsa-miR-27a-3p | [FCMR](http://www.ncbi.nlm.nih.gov/entrez/query.fcgi?db=gene&cmd=Retrieve&dopt=full_report&list_uids=9214) | Fc fragment of IgM receptor |
| [Details](http://mirdb.org/cgi-bin/target_detail.cgi?targetID=3073477) | 1380 | 54 | hsa-miR-27a-3p | [SLX4IP](http://www.ncbi.nlm.nih.gov/entrez/query.fcgi?db=gene&cmd=Retrieve&dopt=full_report&list_uids=128710) | SLX4 interacting protein |
| [Details](http://mirdb.org/cgi-bin/target_detail.cgi?targetID=3073531) | 1381 | 54 | hsa-miR-27a-3p | [ANKRD18A](http://www.ncbi.nlm.nih.gov/entrez/query.fcgi?db=gene&cmd=Retrieve&dopt=full_report&list_uids=253650) | ankyrin repeat domain 18A |
| [Details](http://mirdb.org/cgi-bin/target_detail.cgi?targetID=3073593) | 1382 | 54 | hsa-miR-27a-3p | [BEND7](http://www.ncbi.nlm.nih.gov/entrez/query.fcgi?db=gene&cmd=Retrieve&dopt=full_report&list_uids=222389) | BEN domain containing 7 |
| [Details](http://mirdb.org/cgi-bin/target_detail.cgi?targetID=3073613) | 1383 | 54 | hsa-miR-27a-3p | [KDM4B](http://www.ncbi.nlm.nih.gov/entrez/query.fcgi?db=gene&cmd=Retrieve&dopt=full_report&list_uids=23030) | lysine demethylase 4B |
| [Details](http://mirdb.org/cgi-bin/target_detail.cgi?targetID=3073620) | 1384 | 54 | hsa-miR-27a-3p | [PHF13](http://www.ncbi.nlm.nih.gov/entrez/query.fcgi?db=gene&cmd=Retrieve&dopt=full_report&list_uids=148479) | PHD finger protein 13 |
| [Details](http://mirdb.org/cgi-bin/target_detail.cgi?targetID=3073705) | 1385 | 54 | hsa-miR-27a-3p | [GABRB3](http://www.ncbi.nlm.nih.gov/entrez/query.fcgi?db=gene&cmd=Retrieve&dopt=full_report&list_uids=2562) | gamma-aminobutyric acid type A receptor beta3 subunit |
| [Details](http://mirdb.org/cgi-bin/target_detail.cgi?targetID=3073711) | 1386 | 54 | hsa-miR-27a-3p | [TENT4A](http://www.ncbi.nlm.nih.gov/entrez/query.fcgi?db=gene&cmd=Retrieve&dopt=full_report&list_uids=11044) | terminal nucleotidyltransferase 4A |
| [Details](http://mirdb.org/cgi-bin/target_detail.cgi?targetID=3073731) | 1387 | 54 | hsa-miR-27a-3p | [PIGA](http://www.ncbi.nlm.nih.gov/entrez/query.fcgi?db=gene&cmd=Retrieve&dopt=full_report&list_uids=5277) | phosphatidylinositol glycan anchor biosynthesis class A |
| [Details](http://mirdb.org/cgi-bin/target_detail.cgi?targetID=3073807) | 1388 | 54 | hsa-miR-27a-3p | [CLDN3](http://www.ncbi.nlm.nih.gov/entrez/query.fcgi?db=gene&cmd=Retrieve&dopt=full_report&list_uids=1365) | claudin 3 |
| [Details](http://mirdb.org/cgi-bin/target_detail.cgi?targetID=3073821) | 1389 | 54 | hsa-miR-27a-3p | [ITIH6](http://www.ncbi.nlm.nih.gov/entrez/query.fcgi?db=gene&cmd=Retrieve&dopt=full_report&list_uids=347365) | inter-alpha-trypsin inhibitor heavy chain family member 6 |
| [Details](http://mirdb.org/cgi-bin/target_detail.cgi?targetID=3073853) | 1390 | 54 | hsa-miR-27a-3p | [DDX58](http://www.ncbi.nlm.nih.gov/entrez/query.fcgi?db=gene&cmd=Retrieve&dopt=full_report&list_uids=23586) | DExD/H-box helicase 58 |
| [Details](http://mirdb.org/cgi-bin/target_detail.cgi?targetID=3073882) | 1391 | 54 | hsa-miR-27a-3p | [FRMD3](http://www.ncbi.nlm.nih.gov/entrez/query.fcgi?db=gene&cmd=Retrieve&dopt=full_report&list_uids=257019) | FERM domain containing 3 |
| [Details](http://mirdb.org/cgi-bin/target_detail.cgi?targetID=3073884) | 1392 | 54 | hsa-miR-27a-3p | [DEFB134](http://www.ncbi.nlm.nih.gov/entrez/query.fcgi?db=gene&cmd=Retrieve&dopt=full_report&list_uids=613211) | defensin beta 134 |
| [Details](http://mirdb.org/cgi-bin/target_detail.cgi?targetID=3072556) | 1393 | 53 | hsa-miR-27a-3p | [GPATCH2L](http://www.ncbi.nlm.nih.gov/entrez/query.fcgi?db=gene&cmd=Retrieve&dopt=full_report&list_uids=55668) | G-patch domain containing 2 like |
| [Details](http://mirdb.org/cgi-bin/target_detail.cgi?targetID=3072629) | 1394 | 53 | hsa-miR-27a-3p | [SALL3](http://www.ncbi.nlm.nih.gov/entrez/query.fcgi?db=gene&cmd=Retrieve&dopt=full_report&list_uids=27164) | spalt like transcription factor 3 |
| [Details](http://mirdb.org/cgi-bin/target_detail.cgi?targetID=3072631) | 1395 | 53 | hsa-miR-27a-3p | [MNDA](http://www.ncbi.nlm.nih.gov/entrez/query.fcgi?db=gene&cmd=Retrieve&dopt=full_report&list_uids=4332) | myeloid cell nuclear differentiation antigen |
| [Details](http://mirdb.org/cgi-bin/target_detail.cgi?targetID=3072636) | 1396 | 53 | hsa-miR-27a-3p | [LSM11](http://www.ncbi.nlm.nih.gov/entrez/query.fcgi?db=gene&cmd=Retrieve&dopt=full_report&list_uids=134353) | LSM11, U7 small nuclear RNA associated |
| [Details](http://mirdb.org/cgi-bin/target_detail.cgi?targetID=3072815) | 1397 | 53 | hsa-miR-27a-3p | [MRTFB](http://www.ncbi.nlm.nih.gov/entrez/query.fcgi?db=gene&cmd=Retrieve&dopt=full_report&list_uids=57496) | myocardin related transcription factor B |
| [Details](http://mirdb.org/cgi-bin/target_detail.cgi?targetID=3072863) | 1398 | 53 | hsa-miR-27a-3p | [SAMD10](http://www.ncbi.nlm.nih.gov/entrez/query.fcgi?db=gene&cmd=Retrieve&dopt=full_report&list_uids=140700) | sterile alpha motif domain containing 10 |
| [Details](http://mirdb.org/cgi-bin/target_detail.cgi?targetID=3072881) | 1399 | 53 | hsa-miR-27a-3p | [SMIM10L1](http://www.ncbi.nlm.nih.gov/entrez/query.fcgi?db=gene&cmd=Retrieve&dopt=full_report&list_uids=100129361) | small integral membrane protein 10 like 1 |
| [Details](http://mirdb.org/cgi-bin/target_detail.cgi?targetID=3072968) | 1400 | 53 | hsa-miR-27a-3p | [HOOK1](http://www.ncbi.nlm.nih.gov/entrez/query.fcgi?db=gene&cmd=Retrieve&dopt=full_report&list_uids=51361) | hook microtubule tethering protein 1 |
| [Details](http://mirdb.org/cgi-bin/target_detail.cgi?targetID=3073000) | 1401 | 53 | hsa-miR-27a-3p | [EPS15](http://www.ncbi.nlm.nih.gov/entrez/query.fcgi?db=gene&cmd=Retrieve&dopt=full_report&list_uids=2060) | epidermal growth factor receptor pathway substrate 15 |
| [Details](http://mirdb.org/cgi-bin/target_detail.cgi?targetID=3073183) | 1402 | 53 | hsa-miR-27a-3p | [ZFP82](http://www.ncbi.nlm.nih.gov/entrez/query.fcgi?db=gene&cmd=Retrieve&dopt=full_report&list_uids=284406) | ZFP82 zinc finger protein |
| [Details](http://mirdb.org/cgi-bin/target_detail.cgi?targetID=3073236) | 1403 | 53 | hsa-miR-27a-3p | [HLA-DOA](http://www.ncbi.nlm.nih.gov/entrez/query.fcgi?db=gene&cmd=Retrieve&dopt=full_report&list_uids=3111) | major histocompatibility complex, class II, DO alpha |
| [Details](http://mirdb.org/cgi-bin/target_detail.cgi?targetID=3073335) | 1404 | 53 | hsa-miR-27a-3p | [RASSF8](http://www.ncbi.nlm.nih.gov/entrez/query.fcgi?db=gene&cmd=Retrieve&dopt=full_report&list_uids=11228) | Ras association domain family member 8 |
| [Details](http://mirdb.org/cgi-bin/target_detail.cgi?targetID=3073399) | 1405 | 53 | hsa-miR-27a-3p | [C9orf72](http://www.ncbi.nlm.nih.gov/entrez/query.fcgi?db=gene&cmd=Retrieve&dopt=full_report&list_uids=203228) | chromosome 9 open reading frame 72 |
| [Details](http://mirdb.org/cgi-bin/target_detail.cgi?targetID=3073448) | 1406 | 53 | hsa-miR-27a-3p | [KIAA0895](http://www.ncbi.nlm.nih.gov/entrez/query.fcgi?db=gene&cmd=Retrieve&dopt=full_report&list_uids=23366) | KIAA0895 |
| [Details](http://mirdb.org/cgi-bin/target_detail.cgi?targetID=3073452) | 1407 | 53 | hsa-miR-27a-3p | [WNK2](http://www.ncbi.nlm.nih.gov/entrez/query.fcgi?db=gene&cmd=Retrieve&dopt=full_report&list_uids=65268) | WNK lysine deficient protein kinase 2 |
| [Details](http://mirdb.org/cgi-bin/target_detail.cgi?targetID=3073458) | 1408 | 53 | hsa-miR-27a-3p | [NTRK2](http://www.ncbi.nlm.nih.gov/entrez/query.fcgi?db=gene&cmd=Retrieve&dopt=full_report&list_uids=4915) | neurotrophic receptor tyrosine kinase 2 |
| [Details](http://mirdb.org/cgi-bin/target_detail.cgi?targetID=3073460) | 1409 | 53 | hsa-miR-27a-3p | [CD302](http://www.ncbi.nlm.nih.gov/entrez/query.fcgi?db=gene&cmd=Retrieve&dopt=full_report&list_uids=9936) | CD302 molecule |
| [Details](http://mirdb.org/cgi-bin/target_detail.cgi?targetID=3073603) | 1410 | 53 | hsa-miR-27a-3p | [GRK6](http://www.ncbi.nlm.nih.gov/entrez/query.fcgi?db=gene&cmd=Retrieve&dopt=full_report&list_uids=2870) | G protein-coupled receptor kinase 6 |
| [Details](http://mirdb.org/cgi-bin/target_detail.cgi?targetID=3073604) | 1411 | 53 | hsa-miR-27a-3p | [MVB12B](http://www.ncbi.nlm.nih.gov/entrez/query.fcgi?db=gene&cmd=Retrieve&dopt=full_report&list_uids=89853) | multivesicular body subunit 12B |
| [Details](http://mirdb.org/cgi-bin/target_detail.cgi?targetID=3073606) | 1412 | 53 | hsa-miR-27a-3p | [UBA5](http://www.ncbi.nlm.nih.gov/entrez/query.fcgi?db=gene&cmd=Retrieve&dopt=full_report&list_uids=79876) | ubiquitin like modifier activating enzyme 5 |
| [Details](http://mirdb.org/cgi-bin/target_detail.cgi?targetID=3073642) | 1413 | 53 | hsa-miR-27a-3p | [TTC39C](http://www.ncbi.nlm.nih.gov/entrez/query.fcgi?db=gene&cmd=Retrieve&dopt=full_report&list_uids=125488) | tetratricopeptide repeat domain 39C |
| [Details](http://mirdb.org/cgi-bin/target_detail.cgi?targetID=3073714) | 1414 | 53 | hsa-miR-27a-3p | [MTX3](http://www.ncbi.nlm.nih.gov/entrez/query.fcgi?db=gene&cmd=Retrieve&dopt=full_report&list_uids=345778) | metaxin 3 |
| [Details](http://mirdb.org/cgi-bin/target_detail.cgi?targetID=3073725) | 1415 | 53 | hsa-miR-27a-3p | [UNC80](http://www.ncbi.nlm.nih.gov/entrez/query.fcgi?db=gene&cmd=Retrieve&dopt=full_report&list_uids=285175) | unc-80 homolog, NALCN channel complex subunit |
| [Details](http://mirdb.org/cgi-bin/target_detail.cgi?targetID=3073823) | 1416 | 53 | hsa-miR-27a-3p | [STIM2](http://www.ncbi.nlm.nih.gov/entrez/query.fcgi?db=gene&cmd=Retrieve&dopt=full_report&list_uids=57620) | stromal interaction molecule 2 |
| [Details](http://mirdb.org/cgi-bin/target_detail.cgi?targetID=3073841) | 1417 | 53 | hsa-miR-27a-3p | [LY75-CD302](http://www.ncbi.nlm.nih.gov/entrez/query.fcgi?db=gene&cmd=Retrieve&dopt=full_report&list_uids=100526664) | LY75-CD302 readthrough |
| [Details](http://mirdb.org/cgi-bin/target_detail.cgi?targetID=3073873) | 1418 | 53 | hsa-miR-27a-3p | [KCTD21](http://www.ncbi.nlm.nih.gov/entrez/query.fcgi?db=gene&cmd=Retrieve&dopt=full_report&list_uids=283219) | potassium channel tetramerization domain containing 21 |
| [Details](http://mirdb.org/cgi-bin/target_detail.cgi?targetID=3072443) | 1419 | 52 | hsa-miR-27a-3p | [MAP3K9](http://www.ncbi.nlm.nih.gov/entrez/query.fcgi?db=gene&cmd=Retrieve&dopt=full_report&list_uids=4293) | mitogen-activated protein kinase kinase kinase 9 |
| [Details](http://mirdb.org/cgi-bin/target_detail.cgi?targetID=3072474) | 1420 | 52 | hsa-miR-27a-3p | [MRS2](http://www.ncbi.nlm.nih.gov/entrez/query.fcgi?db=gene&cmd=Retrieve&dopt=full_report&list_uids=57380) | magnesium transporter MRS2 |
| [Details](http://mirdb.org/cgi-bin/target_detail.cgi?targetID=3072506) | 1421 | 52 | hsa-miR-27a-3p | [SEC22C](http://www.ncbi.nlm.nih.gov/entrez/query.fcgi?db=gene&cmd=Retrieve&dopt=full_report&list_uids=9117) | SEC22 homolog C, vesicle trafficking protein |
| [Details](http://mirdb.org/cgi-bin/target_detail.cgi?targetID=3072545) | 1422 | 52 | hsa-miR-27a-3p | [DNAJC5B](http://www.ncbi.nlm.nih.gov/entrez/query.fcgi?db=gene&cmd=Retrieve&dopt=full_report&list_uids=85479) | DnaJ heat shock protein family (Hsp40) member C5 beta |
| [Details](http://mirdb.org/cgi-bin/target_detail.cgi?targetID=3072546) | 1423 | 52 | hsa-miR-27a-3p | [C1QL4](http://www.ncbi.nlm.nih.gov/entrez/query.fcgi?db=gene&cmd=Retrieve&dopt=full_report&list_uids=338761) | complement C1q like 4 |
| [Details](http://mirdb.org/cgi-bin/target_detail.cgi?targetID=3072577) | 1424 | 52 | hsa-miR-27a-3p | [CCDC82](http://www.ncbi.nlm.nih.gov/entrez/query.fcgi?db=gene&cmd=Retrieve&dopt=full_report&list_uids=79780) | coiled-coil domain containing 82 |
| [Details](http://mirdb.org/cgi-bin/target_detail.cgi?targetID=3072584) | 1425 | 52 | hsa-miR-27a-3p | [PTAFR](http://www.ncbi.nlm.nih.gov/entrez/query.fcgi?db=gene&cmd=Retrieve&dopt=full_report&list_uids=5724) | platelet activating factor receptor |
| [Details](http://mirdb.org/cgi-bin/target_detail.cgi?targetID=3072649) | 1426 | 52 | hsa-miR-27a-3p | [ELMOD1](http://www.ncbi.nlm.nih.gov/entrez/query.fcgi?db=gene&cmd=Retrieve&dopt=full_report&list_uids=55531) | ELMO domain containing 1 |
| [Details](http://mirdb.org/cgi-bin/target_detail.cgi?targetID=3072673) | 1427 | 52 | hsa-miR-27a-3p | [CASP2](http://www.ncbi.nlm.nih.gov/entrez/query.fcgi?db=gene&cmd=Retrieve&dopt=full_report&list_uids=835) | caspase 2 |
| [Details](http://mirdb.org/cgi-bin/target_detail.cgi?targetID=3072781) | 1428 | 52 | hsa-miR-27a-3p | [VGF](http://www.ncbi.nlm.nih.gov/entrez/query.fcgi?db=gene&cmd=Retrieve&dopt=full_report&list_uids=7425) | VGF nerve growth factor inducible |
| [Details](http://mirdb.org/cgi-bin/target_detail.cgi?targetID=3072916) | 1429 | 52 | hsa-miR-27a-3p | [MAPK8IP3](http://www.ncbi.nlm.nih.gov/entrez/query.fcgi?db=gene&cmd=Retrieve&dopt=full_report&list_uids=23162) | mitogen-activated protein kinase 8 interacting protein 3 |
| [Details](http://mirdb.org/cgi-bin/target_detail.cgi?targetID=3073108) | 1430 | 52 | hsa-miR-27a-3p | [ZNF577](http://www.ncbi.nlm.nih.gov/entrez/query.fcgi?db=gene&cmd=Retrieve&dopt=full_report&list_uids=84765) | zinc finger protein 577 |
| [Details](http://mirdb.org/cgi-bin/target_detail.cgi?targetID=3073138) | 1431 | 52 | hsa-miR-27a-3p | [HYDIN](http://www.ncbi.nlm.nih.gov/entrez/query.fcgi?db=gene&cmd=Retrieve&dopt=full_report&list_uids=54768) | HYDIN, axonemal central pair apparatus protein |
| [Details](http://mirdb.org/cgi-bin/target_detail.cgi?targetID=3073266) | 1432 | 52 | hsa-miR-27a-3p | [PDE1A](http://www.ncbi.nlm.nih.gov/entrez/query.fcgi?db=gene&cmd=Retrieve&dopt=full_report&list_uids=5136) | phosphodiesterase 1A |
| [Details](http://mirdb.org/cgi-bin/target_detail.cgi?targetID=3073351) | 1433 | 52 | hsa-miR-27a-3p | [SV2B](http://www.ncbi.nlm.nih.gov/entrez/query.fcgi?db=gene&cmd=Retrieve&dopt=full_report&list_uids=9899) | synaptic vesicle glycoprotein 2B |
| [Details](http://mirdb.org/cgi-bin/target_detail.cgi?targetID=3073372) | 1434 | 52 | hsa-miR-27a-3p | [CADM1](http://www.ncbi.nlm.nih.gov/entrez/query.fcgi?db=gene&cmd=Retrieve&dopt=full_report&list_uids=23705) | cell adhesion molecule 1 |
| [Details](http://mirdb.org/cgi-bin/target_detail.cgi?targetID=3073433) | 1435 | 52 | hsa-miR-27a-3p | [PPARGC1B](http://www.ncbi.nlm.nih.gov/entrez/query.fcgi?db=gene&cmd=Retrieve&dopt=full_report&list_uids=133522) | PPARG coactivator 1 beta |
| [Details](http://mirdb.org/cgi-bin/target_detail.cgi?targetID=3073470) | 1436 | 52 | hsa-miR-27a-3p | [ZNF705D](http://www.ncbi.nlm.nih.gov/entrez/query.fcgi?db=gene&cmd=Retrieve&dopt=full_report&list_uids=728957) | zinc finger protein 705D |
| [Details](http://mirdb.org/cgi-bin/target_detail.cgi?targetID=3073489) | 1437 | 52 | hsa-miR-27a-3p | [ZNF614](http://www.ncbi.nlm.nih.gov/entrez/query.fcgi?db=gene&cmd=Retrieve&dopt=full_report&list_uids=80110) | zinc finger protein 614 |
| [Details](http://mirdb.org/cgi-bin/target_detail.cgi?targetID=3073500) | 1438 | 52 | hsa-miR-27a-3p | [SNX18](http://www.ncbi.nlm.nih.gov/entrez/query.fcgi?db=gene&cmd=Retrieve&dopt=full_report&list_uids=112574) | sorting nexin 18 |
| [Details](http://mirdb.org/cgi-bin/target_detail.cgi?targetID=3073525) | 1439 | 52 | hsa-miR-27a-3p | [GPR180](http://www.ncbi.nlm.nih.gov/entrez/query.fcgi?db=gene&cmd=Retrieve&dopt=full_report&list_uids=160897) | G protein-coupled receptor 180 |
| [Details](http://mirdb.org/cgi-bin/target_detail.cgi?targetID=3073636) | 1440 | 52 | hsa-miR-27a-3p | [PELI1](http://www.ncbi.nlm.nih.gov/entrez/query.fcgi?db=gene&cmd=Retrieve&dopt=full_report&list_uids=57162) | pellino E3 ubiquitin protein ligase 1 |
| [Details](http://mirdb.org/cgi-bin/target_detail.cgi?targetID=3073639) | 1441 | 52 | hsa-miR-27a-3p | [KLF12](http://www.ncbi.nlm.nih.gov/entrez/query.fcgi?db=gene&cmd=Retrieve&dopt=full_report&list_uids=11278) | Kruppel like factor 12 |
| [Details](http://mirdb.org/cgi-bin/target_detail.cgi?targetID=3073700) | 1442 | 52 | hsa-miR-27a-3p | [RIMS4](http://www.ncbi.nlm.nih.gov/entrez/query.fcgi?db=gene&cmd=Retrieve&dopt=full_report&list_uids=140730) | regulating synaptic membrane exocytosis 4 |
| [Details](http://mirdb.org/cgi-bin/target_detail.cgi?targetID=3073701) | 1443 | 52 | hsa-miR-27a-3p | [TMEM199](http://www.ncbi.nlm.nih.gov/entrez/query.fcgi?db=gene&cmd=Retrieve&dopt=full_report&list_uids=147007) | transmembrane protein 199 |
| [Details](http://mirdb.org/cgi-bin/target_detail.cgi?targetID=3073753) | 1444 | 52 | hsa-miR-27a-3p | [MMS22L](http://www.ncbi.nlm.nih.gov/entrez/query.fcgi?db=gene&cmd=Retrieve&dopt=full_report&list_uids=253714) | MMS22 like, DNA repair protein |
| [Details](http://mirdb.org/cgi-bin/target_detail.cgi?targetID=3073773) | 1445 | 52 | hsa-miR-27a-3p | [AGBL3](http://www.ncbi.nlm.nih.gov/entrez/query.fcgi?db=gene&cmd=Retrieve&dopt=full_report&list_uids=340351) | ATP/GTP binding protein like 3 |
| [Details](http://mirdb.org/cgi-bin/target_detail.cgi?targetID=3073850) | 1446 | 52 | hsa-miR-27a-3p | [CCBE1](http://www.ncbi.nlm.nih.gov/entrez/query.fcgi?db=gene&cmd=Retrieve&dopt=full_report&list_uids=147372) | collagen and calcium binding EGF domains 1 |
| [Details](http://mirdb.org/cgi-bin/target_detail.cgi?targetID=3073883) | 1447 | 52 | hsa-miR-27a-3p | [NAT8L](http://www.ncbi.nlm.nih.gov/entrez/query.fcgi?db=gene&cmd=Retrieve&dopt=full_report&list_uids=339983) | N-acetyltransferase 8 like |
| [Details](http://mirdb.org/cgi-bin/target_detail.cgi?targetID=3073900) | 1448 | 52 | hsa-miR-27a-3p | [ODF2](http://www.ncbi.nlm.nih.gov/entrez/query.fcgi?db=gene&cmd=Retrieve&dopt=full_report&list_uids=4957) | outer dense fiber of sperm tails 2 |
| [Details](http://mirdb.org/cgi-bin/target_detail.cgi?targetID=3073910) | 1449 | 52 | hsa-miR-27a-3p | [ADAM22](http://www.ncbi.nlm.nih.gov/entrez/query.fcgi?db=gene&cmd=Retrieve&dopt=full_report&list_uids=53616) | ADAM metallopeptidase domain 22 |
| [Details](http://mirdb.org/cgi-bin/target_detail.cgi?targetID=3072434) | 1450 | 51 | hsa-miR-27a-3p | [RAD1](http://www.ncbi.nlm.nih.gov/entrez/query.fcgi?db=gene&cmd=Retrieve&dopt=full_report&list_uids=5810) | RAD1 checkpoint DNA exonuclease |
| [Details](http://mirdb.org/cgi-bin/target_detail.cgi?targetID=3072439) | 1451 | 51 | hsa-miR-27a-3p | [C17orf51](http://www.ncbi.nlm.nih.gov/entrez/query.fcgi?db=gene&cmd=Retrieve&dopt=full_report&list_uids=339263) | chromosome 17 open reading frame 51 |
| [Details](http://mirdb.org/cgi-bin/target_detail.cgi?targetID=3072440) | 1452 | 51 | hsa-miR-27a-3p | [VASH2](http://www.ncbi.nlm.nih.gov/entrez/query.fcgi?db=gene&cmd=Retrieve&dopt=full_report&list_uids=79805) | vasohibin 2 |
| [Details](http://mirdb.org/cgi-bin/target_detail.cgi?targetID=3072560) | 1453 | 51 | hsa-miR-27a-3p | [ATXN3](http://www.ncbi.nlm.nih.gov/entrez/query.fcgi?db=gene&cmd=Retrieve&dopt=full_report&list_uids=4287) | ataxin 3 |
| [Details](http://mirdb.org/cgi-bin/target_detail.cgi?targetID=3072663) | 1454 | 51 | hsa-miR-27a-3p | [ZNF502](http://www.ncbi.nlm.nih.gov/entrez/query.fcgi?db=gene&cmd=Retrieve&dopt=full_report&list_uids=91392) | zinc finger protein 502 |
| [Details](http://mirdb.org/cgi-bin/target_detail.cgi?targetID=3072699) | 1455 | 51 | hsa-miR-27a-3p | [HMGA2-AS1](http://www.ncbi.nlm.nih.gov/entrez/query.fcgi?db=gene&cmd=Retrieve&dopt=full_report&list_uids=100129940) | HMGA2 antisense RNA 1 |
| [Details](http://mirdb.org/cgi-bin/target_detail.cgi?targetID=3072722) | 1456 | 51 | hsa-miR-27a-3p | [MOSPD3](http://www.ncbi.nlm.nih.gov/entrez/query.fcgi?db=gene&cmd=Retrieve&dopt=full_report&list_uids=64598) | motile sperm domain containing 3 |
| [Details](http://mirdb.org/cgi-bin/target_detail.cgi?targetID=3072769) | 1457 | 51 | hsa-miR-27a-3p | [TUSC3](http://www.ncbi.nlm.nih.gov/entrez/query.fcgi?db=gene&cmd=Retrieve&dopt=full_report&list_uids=7991) | tumor suppressor candidate 3 |
| [Details](http://mirdb.org/cgi-bin/target_detail.cgi?targetID=3072842) | 1458 | 51 | hsa-miR-27a-3p | [MARCH1](http://www.ncbi.nlm.nih.gov/entrez/query.fcgi?db=gene&cmd=Retrieve&dopt=full_report&list_uids=55016) | membrane associated ring-CH-type finger 1 |
| [Details](http://mirdb.org/cgi-bin/target_detail.cgi?targetID=3072845) | 1459 | 51 | hsa-miR-27a-3p | [ZNF286A](http://www.ncbi.nlm.nih.gov/entrez/query.fcgi?db=gene&cmd=Retrieve&dopt=full_report&list_uids=57335) | zinc finger protein 286A |
| [Details](http://mirdb.org/cgi-bin/target_detail.cgi?targetID=3072892) | 1460 | 51 | hsa-miR-27a-3p | [ITGB8](http://www.ncbi.nlm.nih.gov/entrez/query.fcgi?db=gene&cmd=Retrieve&dopt=full_report&list_uids=3696) | integrin subunit beta 8 |
| [Details](http://mirdb.org/cgi-bin/target_detail.cgi?targetID=3072899) | 1461 | 51 | hsa-miR-27a-3p | [GPATCH11](http://www.ncbi.nlm.nih.gov/entrez/query.fcgi?db=gene&cmd=Retrieve&dopt=full_report&list_uids=253635) | G-patch domain containing 11 |
| [Details](http://mirdb.org/cgi-bin/target_detail.cgi?targetID=3072926) | 1462 | 51 | hsa-miR-27a-3p | [ULBP3](http://www.ncbi.nlm.nih.gov/entrez/query.fcgi?db=gene&cmd=Retrieve&dopt=full_report&list_uids=79465) | UL16 binding protein 3 |
| [Details](http://mirdb.org/cgi-bin/target_detail.cgi?targetID=3072974) | 1463 | 51 | hsa-miR-27a-3p | [SPICE1](http://www.ncbi.nlm.nih.gov/entrez/query.fcgi?db=gene&cmd=Retrieve&dopt=full_report&list_uids=152185) | spindle and centriole associated protein 1 |
| [Details](http://mirdb.org/cgi-bin/target_detail.cgi?targetID=3073027) | 1464 | 51 | hsa-miR-27a-3p | [SHISAL1](http://www.ncbi.nlm.nih.gov/entrez/query.fcgi?db=gene&cmd=Retrieve&dopt=full_report&list_uids=85352) | shisa like 1 |
| [Details](http://mirdb.org/cgi-bin/target_detail.cgi?targetID=3073258) | 1465 | 51 | hsa-miR-27a-3p | [LAX1](http://www.ncbi.nlm.nih.gov/entrez/query.fcgi?db=gene&cmd=Retrieve&dopt=full_report&list_uids=54900) | lymphocyte transmembrane adaptor 1 |
| [Details](http://mirdb.org/cgi-bin/target_detail.cgi?targetID=3073305) | 1466 | 51 | hsa-miR-27a-3p | [GZF1](http://www.ncbi.nlm.nih.gov/entrez/query.fcgi?db=gene&cmd=Retrieve&dopt=full_report&list_uids=64412) | GDNF inducible zinc finger protein 1 |
| [Details](http://mirdb.org/cgi-bin/target_detail.cgi?targetID=3073383) | 1467 | 51 | hsa-miR-27a-3p | [KCNA6](http://www.ncbi.nlm.nih.gov/entrez/query.fcgi?db=gene&cmd=Retrieve&dopt=full_report&list_uids=3742) | potassium voltage-gated channel subfamily A member 6 |
| [Details](http://mirdb.org/cgi-bin/target_detail.cgi?targetID=3073553) | 1468 | 51 | hsa-miR-27a-3p | [SSTR1](http://www.ncbi.nlm.nih.gov/entrez/query.fcgi?db=gene&cmd=Retrieve&dopt=full_report&list_uids=6751) | somatostatin receptor 1 |
| [Details](http://mirdb.org/cgi-bin/target_detail.cgi?targetID=3073587) | 1469 | 51 | hsa-miR-27a-3p | [COA7](http://www.ncbi.nlm.nih.gov/entrez/query.fcgi?db=gene&cmd=Retrieve&dopt=full_report&list_uids=65260) | cytochrome c oxidase assembly factor 7 (putative) |
| [Details](http://mirdb.org/cgi-bin/target_detail.cgi?targetID=3073611) | 1470 | 51 | hsa-miR-27a-3p | [ZNF579](http://www.ncbi.nlm.nih.gov/entrez/query.fcgi?db=gene&cmd=Retrieve&dopt=full_report&list_uids=163033) | zinc finger protein 579 |
| [Details](http://mirdb.org/cgi-bin/target_detail.cgi?targetID=3073632) | 1471 | 51 | hsa-miR-27a-3p | [ATP6V1G2](http://www.ncbi.nlm.nih.gov/entrez/query.fcgi?db=gene&cmd=Retrieve&dopt=full_report&list_uids=534) | ATPase H+ transporting V1 subunit G2 |
| [Details](http://mirdb.org/cgi-bin/target_detail.cgi?targetID=3073688) | 1472 | 51 | hsa-miR-27a-3p | [TATDN3](http://www.ncbi.nlm.nih.gov/entrez/query.fcgi?db=gene&cmd=Retrieve&dopt=full_report&list_uids=128387) | TatD DNase domain containing 3 |
| [Details](http://mirdb.org/cgi-bin/target_detail.cgi?targetID=3073691) | 1473 | 51 | hsa-miR-27a-3p | [PLEKHA2](http://www.ncbi.nlm.nih.gov/entrez/query.fcgi?db=gene&cmd=Retrieve&dopt=full_report&list_uids=59339) | pleckstrin homology domain containing A2 |
| [Details](http://mirdb.org/cgi-bin/target_detail.cgi?targetID=3073780) | 1474 | 51 | hsa-miR-27a-3p | [PAPSS2](http://www.ncbi.nlm.nih.gov/entrez/query.fcgi?db=gene&cmd=Retrieve&dopt=full_report&list_uids=9060) | 3'-phosphoadenosine 5'-phosphosulfate synthase 2 |
| [Details](http://mirdb.org/cgi-bin/target_detail.cgi?targetID=3073822) | 1475 | 51 | hsa-miR-27a-3p | [RAPGEF6](http://www.ncbi.nlm.nih.gov/entrez/query.fcgi?db=gene&cmd=Retrieve&dopt=full_report&list_uids=51735) | Rap guanine nucleotide exchange factor 6 |
| [Details](http://mirdb.org/cgi-bin/target_detail.cgi?targetID=3073833) | 1476 | 51 | hsa-miR-27a-3p | [CMTM4](http://www.ncbi.nlm.nih.gov/entrez/query.fcgi?db=gene&cmd=Retrieve&dopt=full_report&list_uids=146223) | CKLF like MARVEL transmembrane domain containing 4 |
| [Details](http://mirdb.org/cgi-bin/target_detail.cgi?targetID=3072505) | 1477 | 50 | hsa-miR-27a-3p | [DDX60L](http://www.ncbi.nlm.nih.gov/entrez/query.fcgi?db=gene&cmd=Retrieve&dopt=full_report&list_uids=91351) | DExD/H-box 60 like |
| [Details](http://mirdb.org/cgi-bin/target_detail.cgi?targetID=3072706) | 1478 | 50 | hsa-miR-27a-3p | [CRB1](http://www.ncbi.nlm.nih.gov/entrez/query.fcgi?db=gene&cmd=Retrieve&dopt=full_report&list_uids=23418) | crumbs cell polarity complex component 1 |
| [Details](http://mirdb.org/cgi-bin/target_detail.cgi?targetID=3072800) | 1479 | 50 | hsa-miR-27a-3p | [LY6G5B](http://www.ncbi.nlm.nih.gov/entrez/query.fcgi?db=gene&cmd=Retrieve&dopt=full_report&list_uids=58496) | lymphocyte antigen 6 family member G5B |
| [Details](http://mirdb.org/cgi-bin/target_detail.cgi?targetID=3072878) | 1480 | 50 | hsa-miR-27a-3p | [STMN1](http://www.ncbi.nlm.nih.gov/entrez/query.fcgi?db=gene&cmd=Retrieve&dopt=full_report&list_uids=3925) | stathmin 1 |
| [Details](http://mirdb.org/cgi-bin/target_detail.cgi?targetID=3072987) | 1481 | 50 | hsa-miR-27a-3p | [ZEB1](http://www.ncbi.nlm.nih.gov/entrez/query.fcgi?db=gene&cmd=Retrieve&dopt=full_report&list_uids=6935) | zinc finger E-box binding homeobox 1 |
| [Details](http://mirdb.org/cgi-bin/target_detail.cgi?targetID=3073085) | 1482 | 50 | hsa-miR-27a-3p | [SARM1](http://www.ncbi.nlm.nih.gov/entrez/query.fcgi?db=gene&cmd=Retrieve&dopt=full_report&list_uids=23098) | sterile alpha and TIR motif containing 1 |
| [Details](http://mirdb.org/cgi-bin/target_detail.cgi?targetID=3073141) | 1483 | 50 | hsa-miR-27a-3p | [PDE3A](http://www.ncbi.nlm.nih.gov/entrez/query.fcgi?db=gene&cmd=Retrieve&dopt=full_report&list_uids=5139) | phosphodiesterase 3A |
| [Details](http://mirdb.org/cgi-bin/target_detail.cgi?targetID=3073216) | 1484 | 50 | hsa-miR-27a-3p | [LMOD1](http://www.ncbi.nlm.nih.gov/entrez/query.fcgi?db=gene&cmd=Retrieve&dopt=full_report&list_uids=25802) | leiomodin 1 |
| [Details](http://mirdb.org/cgi-bin/target_detail.cgi?targetID=3073288) | 1485 | 50 | hsa-miR-27a-3p | [CA7](http://www.ncbi.nlm.nih.gov/entrez/query.fcgi?db=gene&cmd=Retrieve&dopt=full_report&list_uids=766) | carbonic anhydrase 7 |
| [Details](http://mirdb.org/cgi-bin/target_detail.cgi?targetID=3073347) | 1486 | 50 | hsa-miR-27a-3p | [JPH1](http://www.ncbi.nlm.nih.gov/entrez/query.fcgi?db=gene&cmd=Retrieve&dopt=full_report&list_uids=56704) | junctophilin 1 |
| [Details](http://mirdb.org/cgi-bin/target_detail.cgi?targetID=3073350) | 1487 | 50 | hsa-miR-27a-3p | [ASB3](http://www.ncbi.nlm.nih.gov/entrez/query.fcgi?db=gene&cmd=Retrieve&dopt=full_report&list_uids=51130) | ankyrin repeat and SOCS box containing 3 |
| [Details](http://mirdb.org/cgi-bin/target_detail.cgi?targetID=3073442) | 1488 | 50 | hsa-miR-27a-3p | [CSDC2](http://www.ncbi.nlm.nih.gov/entrez/query.fcgi?db=gene&cmd=Retrieve&dopt=full_report&list_uids=27254) | cold shock domain containing C2 |
| [Details](http://mirdb.org/cgi-bin/target_detail.cgi?targetID=3073469) | 1489 | 50 | hsa-miR-27a-3p | [TNNT3](http://www.ncbi.nlm.nih.gov/entrez/query.fcgi?db=gene&cmd=Retrieve&dopt=full_report&list_uids=7140) | troponin T3, fast skeletal type |
| [Details](http://mirdb.org/cgi-bin/target_detail.cgi?targetID=3073502) | 1490 | 50 | hsa-miR-27a-3p | [ITPKC](http://www.ncbi.nlm.nih.gov/entrez/query.fcgi?db=gene&cmd=Retrieve&dopt=full_report&list_uids=80271) | inositol-trisphosphate 3-kinase C |
| [Details](http://mirdb.org/cgi-bin/target_detail.cgi?targetID=3073523) | 1491 | 50 | hsa-miR-27a-3p | [SCAMP3](http://www.ncbi.nlm.nih.gov/entrez/query.fcgi?db=gene&cmd=Retrieve&dopt=full_report&list_uids=10067) | secretory carrier membrane protein 3 |
| [Details](http://mirdb.org/cgi-bin/target_detail.cgi?targetID=3073554) | 1492 | 50 | hsa-miR-27a-3p | [ZFP90](http://www.ncbi.nlm.nih.gov/entrez/query.fcgi?db=gene&cmd=Retrieve&dopt=full_report&list_uids=146198) | ZFP90 zinc finger protein |
| [Details](http://mirdb.org/cgi-bin/target_detail.cgi?targetID=3073651) | 1493 | 50 | hsa-miR-27a-3p | [TRPM3](http://www.ncbi.nlm.nih.gov/entrez/query.fcgi?db=gene&cmd=Retrieve&dopt=full_report&list_uids=80036) | transient receptor potential cation channel subfamily M member 3 |
| [Details](http://mirdb.org/cgi-bin/target_detail.cgi?targetID=3073669) | 1494 | 50 | hsa-miR-27a-3p | [COL11A2](http://www.ncbi.nlm.nih.gov/entrez/query.fcgi?db=gene&cmd=Retrieve&dopt=full_report&list_uids=1302) | collagen type XI alpha 2 chain |
| [Details](http://mirdb.org/cgi-bin/target_detail.cgi?targetID=3073806) | 1495 | 50 | hsa-miR-27a-3p | [NPTXR](http://www.ncbi.nlm.nih.gov/entrez/query.fcgi?db=gene&cmd=Retrieve&dopt=full_report&list_uids=23467) | neuronal pentraxin receptor |
| [Details](http://mirdb.org/cgi-bin/target_detail.cgi?targetID=3073858) | 1496 | 50 | hsa-miR-27a-3p | [ASAP1](http://www.ncbi.nlm.nih.gov/entrez/query.fcgi?db=gene&cmd=Retrieve&dopt=full_report&list_uids=50807) | ArfGAP with SH3 domain, ankyrin repeat and PH domain 1 |
| [Details](http://mirdb.org/cgi-bin/target_detail.cgi?targetID=3073906) | 1497 | 50 | hsa-miR-27a-3p | [C16orf82](http://www.ncbi.nlm.nih.gov/entrez/query.fcgi?db=gene&cmd=Retrieve&dopt=full_report&list_uids=162083) | chromosome 16 open reading frame 82 |

Data generated from the online database for prediction of functional microRNA targets (Chen & Wang, 2020)

Chen, Y., & Wang, X. (2020). MiRDB: An online database for prediction of functional microRNA targets. *Nucleic Acids Research*, *48*(D1), D127–D131. https://doi.org/10.1093/nar/gkz757
