# Supplementary material for: The Role of microRNAs in Organismal and Skin Aging
Source: Int J Mol Sci. 2020 Jul 25;21(15):5281. doi: 10.3390/ijms21155281 (PMC7432402; doi:10.3390/ijms21155281)
Supplement: Supplementary file 1 [file ijms-21-05281-s001.zip › Appendix 9 hsa-miR-148a-3p .docx]

**There are 839 predicted targets for hsa-miR-148a-3p in miRDB**

| **Target Detail** | **Target Rank** | **Target Score** | **miRNA Name** | **Gene Symbol** | **Gene Description** |
| --- | --- | --- | --- | --- | --- |
| [Details](http://mirdb.org/cgi-bin/target_detail.cgi?targetID=598420) | 1 | 100 | hsa-miR-148a-3p | [SOS2](http://www.ncbi.nlm.nih.gov/entrez/query.fcgi?db=gene&cmd=Retrieve&dopt=full_report&list_uids=6655) | SOS Ras/Rho guanine nucleotide exchange factor 2 |
| [Details](http://mirdb.org/cgi-bin/target_detail.cgi?targetID=598294) | 2 | 100 | hsa-miR-148a-3p | [CDK19](http://www.ncbi.nlm.nih.gov/entrez/query.fcgi?db=gene&cmd=Retrieve&dopt=full_report&list_uids=23097) | cyclin dependent kinase 19 |
| [Details](http://mirdb.org/cgi-bin/target_detail.cgi?targetID=598328) | 3 | 99 | hsa-miR-148a-3p | [HOMER1](http://www.ncbi.nlm.nih.gov/entrez/query.fcgi?db=gene&cmd=Retrieve&dopt=full_report&list_uids=9456) | homer scaffold protein 1 |
| [Details](http://mirdb.org/cgi-bin/target_detail.cgi?targetID=598597) | 4 | 99 | hsa-miR-148a-3p | [MEOX2](http://www.ncbi.nlm.nih.gov/entrez/query.fcgi?db=gene&cmd=Retrieve&dopt=full_report&list_uids=4223) | mesenchyme homeobox 2 |
| [Details](http://mirdb.org/cgi-bin/target_detail.cgi?targetID=598541) | 5 | 99 | hsa-miR-148a-3p | [BCL2L11](http://www.ncbi.nlm.nih.gov/entrez/query.fcgi?db=gene&cmd=Retrieve&dopt=full_report&list_uids=10018) | BCL2 like 11 |
| [Details](http://mirdb.org/cgi-bin/target_detail.cgi?targetID=598663) | 6 | 99 | hsa-miR-148a-3p | [KMT2A](http://www.ncbi.nlm.nih.gov/entrez/query.fcgi?db=gene&cmd=Retrieve&dopt=full_report&list_uids=4297) | lysine methyltransferase 2A |
| [Details](http://mirdb.org/cgi-bin/target_detail.cgi?targetID=598143) | 7 | 99 | hsa-miR-148a-3p | [RPS6KA5](http://www.ncbi.nlm.nih.gov/entrez/query.fcgi?db=gene&cmd=Retrieve&dopt=full_report&list_uids=9252) | ribosomal protein S6 kinase A5 |
| [Details](http://mirdb.org/cgi-bin/target_detail.cgi?targetID=598617) | 8 | 99 | hsa-miR-148a-3p | [CBLB](http://www.ncbi.nlm.nih.gov/entrez/query.fcgi?db=gene&cmd=Retrieve&dopt=full_report&list_uids=868) | Cbl proto-oncogene B |
| [Details](http://mirdb.org/cgi-bin/target_detail.cgi?targetID=598563) | 9 | 99 | hsa-miR-148a-3p | [UBE2D3](http://www.ncbi.nlm.nih.gov/entrez/query.fcgi?db=gene&cmd=Retrieve&dopt=full_report&list_uids=7323) | ubiquitin conjugating enzyme E2 D3 |
| [Details](http://mirdb.org/cgi-bin/target_detail.cgi?targetID=598348) | 10 | 99 | hsa-miR-148a-3p | [WDR47](http://www.ncbi.nlm.nih.gov/entrez/query.fcgi?db=gene&cmd=Retrieve&dopt=full_report&list_uids=22911) | WD repeat domain 47 |
| [Details](http://mirdb.org/cgi-bin/target_detail.cgi?targetID=598530) | 11 | 99 | hsa-miR-148a-3p | [LDLR](http://www.ncbi.nlm.nih.gov/entrez/query.fcgi?db=gene&cmd=Retrieve&dopt=full_report&list_uids=3949) | low density lipoprotein receptor |
| [Details](http://mirdb.org/cgi-bin/target_detail.cgi?targetID=598565) | 12 | 99 | hsa-miR-148a-3p | [NPTN](http://www.ncbi.nlm.nih.gov/entrez/query.fcgi?db=gene&cmd=Retrieve&dopt=full_report&list_uids=27020) | neuroplastin |
| [Details](http://mirdb.org/cgi-bin/target_detail.cgi?targetID=598543) | 13 | 98 | hsa-miR-148a-3p | [AKAP1](http://www.ncbi.nlm.nih.gov/entrez/query.fcgi?db=gene&cmd=Retrieve&dopt=full_report&list_uids=8165) | A-kinase anchoring protein 1 |
| [Details](http://mirdb.org/cgi-bin/target_detail.cgi?targetID=598556) | 14 | 98 | hsa-miR-148a-3p | [B4GALT6](http://www.ncbi.nlm.nih.gov/entrez/query.fcgi?db=gene&cmd=Retrieve&dopt=full_report&list_uids=9331) | beta-1,4-galactosyltransferase 6 |
| [Details](http://mirdb.org/cgi-bin/target_detail.cgi?targetID=598130) | 15 | 98 | hsa-miR-148a-3p | [FXR1](http://www.ncbi.nlm.nih.gov/entrez/query.fcgi?db=gene&cmd=Retrieve&dopt=full_report&list_uids=8087) | FMR1 autosomal homolog 1 |
| [Details](http://mirdb.org/cgi-bin/target_detail.cgi?targetID=598493) | 16 | 98 | hsa-miR-148a-3p | [SZRD1](http://www.ncbi.nlm.nih.gov/entrez/query.fcgi?db=gene&cmd=Retrieve&dopt=full_report&list_uids=26099) | SUZ RNA binding domain containing 1 |
| [Details](http://mirdb.org/cgi-bin/target_detail.cgi?targetID=598287) | 17 | 98 | hsa-miR-148a-3p | [C5orf30](http://www.ncbi.nlm.nih.gov/entrez/query.fcgi?db=gene&cmd=Retrieve&dopt=full_report&list_uids=90355) | chromosome 5 open reading frame 30 |
| [Details](http://mirdb.org/cgi-bin/target_detail.cgi?targetID=598023) | 18 | 98 | hsa-miR-148a-3p | [DENND4C](http://www.ncbi.nlm.nih.gov/entrez/query.fcgi?db=gene&cmd=Retrieve&dopt=full_report&list_uids=55667) | DENN domain containing 4C |
| [Details](http://mirdb.org/cgi-bin/target_detail.cgi?targetID=598274) | 19 | 98 | hsa-miR-148a-3p | [ZFYVE26](http://www.ncbi.nlm.nih.gov/entrez/query.fcgi?db=gene&cmd=Retrieve&dopt=full_report&list_uids=23503) | zinc finger FYVE-type containing 26 |
| [Details](http://mirdb.org/cgi-bin/target_detail.cgi?targetID=598059) | 20 | 98 | hsa-miR-148a-3p | [ROBO1](http://www.ncbi.nlm.nih.gov/entrez/query.fcgi?db=gene&cmd=Retrieve&dopt=full_report&list_uids=6091) | roundabout guidance receptor 1 |
| [Details](http://mirdb.org/cgi-bin/target_detail.cgi?targetID=598275) | 21 | 98 | hsa-miR-148a-3p | [UHMK1](http://www.ncbi.nlm.nih.gov/entrez/query.fcgi?db=gene&cmd=Retrieve&dopt=full_report&list_uids=127933) | U2AF homology motif kinase 1 |
| [Details](http://mirdb.org/cgi-bin/target_detail.cgi?targetID=597905) | 22 | 97 | hsa-miR-148a-3p | [BTAF1](http://www.ncbi.nlm.nih.gov/entrez/query.fcgi?db=gene&cmd=Retrieve&dopt=full_report&list_uids=9044) | B-TFIID TATA-box binding protein associated factor 1 |
| [Details](http://mirdb.org/cgi-bin/target_detail.cgi?targetID=598423) | 23 | 97 | hsa-miR-148a-3p | [QKI](http://www.ncbi.nlm.nih.gov/entrez/query.fcgi?db=gene&cmd=Retrieve&dopt=full_report&list_uids=9444) | QKI, KH domain containing RNA binding |
| [Details](http://mirdb.org/cgi-bin/target_detail.cgi?targetID=598494) | 24 | 97 | hsa-miR-148a-3p | [KAT7](http://www.ncbi.nlm.nih.gov/entrez/query.fcgi?db=gene&cmd=Retrieve&dopt=full_report&list_uids=11143) | lysine acetyltransferase 7 |
| [Details](http://mirdb.org/cgi-bin/target_detail.cgi?targetID=598093) | 25 | 97 | hsa-miR-148a-3p | [RMND5A](http://www.ncbi.nlm.nih.gov/entrez/query.fcgi?db=gene&cmd=Retrieve&dopt=full_report&list_uids=64795) | required for meiotic nuclear division 5 homolog A |
| [Details](http://mirdb.org/cgi-bin/target_detail.cgi?targetID=598006) | 26 | 97 | hsa-miR-148a-3p | [JMY](http://www.ncbi.nlm.nih.gov/entrez/query.fcgi?db=gene&cmd=Retrieve&dopt=full_report&list_uids=133746) | junction mediating and regulatory protein, p53 cofactor |
| [Details](http://mirdb.org/cgi-bin/target_detail.cgi?targetID=597971) | 27 | 97 | hsa-miR-148a-3p | [BICC1](http://www.ncbi.nlm.nih.gov/entrez/query.fcgi?db=gene&cmd=Retrieve&dopt=full_report&list_uids=80114) | BicC family RNA binding protein 1 |
| [Details](http://mirdb.org/cgi-bin/target_detail.cgi?targetID=598516) | 28 | 97 | hsa-miR-148a-3p | [ZDHHC7](http://www.ncbi.nlm.nih.gov/entrez/query.fcgi?db=gene&cmd=Retrieve&dopt=full_report&list_uids=55625) | zinc finger DHHC-type containing 7 |
| [Details](http://mirdb.org/cgi-bin/target_detail.cgi?targetID=598139) | 29 | 97 | hsa-miR-148a-3p | [B4GALT5](http://www.ncbi.nlm.nih.gov/entrez/query.fcgi?db=gene&cmd=Retrieve&dopt=full_report&list_uids=9334) | beta-1,4-galactosyltransferase 5 |
| [Details](http://mirdb.org/cgi-bin/target_detail.cgi?targetID=598393) | 30 | 97 | hsa-miR-148a-3p | [DCP2](http://www.ncbi.nlm.nih.gov/entrez/query.fcgi?db=gene&cmd=Retrieve&dopt=full_report&list_uids=167227) | decapping mRNA 2 |
| [Details](http://mirdb.org/cgi-bin/target_detail.cgi?targetID=597924) | 31 | 97 | hsa-miR-148a-3p | [NPEPL1](http://www.ncbi.nlm.nih.gov/entrez/query.fcgi?db=gene&cmd=Retrieve&dopt=full_report&list_uids=79716) | aminopeptidase like 1 |
| [Details](http://mirdb.org/cgi-bin/target_detail.cgi?targetID=598082) | 32 | 97 | hsa-miR-148a-3p | [ABCB7](http://www.ncbi.nlm.nih.gov/entrez/query.fcgi?db=gene&cmd=Retrieve&dopt=full_report&list_uids=22) | ATP binding cassette subfamily B member 7 |
| [Details](http://mirdb.org/cgi-bin/target_detail.cgi?targetID=598080) | 33 | 97 | hsa-miR-148a-3p | [VPS37A](http://www.ncbi.nlm.nih.gov/entrez/query.fcgi?db=gene&cmd=Retrieve&dopt=full_report&list_uids=137492) | VPS37A, ESCRT-I subunit |
| [Details](http://mirdb.org/cgi-bin/target_detail.cgi?targetID=598652) | 34 | 97 | hsa-miR-148a-3p | [TMEM9B](http://www.ncbi.nlm.nih.gov/entrez/query.fcgi?db=gene&cmd=Retrieve&dopt=full_report&list_uids=56674) | TMEM9 domain family member B |
| [Details](http://mirdb.org/cgi-bin/target_detail.cgi?targetID=598593) | 35 | 97 | hsa-miR-148a-3p | [OSBPL11](http://www.ncbi.nlm.nih.gov/entrez/query.fcgi?db=gene&cmd=Retrieve&dopt=full_report&list_uids=114885) | oxysterol binding protein like 11 |
| [Details](http://mirdb.org/cgi-bin/target_detail.cgi?targetID=598040) | 36 | 97 | hsa-miR-148a-3p | [USP33](http://www.ncbi.nlm.nih.gov/entrez/query.fcgi?db=gene&cmd=Retrieve&dopt=full_report&list_uids=23032) | ubiquitin specific peptidase 33 |
| [Details](http://mirdb.org/cgi-bin/target_detail.cgi?targetID=597878) | 37 | 97 | hsa-miR-148a-3p | [MED12L](http://www.ncbi.nlm.nih.gov/entrez/query.fcgi?db=gene&cmd=Retrieve&dopt=full_report&list_uids=116931) | mediator complex subunit 12 like |
| [Details](http://mirdb.org/cgi-bin/target_detail.cgi?targetID=598026) | 38 | 97 | hsa-miR-148a-3p | [PHF3](http://www.ncbi.nlm.nih.gov/entrez/query.fcgi?db=gene&cmd=Retrieve&dopt=full_report&list_uids=23469) | PHD finger protein 3 |
| [Details](http://mirdb.org/cgi-bin/target_detail.cgi?targetID=598073) | 39 | 97 | hsa-miR-148a-3p | [ARHGAP21](http://www.ncbi.nlm.nih.gov/entrez/query.fcgi?db=gene&cmd=Retrieve&dopt=full_report&list_uids=57584) | Rho GTPase activating protein 21 |
| [Details](http://mirdb.org/cgi-bin/target_detail.cgi?targetID=598030) | 40 | 97 | hsa-miR-148a-3p | [ATP6AP2](http://www.ncbi.nlm.nih.gov/entrez/query.fcgi?db=gene&cmd=Retrieve&dopt=full_report&list_uids=10159) | ATPase H+ transporting accessory protein 2 |
| [Details](http://mirdb.org/cgi-bin/target_detail.cgi?targetID=597862) | 41 | 97 | hsa-miR-148a-3p | [MAP3K9](http://www.ncbi.nlm.nih.gov/entrez/query.fcgi?db=gene&cmd=Retrieve&dopt=full_report&list_uids=4293) | mitogen-activated protein kinase kinase kinase 9 |
| [Details](http://mirdb.org/cgi-bin/target_detail.cgi?targetID=598490) | 42 | 96 | hsa-miR-148a-3p | [CLCN6](http://www.ncbi.nlm.nih.gov/entrez/query.fcgi?db=gene&cmd=Retrieve&dopt=full_report&list_uids=1185) | chloride voltage-gated channel 6 |
| [Details](http://mirdb.org/cgi-bin/target_detail.cgi?targetID=597915) | 43 | 96 | hsa-miR-148a-3p | [ADAMTS19](http://www.ncbi.nlm.nih.gov/entrez/query.fcgi?db=gene&cmd=Retrieve&dopt=full_report&list_uids=171019) | ADAM metallopeptidase with thrombospondin type 1 motif 19 |
| [Details](http://mirdb.org/cgi-bin/target_detail.cgi?targetID=598416) | 44 | 96 | hsa-miR-148a-3p | [GTF2H1](http://www.ncbi.nlm.nih.gov/entrez/query.fcgi?db=gene&cmd=Retrieve&dopt=full_report&list_uids=2965) | general transcription factor IIH subunit 1 |
| [Details](http://mirdb.org/cgi-bin/target_detail.cgi?targetID=597962) | 45 | 96 | hsa-miR-148a-3p | [TGFA](http://www.ncbi.nlm.nih.gov/entrez/query.fcgi?db=gene&cmd=Retrieve&dopt=full_report&list_uids=7039) | transforming growth factor alpha |
| [Details](http://mirdb.org/cgi-bin/target_detail.cgi?targetID=598024) | 46 | 96 | hsa-miR-148a-3p | [NEURL4](http://www.ncbi.nlm.nih.gov/entrez/query.fcgi?db=gene&cmd=Retrieve&dopt=full_report&list_uids=84461) | neuralized E3 ubiquitin protein ligase 4 |
| [Details](http://mirdb.org/cgi-bin/target_detail.cgi?targetID=598333) | 47 | 96 | hsa-miR-148a-3p | [INO80](http://www.ncbi.nlm.nih.gov/entrez/query.fcgi?db=gene&cmd=Retrieve&dopt=full_report&list_uids=54617) | INO80 complex subunit |
| [Details](http://mirdb.org/cgi-bin/target_detail.cgi?targetID=598487) | 48 | 96 | hsa-miR-148a-3p | [HOXC8](http://www.ncbi.nlm.nih.gov/entrez/query.fcgi?db=gene&cmd=Retrieve&dopt=full_report&list_uids=3224) | homeobox C8 |
| [Details](http://mirdb.org/cgi-bin/target_detail.cgi?targetID=598555) | 49 | 96 | hsa-miR-148a-3p | [ERBB3](http://www.ncbi.nlm.nih.gov/entrez/query.fcgi?db=gene&cmd=Retrieve&dopt=full_report&list_uids=2065) | erb-b2 receptor tyrosine kinase 3 |
| [Details](http://mirdb.org/cgi-bin/target_detail.cgi?targetID=598403) | 50 | 96 | hsa-miR-148a-3p | [TNRC6C](http://www.ncbi.nlm.nih.gov/entrez/query.fcgi?db=gene&cmd=Retrieve&dopt=full_report&list_uids=57690) | trinucleotide repeat containing 6C |
| [Details](http://mirdb.org/cgi-bin/target_detail.cgi?targetID=598226) | 51 | 96 | hsa-miR-148a-3p | [DNMT1](http://www.ncbi.nlm.nih.gov/entrez/query.fcgi?db=gene&cmd=Retrieve&dopt=full_report&list_uids=1786) | DNA methyltransferase 1 |
| [Details](http://mirdb.org/cgi-bin/target_detail.cgi?targetID=597935) | 52 | 96 | hsa-miR-148a-3p | [BTBD3](http://www.ncbi.nlm.nih.gov/entrez/query.fcgi?db=gene&cmd=Retrieve&dopt=full_report&list_uids=22903) | BTB domain containing 3 |
| [Details](http://mirdb.org/cgi-bin/target_detail.cgi?targetID=597853) | 53 | 96 | hsa-miR-148a-3p | [KIAA1217](http://www.ncbi.nlm.nih.gov/entrez/query.fcgi?db=gene&cmd=Retrieve&dopt=full_report&list_uids=56243) | KIAA1217 |
| [Details](http://mirdb.org/cgi-bin/target_detail.cgi?targetID=598255) | 54 | 96 | hsa-miR-148a-3p | [TNRC6A](http://www.ncbi.nlm.nih.gov/entrez/query.fcgi?db=gene&cmd=Retrieve&dopt=full_report&list_uids=27327) | trinucleotide repeat containing 6A |
| [Details](http://mirdb.org/cgi-bin/target_detail.cgi?targetID=598384) | 55 | 96 | hsa-miR-148a-3p | [GADD45A](http://www.ncbi.nlm.nih.gov/entrez/query.fcgi?db=gene&cmd=Retrieve&dopt=full_report&list_uids=1647) | growth arrest and DNA damage inducible alpha |
| [Details](http://mirdb.org/cgi-bin/target_detail.cgi?targetID=597893) | 56 | 96 | hsa-miR-148a-3p | [ESR1](http://www.ncbi.nlm.nih.gov/entrez/query.fcgi?db=gene&cmd=Retrieve&dopt=full_report&list_uids=2099) | estrogen receptor 1 |
| [Details](http://mirdb.org/cgi-bin/target_detail.cgi?targetID=598241) | 57 | 96 | hsa-miR-148a-3p | [PPP1R10](http://www.ncbi.nlm.nih.gov/entrez/query.fcgi?db=gene&cmd=Retrieve&dopt=full_report&list_uids=5514) | protein phosphatase 1 regulatory subunit 10 |
| [Details](http://mirdb.org/cgi-bin/target_detail.cgi?targetID=598365) | 58 | 96 | hsa-miR-148a-3p | [CCT6A](http://www.ncbi.nlm.nih.gov/entrez/query.fcgi?db=gene&cmd=Retrieve&dopt=full_report&list_uids=908) | chaperonin containing TCP1 subunit 6A |
| [Details](http://mirdb.org/cgi-bin/target_detail.cgi?targetID=598228) | 59 | 95 | hsa-miR-148a-3p | [AGO4](http://www.ncbi.nlm.nih.gov/entrez/query.fcgi?db=gene&cmd=Retrieve&dopt=full_report&list_uids=192670) | argonaute RISC catalytic component 4 |
| [Details](http://mirdb.org/cgi-bin/target_detail.cgi?targetID=598454) | 60 | 95 | hsa-miR-148a-3p | [LMTK2](http://www.ncbi.nlm.nih.gov/entrez/query.fcgi?db=gene&cmd=Retrieve&dopt=full_report&list_uids=22853) | lemur tyrosine kinase 2 |
| [Details](http://mirdb.org/cgi-bin/target_detail.cgi?targetID=598096) | 61 | 95 | hsa-miR-148a-3p | [ARL6IP1](http://www.ncbi.nlm.nih.gov/entrez/query.fcgi?db=gene&cmd=Retrieve&dopt=full_report&list_uids=23204) | ADP ribosylation factor like GTPase 6 interacting protein 1 |
| [Details](http://mirdb.org/cgi-bin/target_detail.cgi?targetID=597920) | 62 | 95 | hsa-miR-148a-3p | [KIAA0232](http://www.ncbi.nlm.nih.gov/entrez/query.fcgi?db=gene&cmd=Retrieve&dopt=full_report&list_uids=9778) | KIAA0232 |
| [Details](http://mirdb.org/cgi-bin/target_detail.cgi?targetID=598160) | 63 | 95 | hsa-miR-148a-3p | [MXD1](http://www.ncbi.nlm.nih.gov/entrez/query.fcgi?db=gene&cmd=Retrieve&dopt=full_report&list_uids=4084) | MAX dimerization protein 1 |
| [Details](http://mirdb.org/cgi-bin/target_detail.cgi?targetID=598441) | 64 | 95 | hsa-miR-148a-3p | [CABP7](http://www.ncbi.nlm.nih.gov/entrez/query.fcgi?db=gene&cmd=Retrieve&dopt=full_report&list_uids=164633) | calcium binding protein 7 |
| [Details](http://mirdb.org/cgi-bin/target_detail.cgi?targetID=598035) | 65 | 95 | hsa-miR-148a-3p | [XPO4](http://www.ncbi.nlm.nih.gov/entrez/query.fcgi?db=gene&cmd=Retrieve&dopt=full_report&list_uids=64328) | exportin 4 |
| [Details](http://mirdb.org/cgi-bin/target_detail.cgi?targetID=598018) | 66 | 95 | hsa-miR-148a-3p | [POU3F2](http://www.ncbi.nlm.nih.gov/entrez/query.fcgi?db=gene&cmd=Retrieve&dopt=full_report&list_uids=5454) | POU class 3 homeobox 2 |
| [Details](http://mirdb.org/cgi-bin/target_detail.cgi?targetID=598140) | 67 | 95 | hsa-miR-148a-3p | [FAM168B](http://www.ncbi.nlm.nih.gov/entrez/query.fcgi?db=gene&cmd=Retrieve&dopt=full_report&list_uids=130074) | family with sequence similarity 168 member B |
| [Details](http://mirdb.org/cgi-bin/target_detail.cgi?targetID=598364) | 68 | 95 | hsa-miR-148a-3p | [LIPA](http://www.ncbi.nlm.nih.gov/entrez/query.fcgi?db=gene&cmd=Retrieve&dopt=full_report&list_uids=3988) | lipase A, lysosomal acid type |
| [Details](http://mirdb.org/cgi-bin/target_detail.cgi?targetID=598120) | 69 | 95 | hsa-miR-148a-3p | [GLRX5](http://www.ncbi.nlm.nih.gov/entrez/query.fcgi?db=gene&cmd=Retrieve&dopt=full_report&list_uids=51218) | glutaredoxin 5 |
| [Details](http://mirdb.org/cgi-bin/target_detail.cgi?targetID=598145) | 70 | 95 | hsa-miR-148a-3p | [ERRFI1](http://www.ncbi.nlm.nih.gov/entrez/query.fcgi?db=gene&cmd=Retrieve&dopt=full_report&list_uids=54206) | ERBB receptor feedback inhibitor 1 |
| [Details](http://mirdb.org/cgi-bin/target_detail.cgi?targetID=598342) | 71 | 95 | hsa-miR-148a-3p | [COL4A1](http://www.ncbi.nlm.nih.gov/entrez/query.fcgi?db=gene&cmd=Retrieve&dopt=full_report&list_uids=1282) | collagen type IV alpha 1 chain |
| [Details](http://mirdb.org/cgi-bin/target_detail.cgi?targetID=598276) | 72 | 95 | hsa-miR-148a-3p | [YWHAB](http://www.ncbi.nlm.nih.gov/entrez/query.fcgi?db=gene&cmd=Retrieve&dopt=full_report&list_uids=7529) | tyrosine 3-monooxygenase/tryptophan 5-monooxygenase activation protein beta |
| [Details](http://mirdb.org/cgi-bin/target_detail.cgi?targetID=598576) | 73 | 95 | hsa-miR-148a-3p | [INHBB](http://www.ncbi.nlm.nih.gov/entrez/query.fcgi?db=gene&cmd=Retrieve&dopt=full_report&list_uids=3625) | inhibin subunit beta B |
| [Details](http://mirdb.org/cgi-bin/target_detail.cgi?targetID=598075) | 74 | 94 | hsa-miR-148a-3p | [RNF38](http://www.ncbi.nlm.nih.gov/entrez/query.fcgi?db=gene&cmd=Retrieve&dopt=full_report&list_uids=152006) | ring finger protein 38 |
| [Details](http://mirdb.org/cgi-bin/target_detail.cgi?targetID=598546) | 75 | 94 | hsa-miR-148a-3p | [RALBP1](http://www.ncbi.nlm.nih.gov/entrez/query.fcgi?db=gene&cmd=Retrieve&dopt=full_report&list_uids=10928) | ralA binding protein 1 |
| [Details](http://mirdb.org/cgi-bin/target_detail.cgi?targetID=598535) | 76 | 94 | hsa-miR-148a-3p | [NRP1](http://www.ncbi.nlm.nih.gov/entrez/query.fcgi?db=gene&cmd=Retrieve&dopt=full_report&list_uids=8829) | neuropilin 1 |
| [Details](http://mirdb.org/cgi-bin/target_detail.cgi?targetID=598099) | 77 | 94 | hsa-miR-148a-3p | [ARL8B](http://www.ncbi.nlm.nih.gov/entrez/query.fcgi?db=gene&cmd=Retrieve&dopt=full_report&list_uids=55207) | ADP ribosylation factor like GTPase 8B |
| [Details](http://mirdb.org/cgi-bin/target_detail.cgi?targetID=598602) | 78 | 94 | hsa-miR-148a-3p | [SESTD1](http://www.ncbi.nlm.nih.gov/entrez/query.fcgi?db=gene&cmd=Retrieve&dopt=full_report&list_uids=91404) | SEC14 and spectrin domain containing 1 |
| [Details](http://mirdb.org/cgi-bin/target_detail.cgi?targetID=598220) | 79 | 94 | hsa-miR-148a-3p | [KRT76](http://www.ncbi.nlm.nih.gov/entrez/query.fcgi?db=gene&cmd=Retrieve&dopt=full_report&list_uids=51350) | keratin 76 |
| [Details](http://mirdb.org/cgi-bin/target_detail.cgi?targetID=598015) | 80 | 94 | hsa-miR-148a-3p | [MRGPRX3](http://www.ncbi.nlm.nih.gov/entrez/query.fcgi?db=gene&cmd=Retrieve&dopt=full_report&list_uids=117195) | MAS related GPR family member X3 |
| [Details](http://mirdb.org/cgi-bin/target_detail.cgi?targetID=598295) | 81 | 94 | hsa-miR-148a-3p | [LRP2](http://www.ncbi.nlm.nih.gov/entrez/query.fcgi?db=gene&cmd=Retrieve&dopt=full_report&list_uids=4036) | LDL receptor related protein 2 |
| [Details](http://mirdb.org/cgi-bin/target_detail.cgi?targetID=598561) | 82 | 94 | hsa-miR-148a-3p | [SESN2](http://www.ncbi.nlm.nih.gov/entrez/query.fcgi?db=gene&cmd=Retrieve&dopt=full_report&list_uids=83667) | sestrin 2 |
| [Details](http://mirdb.org/cgi-bin/target_detail.cgi?targetID=598038) | 83 | 94 | hsa-miR-148a-3p | [CAND1](http://www.ncbi.nlm.nih.gov/entrez/query.fcgi?db=gene&cmd=Retrieve&dopt=full_report&list_uids=55832) | cullin associated and neddylation dissociated 1 |
| [Details](http://mirdb.org/cgi-bin/target_detail.cgi?targetID=598154) | 84 | 94 | hsa-miR-148a-3p | [WNT1](http://www.ncbi.nlm.nih.gov/entrez/query.fcgi?db=gene&cmd=Retrieve&dopt=full_report&list_uids=7471) | Wnt family member 1 |
| [Details](http://mirdb.org/cgi-bin/target_detail.cgi?targetID=598611) | 85 | 94 | hsa-miR-148a-3p | [BAZ2B](http://www.ncbi.nlm.nih.gov/entrez/query.fcgi?db=gene&cmd=Retrieve&dopt=full_report&list_uids=29994) | bromodomain adjacent to zinc finger domain 2B |
| [Details](http://mirdb.org/cgi-bin/target_detail.cgi?targetID=598009) | 86 | 94 | hsa-miR-148a-3p | [SMS](http://www.ncbi.nlm.nih.gov/entrez/query.fcgi?db=gene&cmd=Retrieve&dopt=full_report&list_uids=6611) | spermine synthase |
| [Details](http://mirdb.org/cgi-bin/target_detail.cgi?targetID=598331) | 87 | 94 | hsa-miR-148a-3p | [FBN1](http://www.ncbi.nlm.nih.gov/entrez/query.fcgi?db=gene&cmd=Retrieve&dopt=full_report&list_uids=2200) | fibrillin 1 |
| [Details](http://mirdb.org/cgi-bin/target_detail.cgi?targetID=597889) | 88 | 94 | hsa-miR-148a-3p | [DDX6](http://www.ncbi.nlm.nih.gov/entrez/query.fcgi?db=gene&cmd=Retrieve&dopt=full_report&list_uids=1656) | DEAD-box helicase 6 |
| [Details](http://mirdb.org/cgi-bin/target_detail.cgi?targetID=598261) | 89 | 94 | hsa-miR-148a-3p | [ADGRB3](http://www.ncbi.nlm.nih.gov/entrez/query.fcgi?db=gene&cmd=Retrieve&dopt=full_report&list_uids=577) | adhesion G protein-coupled receptor B3 |
| [Details](http://mirdb.org/cgi-bin/target_detail.cgi?targetID=597976) | 90 | 94 | hsa-miR-148a-3p | [ROBO2](http://www.ncbi.nlm.nih.gov/entrez/query.fcgi?db=gene&cmd=Retrieve&dopt=full_report&list_uids=6092) | roundabout guidance receptor 2 |
| [Details](http://mirdb.org/cgi-bin/target_detail.cgi?targetID=598345) | 91 | 94 | hsa-miR-148a-3p | [EOGT](http://www.ncbi.nlm.nih.gov/entrez/query.fcgi?db=gene&cmd=Retrieve&dopt=full_report&list_uids=285203) | EGF domain specific O-linked N-acetylglucosamine transferase |
| [Details](http://mirdb.org/cgi-bin/target_detail.cgi?targetID=598324) | 92 | 94 | hsa-miR-148a-3p | [LEPROTL1](http://www.ncbi.nlm.nih.gov/entrez/query.fcgi?db=gene&cmd=Retrieve&dopt=full_report&list_uids=23484) | leptin receptor overlapping transcript like 1 |
| [Details](http://mirdb.org/cgi-bin/target_detail.cgi?targetID=597894) | 93 | 94 | hsa-miR-148a-3p | [TENT2](http://www.ncbi.nlm.nih.gov/entrez/query.fcgi?db=gene&cmd=Retrieve&dopt=full_report&list_uids=167153) | terminal nucleotidyltransferase 2 |
| [Details](http://mirdb.org/cgi-bin/target_detail.cgi?targetID=598683) | 94 | 93 | hsa-miR-148a-3p | [ADAM22](http://www.ncbi.nlm.nih.gov/entrez/query.fcgi?db=gene&cmd=Retrieve&dopt=full_report&list_uids=53616) | ADAM metallopeptidase domain 22 |
| [Details](http://mirdb.org/cgi-bin/target_detail.cgi?targetID=598004) | 95 | 93 | hsa-miR-148a-3p | [TBC1D8](http://www.ncbi.nlm.nih.gov/entrez/query.fcgi?db=gene&cmd=Retrieve&dopt=full_report&list_uids=11138) | TBC1 domain family member 8 |
| [Details](http://mirdb.org/cgi-bin/target_detail.cgi?targetID=598672) | 96 | 93 | hsa-miR-148a-3p | [AGFG1](http://www.ncbi.nlm.nih.gov/entrez/query.fcgi?db=gene&cmd=Retrieve&dopt=full_report&list_uids=3267) | ArfGAP with FG repeats 1 |
| [Details](http://mirdb.org/cgi-bin/target_detail.cgi?targetID=598074) | 97 | 93 | hsa-miR-148a-3p | [PNPLA6](http://www.ncbi.nlm.nih.gov/entrez/query.fcgi?db=gene&cmd=Retrieve&dopt=full_report&list_uids=10908) | patatin like phospholipase domain containing 6 |
| [Details](http://mirdb.org/cgi-bin/target_detail.cgi?targetID=598159) | 98 | 93 | hsa-miR-148a-3p | [SNX27](http://www.ncbi.nlm.nih.gov/entrez/query.fcgi?db=gene&cmd=Retrieve&dopt=full_report&list_uids=81609) | sorting nexin family member 27 |
| [Details](http://mirdb.org/cgi-bin/target_detail.cgi?targetID=598412) | 99 | 93 | hsa-miR-148a-3p | [GMFB](http://www.ncbi.nlm.nih.gov/entrez/query.fcgi?db=gene&cmd=Retrieve&dopt=full_report&list_uids=2764) | glia maturation factor beta |
| [Details](http://mirdb.org/cgi-bin/target_detail.cgi?targetID=598409) | 100 | 93 | hsa-miR-148a-3p | [CCDC6](http://www.ncbi.nlm.nih.gov/entrez/query.fcgi?db=gene&cmd=Retrieve&dopt=full_report&list_uids=8030) | coiled-coil domain containing 6 |
| [Details](http://mirdb.org/cgi-bin/target_detail.cgi?targetID=598063) | 101 | 93 | hsa-miR-148a-3p | [ATG14](http://www.ncbi.nlm.nih.gov/entrez/query.fcgi?db=gene&cmd=Retrieve&dopt=full_report&list_uids=22863) | autophagy related 14 |
| [Details](http://mirdb.org/cgi-bin/target_detail.cgi?targetID=598550) | 102 | 93 | hsa-miR-148a-3p | [PHF20](http://www.ncbi.nlm.nih.gov/entrez/query.fcgi?db=gene&cmd=Retrieve&dopt=full_report&list_uids=51230) | PHD finger protein 20 |
| [Details](http://mirdb.org/cgi-bin/target_detail.cgi?targetID=598273) | 103 | 93 | hsa-miR-148a-3p | [PLAA](http://www.ncbi.nlm.nih.gov/entrez/query.fcgi?db=gene&cmd=Retrieve&dopt=full_report&list_uids=9373) | phospholipase A2 activating protein |
| [Details](http://mirdb.org/cgi-bin/target_detail.cgi?targetID=598608) | 104 | 93 | hsa-miR-148a-3p | [SGCB](http://www.ncbi.nlm.nih.gov/entrez/query.fcgi?db=gene&cmd=Retrieve&dopt=full_report&list_uids=6443) | sarcoglycan beta |
| [Details](http://mirdb.org/cgi-bin/target_detail.cgi?targetID=598043) | 105 | 93 | hsa-miR-148a-3p | [RAB34](http://www.ncbi.nlm.nih.gov/entrez/query.fcgi?db=gene&cmd=Retrieve&dopt=full_report&list_uids=83871) | RAB34, member RAS oncogene family |
| [Details](http://mirdb.org/cgi-bin/target_detail.cgi?targetID=597898) | 106 | 93 | hsa-miR-148a-3p | [ZDHHC23](http://www.ncbi.nlm.nih.gov/entrez/query.fcgi?db=gene&cmd=Retrieve&dopt=full_report&list_uids=254887) | zinc finger DHHC-type containing 23 |
| [Details](http://mirdb.org/cgi-bin/target_detail.cgi?targetID=598300) | 107 | 93 | hsa-miR-148a-3p | [ATP11A](http://www.ncbi.nlm.nih.gov/entrez/query.fcgi?db=gene&cmd=Retrieve&dopt=full_report&list_uids=23250) | ATPase phospholipid transporting 11A |
| [Details](http://mirdb.org/cgi-bin/target_detail.cgi?targetID=598002) | 108 | 93 | hsa-miR-148a-3p | [ACVR1](http://www.ncbi.nlm.nih.gov/entrez/query.fcgi?db=gene&cmd=Retrieve&dopt=full_report&list_uids=90) | activin A receptor type 1 |
| [Details](http://mirdb.org/cgi-bin/target_detail.cgi?targetID=598351) | 109 | 93 | hsa-miR-148a-3p | [RASSF8](http://www.ncbi.nlm.nih.gov/entrez/query.fcgi?db=gene&cmd=Retrieve&dopt=full_report&list_uids=11228) | Ras association domain family member 8 |
| [Details](http://mirdb.org/cgi-bin/target_detail.cgi?targetID=597858) | 110 | 93 | hsa-miR-148a-3p | [STARD13](http://www.ncbi.nlm.nih.gov/entrez/query.fcgi?db=gene&cmd=Retrieve&dopt=full_report&list_uids=90627) | StAR related lipid transfer domain containing 13 |
| [Details](http://mirdb.org/cgi-bin/target_detail.cgi?targetID=597969) | 111 | 93 | hsa-miR-148a-3p | [FAM43A](http://www.ncbi.nlm.nih.gov/entrez/query.fcgi?db=gene&cmd=Retrieve&dopt=full_report&list_uids=131583) | family with sequence similarity 43 member A |
| [Details](http://mirdb.org/cgi-bin/target_detail.cgi?targetID=598053) | 112 | 93 | hsa-miR-148a-3p | [ESRRG](http://www.ncbi.nlm.nih.gov/entrez/query.fcgi?db=gene&cmd=Retrieve&dopt=full_report&list_uids=2104) | estrogen related receptor gamma |
| [Details](http://mirdb.org/cgi-bin/target_detail.cgi?targetID=598386) | 113 | 93 | hsa-miR-148a-3p | [TMED7](http://www.ncbi.nlm.nih.gov/entrez/query.fcgi?db=gene&cmd=Retrieve&dopt=full_report&list_uids=51014) | transmembrane p24 trafficking protein 7 |
| [Details](http://mirdb.org/cgi-bin/target_detail.cgi?targetID=598107) | 114 | 93 | hsa-miR-148a-3p | [LBR](http://www.ncbi.nlm.nih.gov/entrez/query.fcgi?db=gene&cmd=Retrieve&dopt=full_report&list_uids=3930) | lamin B receptor |
| [Details](http://mirdb.org/cgi-bin/target_detail.cgi?targetID=598356) | 115 | 93 | hsa-miR-148a-3p | [KLF6](http://www.ncbi.nlm.nih.gov/entrez/query.fcgi?db=gene&cmd=Retrieve&dopt=full_report&list_uids=1316) | Kruppel like factor 6 |
| [Details](http://mirdb.org/cgi-bin/target_detail.cgi?targetID=598371) | 116 | 92 | hsa-miR-148a-3p | [HECW2](http://www.ncbi.nlm.nih.gov/entrez/query.fcgi?db=gene&cmd=Retrieve&dopt=full_report&list_uids=57520) | HECT, C2 and WW domain containing E3 ubiquitin protein ligase 2 |
| [Details](http://mirdb.org/cgi-bin/target_detail.cgi?targetID=598660) | 117 | 92 | hsa-miR-148a-3p | [FAM104A](http://www.ncbi.nlm.nih.gov/entrez/query.fcgi?db=gene&cmd=Retrieve&dopt=full_report&list_uids=84923) | family with sequence similarity 104 member A |
| [Details](http://mirdb.org/cgi-bin/target_detail.cgi?targetID=598079) | 118 | 92 | hsa-miR-148a-3p | [ITGB8](http://www.ncbi.nlm.nih.gov/entrez/query.fcgi?db=gene&cmd=Retrieve&dopt=full_report&list_uids=3696) | integrin subunit beta 8 |
| [Details](http://mirdb.org/cgi-bin/target_detail.cgi?targetID=598378) | 119 | 92 | hsa-miR-148a-3p | [SNAP91](http://www.ncbi.nlm.nih.gov/entrez/query.fcgi?db=gene&cmd=Retrieve&dopt=full_report&list_uids=9892) | synaptosome associated protein 91 |
| [Details](http://mirdb.org/cgi-bin/target_detail.cgi?targetID=597876) | 120 | 92 | hsa-miR-148a-3p | [TGIF2](http://www.ncbi.nlm.nih.gov/entrez/query.fcgi?db=gene&cmd=Retrieve&dopt=full_report&list_uids=60436) | TGFB induced factor homeobox 2 |
| [Details](http://mirdb.org/cgi-bin/target_detail.cgi?targetID=598512) | 121 | 92 | hsa-miR-148a-3p | [C1GALT1](http://www.ncbi.nlm.nih.gov/entrez/query.fcgi?db=gene&cmd=Retrieve&dopt=full_report&list_uids=56913) | core 1 synthase, glycoprotein-N-acetylgalactosamine 3-beta-galactosyltransferase 1 |
| [Details](http://mirdb.org/cgi-bin/target_detail.cgi?targetID=598373) | 122 | 92 | hsa-miR-148a-3p | [CS](http://www.ncbi.nlm.nih.gov/entrez/query.fcgi?db=gene&cmd=Retrieve&dopt=full_report&list_uids=1431) | citrate synthase |
| [Details](http://mirdb.org/cgi-bin/target_detail.cgi?targetID=598529) | 123 | 92 | hsa-miR-148a-3p | [ZDHHC17](http://www.ncbi.nlm.nih.gov/entrez/query.fcgi?db=gene&cmd=Retrieve&dopt=full_report&list_uids=23390) | zinc finger DHHC-type containing 17 |
| [Details](http://mirdb.org/cgi-bin/target_detail.cgi?targetID=597983) | 124 | 92 | hsa-miR-148a-3p | [DOCK6](http://www.ncbi.nlm.nih.gov/entrez/query.fcgi?db=gene&cmd=Retrieve&dopt=full_report&list_uids=57572) | dedicator of cytokinesis 6 |
| [Details](http://mirdb.org/cgi-bin/target_detail.cgi?targetID=598125) | 125 | 92 | hsa-miR-148a-3p | [SLC24A3](http://www.ncbi.nlm.nih.gov/entrez/query.fcgi?db=gene&cmd=Retrieve&dopt=full_report&list_uids=57419) | solute carrier family 24 member 3 |
| [Details](http://mirdb.org/cgi-bin/target_detail.cgi?targetID=597977) | 126 | 92 | hsa-miR-148a-3p | [RBM24](http://www.ncbi.nlm.nih.gov/entrez/query.fcgi?db=gene&cmd=Retrieve&dopt=full_report&list_uids=221662) | RNA binding motif protein 24 |
| [Details](http://mirdb.org/cgi-bin/target_detail.cgi?targetID=598221) | 127 | 92 | hsa-miR-148a-3p | [SKIDA1](http://www.ncbi.nlm.nih.gov/entrez/query.fcgi?db=gene&cmd=Retrieve&dopt=full_report&list_uids=387640) | SKI/DACH domain containing 1 |
| [Details](http://mirdb.org/cgi-bin/target_detail.cgi?targetID=598175) | 128 | 92 | hsa-miR-148a-3p | [GRID2](http://www.ncbi.nlm.nih.gov/entrez/query.fcgi?db=gene&cmd=Retrieve&dopt=full_report&list_uids=2895) | glutamate ionotropic receptor delta type subunit 2 |
| [Details](http://mirdb.org/cgi-bin/target_detail.cgi?targetID=598234) | 129 | 92 | hsa-miR-148a-3p | [SLC25A44](http://www.ncbi.nlm.nih.gov/entrez/query.fcgi?db=gene&cmd=Retrieve&dopt=full_report&list_uids=9673) | solute carrier family 25 member 44 |
| [Details](http://mirdb.org/cgi-bin/target_detail.cgi?targetID=598352) | 130 | 92 | hsa-miR-148a-3p | [E2F7](http://www.ncbi.nlm.nih.gov/entrez/query.fcgi?db=gene&cmd=Retrieve&dopt=full_report&list_uids=144455) | E2F transcription factor 7 |
| [Details](http://mirdb.org/cgi-bin/target_detail.cgi?targetID=598025) | 131 | 92 | hsa-miR-148a-3p | [DICER1](http://www.ncbi.nlm.nih.gov/entrez/query.fcgi?db=gene&cmd=Retrieve&dopt=full_report&list_uids=23405) | dicer 1, ribonuclease III |
| [Details](http://mirdb.org/cgi-bin/target_detail.cgi?targetID=598258) | 132 | 92 | hsa-miR-148a-3p | [SIK1B](http://www.ncbi.nlm.nih.gov/entrez/query.fcgi?db=gene&cmd=Retrieve&dopt=full_report&list_uids=102724428) | salt inducible kinase 1B (putative) |
| [Details](http://mirdb.org/cgi-bin/target_detail.cgi?targetID=598689) | 133 | 92 | hsa-miR-148a-3p | [CYTH3](http://www.ncbi.nlm.nih.gov/entrez/query.fcgi?db=gene&cmd=Retrieve&dopt=full_report&list_uids=9265) | cytohesin 3 |
| [Details](http://mirdb.org/cgi-bin/target_detail.cgi?targetID=598158) | 134 | 92 | hsa-miR-148a-3p | [VSIG1](http://www.ncbi.nlm.nih.gov/entrez/query.fcgi?db=gene&cmd=Retrieve&dopt=full_report&list_uids=340547) | V-set and immunoglobulin domain containing 1 |
| [Details](http://mirdb.org/cgi-bin/target_detail.cgi?targetID=598062) | 135 | 92 | hsa-miR-148a-3p | [SIK1](http://www.ncbi.nlm.nih.gov/entrez/query.fcgi?db=gene&cmd=Retrieve&dopt=full_report&list_uids=150094) | salt inducible kinase 1 |
| [Details](http://mirdb.org/cgi-bin/target_detail.cgi?targetID=598450) | 136 | 92 | hsa-miR-148a-3p | [CDKN1B](http://www.ncbi.nlm.nih.gov/entrez/query.fcgi?db=gene&cmd=Retrieve&dopt=full_report&list_uids=1027) | cyclin dependent kinase inhibitor 1B |
| [Details](http://mirdb.org/cgi-bin/target_detail.cgi?targetID=598150) | 137 | 92 | hsa-miR-148a-3p | [DMXL1](http://www.ncbi.nlm.nih.gov/entrez/query.fcgi?db=gene&cmd=Retrieve&dopt=full_report&list_uids=1657) | Dmx like 1 |
| [Details](http://mirdb.org/cgi-bin/target_detail.cgi?targetID=598148) | 138 | 92 | hsa-miR-148a-3p | [F3](http://www.ncbi.nlm.nih.gov/entrez/query.fcgi?db=gene&cmd=Retrieve&dopt=full_report&list_uids=2152) | coagulation factor III, tissue factor |
| [Details](http://mirdb.org/cgi-bin/target_detail.cgi?targetID=598281) | 139 | 92 | hsa-miR-148a-3p | [PPP6R1](http://www.ncbi.nlm.nih.gov/entrez/query.fcgi?db=gene&cmd=Retrieve&dopt=full_report&list_uids=22870) | protein phosphatase 6 regulatory subunit 1 |
| [Details](http://mirdb.org/cgi-bin/target_detail.cgi?targetID=598246) | 140 | 92 | hsa-miR-148a-3p | [JARID2](http://www.ncbi.nlm.nih.gov/entrez/query.fcgi?db=gene&cmd=Retrieve&dopt=full_report&list_uids=3720) | jumonji and AT-rich interaction domain containing 2 |
| [Details](http://mirdb.org/cgi-bin/target_detail.cgi?targetID=598308) | 141 | 91 | hsa-miR-148a-3p | [NCKIPSD](http://www.ncbi.nlm.nih.gov/entrez/query.fcgi?db=gene&cmd=Retrieve&dopt=full_report&list_uids=51517) | NCK interacting protein with SH3 domain |
| [Details](http://mirdb.org/cgi-bin/target_detail.cgi?targetID=598054) | 142 | 91 | hsa-miR-148a-3p | [CDH20](http://www.ncbi.nlm.nih.gov/entrez/query.fcgi?db=gene&cmd=Retrieve&dopt=full_report&list_uids=28316) | cadherin 20 |
| [Details](http://mirdb.org/cgi-bin/target_detail.cgi?targetID=597860) | 143 | 91 | hsa-miR-148a-3p | [ANGEL2](http://www.ncbi.nlm.nih.gov/entrez/query.fcgi?db=gene&cmd=Retrieve&dopt=full_report&list_uids=90806) | angel homolog 2 |
| [Details](http://mirdb.org/cgi-bin/target_detail.cgi?targetID=597863) | 144 | 91 | hsa-miR-148a-3p | [MMP10](http://www.ncbi.nlm.nih.gov/entrez/query.fcgi?db=gene&cmd=Retrieve&dopt=full_report&list_uids=4319) | matrix metallopeptidase 10 |
| [Details](http://mirdb.org/cgi-bin/target_detail.cgi?targetID=598667) | 145 | 91 | hsa-miR-148a-3p | [PITPNM2](http://www.ncbi.nlm.nih.gov/entrez/query.fcgi?db=gene&cmd=Retrieve&dopt=full_report&list_uids=57605) | phosphatidylinositol transfer protein membrane associated 2 |
| [Details](http://mirdb.org/cgi-bin/target_detail.cgi?targetID=598340) | 146 | 91 | hsa-miR-148a-3p | [MMP15](http://www.ncbi.nlm.nih.gov/entrez/query.fcgi?db=gene&cmd=Retrieve&dopt=full_report&list_uids=4324) | matrix metallopeptidase 15 |
| [Details](http://mirdb.org/cgi-bin/target_detail.cgi?targetID=598678) | 147 | 91 | hsa-miR-148a-3p | [TMEM170A](http://www.ncbi.nlm.nih.gov/entrez/query.fcgi?db=gene&cmd=Retrieve&dopt=full_report&list_uids=124491) | transmembrane protein 170A |
| [Details](http://mirdb.org/cgi-bin/target_detail.cgi?targetID=598668) | 148 | 91 | hsa-miR-148a-3p | [A4GNT](http://www.ncbi.nlm.nih.gov/entrez/query.fcgi?db=gene&cmd=Retrieve&dopt=full_report&list_uids=51146) | alpha-1,4-N-acetylglucosaminyltransferase |
| [Details](http://mirdb.org/cgi-bin/target_detail.cgi?targetID=598623) | 149 | 91 | hsa-miR-148a-3p | [SECISBP2L](http://www.ncbi.nlm.nih.gov/entrez/query.fcgi?db=gene&cmd=Retrieve&dopt=full_report&list_uids=9728) | SECIS binding protein 2 like |
| [Details](http://mirdb.org/cgi-bin/target_detail.cgi?targetID=598466) | 150 | 91 | hsa-miR-148a-3p | [KCTD16](http://www.ncbi.nlm.nih.gov/entrez/query.fcgi?db=gene&cmd=Retrieve&dopt=full_report&list_uids=57528) | potassium channel tetramerization domain containing 16 |
| [Details](http://mirdb.org/cgi-bin/target_detail.cgi?targetID=598496) | 151 | 91 | hsa-miR-148a-3p | [HBS1L](http://www.ncbi.nlm.nih.gov/entrez/query.fcgi?db=gene&cmd=Retrieve&dopt=full_report&list_uids=10767) | HBS1 like translational GTPase |
| [Details](http://mirdb.org/cgi-bin/target_detail.cgi?targetID=598213) | 152 | 91 | hsa-miR-148a-3p | [BMP3](http://www.ncbi.nlm.nih.gov/entrez/query.fcgi?db=gene&cmd=Retrieve&dopt=full_report&list_uids=651) | bone morphogenetic protein 3 |
| [Details](http://mirdb.org/cgi-bin/target_detail.cgi?targetID=598570) | 153 | 91 | hsa-miR-148a-3p | [NR2C2AP](http://www.ncbi.nlm.nih.gov/entrez/query.fcgi?db=gene&cmd=Retrieve&dopt=full_report&list_uids=126382) | nuclear receptor 2C2 associated protein |
| [Details](http://mirdb.org/cgi-bin/target_detail.cgi?targetID=598481) | 154 | 91 | hsa-miR-148a-3p | [SRSF11](http://www.ncbi.nlm.nih.gov/entrez/query.fcgi?db=gene&cmd=Retrieve&dopt=full_report&list_uids=9295) | serine and arginine rich splicing factor 11 |
| [Details](http://mirdb.org/cgi-bin/target_detail.cgi?targetID=598472) | 155 | 91 | hsa-miR-148a-3p | [CDC14A](http://www.ncbi.nlm.nih.gov/entrez/query.fcgi?db=gene&cmd=Retrieve&dopt=full_report&list_uids=8556) | cell division cycle 14A |
| [Details](http://mirdb.org/cgi-bin/target_detail.cgi?targetID=598629) | 156 | 91 | hsa-miR-148a-3p | [CYB5R4](http://www.ncbi.nlm.nih.gov/entrez/query.fcgi?db=gene&cmd=Retrieve&dopt=full_report&list_uids=51167) | cytochrome b5 reductase 4 |
| [Details](http://mirdb.org/cgi-bin/target_detail.cgi?targetID=598391) | 157 | 91 | hsa-miR-148a-3p | [NHS](http://www.ncbi.nlm.nih.gov/entrez/query.fcgi?db=gene&cmd=Retrieve&dopt=full_report&list_uids=4810) | NHS actin remodeling regulator |
| [Details](http://mirdb.org/cgi-bin/target_detail.cgi?targetID=598296) | 158 | 91 | hsa-miR-148a-3p | [NFAT5](http://www.ncbi.nlm.nih.gov/entrez/query.fcgi?db=gene&cmd=Retrieve&dopt=full_report&list_uids=10725) | nuclear factor of activated T cells 5 |
| [Details](http://mirdb.org/cgi-bin/target_detail.cgi?targetID=597896) | 159 | 91 | hsa-miR-148a-3p | [S1PR1](http://www.ncbi.nlm.nih.gov/entrez/query.fcgi?db=gene&cmd=Retrieve&dopt=full_report&list_uids=1901) | sphingosine-1-phosphate receptor 1 |
| [Details](http://mirdb.org/cgi-bin/target_detail.cgi?targetID=598592) | 160 | 91 | hsa-miR-148a-3p | [TMEM63B](http://www.ncbi.nlm.nih.gov/entrez/query.fcgi?db=gene&cmd=Retrieve&dopt=full_report&list_uids=55362) | transmembrane protein 63B |
| [Details](http://mirdb.org/cgi-bin/target_detail.cgi?targetID=598527) | 161 | 91 | hsa-miR-148a-3p | [RANGAP1](http://www.ncbi.nlm.nih.gov/entrez/query.fcgi?db=gene&cmd=Retrieve&dopt=full_report&list_uids=5905) | Ran GTPase activating protein 1 |
| [Details](http://mirdb.org/cgi-bin/target_detail.cgi?targetID=598641) | 162 | 90 | hsa-miR-148a-3p | [ADGRF5](http://www.ncbi.nlm.nih.gov/entrez/query.fcgi?db=gene&cmd=Retrieve&dopt=full_report&list_uids=221395) | adhesion G protein-coupled receptor F5 |
| [Details](http://mirdb.org/cgi-bin/target_detail.cgi?targetID=598448) | 163 | 90 | hsa-miR-148a-3p | [SNN](http://www.ncbi.nlm.nih.gov/entrez/query.fcgi?db=gene&cmd=Retrieve&dopt=full_report&list_uids=8303) | stannin |
| [Details](http://mirdb.org/cgi-bin/target_detail.cgi?targetID=598037) | 164 | 90 | hsa-miR-148a-3p | [PIK3C2A](http://www.ncbi.nlm.nih.gov/entrez/query.fcgi?db=gene&cmd=Retrieve&dopt=full_report&list_uids=5286) | phosphatidylinositol-4-phosphate 3-kinase catalytic subunit type 2 alpha |
| [Details](http://mirdb.org/cgi-bin/target_detail.cgi?targetID=598579) | 165 | 90 | hsa-miR-148a-3p | [SPTY2D1](http://www.ncbi.nlm.nih.gov/entrez/query.fcgi?db=gene&cmd=Retrieve&dopt=full_report&list_uids=144108) | SPT2 chromatin protein domain containing 1 |
| [Details](http://mirdb.org/cgi-bin/target_detail.cgi?targetID=598418) | 166 | 90 | hsa-miR-148a-3p | [MAP2K1](http://www.ncbi.nlm.nih.gov/entrez/query.fcgi?db=gene&cmd=Retrieve&dopt=full_report&list_uids=5604) | mitogen-activated protein kinase kinase 1 |
| [Details](http://mirdb.org/cgi-bin/target_detail.cgi?targetID=598673) | 167 | 90 | hsa-miR-148a-3p | [ZNF804A](http://www.ncbi.nlm.nih.gov/entrez/query.fcgi?db=gene&cmd=Retrieve&dopt=full_report&list_uids=91752) | zinc finger protein 804A |
| [Details](http://mirdb.org/cgi-bin/target_detail.cgi?targetID=598227) | 168 | 90 | hsa-miR-148a-3p | [EYA3](http://www.ncbi.nlm.nih.gov/entrez/query.fcgi?db=gene&cmd=Retrieve&dopt=full_report&list_uids=2140) | EYA transcriptional coactivator and phosphatase 3 |
| [Details](http://mirdb.org/cgi-bin/target_detail.cgi?targetID=598032) | 169 | 90 | hsa-miR-148a-3p | [EPN2](http://www.ncbi.nlm.nih.gov/entrez/query.fcgi?db=gene&cmd=Retrieve&dopt=full_report&list_uids=22905) | epsin 2 |
| [Details](http://mirdb.org/cgi-bin/target_detail.cgi?targetID=598290) | 170 | 90 | hsa-miR-148a-3p | [TANC1](http://www.ncbi.nlm.nih.gov/entrez/query.fcgi?db=gene&cmd=Retrieve&dopt=full_report&list_uids=85461) | tetratricopeptide repeat, ankyrin repeat and coiled-coil containing 1 |
| [Details](http://mirdb.org/cgi-bin/target_detail.cgi?targetID=598303) | 171 | 90 | hsa-miR-148a-3p | [YTHDC2](http://www.ncbi.nlm.nih.gov/entrez/query.fcgi?db=gene&cmd=Retrieve&dopt=full_report&list_uids=64848) | YTH domain containing 2 |
| [Details](http://mirdb.org/cgi-bin/target_detail.cgi?targetID=598257) | 172 | 90 | hsa-miR-148a-3p | [NPTX1](http://www.ncbi.nlm.nih.gov/entrez/query.fcgi?db=gene&cmd=Retrieve&dopt=full_report&list_uids=4884) | neuronal pentraxin 1 |
| [Details](http://mirdb.org/cgi-bin/target_detail.cgi?targetID=597958) | 173 | 90 | hsa-miR-148a-3p | [SYNJ1](http://www.ncbi.nlm.nih.gov/entrez/query.fcgi?db=gene&cmd=Retrieve&dopt=full_report&list_uids=8867) | synaptojanin 1 |
| [Details](http://mirdb.org/cgi-bin/target_detail.cgi?targetID=598133) | 174 | 90 | hsa-miR-148a-3p | [TXNIP](http://www.ncbi.nlm.nih.gov/entrez/query.fcgi?db=gene&cmd=Retrieve&dopt=full_report&list_uids=10628) | thioredoxin interacting protein |
| [Details](http://mirdb.org/cgi-bin/target_detail.cgi?targetID=598510) | 175 | 90 | hsa-miR-148a-3p | [RC3H1](http://www.ncbi.nlm.nih.gov/entrez/query.fcgi?db=gene&cmd=Retrieve&dopt=full_report&list_uids=149041) | ring finger and CCCH-type domains 1 |
| [Details](http://mirdb.org/cgi-bin/target_detail.cgi?targetID=598566) | 176 | 90 | hsa-miR-148a-3p | [PIGA](http://www.ncbi.nlm.nih.gov/entrez/query.fcgi?db=gene&cmd=Retrieve&dopt=full_report&list_uids=5277) | phosphatidylinositol glycan anchor biosynthesis class A |
| [Details](http://mirdb.org/cgi-bin/target_detail.cgi?targetID=597906) | 177 | 90 | hsa-miR-148a-3p | [ARHGEF12](http://www.ncbi.nlm.nih.gov/entrez/query.fcgi?db=gene&cmd=Retrieve&dopt=full_report&list_uids=23365) | Rho guanine nucleotide exchange factor 12 |
| [Details](http://mirdb.org/cgi-bin/target_detail.cgi?targetID=598401) | 178 | 90 | hsa-miR-148a-3p | [PRKCZ](http://www.ncbi.nlm.nih.gov/entrez/query.fcgi?db=gene&cmd=Retrieve&dopt=full_report&list_uids=5590) | protein kinase C zeta |
| [Details](http://mirdb.org/cgi-bin/target_detail.cgi?targetID=598279) | 179 | 90 | hsa-miR-148a-3p | [MTCL1](http://www.ncbi.nlm.nih.gov/entrez/query.fcgi?db=gene&cmd=Retrieve&dopt=full_report&list_uids=23255) | microtubule crosslinking factor 1 |
| [Details](http://mirdb.org/cgi-bin/target_detail.cgi?targetID=598675) | 180 | 90 | hsa-miR-148a-3p | [GPM6A](http://www.ncbi.nlm.nih.gov/entrez/query.fcgi?db=gene&cmd=Retrieve&dopt=full_report&list_uids=2823) | glycoprotein M6A |
| [Details](http://mirdb.org/cgi-bin/target_detail.cgi?targetID=598664) | 181 | 90 | hsa-miR-148a-3p | [AP4E1](http://www.ncbi.nlm.nih.gov/entrez/query.fcgi?db=gene&cmd=Retrieve&dopt=full_report&list_uids=23431) | adaptor related protein complex 4 subunit epsilon 1 |
| [Details](http://mirdb.org/cgi-bin/target_detail.cgi?targetID=597891) | 182 | 90 | hsa-miR-148a-3p | [CDK5R1](http://www.ncbi.nlm.nih.gov/entrez/query.fcgi?db=gene&cmd=Retrieve&dopt=full_report&list_uids=8851) | cyclin dependent kinase 5 regulatory subunit 1 |
| [Details](http://mirdb.org/cgi-bin/target_detail.cgi?targetID=598194) | 183 | 90 | hsa-miR-148a-3p | [ATP4B](http://www.ncbi.nlm.nih.gov/entrez/query.fcgi?db=gene&cmd=Retrieve&dopt=full_report&list_uids=496) | ATPase H+/K+ transporting subunit beta |
| [Details](http://mirdb.org/cgi-bin/target_detail.cgi?targetID=598069) | 184 | 89 | hsa-miR-148a-3p | [RICTOR](http://www.ncbi.nlm.nih.gov/entrez/query.fcgi?db=gene&cmd=Retrieve&dopt=full_report&list_uids=253260) | RPTOR independent companion of MTOR complex 2 |
| [Details](http://mirdb.org/cgi-bin/target_detail.cgi?targetID=598173) | 185 | 89 | hsa-miR-148a-3p | [TBL1XR1](http://www.ncbi.nlm.nih.gov/entrez/query.fcgi?db=gene&cmd=Retrieve&dopt=full_report&list_uids=79718) | transducin beta like 1 X-linked receptor 1 |
| [Details](http://mirdb.org/cgi-bin/target_detail.cgi?targetID=598358) | 186 | 89 | hsa-miR-148a-3p | [FAM234A](http://www.ncbi.nlm.nih.gov/entrez/query.fcgi?db=gene&cmd=Retrieve&dopt=full_report&list_uids=83986) | family with sequence similarity 234 member A |
| [Details](http://mirdb.org/cgi-bin/target_detail.cgi?targetID=598486) | 187 | 89 | hsa-miR-148a-3p | [C18orf25](http://www.ncbi.nlm.nih.gov/entrez/query.fcgi?db=gene&cmd=Retrieve&dopt=full_report&list_uids=147339) | chromosome 18 open reading frame 25 |
| [Details](http://mirdb.org/cgi-bin/target_detail.cgi?targetID=598102) | 188 | 89 | hsa-miR-148a-3p | [ITSN2](http://www.ncbi.nlm.nih.gov/entrez/query.fcgi?db=gene&cmd=Retrieve&dopt=full_report&list_uids=50618) | intersectin 2 |
| [Details](http://mirdb.org/cgi-bin/target_detail.cgi?targetID=598155) | 189 | 89 | hsa-miR-148a-3p | [EPS15](http://www.ncbi.nlm.nih.gov/entrez/query.fcgi?db=gene&cmd=Retrieve&dopt=full_report&list_uids=2060) | epidermal growth factor receptor pathway substrate 15 |
| [Details](http://mirdb.org/cgi-bin/target_detail.cgi?targetID=598229) | 190 | 89 | hsa-miR-148a-3p | [CELSR1](http://www.ncbi.nlm.nih.gov/entrez/query.fcgi?db=gene&cmd=Retrieve&dopt=full_report&list_uids=9620) | cadherin EGF LAG seven-pass G-type receptor 1 |
| [Details](http://mirdb.org/cgi-bin/target_detail.cgi?targetID=598141) | 191 | 89 | hsa-miR-148a-3p | [CBLL1](http://www.ncbi.nlm.nih.gov/entrez/query.fcgi?db=gene&cmd=Retrieve&dopt=full_report&list_uids=79872) | Cbl proto-oncogene like 1 |
| [Details](http://mirdb.org/cgi-bin/target_detail.cgi?targetID=598119) | 192 | 89 | hsa-miR-148a-3p | [ZBTB18](http://www.ncbi.nlm.nih.gov/entrez/query.fcgi?db=gene&cmd=Retrieve&dopt=full_report&list_uids=10472) | zinc finger and BTB domain containing 18 |
| [Details](http://mirdb.org/cgi-bin/target_detail.cgi?targetID=598380) | 193 | 89 | hsa-miR-148a-3p | [FMR1](http://www.ncbi.nlm.nih.gov/entrez/query.fcgi?db=gene&cmd=Retrieve&dopt=full_report&list_uids=2332) | fragile X mental retardation 1 |
| [Details](http://mirdb.org/cgi-bin/target_detail.cgi?targetID=597997) | 194 | 89 | hsa-miR-148a-3p | [PRICKLE2](http://www.ncbi.nlm.nih.gov/entrez/query.fcgi?db=gene&cmd=Retrieve&dopt=full_report&list_uids=166336) | prickle planar cell polarity protein 2 |
| [Details](http://mirdb.org/cgi-bin/target_detail.cgi?targetID=598613) | 195 | 89 | hsa-miR-148a-3p | [UBAP2L](http://www.ncbi.nlm.nih.gov/entrez/query.fcgi?db=gene&cmd=Retrieve&dopt=full_report&list_uids=9898) | ubiquitin associated protein 2 like |
| [Details](http://mirdb.org/cgi-bin/target_detail.cgi?targetID=597961) | 196 | 89 | hsa-miR-148a-3p | [CNTNAP3B](http://www.ncbi.nlm.nih.gov/entrez/query.fcgi?db=gene&cmd=Retrieve&dopt=full_report&list_uids=728577) | contactin associated protein like 3B |
| [Details](http://mirdb.org/cgi-bin/target_detail.cgi?targetID=597890) | 197 | 89 | hsa-miR-148a-3p | [MAFB](http://www.ncbi.nlm.nih.gov/entrez/query.fcgi?db=gene&cmd=Retrieve&dopt=full_report&list_uids=9935) | MAF bZIP transcription factor B |
| [Details](http://mirdb.org/cgi-bin/target_detail.cgi?targetID=598635) | 198 | 89 | hsa-miR-148a-3p | [GPATCH8](http://www.ncbi.nlm.nih.gov/entrez/query.fcgi?db=gene&cmd=Retrieve&dopt=full_report&list_uids=23131) | G-patch domain containing 8 |
| [Details](http://mirdb.org/cgi-bin/target_detail.cgi?targetID=597956) | 199 | 88 | hsa-miR-148a-3p | [TEK](http://www.ncbi.nlm.nih.gov/entrez/query.fcgi?db=gene&cmd=Retrieve&dopt=full_report&list_uids=7010) | TEK receptor tyrosine kinase |
| [Details](http://mirdb.org/cgi-bin/target_detail.cgi?targetID=597991) | 200 | 88 | hsa-miR-148a-3p | [CSF1](http://www.ncbi.nlm.nih.gov/entrez/query.fcgi?db=gene&cmd=Retrieve&dopt=full_report&list_uids=1435) | colony stimulating factor 1 |
| [Details](http://mirdb.org/cgi-bin/target_detail.cgi?targetID=597868) | 201 | 88 | hsa-miR-148a-3p | [ABCD3](http://www.ncbi.nlm.nih.gov/entrez/query.fcgi?db=gene&cmd=Retrieve&dopt=full_report&list_uids=5825) | ATP binding cassette subfamily D member 3 |
| [Details](http://mirdb.org/cgi-bin/target_detail.cgi?targetID=598101) | 202 | 88 | hsa-miR-148a-3p | [FAM161A](http://www.ncbi.nlm.nih.gov/entrez/query.fcgi?db=gene&cmd=Retrieve&dopt=full_report&list_uids=84140) | FAM161A, centrosomal protein |
| [Details](http://mirdb.org/cgi-bin/target_detail.cgi?targetID=597895) | 203 | 88 | hsa-miR-148a-3p | [SLC2A1](http://www.ncbi.nlm.nih.gov/entrez/query.fcgi?db=gene&cmd=Retrieve&dopt=full_report&list_uids=6513) | solute carrier family 2 member 1 |
| [Details](http://mirdb.org/cgi-bin/target_detail.cgi?targetID=598690) | 204 | 88 | hsa-miR-148a-3p | [PHACTR2](http://www.ncbi.nlm.nih.gov/entrez/query.fcgi?db=gene&cmd=Retrieve&dopt=full_report&list_uids=9749) | phosphatase and actin regulator 2 |
| [Details](http://mirdb.org/cgi-bin/target_detail.cgi?targetID=598001) | 205 | 88 | hsa-miR-148a-3p | [DLG2](http://www.ncbi.nlm.nih.gov/entrez/query.fcgi?db=gene&cmd=Retrieve&dopt=full_report&list_uids=1740) | discs large MAGUK scaffold protein 2 |
| [Details](http://mirdb.org/cgi-bin/target_detail.cgi?targetID=598442) | 206 | 88 | hsa-miR-148a-3p | [GAP43](http://www.ncbi.nlm.nih.gov/entrez/query.fcgi?db=gene&cmd=Retrieve&dopt=full_report&list_uids=2596) | growth associated protein 43 |
| [Details](http://mirdb.org/cgi-bin/target_detail.cgi?targetID=598098) | 207 | 88 | hsa-miR-148a-3p | [CASZ1](http://www.ncbi.nlm.nih.gov/entrez/query.fcgi?db=gene&cmd=Retrieve&dopt=full_report&list_uids=54897) | castor zinc finger 1 |
| [Details](http://mirdb.org/cgi-bin/target_detail.cgi?targetID=598149) | 208 | 88 | hsa-miR-148a-3p | [ARRDC3](http://www.ncbi.nlm.nih.gov/entrez/query.fcgi?db=gene&cmd=Retrieve&dopt=full_report&list_uids=57561) | arrestin domain containing 3 |
| [Details](http://mirdb.org/cgi-bin/target_detail.cgi?targetID=598086) | 209 | 88 | hsa-miR-148a-3p | [ELAVL2](http://www.ncbi.nlm.nih.gov/entrez/query.fcgi?db=gene&cmd=Retrieve&dopt=full_report&list_uids=1993) | ELAV like RNA binding protein 2 |
| [Details](http://mirdb.org/cgi-bin/target_detail.cgi?targetID=598582) | 210 | 88 | hsa-miR-148a-3p | [MIER1](http://www.ncbi.nlm.nih.gov/entrez/query.fcgi?db=gene&cmd=Retrieve&dopt=full_report&list_uids=57708) | MIER1 transcriptional regulator |
| [Details](http://mirdb.org/cgi-bin/target_detail.cgi?targetID=598625) | 211 | 88 | hsa-miR-148a-3p | [UBE4B](http://www.ncbi.nlm.nih.gov/entrez/query.fcgi?db=gene&cmd=Retrieve&dopt=full_report&list_uids=10277) | ubiquitination factor E4B |
| [Details](http://mirdb.org/cgi-bin/target_detail.cgi?targetID=598477) | 212 | 88 | hsa-miR-148a-3p | [ARF4](http://www.ncbi.nlm.nih.gov/entrez/query.fcgi?db=gene&cmd=Retrieve&dopt=full_report&list_uids=378) | ADP ribosylation factor 4 |
| [Details](http://mirdb.org/cgi-bin/target_detail.cgi?targetID=597986) | 213 | 88 | hsa-miR-148a-3p | [ADAMTS15](http://www.ncbi.nlm.nih.gov/entrez/query.fcgi?db=gene&cmd=Retrieve&dopt=full_report&list_uids=170689) | ADAM metallopeptidase with thrombospondin type 1 motif 15 |
| [Details](http://mirdb.org/cgi-bin/target_detail.cgi?targetID=598642) | 214 | 88 | hsa-miR-148a-3p | [RNF219](http://www.ncbi.nlm.nih.gov/entrez/query.fcgi?db=gene&cmd=Retrieve&dopt=full_report&list_uids=79596) | ring finger protein 219 |
| [Details](http://mirdb.org/cgi-bin/target_detail.cgi?targetID=597907) | 215 | 88 | hsa-miR-148a-3p | [UCP3](http://www.ncbi.nlm.nih.gov/entrez/query.fcgi?db=gene&cmd=Retrieve&dopt=full_report&list_uids=7352) | uncoupling protein 3 |
| [Details](http://mirdb.org/cgi-bin/target_detail.cgi?targetID=598621) | 216 | 88 | hsa-miR-148a-3p | [VMP1](http://www.ncbi.nlm.nih.gov/entrez/query.fcgi?db=gene&cmd=Retrieve&dopt=full_report&list_uids=81671) | vacuole membrane protein 1 |
| [Details](http://mirdb.org/cgi-bin/target_detail.cgi?targetID=597979) | 217 | 88 | hsa-miR-148a-3p | [TGFB2](http://www.ncbi.nlm.nih.gov/entrez/query.fcgi?db=gene&cmd=Retrieve&dopt=full_report&list_uids=7042) | transforming growth factor beta 2 |
| [Details](http://mirdb.org/cgi-bin/target_detail.cgi?targetID=598650) | 218 | 88 | hsa-miR-148a-3p | [CNTN4](http://www.ncbi.nlm.nih.gov/entrez/query.fcgi?db=gene&cmd=Retrieve&dopt=full_report&list_uids=152330) | contactin 4 |
| [Details](http://mirdb.org/cgi-bin/target_detail.cgi?targetID=598110) | 219 | 87 | hsa-miR-148a-3p | [ITGA5](http://www.ncbi.nlm.nih.gov/entrez/query.fcgi?db=gene&cmd=Retrieve&dopt=full_report&list_uids=3678) | integrin subunit alpha 5 |
| [Details](http://mirdb.org/cgi-bin/target_detail.cgi?targetID=597884) | 220 | 87 | hsa-miR-148a-3p | [MLLT10](http://www.ncbi.nlm.nih.gov/entrez/query.fcgi?db=gene&cmd=Retrieve&dopt=full_report&list_uids=8028) | MLLT10, histone lysine methyltransferase DOT1L cofactor |
| [Details](http://mirdb.org/cgi-bin/target_detail.cgi?targetID=598136) | 221 | 87 | hsa-miR-148a-3p | [ALCAM](http://www.ncbi.nlm.nih.gov/entrez/query.fcgi?db=gene&cmd=Retrieve&dopt=full_report&list_uids=214) | activated leukocyte cell adhesion molecule |
| [Details](http://mirdb.org/cgi-bin/target_detail.cgi?targetID=598366) | 222 | 87 | hsa-miR-148a-3p | [MTMR9](http://www.ncbi.nlm.nih.gov/entrez/query.fcgi?db=gene&cmd=Retrieve&dopt=full_report&list_uids=66036) | myotubularin related protein 9 |
| [Details](http://mirdb.org/cgi-bin/target_detail.cgi?targetID=598422) | 223 | 87 | hsa-miR-148a-3p | [NCOA1](http://www.ncbi.nlm.nih.gov/entrez/query.fcgi?db=gene&cmd=Retrieve&dopt=full_report&list_uids=8648) | nuclear receptor coactivator 1 |
| [Details](http://mirdb.org/cgi-bin/target_detail.cgi?targetID=598359) | 224 | 87 | hsa-miR-148a-3p | [RGMA](http://www.ncbi.nlm.nih.gov/entrez/query.fcgi?db=gene&cmd=Retrieve&dopt=full_report&list_uids=56963) | repulsive guidance molecule BMP co-receptor a |
| [Details](http://mirdb.org/cgi-bin/target_detail.cgi?targetID=598293) | 225 | 87 | hsa-miR-148a-3p | [NRARP](http://www.ncbi.nlm.nih.gov/entrez/query.fcgi?db=gene&cmd=Retrieve&dopt=full_report&list_uids=441478) | NOTCH regulated ankyrin repeat protein |
| [Details](http://mirdb.org/cgi-bin/target_detail.cgi?targetID=598239) | 226 | 87 | hsa-miR-148a-3p | [KLF5](http://www.ncbi.nlm.nih.gov/entrez/query.fcgi?db=gene&cmd=Retrieve&dopt=full_report&list_uids=688) | Kruppel like factor 5 |
| [Details](http://mirdb.org/cgi-bin/target_detail.cgi?targetID=598247) | 227 | 87 | hsa-miR-148a-3p | [LTBP1](http://www.ncbi.nlm.nih.gov/entrez/query.fcgi?db=gene&cmd=Retrieve&dopt=full_report&list_uids=4052) | latent transforming growth factor beta binding protein 1 |
| [Details](http://mirdb.org/cgi-bin/target_detail.cgi?targetID=598329) | 228 | 87 | hsa-miR-148a-3p | [CUL5](http://www.ncbi.nlm.nih.gov/entrez/query.fcgi?db=gene&cmd=Retrieve&dopt=full_report&list_uids=8065) | cullin 5 |
| [Details](http://mirdb.org/cgi-bin/target_detail.cgi?targetID=597990) | 229 | 87 | hsa-miR-148a-3p | [MDFIC](http://www.ncbi.nlm.nih.gov/entrez/query.fcgi?db=gene&cmd=Retrieve&dopt=full_report&list_uids=29969) | MyoD family inhibitor domain containing |
| [Details](http://mirdb.org/cgi-bin/target_detail.cgi?targetID=597925) | 230 | 87 | hsa-miR-148a-3p | [ADAM23](http://www.ncbi.nlm.nih.gov/entrez/query.fcgi?db=gene&cmd=Retrieve&dopt=full_report&list_uids=8745) | ADAM metallopeptidase domain 23 |
| [Details](http://mirdb.org/cgi-bin/target_detail.cgi?targetID=598214) | 231 | 87 | hsa-miR-148a-3p | [CCDC141](http://www.ncbi.nlm.nih.gov/entrez/query.fcgi?db=gene&cmd=Retrieve&dopt=full_report&list_uids=285025) | coiled-coil domain containing 141 |
| [Details](http://mirdb.org/cgi-bin/target_detail.cgi?targetID=598554) | 232 | 87 | hsa-miR-148a-3p | [ELAVL4](http://www.ncbi.nlm.nih.gov/entrez/query.fcgi?db=gene&cmd=Retrieve&dopt=full_report&list_uids=1996) | ELAV like RNA binding protein 4 |
| [Details](http://mirdb.org/cgi-bin/target_detail.cgi?targetID=598564) | 233 | 87 | hsa-miR-148a-3p | [MTMR10](http://www.ncbi.nlm.nih.gov/entrez/query.fcgi?db=gene&cmd=Retrieve&dopt=full_report&list_uids=54893) | myotubularin related protein 10 |
| [Details](http://mirdb.org/cgi-bin/target_detail.cgi?targetID=598171) | 234 | 87 | hsa-miR-148a-3p | [TOMM70](http://www.ncbi.nlm.nih.gov/entrez/query.fcgi?db=gene&cmd=Retrieve&dopt=full_report&list_uids=9868) | translocase of outer mitochondrial membrane 70 |
| [Details](http://mirdb.org/cgi-bin/target_detail.cgi?targetID=598571) | 235 | 87 | hsa-miR-148a-3p | [FEZ2](http://www.ncbi.nlm.nih.gov/entrez/query.fcgi?db=gene&cmd=Retrieve&dopt=full_report&list_uids=9637) | fasciculation and elongation protein zeta 2 |
| [Details](http://mirdb.org/cgi-bin/target_detail.cgi?targetID=598049) | 236 | 87 | hsa-miR-148a-3p | [AGO1](http://www.ncbi.nlm.nih.gov/entrez/query.fcgi?db=gene&cmd=Retrieve&dopt=full_report&list_uids=26523) | argonaute RISC catalytic component 1 |
| [Details](http://mirdb.org/cgi-bin/target_detail.cgi?targetID=598319) | 237 | 87 | hsa-miR-148a-3p | [USP31](http://www.ncbi.nlm.nih.gov/entrez/query.fcgi?db=gene&cmd=Retrieve&dopt=full_report&list_uids=57478) | ubiquitin specific peptidase 31 |
| [Details](http://mirdb.org/cgi-bin/target_detail.cgi?targetID=598012) | 238 | 87 | hsa-miR-148a-3p | [TMEM54](http://www.ncbi.nlm.nih.gov/entrez/query.fcgi?db=gene&cmd=Retrieve&dopt=full_report&list_uids=113452) | transmembrane protein 54 |
| [Details](http://mirdb.org/cgi-bin/target_detail.cgi?targetID=598585) | 239 | 87 | hsa-miR-148a-3p | [ARFIP1](http://www.ncbi.nlm.nih.gov/entrez/query.fcgi?db=gene&cmd=Retrieve&dopt=full_report&list_uids=27236) | ADP ribosylation factor interacting protein 1 |
| [Details](http://mirdb.org/cgi-bin/target_detail.cgi?targetID=598129) | 240 | 86 | hsa-miR-148a-3p | [CD72](http://www.ncbi.nlm.nih.gov/entrez/query.fcgi?db=gene&cmd=Retrieve&dopt=full_report&list_uids=971) | CD72 molecule |
| [Details](http://mirdb.org/cgi-bin/target_detail.cgi?targetID=598684) | 241 | 86 | hsa-miR-148a-3p | [FOXF1](http://www.ncbi.nlm.nih.gov/entrez/query.fcgi?db=gene&cmd=Retrieve&dopt=full_report&list_uids=2294) | forkhead box F1 |
| [Details](http://mirdb.org/cgi-bin/target_detail.cgi?targetID=598168) | 242 | 86 | hsa-miR-148a-3p | [H2AFY](http://www.ncbi.nlm.nih.gov/entrez/query.fcgi?db=gene&cmd=Retrieve&dopt=full_report&list_uids=9555) | H2A histone family member Y |
| [Details](http://mirdb.org/cgi-bin/target_detail.cgi?targetID=598065) | 243 | 86 | hsa-miR-148a-3p | [MLLT6](http://www.ncbi.nlm.nih.gov/entrez/query.fcgi?db=gene&cmd=Retrieve&dopt=full_report&list_uids=4302) | MLLT6, PHD finger containing |
| [Details](http://mirdb.org/cgi-bin/target_detail.cgi?targetID=598574) | 244 | 86 | hsa-miR-148a-3p | [NAT14](http://www.ncbi.nlm.nih.gov/entrez/query.fcgi?db=gene&cmd=Retrieve&dopt=full_report&list_uids=57106) | N-acetyltransferase 14 (putative) |
| [Details](http://mirdb.org/cgi-bin/target_detail.cgi?targetID=598128) | 245 | 86 | hsa-miR-148a-3p | [ITGA9](http://www.ncbi.nlm.nih.gov/entrez/query.fcgi?db=gene&cmd=Retrieve&dopt=full_report&list_uids=3680) | integrin subunit alpha 9 |
| [Details](http://mirdb.org/cgi-bin/target_detail.cgi?targetID=598046) | 246 | 86 | hsa-miR-148a-3p | [MOSPD1](http://www.ncbi.nlm.nih.gov/entrez/query.fcgi?db=gene&cmd=Retrieve&dopt=full_report&list_uids=56180) | motile sperm domain containing 1 |
| [Details](http://mirdb.org/cgi-bin/target_detail.cgi?targetID=597881) | 247 | 86 | hsa-miR-148a-3p | [EPAS1](http://www.ncbi.nlm.nih.gov/entrez/query.fcgi?db=gene&cmd=Retrieve&dopt=full_report&list_uids=2034) | endothelial PAS domain protein 1 |
| [Details](http://mirdb.org/cgi-bin/target_detail.cgi?targetID=597934) | 248 | 86 | hsa-miR-148a-3p | [NOG](http://www.ncbi.nlm.nih.gov/entrez/query.fcgi?db=gene&cmd=Retrieve&dopt=full_report&list_uids=9241) | noggin |
| [Details](http://mirdb.org/cgi-bin/target_detail.cgi?targetID=598671) | 249 | 86 | hsa-miR-148a-3p | [LRRC41](http://www.ncbi.nlm.nih.gov/entrez/query.fcgi?db=gene&cmd=Retrieve&dopt=full_report&list_uids=10489) | leucine rich repeat containing 41 |
| [Details](http://mirdb.org/cgi-bin/target_detail.cgi?targetID=597933) | 250 | 86 | hsa-miR-148a-3p | [CDKL5](http://www.ncbi.nlm.nih.gov/entrez/query.fcgi?db=gene&cmd=Retrieve&dopt=full_report&list_uids=6792) | cyclin dependent kinase like 5 |
| [Details](http://mirdb.org/cgi-bin/target_detail.cgi?targetID=598248) | 251 | 86 | hsa-miR-148a-3p | [USP48](http://www.ncbi.nlm.nih.gov/entrez/query.fcgi?db=gene&cmd=Retrieve&dopt=full_report&list_uids=84196) | ubiquitin specific peptidase 48 |
| [Details](http://mirdb.org/cgi-bin/target_detail.cgi?targetID=597903) | 252 | 86 | hsa-miR-148a-3p | [SCN9A](http://www.ncbi.nlm.nih.gov/entrez/query.fcgi?db=gene&cmd=Retrieve&dopt=full_report&list_uids=6335) | sodium voltage-gated channel alpha subunit 9 |
| [Details](http://mirdb.org/cgi-bin/target_detail.cgi?targetID=598372) | 253 | 86 | hsa-miR-148a-3p | [CANX](http://www.ncbi.nlm.nih.gov/entrez/query.fcgi?db=gene&cmd=Retrieve&dopt=full_report&list_uids=821) | calnexin |
| [Details](http://mirdb.org/cgi-bin/target_detail.cgi?targetID=598534) | 254 | 86 | hsa-miR-148a-3p | [MAP3K4](http://www.ncbi.nlm.nih.gov/entrez/query.fcgi?db=gene&cmd=Retrieve&dopt=full_report&list_uids=4216) | mitogen-activated protein kinase kinase kinase 4 |
| [Details](http://mirdb.org/cgi-bin/target_detail.cgi?targetID=598200) | 255 | 86 | hsa-miR-148a-3p | [SSR1](http://www.ncbi.nlm.nih.gov/entrez/query.fcgi?db=gene&cmd=Retrieve&dopt=full_report&list_uids=6745) | signal sequence receptor subunit 1 |
| [Details](http://mirdb.org/cgi-bin/target_detail.cgi?targetID=597944) | 256 | 86 | hsa-miR-148a-3p | [MRAS](http://www.ncbi.nlm.nih.gov/entrez/query.fcgi?db=gene&cmd=Retrieve&dopt=full_report&list_uids=22808) | muscle RAS oncogene homolog |
| [Details](http://mirdb.org/cgi-bin/target_detail.cgi?targetID=598666) | 257 | 86 | hsa-miR-148a-3p | [KDM7A](http://www.ncbi.nlm.nih.gov/entrez/query.fcgi?db=gene&cmd=Retrieve&dopt=full_report&list_uids=80853) | lysine demethylase 7A |
| [Details](http://mirdb.org/cgi-bin/target_detail.cgi?targetID=598545) | 258 | 86 | hsa-miR-148a-3p | [USP32](http://www.ncbi.nlm.nih.gov/entrez/query.fcgi?db=gene&cmd=Retrieve&dopt=full_report&list_uids=84669) | ubiquitin specific peptidase 32 |
| [Details](http://mirdb.org/cgi-bin/target_detail.cgi?targetID=598387) | 259 | 86 | hsa-miR-148a-3p | [NT5C3A](http://www.ncbi.nlm.nih.gov/entrez/query.fcgi?db=gene&cmd=Retrieve&dopt=full_report&list_uids=51251) | 5'-nucleotidase, cytosolic IIIA |
| [Details](http://mirdb.org/cgi-bin/target_detail.cgi?targetID=598278) | 260 | 85 | hsa-miR-148a-3p | [TGOLN2](http://www.ncbi.nlm.nih.gov/entrez/query.fcgi?db=gene&cmd=Retrieve&dopt=full_report&list_uids=10618) | trans-golgi network protein 2 |
| [Details](http://mirdb.org/cgi-bin/target_detail.cgi?targetID=598259) | 261 | 85 | hsa-miR-148a-3p | [ING2](http://www.ncbi.nlm.nih.gov/entrez/query.fcgi?db=gene&cmd=Retrieve&dopt=full_report&list_uids=3622) | inhibitor of growth family member 2 |
| [Details](http://mirdb.org/cgi-bin/target_detail.cgi?targetID=598511) | 262 | 85 | hsa-miR-148a-3p | [WDR20](http://www.ncbi.nlm.nih.gov/entrez/query.fcgi?db=gene&cmd=Retrieve&dopt=full_report&list_uids=91833) | WD repeat domain 20 |
| [Details](http://mirdb.org/cgi-bin/target_detail.cgi?targetID=598172) | 263 | 85 | hsa-miR-148a-3p | [WNT10B](http://www.ncbi.nlm.nih.gov/entrez/query.fcgi?db=gene&cmd=Retrieve&dopt=full_report&list_uids=7480) | Wnt family member 10B |
| [Details](http://mirdb.org/cgi-bin/target_detail.cgi?targetID=597982) | 264 | 85 | hsa-miR-148a-3p | [MTMR14](http://www.ncbi.nlm.nih.gov/entrez/query.fcgi?db=gene&cmd=Retrieve&dopt=full_report&list_uids=64419) | myotubularin related protein 14 |
| [Details](http://mirdb.org/cgi-bin/target_detail.cgi?targetID=598459) | 265 | 85 | hsa-miR-148a-3p | [ROCK1](http://www.ncbi.nlm.nih.gov/entrez/query.fcgi?db=gene&cmd=Retrieve&dopt=full_report&list_uids=6093) | Rho associated coiled-coil containing protein kinase 1 |
| [Details](http://mirdb.org/cgi-bin/target_detail.cgi?targetID=598346) | 266 | 85 | hsa-miR-148a-3p | [CERS6](http://www.ncbi.nlm.nih.gov/entrez/query.fcgi?db=gene&cmd=Retrieve&dopt=full_report&list_uids=253782) | ceramide synthase 6 |
| [Details](http://mirdb.org/cgi-bin/target_detail.cgi?targetID=598444) | 267 | 85 | hsa-miR-148a-3p | [MMD](http://www.ncbi.nlm.nih.gov/entrez/query.fcgi?db=gene&cmd=Retrieve&dopt=full_report&list_uids=23531) | monocyte to macrophage differentiation associated |
| [Details](http://mirdb.org/cgi-bin/target_detail.cgi?targetID=598532) | 268 | 85 | hsa-miR-148a-3p | [CAMSAP2](http://www.ncbi.nlm.nih.gov/entrez/query.fcgi?db=gene&cmd=Retrieve&dopt=full_report&list_uids=23271) | calmodulin regulated spectrin associated protein family member 2 |
| [Details](http://mirdb.org/cgi-bin/target_detail.cgi?targetID=598680) | 269 | 85 | hsa-miR-148a-3p | [IL15](http://www.ncbi.nlm.nih.gov/entrez/query.fcgi?db=gene&cmd=Retrieve&dopt=full_report&list_uids=3600) | interleukin 15 |
| [Details](http://mirdb.org/cgi-bin/target_detail.cgi?targetID=598245) | 270 | 85 | hsa-miR-148a-3p | [CLTA](http://www.ncbi.nlm.nih.gov/entrez/query.fcgi?db=gene&cmd=Retrieve&dopt=full_report&list_uids=1211) | clathrin light chain A |
| [Details](http://mirdb.org/cgi-bin/target_detail.cgi?targetID=598286) | 271 | 85 | hsa-miR-148a-3p | [MNT](http://www.ncbi.nlm.nih.gov/entrez/query.fcgi?db=gene&cmd=Retrieve&dopt=full_report&list_uids=4335) | MAX network transcriptional repressor |
| [Details](http://mirdb.org/cgi-bin/target_detail.cgi?targetID=597975) | 272 | 85 | hsa-miR-148a-3p | [NOL4L](http://www.ncbi.nlm.nih.gov/entrez/query.fcgi?db=gene&cmd=Retrieve&dopt=full_report&list_uids=140688) | nucleolar protein 4 like |
| [Details](http://mirdb.org/cgi-bin/target_detail.cgi?targetID=598240) | 273 | 85 | hsa-miR-148a-3p | [SIX4](http://www.ncbi.nlm.nih.gov/entrez/query.fcgi?db=gene&cmd=Retrieve&dopt=full_report&list_uids=51804) | SIX homeobox 4 |
| [Details](http://mirdb.org/cgi-bin/target_detail.cgi?targetID=598560) | 274 | 85 | hsa-miR-148a-3p | [MMP13](http://www.ncbi.nlm.nih.gov/entrez/query.fcgi?db=gene&cmd=Retrieve&dopt=full_report&list_uids=4322) | matrix metallopeptidase 13 |
| [Details](http://mirdb.org/cgi-bin/target_detail.cgi?targetID=598610) | 275 | 84 | hsa-miR-148a-3p | [CFL2](http://www.ncbi.nlm.nih.gov/entrez/query.fcgi?db=gene&cmd=Retrieve&dopt=full_report&list_uids=1073) | cofilin 2 |
| [Details](http://mirdb.org/cgi-bin/target_detail.cgi?targetID=598164) | 276 | 84 | hsa-miR-148a-3p | [PGRMC2](http://www.ncbi.nlm.nih.gov/entrez/query.fcgi?db=gene&cmd=Retrieve&dopt=full_report&list_uids=10424) | progesterone receptor membrane component 2 |
| [Details](http://mirdb.org/cgi-bin/target_detail.cgi?targetID=598504) | 277 | 84 | hsa-miR-148a-3p | [MITF](http://www.ncbi.nlm.nih.gov/entrez/query.fcgi?db=gene&cmd=Retrieve&dopt=full_report&list_uids=4286) | melanocyte inducing transcription factor |
| [Details](http://mirdb.org/cgi-bin/target_detail.cgi?targetID=597954) | 278 | 84 | hsa-miR-148a-3p | [PEAK1](http://www.ncbi.nlm.nih.gov/entrez/query.fcgi?db=gene&cmd=Retrieve&dopt=full_report&list_uids=79834) | pseudopodium enriched atypical kinase 1 |
| [Details](http://mirdb.org/cgi-bin/target_detail.cgi?targetID=597892) | 279 | 84 | hsa-miR-148a-3p | [BTBD10](http://www.ncbi.nlm.nih.gov/entrez/query.fcgi?db=gene&cmd=Retrieve&dopt=full_report&list_uids=84280) | BTB domain containing 10 |
| [Details](http://mirdb.org/cgi-bin/target_detail.cgi?targetID=598163) | 280 | 84 | hsa-miR-148a-3p | [NOL4](http://www.ncbi.nlm.nih.gov/entrez/query.fcgi?db=gene&cmd=Retrieve&dopt=full_report&list_uids=8715) | nucleolar protein 4 |
| [Details](http://mirdb.org/cgi-bin/target_detail.cgi?targetID=598398) | 281 | 84 | hsa-miR-148a-3p | [ZCCHC2](http://www.ncbi.nlm.nih.gov/entrez/query.fcgi?db=gene&cmd=Retrieve&dopt=full_report&list_uids=54877) | zinc finger CCHC-type containing 2 |
| [Details](http://mirdb.org/cgi-bin/target_detail.cgi?targetID=598014) | 282 | 84 | hsa-miR-148a-3p | [CHUK](http://www.ncbi.nlm.nih.gov/entrez/query.fcgi?db=gene&cmd=Retrieve&dopt=full_report&list_uids=1147) | conserved helix-loop-helix ubiquitous kinase |
| [Details](http://mirdb.org/cgi-bin/target_detail.cgi?targetID=598016) | 283 | 84 | hsa-miR-148a-3p | [CHD7](http://www.ncbi.nlm.nih.gov/entrez/query.fcgi?db=gene&cmd=Retrieve&dopt=full_report&list_uids=55636) | chromodomain helicase DNA binding protein 7 |
| [Details](http://mirdb.org/cgi-bin/target_detail.cgi?targetID=598515) | 284 | 84 | hsa-miR-148a-3p | [TRIM59](http://www.ncbi.nlm.nih.gov/entrez/query.fcgi?db=gene&cmd=Retrieve&dopt=full_report&list_uids=286827) | tripartite motif containing 59 |
| [Details](http://mirdb.org/cgi-bin/target_detail.cgi?targetID=598524) | 285 | 84 | hsa-miR-148a-3p | [KLF4](http://www.ncbi.nlm.nih.gov/entrez/query.fcgi?db=gene&cmd=Retrieve&dopt=full_report&list_uids=9314) | Kruppel like factor 4 |
| [Details](http://mirdb.org/cgi-bin/target_detail.cgi?targetID=598357) | 286 | 84 | hsa-miR-148a-3p | [GPR137C](http://www.ncbi.nlm.nih.gov/entrez/query.fcgi?db=gene&cmd=Retrieve&dopt=full_report&list_uids=283554) | G protein-coupled receptor 137C |
| [Details](http://mirdb.org/cgi-bin/target_detail.cgi?targetID=598011) | 287 | 84 | hsa-miR-148a-3p | [PRKAG2](http://www.ncbi.nlm.nih.gov/entrez/query.fcgi?db=gene&cmd=Retrieve&dopt=full_report&list_uids=51422) | protein kinase AMP-activated non-catalytic subunit gamma 2 |
| [Details](http://mirdb.org/cgi-bin/target_detail.cgi?targetID=597916) | 288 | 84 | hsa-miR-148a-3p | [TRAK2](http://www.ncbi.nlm.nih.gov/entrez/query.fcgi?db=gene&cmd=Retrieve&dopt=full_report&list_uids=66008) | trafficking kinesin protein 2 |
| [Details](http://mirdb.org/cgi-bin/target_detail.cgi?targetID=598231) | 289 | 84 | hsa-miR-148a-3p | [SLC16A6](http://www.ncbi.nlm.nih.gov/entrez/query.fcgi?db=gene&cmd=Retrieve&dopt=full_report&list_uids=9120) | solute carrier family 16 member 6 |
| [Details](http://mirdb.org/cgi-bin/target_detail.cgi?targetID=598580) | 290 | 83 | hsa-miR-148a-3p | [PPP1R9B](http://www.ncbi.nlm.nih.gov/entrez/query.fcgi?db=gene&cmd=Retrieve&dopt=full_report&list_uids=84687) | protein phosphatase 1 regulatory subunit 9B |
| [Details](http://mirdb.org/cgi-bin/target_detail.cgi?targetID=598268) | 291 | 83 | hsa-miR-148a-3p | [ADAM17](http://www.ncbi.nlm.nih.gov/entrez/query.fcgi?db=gene&cmd=Retrieve&dopt=full_report&list_uids=6868) | ADAM metallopeptidase domain 17 |
| [Details](http://mirdb.org/cgi-bin/target_detail.cgi?targetID=598013) | 292 | 83 | hsa-miR-148a-3p | [IGFBP5](http://www.ncbi.nlm.nih.gov/entrez/query.fcgi?db=gene&cmd=Retrieve&dopt=full_report&list_uids=3488) | insulin like growth factor binding protein 5 |
| [Details](http://mirdb.org/cgi-bin/target_detail.cgi?targetID=598627) | 293 | 83 | hsa-miR-148a-3p | [CCKBR](http://www.ncbi.nlm.nih.gov/entrez/query.fcgi?db=gene&cmd=Retrieve&dopt=full_report&list_uids=887) | cholecystokinin B receptor |
| [Details](http://mirdb.org/cgi-bin/target_detail.cgi?targetID=597867) | 294 | 83 | hsa-miR-148a-3p | [NME7](http://www.ncbi.nlm.nih.gov/entrez/query.fcgi?db=gene&cmd=Retrieve&dopt=full_report&list_uids=29922) | NME/NM23 family member 7 |
| [Details](http://mirdb.org/cgi-bin/target_detail.cgi?targetID=597866) | 295 | 83 | hsa-miR-148a-3p | [CIITA](http://www.ncbi.nlm.nih.gov/entrez/query.fcgi?db=gene&cmd=Retrieve&dopt=full_report&list_uids=4261) | class II major histocompatibility complex transactivator |
| [Details](http://mirdb.org/cgi-bin/target_detail.cgi?targetID=598084) | 296 | 83 | hsa-miR-148a-3p | [ATP2B4](http://www.ncbi.nlm.nih.gov/entrez/query.fcgi?db=gene&cmd=Retrieve&dopt=full_report&list_uids=493) | ATPase plasma membrane Ca2+ transporting 4 |
| [Details](http://mirdb.org/cgi-bin/target_detail.cgi?targetID=597904) | 297 | 83 | hsa-miR-148a-3p | [DUSP1](http://www.ncbi.nlm.nih.gov/entrez/query.fcgi?db=gene&cmd=Retrieve&dopt=full_report&list_uids=1843) | dual specificity phosphatase 1 |
| [Details](http://mirdb.org/cgi-bin/target_detail.cgi?targetID=598590) | 298 | 83 | hsa-miR-148a-3p | [STOX2](http://www.ncbi.nlm.nih.gov/entrez/query.fcgi?db=gene&cmd=Retrieve&dopt=full_report&list_uids=56977) | storkhead box 2 |
| [Details](http://mirdb.org/cgi-bin/target_detail.cgi?targetID=598338) | 299 | 83 | hsa-miR-148a-3p | [ANXA4](http://www.ncbi.nlm.nih.gov/entrez/query.fcgi?db=gene&cmd=Retrieve&dopt=full_report&list_uids=307) | annexin A4 |
| [Details](http://mirdb.org/cgi-bin/target_detail.cgi?targetID=598438) | 300 | 83 | hsa-miR-148a-3p | [EFNB2](http://www.ncbi.nlm.nih.gov/entrez/query.fcgi?db=gene&cmd=Retrieve&dopt=full_report&list_uids=1948) | ephrin B2 |
| [Details](http://mirdb.org/cgi-bin/target_detail.cgi?targetID=598429) | 301 | 83 | hsa-miR-148a-3p | [ATP8A1](http://www.ncbi.nlm.nih.gov/entrez/query.fcgi?db=gene&cmd=Retrieve&dopt=full_report&list_uids=10396) | ATPase phospholipid transporting 8A1 |
| [Details](http://mirdb.org/cgi-bin/target_detail.cgi?targetID=598600) | 302 | 83 | hsa-miR-148a-3p | [KRTAP2-3](http://www.ncbi.nlm.nih.gov/entrez/query.fcgi?db=gene&cmd=Retrieve&dopt=full_report&list_uids=730755) | keratin associated protein 2-3 |
| [Details](http://mirdb.org/cgi-bin/target_detail.cgi?targetID=598042) | 303 | 83 | hsa-miR-148a-3p | [ITPK1](http://www.ncbi.nlm.nih.gov/entrez/query.fcgi?db=gene&cmd=Retrieve&dopt=full_report&list_uids=3705) | inositol-tetrakisphosphate 1-kinase |
| [Details](http://mirdb.org/cgi-bin/target_detail.cgi?targetID=598638) | 304 | 83 | hsa-miR-148a-3p | [OTUD4](http://www.ncbi.nlm.nih.gov/entrez/query.fcgi?db=gene&cmd=Retrieve&dopt=full_report&list_uids=54726) | OTU deubiquitinase 4 |
| [Details](http://mirdb.org/cgi-bin/target_detail.cgi?targetID=598417) | 305 | 83 | hsa-miR-148a-3p | [PDE1C](http://www.ncbi.nlm.nih.gov/entrez/query.fcgi?db=gene&cmd=Retrieve&dopt=full_report&list_uids=5137) | phosphodiesterase 1C |
| [Details](http://mirdb.org/cgi-bin/target_detail.cgi?targetID=597921) | 306 | 82 | hsa-miR-148a-3p | [EPM2A](http://www.ncbi.nlm.nih.gov/entrez/query.fcgi?db=gene&cmd=Retrieve&dopt=full_report&list_uids=7957) | EPM2A, laforin glucan phosphatase |
| [Details](http://mirdb.org/cgi-bin/target_detail.cgi?targetID=598430) | 307 | 82 | hsa-miR-148a-3p | [SESN3](http://www.ncbi.nlm.nih.gov/entrez/query.fcgi?db=gene&cmd=Retrieve&dopt=full_report&list_uids=143686) | sestrin 3 |
| [Details](http://mirdb.org/cgi-bin/target_detail.cgi?targetID=598124) | 308 | 82 | hsa-miR-148a-3p | [PRKAA1](http://www.ncbi.nlm.nih.gov/entrez/query.fcgi?db=gene&cmd=Retrieve&dopt=full_report&list_uids=5562) | protein kinase AMP-activated catalytic subunit alpha 1 |
| [Details](http://mirdb.org/cgi-bin/target_detail.cgi?targetID=598581) | 309 | 82 | hsa-miR-148a-3p | [ZNF821](http://www.ncbi.nlm.nih.gov/entrez/query.fcgi?db=gene&cmd=Retrieve&dopt=full_report&list_uids=55565) | zinc finger protein 821 |
| [Details](http://mirdb.org/cgi-bin/target_detail.cgi?targetID=598267) | 310 | 82 | hsa-miR-148a-3p | [MPL](http://www.ncbi.nlm.nih.gov/entrez/query.fcgi?db=gene&cmd=Retrieve&dopt=full_report&list_uids=4352) | MPL proto-oncogene, thrombopoietin receptor |
| [Details](http://mirdb.org/cgi-bin/target_detail.cgi?targetID=598315) | 311 | 82 | hsa-miR-148a-3p | [RUNX2](http://www.ncbi.nlm.nih.gov/entrez/query.fcgi?db=gene&cmd=Retrieve&dopt=full_report&list_uids=860) | runt related transcription factor 2 |
| [Details](http://mirdb.org/cgi-bin/target_detail.cgi?targetID=598665) | 312 | 82 | hsa-miR-148a-3p | [RTN4](http://www.ncbi.nlm.nih.gov/entrez/query.fcgi?db=gene&cmd=Retrieve&dopt=full_report&list_uids=57142) | reticulon 4 |
| [Details](http://mirdb.org/cgi-bin/target_detail.cgi?targetID=598251) | 313 | 82 | hsa-miR-148a-3p | [DYRK2](http://www.ncbi.nlm.nih.gov/entrez/query.fcgi?db=gene&cmd=Retrieve&dopt=full_report&list_uids=8445) | dual specificity tyrosine phosphorylation regulated kinase 2 |
| [Details](http://mirdb.org/cgi-bin/target_detail.cgi?targetID=598289) | 314 | 82 | hsa-miR-148a-3p | [MAFG](http://www.ncbi.nlm.nih.gov/entrez/query.fcgi?db=gene&cmd=Retrieve&dopt=full_report&list_uids=4097) | MAF bZIP transcription factor G |
| [Details](http://mirdb.org/cgi-bin/target_detail.cgi?targetID=597928) | 315 | 82 | hsa-miR-148a-3p | [ATXN7L1](http://www.ncbi.nlm.nih.gov/entrez/query.fcgi?db=gene&cmd=Retrieve&dopt=full_report&list_uids=222255) | ataxin 7 like 1 |
| [Details](http://mirdb.org/cgi-bin/target_detail.cgi?targetID=598335) | 316 | 82 | hsa-miR-148a-3p | [GPRC5A](http://www.ncbi.nlm.nih.gov/entrez/query.fcgi?db=gene&cmd=Retrieve&dopt=full_report&list_uids=9052) | G protein-coupled receptor class C group 5 member A |
| [Details](http://mirdb.org/cgi-bin/target_detail.cgi?targetID=598299) | 317 | 81 | hsa-miR-148a-3p | [MGAT4A](http://www.ncbi.nlm.nih.gov/entrez/query.fcgi?db=gene&cmd=Retrieve&dopt=full_report&list_uids=11320) | alpha-1,3-mannosyl-glycoprotein 4-beta-N-acetylglucosaminyltransferase A |
| [Details](http://mirdb.org/cgi-bin/target_detail.cgi?targetID=597950) | 318 | 81 | hsa-miR-148a-3p | [ABCA1](http://www.ncbi.nlm.nih.gov/entrez/query.fcgi?db=gene&cmd=Retrieve&dopt=full_report&list_uids=19) | ATP binding cassette subfamily A member 1 |
| [Details](http://mirdb.org/cgi-bin/target_detail.cgi?targetID=598317) | 319 | 81 | hsa-miR-148a-3p | [PIGS](http://www.ncbi.nlm.nih.gov/entrez/query.fcgi?db=gene&cmd=Retrieve&dopt=full_report&list_uids=94005) | phosphatidylinositol glycan anchor biosynthesis class S |
| [Details](http://mirdb.org/cgi-bin/target_detail.cgi?targetID=598305) | 320 | 81 | hsa-miR-148a-3p | [PRNP](http://www.ncbi.nlm.nih.gov/entrez/query.fcgi?db=gene&cmd=Retrieve&dopt=full_report&list_uids=5621) | prion protein |
| [Details](http://mirdb.org/cgi-bin/target_detail.cgi?targetID=598396) | 321 | 81 | hsa-miR-148a-3p | [CDS1](http://www.ncbi.nlm.nih.gov/entrez/query.fcgi?db=gene&cmd=Retrieve&dopt=full_report&list_uids=1040) | CDP-diacylglycerol synthase 1 |
| [Details](http://mirdb.org/cgi-bin/target_detail.cgi?targetID=598470) | 322 | 81 | hsa-miR-148a-3p | [BACH2](http://www.ncbi.nlm.nih.gov/entrez/query.fcgi?db=gene&cmd=Retrieve&dopt=full_report&list_uids=60468) | BTB domain and CNC homolog 2 |
| [Details](http://mirdb.org/cgi-bin/target_detail.cgi?targetID=598624) | 323 | 81 | hsa-miR-148a-3p | [LYSMD2](http://www.ncbi.nlm.nih.gov/entrez/query.fcgi?db=gene&cmd=Retrieve&dopt=full_report&list_uids=256586) | LysM domain containing 2 |
| [Details](http://mirdb.org/cgi-bin/target_detail.cgi?targetID=598253) | 324 | 81 | hsa-miR-148a-3p | [GPCPD1](http://www.ncbi.nlm.nih.gov/entrez/query.fcgi?db=gene&cmd=Retrieve&dopt=full_report&list_uids=56261) | glycerophosphocholine phosphodiesterase 1 |
| [Details](http://mirdb.org/cgi-bin/target_detail.cgi?targetID=597947) | 325 | 81 | hsa-miR-148a-3p | [ATXN1](http://www.ncbi.nlm.nih.gov/entrez/query.fcgi?db=gene&cmd=Retrieve&dopt=full_report&list_uids=6310) | ataxin 1 |
| [Details](http://mirdb.org/cgi-bin/target_detail.cgi?targetID=598316) | 326 | 81 | hsa-miR-148a-3p | [TMEM266](http://www.ncbi.nlm.nih.gov/entrez/query.fcgi?db=gene&cmd=Retrieve&dopt=full_report&list_uids=123591) | transmembrane protein 266 |
| [Details](http://mirdb.org/cgi-bin/target_detail.cgi?targetID=598176) | 327 | 81 | hsa-miR-148a-3p | [RAB14](http://www.ncbi.nlm.nih.gov/entrez/query.fcgi?db=gene&cmd=Retrieve&dopt=full_report&list_uids=51552) | RAB14, member RAS oncogene family |
| [Details](http://mirdb.org/cgi-bin/target_detail.cgi?targetID=598622) | 328 | 81 | hsa-miR-148a-3p | [AHDC1](http://www.ncbi.nlm.nih.gov/entrez/query.fcgi?db=gene&cmd=Retrieve&dopt=full_report&list_uids=27245) | AT-hook DNA binding motif containing 1 |
| [Details](http://mirdb.org/cgi-bin/target_detail.cgi?targetID=598077) | 329 | 81 | hsa-miR-148a-3p | [SLC26A4](http://www.ncbi.nlm.nih.gov/entrez/query.fcgi?db=gene&cmd=Retrieve&dopt=full_report&list_uids=5172) | solute carrier family 26 member 4 |
| [Details](http://mirdb.org/cgi-bin/target_detail.cgi?targetID=598598) | 330 | 81 | hsa-miR-148a-3p | [RFX7](http://www.ncbi.nlm.nih.gov/entrez/query.fcgi?db=gene&cmd=Retrieve&dopt=full_report&list_uids=64864) | regulatory factor X7 |
| [Details](http://mirdb.org/cgi-bin/target_detail.cgi?targetID=598323) | 331 | 81 | hsa-miR-148a-3p | [ADCY2](http://www.ncbi.nlm.nih.gov/entrez/query.fcgi?db=gene&cmd=Retrieve&dopt=full_report&list_uids=108) | adenylate cyclase 2 |
| [Details](http://mirdb.org/cgi-bin/target_detail.cgi?targetID=598375) | 332 | 81 | hsa-miR-148a-3p | [CADM1](http://www.ncbi.nlm.nih.gov/entrez/query.fcgi?db=gene&cmd=Retrieve&dopt=full_report&list_uids=23705) | cell adhesion molecule 1 |
| [Details](http://mirdb.org/cgi-bin/target_detail.cgi?targetID=598657) | 333 | 80 | hsa-miR-148a-3p | [ISM1](http://www.ncbi.nlm.nih.gov/entrez/query.fcgi?db=gene&cmd=Retrieve&dopt=full_report&list_uids=140862) | isthmin 1 |
| [Details](http://mirdb.org/cgi-bin/target_detail.cgi?targetID=597855) | 334 | 80 | hsa-miR-148a-3p | [BRPF1](http://www.ncbi.nlm.nih.gov/entrez/query.fcgi?db=gene&cmd=Retrieve&dopt=full_report&list_uids=7862) | bromodomain and PHD finger containing 1 |
| [Details](http://mirdb.org/cgi-bin/target_detail.cgi?targetID=597998) | 335 | 80 | hsa-miR-148a-3p | [PTGES3](http://www.ncbi.nlm.nih.gov/entrez/query.fcgi?db=gene&cmd=Retrieve&dopt=full_report&list_uids=10728) | prostaglandin E synthase 3 |
| [Details](http://mirdb.org/cgi-bin/target_detail.cgi?targetID=598219) | 336 | 80 | hsa-miR-148a-3p | [TFRC](http://www.ncbi.nlm.nih.gov/entrez/query.fcgi?db=gene&cmd=Retrieve&dopt=full_report&list_uids=7037) | transferrin receptor |
| [Details](http://mirdb.org/cgi-bin/target_detail.cgi?targetID=598439) | 337 | 80 | hsa-miR-148a-3p | [WDFY4](http://www.ncbi.nlm.nih.gov/entrez/query.fcgi?db=gene&cmd=Retrieve&dopt=full_report&list_uids=57705) | WDFY family member 4 |
| [Details](http://mirdb.org/cgi-bin/target_detail.cgi?targetID=597942) | 338 | 80 | hsa-miR-148a-3p | [CTSA](http://www.ncbi.nlm.nih.gov/entrez/query.fcgi?db=gene&cmd=Retrieve&dopt=full_report&list_uids=5476) | cathepsin A |
| [Details](http://mirdb.org/cgi-bin/target_detail.cgi?targetID=598376) | 339 | 80 | hsa-miR-148a-3p | [ZNRF1](http://www.ncbi.nlm.nih.gov/entrez/query.fcgi?db=gene&cmd=Retrieve&dopt=full_report&list_uids=84937) | zinc and ring finger 1 |
| [Details](http://mirdb.org/cgi-bin/target_detail.cgi?targetID=597945) | 340 | 80 | hsa-miR-148a-3p | [KIF14](http://www.ncbi.nlm.nih.gov/entrez/query.fcgi?db=gene&cmd=Retrieve&dopt=full_report&list_uids=9928) | kinesin family member 14 |
| [Details](http://mirdb.org/cgi-bin/target_detail.cgi?targetID=598181) | 341 | 80 | hsa-miR-148a-3p | [SIRT7](http://www.ncbi.nlm.nih.gov/entrez/query.fcgi?db=gene&cmd=Retrieve&dopt=full_report&list_uids=51547) | sirtuin 7 |
| [Details](http://mirdb.org/cgi-bin/target_detail.cgi?targetID=598115) | 342 | 80 | hsa-miR-148a-3p | [ADAM10](http://www.ncbi.nlm.nih.gov/entrez/query.fcgi?db=gene&cmd=Retrieve&dopt=full_report&list_uids=102) | ADAM metallopeptidase domain 10 |
| [Details](http://mirdb.org/cgi-bin/target_detail.cgi?targetID=597852) | 343 | 80 | hsa-miR-148a-3p | [SBF2](http://www.ncbi.nlm.nih.gov/entrez/query.fcgi?db=gene&cmd=Retrieve&dopt=full_report&list_uids=81846) | SET binding factor 2 |
| [Details](http://mirdb.org/cgi-bin/target_detail.cgi?targetID=597995) | 344 | 80 | hsa-miR-148a-3p | [CEP55](http://www.ncbi.nlm.nih.gov/entrez/query.fcgi?db=gene&cmd=Retrieve&dopt=full_report&list_uids=55165) | centrosomal protein 55 |
| [Details](http://mirdb.org/cgi-bin/target_detail.cgi?targetID=598223) | 345 | 79 | hsa-miR-148a-3p | [MDM4](http://www.ncbi.nlm.nih.gov/entrez/query.fcgi?db=gene&cmd=Retrieve&dopt=full_report&list_uids=4194) | MDM4, p53 regulator |
| [Details](http://mirdb.org/cgi-bin/target_detail.cgi?targetID=598362) | 346 | 79 | hsa-miR-148a-3p | [GOLT1B](http://www.ncbi.nlm.nih.gov/entrez/query.fcgi?db=gene&cmd=Retrieve&dopt=full_report&list_uids=51026) | golgi transport 1B |
| [Details](http://mirdb.org/cgi-bin/target_detail.cgi?targetID=597902) | 347 | 79 | hsa-miR-148a-3p | [SNX3](http://www.ncbi.nlm.nih.gov/entrez/query.fcgi?db=gene&cmd=Retrieve&dopt=full_report&list_uids=8724) | sorting nexin 3 |
| [Details](http://mirdb.org/cgi-bin/target_detail.cgi?targetID=598116) | 348 | 79 | hsa-miR-148a-3p | [GABPB2](http://www.ncbi.nlm.nih.gov/entrez/query.fcgi?db=gene&cmd=Retrieve&dopt=full_report&list_uids=126626) | GA binding protein transcription factor subunit beta 2 |
| [Details](http://mirdb.org/cgi-bin/target_detail.cgi?targetID=597980) | 349 | 79 | hsa-miR-148a-3p | [MTMR12](http://www.ncbi.nlm.nih.gov/entrez/query.fcgi?db=gene&cmd=Retrieve&dopt=full_report&list_uids=54545) | myotubularin related protein 12 |
| [Details](http://mirdb.org/cgi-bin/target_detail.cgi?targetID=597899) | 350 | 79 | hsa-miR-148a-3p | [CLCN3](http://www.ncbi.nlm.nih.gov/entrez/query.fcgi?db=gene&cmd=Retrieve&dopt=full_report&list_uids=1182) | chloride voltage-gated channel 3 |
| [Details](http://mirdb.org/cgi-bin/target_detail.cgi?targetID=598521) | 351 | 79 | hsa-miR-148a-3p | [CRISP1](http://www.ncbi.nlm.nih.gov/entrez/query.fcgi?db=gene&cmd=Retrieve&dopt=full_report&list_uids=167) | cysteine rich secretory protein 1 |
| [Details](http://mirdb.org/cgi-bin/target_detail.cgi?targetID=598165) | 352 | 79 | hsa-miR-148a-3p | [TRIP12](http://www.ncbi.nlm.nih.gov/entrez/query.fcgi?db=gene&cmd=Retrieve&dopt=full_report&list_uids=9320) | thyroid hormone receptor interactor 12 |
| [Details](http://mirdb.org/cgi-bin/target_detail.cgi?targetID=598334) | 353 | 79 | hsa-miR-148a-3p | [IGF1](http://www.ncbi.nlm.nih.gov/entrez/query.fcgi?db=gene&cmd=Retrieve&dopt=full_report&list_uids=3479) | insulin like growth factor 1 |
| [Details](http://mirdb.org/cgi-bin/target_detail.cgi?targetID=598659) | 354 | 79 | hsa-miR-148a-3p | [ATP7A](http://www.ncbi.nlm.nih.gov/entrez/query.fcgi?db=gene&cmd=Retrieve&dopt=full_report&list_uids=538) | ATPase copper transporting alpha |
| [Details](http://mirdb.org/cgi-bin/target_detail.cgi?targetID=597984) | 355 | 79 | hsa-miR-148a-3p | [SNAPIN](http://www.ncbi.nlm.nih.gov/entrez/query.fcgi?db=gene&cmd=Retrieve&dopt=full_report&list_uids=23557) | SNAP associated protein |
| [Details](http://mirdb.org/cgi-bin/target_detail.cgi?targetID=598452) | 356 | 79 | hsa-miR-148a-3p | [TNPO1](http://www.ncbi.nlm.nih.gov/entrez/query.fcgi?db=gene&cmd=Retrieve&dopt=full_report&list_uids=3842) | transportin 1 |
| [Details](http://mirdb.org/cgi-bin/target_detail.cgi?targetID=598628) | 357 | 79 | hsa-miR-148a-3p | [RBFOX2](http://www.ncbi.nlm.nih.gov/entrez/query.fcgi?db=gene&cmd=Retrieve&dopt=full_report&list_uids=23543) | RNA binding fox-1 homolog 2 |
| [Details](http://mirdb.org/cgi-bin/target_detail.cgi?targetID=598377) | 358 | 79 | hsa-miR-148a-3p | [DCUN1D4](http://www.ncbi.nlm.nih.gov/entrez/query.fcgi?db=gene&cmd=Retrieve&dopt=full_report&list_uids=23142) | defective in cullin neddylation 1 domain containing 4 |
| [Details](http://mirdb.org/cgi-bin/target_detail.cgi?targetID=598076) | 359 | 79 | hsa-miR-148a-3p | [SKP1](http://www.ncbi.nlm.nih.gov/entrez/query.fcgi?db=gene&cmd=Retrieve&dopt=full_report&list_uids=6500) | S-phase kinase associated protein 1 |
| [Details](http://mirdb.org/cgi-bin/target_detail.cgi?targetID=598645) | 360 | 79 | hsa-miR-148a-3p | [SERPINE1](http://www.ncbi.nlm.nih.gov/entrez/query.fcgi?db=gene&cmd=Retrieve&dopt=full_report&list_uids=5054) | serpin family E member 1 |
| [Details](http://mirdb.org/cgi-bin/target_detail.cgi?targetID=597908) | 361 | 79 | hsa-miR-148a-3p | [TAF4](http://www.ncbi.nlm.nih.gov/entrez/query.fcgi?db=gene&cmd=Retrieve&dopt=full_report&list_uids=6874) | TATA-box binding protein associated factor 4 |
| [Details](http://mirdb.org/cgi-bin/target_detail.cgi?targetID=598476) | 362 | 79 | hsa-miR-148a-3p | [KCND3](http://www.ncbi.nlm.nih.gov/entrez/query.fcgi?db=gene&cmd=Retrieve&dopt=full_report&list_uids=3752) | potassium voltage-gated channel subfamily D member 3 |
| [Details](http://mirdb.org/cgi-bin/target_detail.cgi?targetID=597963) | 363 | 79 | hsa-miR-148a-3p | [ST8SIA3](http://www.ncbi.nlm.nih.gov/entrez/query.fcgi?db=gene&cmd=Retrieve&dopt=full_report&list_uids=51046) | ST8 alpha-N-acetyl-neuraminide alpha-2,8-sialyltransferase 3 |
| [Details](http://mirdb.org/cgi-bin/target_detail.cgi?targetID=598041) | 364 | 78 | hsa-miR-148a-3p | [CHD1](http://www.ncbi.nlm.nih.gov/entrez/query.fcgi?db=gene&cmd=Retrieve&dopt=full_report&list_uids=1105) | chromodomain helicase DNA binding protein 1 |
| [Details](http://mirdb.org/cgi-bin/target_detail.cgi?targetID=598569) | 365 | 78 | hsa-miR-148a-3p | [BAHCC1](http://www.ncbi.nlm.nih.gov/entrez/query.fcgi?db=gene&cmd=Retrieve&dopt=full_report&list_uids=57597) | BAH domain and coiled-coil containing 1 |
| [Details](http://mirdb.org/cgi-bin/target_detail.cgi?targetID=598244) | 366 | 78 | hsa-miR-148a-3p | [CTTNBP2NL](http://www.ncbi.nlm.nih.gov/entrez/query.fcgi?db=gene&cmd=Retrieve&dopt=full_report&list_uids=55917) | CTTNBP2 N-terminal like |
| [Details](http://mirdb.org/cgi-bin/target_detail.cgi?targetID=598183) | 367 | 78 | hsa-miR-148a-3p | [SH2B3](http://www.ncbi.nlm.nih.gov/entrez/query.fcgi?db=gene&cmd=Retrieve&dopt=full_report&list_uids=10019) | SH2B adaptor protein 3 |
| [Details](http://mirdb.org/cgi-bin/target_detail.cgi?targetID=598491) | 368 | 78 | hsa-miR-148a-3p | [ITGA11](http://www.ncbi.nlm.nih.gov/entrez/query.fcgi?db=gene&cmd=Retrieve&dopt=full_report&list_uids=22801) | integrin subunit alpha 11 |
| [Details](http://mirdb.org/cgi-bin/target_detail.cgi?targetID=598047) | 369 | 78 | hsa-miR-148a-3p | [RAB10](http://www.ncbi.nlm.nih.gov/entrez/query.fcgi?db=gene&cmd=Retrieve&dopt=full_report&list_uids=10890) | RAB10, member RAS oncogene family |
| [Details](http://mirdb.org/cgi-bin/target_detail.cgi?targetID=598151) | 370 | 78 | hsa-miR-148a-3p | [PPP1R12A](http://www.ncbi.nlm.nih.gov/entrez/query.fcgi?db=gene&cmd=Retrieve&dopt=full_report&list_uids=4659) | protein phosphatase 1 regulatory subunit 12A |
| [Details](http://mirdb.org/cgi-bin/target_detail.cgi?targetID=598446) | 371 | 78 | hsa-miR-148a-3p | [CAST](http://www.ncbi.nlm.nih.gov/entrez/query.fcgi?db=gene&cmd=Retrieve&dopt=full_report&list_uids=831) | calpastatin |
| [Details](http://mirdb.org/cgi-bin/target_detail.cgi?targetID=598089) | 372 | 78 | hsa-miR-148a-3p | [ZNF3](http://www.ncbi.nlm.nih.gov/entrez/query.fcgi?db=gene&cmd=Retrieve&dopt=full_report&list_uids=7551) | zinc finger protein 3 |
| [Details](http://mirdb.org/cgi-bin/target_detail.cgi?targetID=598381) | 373 | 78 | hsa-miR-148a-3p | [CDK8](http://www.ncbi.nlm.nih.gov/entrez/query.fcgi?db=gene&cmd=Retrieve&dopt=full_report&list_uids=1024) | cyclin dependent kinase 8 |
| [Details](http://mirdb.org/cgi-bin/target_detail.cgi?targetID=598498) | 374 | 78 | hsa-miR-148a-3p | [FBXL19](http://www.ncbi.nlm.nih.gov/entrez/query.fcgi?db=gene&cmd=Retrieve&dopt=full_report&list_uids=54620) | F-box and leucine rich repeat protein 19 |
| [Details](http://mirdb.org/cgi-bin/target_detail.cgi?targetID=598500) | 375 | 78 | hsa-miR-148a-3p | [PHAX](http://www.ncbi.nlm.nih.gov/entrez/query.fcgi?db=gene&cmd=Retrieve&dopt=full_report&list_uids=51808) | phosphorylated adaptor for RNA export |
| [Details](http://mirdb.org/cgi-bin/target_detail.cgi?targetID=598413) | 376 | 78 | hsa-miR-148a-3p | [NFYA](http://www.ncbi.nlm.nih.gov/entrez/query.fcgi?db=gene&cmd=Retrieve&dopt=full_report&list_uids=4800) | nuclear transcription factor Y subunit alpha |
| [Details](http://mirdb.org/cgi-bin/target_detail.cgi?targetID=597966) | 377 | 78 | hsa-miR-148a-3p | [COL6A3](http://www.ncbi.nlm.nih.gov/entrez/query.fcgi?db=gene&cmd=Retrieve&dopt=full_report&list_uids=1293) | collagen type VI alpha 3 chain |
| [Details](http://mirdb.org/cgi-bin/target_detail.cgi?targetID=598301) | 378 | 78 | hsa-miR-148a-3p | [PICALM](http://www.ncbi.nlm.nih.gov/entrez/query.fcgi?db=gene&cmd=Retrieve&dopt=full_report&list_uids=8301) | phosphatidylinositol binding clathrin assembly protein |
| [Details](http://mirdb.org/cgi-bin/target_detail.cgi?targetID=598478) | 379 | 78 | hsa-miR-148a-3p | [BMT2](http://www.ncbi.nlm.nih.gov/entrez/query.fcgi?db=gene&cmd=Retrieve&dopt=full_report&list_uids=154743) | base methyltransferase of 25S rRNA 2 homolog |
| [Details](http://mirdb.org/cgi-bin/target_detail.cgi?targetID=598644) | 380 | 77 | hsa-miR-148a-3p | [STT3A](http://www.ncbi.nlm.nih.gov/entrez/query.fcgi?db=gene&cmd=Retrieve&dopt=full_report&list_uids=3703) | STT3A, catalytic subunit of the oligosaccharyltransferase complex |
| [Details](http://mirdb.org/cgi-bin/target_detail.cgi?targetID=598553) | 381 | 77 | hsa-miR-148a-3p | [MYO1D](http://www.ncbi.nlm.nih.gov/entrez/query.fcgi?db=gene&cmd=Retrieve&dopt=full_report&list_uids=4642) | myosin ID |
| [Details](http://mirdb.org/cgi-bin/target_detail.cgi?targetID=598108) | 382 | 77 | hsa-miR-148a-3p | [C12orf4](http://www.ncbi.nlm.nih.gov/entrez/query.fcgi?db=gene&cmd=Retrieve&dopt=full_report&list_uids=57102) | chromosome 12 open reading frame 4 |
| [Details](http://mirdb.org/cgi-bin/target_detail.cgi?targetID=598636) | 383 | 77 | hsa-miR-148a-3p | [SELENOT](http://www.ncbi.nlm.nih.gov/entrez/query.fcgi?db=gene&cmd=Retrieve&dopt=full_report&list_uids=51714) | selenoprotein T |
| [Details](http://mirdb.org/cgi-bin/target_detail.cgi?targetID=598134) | 384 | 77 | hsa-miR-148a-3p | [KIT](http://www.ncbi.nlm.nih.gov/entrez/query.fcgi?db=gene&cmd=Retrieve&dopt=full_report&list_uids=3815) | KIT proto-oncogene receptor tyrosine kinase |
| [Details](http://mirdb.org/cgi-bin/target_detail.cgi?targetID=597880) | 385 | 77 | hsa-miR-148a-3p | [MTMR1](http://www.ncbi.nlm.nih.gov/entrez/query.fcgi?db=gene&cmd=Retrieve&dopt=full_report&list_uids=8776) | myotubularin related protein 1 |
| [Details](http://mirdb.org/cgi-bin/target_detail.cgi?targetID=598507) | 386 | 77 | hsa-miR-148a-3p | [LUC7L](http://www.ncbi.nlm.nih.gov/entrez/query.fcgi?db=gene&cmd=Retrieve&dopt=full_report&list_uids=55692) | LUC7 like |
| [Details](http://mirdb.org/cgi-bin/target_detail.cgi?targetID=598020) | 387 | 77 | hsa-miR-148a-3p | [DLEU7](http://www.ncbi.nlm.nih.gov/entrez/query.fcgi?db=gene&cmd=Retrieve&dopt=full_report&list_uids=220107) | deleted in lymphocytic leukemia 7 |
| [Details](http://mirdb.org/cgi-bin/target_detail.cgi?targetID=598122) | 388 | 77 | hsa-miR-148a-3p | [FAM104B](http://www.ncbi.nlm.nih.gov/entrez/query.fcgi?db=gene&cmd=Retrieve&dopt=full_report&list_uids=90736) | family with sequence similarity 104 member B |
| [Details](http://mirdb.org/cgi-bin/target_detail.cgi?targetID=598320) | 389 | 77 | hsa-miR-148a-3p | [USP7](http://www.ncbi.nlm.nih.gov/entrez/query.fcgi?db=gene&cmd=Retrieve&dopt=full_report&list_uids=7874) | ubiquitin specific peptidase 7 |
| [Details](http://mirdb.org/cgi-bin/target_detail.cgi?targetID=597978) | 390 | 77 | hsa-miR-148a-3p | [TBP](http://www.ncbi.nlm.nih.gov/entrez/query.fcgi?db=gene&cmd=Retrieve&dopt=full_report&list_uids=6908) | TATA-box binding protein |
| [Details](http://mirdb.org/cgi-bin/target_detail.cgi?targetID=598206) | 391 | 77 | hsa-miR-148a-3p | [CDK1](http://www.ncbi.nlm.nih.gov/entrez/query.fcgi?db=gene&cmd=Retrieve&dopt=full_report&list_uids=983) | cyclin dependent kinase 1 |
| [Details](http://mirdb.org/cgi-bin/target_detail.cgi?targetID=598369) | 392 | 77 | hsa-miR-148a-3p | [DIPK2A](http://www.ncbi.nlm.nih.gov/entrez/query.fcgi?db=gene&cmd=Retrieve&dopt=full_report&list_uids=205428) | divergent protein kinase domain 2A |
| [Details](http://mirdb.org/cgi-bin/target_detail.cgi?targetID=598519) | 393 | 76 | hsa-miR-148a-3p | [SSBP2](http://www.ncbi.nlm.nih.gov/entrez/query.fcgi?db=gene&cmd=Retrieve&dopt=full_report&list_uids=23635) | single stranded DNA binding protein 2 |
| [Details](http://mirdb.org/cgi-bin/target_detail.cgi?targetID=597885) | 394 | 76 | hsa-miR-148a-3p | [DYRK1B](http://www.ncbi.nlm.nih.gov/entrez/query.fcgi?db=gene&cmd=Retrieve&dopt=full_report&list_uids=9149) | dual specificity tyrosine phosphorylation regulated kinase 1B |
| [Details](http://mirdb.org/cgi-bin/target_detail.cgi?targetID=597883) | 395 | 76 | hsa-miR-148a-3p | [ST18](http://www.ncbi.nlm.nih.gov/entrez/query.fcgi?db=gene&cmd=Retrieve&dopt=full_report&list_uids=9705) | ST18, C2H2C-type zinc finger |
| [Details](http://mirdb.org/cgi-bin/target_detail.cgi?targetID=598485) | 396 | 76 | hsa-miR-148a-3p | [DYRK1A](http://www.ncbi.nlm.nih.gov/entrez/query.fcgi?db=gene&cmd=Retrieve&dopt=full_report&list_uids=1859) | dual specificity tyrosine phosphorylation regulated kinase 1A |
| [Details](http://mirdb.org/cgi-bin/target_detail.cgi?targetID=598355) | 397 | 76 | hsa-miR-148a-3p | [PKHD1](http://www.ncbi.nlm.nih.gov/entrez/query.fcgi?db=gene&cmd=Retrieve&dopt=full_report&list_uids=5314) | PKHD1, fibrocystin/polyductin |
| [Details](http://mirdb.org/cgi-bin/target_detail.cgi?targetID=598060) | 398 | 76 | hsa-miR-148a-3p | [PSMD9](http://www.ncbi.nlm.nih.gov/entrez/query.fcgi?db=gene&cmd=Retrieve&dopt=full_report&list_uids=5715) | proteasome 26S subunit, non-ATPase 9 |
| [Details](http://mirdb.org/cgi-bin/target_detail.cgi?targetID=598573) | 399 | 76 | hsa-miR-148a-3p | [NETO1](http://www.ncbi.nlm.nih.gov/entrez/query.fcgi?db=gene&cmd=Retrieve&dopt=full_report&list_uids=81832) | neuropilin and tolloid like 1 |
| [Details](http://mirdb.org/cgi-bin/target_detail.cgi?targetID=598353) | 400 | 76 | hsa-miR-148a-3p | [SOS1](http://www.ncbi.nlm.nih.gov/entrez/query.fcgi?db=gene&cmd=Retrieve&dopt=full_report&list_uids=6654) | SOS Ras/Rac guanine nucleotide exchange factor 1 |
| [Details](http://mirdb.org/cgi-bin/target_detail.cgi?targetID=598589) | 401 | 76 | hsa-miR-148a-3p | [AGBL2](http://www.ncbi.nlm.nih.gov/entrez/query.fcgi?db=gene&cmd=Retrieve&dopt=full_report&list_uids=79841) | ATP/GTP binding protein like 2 |
| [Details](http://mirdb.org/cgi-bin/target_detail.cgi?targetID=598656) | 402 | 76 | hsa-miR-148a-3p | [STX3](http://www.ncbi.nlm.nih.gov/entrez/query.fcgi?db=gene&cmd=Retrieve&dopt=full_report&list_uids=6809) | syntaxin 3 |
| [Details](http://mirdb.org/cgi-bin/target_detail.cgi?targetID=598419) | 403 | 76 | hsa-miR-148a-3p | [KDM2B](http://www.ncbi.nlm.nih.gov/entrez/query.fcgi?db=gene&cmd=Retrieve&dopt=full_report&list_uids=84678) | lysine demethylase 2B |
| [Details](http://mirdb.org/cgi-bin/target_detail.cgi?targetID=598187) | 404 | 76 | hsa-miR-148a-3p | [TMEM121B](http://www.ncbi.nlm.nih.gov/entrez/query.fcgi?db=gene&cmd=Retrieve&dopt=full_report&list_uids=27439) | transmembrane protein 121B |
| [Details](http://mirdb.org/cgi-bin/target_detail.cgi?targetID=597967) | 405 | 76 | hsa-miR-148a-3p | [TAF4B](http://www.ncbi.nlm.nih.gov/entrez/query.fcgi?db=gene&cmd=Retrieve&dopt=full_report&list_uids=6875) | TATA-box binding protein associated factor 4b |
| [Details](http://mirdb.org/cgi-bin/target_detail.cgi?targetID=598322) | 406 | 76 | hsa-miR-148a-3p | [PAG1](http://www.ncbi.nlm.nih.gov/entrez/query.fcgi?db=gene&cmd=Retrieve&dopt=full_report&list_uids=55824) | phosphoprotein membrane anchor with glycosphingolipid microdomains 1 |
| [Details](http://mirdb.org/cgi-bin/target_detail.cgi?targetID=598604) | 407 | 76 | hsa-miR-148a-3p | [UBN2](http://www.ncbi.nlm.nih.gov/entrez/query.fcgi?db=gene&cmd=Retrieve&dopt=full_report&list_uids=254048) | ubinuclein 2 |
| [Details](http://mirdb.org/cgi-bin/target_detail.cgi?targetID=598242) | 408 | 76 | hsa-miR-148a-3p | [PTEN](http://www.ncbi.nlm.nih.gov/entrez/query.fcgi?db=gene&cmd=Retrieve&dopt=full_report&list_uids=5728) | phosphatase and tensin homolog |
| [Details](http://mirdb.org/cgi-bin/target_detail.cgi?targetID=598111) | 409 | 76 | hsa-miR-148a-3p | [USP6](http://www.ncbi.nlm.nih.gov/entrez/query.fcgi?db=gene&cmd=Retrieve&dopt=full_report&list_uids=9098) | ubiquitin specific peptidase 6 |
| [Details](http://mirdb.org/cgi-bin/target_detail.cgi?targetID=598332) | 410 | 76 | hsa-miR-148a-3p | [BBX](http://www.ncbi.nlm.nih.gov/entrez/query.fcgi?db=gene&cmd=Retrieve&dopt=full_report&list_uids=56987) | BBX, HMG-box containing |
| [Details](http://mirdb.org/cgi-bin/target_detail.cgi?targetID=597930) | 411 | 76 | hsa-miR-148a-3p | [MARF1](http://www.ncbi.nlm.nih.gov/entrez/query.fcgi?db=gene&cmd=Retrieve&dopt=full_report&list_uids=9665) | meiosis regulator and mRNA stability factor 1 |
| [Details](http://mirdb.org/cgi-bin/target_detail.cgi?targetID=598609) | 412 | 76 | hsa-miR-148a-3p | [LILRB4](http://www.ncbi.nlm.nih.gov/entrez/query.fcgi?db=gene&cmd=Retrieve&dopt=full_report&list_uids=11006) | leukocyte immunoglobulin like receptor B4 |
| [Details](http://mirdb.org/cgi-bin/target_detail.cgi?targetID=598421) | 413 | 76 | hsa-miR-148a-3p | [MTMR6](http://www.ncbi.nlm.nih.gov/entrez/query.fcgi?db=gene&cmd=Retrieve&dopt=full_report&list_uids=9107) | myotubularin related protein 6 |
| [Details](http://mirdb.org/cgi-bin/target_detail.cgi?targetID=598117) | 414 | 76 | hsa-miR-148a-3p | [PIK3CA](http://www.ncbi.nlm.nih.gov/entrez/query.fcgi?db=gene&cmd=Retrieve&dopt=full_report&list_uids=5290) | phosphatidylinositol-4,5-bisphosphate 3-kinase catalytic subunit alpha |
| [Details](http://mirdb.org/cgi-bin/target_detail.cgi?targetID=597987) | 415 | 75 | hsa-miR-148a-3p | [ELF5](http://www.ncbi.nlm.nih.gov/entrez/query.fcgi?db=gene&cmd=Retrieve&dopt=full_report&list_uids=2001) | E74 like ETS transcription factor 5 |
| [Details](http://mirdb.org/cgi-bin/target_detail.cgi?targetID=597872) | 416 | 75 | hsa-miR-148a-3p | [FAM149A](http://www.ncbi.nlm.nih.gov/entrez/query.fcgi?db=gene&cmd=Retrieve&dopt=full_report&list_uids=25854) | family with sequence similarity 149 member A |
| [Details](http://mirdb.org/cgi-bin/target_detail.cgi?targetID=597922) | 417 | 75 | hsa-miR-148a-3p | [ATG4D](http://www.ncbi.nlm.nih.gov/entrez/query.fcgi?db=gene&cmd=Retrieve&dopt=full_report&list_uids=84971) | autophagy related 4D cysteine peptidase |
| [Details](http://mirdb.org/cgi-bin/target_detail.cgi?targetID=598112) | 418 | 75 | hsa-miR-148a-3p | [DDAH1](http://www.ncbi.nlm.nih.gov/entrez/query.fcgi?db=gene&cmd=Retrieve&dopt=full_report&list_uids=23576) | dimethylarginine dimethylaminohydrolase 1 |
| [Details](http://mirdb.org/cgi-bin/target_detail.cgi?targetID=598410) | 419 | 75 | hsa-miR-148a-3p | [PPARGC1B](http://www.ncbi.nlm.nih.gov/entrez/query.fcgi?db=gene&cmd=Retrieve&dopt=full_report&list_uids=133522) | PPARG coactivator 1 beta |
| [Details](http://mirdb.org/cgi-bin/target_detail.cgi?targetID=598591) | 420 | 75 | hsa-miR-148a-3p | [GNS](http://www.ncbi.nlm.nih.gov/entrez/query.fcgi?db=gene&cmd=Retrieve&dopt=full_report&list_uids=2799) | glucosamine (N-acetyl)-6-sulfatase |
| [Details](http://mirdb.org/cgi-bin/target_detail.cgi?targetID=598607) | 421 | 75 | hsa-miR-148a-3p | [DCP1A](http://www.ncbi.nlm.nih.gov/entrez/query.fcgi?db=gene&cmd=Retrieve&dopt=full_report&list_uids=55802) | decapping mRNA 1A |
| [Details](http://mirdb.org/cgi-bin/target_detail.cgi?targetID=598647) | 422 | 75 | hsa-miR-148a-3p | [RHD](http://www.ncbi.nlm.nih.gov/entrez/query.fcgi?db=gene&cmd=Retrieve&dopt=full_report&list_uids=6007) | Rh blood group D antigen |
| [Details](http://mirdb.org/cgi-bin/target_detail.cgi?targetID=598414) | 423 | 75 | hsa-miR-148a-3p | [MAFF](http://www.ncbi.nlm.nih.gov/entrez/query.fcgi?db=gene&cmd=Retrieve&dopt=full_report&list_uids=23764) | MAF bZIP transcription factor F |
| [Details](http://mirdb.org/cgi-bin/target_detail.cgi?targetID=598588) | 424 | 75 | hsa-miR-148a-3p | [TFDP2](http://www.ncbi.nlm.nih.gov/entrez/query.fcgi?db=gene&cmd=Retrieve&dopt=full_report&list_uids=7029) | transcription factor Dp-2 |
| [Details](http://mirdb.org/cgi-bin/target_detail.cgi?targetID=598256) | 425 | 75 | hsa-miR-148a-3p | [SLC12A7](http://www.ncbi.nlm.nih.gov/entrez/query.fcgi?db=gene&cmd=Retrieve&dopt=full_report&list_uids=10723) | solute carrier family 12 member 7 |
| [Details](http://mirdb.org/cgi-bin/target_detail.cgi?targetID=598306) | 426 | 75 | hsa-miR-148a-3p | [KRR1](http://www.ncbi.nlm.nih.gov/entrez/query.fcgi?db=gene&cmd=Retrieve&dopt=full_report&list_uids=11103) | KRR1, small subunit processome component homolog |
| [Details](http://mirdb.org/cgi-bin/target_detail.cgi?targetID=598349) | 427 | 74 | hsa-miR-148a-3p | [CHST1](http://www.ncbi.nlm.nih.gov/entrez/query.fcgi?db=gene&cmd=Retrieve&dopt=full_report&list_uids=8534) | carbohydrate sulfotransferase 1 |
| [Details](http://mirdb.org/cgi-bin/target_detail.cgi?targetID=598311) | 428 | 74 | hsa-miR-148a-3p | [FBXO28](http://www.ncbi.nlm.nih.gov/entrez/query.fcgi?db=gene&cmd=Retrieve&dopt=full_report&list_uids=23219) | F-box protein 28 |
| [Details](http://mirdb.org/cgi-bin/target_detail.cgi?targetID=598528) | 429 | 74 | hsa-miR-148a-3p | [DSC2](http://www.ncbi.nlm.nih.gov/entrez/query.fcgi?db=gene&cmd=Retrieve&dopt=full_report&list_uids=1824) | desmocollin 2 |
| [Details](http://mirdb.org/cgi-bin/target_detail.cgi?targetID=598095) | 430 | 74 | hsa-miR-148a-3p | [SPIRE1](http://www.ncbi.nlm.nih.gov/entrez/query.fcgi?db=gene&cmd=Retrieve&dopt=full_report&list_uids=56907) | spire type actin nucleation factor 1 |
| [Details](http://mirdb.org/cgi-bin/target_detail.cgi?targetID=598236) | 431 | 74 | hsa-miR-148a-3p | [SYT10](http://www.ncbi.nlm.nih.gov/entrez/query.fcgi?db=gene&cmd=Retrieve&dopt=full_report&list_uids=341359) | synaptotagmin 10 |
| [Details](http://mirdb.org/cgi-bin/target_detail.cgi?targetID=598142) | 432 | 74 | hsa-miR-148a-3p | [NPTX2](http://www.ncbi.nlm.nih.gov/entrez/query.fcgi?db=gene&cmd=Retrieve&dopt=full_report&list_uids=4885) | neuronal pentraxin 2 |
| [Details](http://mirdb.org/cgi-bin/target_detail.cgi?targetID=598615) | 433 | 74 | hsa-miR-148a-3p | [SOX11](http://www.ncbi.nlm.nih.gov/entrez/query.fcgi?db=gene&cmd=Retrieve&dopt=full_report&list_uids=6664) | SRY-box 11 |
| [Details](http://mirdb.org/cgi-bin/target_detail.cgi?targetID=598605) | 434 | 74 | hsa-miR-148a-3p | [RCC2](http://www.ncbi.nlm.nih.gov/entrez/query.fcgi?db=gene&cmd=Retrieve&dopt=full_report&list_uids=55920) | regulator of chromosome condensation 2 |
| [Details](http://mirdb.org/cgi-bin/target_detail.cgi?targetID=598044) | 435 | 74 | hsa-miR-148a-3p | [RUBCN](http://www.ncbi.nlm.nih.gov/entrez/query.fcgi?db=gene&cmd=Retrieve&dopt=full_report&list_uids=9711) | rubicon autophagy regulator |
| [Details](http://mirdb.org/cgi-bin/target_detail.cgi?targetID=598557) | 436 | 74 | hsa-miR-148a-3p | [TRPM5](http://www.ncbi.nlm.nih.gov/entrez/query.fcgi?db=gene&cmd=Retrieve&dopt=full_report&list_uids=29850) | transient receptor potential cation channel subfamily M member 5 |
| [Details](http://mirdb.org/cgi-bin/target_detail.cgi?targetID=598270) | 437 | 74 | hsa-miR-148a-3p | [LIPI](http://www.ncbi.nlm.nih.gov/entrez/query.fcgi?db=gene&cmd=Retrieve&dopt=full_report&list_uids=149998) | lipase I |
| [Details](http://mirdb.org/cgi-bin/target_detail.cgi?targetID=598464) | 438 | 74 | hsa-miR-148a-3p | [UBE2W](http://www.ncbi.nlm.nih.gov/entrez/query.fcgi?db=gene&cmd=Retrieve&dopt=full_report&list_uids=55284) | ubiquitin conjugating enzyme E2 W |
| [Details](http://mirdb.org/cgi-bin/target_detail.cgi?targetID=598185) | 439 | 74 | hsa-miR-148a-3p | [FAM13B](http://www.ncbi.nlm.nih.gov/entrez/query.fcgi?db=gene&cmd=Retrieve&dopt=full_report&list_uids=51306) | family with sequence similarity 13 member B |
| [Details](http://mirdb.org/cgi-bin/target_detail.cgi?targetID=598092) | 440 | 74 | hsa-miR-148a-3p | [DPP4](http://www.ncbi.nlm.nih.gov/entrez/query.fcgi?db=gene&cmd=Retrieve&dopt=full_report&list_uids=1803) | dipeptidyl peptidase 4 |
| [Details](http://mirdb.org/cgi-bin/target_detail.cgi?targetID=598525) | 441 | 73 | hsa-miR-148a-3p | [SCML4](http://www.ncbi.nlm.nih.gov/entrez/query.fcgi?db=gene&cmd=Retrieve&dopt=full_report&list_uids=256380) | Scm polycomb group protein like 4 |
| [Details](http://mirdb.org/cgi-bin/target_detail.cgi?targetID=598437) | 442 | 73 | hsa-miR-148a-3p | [MASP1](http://www.ncbi.nlm.nih.gov/entrez/query.fcgi?db=gene&cmd=Retrieve&dopt=full_report&list_uids=5648) | mannan binding lectin serine peptidase 1 |
| [Details](http://mirdb.org/cgi-bin/target_detail.cgi?targetID=598383) | 443 | 73 | hsa-miR-148a-3p | [CTSL](http://www.ncbi.nlm.nih.gov/entrez/query.fcgi?db=gene&cmd=Retrieve&dopt=full_report&list_uids=1514) | cathepsin L |
| [Details](http://mirdb.org/cgi-bin/target_detail.cgi?targetID=598212) | 444 | 73 | hsa-miR-148a-3p | [TCF4](http://www.ncbi.nlm.nih.gov/entrez/query.fcgi?db=gene&cmd=Retrieve&dopt=full_report&list_uids=6925) | transcription factor 4 |
| [Details](http://mirdb.org/cgi-bin/target_detail.cgi?targetID=598449) | 445 | 73 | hsa-miR-148a-3p | [NUDT12](http://www.ncbi.nlm.nih.gov/entrez/query.fcgi?db=gene&cmd=Retrieve&dopt=full_report&list_uids=83594) | nudix hydrolase 12 |
| [Details](http://mirdb.org/cgi-bin/target_detail.cgi?targetID=597871) | 446 | 73 | hsa-miR-148a-3p | [ERMP1](http://www.ncbi.nlm.nih.gov/entrez/query.fcgi?db=gene&cmd=Retrieve&dopt=full_report&list_uids=79956) | endoplasmic reticulum metallopeptidase 1 |
| [Details](http://mirdb.org/cgi-bin/target_detail.cgi?targetID=598264) | 447 | 73 | hsa-miR-148a-3p | [ARPP19](http://www.ncbi.nlm.nih.gov/entrez/query.fcgi?db=gene&cmd=Retrieve&dopt=full_report&list_uids=10776) | cAMP regulated phosphoprotein 19 |
| [Details](http://mirdb.org/cgi-bin/target_detail.cgi?targetID=597856) | 448 | 73 | hsa-miR-148a-3p | [NRAS](http://www.ncbi.nlm.nih.gov/entrez/query.fcgi?db=gene&cmd=Retrieve&dopt=full_report&list_uids=4893) | NRAS proto-oncogene, GTPase |
| [Details](http://mirdb.org/cgi-bin/target_detail.cgi?targetID=598559) | 449 | 73 | hsa-miR-148a-3p | [COL2A1](http://www.ncbi.nlm.nih.gov/entrez/query.fcgi?db=gene&cmd=Retrieve&dopt=full_report&list_uids=1280) | collagen type II alpha 1 chain |
| [Details](http://mirdb.org/cgi-bin/target_detail.cgi?targetID=598285) | 450 | 73 | hsa-miR-148a-3p | [DMPK](http://www.ncbi.nlm.nih.gov/entrez/query.fcgi?db=gene&cmd=Retrieve&dopt=full_report&list_uids=1760) | DM1 protein kinase |
| [Details](http://mirdb.org/cgi-bin/target_detail.cgi?targetID=598266) | 451 | 73 | hsa-miR-148a-3p | [LRP4](http://www.ncbi.nlm.nih.gov/entrez/query.fcgi?db=gene&cmd=Retrieve&dopt=full_report&list_uids=4038) | LDL receptor related protein 4 |
| [Details](http://mirdb.org/cgi-bin/target_detail.cgi?targetID=598161) | 452 | 73 | hsa-miR-148a-3p | [PIK3R3](http://www.ncbi.nlm.nih.gov/entrez/query.fcgi?db=gene&cmd=Retrieve&dopt=full_report&list_uids=8503) | phosphoinositide-3-kinase regulatory subunit 3 |
| [Details](http://mirdb.org/cgi-bin/target_detail.cgi?targetID=598249) | 453 | 73 | hsa-miR-148a-3p | [DNER](http://www.ncbi.nlm.nih.gov/entrez/query.fcgi?db=gene&cmd=Retrieve&dopt=full_report&list_uids=92737) | delta/notch like EGF repeat containing |
| [Details](http://mirdb.org/cgi-bin/target_detail.cgi?targetID=597992) | 454 | 73 | hsa-miR-148a-3p | [CDK14](http://www.ncbi.nlm.nih.gov/entrez/query.fcgi?db=gene&cmd=Retrieve&dopt=full_report&list_uids=5218) | cyclin dependent kinase 14 |
| [Details](http://mirdb.org/cgi-bin/target_detail.cgi?targetID=598531) | 455 | 73 | hsa-miR-148a-3p | [GAPVD1](http://www.ncbi.nlm.nih.gov/entrez/query.fcgi?db=gene&cmd=Retrieve&dopt=full_report&list_uids=26130) | GTPase activating protein and VPS9 domains 1 |
| [Details](http://mirdb.org/cgi-bin/target_detail.cgi?targetID=598201) | 456 | 72 | hsa-miR-148a-3p | [EMP1](http://www.ncbi.nlm.nih.gov/entrez/query.fcgi?db=gene&cmd=Retrieve&dopt=full_report&list_uids=2012) | epithelial membrane protein 1 |
| [Details](http://mirdb.org/cgi-bin/target_detail.cgi?targetID=597974) | 457 | 72 | hsa-miR-148a-3p | [CHCHD3](http://www.ncbi.nlm.nih.gov/entrez/query.fcgi?db=gene&cmd=Retrieve&dopt=full_report&list_uids=54927) | coiled-coil-helix-coiled-coil-helix domain containing 3 |
| [Details](http://mirdb.org/cgi-bin/target_detail.cgi?targetID=598071) | 458 | 72 | hsa-miR-148a-3p | [ZNF274](http://www.ncbi.nlm.nih.gov/entrez/query.fcgi?db=gene&cmd=Retrieve&dopt=full_report&list_uids=10782) | zinc finger protein 274 |
| [Details](http://mirdb.org/cgi-bin/target_detail.cgi?targetID=598537) | 459 | 72 | hsa-miR-148a-3p | [SULF1](http://www.ncbi.nlm.nih.gov/entrez/query.fcgi?db=gene&cmd=Retrieve&dopt=full_report&list_uids=23213) | sulfatase 1 |
| [Details](http://mirdb.org/cgi-bin/target_detail.cgi?targetID=598339) | 460 | 72 | hsa-miR-148a-3p | [RASAL1](http://www.ncbi.nlm.nih.gov/entrez/query.fcgi?db=gene&cmd=Retrieve&dopt=full_report&list_uids=8437) | RAS protein activator like 1 |
| [Details](http://mirdb.org/cgi-bin/target_detail.cgi?targetID=598542) | 461 | 72 | hsa-miR-148a-3p | [NSD2](http://www.ncbi.nlm.nih.gov/entrez/query.fcgi?db=gene&cmd=Retrieve&dopt=full_report&list_uids=7468) | nuclear receptor binding SET domain protein 2 |
| [Details](http://mirdb.org/cgi-bin/target_detail.cgi?targetID=597965) | 462 | 72 | hsa-miR-148a-3p | [SLC5A7](http://www.ncbi.nlm.nih.gov/entrez/query.fcgi?db=gene&cmd=Retrieve&dopt=full_report&list_uids=60482) | solute carrier family 5 member 7 |
| [Details](http://mirdb.org/cgi-bin/target_detail.cgi?targetID=598567) | 463 | 72 | hsa-miR-148a-3p | [TMEM52B](http://www.ncbi.nlm.nih.gov/entrez/query.fcgi?db=gene&cmd=Retrieve&dopt=full_report&list_uids=120939) | transmembrane protein 52B |
| [Details](http://mirdb.org/cgi-bin/target_detail.cgi?targetID=597936) | 464 | 72 | hsa-miR-148a-3p | [STXBP5](http://www.ncbi.nlm.nih.gov/entrez/query.fcgi?db=gene&cmd=Retrieve&dopt=full_report&list_uids=134957) | syntaxin binding protein 5 |
| [Details](http://mirdb.org/cgi-bin/target_detail.cgi?targetID=598443) | 465 | 72 | hsa-miR-148a-3p | [EPB41L5](http://www.ncbi.nlm.nih.gov/entrez/query.fcgi?db=gene&cmd=Retrieve&dopt=full_report&list_uids=57669) | erythrocyte membrane protein band 4.1 like 5 |
| [Details](http://mirdb.org/cgi-bin/target_detail.cgi?targetID=598653) | 466 | 72 | hsa-miR-148a-3p | [ZC3H12C](http://www.ncbi.nlm.nih.gov/entrez/query.fcgi?db=gene&cmd=Retrieve&dopt=full_report&list_uids=85463) | zinc finger CCCH-type containing 12C |
| [Details](http://mirdb.org/cgi-bin/target_detail.cgi?targetID=598203) | 467 | 72 | hsa-miR-148a-3p | [DDHD2](http://www.ncbi.nlm.nih.gov/entrez/query.fcgi?db=gene&cmd=Retrieve&dopt=full_report&list_uids=23259) | DDHD domain containing 2 |
| [Details](http://mirdb.org/cgi-bin/target_detail.cgi?targetID=597938) | 468 | 72 | hsa-miR-148a-3p | [MSI2](http://www.ncbi.nlm.nih.gov/entrez/query.fcgi?db=gene&cmd=Retrieve&dopt=full_report&list_uids=124540) | musashi RNA binding protein 2 |
| [Details](http://mirdb.org/cgi-bin/target_detail.cgi?targetID=598639) | 469 | 72 | hsa-miR-148a-3p | [C7orf71](http://www.ncbi.nlm.nih.gov/entrez/query.fcgi?db=gene&cmd=Retrieve&dopt=full_report&list_uids=285941) | chromosome 7 open reading frame 71 |
| [Details](http://mirdb.org/cgi-bin/target_detail.cgi?targetID=598252) | 470 | 72 | hsa-miR-148a-3p | [LAMA4](http://www.ncbi.nlm.nih.gov/entrez/query.fcgi?db=gene&cmd=Retrieve&dopt=full_report&list_uids=3910) | laminin subunit alpha 4 |
| [Details](http://mirdb.org/cgi-bin/target_detail.cgi?targetID=598467) | 471 | 72 | hsa-miR-148a-3p | [SYT6](http://www.ncbi.nlm.nih.gov/entrez/query.fcgi?db=gene&cmd=Retrieve&dopt=full_report&list_uids=148281) | synaptotagmin 6 |
| [Details](http://mirdb.org/cgi-bin/target_detail.cgi?targetID=598468) | 472 | 72 | hsa-miR-148a-3p | [MET](http://www.ncbi.nlm.nih.gov/entrez/query.fcgi?db=gene&cmd=Retrieve&dopt=full_report&list_uids=4233) | MET proto-oncogene, receptor tyrosine kinase |
| [Details](http://mirdb.org/cgi-bin/target_detail.cgi?targetID=598428) | 473 | 71 | hsa-miR-148a-3p | [DNAJB4](http://www.ncbi.nlm.nih.gov/entrez/query.fcgi?db=gene&cmd=Retrieve&dopt=full_report&list_uids=11080) | DnaJ heat shock protein family (Hsp40) member B4 |
| [Details](http://mirdb.org/cgi-bin/target_detail.cgi?targetID=598010) | 474 | 71 | hsa-miR-148a-3p | [CYFIP2](http://www.ncbi.nlm.nih.gov/entrez/query.fcgi?db=gene&cmd=Retrieve&dopt=full_report&list_uids=26999) | cytoplasmic FMR1 interacting protein 2 |
| [Details](http://mirdb.org/cgi-bin/target_detail.cgi?targetID=598451) | 475 | 71 | hsa-miR-148a-3p | [BCL7B](http://www.ncbi.nlm.nih.gov/entrez/query.fcgi?db=gene&cmd=Retrieve&dopt=full_report&list_uids=9275) | BCL7B, BAF complex component |
| [Details](http://mirdb.org/cgi-bin/target_detail.cgi?targetID=598280) | 476 | 71 | hsa-miR-148a-3p | [CCDC170](http://www.ncbi.nlm.nih.gov/entrez/query.fcgi?db=gene&cmd=Retrieve&dopt=full_report&list_uids=80129) | coiled-coil domain containing 170 |
| [Details](http://mirdb.org/cgi-bin/target_detail.cgi?targetID=598118) | 477 | 71 | hsa-miR-148a-3p | [MARCH2](http://www.ncbi.nlm.nih.gov/entrez/query.fcgi?db=gene&cmd=Retrieve&dopt=full_report&list_uids=51257) | membrane associated ring-CH-type finger 2 |
| [Details](http://mirdb.org/cgi-bin/target_detail.cgi?targetID=598019) | 478 | 71 | hsa-miR-148a-3p | [MLH1](http://www.ncbi.nlm.nih.gov/entrez/query.fcgi?db=gene&cmd=Retrieve&dopt=full_report&list_uids=4292) | mutL homolog 1 |
| [Details](http://mirdb.org/cgi-bin/target_detail.cgi?targetID=598034) | 479 | 71 | hsa-miR-148a-3p | [AK2](http://www.ncbi.nlm.nih.gov/entrez/query.fcgi?db=gene&cmd=Retrieve&dopt=full_report&list_uids=204) | adenylate kinase 2 |
| [Details](http://mirdb.org/cgi-bin/target_detail.cgi?targetID=597957) | 480 | 71 | hsa-miR-148a-3p | [LSM11](http://www.ncbi.nlm.nih.gov/entrez/query.fcgi?db=gene&cmd=Retrieve&dopt=full_report&list_uids=134353) | LSM11, U7 small nuclear RNA associated |
| [Details](http://mirdb.org/cgi-bin/target_detail.cgi?targetID=598354) | 481 | 71 | hsa-miR-148a-3p | [SNRK](http://www.ncbi.nlm.nih.gov/entrez/query.fcgi?db=gene&cmd=Retrieve&dopt=full_report&list_uids=54861) | SNF related kinase |
| [Details](http://mirdb.org/cgi-bin/target_detail.cgi?targetID=598578) | 482 | 71 | hsa-miR-148a-3p | [RBMS2](http://www.ncbi.nlm.nih.gov/entrez/query.fcgi?db=gene&cmd=Retrieve&dopt=full_report&list_uids=5939) | RNA binding motif single stranded interacting protein 2 |
| [Details](http://mirdb.org/cgi-bin/target_detail.cgi?targetID=598688) | 483 | 71 | hsa-miR-148a-3p | [TEAD1](http://www.ncbi.nlm.nih.gov/entrez/query.fcgi?db=gene&cmd=Retrieve&dopt=full_report&list_uids=7003) | TEA domain transcription factor 1 |
| [Details](http://mirdb.org/cgi-bin/target_detail.cgi?targetID=598189) | 484 | 71 | hsa-miR-148a-3p | [EPB41L3](http://www.ncbi.nlm.nih.gov/entrez/query.fcgi?db=gene&cmd=Retrieve&dopt=full_report&list_uids=23136) | erythrocyte membrane protein band 4.1 like 3 |
| [Details](http://mirdb.org/cgi-bin/target_detail.cgi?targetID=598408) | 485 | 70 | hsa-miR-148a-3p | [C2orf80](http://www.ncbi.nlm.nih.gov/entrez/query.fcgi?db=gene&cmd=Retrieve&dopt=full_report&list_uids=389073) | chromosome 2 open reading frame 80 |
| [Details](http://mirdb.org/cgi-bin/target_detail.cgi?targetID=598655) | 486 | 70 | hsa-miR-148a-3p | [KIAA1324L](http://www.ncbi.nlm.nih.gov/entrez/query.fcgi?db=gene&cmd=Retrieve&dopt=full_report&list_uids=222223) | KIAA1324 like |
| [Details](http://mirdb.org/cgi-bin/target_detail.cgi?targetID=598284) | 487 | 70 | hsa-miR-148a-3p | [SGMS2](http://www.ncbi.nlm.nih.gov/entrez/query.fcgi?db=gene&cmd=Retrieve&dopt=full_report&list_uids=166929) | sphingomyelin synthase 2 |
| [Details](http://mirdb.org/cgi-bin/target_detail.cgi?targetID=598088) | 488 | 70 | hsa-miR-148a-3p | [CNR1](http://www.ncbi.nlm.nih.gov/entrez/query.fcgi?db=gene&cmd=Retrieve&dopt=full_report&list_uids=1268) | cannabinoid receptor 1 |
| [Details](http://mirdb.org/cgi-bin/target_detail.cgi?targetID=598463) | 489 | 70 | hsa-miR-148a-3p | [PLPP3](http://www.ncbi.nlm.nih.gov/entrez/query.fcgi?db=gene&cmd=Retrieve&dopt=full_report&list_uids=8613) | phospholipid phosphatase 3 |
| [Details](http://mirdb.org/cgi-bin/target_detail.cgi?targetID=597864) | 490 | 70 | hsa-miR-148a-3p | [IPO8](http://www.ncbi.nlm.nih.gov/entrez/query.fcgi?db=gene&cmd=Retrieve&dopt=full_report&list_uids=10526) | importin 8 |
| [Details](http://mirdb.org/cgi-bin/target_detail.cgi?targetID=598174) | 491 | 70 | hsa-miR-148a-3p | [MTMR3](http://www.ncbi.nlm.nih.gov/entrez/query.fcgi?db=gene&cmd=Retrieve&dopt=full_report&list_uids=8897) | myotubularin related protein 3 |
| [Details](http://mirdb.org/cgi-bin/target_detail.cgi?targetID=598526) | 492 | 70 | hsa-miR-148a-3p | [PI4KA](http://www.ncbi.nlm.nih.gov/entrez/query.fcgi?db=gene&cmd=Retrieve&dopt=full_report&list_uids=5297) | phosphatidylinositol 4-kinase alpha |
| [Details](http://mirdb.org/cgi-bin/target_detail.cgi?targetID=598634) | 493 | 70 | hsa-miR-148a-3p | [SACS](http://www.ncbi.nlm.nih.gov/entrez/query.fcgi?db=gene&cmd=Retrieve&dopt=full_report&list_uids=26278) | sacsin molecular chaperone |
| [Details](http://mirdb.org/cgi-bin/target_detail.cgi?targetID=598005) | 494 | 70 | hsa-miR-148a-3p | [ZNF217](http://www.ncbi.nlm.nih.gov/entrez/query.fcgi?db=gene&cmd=Retrieve&dopt=full_report&list_uids=7764) | zinc finger protein 217 |
| [Details](http://mirdb.org/cgi-bin/target_detail.cgi?targetID=598204) | 495 | 70 | hsa-miR-148a-3p | [USP12](http://www.ncbi.nlm.nih.gov/entrez/query.fcgi?db=gene&cmd=Retrieve&dopt=full_report&list_uids=219333) | ubiquitin specific peptidase 12 |
| [Details](http://mirdb.org/cgi-bin/target_detail.cgi?targetID=598631) | 496 | 70 | hsa-miR-148a-3p | [DNAJB12](http://www.ncbi.nlm.nih.gov/entrez/query.fcgi?db=gene&cmd=Retrieve&dopt=full_report&list_uids=54788) | DnaJ heat shock protein family (Hsp40) member B12 |
| [Details](http://mirdb.org/cgi-bin/target_detail.cgi?targetID=598509) | 497 | 69 | hsa-miR-148a-3p | [STXBP5L](http://www.ncbi.nlm.nih.gov/entrez/query.fcgi?db=gene&cmd=Retrieve&dopt=full_report&list_uids=9515) | syntaxin binding protein 5 like |
| [Details](http://mirdb.org/cgi-bin/target_detail.cgi?targetID=598367) | 498 | 69 | hsa-miR-148a-3p | [KRT85](http://www.ncbi.nlm.nih.gov/entrez/query.fcgi?db=gene&cmd=Retrieve&dopt=full_report&list_uids=3891) | keratin 85 |
| [Details](http://mirdb.org/cgi-bin/target_detail.cgi?targetID=598479) | 499 | 69 | hsa-miR-148a-3p | [RNASEL](http://www.ncbi.nlm.nih.gov/entrez/query.fcgi?db=gene&cmd=Retrieve&dopt=full_report&list_uids=6041) | ribonuclease L |
| [Details](http://mirdb.org/cgi-bin/target_detail.cgi?targetID=598681) | 500 | 69 | hsa-miR-148a-3p | [SLC38A2](http://www.ncbi.nlm.nih.gov/entrez/query.fcgi?db=gene&cmd=Retrieve&dopt=full_report&list_uids=54407) | solute carrier family 38 member 2 |
| [Details](http://mirdb.org/cgi-bin/target_detail.cgi?targetID=598392) | 501 | 69 | hsa-miR-148a-3p | [LAMB2](http://www.ncbi.nlm.nih.gov/entrez/query.fcgi?db=gene&cmd=Retrieve&dopt=full_report&list_uids=3913) | laminin subunit beta 2 |
| [Details](http://mirdb.org/cgi-bin/target_detail.cgi?targetID=598662) | 502 | 69 | hsa-miR-148a-3p | [MATN3](http://www.ncbi.nlm.nih.gov/entrez/query.fcgi?db=gene&cmd=Retrieve&dopt=full_report&list_uids=4148) | matrilin 3 |
| [Details](http://mirdb.org/cgi-bin/target_detail.cgi?targetID=598326) | 503 | 69 | hsa-miR-148a-3p | [HIF1AN](http://www.ncbi.nlm.nih.gov/entrez/query.fcgi?db=gene&cmd=Retrieve&dopt=full_report&list_uids=55662) | hypoxia inducible factor 1 subunit alpha inhibitor |
| [Details](http://mirdb.org/cgi-bin/target_detail.cgi?targetID=597955) | 504 | 69 | hsa-miR-148a-3p | [SLF2](http://www.ncbi.nlm.nih.gov/entrez/query.fcgi?db=gene&cmd=Retrieve&dopt=full_report&list_uids=55719) | SMC5-SMC6 complex localization factor 2 |
| [Details](http://mirdb.org/cgi-bin/target_detail.cgi?targetID=598298) | 505 | 69 | hsa-miR-148a-3p | [GTPBP10](http://www.ncbi.nlm.nih.gov/entrez/query.fcgi?db=gene&cmd=Retrieve&dopt=full_report&list_uids=85865) | GTP binding protein 10 |
| [Details](http://mirdb.org/cgi-bin/target_detail.cgi?targetID=598216) | 506 | 69 | hsa-miR-148a-3p | [CPD](http://www.ncbi.nlm.nih.gov/entrez/query.fcgi?db=gene&cmd=Retrieve&dopt=full_report&list_uids=1362) | carboxypeptidase D |
| [Details](http://mirdb.org/cgi-bin/target_detail.cgi?targetID=597886) | 507 | 69 | hsa-miR-148a-3p | [MRPS25](http://www.ncbi.nlm.nih.gov/entrez/query.fcgi?db=gene&cmd=Retrieve&dopt=full_report&list_uids=64432) | mitochondrial ribosomal protein S25 |
| [Details](http://mirdb.org/cgi-bin/target_detail.cgi?targetID=598277) | 508 | 69 | hsa-miR-148a-3p | [TUBE1](http://www.ncbi.nlm.nih.gov/entrez/query.fcgi?db=gene&cmd=Retrieve&dopt=full_report&list_uids=51175) | tubulin epsilon 1 |
| [Details](http://mirdb.org/cgi-bin/target_detail.cgi?targetID=598482) | 509 | 69 | hsa-miR-148a-3p | [PQLC3](http://www.ncbi.nlm.nih.gov/entrez/query.fcgi?db=gene&cmd=Retrieve&dopt=full_report&list_uids=130814) | PQ loop repeat containing 3 |
| [Details](http://mirdb.org/cgi-bin/target_detail.cgi?targetID=597887) | 510 | 69 | hsa-miR-148a-3p | [MAGI1](http://www.ncbi.nlm.nih.gov/entrez/query.fcgi?db=gene&cmd=Retrieve&dopt=full_report&list_uids=9223) | membrane associated guanylate kinase, WW and PDZ domain containing 1 |
| [Details](http://mirdb.org/cgi-bin/target_detail.cgi?targetID=598087) | 511 | 69 | hsa-miR-148a-3p | [TIMM23](http://www.ncbi.nlm.nih.gov/entrez/query.fcgi?db=gene&cmd=Retrieve&dopt=full_report&list_uids=100287932) | translocase of inner mitochondrial membrane 23 |
| [Details](http://mirdb.org/cgi-bin/target_detail.cgi?targetID=597999) | 512 | 69 | hsa-miR-148a-3p | [MOB1B](http://www.ncbi.nlm.nih.gov/entrez/query.fcgi?db=gene&cmd=Retrieve&dopt=full_report&list_uids=92597) | MOB kinase activator 1B |
| [Details](http://mirdb.org/cgi-bin/target_detail.cgi?targetID=598083) | 513 | 69 | hsa-miR-148a-3p | [OXSR1](http://www.ncbi.nlm.nih.gov/entrez/query.fcgi?db=gene&cmd=Retrieve&dopt=full_report&list_uids=9943) | oxidative stress responsive kinase 1 |
| [Details](http://mirdb.org/cgi-bin/target_detail.cgi?targetID=598028) | 514 | 69 | hsa-miR-148a-3p | [CYREN](http://www.ncbi.nlm.nih.gov/entrez/query.fcgi?db=gene&cmd=Retrieve&dopt=full_report&list_uids=78996) | cell cycle regulator of NHEJ |
| [Details](http://mirdb.org/cgi-bin/target_detail.cgi?targetID=598081) | 515 | 69 | hsa-miR-148a-3p | [AK4](http://www.ncbi.nlm.nih.gov/entrez/query.fcgi?db=gene&cmd=Retrieve&dopt=full_report&list_uids=205) | adenylate kinase 4 |
| [Details](http://mirdb.org/cgi-bin/target_detail.cgi?targetID=598209) | 516 | 68 | hsa-miR-148a-3p | [RUFY2](http://www.ncbi.nlm.nih.gov/entrez/query.fcgi?db=gene&cmd=Retrieve&dopt=full_report&list_uids=55680) | RUN and FYVE domain containing 2 |
| [Details](http://mirdb.org/cgi-bin/target_detail.cgi?targetID=598152) | 517 | 68 | hsa-miR-148a-3p | [UBXN1](http://www.ncbi.nlm.nih.gov/entrez/query.fcgi?db=gene&cmd=Retrieve&dopt=full_report&list_uids=51035) | UBX domain protein 1 |
| [Details](http://mirdb.org/cgi-bin/target_detail.cgi?targetID=598382) | 518 | 68 | hsa-miR-148a-3p | [TMEM187](http://www.ncbi.nlm.nih.gov/entrez/query.fcgi?db=gene&cmd=Retrieve&dopt=full_report&list_uids=8269) | transmembrane protein 187 |
| [Details](http://mirdb.org/cgi-bin/target_detail.cgi?targetID=598078) | 519 | 68 | hsa-miR-148a-3p | [ZNF736](http://www.ncbi.nlm.nih.gov/entrez/query.fcgi?db=gene&cmd=Retrieve&dopt=full_report&list_uids=728927) | zinc finger protein 736 |
| [Details](http://mirdb.org/cgi-bin/target_detail.cgi?targetID=598558) | 520 | 68 | hsa-miR-148a-3p | [DST](http://www.ncbi.nlm.nih.gov/entrez/query.fcgi?db=gene&cmd=Retrieve&dopt=full_report&list_uids=667) | dystonin |
| [Details](http://mirdb.org/cgi-bin/target_detail.cgi?targetID=598503) | 521 | 68 | hsa-miR-148a-3p | [IL6ST](http://www.ncbi.nlm.nih.gov/entrez/query.fcgi?db=gene&cmd=Retrieve&dopt=full_report&list_uids=3572) | interleukin 6 signal transducer |
| [Details](http://mirdb.org/cgi-bin/target_detail.cgi?targetID=598539) | 522 | 68 | hsa-miR-148a-3p | [NOVA2](http://www.ncbi.nlm.nih.gov/entrez/query.fcgi?db=gene&cmd=Retrieve&dopt=full_report&list_uids=4858) | NOVA alternative splicing regulator 2 |
| [Details](http://mirdb.org/cgi-bin/target_detail.cgi?targetID=597937) | 523 | 68 | hsa-miR-148a-3p | [GABPB1](http://www.ncbi.nlm.nih.gov/entrez/query.fcgi?db=gene&cmd=Retrieve&dopt=full_report&list_uids=2553) | GA binding protein transcription factor subunit beta 1 |
| [Details](http://mirdb.org/cgi-bin/target_detail.cgi?targetID=598637) | 524 | 68 | hsa-miR-148a-3p | [KPNB1](http://www.ncbi.nlm.nih.gov/entrez/query.fcgi?db=gene&cmd=Retrieve&dopt=full_report&list_uids=3837) | karyopherin subunit beta 1 |
| [Details](http://mirdb.org/cgi-bin/target_detail.cgi?targetID=598309) | 525 | 68 | hsa-miR-148a-3p | [FAM184A](http://www.ncbi.nlm.nih.gov/entrez/query.fcgi?db=gene&cmd=Retrieve&dopt=full_report&list_uids=79632) | family with sequence similarity 184 member A |
| [Details](http://mirdb.org/cgi-bin/target_detail.cgi?targetID=598361) | 526 | 68 | hsa-miR-148a-3p | [NUCKS1](http://www.ncbi.nlm.nih.gov/entrez/query.fcgi?db=gene&cmd=Retrieve&dopt=full_report&list_uids=64710) | nuclear casein kinase and cyclin dependent kinase substrate 1 |
| [Details](http://mirdb.org/cgi-bin/target_detail.cgi?targetID=597897) | 527 | 68 | hsa-miR-148a-3p | [NUP133](http://www.ncbi.nlm.nih.gov/entrez/query.fcgi?db=gene&cmd=Retrieve&dopt=full_report&list_uids=55746) | nucleoporin 133 |
| [Details](http://mirdb.org/cgi-bin/target_detail.cgi?targetID=597901) | 528 | 68 | hsa-miR-148a-3p | [RNF217](http://www.ncbi.nlm.nih.gov/entrez/query.fcgi?db=gene&cmd=Retrieve&dopt=full_report&list_uids=154214) | ring finger protein 217 |
| [Details](http://mirdb.org/cgi-bin/target_detail.cgi?targetID=597874) | 529 | 68 | hsa-miR-148a-3p | [ZNF445](http://www.ncbi.nlm.nih.gov/entrez/query.fcgi?db=gene&cmd=Retrieve&dopt=full_report&list_uids=353274) | zinc finger protein 445 |
| [Details](http://mirdb.org/cgi-bin/target_detail.cgi?targetID=597873) | 530 | 68 | hsa-miR-148a-3p | [HMGA2](http://www.ncbi.nlm.nih.gov/entrez/query.fcgi?db=gene&cmd=Retrieve&dopt=full_report&list_uids=8091) | high mobility group AT-hook 2 |
| [Details](http://mirdb.org/cgi-bin/target_detail.cgi?targetID=598127) | 531 | 67 | hsa-miR-148a-3p | [KCNH1](http://www.ncbi.nlm.nih.gov/entrez/query.fcgi?db=gene&cmd=Retrieve&dopt=full_report&list_uids=3756) | potassium voltage-gated channel subfamily H member 1 |
| [Details](http://mirdb.org/cgi-bin/target_detail.cgi?targetID=598484) | 532 | 67 | hsa-miR-148a-3p | [IRS1](http://www.ncbi.nlm.nih.gov/entrez/query.fcgi?db=gene&cmd=Retrieve&dopt=full_report&list_uids=3667) | insulin receptor substrate 1 |
| [Details](http://mirdb.org/cgi-bin/target_detail.cgi?targetID=598146) | 533 | 67 | hsa-miR-148a-3p | [ADAMTS5](http://www.ncbi.nlm.nih.gov/entrez/query.fcgi?db=gene&cmd=Retrieve&dopt=full_report&list_uids=11096) | ADAM metallopeptidase with thrombospondin type 1 motif 5 |
| [Details](http://mirdb.org/cgi-bin/target_detail.cgi?targetID=598400) | 534 | 67 | hsa-miR-148a-3p | [RUNX1T1](http://www.ncbi.nlm.nih.gov/entrez/query.fcgi?db=gene&cmd=Retrieve&dopt=full_report&list_uids=862) | RUNX1 translocation partner 1 |
| [Details](http://mirdb.org/cgi-bin/target_detail.cgi?targetID=598426) | 535 | 67 | hsa-miR-148a-3p | [LYSMD3](http://www.ncbi.nlm.nih.gov/entrez/query.fcgi?db=gene&cmd=Retrieve&dopt=full_report&list_uids=116068) | LysM domain containing 3 |
| [Details](http://mirdb.org/cgi-bin/target_detail.cgi?targetID=598435) | 536 | 67 | hsa-miR-148a-3p | [PRDM15](http://www.ncbi.nlm.nih.gov/entrez/query.fcgi?db=gene&cmd=Retrieve&dopt=full_report&list_uids=63977) | PR/SET domain 15 |
| [Details](http://mirdb.org/cgi-bin/target_detail.cgi?targetID=598616) | 537 | 67 | hsa-miR-148a-3p | [MRPL37](http://www.ncbi.nlm.nih.gov/entrez/query.fcgi?db=gene&cmd=Retrieve&dopt=full_report&list_uids=51253) | mitochondrial ribosomal protein L37 |
| [Details](http://mirdb.org/cgi-bin/target_detail.cgi?targetID=597879) | 538 | 67 | hsa-miR-148a-3p | [MAGIX](http://www.ncbi.nlm.nih.gov/entrez/query.fcgi?db=gene&cmd=Retrieve&dopt=full_report&list_uids=79917) | MAGI family member, X-linked |
| [Details](http://mirdb.org/cgi-bin/target_detail.cgi?targetID=598596) | 539 | 67 | hsa-miR-148a-3p | [COLEC10](http://www.ncbi.nlm.nih.gov/entrez/query.fcgi?db=gene&cmd=Retrieve&dopt=full_report&list_uids=10584) | collectin subfamily member 10 |
| [Details](http://mirdb.org/cgi-bin/target_detail.cgi?targetID=598029) | 540 | 67 | hsa-miR-148a-3p | [CNIH4](http://www.ncbi.nlm.nih.gov/entrez/query.fcgi?db=gene&cmd=Retrieve&dopt=full_report&list_uids=29097) | cornichon family AMPA receptor auxiliary protein 4 |
| [Details](http://mirdb.org/cgi-bin/target_detail.cgi?targetID=597923) | 541 | 67 | hsa-miR-148a-3p | [STAP1](http://www.ncbi.nlm.nih.gov/entrez/query.fcgi?db=gene&cmd=Retrieve&dopt=full_report&list_uids=26228) | signal transducing adaptor family member 1 |
| [Details](http://mirdb.org/cgi-bin/target_detail.cgi?targetID=597968) | 542 | 67 | hsa-miR-148a-3p | [ARHGAP20](http://www.ncbi.nlm.nih.gov/entrez/query.fcgi?db=gene&cmd=Retrieve&dopt=full_report&list_uids=57569) | Rho GTPase activating protein 20 |
| [Details](http://mirdb.org/cgi-bin/target_detail.cgi?targetID=597857) | 543 | 66 | hsa-miR-148a-3p | [VASH2](http://www.ncbi.nlm.nih.gov/entrez/query.fcgi?db=gene&cmd=Retrieve&dopt=full_report&list_uids=79805) | vasohibin 2 |
| [Details](http://mirdb.org/cgi-bin/target_detail.cgi?targetID=598055) | 544 | 66 | hsa-miR-148a-3p | [EPG5](http://www.ncbi.nlm.nih.gov/entrez/query.fcgi?db=gene&cmd=Retrieve&dopt=full_report&list_uids=57724) | ectopic P-granules autophagy protein 5 homolog |
| [Details](http://mirdb.org/cgi-bin/target_detail.cgi?targetID=598577) | 545 | 66 | hsa-miR-148a-3p | [C16orf91](http://www.ncbi.nlm.nih.gov/entrez/query.fcgi?db=gene&cmd=Retrieve&dopt=full_report&list_uids=283951) | chromosome 16 open reading frame 91 |
| [Details](http://mirdb.org/cgi-bin/target_detail.cgi?targetID=598587) | 546 | 66 | hsa-miR-148a-3p | [ZBTB20](http://www.ncbi.nlm.nih.gov/entrez/query.fcgi?db=gene&cmd=Retrieve&dopt=full_report&list_uids=26137) | zinc finger and BTB domain containing 20 |
| [Details](http://mirdb.org/cgi-bin/target_detail.cgi?targetID=598232) | 547 | 66 | hsa-miR-148a-3p | [CCDC85A](http://www.ncbi.nlm.nih.gov/entrez/query.fcgi?db=gene&cmd=Retrieve&dopt=full_report&list_uids=114800) | coiled-coil domain containing 85A |
| [Details](http://mirdb.org/cgi-bin/target_detail.cgi?targetID=598483) | 548 | 66 | hsa-miR-148a-3p | [ZNF605](http://www.ncbi.nlm.nih.gov/entrez/query.fcgi?db=gene&cmd=Retrieve&dopt=full_report&list_uids=100289635) | zinc finger protein 605 |
| [Details](http://mirdb.org/cgi-bin/target_detail.cgi?targetID=598177) | 549 | 66 | hsa-miR-148a-3p | [EPDR1](http://www.ncbi.nlm.nih.gov/entrez/query.fcgi?db=gene&cmd=Retrieve&dopt=full_report&list_uids=54749) | ependymin related 1 |
| [Details](http://mirdb.org/cgi-bin/target_detail.cgi?targetID=598067) | 550 | 66 | hsa-miR-148a-3p | [SIGLEC8](http://www.ncbi.nlm.nih.gov/entrez/query.fcgi?db=gene&cmd=Retrieve&dopt=full_report&list_uids=27181) | sialic acid binding Ig like lectin 8 |
| [Details](http://mirdb.org/cgi-bin/target_detail.cgi?targetID=598648) | 551 | 66 | hsa-miR-148a-3p | [NDUFC2-KCTD14](http://www.ncbi.nlm.nih.gov/entrez/query.fcgi?db=gene&cmd=Retrieve&dopt=full_report&list_uids=100532726) | NDUFC2-KCTD14 readthrough |
| [Details](http://mirdb.org/cgi-bin/target_detail.cgi?targetID=597926) | 552 | 66 | hsa-miR-148a-3p | [FBXL20](http://www.ncbi.nlm.nih.gov/entrez/query.fcgi?db=gene&cmd=Retrieve&dopt=full_report&list_uids=84961) | F-box and leucine rich repeat protein 20 |
| [Details](http://mirdb.org/cgi-bin/target_detail.cgi?targetID=598379) | 553 | 66 | hsa-miR-148a-3p | [IPCEF1](http://www.ncbi.nlm.nih.gov/entrez/query.fcgi?db=gene&cmd=Retrieve&dopt=full_report&list_uids=26034) | interaction protein for cytohesin exchange factors 1 |
| [Details](http://mirdb.org/cgi-bin/target_detail.cgi?targetID=598147) | 554 | 66 | hsa-miR-148a-3p | [UGT8](http://www.ncbi.nlm.nih.gov/entrez/query.fcgi?db=gene&cmd=Retrieve&dopt=full_report&list_uids=7368) | UDP glycosyltransferase 8 |
| [Details](http://mirdb.org/cgi-bin/target_detail.cgi?targetID=598337) | 555 | 66 | hsa-miR-148a-3p | [BHLHE41](http://www.ncbi.nlm.nih.gov/entrez/query.fcgi?db=gene&cmd=Retrieve&dopt=full_report&list_uids=79365) | basic helix-loop-helix family member e41 |
| [Details](http://mirdb.org/cgi-bin/target_detail.cgi?targetID=598162) | 556 | 66 | hsa-miR-148a-3p | [HSPA4L](http://www.ncbi.nlm.nih.gov/entrez/query.fcgi?db=gene&cmd=Retrieve&dopt=full_report&list_uids=22824) | heat shock protein family A (Hsp70) member 4 like |
| [Details](http://mirdb.org/cgi-bin/target_detail.cgi?targetID=598388) | 557 | 66 | hsa-miR-148a-3p | [TMSB4X](http://www.ncbi.nlm.nih.gov/entrez/query.fcgi?db=gene&cmd=Retrieve&dopt=full_report&list_uids=7114) | thymosin beta 4 X-linked |
| [Details](http://mirdb.org/cgi-bin/target_detail.cgi?targetID=598325) | 558 | 66 | hsa-miR-148a-3p | [FLT1](http://www.ncbi.nlm.nih.gov/entrez/query.fcgi?db=gene&cmd=Retrieve&dopt=full_report&list_uids=2321) | fms related tyrosine kinase 1 |
| [Details](http://mirdb.org/cgi-bin/target_detail.cgi?targetID=598218) | 559 | 66 | hsa-miR-148a-3p | [OTX2](http://www.ncbi.nlm.nih.gov/entrez/query.fcgi?db=gene&cmd=Retrieve&dopt=full_report&list_uids=5015) | orthodenticle homeobox 2 |
| [Details](http://mirdb.org/cgi-bin/target_detail.cgi?targetID=597985) | 560 | 66 | hsa-miR-148a-3p | [EPHA4](http://www.ncbi.nlm.nih.gov/entrez/query.fcgi?db=gene&cmd=Retrieve&dopt=full_report&list_uids=2043) | EPH receptor A4 |
| [Details](http://mirdb.org/cgi-bin/target_detail.cgi?targetID=598433) | 561 | 66 | hsa-miR-148a-3p | [TGFBRAP1](http://www.ncbi.nlm.nih.gov/entrez/query.fcgi?db=gene&cmd=Retrieve&dopt=full_report&list_uids=9392) | transforming growth factor beta receptor associated protein 1 |
| [Details](http://mirdb.org/cgi-bin/target_detail.cgi?targetID=598153) | 562 | 66 | hsa-miR-148a-3p | [ISM2](http://www.ncbi.nlm.nih.gov/entrez/query.fcgi?db=gene&cmd=Retrieve&dopt=full_report&list_uids=145501) | isthmin 2 |
| [Details](http://mirdb.org/cgi-bin/target_detail.cgi?targetID=598501) | 563 | 65 | hsa-miR-148a-3p | [TSPOAP1](http://www.ncbi.nlm.nih.gov/entrez/query.fcgi?db=gene&cmd=Retrieve&dopt=full_report&list_uids=9256) | TSPO associated protein 1 |
| [Details](http://mirdb.org/cgi-bin/target_detail.cgi?targetID=598456) | 564 | 65 | hsa-miR-148a-3p | [RAB9B](http://www.ncbi.nlm.nih.gov/entrez/query.fcgi?db=gene&cmd=Retrieve&dopt=full_report&list_uids=51209) | RAB9B, member RAS oncogene family |
| [Details](http://mirdb.org/cgi-bin/target_detail.cgi?targetID=598544) | 565 | 65 | hsa-miR-148a-3p | [WDR19](http://www.ncbi.nlm.nih.gov/entrez/query.fcgi?db=gene&cmd=Retrieve&dopt=full_report&list_uids=57728) | WD repeat domain 19 |
| [Details](http://mirdb.org/cgi-bin/target_detail.cgi?targetID=597953) | 566 | 65 | hsa-miR-148a-3p | [PHF24](http://www.ncbi.nlm.nih.gov/entrez/query.fcgi?db=gene&cmd=Retrieve&dopt=full_report&list_uids=23349) | PHD finger protein 24 |
| [Details](http://mirdb.org/cgi-bin/target_detail.cgi?targetID=598646) | 567 | 65 | hsa-miR-148a-3p | [PTPN14](http://www.ncbi.nlm.nih.gov/entrez/query.fcgi?db=gene&cmd=Retrieve&dopt=full_report&list_uids=5784) | protein tyrosine phosphatase, non-receptor type 14 |
| [Details](http://mirdb.org/cgi-bin/target_detail.cgi?targetID=598640) | 568 | 65 | hsa-miR-148a-3p | [PARPBP](http://www.ncbi.nlm.nih.gov/entrez/query.fcgi?db=gene&cmd=Retrieve&dopt=full_report&list_uids=55010) | PARP1 binding protein |
| [Details](http://mirdb.org/cgi-bin/target_detail.cgi?targetID=598341) | 569 | 65 | hsa-miR-148a-3p | [NCOR1](http://www.ncbi.nlm.nih.gov/entrez/query.fcgi?db=gene&cmd=Retrieve&dopt=full_report&list_uids=9611) | nuclear receptor corepressor 1 |
| [Details](http://mirdb.org/cgi-bin/target_detail.cgi?targetID=598288) | 570 | 65 | hsa-miR-148a-3p | [LARP1B](http://www.ncbi.nlm.nih.gov/entrez/query.fcgi?db=gene&cmd=Retrieve&dopt=full_report&list_uids=55132) | La ribonucleoprotein domain family member 1B |
| [Details](http://mirdb.org/cgi-bin/target_detail.cgi?targetID=597861) | 571 | 65 | hsa-miR-148a-3p | [FRY](http://www.ncbi.nlm.nih.gov/entrez/query.fcgi?db=gene&cmd=Retrieve&dopt=full_report&list_uids=10129) | FRY microtubule binding protein |
| [Details](http://mirdb.org/cgi-bin/target_detail.cgi?targetID=598397) | 572 | 65 | hsa-miR-148a-3p | [ATP2A2](http://www.ncbi.nlm.nih.gov/entrez/query.fcgi?db=gene&cmd=Retrieve&dopt=full_report&list_uids=488) | ATPase sarcoplasmic/endoplasmic reticulum Ca2+ transporting 2 |
| [Details](http://mirdb.org/cgi-bin/target_detail.cgi?targetID=598008) | 573 | 65 | hsa-miR-148a-3p | [UHRF1](http://www.ncbi.nlm.nih.gov/entrez/query.fcgi?db=gene&cmd=Retrieve&dopt=full_report&list_uids=29128) | ubiquitin like with PHD and ring finger domains 1 |
| [Details](http://mirdb.org/cgi-bin/target_detail.cgi?targetID=597972) | 574 | 65 | hsa-miR-148a-3p | [SBNO1](http://www.ncbi.nlm.nih.gov/entrez/query.fcgi?db=gene&cmd=Retrieve&dopt=full_report&list_uids=55206) | strawberry notch homolog 1 |
| [Details](http://mirdb.org/cgi-bin/target_detail.cgi?targetID=598061) | 575 | 65 | hsa-miR-148a-3p | [USP38](http://www.ncbi.nlm.nih.gov/entrez/query.fcgi?db=gene&cmd=Retrieve&dopt=full_report&list_uids=84640) | ubiquitin specific peptidase 38 |
| [Details](http://mirdb.org/cgi-bin/target_detail.cgi?targetID=598283) | 576 | 64 | hsa-miR-148a-3p | [WAPL](http://www.ncbi.nlm.nih.gov/entrez/query.fcgi?db=gene&cmd=Retrieve&dopt=full_report&list_uids=23063) | WAPL cohesin release factor |
| [Details](http://mirdb.org/cgi-bin/target_detail.cgi?targetID=597970) | 577 | 64 | hsa-miR-148a-3p | [RXYLT1](http://www.ncbi.nlm.nih.gov/entrez/query.fcgi?db=gene&cmd=Retrieve&dopt=full_report&list_uids=10329) | ribitol xylosyltransferase 1 |
| [Details](http://mirdb.org/cgi-bin/target_detail.cgi?targetID=598217) | 578 | 64 | hsa-miR-148a-3p | [RXFP1](http://www.ncbi.nlm.nih.gov/entrez/query.fcgi?db=gene&cmd=Retrieve&dopt=full_report&list_uids=59350) | relaxin family peptide receptor 1 |
| [Details](http://mirdb.org/cgi-bin/target_detail.cgi?targetID=598606) | 579 | 64 | hsa-miR-148a-3p | [NECTIN3](http://www.ncbi.nlm.nih.gov/entrez/query.fcgi?db=gene&cmd=Retrieve&dopt=full_report&list_uids=25945) | nectin cell adhesion molecule 3 |
| [Details](http://mirdb.org/cgi-bin/target_detail.cgi?targetID=598310) | 580 | 64 | hsa-miR-148a-3p | [NR2E1](http://www.ncbi.nlm.nih.gov/entrez/query.fcgi?db=gene&cmd=Retrieve&dopt=full_report&list_uids=7101) | nuclear receptor subfamily 2 group E member 1 |
| [Details](http://mirdb.org/cgi-bin/target_detail.cgi?targetID=598518) | 581 | 64 | hsa-miR-148a-3p | [BCCIP](http://www.ncbi.nlm.nih.gov/entrez/query.fcgi?db=gene&cmd=Retrieve&dopt=full_report&list_uids=56647) | BRCA2 and CDKN1A interacting protein |
| [Details](http://mirdb.org/cgi-bin/target_detail.cgi?targetID=598458) | 582 | 64 | hsa-miR-148a-3p | [TRIM45](http://www.ncbi.nlm.nih.gov/entrez/query.fcgi?db=gene&cmd=Retrieve&dopt=full_report&list_uids=80263) | tripartite motif containing 45 |
| [Details](http://mirdb.org/cgi-bin/target_detail.cgi?targetID=598193) | 583 | 64 | hsa-miR-148a-3p | [TRAK1](http://www.ncbi.nlm.nih.gov/entrez/query.fcgi?db=gene&cmd=Retrieve&dopt=full_report&list_uids=22906) | trafficking kinesin protein 1 |
| [Details](http://mirdb.org/cgi-bin/target_detail.cgi?targetID=598260) | 584 | 64 | hsa-miR-148a-3p | [PWWP3B](http://www.ncbi.nlm.nih.gov/entrez/query.fcgi?db=gene&cmd=Retrieve&dopt=full_report&list_uids=139221) | PWWP domain containing 3B |
| [Details](http://mirdb.org/cgi-bin/target_detail.cgi?targetID=598003) | 585 | 64 | hsa-miR-148a-3p | [KLC2](http://www.ncbi.nlm.nih.gov/entrez/query.fcgi?db=gene&cmd=Retrieve&dopt=full_report&list_uids=64837) | kinesin light chain 2 |
| [Details](http://mirdb.org/cgi-bin/target_detail.cgi?targetID=598424) | 586 | 64 | hsa-miR-148a-3p | [MLEC](http://www.ncbi.nlm.nih.gov/entrez/query.fcgi?db=gene&cmd=Retrieve&dopt=full_report&list_uids=9761) | malectin |
| [Details](http://mirdb.org/cgi-bin/target_detail.cgi?targetID=598674) | 587 | 64 | hsa-miR-148a-3p | [STEAP4](http://www.ncbi.nlm.nih.gov/entrez/query.fcgi?db=gene&cmd=Retrieve&dopt=full_report&list_uids=79689) | STEAP4 metalloreductase |
| [Details](http://mirdb.org/cgi-bin/target_detail.cgi?targetID=598399) | 588 | 64 | hsa-miR-148a-3p | [SLC22A5](http://www.ncbi.nlm.nih.gov/entrez/query.fcgi?db=gene&cmd=Retrieve&dopt=full_report&list_uids=6584) | solute carrier family 22 member 5 |
| [Details](http://mirdb.org/cgi-bin/target_detail.cgi?targetID=598394) | 589 | 64 | hsa-miR-148a-3p | [CNOT6](http://www.ncbi.nlm.nih.gov/entrez/query.fcgi?db=gene&cmd=Retrieve&dopt=full_report&list_uids=57472) | CCR4-NOT transcription complex subunit 6 |
| [Details](http://mirdb.org/cgi-bin/target_detail.cgi?targetID=597882) | 590 | 63 | hsa-miR-148a-3p | [PNRC2](http://www.ncbi.nlm.nih.gov/entrez/query.fcgi?db=gene&cmd=Retrieve&dopt=full_report&list_uids=55629) | proline rich nuclear receptor coactivator 2 |
| [Details](http://mirdb.org/cgi-bin/target_detail.cgi?targetID=597946) | 591 | 63 | hsa-miR-148a-3p | [LIX1](http://www.ncbi.nlm.nih.gov/entrez/query.fcgi?db=gene&cmd=Retrieve&dopt=full_report&list_uids=167410) | limb and CNS expressed 1 |
| [Details](http://mirdb.org/cgi-bin/target_detail.cgi?targetID=598614) | 592 | 63 | hsa-miR-148a-3p | [MYBL1](http://www.ncbi.nlm.nih.gov/entrez/query.fcgi?db=gene&cmd=Retrieve&dopt=full_report&list_uids=4603) | MYB proto-oncogene like 1 |
| [Details](http://mirdb.org/cgi-bin/target_detail.cgi?targetID=598551) | 593 | 63 | hsa-miR-148a-3p | [NCLN](http://www.ncbi.nlm.nih.gov/entrez/query.fcgi?db=gene&cmd=Retrieve&dopt=full_report&list_uids=56926) | nicalin |
| [Details](http://mirdb.org/cgi-bin/target_detail.cgi?targetID=598572) | 594 | 63 | hsa-miR-148a-3p | [SLC25A46](http://www.ncbi.nlm.nih.gov/entrez/query.fcgi?db=gene&cmd=Retrieve&dopt=full_report&list_uids=91137) | solute carrier family 25 member 46 |
| [Details](http://mirdb.org/cgi-bin/target_detail.cgi?targetID=597996) | 595 | 63 | hsa-miR-148a-3p | [SYNC](http://www.ncbi.nlm.nih.gov/entrez/query.fcgi?db=gene&cmd=Retrieve&dopt=full_report&list_uids=81493) | syncoilin, intermediate filament protein |
| [Details](http://mirdb.org/cgi-bin/target_detail.cgi?targetID=597993) | 596 | 63 | hsa-miR-148a-3p | [IKBKB](http://www.ncbi.nlm.nih.gov/entrez/query.fcgi?db=gene&cmd=Retrieve&dopt=full_report&list_uids=3551) | inhibitor of nuclear factor kappa B kinase subunit beta |
| [Details](http://mirdb.org/cgi-bin/target_detail.cgi?targetID=598091) | 597 | 63 | hsa-miR-148a-3p | [SLC24A4](http://www.ncbi.nlm.nih.gov/entrez/query.fcgi?db=gene&cmd=Retrieve&dopt=full_report&list_uids=123041) | solute carrier family 24 member 4 |
| [Details](http://mirdb.org/cgi-bin/target_detail.cgi?targetID=598522) | 598 | 63 | hsa-miR-148a-3p | [ABHD12](http://www.ncbi.nlm.nih.gov/entrez/query.fcgi?db=gene&cmd=Retrieve&dopt=full_report&list_uids=26090) | abhydrolase domain containing 12 |
| [Details](http://mirdb.org/cgi-bin/target_detail.cgi?targetID=598508) | 599 | 63 | hsa-miR-148a-3p | [PHF21B](http://www.ncbi.nlm.nih.gov/entrez/query.fcgi?db=gene&cmd=Retrieve&dopt=full_report&list_uids=112885) | PHD finger protein 21B |
| [Details](http://mirdb.org/cgi-bin/target_detail.cgi?targetID=598595) | 600 | 63 | hsa-miR-148a-3p | [PAPSS2](http://www.ncbi.nlm.nih.gov/entrez/query.fcgi?db=gene&cmd=Retrieve&dopt=full_report&list_uids=9060) | 3'-phosphoadenosine 5'-phosphosulfate synthase 2 |
| [Details](http://mirdb.org/cgi-bin/target_detail.cgi?targetID=598506) | 601 | 63 | hsa-miR-148a-3p | [MPPED2](http://www.ncbi.nlm.nih.gov/entrez/query.fcgi?db=gene&cmd=Retrieve&dopt=full_report&list_uids=744) | metallophosphoesterase domain containing 2 |
| [Details](http://mirdb.org/cgi-bin/target_detail.cgi?targetID=598097) | 602 | 63 | hsa-miR-148a-3p | [KAT14](http://www.ncbi.nlm.nih.gov/entrez/query.fcgi?db=gene&cmd=Retrieve&dopt=full_report&list_uids=57325) | lysine acetyltransferase 14 |
| [Details](http://mirdb.org/cgi-bin/target_detail.cgi?targetID=598327) | 603 | 63 | hsa-miR-148a-3p | [CHMP4B](http://www.ncbi.nlm.nih.gov/entrez/query.fcgi?db=gene&cmd=Retrieve&dopt=full_report&list_uids=128866) | charged multivesicular body protein 4B |
| [Details](http://mirdb.org/cgi-bin/target_detail.cgi?targetID=598254) | 604 | 63 | hsa-miR-148a-3p | [TMEM132B](http://www.ncbi.nlm.nih.gov/entrez/query.fcgi?db=gene&cmd=Retrieve&dopt=full_report&list_uids=114795) | transmembrane protein 132B |
| [Details](http://mirdb.org/cgi-bin/target_detail.cgi?targetID=598347) | 605 | 63 | hsa-miR-148a-3p | [ZNF704](http://www.ncbi.nlm.nih.gov/entrez/query.fcgi?db=gene&cmd=Retrieve&dopt=full_report&list_uids=619279) | zinc finger protein 704 |
| [Details](http://mirdb.org/cgi-bin/target_detail.cgi?targetID=598385) | 606 | 63 | hsa-miR-148a-3p | [ELMO1](http://www.ncbi.nlm.nih.gov/entrez/query.fcgi?db=gene&cmd=Retrieve&dopt=full_report&list_uids=9844) | engulfment and cell motility 1 |
| [Details](http://mirdb.org/cgi-bin/target_detail.cgi?targetID=598262) | 607 | 63 | hsa-miR-148a-3p | [RELCH](http://www.ncbi.nlm.nih.gov/entrez/query.fcgi?db=gene&cmd=Retrieve&dopt=full_report&list_uids=57614) | RAB11 binding and LisH domain, coiled-coil and HEAT repeat containing |
| [Details](http://mirdb.org/cgi-bin/target_detail.cgi?targetID=598523) | 608 | 62 | hsa-miR-148a-3p | [HLA-DQB2](http://www.ncbi.nlm.nih.gov/entrez/query.fcgi?db=gene&cmd=Retrieve&dopt=full_report&list_uids=3120) | major histocompatibility complex, class II, DQ beta 2 |
| [Details](http://mirdb.org/cgi-bin/target_detail.cgi?targetID=598057) | 609 | 62 | hsa-miR-148a-3p | [KRBOX4](http://www.ncbi.nlm.nih.gov/entrez/query.fcgi?db=gene&cmd=Retrieve&dopt=full_report&list_uids=55634) | KRAB box domain containing 4 |
| [Details](http://mirdb.org/cgi-bin/target_detail.cgi?targetID=598330) | 610 | 62 | hsa-miR-148a-3p | [NOL10](http://www.ncbi.nlm.nih.gov/entrez/query.fcgi?db=gene&cmd=Retrieve&dopt=full_report&list_uids=79954) | nucleolar protein 10 |
| [Details](http://mirdb.org/cgi-bin/target_detail.cgi?targetID=597900) | 611 | 62 | hsa-miR-148a-3p | [KRTAP4-5](http://www.ncbi.nlm.nih.gov/entrez/query.fcgi?db=gene&cmd=Retrieve&dopt=full_report&list_uids=85289) | keratin associated protein 4-5 |
| [Details](http://mirdb.org/cgi-bin/target_detail.cgi?targetID=597988) | 612 | 62 | hsa-miR-148a-3p | [RAD51B](http://www.ncbi.nlm.nih.gov/entrez/query.fcgi?db=gene&cmd=Retrieve&dopt=full_report&list_uids=5890) | RAD51 paralog B |
| [Details](http://mirdb.org/cgi-bin/target_detail.cgi?targetID=598389) | 613 | 62 | hsa-miR-148a-3p | [ZBTB1](http://www.ncbi.nlm.nih.gov/entrez/query.fcgi?db=gene&cmd=Retrieve&dopt=full_report&list_uids=22890) | zinc finger and BTB domain containing 1 |
| [Details](http://mirdb.org/cgi-bin/target_detail.cgi?targetID=597959) | 614 | 62 | hsa-miR-148a-3p | [SNRPD1](http://www.ncbi.nlm.nih.gov/entrez/query.fcgi?db=gene&cmd=Retrieve&dopt=full_report&list_uids=6632) | small nuclear ribonucleoprotein D1 polypeptide |
| [Details](http://mirdb.org/cgi-bin/target_detail.cgi?targetID=598250) | 615 | 62 | hsa-miR-148a-3p | [RASGRP1](http://www.ncbi.nlm.nih.gov/entrez/query.fcgi?db=gene&cmd=Retrieve&dopt=full_report&list_uids=10125) | RAS guanyl releasing protein 1 |
| [Details](http://mirdb.org/cgi-bin/target_detail.cgi?targetID=597948) | 616 | 62 | hsa-miR-148a-3p | [BCL11A](http://www.ncbi.nlm.nih.gov/entrez/query.fcgi?db=gene&cmd=Retrieve&dopt=full_report&list_uids=53335) | BCL11A, BAF complex component |
| [Details](http://mirdb.org/cgi-bin/target_detail.cgi?targetID=598620) | 617 | 62 | hsa-miR-148a-3p | [TNFRSF6B](http://www.ncbi.nlm.nih.gov/entrez/query.fcgi?db=gene&cmd=Retrieve&dopt=full_report&list_uids=8771) | TNF receptor superfamily member 6b |
| [Details](http://mirdb.org/cgi-bin/target_detail.cgi?targetID=598050) | 618 | 62 | hsa-miR-148a-3p | [ADPRH](http://www.ncbi.nlm.nih.gov/entrez/query.fcgi?db=gene&cmd=Retrieve&dopt=full_report&list_uids=141) | ADP-ribosylarginine hydrolase |
| [Details](http://mirdb.org/cgi-bin/target_detail.cgi?targetID=598649) | 619 | 62 | hsa-miR-148a-3p | [MEX3C](http://www.ncbi.nlm.nih.gov/entrez/query.fcgi?db=gene&cmd=Retrieve&dopt=full_report&list_uids=51320) | mex-3 RNA binding family member C |
| [Details](http://mirdb.org/cgi-bin/target_detail.cgi?targetID=598427) | 620 | 62 | hsa-miR-148a-3p | [GLG1](http://www.ncbi.nlm.nih.gov/entrez/query.fcgi?db=gene&cmd=Retrieve&dopt=full_report&list_uids=2734) | golgi glycoprotein 1 |
| [Details](http://mirdb.org/cgi-bin/target_detail.cgi?targetID=598547) | 621 | 61 | hsa-miR-148a-3p | [BLCAP](http://www.ncbi.nlm.nih.gov/entrez/query.fcgi?db=gene&cmd=Retrieve&dopt=full_report&list_uids=10904) | BLCAP, apoptosis inducing factor |
| [Details](http://mirdb.org/cgi-bin/target_detail.cgi?targetID=597913) | 622 | 61 | hsa-miR-148a-3p | [ZPLD1](http://www.ncbi.nlm.nih.gov/entrez/query.fcgi?db=gene&cmd=Retrieve&dopt=full_report&list_uids=131368) | zona pellucida like domain containing 1 |
| [Details](http://mirdb.org/cgi-bin/target_detail.cgi?targetID=598052) | 623 | 61 | hsa-miR-148a-3p | [MTF1](http://www.ncbi.nlm.nih.gov/entrez/query.fcgi?db=gene&cmd=Retrieve&dopt=full_report&list_uids=4520) | metal regulatory transcription factor 1 |
| [Details](http://mirdb.org/cgi-bin/target_detail.cgi?targetID=598090) | 624 | 61 | hsa-miR-148a-3p | [TET2](http://www.ncbi.nlm.nih.gov/entrez/query.fcgi?db=gene&cmd=Retrieve&dopt=full_report&list_uids=54790) | tet methylcytosine dioxygenase 2 |
| [Details](http://mirdb.org/cgi-bin/target_detail.cgi?targetID=598094) | 625 | 61 | hsa-miR-148a-3p | [PAPPA](http://www.ncbi.nlm.nih.gov/entrez/query.fcgi?db=gene&cmd=Retrieve&dopt=full_report&list_uids=5069) | pappalysin 1 |
| [Details](http://mirdb.org/cgi-bin/target_detail.cgi?targetID=597940) | 626 | 61 | hsa-miR-148a-3p | [MYO19](http://www.ncbi.nlm.nih.gov/entrez/query.fcgi?db=gene&cmd=Retrieve&dopt=full_report&list_uids=80179) | myosin XIX |
| [Details](http://mirdb.org/cgi-bin/target_detail.cgi?targetID=598207) | 627 | 61 | hsa-miR-148a-3p | [MAF1](http://www.ncbi.nlm.nih.gov/entrez/query.fcgi?db=gene&cmd=Retrieve&dopt=full_report&list_uids=84232) | MAF1 homolog, negative regulator of RNA polymerase III |
| [Details](http://mirdb.org/cgi-bin/target_detail.cgi?targetID=598033) | 628 | 61 | hsa-miR-148a-3p | [COL10A1](http://www.ncbi.nlm.nih.gov/entrez/query.fcgi?db=gene&cmd=Retrieve&dopt=full_report&list_uids=1300) | collagen type X alpha 1 chain |
| [Details](http://mirdb.org/cgi-bin/target_detail.cgi?targetID=598188) | 629 | 61 | hsa-miR-148a-3p | [PDK4](http://www.ncbi.nlm.nih.gov/entrez/query.fcgi?db=gene&cmd=Retrieve&dopt=full_report&list_uids=5166) | pyruvate dehydrogenase kinase 4 |
| [Details](http://mirdb.org/cgi-bin/target_detail.cgi?targetID=598238) | 630 | 61 | hsa-miR-148a-3p | [RPL36A](http://www.ncbi.nlm.nih.gov/entrez/query.fcgi?db=gene&cmd=Retrieve&dopt=full_report&list_uids=6173) | ribosomal protein L36a |
| [Details](http://mirdb.org/cgi-bin/target_detail.cgi?targetID=598548) | 631 | 61 | hsa-miR-148a-3p | [C5orf24](http://www.ncbi.nlm.nih.gov/entrez/query.fcgi?db=gene&cmd=Retrieve&dopt=full_report&list_uids=134553) | chromosome 5 open reading frame 24 |
| [Details](http://mirdb.org/cgi-bin/target_detail.cgi?targetID=598045) | 632 | 61 | hsa-miR-148a-3p | [SENP6](http://www.ncbi.nlm.nih.gov/entrez/query.fcgi?db=gene&cmd=Retrieve&dopt=full_report&list_uids=26054) | SUMO specific peptidase 6 |
| [Details](http://mirdb.org/cgi-bin/target_detail.cgi?targetID=598676) | 633 | 61 | hsa-miR-148a-3p | [STK38L](http://www.ncbi.nlm.nih.gov/entrez/query.fcgi?db=gene&cmd=Retrieve&dopt=full_report&list_uids=23012) | serine/threonine kinase 38 like |
| [Details](http://mirdb.org/cgi-bin/target_detail.cgi?targetID=598562) | 634 | 61 | hsa-miR-148a-3p | [DCAF5](http://www.ncbi.nlm.nih.gov/entrez/query.fcgi?db=gene&cmd=Retrieve&dopt=full_report&list_uids=8816) | DDB1 and CUL4 associated factor 5 |
| [Details](http://mirdb.org/cgi-bin/target_detail.cgi?targetID=598166) | 635 | 61 | hsa-miR-148a-3p | [PDE4B](http://www.ncbi.nlm.nih.gov/entrez/query.fcgi?db=gene&cmd=Retrieve&dopt=full_report&list_uids=5142) | phosphodiesterase 4B |
| [Details](http://mirdb.org/cgi-bin/target_detail.cgi?targetID=598109) | 636 | 61 | hsa-miR-148a-3p | [YTHDC1](http://www.ncbi.nlm.nih.gov/entrez/query.fcgi?db=gene&cmd=Retrieve&dopt=full_report&list_uids=91746) | YTH domain containing 1 |
| [Details](http://mirdb.org/cgi-bin/target_detail.cgi?targetID=598586) | 637 | 60 | hsa-miR-148a-3p | [CDON](http://www.ncbi.nlm.nih.gov/entrez/query.fcgi?db=gene&cmd=Retrieve&dopt=full_report&list_uids=50937) | cell adhesion associated, oncogene regulated |
| [Details](http://mirdb.org/cgi-bin/target_detail.cgi?targetID=597939) | 638 | 60 | hsa-miR-148a-3p | [ZBED4](http://www.ncbi.nlm.nih.gov/entrez/query.fcgi?db=gene&cmd=Retrieve&dopt=full_report&list_uids=9889) | zinc finger BED-type containing 4 |
| [Details](http://mirdb.org/cgi-bin/target_detail.cgi?targetID=597973) | 639 | 60 | hsa-miR-148a-3p | [INSM2](http://www.ncbi.nlm.nih.gov/entrez/query.fcgi?db=gene&cmd=Retrieve&dopt=full_report&list_uids=84684) | INSM transcriptional repressor 2 |
| [Details](http://mirdb.org/cgi-bin/target_detail.cgi?targetID=598313) | 640 | 60 | hsa-miR-148a-3p | [ERC2](http://www.ncbi.nlm.nih.gov/entrez/query.fcgi?db=gene&cmd=Retrieve&dopt=full_report&list_uids=26059) | ELKS/RAB6-interacting/CAST family member 2 |
| [Details](http://mirdb.org/cgi-bin/target_detail.cgi?targetID=597949) | 641 | 60 | hsa-miR-148a-3p | [ADGRE1](http://www.ncbi.nlm.nih.gov/entrez/query.fcgi?db=gene&cmd=Retrieve&dopt=full_report&list_uids=2015) | adhesion G protein-coupled receptor E1 |
| [Details](http://mirdb.org/cgi-bin/target_detail.cgi?targetID=598105) | 642 | 60 | hsa-miR-148a-3p | [MBOAT2](http://www.ncbi.nlm.nih.gov/entrez/query.fcgi?db=gene&cmd=Retrieve&dopt=full_report&list_uids=129642) | membrane bound O-acyltransferase domain containing 2 |
| [Details](http://mirdb.org/cgi-bin/target_detail.cgi?targetID=598271) | 643 | 60 | hsa-miR-148a-3p | [MAP1B](http://www.ncbi.nlm.nih.gov/entrez/query.fcgi?db=gene&cmd=Retrieve&dopt=full_report&list_uids=4131) | microtubule associated protein 1B |
| [Details](http://mirdb.org/cgi-bin/target_detail.cgi?targetID=598612) | 644 | 60 | hsa-miR-148a-3p | [NREP](http://www.ncbi.nlm.nih.gov/entrez/query.fcgi?db=gene&cmd=Retrieve&dopt=full_report&list_uids=9315) | neuronal regeneration related protein |
| [Details](http://mirdb.org/cgi-bin/target_detail.cgi?targetID=598514) | 645 | 60 | hsa-miR-148a-3p | [RBM38](http://www.ncbi.nlm.nih.gov/entrez/query.fcgi?db=gene&cmd=Retrieve&dopt=full_report&list_uids=55544) | RNA binding motif protein 38 |
| [Details](http://mirdb.org/cgi-bin/target_detail.cgi?targetID=598199) | 646 | 60 | hsa-miR-148a-3p | [S1PR2](http://www.ncbi.nlm.nih.gov/entrez/query.fcgi?db=gene&cmd=Retrieve&dopt=full_report&list_uids=9294) | sphingosine-1-phosphate receptor 2 |
| [Details](http://mirdb.org/cgi-bin/target_detail.cgi?targetID=597952) | 647 | 60 | hsa-miR-148a-3p | [PANX1](http://www.ncbi.nlm.nih.gov/entrez/query.fcgi?db=gene&cmd=Retrieve&dopt=full_report&list_uids=24145) | pannexin 1 |
| [Details](http://mirdb.org/cgi-bin/target_detail.cgi?targetID=598471) | 648 | 60 | hsa-miR-148a-3p | [COLEC12](http://www.ncbi.nlm.nih.gov/entrez/query.fcgi?db=gene&cmd=Retrieve&dopt=full_report&list_uids=81035) | collectin subfamily member 12 |
| [Details](http://mirdb.org/cgi-bin/target_detail.cgi?targetID=598184) | 649 | 60 | hsa-miR-148a-3p | [C11orf53](http://www.ncbi.nlm.nih.gov/entrez/query.fcgi?db=gene&cmd=Retrieve&dopt=full_report&list_uids=341032) | chromosome 11 open reading frame 53 |
| [Details](http://mirdb.org/cgi-bin/target_detail.cgi?targetID=598178) | 650 | 60 | hsa-miR-148a-3p | [PSMD1](http://www.ncbi.nlm.nih.gov/entrez/query.fcgi?db=gene&cmd=Retrieve&dopt=full_report&list_uids=5707) | proteasome 26S subunit, non-ATPase 1 |
| [Details](http://mirdb.org/cgi-bin/target_detail.cgi?targetID=598058) | 651 | 60 | hsa-miR-148a-3p | [BBC3](http://www.ncbi.nlm.nih.gov/entrez/query.fcgi?db=gene&cmd=Retrieve&dopt=full_report&list_uids=27113) | BCL2 binding component 3 |
| [Details](http://mirdb.org/cgi-bin/target_detail.cgi?targetID=598230) | 652 | 60 | hsa-miR-148a-3p | [PRRG4](http://www.ncbi.nlm.nih.gov/entrez/query.fcgi?db=gene&cmd=Retrieve&dopt=full_report&list_uids=79056) | proline rich and Gla domain 4 |
| [Details](http://mirdb.org/cgi-bin/target_detail.cgi?targetID=598196) | 653 | 60 | hsa-miR-148a-3p | [LRPPRC](http://www.ncbi.nlm.nih.gov/entrez/query.fcgi?db=gene&cmd=Retrieve&dopt=full_report&list_uids=10128) | leucine rich pentatricopeptide repeat containing |
| [Details](http://mirdb.org/cgi-bin/target_detail.cgi?targetID=598488) | 654 | 60 | hsa-miR-148a-3p | [MAB21L3](http://www.ncbi.nlm.nih.gov/entrez/query.fcgi?db=gene&cmd=Retrieve&dopt=full_report&list_uids=126868) | mab-21 like 3 |
| [Details](http://mirdb.org/cgi-bin/target_detail.cgi?targetID=598651) | 655 | 60 | hsa-miR-148a-3p | [ASAP1](http://www.ncbi.nlm.nih.gov/entrez/query.fcgi?db=gene&cmd=Retrieve&dopt=full_report&list_uids=50807) | ArfGAP with SH3 domain, ankyrin repeat and PH domain 1 |
| [Details](http://mirdb.org/cgi-bin/target_detail.cgi?targetID=598465) | 656 | 60 | hsa-miR-148a-3p | [EMG1](http://www.ncbi.nlm.nih.gov/entrez/query.fcgi?db=gene&cmd=Retrieve&dopt=full_report&list_uids=10436) | EMG1, N1-specific pseudouridine methyltransferase |
| [Details](http://mirdb.org/cgi-bin/target_detail.cgi?targetID=598455) | 657 | 60 | hsa-miR-148a-3p | [EMX2](http://www.ncbi.nlm.nih.gov/entrez/query.fcgi?db=gene&cmd=Retrieve&dopt=full_report&list_uids=2018) | empty spiracles homeobox 2 |
| [Details](http://mirdb.org/cgi-bin/target_detail.cgi?targetID=598156) | 658 | 60 | hsa-miR-148a-3p | [SYT1](http://www.ncbi.nlm.nih.gov/entrez/query.fcgi?db=gene&cmd=Retrieve&dopt=full_report&list_uids=6857) | synaptotagmin 1 |
| [Details](http://mirdb.org/cgi-bin/target_detail.cgi?targetID=597854) | 659 | 59 | hsa-miR-148a-3p | [MRE11](http://www.ncbi.nlm.nih.gov/entrez/query.fcgi?db=gene&cmd=Retrieve&dopt=full_report&list_uids=4361) | MRE11 homolog, double strand break repair nuclease |
| [Details](http://mirdb.org/cgi-bin/target_detail.cgi?targetID=598415) | 660 | 59 | hsa-miR-148a-3p | [PTGER3](http://www.ncbi.nlm.nih.gov/entrez/query.fcgi?db=gene&cmd=Retrieve&dopt=full_report&list_uids=5733) | prostaglandin E receptor 3 |
| [Details](http://mirdb.org/cgi-bin/target_detail.cgi?targetID=598474) | 661 | 59 | hsa-miR-148a-3p | [MOB1A](http://www.ncbi.nlm.nih.gov/entrez/query.fcgi?db=gene&cmd=Retrieve&dopt=full_report&list_uids=55233) | MOB kinase activator 1A |
| [Details](http://mirdb.org/cgi-bin/target_detail.cgi?targetID=598584) | 662 | 59 | hsa-miR-148a-3p | [ROGDI](http://www.ncbi.nlm.nih.gov/entrez/query.fcgi?db=gene&cmd=Retrieve&dopt=full_report&list_uids=79641) | rogdi atypical leucine zipper |
| [Details](http://mirdb.org/cgi-bin/target_detail.cgi?targetID=597981) | 663 | 59 | hsa-miR-148a-3p | [ARID3A](http://www.ncbi.nlm.nih.gov/entrez/query.fcgi?db=gene&cmd=Retrieve&dopt=full_report&list_uids=1820) | AT-rich interaction domain 3A |
| [Details](http://mirdb.org/cgi-bin/target_detail.cgi?targetID=598461) | 664 | 59 | hsa-miR-148a-3p | [EAPP](http://www.ncbi.nlm.nih.gov/entrez/query.fcgi?db=gene&cmd=Retrieve&dopt=full_report&list_uids=55837) | E2F associated phosphoprotein |
| [Details](http://mirdb.org/cgi-bin/target_detail.cgi?targetID=598179) | 665 | 59 | hsa-miR-148a-3p | [RAB12](http://www.ncbi.nlm.nih.gov/entrez/query.fcgi?db=gene&cmd=Retrieve&dopt=full_report&list_uids=201475) | RAB12, member RAS oncogene family |
| [Details](http://mirdb.org/cgi-bin/target_detail.cgi?targetID=598282) | 666 | 59 | hsa-miR-148a-3p | [KPNA1](http://www.ncbi.nlm.nih.gov/entrez/query.fcgi?db=gene&cmd=Retrieve&dopt=full_report&list_uids=3836) | karyopherin subunit alpha 1 |
| [Details](http://mirdb.org/cgi-bin/target_detail.cgi?targetID=598489) | 667 | 59 | hsa-miR-148a-3p | [KCNQ5](http://www.ncbi.nlm.nih.gov/entrez/query.fcgi?db=gene&cmd=Retrieve&dopt=full_report&list_uids=56479) | potassium voltage-gated channel subfamily Q member 5 |
| [Details](http://mirdb.org/cgi-bin/target_detail.cgi?targetID=598568) | 668 | 59 | hsa-miR-148a-3p | [PDIK1L](http://www.ncbi.nlm.nih.gov/entrez/query.fcgi?db=gene&cmd=Retrieve&dopt=full_report&list_uids=149420) | PDLIM1 interacting kinase 1 like |
| [Details](http://mirdb.org/cgi-bin/target_detail.cgi?targetID=598552) | 669 | 59 | hsa-miR-148a-3p | [PRR15](http://www.ncbi.nlm.nih.gov/entrez/query.fcgi?db=gene&cmd=Retrieve&dopt=full_report&list_uids=222171) | proline rich 15 |
| [Details](http://mirdb.org/cgi-bin/target_detail.cgi?targetID=598070) | 670 | 59 | hsa-miR-148a-3p | [PHIP](http://www.ncbi.nlm.nih.gov/entrez/query.fcgi?db=gene&cmd=Retrieve&dopt=full_report&list_uids=55023) | pleckstrin homology domain interacting protein |
| [Details](http://mirdb.org/cgi-bin/target_detail.cgi?targetID=597919) | 671 | 59 | hsa-miR-148a-3p | [ATXN3](http://www.ncbi.nlm.nih.gov/entrez/query.fcgi?db=gene&cmd=Retrieve&dopt=full_report&list_uids=4287) | ataxin 3 |
| [Details](http://mirdb.org/cgi-bin/target_detail.cgi?targetID=598480) | 672 | 58 | hsa-miR-148a-3p | [PPP1R7](http://www.ncbi.nlm.nih.gov/entrez/query.fcgi?db=gene&cmd=Retrieve&dopt=full_report&list_uids=5510) | protein phosphatase 1 regulatory subunit 7 |
| [Details](http://mirdb.org/cgi-bin/target_detail.cgi?targetID=598540) | 673 | 58 | hsa-miR-148a-3p | [PIK3IP1](http://www.ncbi.nlm.nih.gov/entrez/query.fcgi?db=gene&cmd=Retrieve&dopt=full_report&list_uids=113791) | phosphoinositide-3-kinase interacting protein 1 |
| [Details](http://mirdb.org/cgi-bin/target_detail.cgi?targetID=598235) | 674 | 58 | hsa-miR-148a-3p | [SEC23A](http://www.ncbi.nlm.nih.gov/entrez/query.fcgi?db=gene&cmd=Retrieve&dopt=full_report&list_uids=10484) | Sec23 homolog A, coat complex II component |
| [Details](http://mirdb.org/cgi-bin/target_detail.cgi?targetID=598007) | 675 | 58 | hsa-miR-148a-3p | [FBXO33](http://www.ncbi.nlm.nih.gov/entrez/query.fcgi?db=gene&cmd=Retrieve&dopt=full_report&list_uids=254170) | F-box protein 33 |
| [Details](http://mirdb.org/cgi-bin/target_detail.cgi?targetID=598453) | 676 | 58 | hsa-miR-148a-3p | [GPR180](http://www.ncbi.nlm.nih.gov/entrez/query.fcgi?db=gene&cmd=Retrieve&dopt=full_report&list_uids=160897) | G protein-coupled receptor 180 |
| [Details](http://mirdb.org/cgi-bin/target_detail.cgi?targetID=597875) | 677 | 58 | hsa-miR-148a-3p | [HSP90B1](http://www.ncbi.nlm.nih.gov/entrez/query.fcgi?db=gene&cmd=Retrieve&dopt=full_report&list_uids=7184) | heat shock protein 90 beta family member 1 |
| [Details](http://mirdb.org/cgi-bin/target_detail.cgi?targetID=598066) | 678 | 58 | hsa-miR-148a-3p | [DEDD](http://www.ncbi.nlm.nih.gov/entrez/query.fcgi?db=gene&cmd=Retrieve&dopt=full_report&list_uids=9191) | death effector domain containing |
| [Details](http://mirdb.org/cgi-bin/target_detail.cgi?targetID=598434) | 679 | 58 | hsa-miR-148a-3p | [ZNF614](http://www.ncbi.nlm.nih.gov/entrez/query.fcgi?db=gene&cmd=Retrieve&dopt=full_report&list_uids=80110) | zinc finger protein 614 |
| [Details](http://mirdb.org/cgi-bin/target_detail.cgi?targetID=598170) | 680 | 58 | hsa-miR-148a-3p | [TPBGL](http://www.ncbi.nlm.nih.gov/entrez/query.fcgi?db=gene&cmd=Retrieve&dopt=full_report&list_uids=100507050) | trophoblast glycoprotein like |
| [Details](http://mirdb.org/cgi-bin/target_detail.cgi?targetID=598654) | 681 | 58 | hsa-miR-148a-3p | [SCAF11](http://www.ncbi.nlm.nih.gov/entrez/query.fcgi?db=gene&cmd=Retrieve&dopt=full_report&list_uids=9169) | SR-related CTD associated factor 11 |
| [Details](http://mirdb.org/cgi-bin/target_detail.cgi?targetID=598633) | 682 | 58 | hsa-miR-148a-3p | [SCAP](http://www.ncbi.nlm.nih.gov/entrez/query.fcgi?db=gene&cmd=Retrieve&dopt=full_report&list_uids=22937) | SREBF chaperone |
| [Details](http://mirdb.org/cgi-bin/target_detail.cgi?targetID=598180) | 683 | 58 | hsa-miR-148a-3p | [PTPRM](http://www.ncbi.nlm.nih.gov/entrez/query.fcgi?db=gene&cmd=Retrieve&dopt=full_report&list_uids=5797) | protein tyrosine phosphatase, receptor type M |
| [Details](http://mirdb.org/cgi-bin/target_detail.cgi?targetID=598186) | 684 | 58 | hsa-miR-148a-3p | [STXBP1](http://www.ncbi.nlm.nih.gov/entrez/query.fcgi?db=gene&cmd=Retrieve&dopt=full_report&list_uids=6812) | syntaxin binding protein 1 |
| [Details](http://mirdb.org/cgi-bin/target_detail.cgi?targetID=597929) | 685 | 58 | hsa-miR-148a-3p | [CCDC71](http://www.ncbi.nlm.nih.gov/entrez/query.fcgi?db=gene&cmd=Retrieve&dopt=full_report&list_uids=64925) | coiled-coil domain containing 71 |
| [Details](http://mirdb.org/cgi-bin/target_detail.cgi?targetID=598056) | 686 | 57 | hsa-miR-148a-3p | [NAA15](http://www.ncbi.nlm.nih.gov/entrez/query.fcgi?db=gene&cmd=Retrieve&dopt=full_report&list_uids=80155) | N(alpha)-acetyltransferase 15, NatA auxiliary subunit |
| [Details](http://mirdb.org/cgi-bin/target_detail.cgi?targetID=598068) | 687 | 57 | hsa-miR-148a-3p | [RAB11FIP4](http://www.ncbi.nlm.nih.gov/entrez/query.fcgi?db=gene&cmd=Retrieve&dopt=full_report&list_uids=84440) | RAB11 family interacting protein 4 |
| [Details](http://mirdb.org/cgi-bin/target_detail.cgi?targetID=598072) | 688 | 57 | hsa-miR-148a-3p | [E2F3](http://www.ncbi.nlm.nih.gov/entrez/query.fcgi?db=gene&cmd=Retrieve&dopt=full_report&list_uids=1871) | E2F transcription factor 3 |
| [Details](http://mirdb.org/cgi-bin/target_detail.cgi?targetID=598027) | 689 | 57 | hsa-miR-148a-3p | [CEP85L](http://www.ncbi.nlm.nih.gov/entrez/query.fcgi?db=gene&cmd=Retrieve&dopt=full_report&list_uids=387119) | centrosomal protein 85 like |
| [Details](http://mirdb.org/cgi-bin/target_detail.cgi?targetID=598198) | 690 | 57 | hsa-miR-148a-3p | [MMP19](http://www.ncbi.nlm.nih.gov/entrez/query.fcgi?db=gene&cmd=Retrieve&dopt=full_report&list_uids=4327) | matrix metallopeptidase 19 |
| [Details](http://mirdb.org/cgi-bin/target_detail.cgi?targetID=597960) | 691 | 57 | hsa-miR-148a-3p | [NECTIN1](http://www.ncbi.nlm.nih.gov/entrez/query.fcgi?db=gene&cmd=Retrieve&dopt=full_report&list_uids=5818) | nectin cell adhesion molecule 1 |
| [Details](http://mirdb.org/cgi-bin/target_detail.cgi?targetID=598599) | 692 | 57 | hsa-miR-148a-3p | [LGALS8](http://www.ncbi.nlm.nih.gov/entrez/query.fcgi?db=gene&cmd=Retrieve&dopt=full_report&list_uids=3964) | galectin 8 |
| [Details](http://mirdb.org/cgi-bin/target_detail.cgi?targetID=598144) | 693 | 57 | hsa-miR-148a-3p | [HLA-A](http://www.ncbi.nlm.nih.gov/entrez/query.fcgi?db=gene&cmd=Retrieve&dopt=full_report&list_uids=3105) | major histocompatibility complex, class I, A |
| [Details](http://mirdb.org/cgi-bin/target_detail.cgi?targetID=597931) | 694 | 57 | hsa-miR-148a-3p | [DGCR2](http://www.ncbi.nlm.nih.gov/entrez/query.fcgi?db=gene&cmd=Retrieve&dopt=full_report&list_uids=9993) | DiGeorge syndrome critical region gene 2 |
| [Details](http://mirdb.org/cgi-bin/target_detail.cgi?targetID=598499) | 695 | 57 | hsa-miR-148a-3p | [TNFRSF1B](http://www.ncbi.nlm.nih.gov/entrez/query.fcgi?db=gene&cmd=Retrieve&dopt=full_report&list_uids=7133) | TNF receptor superfamily member 1B |
| [Details](http://mirdb.org/cgi-bin/target_detail.cgi?targetID=598211) | 696 | 57 | hsa-miR-148a-3p | [STX6](http://www.ncbi.nlm.nih.gov/entrez/query.fcgi?db=gene&cmd=Retrieve&dopt=full_report&list_uids=10228) | syntaxin 6 |
| [Details](http://mirdb.org/cgi-bin/target_detail.cgi?targetID=598495) | 697 | 57 | hsa-miR-148a-3p | [RPS6KB1](http://www.ncbi.nlm.nih.gov/entrez/query.fcgi?db=gene&cmd=Retrieve&dopt=full_report&list_uids=6198) | ribosomal protein S6 kinase B1 |
| [Details](http://mirdb.org/cgi-bin/target_detail.cgi?targetID=598195) | 698 | 57 | hsa-miR-148a-3p | [NEUROD1](http://www.ncbi.nlm.nih.gov/entrez/query.fcgi?db=gene&cmd=Retrieve&dopt=full_report&list_uids=4760) | neuronal differentiation 1 |
| [Details](http://mirdb.org/cgi-bin/target_detail.cgi?targetID=598123) | 699 | 57 | hsa-miR-148a-3p | [USP4](http://www.ncbi.nlm.nih.gov/entrez/query.fcgi?db=gene&cmd=Retrieve&dopt=full_report&list_uids=7375) | ubiquitin specific peptidase 4 |
| [Details](http://mirdb.org/cgi-bin/target_detail.cgi?targetID=598265) | 700 | 57 | hsa-miR-148a-3p | [IGSF21](http://www.ncbi.nlm.nih.gov/entrez/query.fcgi?db=gene&cmd=Retrieve&dopt=full_report&list_uids=84966) | immunoglobin superfamily member 21 |
| [Details](http://mirdb.org/cgi-bin/target_detail.cgi?targetID=598601) | 701 | 57 | hsa-miR-148a-3p | [TNRC6B](http://www.ncbi.nlm.nih.gov/entrez/query.fcgi?db=gene&cmd=Retrieve&dopt=full_report&list_uids=23112) | trinucleotide repeat containing 6B |
| [Details](http://mirdb.org/cgi-bin/target_detail.cgi?targetID=598583) | 702 | 57 | hsa-miR-148a-3p | [NAALADL2](http://www.ncbi.nlm.nih.gov/entrez/query.fcgi?db=gene&cmd=Retrieve&dopt=full_report&list_uids=254827) | N-acetylated alpha-linked acidic dipeptidase like 2 |
| [Details](http://mirdb.org/cgi-bin/target_detail.cgi?targetID=598406) | 703 | 57 | hsa-miR-148a-3p | [ALG13](http://www.ncbi.nlm.nih.gov/entrez/query.fcgi?db=gene&cmd=Retrieve&dopt=full_report&list_uids=79868) | ALG13, UDP-N-acetylglucosaminyltransferase subunit |
| [Details](http://mirdb.org/cgi-bin/target_detail.cgi?targetID=598269) | 704 | 57 | hsa-miR-148a-3p | [LENG8](http://www.ncbi.nlm.nih.gov/entrez/query.fcgi?db=gene&cmd=Retrieve&dopt=full_report&list_uids=114823) | leukocyte receptor cluster member 8 |
| [Details](http://mirdb.org/cgi-bin/target_detail.cgi?targetID=598687) | 705 | 56 | hsa-miR-148a-3p | [TNFRSF11A](http://www.ncbi.nlm.nih.gov/entrez/query.fcgi?db=gene&cmd=Retrieve&dopt=full_report&list_uids=8792) | TNF receptor superfamily member 11a |
| [Details](http://mirdb.org/cgi-bin/target_detail.cgi?targetID=598677) | 706 | 56 | hsa-miR-148a-3p | [MEF2A](http://www.ncbi.nlm.nih.gov/entrez/query.fcgi?db=gene&cmd=Retrieve&dopt=full_report&list_uids=4205) | myocyte enhancer factor 2A |
| [Details](http://mirdb.org/cgi-bin/target_detail.cgi?targetID=598370) | 707 | 56 | hsa-miR-148a-3p | [ECEL1](http://www.ncbi.nlm.nih.gov/entrez/query.fcgi?db=gene&cmd=Retrieve&dopt=full_report&list_uids=9427) | endothelin converting enzyme like 1 |
| [Details](http://mirdb.org/cgi-bin/target_detail.cgi?targetID=597910) | 708 | 56 | hsa-miR-148a-3p | [FOXK2](http://www.ncbi.nlm.nih.gov/entrez/query.fcgi?db=gene&cmd=Retrieve&dopt=full_report&list_uids=3607) | forkhead box K2 |
| [Details](http://mirdb.org/cgi-bin/target_detail.cgi?targetID=598017) | 709 | 56 | hsa-miR-148a-3p | [MRPL28](http://www.ncbi.nlm.nih.gov/entrez/query.fcgi?db=gene&cmd=Retrieve&dopt=full_report&list_uids=10573) | mitochondrial ribosomal protein L28 |
| [Details](http://mirdb.org/cgi-bin/target_detail.cgi?targetID=598360) | 710 | 56 | hsa-miR-148a-3p | [MCMDC2](http://www.ncbi.nlm.nih.gov/entrez/query.fcgi?db=gene&cmd=Retrieve&dopt=full_report&list_uids=157777) | minichromosome maintenance domain containing 2 |
| [Details](http://mirdb.org/cgi-bin/target_detail.cgi?targetID=598131) | 711 | 56 | hsa-miR-148a-3p | [BCLAF3](http://www.ncbi.nlm.nih.gov/entrez/query.fcgi?db=gene&cmd=Retrieve&dopt=full_report&list_uids=256643) | BCLAF1 and THRAP3 family member 3 |
| [Details](http://mirdb.org/cgi-bin/target_detail.cgi?targetID=597994) | 712 | 56 | hsa-miR-148a-3p | [KDM1B](http://www.ncbi.nlm.nih.gov/entrez/query.fcgi?db=gene&cmd=Retrieve&dopt=full_report&list_uids=221656) | lysine demethylase 1B |
| [Details](http://mirdb.org/cgi-bin/target_detail.cgi?targetID=598643) | 713 | 56 | hsa-miR-148a-3p | [AKT2](http://www.ncbi.nlm.nih.gov/entrez/query.fcgi?db=gene&cmd=Retrieve&dopt=full_report&list_uids=208) | AKT serine/threonine kinase 2 |
| [Details](http://mirdb.org/cgi-bin/target_detail.cgi?targetID=598021) | 714 | 56 | hsa-miR-148a-3p | [SLC6A15](http://www.ncbi.nlm.nih.gov/entrez/query.fcgi?db=gene&cmd=Retrieve&dopt=full_report&list_uids=55117) | solute carrier family 6 member 15 |
| [Details](http://mirdb.org/cgi-bin/target_detail.cgi?targetID=598520) | 715 | 56 | hsa-miR-148a-3p | [CMTR1](http://www.ncbi.nlm.nih.gov/entrez/query.fcgi?db=gene&cmd=Retrieve&dopt=full_report&list_uids=23070) | cap methyltransferase 1 |
| [Details](http://mirdb.org/cgi-bin/target_detail.cgi?targetID=598233) | 716 | 56 | hsa-miR-148a-3p | [IRX4](http://www.ncbi.nlm.nih.gov/entrez/query.fcgi?db=gene&cmd=Retrieve&dopt=full_report&list_uids=50805) | iroquois homeobox 4 |
| [Details](http://mirdb.org/cgi-bin/target_detail.cgi?targetID=598237) | 717 | 56 | hsa-miR-148a-3p | [PPP1CB](http://www.ncbi.nlm.nih.gov/entrez/query.fcgi?db=gene&cmd=Retrieve&dopt=full_report&list_uids=5500) | protein phosphatase 1 catalytic subunit beta |
| [Details](http://mirdb.org/cgi-bin/target_detail.cgi?targetID=598104) | 718 | 56 | hsa-miR-148a-3p | [DSTYK](http://www.ncbi.nlm.nih.gov/entrez/query.fcgi?db=gene&cmd=Retrieve&dopt=full_report&list_uids=25778) | dual serine/threonine and tyrosine protein kinase |
| [Details](http://mirdb.org/cgi-bin/target_detail.cgi?targetID=598517) | 719 | 56 | hsa-miR-148a-3p | [SH3PXD2A](http://www.ncbi.nlm.nih.gov/entrez/query.fcgi?db=gene&cmd=Retrieve&dopt=full_report&list_uids=9644) | SH3 and PX domains 2A |
| [Details](http://mirdb.org/cgi-bin/target_detail.cgi?targetID=598425) | 720 | 56 | hsa-miR-148a-3p | [UBA6](http://www.ncbi.nlm.nih.gov/entrez/query.fcgi?db=gene&cmd=Retrieve&dopt=full_report&list_uids=55236) | ubiquitin like modifier activating enzyme 6 |
| [Details](http://mirdb.org/cgi-bin/target_detail.cgi?targetID=598475) | 721 | 56 | hsa-miR-148a-3p | [EIF4E3](http://www.ncbi.nlm.nih.gov/entrez/query.fcgi?db=gene&cmd=Retrieve&dopt=full_report&list_uids=317649) | eukaryotic translation initiation factor 4E family member 3 |
| [Details](http://mirdb.org/cgi-bin/target_detail.cgi?targetID=598538) | 722 | 56 | hsa-miR-148a-3p | [SLC7A11](http://www.ncbi.nlm.nih.gov/entrez/query.fcgi?db=gene&cmd=Retrieve&dopt=full_report&list_uids=23657) | solute carrier family 7 member 11 |
| [Details](http://mirdb.org/cgi-bin/target_detail.cgi?targetID=598103) | 723 | 55 | hsa-miR-148a-3p | [LMAN1](http://www.ncbi.nlm.nih.gov/entrez/query.fcgi?db=gene&cmd=Retrieve&dopt=full_report&list_uids=3998) | lectin, mannose binding 1 |
| [Details](http://mirdb.org/cgi-bin/target_detail.cgi?targetID=598307) | 724 | 55 | hsa-miR-148a-3p | [ADAMTS18](http://www.ncbi.nlm.nih.gov/entrez/query.fcgi?db=gene&cmd=Retrieve&dopt=full_report&list_uids=170692) | ADAM metallopeptidase with thrombospondin type 1 motif 18 |
| [Details](http://mirdb.org/cgi-bin/target_detail.cgi?targetID=598679) | 725 | 55 | hsa-miR-148a-3p | [NAGK](http://www.ncbi.nlm.nih.gov/entrez/query.fcgi?db=gene&cmd=Retrieve&dopt=full_report&list_uids=55577) | N-acetylglucosamine kinase |
| [Details](http://mirdb.org/cgi-bin/target_detail.cgi?targetID=598594) | 726 | 55 | hsa-miR-148a-3p | [DNM3](http://www.ncbi.nlm.nih.gov/entrez/query.fcgi?db=gene&cmd=Retrieve&dopt=full_report&list_uids=26052) | dynamin 3 |
| [Details](http://mirdb.org/cgi-bin/target_detail.cgi?targetID=597877) | 727 | 55 | hsa-miR-148a-3p | [UBE3B](http://www.ncbi.nlm.nih.gov/entrez/query.fcgi?db=gene&cmd=Retrieve&dopt=full_report&list_uids=89910) | ubiquitin protein ligase E3B |
| [Details](http://mirdb.org/cgi-bin/target_detail.cgi?targetID=598272) | 728 | 55 | hsa-miR-148a-3p | [PPP1R3E](http://www.ncbi.nlm.nih.gov/entrez/query.fcgi?db=gene&cmd=Retrieve&dopt=full_report&list_uids=90673) | protein phosphatase 1 regulatory subunit 3E |
| [Details](http://mirdb.org/cgi-bin/target_detail.cgi?targetID=598222) | 729 | 55 | hsa-miR-148a-3p | [PPARD](http://www.ncbi.nlm.nih.gov/entrez/query.fcgi?db=gene&cmd=Retrieve&dopt=full_report&list_uids=5467) | peroxisome proliferator activated receptor delta |
| [Details](http://mirdb.org/cgi-bin/target_detail.cgi?targetID=597964) | 730 | 55 | hsa-miR-148a-3p | [XXYLT1](http://www.ncbi.nlm.nih.gov/entrez/query.fcgi?db=gene&cmd=Retrieve&dopt=full_report&list_uids=152002) | xyloside xylosyltransferase 1 |
| [Details](http://mirdb.org/cgi-bin/target_detail.cgi?targetID=598603) | 731 | 55 | hsa-miR-148a-3p | [SGMS1](http://www.ncbi.nlm.nih.gov/entrez/query.fcgi?db=gene&cmd=Retrieve&dopt=full_report&list_uids=259230) | sphingomyelin synthase 1 |
| [Details](http://mirdb.org/cgi-bin/target_detail.cgi?targetID=598000) | 732 | 55 | hsa-miR-148a-3p | [TMEM50B](http://www.ncbi.nlm.nih.gov/entrez/query.fcgi?db=gene&cmd=Retrieve&dopt=full_report&list_uids=757) | transmembrane protein 50B |
| [Details](http://mirdb.org/cgi-bin/target_detail.cgi?targetID=598658) | 733 | 55 | hsa-miR-148a-3p | [C6orf62](http://www.ncbi.nlm.nih.gov/entrez/query.fcgi?db=gene&cmd=Retrieve&dopt=full_report&list_uids=81688) | chromosome 6 open reading frame 62 |
| [Details](http://mirdb.org/cgi-bin/target_detail.cgi?targetID=598685) | 734 | 55 | hsa-miR-148a-3p | [IKZF1](http://www.ncbi.nlm.nih.gov/entrez/query.fcgi?db=gene&cmd=Retrieve&dopt=full_report&list_uids=10320) | IKAROS family zinc finger 1 |
| [Details](http://mirdb.org/cgi-bin/target_detail.cgi?targetID=598343) | 735 | 55 | hsa-miR-148a-3p | [UBE2D1](http://www.ncbi.nlm.nih.gov/entrez/query.fcgi?db=gene&cmd=Retrieve&dopt=full_report&list_uids=7321) | ubiquitin conjugating enzyme E2 D1 |
| [Details](http://mirdb.org/cgi-bin/target_detail.cgi?targetID=597917) | 736 | 55 | hsa-miR-148a-3p | [RRM2B](http://www.ncbi.nlm.nih.gov/entrez/query.fcgi?db=gene&cmd=Retrieve&dopt=full_report&list_uids=50484) | ribonucleotide reductase regulatory TP53 inducible subunit M2B |
| [Details](http://mirdb.org/cgi-bin/target_detail.cgi?targetID=598457) | 737 | 55 | hsa-miR-148a-3p | [SLC24A2](http://www.ncbi.nlm.nih.gov/entrez/query.fcgi?db=gene&cmd=Retrieve&dopt=full_report&list_uids=25769) | solute carrier family 24 member 2 |
| [Details](http://mirdb.org/cgi-bin/target_detail.cgi?targetID=598064) | 738 | 55 | hsa-miR-148a-3p | [SRA1](http://www.ncbi.nlm.nih.gov/entrez/query.fcgi?db=gene&cmd=Retrieve&dopt=full_report&list_uids=10011) | steroid receptor RNA activator 1 |
| [Details](http://mirdb.org/cgi-bin/target_detail.cgi?targetID=598167) | 739 | 55 | hsa-miR-148a-3p | [YPEL3](http://www.ncbi.nlm.nih.gov/entrez/query.fcgi?db=gene&cmd=Retrieve&dopt=full_report&list_uids=83719) | yippee like 3 |
| [Details](http://mirdb.org/cgi-bin/target_detail.cgi?targetID=597859) | 740 | 55 | hsa-miR-148a-3p | [ZNF532](http://www.ncbi.nlm.nih.gov/entrez/query.fcgi?db=gene&cmd=Retrieve&dopt=full_report&list_uids=55205) | zinc finger protein 532 |
| [Details](http://mirdb.org/cgi-bin/target_detail.cgi?targetID=598402) | 741 | 55 | hsa-miR-148a-3p | [ZNF778](http://www.ncbi.nlm.nih.gov/entrez/query.fcgi?db=gene&cmd=Retrieve&dopt=full_report&list_uids=197320) | zinc finger protein 778 |
| [Details](http://mirdb.org/cgi-bin/target_detail.cgi?targetID=598208) | 742 | 54 | hsa-miR-148a-3p | [MOSMO](http://www.ncbi.nlm.nih.gov/entrez/query.fcgi?db=gene&cmd=Retrieve&dopt=full_report&list_uids=730094) | modulator of smoothened |
| [Details](http://mirdb.org/cgi-bin/target_detail.cgi?targetID=598513) | 743 | 54 | hsa-miR-148a-3p | [ACSL3](http://www.ncbi.nlm.nih.gov/entrez/query.fcgi?db=gene&cmd=Retrieve&dopt=full_report&list_uids=2181) | acyl-CoA synthetase long chain family member 3 |
| [Details](http://mirdb.org/cgi-bin/target_detail.cgi?targetID=598169) | 744 | 54 | hsa-miR-148a-3p | [MARCH3](http://www.ncbi.nlm.nih.gov/entrez/query.fcgi?db=gene&cmd=Retrieve&dopt=full_report&list_uids=115123) | membrane associated ring-CH-type finger 3 |
| [Details](http://mirdb.org/cgi-bin/target_detail.cgi?targetID=598321) | 745 | 54 | hsa-miR-148a-3p | [TMEM246](http://www.ncbi.nlm.nih.gov/entrez/query.fcgi?db=gene&cmd=Retrieve&dopt=full_report&list_uids=84302) | transmembrane protein 246 |
| [Details](http://mirdb.org/cgi-bin/target_detail.cgi?targetID=598197) | 746 | 54 | hsa-miR-148a-3p | [DYNLL2](http://www.ncbi.nlm.nih.gov/entrez/query.fcgi?db=gene&cmd=Retrieve&dopt=full_report&list_uids=140735) | dynein light chain LC8-type 2 |
| [Details](http://mirdb.org/cgi-bin/target_detail.cgi?targetID=598497) | 747 | 54 | hsa-miR-148a-3p | [LDLRAD1](http://www.ncbi.nlm.nih.gov/entrez/query.fcgi?db=gene&cmd=Retrieve&dopt=full_report&list_uids=388633) | low density lipoprotein receptor class A domain containing 1 |
| [Details](http://mirdb.org/cgi-bin/target_detail.cgi?targetID=598137) | 748 | 54 | hsa-miR-148a-3p | [DGCR8](http://www.ncbi.nlm.nih.gov/entrez/query.fcgi?db=gene&cmd=Retrieve&dopt=full_report&list_uids=54487) | DGCR8, microprocessor complex subunit |
| [Details](http://mirdb.org/cgi-bin/target_detail.cgi?targetID=598138) | 749 | 54 | hsa-miR-148a-3p | [TSPAN18](http://www.ncbi.nlm.nih.gov/entrez/query.fcgi?db=gene&cmd=Retrieve&dopt=full_report&list_uids=90139) | tetraspanin 18 |
| [Details](http://mirdb.org/cgi-bin/target_detail.cgi?targetID=598473) | 750 | 54 | hsa-miR-148a-3p | [AP1G1](http://www.ncbi.nlm.nih.gov/entrez/query.fcgi?db=gene&cmd=Retrieve&dopt=full_report&list_uids=164) | adaptor related protein complex 1 subunit gamma 1 |
| [Details](http://mirdb.org/cgi-bin/target_detail.cgi?targetID=597932) | 751 | 54 | hsa-miR-148a-3p | [PDE7A](http://www.ncbi.nlm.nih.gov/entrez/query.fcgi?db=gene&cmd=Retrieve&dopt=full_report&list_uids=5150) | phosphodiesterase 7A |
| [Details](http://mirdb.org/cgi-bin/target_detail.cgi?targetID=598318) | 752 | 54 | hsa-miR-148a-3p | [TMEM178B](http://www.ncbi.nlm.nih.gov/entrez/query.fcgi?db=gene&cmd=Retrieve&dopt=full_report&list_uids=100507421) | transmembrane protein 178B |
| [Details](http://mirdb.org/cgi-bin/target_detail.cgi?targetID=598632) | 753 | 54 | hsa-miR-148a-3p | [TIGD5](http://www.ncbi.nlm.nih.gov/entrez/query.fcgi?db=gene&cmd=Retrieve&dopt=full_report&list_uids=84948) | tigger transposable element derived 5 |
| [Details](http://mirdb.org/cgi-bin/target_detail.cgi?targetID=598182) | 754 | 54 | hsa-miR-148a-3p | [GLIPR2](http://www.ncbi.nlm.nih.gov/entrez/query.fcgi?db=gene&cmd=Retrieve&dopt=full_report&list_uids=152007) | GLI pathogenesis related 2 |
| [Details](http://mirdb.org/cgi-bin/target_detail.cgi?targetID=598431) | 755 | 54 | hsa-miR-148a-3p | [SPIN1](http://www.ncbi.nlm.nih.gov/entrez/query.fcgi?db=gene&cmd=Retrieve&dopt=full_report&list_uids=10927) | spindlin 1 |
| [Details](http://mirdb.org/cgi-bin/target_detail.cgi?targetID=598291) | 756 | 54 | hsa-miR-148a-3p | [ASPH](http://www.ncbi.nlm.nih.gov/entrez/query.fcgi?db=gene&cmd=Retrieve&dopt=full_report&list_uids=444) | aspartate beta-hydroxylase |
| [Details](http://mirdb.org/cgi-bin/target_detail.cgi?targetID=598225) | 757 | 54 | hsa-miR-148a-3p | [KCNQ4](http://www.ncbi.nlm.nih.gov/entrez/query.fcgi?db=gene&cmd=Retrieve&dopt=full_report&list_uids=9132) | potassium voltage-gated channel subfamily Q member 4 |
| [Details](http://mirdb.org/cgi-bin/target_detail.cgi?targetID=598536) | 758 | 54 | hsa-miR-148a-3p | [LIMD1](http://www.ncbi.nlm.nih.gov/entrez/query.fcgi?db=gene&cmd=Retrieve&dopt=full_report&list_uids=8994) | LIM domains containing 1 |
| [Details](http://mirdb.org/cgi-bin/target_detail.cgi?targetID=598085) | 759 | 54 | hsa-miR-148a-3p | [LIPG](http://www.ncbi.nlm.nih.gov/entrez/query.fcgi?db=gene&cmd=Retrieve&dopt=full_report&list_uids=9388) | lipase G, endothelial type |
| [Details](http://mirdb.org/cgi-bin/target_detail.cgi?targetID=598686) | 760 | 54 | hsa-miR-148a-3p | [C6orf136](http://www.ncbi.nlm.nih.gov/entrez/query.fcgi?db=gene&cmd=Retrieve&dopt=full_report&list_uids=221545) | chromosome 6 open reading frame 136 |
| [Details](http://mirdb.org/cgi-bin/target_detail.cgi?targetID=598336) | 761 | 54 | hsa-miR-148a-3p | [TMEM165](http://www.ncbi.nlm.nih.gov/entrez/query.fcgi?db=gene&cmd=Retrieve&dopt=full_report&list_uids=55858) | transmembrane protein 165 |
| [Details](http://mirdb.org/cgi-bin/target_detail.cgi?targetID=598036) | 762 | 54 | hsa-miR-148a-3p | [MCM3AP](http://www.ncbi.nlm.nih.gov/entrez/query.fcgi?db=gene&cmd=Retrieve&dopt=full_report&list_uids=8888) | minichromosome maintenance complex component 3 associated protein |
| [Details](http://mirdb.org/cgi-bin/target_detail.cgi?targetID=598224) | 763 | 54 | hsa-miR-148a-3p | [SMAD5](http://www.ncbi.nlm.nih.gov/entrez/query.fcgi?db=gene&cmd=Retrieve&dopt=full_report&list_uids=4090) | SMAD family member 5 |
| [Details](http://mirdb.org/cgi-bin/target_detail.cgi?targetID=598192) | 764 | 54 | hsa-miR-148a-3p | [CLOCK](http://www.ncbi.nlm.nih.gov/entrez/query.fcgi?db=gene&cmd=Retrieve&dopt=full_report&list_uids=9575) | clock circadian regulator |
| [Details](http://mirdb.org/cgi-bin/target_detail.cgi?targetID=597888) | 765 | 53 | hsa-miR-148a-3p | [KIAA1210](http://www.ncbi.nlm.nih.gov/entrez/query.fcgi?db=gene&cmd=Retrieve&dopt=full_report&list_uids=57481) | KIAA1210 |
| [Details](http://mirdb.org/cgi-bin/target_detail.cgi?targetID=598460) | 766 | 53 | hsa-miR-148a-3p | [ZNF784](http://www.ncbi.nlm.nih.gov/entrez/query.fcgi?db=gene&cmd=Retrieve&dopt=full_report&list_uids=147808) | zinc finger protein 784 |
| [Details](http://mirdb.org/cgi-bin/target_detail.cgi?targetID=598505) | 767 | 53 | hsa-miR-148a-3p | [PSMC2](http://www.ncbi.nlm.nih.gov/entrez/query.fcgi?db=gene&cmd=Retrieve&dopt=full_report&list_uids=5701) | proteasome 26S subunit, ATPase 2 |
| [Details](http://mirdb.org/cgi-bin/target_detail.cgi?targetID=598492) | 768 | 53 | hsa-miR-148a-3p | [CGGBP1](http://www.ncbi.nlm.nih.gov/entrez/query.fcgi?db=gene&cmd=Retrieve&dopt=full_report&list_uids=8545) | CGG triplet repeat binding protein 1 |
| [Details](http://mirdb.org/cgi-bin/target_detail.cgi?targetID=598350) | 769 | 53 | hsa-miR-148a-3p | [MECP2](http://www.ncbi.nlm.nih.gov/entrez/query.fcgi?db=gene&cmd=Retrieve&dopt=full_report&list_uids=4204) | methyl-CpG binding protein 2 |
| [Details](http://mirdb.org/cgi-bin/target_detail.cgi?targetID=597870) | 770 | 53 | hsa-miR-148a-3p | [MAF](http://www.ncbi.nlm.nih.gov/entrez/query.fcgi?db=gene&cmd=Retrieve&dopt=full_report&list_uids=4094) | MAF bZIP transcription factor |
| [Details](http://mirdb.org/cgi-bin/target_detail.cgi?targetID=598312) | 771 | 53 | hsa-miR-148a-3p | [PPP2R2C](http://www.ncbi.nlm.nih.gov/entrez/query.fcgi?db=gene&cmd=Retrieve&dopt=full_report&list_uids=5522) | protein phosphatase 2 regulatory subunit Bgamma |
| [Details](http://mirdb.org/cgi-bin/target_detail.cgi?targetID=598344) | 772 | 53 | hsa-miR-148a-3p | [RSBN1L](http://www.ncbi.nlm.nih.gov/entrez/query.fcgi?db=gene&cmd=Retrieve&dopt=full_report&list_uids=222194) | round spermatid basic protein 1 like |
| [Details](http://mirdb.org/cgi-bin/target_detail.cgi?targetID=598411) | 773 | 53 | hsa-miR-148a-3p | [SHC3](http://www.ncbi.nlm.nih.gov/entrez/query.fcgi?db=gene&cmd=Retrieve&dopt=full_report&list_uids=53358) | SHC adaptor protein 3 |
| [Details](http://mirdb.org/cgi-bin/target_detail.cgi?targetID=598447) | 774 | 53 | hsa-miR-148a-3p | [EGR3](http://www.ncbi.nlm.nih.gov/entrez/query.fcgi?db=gene&cmd=Retrieve&dopt=full_report&list_uids=1960) | early growth response 3 |
| [Details](http://mirdb.org/cgi-bin/target_detail.cgi?targetID=597912) | 775 | 53 | hsa-miR-148a-3p | [TMEM239](http://www.ncbi.nlm.nih.gov/entrez/query.fcgi?db=gene&cmd=Retrieve&dopt=full_report&list_uids=100288797) | transmembrane protein 239 |
| [Details](http://mirdb.org/cgi-bin/target_detail.cgi?targetID=598039) | 776 | 53 | hsa-miR-148a-3p | [RAI14](http://www.ncbi.nlm.nih.gov/entrez/query.fcgi?db=gene&cmd=Retrieve&dopt=full_report&list_uids=26064) | retinoic acid induced 14 |
| [Details](http://mirdb.org/cgi-bin/target_detail.cgi?targetID=597911) | 777 | 53 | hsa-miR-148a-3p | [EPB41L2](http://www.ncbi.nlm.nih.gov/entrez/query.fcgi?db=gene&cmd=Retrieve&dopt=full_report&list_uids=2037) | erythrocyte membrane protein band 4.1 like 2 |
| [Details](http://mirdb.org/cgi-bin/target_detail.cgi?targetID=598126) | 778 | 53 | hsa-miR-148a-3p | [PSEN1](http://www.ncbi.nlm.nih.gov/entrez/query.fcgi?db=gene&cmd=Retrieve&dopt=full_report&list_uids=5663) | presenilin 1 |
| [Details](http://mirdb.org/cgi-bin/target_detail.cgi?targetID=598390) | 779 | 53 | hsa-miR-148a-3p | [PDPR](http://www.ncbi.nlm.nih.gov/entrez/query.fcgi?db=gene&cmd=Retrieve&dopt=full_report&list_uids=55066) | pyruvate dehydrogenase phosphatase regulatory subunit |
| [Details](http://mirdb.org/cgi-bin/target_detail.cgi?targetID=598395) | 780 | 53 | hsa-miR-148a-3p | [ETNK1](http://www.ncbi.nlm.nih.gov/entrez/query.fcgi?db=gene&cmd=Retrieve&dopt=full_report&list_uids=55500) | ethanolamine kinase 1 |
| [Details](http://mirdb.org/cgi-bin/target_detail.cgi?targetID=597951) | 781 | 53 | hsa-miR-148a-3p | [ARHGEF7](http://www.ncbi.nlm.nih.gov/entrez/query.fcgi?db=gene&cmd=Retrieve&dopt=full_report&list_uids=8874) | Rho guanine nucleotide exchange factor 7 |
| [Details](http://mirdb.org/cgi-bin/target_detail.cgi?targetID=598292) | 782 | 53 | hsa-miR-148a-3p | [ZNF618](http://www.ncbi.nlm.nih.gov/entrez/query.fcgi?db=gene&cmd=Retrieve&dopt=full_report&list_uids=114991) | zinc finger protein 618 |
| [Details](http://mirdb.org/cgi-bin/target_detail.cgi?targetID=598618) | 783 | 53 | hsa-miR-148a-3p | [AJUBA](http://www.ncbi.nlm.nih.gov/entrez/query.fcgi?db=gene&cmd=Retrieve&dopt=full_report&list_uids=84962) | ajuba LIM protein |
| [Details](http://mirdb.org/cgi-bin/target_detail.cgi?targetID=598121) | 784 | 53 | hsa-miR-148a-3p | [VSNL1](http://www.ncbi.nlm.nih.gov/entrez/query.fcgi?db=gene&cmd=Retrieve&dopt=full_report&list_uids=7447) | visinin like 1 |
| [Details](http://mirdb.org/cgi-bin/target_detail.cgi?targetID=598469) | 785 | 52 | hsa-miR-148a-3p | [GRM6](http://www.ncbi.nlm.nih.gov/entrez/query.fcgi?db=gene&cmd=Retrieve&dopt=full_report&list_uids=2916) | glutamate metabotropic receptor 6 |
| [Details](http://mirdb.org/cgi-bin/target_detail.cgi?targetID=598445) | 786 | 52 | hsa-miR-148a-3p | [CABLES1](http://www.ncbi.nlm.nih.gov/entrez/query.fcgi?db=gene&cmd=Retrieve&dopt=full_report&list_uids=91768) | Cdk5 and Abl enzyme substrate 1 |
| [Details](http://mirdb.org/cgi-bin/target_detail.cgi?targetID=598114) | 787 | 52 | hsa-miR-148a-3p | [VANGL1](http://www.ncbi.nlm.nih.gov/entrez/query.fcgi?db=gene&cmd=Retrieve&dopt=full_report&list_uids=81839) | VANGL planar cell polarity protein 1 |
| [Details](http://mirdb.org/cgi-bin/target_detail.cgi?targetID=598048) | 788 | 52 | hsa-miR-148a-3p | [LOC389602](http://www.ncbi.nlm.nih.gov/entrez/query.fcgi?db=gene&cmd=Retrieve&dopt=full_report&list_uids=389602) | uncharacterized LOC389602 |
| [Details](http://mirdb.org/cgi-bin/target_detail.cgi?targetID=598297) | 789 | 52 | hsa-miR-148a-3p | [NEXMIF](http://www.ncbi.nlm.nih.gov/entrez/query.fcgi?db=gene&cmd=Retrieve&dopt=full_report&list_uids=340533) | neurite extension and migration factor |
| [Details](http://mirdb.org/cgi-bin/target_detail.cgi?targetID=598436) | 790 | 52 | hsa-miR-148a-3p | [KITLG](http://www.ncbi.nlm.nih.gov/entrez/query.fcgi?db=gene&cmd=Retrieve&dopt=full_report&list_uids=4254) | KIT ligand |
| [Details](http://mirdb.org/cgi-bin/target_detail.cgi?targetID=598682) | 791 | 52 | hsa-miR-148a-3p | [VSIG10](http://www.ncbi.nlm.nih.gov/entrez/query.fcgi?db=gene&cmd=Retrieve&dopt=full_report&list_uids=54621) | V-set and immunoglobulin domain containing 10 |
| [Details](http://mirdb.org/cgi-bin/target_detail.cgi?targetID=598106) | 792 | 52 | hsa-miR-148a-3p | [CPEB4](http://www.ncbi.nlm.nih.gov/entrez/query.fcgi?db=gene&cmd=Retrieve&dopt=full_report&list_uids=80315) | cytoplasmic polyadenylation element binding protein 4 |
| [Details](http://mirdb.org/cgi-bin/target_detail.cgi?targetID=598405) | 793 | 52 | hsa-miR-148a-3p | [CDRT1](http://www.ncbi.nlm.nih.gov/entrez/query.fcgi?db=gene&cmd=Retrieve&dopt=full_report&list_uids=374286) | CMT1A duplicated region transcript 1 |
| [Details](http://mirdb.org/cgi-bin/target_detail.cgi?targetID=598363) | 794 | 52 | hsa-miR-148a-3p | [GUCY2C](http://www.ncbi.nlm.nih.gov/entrez/query.fcgi?db=gene&cmd=Retrieve&dopt=full_report&list_uids=2984) | guanylate cyclase 2C |
| [Details](http://mirdb.org/cgi-bin/target_detail.cgi?targetID=598304) | 795 | 52 | hsa-miR-148a-3p | [ARFGEF1](http://www.ncbi.nlm.nih.gov/entrez/query.fcgi?db=gene&cmd=Retrieve&dopt=full_report&list_uids=10565) | ADP ribosylation factor guanine nucleotide exchange factor 1 |
| [Details](http://mirdb.org/cgi-bin/target_detail.cgi?targetID=598132) | 796 | 52 | hsa-miR-148a-3p | [CEBPG](http://www.ncbi.nlm.nih.gov/entrez/query.fcgi?db=gene&cmd=Retrieve&dopt=full_report&list_uids=1054) | CCAAT enhancer binding protein gamma |
| [Details](http://mirdb.org/cgi-bin/target_detail.cgi?targetID=598669) | 797 | 52 | hsa-miR-148a-3p | [NAT8L](http://www.ncbi.nlm.nih.gov/entrez/query.fcgi?db=gene&cmd=Retrieve&dopt=full_report&list_uids=339983) | N-acetyltransferase 8 like |
| [Details](http://mirdb.org/cgi-bin/target_detail.cgi?targetID=598215) | 798 | 52 | hsa-miR-148a-3p | [LHX6](http://www.ncbi.nlm.nih.gov/entrez/query.fcgi?db=gene&cmd=Retrieve&dopt=full_report&list_uids=26468) | LIM homeobox 6 |
| [Details](http://mirdb.org/cgi-bin/target_detail.cgi?targetID=598100) | 799 | 52 | hsa-miR-148a-3p | [FBXO11](http://www.ncbi.nlm.nih.gov/entrez/query.fcgi?db=gene&cmd=Retrieve&dopt=full_report&list_uids=80204) | F-box protein 11 |
| [Details](http://mirdb.org/cgi-bin/target_detail.cgi?targetID=598190) | 800 | 51 | hsa-miR-148a-3p | [TGM1](http://www.ncbi.nlm.nih.gov/entrez/query.fcgi?db=gene&cmd=Retrieve&dopt=full_report&list_uids=7051) | transglutaminase 1 |
| [Details](http://mirdb.org/cgi-bin/target_detail.cgi?targetID=597914) | 801 | 51 | hsa-miR-148a-3p | [PIGK](http://www.ncbi.nlm.nih.gov/entrez/query.fcgi?db=gene&cmd=Retrieve&dopt=full_report&list_uids=10026) | phosphatidylinositol glycan anchor biosynthesis class K |
| [Details](http://mirdb.org/cgi-bin/target_detail.cgi?targetID=598157) | 802 | 51 | hsa-miR-148a-3p | [PDS5A](http://www.ncbi.nlm.nih.gov/entrez/query.fcgi?db=gene&cmd=Retrieve&dopt=full_report&list_uids=23244) | PDS5 cohesin associated factor A |
| [Details](http://mirdb.org/cgi-bin/target_detail.cgi?targetID=598051) | 803 | 51 | hsa-miR-148a-3p | [MLPH](http://www.ncbi.nlm.nih.gov/entrez/query.fcgi?db=gene&cmd=Retrieve&dopt=full_report&list_uids=79083) | melanophilin |
| [Details](http://mirdb.org/cgi-bin/target_detail.cgi?targetID=598619) | 804 | 51 | hsa-miR-148a-3p | [RSPH10B2](http://www.ncbi.nlm.nih.gov/entrez/query.fcgi?db=gene&cmd=Retrieve&dopt=full_report&list_uids=728194) | radial spoke head 10 homolog B2 |
| [Details](http://mirdb.org/cgi-bin/target_detail.cgi?targetID=598407) | 805 | 51 | hsa-miR-148a-3p | [AK3](http://www.ncbi.nlm.nih.gov/entrez/query.fcgi?db=gene&cmd=Retrieve&dopt=full_report&list_uids=50808) | adenylate kinase 3 |
| [Details](http://mirdb.org/cgi-bin/target_detail.cgi?targetID=598661) | 806 | 51 | hsa-miR-148a-3p | [RSPH10B](http://www.ncbi.nlm.nih.gov/entrez/query.fcgi?db=gene&cmd=Retrieve&dopt=full_report&list_uids=222967) | radial spoke head 10 homolog B |
| [Details](http://mirdb.org/cgi-bin/target_detail.cgi?targetID=598630) | 807 | 51 | hsa-miR-148a-3p | [ZDHHC6](http://www.ncbi.nlm.nih.gov/entrez/query.fcgi?db=gene&cmd=Retrieve&dopt=full_report&list_uids=64429) | zinc finger DHHC-type containing 6 |
| [Details](http://mirdb.org/cgi-bin/target_detail.cgi?targetID=598502) | 808 | 51 | hsa-miR-148a-3p | [SLC25A21](http://www.ncbi.nlm.nih.gov/entrez/query.fcgi?db=gene&cmd=Retrieve&dopt=full_report&list_uids=89874) | solute carrier family 25 member 21 |
| [Details](http://mirdb.org/cgi-bin/target_detail.cgi?targetID=598533) | 809 | 51 | hsa-miR-148a-3p | [LDB2](http://www.ncbi.nlm.nih.gov/entrez/query.fcgi?db=gene&cmd=Retrieve&dopt=full_report&list_uids=9079) | LIM domain binding 2 |
| [Details](http://mirdb.org/cgi-bin/target_detail.cgi?targetID=598022) | 810 | 51 | hsa-miR-148a-3p | [LOC110117498-PIK3R3](http://www.ncbi.nlm.nih.gov/entrez/query.fcgi?db=gene&cmd=Retrieve&dopt=full_report&list_uids=110117499) | LOC110117498-PIK3R3 readthrough |
| [Details](http://mirdb.org/cgi-bin/target_detail.cgi?targetID=598113) | 811 | 51 | hsa-miR-148a-3p | [KRTAP4-12](http://www.ncbi.nlm.nih.gov/entrez/query.fcgi?db=gene&cmd=Retrieve&dopt=full_report&list_uids=83755) | keratin associated protein 4-12 |
| [Details](http://mirdb.org/cgi-bin/target_detail.cgi?targetID=598263) | 812 | 51 | hsa-miR-148a-3p | [MLXIP](http://www.ncbi.nlm.nih.gov/entrez/query.fcgi?db=gene&cmd=Retrieve&dopt=full_report&list_uids=22877) | MLX interacting protein |
| [Details](http://mirdb.org/cgi-bin/target_detail.cgi?targetID=598031) | 813 | 50 | hsa-miR-148a-3p | [GRK3](http://www.ncbi.nlm.nih.gov/entrez/query.fcgi?db=gene&cmd=Retrieve&dopt=full_report&list_uids=157) | G protein-coupled receptor kinase 3 |
| [Details](http://mirdb.org/cgi-bin/target_detail.cgi?targetID=597943) | 814 | 50 | hsa-miR-148a-3p | [SEC22A](http://www.ncbi.nlm.nih.gov/entrez/query.fcgi?db=gene&cmd=Retrieve&dopt=full_report&list_uids=26984) | SEC22 homolog A, vesicle trafficking protein |
| [Details](http://mirdb.org/cgi-bin/target_detail.cgi?targetID=598191) | 815 | 50 | hsa-miR-148a-3p | [IL1RL1](http://www.ncbi.nlm.nih.gov/entrez/query.fcgi?db=gene&cmd=Retrieve&dopt=full_report&list_uids=9173) | interleukin 1 receptor like 1 |
| [Details](http://mirdb.org/cgi-bin/target_detail.cgi?targetID=598243) | 816 | 50 | hsa-miR-148a-3p | [TAGLN2](http://www.ncbi.nlm.nih.gov/entrez/query.fcgi?db=gene&cmd=Retrieve&dopt=full_report&list_uids=8407) | transgelin 2 |
| [Details](http://mirdb.org/cgi-bin/target_detail.cgi?targetID=598549) | 817 | 50 | hsa-miR-148a-3p | [PREX1](http://www.ncbi.nlm.nih.gov/entrez/query.fcgi?db=gene&cmd=Retrieve&dopt=full_report&list_uids=57580) | phosphatidylinositol-3,4,5-trisphosphate dependent Rac exchange factor 1 |
| [Details](http://mirdb.org/cgi-bin/target_detail.cgi?targetID=598404) | 818 | 50 | hsa-miR-148a-3p | [ETS1](http://www.ncbi.nlm.nih.gov/entrez/query.fcgi?db=gene&cmd=Retrieve&dopt=full_report&list_uids=2113) | ETS proto-oncogene 1, transcription factor |
| [Details](http://mirdb.org/cgi-bin/target_detail.cgi?targetID=598575) | 819 | 50 | hsa-miR-148a-3p | [TAX1BP1](http://www.ncbi.nlm.nih.gov/entrez/query.fcgi?db=gene&cmd=Retrieve&dopt=full_report&list_uids=8887) | Tax1 binding protein 1 |
| [Details](http://mirdb.org/cgi-bin/target_detail.cgi?targetID=597869) | 820 | 50 | hsa-miR-148a-3p | [JPH3](http://www.ncbi.nlm.nih.gov/entrez/query.fcgi?db=gene&cmd=Retrieve&dopt=full_report&list_uids=57338) | junctophilin 3 |
| [Details](http://mirdb.org/cgi-bin/target_detail.cgi?targetID=597865) | 821 | 50 | hsa-miR-148a-3p | [GOLT1A](http://www.ncbi.nlm.nih.gov/entrez/query.fcgi?db=gene&cmd=Retrieve&dopt=full_report&list_uids=127845) | golgi transport 1A |
| [Details](http://mirdb.org/cgi-bin/target_detail.cgi?targetID=598462) | 822 | 50 | hsa-miR-148a-3p | [GLCE](http://www.ncbi.nlm.nih.gov/entrez/query.fcgi?db=gene&cmd=Retrieve&dopt=full_report&list_uids=26035) | glucuronic acid epimerase |
| [Details](http://mirdb.org/cgi-bin/target_detail.cgi?targetID=597989) | 823 | 50 | hsa-miR-148a-3p | [ZBTB7A](http://www.ncbi.nlm.nih.gov/entrez/query.fcgi?db=gene&cmd=Retrieve&dopt=full_report&list_uids=51341) | zinc finger and BTB domain containing 7A |
| [Details](http://mirdb.org/cgi-bin/target_detail.cgi?targetID=597918) | 824 | 50 | hsa-miR-148a-3p | [SOX5](http://www.ncbi.nlm.nih.gov/entrez/query.fcgi?db=gene&cmd=Retrieve&dopt=full_report&list_uids=6660) | SRY-box 5 |
| [Details](http://mirdb.org/cgi-bin/target_detail.cgi?targetID=598314) | 825 | 50 | hsa-miR-148a-3p | [C1orf74](http://www.ncbi.nlm.nih.gov/entrez/query.fcgi?db=gene&cmd=Retrieve&dopt=full_report&list_uids=148304) | chromosome 1 open reading frame 74 |
| [Details](http://mirdb.org/cgi-bin/target_detail.cgi?targetID=598210) | 826 | 50 | hsa-miR-148a-3p | [RIBC1](http://www.ncbi.nlm.nih.gov/entrez/query.fcgi?db=gene&cmd=Retrieve&dopt=full_report&list_uids=158787) | RIB43A domain with coiled-coils 1 |
| [Details](http://mirdb.org/cgi-bin/target_detail.cgi?targetID=598205) | 827 | 50 | hsa-miR-148a-3p | [PBXIP1](http://www.ncbi.nlm.nih.gov/entrez/query.fcgi?db=gene&cmd=Retrieve&dopt=full_report&list_uids=57326) | PBX homeobox interacting protein 1 |
| [Details](http://mirdb.org/cgi-bin/target_detail.cgi?targetID=597927) | 828 | 50 | hsa-miR-148a-3p | [ABHD15](http://www.ncbi.nlm.nih.gov/entrez/query.fcgi?db=gene&cmd=Retrieve&dopt=full_report&list_uids=116236) | abhydrolase domain containing 15 |
| [Details](http://mirdb.org/cgi-bin/target_detail.cgi?targetID=597909) | 829 | 50 | hsa-miR-148a-3p | [HIPK3](http://www.ncbi.nlm.nih.gov/entrez/query.fcgi?db=gene&cmd=Retrieve&dopt=full_report&list_uids=10114) | homeodomain interacting protein kinase 3 |
| [Details](http://mirdb.org/cgi-bin/target_detail.cgi?targetID=598374) | 830 | 50 | hsa-miR-148a-3p | [POLM](http://www.ncbi.nlm.nih.gov/entrez/query.fcgi?db=gene&cmd=Retrieve&dopt=full_report&list_uids=27434) | DNA polymerase mu |
| [Details](http://mirdb.org/cgi-bin/target_detail.cgi?targetID=598440) | 831 | 50 | hsa-miR-148a-3p | [TNFSF8](http://www.ncbi.nlm.nih.gov/entrez/query.fcgi?db=gene&cmd=Retrieve&dopt=full_report&list_uids=944) | TNF superfamily member 8 |
| [Details](http://mirdb.org/cgi-bin/target_detail.cgi?targetID=597941) | 832 | 50 | hsa-miR-148a-3p | [PDE5A](http://www.ncbi.nlm.nih.gov/entrez/query.fcgi?db=gene&cmd=Retrieve&dopt=full_report&list_uids=8654) | phosphodiesterase 5A |
| [Details](http://mirdb.org/cgi-bin/target_detail.cgi?targetID=598368) | 833 | 50 | hsa-miR-148a-3p | [SLC30A4](http://www.ncbi.nlm.nih.gov/entrez/query.fcgi?db=gene&cmd=Retrieve&dopt=full_report&list_uids=7782) | solute carrier family 30 member 4 |
| [Details](http://mirdb.org/cgi-bin/target_detail.cgi?targetID=598626) | 834 | 50 | hsa-miR-148a-3p | [SYNCRIP](http://www.ncbi.nlm.nih.gov/entrez/query.fcgi?db=gene&cmd=Retrieve&dopt=full_report&list_uids=10492) | synaptotagmin binding cytoplasmic RNA interacting protein |
| [Details](http://mirdb.org/cgi-bin/target_detail.cgi?targetID=598135) | 835 | 50 | hsa-miR-148a-3p | [MYLK4](http://www.ncbi.nlm.nih.gov/entrez/query.fcgi?db=gene&cmd=Retrieve&dopt=full_report&list_uids=340156) | myosin light chain kinase family member 4 |
| [Details](http://mirdb.org/cgi-bin/target_detail.cgi?targetID=598670) | 836 | 50 | hsa-miR-148a-3p | [ESYT2](http://www.ncbi.nlm.nih.gov/entrez/query.fcgi?db=gene&cmd=Retrieve&dopt=full_report&list_uids=57488) | extended synaptotagmin 2 |
| [Details](http://mirdb.org/cgi-bin/target_detail.cgi?targetID=598302) | 837 | 50 | hsa-miR-148a-3p | [PHC3](http://www.ncbi.nlm.nih.gov/entrez/query.fcgi?db=gene&cmd=Retrieve&dopt=full_report&list_uids=80012) | polyhomeotic homolog 3 |
| [Details](http://mirdb.org/cgi-bin/target_detail.cgi?targetID=598432) | 838 | 50 | hsa-miR-148a-3p | [VWA5A](http://www.ncbi.nlm.nih.gov/entrez/query.fcgi?db=gene&cmd=Retrieve&dopt=full_report&list_uids=4013) | von Willebrand factor A domain containing 5A |
| [Details](http://mirdb.org/cgi-bin/target_detail.cgi?targetID=598202) | 839 | 50 | hsa-miR-148a-3p | [SPON2](http://www.ncbi.nlm.nih.gov/entrez/query.fcgi?db=gene&cmd=Retrieve&dopt=full_report&list_uids=10417) | spondin 2 |

Data generated from the online database for prediction of functional microRNA targets (Chen & Wang, 2020)

Chen, Y., & Wang, X. (2020). MiRDB: An online database for prediction of functional microRNA targets. *Nucleic Acids Research*, *48*(D1), D127–D131. https://doi.org/10.1093/nar/gkz757
