# Supplementary material for: The Role of microRNAs in Organismal and Skin Aging
Source: Int J Mol Sci. 2020 Jul 25;21(15):5281. doi: 10.3390/ijms21155281 (PMC7432402; doi:10.3390/ijms21155281)
Supplement: Supplementary file 1 [file ijms-21-05281-s001.zip › Supplementary Table 1. miRNAs in long-lived individuals.aging.docx]

**Table 3. MicroRNA expression in the skin of long-lived individuals with a prediction of functional miRNA targets**

| **MicroRNAs in long‐lived individuals** | **Skin aging** | **Predicted gene targets for microRNA*** | **Role of the gene*#** |
| --- | --- | --- | --- |
| **Up‐regulated miRNAs in long‐lived individuals** | | | |
| miR‐574‐3p | Up-regulated in senescent fibroblasts (Elsharawy et al., 2012; Markopoulos et al., 2017) | [ADAM28](http://www.ncbi.nlm.nih.gov/entrez/query.fcgi?db=gene&cmd=Retrieve&dopt=full_report&list_uids=10863) | involved in cell-cell and cell-matrix interactions, muscle development, and neurogenesis (PMID: 23010875; 29254295) |
|  |  | [ATP2A2](http://www.ncbi.nlm.nih.gov/entrez/query.fcgi?db=gene&cmd=Retrieve&dopt=full_report&list_uids=488) | catalyzes calcium transport from the cytosol into the sarcoplasmic reticulum; regulates the contraction/relaxation cycle; this gene mutation causes keratosis follicularis, associated with loss of adhesion between epidermal cells and abnormal keratinization (PMID: 30663125) |
|  |  | [BACE1](http://www.ncbi.nlm.nih.gov/entrez/query.fcgi?db=gene&cmd=Retrieve&dopt=full_report&list_uids=23621) | involved in the amyloid beta peptide generation from amyloid precursor protein that accumulated in Alzheimer's disease patients in amyloid beta plaques (PMID: 29581300) |
|  |  | [BTG3](http://www.ncbi.nlm.nih.gov/entrez/query.fcgi?db=gene&cmd=Retrieve&dopt=full_report&list_uids=10950) | demonstrate antiproliferative properties; involved in neurogenesis (PMID: 29270670); highly presented in skin |
|  |  | [CLTC](http://www.ncbi.nlm.nih.gov/entrez/query.fcgi?db=gene&cmd=Retrieve&dopt=full_report&list_uids=1213) | implicated in the intracellular trafficking of receptors and endocytosis of a different of macromolecules by specialized organelles (PMID 29973118); regulates lymphocytes migration (PMID: 27405273) |
|  |  | [CSE1L](http://www.ncbi.nlm.nih.gov/entrez/query.fcgi?db=gene&cmd=Retrieve&dopt=full_report&list_uids=1434) | in the cytoplasm cooperate with RANBP1 and RANGAP1; participates in apoptosis and in cell proliferation; regulates lymphocyte migration (PMID: 27405273; 27941559) |
|  |  | [IL6](http://www.ncbi.nlm.nih.gov/entrez/query.fcgi?db=gene&cmd=Retrieve&dopt=full_report&list_uids=3569) | involved in acute/chronic inflammation, inflammation-associated conditions, maturation of B cells (PMID: 32386656 ) |
|  |  | [RXRA](http://www.ncbi.nlm.nih.gov/entrez/query.fcgi?db=gene&cmd=Retrieve&dopt=full_report&list_uids=6256) | modulate retinoic acid-mediated gene activation; regulates cellular senescence (PMID: 30216632); highly presented in skin |
|  |  | [USP45](http://www.ncbi.nlm.nih.gov/entrez/query.fcgi?db=gene&cmd=Retrieve&dopt=full_report&list_uids=85015) | modulate the DNA repair ability of XPF-ERCC1 endonuclease (PMID: 25538220); highly presented in skin |
| miR‐30c | Up-regulated in photoaged primary human fibroblasts irradiated UVA (Elsharawy et al., 2012; Fuster et al., 2013; Li, Zhou, Hua, Guo, & Luo, 2013)  . | [B3GNT5](http://www.ncbi.nlm.nih.gov/entrez/query.fcgi?db=gene&cmd=Retrieve&dopt=full_report&list_uids=84002) | essential component of glycolipids to express the Lewis X epitopes (PMID: 20734064) |
|  |  | [BNIP3L](http://www.ncbi.nlm.nih.gov/entrez/query.fcgi?db=gene&cmd=Retrieve&dopt=full_report&list_uids=665) | binds to Bcl-2 pro-apoptotic protein; directly targets mitochondria; stimulate apoptosis: induces loss of membrane potential and the secretion of cytochrome c (PMID: 30618073) |
|  |  | [CNR1](http://www.ncbi.nlm.nih.gov/entrez/query.fcgi?db=gene&cmd=Retrieve&dopt=full_report&list_uids=1268) | known as CB1, G-protein coupled receptor; inhibit adenylate cyclase activity in a dose-dependent, stereoselective and pertussis toxin-sensitive manner; abundantly present in the CNS and throughout the body (PMID: 31599069) |
|  |  | [COL25A1](http://www.ncbi.nlm.nih.gov/entrez/query.fcgi?db=gene&cmd=Retrieve&dopt=full_report&list_uids=84570) | a brain-specific membrane associated collagen; encoded protein, CLAC (collagenous Alzheimer amyloid plaque component), CLAC inhibits rather than facilitates amyloid fibril elongation (PMID:: 16300410); prone to amyloid plaque formation (PMID:: 19548013) |
|  |  | [EED](http://www.ncbi.nlm.nih.gov/entrez/query.fcgi?db=gene&cmd=Retrieve&dopt=full_report&list_uids=8726) | maintains the transcriptional repressive state of genes over successive cell generations via histone deacetylation; cooperates with enhancer of zeste 2, the cytoplasmic tail of integrin beta7, immunodeficiency virus type 1 (HIV-1) MA protein, and histone deacetylase proteins; regulates integrin function (PMID: 31395608); highly presented in skin |
|  |  | [MAP4K4](http://www.ncbi.nlm.nih.gov/entrez/query.fcgi?db=gene&cmd=Retrieve&dopt=full_report&list_uids=9448) | activates MAPK8/JNK (PMID: 28306189) |
|  |  | [NFAT5](http://www.ncbi.nlm.nih.gov/entrez/query.fcgi?db=gene&cmd=Retrieve&dopt=full_report&list_uids=10725) | activates T cells family of transcription factors; involved in inducible gene transcription during the immune response; modulated gene expression induced by osmotic stress in mammalian cells (PMID: 28485155); highly presented in skin |
|  |  | [SMAD1](http://www.ncbi.nlm.nih.gov/entrez/query.fcgi?db=gene&cmd=Retrieve&dopt=full_report&list_uids=4086) | modulates the signals of the bone morphogenetic proteins (BMPs), which are participated in cell growth, morphogenesis, development and immune responses and apoptosis; in the phosphorylated form of this protein generate a complex with SMAD4 (PMID:: 31022296); highly presented in skin |
|  |  | [WDR7](http://www.ncbi.nlm.nih.gov/entrez/query.fcgi?db=gene&cmd=Retrieve&dopt=full_report&list_uids=23335) | regulate cell cycle progression, signal transduction, apoptosis; binds directly with Rab3A GDP/GTP exchange protein and indirectly with Rab3A GDP/GTP activating protein, which modulate Rab3 small G protein family members involved in control of the calcium-dependant exocytosis of neurotransmitters (PMID: 12786944) |
| **Down‐regulated miRNAs in long‐lived individuals** | | | |
| let‐7d | Up-regulated in senescent fibroblasts  (Elsharawy et al., 2012; Markopoulos et al., 2017; Rossi & Gorospe, 2020) | [CCND2](http://www.ncbi.nlm.nih.gov/entrez/query.fcgi?db=gene&cmd=Retrieve&dopt=full_report&list_uids=894) | regulator of CDK kinases; forms a complex with CDK4 or CDK6; required for cell cycle G1/S transition; involved in the phosphorylation of tumor suppressor protein Rb (PMID: 30193733) |
|  |  | [COL1A2](http://www.ncbi.nlm.nih.gov/entrez/query.fcgi?db=gene&cmd=Retrieve&dopt=full_report&list_uids=1278) | a fibril-forming collagen present in connective tissues and is ubiquitous in dermis, cornea, bone, and tendon (PMID:: **30683734**); gene mutations linked to Ehlers-Danlos syndrome type VIIB and recessive classical type, atypical Marfan syndrome, idiopathic osteoporosis, and osteogenesis imperfecta types I-IV(PMID: 29636545) |
|  |  | [COL3A1](http://www.ncbi.nlm.nih.gov/entrez/query.fcgi?db=gene&cmd=Retrieve&dopt=full_report&list_uids=1281) | a fibrillar collagen that is present in extensible connective tissues, including skin, intestine and the vascular system, lung, uterus; associated with type I collagen in wound healing; gene mutations are found in aortic and arterial aneurysms (PMID: 31075413), Ehlers-Danlos syndrome types IV (PMID: 30837697) |
|  |  | [GPR26](http://www.ncbi.nlm.nih.gov/entrez/query.fcgi?db=gene&cmd=Retrieve&dopt=full_report&list_uids=2849) | play role in cellular responses to environmental stimuli, hormones, and neurotransmitters (PMID: 17363172); involved in neurodegenerative diseases (PMID: 26303144) |
|  |  | [HAS2](http://www.ncbi.nlm.nih.gov/entrez/query.fcgi?db=gene&cmd=Retrieve&dopt=full_report&list_uids=3037) | hyaluronic acid (HA) is a component of the extracellular matrix; mediate space filling, lubrication of joints, facilitate cellular migration, wound healing and tissue repair to supply scaffolding for fibroblasts and new blood vessels (PMID: 28188289); modulate leukocytes homing via leukocyte receptor CD44; decreased expression of HA receptors associated with dermal aging, while overexpression – correlated with tumor metastasis (PMID: 31102316) |
|  |  | [IGF1R](http://www.ncbi.nlm.nih.gov/entrez/query.fcgi?db=gene&cmd=Retrieve&dopt=full_report&list_uids=3480) | binds insulin-like growth factor; enhances cell survival and overexpressed in malignant tissues where it acts as an anti-apoptotic factor; ubiquitously expressed in skin (PMID: 31122679) |
|  |  | [KRT5](http://www.ncbi.nlm.nih.gov/entrez/query.fcgi?db=gene&cmd=Retrieve&dopt=full_report&list_uids=3852) | type II cytokeratins component of basic or neutral proteins; expressed in the basal layer of the epidermis with family member KRT14; involved in differentiation of simple and stratified epithelial tissues; gene mutations associated with epidermolysis bullosa simplex (PMID: 31302245) |
| miR-17 | Down-regulated in replicative senescence and organismal aging (Elsharawy et al., 2012; Grillari, Hackl, & Grillari-Voglauer, 2010; Hackl et al., 2010; Kuo, Wu, & Yang, 2019; Otsuka et al., 2008) | [CCND1](http://www.ncbi.nlm.nih.gov/entrez/query.fcgi?db=gene&cmd=Retrieve&dopt=full_report&list_uids=595) | regulator of CDK kinases; forms a complex with CDK4 or CDK6; required for cell cycle G1/S transition; involved in the phosphorylation of tumor suppressor protein Rb; gene overexpression affects cell cycle progression and are found in numerous cancers (PMID: 31450922) |
|  |  | [CNRIP1](http://www.ncbi.nlm.nih.gov/entrez/query.fcgi?db=gene&cmd=Retrieve&dopt=full_report&list_uids=25927) | interacts with the C-terminal tail of cannabinoid receptor 1(PMID: 31614728) |
|  |  | [GAB1](http://www.ncbi.nlm.nih.gov/entrez/query.fcgi?db=gene&cmd=Retrieve&dopt=full_report&list_uids=2549) | mediate branching tubulogenesis; involved in cellular growth response, transformation and apoptosis (PMID: 30697991) |
|  |  | [MMP24](http://www.ncbi.nlm.nih.gov/entrez/query.fcgi?db=gene&cmd=Retrieve&dopt=full_report&list_uids=10893) | expressed at the cell surface; degrades extracellular matrix in normal physiological processes including embryonic development, reproduction, and tissue remodeling; in pathological processes involved in arthritis and metastasis (PMID: **24669030)** |
|  |  | [PAK5](http://www.ncbi.nlm.nih.gov/entrez/query.fcgi?db=gene&cmd=Retrieve&dopt=full_report&list_uids=57144) | effector of Rac/Cdc42 GTPases, that regulate cytoskeletal dynamics, cell cycle progression, proliferation, and survival signalling (PMID: 27323857) |
|  |  | [SIRT5](http://www.ncbi.nlm.nih.gov/entrez/query.fcgi?db=gene&cmd=Retrieve&dopt=full_report&list_uids=23408) | mediates mitochondrial enzymes activity in response to fasting and calorie restriction; supply cellular antioxidant defense (PMID: 30559718) |
| miR-20a | Down-regulated in skin fibroblasts replicative senescence and organismal aging  (Elsharawy et al., 2012; Hackl et al., 2010) | [ATG2B](http://www.ncbi.nlm.nih.gov/entrez/query.fcgi?db=gene&cmd=Retrieve&dopt=full_report&list_uids=55102) | implicated in autophagosome formation and apoptosis (PMID: 28800131); related to myeloid malignancies predisposition (PMID: 26280900); ubiquitously expressed in skin |
|  |  | [BMP2](http://www.ncbi.nlm.nih.gov/entrez/query.fcgi?db=gene&cmd=Retrieve&dopt=full_report&list_uids=650) | encodes a secreted ligand of the TGF-β superfamily of proteins and bind different TGF-β receptors; recruits and activates SMAD family TFs; involved in bone, cartilage (PMID 31413943), and epidermal keratinocytes development (PMID: 21984808) |
|  |  | [HIF1A](http://www.ncbi.nlm.nih.gov/entrez/query.fcgi?db=gene&cmd=Retrieve&dopt=full_report&list_uids=3091) | regulates cellular and systemic homeostatic response to hypoxia by increasing oxygen delivery or facilitates metabolic adaptation to hypoxia; modulates energy metabolism, angiogenesis, and apoptosis; directly involved in embryonic vascularization, pathogenesis of ischemic disease and tumor angiogenesis (PMID: 31744467) |
|  |  | [JAK1](http://www.ncbi.nlm.nih.gov/entrez/query.fcgi?db=gene&cmd=Retrieve&dopt=full_report&list_uids=3716) | phosphorylates STAT proteins (signal transducers and activators of transcription); mediate INF-α/β and INF-ɣ signal transduction; associated with cutaneous aging, and skin diseases such as vitiligo and psoriasis) (PMID: 29127481). |
|  |  | [KRT10](http://www.ncbi.nlm.nih.gov/entrez/query.fcgi?db=gene&cmd=Retrieve&dopt=full_report&list_uids=3858) | component of the epithelial cells cytoskeleton along with actin microfilaments and microtubules; involved in renewal of the cutaneous barrier; gene mutations associated with epidermolytic hyperkeratosis (PMID: 26373619) |
|  |  | [PDGFRA](http://www.ncbi.nlm.nih.gov/entrez/query.fcgi?db=gene&cmd=Retrieve&dopt=full_report&list_uids=5156) | stimulate development and cellular maturation of mesenchymal origin; involved in papillary and reticular fibroblasts formation, organ development, wound healing (PMID: 29244903), and tumor progression; gene mutations linked to idiopathic hypereosinophilic syndrome, and numerous cancers (PMID: 3059807) |
| miR-27a | Up-regulation in UVB-photoaged keratinocytes.  (Zhang, Yang, Yang, & Guo, 2020) | [CDK6](http://www.ncbi.nlm.nih.gov/entrez/query.fcgi?db=gene&cmd=Retrieve&dopt=full_report&list_uids=1021) | involved in cell cycle G1 phase progression and G1/S transition; controlled by D-type cyclins and members of INK4 family of CDK inhibitors; mediate tumor suppressor protein Rb activity; gene mutation resulted in decreased cell proliferation, altered cell motility and polarity (PMID: 30635286); modified gene expression associated with numerous human cancers (PMID: 29806604; 18381414). |
|  |  | [CNR1](http://www.ncbi.nlm.nih.gov/entrez/query.fcgi?db=gene&cmd=Retrieve&dopt=full_report&list_uids=1268) | known as CB1, G-protein coupled receptor; inhibit adenylate cyclase activity in a dose-dependent, stereoselective and pertussis toxin-sensitive manner; abundantly present in the CNS and throughout the body (PMID: 31599069) |
|  |  | [COL5A1](http://www.ncbi.nlm.nih.gov/entrez/query.fcgi?db=gene&cmd=Retrieve&dopt=full_report&list_uids=1289) | fibrillar collagen that present in tissues containing type I collagen; regulates the assembly of heterotypic fibers composed of both type I and type V collagen; altered expression associated with fibrosis (PMID: 31178721); gene mutations linked to Ehlers-Danlos syndrome, types I and II (PMID 29520887) |
|  |  | [CSF1](http://www.ncbi.nlm.nih.gov/entrez/query.fcgi?db=gene&cmd=Retrieve&dopt=full_report&list_uids=1435) | controls the production, differentiation, and function of macrophages (PMID: 30816518); mediate homeostasis and development of LCs (PMID: 27191270) |
|  |  | [MAPK8IP3](http://www.ncbi.nlm.nih.gov/entrez/query.fcgi?db=gene&cmd=Retrieve&dopt=full_report&list_uids=23162) | mediates activity of different protein kinases of the JNK signaling pathway (PMID: 30969891) |
|  |  | [MITF](http://www.ncbi.nlm.nih.gov/entrez/query.fcgi?db=gene&cmd=Retrieve&dopt=full_report&list_uids=4286) | mediates melanocyte development; controls survival, proliferation, pigmentation, invasion, and oxygen stress in melanocytes (PMID: 32034251); gene mutations associated with auditory-pigmentary syndromes, including Waardenburg syndrome type 2 and Tietz syndrome (PMID 30549420) |
| miR-93 | Highly expressed in the epidermis, declined with age (Ameling et al., 2015; Elsharawy et al., 2012) | [E2F1](http://www.ncbi.nlm.nih.gov/entrez/query.fcgi?db=gene&cmd=Retrieve&dopt=full_report&list_uids=1869) | binds to retinoblastoma protein pRB; can modulate cell proliferation and p53-dependent/independent apoptosis (PMID: 31124185) |
|  |  | [MAP3K2](http://www.ncbi.nlm.nih.gov/entrez/query.fcgi?db=gene&cmd=Retrieve&dopt=full_report&list_uids=10746) | directly phosphorylate and activate Ikappa B kinases, and thereby involved in NF-κB signaling pathway; bind and activate protein kinase C-related kinase 2; highly presented in skin (PMID: 29309787) |
|  |  | [MMP3](http://www.ncbi.nlm.nih.gov/entrez/query.fcgi?db=gene&cmd=Retrieve&dopt=full_report&list_uids=4314) | mediate the breakdown of extracellular matrix in normal physiological processes: embryonic development, reproduction, and tissue remodeling, wound repair and pathological conditions, including cancerogenesis, metastasis, atherosclerosis and arthritis; play role in degradation of collagens III, IV, IX, and X, fibronectin, cartilage proteoglycans, and laminin (PMID: 31055827) |
|  |  | [SMAD4](http://www.ncbi.nlm.nih.gov/entrez/query.fcgi?db=gene&cmd=Retrieve&dopt=full_report&list_uids=4089) | activated in TGF-β signaling; regulate the transcription of target genes; tumor suppressor; inhibits epithelial cell proliferation; ubiquitously expressed in skin (PMID: 31082421) |
|  |  | [TGFBR2](http://www.ncbi.nlm.nih.gov/entrez/query.fcgi?db=gene&cmd=Retrieve&dopt=full_report&list_uids=7048) | binds TGF-β; phosphorylates proteins, which regulate in the nucleus transcription of genes that maintain cell proliferation, wound healing, immunosuppression, cell cycle arrest, and cancerogenesis (PMID: 29888864) |
| miR-106a | Down-regulated in replicative senescence and organismal aging (Elsharawy et al., 2012; Hackl et al., 2010; Stratz et al., 2012) | [CNRIP1](http://www.ncbi.nlm.nih.gov/entrez/query.fcgi?db=gene&cmd=Retrieve&dopt=full_report&list_uids=25927) | interacts with the C-terminal tail of cannabinoid receptor 1(PMID: 31614728) |
|  |  | [E2F3](http://www.ncbi.nlm.nih.gov/entrez/query.fcgi?db=gene&cmd=Retrieve&dopt=full_report&list_uids=1871) | involved in the cell cycle regulation via direct interaction with the pRB; protects dermal fibroblasts from UVB-induced premature senescence as it regulates the senescence-related genes (e.g., p53 and p21WAF-1) (PMID: 23983607); gene alteration associated with multiple human cancers (PMID: 30740539) |
|  |  | [FGF4](http://www.ncbi.nlm.nih.gov/entrez/query.fcgi?db=gene&cmd=Retrieve&dopt=full_report&list_uids=2249) | mediates various mitogenic and cell survival activities; regulates embryonic development, cell growth, morphogenesis, and tissue repair (PMID: 18192227); appears oncogenic transforming activity, involved in tumor growth and invasion (PMID: 29936056) |
|  |  | [NOTCH2NLA](http://www.ncbi.nlm.nih.gov/entrez/query.fcgi?db=gene&cmd=Retrieve&dopt=full_report&list_uids=388677) | involved in cellular differentiation, alternative splicing; modulate brain neuronal development (PMID: 29856955); interacts with neutrophil elastase; involved in hereditary neutropenia (PMID: 14673143) |
| miR-148a | Up-regulated in photoaged primary human fibroblasts irradiated with UVA (Elsharawy et al., 2012; Li et al., 2013). | [CNR1](http://www.ncbi.nlm.nih.gov/entrez/query.fcgi?db=gene&cmd=Retrieve&dopt=full_report&list_uids=1268) | known as CB1, G-protein coupled receptor; inhibit adenylate cyclase activity in a dose-dependent, stereoselective and pertussis toxin-sensitive manner; abundantly present in the CNS and throughout the body (PMID: 31599069) |
|  |  | [ERBB3](http://www.ncbi.nlm.nih.gov/entrez/query.fcgi?db=gene&cmd=Retrieve&dopt=full_report&list_uids=2065) | a member of the epidermal growth factor receptor (EGFR) family of receptor tyrosine kinases; maintains the activation of cell proliferation and differentiation pathways; reduced expression linked to accelerated apoptosis and inhibited cell growth (PMID: 31178721); gene deregulation and/or overexpression of its protein associated with bladder, breast, and prostate cancers (PMID: 29413684) |
|  |  | [PTEN](http://www.ncbi.nlm.nih.gov/entrez/query.fcgi?db=gene&cmd=Retrieve&dopt=full_report&list_uids=5728) | negatively regulates intracellular levels of PIP_3_ in cells; facilitates energy metabolism in the mitochondria; acts as a tumor suppressor by negatively regulating AKT/PKB signaling pathway (PMID: 31530568); phosphatidylinositol 3-kinase/PTEN/AKT signaling pathway is involved in proliferation, migration, cell growth, cell survival, and tumorigenesis (PMID: 25357050) |
|  |  | [SIRT7](http://www.ncbi.nlm.nih.gov/entrez/query.fcgi?db=gene&cmd=Retrieve&dopt=full_report&list_uids=51547) | nuclear sirtuin that mediates the functionality of TFs and cofactors in the most of tissues with the cellular responses to energy demands; regulates telomere length and integrity via deacetylase activity that also facilitates chromatin condensation and histone modification (PMID: 31528700); modulates the TGF-β1-induced proliferation and migration (PMID 29843083); like SIRT4 reduces fatty acid oxidation and insulin secretion (PMID: **30559718)** |
| miR‐222 | Up-regulated during replicative senescence (Elsharawy et al., 2012; Felicetti et al., 2016; Liu, Cheng, Yang, Xu, & Zhang, 2012; Markopoulos et al., 2017) | [CASP3](http://www.ncbi.nlm.nih.gov/entrez/query.fcgi?db=gene&cmd=Retrieve&dopt=full_report&list_uids=836) | modulates a central role in the execution-phase of cell apoptosis; activates caspases 6, 7, and 9 itself is processed by caspases 8, 9, and 10; associated with the cleavage of amyloid-beta 4A precursor protein, which is related to neuronal death in Alzheimer's disease (PMID: 3090893); involved in the colon cancer cells migration, invasion and metastasis (MID: 29524226) |
|  |  | [CNR1](http://www.ncbi.nlm.nih.gov/entrez/query.fcgi?db=gene&cmd=Retrieve&dopt=full_report&list_uids=1268) | known as CB1, G-protein coupled receptor; inhibit adenylate cyclase activity in a dose-dependent, stereoselective and pertussis toxin-sensitive manner; abundantly present in the CNS and throughout the body (PMID: 31599069) |
|  |  | [GNAI3](http://www.ncbi.nlm.nih.gov/entrez/query.fcgi?db=gene&cmd=Retrieve&dopt=full_report&list_uids=2773) | involved in numerous transmembrane signaling pathways; gene mutations affect downstream targets in the G protein-coupled endothelin receptor pathway, and linked with auriculocondylar syndrome (PMID: 25026904); highly expressed in skin. |
|  |  | [SPTBN1](http://www.ncbi.nlm.nih.gov/entrez/query.fcgi?db=gene&cmd=Retrieve&dopt=full_report&list_uids=6711) | is an actin crosslinking and molecular scaffold protein that connects the plasma membrane to the actin cytoskeleton, mediates cell shape, arrangement of transmembrane proteins, and organization of organelles (PMID: 29555987) |

*, data generated from the online database for prediction of functional microRNA targets (Chen & Wang, 2020);

#, Gene description based on materials of <https://www.ncbi.nlm.nih.gov/gene>?

ADAM28, ADAM metallopeptidase domain 28; ATG2B, autophagy related 2B; ATP2A2, ATPase sarcoplasmic/endoplasmic reticulum Ca2+ transporting 2; B3GNT5, UDP-GlcNAc:betaGal beta-1,3-N-acetylglucosaminyltransferase 5; BACE1, beta-secretase 1; BMP2, bone morphogenetic protein 2; BNIP3L, BCL2 interacting protein 3 like; BTG3, BTG anti-proliferation factor 3; CASP3, caspase 3; CCND1, cyclin D1; CCND2, cyclin D2; CDK6, cyclin dependent kinase 6; CLTC, clathrin heavy chain; CNR1, cannabinoid receptor 1 (CB1); CNRIP1 cannabinoid receptor interacting protein 1; COL1A2, collagen type I alpha 2 chain; COL3A1, collagen type III alpha 1 chain; COL5A1, collagen type V alpha 1 chain; COL25A1, collagen type XXV alpha 1 chain; CSE1L, chromosome segregation 1 like; CSF1, colony stimulating factor 1; E2F3, E2F transcription factor 3;  EED, embryonic ectoderm development; EGFR, epidermal growth factor receptor; ERBB3, erb-b2 receptor tyrosine kinase 3; FGF4, fibroblast growth factor 4; GAB1, GRB2 associated binding protein 1; GNAI3, G protein subunit alpha i3; GPR26, G protein-coupled receptor 26; HAS2, hyaluronan synthase 2; HIF1A, hypoxia inducible factor 1 subunit alpha; IGF1R, insulin like growth factor 1 receptor; IL6,  interleukin 6; INF-α/β/ɣ, interferon-alpha/beta/gamma; JAK1, Janus kinase 1;  KRT5, keratin 5; KRT10, keratin 10; LCs, Langerhans cells; MAP3K2, mitogen-activated protein kinase kinase kinase 2; MAP4K4, mitogen-activated protein kinase kinase kinase kinase 4; MITF,  melanocyte inducing transcription factor; MMP3, matrix metallopeptidase 3; MMP24, matrix metallopeptidase 24; NFAT5, nuclear factor of activated T cells 5; NOTCH2NLA, notch 2 N-terminal like A; PAK5, p21 (RAC1) activated kinase 5; PDGFRA, platelet derived growth factor receptor alpha;  PIP_3,_ phosphatidylinositol-3,4,5-trisphosphate; pRB, retinoblastoma protein; PTEN, phosphatase and tensin homolog; RXRA, retinoid X receptor alpha; SIRT5, sirtuin 5; SIRT7, sirtuin 7;  SMAD1, SMAD family member 1; SMAD4, SMAD family member 4; SPTBN1, spectrin beta, non-erythrocytic 1; TF, transcription factors; TGF-β, transforming growth factor-beta; TGFBR2, transforming growth factor beta receptor 2; USP45, ubiquitin specific peptidase 45; WDR7, WD repeat domain 7

**References**

Ameling, S., Kacprowski, T., Chilukoti, R. K., Malsch, C., Liebscher, V., Suhre, K., … Völker, U. (2015). Associations of circulating plasma microRNAs with age, body mass index and sex in a population-based study. *BMC Medical Genomics*, *8*(1), 1–9. https://doi.org/10.1186/s12920-015-0136-7

Chen, Y., & Wang, X. (2020). MiRDB: An online database for prediction of functional microRNA targets. *Nucleic Acids Research*, *48*(D1), D127–D131. https://doi.org/10.1093/nar/gkz757

Elsharawy, A., Keller, A., Flachsbart, F., Wendschlag, A., Jacobs, G., Kefer, N., … Nebel, A. (2012). Genome-wide miRNA signatures of human longevity. *Aging Cell*, *11*(4), 607–616. https://doi.org/10.1111/j.1474-9726.2012.00824.x

Felicetti, F., Feo, A. De, Coscia, C., Puglisi, R., Pedini, F., Pasquini, L., … Carè, A. (2016). Exosome‑mediated transfer of miR‑222 is sufficient to increase tumor malignancy in melanoma. *Journal of Translational Medicine*, *14*, 56. https://doi.org/10.1186/s12967-016-0811-2

Fuster, Ó., Llop, M., Dolz, S., García, P., Such, E., Luna, I., … Barragán, E. (2013). Adverse prognostic value of MYBL2 overexpression and association with microRNA-30 family in acute myeloid leukemia patients. *Leukemia Research*, *37*(12), 1690–1696. https://doi.org/10.1016/j.leukres.2013.09.015

Grillari, J., Hackl, M., & Grillari-Voglauer, R. (2010). miR-17-92 cluster: ups and downs in cancer and aging. *Biogerontology*, *11*(4), 501–506. https://doi.org/10.1007/s10522-010-9272-9

Hackl, M., Brunner, S., Fortschegger, K., Schreiner, C., Micutkova, L., Mück, C., … Grillari, J. (2010). miR-17, miR-19b, miR-20a, and miR-106a are down-regulated in human aging. *Aging Cell*, *9*(2), 291–296. https://doi.org/10.1111/j.1474-9726.2010.00549.x

Kuo, G., Wu, C. Y., & Yang, H. Y. (2019). MiR-17-92 cluster and immunity. *Journal of the Formosan Medical Association*, *118*(1 Pt1), 2–6. https://doi.org/10.1016/j.jfma.2018.04.013

Li, W., Zhou, B. R., Hua, L. J., Guo, Z., & Luo, D. (2013). Differential miRNA profile on photoaged primary human fibroblasts irradiated with ultraviolet A. *Tumor Biology*, *34*(6), 3491–3500. https://doi.org/10.1007/s13277-013-0927-4

Liu, X., Cheng, Y., Yang, J., Xu, L., & Zhang, C. (2012). Cell-specific effects of miR-221/222 in vessels: molecular mechanism and therapeutic application. *Journal of Molecular and Cellular Cardiology*, *52*(1), 245–255. https://doi.org/10.1016/j.yjmcc.2011.11.008

Markopoulos, G. S., Roupakia, E., Tokamani, M., Vartholomatos, G., Tzavaras, T., Hatziapostolou, M., … Kolettas, E. (2017). Senescence-associated microRNAs target cell cycle regulatory genes in normal human lung fibroblasts. *Experimental Gerontology*, *96*, 110–122. https://doi.org/10.1016/j.exger.2017.06.017

Otsuka, M., Zheng, M., Hayashi, M., Lee, J.-D., Yoshino, O., Lin, S., & Han, J. (2008). Impaired microRNA processing causes corpus luteum insufficiency and infertility in mice. *The Journal of Clinical Investigation*, *118*(5), 1944–1954. https://doi.org/10.1172/JCI33680.1944

Rossi, M., & Gorospe, M. (2020). Noncoding RNAs controlling telomere homeostasis in senescence and aging. *Trends in Molecular Medicine*, *26*(4), 422–433. https://doi.org/10.1016/j.molmed.2020.01.010

Stratz, C., Nührenberg, T. G., Binder, H., Valina, C. M., Trenk, D., Hochholzer, W., … Fiebich, B. L. (2012). Micro-array profiling exhibits remarkable intra-individual stability of human platelet micro-RNA. *Thrombosis and Haemostasis*, *107*(4), 634–641. https://doi.org/10.1160/TH11-10-0742

Zhang, Y., Yang, C., Yang, S., & Guo, Z. (2020). MiRNA ‐ 27a decreases ultraviolet B irradiation‐induced cell damage. *Journal of Cellular Biochemistry*, *121*(2), 1032–1038. https://doi.org/10.1002/jcb.29337
